# Eosinophilia and Risk of Thrombosis and Mortality in Hospitalized Patients: A Retrospective Cohort Study

**DOI:** 10.3390/life16020241

**Published:** 2026-02-02

**Authors:** Ronen Shavit, Adi Kidron, Ramit Maoz Segal, Stanley Niznik, Soad Haj Yahia, Mona Iancovici-Kidon, Irena Offengenden, Diti Machnes Maayan, Yulia Lifshitz-Tunitsky, Liraz Olmer, Nancy Agmon-Levin

**Affiliations:** 1 The Zabludowicz Center for Autoimmune Diseases, Sheba Medical Center, Tel Aviv 52621, Israel; 2 Faculty of Medical & Health Sciences, Tel Aviv University, Tel Aviv 69978, Israel; 3 Department of Urology, Tel Aviv Soourasky Medical Center, Tel Aviv 64239, Israel; 4 Bio-Statistical and Bio-Mathematical Unit, The Gertner Institute of Epidemiology and Health Policy Research, Sheba Medical Center, Tel Aviv 52621, Israel

**Keywords:** eosinophil count, eosinophilia, hypereosinophilia, mortality, risk factors, thromboembolic event, thrombosis

## Abstract

Background: Eosinophilia, defined as peripheral blood eosinophil counts > 0.5 K/μL, is associated with various clinical conditions, including allergic, infectious, and malignant diseases. Emerging evidence suggests that eosinophils may contribute to thrombo-inflammatory processes, but their association with thromboembolic events and mortality remains insufficiently characterized. This study aimed to evaluate whether eosinophilia is independently associated with increased risk of thromboembolic events and mortality in hospitalized patients. Methods: We conducted a retrospective cohort study using electronic medical records from Sheba Medical Center (2011–2020). Eosinophilia was classified as mild (0.5–1.5 K/μL) or hypereosinophilia (HE, >1.5 K/μL). Patients with eosinophilia were matched 1:1 to controls with normal eosinophil counts based on age, sex, and follow-up duration. Results: Among 93,320 patients (46,660 with eosinophilia and 46,660 controls), thromboembolic events occurred in 20.9% of eosinophilic patients vs. 9.8% of controls. Eosinophilia was independently associated with thrombosis (OR = 1.33; 95% CI, 1.28–1.38; *p* < 0.0001), with increased risk from counts ≥ 0.7 K/μL. Mortality was also higher among eosinophilic patients (HR = 1.17; 95% CI, 1.13–1.20; *p* < 0.0001). Conclusions: Eosinophilia is associated with increased thromboembolic and mortality risk, highlighting the importance of eosinophil monitoring in clinical practice.

## 1. Introduction

Eosinophilia, defined as an elevated eosinophil count in peripheral blood, is traditionally associated with a range of clinical conditions, including allergic disorders, parasitic infections, autoimmune diseases, and solid and hematological malignancies. While eosinophils play a physiologic role in immune responses, such as combating helminthic infections, their hyperactivity is also implicated in other pathological processes [1]. One potential such process is hyper-coagulation. Mechanistically, eosinophils may promote thrombosis through several pathways, including endothelial injury, platelet activation, and the release of granule proteins and eosinophil extracellular traps (EETs), all of which exert pro-thrombotic effects. Preclinical studies have further shown that eosinophil granules contain inflammatory, oxidative, and coagulation-enhancing components such as major basic protein and eosinophil peroxidase which can amplify thrombin generation and lower the threshold for clot formation. Together, these biological processes provide a plausible mechanistic basis for the association between elevated eosinophil counts and increased thrombotic risk [1,2,3,4]. In addition, increased tissue factor expression, which is highly associated with coagulation, was documented following eosinophils degranulation in patients with hypereosinophilic syndrome (HES) [2], alluding to a plausible direct effect of eosinophil degranulation on the coagulation state.

Thrombotic events are one of the major public health priorities as they are considered responsible for 1 in 4 deaths worldwide [5] and the imminent cause of most heart attacks, strokes, and venous thromboembolism (VTE), such as deep vein thrombosis (DVT) and pulmonary embolism (PE). Established causes of thrombophilia such as antiphospholipid syndrome, inherited thrombophilic disorders, malignancy, and systemic inflammatory conditions are well-recognized contributors to thrombotic risk [6]. However, despite extensive research into thrombosis risk factors, the role of eosinophilia in thrombotic complications remains inadequately explored. Evidence suggests that eosinophils may interact with key pathways involved in clot formation, potentially amplifying both localized and systemic inflammatory responses that underpin vascular injury. In recent years, several case reports [7,8,9,10,11,12,13,14,15,16], retrospective cohort studies [17,18,19] and a recent systematic review and meta-analysis [20] have described thrombotic complications in patients with elevated blood eosinophil counts and/or HES. These studies, although valuable, were limited by modest sample sizes, heterogeneous inclusion criteria, and incomplete adjustment for confounders, leaving uncertainty regarding the magnitude and lower threshold of thrombosis risk associated with more common, mild eosinophil elevations. Epidemiologic surveys indicate that transient eosinophilia is far from rare: up to 3% of healthy adults register at least one eosinophil count above 0.5 K/µL during routine blood tests [21]. With rising global prevalence of allergic and immune-mediated diseases, the absolute number of individuals exhibiting either transient or chronic eosinophilia is poised to increase, underscoring the need to clarify downstream clinical consequences. Complementing these observations, recent multi-omics and single-cell studies have positioned eosinophils as immunologic sentinels at epithelial barriers that can influence systemic coagulation pathways, thereby framing eosinophils as a potentially modifiable nexus between inflammation and thrombosis [22,23]. The present study aimed to leverage a large, longitudinal electronic health-record cohort to clarify whether modest elevations in peripheral eosinophil counts can independently predict thromboembolic events and all-cause mortality in hospitalized patients and to delineate the eosinophil threshold at which these risks manifest.

## 2. Methods

In this retrospective cohort study, we queried the MDClone © analytics platform (MDClone Ltd., Be’er Sheva, Israel) at Sheba Medical Center-Israel’s largest tertiary-care hospital- to obtain a fully de-identified dataset. MDClone integrates data from the institution’s operational systems, harmonizing electronic health records, inpatient-pharmacy transactions, laboratory information systems, and the national mortality registry into a unified common-data model. This design reflects a real-world inpatient hospital-based cohort, rather than a predefined population registry. We retrieved data on all consecutive adult patients (age ≥ 18 years) hospitalized between January 2011 and December 2020, who had at least two eosinophil counts > 0.5 K/μL within a one-month interval (either before or after each other). This criterion was used to ensure the presence of a true eosinophilic state and to exclude potential laboratory error.

For each patient, the highest recorded eosinophil count served as the index date. Follow-up time was calculated from this index date until the end of the study period or death, whichever occurred first. A control group of hospitalized patients was matched in a 1:1 ratio according to age, sex, and follow-up strata and included only individuals who had no documented eosinophil count above 0.5 K/μL.

We defined mild eosinophilia as an eosinophil count of 0.5–1.5 K/μL and hypereosinophilia (HE) count higher than >1.5 K/μL. Using the MDclone, data was collected and analyzed for each patient including the following: baseline demographic; laboratory results; clinical comorbidities such as smoking, hypertension, dyslipidemia, obesity, infections, malignant solid tumors, hematologic malignancies including acute and chronic lymphoid leukemias, acute and chronic myeloid leukemias, chronic myelomonocytic leukemia, Hodgkin lymphoma and non-Hodgkin lymphoma, non-thrombotic cardiac and neurologic diseases (e.g., heart failure, valve diseases, cardiomyopathies, dementia, movement disorders, etc.), kidney diseases (acute and chronic kidney failure, glomerulonephritis, interstitial nephritis, etc.), hepatic diseases (e.g., viral, drug induced, alcoholic cirrhosis, etc.), lung diseases (asthma, chronic obstructive lung disease, interstitial lung disease, etc.), as well as arterial and venous thromboembolism and death during the follow-up time. The prevalence of these comorbidities was analyzed and compared between the eosinophilia and control group.

This study was approved by the ethical committee of the Sheba Medical Center, in accordance with the Declaration of Helsinki (IRB approval number: 8344-21-SMC).

## 3. Statistical Analysis

All statistical analyses were conducted using SAS software version 9.4 (SAS Institute Inc., Cary, NC, USA). Descriptive statistics were used to characterize the study population. Continuous variables were summarized as means ± standard deviation (SD), while categorical variables were expressed as frequencies and percentages. Comparisons between the eosinophilia and control groups, as well as between subgroups with mild eosinophilia and hypereosinophilia (HE), were performed using the unpaired Student’s *t*-test for continuous variables and the Chi-square test for categorical variables. Given the very large sample size, formal tests for normality were not applied, and parametric tests were considered appropriate based on the robustness afforded by the central limit theorem. Statistical significance was defined as a *p*-value < 0.05.

### 3.1. Multivariate Analysis

To assess the association between eosinophilia and thromboembolism while adjusting for potential confounders, a two-step regression analysis was employed:

1. Step 1: Variables associated with thromboembolism in univariate analyses (*p* < 0.05), present in ≥10% of cases and without evidence of substantial collinearity were included in a multivariable logistic regression model. Collinearity was evaluated using variance inflation factors (VIFs) and pairwise correlation matrices, and variables with VIF > 5 were not entered simultaneously.
2. Step 2: Significant variables from step 1 were retained, and eosinophilia was added to the model to assess its adjusted association with thromboembolism. Adjusted odds teratios (ORs) with 95% confidence intervals (CIs) were calculated. Subgroup analyses were conducted to compare ORs for thromboembolism between patients with mild eosinophilia and HE.

To evaluate dose-dependent mortality risk, eosinophil count was also analyzed as a continuous variable in a multivariate logistic regression model.

We performed additional stratified multivariate logistic regression analyses across key clinical subgroups. Specifically, separate models were constructed for patients with the following comorbidities: smoking status, hypertension, diabetes mellitus, dyslipidemia, non-thrombotic cardiac disease, non-thrombotic neurologic disease, infectious diseases, and kidney disease. In each subgroup, we assessed whether eosinophilia remained independently associated with thrombotic outcomes.

### 3.2. Outcome Analysis for Mortality

Cox proportional hazards regression analysis was utilized to examine the relationship between eosinophilia and mortality. The same two-step approach described for thromboembolism was applied. Adjusted hazard ratios (HRs) with 95% CIs were calculated, and subgroup analyses were performed to compare HRs for mortality among patients with mild eosinophilia and HE.

Kaplan–Meier survival analysis was performed to compare all-cause mortality among patients with no eosinophilia, mild, and moderate/severe eosinophilia. Survival curves were compared using the log-rank test.

### 3.3. Threshold Analysis

The optimal eosinophil count threshold for increased risk of thromboembolism and mortality was determined using the “distance to (0, 1)” method, sensitivity–specificity equality, and the Youden index.

## 4. Results

Our cohort included 93,320 patients who were hospitalized at Sheba Medical Center between January 2011 and December 2020, of whom 46,660 were diagnosed with eosinophilia (study group), and from a control pool of 65,535 hospitalized patients with no-eosinophilia, we matched the closest controls of 46,660 patients, according to sex, age, and follow-up duration. (Table 1). Of note, the mean age and follow-up duration were statistically different between groups; however, this reflects the very large sample size, where even minimal differences may reach statistical significance. Importantly, the observed differences (approximately 2 years in mean age and less than 1 year in mean follow-up) are not clinically meaningful. In addition, in all subsequent multivariable analyses we adjusted for both age and follow-up duration to eliminate any potential residual confounding.

In our study group, mild–moderate eosinophilia (eosinophil counts of 0.5–1.5 K/μL) was observed in 43,000 patients, while 3660 patients exhibited hypereosinophilia (HE; eosinophil count > 1.5 K/μL).

### 4.1. Eosinophilia and Comorbidities

Comorbidities and risk factors for thromboembolism and mortality for both study and control groups are delineated in Table 1. In univariate analysis, higher prevalence of known thromboembolic risk factors such as smoking, hypertension, diabetes mellitus, dyslipidemia, and obesity were observed in our study group compared to the control group. Moreover, other comorbidities, such as infections, non-thrombotic cardiac disease, neurologic, hepatic lung, kidney, as well as both solid and hematological malignancies, were more common in patients with elevated eosinophil counts (Table 1).

### 4.2. Eosinophilia and Thrombosis

In our entire cohort of 93,320 patients, 16,666 suffered a thrombotic event within the study period. Of which, 4502/93,320 (4.8%) were diagnosed with venous thrombosis and 12,164/93,320 (13%) with arterial events. As expected, participants of the female gender and younger age were inversely associated with thrombosis while traditional risk factors and certain comorbidities were linked with this worse outcome (Table 2). Nonetheless, our data support an association between eosinophilia and both venous and arterial thrombosis, as absolute event rates were higher in the eosinophilia group compared with controls. Venous thromboembolic events occurred in 3013 of 46,660 patients (6.5%) in the eosinophilia group vs. 1489 of 46,660 patients (3.2%) in the control group, corresponding to an absolute risk difference of 3.3% (*p* < 0.0001). Similarly, arterial thrombotic events were observed in 7481 of 46,660 patients (16.0%) compared with 4683 of 46,660 patients (10.0%), yielding an absolute risk difference of 6.0% (*p* < 0.001). Given the large cohort size, small differences in event rates may reach statistical significance; thus, clinical interpretation should consider the absolute risk differences alongside the adjusted effect estimates. When adjusting for potential confounders in the multivariate logistic regression model, several comorbid conditions were independently associated with thromboembolic events in the eosinophilic cohort. These included older age, male sex, smoking, hypertension, diabetes mellitus, dyslipidemia, infections, non-thrombotic cardiac and neurologic diseases, and kidney disease. Importantly, eosinophil count remained an independent risk factor for thrombosis in our cohort with OR = 1.33 95%CI (1.28–1.38) *p* < 0.0001, suggesting its additive contribution beyond traditional thrombotic risk factors (Table 2).

Furthermore, this increased thrombotic risk, although numerically higher in patients with HE compared to mild–moderate counts (Table 3), was eventually not related to the defined levels of eosinophilia in a multivariate analysis OR = 1.32 95%CI (1.27–1.38) and 1.36 95%CI (1.24–1.49) for mild and HE subgroups, respectively, with no statistical difference between these subgroups OR 1.04 (C.I. 0.95–1.13); *p* = 0.44.

In subgroup analyses across various clinical conditions, eosinophilia consistently emerged as an independent predictor of thrombotic events. As shown in Table 4, odds ratios ranged from 1.14 to 1.26 across different subgroups, all statistically significant. For example, among patients with hypertension (n = 28,003), eosinophilia was associated with a 20% increase in thrombosis risk (OR 1.2, 95% CI 1.14–1.26, *p* < 0.0001). Notably, even in traditionally non-thrombotic domains such as neurologic disease (n = 4299), eosinophilia remained a significant risk factor (OR 1.26, 95% CI 1.1–1.43, *p* = 0.0006). These findings strengthen the robustness of our primary model by demonstrating that the pro-thrombotic signal of eosinophilia is reproducible across diverse patient groups.

Lastly, we wished to define a cutoff for eosinophil counts linked with increased thrombosis in our entire cohort. This was performed using several statistical methods (see Section 2), in all of which approximate counts of eosinophils equal or above ~>0.7 K/μL were linked with thrombosis.

### 4.3. Eosinophilia and Mortality

During the study period, 21,060 out of 93,320 patients (22.5%) died. Among them, 12,830 out of 46,660 (27.5%) were in the eosinophilia group, compared to 8230 out of 46,660 (17.6%) in the control group (*p* < 0.0001). The observed difference in mortality rates, though statistically significant, should be interpreted cautiously in light of the large sample size, which can amplify the detection of small differences.

In multivariate cox regression analysis of various factors associated with mortality, eosinophilia remained an independent risk factor for this grave outcome with HR of 1.17; 95% CI (1.13–1.2) *p* < 0.0001 (Table 5). In our cohort, the risk of mortality was directly related to the levels of eosinophilia. Particularly mortality was documented in 43.3% vs. 26.2% of patients in our HE and mild eosinophilia subgroups (Table 5), with a HR between these subgroups of 1.35, CI 1.28–1.43, (*p* < 0.0001). When analyzed as a continuous variable, each 1000 cells/μL increase in eosinophil count was associated with 9% higher odds of death (OR = 1.09, 95% CI: 1.07–1.11, *p* < 0.001).

**Figure 1:**
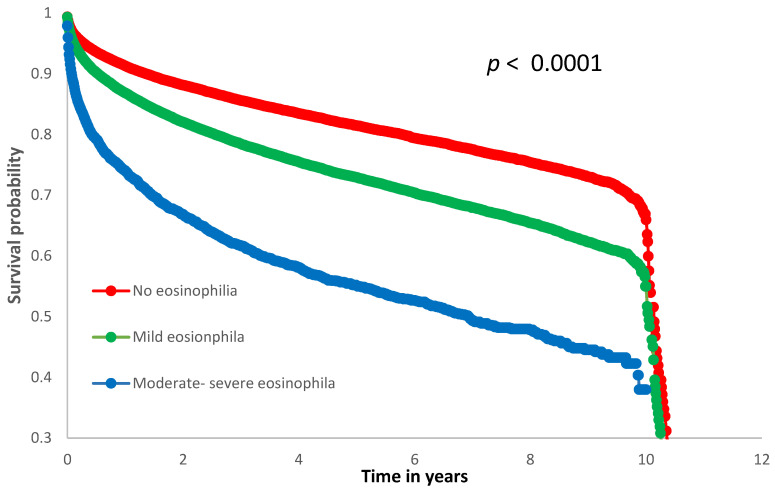
Kaplan–Meier survival curves stratified by eosinophil count. Legend: Kaplan–Meier curves illustrating survival probability over time in years among hospitalized patients, stratified by eosinophil count groups: no eosionophilia (eosinophil count ≤ 0.5 K/μL; red), mild eosinophilia (0.5–1.5 K/μL; green), and moderate/severe eosinophilia (>1.5 K/μL; blue). The survival probability decreased progressively with increasing eosinophil count. A significant difference in survival was observed between the groups (log-rank *p* < 0.0001).

Kaplan–Meier analysis revealed significant differences in survival among the three eosinophil subgroups (log-rank *p* < 0.0001), as illustrated in Figure 1 (the corresponding survival table is available in the Supplementary Material). Patients with moderate/severe eosinophilia showed the lowest survival probability throughout the follow-up period, while those without eosinophilia had the most favorable survival.

## 5. Discussion

In the current study, we aimed to assess the links between eosinophilia and thrombosis in a large cohort of hospitalized patients. We found an association between eosinophilia and various comorbidities, many of which are related to thrombosis; however, in a multivariate analysis, elevated eosinophil count remained an independent risk of thrombosis, with an odds ratio of 1.33. These findings challenge the notion that eosinophils-associated thrombosis is solely attributable to the underlying chronic inflammatory processes, rather supporting the hypothesis that eosinophils themselves promote a pro-coagulative state.

### 5.1. Eosinophilia and Thrombosis

Historically, the connections between eosinophils and thromboembolic events were documented in patients with very high levels of eosinophils such as the studies of Orly Leiva et al., who reported thrombotic events in 17 out of 71 (24%) patients with HES (eosinophil counts > 1.500 G/L [17], and Réau et al., who reported thrombotic events among 29 out of 54 (54%) patients with eosinophil-related diseases and blood eosinophil counts of ≥1 G/L, [19]. A recent meta-analysis by Xu et al. [20], pooling 22 studies across various clinical settings and eosinophilic diseases, further reinforced the association between eosinophilia and thrombosis, particularly in the context of hypereosinophilia. However, many of the included studies were limited by small sample sizes, high clinical heterogeneity, and lacked uniform thresholds for eosinophilia, thereby limiting their ability to establish practical risk stratification tools. In our study, we identified an augmented risk of thrombosis even in patients with mild to moderate eosinophilia, suggesting that the pro-thrombotic potential of eosinophils is not restricted to extreme elevations. To the best of our knowledge, this is the first large-scale study to define a quantitative threshold for thrombotic risk (~0.7 K/μL), a level below traditional HES cutoffs, which may inform broader clinical decision-making. Nevertheless, we fully acknowledge that this threshold requires validation in independent prospective studies. Lastly, vasculitis, which is thrombogenic by itself, was more prevalent in our study group as expected. However, it was present in less than 1% of our entire cohort and therefore cannot explain the increased thrombotic rate of 20–25.6% observed in our study group.

Taking it all together, this underscores the necessity for clinicians to incorporate eosinophil counts into their evaluation and management strategies of patients at high risk of thrombotic events and particularly in patients presenting with thrombosis or other cardiovascular risks. However, given the observational nature of our study and the multifactorial background of eosinophilia, we refrained from making therapeutic recommendations such as the routine use of antiplatelet prophylaxis and instead advocate for future prospective trials to clarify whether eosinophil-lowering or anti-thrombotic interventions may be warranted in selected populations. Importantly, although the adjusted odds ratios indicated an independent association between eosinophilia and thrombotic outcomes, the absolute risk differences were modest, amounting to 3.3% for venous and 6.0% for arterial thrombotic events. These figures help contextualize the statistically significant associations observed in this large cohort and emphasize that eosinophilia should be viewed as a contributory risk marker rather than a dominant determinant of thrombotic risk.

### 5.2. Eosinophilia and Mortality

Another observation of the current study is the escalation in mortality among patients with elevated eosinophil corroborating previous data on this subject, particularly among those with hypereosinophilia of more than 1.5 K/μL. Our results are consistent with the findings of Deniz Yilmaz et al. [24], who reported higher mortality rates in patients with higher eosinophil counts compared to those with lower eosinophil counts. In our cohort, a higher prevalence of many comorbidities and predominant malignancies among patients with eosinophilia was documented, a connection that is well established in the literature [25,26]. In this regard, using multivariate analysis revealed that elevated counts particularly in the HE group were independently related to mortality. Importantly, when eosinophil count was analyzed as a continuous variable, each 1000 cells/μL increase was associated with a 9% rise in the odds of death (OR = 1.09, 95% CI: 1.07–1.11, *p* < 0.001), further supporting a dose–response relationship between eosinophilia and mortality. The Kaplan–Meier survival curves further support the association between increasing eosinophil levels and reduced survival, with a clear gradient observed between the groups. This visual evidence complements our multivariate findings and strengthens the relevance of eosinophil count as a potential prognostic marker, even at mild to moderate levels.

### 5.3. Eosinophilia and Other Comorbidities

We also observed higher rates of other comorbidities, such as cardiovascular and metabolic diseases (hypertension, diabetes, dyslipidemia), among patients with elevated eosinophils. However, unlike the well-established link between eosinophilia and malignancy, the association between eosinophilia and these comorbidities remains unclear. Several studies have explored the relationship between eosinophilia and metabolic diseases [27,28,29,30]. For example, Naharro-González et al. reported higher eosinophil counts in asthmatic patients with dyslipidemia, hypertension, and other cardiovascular conditions compared to those with asthma and without additional comorbidities [30]. In contrast, Hartl et al. found no association between eosinophilia and hypertension or cardiovascular disease in the general population [31], while Amini et al. found no association between eosinophilia and blood pressure [32]. Therefore, further research is needed for the establishment of these relationships, while potentially differentiating between hospitalized and non-hospitalized patients.

### 5.4. Limitations

While our study provides valuable insights, it is important to acknowledge its limitations. Although eosinophilia emerged as an independent predictor of thromboembolism and mortality, it is important to emphasize that the magnitude of this association was modest relative to other well-established risk factors, such as advanced age, malignancy, and cardiovascular disease. Given the large sample size, some statistically significant findings should be interpreted with caution, as small numerical differences may achieve low *p*-values. The adjusted effect sizes indicate that eosinophilia likely contributes to thrombotic and mortality risk but is not the sole or dominant driver. Clinical decisions should integrate these findings alongside other risk factors.

The retrospective design restricts our ability to establish definitive causality, and there may be unmeasured confounding factors. Furthermore, data were retrieved from electronic medical records and diagnostic codes provided by physicians, which may not capture the full medical history of patients. There were significant gaps in the availability of certain clinical parameters. Specifically, many tests that could have provided insight into the etiology of eosinophilia—such as parasitological evaluations, allergy workups, genetic testing, and dedicated laboratory investigations for hypereosinophilic syndromes—were either missing or incomplete in a substantial number of patients. As a result, we were unable to draw firm conclusions regarding the underlying causes of eosinophilia in many cases and therefore could not stratify patients by eosinophilia etiology (e.g., secondary vs. primary). Future prospective studies are essential to further elucidate the mechanisms driving the observed associations and to explore potential therapeutic interventions aimed at modulating eosinophil activity. Even so, the size of our cohort of over 93,000 patients allows a robust analysis that we believe minimizes many of the detailed limitations and thus may have significant implications for clinical practice.

Future directions should include prospective interventional studies to determine whether anti-aggression/coagulation therapy or pharmacological eosinophil suppression (e.g., anti-IL-5 or anti-Siglec-8 monoclonal antibodies) can translate into tangible reductions in thrombosis or mortality. Integration of high-dimensional single-cell transcriptomics with longitudinal biobanking may help elucidate whether distinct eosinophil phenotypes account for the heterogeneous risk observed.

### 5.5. Conclusions

Our findings reinforce the independent association between eosinophilia and thrombotic as well as mortality risk, even at non-extreme levels. While causal mechanisms remain to be fully elucidated, these results suggest that eosinophil counts—readily available in clinical practice—may warrant consideration as part of comprehensive thrombotic risk assessment, particularly in hospitalized patients.

## Figures and Tables

**Table 1:** Demographics and commodities among hospitalized patients with and without eosinphilia.

Risk Factors Related to Thrombosis and Mortality	Study Group (*n* = 46,600) No. (%)	Control Group (*n* = 46,600) No. (%)	*p* Value
Gender			
Female	20,719 (44.4)	20,719 (44.4)	1
Male	25,941 (55.6)	25,941 (55.6)	1
Age, mean (SD) years	56.29 (20.65)	54.66 (19.89)	<0.0001
Follow-up, mean (SD) years	3.86 (2.98)	4.04 (2.97)	<0.0001
Smoking	11,745 (25.2)	8187 (17.5)	<0.0001
Hypertension	16,123 (34.6)	11,880 (25.5)	<0.0001
Obesity	3813 (8.2)	2966 (6.4)	<0.0001
Diabetes mellitus	9369 (20.1)	6097 (13.1)	<0.0001
Dyslipidemia	10,824 (23.2)	8251 (17.7)	<0.0001
Infections	10,594 (22.7)	5017 (10.8)	<0.0001
Solid malignancy	5095(10.9)	3329 (7.1)	<0.0001
Hematologic malignancy	1759 (3.8)	546 (1.2)	<0.0001
Non-thrombotic Neurological conditions	2543 (5.5)	1756 (3.8)	<0.0001
Non thrombotic Cardiac disease	6047 (13.0)	3268 (7.0)	<0.0001
Lung disease	6053 (12.9)	2630 (5.6)	<0.0001
Hepatic disease	1343 (2.9)	795 (1.7)	<0.0001
Kidney disease	8392 (18.0)	3846 (8.2)	<0.0001
Vasculitis	239 (0.5)	118 (0.3)	<0.0001

**Table 2:** Multivariate logistic regression analysis for thrombosis.

Parameter	Odds Ratio	Lower 95% CI	Upper 95% CI	*p*-Value
Female	0.85	0.82	0.88	<0.0001
Age group 19–39 vs. above 65	0.19	0.17	0.21	<0.0001
Age group 40–65 vs. above 65	0.66	0.63	0.69	<0.0001
Smoking	1.56	1.49	1.63	<0.0001
Hypertension	1.92	1.83	2.01	<0.0001
Diabetes mellitus	1.37	1.31	1.44	<0.0001
Dyslipidemia	1.79	1.71	1.87	<0.0001
Infections	2.09	1.99	2.19	<0.0001
Non-thrombotic cardiac disease	1.78	1.68	1.87	<0.0001
Non-thrombotic neurologic disease	1.61	1.50	1.73	<0.0001
Kidney disease	1.38	1.31	1.45	<0.0001
Eosinophilia	1.33	1.28	1.38	<0.0001

**Table 3:** Demographics and comorbidities among hospitalized patients with mild eosinophilia vs. hypereosinophilia (HE).

	Mild-Moderate Eosinophilia * (n = 43,000) No. (%)	Hyper Eosinophilia ** (n = 3660) No. (%)	*p*-Value
Gender			
female	19,060 (44.3)	1659 (45.3)	0.2414
Male	23,940 (55.7)	2001 (54.7)	0.2414
Age, mean (SD)	55.94 (20.68)	60.37 (19.84)	<0.0001
Follow-up, mean (SD), y	3.91 (2.97)	3.24 (2.94)	<0.0001
Smoking	10,812 (25.1)	933 (25.5)	0.6418
Hypertension	14,646 (34.1)	1477 (40.4)	<0.0001
Obesity	3537 (8.2)	276 (7.5)	0.1467
Diabetes mellitus	8539 (19.9)	830 (22.7)	<0.001
Dyslipidemia	9946 (23.1)	878 (24)	0.2373
Infections	9216 (21.4)	1378 (37.7)	<0.0001
Solid malignancy	4557 (10.6)	538 (14.7)	<0.0001
Hematologic malignancy	1421 (3.3)	338 (9.2)	<0.0001
Non-thrombotic Neurological conditions	2295 (5.3)	248 (6.8)	0.0002
Non thrombotic Cardiac disease	5425 (12.6)	622 (17)	<0.0001
Lung disease	5452 (12.67)	601 (16.4)	<0.0001
Hepatic disease	1174 (2.7)	169 (0.1)	<0.0001
Kidney disease	7384 (17.2)	1008 (27.5)	<0.0001
Thrombosis	8791 (20.4)	948 (25.9)	<0.0001
Death	11,247 (26.2)	1583 (43.3)	<0.0001

Abbreviations: SD—standard deviation. * Mild eosinophilia—500–1500 K/μL; ** Hypereosinophilia > 1500 K/μL.

**Table 4:** Adjusted odds ratios for thrombotic events associated with eosinophilia across major clinical subgroups.

Subgroup	n (Patients)	Odds Ratio	Lower 95% CI	Upper 95% CI	*p*-Value
Smoking	19,932	1.23	1.13	1.33	<0.0001
Hypertension	28,003	1.2	1.14	1.26	<0.0001
Diabetes mellitus	15,466	1.18	1.09	1.26	<0.0001
Dyslipidemia	15,466	1.19	1.12	1.27	<0.0001
Infections	15,611	1.26	1.17	1.36	<0.0001
Non-thrombotic cardiac disease	9315	1.14	1.04	1.25	<0.0001
Non-thrombotic neurologic disease	4299	1.26	1.1	1.43	0.0006
Kidney disease	12,238	1.21	1.12	1.32	<0.0001

Abbreviations: CI—confidence interval.

**Table 5:** Multivariate Cox regression for mortality.

Parameter	Hazard Ratio	Lower 95% CI	Upper 95% CI	*p*-Value
Female	1.05	1.02	1.08	0.0022
Age group 40–65	5.50	5.02	6.03	<0.0001
Age group > 65	18.17	16.62	19.88	<0.0001
Thromboembolism	1.19	1.15	1.23	<0.0001
Malignancy	1.70	1.65	1.76	<0.0001
Smoking	1.15	1.11	1.19	<0.0001
Diabetes mellitus	1.19	1.15	1.22	<0.0001
Dyslipidemia	0.71	0.68	0.73	<0.0001
Infections	2.30	2.22	2.37	<0.0001
Non-thrombotic cardiac disease	1.23	1.18	1.27	<0.0001
Non-thrombotic neurologic disease	1.43	1.37	1.49	<0.0001
Kidney disease	1.53	1.48	1.58	<0.0001
Eosinophilia	1.17	1.13	1.20	<0.0001

Abbreviations: CI—confidence interval.

## Data Availability

The data presented in this study are available on request from the corresponding author due to restrictions imposed by patient-privacy legislation and the Sheba Medical Center Institutional Review Board.

## Supplementary Materials

The following supporting information can be downloaded at: https://www.mdpi.com/article/10.3390/life16020241/s1, Table S1: Detailed survival analysis data underlying the Kaplan–Meier curves, including numbers at risk and event counts across follow-up intervals for patients with no eosinophilia, mild eosinophilia, and hypereosinophilia.
