# Supplementary material for: Eosinophilia and Risk of Thrombosis and Mortality in Hospitalized Patients: A Retrospective Cohort Study
Source: Life (Basel). 2026 Feb 2;16(2):241. doi: 10.3390/life16020241 (PMC12942559; doi:10.3390/life16020241)
Supplement: Supplementary file 1 [file life-16-00241-s001.zip › KM survival table 4.1.2026.pdf]

Obs	Sub_group	fuptimey	_CENSOR_	SURVIVAL	SDF_LCL	SDF_UCL	STRATUM
1	0	0.00	.	1.00000	1.00000	1.00000	1
2	0	0.00	0	0.99366	0.99289	0.99434	1
3	0	0.00	1	0.99366	.	.	1
4	0	0.00	1	0.99366	.	.	1
5	0	0.00	1	0.99366	.	.	1
6	0	0.00	1	0.99366	.	.	1
7	0	0.00	1	0.99366	.	.	1
8	0	0.00	1	0.99366	.	.	1
9	0	0.00	1	0.99366	.	.	1
10	0	0.00	1	0.99366	.	.	1
11	0	0.00	1	0.99366	.	.	1
12	0	0.00	1	0.99366	.	.	1
13	0	0.00	1	0.99366	.	.	1
14	0	0.00	1	0.99366	.	.	1
15	0	0.00	1	0.99366	.	.	1
16	0	0.00	1	0.99366	.	.	1
17	0	0.00	1	0.99366	.	.	1
18	0	0.00	1	0.99366	.	.	1
19	0	0.00	1	0.99366	.	.	1
20	0	0.00	1	0.99366	.	.	1
21	0	0.00	1	0.99366	.	.	1
22	0	0.00	1	0.99366	.	.	1
23	0	0.00	1	0.99366	.	.	1
24	0	0.00	1	0.99366	.	.	1
25	0	0.00	1	0.99366	.	.	1
26	0	0.00	1	0.99366	.	.	1
27	0	0.00	1	0.99366	.	.	1
28	0	0.00	1	0.99366	.	.	1
29	0	0.00	1	0.99366	.	.	1
30	0	0.00	1	0.99366	.	.	1
31	0	0.00	1	0.99366	.	.	1
32	0	0.00	1	0.99366	.	.	1
33	0	0.00	1	0.99366	.	.	1
34	0	0.00	1	0.99366	.	.	1
35	0	0.00	1	0.99366	.	.	1

Obs	Sub_group	fuptimey	_CENSOR_	SURVIVAL	SDF_LCL	SDF_UCL	STRATUM
36	0	0.00	1	0.99366	.	.	1
37	0	0.00	1	0.99366	.	.	1
38	0	0.00	1	0.99366	.	.	1
39	0	0.00	1	0.99366	.	.	1
40	0	0.01	0	0.98801	0.98698	0.98896	1
41	0	0.01	1	0.98801	.	.	1
42	0	0.01	1	0.98801	.	.	1
43	0	0.01	1	0.98801	.	.	1
44	0	0.01	1	0.98801	.	.	1
45	0	0.01	1	0.98801	.	.	1
46	0	0.01	1	0.98801	.	.	1
47	0	0.01	1	0.98801	.	.	1
48	0	0.01	1	0.98801	.	.	1
49	0	0.01	1	0.98801	.	.	1
50	0	0.01	1	0.98801	.	.	1
51	0	0.01	1	0.98801	.	.	1
52	0	0.01	1	0.98801	.	.	1
53	0	0.01	1	0.98801	.	.	1
54	0	0.01	1	0.98801	.	.	1
55	0	0.01	1	0.98801	.	.	1
56	0	0.01	1	0.98801	.	.	1
57	0	0.01	1	0.98801	.	.	1
58	0	0.01	1	0.98801	.	.	1
59	0	0.01	1	0.98801	.	.	1
60	0	0.01	1	0.98801	.	.	1
61	0	0.01	1	0.98801	.	.	1
62	0	0.01	1	0.98801	.	.	1
63	0	0.01	1	0.98801	.	.	1
64	0	0.01	1	0.98801	.	.	1
65	0	0.01	1	0.98801	.	.	1
66	0	0.01	1	0.98801	.	.	1
67	0	0.01	1	0.98801	.	.	1
68	0	0.01	1	0.98801	.	.	1
69	0	0.01	1	0.98801	.	.	1
70	0	0.01	1	0.98801	.	.	1

Obs	Sub_group	fuptimey	_CENSOR_	SURVIVAL	SDF_LCL	SDF_UCL	STRATUM
71	0	0.01	1	0.98801	.	.	1
72	0	0.01	1	0.98801	.	.	1
73	0	0.01	1	0.98801	.	.	1
74	0	0.01	1	0.98801	.	.	1
75	0	0.01	1	0.98801	.	.	1
76	0	0.01	1	0.98801	.	.	1
77	0	0.01	1	0.98801	.	.	1
78	0	0.01	1	0.98801	.	.	1
79	0	0.01	1	0.98801	.	.	1
80	0	0.01	1	0.98801	.	.	1
81	0	0.01	1	0.98801	.	.	1
82	0	0.01	1	0.98801	.	.	1
83	0	0.01	1	0.98801	.	.	1
84	0	0.01	1	0.98801	.	.	1
85	0	0.01	1	0.98801	.	.	1
86	0	0.01	1	0.98801	.	.	1
87	0	0.01	1	0.98801	.	.	1
88	0	0.01	1	0.98801	.	.	1
89	0	0.01	1	0.98801	.	.	1
90	0	0.01	1	0.98801	.	.	1
91	0	0.01	1	0.98801	.	.	1
92	0	0.01	1	0.98801	.	.	1
93	0	0.01	1	0.98801	.	.	1
94	0	0.01	1	0.98801	.	.	1
95	0	0.01	1	0.98801	.	.	1
96	0	0.01	1	0.98801	.	.	1
97	0	0.01	1	0.98801	.	.	1
98	0	0.01	1	0.98801	.	.	1
99	0	0.01	1	0.98801	.	.	1
100	0	0.01	1	0.98801	.	.	1
101	0	0.01	1	0.98801	.	.	1
102	0	0.01	1	0.98801	.	.	1
103	0	0.01	1	0.98801	.	.	1
104	0	0.01	1	0.98801	.	.	1
105	0	0.01	1	0.98801	.	.	1

Obs	Sub_group	fuptimey	_CENSOR_	SURVIVAL	SDF_LCL	SDF_UCL	STRATUM
**106**	0	0.01	1	0.98801	.	.	1
**107**	0	0.01	1	0.98801	.	.	1
**108**	0	0.01	1	0.98801	.	.	1
**109**	0	0.02	0	0.98395	0.98277	0.98505	1
**110**	0	0.02	1	0.98395	.	.	1
**111**	0	0.02	1	0.98395	.	.	1
**112**	0	0.02	1	0.98395	.	.	1
**113**	0	0.02	1	0.98395	.	.	1
**114**	0	0.02	1	0.98395	.	.	1
**115**	0	0.02	1	0.98395	.	.	1
**116**	0	0.02	1	0.98395	.	.	1
**117**	0	0.02	1	0.98395	.	.	1
**118**	0	0.02	1	0.98395	.	.	1
**119**	0	0.02	1	0.98395	.	.	1
**120**	0	0.02	1	0.98395	.	.	1
**121**	0	0.02	1	0.98395	.	.	1
**122**	0	0.02	1	0.98395	.	.	1
**123**	0	0.02	1	0.98395	.	.	1
**124**	0	0.02	1	0.98395	.	.	1
**125**	0	0.02	1	0.98395	.	.	1
**126**	0	0.02	1	0.98395	.	.	1
**127**	0	0.02	1	0.98395	.	.	1
**128**	0	0.02	1	0.98395	.	.	1
**129**	0	0.02	1	0.98395	.	.	1
**130**	0	0.02	1	0.98395	.	.	1
**131**	0	0.02	1	0.98395	.	.	1
**132**	0	0.02	1	0.98395	.	.	1
**133**	0	0.02	1	0.98395	.	.	1
**134**	0	0.02	1	0.98395	.	.	1
**135**	0	0.02	1	0.98395	.	.	1
**136**	0	0.02	1	0.98395	.	.	1
**137**	0	0.02	1	0.98395	.	.	1
**138**	0	0.02	1	0.98395	.	.	1
**139**	0	0.02	1	0.98395	.	.	1
**140**	0	0.02	1	0.98395	.	.	1

Obs	Sub_group	fuptimey	_CENSOR_	SURVIVAL	SDF_LCL	SDF_UCL	STRATUM
**141**	0	0.02	1	0.98395	.	.	1
**142**	0	0.02	1	0.98395	.	.	1
**143**	0	0.02	1	0.98395	.	.	1
**144**	0	0.02	1	0.98395	.	.	1
**145**	0	0.02	1	0.98395	.	.	1
**146**	0	0.02	1	0.98395	.	.	1
**147**	0	0.02	1	0.98395	.	.	1
**148**	0	0.02	1	0.98395	.	.	1
**149**	0	0.02	1	0.98395	.	.	1
**150**	0	0.02	1	0.98395	.	.	1
**151**	0	0.02	1	0.98395	.	.	1
**152**	0	0.02	1	0.98395	.	.	1
**153**	0	0.02	1	0.98395	.	.	1
**154**	0	0.02	1	0.98395	.	.	1
**155**	0	0.02	1	0.98395	.	.	1
**156**	0	0.02	1	0.98395	.	.	1
**157**	0	0.02	1	0.98395	.	.	1
**158**	0	0.02	1	0.98395	.	.	1
**159**	0	0.02	1	0.98395	.	.	1
**160**	0	0.02	1	0.98395	.	.	1
**161**	0	0.02	1	0.98395	.	.	1
**162**	0	0.02	1	0.98395	.	.	1
**163**	0	0.02	1	0.98395	.	.	1
**164**	0	0.02	1	0.98395	.	.	1
**165**	0	0.02	1	0.98395	.	.	1
**166**	0	0.02	1	0.98395	.	.	1
**167**	0	0.02	1	0.98395	.	.	1
**168**	0	0.03	0	0.98109	0.97981	0.98229	1
**169**	0	0.03	1	0.98109	.	.	1
**170**	0	0.03	1	0.98109	.	.	1
**171**	0	0.03	1	0.98109	.	.	1
**172**	0	0.03	1	0.98109	.	.	1
**173**	0	0.03	1	0.98109	.	.	1
**174**	0	0.03	1	0.98109	.	.	1
**175**	0	0.03	1	0.98109	.	.	1

Obs	Sub_group	fuptimey	_CENSOR_	SURVIVAL	SDF_LCL	SDF_UCL	STRATUM
176	0	0.03	1	0.98109	.	.	1
177	0	0.03	1	0.98109	.	.	1
178	0	0.03	1	0.98109	.	.	1
179	0	0.03	1	0.98109	.	.	1
180	0	0.03	1	0.98109	.	.	1
181	0	0.03	1	0.98109	.	.	1
182	0	0.03	1	0.98109	.	.	1
183	0	0.03	1	0.98109	.	.	1
184	0	0.03	1	0.98109	.	.	1
185	0	0.03	1	0.98109	.	.	1
186	0	0.03	1	0.98109	.	.	1
187	0	0.03	1	0.98109	.	.	1
188	0	0.03	1	0.98109	.	.	1
189	0	0.03	1	0.98109	.	.	1
190	0	0.03	1	0.98109	.	.	1
191	0	0.03	1	0.98109	.	.	1
192	0	0.03	1	0.98109	.	.	1
193	0	0.03	1	0.98109	.	.	1
194	0	0.03	1	0.98109	.	.	1
195	0	0.03	1	0.98109	.	.	1
196	0	0.03	1	0.98109	.	.	1
197	0	0.03	1	0.98109	.	.	1
198	0	0.03	1	0.98109	.	.	1
199	0	0.03	1	0.98109	.	.	1
200	0	0.03	1	0.98109	.	.	1
201	0	0.03	1	0.98109	.	.	1
202	0	0.03	1	0.98109	.	.	1
203	0	0.03	1	0.98109	.	.	1
204	0	0.03	1	0.98109	.	.	1
205	0	0.03	1	0.98109	.	.	1
206	0	0.03	1	0.98109	.	.	1
207	0	0.03	1	0.98109	.	.	1
208	0	0.03	1	0.98109	.	.	1
209	0	0.03	1	0.98109	.	.	1
210	0	0.03	1	0.98109	.	.	1

Obs	Sub_group	fuptimey	_CENSOR_	SURVIVAL	SDF_LCL	SDF_UCL	STRATUM
211	0	0.03	1	0.98109	.	.	1
212	0	0.03	1	0.98109	.	.	1
213	0	0.03	1	0.98109	.	.	1
214	0	0.03	1	0.98109	.	.	1
215	0	0.03	1	0.98109	.	.	1
216	0	0.03	1	0.98109	.	.	1
217	0	0.03	1	0.98109	.	.	1
218	0	0.03	1	0.98109	.	.	1
219	0	0.03	1	0.98109	.	.	1
220	0	0.03	1	0.98109	.	.	1
221	0	0.03	1	0.98109	.	.	1
222	0	0.03	1	0.98109	.	.	1
223	0	0.04	0	0.97834	0.97697	0.97962	1
224	0	0.04	1	0.97834	.	.	1
225	0	0.04	1	0.97834	.	.	1
226	0	0.04	1	0.97834	.	.	1
227	0	0.04	1	0.97834	.	.	1
228	0	0.04	1	0.97834	.	.	1
229	0	0.04	1	0.97834	.	.	1
230	0	0.04	1	0.97834	.	.	1
231	0	0.04	1	0.97834	.	.	1
232	0	0.04	1	0.97834	.	.	1
233	0	0.04	1	0.97834	.	.	1
234	0	0.04	1	0.97834	.	.	1
235	0	0.04	1	0.97834	.	.	1
236	0	0.04	1	0.97834	.	.	1
237	0	0.04	1	0.97834	.	.	1
238	0	0.04	1	0.97834	.	.	1
239	0	0.04	1	0.97834	.	.	1
240	0	0.04	1	0.97834	.	.	1
241	0	0.04	1	0.97834	.	.	1
242	0	0.04	1	0.97834	.	.	1
243	0	0.04	1	0.97834	.	.	1
244	0	0.04	1	0.97834	.	.	1
245	0	0.04	1	0.97834	.	.	1

Obs	Sub_group	fuptimey	_CENSOR_	SURVIVAL	SDF_LCL	SDF_UCL	STRATUM
246	0	0.04	1	0.97834	.	.	1
247	0	0.04	1	0.97834	.	.	1
248	0	0.04	1	0.97834	.	.	1
249	0	0.04	1	0.97834	.	.	1
250	0	0.04	1	0.97834	.	.	1
251	0	0.04	1	0.97834	.	.	1
252	0	0.04	1	0.97834	.	.	1
253	0	0.04	1	0.97834	.	.	1
254	0	0.04	1	0.97834	.	.	1
255	0	0.04	1	0.97834	.	.	1
256	0	0.04	1	0.97834	.	.	1
257	0	0.04	1	0.97834	.	.	1
258	0	0.04	1	0.97834	.	.	1
259	0	0.04	1	0.97834	.	.	1
260	0	0.04	1	0.97834	.	.	1
261	0	0.04	1	0.97834	.	.	1
262	0	0.04	1	0.97834	.	.	1
263	0	0.04	1	0.97834	.	.	1
264	0	0.04	1	0.97834	.	.	1
265	0	0.04	1	0.97834	.	.	1
266	0	0.04	1	0.97834	.	.	1
267	0	0.04	1	0.97834	.	.	1
268	0	0.04	1	0.97834	.	.	1
269	0	0.04	1	0.97834	.	.	1
270	0	0.04	1	0.97834	.	.	1
271	0	0.04	1	0.97834	.	.	1
272	0	0.04	1	0.97834	.	.	1
273	0	0.04	1	0.97834	.	.	1
274	0	0.04	1	0.97834	.	.	1
275	0	0.04	1	0.97834	.	.	1
276	0	0.04	1	0.97834	.	.	1
277	0	0.04	1	0.97834	.	.	1
278	0	0.04	1	0.97834	.	.	1
279	0	0.04	1	0.97834	.	.	1
280	0	0.04	1	0.97834	.	.	1

Obs	Sub_group	fuptimey	_CENSOR_	SURVIVAL	SDF_LCL	SDF_UCL	STRATUM
281	0	0.04	1	0.97834	.	.	1
282	0	0.04	1	0.97834	.	.	1
283	0	0.04	1	0.97834	.	.	1
284	0	0.04	1	0.97834	.	.	1
285	0	0.04	1	0.97834	.	.	1
286	0	0.05	0	0.97579	0.97435	0.97715	1
287	0	0.05	1	0.97579	.	.	1
288	0	0.05	1	0.97579	.	.	1
289	0	0.05	1	0.97579	.	.	1
290	0	0.05	1	0.97579	.	.	1
291	0	0.05	1	0.97579	.	.	1
292	0	0.05	1	0.97579	.	.	1
293	0	0.05	1	0.97579	.	.	1
294	0	0.05	1	0.97579	.	.	1
295	0	0.05	1	0.97579	.	.	1
296	0	0.05	1	0.97579	.	.	1
297	0	0.05	1	0.97579	.	.	1
298	0	0.05	1	0.97579	.	.	1
299	0	0.05	1	0.97579	.	.	1
300	0	0.05	1	0.97579	.	.	1
301	0	0.05	1	0.97579	.	.	1
302	0	0.05	1	0.97579	.	.	1
303	0	0.05	1	0.97579	.	.	1
304	0	0.05	1	0.97579	.	.	1
305	0	0.05	1	0.97579	.	.	1
306	0	0.05	1	0.97579	.	.	1
307	0	0.05	1	0.97579	.	.	1
308	0	0.05	1	0.97579	.	.	1
309	0	0.05	1	0.97579	.	.	1
310	0	0.05	1	0.97579	.	.	1
311	0	0.05	1	0.97579	.	.	1
312	0	0.05	1	0.97579	.	.	1
313	0	0.05	1	0.97579	.	.	1
314	0	0.05	1	0.97579	.	.	1
315	0	0.05	1	0.97579	.	.	1

Obs	Sub_group	fuptimey	_CENSOR_	SURVIVAL	SDF_LCL	SDF_UCL	STRATUM
316	0	0.05	1	0.97579	.	.	1
317	0	0.05	1	0.97579	.	.	1
318	0	0.05	1	0.97579	.	.	1
319	0	0.05	1	0.97579	.	.	1
320	0	0.05	1	0.97579	.	.	1
321	0	0.05	1	0.97579	.	.	1
322	0	0.05	1	0.97579	.	.	1
323	0	0.05	1	0.97579	.	.	1
324	0	0.05	1	0.97579	.	.	1
325	0	0.05	1	0.97579	.	.	1
326	0	0.05	1	0.97579	.	.	1
327	0	0.05	1	0.97579	.	.	1
328	0	0.05	1	0.97579	.	.	1
329	0	0.05	1	0.97579	.	.	1
330	0	0.05	1	0.97579	.	.	1
331	0	0.05	1	0.97579	.	.	1
332	0	0.05	1	0.97579	.	.	1
333	0	0.05	1	0.97579	.	.	1
334	0	0.05	1	0.97579	.	.	1
335	0	0.05	1	0.97579	.	.	1
336	0	0.06	0	0.97396	0.97247	0.97536	1
337	0	0.06	1	0.97396	.	.	1
338	0	0.06	1	0.97396	.	.	1
339	0	0.06	1	0.97396	.	.	1
340	0	0.06	1	0.97396	.	.	1
341	0	0.06	1	0.97396	.	.	1
342	0	0.06	1	0.97396	.	.	1
343	0	0.06	1	0.97396	.	.	1
344	0	0.06	1	0.97396	.	.	1
345	0	0.06	1	0.97396	.	.	1
346	0	0.06	1	0.97396	.	.	1
347	0	0.06	1	0.97396	.	.	1
348	0	0.06	1	0.97396	.	.	1
349	0	0.06	1	0.97396	.	.	1
350	0	0.06	1	0.97396	.	.	1

Obs	Sub_group	fuptimey	_CENSOR_	SURVIVAL	SDF_LCL	SDF_UCL	STRATUM
**351**	0	0.06	1	0.97396	.	.	1
**352**	0	0.06	1	0.97396	.	.	1
**353**	0	0.06	1	0.97396	.	.	1
**354**	0	0.06	1	0.97396	.	.	1
**355**	0	0.06	1	0.97396	.	.	1
**356**	0	0.06	1	0.97396	.	.	1
**357**	0	0.06	1	0.97396	.	.	1
**358**	0	0.06	1	0.97396	.	.	1
**359**	0	0.06	1	0.97396	.	.	1
**360**	0	0.06	1	0.97396	.	.	1
**361**	0	0.06	1	0.97396	.	.	1
**362**	0	0.06	1	0.97396	.	.	1
**363**	0	0.06	1	0.97396	.	.	1
**364**	0	0.06	1	0.97396	.	.	1
**365**	0	0.06	1	0.97396	.	.	1
**366**	0	0.06	1	0.97396	.	.	1
**367**	0	0.06	1	0.97396	.	.	1
**368**	0	0.06	1	0.97396	.	.	1
**369**	0	0.06	1	0.97396	.	.	1
**370**	0	0.06	1	0.97396	.	.	1
**371**	0	0.06	1	0.97396	.	.	1
**372**	0	0.06	1	0.97396	.	.	1
**373**	0	0.06	1	0.97396	.	.	1
**374**	0	0.06	1	0.97396	.	.	1
**375**	0	0.06	1	0.97396	.	.	1
**376**	0	0.06	1	0.97396	.	.	1
**377**	0	0.06	1	0.97396	.	.	1
**378**	0	0.06	1	0.97396	.	.	1
**379**	0	0.06	1	0.97396	.	.	1
**380**	0	0.06	1	0.97396	.	.	1
**381**	0	0.06	1	0.97396	.	.	1
**382**	0	0.06	1	0.97396	.	.	1
**383**	0	0.06	1	0.97396	.	.	1
**384**	0	0.06	1	0.97396	.	.	1
**385**	0	0.06	1	0.97396	.	.	1

Obs	Sub_group	fuptimey	_CENSOR_	SURVIVAL	SDF_LCL	SDF_UCL	STRATUM
386	0	0.06	1	0.97396	.	.	1
387	0	0.06	1	0.97396	.	.	1
388	0	0.06	1	0.97396	.	.	1
389	0	0.06	1	0.97396	.	.	1
390	0	0.06	1	0.97396	.	.	1
391	0	0.06	1	0.97396	.	.	1
392	0	0.06	1	0.97396	.	.	1
393	0	0.06	1	0.97396	.	.	1
394	0	0.06	1	0.97396	.	.	1
395	0	0.06	1	0.97396	.	.	1
396	0	0.06	1	0.97396	.	.	1
397	0	0.06	1	0.97396	.	.	1
398	0	0.06	1	0.97396	.	.	1
399	0	0.06	1	0.97396	.	.	1
400	0	0.06	1	0.97396	.	.	1
401	0	0.06	1	0.97396	.	.	1
402	0	0.06	1	0.97396	.	.	1
403	0	0.06	1	0.97396	.	.	1
404	0	0.06	1	0.97396	.	.	1
405	0	0.06	1	0.97396	.	.	1
406	0	0.06	1	0.97396	.	.	1
407	0	0.06	1	0.97396	.	.	1
408	0	0.06	1	0.97396	.	.	1
409	0	0.07	0	0.97179	0.97025	0.97326	1
410	0	0.07	1	0.97179	.	.	1
411	0	0.07	1	0.97179	.	.	1
412	0	0.07	1	0.97179	.	.	1
413	0	0.07	1	0.97179	.	.	1
414	0	0.07	1	0.97179	.	.	1
415	0	0.07	1	0.97179	.	.	1
416	0	0.07	1	0.97179	.	.	1
417	0	0.07	1	0.97179	.	.	1
418	0	0.07	1	0.97179	.	.	1
419	0	0.07	1	0.97179	.	.	1
420	0	0.07	1	0.97179	.	.	1

Obs	Sub_group	fuptimey	_CENSOR_	SURVIVAL	SDF_LCL	SDF_UCL	STRATUM
421	0	0.07	1	0.97179	.	.	1
422	0	0.07	1	0.97179	.	.	1
423	0	0.07	1	0.97179	.	.	1
424	0	0.07	1	0.97179	.	.	1
425	0	0.07	1	0.97179	.	.	1
426	0	0.07	1	0.97179	.	.	1
427	0	0.07	1	0.97179	.	.	1
428	0	0.07	1	0.97179	.	.	1
429	0	0.07	1	0.97179	.	.	1
430	0	0.07	1	0.97179	.	.	1
431	0	0.07	1	0.97179	.	.	1
432	0	0.07	1	0.97179	.	.	1
433	0	0.07	1	0.97179	.	.	1
434	0	0.07	1	0.97179	.	.	1
435	0	0.07	1	0.97179	.	.	1
436	0	0.07	1	0.97179	.	.	1
437	0	0.07	1	0.97179	.	.	1
438	0	0.07	1	0.97179	.	.	1
439	0	0.07	1	0.97179	.	.	1
440	0	0.07	1	0.97179	.	.	1
441	0	0.07	1	0.97179	.	.	1
442	0	0.07	1	0.97179	.	.	1
443	0	0.07	1	0.97179	.	.	1
444	0	0.07	1	0.97179	.	.	1
445	0	0.07	1	0.97179	.	.	1
446	0	0.07	1	0.97179	.	.	1
447	0	0.07	1	0.97179	.	.	1
448	0	0.07	1	0.97179	.	.	1
449	0	0.07	1	0.97179	.	.	1
450	0	0.07	1	0.97179	.	.	1
451	0	0.07	1	0.97179	.	.	1
452	0	0.07	1	0.97179	.	.	1
453	0	0.07	1	0.97179	.	.	1
454	0	0.07	1	0.97179	.	.	1
455	0	0.07	1	0.97179	.	.	1

Obs	Sub_group	fuptimey	_CENSOR_	SURVIVAL	SDF_LCL	SDF_UCL	STRATUM
456	0	0.07	1	0.97179	.	.	1
457	0	0.07	1	0.97179	.	.	1
458	0	0.07	1	0.97179	.	.	1
459	0	0.07	1	0.97179	.	.	1
460	0	0.07	1	0.97179	.	.	1
461	0	0.07	1	0.97179	.	.	1
462	0	0.07	1	0.97179	.	.	1
463	0	0.07	1	0.97179	.	.	1
464	0	0.07	1	0.97179	.	.	1
465	0	0.07	1	0.97179	.	.	1
466	0	0.07	1	0.97179	.	.	1
467	0	0.07	1	0.97179	.	.	1
468	0	0.07	1	0.97179	.	.	1
469	0	0.08	0	0.97023	0.96865	0.97174	1
470	0	0.08	1	0.97023	.	.	1
471	0	0.08	1	0.97023	.	.	1
472	0	0.08	1	0.97023	.	.	1
473	0	0.08	1	0.97023	.	.	1
474	0	0.08	1	0.97023	.	.	1
475	0	0.08	1	0.97023	.	.	1
476	0	0.08	1	0.97023	.	.	1
477	0	0.08	1	0.97023	.	.	1
478	0	0.08	1	0.97023	.	.	1
479	0	0.08	1	0.97023	.	.	1
480	0	0.08	1	0.97023	.	.	1
481	0	0.08	1	0.97023	.	.	1
482	0	0.08	1	0.97023	.	.	1
483	0	0.08	1	0.97023	.	.	1
484	0	0.08	1	0.97023	.	.	1
485	0	0.08	1	0.97023	.	.	1
486	0	0.08	1	0.97023	.	.	1
487	0	0.08	1	0.97023	.	.	1
488	0	0.08	1	0.97023	.	.	1
489	0	0.08	1	0.97023	.	.	1
490	0	0.08	1	0.97023	.	.	1

Obs	Sub_group	fuptimey	_CENSOR_	SURVIVAL	SDF_LCL	SDF_UCL	STRATUM
491	0	0.08	1	0.97023	.	.	1
492	0	0.08	1	0.97023	.	.	1
493	0	0.08	1	0.97023	.	.	1
494	0	0.08	1	0.97023	.	.	1
495	0	0.08	1	0.97023	.	.	1
496	0	0.08	1	0.97023	.	.	1
497	0	0.08	1	0.97023	.	.	1
498	0	0.08	1	0.97023	.	.	1
499	0	0.08	1	0.97023	.	.	1
500	0	0.08	1	0.97023	.	.	1
501	0	0.08	1	0.97023	.	.	1
502	0	0.08	1	0.97023	.	.	1
503	0	0.08	1	0.97023	.	.	1
504	0	0.08	1	0.97023	.	.	1
505	0	0.08	1	0.97023	.	.	1
506	0	0.08	1	0.97023	.	.	1
507	0	0.08	1	0.97023	.	.	1
508	0	0.08	1	0.97023	.	.	1
509	0	0.08	1	0.97023	.	.	1
510	0	0.08	1	0.97023	.	.	1
511	0	0.08	1	0.97023	.	.	1
512	0	0.08	1	0.97023	.	.	1
513	0	0.08	1	0.97023	.	.	1
514	0	0.08	1	0.97023	.	.	1
515	0	0.08	1	0.97023	.	.	1
516	0	0.08	1	0.97023	.	.	1
517	0	0.08	1	0.97023	.	.	1
518	0	0.08	1	0.97023	.	.	1
519	0	0.08	1	0.97023	.	.	1
520	0	0.08	1	0.97023	.	.	1
521	0	0.08	1	0.97023	.	.	1
522	0	0.08	1	0.97023	.	.	1
523	0	0.08	1	0.97023	.	.	1
524	0	0.08	1	0.97023	.	.	1
525	0	0.08	1	0.97023	.	.	1

Obs	Sub_group	fuptimey	_CENSOR_	SURVIVAL	SDF_LCL	SDF_UCL	STRATUM
526	0	0.08	1	0.97023	.	.	1
527	0	0.08	1	0.97023	.	.	1
528	0	0.09	0	0.96874	0.96712	0.97028	1
529	0	0.09	1	0.96874	.	.	1
530	0	0.09	1	0.96874	.	.	1
531	0	0.09	1	0.96874	.	.	1
532	0	0.09	1	0.96874	.	.	1
533	0	0.09	1	0.96874	.	.	1
534	0	0.09	1	0.96874	.	.	1
535	0	0.09	1	0.96874	.	.	1
536	0	0.09	1	0.96874	.	.	1
537	0	0.09	1	0.96874	.	.	1
538	0	0.09	1	0.96874	.	.	1
539	0	0.09	1	0.96874	.	.	1
540	0	0.09	1	0.96874	.	.	1
541	0	0.09	1	0.96874	.	.	1
542	0	0.09	1	0.96874	.	.	1
543	0	0.09	1	0.96874	.	.	1
544	0	0.09	1	0.96874	.	.	1
545	0	0.09	1	0.96874	.	.	1
546	0	0.09	1	0.96874	.	.	1
547	0	0.09	1	0.96874	.	.	1
548	0	0.09	1	0.96874	.	.	1
549	0	0.09	1	0.96874	.	.	1
550	0	0.09	1	0.96874	.	.	1
551	0	0.09	1	0.96874	.	.	1
552	0	0.09	1	0.96874	.	.	1
553	0	0.09	1	0.96874	.	.	1
554	0	0.09	1	0.96874	.	.	1
555	0	0.09	1	0.96874	.	.	1
556	0	0.09	1	0.96874	.	.	1
557	0	0.09	1	0.96874	.	.	1
558	0	0.09	1	0.96874	.	.	1
559	0	0.09	1	0.96874	.	.	1
560	0	0.09	1	0.96874	.	.	1

Obs	Sub_group	fuptimey	_CENSOR_	SURVIVAL	SDF_LCL	SDF_UCL	STRATUM
561	0	0.09	1	0.96874	.	.	1
562	0	0.09	1	0.96874	.	.	1
563	0	0.09	1	0.96874	.	.	1
564	0	0.09	1	0.96874	.	.	1
565	0	0.09	1	0.96874	.	.	1
566	0	0.09	1	0.96874	.	.	1
567	0	0.09	1	0.96874	.	.	1
568	0	0.09	1	0.96874	.	.	1
569	0	0.09	1	0.96874	.	.	1
570	0	0.09	1	0.96874	.	.	1
571	0	0.09	1	0.96874	.	.	1
572	0	0.09	1	0.96874	.	.	1
573	0	0.09	1	0.96874	.	.	1
574	0	0.09	1	0.96874	.	.	1
575	0	0.09	1	0.96874	.	.	1
576	0	0.09	1	0.96874	.	.	1
577	0	0.09	1	0.96874	.	.	1
578	0	0.09	1	0.96874	.	.	1
579	0	0.09	1	0.96874	.	.	1
580	0	0.09	1	0.96874	.	.	1
581	0	0.09	1	0.96874	.	.	1
582	0	0.09	1	0.96874	.	.	1
583	0	0.09	1	0.96874	.	.	1
584	0	0.09	1	0.96874	.	.	1
585	0	0.09	1	0.96874	.	.	1
586	0	0.09	1	0.96874	.	.	1
587	0	0.09	1	0.96874	.	.	1
588	0	0.09	1	0.96874	.	.	1
589	0	0.09	1	0.96874	.	.	1
590	0	0.09	1	0.96874	.	.	1
591	0	0.09	1	0.96874	.	.	1
592	0	0.09	1	0.96874	.	.	1
593	0	0.09	1	0.96874	.	.	1
594	0	0.10	0	0.96715	0.96549	0.96874	1
595	0	0.10	1	0.96715	.	.	1

Obs	Sub_group	fuptimey	_CENSOR_	SURVIVAL	SDF_LCL	SDF_UCL	STRATUM
596	0	0.10	1	0.96715	.	.	1
597	0	0.10	1	0.96715	.	.	1
598	0	0.10	1	0.96715	.	.	1
599	0	0.10	1	0.96715	.	.	1
600	0	0.10	1	0.96715	.	.	1
601	0	0.10	1	0.96715	.	.	1
602	0	0.10	1	0.96715	.	.	1
603	0	0.10	1	0.96715	.	.	1
604	0	0.10	1	0.96715	.	.	1
605	0	0.10	1	0.96715	.	.	1
606	0	0.10	1	0.96715	.	.	1
607	0	0.10	1	0.96715	.	.	1
608	0	0.10	1	0.96715	.	.	1
609	0	0.10	1	0.96715	.	.	1
610	0	0.10	1	0.96715	.	.	1
611	0	0.10	1	0.96715	.	.	1
612	0	0.10	1	0.96715	.	.	1
613	0	0.10	1	0.96715	.	.	1
614	0	0.10	1	0.96715	.	.	1
615	0	0.10	1	0.96715	.	.	1
616	0	0.10	1	0.96715	.	.	1
617	0	0.10	1	0.96715	.	.	1
618	0	0.10	1	0.96715	.	.	1
619	0	0.10	1	0.96715	.	.	1
620	0	0.10	1	0.96715	.	.	1
621	0	0.10	1	0.96715	.	.	1
622	0	0.10	1	0.96715	.	.	1
623	0	0.10	1	0.96715	.	.	1
624	0	0.10	1	0.96715	.	.	1
625	0	0.10	1	0.96715	.	.	1
626	0	0.10	1	0.96715	.	.	1
627	0	0.10	1	0.96715	.	.	1
628	0	0.10	1	0.96715	.	.	1
629	0	0.10	1	0.96715	.	.	1
630	0	0.10	1	0.96715	.	.	1

Obs	Sub_group	fuptimey	_CENSOR_	SURVIVAL	SDF_LCL	SDF_UCL	STRATUM
631	0	0.10	1	0.96715	.	.	1
632	0	0.10	1	0.96715	.	.	1
633	0	0.10	1	0.96715	.	.	1
634	0	0.10	1	0.96715	.	.	1
635	0	0.10	1	0.96715	.	.	1
636	0	0.10	1	0.96715	.	.	1
637	0	0.10	1	0.96715	.	.	1
638	0	0.10	1	0.96715	.	.	1
639	0	0.10	1	0.96715	.	.	1
640	0	0.10	1	0.96715	.	.	1
641	0	0.10	1	0.96715	.	.	1
642	0	0.10	1	0.96715	.	.	1
643	0	0.10	1	0.96715	.	.	1
644	0	0.10	1	0.96715	.	.	1
645	0	0.10	1	0.96715	.	.	1
646	0	0.10	1	0.96715	.	.	1
647	0	0.10	1	0.96715	.	.	1
648	0	0.10	1	0.96715	.	.	1
649	0	0.10	1	0.96715	.	.	1
650	0	0.10	1	0.96715	.	.	1
651	0	0.10	1	0.96715	.	.	1
652	0	0.10	1	0.96715	.	.	1
653	0	0.10	1	0.96715	.	.	1
654	0	0.10	1	0.96715	.	.	1
655	0	0.10	1	0.96715	.	.	1
656	0	0.10	1	0.96715	.	.	1
657	0	0.10	1	0.96715	.	.	1
658	0	0.11	0	0.96576	0.96407	0.96738	1
659	0	0.11	1	0.96576	.	.	1
660	0	0.11	1	0.96576	.	.	1
661	0	0.11	1	0.96576	.	.	1
662	0	0.11	1	0.96576	.	.	1
663	0	0.11	1	0.96576	.	.	1
664	0	0.11	1	0.96576	.	.	1
665	0	0.11	1	0.96576	.	.	1

Obs	Sub_group	fuptimey	_CENSOR_	SURVIVAL	SDF_LCL	SDF_UCL	STRATUM
666	0	0.11	1	0.96576	.	.	1
667	0	0.11	1	0.96576	.	.	1
668	0	0.11	1	0.96576	.	.	1
669	0	0.11	1	0.96576	.	.	1
670	0	0.11	1	0.96576	.	.	1
671	0	0.11	1	0.96576	.	.	1
672	0	0.11	1	0.96576	.	.	1
673	0	0.11	1	0.96576	.	.	1
674	0	0.11	1	0.96576	.	.	1
675	0	0.11	1	0.96576	.	.	1
676	0	0.11	1	0.96576	.	.	1
677	0	0.11	1	0.96576	.	.	1
678	0	0.11	1	0.96576	.	.	1
679	0	0.11	1	0.96576	.	.	1
680	0	0.11	1	0.96576	.	.	1
681	0	0.11	1	0.96576	.	.	1
682	0	0.11	1	0.96576	.	.	1
683	0	0.11	1	0.96576	.	.	1
684	0	0.11	1	0.96576	.	.	1
685	0	0.11	1	0.96576	.	.	1
686	0	0.11	1	0.96576	.	.	1
687	0	0.11	1	0.96576	.	.	1
688	0	0.11	1	0.96576	.	.	1
689	0	0.11	1	0.96576	.	.	1
690	0	0.11	1	0.96576	.	.	1
691	0	0.11	1	0.96576	.	.	1
692	0	0.11	1	0.96576	.	.	1
693	0	0.11	1	0.96576	.	.	1
694	0	0.11	1	0.96576	.	.	1
695	0	0.11	1	0.96576	.	.	1
696	0	0.11	1	0.96576	.	.	1
697	0	0.11	1	0.96576	.	.	1
698	0	0.11	1	0.96576	.	.	1
699	0	0.11	1	0.96576	.	.	1
700	0	0.11	1	0.96576	.	.	1

Obs	Sub_group	fuptimey	_CENSOR_	SURVIVAL	SDF_LCL	SDF_UCL	STRATUM
**701**	0	0.11	1	0.96576	.	.	1
**702**	0	0.11	1	0.96576	.	.	1
**703**	0	0.11	1	0.96576	.	.	1
**704**	0	0.11	1	0.96576	.	.	1
**705**	0	0.12	0	0.96474	0.96302	0.96638	1
**706**	0	0.12	1	0.96474	.	.	1
**707**	0	0.12	1	0.96474	.	.	1
**708**	0	0.12	1	0.96474	.	.	1
**709**	0	0.12	1	0.96474	.	.	1
**710**	0	0.12	1	0.96474	.	.	1
**711**	0	0.12	1	0.96474	.	.	1
**712**	0	0.12	1	0.96474	.	.	1
**713**	0	0.12	1	0.96474	.	.	1
**714**	0	0.12	1	0.96474	.	.	1
**715**	0	0.12	1	0.96474	.	.	1
**716**	0	0.12	1	0.96474	.	.	1
**717**	0	0.12	1	0.96474	.	.	1
**718**	0	0.12	1	0.96474	.	.	1
**719**	0	0.12	1	0.96474	.	.	1
**720**	0	0.12	1	0.96474	.	.	1
**721**	0	0.12	1	0.96474	.	.	1
**722**	0	0.12	1	0.96474	.	.	1
**723**	0	0.12	1	0.96474	.	.	1
**724**	0	0.12	1	0.96474	.	.	1
**725**	0	0.12	1	0.96474	.	.	1
**726**	0	0.12	1	0.96474	.	.	1
**727**	0	0.12	1	0.96474	.	.	1
**728**	0	0.12	1	0.96474	.	.	1
**729**	0	0.12	1	0.96474	.	.	1
**730**	0	0.12	1	0.96474	.	.	1
**731**	0	0.12	1	0.96474	.	.	1
**732**	0	0.12	1	0.96474	.	.	1
**733**	0	0.12	1	0.96474	.	.	1
**734**	0	0.12	1	0.96474	.	.	1
**735**	0	0.12	1	0.96474	.	.	1

Obs	Sub_group	fuptimey	_CENSOR_	SURVIVAL	SDF_LCL	SDF_UCL	STRATUM
736	0	0.12	1	0.96474	.	.	1
737	0	0.12	1	0.96474	.	.	1
738	0	0.12	1	0.96474	.	.	1
739	0	0.12	1	0.96474	.	.	1
740	0	0.12	1	0.96474	.	.	1
741	0	0.12	1	0.96474	.	.	1
742	0	0.12	1	0.96474	.	.	1
743	0	0.12	1	0.96474	.	.	1
744	0	0.12	1	0.96474	.	.	1
745	0	0.12	1	0.96474	.	.	1
746	0	0.12	1	0.96474	.	.	1
747	0	0.12	1	0.96474	.	.	1
748	0	0.12	1	0.96474	.	.	1
749	0	0.12	1	0.96474	.	.	1
750	0	0.12	1	0.96474	.	.	1
751	0	0.12	1	0.96474	.	.	1
752	0	0.12	1	0.96474	.	.	1
753	0	0.12	1	0.96474	.	.	1
754	0	0.12	1	0.96474	.	.	1
755	0	0.12	1	0.96474	.	.	1
756	0	0.12	1	0.96474	.	.	1
757	0	0.12	1	0.96474	.	.	1
758	0	0.12	1	0.96474	.	.	1
759	0	0.12	1	0.96474	.	.	1
760	0	0.12	1	0.96474	.	.	1
761	0	0.12	1	0.96474	.	.	1
762	0	0.12	1	0.96474	.	.	1
763	0	0.12	1	0.96474	.	.	1
764	0	0.12	1	0.96474	.	.	1
765	0	0.12	1	0.96474	.	.	1
766	0	0.12	1	0.96474	.	.	1
767	0	0.12	1	0.96474	.	.	1
768	0	0.12	1	0.96474	.	.	1
769	0	0.12	1	0.96474	.	.	1
770	0	0.12	1	0.96474	.	.	1

Obs	Sub_group	fuptimey	_CENSOR_	SURVIVAL	SDF_LCL	SDF_UCL	STRATUM
**771**	0	0.12	1	0.96474	.	.	1
**772**	0	0.12	1	0.96474	.	.	1
**773**	0	0.12	1	0.96474	.	.	1
**774**	0	0.13	0	0.96361	0.96186	0.96527	1
**775**	0	0.13	1	0.96361	.	.	1
**776**	0	0.13	1	0.96361	.	.	1
**777**	0	0.13	1	0.96361	.	.	1
**778**	0	0.13	1	0.96361	.	.	1
**779**	0	0.13	1	0.96361	.	.	1
**780**	0	0.13	1	0.96361	.	.	1
**781**	0	0.13	1	0.96361	.	.	1
**782**	0	0.13	1	0.96361	.	.	1
**783**	0	0.13	1	0.96361	.	.	1
**784**	0	0.13	1	0.96361	.	.	1
**785**	0	0.13	1	0.96361	.	.	1
**786**	0	0.13	1	0.96361	.	.	1
**787**	0	0.13	1	0.96361	.	.	1
**788**	0	0.13	1	0.96361	.	.	1
**789**	0	0.13	1	0.96361	.	.	1
**790**	0	0.13	1	0.96361	.	.	1
**791**	0	0.13	1	0.96361	.	.	1
**792**	0	0.13	1	0.96361	.	.	1
**793**	0	0.13	1	0.96361	.	.	1
**794**	0	0.13	1	0.96361	.	.	1
**795**	0	0.13	1	0.96361	.	.	1
**796**	0	0.13	1	0.96361	.	.	1
**797**	0	0.13	1	0.96361	.	.	1
**798**	0	0.13	1	0.96361	.	.	1
**799**	0	0.13	1	0.96361	.	.	1
**800**	0	0.13	1	0.96361	.	.	1
**801**	0	0.13	1	0.96361	.	.	1
**802**	0	0.13	1	0.96361	.	.	1
**803**	0	0.13	1	0.96361	.	.	1
**804**	0	0.13	1	0.96361	.	.	1
**805**	0	0.13	1	0.96361	.	.	1

Obs	Sub_group	fuptimey	_CENSOR_	SURVIVAL	SDF_LCL	SDF_UCL	STRATUM
806	0	0.13	1	0.96361	.	.	1
807	0	0.13	1	0.96361	.	.	1
808	0	0.13	1	0.96361	.	.	1
809	0	0.13	1	0.96361	.	.	1
810	0	0.13	1	0.96361	.	.	1
811	0	0.13	1	0.96361	.	.	1
812	0	0.13	1	0.96361	.	.	1
813	0	0.13	1	0.96361	.	.	1
814	0	0.13	1	0.96361	.	.	1
815	0	0.13	1	0.96361	.	.	1
816	0	0.13	1	0.96361	.	.	1
817	0	0.13	1	0.96361	.	.	1
818	0	0.13	1	0.96361	.	.	1
819	0	0.13	1	0.96361	.	.	1
820	0	0.13	1	0.96361	.	.	1
821	0	0.13	1	0.96361	.	.	1
822	0	0.13	1	0.96361	.	.	1
823	0	0.14	0	0.96245	0.96068	0.96414	1
824	0	0.14	1	0.96245	.	.	1
825	0	0.14	1	0.96245	.	.	1
826	0	0.14	1	0.96245	.	.	1
827	0	0.14	1	0.96245	.	.	1
828	0	0.14	1	0.96245	.	.	1
829	0	0.14	1	0.96245	.	.	1
830	0	0.14	1	0.96245	.	.	1
831	0	0.14	1	0.96245	.	.	1
832	0	0.14	1	0.96245	.	.	1
833	0	0.14	1	0.96245	.	.	1
834	0	0.14	1	0.96245	.	.	1
835	0	0.14	1	0.96245	.	.	1
836	0	0.14	1	0.96245	.	.	1
837	0	0.14	1	0.96245	.	.	1
838	0	0.14	1	0.96245	.	.	1
839	0	0.14	1	0.96245	.	.	1
840	0	0.14	1	0.96245	.	.	1

Obs	Sub_group	fuptimey	_CENSOR_	SURVIVAL	SDF_LCL	SDF_UCL	STRATUM
841	0	0.14	1	0.96245	.	.	1
842	0	0.14	1	0.96245	.	.	1
843	0	0.14	1	0.96245	.	.	1
844	0	0.14	1	0.96245	.	.	1
845	0	0.14	1	0.96245	.	.	1
846	0	0.14	1	0.96245	.	.	1
847	0	0.14	1	0.96245	.	.	1
848	0	0.14	1	0.96245	.	.	1
849	0	0.14	1	0.96245	.	.	1
850	0	0.14	1	0.96245	.	.	1
851	0	0.14	1	0.96245	.	.	1
852	0	0.14	1	0.96245	.	.	1
853	0	0.14	1	0.96245	.	.	1
854	0	0.14	1	0.96245	.	.	1
855	0	0.14	1	0.96245	.	.	1
856	0	0.14	1	0.96245	.	.	1
857	0	0.14	1	0.96245	.	.	1
858	0	0.14	1	0.96245	.	.	1
859	0	0.14	1	0.96245	.	.	1
860	0	0.14	1	0.96245	.	.	1
861	0	0.14	1	0.96245	.	.	1
862	0	0.14	1	0.96245	.	.	1
863	0	0.14	1	0.96245	.	.	1
864	0	0.14	1	0.96245	.	.	1
865	0	0.14	1	0.96245	.	.	1
866	0	0.14	1	0.96245	.	.	1
867	0	0.14	1	0.96245	.	.	1
868	0	0.14	1	0.96245	.	.	1
869	0	0.14	1	0.96245	.	.	1
870	0	0.14	1	0.96245	.	.	1
871	0	0.14	1	0.96245	.	.	1
872	0	0.14	1	0.96245	.	.	1
873	0	0.14	1	0.96245	.	.	1
874	0	0.14	1	0.96245	.	.	1
875	0	0.14	1	0.96245	.	.	1

Obs	Sub_group	fuptimey	_CENSOR_	SURVIVAL	SDF_LCL	SDF_UCL	STRATUM
**876**	0	0.14	1	0.96245	.	.	1
**877**	0	0.14	1	0.96245	.	.	1
**878**	0	0.14	1	0.96245	.	.	1
**879**	0	0.14	1	0.96245	.	.	1
**880**	0	0.14	1	0.96245	.	.	1
**881**	0	0.14	1	0.96245	.	.	1
**882**	0	0.14	1	0.96245	.	.	1
**883**	0	0.14	1	0.96245	.	.	1
**884**	0	0.14	1	0.96245	.	.	1
**885**	0	0.14	1	0.96245	.	.	1
**886**	0	0.14	1	0.96245	.	.	1
**887**	0	0.14	1	0.96245	.	.	1
**888**	0	0.14	1	0.96245	.	.	1
**889**	0	0.14	1	0.96245	.	.	1
**890**	0	0.15	0	0.96160	0.95981	0.96331	1
**891**	0	0.15	1	0.96160	.	.	1
**892**	0	0.15	1	0.96160	.	.	1
**893**	0	0.15	1	0.96160	.	.	1
**894**	0	0.15	1	0.96160	.	.	1
**895**	0	0.15	1	0.96160	.	.	1
**896**	0	0.15	1	0.96160	.	.	1
**897**	0	0.15	1	0.96160	.	.	1
**898**	0	0.15	1	0.96160	.	.	1
**899**	0	0.15	1	0.96160	.	.	1
**900**	0	0.15	1	0.96160	.	.	1
**901**	0	0.15	1	0.96160	.	.	1
**902**	0	0.15	1	0.96160	.	.	1
**903**	0	0.15	1	0.96160	.	.	1
**904**	0	0.15	1	0.96160	.	.	1
**905**	0	0.15	1	0.96160	.	.	1
**906**	0	0.15	1	0.96160	.	.	1
**907**	0	0.15	1	0.96160	.	.	1
**908**	0	0.15	1	0.96160	.	.	1
**909**	0	0.15	1	0.96160	.	.	1
**910**	0	0.15	1	0.96160	.	.	1

Obs	Sub_group	fuptimey	_CENSOR_	SURVIVAL	SDF_LCL	SDF_UCL	STRATUM
**911**	0	0.15	1	0.96160	.	.	1
**912**	0	0.15	1	0.96160	.	.	1
**913**	0	0.15	1	0.96160	.	.	1
**914**	0	0.15	1	0.96160	.	.	1
**915**	0	0.15	1	0.96160	.	.	1
**916**	0	0.15	1	0.96160	.	.	1
**917**	0	0.15	1	0.96160	.	.	1
**918**	0	0.15	1	0.96160	.	.	1
**919**	0	0.15	1	0.96160	.	.	1
**920**	0	0.15	1	0.96160	.	.	1
**921**	0	0.15	1	0.96160	.	.	1
**922**	0	0.15	1	0.96160	.	.	1
**923**	0	0.15	1	0.96160	.	.	1
**924**	0	0.15	1	0.96160	.	.	1
**925**	0	0.15	1	0.96160	.	.	1
**926**	0	0.15	1	0.96160	.	.	1
**927**	0	0.15	1	0.96160	.	.	1
**928**	0	0.15	1	0.96160	.	.	1
**929**	0	0.15	1	0.96160	.	.	1
**930**	0	0.15	1	0.96160	.	.	1
**931**	0	0.15	1	0.96160	.	.	1
**932**	0	0.15	1	0.96160	.	.	1
**933**	0	0.15	1	0.96160	.	.	1
**934**	0	0.15	1	0.96160	.	.	1
**935**	0	0.15	1	0.96160	.	.	1
**936**	0	0.15	1	0.96160	.	.	1
**937**	0	0.15	1	0.96160	.	.	1
**938**	0	0.15	1	0.96160	.	.	1
**939**	0	0.15	1	0.96160	.	.	1
**940**	0	0.16	0	0.96059	0.95878	0.96232	1
**941**	0	0.16	1	0.96059	.	.	1
**942**	0	0.16	1	0.96059	.	.	1
**943**	0	0.16	1	0.96059	.	.	1
**944**	0	0.16	1	0.96059	.	.	1
**945**	0	0.16	1	0.96059	.	.	1

Obs	Sub_group	fuptimey	_CENSOR_	SURVIVAL	SDF_LCL	SDF_UCL	STRATUM
946	0	0.16	1	0.96059	.	.	1
947	0	0.16	1	0.96059	.	.	1
948	0	0.16	1	0.96059	.	.	1
949	0	0.16	1	0.96059	.	.	1
950	0	0.16	1	0.96059	.	.	1
951	0	0.16	1	0.96059	.	.	1
952	0	0.16	1	0.96059	.	.	1
953	0	0.16	1	0.96059	.	.	1
954	0	0.16	1	0.96059	.	.	1
955	0	0.16	1	0.96059	.	.	1
956	0	0.16	1	0.96059	.	.	1
957	0	0.16	1	0.96059	.	.	1
958	0	0.16	1	0.96059	.	.	1
959	0	0.16	1	0.96059	.	.	1
960	0	0.16	1	0.96059	.	.	1
961	0	0.16	1	0.96059	.	.	1
962	0	0.16	1	0.96059	.	.	1
963	0	0.16	1	0.96059	.	.	1
964	0	0.16	1	0.96059	.	.	1
965	0	0.16	1	0.96059	.	.	1
966	0	0.16	1	0.96059	.	.	1
967	0	0.16	1	0.96059	.	.	1
968	0	0.16	1	0.96059	.	.	1
969	0	0.16	1	0.96059	.	.	1
970	0	0.16	1	0.96059	.	.	1
971	0	0.16	1	0.96059	.	.	1
972	0	0.16	1	0.96059	.	.	1
973	0	0.16	1	0.96059	.	.	1
974	0	0.16	1	0.96059	.	.	1
975	0	0.16	1	0.96059	.	.	1
976	0	0.16	1	0.96059	.	.	1
977	0	0.16	1	0.96059	.	.	1
978	0	0.16	1	0.96059	.	.	1
979	0	0.16	1	0.96059	.	.	1
980	0	0.16	1	0.96059	.	.	1

Obs	Sub_group	fuptimey	_CENSOR_	SURVIVAL	SDF_LCL	SDF_UCL	STRATUM
981	0	0.16	1	0.96059	.	.	1
982	0	0.16	1	0.96059	.	.	1
983	0	0.16	1	0.96059	.	.	1
984	0	0.16	1	0.96059	.	.	1
985	0	0.16	1	0.96059	.	.	1
986	0	0.16	1	0.96059	.	.	1
987	0	0.16	1	0.96059	.	.	1
988	0	0.16	1	0.96059	.	.	1
989	0	0.16	1	0.96059	.	.	1
990	0	0.16	1	0.96059	.	.	1
991	0	0.16	1	0.96059	.	.	1
992	0	0.16	1	0.96059	.	.	1
993	0	0.16	1	0.96059	.	.	1
994	0	0.16	1	0.96059	.	.	1
995	0	0.16	1	0.96059	.	.	1
996	0	0.16	1	0.96059	.	.	1
997	0	0.16	1	0.96059	.	.	1
998	0	0.16	1	0.96059	.	.	1
999	0	0.16	1	0.96059	.	.	1
1000	0	0.17	0	0.95963	0.95779	0.96138	1
1001	0	0.17	1	0.95963	.	.	1
1002	0	0.17	1	0.95963	.	.	1
1003	0	0.17	1	0.95963	.	.	1
1004	0	0.17	1	0.95963	.	.	1
1005	0	0.17	1	0.95963	.	.	1
1006	0	0.17	1	0.95963	.	.	1
1007	0	0.17	1	0.95963	.	.	1
1008	0	0.17	1	0.95963	.	.	1
1009	0	0.17	1	0.95963	.	.	1
1010	0	0.17	1	0.95963	.	.	1
1011	0	0.17	1	0.95963	.	.	1
1012	0	0.17	1	0.95963	.	.	1
1013	0	0.17	1	0.95963	.	.	1
1014	0	0.17	1	0.95963	.	.	1
1015	0	0.17	1	0.95963	.	.	1

Obs	Sub_group	fuptimey	_CENSOR_	SURVIVAL	SDF_LCL	SDF_UCL	STRATUM
1016	0	0.17	1	0.95963	.	.	1
1017	0	0.17	1	0.95963	.	.	1
1018	0	0.17	1	0.95963	.	.	1
1019	0	0.17	1	0.95963	.	.	1
1020	0	0.17	1	0.95963	.	.	1
1021	0	0.17	1	0.95963	.	.	1
1022	0	0.17	1	0.95963	.	.	1
1023	0	0.17	1	0.95963	.	.	1
1024	0	0.17	1	0.95963	.	.	1
1025	0	0.17	1	0.95963	.	.	1
1026	0	0.17	1	0.95963	.	.	1
1027	0	0.17	1	0.95963	.	.	1
1028	0	0.17	1	0.95963	.	.	1
1029	0	0.17	1	0.95963	.	.	1
1030	0	0.17	1	0.95963	.	.	1
1031	0	0.17	1	0.95963	.	.	1
1032	0	0.17	1	0.95963	.	.	1
1033	0	0.17	1	0.95963	.	.	1
1034	0	0.17	1	0.95963	.	.	1
1035	0	0.17	1	0.95963	.	.	1
1036	0	0.17	1	0.95963	.	.	1
1037	0	0.17	1	0.95963	.	.	1
1038	0	0.17	1	0.95963	.	.	1
1039	0	0.17	1	0.95963	.	.	1
1040	0	0.17	1	0.95963	.	.	1
1041	0	0.17	1	0.95963	.	.	1
1042	0	0.17	1	0.95963	.	.	1
1043	0	0.17	1	0.95963	.	.	1
1044	0	0.17	1	0.95963	.	.	1
1045	0	0.17	1	0.95963	.	.	1
1046	0	0.17	1	0.95963	.	.	1
1047	0	0.17	1	0.95963	.	.	1
1048	0	0.17	1	0.95963	.	.	1
1049	0	0.17	1	0.95963	.	.	1
1050	0	0.17	1	0.95963	.	.	1

Obs	Sub_group	fuptimey	_CENSOR_	SURVIVAL	SDF_LCL	SDF_UCL	STRATUM
1051	0	0.17	1	0.95963	.	.	1
1052	0	0.17	1	0.95963	.	.	1
1053	0	0.17	1	0.95963	.	.	1
1054	0	0.17	1	0.95963	.	.	1
1055	0	0.17	1	0.95963	.	.	1
1056	0	0.17	1	0.95963	.	.	1
1057	0	0.18	0	0.95873	0.95688	0.96050	1
1058	0	0.18	1	0.95873	.	.	1
1059	0	0.18	1	0.95873	.	.	1
1060	0	0.18	1	0.95873	.	.	1
1061	0	0.18	1	0.95873	.	.	1
1062	0	0.18	1	0.95873	.	.	1
1063	0	0.18	1	0.95873	.	.	1
1064	0	0.18	1	0.95873	.	.	1
1065	0	0.18	1	0.95873	.	.	1
1066	0	0.18	1	0.95873	.	.	1
1067	0	0.18	1	0.95873	.	.	1
1068	0	0.18	1	0.95873	.	.	1
1069	0	0.18	1	0.95873	.	.	1
1070	0	0.18	1	0.95873	.	.	1
1071	0	0.18	1	0.95873	.	.	1
1072	0	0.18	1	0.95873	.	.	1
1073	0	0.18	1	0.95873	.	.	1
1074	0	0.18	1	0.95873	.	.	1
1075	0	0.18	1	0.95873	.	.	1
1076	0	0.18	1	0.95873	.	.	1
1077	0	0.18	1	0.95873	.	.	1
1078	0	0.18	1	0.95873	.	.	1
1079	0	0.18	1	0.95873	.	.	1
1080	0	0.18	1	0.95873	.	.	1
1081	0	0.18	1	0.95873	.	.	1
1082	0	0.18	1	0.95873	.	.	1
1083	0	0.18	1	0.95873	.	.	1
1084	0	0.18	1	0.95873	.	.	1
1085	0	0.18	1	0.95873	.	.	1

Obs	Sub_group	fuptimey	_CENSOR_	SURVIVAL	SDF_LCL	SDF_UCL	STRATUM
1086	0	0.18	1	0.95873	.	.	1
1087	0	0.18	1	0.95873	.	.	1
1088	0	0.18	1	0.95873	.	.	1
1089	0	0.18	1	0.95873	.	.	1
1090	0	0.18	1	0.95873	.	.	1
1091	0	0.18	1	0.95873	.	.	1
1092	0	0.18	1	0.95873	.	.	1
1093	0	0.18	1	0.95873	.	.	1
1094	0	0.18	1	0.95873	.	.	1
1095	0	0.18	1	0.95873	.	.	1
1096	0	0.18	1	0.95873	.	.	1
1097	0	0.18	1	0.95873	.	.	1
1098	0	0.18	1	0.95873	.	.	1
1099	0	0.18	1	0.95873	.	.	1
1100	0	0.18	1	0.95873	.	.	1
1101	0	0.18	1	0.95873	.	.	1
1102	0	0.18	1	0.95873	.	.	1
1103	0	0.18	1	0.95873	.	.	1
1104	0	0.18	1	0.95873	.	.	1
1105	0	0.18	1	0.95873	.	.	1
1106	0	0.18	1	0.95873	.	.	1
1107	0	0.18	1	0.95873	.	.	1
1108	0	0.18	1	0.95873	.	.	1
1109	0	0.18	1	0.95873	.	.	1
1110	0	0.18	1	0.95873	.	.	1
1111	0	0.18	1	0.95873	.	.	1
1112	0	0.18	1	0.95873	.	.	1
1113	0	0.18	1	0.95873	.	.	1
1114	0	0.18	1	0.95873	.	.	1
1115	0	0.18	1	0.95873	.	.	1
1116	0	0.18	1	0.95873	.	.	1
1117	0	0.18	1	0.95873	.	.	1
1118	0	0.18	1	0.95873	.	.	1
1119	0	0.18	1	0.95873	.	.	1
1120	0	0.19	0	0.95778	0.95591	0.95958	1

Obs	Sub_group	fuptimey	_CENSOR_	SURVIVAL	SDF_LCL	SDF_UCL	STRATUM
1121	0	0.19	1	0.95778	.	.	1
1122	0	0.19	1	0.95778	.	.	1
1123	0	0.19	1	0.95778	.	.	1
1124	0	0.19	1	0.95778	.	.	1
1125	0	0.19	1	0.95778	.	.	1
1126	0	0.19	1	0.95778	.	.	1
1127	0	0.19	1	0.95778	.	.	1
1128	0	0.19	1	0.95778	.	.	1
1129	0	0.19	1	0.95778	.	.	1
1130	0	0.19	1	0.95778	.	.	1
1131	0	0.19	1	0.95778	.	.	1
1132	0	0.19	1	0.95778	.	.	1
1133	0	0.19	1	0.95778	.	.	1
1134	0	0.19	1	0.95778	.	.	1
1135	0	0.19	1	0.95778	.	.	1
1136	0	0.19	1	0.95778	.	.	1
1137	0	0.19	1	0.95778	.	.	1
1138	0	0.19	1	0.95778	.	.	1
1139	0	0.19	1	0.95778	.	.	1
1140	0	0.19	1	0.95778	.	.	1
1141	0	0.19	1	0.95778	.	.	1
1142	0	0.19	1	0.95778	.	.	1
1143	0	0.19	1	0.95778	.	.	1
1144	0	0.19	1	0.95778	.	.	1
1145	0	0.19	1	0.95778	.	.	1
1146	0	0.19	1	0.95778	.	.	1
1147	0	0.19	1	0.95778	.	.	1
1148	0	0.19	1	0.95778	.	.	1
1149	0	0.19	1	0.95778	.	.	1
1150	0	0.19	1	0.95778	.	.	1
1151	0	0.19	1	0.95778	.	.	1
1152	0	0.19	1	0.95778	.	.	1
1153	0	0.19	1	0.95778	.	.	1
1154	0	0.19	1	0.95778	.	.	1
1155	0	0.19	1	0.95778	.	.	1

Obs	Sub_group	fuptimey	_CENSOR_	SURVIVAL	SDF_LCL	SDF_UCL	STRATUM
**1156**	0	0.19	1	0.95778	.	.	1
**1157**	0	0.19	1	0.95778	.	.	1
**1158**	0	0.19	1	0.95778	.	.	1
**1159**	0	0.19	1	0.95778	.	.	1
**1160**	0	0.19	1	0.95778	.	.	1
**1161**	0	0.19	1	0.95778	.	.	1
**1162**	0	0.19	1	0.95778	.	.	1
**1163**	0	0.19	1	0.95778	.	.	1
**1164**	0	0.19	1	0.95778	.	.	1
**1165**	0	0.19	1	0.95778	.	.	1
**1166**	0	0.19	1	0.95778	.	.	1
**1167**	0	0.19	1	0.95778	.	.	1
**1168**	0	0.19	1	0.95778	.	.	1
**1169**	0	0.19	1	0.95778	.	.	1
**1170**	0	0.19	1	0.95778	.	.	1
**1171**	0	0.19	1	0.95778	.	.	1
**1172**	0	0.19	1	0.95778	.	.	1
**1173**	0	0.19	1	0.95778	.	.	1
**1174**	0	0.19	1	0.95778	.	.	1
**1175**	0	0.19	1	0.95778	.	.	1
**1176**	0	0.20	0	0.95675	0.95486	0.95856	1
**1177**	0	0.20	1	0.95675	.	.	1
**1178**	0	0.20	1	0.95675	.	.	1
**1179**	0	0.20	1	0.95675	.	.	1
**1180**	0	0.20	1	0.95675	.	.	1
**1181**	0	0.20	1	0.95675	.	.	1
**1182**	0	0.20	1	0.95675	.	.	1
**1183**	0	0.20	1	0.95675	.	.	1
**1184**	0	0.20	1	0.95675	.	.	1
**1185**	0	0.20	1	0.95675	.	.	1
**1186**	0	0.20	1	0.95675	.	.	1
**1187**	0	0.20	1	0.95675	.	.	1
**1188**	0	0.20	1	0.95675	.	.	1
**1189**	0	0.20	1	0.95675	.	.	1
**1190**	0	0.20	1	0.95675	.	.	1

Obs	Sub_group	fuptimey	_CENSOR_	SURVIVAL	SDF_LCL	SDF_UCL	STRATUM
1191	0	0.20	1	0.95675	.	.	1
1192	0	0.20	1	0.95675	.	.	1
1193	0	0.20	1	0.95675	.	.	1
1194	0	0.20	1	0.95675	.	.	1
1195	0	0.20	1	0.95675	.	.	1
1196	0	0.20	1	0.95675	.	.	1
1197	0	0.20	1	0.95675	.	.	1
1198	0	0.20	1	0.95675	.	.	1
1199	0	0.20	1	0.95675	.	.	1
1200	0	0.20	1	0.95675	.	.	1
1201	0	0.20	1	0.95675	.	.	1
1202	0	0.20	1	0.95675	.	.	1
1203	0	0.20	1	0.95675	.	.	1
1204	0	0.20	1	0.95675	.	.	1
1205	0	0.20	1	0.95675	.	.	1
1206	0	0.20	1	0.95675	.	.	1
1207	0	0.20	1	0.95675	.	.	1
1208	0	0.20	1	0.95675	.	.	1
1209	0	0.20	1	0.95675	.	.	1
1210	0	0.20	1	0.95675	.	.	1
1211	0	0.20	1	0.95675	.	.	1
1212	0	0.20	1	0.95675	.	.	1
1213	0	0.20	1	0.95675	.	.	1
1214	0	0.20	1	0.95675	.	.	1
1215	0	0.20	1	0.95675	.	.	1
1216	0	0.20	1	0.95675	.	.	1
1217	0	0.20	1	0.95675	.	.	1
1218	0	0.20	1	0.95675	.	.	1
1219	0	0.20	1	0.95675	.	.	1
1220	0	0.20	1	0.95675	.	.	1
1221	0	0.20	1	0.95675	.	.	1
1222	0	0.20	1	0.95675	.	.	1
1223	0	0.20	1	0.95675	.	.	1
1224	0	0.20	1	0.95675	.	.	1
1225	0	0.20	1	0.95675	.	.	1

Obs	Sub_group	fuptimey	_CENSOR_	SURVIVAL	SDF_LCL	SDF_UCL	STRATUM
1226	0	0.20	1	0.95675	.	.	1
1227	0	0.20	1	0.95675	.	.	1
1228	0	0.20	1	0.95675	.	.	1
1229	0	0.20	1	0.95675	.	.	1
1230	0	0.20	1	0.95675	.	.	1
1231	0	0.20	1	0.95675	.	.	1
1232	0	0.20	1	0.95675	.	.	1
1233	0	0.20	1	0.95675	.	.	1
1234	0	0.20	1	0.95675	.	.	1
1235	0	0.20	1	0.95675	.	.	1
1236	0	0.20	1	0.95675	.	.	1
1237	0	0.20	1	0.95675	.	.	1
1238	0	0.20	1	0.95675	.	.	1
1239	0	0.20	1	0.95675	.	.	1
1240	0	0.20	1	0.95675	.	.	1
1241	0	0.20	1	0.95675	.	.	1
1242	0	0.20	1	0.95675	.	.	1
1243	0	0.20	1	0.95675	.	.	1
1244	0	0.20	1	0.95675	.	.	1
1245	0	0.20	1	0.95675	.	.	1
1246	0	0.20	1	0.95675	.	.	1
1247	0	0.20	1	0.95675	.	.	1
1248	0	0.20	1	0.95675	.	.	1
1249	0	0.20	1	0.95675	.	.	1
1250	0	0.21	0	0.95598	0.95407	0.95781	1
1251	0	0.21	1	0.95598	.	.	1
1252	0	0.21	1	0.95598	.	.	1
1253	0	0.21	1	0.95598	.	.	1
1254	0	0.21	1	0.95598	.	.	1
1255	0	0.21	1	0.95598	.	.	1
1256	0	0.21	1	0.95598	.	.	1
1257	0	0.21	1	0.95598	.	.	1
1258	0	0.21	1	0.95598	.	.	1
1259	0	0.21	1	0.95598	.	.	1
1260	0	0.21	1	0.95598	.	.	1

Obs	Sub_group	fuptimey	_CENSOR_	SURVIVAL	SDF_LCL	SDF_UCL	STRATUM
1261	0	0.21	1	0.95598	.	.	1
1262	0	0.21	1	0.95598	.	.	1
1263	0	0.21	1	0.95598	.	.	1
1264	0	0.21	1	0.95598	.	.	1
1265	0	0.21	1	0.95598	.	.	1
1266	0	0.21	1	0.95598	.	.	1
1267	0	0.21	1	0.95598	.	.	1
1268	0	0.21	1	0.95598	.	.	1
1269	0	0.21	1	0.95598	.	.	1
1270	0	0.21	1	0.95598	.	.	1
1271	0	0.21	1	0.95598	.	.	1
1272	0	0.21	1	0.95598	.	.	1
1273	0	0.21	1	0.95598	.	.	1
1274	0	0.21	1	0.95598	.	.	1
1275	0	0.21	1	0.95598	.	.	1
1276	0	0.21	1	0.95598	.	.	1
1277	0	0.21	1	0.95598	.	.	1
1278	0	0.21	1	0.95598	.	.	1
1279	0	0.21	1	0.95598	.	.	1
1280	0	0.21	1	0.95598	.	.	1
1281	0	0.21	1	0.95598	.	.	1
1282	0	0.21	1	0.95598	.	.	1
1283	0	0.21	1	0.95598	.	.	1
1284	0	0.21	1	0.95598	.	.	1
1285	0	0.21	1	0.95598	.	.	1
1286	0	0.21	1	0.95598	.	.	1
1287	0	0.21	1	0.95598	.	.	1
1288	0	0.21	1	0.95598	.	.	1
1289	0	0.21	1	0.95598	.	.	1
1290	0	0.21	1	0.95598	.	.	1
1291	0	0.21	1	0.95598	.	.	1
1292	0	0.21	1	0.95598	.	.	1
1293	0	0.21	1	0.95598	.	.	1
1294	0	0.21	1	0.95598	.	.	1
1295	0	0.21	1	0.95598	.	.	1

Obs	Sub_group	fuptimey	_CENSOR_	SURVIVAL	SDF_LCL	SDF_UCL	STRATUM
1296	0	0.21	1	0.95598	.	.	1
1297	0	0.21	1	0.95598	.	.	1
1298	0	0.22	0	0.95483	0.95290	0.95668	1
1299	0	0.22	1	0.95483	.	.	1
1300	0	0.22	1	0.95483	.	.	1
1301	0	0.22	1	0.95483	.	.	1
1302	0	0.22	1	0.95483	.	.	1
1303	0	0.22	1	0.95483	.	.	1
1304	0	0.22	1	0.95483	.	.	1
1305	0	0.22	1	0.95483	.	.	1
1306	0	0.22	1	0.95483	.	.	1
1307	0	0.22	1	0.95483	.	.	1
1308	0	0.22	1	0.95483	.	.	1
1309	0	0.22	1	0.95483	.	.	1
1310	0	0.22	1	0.95483	.	.	1
1311	0	0.22	1	0.95483	.	.	1
1312	0	0.22	1	0.95483	.	.	1
1313	0	0.22	1	0.95483	.	.	1
1314	0	0.22	1	0.95483	.	.	1
1315	0	0.22	1	0.95483	.	.	1
1316	0	0.22	1	0.95483	.	.	1
1317	0	0.22	1	0.95483	.	.	1
1318	0	0.22	1	0.95483	.	.	1
1319	0	0.22	1	0.95483	.	.	1
1320	0	0.22	1	0.95483	.	.	1
1321	0	0.22	1	0.95483	.	.	1
1322	0	0.22	1	0.95483	.	.	1
1323	0	0.22	1	0.95483	.	.	1
1324	0	0.22	1	0.95483	.	.	1
1325	0	0.22	1	0.95483	.	.	1
1326	0	0.22	1	0.95483	.	.	1
1327	0	0.22	1	0.95483	.	.	1
1328	0	0.22	1	0.95483	.	.	1
1329	0	0.22	1	0.95483	.	.	1
1330	0	0.22	1	0.95483	.	.	1

Obs	Sub_group	fuptimey	_CENSOR_	SURVIVAL	SDF_LCL	SDF_UCL	STRATUM
**1331**	0	0.22	1	0.95483	.	.	1
**1332**	0	0.22	1	0.95483	.	.	1
**1333**	0	0.22	1	0.95483	.	.	1
**1334**	0	0.22	1	0.95483	.	.	1
**1335**	0	0.22	1	0.95483	.	.	1
**1336**	0	0.22	1	0.95483	.	.	1
**1337**	0	0.22	1	0.95483	.	.	1
**1338**	0	0.22	1	0.95483	.	.	1
**1339**	0	0.22	1	0.95483	.	.	1
**1340**	0	0.22	1	0.95483	.	.	1
**1341**	0	0.22	1	0.95483	.	.	1
**1342**	0	0.22	1	0.95483	.	.	1
**1343**	0	0.22	1	0.95483	.	.	1
**1344**	0	0.22	1	0.95483	.	.	1
**1345**	0	0.22	1	0.95483	.	.	1
**1346**	0	0.22	1	0.95483	.	.	1
**1347**	0	0.22	1	0.95483	.	.	1
**1348**	0	0.22	1	0.95483	.	.	1
**1349**	0	0.22	1	0.95483	.	.	1
**1350**	0	0.22	1	0.95483	.	.	1
**1351**	0	0.22	1	0.95483	.	.	1
**1352**	0	0.22	1	0.95483	.	.	1
**1353**	0	0.22	1	0.95483	.	.	1
**1354**	0	0.22	1	0.95483	.	.	1
**1355**	0	0.22	1	0.95483	.	.	1
**1356**	0	0.22	1	0.95483	.	.	1
**1357**	0	0.22	1	0.95483	.	.	1
**1358**	0	0.22	1	0.95483	.	.	1
**1359**	0	0.23	0	0.95401	0.95206	0.95588	1
**1360**	0	0.23	1	0.95401	.	.	1
**1361**	0	0.23	1	0.95401	.	.	1
**1362**	0	0.23	1	0.95401	.	.	1
**1363**	0	0.23	1	0.95401	.	.	1
**1364**	0	0.23	1	0.95401	.	.	1
**1365**	0	0.23	1	0.95401	.	.	1

Obs	Sub_group	fuptimey	_CENSOR_	SURVIVAL	SDF_LCL	SDF_UCL	STRATUM
**1366**	0	0.23	1	0.95401	.	.	1
**1367**	0	0.23	1	0.95401	.	.	1
**1368**	0	0.23	1	0.95401	.	.	1
**1369**	0	0.23	1	0.95401	.	.	1
**1370**	0	0.23	1	0.95401	.	.	1
**1371**	0	0.23	1	0.95401	.	.	1
**1372**	0	0.23	1	0.95401	.	.	1
**1373**	0	0.23	1	0.95401	.	.	1
**1374**	0	0.23	1	0.95401	.	.	1
**1375**	0	0.23	1	0.95401	.	.	1
**1376**	0	0.23	1	0.95401	.	.	1
**1377**	0	0.23	1	0.95401	.	.	1
**1378**	0	0.23	1	0.95401	.	.	1
**1379**	0	0.23	1	0.95401	.	.	1
**1380**	0	0.23	1	0.95401	.	.	1
**1381**	0	0.23	1	0.95401	.	.	1
**1382**	0	0.23	1	0.95401	.	.	1
**1383**	0	0.23	1	0.95401	.	.	1
**1384**	0	0.23	1	0.95401	.	.	1
**1385**	0	0.23	1	0.95401	.	.	1
**1386**	0	0.23	1	0.95401	.	.	1
**1387**	0	0.23	1	0.95401	.	.	1
**1388**	0	0.23	1	0.95401	.	.	1
**1389**	0	0.23	1	0.95401	.	.	1
**1390**	0	0.23	1	0.95401	.	.	1
**1391**	0	0.23	1	0.95401	.	.	1
**1392**	0	0.23	1	0.95401	.	.	1
**1393**	0	0.23	1	0.95401	.	.	1
**1394**	0	0.23	1	0.95401	.	.	1
**1395**	0	0.23	1	0.95401	.	.	1
**1396**	0	0.23	1	0.95401	.	.	1
**1397**	0	0.24	0	0.95326	0.95130	0.95515	1
**1398**	0	0.24	1	0.95326	.	.	1
**1399**	0	0.24	1	0.95326	.	.	1
**1400**	0	0.24	1	0.95326	.	.	1

Obs	Sub_group	fuptimey	_CENSOR_	SURVIVAL	SDF_LCL	SDF_UCL	STRATUM
1401	0	0.24	1	0.95326	.	.	1
1402	0	0.24	1	0.95326	.	.	1
1403	0	0.24	1	0.95326	.	.	1
1404	0	0.24	1	0.95326	.	.	1
1405	0	0.24	1	0.95326	.	.	1
1406	0	0.24	1	0.95326	.	.	1
1407	0	0.24	1	0.95326	.	.	1
1408	0	0.24	1	0.95326	.	.	1
1409	0	0.24	1	0.95326	.	.	1
1410	0	0.24	1	0.95326	.	.	1
1411	0	0.24	1	0.95326	.	.	1
1412	0	0.24	1	0.95326	.	.	1
1413	0	0.24	1	0.95326	.	.	1
1414	0	0.24	1	0.95326	.	.	1
1415	0	0.24	1	0.95326	.	.	1
1416	0	0.24	1	0.95326	.	.	1
1417	0	0.24	1	0.95326	.	.	1
1418	0	0.24	1	0.95326	.	.	1
1419	0	0.24	1	0.95326	.	.	1
1420	0	0.24	1	0.95326	.	.	1
1421	0	0.24	1	0.95326	.	.	1
1422	0	0.24	1	0.95326	.	.	1
1423	0	0.24	1	0.95326	.	.	1
1424	0	0.24	1	0.95326	.	.	1
1425	0	0.24	1	0.95326	.	.	1
1426	0	0.24	1	0.95326	.	.	1
1427	0	0.24	1	0.95326	.	.	1
1428	0	0.24	1	0.95326	.	.	1
1429	0	0.24	1	0.95326	.	.	1
1430	0	0.24	1	0.95326	.	.	1
1431	0	0.24	1	0.95326	.	.	1
1432	0	0.24	1	0.95326	.	.	1
1433	0	0.24	1	0.95326	.	.	1
1434	0	0.24	1	0.95326	.	.	1
1435	0	0.24	1	0.95326	.	.	1

Obs	Sub_group	fuptimey	_CENSOR_	SURVIVAL	SDF_LCL	SDF_UCL	STRATUM
**1436**	0	0.24	1	0.95326	.	.	1
**1437**	0	0.24	1	0.95326	.	.	1
**1438**	0	0.24	1	0.95326	.	.	1
**1439**	0	0.24	1	0.95326	.	.	1
**1440**	0	0.24	1	0.95326	.	.	1
**1441**	0	0.24	1	0.95326	.	.	1
**1442**	0	0.24	1	0.95326	.	.	1
**1443**	0	0.24	1	0.95326	.	.	1
**1444**	0	0.24	1	0.95326	.	.	1
**1445**	0	0.24	1	0.95326	.	.	1
**1446**	0	0.24	1	0.95326	.	.	1
**1447**	0	0.24	1	0.95326	.	.	1
**1448**	0	0.24	1	0.95326	.	.	1
**1449**	0	0.24	1	0.95326	.	.	1
**1450**	0	0.24	1	0.95326	.	.	1
**1451**	0	0.24	1	0.95326	.	.	1
**1452**	0	0.24	1	0.95326	.	.	1
**1453**	0	0.24	1	0.95326	.	.	1
**1454**	0	0.24	1	0.95326	.	.	1
**1455**	0	0.24	1	0.95326	.	.	1
**1456**	0	0.24	1	0.95326	.	.	1
**1457**	0	0.24	1	0.95326	.	.	1
**1458**	0	0.25	0	0.95229	0.95030	0.95419	1
**1459**	0	0.25	1	0.95229	.	.	1
**1460**	0	0.25	1	0.95229	.	.	1
**1461**	0	0.25	1	0.95229	.	.	1
**1462**	0	0.25	1	0.95229	.	.	1
**1463**	0	0.25	1	0.95229	.	.	1
**1464**	0	0.25	1	0.95229	.	.	1
**1465**	0	0.25	1	0.95229	.	.	1
**1466**	0	0.25	1	0.95229	.	.	1
**1467**	0	0.25	1	0.95229	.	.	1
**1468**	0	0.25	1	0.95229	.	.	1
**1469**	0	0.25	1	0.95229	.	.	1
**1470**	0	0.25	1	0.95229	.	.	1

Obs	Sub_group	fuptimey	_CENSOR_	SURVIVAL	SDF_LCL	SDF_UCL	STRATUM
**1471**	0	0.25	1	0.95229	.	.	1
**1472**	0	0.25	1	0.95229	.	.	1
**1473**	0	0.25	1	0.95229	.	.	1
**1474**	0	0.25	1	0.95229	.	.	1
**1475**	0	0.25	1	0.95229	.	.	1
**1476**	0	0.25	1	0.95229	.	.	1
**1477**	0	0.25	1	0.95229	.	.	1
**1478**	0	0.25	1	0.95229	.	.	1
**1479**	0	0.25	1	0.95229	.	.	1
**1480**	0	0.25	1	0.95229	.	.	1
**1481**	0	0.25	1	0.95229	.	.	1
**1482**	0	0.25	1	0.95229	.	.	1
**1483**	0	0.25	1	0.95229	.	.	1
**1484**	0	0.25	1	0.95229	.	.	1
**1485**	0	0.25	1	0.95229	.	.	1
**1486**	0	0.25	1	0.95229	.	.	1
**1487**	0	0.25	1	0.95229	.	.	1
**1488**	0	0.25	1	0.95229	.	.	1
**1489**	0	0.25	1	0.95229	.	.	1
**1490**	0	0.25	1	0.95229	.	.	1
**1491**	0	0.25	1	0.95229	.	.	1
**1492**	0	0.25	1	0.95229	.	.	1
**1493**	0	0.25	1	0.95229	.	.	1
**1494**	0	0.25	1	0.95229	.	.	1
**1495**	0	0.25	1	0.95229	.	.	1
**1496**	0	0.25	1	0.95229	.	.	1
**1497**	0	0.25	1	0.95229	.	.	1
**1498**	0	0.25	1	0.95229	.	.	1
**1499**	0	0.25	1	0.95229	.	.	1
**1500**	0	0.25	1	0.95229	.	.	1
**1501**	0	0.26	0	0.95136	0.94935	0.95328	1
**1502**	0	0.26	1	0.95136	.	.	1
**1503**	0	0.26	1	0.95136	.	.	1
**1504**	0	0.26	1	0.95136	.	.	1
**1505**	0	0.26	1	0.95136	.	.	1

Obs	Sub_group	fuptimey	_CENSOR_	SURVIVAL	SDF_LCL	SDF_UCL	STRATUM
1506	0	0.26	1	0.95136	.	.	1
1507	0	0.26	1	0.95136	.	.	1
1508	0	0.26	1	0.95136	.	.	1
1509	0	0.26	1	0.95136	.	.	1
1510	0	0.26	1	0.95136	.	.	1
1511	0	0.26	1	0.95136	.	.	1
1512	0	0.26	1	0.95136	.	.	1
1513	0	0.26	1	0.95136	.	.	1
1514	0	0.26	1	0.95136	.	.	1
1515	0	0.26	1	0.95136	.	.	1
1516	0	0.26	1	0.95136	.	.	1
1517	0	0.26	1	0.95136	.	.	1
1518	0	0.26	1	0.95136	.	.	1
1519	0	0.26	1	0.95136	.	.	1
1520	0	0.26	1	0.95136	.	.	1
1521	0	0.26	1	0.95136	.	.	1
1522	0	0.26	1	0.95136	.	.	1
1523	0	0.26	1	0.95136	.	.	1
1524	0	0.26	1	0.95136	.	.	1
1525	0	0.26	1	0.95136	.	.	1
1526	0	0.26	1	0.95136	.	.	1
1527	0	0.26	1	0.95136	.	.	1
1528	0	0.26	1	0.95136	.	.	1
1529	0	0.26	1	0.95136	.	.	1
1530	0	0.26	1	0.95136	.	.	1
1531	0	0.26	1	0.95136	.	.	1
1532	0	0.26	1	0.95136	.	.	1
1533	0	0.26	1	0.95136	.	.	1
1534	0	0.26	1	0.95136	.	.	1
1535	0	0.26	1	0.95136	.	.	1
1536	0	0.26	1	0.95136	.	.	1
1537	0	0.26	1	0.95136	.	.	1
1538	0	0.26	1	0.95136	.	.	1
1539	0	0.26	1	0.95136	.	.	1
1540	0	0.26	1	0.95136	.	.	1

Obs	Sub_group	fuptimey	_CENSOR_	SURVIVAL	SDF_LCL	SDF_UCL	STRATUM
**1541**	0	0.26	1	0.95136	.	.	1
**1542**	0	0.26	1	0.95136	.	.	1
**1543**	0	0.26	1	0.95136	.	.	1
**1544**	0	0.26	1	0.95136	.	.	1
**1545**	0	0.26	1	0.95136	.	.	1
**1546**	0	0.26	1	0.95136	.	.	1
**1547**	0	0.26	1	0.95136	.	.	1
**1548**	0	0.26	1	0.95136	.	.	1
**1549**	0	0.26	1	0.95136	.	.	1
**1550**	0	0.26	1	0.95136	.	.	1
**1551**	0	0.26	1	0.95136	.	.	1
**1552**	0	0.26	1	0.95136	.	.	1
**1553**	0	0.26	1	0.95136	.	.	1
**1554**	0	0.26	1	0.95136	.	.	1
**1555**	0	0.26	1	0.95136	.	.	1
**1556**	0	0.26	1	0.95136	.	.	1
**1557**	0	0.27	0	0.95096	0.94895	0.95289	1
**1558**	0	0.27	1	0.95096	.	.	1
**1559**	0	0.27	1	0.95096	.	.	1
**1560**	0	0.27	1	0.95096	.	.	1
**1561**	0	0.27	1	0.95096	.	.	1
**1562**	0	0.27	1	0.95096	.	.	1
**1563**	0	0.27	1	0.95096	.	.	1
**1564**	0	0.27	1	0.95096	.	.	1
**1565**	0	0.27	1	0.95096	.	.	1
**1566**	0	0.27	1	0.95096	.	.	1
**1567**	0	0.27	1	0.95096	.	.	1
**1568**	0	0.27	1	0.95096	.	.	1
**1569**	0	0.27	1	0.95096	.	.	1
**1570**	0	0.27	1	0.95096	.	.	1
**1571**	0	0.27	1	0.95096	.	.	1
**1572**	0	0.27	1	0.95096	.	.	1
**1573**	0	0.27	1	0.95096	.	.	1
**1574**	0	0.27	1	0.95096	.	.	1
**1575**	0	0.27	1	0.95096	.	.	1

Obs	Sub_group	fuptimey	_CENSOR_	SURVIVAL	SDF_LCL	SDF_UCL	STRATUM
1576	0	0.27	1	0.95096	.	.	1
1577	0	0.27	1	0.95096	.	.	1
1578	0	0.27	1	0.95096	.	.	1
1579	0	0.27	1	0.95096	.	.	1
1580	0	0.27	1	0.95096	.	.	1
1581	0	0.27	1	0.95096	.	.	1
1582	0	0.27	1	0.95096	.	.	1
1583	0	0.27	1	0.95096	.	.	1
1584	0	0.27	1	0.95096	.	.	1
1585	0	0.27	1	0.95096	.	.	1
1586	0	0.27	1	0.95096	.	.	1
1587	0	0.27	1	0.95096	.	.	1
1588	0	0.27	1	0.95096	.	.	1
1589	0	0.27	1	0.95096	.	.	1
1590	0	0.27	1	0.95096	.	.	1
1591	0	0.27	1	0.95096	.	.	1
1592	0	0.27	1	0.95096	.	.	1
1593	0	0.27	1	0.95096	.	.	1
1594	0	0.27	1	0.95096	.	.	1
1595	0	0.27	1	0.95096	.	.	1
1596	0	0.27	1	0.95096	.	.	1
1597	0	0.27	1	0.95096	.	.	1
1598	0	0.27	1	0.95096	.	.	1
1599	0	0.27	1	0.95096	.	.	1
1600	0	0.27	1	0.95096	.	.	1
1601	0	0.27	1	0.95096	.	.	1
1602	0	0.27	1	0.95096	.	.	1
1603	0	0.27	1	0.95096	.	.	1
1604	0	0.27	1	0.95096	.	.	1
1605	0	0.27	1	0.95096	.	.	1
1606	0	0.27	1	0.95096	.	.	1
1607	0	0.27	1	0.95096	.	.	1
1608	0	0.27	1	0.95096	.	.	1
1609	0	0.27	1	0.95096	.	.	1
1610	0	0.27	1	0.95096	.	.	1

Obs	Sub_group	fuptimey	_CENSOR_	SURVIVAL	SDF_LCL	SDF_UCL	STRATUM
1611	0	0.27	1	0.95096	.	.	1
1612	0	0.27	1	0.95096	.	.	1
1613	0	0.27	1	0.95096	.	.	1
1614	0	0.27	1	0.95096	.	.	1
1615	0	0.28	0	0.95009	0.94806	0.95204	1
1616	0	0.28	1	0.95009	.	.	1
1617	0	0.28	1	0.95009	.	.	1
1618	0	0.28	1	0.95009	.	.	1
1619	0	0.28	1	0.95009	.	.	1
1620	0	0.28	1	0.95009	.	.	1
1621	0	0.28	1	0.95009	.	.	1
1622	0	0.28	1	0.95009	.	.	1
1623	0	0.28	1	0.95009	.	.	1
1624	0	0.28	1	0.95009	.	.	1
1625	0	0.28	1	0.95009	.	.	1
1626	0	0.28	1	0.95009	.	.	1
1627	0	0.28	1	0.95009	.	.	1
1628	0	0.28	1	0.95009	.	.	1
1629	0	0.28	1	0.95009	.	.	1
1630	0	0.28	1	0.95009	.	.	1
1631	0	0.28	1	0.95009	.	.	1
1632	0	0.28	1	0.95009	.	.	1
1633	0	0.28	1	0.95009	.	.	1
1634	0	0.28	1	0.95009	.	.	1
1635	0	0.28	1	0.95009	.	.	1
1636	0	0.28	1	0.95009	.	.	1
1637	0	0.28	1	0.95009	.	.	1
1638	0	0.28	1	0.95009	.	.	1
1639	0	0.28	1	0.95009	.	.	1
1640	0	0.28	1	0.95009	.	.	1
1641	0	0.28	1	0.95009	.	.	1
1642	0	0.28	1	0.95009	.	.	1
1643	0	0.28	1	0.95009	.	.	1
1644	0	0.28	1	0.95009	.	.	1
1645	0	0.28	1	0.95009	.	.	1

Obs	Sub_group	fuptimey	_CENSOR_	SURVIVAL	SDF_LCL	SDF_UCL	STRATUM
**1646**	0	0.28	1	0.95009	.	.	1
**1647**	0	0.28	1	0.95009	.	.	1
**1648**	0	0.28	1	0.95009	.	.	1
**1649**	0	0.28	1	0.95009	.	.	1
**1650**	0	0.28	1	0.95009	.	.	1
**1651**	0	0.28	1	0.95009	.	.	1
**1652**	0	0.28	1	0.95009	.	.	1
**1653**	0	0.28	1	0.95009	.	.	1
**1654**	0	0.28	1	0.95009	.	.	1
**1655**	0	0.28	1	0.95009	.	.	1
**1656**	0	0.28	1	0.95009	.	.	1
**1657**	0	0.28	1	0.95009	.	.	1
**1658**	0	0.28	1	0.95009	.	.	1
**1659**	0	0.28	1	0.95009	.	.	1
**1660**	0	0.28	1	0.95009	.	.	1
**1661**	0	0.28	1	0.95009	.	.	1
**1662**	0	0.28	1	0.95009	.	.	1
**1663**	0	0.28	1	0.95009	.	.	1
**1664**	0	0.28	1	0.95009	.	.	1
**1665**	0	0.29	0	0.94945	0.94741	0.95141	1
**1666**	0	0.29	1	0.94945	.	.	1
**1667**	0	0.29	1	0.94945	.	.	1
**1668**	0	0.29	1	0.94945	.	.	1
**1669**	0	0.29	1	0.94945	.	.	1
**1670**	0	0.29	1	0.94945	.	.	1
**1671**	0	0.29	1	0.94945	.	.	1
**1672**	0	0.29	1	0.94945	.	.	1
**1673**	0	0.29	1	0.94945	.	.	1
**1674**	0	0.29	1	0.94945	.	.	1
**1675**	0	0.29	1	0.94945	.	.	1
**1676**	0	0.29	1	0.94945	.	.	1
**1677**	0	0.29	1	0.94945	.	.	1
**1678**	0	0.29	1	0.94945	.	.	1
**1679**	0	0.29	1	0.94945	.	.	1
**1680**	0	0.29	1	0.94945	.	.	1

Obs	Sub_group	fuptimey	_CENSOR_	SURVIVAL	SDF_LCL	SDF_UCL	STRATUM
1681	0	0.29	1	0.94945	.	.	1
1682	0	0.29	1	0.94945	.	.	1
1683	0	0.29	1	0.94945	.	.	1
1684	0	0.29	1	0.94945	.	.	1
1685	0	0.29	1	0.94945	.	.	1
1686	0	0.29	1	0.94945	.	.	1
1687	0	0.29	1	0.94945	.	.	1
1688	0	0.29	1	0.94945	.	.	1
1689	0	0.29	1	0.94945	.	.	1
1690	0	0.29	1	0.94945	.	.	1
1691	0	0.29	1	0.94945	.	.	1
1692	0	0.29	1	0.94945	.	.	1
1693	0	0.29	1	0.94945	.	.	1
1694	0	0.29	1	0.94945	.	.	1
1695	0	0.29	1	0.94945	.	.	1
1696	0	0.29	1	0.94945	.	.	1
1697	0	0.29	1	0.94945	.	.	1
1698	0	0.29	1	0.94945	.	.	1
1699	0	0.29	1	0.94945	.	.	1
1700	0	0.29	1	0.94945	.	.	1
1701	0	0.29	1	0.94945	.	.	1
1702	0	0.29	1	0.94945	.	.	1
1703	0	0.29	1	0.94945	.	.	1
1704	0	0.29	1	0.94945	.	.	1
1705	0	0.29	1	0.94945	.	.	1
1706	0	0.29	1	0.94945	.	.	1
1707	0	0.29	1	0.94945	.	.	1
1708	0	0.29	1	0.94945	.	.	1
1709	0	0.29	1	0.94945	.	.	1
1710	0	0.29	1	0.94945	.	.	1
1711	0	0.29	1	0.94945	.	.	1
1712	0	0.29	1	0.94945	.	.	1
1713	0	0.29	1	0.94945	.	.	1
1714	0	0.29	1	0.94945	.	.	1
1715	0	0.29	1	0.94945	.	.	1

Obs	Sub_group	fuptimey	_CENSOR_	SURVIVAL	SDF_LCL	SDF_UCL	STRATUM
1716	0	0.29	1	0.94945	.	.	1
1717	0	0.29	1	0.94945	.	.	1
1718	0	0.29	1	0.94945	.	.	1
1719	0	0.29	1	0.94945	.	.	1
1720	0	0.29	1	0.94945	.	.	1
1721	0	0.29	1	0.94945	.	.	1
1722	0	0.29	1	0.94945	.	.	1
1723	0	0.29	1	0.94945	.	.	1
1724	0	0.30	0	0.94873	0.94668	0.95071	1
1725	0	0.30	1	0.94873	.	.	1
1726	0	0.30	1	0.94873	.	.	1
1727	0	0.30	1	0.94873	.	.	1
1728	0	0.30	1	0.94873	.	.	1
1729	0	0.30	1	0.94873	.	.	1
1730	0	0.30	1	0.94873	.	.	1
1731	0	0.30	1	0.94873	.	.	1
1732	0	0.30	1	0.94873	.	.	1
1733	0	0.30	1	0.94873	.	.	1
1734	0	0.30	1	0.94873	.	.	1
1735	0	0.30	1	0.94873	.	.	1
1736	0	0.30	1	0.94873	.	.	1
1737	0	0.30	1	0.94873	.	.	1
1738	0	0.30	1	0.94873	.	.	1
1739	0	0.30	1	0.94873	.	.	1
1740	0	0.30	1	0.94873	.	.	1
1741	0	0.30	1	0.94873	.	.	1
1742	0	0.30	1	0.94873	.	.	1
1743	0	0.30	1	0.94873	.	.	1
1744	0	0.30	1	0.94873	.	.	1
1745	0	0.30	1	0.94873	.	.	1
1746	0	0.30	1	0.94873	.	.	1
1747	0	0.30	1	0.94873	.	.	1
1748	0	0.30	1	0.94873	.	.	1
1749	0	0.30	1	0.94873	.	.	1
1750	0	0.30	1	0.94873	.	.	1

Obs	Sub_group	fuptimey	_CENSOR_	SURVIVAL	SDF_LCL	SDF_UCL	STRATUM
**1751**	0	0.30	1	0.94873	.	.	1
**1752**	0	0.30	1	0.94873	.	.	1
**1753**	0	0.30	1	0.94873	.	.	1
**1754**	0	0.30	1	0.94873	.	.	1
**1755**	0	0.30	1	0.94873	.	.	1
**1756**	0	0.30	1	0.94873	.	.	1
**1757**	0	0.30	1	0.94873	.	.	1
**1758**	0	0.30	1	0.94873	.	.	1
**1759**	0	0.30	1	0.94873	.	.	1
**1760**	0	0.30	1	0.94873	.	.	1
**1761**	0	0.30	1	0.94873	.	.	1
**1762**	0	0.30	1	0.94873	.	.	1
**1763**	0	0.30	1	0.94873	.	.	1
**1764**	0	0.30	1	0.94873	.	.	1
**1765**	0	0.30	1	0.94873	.	.	1
**1766**	0	0.30	1	0.94873	.	.	1
**1767**	0	0.30	1	0.94873	.	.	1
**1768**	0	0.30	1	0.94873	.	.	1
**1769**	0	0.30	1	0.94873	.	.	1
**1770**	0	0.31	0	0.94789	0.94582	0.94988	1
**1771**	0	0.31	1	0.94789	.	.	1
**1772**	0	0.31	1	0.94789	.	.	1
**1773**	0	0.31	1	0.94789	.	.	1
**1774**	0	0.31	1	0.94789	.	.	1
**1775**	0	0.31	1	0.94789	.	.	1
**1776**	0	0.31	1	0.94789	.	.	1
**1777**	0	0.31	1	0.94789	.	.	1
**1778**	0	0.31	1	0.94789	.	.	1
**1779**	0	0.31	1	0.94789	.	.	1
**1780**	0	0.31	1	0.94789	.	.	1
**1781**	0	0.31	1	0.94789	.	.	1
**1782**	0	0.31	1	0.94789	.	.	1
**1783**	0	0.31	1	0.94789	.	.	1
**1784**	0	0.31	1	0.94789	.	.	1
**1785**	0	0.31	1	0.94789	.	.	1

Obs	Sub_group	fuptimey	_CENSOR_	SURVIVAL	SDF_LCL	SDF_UCL	STRATUM
1786	0	0.31	1	0.94789	.	.	1
1787	0	0.31	1	0.94789	.	.	1
1788	0	0.31	1	0.94789	.	.	1
1789	0	0.31	1	0.94789	.	.	1
1790	0	0.31	1	0.94789	.	.	1
1791	0	0.31	1	0.94789	.	.	1
1792	0	0.31	1	0.94789	.	.	1
1793	0	0.31	1	0.94789	.	.	1
1794	0	0.31	1	0.94789	.	.	1
1795	0	0.31	1	0.94789	.	.	1
1796	0	0.31	1	0.94789	.	.	1
1797	0	0.31	1	0.94789	.	.	1
1798	0	0.31	1	0.94789	.	.	1
1799	0	0.31	1	0.94789	.	.	1
1800	0	0.31	1	0.94789	.	.	1
1801	0	0.31	1	0.94789	.	.	1
1802	0	0.31	1	0.94789	.	.	1
1803	0	0.31	1	0.94789	.	.	1
1804	0	0.31	1	0.94789	.	.	1
1805	0	0.31	1	0.94789	.	.	1
1806	0	0.31	1	0.94789	.	.	1
1807	0	0.31	1	0.94789	.	.	1
1808	0	0.31	1	0.94789	.	.	1
1809	0	0.31	1	0.94789	.	.	1
1810	0	0.31	1	0.94789	.	.	1
1811	0	0.31	1	0.94789	.	.	1
1812	0	0.31	1	0.94789	.	.	1
1813	0	0.31	1	0.94789	.	.	1
1814	0	0.31	1	0.94789	.	.	1
1815	0	0.31	1	0.94789	.	.	1
1816	0	0.31	1	0.94789	.	.	1
1817	0	0.31	1	0.94789	.	.	1
1818	0	0.31	1	0.94789	.	.	1
1819	0	0.31	1	0.94789	.	.	1
1820	0	0.31	1	0.94789	.	.	1

Obs	Sub_group	fuptimey	_CENSOR_	SURVIVAL	SDF_LCL	SDF_UCL	STRATUM
1821	0	0.31	1	0.94789	.	.	1
1822	0	0.31	1	0.94789	.	.	1
1823	0	0.31	1	0.94789	.	.	1
1824	0	0.31	1	0.94789	.	.	1
1825	0	0.31	1	0.94789	.	.	1
1826	0	0.31	1	0.94789	.	.	1
1827	0	0.32	0	0.94726	0.94518	0.94926	1
1828	0	0.32	1	0.94726	.	.	1
1829	0	0.32	1	0.94726	.	.	1
1830	0	0.32	1	0.94726	.	.	1
1831	0	0.32	1	0.94726	.	.	1
1832	0	0.32	1	0.94726	.	.	1
1833	0	0.32	1	0.94726	.	.	1
1834	0	0.32	1	0.94726	.	.	1
1835	0	0.32	1	0.94726	.	.	1
1836	0	0.32	1	0.94726	.	.	1
1837	0	0.32	1	0.94726	.	.	1
1838	0	0.32	1	0.94726	.	.	1
1839	0	0.32	1	0.94726	.	.	1
1840	0	0.32	1	0.94726	.	.	1
1841	0	0.32	1	0.94726	.	.	1
1842	0	0.32	1	0.94726	.	.	1
1843	0	0.32	1	0.94726	.	.	1
1844	0	0.32	1	0.94726	.	.	1
1845	0	0.32	1	0.94726	.	.	1
1846	0	0.32	1	0.94726	.	.	1
1847	0	0.32	1	0.94726	.	.	1
1848	0	0.32	1	0.94726	.	.	1
1849	0	0.32	1	0.94726	.	.	1
1850	0	0.32	1	0.94726	.	.	1
1851	0	0.32	1	0.94726	.	.	1
1852	0	0.32	1	0.94726	.	.	1
1853	0	0.32	1	0.94726	.	.	1
1854	0	0.32	1	0.94726	.	.	1
1855	0	0.32	1	0.94726	.	.	1

Obs	Sub_group	fuptimey	_CENSOR_	SURVIVAL	SDF_LCL	SDF_UCL	STRATUM
**1856**	0	0.32	1	0.94726	.	.	1
**1857**	0	0.32	1	0.94726	.	.	1
**1858**	0	0.32	1	0.94726	.	.	1
**1859**	0	0.32	1	0.94726	.	.	1
**1860**	0	0.32	1	0.94726	.	.	1
**1861**	0	0.32	1	0.94726	.	.	1
**1862**	0	0.33	0	0.94641	0.94432	0.94843	1
**1863**	0	0.33	1	0.94641	.	.	1
**1864**	0	0.33	1	0.94641	.	.	1
**1865**	0	0.33	1	0.94641	.	.	1
**1866**	0	0.33	1	0.94641	.	.	1
**1867**	0	0.33	1	0.94641	.	.	1
**1868**	0	0.33	1	0.94641	.	.	1
**1869**	0	0.33	1	0.94641	.	.	1
**1870**	0	0.33	1	0.94641	.	.	1
**1871**	0	0.33	1	0.94641	.	.	1
**1872**	0	0.33	1	0.94641	.	.	1
**1873**	0	0.33	1	0.94641	.	.	1
**1874**	0	0.33	1	0.94641	.	.	1
**1875**	0	0.33	1	0.94641	.	.	1
**1876**	0	0.33	1	0.94641	.	.	1
**1877**	0	0.33	1	0.94641	.	.	1
**1878**	0	0.33	1	0.94641	.	.	1
**1879**	0	0.33	1	0.94641	.	.	1
**1880**	0	0.33	1	0.94641	.	.	1
**1881**	0	0.33	1	0.94641	.	.	1
**1882**	0	0.33	1	0.94641	.	.	1
**1883**	0	0.33	1	0.94641	.	.	1
**1884**	0	0.33	1	0.94641	.	.	1
**1885**	0	0.33	1	0.94641	.	.	1
**1886**	0	0.33	1	0.94641	.	.	1
**1887**	0	0.33	1	0.94641	.	.	1
**1888**	0	0.33	1	0.94641	.	.	1
**1889**	0	0.33	1	0.94641	.	.	1
**1890**	0	0.33	1	0.94641	.	.	1

Obs	Sub_group	fuptimey	_CENSOR_	SURVIVAL	SDF_LCL	SDF_UCL	STRATUM
**1891**	0	0.33	1	0.94641	.	.	1
**1892**	0	0.33	1	0.94641	.	.	1
**1893**	0	0.33	1	0.94641	.	.	1
**1894**	0	0.33	1	0.94641	.	.	1
**1895**	0	0.33	1	0.94641	.	.	1
**1896**	0	0.33	1	0.94641	.	.	1
**1897**	0	0.33	1	0.94641	.	.	1
**1898**	0	0.33	1	0.94641	.	.	1
**1899**	0	0.33	1	0.94641	.	.	1
**1900**	0	0.33	1	0.94641	.	.	1
**1901**	0	0.33	1	0.94641	.	.	1
**1902**	0	0.33	1	0.94641	.	.	1
**1903**	0	0.33	1	0.94641	.	.	1
**1904**	0	0.33	1	0.94641	.	.	1
**1905**	0	0.33	1	0.94641	.	.	1
**1906**	0	0.33	1	0.94641	.	.	1
**1907**	0	0.33	1	0.94641	.	.	1
**1908**	0	0.33	1	0.94641	.	.	1
**1909**	0	0.33	1	0.94641	.	.	1
**1910**	0	0.33	1	0.94641	.	.	1
**1911**	0	0.33	1	0.94641	.	.	1
**1912**	0	0.33	1	0.94641	.	.	1
**1913**	0	0.33	1	0.94641	.	.	1
**1914**	0	0.33	1	0.94641	.	.	1
**1915**	0	0.33	1	0.94641	.	.	1
**1916**	0	0.33	1	0.94641	.	.	1
**1917**	0	0.33	1	0.94641	.	.	1
**1918**	0	0.33	1	0.94641	.	.	1
**1919**	0	0.33	1	0.94641	.	.	1
**1920**	0	0.33	1	0.94641	.	.	1
**1921**	0	0.33	1	0.94641	.	.	1
**1922**	0	0.33	1	0.94641	.	.	1
**1923**	0	0.33	1	0.94641	.	.	1
**1924**	0	0.33	1	0.94641	.	.	1
**1925**	0	0.33	1	0.94641	.	.	1

Obs	Sub_group	fuptimey	_CENSOR_	SURVIVAL	SDF_LCL	SDF_UCL	STRATUM
1926	0	0.33	1	0.94641	.	.	1
1927	0	0.33	1	0.94641	.	.	1
1928	0	0.33	1	0.94641	.	.	1
1929	0	0.33	1	0.94641	.	.	1
1930	0	0.34	0	0.94574	0.94363	0.94777	1
1931	0	0.34	1	0.94574	.	.	1
1932	0	0.34	1	0.94574	.	.	1
1933	0	0.34	1	0.94574	.	.	1
1934	0	0.34	1	0.94574	.	.	1
1935	0	0.34	1	0.94574	.	.	1
1936	0	0.34	1	0.94574	.	.	1
1937	0	0.34	1	0.94574	.	.	1
1938	0	0.34	1	0.94574	.	.	1
1939	0	0.34	1	0.94574	.	.	1
1940	0	0.34	1	0.94574	.	.	1
1941	0	0.34	1	0.94574	.	.	1
1942	0	0.34	1	0.94574	.	.	1
1943	0	0.34	1	0.94574	.	.	1
1944	0	0.34	1	0.94574	.	.	1
1945	0	0.34	1	0.94574	.	.	1
1946	0	0.34	1	0.94574	.	.	1
1947	0	0.34	1	0.94574	.	.	1
1948	0	0.34	1	0.94574	.	.	1
1949	0	0.34	1	0.94574	.	.	1
1950	0	0.34	1	0.94574	.	.	1
1951	0	0.34	1	0.94574	.	.	1
1952	0	0.34	1	0.94574	.	.	1
1953	0	0.34	1	0.94574	.	.	1
1954	0	0.34	1	0.94574	.	.	1
1955	0	0.34	1	0.94574	.	.	1
1956	0	0.34	1	0.94574	.	.	1
1957	0	0.34	1	0.94574	.	.	1
1958	0	0.34	1	0.94574	.	.	1
1959	0	0.34	1	0.94574	.	.	1
1960	0	0.34	1	0.94574	.	.	1

Obs	Sub_group	fuptimey	_CENSOR_	SURVIVAL	SDF_LCL	SDF_UCL	STRATUM
**1961**	0	0.34	1	0.94574	.	.	1
**1962**	0	0.34	1	0.94574	.	.	1
**1963**	0	0.34	1	0.94574	.	.	1
**1964**	0	0.34	1	0.94574	.	.	1
**1965**	0	0.34	1	0.94574	.	.	1
**1966**	0	0.34	1	0.94574	.	.	1
**1967**	0	0.34	1	0.94574	.	.	1
**1968**	0	0.34	1	0.94574	.	.	1
**1969**	0	0.34	1	0.94574	.	.	1
**1970**	0	0.34	1	0.94574	.	.	1
**1971**	0	0.34	1	0.94574	.	.	1
**1972**	0	0.34	1	0.94574	.	.	1
**1973**	0	0.34	1	0.94574	.	.	1
**1974**	0	0.34	1	0.94574	.	.	1
**1975**	0	0.34	1	0.94574	.	.	1
**1976**	0	0.34	1	0.94574	.	.	1
**1977**	0	0.34	1	0.94574	.	.	1
**1978**	0	0.34	1	0.94574	.	.	1
**1979**	0	0.34	1	0.94574	.	.	1
**1980**	0	0.34	1	0.94574	.	.	1
**1981**	0	0.34	1	0.94574	.	.	1
**1982**	0	0.34	1	0.94574	.	.	1
**1983**	0	0.34	1	0.94574	.	.	1
**1984**	0	0.34	1	0.94574	.	.	1
**1985**	0	0.35	0	0.94491	0.94279	0.94696	1
**1986**	0	0.35	1	0.94491	.	.	1
**1987**	0	0.35	1	0.94491	.	.	1
**1988**	0	0.35	1	0.94491	.	.	1
**1989**	0	0.35	1	0.94491	.	.	1
**1990**	0	0.35	1	0.94491	.	.	1
**1991**	0	0.35	1	0.94491	.	.	1
**1992**	0	0.35	1	0.94491	.	.	1
**1993**	0	0.35	1	0.94491	.	.	1
**1994**	0	0.35	1	0.94491	.	.	1
**1995**	0	0.35	1	0.94491	.	.	1

Obs	Sub_group	fuptimey	_CENSOR_	SURVIVAL	SDF_LCL	SDF_UCL	STRATUM
1996	0	0.35	1	0.94491	.	.	1
1997	0	0.35	1	0.94491	.	.	1
1998	0	0.35	1	0.94491	.	.	1
1999	0	0.35	1	0.94491	.	.	1
2000	0	0.35	1	0.94491	.	.	1
2001	0	0.35	1	0.94491	.	.	1
2002	0	0.35	1	0.94491	.	.	1
2003	0	0.35	1	0.94491	.	.	1
2004	0	0.35	1	0.94491	.	.	1
2005	0	0.35	1	0.94491	.	.	1
2006	0	0.35	1	0.94491	.	.	1
2007	0	0.35	1	0.94491	.	.	1
2008	0	0.35	1	0.94491	.	.	1
2009	0	0.35	1	0.94491	.	.	1
2010	0	0.35	1	0.94491	.	.	1
2011	0	0.35	1	0.94491	.	.	1
2012	0	0.35	1	0.94491	.	.	1
2013	0	0.35	1	0.94491	.	.	1
2014	0	0.35	1	0.94491	.	.	1
2015	0	0.35	1	0.94491	.	.	1
2016	0	0.35	1	0.94491	.	.	1
2017	0	0.35	1	0.94491	.	.	1
2018	0	0.35	1	0.94491	.	.	1
2019	0	0.35	1	0.94491	.	.	1
2020	0	0.35	1	0.94491	.	.	1
2021	0	0.35	1	0.94491	.	.	1
2022	0	0.35	1	0.94491	.	.	1
2023	0	0.35	1	0.94491	.	.	1
2024	0	0.35	1	0.94491	.	.	1
2025	0	0.35	1	0.94491	.	.	1
2026	0	0.35	1	0.94491	.	.	1
2027	0	0.35	1	0.94491	.	.	1
2028	0	0.35	1	0.94491	.	.	1
2029	0	0.35	1	0.94491	.	.	1
2030	0	0.35	1	0.94491	.	.	1

Obs	Sub_group	fuptimey	_CENSOR_	SURVIVAL	SDF_LCL	SDF_UCL	STRATUM
**2031**	0	0.35	1	0.94491	.	.	1
**2032**	0	0.35	1	0.94491	.	.	1
**2033**	0	0.35	1	0.94491	.	.	1
**2034**	0	0.35	1	0.94491	.	.	1
**2035**	0	0.35	1	0.94491	.	.	1
**2036**	0	0.35	1	0.94491	.	.	1
**2037**	0	0.35	1	0.94491	.	.	1
**2038**	0	0.35	1	0.94491	.	.	1
**2039**	0	0.35	1	0.94491	.	.	1
**2040**	0	0.35	1	0.94491	.	.	1
**2041**	0	0.36	0	0.94437	0.94224	0.94643	1
**2042**	0	0.36	1	0.94437	.	.	1
**2043**	0	0.36	1	0.94437	.	.	1
**2044**	0	0.36	1	0.94437	.	.	1
**2045**	0	0.36	1	0.94437	.	.	1
**2046**	0	0.36	1	0.94437	.	.	1
**2047**	0	0.36	1	0.94437	.	.	1
**2048**	0	0.36	1	0.94437	.	.	1
**2049**	0	0.36	1	0.94437	.	.	1
**2050**	0	0.36	1	0.94437	.	.	1
**2051**	0	0.36	1	0.94437	.	.	1
**2052**	0	0.36	1	0.94437	.	.	1
**2053**	0	0.36	1	0.94437	.	.	1
**2054**	0	0.36	1	0.94437	.	.	1
**2055**	0	0.36	1	0.94437	.	.	1
**2056**	0	0.36	1	0.94437	.	.	1
**2057**	0	0.36	1	0.94437	.	.	1
**2058**	0	0.36	1	0.94437	.	.	1
**2059**	0	0.36	1	0.94437	.	.	1
**2060**	0	0.36	1	0.94437	.	.	1
**2061**	0	0.36	1	0.94437	.	.	1
**2062**	0	0.36	1	0.94437	.	.	1
**2063**	0	0.36	1	0.94437	.	.	1
**2064**	0	0.36	1	0.94437	.	.	1
**2065**	0	0.36	1	0.94437	.	.	1

Obs	Sub_group	fuptimey	_CENSOR_	SURVIVAL	SDF_LCL	SDF_UCL	STRATUM
2066	0	0.36	1	0.94437	.	.	1
2067	0	0.36	1	0.94437	.	.	1
2068	0	0.36	1	0.94437	.	.	1
2069	0	0.36	1	0.94437	.	.	1
2070	0	0.36	1	0.94437	.	.	1
2071	0	0.36	1	0.94437	.	.	1
2072	0	0.36	1	0.94437	.	.	1
2073	0	0.36	1	0.94437	.	.	1
2074	0	0.36	1	0.94437	.	.	1
2075	0	0.36	1	0.94437	.	.	1
2076	0	0.36	1	0.94437	.	.	1
2077	0	0.36	1	0.94437	.	.	1
2078	0	0.36	1	0.94437	.	.	1
2079	0	0.36	1	0.94437	.	.	1
2080	0	0.36	1	0.94437	.	.	1
2081	0	0.36	1	0.94437	.	.	1
2082	0	0.36	1	0.94437	.	.	1
2083	0	0.36	1	0.94437	.	.	1
2084	0	0.36	1	0.94437	.	.	1
2085	0	0.36	1	0.94437	.	.	1
2086	0	0.36	1	0.94437	.	.	1
2087	0	0.36	1	0.94437	.	.	1
2088	0	0.36	1	0.94437	.	.	1
2089	0	0.36	1	0.94437	.	.	1
2090	0	0.36	1	0.94437	.	.	1
2091	0	0.36	1	0.94437	.	.	1
2092	0	0.36	1	0.94437	.	.	1
2093	0	0.37	0	0.94377	0.94162	0.94583	1
2094	0	0.37	1	0.94377	.	.	1
2095	0	0.37	1	0.94377	.	.	1
2096	0	0.37	1	0.94377	.	.	1
2097	0	0.37	1	0.94377	.	.	1
2098	0	0.37	1	0.94377	.	.	1
2099	0	0.37	1	0.94377	.	.	1
2100	0	0.37	1	0.94377	.	.	1

Obs	Sub_group	fuptimey	_CENSOR_	SURVIVAL	SDF_LCL	SDF_UCL	STRATUM
2101	0	0.37	1	0.94377	.	.	1
2102	0	0.37	1	0.94377	.	.	1
2103	0	0.37	1	0.94377	.	.	1
2104	0	0.37	1	0.94377	.	.	1
2105	0	0.37	1	0.94377	.	.	1
2106	0	0.37	1	0.94377	.	.	1
2107	0	0.37	1	0.94377	.	.	1
2108	0	0.37	1	0.94377	.	.	1
2109	0	0.37	1	0.94377	.	.	1
2110	0	0.37	1	0.94377	.	.	1
2111	0	0.37	1	0.94377	.	.	1
2112	0	0.37	1	0.94377	.	.	1
2113	0	0.37	1	0.94377	.	.	1
2114	0	0.37	1	0.94377	.	.	1
2115	0	0.37	1	0.94377	.	.	1
2116	0	0.37	1	0.94377	.	.	1
2117	0	0.37	1	0.94377	.	.	1
2118	0	0.37	1	0.94377	.	.	1
2119	0	0.37	1	0.94377	.	.	1
2120	0	0.37	1	0.94377	.	.	1
2121	0	0.37	1	0.94377	.	.	1
2122	0	0.37	1	0.94377	.	.	1
2123	0	0.37	1	0.94377	.	.	1
2124	0	0.37	1	0.94377	.	.	1
2125	0	0.37	1	0.94377	.	.	1
2126	0	0.37	1	0.94377	.	.	1
2127	0	0.37	1	0.94377	.	.	1
2128	0	0.37	1	0.94377	.	.	1
2129	0	0.37	1	0.94377	.	.	1
2130	0	0.37	1	0.94377	.	.	1
2131	0	0.37	1	0.94377	.	.	1
2132	0	0.37	1	0.94377	.	.	1
2133	0	0.37	1	0.94377	.	.	1
2134	0	0.37	1	0.94377	.	.	1
2135	0	0.37	1	0.94377	.	.	1

Obs	Sub_group	fuptimey	_CENSOR_	SURVIVAL	SDF_LCL	SDF_UCL	STRATUM
2136	0	0.37	1	0.94377	.	.	1
2137	0	0.37	1	0.94377	.	.	1
2138	0	0.37	1	0.94377	.	.	1
2139	0	0.37	1	0.94377	.	.	1
2140	0	0.37	1	0.94377	.	.	1
2141	0	0.37	1	0.94377	.	.	1
2142	0	0.37	1	0.94377	.	.	1
2143	0	0.37	1	0.94377	.	.	1
2144	0	0.37	1	0.94377	.	.	1
2145	0	0.37	1	0.94377	.	.	1
2146	0	0.37	1	0.94377	.	.	1
2147	0	0.37	1	0.94377	.	.	1
2148	0	0.37	1	0.94377	.	.	1
2149	0	0.37	1	0.94377	.	.	1
2150	0	0.37	1	0.94377	.	.	1
2151	0	0.37	1	0.94377	.	.	1
2152	0	0.37	1	0.94377	.	.	1
2153	0	0.37	1	0.94377	.	.	1
2154	0	0.37	1	0.94377	.	.	1
2155	0	0.37	1	0.94377	.	.	1
2156	0	0.37	1	0.94377	.	.	1
2157	0	0.37	1	0.94377	.	.	1
2158	0	0.37	1	0.94377	.	.	1
2159	0	0.37	1	0.94377	.	.	1
2160	0	0.37	1	0.94377	.	.	1
2161	0	0.38	0	0.94307	0.94091	0.94515	1
2162	0	0.38	1	0.94307	.	.	1
2163	0	0.38	1	0.94307	.	.	1
2164	0	0.38	1	0.94307	.	.	1
2165	0	0.38	1	0.94307	.	.	1
2166	0	0.38	1	0.94307	.	.	1
2167	0	0.38	1	0.94307	.	.	1
2168	0	0.38	1	0.94307	.	.	1
2169	0	0.38	1	0.94307	.	.	1
2170	0	0.38	1	0.94307	.	.	1

Obs	Sub_group	fuptimey	_CENSOR_	SURVIVAL	SDF_LCL	SDF_UCL	STRATUM
2171	0	0.38	1	0.94307	.	.	1
2172	0	0.38	1	0.94307	.	.	1
2173	0	0.38	1	0.94307	.	.	1
2174	0	0.38	1	0.94307	.	.	1
2175	0	0.38	1	0.94307	.	.	1
2176	0	0.38	1	0.94307	.	.	1
2177	0	0.38	1	0.94307	.	.	1
2178	0	0.38	1	0.94307	.	.	1
2179	0	0.38	1	0.94307	.	.	1
2180	0	0.38	1	0.94307	.	.	1
2181	0	0.38	1	0.94307	.	.	1
2182	0	0.38	1	0.94307	.	.	1
2183	0	0.38	1	0.94307	.	.	1
2184	0	0.38	1	0.94307	.	.	1
2185	0	0.38	1	0.94307	.	.	1
2186	0	0.38	1	0.94307	.	.	1
2187	0	0.38	1	0.94307	.	.	1
2188	0	0.38	1	0.94307	.	.	1
2189	0	0.38	1	0.94307	.	.	1
2190	0	0.38	1	0.94307	.	.	1
2191	0	0.38	1	0.94307	.	.	1
2192	0	0.38	1	0.94307	.	.	1
2193	0	0.38	1	0.94307	.	.	1
2194	0	0.38	1	0.94307	.	.	1
2195	0	0.38	1	0.94307	.	.	1
2196	0	0.38	1	0.94307	.	.	1
2197	0	0.38	1	0.94307	.	.	1
2198	0	0.38	1	0.94307	.	.	1
2199	0	0.38	1	0.94307	.	.	1
2200	0	0.38	1	0.94307	.	.	1
2201	0	0.38	1	0.94307	.	.	1
2202	0	0.38	1	0.94307	.	.	1
2203	0	0.38	1	0.94307	.	.	1
2204	0	0.38	1	0.94307	.	.	1
2205	0	0.38	1	0.94307	.	.	1

Obs	Sub_group	fuptimey	_CENSOR_	SURVIVAL	SDF_LCL	SDF_UCL	STRATUM
2206	0	0.38	1	0.94307	.	.	1
2207	0	0.38	1	0.94307	.	.	1
2208	0	0.38	1	0.94307	.	.	1
2209	0	0.38	1	0.94307	.	.	1
2210	0	0.38	1	0.94307	.	.	1
2211	0	0.38	1	0.94307	.	.	1
2212	0	0.38	1	0.94307	.	.	1
2213	0	0.39	0	0.94275	0.94059	0.94484	1
2214	0	0.39	1	0.94275	.	.	1
2215	0	0.39	1	0.94275	.	.	1
2216	0	0.39	1	0.94275	.	.	1
2217	0	0.39	1	0.94275	.	.	1
2218	0	0.39	1	0.94275	.	.	1
2219	0	0.39	1	0.94275	.	.	1
2220	0	0.39	1	0.94275	.	.	1
2221	0	0.39	1	0.94275	.	.	1
2222	0	0.39	1	0.94275	.	.	1
2223	0	0.39	1	0.94275	.	.	1
2224	0	0.39	1	0.94275	.	.	1
2225	0	0.39	1	0.94275	.	.	1
2226	0	0.39	1	0.94275	.	.	1
2227	0	0.39	1	0.94275	.	.	1
2228	0	0.39	1	0.94275	.	.	1
2229	0	0.39	1	0.94275	.	.	1
2230	0	0.39	1	0.94275	.	.	1
2231	0	0.39	1	0.94275	.	.	1
2232	0	0.39	1	0.94275	.	.	1
2233	0	0.39	1	0.94275	.	.	1
2234	0	0.39	1	0.94275	.	.	1
2235	0	0.39	1	0.94275	.	.	1
2236	0	0.39	1	0.94275	.	.	1
2237	0	0.39	1	0.94275	.	.	1
2238	0	0.39	1	0.94275	.	.	1
2239	0	0.39	1	0.94275	.	.	1
2240	0	0.39	1	0.94275	.	.	1

Obs	Sub_group	fuptimey	_CENSOR_	SURVIVAL	SDF_LCL	SDF_UCL	STRATUM
2241	0	0.39	1	0.94275	.	.	1
2242	0	0.39	1	0.94275	.	.	1
2243	0	0.39	1	0.94275	.	.	1
2244	0	0.39	1	0.94275	.	.	1
2245	0	0.39	1	0.94275	.	.	1
2246	0	0.39	1	0.94275	.	.	1
2247	0	0.39	1	0.94275	.	.	1
2248	0	0.39	1	0.94275	.	.	1
2249	0	0.39	1	0.94275	.	.	1
2250	0	0.39	1	0.94275	.	.	1
2251	0	0.39	1	0.94275	.	.	1
2252	0	0.39	1	0.94275	.	.	1
2253	0	0.39	1	0.94275	.	.	1
2254	0	0.39	1	0.94275	.	.	1
2255	0	0.39	1	0.94275	.	.	1
2256	0	0.39	1	0.94275	.	.	1
2257	0	0.39	1	0.94275	.	.	1
2258	0	0.39	1	0.94275	.	.	1
2259	0	0.39	1	0.94275	.	.	1
2260	0	0.39	1	0.94275	.	.	1
2261	0	0.39	1	0.94275	.	.	1
2262	0	0.39	1	0.94275	.	.	1
2263	0	0.39	1	0.94275	.	.	1
2264	0	0.39	1	0.94275	.	.	1
2265	0	0.39	1	0.94275	.	.	1
2266	0	0.39	1	0.94275	.	.	1
2267	0	0.39	1	0.94275	.	.	1
2268	0	0.39	1	0.94275	.	.	1
2269	0	0.39	1	0.94275	.	.	1
2270	0	0.39	1	0.94275	.	.	1
2271	0	0.39	1	0.94275	.	.	1
2272	0	0.39	1	0.94275	.	.	1
2273	0	0.39	1	0.94275	.	.	1
2274	0	0.39	1	0.94275	.	.	1
2275	0	0.39	1	0.94275	.	.	1

Obs	Sub_group	fuptimey	_CENSOR_	SURVIVAL	SDF_LCL	SDF_UCL	STRATUM
**2276**	0	0.40	0	0.94208	0.93990	0.94418	1
**2277**	0	0.40	1	0.94208	.	.	1
**2278**	0	0.40	1	0.94208	.	.	1
**2279**	0	0.40	1	0.94208	.	.	1
**2280**	0	0.40	1	0.94208	.	.	1
**2281**	0	0.40	1	0.94208	.	.	1
**2282**	0	0.40	1	0.94208	.	.	1
**2283**	0	0.40	1	0.94208	.	.	1
**2284**	0	0.40	1	0.94208	.	.	1
**2285**	0	0.40	1	0.94208	.	.	1
**2286**	0	0.40	1	0.94208	.	.	1
**2287**	0	0.40	1	0.94208	.	.	1
**2288**	0	0.40	1	0.94208	.	.	1
**2289**	0	0.40	1	0.94208	.	.	1
**2290**	0	0.40	1	0.94208	.	.	1
**2291**	0	0.40	1	0.94208	.	.	1
**2292**	0	0.40	1	0.94208	.	.	1
**2293**	0	0.40	1	0.94208	.	.	1
**2294**	0	0.40	1	0.94208	.	.	1
**2295**	0	0.40	1	0.94208	.	.	1
**2296**	0	0.40	1	0.94208	.	.	1
**2297**	0	0.40	1	0.94208	.	.	1
**2298**	0	0.40	1	0.94208	.	.	1
**2299**	0	0.40	1	0.94208	.	.	1
**2300**	0	0.40	1	0.94208	.	.	1
**2301**	0	0.40	1	0.94208	.	.	1
**2302**	0	0.40	1	0.94208	.	.	1
**2303**	0	0.40	1	0.94208	.	.	1
**2304**	0	0.40	1	0.94208	.	.	1
**2305**	0	0.40	1	0.94208	.	.	1
**2306**	0	0.40	1	0.94208	.	.	1
**2307**	0	0.40	1	0.94208	.	.	1
**2308**	0	0.40	1	0.94208	.	.	1
**2309**	0	0.40	1	0.94208	.	.	1
**2310**	0	0.40	1	0.94208	.	.	1

Obs	Sub_group	fuptimey	_CENSOR_	SURVIVAL	SDF_LCL	SDF_UCL	STRATUM
2311	0	0.40	1	0.94208	.	.	1
2312	0	0.40	1	0.94208	.	.	1
2313	0	0.40	1	0.94208	.	.	1
2314	0	0.40	1	0.94208	.	.	1
2315	0	0.40	1	0.94208	.	.	1
2316	0	0.40	1	0.94208	.	.	1
2317	0	0.40	1	0.94208	.	.	1
2318	0	0.40	1	0.94208	.	.	1
2319	0	0.40	1	0.94208	.	.	1
2320	0	0.40	1	0.94208	.	.	1
2321	0	0.40	1	0.94208	.	.	1
2322	0	0.40	1	0.94208	.	.	1
2323	0	0.40	1	0.94208	.	.	1
2324	0	0.40	1	0.94208	.	.	1
2325	0	0.40	1	0.94208	.	.	1
2326	0	0.40	1	0.94208	.	.	1
2327	0	0.40	1	0.94208	.	.	1
2328	0	0.40	1	0.94208	.	.	1
2329	0	0.40	1	0.94208	.	.	1
2330	0	0.40	1	0.94208	.	.	1
2331	0	0.40	1	0.94208	.	.	1
2332	0	0.40	1	0.94208	.	.	1
2333	0	0.40	1	0.94208	.	.	1
2334	0	0.40	1	0.94208	.	.	1
2335	0	0.40	1	0.94208	.	.	1
2336	0	0.40	1	0.94208	.	.	1
2337	0	0.40	1	0.94208	.	.	1
2338	0	0.40	1	0.94208	.	.	1
2339	0	0.41	0	0.94133	0.93914	0.94344	1
2340	0	0.41	1	0.94133	.	.	1
2341	0	0.41	1	0.94133	.	.	1
2342	0	0.41	1	0.94133	.	.	1
2343	0	0.41	1	0.94133	.	.	1
2344	0	0.41	1	0.94133	.	.	1
2345	0	0.41	1	0.94133	.	.	1

Obs	Sub_group	fuptimey	_CENSOR_	SURVIVAL	SDF_LCL	SDF_UCL	STRATUM
2346	0	0.41	1	0.94133	.	.	1
2347	0	0.41	1	0.94133	.	.	1
2348	0	0.41	1	0.94133	.	.	1
2349	0	0.41	1	0.94133	.	.	1
2350	0	0.41	1	0.94133	.	.	1
2351	0	0.41	1	0.94133	.	.	1
2352	0	0.41	1	0.94133	.	.	1
2353	0	0.41	1	0.94133	.	.	1
2354	0	0.41	1	0.94133	.	.	1
2355	0	0.41	1	0.94133	.	.	1
2356	0	0.41	1	0.94133	.	.	1
2357	0	0.41	1	0.94133	.	.	1
2358	0	0.41	1	0.94133	.	.	1
2359	0	0.41	1	0.94133	.	.	1
2360	0	0.41	1	0.94133	.	.	1
2361	0	0.41	1	0.94133	.	.	1
2362	0	0.41	1	0.94133	.	.	1
2363	0	0.41	1	0.94133	.	.	1
2364	0	0.41	1	0.94133	.	.	1
2365	0	0.41	1	0.94133	.	.	1
2366	0	0.41	1	0.94133	.	.	1
2367	0	0.41	1	0.94133	.	.	1
2368	0	0.41	1	0.94133	.	.	1
2369	0	0.41	1	0.94133	.	.	1
2370	0	0.41	1	0.94133	.	.	1
2371	0	0.41	1	0.94133	.	.	1
2372	0	0.41	1	0.94133	.	.	1
2373	0	0.41	1	0.94133	.	.	1
2374	0	0.41	1	0.94133	.	.	1
2375	0	0.41	1	0.94133	.	.	1
2376	0	0.41	1	0.94133	.	.	1
2377	0	0.41	1	0.94133	.	.	1
2378	0	0.41	1	0.94133	.	.	1
2379	0	0.41	1	0.94133	.	.	1
2380	0	0.41	1	0.94133	.	.	1

Obs	Sub_group	fuptimey	_CENSOR_	SURVIVAL	SDF_LCL	SDF_UCL	STRATUM
2381	0	0.41	1	0.94133	.	.	1
2382	0	0.41	1	0.94133	.	.	1
2383	0	0.41	1	0.94133	.	.	1
2384	0	0.41	1	0.94133	.	.	1
2385	0	0.41	1	0.94133	.	.	1
2386	0	0.41	1	0.94133	.	.	1
2387	0	0.41	1	0.94133	.	.	1
2388	0	0.41	1	0.94133	.	.	1
2389	0	0.41	1	0.94133	.	.	1
2390	0	0.41	1	0.94133	.	.	1
2391	0	0.41	1	0.94133	.	.	1
2392	0	0.41	1	0.94133	.	.	1
2393	0	0.41	1	0.94133	.	.	1
2394	0	0.41	1	0.94133	.	.	1
2395	0	0.41	1	0.94133	.	.	1
2396	0	0.41	1	0.94133	.	.	1
2397	0	0.41	1	0.94133	.	.	1
2398	0	0.41	1	0.94133	.	.	1
2399	0	0.41	1	0.94133	.	.	1
2400	0	0.41	1	0.94133	.	.	1
2401	0	0.41	1	0.94133	.	.	1
2402	0	0.41	1	0.94133	.	.	1
2403	0	0.41	1	0.94133	.	.	1
2404	0	0.41	1	0.94133	.	.	1
2405	0	0.41	1	0.94133	.	.	1
2406	0	0.41	1	0.94133	.	.	1
2407	0	0.41	1	0.94133	.	.	1
2408	0	0.42	0	0.94072	0.93852	0.94284	1
2409	0	0.42	1	0.94072	.	.	1
2410	0	0.42	1	0.94072	.	.	1
2411	0	0.42	1	0.94072	.	.	1
2412	0	0.42	1	0.94072	.	.	1
2413	0	0.42	1	0.94072	.	.	1
2414	0	0.42	1	0.94072	.	.	1
2415	0	0.42	1	0.94072	.	.	1

Obs	Sub_group	fuptimey	_CENSOR_	SURVIVAL	SDF_LCL	SDF_UCL	STRATUM
**2416**	0	0.42	1	0.94072	.	.	1
**2417**	0	0.42	1	0.94072	.	.	1
**2418**	0	0.42	1	0.94072	.	.	1
**2419**	0	0.42	1	0.94072	.	.	1
**2420**	0	0.42	1	0.94072	.	.	1
**2421**	0	0.42	1	0.94072	.	.	1
**2422**	0	0.42	1	0.94072	.	.	1
**2423**	0	0.42	1	0.94072	.	.	1
**2424**	0	0.42	1	0.94072	.	.	1
**2425**	0	0.42	1	0.94072	.	.	1
**2426**	0	0.42	1	0.94072	.	.	1
**2427**	0	0.42	1	0.94072	.	.	1
**2428**	0	0.42	1	0.94072	.	.	1
**2429**	0	0.42	1	0.94072	.	.	1
**2430**	0	0.42	1	0.94072	.	.	1
**2431**	0	0.42	1	0.94072	.	.	1
**2432**	0	0.42	1	0.94072	.	.	1
**2433**	0	0.42	1	0.94072	.	.	1
**2434**	0	0.42	1	0.94072	.	.	1
**2435**	0	0.42	1	0.94072	.	.	1
**2436**	0	0.42	1	0.94072	.	.	1
**2437**	0	0.42	1	0.94072	.	.	1
**2438**	0	0.42	1	0.94072	.	.	1
**2439**	0	0.42	1	0.94072	.	.	1
**2440**	0	0.42	1	0.94072	.	.	1
**2441**	0	0.42	1	0.94072	.	.	1
**2442**	0	0.42	1	0.94072	.	.	1
**2443**	0	0.42	1	0.94072	.	.	1
**2444**	0	0.42	1	0.94072	.	.	1
**2445**	0	0.42	1	0.94072	.	.	1
**2446**	0	0.42	1	0.94072	.	.	1
**2447**	0	0.42	1	0.94072	.	.	1
**2448**	0	0.42	1	0.94072	.	.	1
**2449**	0	0.42	1	0.94072	.	.	1
**2450**	0	0.43	0	0.94009	0.93787	0.94222	1

Obs	Sub_group	fuptimey	_CENSOR_	SURVIVAL	SDF_LCL	SDF_UCL	STRATUM
2451	0	0.43	1	0.94009	.	.	1
2452	0	0.43	1	0.94009	.	.	1
2453	0	0.43	1	0.94009	.	.	1
2454	0	0.43	1	0.94009	.	.	1
2455	0	0.43	1	0.94009	.	.	1
2456	0	0.43	1	0.94009	.	.	1
2457	0	0.43	1	0.94009	.	.	1
2458	0	0.43	1	0.94009	.	.	1
2459	0	0.43	1	0.94009	.	.	1
2460	0	0.43	1	0.94009	.	.	1
2461	0	0.43	1	0.94009	.	.	1
2462	0	0.43	1	0.94009	.	.	1
2463	0	0.43	1	0.94009	.	.	1
2464	0	0.43	1	0.94009	.	.	1
2465	0	0.43	1	0.94009	.	.	1
2466	0	0.43	1	0.94009	.	.	1
2467	0	0.43	1	0.94009	.	.	1
2468	0	0.43	1	0.94009	.	.	1
2469	0	0.43	1	0.94009	.	.	1
2470	0	0.43	1	0.94009	.	.	1
2471	0	0.43	1	0.94009	.	.	1
2472	0	0.43	1	0.94009	.	.	1
2473	0	0.43	1	0.94009	.	.	1
2474	0	0.43	1	0.94009	.	.	1
2475	0	0.43	1	0.94009	.	.	1
2476	0	0.43	1	0.94009	.	.	1
2477	0	0.43	1	0.94009	.	.	1
2478	0	0.43	1	0.94009	.	.	1
2479	0	0.43	1	0.94009	.	.	1
2480	0	0.43	1	0.94009	.	.	1
2481	0	0.43	1	0.94009	.	.	1
2482	0	0.43	1	0.94009	.	.	1
2483	0	0.43	1	0.94009	.	.	1
2484	0	0.43	1	0.94009	.	.	1
2485	0	0.43	1	0.94009	.	.	1

Obs	Sub_group	fuptimey	_CENSOR_	SURVIVAL	SDF_LCL	SDF_UCL	STRATUM
**2486**	0	0.43	1	0.94009	.	.	1
**2487**	0	0.43	1	0.94009	.	.	1
**2488**	0	0.43	1	0.94009	.	.	1
**2489**	0	0.43	1	0.94009	.	.	1
**2490**	0	0.43	1	0.94009	.	.	1
**2491**	0	0.43	1	0.94009	.	.	1
**2492**	0	0.43	1	0.94009	.	.	1
**2493**	0	0.43	1	0.94009	.	.	1
**2494**	0	0.43	1	0.94009	.	.	1
**2495**	0	0.43	1	0.94009	.	.	1
**2496**	0	0.43	1	0.94009	.	.	1
**2497**	0	0.43	1	0.94009	.	.	1
**2498**	0	0.43	1	0.94009	.	.	1
**2499**	0	0.43	1	0.94009	.	.	1
**2500**	0	0.43	1	0.94009	.	.	1
**2501**	0	0.43	1	0.94009	.	.	1
**2502**	0	0.43	1	0.94009	.	.	1
**2503**	0	0.43	1	0.94009	.	.	1
**2504**	0	0.43	1	0.94009	.	.	1
**2505**	0	0.43	1	0.94009	.	.	1
**2506**	0	0.43	1	0.94009	.	.	1
**2507**	0	0.43	1	0.94009	.	.	1
**2508**	0	0.43	1	0.94009	.	.	1
**2509**	0	0.43	1	0.94009	.	.	1
**2510**	0	0.43	1	0.94009	.	.	1
**2511**	0	0.43	1	0.94009	.	.	1
**2512**	0	0.43	1	0.94009	.	.	1
**2513**	0	0.43	1	0.94009	.	.	1
**2514**	0	0.43	1	0.94009	.	.	1
**2515**	0	0.43	1	0.94009	.	.	1
**2516**	0	0.43	1	0.94009	.	.	1
**2517**	0	0.43	1	0.94009	.	.	1
**2518**	0	0.43	1	0.94009	.	.	1
**2519**	0	0.44	0	0.93945	0.93723	0.94160	1
**2520**	0	0.44	1	0.93945	.	.	1

Obs	Sub_group	fuptimey	_CENSOR_	SURVIVAL	SDF_LCL	SDF_UCL	STRATUM
2521	0	0.44	1	0.93945	.	.	1
2522	0	0.44	1	0.93945	.	.	1
2523	0	0.44	1	0.93945	.	.	1
2524	0	0.44	1	0.93945	.	.	1
2525	0	0.44	1	0.93945	.	.	1
2526	0	0.44	1	0.93945	.	.	1
2527	0	0.44	1	0.93945	.	.	1
2528	0	0.44	1	0.93945	.	.	1
2529	0	0.44	1	0.93945	.	.	1
2530	0	0.44	1	0.93945	.	.	1
2531	0	0.44	1	0.93945	.	.	1
2532	0	0.44	1	0.93945	.	.	1
2533	0	0.44	1	0.93945	.	.	1
2534	0	0.44	1	0.93945	.	.	1
2535	0	0.44	1	0.93945	.	.	1
2536	0	0.44	1	0.93945	.	.	1
2537	0	0.44	1	0.93945	.	.	1
2538	0	0.44	1	0.93945	.	.	1
2539	0	0.44	1	0.93945	.	.	1
2540	0	0.44	1	0.93945	.	.	1
2541	0	0.44	1	0.93945	.	.	1
2542	0	0.44	1	0.93945	.	.	1
2543	0	0.44	1	0.93945	.	.	1
2544	0	0.44	1	0.93945	.	.	1
2545	0	0.44	1	0.93945	.	.	1
2546	0	0.44	1	0.93945	.	.	1
2547	0	0.44	1	0.93945	.	.	1
2548	0	0.44	1	0.93945	.	.	1
2549	0	0.44	1	0.93945	.	.	1
2550	0	0.44	1	0.93945	.	.	1
2551	0	0.44	1	0.93945	.	.	1
2552	0	0.44	1	0.93945	.	.	1
2553	0	0.45	0	0.93902	0.93679	0.94117	1
2554	0	0.45	1	0.93902	.	.	1
2555	0	0.45	1	0.93902	.	.	1

Obs	Sub_group	fuptimey	_CENSOR_	SURVIVAL	SDF_LCL	SDF_UCL	STRATUM
2556	0	0.45	1	0.93902	.	.	1
2557	0	0.45	1	0.93902	.	.	1
2558	0	0.45	1	0.93902	.	.	1
2559	0	0.45	1	0.93902	.	.	1
2560	0	0.45	1	0.93902	.	.	1
2561	0	0.45	1	0.93902	.	.	1
2562	0	0.45	1	0.93902	.	.	1
2563	0	0.45	1	0.93902	.	.	1
2564	0	0.45	1	0.93902	.	.	1
2565	0	0.45	1	0.93902	.	.	1
2566	0	0.45	1	0.93902	.	.	1
2567	0	0.45	1	0.93902	.	.	1
2568	0	0.45	1	0.93902	.	.	1
2569	0	0.45	1	0.93902	.	.	1
2570	0	0.45	1	0.93902	.	.	1
2571	0	0.45	1	0.93902	.	.	1
2572	0	0.45	1	0.93902	.	.	1
2573	0	0.45	1	0.93902	.	.	1
2574	0	0.45	1	0.93902	.	.	1
2575	0	0.45	1	0.93902	.	.	1
2576	0	0.45	1	0.93902	.	.	1
2577	0	0.45	1	0.93902	.	.	1
2578	0	0.45	1	0.93902	.	.	1
2579	0	0.45	1	0.93902	.	.	1
2580	0	0.45	1	0.93902	.	.	1
2581	0	0.45	1	0.93902	.	.	1
2582	0	0.45	1	0.93902	.	.	1
2583	0	0.45	1	0.93902	.	.	1
2584	0	0.45	1	0.93902	.	.	1
2585	0	0.45	1	0.93902	.	.	1
2586	0	0.45	1	0.93902	.	.	1
2587	0	0.45	1	0.93902	.	.	1
2588	0	0.45	1	0.93902	.	.	1
2589	0	0.45	1	0.93902	.	.	1
2590	0	0.45	1	0.93902	.	.	1

Obs	Sub_group	fuptimey	_CENSOR_	SURVIVAL	SDF_LCL	SDF_UCL	STRATUM
**2591**	0	0.45	1	0.93902	.	.	1
**2592**	0	0.45	1	0.93902	.	.	1
**2593**	0	0.45	1	0.93902	.	.	1
**2594**	0	0.45	1	0.93902	.	.	1
**2595**	0	0.45	1	0.93902	.	.	1
**2596**	0	0.45	1	0.93902	.	.	1
**2597**	0	0.45	1	0.93902	.	.	1
**2598**	0	0.45	1	0.93902	.	.	1
**2599**	0	0.45	1	0.93902	.	.	1
**2600**	0	0.45	1	0.93902	.	.	1
**2601**	0	0.45	1	0.93902	.	.	1
**2602**	0	0.45	1	0.93902	.	.	1
**2603**	0	0.45	1	0.93902	.	.	1
**2604**	0	0.45	1	0.93902	.	.	1
**2605**	0	0.45	1	0.93902	.	.	1
**2606**	0	0.45	1	0.93902	.	.	1
**2607**	0	0.45	1	0.93902	.	.	1
**2608**	0	0.45	1	0.93902	.	.	1
**2609**	0	0.45	1	0.93902	.	.	1
**2610**	0	0.45	1	0.93902	.	.	1
**2611**	0	0.45	1	0.93902	.	.	1
**2612**	0	0.45	1	0.93902	.	.	1
**2613**	0	0.45	1	0.93902	.	.	1
**2614**	0	0.45	1	0.93902	.	.	1
**2615**	0	0.45	1	0.93902	.	.	1
**2616**	0	0.45	1	0.93902	.	.	1
**2617**	0	0.45	1	0.93902	.	.	1
**2618**	0	0.45	1	0.93902	.	.	1
**2619**	0	0.45	1	0.93902	.	.	1
**2620**	0	0.45	1	0.93902	.	.	1
**2621**	0	0.45	1	0.93902	.	.	1
**2622**	0	0.45	1	0.93902	.	.	1
**2623**	0	0.45	1	0.93902	.	.	1
**2624**	0	0.45	1	0.93902	.	.	1
**2625**	0	0.45	1	0.93902	.	.	1

Obs	Sub_group	fuptimey	_CENSOR_	SURVIVAL	SDF_LCL	SDF_UCL	STRATUM
2626	0	0.45	1	0.93902	.	.	1
2627	0	0.45	1	0.93902	.	.	1
2628	0	0.45	1	0.93902	.	.	1
2629	0	0.46	0	0.93822	0.93598	0.94039	1
2630	0	0.46	1	0.93822	.	.	1
2631	0	0.46	1	0.93822	.	.	1
2632	0	0.46	1	0.93822	.	.	1
2633	0	0.46	1	0.93822	.	.	1
2634	0	0.46	1	0.93822	.	.	1
2635	0	0.46	1	0.93822	.	.	1
2636	0	0.46	1	0.93822	.	.	1
2637	0	0.46	1	0.93822	.	.	1
2638	0	0.46	1	0.93822	.	.	1
2639	0	0.46	1	0.93822	.	.	1
2640	0	0.46	1	0.93822	.	.	1
2641	0	0.46	1	0.93822	.	.	1
2642	0	0.46	1	0.93822	.	.	1
2643	0	0.46	1	0.93822	.	.	1
2644	0	0.46	1	0.93822	.	.	1
2645	0	0.46	1	0.93822	.	.	1
2646	0	0.46	1	0.93822	.	.	1
2647	0	0.46	1	0.93822	.	.	1
2648	0	0.46	1	0.93822	.	.	1
2649	0	0.46	1	0.93822	.	.	1
2650	0	0.46	1	0.93822	.	.	1
2651	0	0.46	1	0.93822	.	.	1
2652	0	0.46	1	0.93822	.	.	1
2653	0	0.46	1	0.93822	.	.	1
2654	0	0.46	1	0.93822	.	.	1
2655	0	0.46	1	0.93822	.	.	1
2656	0	0.46	1	0.93822	.	.	1
2657	0	0.46	1	0.93822	.	.	1
2658	0	0.46	1	0.93822	.	.	1
2659	0	0.46	1	0.93822	.	.	1
2660	0	0.46	1	0.93822	.	.	1

Obs	Sub_group	fuptimey	_CENSOR_	SURVIVAL	SDF_LCL	SDF_UCL	STRATUM
2661	0	0.46	1	0.93822	.	.	1
2662	0	0.46	1	0.93822	.	.	1
2663	0	0.46	1	0.93822	.	.	1
2664	0	0.46	1	0.93822	.	.	1
2665	0	0.46	1	0.93822	.	.	1
2666	0	0.46	1	0.93822	.	.	1
2667	0	0.46	1	0.93822	.	.	1
2668	0	0.46	1	0.93822	.	.	1
2669	0	0.46	1	0.93822	.	.	1
2670	0	0.46	1	0.93822	.	.	1
2671	0	0.46	1	0.93822	.	.	1
2672	0	0.46	1	0.93822	.	.	1
2673	0	0.47	0	0.93768	0.93542	0.93985	1
2674	0	0.47	1	0.93768	.	.	1
2675	0	0.47	1	0.93768	.	.	1
2676	0	0.47	1	0.93768	.	.	1
2677	0	0.47	1	0.93768	.	.	1
2678	0	0.47	1	0.93768	.	.	1
2679	0	0.47	1	0.93768	.	.	1
2680	0	0.47	1	0.93768	.	.	1
2681	0	0.47	1	0.93768	.	.	1
2682	0	0.47	1	0.93768	.	.	1
2683	0	0.47	1	0.93768	.	.	1
2684	0	0.47	1	0.93768	.	.	1
2685	0	0.47	1	0.93768	.	.	1
2686	0	0.47	1	0.93768	.	.	1
2687	0	0.47	1	0.93768	.	.	1
2688	0	0.47	1	0.93768	.	.	1
2689	0	0.47	1	0.93768	.	.	1
2690	0	0.47	1	0.93768	.	.	1
2691	0	0.47	1	0.93768	.	.	1
2692	0	0.47	1	0.93768	.	.	1
2693	0	0.47	1	0.93768	.	.	1
2694	0	0.47	1	0.93768	.	.	1
2695	0	0.47	1	0.93768	.	.	1

Obs	Sub_group	fuptimey	_CENSOR_	SURVIVAL	SDF_LCL	SDF_UCL	STRATUM
2696	0	0.47	1	0.93768	.	.	1
2697	0	0.47	1	0.93768	.	.	1
2698	0	0.47	1	0.93768	.	.	1
2699	0	0.47	1	0.93768	.	.	1
2700	0	0.47	1	0.93768	.	.	1
2701	0	0.47	1	0.93768	.	.	1
2702	0	0.47	1	0.93768	.	.	1
2703	0	0.47	1	0.93768	.	.	1
2704	0	0.47	1	0.93768	.	.	1
2705	0	0.47	1	0.93768	.	.	1
2706	0	0.47	1	0.93768	.	.	1
2707	0	0.47	1	0.93768	.	.	1
2708	0	0.47	1	0.93768	.	.	1
2709	0	0.47	1	0.93768	.	.	1
2710	0	0.47	1	0.93768	.	.	1
2711	0	0.47	1	0.93768	.	.	1
2712	0	0.47	1	0.93768	.	.	1
2713	0	0.47	1	0.93768	.	.	1
2714	0	0.47	1	0.93768	.	.	1
2715	0	0.47	1	0.93768	.	.	1
2716	0	0.47	1	0.93768	.	.	1
2717	0	0.47	1	0.93768	.	.	1
2718	0	0.47	1	0.93768	.	.	1
2719	0	0.47	1	0.93768	.	.	1
2720	0	0.47	1	0.93768	.	.	1
2721	0	0.47	1	0.93768	.	.	1
2722	0	0.47	1	0.93768	.	.	1
2723	0	0.47	1	0.93768	.	.	1
2724	0	0.47	1	0.93768	.	.	1
2725	0	0.47	1	0.93768	.	.	1
2726	0	0.47	1	0.93768	.	.	1
2727	0	0.47	1	0.93768	.	.	1
2728	0	0.47	1	0.93768	.	.	1
2729	0	0.48	0	0.93717	0.93491	0.93936	1
2730	0	0.48	1	0.93717	.	.	1

Obs	Sub_group	fuptimey	_CENSOR_	SURVIVAL	SDF_LCL	SDF_UCL	STRATUM
2731	0	0.48	1	0.93717	.	.	1
2732	0	0.48	1	0.93717	.	.	1
2733	0	0.48	1	0.93717	.	.	1
2734	0	0.48	1	0.93717	.	.	1
2735	0	0.48	1	0.93717	.	.	1
2736	0	0.48	1	0.93717	.	.	1
2737	0	0.48	1	0.93717	.	.	1
2738	0	0.48	1	0.93717	.	.	1
2739	0	0.48	1	0.93717	.	.	1
2740	0	0.48	1	0.93717	.	.	1
2741	0	0.48	1	0.93717	.	.	1
2742	0	0.48	1	0.93717	.	.	1
2743	0	0.48	1	0.93717	.	.	1
2744	0	0.48	1	0.93717	.	.	1
2745	0	0.48	1	0.93717	.	.	1
2746	0	0.48	1	0.93717	.	.	1
2747	0	0.48	1	0.93717	.	.	1
2748	0	0.48	1	0.93717	.	.	1
2749	0	0.48	1	0.93717	.	.	1
2750	0	0.48	1	0.93717	.	.	1
2751	0	0.48	1	0.93717	.	.	1
2752	0	0.48	1	0.93717	.	.	1
2753	0	0.48	1	0.93717	.	.	1
2754	0	0.48	1	0.93717	.	.	1
2755	0	0.48	1	0.93717	.	.	1
2756	0	0.48	1	0.93717	.	.	1
2757	0	0.48	1	0.93717	.	.	1
2758	0	0.48	1	0.93717	.	.	1
2759	0	0.48	1	0.93717	.	.	1
2760	0	0.48	1	0.93717	.	.	1
2761	0	0.48	1	0.93717	.	.	1
2762	0	0.48	1	0.93717	.	.	1
2763	0	0.48	1	0.93717	.	.	1
2764	0	0.48	1	0.93717	.	.	1
2765	0	0.48	1	0.93717	.	.	1

Obs	Sub_group	fuptimey	_CENSOR_	SURVIVAL	SDF_LCL	SDF_UCL	STRATUM
2766	0	0.48	1	0.93717	.	.	1
2767	0	0.48	1	0.93717	.	.	1
2768	0	0.48	1	0.93717	.	.	1
2769	0	0.48	1	0.93717	.	.	1
2770	0	0.48	1	0.93717	.	.	1
2771	0	0.48	1	0.93717	.	.	1
2772	0	0.48	1	0.93717	.	.	1
2773	0	0.49	0	0.93656	0.93429	0.93875	1
2774	0	0.49	1	0.93656	.	.	1
2775	0	0.49	1	0.93656	.	.	1
2776	0	0.49	1	0.93656	.	.	1
2777	0	0.49	1	0.93656	.	.	1
2778	0	0.49	1	0.93656	.	.	1
2779	0	0.49	1	0.93656	.	.	1
2780	0	0.49	1	0.93656	.	.	1
2781	0	0.49	1	0.93656	.	.	1
2782	0	0.49	1	0.93656	.	.	1
2783	0	0.49	1	0.93656	.	.	1
2784	0	0.49	1	0.93656	.	.	1
2785	0	0.49	1	0.93656	.	.	1
2786	0	0.49	1	0.93656	.	.	1
2787	0	0.49	1	0.93656	.	.	1
2788	0	0.49	1	0.93656	.	.	1
2789	0	0.49	1	0.93656	.	.	1
2790	0	0.49	1	0.93656	.	.	1
2791	0	0.49	1	0.93656	.	.	1
2792	0	0.49	1	0.93656	.	.	1
2793	0	0.49	1	0.93656	.	.	1
2794	0	0.49	1	0.93656	.	.	1
2795	0	0.49	1	0.93656	.	.	1
2796	0	0.49	1	0.93656	.	.	1
2797	0	0.49	1	0.93656	.	.	1
2798	0	0.49	1	0.93656	.	.	1
2799	0	0.49	1	0.93656	.	.	1
2800	0	0.49	1	0.93656	.	.	1

Obs	Sub_group	fuptimey	_CENSOR_	SURVIVAL	SDF_LCL	SDF_UCL	STRATUM
2801	0	0.49	1	0.93656	.	.	1
2802	0	0.49	1	0.93656	.	.	1
2803	0	0.49	1	0.93656	.	.	1
2804	0	0.49	1	0.93656	.	.	1
2805	0	0.49	1	0.93656	.	.	1
2806	0	0.49	1	0.93656	.	.	1
2807	0	0.49	1	0.93656	.	.	1
2808	0	0.49	1	0.93656	.	.	1
2809	0	0.49	1	0.93656	.	.	1
2810	0	0.49	1	0.93656	.	.	1
2811	0	0.49	1	0.93656	.	.	1
2812	0	0.49	1	0.93656	.	.	1
2813	0	0.49	1	0.93656	.	.	1
2814	0	0.49	1	0.93656	.	.	1
2815	0	0.49	1	0.93656	.	.	1
2816	0	0.49	1	0.93656	.	.	1
2817	0	0.49	1	0.93656	.	.	1
2818	0	0.49	1	0.93656	.	.	1
2819	0	0.49	1	0.93656	.	.	1
2820	0	0.49	1	0.93656	.	.	1
2821	0	0.49	1	0.93656	.	.	1
2822	0	0.49	1	0.93656	.	.	1
2823	0	0.49	1	0.93656	.	.	1
2824	0	0.49	1	0.93656	.	.	1
2825	0	0.49	1	0.93656	.	.	1
2826	0	0.49	1	0.93656	.	.	1
2827	0	0.49	1	0.93656	.	.	1
2828	0	0.49	1	0.93656	.	.	1
2829	0	0.49	1	0.93656	.	.	1
2830	0	0.49	1	0.93656	.	.	1
2831	0	0.49	1	0.93656	.	.	1
2832	0	0.49	1	0.93656	.	.	1
2833	0	0.49	1	0.93656	.	.	1
2834	0	0.49	1	0.93656	.	.	1
2835	0	0.49	1	0.93656	.	.	1

Obs	Sub_group	fuptimey	_CENSOR_	SURVIVAL	SDF_LCL	SDF_UCL	STRATUM
2836	0	0.49	1	0.93656	.	.	1
2837	0	0.49	1	0.93656	.	.	1
2838	0	0.49	1	0.93656	.	.	1
2839	0	0.49	1	0.93656	.	.	1
2840	0	0.49	1	0.93656	.	.	1
2841	0	0.49	1	0.93656	.	.	1
2842	0	0.49	1	0.93656	.	.	1
2843	0	0.50	0	0.93601	0.93373	0.93822	1
2844	0	0.50	1	0.93601	.	.	1
2845	0	0.50	1	0.93601	.	.	1
2846	0	0.50	1	0.93601	.	.	1
2847	0	0.50	1	0.93601	.	.	1
2848	0	0.50	1	0.93601	.	.	1
2849	0	0.50	1	0.93601	.	.	1
2850	0	0.50	1	0.93601	.	.	1
2851	0	0.50	1	0.93601	.	.	1
2852	0	0.50	1	0.93601	.	.	1
2853	0	0.50	1	0.93601	.	.	1
2854	0	0.50	1	0.93601	.	.	1
2855	0	0.50	1	0.93601	.	.	1
2856	0	0.50	1	0.93601	.	.	1
2857	0	0.50	1	0.93601	.	.	1
2858	0	0.50	1	0.93601	.	.	1
2859	0	0.50	1	0.93601	.	.	1
2860	0	0.50	1	0.93601	.	.	1
2861	0	0.50	1	0.93601	.	.	1
2862	0	0.50	1	0.93601	.	.	1
2863	0	0.50	1	0.93601	.	.	1
2864	0	0.50	1	0.93601	.	.	1
2865	0	0.50	1	0.93601	.	.	1
2866	0	0.50	1	0.93601	.	.	1
2867	0	0.50	1	0.93601	.	.	1
2868	0	0.50	1	0.93601	.	.	1
2869	0	0.50	1	0.93601	.	.	1
2870	0	0.50	1	0.93601	.	.	1

Obs	Sub_group	fuptimey	_CENSOR_	SURVIVAL	SDF_LCL	SDF_UCL	STRATUM
2871	0	0.50	1	0.93601	.	.	1
2872	0	0.50	1	0.93601	.	.	1
2873	0	0.50	1	0.93601	.	.	1
2874	0	0.50	1	0.93601	.	.	1
2875	0	0.50	1	0.93601	.	.	1
2876	0	0.50	1	0.93601	.	.	1
2877	0	0.50	1	0.93601	.	.	1
2878	0	0.50	1	0.93601	.	.	1
2879	0	0.50	1	0.93601	.	.	1
2880	0	0.50	1	0.93601	.	.	1
2881	0	0.51	0	0.93546	0.93317	0.93768	1
2882	0	0.51	1	0.93546	.	.	1
2883	0	0.51	1	0.93546	.	.	1
2884	0	0.51	1	0.93546	.	.	1
2885	0	0.51	1	0.93546	.	.	1
2886	0	0.51	1	0.93546	.	.	1
2887	0	0.51	1	0.93546	.	.	1
2888	0	0.51	1	0.93546	.	.	1
2889	0	0.51	1	0.93546	.	.	1
2890	0	0.51	1	0.93546	.	.	1
2891	0	0.51	1	0.93546	.	.	1
2892	0	0.51	1	0.93546	.	.	1
2893	0	0.51	1	0.93546	.	.	1
2894	0	0.51	1	0.93546	.	.	1
2895	0	0.51	1	0.93546	.	.	1
2896	0	0.51	1	0.93546	.	.	1
2897	0	0.51	1	0.93546	.	.	1
2898	0	0.51	1	0.93546	.	.	1
2899	0	0.51	1	0.93546	.	.	1
2900	0	0.51	1	0.93546	.	.	1
2901	0	0.51	1	0.93546	.	.	1
2902	0	0.51	1	0.93546	.	.	1
2903	0	0.51	1	0.93546	.	.	1
2904	0	0.51	1	0.93546	.	.	1
2905	0	0.51	1	0.93546	.	.	1

Obs	Sub_group	fuptimey	_CENSOR_	SURVIVAL	SDF_LCL	SDF_UCL	STRATUM
2906	0	0.51	1	0.93546	.	.	1
2907	0	0.51	1	0.93546	.	.	1
2908	0	0.51	1	0.93546	.	.	1
2909	0	0.51	1	0.93546	.	.	1
2910	0	0.51	1	0.93546	.	.	1
2911	0	0.51	1	0.93546	.	.	1
2912	0	0.51	1	0.93546	.	.	1
2913	0	0.51	1	0.93546	.	.	1
2914	0	0.51	1	0.93546	.	.	1
2915	0	0.51	1	0.93546	.	.	1
2916	0	0.51	1	0.93546	.	.	1
2917	0	0.51	1	0.93546	.	.	1
2918	0	0.51	1	0.93546	.	.	1
2919	0	0.51	1	0.93546	.	.	1
2920	0	0.51	1	0.93546	.	.	1
2921	0	0.51	1	0.93546	.	.	1
2922	0	0.51	1	0.93546	.	.	1
2923	0	0.51	1	0.93546	.	.	1
2924	0	0.51	1	0.93546	.	.	1
2925	0	0.51	1	0.93546	.	.	1
2926	0	0.51	1	0.93546	.	.	1
2927	0	0.51	1	0.93546	.	.	1
2928	0	0.51	1	0.93546	.	.	1
2929	0	0.51	1	0.93546	.	.	1
2930	0	0.51	1	0.93546	.	.	1
2931	0	0.51	1	0.93546	.	.	1
2932	0	0.51	1	0.93546	.	.	1
2933	0	0.51	1	0.93546	.	.	1
2934	0	0.52	0	0.93482	0.93252	0.93704	1
2935	0	0.52	1	0.93482	.	.	1
2936	0	0.52	1	0.93482	.	.	1
2937	0	0.52	1	0.93482	.	.	1
2938	0	0.52	1	0.93482	.	.	1
2939	0	0.52	1	0.93482	.	.	1
2940	0	0.52	1	0.93482	.	.	1

Obs	Sub_group	fuptimey	_CENSOR_	SURVIVAL	SDF_LCL	SDF_UCL	STRATUM
2941	0	0.52	1	0.93482	.	.	1
2942	0	0.52	1	0.93482	.	.	1
2943	0	0.52	1	0.93482	.	.	1
2944	0	0.52	1	0.93482	.	.	1
2945	0	0.52	1	0.93482	.	.	1
2946	0	0.52	1	0.93482	.	.	1
2947	0	0.52	1	0.93482	.	.	1
2948	0	0.52	1	0.93482	.	.	1
2949	0	0.52	1	0.93482	.	.	1
2950	0	0.52	1	0.93482	.	.	1
2951	0	0.52	1	0.93482	.	.	1
2952	0	0.52	1	0.93482	.	.	1
2953	0	0.52	1	0.93482	.	.	1
2954	0	0.52	1	0.93482	.	.	1
2955	0	0.52	1	0.93482	.	.	1
2956	0	0.52	1	0.93482	.	.	1
2957	0	0.52	1	0.93482	.	.	1
2958	0	0.52	1	0.93482	.	.	1
2959	0	0.52	1	0.93482	.	.	1
2960	0	0.52	1	0.93482	.	.	1
2961	0	0.52	1	0.93482	.	.	1
2962	0	0.52	1	0.93482	.	.	1
2963	0	0.52	1	0.93482	.	.	1
2964	0	0.52	1	0.93482	.	.	1
2965	0	0.52	1	0.93482	.	.	1
2966	0	0.52	1	0.93482	.	.	1
2967	0	0.52	1	0.93482	.	.	1
2968	0	0.52	1	0.93482	.	.	1
2969	0	0.52	1	0.93482	.	.	1
2970	0	0.52	1	0.93482	.	.	1
2971	0	0.52	1	0.93482	.	.	1
2972	0	0.52	1	0.93482	.	.	1
2973	0	0.52	1	0.93482	.	.	1
2974	0	0.52	1	0.93482	.	.	1
2975	0	0.52	1	0.93482	.	.	1

Obs	Sub_group	fuptimey	_CENSOR_	SURVIVAL	SDF_LCL	SDF_UCL	STRATUM
2976	0	0.52	1	0.93482	.	.	1
2977	0	0.52	1	0.93482	.	.	1
2978	0	0.52	1	0.93482	.	.	1
2979	0	0.52	1	0.93482	.	.	1
2980	0	0.52	1	0.93482	.	.	1
2981	0	0.52	1	0.93482	.	.	1
2982	0	0.52	1	0.93482	.	.	1
2983	0	0.52	1	0.93482	.	.	1
2984	0	0.52	1	0.93482	.	.	1
2985	0	0.53	0	0.93431	0.93200	0.93655	1
2986	0	0.53	1	0.93431	.	.	1
2987	0	0.53	1	0.93431	.	.	1
2988	0	0.53	1	0.93431	.	.	1
2989	0	0.53	1	0.93431	.	.	1
2990	0	0.53	1	0.93431	.	.	1
2991	0	0.53	1	0.93431	.	.	1
2992	0	0.53	1	0.93431	.	.	1
2993	0	0.53	1	0.93431	.	.	1
2994	0	0.53	1	0.93431	.	.	1
2995	0	0.53	1	0.93431	.	.	1
2996	0	0.53	1	0.93431	.	.	1
2997	0	0.53	1	0.93431	.	.	1
2998	0	0.53	1	0.93431	.	.	1
2999	0	0.53	1	0.93431	.	.	1
3000	0	0.53	1	0.93431	.	.	1
3001	0	0.53	1	0.93431	.	.	1
3002	0	0.53	1	0.93431	.	.	1
3003	0	0.53	1	0.93431	.	.	1
3004	0	0.53	1	0.93431	.	.	1
3005	0	0.53	1	0.93431	.	.	1
3006	0	0.53	1	0.93431	.	.	1
3007	0	0.53	1	0.93431	.	.	1
3008	0	0.53	1	0.93431	.	.	1
3009	0	0.53	1	0.93431	.	.	1
3010	0	0.53	1	0.93431	.	.	1

Obs	Sub_group	fuptimey	_CENSOR_	SURVIVAL	SDF_LCL	SDF_UCL	STRATUM
3011	0	0.53	1	0.93431	.	.	1
3012	0	0.53	1	0.93431	.	.	1
3013	0	0.53	1	0.93431	.	.	1
3014	0	0.53	1	0.93431	.	.	1
3015	0	0.53	1	0.93431	.	.	1
3016	0	0.53	1	0.93431	.	.	1
3017	0	0.53	1	0.93431	.	.	1
3018	0	0.53	1	0.93431	.	.	1
3019	0	0.53	1	0.93431	.	.	1
3020	0	0.53	1	0.93431	.	.	1
3021	0	0.53	1	0.93431	.	.	1
3022	0	0.53	1	0.93431	.	.	1
3023	0	0.53	1	0.93431	.	.	1
3024	0	0.53	1	0.93431	.	.	1
3025	0	0.53	1	0.93431	.	.	1
3026	0	0.53	1	0.93431	.	.	1
3027	0	0.53	1	0.93431	.	.	1
3028	0	0.53	1	0.93431	.	.	1
3029	0	0.53	1	0.93431	.	.	1
3030	0	0.53	1	0.93431	.	.	1
3031	0	0.53	1	0.93431	.	.	1
3032	0	0.53	1	0.93431	.	.	1
3033	0	0.53	1	0.93431	.	.	1
3034	0	0.53	1	0.93431	.	.	1
3035	0	0.53	1	0.93431	.	.	1
3036	0	0.54	0	0.93365	0.93133	0.93589	1
3037	0	0.54	1	0.93365	.	.	1
3038	0	0.54	1	0.93365	.	.	1
3039	0	0.54	1	0.93365	.	.	1
3040	0	0.54	1	0.93365	.	.	1
3041	0	0.54	1	0.93365	.	.	1
3042	0	0.54	1	0.93365	.	.	1
3043	0	0.54	1	0.93365	.	.	1
3044	0	0.54	1	0.93365	.	.	1
3045	0	0.54	1	0.93365	.	.	1

Obs	Sub_group	fuptimey	_CENSOR_	SURVIVAL	SDF_LCL	SDF_UCL	STRATUM
3046	0	0.54	1	0.93365	.	.	1
3047	0	0.54	1	0.93365	.	.	1
3048	0	0.54	1	0.93365	.	.	1
3049	0	0.54	1	0.93365	.	.	1
3050	0	0.54	1	0.93365	.	.	1
3051	0	0.54	1	0.93365	.	.	1
3052	0	0.54	1	0.93365	.	.	1
3053	0	0.54	1	0.93365	.	.	1
3054	0	0.54	1	0.93365	.	.	1
3055	0	0.54	1	0.93365	.	.	1
3056	0	0.54	1	0.93365	.	.	1
3057	0	0.54	1	0.93365	.	.	1
3058	0	0.54	1	0.93365	.	.	1
3059	0	0.54	1	0.93365	.	.	1
3060	0	0.54	1	0.93365	.	.	1
3061	0	0.54	1	0.93365	.	.	1
3062	0	0.54	1	0.93365	.	.	1
3063	0	0.54	1	0.93365	.	.	1
3064	0	0.54	1	0.93365	.	.	1
3065	0	0.54	1	0.93365	.	.	1
3066	0	0.54	1	0.93365	.	.	1
3067	0	0.54	1	0.93365	.	.	1
3068	0	0.54	1	0.93365	.	.	1
3069	0	0.54	1	0.93365	.	.	1
3070	0	0.54	1	0.93365	.	.	1
3071	0	0.54	1	0.93365	.	.	1
3072	0	0.54	1	0.93365	.	.	1
3073	0	0.54	1	0.93365	.	.	1
3074	0	0.54	1	0.93365	.	.	1
3075	0	0.54	1	0.93365	.	.	1
3076	0	0.54	1	0.93365	.	.	1
3077	0	0.54	1	0.93365	.	.	1
3078	0	0.54	1	0.93365	.	.	1
3079	0	0.54	1	0.93365	.	.	1
3080	0	0.54	1	0.93365	.	.	1

Obs	Sub_group	fuptimey	_CENSOR_	SURVIVAL	SDF_LCL	SDF_UCL	STRATUM
3081	0	0.54	1	0.93365	.	.	1
3082	0	0.54	1	0.93365	.	.	1
3083	0	0.54	1	0.93365	.	.	1
3084	0	0.54	1	0.93365	.	.	1
3085	0	0.54	1	0.93365	.	.	1
3086	0	0.55	0	0.93307	0.93074	0.93533	1
3087	0	0.55	1	0.93307	.	.	1
3088	0	0.55	1	0.93307	.	.	1
3089	0	0.55	1	0.93307	.	.	1
3090	0	0.55	1	0.93307	.	.	1
3091	0	0.55	1	0.93307	.	.	1
3092	0	0.55	1	0.93307	.	.	1
3093	0	0.55	1	0.93307	.	.	1
3094	0	0.55	1	0.93307	.	.	1
3095	0	0.55	1	0.93307	.	.	1
3096	0	0.55	1	0.93307	.	.	1
3097	0	0.55	1	0.93307	.	.	1
3098	0	0.55	1	0.93307	.	.	1
3099	0	0.55	1	0.93307	.	.	1
3100	0	0.55	1	0.93307	.	.	1
3101	0	0.55	1	0.93307	.	.	1
3102	0	0.55	1	0.93307	.	.	1
3103	0	0.55	1	0.93307	.	.	1
3104	0	0.55	1	0.93307	.	.	1
3105	0	0.55	1	0.93307	.	.	1
3106	0	0.55	1	0.93307	.	.	1
3107	0	0.55	1	0.93307	.	.	1
3108	0	0.55	1	0.93307	.	.	1
3109	0	0.55	1	0.93307	.	.	1
3110	0	0.55	1	0.93307	.	.	1
3111	0	0.55	1	0.93307	.	.	1
3112	0	0.55	1	0.93307	.	.	1
3113	0	0.55	1	0.93307	.	.	1
3114	0	0.55	1	0.93307	.	.	1
3115	0	0.55	1	0.93307	.	.	1

Obs	Sub_group	fuptimey	_CENSOR_	SURVIVAL	SDF_LCL	SDF_UCL	STRATUM
3116	0	0.55	1	0.93307	.	.	1
3117	0	0.55	1	0.93307	.	.	1
3118	0	0.55	1	0.93307	.	.	1
3119	0	0.55	1	0.93307	.	.	1
3120	0	0.55	1	0.93307	.	.	1
3121	0	0.55	1	0.93307	.	.	1
3122	0	0.55	1	0.93307	.	.	1
3123	0	0.55	1	0.93307	.	.	1
3124	0	0.55	1	0.93307	.	.	1
3125	0	0.55	1	0.93307	.	.	1
3126	0	0.55	1	0.93307	.	.	1
3127	0	0.55	1	0.93307	.	.	1
3128	0	0.55	1	0.93307	.	.	1
3129	0	0.55	1	0.93307	.	.	1
3130	0	0.55	1	0.93307	.	.	1
3131	0	0.55	1	0.93307	.	.	1
3132	0	0.55	1	0.93307	.	.	1
3133	0	0.55	1	0.93307	.	.	1
3134	0	0.55	1	0.93307	.	.	1
3135	0	0.55	1	0.93307	.	.	1
3136	0	0.55	1	0.93307	.	.	1
3137	0	0.55	1	0.93307	.	.	1
3138	0	0.55	1	0.93307	.	.	1
3139	0	0.56	0	0.93266	0.93032	0.93492	1
3140	0	0.56	1	0.93266	.	.	1
3141	0	0.56	1	0.93266	.	.	1
3142	0	0.56	1	0.93266	.	.	1
3143	0	0.56	1	0.93266	.	.	1
3144	0	0.56	1	0.93266	.	.	1
3145	0	0.56	1	0.93266	.	.	1
3146	0	0.56	1	0.93266	.	.	1
3147	0	0.56	1	0.93266	.	.	1
3148	0	0.56	1	0.93266	.	.	1
3149	0	0.56	1	0.93266	.	.	1
3150	0	0.56	1	0.93266	.	.	1

Obs	Sub_group	fuptimey	_CENSOR_	SURVIVAL	SDF_LCL	SDF_UCL	STRATUM
3151	0	0.56	1	0.93266	.	.	1
3152	0	0.56	1	0.93266	.	.	1
3153	0	0.56	1	0.93266	.	.	1
3154	0	0.56	1	0.93266	.	.	1
3155	0	0.56	1	0.93266	.	.	1
3156	0	0.56	1	0.93266	.	.	1
3157	0	0.56	1	0.93266	.	.	1
3158	0	0.56	1	0.93266	.	.	1
3159	0	0.56	1	0.93266	.	.	1
3160	0	0.56	1	0.93266	.	.	1
3161	0	0.56	1	0.93266	.	.	1
3162	0	0.56	1	0.93266	.	.	1
3163	0	0.56	1	0.93266	.	.	1
3164	0	0.56	1	0.93266	.	.	1
3165	0	0.56	1	0.93266	.	.	1
3166	0	0.56	1	0.93266	.	.	1
3167	0	0.56	1	0.93266	.	.	1
3168	0	0.56	1	0.93266	.	.	1
3169	0	0.56	1	0.93266	.	.	1
3170	0	0.56	1	0.93266	.	.	1
3171	0	0.56	1	0.93266	.	.	1
3172	0	0.56	1	0.93266	.	.	1
3173	0	0.56	1	0.93266	.	.	1
3174	0	0.56	1	0.93266	.	.	1
3175	0	0.56	1	0.93266	.	.	1
3176	0	0.56	1	0.93266	.	.	1
3177	0	0.56	1	0.93266	.	.	1
3178	0	0.56	1	0.93266	.	.	1
3179	0	0.56	1	0.93266	.	.	1
3180	0	0.56	1	0.93266	.	.	1
3181	0	0.56	1	0.93266	.	.	1
3182	0	0.56	1	0.93266	.	.	1
3183	0	0.56	1	0.93266	.	.	1
3184	0	0.56	1	0.93266	.	.	1
3185	0	0.56	1	0.93266	.	.	1

Obs	Sub_group	fuptimey	_CENSOR_	SURVIVAL	SDF_LCL	SDF_UCL	STRATUM
3186	0	0.56	1	0.93266	.	.	1
3187	0	0.56	1	0.93266	.	.	1
3188	0	0.56	1	0.93266	.	.	1
3189	0	0.56	1	0.93266	.	.	1
3190	0	0.56	1	0.93266	.	.	1
3191	0	0.56	1	0.93266	.	.	1
3192	0	0.56	1	0.93266	.	.	1
3193	0	0.56	1	0.93266	.	.	1
3194	0	0.56	1	0.93266	.	.	1
3195	0	0.56	1	0.93266	.	.	1
3196	0	0.57	0	0.93229	0.92994	0.93456	1
3197	0	0.57	1	0.93229	.	.	1
3198	0	0.57	1	0.93229	.	.	1
3199	0	0.57	1	0.93229	.	.	1
3200	0	0.57	1	0.93229	.	.	1
3201	0	0.57	1	0.93229	.	.	1
3202	0	0.57	1	0.93229	.	.	1
3203	0	0.57	1	0.93229	.	.	1
3204	0	0.57	1	0.93229	.	.	1
3205	0	0.57	1	0.93229	.	.	1
3206	0	0.57	1	0.93229	.	.	1
3207	0	0.57	1	0.93229	.	.	1
3208	0	0.57	1	0.93229	.	.	1
3209	0	0.57	1	0.93229	.	.	1
3210	0	0.57	1	0.93229	.	.	1
3211	0	0.57	1	0.93229	.	.	1
3212	0	0.57	1	0.93229	.	.	1
3213	0	0.57	1	0.93229	.	.	1
3214	0	0.57	1	0.93229	.	.	1
3215	0	0.57	1	0.93229	.	.	1
3216	0	0.57	1	0.93229	.	.	1
3217	0	0.57	1	0.93229	.	.	1
3218	0	0.57	1	0.93229	.	.	1
3219	0	0.57	1	0.93229	.	.	1
3220	0	0.57	1	0.93229	.	.	1

Obs	Sub_group	fuptimey	_CENSOR_	SURVIVAL	SDF_LCL	SDF_UCL	STRATUM
3221	0	0.57	1	0.93229	.	.	1
3222	0	0.57	1	0.93229	.	.	1
3223	0	0.57	1	0.93229	.	.	1
3224	0	0.57	1	0.93229	.	.	1
3225	0	0.57	1	0.93229	.	.	1
3226	0	0.57	1	0.93229	.	.	1
3227	0	0.57	1	0.93229	.	.	1
3228	0	0.57	1	0.93229	.	.	1
3229	0	0.57	1	0.93229	.	.	1
3230	0	0.57	1	0.93229	.	.	1
3231	0	0.57	1	0.93229	.	.	1
3232	0	0.57	1	0.93229	.	.	1
3233	0	0.57	1	0.93229	.	.	1
3234	0	0.57	1	0.93229	.	.	1
3235	0	0.57	1	0.93229	.	.	1
3236	0	0.57	1	0.93229	.	.	1
3237	0	0.57	1	0.93229	.	.	1
3238	0	0.57	1	0.93229	.	.	1
3239	0	0.57	1	0.93229	.	.	1
3240	0	0.57	1	0.93229	.	.	1
3241	0	0.57	1	0.93229	.	.	1
3242	0	0.57	1	0.93229	.	.	1
3243	0	0.58	0	0.93167	0.92931	0.93394	1
3244	0	0.58	1	0.93167	.	.	1
3245	0	0.58	1	0.93167	.	.	1
3246	0	0.58	1	0.93167	.	.	1
3247	0	0.58	1	0.93167	.	.	1
3248	0	0.58	1	0.93167	.	.	1
3249	0	0.58	1	0.93167	.	.	1
3250	0	0.58	1	0.93167	.	.	1
3251	0	0.58	1	0.93167	.	.	1
3252	0	0.58	1	0.93167	.	.	1
3253	0	0.58	1	0.93167	.	.	1
3254	0	0.58	1	0.93167	.	.	1
3255	0	0.58	1	0.93167	.	.	1

Obs	Sub_group	fuptimey	_CENSOR_	SURVIVAL	SDF_LCL	SDF_UCL	STRATUM
3256	0	0.58	1	0.93167	.	.	1
3257	0	0.58	1	0.93167	.	.	1
3258	0	0.58	1	0.93167	.	.	1
3259	0	0.58	1	0.93167	.	.	1
3260	0	0.58	1	0.93167	.	.	1
3261	0	0.58	1	0.93167	.	.	1
3262	0	0.58	1	0.93167	.	.	1
3263	0	0.58	1	0.93167	.	.	1
3264	0	0.58	1	0.93167	.	.	1
3265	0	0.58	1	0.93167	.	.	1
3266	0	0.58	1	0.93167	.	.	1
3267	0	0.58	1	0.93167	.	.	1
3268	0	0.58	1	0.93167	.	.	1
3269	0	0.58	1	0.93167	.	.	1
3270	0	0.58	1	0.93167	.	.	1
3271	0	0.58	1	0.93167	.	.	1
3272	0	0.58	1	0.93167	.	.	1
3273	0	0.58	1	0.93167	.	.	1
3274	0	0.58	1	0.93167	.	.	1
3275	0	0.58	1	0.93167	.	.	1
3276	0	0.58	1	0.93167	.	.	1
3277	0	0.58	1	0.93167	.	.	1
3278	0	0.58	1	0.93167	.	.	1
3279	0	0.58	1	0.93167	.	.	1
3280	0	0.58	1	0.93167	.	.	1
3281	0	0.58	1	0.93167	.	.	1
3282	0	0.58	1	0.93167	.	.	1
3283	0	0.58	1	0.93167	.	.	1
3284	0	0.58	1	0.93167	.	.	1
3285	0	0.58	1	0.93167	.	.	1
3286	0	0.58	1	0.93167	.	.	1
3287	0	0.58	1	0.93167	.	.	1
3288	0	0.58	1	0.93167	.	.	1
3289	0	0.58	1	0.93167	.	.	1
3290	0	0.58	1	0.93167	.	.	1

Obs	Sub_group	fuptimey	_CENSOR_	SURVIVAL	SDF_LCL	SDF_UCL	STRATUM
3291	0	0.58	1	0.93167	.	.	1
3292	0	0.58	1	0.93167	.	.	1
3293	0	0.58	1	0.93167	.	.	1
3294	0	0.58	1	0.93167	.	.	1
3295	0	0.58	1	0.93167	.	.	1
3296	0	0.58	1	0.93167	.	.	1
3297	0	0.58	1	0.93167	.	.	1
3298	0	0.58	1	0.93167	.	.	1
3299	0	0.58	1	0.93167	.	.	1
3300	0	0.59	0	0.93125	0.92889	0.93354	1
3301	0	0.59	1	0.93125	.	.	1
3302	0	0.59	1	0.93125	.	.	1
3303	0	0.59	1	0.93125	.	.	1
3304	0	0.59	1	0.93125	.	.	1
3305	0	0.59	1	0.93125	.	.	1
3306	0	0.59	1	0.93125	.	.	1
3307	0	0.59	1	0.93125	.	.	1
3308	0	0.59	1	0.93125	.	.	1
3309	0	0.59	1	0.93125	.	.	1
3310	0	0.59	1	0.93125	.	.	1
3311	0	0.59	1	0.93125	.	.	1
3312	0	0.59	1	0.93125	.	.	1
3313	0	0.59	1	0.93125	.	.	1
3314	0	0.59	1	0.93125	.	.	1
3315	0	0.59	1	0.93125	.	.	1
3316	0	0.59	1	0.93125	.	.	1
3317	0	0.59	1	0.93125	.	.	1
3318	0	0.59	1	0.93125	.	.	1
3319	0	0.59	1	0.93125	.	.	1
3320	0	0.59	1	0.93125	.	.	1
3321	0	0.59	1	0.93125	.	.	1
3322	0	0.59	1	0.93125	.	.	1
3323	0	0.59	1	0.93125	.	.	1
3324	0	0.59	1	0.93125	.	.	1
3325	0	0.59	1	0.93125	.	.	1

Obs	Sub_group	fuptimey	_CENSOR_	SURVIVAL	SDF_LCL	SDF_UCL	STRATUM
3326	0	0.59	1	0.93125	.	.	1
3327	0	0.59	1	0.93125	.	.	1
3328	0	0.59	1	0.93125	.	.	1
3329	0	0.59	1	0.93125	.	.	1
3330	0	0.59	1	0.93125	.	.	1
3331	0	0.59	1	0.93125	.	.	1
3332	0	0.59	1	0.93125	.	.	1
3333	0	0.59	1	0.93125	.	.	1
3334	0	0.59	1	0.93125	.	.	1
3335	0	0.59	1	0.93125	.	.	1
3336	0	0.59	1	0.93125	.	.	1
3337	0	0.59	1	0.93125	.	.	1
3338	0	0.59	1	0.93125	.	.	1
3339	0	0.60	0	0.93093	0.92856	0.93322	1
3340	0	0.60	1	0.93093	.	.	1
3341	0	0.60	1	0.93093	.	.	1
3342	0	0.60	1	0.93093	.	.	1
3343	0	0.60	1	0.93093	.	.	1
3344	0	0.60	1	0.93093	.	.	1
3345	0	0.60	1	0.93093	.	.	1
3346	0	0.60	1	0.93093	.	.	1
3347	0	0.60	1	0.93093	.	.	1
3348	0	0.60	1	0.93093	.	.	1
3349	0	0.60	1	0.93093	.	.	1
3350	0	0.60	1	0.93093	.	.	1
3351	0	0.60	1	0.93093	.	.	1
3352	0	0.60	1	0.93093	.	.	1
3353	0	0.60	1	0.93093	.	.	1
3354	0	0.60	1	0.93093	.	.	1
3355	0	0.60	1	0.93093	.	.	1
3356	0	0.60	1	0.93093	.	.	1
3357	0	0.60	1	0.93093	.	.	1
3358	0	0.60	1	0.93093	.	.	1
3359	0	0.60	1	0.93093	.	.	1
3360	0	0.60	1	0.93093	.	.	1

Obs	Sub_group	fuptimey	_CENSOR_	SURVIVAL	SDF_LCL	SDF_UCL	STRATUM
3361	0	0.60	1	0.93093	.	.	1
3362	0	0.60	1	0.93093	.	.	1
3363	0	0.60	1	0.93093	.	.	1
3364	0	0.60	1	0.93093	.	.	1
3365	0	0.60	1	0.93093	.	.	1
3366	0	0.60	1	0.93093	.	.	1
3367	0	0.60	1	0.93093	.	.	1
3368	0	0.60	1	0.93093	.	.	1
3369	0	0.60	1	0.93093	.	.	1
3370	0	0.60	1	0.93093	.	.	1
3371	0	0.60	1	0.93093	.	.	1
3372	0	0.60	1	0.93093	.	.	1
3373	0	0.60	1	0.93093	.	.	1
3374	0	0.60	1	0.93093	.	.	1
3375	0	0.60	1	0.93093	.	.	1
3376	0	0.60	1	0.93093	.	.	1
3377	0	0.60	1	0.93093	.	.	1
3378	0	0.60	1	0.93093	.	.	1
3379	0	0.60	1	0.93093	.	.	1
3380	0	0.60	1	0.93093	.	.	1
3381	0	0.60	1	0.93093	.	.	1
3382	0	0.60	1	0.93093	.	.	1
3383	0	0.60	1	0.93093	.	.	1
3384	0	0.60	1	0.93093	.	.	1
3385	0	0.60	1	0.93093	.	.	1
3386	0	0.60	1	0.93093	.	.	1
3387	0	0.60	1	0.93093	.	.	1
3388	0	0.60	1	0.93093	.	.	1
3389	0	0.60	1	0.93093	.	.	1
3390	0	0.60	1	0.93093	.	.	1
3391	0	0.60	1	0.93093	.	.	1
3392	0	0.60	1	0.93093	.	.	1
3393	0	0.60	1	0.93093	.	.	1
3394	0	0.60	1	0.93093	.	.	1
3395	0	0.60	1	0.93093	.	.	1

Obs	Sub_group	fuptimey	_CENSOR_	SURVIVAL	SDF_LCL	SDF_UCL	STRATUM
3396	0	0.60	1	0.93093	.	.	1
3397	0	0.60	1	0.93093	.	.	1
3398	0	0.61	0	0.93046	0.92809	0.93276	1
3399	0	0.61	1	0.93046	.	.	1
3400	0	0.61	1	0.93046	.	.	1
3401	0	0.61	1	0.93046	.	.	1
3402	0	0.61	1	0.93046	.	.	1
3403	0	0.61	1	0.93046	.	.	1
3404	0	0.61	1	0.93046	.	.	1
3405	0	0.61	1	0.93046	.	.	1
3406	0	0.61	1	0.93046	.	.	1
3407	0	0.61	1	0.93046	.	.	1
3408	0	0.61	1	0.93046	.	.	1
3409	0	0.61	1	0.93046	.	.	1
3410	0	0.61	1	0.93046	.	.	1
3411	0	0.61	1	0.93046	.	.	1
3412	0	0.61	1	0.93046	.	.	1
3413	0	0.61	1	0.93046	.	.	1
3414	0	0.61	1	0.93046	.	.	1
3415	0	0.61	1	0.93046	.	.	1
3416	0	0.61	1	0.93046	.	.	1
3417	0	0.61	1	0.93046	.	.	1
3418	0	0.61	1	0.93046	.	.	1
3419	0	0.61	1	0.93046	.	.	1
3420	0	0.61	1	0.93046	.	.	1
3421	0	0.61	1	0.93046	.	.	1
3422	0	0.61	1	0.93046	.	.	1
3423	0	0.61	1	0.93046	.	.	1
3424	0	0.61	1	0.93046	.	.	1
3425	0	0.61	1	0.93046	.	.	1
3426	0	0.61	1	0.93046	.	.	1
3427	0	0.61	1	0.93046	.	.	1
3428	0	0.61	1	0.93046	.	.	1
3429	0	0.61	1	0.93046	.	.	1
3430	0	0.61	1	0.93046	.	.	1

Obs	Sub_group	fuptimey	_CENSOR_	SURVIVAL	SDF_LCL	SDF_UCL	STRATUM
**3431**	0	0.61	1	0.93046	.	.	1
**3432**	0	0.61	1	0.93046	.	.	1
**3433**	0	0.61	1	0.93046	.	.	1
**3434**	0	0.61	1	0.93046	.	.	1
**3435**	0	0.61	1	0.93046	.	.	1
**3436**	0	0.61	1	0.93046	.	.	1
**3437**	0	0.61	1	0.93046	.	.	1
**3438**	0	0.61	1	0.93046	.	.	1
**3439**	0	0.61	1	0.93046	.	.	1
**3440**	0	0.61	1	0.93046	.	.	1
**3441**	0	0.61	1	0.93046	.	.	1
**3442**	0	0.61	1	0.93046	.	.	1
**3443**	0	0.61	1	0.93046	.	.	1
**3444**	0	0.61	1	0.93046	.	.	1
**3445**	0	0.61	1	0.93046	.	.	1
**3446**	0	0.61	1	0.93046	.	.	1
**3447**	0	0.61	1	0.93046	.	.	1
**3448**	0	0.61	1	0.93046	.	.	1
**3449**	0	0.61	1	0.93046	.	.	1
**3450**	0	0.61	1	0.93046	.	.	1
**3451**	0	0.61	1	0.93046	.	.	1
**3452**	0	0.62	0	0.92979	0.92740	0.93210	1
**3453**	0	0.62	1	0.92979	.	.	1
**3454**	0	0.62	1	0.92979	.	.	1
**3455**	0	0.62	1	0.92979	.	.	1
**3456**	0	0.62	1	0.92979	.	.	1
**3457**	0	0.62	1	0.92979	.	.	1
**3458**	0	0.62	1	0.92979	.	.	1
**3459**	0	0.62	1	0.92979	.	.	1
**3460**	0	0.62	1	0.92979	.	.	1
**3461**	0	0.62	1	0.92979	.	.	1
**3462**	0	0.62	1	0.92979	.	.	1
**3463**	0	0.62	1	0.92979	.	.	1
**3464**	0	0.62	1	0.92979	.	.	1
**3465**	0	0.62	1	0.92979	.	.	1

Obs	Sub_group	fuptimey	_CENSOR_	SURVIVAL	SDF_LCL	SDF_UCL	STRATUM
3466	0	0.62	1	0.92979	.	.	1
3467	0	0.62	1	0.92979	.	.	1
3468	0	0.62	1	0.92979	.	.	1
3469	0	0.62	1	0.92979	.	.	1
3470	0	0.62	1	0.92979	.	.	1
3471	0	0.62	1	0.92979	.	.	1
3472	0	0.62	1	0.92979	.	.	1
3473	0	0.62	1	0.92979	.	.	1
3474	0	0.62	1	0.92979	.	.	1
3475	0	0.62	1	0.92979	.	.	1
3476	0	0.62	1	0.92979	.	.	1
3477	0	0.62	1	0.92979	.	.	1
3478	0	0.62	1	0.92979	.	.	1
3479	0	0.62	1	0.92979	.	.	1
3480	0	0.62	1	0.92979	.	.	1
3481	0	0.62	1	0.92979	.	.	1
3482	0	0.62	1	0.92979	.	.	1
3483	0	0.62	1	0.92979	.	.	1
3484	0	0.62	1	0.92979	.	.	1
3485	0	0.62	1	0.92979	.	.	1
3486	0	0.62	1	0.92979	.	.	1
3487	0	0.62	1	0.92979	.	.	1
3488	0	0.62	1	0.92979	.	.	1
3489	0	0.62	1	0.92979	.	.	1
3490	0	0.62	1	0.92979	.	.	1
3491	0	0.62	1	0.92979	.	.	1
3492	0	0.62	1	0.92979	.	.	1
3493	0	0.62	1	0.92979	.	.	1
3494	0	0.62	1	0.92979	.	.	1
3495	0	0.62	1	0.92979	.	.	1
3496	0	0.62	1	0.92979	.	.	1
3497	0	0.62	1	0.92979	.	.	1
3498	0	0.62	1	0.92979	.	.	1
3499	0	0.62	1	0.92979	.	.	1
3500	0	0.62	1	0.92979	.	.	1

Obs	Sub_group	fuptimey	_CENSOR_	SURVIVAL	SDF_LCL	SDF_UCL	STRATUM
3501	0	0.62	1	0.92979	.	.	1
3502	0	0.62	1	0.92979	.	.	1
3503	0	0.63	0	0.92921	0.92681	0.93153	1
3504	0	0.63	1	0.92921	.	.	1
3505	0	0.63	1	0.92921	.	.	1
3506	0	0.63	1	0.92921	.	.	1
3507	0	0.63	1	0.92921	.	.	1
3508	0	0.63	1	0.92921	.	.	1
3509	0	0.63	1	0.92921	.	.	1
3510	0	0.63	1	0.92921	.	.	1
3511	0	0.63	1	0.92921	.	.	1
3512	0	0.63	1	0.92921	.	.	1
3513	0	0.63	1	0.92921	.	.	1
3514	0	0.63	1	0.92921	.	.	1
3515	0	0.63	1	0.92921	.	.	1
3516	0	0.63	1	0.92921	.	.	1
3517	0	0.63	1	0.92921	.	.	1
3518	0	0.63	1	0.92921	.	.	1
3519	0	0.63	1	0.92921	.	.	1
3520	0	0.63	1	0.92921	.	.	1
3521	0	0.63	1	0.92921	.	.	1
3522	0	0.63	1	0.92921	.	.	1
3523	0	0.63	1	0.92921	.	.	1
3524	0	0.63	1	0.92921	.	.	1
3525	0	0.63	1	0.92921	.	.	1
3526	0	0.63	1	0.92921	.	.	1
3527	0	0.63	1	0.92921	.	.	1
3528	0	0.63	1	0.92921	.	.	1
3529	0	0.63	1	0.92921	.	.	1
3530	0	0.63	1	0.92921	.	.	1
3531	0	0.63	1	0.92921	.	.	1
3532	0	0.63	1	0.92921	.	.	1
3533	0	0.63	1	0.92921	.	.	1
3534	0	0.63	1	0.92921	.	.	1
3535	0	0.63	1	0.92921	.	.	1

Obs	Sub_group	fuptimey	_CENSOR_	SURVIVAL	SDF_LCL	SDF_UCL	STRATUM
3536	0	0.63	1	0.92921	.	.	1
3537	0	0.63	1	0.92921	.	.	1
3538	0	0.63	1	0.92921	.	.	1
3539	0	0.63	1	0.92921	.	.	1
3540	0	0.63	1	0.92921	.	.	1
3541	0	0.63	1	0.92921	.	.	1
3542	0	0.63	1	0.92921	.	.	1
3543	0	0.63	1	0.92921	.	.	1
3544	0	0.63	1	0.92921	.	.	1
3545	0	0.63	1	0.92921	.	.	1
3546	0	0.63	1	0.92921	.	.	1
3547	0	0.63	1	0.92921	.	.	1
3548	0	0.64	0	0.92877	0.92636	0.93109	1
3549	0	0.64	1	0.92877	.	.	1
3550	0	0.64	1	0.92877	.	.	1
3551	0	0.64	1	0.92877	.	.	1
3552	0	0.64	1	0.92877	.	.	1
3553	0	0.64	1	0.92877	.	.	1
3554	0	0.64	1	0.92877	.	.	1
3555	0	0.64	1	0.92877	.	.	1
3556	0	0.64	1	0.92877	.	.	1
3557	0	0.64	1	0.92877	.	.	1
3558	0	0.64	1	0.92877	.	.	1
3559	0	0.64	1	0.92877	.	.	1
3560	0	0.64	1	0.92877	.	.	1
3561	0	0.64	1	0.92877	.	.	1
3562	0	0.64	1	0.92877	.	.	1
3563	0	0.64	1	0.92877	.	.	1
3564	0	0.64	1	0.92877	.	.	1
3565	0	0.64	1	0.92877	.	.	1
3566	0	0.64	1	0.92877	.	.	1
3567	0	0.64	1	0.92877	.	.	1
3568	0	0.64	1	0.92877	.	.	1
3569	0	0.64	1	0.92877	.	.	1
3570	0	0.64	1	0.92877	.	.	1

Obs	Sub_group	fuptimey	_CENSOR_	SURVIVAL	SDF_LCL	SDF_UCL	STRATUM
3571	0	0.64	1	0.92877	.	.	1
3572	0	0.64	1	0.92877	.	.	1
3573	0	0.64	1	0.92877	.	.	1
3574	0	0.64	1	0.92877	.	.	1
3575	0	0.64	1	0.92877	.	.	1
3576	0	0.64	1	0.92877	.	.	1
3577	0	0.64	1	0.92877	.	.	1
3578	0	0.64	1	0.92877	.	.	1
3579	0	0.64	1	0.92877	.	.	1
3580	0	0.64	1	0.92877	.	.	1
3581	0	0.64	1	0.92877	.	.	1
3582	0	0.64	1	0.92877	.	.	1
3583	0	0.64	1	0.92877	.	.	1
3584	0	0.64	1	0.92877	.	.	1
3585	0	0.64	1	0.92877	.	.	1
3586	0	0.64	1	0.92877	.	.	1
3587	0	0.64	1	0.92877	.	.	1
3588	0	0.64	1	0.92877	.	.	1
3589	0	0.64	1	0.92877	.	.	1
3590	0	0.64	1	0.92877	.	.	1
3591	0	0.64	1	0.92877	.	.	1
3592	0	0.64	1	0.92877	.	.	1
3593	0	0.64	1	0.92877	.	.	1
3594	0	0.64	1	0.92877	.	.	1
3595	0	0.64	1	0.92877	.	.	1
3596	0	0.64	1	0.92877	.	.	1
3597	0	0.64	1	0.92877	.	.	1
3598	0	0.64	1	0.92877	.	.	1
3599	0	0.64	1	0.92877	.	.	1
3600	0	0.64	1	0.92877	.	.	1
3601	0	0.65	0	0.92828	0.92587	0.93061	1
3602	0	0.65	1	0.92828	.	.	1
3603	0	0.65	1	0.92828	.	.	1
3604	0	0.65	1	0.92828	.	.	1
3605	0	0.65	1	0.92828	.	.	1

Obs	Sub_group	fuptimey	_CENSOR_	SURVIVAL	SDF_LCL	SDF_UCL	STRATUM
3606	0	0.65	1	0.92828	.	.	1
3607	0	0.65	1	0.92828	.	.	1
3608	0	0.65	1	0.92828	.	.	1
3609	0	0.65	1	0.92828	.	.	1
3610	0	0.65	1	0.92828	.	.	1
3611	0	0.65	1	0.92828	.	.	1
3612	0	0.65	1	0.92828	.	.	1
3613	0	0.65	1	0.92828	.	.	1
3614	0	0.65	1	0.92828	.	.	1
3615	0	0.65	1	0.92828	.	.	1
3616	0	0.65	1	0.92828	.	.	1
3617	0	0.65	1	0.92828	.	.	1
3618	0	0.65	1	0.92828	.	.	1
3619	0	0.65	1	0.92828	.	.	1
3620	0	0.65	1	0.92828	.	.	1
3621	0	0.65	1	0.92828	.	.	1
3622	0	0.65	1	0.92828	.	.	1
3623	0	0.65	1	0.92828	.	.	1
3624	0	0.65	1	0.92828	.	.	1
3625	0	0.65	1	0.92828	.	.	1
3626	0	0.65	1	0.92828	.	.	1
3627	0	0.65	1	0.92828	.	.	1
3628	0	0.65	1	0.92828	.	.	1
3629	0	0.65	1	0.92828	.	.	1
3630	0	0.65	1	0.92828	.	.	1
3631	0	0.65	1	0.92828	.	.	1
3632	0	0.65	1	0.92828	.	.	1
3633	0	0.65	1	0.92828	.	.	1
3634	0	0.65	1	0.92828	.	.	1
3635	0	0.65	1	0.92828	.	.	1
3636	0	0.65	1	0.92828	.	.	1
3637	0	0.65	1	0.92828	.	.	1
3638	0	0.65	1	0.92828	.	.	1
3639	0	0.65	1	0.92828	.	.	1
3640	0	0.65	1	0.92828	.	.	1

Obs	Sub_group	fuptimey	_CENSOR_	SURVIVAL	SDF_LCL	SDF_UCL	STRATUM
3641	0	0.65	1	0.92828	.	.	1
3642	0	0.65	1	0.92828	.	.	1
3643	0	0.65	1	0.92828	.	.	1
3644	0	0.65	1	0.92828	.	.	1
3645	0	0.66	0	0.92772	0.92530	0.93006	1
3646	0	0.66	1	0.92772	.	.	1
3647	0	0.66	1	0.92772	.	.	1
3648	0	0.66	1	0.92772	.	.	1
3649	0	0.66	1	0.92772	.	.	1
3650	0	0.66	1	0.92772	.	.	1
3651	0	0.66	1	0.92772	.	.	1
3652	0	0.66	1	0.92772	.	.	1
3653	0	0.66	1	0.92772	.	.	1
3654	0	0.66	1	0.92772	.	.	1
3655	0	0.66	1	0.92772	.	.	1
3656	0	0.66	1	0.92772	.	.	1
3657	0	0.66	1	0.92772	.	.	1
3658	0	0.66	1	0.92772	.	.	1
3659	0	0.66	1	0.92772	.	.	1
3660	0	0.66	1	0.92772	.	.	1
3661	0	0.66	1	0.92772	.	.	1
3662	0	0.66	1	0.92772	.	.	1
3663	0	0.66	1	0.92772	.	.	1
3664	0	0.66	1	0.92772	.	.	1
3665	0	0.66	1	0.92772	.	.	1
3666	0	0.66	1	0.92772	.	.	1
3667	0	0.66	1	0.92772	.	.	1
3668	0	0.66	1	0.92772	.	.	1
3669	0	0.66	1	0.92772	.	.	1
3670	0	0.66	1	0.92772	.	.	1
3671	0	0.66	1	0.92772	.	.	1
3672	0	0.66	1	0.92772	.	.	1
3673	0	0.66	1	0.92772	.	.	1
3674	0	0.66	1	0.92772	.	.	1
3675	0	0.66	1	0.92772	.	.	1

Obs	Sub_group	fuptimey	_CENSOR_	SURVIVAL	SDF_LCL	SDF_UCL	STRATUM
3676	0	0.66	1	0.92772	.	.	1
3677	0	0.66	1	0.92772	.	.	1
3678	0	0.66	1	0.92772	.	.	1
3679	0	0.66	1	0.92772	.	.	1
3680	0	0.66	1	0.92772	.	.	1
3681	0	0.66	1	0.92772	.	.	1
3682	0	0.66	1	0.92772	.	.	1
3683	0	0.66	1	0.92772	.	.	1
3684	0	0.66	1	0.92772	.	.	1
3685	0	0.66	1	0.92772	.	.	1
3686	0	0.66	1	0.92772	.	.	1
3687	0	0.66	1	0.92772	.	.	1
3688	0	0.66	1	0.92772	.	.	1
3689	0	0.66	1	0.92772	.	.	1
3690	0	0.66	1	0.92772	.	.	1
3691	0	0.66	1	0.92772	.	.	1
3692	0	0.66	1	0.92772	.	.	1
3693	0	0.66	1	0.92772	.	.	1
3694	0	0.66	1	0.92772	.	.	1
3695	0	0.66	1	0.92772	.	.	1
3696	0	0.66	1	0.92772	.	.	1
3697	0	0.67	0	0.92727	0.92485	0.92963	1
3698	0	0.67	1	0.92727	.	.	1
3699	0	0.67	1	0.92727	.	.	1
3700	0	0.67	1	0.92727	.	.	1
3701	0	0.67	1	0.92727	.	.	1
3702	0	0.67	1	0.92727	.	.	1
3703	0	0.67	1	0.92727	.	.	1
3704	0	0.67	1	0.92727	.	.	1
3705	0	0.67	1	0.92727	.	.	1
3706	0	0.67	1	0.92727	.	.	1
3707	0	0.67	1	0.92727	.	.	1
3708	0	0.67	1	0.92727	.	.	1
3709	0	0.67	1	0.92727	.	.	1
3710	0	0.67	1	0.92727	.	.	1

Obs	Sub_group	fuptimey	_CENSOR_	SURVIVAL	SDF_LCL	SDF_UCL	STRATUM
**3711**	0	0.67	1	0.92727	.	.	1
**3712**	0	0.67	1	0.92727	.	.	1
**3713**	0	0.67	1	0.92727	.	.	1
**3714**	0	0.67	1	0.92727	.	.	1
**3715**	0	0.67	1	0.92727	.	.	1
**3716**	0	0.67	1	0.92727	.	.	1
**3717**	0	0.67	1	0.92727	.	.	1
**3718**	0	0.67	1	0.92727	.	.	1
**3719**	0	0.67	1	0.92727	.	.	1
**3720**	0	0.67	1	0.92727	.	.	1
**3721**	0	0.67	1	0.92727	.	.	1
**3722**	0	0.67	1	0.92727	.	.	1
**3723**	0	0.67	1	0.92727	.	.	1
**3724**	0	0.67	1	0.92727	.	.	1
**3725**	0	0.67	1	0.92727	.	.	1
**3726**	0	0.67	1	0.92727	.	.	1
**3727**	0	0.68	0	0.92699	0.92456	0.92935	1
**3728**	0	0.68	1	0.92699	.	.	1
**3729**	0	0.68	1	0.92699	.	.	1
**3730**	0	0.68	1	0.92699	.	.	1
**3731**	0	0.68	1	0.92699	.	.	1
**3732**	0	0.68	1	0.92699	.	.	1
**3733**	0	0.68	1	0.92699	.	.	1
**3734**	0	0.68	1	0.92699	.	.	1
**3735**	0	0.68	1	0.92699	.	.	1
**3736**	0	0.68	1	0.92699	.	.	1
**3737**	0	0.68	1	0.92699	.	.	1
**3738**	0	0.68	1	0.92699	.	.	1
**3739**	0	0.68	1	0.92699	.	.	1
**3740**	0	0.68	1	0.92699	.	.	1
**3741**	0	0.68	1	0.92699	.	.	1
**3742**	0	0.68	1	0.92699	.	.	1
**3743**	0	0.68	1	0.92699	.	.	1
**3744**	0	0.68	1	0.92699	.	.	1
**3745**	0	0.68	1	0.92699	.	.	1

Obs	Sub_group	fuptimey	_CENSOR_	SURVIVAL	SDF_LCL	SDF_UCL	STRATUM
3746	0	0.68	1	0.92699	.	.	1
3747	0	0.68	1	0.92699	.	.	1
3748	0	0.68	1	0.92699	.	.	1
3749	0	0.68	1	0.92699	.	.	1
3750	0	0.68	1	0.92699	.	.	1
3751	0	0.68	1	0.92699	.	.	1
3752	0	0.68	1	0.92699	.	.	1
3753	0	0.68	1	0.92699	.	.	1
3754	0	0.68	1	0.92699	.	.	1
3755	0	0.68	1	0.92699	.	.	1
3756	0	0.68	1	0.92699	.	.	1
3757	0	0.68	1	0.92699	.	.	1
3758	0	0.68	1	0.92699	.	.	1
3759	0	0.68	1	0.92699	.	.	1
3760	0	0.68	1	0.92699	.	.	1
3761	0	0.68	1	0.92699	.	.	1
3762	0	0.68	1	0.92699	.	.	1
3763	0	0.68	1	0.92699	.	.	1
3764	0	0.68	1	0.92699	.	.	1
3765	0	0.68	1	0.92699	.	.	1
3766	0	0.68	1	0.92699	.	.	1
3767	0	0.68	1	0.92699	.	.	1
3768	0	0.68	1	0.92699	.	.	1
3769	0	0.68	1	0.92699	.	.	1
3770	0	0.68	1	0.92699	.	.	1
3771	0	0.68	1	0.92699	.	.	1
3772	0	0.69	0	0.92636	0.92392	0.92873	1
3773	0	0.69	1	0.92636	.	.	1
3774	0	0.69	1	0.92636	.	.	1
3775	0	0.69	1	0.92636	.	.	1
3776	0	0.69	1	0.92636	.	.	1
3777	0	0.69	1	0.92636	.	.	1
3778	0	0.69	1	0.92636	.	.	1
3779	0	0.69	1	0.92636	.	.	1
3780	0	0.69	1	0.92636	.	.	1

Obs	Sub_group	fuptimey	_CENSOR_	SURVIVAL	SDF_LCL	SDF_UCL	STRATUM
3781	0	0.69	1	0.92636	.	.	1
3782	0	0.69	1	0.92636	.	.	1
3783	0	0.69	1	0.92636	.	.	1
3784	0	0.69	1	0.92636	.	.	1
3785	0	0.69	1	0.92636	.	.	1
3786	0	0.69	1	0.92636	.	.	1
3787	0	0.69	1	0.92636	.	.	1
3788	0	0.69	1	0.92636	.	.	1
3789	0	0.69	1	0.92636	.	.	1
3790	0	0.69	1	0.92636	.	.	1
3791	0	0.69	1	0.92636	.	.	1
3792	0	0.69	1	0.92636	.	.	1
3793	0	0.69	1	0.92636	.	.	1
3794	0	0.69	1	0.92636	.	.	1
3795	0	0.69	1	0.92636	.	.	1
3796	0	0.69	1	0.92636	.	.	1
3797	0	0.70	0	0.92606	0.92361	0.92843	1
3798	0	0.70	1	0.92606	.	.	1
3799	0	0.70	1	0.92606	.	.	1
3800	0	0.70	1	0.92606	.	.	1
3801	0	0.70	1	0.92606	.	.	1
3802	0	0.70	1	0.92606	.	.	1
3803	0	0.70	1	0.92606	.	.	1
3804	0	0.70	1	0.92606	.	.	1
3805	0	0.70	1	0.92606	.	.	1
3806	0	0.70	1	0.92606	.	.	1
3807	0	0.70	1	0.92606	.	.	1
3808	0	0.70	1	0.92606	.	.	1
3809	0	0.70	1	0.92606	.	.	1
3810	0	0.70	1	0.92606	.	.	1
3811	0	0.70	1	0.92606	.	.	1
3812	0	0.70	1	0.92606	.	.	1
3813	0	0.70	1	0.92606	.	.	1
3814	0	0.70	1	0.92606	.	.	1
3815	0	0.70	1	0.92606	.	.	1

Obs	Sub_group	fuptimey	_CENSOR_	SURVIVAL	SDF_LCL	SDF_UCL	STRATUM
3816	0	0.70	1	0.92606	.	.	1
3817	0	0.70	1	0.92606	.	.	1
3818	0	0.70	1	0.92606	.	.	1
3819	0	0.70	1	0.92606	.	.	1
3820	0	0.70	1	0.92606	.	.	1
3821	0	0.70	1	0.92606	.	.	1
3822	0	0.70	1	0.92606	.	.	1
3823	0	0.70	1	0.92606	.	.	1
3824	0	0.70	1	0.92606	.	.	1
3825	0	0.70	1	0.92606	.	.	1
3826	0	0.70	1	0.92606	.	.	1
3827	0	0.70	1	0.92606	.	.	1
3828	0	0.70	1	0.92606	.	.	1
3829	0	0.70	1	0.92606	.	.	1
3830	0	0.70	1	0.92606	.	.	1
3831	0	0.70	1	0.92606	.	.	1
3832	0	0.70	1	0.92606	.	.	1
3833	0	0.70	1	0.92606	.	.	1
3834	0	0.70	1	0.92606	.	.	1
3835	0	0.70	1	0.92606	.	.	1
3836	0	0.71	0	0.92547	0.92302	0.92785	1
3837	0	0.71	1	0.92547	.	.	1
3838	0	0.71	1	0.92547	.	.	1
3839	0	0.71	1	0.92547	.	.	1
3840	0	0.71	1	0.92547	.	.	1
3841	0	0.71	1	0.92547	.	.	1
3842	0	0.71	1	0.92547	.	.	1
3843	0	0.71	1	0.92547	.	.	1
3844	0	0.71	1	0.92547	.	.	1
3845	0	0.71	1	0.92547	.	.	1
3846	0	0.71	1	0.92547	.	.	1
3847	0	0.71	1	0.92547	.	.	1
3848	0	0.71	1	0.92547	.	.	1
3849	0	0.71	1	0.92547	.	.	1
3850	0	0.71	1	0.92547	.	.	1

Obs	Sub_group	fuptimey	_CENSOR_	SURVIVAL	SDF_LCL	SDF_UCL	STRATUM
3851	0	0.71	1	0.92547	.	.	1
3852	0	0.71	1	0.92547	.	.	1
3853	0	0.71	1	0.92547	.	.	1
3854	0	0.71	1	0.92547	.	.	1
3855	0	0.71	1	0.92547	.	.	1
3856	0	0.71	1	0.92547	.	.	1
3857	0	0.71	1	0.92547	.	.	1
3858	0	0.71	1	0.92547	.	.	1
3859	0	0.71	1	0.92547	.	.	1
3860	0	0.71	1	0.92547	.	.	1
3861	0	0.71	1	0.92547	.	.	1
3862	0	0.71	1	0.92547	.	.	1
3863	0	0.71	1	0.92547	.	.	1
3864	0	0.71	1	0.92547	.	.	1
3865	0	0.71	1	0.92547	.	.	1
3866	0	0.71	1	0.92547	.	.	1
3867	0	0.71	1	0.92547	.	.	1
3868	0	0.71	1	0.92547	.	.	1
3869	0	0.71	1	0.92547	.	.	1
3870	0	0.71	1	0.92547	.	.	1
3871	0	0.71	1	0.92547	.	.	1
3872	0	0.71	1	0.92547	.	.	1
3873	0	0.71	1	0.92547	.	.	1
3874	0	0.71	1	0.92547	.	.	1
3875	0	0.71	1	0.92547	.	.	1
3876	0	0.71	1	0.92547	.	.	1
3877	0	0.71	1	0.92547	.	.	1
3878	0	0.71	1	0.92547	.	.	1
3879	0	0.71	1	0.92547	.	.	1
3880	0	0.71	1	0.92547	.	.	1
3881	0	0.71	1	0.92547	.	.	1
3882	0	0.71	1	0.92547	.	.	1
3883	0	0.72	0	0.92512	0.92266	0.92751	1
3884	0	0.72	1	0.92512	.	.	1
3885	0	0.72	1	0.92512	.	.	1

Obs	Sub_group	fuptimey	_CENSOR_	SURVIVAL	SDF_LCL	SDF_UCL	STRATUM
**3886**	0	0.72	1	0.92512	.	.	1
**3887**	0	0.72	1	0.92512	.	.	1
**3888**	0	0.72	1	0.92512	.	.	1
**3889**	0	0.72	1	0.92512	.	.	1
**3890**	0	0.72	1	0.92512	.	.	1
**3891**	0	0.72	1	0.92512	.	.	1
**3892**	0	0.72	1	0.92512	.	.	1
**3893**	0	0.72	1	0.92512	.	.	1
**3894**	0	0.72	1	0.92512	.	.	1
**3895**	0	0.72	1	0.92512	.	.	1
**3896**	0	0.72	1	0.92512	.	.	1
**3897**	0	0.72	1	0.92512	.	.	1
**3898**	0	0.72	1	0.92512	.	.	1
**3899**	0	0.72	1	0.92512	.	.	1
**3900**	0	0.72	1	0.92512	.	.	1
**3901**	0	0.72	1	0.92512	.	.	1
**3902**	0	0.72	1	0.92512	.	.	1
**3903**	0	0.72	1	0.92512	.	.	1
**3904**	0	0.72	1	0.92512	.	.	1
**3905**	0	0.72	1	0.92512	.	.	1
**3906**	0	0.72	1	0.92512	.	.	1
**3907**	0	0.72	1	0.92512	.	.	1
**3908**	0	0.72	1	0.92512	.	.	1
**3909**	0	0.72	1	0.92512	.	.	1
**3910**	0	0.72	1	0.92512	.	.	1
**3911**	0	0.72	1	0.92512	.	.	1
**3912**	0	0.72	1	0.92512	.	.	1
**3913**	0	0.72	1	0.92512	.	.	1
**3914**	0	0.72	1	0.92512	.	.	1
**3915**	0	0.72	1	0.92512	.	.	1
**3916**	0	0.72	1	0.92512	.	.	1
**3917**	0	0.72	1	0.92512	.	.	1
**3918**	0	0.73	0	0.92479	0.92232	0.92718	1
**3919**	0	0.73	1	0.92479	.	.	1
**3920**	0	0.73	1	0.92479	.	.	1

Obs	Sub_group	fuptimey	_CENSOR_	SURVIVAL	SDF_LCL	SDF_UCL	STRATUM
3921	0	0.73	1	0.92479	.	.	1
3922	0	0.73	1	0.92479	.	.	1
3923	0	0.73	1	0.92479	.	.	1
3924	0	0.73	1	0.92479	.	.	1
3925	0	0.73	1	0.92479	.	.	1
3926	0	0.73	1	0.92479	.	.	1
3927	0	0.73	1	0.92479	.	.	1
3928	0	0.73	1	0.92479	.	.	1
3929	0	0.73	1	0.92479	.	.	1
3930	0	0.73	1	0.92479	.	.	1
3931	0	0.73	1	0.92479	.	.	1
3932	0	0.73	1	0.92479	.	.	1
3933	0	0.73	1	0.92479	.	.	1
3934	0	0.73	1	0.92479	.	.	1
3935	0	0.73	1	0.92479	.	.	1
3936	0	0.73	1	0.92479	.	.	1
3937	0	0.73	1	0.92479	.	.	1
3938	0	0.73	1	0.92479	.	.	1
3939	0	0.73	1	0.92479	.	.	1
3940	0	0.73	1	0.92479	.	.	1
3941	0	0.73	1	0.92479	.	.	1
3942	0	0.73	1	0.92479	.	.	1
3943	0	0.73	1	0.92479	.	.	1
3944	0	0.73	1	0.92479	.	.	1
3945	0	0.73	1	0.92479	.	.	1
3946	0	0.73	1	0.92479	.	.	1
3947	0	0.74	0	0.92442	0.92194	0.92681	1
3948	0	0.74	1	0.92442	.	.	1
3949	0	0.74	1	0.92442	.	.	1
3950	0	0.74	1	0.92442	.	.	1
3951	0	0.74	1	0.92442	.	.	1
3952	0	0.74	1	0.92442	.	.	1
3953	0	0.74	1	0.92442	.	.	1
3954	0	0.74	1	0.92442	.	.	1
3955	0	0.74	1	0.92442	.	.	1

Obs	Sub_group	fuptimey	_CENSOR_	SURVIVAL	SDF_LCL	SDF_UCL	STRATUM
3956	0	0.74	1	0.92442	.	.	1
3957	0	0.74	1	0.92442	.	.	1
3958	0	0.74	1	0.92442	.	.	1
3959	0	0.74	1	0.92442	.	.	1
3960	0	0.74	1	0.92442	.	.	1
3961	0	0.74	1	0.92442	.	.	1
3962	0	0.74	1	0.92442	.	.	1
3963	0	0.74	1	0.92442	.	.	1
3964	0	0.74	1	0.92442	.	.	1
3965	0	0.74	1	0.92442	.	.	1
3966	0	0.74	1	0.92442	.	.	1
3967	0	0.74	1	0.92442	.	.	1
3968	0	0.74	1	0.92442	.	.	1
3969	0	0.74	1	0.92442	.	.	1
3970	0	0.74	1	0.92442	.	.	1
3971	0	0.74	1	0.92442	.	.	1
3972	0	0.74	1	0.92442	.	.	1
3973	0	0.74	1	0.92442	.	.	1
3974	0	0.74	1	0.92442	.	.	1
3975	0	0.74	1	0.92442	.	.	1
3976	0	0.74	1	0.92442	.	.	1
3977	0	0.74	1	0.92442	.	.	1
3978	0	0.74	1	0.92442	.	.	1
3979	0	0.74	1	0.92442	.	.	1
3980	0	0.74	1	0.92442	.	.	1
3981	0	0.74	1	0.92442	.	.	1
3982	0	0.74	1	0.92442	.	.	1
3983	0	0.74	1	0.92442	.	.	1
3984	0	0.74	1	0.92442	.	.	1
3985	0	0.74	1	0.92442	.	.	1
3986	0	0.74	1	0.92442	.	.	1
3987	0	0.75	0	0.92380	0.92132	0.92621	1
3988	0	0.75	1	0.92380	.	.	1
3989	0	0.75	1	0.92380	.	.	1
3990	0	0.75	1	0.92380	.	.	1

Obs	Sub_group	fuptimey	_CENSOR_	SURVIVAL	SDF_LCL	SDF_UCL	STRATUM
3991	0	0.75	1	0.92380	.	.	1
3992	0	0.75	1	0.92380	.	.	1
3993	0	0.75	1	0.92380	.	.	1
3994	0	0.75	1	0.92380	.	.	1
3995	0	0.75	1	0.92380	.	.	1
3996	0	0.75	1	0.92380	.	.	1
3997	0	0.75	1	0.92380	.	.	1
3998	0	0.75	1	0.92380	.	.	1
3999	0	0.75	1	0.92380	.	.	1
4000	0	0.75	1	0.92380	.	.	1
4001	0	0.75	1	0.92380	.	.	1
4002	0	0.75	1	0.92380	.	.	1
4003	0	0.75	1	0.92380	.	.	1
4004	0	0.75	1	0.92380	.	.	1
4005	0	0.75	1	0.92380	.	.	1
4006	0	0.75	1	0.92380	.	.	1
4007	0	0.75	1	0.92380	.	.	1
4008	0	0.75	1	0.92380	.	.	1
4009	0	0.75	1	0.92380	.	.	1
4010	0	0.75	1	0.92380	.	.	1
4011	0	0.75	1	0.92380	.	.	1
4012	0	0.75	1	0.92380	.	.	1
4013	0	0.75	1	0.92380	.	.	1
4014	0	0.75	1	0.92380	.	.	1
4015	0	0.75	1	0.92380	.	.	1
4016	0	0.75	1	0.92380	.	.	1
4017	0	0.75	1	0.92380	.	.	1
4018	0	0.75	1	0.92380	.	.	1
4019	0	0.75	1	0.92380	.	.	1
4020	0	0.75	1	0.92380	.	.	1
4021	0	0.75	1	0.92380	.	.	1
4022	0	0.75	1	0.92380	.	.	1
4023	0	0.75	1	0.92380	.	.	1
4024	0	0.75	1	0.92380	.	.	1
4025	0	0.75	1	0.92380	.	.	1

Obs	Sub_group	fuptimey	_CENSOR_	SURVIVAL	SDF_LCL	SDF_UCL	STRATUM
**4026**	0	0.75	1	0.92380	.	.	1
**4027**	0	0.75	1	0.92380	.	.	1
**4028**	0	0.75	1	0.92380	.	.	1
**4029**	0	0.75	1	0.92380	.	.	1
**4030**	0	0.75	1	0.92380	.	.	1
**4031**	0	0.75	1	0.92380	.	.	1
**4032**	0	0.76	0	0.92340	0.92092	0.92582	1
**4033**	0	0.76	1	0.92340	.	.	1
**4034**	0	0.76	1	0.92340	.	.	1
**4035**	0	0.76	1	0.92340	.	.	1
**4036**	0	0.76	1	0.92340	.	.	1
**4037**	0	0.76	1	0.92340	.	.	1
**4038**	0	0.76	1	0.92340	.	.	1
**4039**	0	0.76	1	0.92340	.	.	1
**4040**	0	0.76	1	0.92340	.	.	1
**4041**	0	0.76	1	0.92340	.	.	1
**4042**	0	0.76	1	0.92340	.	.	1
**4043**	0	0.76	1	0.92340	.	.	1
**4044**	0	0.76	1	0.92340	.	.	1
**4045**	0	0.76	1	0.92340	.	.	1
**4046**	0	0.76	1	0.92340	.	.	1
**4047**	0	0.76	1	0.92340	.	.	1
**4048**	0	0.76	1	0.92340	.	.	1
**4049**	0	0.76	1	0.92340	.	.	1
**4050**	0	0.76	1	0.92340	.	.	1
**4051**	0	0.76	1	0.92340	.	.	1
**4052**	0	0.76	1	0.92340	.	.	1
**4053**	0	0.76	1	0.92340	.	.	1
**4054**	0	0.76	1	0.92340	.	.	1
**4055**	0	0.76	1	0.92340	.	.	1
**4056**	0	0.76	1	0.92340	.	.	1
**4057**	0	0.76	1	0.92340	.	.	1
**4058**	0	0.76	1	0.92340	.	.	1
**4059**	0	0.76	1	0.92340	.	.	1
**4060**	0	0.76	1	0.92340	.	.	1

Obs	Sub_group	fuptimey	_CENSOR_	SURVIVAL	SDF_LCL	SDF_UCL	STRATUM
4061	0	0.76	1	0.92340	.	.	1
4062	0	0.76	1	0.92340	.	.	1
4063	0	0.76	1	0.92340	.	.	1
4064	0	0.76	1	0.92340	.	.	1
4065	0	0.76	1	0.92340	.	.	1
4066	0	0.76	1	0.92340	.	.	1
4067	0	0.76	1	0.92340	.	.	1
4068	0	0.76	1	0.92340	.	.	1
4069	0	0.76	1	0.92340	.	.	1
4070	0	0.76	1	0.92340	.	.	1
4071	0	0.76	1	0.92340	.	.	1
4072	0	0.76	1	0.92340	.	.	1
4073	0	0.76	1	0.92340	.	.	1
4074	0	0.77	0	0.92282	0.92032	0.92524	1
4075	0	0.77	1	0.92282	.	.	1
4076	0	0.77	1	0.92282	.	.	1
4077	0	0.77	1	0.92282	.	.	1
4078	0	0.77	1	0.92282	.	.	1
4079	0	0.77	1	0.92282	.	.	1
4080	0	0.77	1	0.92282	.	.	1
4081	0	0.77	1	0.92282	.	.	1
4082	0	0.77	1	0.92282	.	.	1
4083	0	0.77	1	0.92282	.	.	1
4084	0	0.77	1	0.92282	.	.	1
4085	0	0.77	1	0.92282	.	.	1
4086	0	0.77	1	0.92282	.	.	1
4087	0	0.77	1	0.92282	.	.	1
4088	0	0.77	1	0.92282	.	.	1
4089	0	0.77	1	0.92282	.	.	1
4090	0	0.77	1	0.92282	.	.	1
4091	0	0.77	1	0.92282	.	.	1
4092	0	0.77	1	0.92282	.	.	1
4093	0	0.77	1	0.92282	.	.	1
4094	0	0.77	1	0.92282	.	.	1
4095	0	0.77	1	0.92282	.	.	1

Obs	Sub_group	fuptimey	_CENSOR_	SURVIVAL	SDF_LCL	SDF_UCL	STRATUM
**4096**	0	0.77	1	0.92282	.	.	1
**4097**	0	0.77	1	0.92282	.	.	1
**4098**	0	0.77	1	0.92282	.	.	1
**4099**	0	0.77	1	0.92282	.	.	1
**4100**	0	0.77	1	0.92282	.	.	1
**4101**	0	0.77	1	0.92282	.	.	1
**4102**	0	0.77	1	0.92282	.	.	1
**4103**	0	0.77	1	0.92282	.	.	1
**4104**	0	0.77	1	0.92282	.	.	1
**4105**	0	0.77	1	0.92282	.	.	1
**4106**	0	0.77	1	0.92282	.	.	1
**4107**	0	0.77	1	0.92282	.	.	1
**4108**	0	0.77	1	0.92282	.	.	1
**4109**	0	0.77	1	0.92282	.	.	1
**4110**	0	0.77	1	0.92282	.	.	1
**4111**	0	0.77	1	0.92282	.	.	1
**4112**	0	0.77	1	0.92282	.	.	1
**4113**	0	0.77	1	0.92282	.	.	1
**4114**	0	0.77	1	0.92282	.	.	1
**4115**	0	0.77	1	0.92282	.	.	1
**4116**	0	0.77	1	0.92282	.	.	1
**4117**	0	0.77	1	0.92282	.	.	1
**4118**	0	0.77	1	0.92282	.	.	1
**4119**	0	0.77	1	0.92282	.	.	1
**4120**	0	0.78	0	0.92234	0.91984	0.92477	1
**4121**	0	0.78	1	0.92234	.	.	1
**4122**	0	0.78	1	0.92234	.	.	1
**4123**	0	0.78	1	0.92234	.	.	1
**4124**	0	0.78	1	0.92234	.	.	1
**4125**	0	0.78	1	0.92234	.	.	1
**4126**	0	0.78	1	0.92234	.	.	1
**4127**	0	0.78	1	0.92234	.	.	1
**4128**	0	0.78	1	0.92234	.	.	1
**4129**	0	0.78	1	0.92234	.	.	1
**4130**	0	0.78	1	0.92234	.	.	1

Obs	Sub_group	fuptimey	_CENSOR_	SURVIVAL	SDF_LCL	SDF_UCL	STRATUM
4131	0	0.78	1	0.92234	.	.	1
4132	0	0.78	1	0.92234	.	.	1
4133	0	0.78	1	0.92234	.	.	1
4134	0	0.78	1	0.92234	.	.	1
4135	0	0.78	1	0.92234	.	.	1
4136	0	0.78	1	0.92234	.	.	1
4137	0	0.78	1	0.92234	.	.	1
4138	0	0.78	1	0.92234	.	.	1
4139	0	0.78	1	0.92234	.	.	1
4140	0	0.78	1	0.92234	.	.	1
4141	0	0.78	1	0.92234	.	.	1
4142	0	0.78	1	0.92234	.	.	1
4143	0	0.78	1	0.92234	.	.	1
4144	0	0.78	1	0.92234	.	.	1
4145	0	0.78	1	0.92234	.	.	1
4146	0	0.78	1	0.92234	.	.	1
4147	0	0.78	1	0.92234	.	.	1
4148	0	0.78	1	0.92234	.	.	1
4149	0	0.78	1	0.92234	.	.	1
4150	0	0.78	1	0.92234	.	.	1
4151	0	0.78	1	0.92234	.	.	1
4152	0	0.78	1	0.92234	.	.	1
4153	0	0.78	1	0.92234	.	.	1
4154	0	0.78	1	0.92234	.	.	1
4155	0	0.78	1	0.92234	.	.	1
4156	0	0.78	1	0.92234	.	.	1
4157	0	0.78	1	0.92234	.	.	1
4158	0	0.79	0	0.92194	0.91943	0.92438	1
4159	0	0.79	1	0.92194	.	.	1
4160	0	0.79	1	0.92194	.	.	1
4161	0	0.79	1	0.92194	.	.	1
4162	0	0.79	1	0.92194	.	.	1
4163	0	0.79	1	0.92194	.	.	1
4164	0	0.79	1	0.92194	.	.	1
4165	0	0.79	1	0.92194	.	.	1

Obs	Sub_group	fuptimey	_CENSOR_	SURVIVAL	SDF_LCL	SDF_UCL	STRATUM
4166	0	0.79	1	0.92194	.	.	1
4167	0	0.79	1	0.92194	.	.	1
4168	0	0.79	1	0.92194	.	.	1
4169	0	0.79	1	0.92194	.	.	1
4170	0	0.79	1	0.92194	.	.	1
4171	0	0.79	1	0.92194	.	.	1
4172	0	0.79	1	0.92194	.	.	1
4173	0	0.79	1	0.92194	.	.	1
4174	0	0.79	1	0.92194	.	.	1
4175	0	0.79	1	0.92194	.	.	1
4176	0	0.79	1	0.92194	.	.	1
4177	0	0.79	1	0.92194	.	.	1
4178	0	0.79	1	0.92194	.	.	1
4179	0	0.79	1	0.92194	.	.	1
4180	0	0.79	1	0.92194	.	.	1
4181	0	0.79	1	0.92194	.	.	1
4182	0	0.79	1	0.92194	.	.	1
4183	0	0.79	1	0.92194	.	.	1
4184	0	0.79	1	0.92194	.	.	1
4185	0	0.79	1	0.92194	.	.	1
4186	0	0.79	1	0.92194	.	.	1
4187	0	0.79	1	0.92194	.	.	1
4188	0	0.79	1	0.92194	.	.	1
4189	0	0.79	1	0.92194	.	.	1
4190	0	0.79	1	0.92194	.	.	1
4191	0	0.79	1	0.92194	.	.	1
4192	0	0.79	1	0.92194	.	.	1
4193	0	0.79	1	0.92194	.	.	1
4194	0	0.79	1	0.92194	.	.	1
4195	0	0.80	0	0.92147	0.91895	0.92391	1
4196	0	0.80	1	0.92147	.	.	1
4197	0	0.80	1	0.92147	.	.	1
4198	0	0.80	1	0.92147	.	.	1
4199	0	0.80	1	0.92147	.	.	1
4200	0	0.80	1	0.92147	.	.	1

Obs	Sub_group	fuptimey	_CENSOR_	SURVIVAL	SDF_LCL	SDF_UCL	STRATUM
4201	0	0.80	1	0.92147	.	.	1
4202	0	0.80	1	0.92147	.	.	1
4203	0	0.80	1	0.92147	.	.	1
4204	0	0.80	1	0.92147	.	.	1
4205	0	0.80	1	0.92147	.	.	1
4206	0	0.80	1	0.92147	.	.	1
4207	0	0.80	1	0.92147	.	.	1
4208	0	0.80	1	0.92147	.	.	1
4209	0	0.80	1	0.92147	.	.	1
4210	0	0.80	1	0.92147	.	.	1
4211	0	0.80	1	0.92147	.	.	1
4212	0	0.80	1	0.92147	.	.	1
4213	0	0.80	1	0.92147	.	.	1
4214	0	0.80	1	0.92147	.	.	1
4215	0	0.80	1	0.92147	.	.	1
4216	0	0.80	1	0.92147	.	.	1
4217	0	0.80	1	0.92147	.	.	1
4218	0	0.80	1	0.92147	.	.	1
4219	0	0.80	1	0.92147	.	.	1
4220	0	0.80	1	0.92147	.	.	1
4221	0	0.80	1	0.92147	.	.	1
4222	0	0.80	1	0.92147	.	.	1
4223	0	0.80	1	0.92147	.	.	1
4224	0	0.80	1	0.92147	.	.	1
4225	0	0.80	1	0.92147	.	.	1
4226	0	0.80	1	0.92147	.	.	1
4227	0	0.81	0	0.92093	0.91840	0.92338	1
4228	0	0.81	1	0.92093	.	.	1
4229	0	0.81	1	0.92093	.	.	1
4230	0	0.81	1	0.92093	.	.	1
4231	0	0.81	1	0.92093	.	.	1
4232	0	0.81	1	0.92093	.	.	1
4233	0	0.81	1	0.92093	.	.	1
4234	0	0.81	1	0.92093	.	.	1
4235	0	0.81	1	0.92093	.	.	1

Obs	Sub_group	fuptimey	_CENSOR_	SURVIVAL	SDF_LCL	SDF_UCL	STRATUM
4236	0	0.81	1	0.92093	.	.	1
4237	0	0.81	1	0.92093	.	.	1
4238	0	0.81	1	0.92093	.	.	1
4239	0	0.81	1	0.92093	.	.	1
4240	0	0.81	1	0.92093	.	.	1
4241	0	0.81	1	0.92093	.	.	1
4242	0	0.81	1	0.92093	.	.	1
4243	0	0.81	1	0.92093	.	.	1
4244	0	0.81	1	0.92093	.	.	1
4245	0	0.81	1	0.92093	.	.	1
4246	0	0.81	1	0.92093	.	.	1
4247	0	0.81	1	0.92093	.	.	1
4248	0	0.81	1	0.92093	.	.	1
4249	0	0.81	1	0.92093	.	.	1
4250	0	0.81	1	0.92093	.	.	1
4251	0	0.81	1	0.92093	.	.	1
4252	0	0.81	1	0.92093	.	.	1
4253	0	0.81	1	0.92093	.	.	1
4254	0	0.81	1	0.92093	.	.	1
4255	0	0.81	1	0.92093	.	.	1
4256	0	0.81	1	0.92093	.	.	1
4257	0	0.81	1	0.92093	.	.	1
4258	0	0.81	1	0.92093	.	.	1
4259	0	0.81	1	0.92093	.	.	1
4260	0	0.81	1	0.92093	.	.	1
4261	0	0.81	1	0.92093	.	.	1
4262	0	0.81	1	0.92093	.	.	1
4263	0	0.81	1	0.92093	.	.	1
4264	0	0.81	1	0.92093	.	.	1
4265	0	0.81	1	0.92093	.	.	1
4266	0	0.81	1	0.92093	.	.	1
4267	0	0.82	0	0.92050	0.91796	0.92296	1
4268	0	0.82	1	0.92050	.	.	1
4269	0	0.82	1	0.92050	.	.	1
4270	0	0.82	1	0.92050	.	.	1

Obs	Sub_group	fuptimey	_CENSOR_	SURVIVAL	SDF_LCL	SDF_UCL	STRATUM
4271	0	0.82	1	0.92050	.	.	1
4272	0	0.82	1	0.92050	.	.	1
4273	0	0.82	1	0.92050	.	.	1
4274	0	0.82	1	0.92050	.	.	1
4275	0	0.82	1	0.92050	.	.	1
4276	0	0.82	1	0.92050	.	.	1
4277	0	0.82	1	0.92050	.	.	1
4278	0	0.82	1	0.92050	.	.	1
4279	0	0.82	1	0.92050	.	.	1
4280	0	0.82	1	0.92050	.	.	1
4281	0	0.82	1	0.92050	.	.	1
4282	0	0.82	1	0.92050	.	.	1
4283	0	0.82	1	0.92050	.	.	1
4284	0	0.82	1	0.92050	.	.	1
4285	0	0.82	1	0.92050	.	.	1
4286	0	0.82	1	0.92050	.	.	1
4287	0	0.82	1	0.92050	.	.	1
4288	0	0.82	1	0.92050	.	.	1
4289	0	0.82	1	0.92050	.	.	1
4290	0	0.82	1	0.92050	.	.	1
4291	0	0.82	1	0.92050	.	.	1
4292	0	0.82	1	0.92050	.	.	1
4293	0	0.82	1	0.92050	.	.	1
4294	0	0.82	1	0.92050	.	.	1
4295	0	0.82	1	0.92050	.	.	1
4296	0	0.82	1	0.92050	.	.	1
4297	0	0.83	0	0.92002	0.91748	0.92249	1
4298	0	0.83	1	0.92002	.	.	1
4299	0	0.83	1	0.92002	.	.	1
4300	0	0.83	1	0.92002	.	.	1
4301	0	0.83	1	0.92002	.	.	1
4302	0	0.83	1	0.92002	.	.	1
4303	0	0.83	1	0.92002	.	.	1
4304	0	0.83	1	0.92002	.	.	1
4305	0	0.83	1	0.92002	.	.	1

Obs	Sub_group	fuptimey	_CENSOR_	SURVIVAL	SDF_LCL	SDF_UCL	STRATUM
**4306**	0	0.83	1	0.92002	.	.	1
**4307**	0	0.83	1	0.92002	.	.	1
**4308**	0	0.83	1	0.92002	.	.	1
**4309**	0	0.83	1	0.92002	.	.	1
**4310**	0	0.83	1	0.92002	.	.	1
**4311**	0	0.83	1	0.92002	.	.	1
**4312**	0	0.83	1	0.92002	.	.	1
**4313**	0	0.83	1	0.92002	.	.	1
**4314**	0	0.83	1	0.92002	.	.	1
**4315**	0	0.83	1	0.92002	.	.	1
**4316**	0	0.83	1	0.92002	.	.	1
**4317**	0	0.83	1	0.92002	.	.	1
**4318**	0	0.83	1	0.92002	.	.	1
**4319**	0	0.83	1	0.92002	.	.	1
**4320**	0	0.83	1	0.92002	.	.	1
**4321**	0	0.83	1	0.92002	.	.	1
**4322**	0	0.83	1	0.92002	.	.	1
**4323**	0	0.83	1	0.92002	.	.	1
**4324**	0	0.83	1	0.92002	.	.	1
**4325**	0	0.83	1	0.92002	.	.	1
**4326**	0	0.83	1	0.92002	.	.	1
**4327**	0	0.83	1	0.92002	.	.	1
**4328**	0	0.83	1	0.92002	.	.	1
**4329**	0	0.83	1	0.92002	.	.	1
**4330**	0	0.83	1	0.92002	.	.	1
**4331**	0	0.83	1	0.92002	.	.	1
**4332**	0	0.83	1	0.92002	.	.	1
**4333**	0	0.83	1	0.92002	.	.	1
**4334**	0	0.83	1	0.92002	.	.	1
**4335**	0	0.83	1	0.92002	.	.	1
**4336**	0	0.83	1	0.92002	.	.	1
**4337**	0	0.83	1	0.92002	.	.	1
**4338**	0	0.83	1	0.92002	.	.	1
**4339**	0	0.83	1	0.92002	.	.	1
**4340**	0	0.83	1	0.92002	.	.	1

Obs	Sub_group	fuptimey	_CENSOR_	SURVIVAL	SDF_LCL	SDF_UCL	STRATUM
4341	0	0.83	1	0.92002	.	.	1
4342	0	0.83	1	0.92002	.	.	1
4343	0	0.83	1	0.92002	.	.	1
4344	0	0.84	0	0.91965	0.91710	0.92212	1
4345	0	0.84	1	0.91965	.	.	1
4346	0	0.84	1	0.91965	.	.	1
4347	0	0.84	1	0.91965	.	.	1
4348	0	0.84	1	0.91965	.	.	1
4349	0	0.84	1	0.91965	.	.	1
4350	0	0.84	1	0.91965	.	.	1
4351	0	0.84	1	0.91965	.	.	1
4352	0	0.84	1	0.91965	.	.	1
4353	0	0.84	1	0.91965	.	.	1
4354	0	0.84	1	0.91965	.	.	1
4355	0	0.84	1	0.91965	.	.	1
4356	0	0.84	1	0.91965	.	.	1
4357	0	0.84	1	0.91965	.	.	1
4358	0	0.84	1	0.91965	.	.	1
4359	0	0.84	1	0.91965	.	.	1
4360	0	0.84	1	0.91965	.	.	1
4361	0	0.84	1	0.91965	.	.	1
4362	0	0.84	1	0.91965	.	.	1
4363	0	0.84	1	0.91965	.	.	1
4364	0	0.84	1	0.91965	.	.	1
4365	0	0.84	1	0.91965	.	.	1
4366	0	0.84	1	0.91965	.	.	1
4367	0	0.84	1	0.91965	.	.	1
4368	0	0.84	1	0.91965	.	.	1
4369	0	0.84	1	0.91965	.	.	1
4370	0	0.84	1	0.91965	.	.	1
4371	0	0.84	1	0.91965	.	.	1
4372	0	0.84	1	0.91965	.	.	1
4373	0	0.84	1	0.91965	.	.	1
4374	0	0.84	1	0.91965	.	.	1
4375	0	0.84	1	0.91965	.	.	1

Obs	Sub_group	fuptimey	_CENSOR_	SURVIVAL	SDF_LCL	SDF_UCL	STRATUM
4376	0	0.84	1	0.91965	.	.	1
4377	0	0.84	1	0.91965	.	.	1
4378	0	0.84	1	0.91965	.	.	1
4379	0	0.84	1	0.91965	.	.	1
4380	0	0.84	1	0.91965	.	.	1
4381	0	0.84	1	0.91965	.	.	1
4382	0	0.84	1	0.91965	.	.	1
4383	0	0.84	1	0.91965	.	.	1
4384	0	0.85	0	0.91924	0.91669	0.92172	1
4385	0	0.85	1	0.91924	.	.	1
4386	0	0.85	1	0.91924	.	.	1
4387	0	0.85	1	0.91924	.	.	1
4388	0	0.85	1	0.91924	.	.	1
4389	0	0.85	1	0.91924	.	.	1
4390	0	0.85	1	0.91924	.	.	1
4391	0	0.85	1	0.91924	.	.	1
4392	0	0.85	1	0.91924	.	.	1
4393	0	0.85	1	0.91924	.	.	1
4394	0	0.85	1	0.91924	.	.	1
4395	0	0.85	1	0.91924	.	.	1
4396	0	0.85	1	0.91924	.	.	1
4397	0	0.85	1	0.91924	.	.	1
4398	0	0.85	1	0.91924	.	.	1
4399	0	0.85	1	0.91924	.	.	1
4400	0	0.85	1	0.91924	.	.	1
4401	0	0.85	1	0.91924	.	.	1
4402	0	0.85	1	0.91924	.	.	1
4403	0	0.85	1	0.91924	.	.	1
4404	0	0.85	1	0.91924	.	.	1
4405	0	0.85	1	0.91924	.	.	1
4406	0	0.85	1	0.91924	.	.	1
4407	0	0.85	1	0.91924	.	.	1
4408	0	0.85	1	0.91924	.	.	1
4409	0	0.85	1	0.91924	.	.	1
4410	0	0.85	1	0.91924	.	.	1

Obs	Sub_group	fuptimey	_CENSOR_	SURVIVAL	SDF_LCL	SDF_UCL	STRATUM
4411	0	0.85	1	0.91924	.	.	1
4412	0	0.85	1	0.91924	.	.	1
4413	0	0.85	1	0.91924	.	.	1
4414	0	0.85	1	0.91924	.	.	1
4415	0	0.85	1	0.91924	.	.	1
4416	0	0.85	1	0.91924	.	.	1
4417	0	0.85	1	0.91924	.	.	1
4418	0	0.85	1	0.91924	.	.	1
4419	0	0.85	1	0.91924	.	.	1
4420	0	0.85	1	0.91924	.	.	1
4421	0	0.85	1	0.91924	.	.	1
4422	0	0.85	1	0.91924	.	.	1
4423	0	0.85	1	0.91924	.	.	1
4424	0	0.85	1	0.91924	.	.	1
4425	0	0.85	1	0.91924	.	.	1
4426	0	0.85	1	0.91924	.	.	1
4427	0	0.85	1	0.91924	.	.	1
4428	0	0.85	1	0.91924	.	.	1
4429	0	0.85	1	0.91924	.	.	1
4430	0	0.85	1	0.91924	.	.	1
4431	0	0.85	1	0.91924	.	.	1
4432	0	0.85	1	0.91924	.	.	1
4433	0	0.85	1	0.91924	.	.	1
4434	0	0.85	1	0.91924	.	.	1
4435	0	0.85	1	0.91924	.	.	1
4436	0	0.86	0	0.91886	0.91630	0.92135	1
4437	0	0.86	1	0.91886	.	.	1
4438	0	0.86	1	0.91886	.	.	1
4439	0	0.86	1	0.91886	.	.	1
4440	0	0.86	1	0.91886	.	.	1
4441	0	0.86	1	0.91886	.	.	1
4442	0	0.86	1	0.91886	.	.	1
4443	0	0.86	1	0.91886	.	.	1
4444	0	0.86	1	0.91886	.	.	1
4445	0	0.86	1	0.91886	.	.	1

Obs	Sub_group	fuptimey	_CENSOR_	SURVIVAL	SDF_LCL	SDF_UCL	STRATUM
**4446**	0	0.86	1	0.91886	.	.	1
**4447**	0	0.86	1	0.91886	.	.	1
**4448**	0	0.86	1	0.91886	.	.	1
**4449**	0	0.86	1	0.91886	.	.	1
**4450**	0	0.86	1	0.91886	.	.	1
**4451**	0	0.86	1	0.91886	.	.	1
**4452**	0	0.86	1	0.91886	.	.	1
**4453**	0	0.86	1	0.91886	.	.	1
**4454**	0	0.86	1	0.91886	.	.	1
**4455**	0	0.86	1	0.91886	.	.	1
**4456**	0	0.86	1	0.91886	.	.	1
**4457**	0	0.86	1	0.91886	.	.	1
**4458**	0	0.86	1	0.91886	.	.	1
**4459**	0	0.86	1	0.91886	.	.	1
**4460**	0	0.86	1	0.91886	.	.	1
**4461**	0	0.86	1	0.91886	.	.	1
**4462**	0	0.86	1	0.91886	.	.	1
**4463**	0	0.86	1	0.91886	.	.	1
**4464**	0	0.86	1	0.91886	.	.	1
**4465**	0	0.86	1	0.91886	.	.	1
**4466**	0	0.86	1	0.91886	.	.	1
**4467**	0	0.86	1	0.91886	.	.	1
**4468**	0	0.86	1	0.91886	.	.	1
**4469**	0	0.86	1	0.91886	.	.	1
**4470**	0	0.86	1	0.91886	.	.	1
**4471**	0	0.86	1	0.91886	.	.	1
**4472**	0	0.86	1	0.91886	.	.	1
**4473**	0	0.86	1	0.91886	.	.	1
**4474**	0	0.86	1	0.91886	.	.	1
**4475**	0	0.86	1	0.91886	.	.	1
**4476**	0	0.86	1	0.91886	.	.	1
**4477**	0	0.86	1	0.91886	.	.	1
**4478**	0	0.87	0	0.91836	0.91579	0.92085	1
**4479**	0	0.87	1	0.91836	.	.	1
**4480**	0	0.87	1	0.91836	.	.	1

Obs	Sub_group	fuptimey	_CENSOR_	SURVIVAL	SDF_LCL	SDF_UCL	STRATUM
4481	0	0.87	1	0.91836	.	.	1
4482	0	0.87	1	0.91836	.	.	1
4483	0	0.87	1	0.91836	.	.	1
4484	0	0.87	1	0.91836	.	.	1
4485	0	0.87	1	0.91836	.	.	1
4486	0	0.87	1	0.91836	.	.	1
4487	0	0.87	1	0.91836	.	.	1
4488	0	0.87	1	0.91836	.	.	1
4489	0	0.87	1	0.91836	.	.	1
4490	0	0.87	1	0.91836	.	.	1
4491	0	0.87	1	0.91836	.	.	1
4492	0	0.87	1	0.91836	.	.	1
4493	0	0.87	1	0.91836	.	.	1
4494	0	0.87	1	0.91836	.	.	1
4495	0	0.87	1	0.91836	.	.	1
4496	0	0.87	1	0.91836	.	.	1
4497	0	0.87	1	0.91836	.	.	1
4498	0	0.87	1	0.91836	.	.	1
4499	0	0.87	1	0.91836	.	.	1
4500	0	0.87	1	0.91836	.	.	1
4501	0	0.87	1	0.91836	.	.	1
4502	0	0.87	1	0.91836	.	.	1
4503	0	0.87	1	0.91836	.	.	1
4504	0	0.87	1	0.91836	.	.	1
4505	0	0.87	1	0.91836	.	.	1
4506	0	0.87	1	0.91836	.	.	1
4507	0	0.87	1	0.91836	.	.	1
4508	0	0.87	1	0.91836	.	.	1
4509	0	0.87	1	0.91836	.	.	1
4510	0	0.87	1	0.91836	.	.	1
4511	0	0.87	1	0.91836	.	.	1
4512	0	0.87	1	0.91836	.	.	1
4513	0	0.87	1	0.91836	.	.	1
4514	0	0.87	1	0.91836	.	.	1
4515	0	0.87	1	0.91836	.	.	1

Obs	Sub_group	fuptimey	_CENSOR_	SURVIVAL	SDF_LCL	SDF_UCL	STRATUM
4516	0	0.87	1	0.91836	.	.	1
4517	0	0.87	1	0.91836	.	.	1
4518	0	0.87	1	0.91836	.	.	1
4519	0	0.87	1	0.91836	.	.	1
4520	0	0.87	1	0.91836	.	.	1
4521	0	0.88	0	0.91810	0.91553	0.92060	1
4522	0	0.88	1	0.91810	.	.	1
4523	0	0.88	1	0.91810	.	.	1
4524	0	0.88	1	0.91810	.	.	1
4525	0	0.88	1	0.91810	.	.	1
4526	0	0.88	1	0.91810	.	.	1
4527	0	0.88	1	0.91810	.	.	1
4528	0	0.88	1	0.91810	.	.	1
4529	0	0.88	1	0.91810	.	.	1
4530	0	0.88	1	0.91810	.	.	1
4531	0	0.88	1	0.91810	.	.	1
4532	0	0.88	1	0.91810	.	.	1
4533	0	0.88	1	0.91810	.	.	1
4534	0	0.88	1	0.91810	.	.	1
4535	0	0.88	1	0.91810	.	.	1
4536	0	0.88	1	0.91810	.	.	1
4537	0	0.88	1	0.91810	.	.	1
4538	0	0.88	1	0.91810	.	.	1
4539	0	0.88	1	0.91810	.	.	1
4540	0	0.88	1	0.91810	.	.	1
4541	0	0.88	1	0.91810	.	.	1
4542	0	0.88	1	0.91810	.	.	1
4543	0	0.88	1	0.91810	.	.	1
4544	0	0.88	1	0.91810	.	.	1
4545	0	0.88	1	0.91810	.	.	1
4546	0	0.88	1	0.91810	.	.	1
4547	0	0.88	1	0.91810	.	.	1
4548	0	0.88	1	0.91810	.	.	1
4549	0	0.88	1	0.91810	.	.	1
4550	0	0.88	1	0.91810	.	.	1

Obs	Sub_group	fuptimey	_CENSOR_	SURVIVAL	SDF_LCL	SDF_UCL	STRATUM
4551	0	0.88	1	0.91810	.	.	1
4552	0	0.88	1	0.91810	.	.	1
4553	0	0.89	0	0.91762	0.91504	0.92013	1
4554	0	0.89	1	0.91762	.	.	1
4555	0	0.89	1	0.91762	.	.	1
4556	0	0.89	1	0.91762	.	.	1
4557	0	0.89	1	0.91762	.	.	1
4558	0	0.89	1	0.91762	.	.	1
4559	0	0.89	1	0.91762	.	.	1
4560	0	0.89	1	0.91762	.	.	1
4561	0	0.89	1	0.91762	.	.	1
4562	0	0.89	1	0.91762	.	.	1
4563	0	0.89	1	0.91762	.	.	1
4564	0	0.89	1	0.91762	.	.	1
4565	0	0.89	1	0.91762	.	.	1
4566	0	0.89	1	0.91762	.	.	1
4567	0	0.89	1	0.91762	.	.	1
4568	0	0.89	1	0.91762	.	.	1
4569	0	0.89	1	0.91762	.	.	1
4570	0	0.89	1	0.91762	.	.	1
4571	0	0.89	1	0.91762	.	.	1
4572	0	0.89	1	0.91762	.	.	1
4573	0	0.89	1	0.91762	.	.	1
4574	0	0.89	1	0.91762	.	.	1
4575	0	0.89	1	0.91762	.	.	1
4576	0	0.89	1	0.91762	.	.	1
4577	0	0.89	1	0.91762	.	.	1
4578	0	0.89	1	0.91762	.	.	1
4579	0	0.89	1	0.91762	.	.	1
4580	0	0.89	1	0.91762	.	.	1
4581	0	0.89	1	0.91762	.	.	1
4582	0	0.89	1	0.91762	.	.	1
4583	0	0.89	1	0.91762	.	.	1
4584	0	0.89	1	0.91762	.	.	1
4585	0	0.89	1	0.91762	.	.	1

Obs	Sub_group	fuptimey	_CENSOR_	SURVIVAL	SDF_LCL	SDF_UCL	STRATUM
4586	0	0.89	1	0.91762	.	.	1
4587	0	0.89	1	0.91762	.	.	1
4588	0	0.89	1	0.91762	.	.	1
4589	0	0.89	1	0.91762	.	.	1
4590	0	0.89	1	0.91762	.	.	1
4591	0	0.89	1	0.91762	.	.	1
4592	0	0.89	1	0.91762	.	.	1
4593	0	0.89	1	0.91762	.	.	1
4594	0	0.89	1	0.91762	.	.	1
4595	0	0.89	1	0.91762	.	.	1
4596	0	0.89	1	0.91762	.	.	1
4597	0	0.89	1	0.91762	.	.	1
4598	0	0.89	1	0.91762	.	.	1
4599	0	0.89	1	0.91762	.	.	1
4600	0	0.89	1	0.91762	.	.	1
4601	0	0.89	1	0.91762	.	.	1
4602	0	0.89	1	0.91762	.	.	1
4603	0	0.89	1	0.91762	.	.	1
4604	0	0.89	1	0.91762	.	.	1
4605	0	0.89	1	0.91762	.	.	1
4606	0	0.89	1	0.91762	.	.	1
4607	0	0.89	1	0.91762	.	.	1
4608	0	0.89	1	0.91762	.	.	1
4609	0	0.89	1	0.91762	.	.	1
4610	0	0.89	1	0.91762	.	.	1
4611	0	0.89	1	0.91762	.	.	1
4612	0	0.89	1	0.91762	.	.	1
4613	0	0.89	1	0.91762	.	.	1
4614	0	0.89	1	0.91762	.	.	1
4615	0	0.89	1	0.91762	.	.	1
4616	0	0.90	0	0.91734	0.91475	0.91984	1
4617	0	0.90	1	0.91734	.	.	1
4618	0	0.90	1	0.91734	.	.	1
4619	0	0.90	1	0.91734	.	.	1
4620	0	0.90	1	0.91734	.	.	1

Obs	Sub_group	fuptimey	_CENSOR_	SURVIVAL	SDF_LCL	SDF_UCL	STRATUM
4621	0	0.90	1	0.91734	.	.	1
4622	0	0.90	1	0.91734	.	.	1
4623	0	0.90	1	0.91734	.	.	1
4624	0	0.90	1	0.91734	.	.	1
4625	0	0.90	1	0.91734	.	.	1
4626	0	0.90	1	0.91734	.	.	1
4627	0	0.90	1	0.91734	.	.	1
4628	0	0.90	1	0.91734	.	.	1
4629	0	0.90	1	0.91734	.	.	1
4630	0	0.90	1	0.91734	.	.	1
4631	0	0.90	1	0.91734	.	.	1
4632	0	0.90	1	0.91734	.	.	1
4633	0	0.90	1	0.91734	.	.	1
4634	0	0.90	1	0.91734	.	.	1
4635	0	0.90	1	0.91734	.	.	1
4636	0	0.90	1	0.91734	.	.	1
4637	0	0.90	1	0.91734	.	.	1
4638	0	0.90	1	0.91734	.	.	1
4639	0	0.90	1	0.91734	.	.	1
4640	0	0.91	0	0.91683	0.91424	0.91935	1
4641	0	0.91	1	0.91683	.	.	1
4642	0	0.91	1	0.91683	.	.	1
4643	0	0.91	1	0.91683	.	.	1
4644	0	0.91	1	0.91683	.	.	1
4645	0	0.91	1	0.91683	.	.	1
4646	0	0.91	1	0.91683	.	.	1
4647	0	0.91	1	0.91683	.	.	1
4648	0	0.91	1	0.91683	.	.	1
4649	0	0.91	1	0.91683	.	.	1
4650	0	0.91	1	0.91683	.	.	1
4651	0	0.91	1	0.91683	.	.	1
4652	0	0.91	1	0.91683	.	.	1
4653	0	0.91	1	0.91683	.	.	1
4654	0	0.91	1	0.91683	.	.	1
4655	0	0.91	1	0.91683	.	.	1

Obs	Sub_group	fuptimey	_CENSOR_	SURVIVAL	SDF_LCL	SDF_UCL	STRATUM
4656	0	0.91	1	0.91683	.	.	1
4657	0	0.91	1	0.91683	.	.	1
4658	0	0.91	1	0.91683	.	.	1
4659	0	0.91	1	0.91683	.	.	1
4660	0	0.91	1	0.91683	.	.	1
4661	0	0.91	1	0.91683	.	.	1
4662	0	0.91	1	0.91683	.	.	1
4663	0	0.91	1	0.91683	.	.	1
4664	0	0.91	1	0.91683	.	.	1
4665	0	0.91	1	0.91683	.	.	1
4666	0	0.91	1	0.91683	.	.	1
4667	0	0.91	1	0.91683	.	.	1
4668	0	0.91	1	0.91683	.	.	1
4669	0	0.91	1	0.91683	.	.	1
4670	0	0.91	1	0.91683	.	.	1
4671	0	0.91	1	0.91683	.	.	1
4672	0	0.91	1	0.91683	.	.	1
4673	0	0.91	1	0.91683	.	.	1
4674	0	0.91	1	0.91683	.	.	1
4675	0	0.91	1	0.91683	.	.	1
4676	0	0.91	1	0.91683	.	.	1
4677	0	0.91	1	0.91683	.	.	1
4678	0	0.91	1	0.91683	.	.	1
4679	0	0.91	1	0.91683	.	.	1
4680	0	0.91	1	0.91683	.	.	1
4681	0	0.91	1	0.91683	.	.	1
4682	0	0.91	1	0.91683	.	.	1
4683	0	0.91	1	0.91683	.	.	1
4684	0	0.91	1	0.91683	.	.	1
4685	0	0.91	1	0.91683	.	.	1
4686	0	0.91	1	0.91683	.	.	1
4687	0	0.91	1	0.91683	.	.	1
4688	0	0.91	1	0.91683	.	.	1
4689	0	0.91	1	0.91683	.	.	1
4690	0	0.91	1	0.91683	.	.	1

Obs	Sub_group	fuptimey	_CENSOR_	SURVIVAL	SDF_LCL	SDF_UCL	STRATUM
4691	0	0.91	1	0.91683	.	.	1
4692	0	0.91	1	0.91683	.	.	1
4693	0	0.91	1	0.91683	.	.	1
4694	0	0.91	1	0.91683	.	.	1
4695	0	0.91	1	0.91683	.	.	1
4696	0	0.91	1	0.91683	.	.	1
4697	0	0.91	1	0.91683	.	.	1
4698	0	0.91	1	0.91683	.	.	1
4699	0	0.91	1	0.91683	.	.	1
4700	0	0.91	1	0.91683	.	.	1
4701	0	0.91	1	0.91683	.	.	1
4702	0	0.91	1	0.91683	.	.	1
4703	0	0.91	1	0.91683	.	.	1
4704	0	0.91	1	0.91683	.	.	1
4705	0	0.91	1	0.91683	.	.	1
4706	0	0.91	1	0.91683	.	.	1
4707	0	0.91	1	0.91683	.	.	1
4708	0	0.91	1	0.91683	.	.	1
4709	0	0.91	1	0.91683	.	.	1
4710	0	0.91	1	0.91683	.	.	1
4711	0	0.91	1	0.91683	.	.	1
4712	0	0.91	1	0.91683	.	.	1
4713	0	0.91	1	0.91683	.	.	1
4714	0	0.92	0	0.91640	0.91381	0.91892	1
4715	0	0.92	1	0.91640	.	.	1
4716	0	0.92	1	0.91640	.	.	1
4717	0	0.92	1	0.91640	.	.	1
4718	0	0.92	1	0.91640	.	.	1
4719	0	0.92	1	0.91640	.	.	1
4720	0	0.92	1	0.91640	.	.	1
4721	0	0.92	1	0.91640	.	.	1
4722	0	0.92	1	0.91640	.	.	1
4723	0	0.92	1	0.91640	.	.	1
4724	0	0.92	1	0.91640	.	.	1
4725	0	0.92	1	0.91640	.	.	1

Obs	Sub_group	fuptimey	_CENSOR_	SURVIVAL	SDF_LCL	SDF_UCL	STRATUM
**4726**	0	0.92	1	0.91640	.	.	1
**4727**	0	0.92	1	0.91640	.	.	1
**4728**	0	0.92	1	0.91640	.	.	1
**4729**	0	0.92	1	0.91640	.	.	1
**4730**	0	0.92	1	0.91640	.	.	1
**4731**	0	0.92	1	0.91640	.	.	1
**4732**	0	0.92	1	0.91640	.	.	1
**4733**	0	0.92	1	0.91640	.	.	1
**4734**	0	0.92	1	0.91640	.	.	1
**4735**	0	0.92	1	0.91640	.	.	1
**4736**	0	0.92	1	0.91640	.	.	1
**4737**	0	0.92	1	0.91640	.	.	1
**4738**	0	0.92	1	0.91640	.	.	1
**4739**	0	0.92	1	0.91640	.	.	1
**4740**	0	0.92	1	0.91640	.	.	1
**4741**	0	0.92	1	0.91640	.	.	1
**4742**	0	0.92	1	0.91640	.	.	1
**4743**	0	0.92	1	0.91640	.	.	1
**4744**	0	0.92	1	0.91640	.	.	1
**4745**	0	0.92	1	0.91640	.	.	1
**4746**	0	0.92	1	0.91640	.	.	1
**4747**	0	0.92	1	0.91640	.	.	1
**4748**	0	0.92	1	0.91640	.	.	1
**4749**	0	0.92	1	0.91640	.	.	1
**4750**	0	0.92	1	0.91640	.	.	1
**4751**	0	0.92	1	0.91640	.	.	1
**4752**	0	0.92	1	0.91640	.	.	1
**4753**	0	0.92	1	0.91640	.	.	1
**4754**	0	0.92	1	0.91640	.	.	1
**4755**	0	0.92	1	0.91640	.	.	1
**4756**	0	0.92	1	0.91640	.	.	1
**4757**	0	0.92	1	0.91640	.	.	1
**4758**	0	0.92	1	0.91640	.	.	1
**4759**	0	0.92	1	0.91640	.	.	1
**4760**	0	0.92	1	0.91640	.	.	1

Obs	Sub_group	fuptimey	_CENSOR_	SURVIVAL	SDF_LCL	SDF_UCL	STRATUM
4761	0	0.92	1	0.91640	.	.	1
4762	0	0.92	1	0.91640	.	.	1
4763	0	0.92	1	0.91640	.	.	1
4764	0	0.92	1	0.91640	.	.	1
4765	0	0.92	1	0.91640	.	.	1
4766	0	0.92	1	0.91640	.	.	1
4767	0	0.92	1	0.91640	.	.	1
4768	0	0.92	1	0.91640	.	.	1
4769	0	0.92	1	0.91640	.	.	1
4770	0	0.92	1	0.91640	.	.	1
4771	0	0.92	1	0.91640	.	.	1
4772	0	0.92	1	0.91640	.	.	1
4773	0	0.92	1	0.91640	.	.	1
4774	0	0.93	0	0.91609	0.91349	0.91862	1
4775	0	0.93	1	0.91609	.	.	1
4776	0	0.93	1	0.91609	.	.	1
4777	0	0.93	1	0.91609	.	.	1
4778	0	0.93	1	0.91609	.	.	1
4779	0	0.93	1	0.91609	.	.	1
4780	0	0.93	1	0.91609	.	.	1
4781	0	0.93	1	0.91609	.	.	1
4782	0	0.93	1	0.91609	.	.	1
4783	0	0.93	1	0.91609	.	.	1
4784	0	0.93	1	0.91609	.	.	1
4785	0	0.93	1	0.91609	.	.	1
4786	0	0.93	1	0.91609	.	.	1
4787	0	0.93	1	0.91609	.	.	1
4788	0	0.93	1	0.91609	.	.	1
4789	0	0.93	1	0.91609	.	.	1
4790	0	0.93	1	0.91609	.	.	1
4791	0	0.93	1	0.91609	.	.	1
4792	0	0.93	1	0.91609	.	.	1
4793	0	0.93	1	0.91609	.	.	1
4794	0	0.93	1	0.91609	.	.	1
4795	0	0.93	1	0.91609	.	.	1

Obs	Sub_group	fuptimey	_CENSOR_	SURVIVAL	SDF_LCL	SDF_UCL	STRATUM
4796	0	0.93	1	0.91609	.	.	1
4797	0	0.93	1	0.91609	.	.	1
4798	0	0.93	1	0.91609	.	.	1
4799	0	0.93	1	0.91609	.	.	1
4800	0	0.93	1	0.91609	.	.	1
4801	0	0.93	1	0.91609	.	.	1
4802	0	0.93	1	0.91609	.	.	1
4803	0	0.93	1	0.91609	.	.	1
4804	0	0.93	1	0.91609	.	.	1
4805	0	0.93	1	0.91609	.	.	1
4806	0	0.93	1	0.91609	.	.	1
4807	0	0.93	1	0.91609	.	.	1
4808	0	0.93	1	0.91609	.	.	1
4809	0	0.93	1	0.91609	.	.	1
4810	0	0.93	1	0.91609	.	.	1
4811	0	0.93	1	0.91609	.	.	1
4812	0	0.93	1	0.91609	.	.	1
4813	0	0.93	1	0.91609	.	.	1
4814	0	0.93	1	0.91609	.	.	1
4815	0	0.93	1	0.91609	.	.	1
4816	0	0.93	1	0.91609	.	.	1
4817	0	0.93	1	0.91609	.	.	1
4818	0	0.93	1	0.91609	.	.	1
4819	0	0.93	1	0.91609	.	.	1
4820	0	0.93	1	0.91609	.	.	1
4821	0	0.93	1	0.91609	.	.	1
4822	0	0.93	1	0.91609	.	.	1
4823	0	0.93	1	0.91609	.	.	1
4824	0	0.93	1	0.91609	.	.	1
4825	0	0.93	1	0.91609	.	.	1
4826	0	0.93	1	0.91609	.	.	1
4827	0	0.93	1	0.91609	.	.	1
4828	0	0.93	1	0.91609	.	.	1
4829	0	0.93	1	0.91609	.	.	1
4830	0	0.93	1	0.91609	.	.	1

Obs	Sub_group	fuptimey	_CENSOR_	SURVIVAL	SDF_LCL	SDF_UCL	STRATUM
4831	0	0.93	1	0.91609	.	.	1
4832	0	0.93	1	0.91609	.	.	1
4833	0	0.94	0	0.91568	0.91307	0.91822	1
4834	0	0.94	1	0.91568	.	.	1
4835	0	0.94	1	0.91568	.	.	1
4836	0	0.94	1	0.91568	.	.	1
4837	0	0.94	1	0.91568	.	.	1
4838	0	0.94	1	0.91568	.	.	1
4839	0	0.94	1	0.91568	.	.	1
4840	0	0.94	1	0.91568	.	.	1
4841	0	0.94	1	0.91568	.	.	1
4842	0	0.94	1	0.91568	.	.	1
4843	0	0.94	1	0.91568	.	.	1
4844	0	0.94	1	0.91568	.	.	1
4845	0	0.94	1	0.91568	.	.	1
4846	0	0.94	1	0.91568	.	.	1
4847	0	0.94	1	0.91568	.	.	1
4848	0	0.94	1	0.91568	.	.	1
4849	0	0.94	1	0.91568	.	.	1
4850	0	0.94	1	0.91568	.	.	1
4851	0	0.94	1	0.91568	.	.	1
4852	0	0.94	1	0.91568	.	.	1
4853	0	0.94	1	0.91568	.	.	1
4854	0	0.94	1	0.91568	.	.	1
4855	0	0.94	1	0.91568	.	.	1
4856	0	0.94	1	0.91568	.	.	1
4857	0	0.94	1	0.91568	.	.	1
4858	0	0.94	1	0.91568	.	.	1
4859	0	0.94	1	0.91568	.	.	1
4860	0	0.94	1	0.91568	.	.	1
4861	0	0.94	1	0.91568	.	.	1
4862	0	0.94	1	0.91568	.	.	1
4863	0	0.94	1	0.91568	.	.	1
4864	0	0.94	1	0.91568	.	.	1
4865	0	0.94	1	0.91568	.	.	1

Obs	Sub_group	fuptimey	_CENSOR_	SURVIVAL	SDF_LCL	SDF_UCL	STRATUM
**4866**	0	0.94	1	0.91568	.	.	1
**4867**	0	0.94	1	0.91568	.	.	1
**4868**	0	0.94	1	0.91568	.	.	1
**4869**	0	0.94	1	0.91568	.	.	1
**4870**	0	0.94	1	0.91568	.	.	1
**4871**	0	0.94	1	0.91568	.	.	1
**4872**	0	0.94	1	0.91568	.	.	1
**4873**	0	0.94	1	0.91568	.	.	1
**4874**	0	0.94	1	0.91568	.	.	1
**4875**	0	0.94	1	0.91568	.	.	1
**4876**	0	0.94	1	0.91568	.	.	1
**4877**	0	0.94	1	0.91568	.	.	1
**4878**	0	0.94	1	0.91568	.	.	1
**4879**	0	0.94	1	0.91568	.	.	1
**4880**	0	0.94	1	0.91568	.	.	1
**4881**	0	0.94	1	0.91568	.	.	1
**4882**	0	0.94	1	0.91568	.	.	1
**4883**	0	0.94	1	0.91568	.	.	1
**4884**	0	0.94	1	0.91568	.	.	1
**4885**	0	0.94	1	0.91568	.	.	1
**4886**	0	0.94	1	0.91568	.	.	1
**4887**	0	0.94	1	0.91568	.	.	1
**4888**	0	0.94	1	0.91568	.	.	1
**4889**	0	0.95	0	0.91515	0.91254	0.91769	1
**4890**	0	0.95	1	0.91515	.	.	1
**4891**	0	0.95	1	0.91515	.	.	1
**4892**	0	0.95	1	0.91515	.	.	1
**4893**	0	0.95	1	0.91515	.	.	1
**4894**	0	0.95	1	0.91515	.	.	1
**4895**	0	0.95	1	0.91515	.	.	1
**4896**	0	0.95	1	0.91515	.	.	1
**4897**	0	0.95	1	0.91515	.	.	1
**4898**	0	0.95	1	0.91515	.	.	1
**4899**	0	0.95	1	0.91515	.	.	1
**4900**	0	0.95	1	0.91515	.	.	1

Obs	Sub_group	fuptimey	_CENSOR_	SURVIVAL	SDF_LCL	SDF_UCL	STRATUM
4901	0	0.95	1	0.91515	.	.	1
4902	0	0.95	1	0.91515	.	.	1
4903	0	0.95	1	0.91515	.	.	1
4904	0	0.95	1	0.91515	.	.	1
4905	0	0.95	1	0.91515	.	.	1
4906	0	0.95	1	0.91515	.	.	1
4907	0	0.95	1	0.91515	.	.	1
4908	0	0.95	1	0.91515	.	.	1
4909	0	0.95	1	0.91515	.	.	1
4910	0	0.95	1	0.91515	.	.	1
4911	0	0.95	1	0.91515	.	.	1
4912	0	0.95	1	0.91515	.	.	1
4913	0	0.95	1	0.91515	.	.	1
4914	0	0.95	1	0.91515	.	.	1
4915	0	0.95	1	0.91515	.	.	1
4916	0	0.95	1	0.91515	.	.	1
4917	0	0.95	1	0.91515	.	.	1
4918	0	0.95	1	0.91515	.	.	1
4919	0	0.95	1	0.91515	.	.	1
4920	0	0.95	1	0.91515	.	.	1
4921	0	0.95	1	0.91515	.	.	1
4922	0	0.95	1	0.91515	.	.	1
4923	0	0.95	1	0.91515	.	.	1
4924	0	0.95	1	0.91515	.	.	1
4925	0	0.95	1	0.91515	.	.	1
4926	0	0.95	1	0.91515	.	.	1
4927	0	0.95	1	0.91515	.	.	1
4928	0	0.95	1	0.91515	.	.	1
4929	0	0.95	1	0.91515	.	.	1
4930	0	0.95	1	0.91515	.	.	1
4931	0	0.95	1	0.91515	.	.	1
4932	0	0.95	1	0.91515	.	.	1
4933	0	0.95	1	0.91515	.	.	1
4934	0	0.95	1	0.91515	.	.	1
4935	0	0.95	1	0.91515	.	.	1

Obs	Sub_group	fuptimey	_CENSOR_	SURVIVAL	SDF_LCL	SDF_UCL	STRATUM
4936	0	0.95	1	0.91515	.	.	1
4937	0	0.95	1	0.91515	.	.	1
4938	0	0.95	1	0.91515	.	.	1
4939	0	0.95	1	0.91515	.	.	1
4940	0	0.95	1	0.91515	.	.	1
4941	0	0.95	1	0.91515	.	.	1
4942	0	0.95	1	0.91515	.	.	1
4943	0	0.95	1	0.91515	.	.	1
4944	0	0.95	1	0.91515	.	.	1
4945	0	0.95	1	0.91515	.	.	1
4946	0	0.95	1	0.91515	.	.	1
4947	0	0.95	1	0.91515	.	.	1
4948	0	0.95	1	0.91515	.	.	1
4949	0	0.96	0	0.91474	0.91212	0.91729	1
4950	0	0.96	1	0.91474	.	.	1
4951	0	0.96	1	0.91474	.	.	1
4952	0	0.96	1	0.91474	.	.	1
4953	0	0.96	1	0.91474	.	.	1
4954	0	0.96	1	0.91474	.	.	1
4955	0	0.96	1	0.91474	.	.	1
4956	0	0.96	1	0.91474	.	.	1
4957	0	0.96	1	0.91474	.	.	1
4958	0	0.96	1	0.91474	.	.	1
4959	0	0.96	1	0.91474	.	.	1
4960	0	0.96	1	0.91474	.	.	1
4961	0	0.96	1	0.91474	.	.	1
4962	0	0.96	1	0.91474	.	.	1
4963	0	0.96	1	0.91474	.	.	1
4964	0	0.96	1	0.91474	.	.	1
4965	0	0.96	1	0.91474	.	.	1
4966	0	0.96	1	0.91474	.	.	1
4967	0	0.96	1	0.91474	.	.	1
4968	0	0.96	1	0.91474	.	.	1
4969	0	0.96	1	0.91474	.	.	1
4970	0	0.96	1	0.91474	.	.	1

Obs	Sub_group	fuptimey	_CENSOR_	SURVIVAL	SDF_LCL	SDF_UCL	STRATUM
4971	0	0.96	1	0.91474	.	.	1
4972	0	0.96	1	0.91474	.	.	1
4973	0	0.96	1	0.91474	.	.	1
4974	0	0.96	1	0.91474	.	.	1
4975	0	0.96	1	0.91474	.	.	1
4976	0	0.96	1	0.91474	.	.	1
4977	0	0.96	1	0.91474	.	.	1
4978	0	0.96	1	0.91474	.	.	1
4979	0	0.96	1	0.91474	.	.	1
4980	0	0.96	1	0.91474	.	.	1
4981	0	0.96	1	0.91474	.	.	1
4982	0	0.96	1	0.91474	.	.	1
4983	0	0.96	1	0.91474	.	.	1
4984	0	0.96	1	0.91474	.	.	1
4985	0	0.96	1	0.91474	.	.	1
4986	0	0.96	1	0.91474	.	.	1
4987	0	0.96	1	0.91474	.	.	1
4988	0	0.96	1	0.91474	.	.	1
4989	0	0.96	1	0.91474	.	.	1
4990	0	0.96	1	0.91474	.	.	1
4991	0	0.97	0	0.91431	0.91168	0.91686	1
4992	0	0.97	1	0.91431	.	.	1
4993	0	0.97	1	0.91431	.	.	1
4994	0	0.97	1	0.91431	.	.	1
4995	0	0.97	1	0.91431	.	.	1
4996	0	0.97	1	0.91431	.	.	1
4997	0	0.97	1	0.91431	.	.	1
4998	0	0.97	1	0.91431	.	.	1
4999	0	0.97	1	0.91431	.	.	1
5000	0	0.97	1	0.91431	.	.	1
5001	0	0.97	1	0.91431	.	.	1
5002	0	0.97	1	0.91431	.	.	1
5003	0	0.97	1	0.91431	.	.	1
5004	0	0.97	1	0.91431	.	.	1
5005	0	0.97	1	0.91431	.	.	1

Obs	Sub_group	fuptimey	_CENSOR_	SURVIVAL	SDF_LCL	SDF_UCL	STRATUM
5006	0	0.97	1	0.91431	.	.	1
5007	0	0.97	1	0.91431	.	.	1
5008	0	0.97	1	0.91431	.	.	1
5009	0	0.97	1	0.91431	.	.	1
5010	0	0.97	1	0.91431	.	.	1
5011	0	0.97	1	0.91431	.	.	1
5012	0	0.97	1	0.91431	.	.	1
5013	0	0.97	1	0.91431	.	.	1
5014	0	0.97	1	0.91431	.	.	1
5015	0	0.97	1	0.91431	.	.	1
5016	0	0.97	1	0.91431	.	.	1
5017	0	0.97	1	0.91431	.	.	1
5018	0	0.97	1	0.91431	.	.	1
5019	0	0.97	1	0.91431	.	.	1
5020	0	0.97	1	0.91431	.	.	1
5021	0	0.97	1	0.91431	.	.	1
5022	0	0.97	1	0.91431	.	.	1
5023	0	0.97	1	0.91431	.	.	1
5024	0	0.97	1	0.91431	.	.	1
5025	0	0.97	1	0.91431	.	.	1
5026	0	0.97	1	0.91431	.	.	1
5027	0	0.97	1	0.91431	.	.	1
5028	0	0.97	1	0.91431	.	.	1
5029	0	0.97	1	0.91431	.	.	1
5030	0	0.97	1	0.91431	.	.	1
5031	0	0.97	1	0.91431	.	.	1
5032	0	0.97	1	0.91431	.	.	1
5033	0	0.97	1	0.91431	.	.	1
5034	0	0.97	1	0.91431	.	.	1
5035	0	0.97	1	0.91431	.	.	1
5036	0	0.97	1	0.91431	.	.	1
5037	0	0.97	1	0.91431	.	.	1
5038	0	0.97	1	0.91431	.	.	1
5039	0	0.97	1	0.91431	.	.	1
5040	0	0.97	1	0.91431	.	.	1

Obs	Sub_group	fuptimey	_CENSOR_	SURVIVAL	SDF_LCL	SDF_UCL	STRATUM
5041	0	0.97	1	0.91431	.	.	1
5042	0	0.97	1	0.91431	.	.	1
5043	0	0.97	1	0.91431	.	.	1
5044	0	0.97	1	0.91431	.	.	1
5045	0	0.97	1	0.91431	.	.	1
5046	0	0.97	1	0.91431	.	.	1
5047	0	0.97	1	0.91431	.	.	1
5048	0	0.97	1	0.91431	.	.	1
5049	0	0.97	1	0.91431	.	.	1
5050	0	0.97	1	0.91431	.	.	1
5051	0	0.97	1	0.91431	.	.	1
5052	0	0.97	1	0.91431	.	.	1
5053	0	0.97	1	0.91431	.	.	1
5054	0	0.97	1	0.91431	.	.	1
5055	0	0.97	1	0.91431	.	.	1
5056	0	0.97	1	0.91431	.	.	1
5057	0	0.97	1	0.91431	.	.	1
5058	0	0.97	1	0.91431	.	.	1
5059	0	0.97	1	0.91431	.	.	1
5060	0	0.97	1	0.91431	.	.	1
5061	0	0.98	0	0.91380	0.91117	0.91636	1
5062	0	0.98	1	0.91380	.	.	1
5063	0	0.98	1	0.91380	.	.	1
5064	0	0.98	1	0.91380	.	.	1
5065	0	0.98	1	0.91380	.	.	1
5066	0	0.98	1	0.91380	.	.	1
5067	0	0.98	1	0.91380	.	.	1
5068	0	0.98	1	0.91380	.	.	1
5069	0	0.98	1	0.91380	.	.	1
5070	0	0.98	1	0.91380	.	.	1
5071	0	0.98	1	0.91380	.	.	1
5072	0	0.98	1	0.91380	.	.	1
5073	0	0.98	1	0.91380	.	.	1
5074	0	0.98	1	0.91380	.	.	1
5075	0	0.98	1	0.91380	.	.	1

Obs	Sub_group	fuptimey	_CENSOR_	SURVIVAL	SDF_LCL	SDF_UCL	STRATUM
5076	0	0.98	1	0.91380	.	.	1
5077	0	0.98	1	0.91380	.	.	1
5078	0	0.98	1	0.91380	.	.	1
5079	0	0.98	1	0.91380	.	.	1
5080	0	0.98	1	0.91380	.	.	1
5081	0	0.98	1	0.91380	.	.	1
5082	0	0.98	1	0.91380	.	.	1
5083	0	0.98	1	0.91380	.	.	1
5084	0	0.98	1	0.91380	.	.	1
5085	0	0.98	1	0.91380	.	.	1
5086	0	0.98	1	0.91380	.	.	1
5087	0	0.98	1	0.91380	.	.	1
5088	0	0.98	1	0.91380	.	.	1
5089	0	0.98	1	0.91380	.	.	1
5090	0	0.98	1	0.91380	.	.	1
5091	0	0.98	1	0.91380	.	.	1
5092	0	0.98	1	0.91380	.	.	1
5093	0	0.98	1	0.91380	.	.	1
5094	0	0.98	1	0.91380	.	.	1
5095	0	0.98	1	0.91380	.	.	1
5096	0	0.98	1	0.91380	.	.	1
5097	0	0.98	1	0.91380	.	.	1
5098	0	0.98	1	0.91380	.	.	1
5099	0	0.98	1	0.91380	.	.	1
5100	0	0.98	1	0.91380	.	.	1
5101	0	0.98	1	0.91380	.	.	1
5102	0	0.98	1	0.91380	.	.	1
5103	0	0.98	1	0.91380	.	.	1
5104	0	0.98	1	0.91380	.	.	1
5105	0	0.98	1	0.91380	.	.	1
5106	0	0.98	1	0.91380	.	.	1
5107	0	0.98	1	0.91380	.	.	1
5108	0	0.98	1	0.91380	.	.	1
5109	0	0.98	1	0.91380	.	.	1
5110	0	0.98	1	0.91380	.	.	1

Obs	Sub_group	fuptimey	_CENSOR_	SURVIVAL	SDF_LCL	SDF_UCL	STRATUM
5111	0	0.98	1	0.91380	.	.	1
5112	0	0.98	1	0.91380	.	.	1
5113	0	0.98	1	0.91380	.	.	1
5114	0	0.98	1	0.91380	.	.	1
5115	0	0.98	1	0.91380	.	.	1
5116	0	0.98	1	0.91380	.	.	1
5117	0	0.98	1	0.91380	.	.	1
5118	0	0.98	1	0.91380	.	.	1
5119	0	0.98	1	0.91380	.	.	1
5120	0	0.98	1	0.91380	.	.	1
5121	0	0.98	1	0.91380	.	.	1
5122	0	0.98	1	0.91380	.	.	1
5123	0	0.98	1	0.91380	.	.	1
5124	0	0.98	1	0.91380	.	.	1
5125	0	0.98	1	0.91380	.	.	1
5126	0	0.98	1	0.91380	.	.	1
5127	0	0.98	1	0.91380	.	.	1
5128	0	0.98	1	0.91380	.	.	1
5129	0	0.98	1	0.91380	.	.	1
5130	0	0.98	1	0.91380	.	.	1
5131	0	0.98	1	0.91380	.	.	1
5132	0	0.99	0	0.91334	0.91070	0.91591	1
5133	0	0.99	1	0.91334	.	.	1
5134	0	0.99	1	0.91334	.	.	1
5135	0	0.99	1	0.91334	.	.	1
5136	0	0.99	1	0.91334	.	.	1
5137	0	0.99	1	0.91334	.	.	1
5138	0	0.99	1	0.91334	.	.	1
5139	0	0.99	1	0.91334	.	.	1
5140	0	0.99	1	0.91334	.	.	1
5141	0	0.99	1	0.91334	.	.	1
5142	0	0.99	1	0.91334	.	.	1
5143	0	0.99	1	0.91334	.	.	1
5144	0	0.99	1	0.91334	.	.	1
5145	0	0.99	1	0.91334	.	.	1

Obs	Sub_group	fuptimey	_CENSOR_	SURVIVAL	SDF_LCL	SDF_UCL	STRATUM
5146	0	0.99	1	0.91334	.	.	1
5147	0	0.99	1	0.91334	.	.	1
5148	0	0.99	1	0.91334	.	.	1
5149	0	0.99	1	0.91334	.	.	1
5150	0	0.99	1	0.91334	.	.	1
5151	0	0.99	1	0.91334	.	.	1
5152	0	0.99	1	0.91334	.	.	1
5153	0	0.99	1	0.91334	.	.	1
5154	0	0.99	1	0.91334	.	.	1
5155	0	0.99	1	0.91334	.	.	1
5156	0	0.99	1	0.91334	.	.	1
5157	0	0.99	1	0.91334	.	.	1
5158	0	0.99	1	0.91334	.	.	1
5159	0	0.99	1	0.91334	.	.	1
5160	0	0.99	1	0.91334	.	.	1
5161	0	0.99	1	0.91334	.	.	1
5162	0	0.99	1	0.91334	.	.	1
5163	0	0.99	1	0.91334	.	.	1
5164	0	0.99	1	0.91334	.	.	1
5165	0	0.99	1	0.91334	.	.	1
5166	0	0.99	1	0.91334	.	.	1
5167	0	0.99	1	0.91334	.	.	1
5168	0	0.99	1	0.91334	.	.	1
5169	0	0.99	1	0.91334	.	.	1
5170	0	0.99	1	0.91334	.	.	1
5171	0	0.99	1	0.91334	.	.	1
5172	0	0.99	1	0.91334	.	.	1
5173	0	0.99	1	0.91334	.	.	1
5174	0	0.99	1	0.91334	.	.	1
5175	0	0.99	1	0.91334	.	.	1
5176	0	0.99	1	0.91334	.	.	1
5177	0	0.99	1	0.91334	.	.	1
5178	0	0.99	1	0.91334	.	.	1
5179	0	0.99	1	0.91334	.	.	1
5180	0	0.99	1	0.91334	.	.	1

Obs	Sub_group	fuptimey	_CENSOR_	SURVIVAL	SDF_LCL	SDF_UCL	STRATUM
5181	0	0.99	1	0.91334	.	.	1
5182	0	0.99	1	0.91334	.	.	1
5183	0	0.99	1	0.91334	.	.	1
5184	0	0.99	1	0.91334	.	.	1
5185	0	0.99	1	0.91334	.	.	1
5186	0	0.99	1	0.91334	.	.	1
5187	0	0.99	1	0.91334	.	.	1
5188	0	0.99	1	0.91334	.	.	1
5189	0	0.99	1	0.91334	.	.	1
5190	0	0.99	1	0.91334	.	.	1
5191	0	0.99	1	0.91334	.	.	1
5192	0	0.99	1	0.91334	.	.	1
5193	0	0.99	1	0.91334	.	.	1
5194	0	1.00	0	0.91293	0.91028	0.91550	1
5195	0	1.00	1	0.91293	.	.	1
5196	0	1.00	1	0.91293	.	.	1
5197	0	1.00	1	0.91293	.	.	1
5198	0	1.00	1	0.91293	.	.	1
5199	0	1.00	1	0.91293	.	.	1
5200	0	1.00	1	0.91293	.	.	1
5201	0	1.00	1	0.91293	.	.	1
5202	0	1.00	1	0.91293	.	.	1
5203	0	1.00	1	0.91293	.	.	1
5204	0	1.00	1	0.91293	.	.	1
5205	0	1.00	1	0.91293	.	.	1
5206	0	1.00	1	0.91293	.	.	1
5207	0	1.00	1	0.91293	.	.	1
5208	0	1.00	1	0.91293	.	.	1
5209	0	1.00	1	0.91293	.	.	1
5210	0	1.00	1	0.91293	.	.	1
5211	0	1.00	1	0.91293	.	.	1
5212	0	1.00	1	0.91293	.	.	1
5213	0	1.00	1	0.91293	.	.	1
5214	0	1.00	1	0.91293	.	.	1
5215	0	1.00	1	0.91293	.	.	1

Obs	Sub_group	fuptimey	_CENSOR_	SURVIVAL	SDF_LCL	SDF_UCL	STRATUM
5216	0	1.00	1	0.91293	.	.	1
5217	0	1.00	1	0.91293	.	.	1
5218	0	1.00	1	0.91293	.	.	1
5219	0	1.00	1	0.91293	.	.	1
5220	0	1.00	1	0.91293	.	.	1
5221	0	1.00	1	0.91293	.	.	1
5222	0	1.00	1	0.91293	.	.	1
5223	0	1.00	1	0.91293	.	.	1
5224	0	1.00	1	0.91293	.	.	1
5225	0	1.00	1	0.91293	.	.	1
5226	0	1.00	1	0.91293	.	.	1
5227	0	1.00	1	0.91293	.	.	1
5228	0	1.00	1	0.91293	.	.	1
5229	0	1.00	1	0.91293	.	.	1
5230	0	1.00	1	0.91293	.	.	1
5231	0	1.00	1	0.91293	.	.	1
5232	0	1.00	1	0.91293	.	.	1
5233	0	1.00	1	0.91293	.	.	1
5234	0	1.00	1	0.91293	.	.	1
5235	0	1.00	1	0.91293	.	.	1
5236	0	1.00	1	0.91293	.	.	1
5237	0	1.00	1	0.91293	.	.	1
5238	0	1.00	1	0.91293	.	.	1
5239	0	1.00	1	0.91293	.	.	1
5240	0	1.00	1	0.91293	.	.	1
5241	0	1.00	1	0.91293	.	.	1
5242	0	1.00	1	0.91293	.	.	1
5243	0	1.00	1	0.91293	.	.	1
5244	0	1.00	1	0.91293	.	.	1
5245	0	1.00	1	0.91293	.	.	1
5246	0	1.00	1	0.91293	.	.	1
5247	0	1.00	1	0.91293	.	.	1
5248	0	1.00	1	0.91293	.	.	1
5249	0	1.00	1	0.91293	.	.	1
5250	0	1.00	1	0.91293	.	.	1

Obs	Sub_group	fuptimey	_CENSOR_	SURVIVAL	SDF_LCL	SDF_UCL	STRATUM
5251	0	1.00	1	0.91293	.	.	1
5252	0	1.00	1	0.91293	.	.	1
5253	0	1.00	1	0.91293	.	.	1
5254	0	1.00	1	0.91293	.	.	1
5255	0	1.00	1	0.91293	.	.	1
5256	0	1.01	0	0.91244	0.90979	0.91503	1
5257	0	1.01	1	0.91244	.	.	1
5258	0	1.01	1	0.91244	.	.	1
5259	0	1.01	1	0.91244	.	.	1
5260	0	1.01	1	0.91244	.	.	1
5261	0	1.01	1	0.91244	.	.	1
5262	0	1.01	1	0.91244	.	.	1
5263	0	1.01	1	0.91244	.	.	1
5264	0	1.01	1	0.91244	.	.	1
5265	0	1.01	1	0.91244	.	.	1
5266	0	1.01	1	0.91244	.	.	1
5267	0	1.01	1	0.91244	.	.	1
5268	0	1.01	1	0.91244	.	.	1
5269	0	1.01	1	0.91244	.	.	1
5270	0	1.01	1	0.91244	.	.	1
5271	0	1.01	1	0.91244	.	.	1
5272	0	1.01	1	0.91244	.	.	1
5273	0	1.01	1	0.91244	.	.	1
5274	0	1.01	1	0.91244	.	.	1
5275	0	1.01	1	0.91244	.	.	1
5276	0	1.01	1	0.91244	.	.	1
5277	0	1.01	1	0.91244	.	.	1
5278	0	1.01	1	0.91244	.	.	1
5279	0	1.01	1	0.91244	.	.	1
5280	0	1.01	1	0.91244	.	.	1
5281	0	1.01	1	0.91244	.	.	1
5282	0	1.01	1	0.91244	.	.	1
5283	0	1.01	1	0.91244	.	.	1
5284	0	1.01	1	0.91244	.	.	1
5285	0	1.01	1	0.91244	.	.	1

Obs	Sub_group	fuptimey	_CENSOR_	SURVIVAL	SDF_LCL	SDF_UCL	STRATUM
**5286**	0	1.01	1	0.91244	.	.	1
**5287**	0	1.01	1	0.91244	.	.	1
**5288**	0	1.01	1	0.91244	.	.	1
**5289**	0	1.01	1	0.91244	.	.	1
**5290**	0	1.01	1	0.91244	.	.	1
**5291**	0	1.01	1	0.91244	.	.	1
**5292**	0	1.01	1	0.91244	.	.	1
**5293**	0	1.01	1	0.91244	.	.	1
**5294**	0	1.01	1	0.91244	.	.	1
**5295**	0	1.01	1	0.91244	.	.	1
**5296**	0	1.01	1	0.91244	.	.	1
**5297**	0	1.01	1	0.91244	.	.	1
**5298**	0	1.01	1	0.91244	.	.	1
**5299**	0	1.01	1	0.91244	.	.	1
**5300**	0	1.01	1	0.91244	.	.	1
**5301**	0	1.02	0	0.91210	0.90944	0.91469	1
**5302**	0	1.02	1	0.91210	.	.	1
**5303**	0	1.02	1	0.91210	.	.	1
**5304**	0	1.02	1	0.91210	.	.	1
**5305**	0	1.02	1	0.91210	.	.	1
**5306**	0	1.02	1	0.91210	.	.	1
**5307**	0	1.02	1	0.91210	.	.	1
**5308**	0	1.02	1	0.91210	.	.	1
**5309**	0	1.02	1	0.91210	.	.	1
**5310**	0	1.02	1	0.91210	.	.	1
**5311**	0	1.02	1	0.91210	.	.	1
**5312**	0	1.02	1	0.91210	.	.	1
**5313**	0	1.02	1	0.91210	.	.	1
**5314**	0	1.02	1	0.91210	.	.	1
**5315**	0	1.02	1	0.91210	.	.	1
**5316**	0	1.02	1	0.91210	.	.	1
**5317**	0	1.02	1	0.91210	.	.	1
**5318**	0	1.02	1	0.91210	.	.	1
**5319**	0	1.02	1	0.91210	.	.	1
**5320**	0	1.02	1	0.91210	.	.	1

Obs	Sub_group	fuptimey	_CENSOR_	SURVIVAL	SDF_LCL	SDF_UCL	STRATUM
5321	0	1.02	1	0.91210	.	.	1
5322	0	1.02	1	0.91210	.	.	1
5323	0	1.02	1	0.91210	.	.	1
5324	0	1.02	1	0.91210	.	.	1
5325	0	1.02	1	0.91210	.	.	1
5326	0	1.02	1	0.91210	.	.	1
5327	0	1.02	1	0.91210	.	.	1
5328	0	1.02	1	0.91210	.	.	1
5329	0	1.02	1	0.91210	.	.	1
5330	0	1.02	1	0.91210	.	.	1
5331	0	1.02	1	0.91210	.	.	1
5332	0	1.02	1	0.91210	.	.	1
5333	0	1.02	1	0.91210	.	.	1
5334	0	1.02	1	0.91210	.	.	1
5335	0	1.02	1	0.91210	.	.	1
5336	0	1.02	1	0.91210	.	.	1
5337	0	1.02	1	0.91210	.	.	1
5338	0	1.02	1	0.91210	.	.	1
5339	0	1.02	1	0.91210	.	.	1
5340	0	1.02	1	0.91210	.	.	1
5341	0	1.02	1	0.91210	.	.	1
5342	0	1.02	1	0.91210	.	.	1
5343	0	1.02	1	0.91210	.	.	1
5344	0	1.02	1	0.91210	.	.	1
5345	0	1.02	1	0.91210	.	.	1
5346	0	1.02	1	0.91210	.	.	1
5347	0	1.02	1	0.91210	.	.	1
5348	0	1.02	1	0.91210	.	.	1
5349	0	1.02	1	0.91210	.	.	1
5350	0	1.02	1	0.91210	.	.	1
5351	0	1.02	1	0.91210	.	.	1
5352	0	1.02	1	0.91210	.	.	1
5353	0	1.02	1	0.91210	.	.	1
5354	0	1.02	1	0.91210	.	.	1
5355	0	1.02	1	0.91210	.	.	1

Obs	Sub_group	fuptimey	_CENSOR_	SURVIVAL	SDF_LCL	SDF_UCL	STRATUM
5356	0	1.02	1	0.91210	.	.	1
5357	0	1.02	1	0.91210	.	.	1
5358	0	1.02	1	0.91210	.	.	1
5359	0	1.02	1	0.91210	.	.	1
5360	0	1.02	1	0.91210	.	.	1
5361	0	1.02	1	0.91210	.	.	1
5362	0	1.02	1	0.91210	.	.	1
5363	0	1.02	1	0.91210	.	.	1
5364	0	1.02	1	0.91210	.	.	1
5365	0	1.02	1	0.91210	.	.	1
5366	0	1.02	1	0.91210	.	.	1
5367	0	1.03	0	0.91162	0.90895	0.91421	1
5368	0	1.03	1	0.91162	.	.	1
5369	0	1.03	1	0.91162	.	.	1
5370	0	1.03	1	0.91162	.	.	1
5371	0	1.03	1	0.91162	.	.	1
5372	0	1.03	1	0.91162	.	.	1
5373	0	1.03	1	0.91162	.	.	1
5374	0	1.03	1	0.91162	.	.	1
5375	0	1.03	1	0.91162	.	.	1
5376	0	1.03	1	0.91162	.	.	1
5377	0	1.03	1	0.91162	.	.	1
5378	0	1.03	1	0.91162	.	.	1
5379	0	1.03	1	0.91162	.	.	1
5380	0	1.03	1	0.91162	.	.	1
5381	0	1.03	1	0.91162	.	.	1
5382	0	1.03	1	0.91162	.	.	1
5383	0	1.03	1	0.91162	.	.	1
5384	0	1.03	1	0.91162	.	.	1
5385	0	1.03	1	0.91162	.	.	1
5386	0	1.03	1	0.91162	.	.	1
5387	0	1.03	1	0.91162	.	.	1
5388	0	1.03	1	0.91162	.	.	1
5389	0	1.03	1	0.91162	.	.	1
5390	0	1.03	1	0.91162	.	.	1

Obs	Sub_group	fuptimey	_CENSOR_	SURVIVAL	SDF_LCL	SDF_UCL	STRATUM
5391	0	1.03	1	0.91162	.	.	1
5392	0	1.03	1	0.91162	.	.	1
5393	0	1.03	1	0.91162	.	.	1
5394	0	1.03	1	0.91162	.	.	1
5395	0	1.03	1	0.91162	.	.	1
5396	0	1.03	1	0.91162	.	.	1
5397	0	1.03	1	0.91162	.	.	1
5398	0	1.03	1	0.91162	.	.	1
5399	0	1.03	1	0.91162	.	.	1
5400	0	1.03	1	0.91162	.	.	1
5401	0	1.03	1	0.91162	.	.	1
5402	0	1.03	1	0.91162	.	.	1
5403	0	1.03	1	0.91162	.	.	1
5404	0	1.03	1	0.91162	.	.	1
5405	0	1.03	1	0.91162	.	.	1
5406	0	1.03	1	0.91162	.	.	1
5407	0	1.03	1	0.91162	.	.	1
5408	0	1.03	1	0.91162	.	.	1
5409	0	1.03	1	0.91162	.	.	1
5410	0	1.03	1	0.91162	.	.	1
5411	0	1.03	1	0.91162	.	.	1
5412	0	1.03	1	0.91162	.	.	1
5413	0	1.04	0	0.91103	0.90835	0.91363	1
5414	0	1.04	1	0.91103	.	.	1
5415	0	1.04	1	0.91103	.	.	1
5416	0	1.04	1	0.91103	.	.	1
5417	0	1.04	1	0.91103	.	.	1
5418	0	1.04	1	0.91103	.	.	1
5419	0	1.04	1	0.91103	.	.	1
5420	0	1.04	1	0.91103	.	.	1
5421	0	1.04	1	0.91103	.	.	1
5422	0	1.04	1	0.91103	.	.	1
5423	0	1.04	1	0.91103	.	.	1
5424	0	1.04	1	0.91103	.	.	1
5425	0	1.04	1	0.91103	.	.	1

Obs	Sub_group	fuptimey	_CENSOR_	SURVIVAL	SDF_LCL	SDF_UCL	STRATUM
5426	0	1.04	1	0.91103	.	.	1
5427	0	1.04	1	0.91103	.	.	1
5428	0	1.04	1	0.91103	.	.	1
5429	0	1.04	1	0.91103	.	.	1
5430	0	1.04	1	0.91103	.	.	1
5431	0	1.04	1	0.91103	.	.	1
5432	0	1.04	1	0.91103	.	.	1
5433	0	1.04	1	0.91103	.	.	1
5434	0	1.04	1	0.91103	.	.	1
5435	0	1.04	1	0.91103	.	.	1
5436	0	1.04	1	0.91103	.	.	1
5437	0	1.04	1	0.91103	.	.	1
5438	0	1.04	1	0.91103	.	.	1
5439	0	1.04	1	0.91103	.	.	1
5440	0	1.04	1	0.91103	.	.	1
5441	0	1.04	1	0.91103	.	.	1
5442	0	1.04	1	0.91103	.	.	1
5443	0	1.04	1	0.91103	.	.	1
5444	0	1.04	1	0.91103	.	.	1
5445	0	1.04	1	0.91103	.	.	1
5446	0	1.04	1	0.91103	.	.	1
5447	0	1.04	1	0.91103	.	.	1
5448	0	1.04	1	0.91103	.	.	1
5449	0	1.04	1	0.91103	.	.	1
5450	0	1.04	1	0.91103	.	.	1
5451	0	1.04	1	0.91103	.	.	1
5452	0	1.04	1	0.91103	.	.	1
5453	0	1.04	1	0.91103	.	.	1
5454	0	1.04	1	0.91103	.	.	1
5455	0	1.04	1	0.91103	.	.	1
5456	0	1.04	1	0.91103	.	.	1
5457	0	1.04	1	0.91103	.	.	1
5458	0	1.04	1	0.91103	.	.	1
5459	0	1.04	1	0.91103	.	.	1
5460	0	1.04	1	0.91103	.	.	1

Obs	Sub_group	fuptimey	_CENSOR_	SURVIVAL	SDF_LCL	SDF_UCL	STRATUM
5461	0	1.04	1	0.91103	.	.	1
5462	0	1.04	1	0.91103	.	.	1
5463	0	1.04	1	0.91103	.	.	1
5464	0	1.04	1	0.91103	.	.	1
5465	0	1.04	1	0.91103	.	.	1
5466	0	1.04	1	0.91103	.	.	1
5467	0	1.04	1	0.91103	.	.	1
5468	0	1.04	1	0.91103	.	.	1
5469	0	1.04	1	0.91103	.	.	1
5470	0	1.04	1	0.91103	.	.	1
5471	0	1.04	1	0.91103	.	.	1
5472	0	1.04	1	0.91103	.	.	1
5473	0	1.04	1	0.91103	.	.	1
5474	0	1.04	1	0.91103	.	.	1
5475	0	1.04	1	0.91103	.	.	1
5476	0	1.04	1	0.91103	.	.	1
5477	0	1.04	1	0.91103	.	.	1
5478	0	1.04	1	0.91103	.	.	1
5479	0	1.04	1	0.91103	.	.	1
5480	0	1.04	1	0.91103	.	.	1
5481	0	1.04	1	0.91103	.	.	1
5482	0	1.04	1	0.91103	.	.	1
5483	0	1.04	1	0.91103	.	.	1
5484	0	1.04	1	0.91103	.	.	1
5485	0	1.05	0	0.91057	0.90788	0.91318	1
5486	0	1.05	1	0.91057	.	.	1
5487	0	1.05	1	0.91057	.	.	1
5488	0	1.05	1	0.91057	.	.	1
5489	0	1.05	1	0.91057	.	.	1
5490	0	1.05	1	0.91057	.	.	1
5491	0	1.05	1	0.91057	.	.	1
5492	0	1.05	1	0.91057	.	.	1
5493	0	1.05	1	0.91057	.	.	1
5494	0	1.05	1	0.91057	.	.	1
5495	0	1.05	1	0.91057	.	.	1

Obs	Sub_group	fuptimey	_CENSOR_	SURVIVAL	SDF_LCL	SDF_UCL	STRATUM
5496	0	1.05	1	0.91057	.	.	1
5497	0	1.05	1	0.91057	.	.	1
5498	0	1.05	1	0.91057	.	.	1
5499	0	1.05	1	0.91057	.	.	1
5500	0	1.05	1	0.91057	.	.	1
5501	0	1.05	1	0.91057	.	.	1
5502	0	1.05	1	0.91057	.	.	1
5503	0	1.05	1	0.91057	.	.	1
5504	0	1.05	1	0.91057	.	.	1
5505	0	1.05	1	0.91057	.	.	1
5506	0	1.05	1	0.91057	.	.	1
5507	0	1.05	1	0.91057	.	.	1
5508	0	1.05	1	0.91057	.	.	1
5509	0	1.05	1	0.91057	.	.	1
5510	0	1.05	1	0.91057	.	.	1
5511	0	1.05	1	0.91057	.	.	1
5512	0	1.05	1	0.91057	.	.	1
5513	0	1.05	1	0.91057	.	.	1
5514	0	1.05	1	0.91057	.	.	1
5515	0	1.05	1	0.91057	.	.	1
5516	0	1.05	1	0.91057	.	.	1
5517	0	1.05	1	0.91057	.	.	1
5518	0	1.05	1	0.91057	.	.	1
5519	0	1.05	1	0.91057	.	.	1
5520	0	1.05	1	0.91057	.	.	1
5521	0	1.05	1	0.91057	.	.	1
5522	0	1.05	1	0.91057	.	.	1
5523	0	1.05	1	0.91057	.	.	1
5524	0	1.05	1	0.91057	.	.	1
5525	0	1.05	1	0.91057	.	.	1
5526	0	1.05	1	0.91057	.	.	1
5527	0	1.05	1	0.91057	.	.	1
5528	0	1.05	1	0.91057	.	.	1
5529	0	1.05	1	0.91057	.	.	1
5530	0	1.05	1	0.91057	.	.	1

Obs	Sub_group	fuptimey	_CENSOR_	SURVIVAL	SDF_LCL	SDF_UCL	STRATUM
5531	0	1.05	1	0.91057	.	.	1
5532	0	1.06	0	0.91032	0.90763	0.91294	1
5533	0	1.06	1	0.91032	.	.	1
5534	0	1.06	1	0.91032	.	.	1
5535	0	1.06	1	0.91032	.	.	1
5536	0	1.06	1	0.91032	.	.	1
5537	0	1.06	1	0.91032	.	.	1
5538	0	1.06	1	0.91032	.	.	1
5539	0	1.06	1	0.91032	.	.	1
5540	0	1.06	1	0.91032	.	.	1
5541	0	1.06	1	0.91032	.	.	1
5542	0	1.06	1	0.91032	.	.	1
5543	0	1.06	1	0.91032	.	.	1
5544	0	1.06	1	0.91032	.	.	1
5545	0	1.06	1	0.91032	.	.	1
5546	0	1.06	1	0.91032	.	.	1
5547	0	1.06	1	0.91032	.	.	1
5548	0	1.06	1	0.91032	.	.	1
5549	0	1.06	1	0.91032	.	.	1
5550	0	1.06	1	0.91032	.	.	1
5551	0	1.06	1	0.91032	.	.	1
5552	0	1.06	1	0.91032	.	.	1
5553	0	1.06	1	0.91032	.	.	1
5554	0	1.06	1	0.91032	.	.	1
5555	0	1.06	1	0.91032	.	.	1
5556	0	1.06	1	0.91032	.	.	1
5557	0	1.06	1	0.91032	.	.	1
5558	0	1.06	1	0.91032	.	.	1
5559	0	1.06	1	0.91032	.	.	1
5560	0	1.06	1	0.91032	.	.	1
5561	0	1.06	1	0.91032	.	.	1
5562	0	1.06	1	0.91032	.	.	1
5563	0	1.06	1	0.91032	.	.	1
5564	0	1.06	1	0.91032	.	.	1
5565	0	1.06	1	0.91032	.	.	1

Obs	Sub_group	fuptimey	_CENSOR_	SURVIVAL	SDF_LCL	SDF_UCL	STRATUM
5566	0	1.06	1	0.91032	.	.	1
5567	0	1.06	1	0.91032	.	.	1
5568	0	1.06	1	0.91032	.	.	1
5569	0	1.06	1	0.91032	.	.	1
5570	0	1.06	1	0.91032	.	.	1
5571	0	1.06	1	0.91032	.	.	1
5572	0	1.06	1	0.91032	.	.	1
5573	0	1.06	1	0.91032	.	.	1
5574	0	1.06	1	0.91032	.	.	1
5575	0	1.06	1	0.91032	.	.	1
5576	0	1.06	1	0.91032	.	.	1
5577	0	1.06	1	0.91032	.	.	1
5578	0	1.06	1	0.91032	.	.	1
5579	0	1.06	1	0.91032	.	.	1
5580	0	1.06	1	0.91032	.	.	1
5581	0	1.06	1	0.91032	.	.	1
5582	0	1.06	1	0.91032	.	.	1
5583	0	1.06	1	0.91032	.	.	1
5584	0	1.06	1	0.91032	.	.	1
5585	0	1.06	1	0.91032	.	.	1
5586	0	1.06	1	0.91032	.	.	1
5587	0	1.06	1	0.91032	.	.	1
5588	0	1.06	1	0.91032	.	.	1
5589	0	1.06	1	0.91032	.	.	1
5590	0	1.06	1	0.91032	.	.	1
5591	0	1.06	1	0.91032	.	.	1
5592	0	1.06	1	0.91032	.	.	1
5593	0	1.06	1	0.91032	.	.	1
5594	0	1.06	1	0.91032	.	.	1
5595	0	1.06	1	0.91032	.	.	1
5596	0	1.06	1	0.91032	.	.	1
5597	0	1.06	1	0.91032	.	.	1
5598	0	1.06	1	0.91032	.	.	1
5599	0	1.06	1	0.91032	.	.	1
5600	0	1.06	1	0.91032	.	.	1

Obs	Sub_group	fuptimey	_CENSOR_	SURVIVAL	SDF_LCL	SDF_UCL	STRATUM
5601	0	1.06	1	0.91032	.	.	1
5602	0	1.06	1	0.91032	.	.	1
5603	0	1.06	1	0.91032	.	.	1
5604	0	1.07	0	0.91005	0.90736	0.91267	1
5605	0	1.07	1	0.91005	.	.	1
5606	0	1.07	1	0.91005	.	.	1
5607	0	1.07	1	0.91005	.	.	1
5608	0	1.07	1	0.91005	.	.	1
5609	0	1.07	1	0.91005	.	.	1
5610	0	1.07	1	0.91005	.	.	1
5611	0	1.07	1	0.91005	.	.	1
5612	0	1.07	1	0.91005	.	.	1
5613	0	1.07	1	0.91005	.	.	1
5614	0	1.07	1	0.91005	.	.	1
5615	0	1.07	1	0.91005	.	.	1
5616	0	1.07	1	0.91005	.	.	1
5617	0	1.07	1	0.91005	.	.	1
5618	0	1.07	1	0.91005	.	.	1
5619	0	1.07	1	0.91005	.	.	1
5620	0	1.07	1	0.91005	.	.	1
5621	0	1.07	1	0.91005	.	.	1
5622	0	1.07	1	0.91005	.	.	1
5623	0	1.07	1	0.91005	.	.	1
5624	0	1.07	1	0.91005	.	.	1
5625	0	1.07	1	0.91005	.	.	1
5626	0	1.07	1	0.91005	.	.	1
5627	0	1.07	1	0.91005	.	.	1
5628	0	1.07	1	0.91005	.	.	1
5629	0	1.07	1	0.91005	.	.	1
5630	0	1.07	1	0.91005	.	.	1
5631	0	1.07	1	0.91005	.	.	1
5632	0	1.07	1	0.91005	.	.	1
5633	0	1.07	1	0.91005	.	.	1
5634	0	1.07	1	0.91005	.	.	1
5635	0	1.07	1	0.91005	.	.	1

Obs	Sub_group	fuptimey	_CENSOR_	SURVIVAL	SDF_LCL	SDF_UCL	STRATUM
5636	0	1.07	1	0.91005	.	.	1
5637	0	1.07	1	0.91005	.	.	1
5638	0	1.07	1	0.91005	.	.	1
5639	0	1.07	1	0.91005	.	.	1
5640	0	1.07	1	0.91005	.	.	1
5641	0	1.07	1	0.91005	.	.	1
5642	0	1.07	1	0.91005	.	.	1
5643	0	1.07	1	0.91005	.	.	1
5644	0	1.08	0	0.90951	0.90681	0.91214	1
5645	0	1.08	1	0.90951	.	.	1
5646	0	1.08	1	0.90951	.	.	1
5647	0	1.08	1	0.90951	.	.	1
5648	0	1.08	1	0.90951	.	.	1
5649	0	1.08	1	0.90951	.	.	1
5650	0	1.08	1	0.90951	.	.	1
5651	0	1.08	1	0.90951	.	.	1
5652	0	1.08	1	0.90951	.	.	1
5653	0	1.08	1	0.90951	.	.	1
5654	0	1.08	1	0.90951	.	.	1
5655	0	1.08	1	0.90951	.	.	1
5656	0	1.08	1	0.90951	.	.	1
5657	0	1.08	1	0.90951	.	.	1
5658	0	1.08	1	0.90951	.	.	1
5659	0	1.08	1	0.90951	.	.	1
5660	0	1.08	1	0.90951	.	.	1
5661	0	1.08	1	0.90951	.	.	1
5662	0	1.08	1	0.90951	.	.	1
5663	0	1.08	1	0.90951	.	.	1
5664	0	1.08	1	0.90951	.	.	1
5665	0	1.08	1	0.90951	.	.	1
5666	0	1.08	1	0.90951	.	.	1
5667	0	1.08	1	0.90951	.	.	1
5668	0	1.08	1	0.90951	.	.	1
5669	0	1.08	1	0.90951	.	.	1
5670	0	1.08	1	0.90951	.	.	1

Obs	Sub_group	fuptimey	_CENSOR_	SURVIVAL	SDF_LCL	SDF_UCL	STRATUM
5671	0	1.08	1	0.90951	.	.	1
5672	0	1.08	1	0.90951	.	.	1
5673	0	1.08	1	0.90951	.	.	1
5674	0	1.08	1	0.90951	.	.	1
5675	0	1.08	1	0.90951	.	.	1
5676	0	1.08	1	0.90951	.	.	1
5677	0	1.08	1	0.90951	.	.	1
5678	0	1.08	1	0.90951	.	.	1
5679	0	1.08	1	0.90951	.	.	1
5680	0	1.08	1	0.90951	.	.	1
5681	0	1.08	1	0.90951	.	.	1
5682	0	1.08	1	0.90951	.	.	1
5683	0	1.08	1	0.90951	.	.	1
5684	0	1.08	1	0.90951	.	.	1
5685	0	1.08	1	0.90951	.	.	1
5686	0	1.08	1	0.90951	.	.	1
5687	0	1.08	1	0.90951	.	.	1
5688	0	1.08	1	0.90951	.	.	1
5689	0	1.08	1	0.90951	.	.	1
5690	0	1.08	1	0.90951	.	.	1
5691	0	1.08	1	0.90951	.	.	1
5692	0	1.08	1	0.90951	.	.	1
5693	0	1.08	1	0.90951	.	.	1
5694	0	1.08	1	0.90951	.	.	1
5695	0	1.08	1	0.90951	.	.	1
5696	0	1.08	1	0.90951	.	.	1
5697	0	1.08	1	0.90951	.	.	1
5698	0	1.08	1	0.90951	.	.	1
5699	0	1.08	1	0.90951	.	.	1
5700	0	1.08	1	0.90951	.	.	1
5701	0	1.08	1	0.90951	.	.	1
5702	0	1.08	1	0.90951	.	.	1
5703	0	1.08	1	0.90951	.	.	1
5704	0	1.08	1	0.90951	.	.	1
5705	0	1.08	1	0.90951	.	.	1

Obs	Sub_group	fuptimey	_CENSOR_	SURVIVAL	SDF_LCL	SDF_UCL	STRATUM
5706	0	1.08	1	0.90951	.	.	1
5707	0	1.08	1	0.90951	.	.	1
5708	0	1.08	1	0.90951	.	.	1
5709	0	1.08	1	0.90951	.	.	1
5710	0	1.08	1	0.90951	.	.	1
5711	0	1.08	1	0.90951	.	.	1
5712	0	1.09	0	0.90902	0.90631	0.91165	1
5713	0	1.09	1	0.90902	.	.	1
5714	0	1.09	1	0.90902	.	.	1
5715	0	1.09	1	0.90902	.	.	1
5716	0	1.09	1	0.90902	.	.	1
5717	0	1.09	1	0.90902	.	.	1
5718	0	1.09	1	0.90902	.	.	1
5719	0	1.09	1	0.90902	.	.	1
5720	0	1.09	1	0.90902	.	.	1
5721	0	1.09	1	0.90902	.	.	1
5722	0	1.09	1	0.90902	.	.	1
5723	0	1.09	1	0.90902	.	.	1
5724	0	1.09	1	0.90902	.	.	1
5725	0	1.09	1	0.90902	.	.	1
5726	0	1.09	1	0.90902	.	.	1
5727	0	1.09	1	0.90902	.	.	1
5728	0	1.09	1	0.90902	.	.	1
5729	0	1.09	1	0.90902	.	.	1
5730	0	1.09	1	0.90902	.	.	1
5731	0	1.09	1	0.90902	.	.	1
5732	0	1.09	1	0.90902	.	.	1
5733	0	1.09	1	0.90902	.	.	1
5734	0	1.09	1	0.90902	.	.	1
5735	0	1.09	1	0.90902	.	.	1
5736	0	1.09	1	0.90902	.	.	1
5737	0	1.09	1	0.90902	.	.	1
5738	0	1.09	1	0.90902	.	.	1
5739	0	1.09	1	0.90902	.	.	1
5740	0	1.09	1	0.90902	.	.	1

Obs	Sub_group	fuptimey	_CENSOR_	SURVIVAL	SDF_LCL	SDF_UCL	STRATUM
5741	0	1.09	1	0.90902	.	.	1
5742	0	1.09	1	0.90902	.	.	1
5743	0	1.09	1	0.90902	.	.	1
5744	0	1.09	1	0.90902	.	.	1
5745	0	1.09	1	0.90902	.	.	1
5746	0	1.09	1	0.90902	.	.	1
5747	0	1.09	1	0.90902	.	.	1
5748	0	1.09	1	0.90902	.	.	1
5749	0	1.09	1	0.90902	.	.	1
5750	0	1.09	1	0.90902	.	.	1
5751	0	1.09	1	0.90902	.	.	1
5752	0	1.09	1	0.90902	.	.	1
5753	0	1.09	1	0.90902	.	.	1
5754	0	1.09	1	0.90902	.	.	1
5755	0	1.09	1	0.90902	.	.	1
5756	0	1.09	1	0.90902	.	.	1
5757	0	1.09	1	0.90902	.	.	1
5758	0	1.09	1	0.90902	.	.	1
5759	0	1.09	1	0.90902	.	.	1
5760	0	1.09	1	0.90902	.	.	1
5761	0	1.09	1	0.90902	.	.	1
5762	0	1.09	1	0.90902	.	.	1
5763	0	1.09	1	0.90902	.	.	1
5764	0	1.09	1	0.90902	.	.	1
5765	0	1.09	1	0.90902	.	.	1
5766	0	1.09	1	0.90902	.	.	1
5767	0	1.09	1	0.90902	.	.	1
5768	0	1.09	1	0.90902	.	.	1
5769	0	1.09	1	0.90902	.	.	1
5770	0	1.09	1	0.90902	.	.	1
5771	0	1.09	1	0.90902	.	.	1
5772	0	1.09	1	0.90902	.	.	1
5773	0	1.09	1	0.90902	.	.	1
5774	0	1.09	1	0.90902	.	.	1
5775	0	1.09	1	0.90902	.	.	1

Obs	Sub_group	fuptimey	_CENSOR_	SURVIVAL	SDF_LCL	SDF_UCL	STRATUM
5776	0	1.10	0	0.90870	0.90599	0.91134	1
5777	0	1.10	1	0.90870	.	.	1
5778	0	1.10	1	0.90870	.	.	1
5779	0	1.10	1	0.90870	.	.	1
5780	0	1.10	1	0.90870	.	.	1
5781	0	1.10	1	0.90870	.	.	1
5782	0	1.10	1	0.90870	.	.	1
5783	0	1.10	1	0.90870	.	.	1
5784	0	1.10	1	0.90870	.	.	1
5785	0	1.10	1	0.90870	.	.	1
5786	0	1.10	1	0.90870	.	.	1
5787	0	1.10	1	0.90870	.	.	1
5788	0	1.10	1	0.90870	.	.	1
5789	0	1.10	1	0.90870	.	.	1
5790	0	1.10	1	0.90870	.	.	1
5791	0	1.10	1	0.90870	.	.	1
5792	0	1.10	1	0.90870	.	.	1
5793	0	1.10	1	0.90870	.	.	1
5794	0	1.10	1	0.90870	.	.	1
5795	0	1.10	1	0.90870	.	.	1
5796	0	1.10	1	0.90870	.	.	1
5797	0	1.10	1	0.90870	.	.	1
5798	0	1.10	1	0.90870	.	.	1
5799	0	1.10	1	0.90870	.	.	1
5800	0	1.10	1	0.90870	.	.	1
5801	0	1.10	1	0.90870	.	.	1
5802	0	1.10	1	0.90870	.	.	1
5803	0	1.10	1	0.90870	.	.	1
5804	0	1.10	1	0.90870	.	.	1
5805	0	1.10	1	0.90870	.	.	1
5806	0	1.10	1	0.90870	.	.	1
5807	0	1.10	1	0.90870	.	.	1
5808	0	1.10	1	0.90870	.	.	1
5809	0	1.10	1	0.90870	.	.	1
5810	0	1.10	1	0.90870	.	.	1

Obs	Sub_group	fuptimey	_CENSOR_	SURVIVAL	SDF_LCL	SDF_UCL	STRATUM
**5811**	0	1.10	1	0.90870	.	.	1
**5812**	0	1.10	1	0.90870	.	.	1
**5813**	0	1.10	1	0.90870	.	.	1
**5814**	0	1.10	1	0.90870	.	.	1
**5815**	0	1.10	1	0.90870	.	.	1
**5816**	0	1.10	1	0.90870	.	.	1
**5817**	0	1.10	1	0.90870	.	.	1
**5818**	0	1.10	1	0.90870	.	.	1
**5819**	0	1.10	1	0.90870	.	.	1
**5820**	0	1.10	1	0.90870	.	.	1
**5821**	0	1.10	1	0.90870	.	.	1
**5822**	0	1.10	1	0.90870	.	.	1
**5823**	0	1.10	1	0.90870	.	.	1
**5824**	0	1.10	1	0.90870	.	.	1
**5825**	0	1.10	1	0.90870	.	.	1
**5826**	0	1.10	1	0.90870	.	.	1
**5827**	0	1.10	1	0.90870	.	.	1
**5828**	0	1.10	1	0.90870	.	.	1
**5829**	0	1.10	1	0.90870	.	.	1
**5830**	0	1.10	1	0.90870	.	.	1
**5831**	0	1.10	1	0.90870	.	.	1
**5832**	0	1.10	1	0.90870	.	.	1
**5833**	0	1.10	1	0.90870	.	.	1
**5834**	0	1.10	1	0.90870	.	.	1
**5835**	0	1.10	1	0.90870	.	.	1
**5836**	0	1.10	1	0.90870	.	.	1
**5837**	0	1.10	1	0.90870	.	.	1
**5838**	0	1.10	1	0.90870	.	.	1
**5839**	0	1.11	0	0.90848	0.90576	0.91112	1
**5840**	0	1.11	1	0.90848	.	.	1
**5841**	0	1.11	1	0.90848	.	.	1
**5842**	0	1.11	1	0.90848	.	.	1
**5843**	0	1.11	1	0.90848	.	.	1
**5844**	0	1.11	1	0.90848	.	.	1
**5845**	0	1.11	1	0.90848	.	.	1

Obs	Sub_group	fuptimey	_CENSOR_	SURVIVAL	SDF_LCL	SDF_UCL	STRATUM
**5846**	0	1.11	1	0.90848	.	.	1
**5847**	0	1.11	1	0.90848	.	.	1
**5848**	0	1.11	1	0.90848	.	.	1
**5849**	0	1.11	1	0.90848	.	.	1
**5850**	0	1.11	1	0.90848	.	.	1
**5851**	0	1.11	1	0.90848	.	.	1
**5852**	0	1.11	1	0.90848	.	.	1
**5853**	0	1.11	1	0.90848	.	.	1
**5854**	0	1.11	1	0.90848	.	.	1
**5855**	0	1.11	1	0.90848	.	.	1
**5856**	0	1.11	1	0.90848	.	.	1
**5857**	0	1.11	1	0.90848	.	.	1
**5858**	0	1.11	1	0.90848	.	.	1
**5859**	0	1.11	1	0.90848	.	.	1
**5860**	0	1.11	1	0.90848	.	.	1
**5861**	0	1.11	1	0.90848	.	.	1
**5862**	0	1.11	1	0.90848	.	.	1
**5863**	0	1.11	1	0.90848	.	.	1
**5864**	0	1.11	1	0.90848	.	.	1
**5865**	0	1.11	1	0.90848	.	.	1
**5866**	0	1.11	1	0.90848	.	.	1
**5867**	0	1.11	1	0.90848	.	.	1
**5868**	0	1.11	1	0.90848	.	.	1
**5869**	0	1.11	1	0.90848	.	.	1
**5870**	0	1.11	1	0.90848	.	.	1
**5871**	0	1.11	1	0.90848	.	.	1
**5872**	0	1.11	1	0.90848	.	.	1
**5873**	0	1.11	1	0.90848	.	.	1
**5874**	0	1.11	1	0.90848	.	.	1
**5875**	0	1.11	1	0.90848	.	.	1
**5876**	0	1.11	1	0.90848	.	.	1
**5877**	0	1.11	1	0.90848	.	.	1
**5878**	0	1.11	1	0.90848	.	.	1
**5879**	0	1.11	1	0.90848	.	.	1
**5880**	0	1.11	1	0.90848	.	.	1

Obs	Sub_group	fuptimey	_CENSOR_	SURVIVAL	SDF_LCL	SDF_UCL	STRATUM
5881	0	1.11	1	0.90848	.	.	1
5882	0	1.11	1	0.90848	.	.	1
5883	0	1.11	1	0.90848	.	.	1
5884	0	1.11	1	0.90848	.	.	1
5885	0	1.11	1	0.90848	.	.	1
5886	0	1.11	1	0.90848	.	.	1
5887	0	1.12	0	0.90794	0.90521	0.91059	1
5888	0	1.12	1	0.90794	.	.	1
5889	0	1.12	1	0.90794	.	.	1
5890	0	1.12	1	0.90794	.	.	1
5891	0	1.12	1	0.90794	.	.	1
5892	0	1.12	1	0.90794	.	.	1
5893	0	1.12	1	0.90794	.	.	1
5894	0	1.12	1	0.90794	.	.	1
5895	0	1.12	1	0.90794	.	.	1
5896	0	1.12	1	0.90794	.	.	1
5897	0	1.12	1	0.90794	.	.	1
5898	0	1.12	1	0.90794	.	.	1
5899	0	1.12	1	0.90794	.	.	1
5900	0	1.12	1	0.90794	.	.	1
5901	0	1.12	1	0.90794	.	.	1
5902	0	1.12	1	0.90794	.	.	1
5903	0	1.12	1	0.90794	.	.	1
5904	0	1.12	1	0.90794	.	.	1
5905	0	1.12	1	0.90794	.	.	1
5906	0	1.12	1	0.90794	.	.	1
5907	0	1.12	1	0.90794	.	.	1
5908	0	1.12	1	0.90794	.	.	1
5909	0	1.12	1	0.90794	.	.	1
5910	0	1.12	1	0.90794	.	.	1
5911	0	1.12	1	0.90794	.	.	1
5912	0	1.12	1	0.90794	.	.	1
5913	0	1.12	1	0.90794	.	.	1
5914	0	1.12	1	0.90794	.	.	1
5915	0	1.12	1	0.90794	.	.	1

Obs	Sub_group	fuptimey	_CENSOR_	SURVIVAL	SDF_LCL	SDF_UCL	STRATUM
5916	0	1.12	1	0.90794	.	.	1
5917	0	1.12	1	0.90794	.	.	1
5918	0	1.12	1	0.90794	.	.	1
5919	0	1.12	1	0.90794	.	.	1
5920	0	1.12	1	0.90794	.	.	1
5921	0	1.12	1	0.90794	.	.	1
5922	0	1.12	1	0.90794	.	.	1
5923	0	1.12	1	0.90794	.	.	1
5924	0	1.12	1	0.90794	.	.	1
5925	0	1.12	1	0.90794	.	.	1
5926	0	1.12	1	0.90794	.	.	1
5927	0	1.12	1	0.90794	.	.	1
5928	0	1.12	1	0.90794	.	.	1
5929	0	1.12	1	0.90794	.	.	1
5930	0	1.12	1	0.90794	.	.	1
5931	0	1.12	1	0.90794	.	.	1
5932	0	1.12	1	0.90794	.	.	1
5933	0	1.12	1	0.90794	.	.	1
5934	0	1.12	1	0.90794	.	.	1
5935	0	1.12	1	0.90794	.	.	1
5936	0	1.12	1	0.90794	.	.	1
5937	0	1.12	1	0.90794	.	.	1
5938	0	1.12	1	0.90794	.	.	1
5939	0	1.12	1	0.90794	.	.	1
5940	0	1.12	1	0.90794	.	.	1
5941	0	1.12	1	0.90794	.	.	1
5942	0	1.12	1	0.90794	.	.	1
5943	0	1.12	1	0.90794	.	.	1
5944	0	1.12	1	0.90794	.	.	1
5945	0	1.12	1	0.90794	.	.	1
5946	0	1.12	1	0.90794	.	.	1
5947	0	1.12	1	0.90794	.	.	1
5948	0	1.12	1	0.90794	.	.	1
5949	0	1.12	1	0.90794	.	.	1
5950	0	1.12	1	0.90794	.	.	1

Obs	Sub_group	fuptimey	_CENSOR_	SURVIVAL	SDF_LCL	SDF_UCL	STRATUM
5951	0	1.12	1	0.90794	.	.	1
5952	0	1.12	1	0.90794	.	.	1
5953	0	1.12	1	0.90794	.	.	1
5954	0	1.12	1	0.90794	.	.	1
5955	0	1.12	1	0.90794	.	.	1
5956	0	1.13	0	0.90761	0.90489	0.91027	1
5957	0	1.13	1	0.90761	.	.	1
5958	0	1.13	1	0.90761	.	.	1
5959	0	1.13	1	0.90761	.	.	1
5960	0	1.13	1	0.90761	.	.	1
5961	0	1.13	1	0.90761	.	.	1
5962	0	1.13	1	0.90761	.	.	1
5963	0	1.13	1	0.90761	.	.	1
5964	0	1.13	1	0.90761	.	.	1
5965	0	1.13	1	0.90761	.	.	1
5966	0	1.13	1	0.90761	.	.	1
5967	0	1.13	1	0.90761	.	.	1
5968	0	1.13	1	0.90761	.	.	1
5969	0	1.13	1	0.90761	.	.	1
5970	0	1.13	1	0.90761	.	.	1
5971	0	1.13	1	0.90761	.	.	1
5972	0	1.13	1	0.90761	.	.	1
5973	0	1.13	1	0.90761	.	.	1
5974	0	1.13	1	0.90761	.	.	1
5975	0	1.13	1	0.90761	.	.	1
5976	0	1.13	1	0.90761	.	.	1
5977	0	1.13	1	0.90761	.	.	1
5978	0	1.13	1	0.90761	.	.	1
5979	0	1.13	1	0.90761	.	.	1
5980	0	1.13	1	0.90761	.	.	1
5981	0	1.13	1	0.90761	.	.	1
5982	0	1.13	1	0.90761	.	.	1
5983	0	1.13	1	0.90761	.	.	1
5984	0	1.13	1	0.90761	.	.	1
5985	0	1.13	1	0.90761	.	.	1

Obs	Sub_group	fuptimey	_CENSOR_	SURVIVAL	SDF_LCL	SDF_UCL	STRATUM
5986	0	1.13	1	0.90761	.	.	1
5987	0	1.13	1	0.90761	.	.	1
5988	0	1.13	1	0.90761	.	.	1
5989	0	1.13	1	0.90761	.	.	1
5990	0	1.13	1	0.90761	.	.	1
5991	0	1.13	1	0.90761	.	.	1
5992	0	1.13	1	0.90761	.	.	1
5993	0	1.13	1	0.90761	.	.	1
5994	0	1.13	1	0.90761	.	.	1
5995	0	1.13	1	0.90761	.	.	1
5996	0	1.13	1	0.90761	.	.	1
5997	0	1.13	1	0.90761	.	.	1
5998	0	1.13	1	0.90761	.	.	1
5999	0	1.13	1	0.90761	.	.	1
6000	0	1.13	1	0.90761	.	.	1
6001	0	1.13	1	0.90761	.	.	1
6002	0	1.13	1	0.90761	.	.	1
6003	0	1.13	1	0.90761	.	.	1
6004	0	1.13	1	0.90761	.	.	1
6005	0	1.13	1	0.90761	.	.	1
6006	0	1.14	0	0.90734	0.90461	0.91000	1
6007	0	1.14	1	0.90734	.	.	1
6008	0	1.14	1	0.90734	.	.	1
6009	0	1.14	1	0.90734	.	.	1
6010	0	1.14	1	0.90734	.	.	1
6011	0	1.14	1	0.90734	.	.	1
6012	0	1.14	1	0.90734	.	.	1
6013	0	1.14	1	0.90734	.	.	1
6014	0	1.14	1	0.90734	.	.	1
6015	0	1.14	1	0.90734	.	.	1
6016	0	1.14	1	0.90734	.	.	1
6017	0	1.14	1	0.90734	.	.	1
6018	0	1.14	1	0.90734	.	.	1
6019	0	1.14	1	0.90734	.	.	1
6020	0	1.14	1	0.90734	.	.	1

Obs	Sub_group	fuptimey	_CENSOR_	SURVIVAL	SDF_LCL	SDF_UCL	STRATUM
6021	0	1.14	1	0.90734	.	.	1
6022	0	1.14	1	0.90734	.	.	1
6023	0	1.14	1	0.90734	.	.	1
6024	0	1.14	1	0.90734	.	.	1
6025	0	1.14	1	0.90734	.	.	1
6026	0	1.14	1	0.90734	.	.	1
6027	0	1.14	1	0.90734	.	.	1
6028	0	1.14	1	0.90734	.	.	1
6029	0	1.14	1	0.90734	.	.	1
6030	0	1.14	1	0.90734	.	.	1
6031	0	1.14	1	0.90734	.	.	1
6032	0	1.14	1	0.90734	.	.	1
6033	0	1.14	1	0.90734	.	.	1
6034	0	1.14	1	0.90734	.	.	1
6035	0	1.14	1	0.90734	.	.	1
6036	0	1.14	1	0.90734	.	.	1
6037	0	1.14	1	0.90734	.	.	1
6038	0	1.14	1	0.90734	.	.	1
6039	0	1.14	1	0.90734	.	.	1
6040	0	1.14	1	0.90734	.	.	1
6041	0	1.14	1	0.90734	.	.	1
6042	0	1.14	1	0.90734	.	.	1
6043	0	1.14	1	0.90734	.	.	1
6044	0	1.14	1	0.90734	.	.	1
6045	0	1.14	1	0.90734	.	.	1
6046	0	1.14	1	0.90734	.	.	1
6047	0	1.14	1	0.90734	.	.	1
6048	0	1.14	1	0.90734	.	.	1
6049	0	1.14	1	0.90734	.	.	1
6050	0	1.14	1	0.90734	.	.	1
6051	0	1.14	1	0.90734	.	.	1
6052	0	1.14	1	0.90734	.	.	1
6053	0	1.14	1	0.90734	.	.	1
6054	0	1.14	1	0.90734	.	.	1
6055	0	1.14	1	0.90734	.	.	1

Obs	Sub_group	fuptimey	_CENSOR_	SURVIVAL	SDF_LCL	SDF_UCL	STRATUM
6056	0	1.14	1	0.90734	.	.	1
6057	0	1.14	1	0.90734	.	.	1
6058	0	1.14	1	0.90734	.	.	1
6059	0	1.14	1	0.90734	.	.	1
6060	0	1.14	1	0.90734	.	.	1
6061	0	1.14	1	0.90734	.	.	1
6062	0	1.14	1	0.90734	.	.	1
6063	0	1.14	1	0.90734	.	.	1
6064	0	1.14	1	0.90734	.	.	1
6065	0	1.14	1	0.90734	.	.	1
6066	0	1.14	1	0.90734	.	.	1
6067	0	1.14	1	0.90734	.	.	1
6068	0	1.14	1	0.90734	.	.	1
6069	0	1.14	1	0.90734	.	.	1
6070	0	1.14	1	0.90734	.	.	1
6071	0	1.14	1	0.90734	.	.	1
6072	0	1.14	1	0.90734	.	.	1
6073	0	1.14	1	0.90734	.	.	1
6074	0	1.14	1	0.90734	.	.	1
6075	0	1.14	1	0.90734	.	.	1
6076	0	1.14	1	0.90734	.	.	1
6077	0	1.14	1	0.90734	.	.	1
6078	0	1.14	1	0.90734	.	.	1
6079	0	1.14	1	0.90734	.	.	1
6080	0	1.15	0	0.90707	0.90433	0.90973	1
6081	0	1.15	1	0.90707	.	.	1
6082	0	1.15	1	0.90707	.	.	1
6083	0	1.15	1	0.90707	.	.	1
6084	0	1.15	1	0.90707	.	.	1
6085	0	1.15	1	0.90707	.	.	1
6086	0	1.15	1	0.90707	.	.	1
6087	0	1.15	1	0.90707	.	.	1
6088	0	1.15	1	0.90707	.	.	1
6089	0	1.15	1	0.90707	.	.	1
6090	0	1.15	1	0.90707	.	.	1

Obs	Sub_group	fuptimey	_CENSOR_	SURVIVAL	SDF_LCL	SDF_UCL	STRATUM
**6091**	0	1.15	1	0.90707	.	.	1
**6092**	0	1.15	1	0.90707	.	.	1
**6093**	0	1.15	1	0.90707	.	.	1
**6094**	0	1.15	1	0.90707	.	.	1
**6095**	0	1.15	1	0.90707	.	.	1
**6096**	0	1.15	1	0.90707	.	.	1
**6097**	0	1.15	1	0.90707	.	.	1
**6098**	0	1.15	1	0.90707	.	.	1
**6099**	0	1.15	1	0.90707	.	.	1
**6100**	0	1.15	1	0.90707	.	.	1
**6101**	0	1.15	1	0.90707	.	.	1
**6102**	0	1.15	1	0.90707	.	.	1
**6103**	0	1.15	1	0.90707	.	.	1
**6104**	0	1.15	1	0.90707	.	.	1
**6105**	0	1.15	1	0.90707	.	.	1
**6106**	0	1.15	1	0.90707	.	.	1
**6107**	0	1.15	1	0.90707	.	.	1
**6108**	0	1.15	1	0.90707	.	.	1
**6109**	0	1.15	1	0.90707	.	.	1
**6110**	0	1.15	1	0.90707	.	.	1
**6111**	0	1.15	1	0.90707	.	.	1
**6112**	0	1.15	1	0.90707	.	.	1
**6113**	0	1.15	1	0.90707	.	.	1
**6114**	0	1.15	1	0.90707	.	.	1
**6115**	0	1.15	1	0.90707	.	.	1
**6116**	0	1.15	1	0.90707	.	.	1
**6117**	0	1.15	1	0.90707	.	.	1
**6118**	0	1.15	1	0.90707	.	.	1
**6119**	0	1.15	1	0.90707	.	.	1
**6120**	0	1.15	1	0.90707	.	.	1
**6121**	0	1.15	1	0.90707	.	.	1
**6122**	0	1.15	1	0.90707	.	.	1
**6123**	0	1.15	1	0.90707	.	.	1
**6124**	0	1.15	1	0.90707	.	.	1
**6125**	0	1.15	1	0.90707	.	.	1

Obs	Sub_group	fuptimey	_CENSOR_	SURVIVAL	SDF_LCL	SDF_UCL	STRATUM
6126	0	1.15	1	0.90707	.	.	1
6127	0	1.16	0	0.90670	0.90395	0.90936	1
6128	0	1.16	1	0.90670	.	.	1
6129	0	1.16	1	0.90670	.	.	1
6130	0	1.16	1	0.90670	.	.	1
6131	0	1.16	1	0.90670	.	.	1
6132	0	1.16	1	0.90670	.	.	1
6133	0	1.16	1	0.90670	.	.	1
6134	0	1.16	1	0.90670	.	.	1
6135	0	1.16	1	0.90670	.	.	1
6136	0	1.16	1	0.90670	.	.	1
6137	0	1.16	1	0.90670	.	.	1
6138	0	1.16	1	0.90670	.	.	1
6139	0	1.16	1	0.90670	.	.	1
6140	0	1.16	1	0.90670	.	.	1
6141	0	1.16	1	0.90670	.	.	1
6142	0	1.16	1	0.90670	.	.	1
6143	0	1.16	1	0.90670	.	.	1
6144	0	1.16	1	0.90670	.	.	1
6145	0	1.16	1	0.90670	.	.	1
6146	0	1.16	1	0.90670	.	.	1
6147	0	1.16	1	0.90670	.	.	1
6148	0	1.16	1	0.90670	.	.	1
6149	0	1.16	1	0.90670	.	.	1
6150	0	1.16	1	0.90670	.	.	1
6151	0	1.16	1	0.90670	.	.	1
6152	0	1.16	1	0.90670	.	.	1
6153	0	1.16	1	0.90670	.	.	1
6154	0	1.16	1	0.90670	.	.	1
6155	0	1.16	1	0.90670	.	.	1
6156	0	1.16	1	0.90670	.	.	1
6157	0	1.16	1	0.90670	.	.	1
6158	0	1.16	1	0.90670	.	.	1
6159	0	1.16	1	0.90670	.	.	1
6160	0	1.16	1	0.90670	.	.	1

Obs	Sub_group	fuptimey	_CENSOR_	SURVIVAL	SDF_LCL	SDF_UCL	STRATUM
**6161**	0	1.16	1	0.90670	.	.	1
**6162**	0	1.16	1	0.90670	.	.	1
**6163**	0	1.16	1	0.90670	.	.	1
**6164**	0	1.16	1	0.90670	.	.	1
**6165**	0	1.16	1	0.90670	.	.	1
**6166**	0	1.16	1	0.90670	.	.	1
**6167**	0	1.16	1	0.90670	.	.	1
**6168**	0	1.16	1	0.90670	.	.	1
**6169**	0	1.16	1	0.90670	.	.	1
**6170**	0	1.16	1	0.90670	.	.	1
**6171**	0	1.16	1	0.90670	.	.	1
**6172**	0	1.16	1	0.90670	.	.	1
**6173**	0	1.16	1	0.90670	.	.	1
**6174**	0	1.16	1	0.90670	.	.	1
**6175**	0	1.16	1	0.90670	.	.	1
**6176**	0	1.16	1	0.90670	.	.	1
**6177**	0	1.16	1	0.90670	.	.	1
**6178**	0	1.16	1	0.90670	.	.	1
**6179**	0	1.16	1	0.90670	.	.	1
**6180**	0	1.16	1	0.90670	.	.	1
**6181**	0	1.16	1	0.90670	.	.	1
**6182**	0	1.16	1	0.90670	.	.	1
**6183**	0	1.16	1	0.90670	.	.	1
**6184**	0	1.16	1	0.90670	.	.	1
**6185**	0	1.17	0	0.90635	0.90360	0.90902	1
**6186**	0	1.17	1	0.90635	.	.	1
**6187**	0	1.17	1	0.90635	.	.	1
**6188**	0	1.17	1	0.90635	.	.	1
**6189**	0	1.17	1	0.90635	.	.	1
**6190**	0	1.17	1	0.90635	.	.	1
**6191**	0	1.17	1	0.90635	.	.	1
**6192**	0	1.17	1	0.90635	.	.	1
**6193**	0	1.17	1	0.90635	.	.	1
**6194**	0	1.17	1	0.90635	.	.	1
**6195**	0	1.17	1	0.90635	.	.	1

Obs	Sub_group	fuptimey	_CENSOR_	SURVIVAL	SDF_LCL	SDF_UCL	STRATUM
6196	0	1.17	1	0.90635	.	.	1
6197	0	1.17	1	0.90635	.	.	1
6198	0	1.17	1	0.90635	.	.	1
6199	0	1.17	1	0.90635	.	.	1
6200	0	1.17	1	0.90635	.	.	1
6201	0	1.17	1	0.90635	.	.	1
6202	0	1.17	1	0.90635	.	.	1
6203	0	1.17	1	0.90635	.	.	1
6204	0	1.17	1	0.90635	.	.	1
6205	0	1.17	1	0.90635	.	.	1
6206	0	1.17	1	0.90635	.	.	1
6207	0	1.17	1	0.90635	.	.	1
6208	0	1.17	1	0.90635	.	.	1
6209	0	1.17	1	0.90635	.	.	1
6210	0	1.17	1	0.90635	.	.	1
6211	0	1.17	1	0.90635	.	.	1
6212	0	1.17	1	0.90635	.	.	1
6213	0	1.17	1	0.90635	.	.	1
6214	0	1.17	1	0.90635	.	.	1
6215	0	1.17	1	0.90635	.	.	1
6216	0	1.17	1	0.90635	.	.	1
6217	0	1.17	1	0.90635	.	.	1
6218	0	1.17	1	0.90635	.	.	1
6219	0	1.17	1	0.90635	.	.	1
6220	0	1.17	1	0.90635	.	.	1
6221	0	1.17	1	0.90635	.	.	1
6222	0	1.17	1	0.90635	.	.	1
6223	0	1.17	1	0.90635	.	.	1
6224	0	1.17	1	0.90635	.	.	1
6225	0	1.17	1	0.90635	.	.	1
6226	0	1.17	1	0.90635	.	.	1
6227	0	1.17	1	0.90635	.	.	1
6228	0	1.17	1	0.90635	.	.	1
6229	0	1.17	1	0.90635	.	.	1
6230	0	1.17	1	0.90635	.	.	1

Obs	Sub_group	fuptimey	_CENSOR_	SURVIVAL	SDF_LCL	SDF_UCL	STRATUM
6231	0	1.17	1	0.90635	.	.	1
6232	0	1.17	1	0.90635	.	.	1
6233	0	1.17	1	0.90635	.	.	1
6234	0	1.17	1	0.90635	.	.	1
6235	0	1.17	1	0.90635	.	.	1
6236	0	1.17	1	0.90635	.	.	1
6237	0	1.17	1	0.90635	.	.	1
6238	0	1.17	1	0.90635	.	.	1
6239	0	1.18	0	0.90595	0.90320	0.90863	1
6240	0	1.18	1	0.90595	.	.	1
6241	0	1.18	1	0.90595	.	.	1
6242	0	1.18	1	0.90595	.	.	1
6243	0	1.18	1	0.90595	.	.	1
6244	0	1.18	1	0.90595	.	.	1
6245	0	1.18	1	0.90595	.	.	1
6246	0	1.18	1	0.90595	.	.	1
6247	0	1.18	1	0.90595	.	.	1
6248	0	1.18	1	0.90595	.	.	1
6249	0	1.18	1	0.90595	.	.	1
6250	0	1.18	1	0.90595	.	.	1
6251	0	1.18	1	0.90595	.	.	1
6252	0	1.18	1	0.90595	.	.	1
6253	0	1.18	1	0.90595	.	.	1
6254	0	1.18	1	0.90595	.	.	1
6255	0	1.18	1	0.90595	.	.	1
6256	0	1.18	1	0.90595	.	.	1
6257	0	1.18	1	0.90595	.	.	1
6258	0	1.18	1	0.90595	.	.	1
6259	0	1.18	1	0.90595	.	.	1
6260	0	1.18	1	0.90595	.	.	1
6261	0	1.18	1	0.90595	.	.	1
6262	0	1.18	1	0.90595	.	.	1
6263	0	1.18	1	0.90595	.	.	1
6264	0	1.18	1	0.90595	.	.	1
6265	0	1.18	1	0.90595	.	.	1

Obs	Sub_group	fuptimey	_CENSOR_	SURVIVAL	SDF_LCL	SDF_UCL	STRATUM
6266	0	1.18	1	0.90595	.	.	1
6267	0	1.18	1	0.90595	.	.	1
6268	0	1.18	1	0.90595	.	.	1
6269	0	1.18	1	0.90595	.	.	1
6270	0	1.18	1	0.90595	.	.	1
6271	0	1.18	1	0.90595	.	.	1
6272	0	1.18	1	0.90595	.	.	1
6273	0	1.18	1	0.90595	.	.	1
6274	0	1.18	1	0.90595	.	.	1
6275	0	1.18	1	0.90595	.	.	1
6276	0	1.18	1	0.90595	.	.	1
6277	0	1.18	1	0.90595	.	.	1
6278	0	1.18	1	0.90595	.	.	1
6279	0	1.18	1	0.90595	.	.	1
6280	0	1.18	1	0.90595	.	.	1
6281	0	1.18	1	0.90595	.	.	1
6282	0	1.18	1	0.90595	.	.	1
6283	0	1.18	1	0.90595	.	.	1
6284	0	1.18	1	0.90595	.	.	1
6285	0	1.18	1	0.90595	.	.	1
6286	0	1.18	1	0.90595	.	.	1
6287	0	1.18	1	0.90595	.	.	1
6288	0	1.18	1	0.90595	.	.	1
6289	0	1.18	1	0.90595	.	.	1
6290	0	1.18	1	0.90595	.	.	1
6291	0	1.18	1	0.90595	.	.	1
6292	0	1.18	1	0.90595	.	.	1
6293	0	1.18	1	0.90595	.	.	1
6294	0	1.18	1	0.90595	.	.	1
6295	0	1.18	1	0.90595	.	.	1
6296	0	1.18	1	0.90595	.	.	1
6297	0	1.18	1	0.90595	.	.	1
6298	0	1.18	1	0.90595	.	.	1
6299	0	1.18	1	0.90595	.	.	1
6300	0	1.18	1	0.90595	.	.	1

Obs	Sub_group	fuptimey	_CENSOR_	SURVIVAL	SDF_LCL	SDF_UCL	STRATUM
6301	0	1.18	1	0.90595	.	.	1
6302	0	1.18	1	0.90595	.	.	1
6303	0	1.18	1	0.90595	.	.	1
6304	0	1.18	1	0.90595	.	.	1
6305	0	1.19	0	0.90552	0.90277	0.90821	1
6306	0	1.19	1	0.90552	.	.	1
6307	0	1.19	1	0.90552	.	.	1
6308	0	1.19	1	0.90552	.	.	1
6309	0	1.19	1	0.90552	.	.	1
6310	0	1.19	1	0.90552	.	.	1
6311	0	1.19	1	0.90552	.	.	1
6312	0	1.19	1	0.90552	.	.	1
6313	0	1.19	1	0.90552	.	.	1
6314	0	1.19	1	0.90552	.	.	1
6315	0	1.19	1	0.90552	.	.	1
6316	0	1.19	1	0.90552	.	.	1
6317	0	1.19	1	0.90552	.	.	1
6318	0	1.19	1	0.90552	.	.	1
6319	0	1.19	1	0.90552	.	.	1
6320	0	1.19	1	0.90552	.	.	1
6321	0	1.19	1	0.90552	.	.	1
6322	0	1.19	1	0.90552	.	.	1
6323	0	1.19	1	0.90552	.	.	1
6324	0	1.19	1	0.90552	.	.	1
6325	0	1.19	1	0.90552	.	.	1
6326	0	1.19	1	0.90552	.	.	1
6327	0	1.19	1	0.90552	.	.	1
6328	0	1.19	1	0.90552	.	.	1
6329	0	1.19	1	0.90552	.	.	1
6330	0	1.19	1	0.90552	.	.	1
6331	0	1.19	1	0.90552	.	.	1
6332	0	1.19	1	0.90552	.	.	1
6333	0	1.19	1	0.90552	.	.	1
6334	0	1.19	1	0.90552	.	.	1
6335	0	1.19	1	0.90552	.	.	1

Obs	Sub_group	fuptimey	_CENSOR_	SURVIVAL	SDF_LCL	SDF_UCL	STRATUM
6336	0	1.19	1	0.90552	.	.	1
6337	0	1.19	1	0.90552	.	.	1
6338	0	1.19	1	0.90552	.	.	1
6339	0	1.19	1	0.90552	.	.	1
6340	0	1.19	1	0.90552	.	.	1
6341	0	1.20	0	0.90515	0.90238	0.90784	1
6342	0	1.20	1	0.90515	.	.	1
6343	0	1.20	1	0.90515	.	.	1
6344	0	1.20	1	0.90515	.	.	1
6345	0	1.20	1	0.90515	.	.	1
6346	0	1.20	1	0.90515	.	.	1
6347	0	1.20	1	0.90515	.	.	1
6348	0	1.20	1	0.90515	.	.	1
6349	0	1.20	1	0.90515	.	.	1
6350	0	1.20	1	0.90515	.	.	1
6351	0	1.20	1	0.90515	.	.	1
6352	0	1.20	1	0.90515	.	.	1
6353	0	1.20	1	0.90515	.	.	1
6354	0	1.20	1	0.90515	.	.	1
6355	0	1.20	1	0.90515	.	.	1
6356	0	1.20	1	0.90515	.	.	1
6357	0	1.20	1	0.90515	.	.	1
6358	0	1.20	1	0.90515	.	.	1
6359	0	1.20	1	0.90515	.	.	1
6360	0	1.20	1	0.90515	.	.	1
6361	0	1.20	1	0.90515	.	.	1
6362	0	1.20	1	0.90515	.	.	1
6363	0	1.20	1	0.90515	.	.	1
6364	0	1.20	1	0.90515	.	.	1
6365	0	1.20	1	0.90515	.	.	1
6366	0	1.20	1	0.90515	.	.	1
6367	0	1.20	1	0.90515	.	.	1
6368	0	1.20	1	0.90515	.	.	1
6369	0	1.20	1	0.90515	.	.	1
6370	0	1.20	1	0.90515	.	.	1

Obs	Sub_group	fuptimey	_CENSOR_	SURVIVAL	SDF_LCL	SDF_UCL	STRATUM
6371	0	1.20	1	0.90515	.	.	1
6372	0	1.20	1	0.90515	.	.	1
6373	0	1.20	1	0.90515	.	.	1
6374	0	1.20	1	0.90515	.	.	1
6375	0	1.20	1	0.90515	.	.	1
6376	0	1.20	1	0.90515	.	.	1
6377	0	1.20	1	0.90515	.	.	1
6378	0	1.20	1	0.90515	.	.	1
6379	0	1.20	1	0.90515	.	.	1
6380	0	1.20	1	0.90515	.	.	1
6381	0	1.20	1	0.90515	.	.	1
6382	0	1.20	1	0.90515	.	.	1
6383	0	1.20	1	0.90515	.	.	1
6384	0	1.20	1	0.90515	.	.	1
6385	0	1.20	1	0.90515	.	.	1
6386	0	1.20	1	0.90515	.	.	1
6387	0	1.20	1	0.90515	.	.	1
6388	0	1.21	0	0.90485	0.90208	0.90754	1
6389	0	1.21	1	0.90485	.	.	1
6390	0	1.21	1	0.90485	.	.	1
6391	0	1.21	1	0.90485	.	.	1
6392	0	1.21	1	0.90485	.	.	1
6393	0	1.21	1	0.90485	.	.	1
6394	0	1.21	1	0.90485	.	.	1
6395	0	1.21	1	0.90485	.	.	1
6396	0	1.21	1	0.90485	.	.	1
6397	0	1.21	1	0.90485	.	.	1
6398	0	1.21	1	0.90485	.	.	1
6399	0	1.21	1	0.90485	.	.	1
6400	0	1.21	1	0.90485	.	.	1
6401	0	1.21	1	0.90485	.	.	1
6402	0	1.21	1	0.90485	.	.	1
6403	0	1.21	1	0.90485	.	.	1
6404	0	1.21	1	0.90485	.	.	1
6405	0	1.21	1	0.90485	.	.	1

Obs	Sub_group	fuptimey	_CENSOR_	SURVIVAL	SDF_LCL	SDF_UCL	STRATUM
6406	0	1.21	1	0.90485	.	.	1
6407	0	1.21	1	0.90485	.	.	1
6408	0	1.21	1	0.90485	.	.	1
6409	0	1.21	1	0.90485	.	.	1
6410	0	1.21	1	0.90485	.	.	1
6411	0	1.21	1	0.90485	.	.	1
6412	0	1.21	1	0.90485	.	.	1
6413	0	1.21	1	0.90485	.	.	1
6414	0	1.21	1	0.90485	.	.	1
6415	0	1.21	1	0.90485	.	.	1
6416	0	1.21	1	0.90485	.	.	1
6417	0	1.21	1	0.90485	.	.	1
6418	0	1.21	1	0.90485	.	.	1
6419	0	1.21	1	0.90485	.	.	1
6420	0	1.21	1	0.90485	.	.	1
6421	0	1.21	1	0.90485	.	.	1
6422	0	1.21	1	0.90485	.	.	1
6423	0	1.21	1	0.90485	.	.	1
6424	0	1.21	1	0.90485	.	.	1
6425	0	1.21	1	0.90485	.	.	1
6426	0	1.21	1	0.90485	.	.	1
6427	0	1.21	1	0.90485	.	.	1
6428	0	1.21	1	0.90485	.	.	1
6429	0	1.21	1	0.90485	.	.	1
6430	0	1.21	1	0.90485	.	.	1
6431	0	1.21	1	0.90485	.	.	1
6432	0	1.21	1	0.90485	.	.	1
6433	0	1.21	1	0.90485	.	.	1
6434	0	1.21	1	0.90485	.	.	1
6435	0	1.21	1	0.90485	.	.	1
6436	0	1.21	1	0.90485	.	.	1
6437	0	1.21	1	0.90485	.	.	1
6438	0	1.21	1	0.90485	.	.	1
6439	0	1.21	1	0.90485	.	.	1
6440	0	1.21	1	0.90485	.	.	1

Obs	Sub_group	fuptimey	_CENSOR_	SURVIVAL	SDF_LCL	SDF_UCL	STRATUM
6441	0	1.21	1	0.90485	.	.	1
6442	0	1.21	1	0.90485	.	.	1
6443	0	1.21	1	0.90485	.	.	1
6444	0	1.21	1	0.90485	.	.	1
6445	0	1.21	1	0.90485	.	.	1
6446	0	1.21	1	0.90485	.	.	1
6447	0	1.21	1	0.90485	.	.	1
6448	0	1.21	1	0.90485	.	.	1
6449	0	1.21	1	0.90485	.	.	1
6450	0	1.21	1	0.90485	.	.	1
6451	0	1.21	1	0.90485	.	.	1
6452	0	1.21	1	0.90485	.	.	1
6453	0	1.22	0	0.90455	0.90177	0.90725	1
6454	0	1.22	1	0.90455	.	.	1
6455	0	1.22	1	0.90455	.	.	1
6456	0	1.22	1	0.90455	.	.	1
6457	0	1.22	1	0.90455	.	.	1
6458	0	1.22	1	0.90455	.	.	1
6459	0	1.22	1	0.90455	.	.	1
6460	0	1.22	1	0.90455	.	.	1
6461	0	1.22	1	0.90455	.	.	1
6462	0	1.22	1	0.90455	.	.	1
6463	0	1.22	1	0.90455	.	.	1
6464	0	1.22	1	0.90455	.	.	1
6465	0	1.22	1	0.90455	.	.	1
6466	0	1.22	1	0.90455	.	.	1
6467	0	1.22	1	0.90455	.	.	1
6468	0	1.22	1	0.90455	.	.	1
6469	0	1.22	1	0.90455	.	.	1
6470	0	1.22	1	0.90455	.	.	1
6471	0	1.22	1	0.90455	.	.	1
6472	0	1.22	1	0.90455	.	.	1
6473	0	1.22	1	0.90455	.	.	1
6474	0	1.22	1	0.90455	.	.	1
6475	0	1.22	1	0.90455	.	.	1

Obs	Sub_group	fuptimey	_CENSOR_	SURVIVAL	SDF_LCL	SDF_UCL	STRATUM
6476	0	1.22	1	0.90455	.	.	1
6477	0	1.22	1	0.90455	.	.	1
6478	0	1.22	1	0.90455	.	.	1
6479	0	1.22	1	0.90455	.	.	1
6480	0	1.22	1	0.90455	.	.	1
6481	0	1.22	1	0.90455	.	.	1
6482	0	1.22	1	0.90455	.	.	1
6483	0	1.22	1	0.90455	.	.	1
6484	0	1.22	1	0.90455	.	.	1
6485	0	1.22	1	0.90455	.	.	1
6486	0	1.22	1	0.90455	.	.	1
6487	0	1.22	1	0.90455	.	.	1
6488	0	1.22	1	0.90455	.	.	1
6489	0	1.22	1	0.90455	.	.	1
6490	0	1.22	1	0.90455	.	.	1
6491	0	1.22	1	0.90455	.	.	1
6492	0	1.22	1	0.90455	.	.	1
6493	0	1.22	1	0.90455	.	.	1
6494	0	1.23	0	0.90420	0.90142	0.90690	1
6495	0	1.23	1	0.90420	.	.	1
6496	0	1.23	1	0.90420	.	.	1
6497	0	1.23	1	0.90420	.	.	1
6498	0	1.23	1	0.90420	.	.	1
6499	0	1.23	1	0.90420	.	.	1
6500	0	1.23	1	0.90420	.	.	1
6501	0	1.23	1	0.90420	.	.	1
6502	0	1.23	1	0.90420	.	.	1
6503	0	1.23	1	0.90420	.	.	1
6504	0	1.23	1	0.90420	.	.	1
6505	0	1.23	1	0.90420	.	.	1
6506	0	1.23	1	0.90420	.	.	1
6507	0	1.23	1	0.90420	.	.	1
6508	0	1.23	1	0.90420	.	.	1
6509	0	1.23	1	0.90420	.	.	1
6510	0	1.23	1	0.90420	.	.	1

Obs	Sub_group	fuptimey	_CENSOR_	SURVIVAL	SDF_LCL	SDF_UCL	STRATUM
6511	0	1.23	1	0.90420	.	.	1
6512	0	1.23	1	0.90420	.	.	1
6513	0	1.23	1	0.90420	.	.	1
6514	0	1.23	1	0.90420	.	.	1
6515	0	1.23	1	0.90420	.	.	1
6516	0	1.23	1	0.90420	.	.	1
6517	0	1.23	1	0.90420	.	.	1
6518	0	1.23	1	0.90420	.	.	1
6519	0	1.23	1	0.90420	.	.	1
6520	0	1.23	1	0.90420	.	.	1
6521	0	1.23	1	0.90420	.	.	1
6522	0	1.23	1	0.90420	.	.	1
6523	0	1.23	1	0.90420	.	.	1
6524	0	1.23	1	0.90420	.	.	1
6525	0	1.23	1	0.90420	.	.	1
6526	0	1.23	1	0.90420	.	.	1
6527	0	1.23	1	0.90420	.	.	1
6528	0	1.23	1	0.90420	.	.	1
6529	0	1.23	1	0.90420	.	.	1
6530	0	1.23	1	0.90420	.	.	1
6531	0	1.23	1	0.90420	.	.	1
6532	0	1.23	1	0.90420	.	.	1
6533	0	1.23	1	0.90420	.	.	1
6534	0	1.23	1	0.90420	.	.	1
6535	0	1.23	1	0.90420	.	.	1
6536	0	1.23	1	0.90420	.	.	1
6537	0	1.23	1	0.90420	.	.	1
6538	0	1.23	1	0.90420	.	.	1
6539	0	1.23	1	0.90420	.	.	1
6540	0	1.23	1	0.90420	.	.	1
6541	0	1.23	1	0.90420	.	.	1
6542	0	1.23	1	0.90420	.	.	1
6543	0	1.23	1	0.90420	.	.	1
6544	0	1.23	1	0.90420	.	.	1
6545	0	1.23	1	0.90420	.	.	1

Obs	Sub_group	fuptimey	_CENSOR_	SURVIVAL	SDF_LCL	SDF_UCL	STRATUM
**6546**	0	1.23	1	0.90420	.	.	1
**6547**	0	1.23	1	0.90420	.	.	1
**6548**	0	1.23	1	0.90420	.	.	1
**6549**	0	1.23	1	0.90420	.	.	1
**6550**	0	1.23	1	0.90420	.	.	1
**6551**	0	1.23	1	0.90420	.	.	1
**6552**	0	1.24	0	0.90392	0.90114	0.90663	1
**6553**	0	1.24	1	0.90392	.	.	1
**6554**	0	1.24	1	0.90392	.	.	1
**6555**	0	1.24	1	0.90392	.	.	1
**6556**	0	1.24	1	0.90392	.	.	1
**6557**	0	1.24	1	0.90392	.	.	1
**6558**	0	1.24	1	0.90392	.	.	1
**6559**	0	1.24	1	0.90392	.	.	1
**6560**	0	1.24	1	0.90392	.	.	1
**6561**	0	1.24	1	0.90392	.	.	1
**6562**	0	1.24	1	0.90392	.	.	1
**6563**	0	1.24	1	0.90392	.	.	1
**6564**	0	1.24	1	0.90392	.	.	1
**6565**	0	1.24	1	0.90392	.	.	1
**6566**	0	1.24	1	0.90392	.	.	1
**6567**	0	1.24	1	0.90392	.	.	1
**6568**	0	1.24	1	0.90392	.	.	1
**6569**	0	1.24	1	0.90392	.	.	1
**6570**	0	1.24	1	0.90392	.	.	1
**6571**	0	1.24	1	0.90392	.	.	1
**6572**	0	1.24	1	0.90392	.	.	1
**6573**	0	1.24	1	0.90392	.	.	1
**6574**	0	1.24	1	0.90392	.	.	1
**6575**	0	1.24	1	0.90392	.	.	1
**6576**	0	1.24	1	0.90392	.	.	1
**6577**	0	1.24	1	0.90392	.	.	1
**6578**	0	1.24	1	0.90392	.	.	1
**6579**	0	1.24	1	0.90392	.	.	1
**6580**	0	1.24	1	0.90392	.	.	1

Obs	Sub_group	fuptimey	_CENSOR_	SURVIVAL	SDF_LCL	SDF_UCL	STRATUM
6581	0	1.24	1	0.90392	.	.	1
6582	0	1.24	1	0.90392	.	.	1
6583	0	1.24	1	0.90392	.	.	1
6584	0	1.24	1	0.90392	.	.	1
6585	0	1.24	1	0.90392	.	.	1
6586	0	1.24	1	0.90392	.	.	1
6587	0	1.24	1	0.90392	.	.	1
6588	0	1.24	1	0.90392	.	.	1
6589	0	1.24	1	0.90392	.	.	1
6590	0	1.24	1	0.90392	.	.	1
6591	0	1.24	1	0.90392	.	.	1
6592	0	1.24	1	0.90392	.	.	1
6593	0	1.24	1	0.90392	.	.	1
6594	0	1.24	1	0.90392	.	.	1
6595	0	1.24	1	0.90392	.	.	1
6596	0	1.24	1	0.90392	.	.	1
6597	0	1.24	1	0.90392	.	.	1
6598	0	1.24	1	0.90392	.	.	1
6599	0	1.25	0	0.90347	0.90068	0.90618	1
6600	0	1.25	1	0.90347	.	.	1
6601	0	1.25	1	0.90347	.	.	1
6602	0	1.25	1	0.90347	.	.	1
6603	0	1.25	1	0.90347	.	.	1
6604	0	1.25	1	0.90347	.	.	1
6605	0	1.25	1	0.90347	.	.	1
6606	0	1.25	1	0.90347	.	.	1
6607	0	1.25	1	0.90347	.	.	1
6608	0	1.25	1	0.90347	.	.	1
6609	0	1.25	1	0.90347	.	.	1
6610	0	1.25	1	0.90347	.	.	1
6611	0	1.25	1	0.90347	.	.	1
6612	0	1.25	1	0.90347	.	.	1
6613	0	1.25	1	0.90347	.	.	1
6614	0	1.25	1	0.90347	.	.	1
6615	0	1.25	1	0.90347	.	.	1

Obs	Sub_group	fuptimey	_CENSOR_	SURVIVAL	SDF_LCL	SDF_UCL	STRATUM
6616	0	1.25	1	0.90347	.	.	1
6617	0	1.25	1	0.90347	.	.	1
6618	0	1.25	1	0.90347	.	.	1
6619	0	1.25	1	0.90347	.	.	1
6620	0	1.25	1	0.90347	.	.	1
6621	0	1.25	1	0.90347	.	.	1
6622	0	1.25	1	0.90347	.	.	1
6623	0	1.25	1	0.90347	.	.	1
6624	0	1.25	1	0.90347	.	.	1
6625	0	1.25	1	0.90347	.	.	1
6626	0	1.25	1	0.90347	.	.	1
6627	0	1.25	1	0.90347	.	.	1
6628	0	1.25	1	0.90347	.	.	1
6629	0	1.25	1	0.90347	.	.	1
6630	0	1.25	1	0.90347	.	.	1
6631	0	1.25	1	0.90347	.	.	1
6632	0	1.25	1	0.90347	.	.	1
6633	0	1.25	1	0.90347	.	.	1
6634	0	1.25	1	0.90347	.	.	1
6635	0	1.25	1	0.90347	.	.	1
6636	0	1.25	1	0.90347	.	.	1
6637	0	1.25	1	0.90347	.	.	1
6638	0	1.25	1	0.90347	.	.	1
6639	0	1.25	1	0.90347	.	.	1
6640	0	1.25	1	0.90347	.	.	1
6641	0	1.25	1	0.90347	.	.	1
6642	0	1.25	1	0.90347	.	.	1
6643	0	1.25	1	0.90347	.	.	1
6644	0	1.25	1	0.90347	.	.	1
6645	0	1.25	1	0.90347	.	.	1
6646	0	1.25	1	0.90347	.	.	1
6647	0	1.26	0	0.90319	0.90040	0.90591	1
6648	0	1.26	1	0.90319	.	.	1
6649	0	1.26	1	0.90319	.	.	1
6650	0	1.26	1	0.90319	.	.	1

Obs	Sub_group	fuptimey	_CENSOR_	SURVIVAL	SDF_LCL	SDF_UCL	STRATUM
6651	0	1.26	1	0.90319	.	.	1
6652	0	1.26	1	0.90319	.	.	1
6653	0	1.26	1	0.90319	.	.	1
6654	0	1.26	1	0.90319	.	.	1
6655	0	1.26	1	0.90319	.	.	1
6656	0	1.26	1	0.90319	.	.	1
6657	0	1.26	1	0.90319	.	.	1
6658	0	1.26	1	0.90319	.	.	1
6659	0	1.26	1	0.90319	.	.	1
6660	0	1.26	1	0.90319	.	.	1
6661	0	1.26	1	0.90319	.	.	1
6662	0	1.26	1	0.90319	.	.	1
6663	0	1.26	1	0.90319	.	.	1
6664	0	1.26	1	0.90319	.	.	1
6665	0	1.26	1	0.90319	.	.	1
6666	0	1.26	1	0.90319	.	.	1
6667	0	1.26	1	0.90319	.	.	1
6668	0	1.26	1	0.90319	.	.	1
6669	0	1.26	1	0.90319	.	.	1
6670	0	1.26	1	0.90319	.	.	1
6671	0	1.26	1	0.90319	.	.	1
6672	0	1.26	1	0.90319	.	.	1
6673	0	1.26	1	0.90319	.	.	1
6674	0	1.26	1	0.90319	.	.	1
6675	0	1.26	1	0.90319	.	.	1
6676	0	1.26	1	0.90319	.	.	1
6677	0	1.26	1	0.90319	.	.	1
6678	0	1.26	1	0.90319	.	.	1
6679	0	1.26	1	0.90319	.	.	1
6680	0	1.26	1	0.90319	.	.	1
6681	0	1.26	1	0.90319	.	.	1
6682	0	1.26	1	0.90319	.	.	1
6683	0	1.26	1	0.90319	.	.	1
6684	0	1.26	1	0.90319	.	.	1
6685	0	1.26	1	0.90319	.	.	1

Obs	Sub_group	fuptimey	_CENSOR_	SURVIVAL	SDF_LCL	SDF_UCL	STRATUM
6686	0	1.26	1	0.90319	.	.	1
6687	0	1.26	1	0.90319	.	.	1
6688	0	1.26	1	0.90319	.	.	1
6689	0	1.26	1	0.90319	.	.	1
6690	0	1.26	1	0.90319	.	.	1
6691	0	1.26	1	0.90319	.	.	1
6692	0	1.26	1	0.90319	.	.	1
6693	0	1.26	1	0.90319	.	.	1
6694	0	1.27	0	0.90274	0.89994	0.90546	1
6695	0	1.27	1	0.90274	.	.	1
6696	0	1.27	1	0.90274	.	.	1
6697	0	1.27	1	0.90274	.	.	1
6698	0	1.27	1	0.90274	.	.	1
6699	0	1.27	1	0.90274	.	.	1
6700	0	1.27	1	0.90274	.	.	1
6701	0	1.27	1	0.90274	.	.	1
6702	0	1.27	1	0.90274	.	.	1
6703	0	1.27	1	0.90274	.	.	1
6704	0	1.27	1	0.90274	.	.	1
6705	0	1.27	1	0.90274	.	.	1
6706	0	1.27	1	0.90274	.	.	1
6707	0	1.27	1	0.90274	.	.	1
6708	0	1.27	1	0.90274	.	.	1
6709	0	1.27	1	0.90274	.	.	1
6710	0	1.27	1	0.90274	.	.	1
6711	0	1.27	1	0.90274	.	.	1
6712	0	1.27	1	0.90274	.	.	1
6713	0	1.27	1	0.90274	.	.	1
6714	0	1.27	1	0.90274	.	.	1
6715	0	1.27	1	0.90274	.	.	1
6716	0	1.27	1	0.90274	.	.	1
6717	0	1.27	1	0.90274	.	.	1
6718	0	1.27	1	0.90274	.	.	1
6719	0	1.27	1	0.90274	.	.	1
6720	0	1.27	1	0.90274	.	.	1

Obs	Sub_group	fuptimey	_CENSOR_	SURVIVAL	SDF_LCL	SDF_UCL	STRATUM
6721	0	1.27	1	0.90274	.	.	1
6722	0	1.27	1	0.90274	.	.	1
6723	0	1.27	1	0.90274	.	.	1
6724	0	1.27	1	0.90274	.	.	1
6725	0	1.27	1	0.90274	.	.	1
6726	0	1.27	1	0.90274	.	.	1
6727	0	1.27	1	0.90274	.	.	1
6728	0	1.27	1	0.90274	.	.	1
6729	0	1.27	1	0.90274	.	.	1
6730	0	1.27	1	0.90274	.	.	1
6731	0	1.27	1	0.90274	.	.	1
6732	0	1.27	1	0.90274	.	.	1
6733	0	1.27	1	0.90274	.	.	1
6734	0	1.27	1	0.90274	.	.	1
6735	0	1.27	1	0.90274	.	.	1
6736	0	1.27	1	0.90274	.	.	1
6737	0	1.27	1	0.90274	.	.	1
6738	0	1.27	1	0.90274	.	.	1
6739	0	1.27	1	0.90274	.	.	1
6740	0	1.27	1	0.90274	.	.	1
6741	0	1.27	1	0.90274	.	.	1
6742	0	1.27	1	0.90274	.	.	1
6743	0	1.27	1	0.90274	.	.	1
6744	0	1.27	1	0.90274	.	.	1
6745	0	1.27	1	0.90274	.	.	1
6746	0	1.27	1	0.90274	.	.	1
6747	0	1.27	1	0.90274	.	.	1
6748	0	1.27	1	0.90274	.	.	1
6749	0	1.27	1	0.90274	.	.	1
6750	0	1.27	1	0.90274	.	.	1
6751	0	1.27	1	0.90274	.	.	1
6752	0	1.27	1	0.90274	.	.	1
6753	0	1.27	1	0.90274	.	.	1
6754	0	1.27	1	0.90274	.	.	1
6755	0	1.27	1	0.90274	.	.	1

Obs	Sub_group	fuptimey	_CENSOR_	SURVIVAL	SDF_LCL	SDF_UCL	STRATUM
6756	0	1.27	1	0.90274	.	.	1
6757	0	1.27	1	0.90274	.	.	1
6758	0	1.27	1	0.90274	.	.	1
6759	0	1.27	1	0.90274	.	.	1
6760	0	1.27	1	0.90274	.	.	1
6761	0	1.28	0	0.90258	0.89978	0.90531	1
6762	0	1.28	1	0.90258	.	.	1
6763	0	1.28	1	0.90258	.	.	1
6764	0	1.28	1	0.90258	.	.	1
6765	0	1.28	1	0.90258	.	.	1
6766	0	1.28	1	0.90258	.	.	1
6767	0	1.28	1	0.90258	.	.	1
6768	0	1.28	1	0.90258	.	.	1
6769	0	1.28	1	0.90258	.	.	1
6770	0	1.28	1	0.90258	.	.	1
6771	0	1.28	1	0.90258	.	.	1
6772	0	1.28	1	0.90258	.	.	1
6773	0	1.28	1	0.90258	.	.	1
6774	0	1.28	1	0.90258	.	.	1
6775	0	1.28	1	0.90258	.	.	1
6776	0	1.28	1	0.90258	.	.	1
6777	0	1.28	1	0.90258	.	.	1
6778	0	1.28	1	0.90258	.	.	1
6779	0	1.28	1	0.90258	.	.	1
6780	0	1.28	1	0.90258	.	.	1
6781	0	1.28	1	0.90258	.	.	1
6782	0	1.28	1	0.90258	.	.	1
6783	0	1.28	1	0.90258	.	.	1
6784	0	1.28	1	0.90258	.	.	1
6785	0	1.28	1	0.90258	.	.	1
6786	0	1.28	1	0.90258	.	.	1
6787	0	1.28	1	0.90258	.	.	1
6788	0	1.28	1	0.90258	.	.	1
6789	0	1.28	1	0.90258	.	.	1
6790	0	1.28	1	0.90258	.	.	1

Obs	Sub_group	fuptimey	_CENSOR_	SURVIVAL	SDF_LCL	SDF_UCL	STRATUM
6791	0	1.28	1	0.90258	.	.	1
6792	0	1.28	1	0.90258	.	.	1
6793	0	1.28	1	0.90258	.	.	1
6794	0	1.28	1	0.90258	.	.	1
6795	0	1.28	1	0.90258	.	.	1
6796	0	1.28	1	0.90258	.	.	1
6797	0	1.28	1	0.90258	.	.	1
6798	0	1.28	1	0.90258	.	.	1
6799	0	1.28	1	0.90258	.	.	1
6800	0	1.28	1	0.90258	.	.	1
6801	0	1.28	1	0.90258	.	.	1
6802	0	1.28	1	0.90258	.	.	1
6803	0	1.28	1	0.90258	.	.	1
6804	0	1.28	1	0.90258	.	.	1
6805	0	1.28	1	0.90258	.	.	1
6806	0	1.28	1	0.90258	.	.	1
6807	0	1.28	1	0.90258	.	.	1
6808	0	1.28	1	0.90258	.	.	1
6809	0	1.28	1	0.90258	.	.	1
6810	0	1.28	1	0.90258	.	.	1
6811	0	1.28	1	0.90258	.	.	1
6812	0	1.28	1	0.90258	.	.	1
6813	0	1.28	1	0.90258	.	.	1
6814	0	1.28	1	0.90258	.	.	1
6815	0	1.28	1	0.90258	.	.	1
6816	0	1.29	0	0.90233	0.89952	0.90506	1
6817	0	1.29	1	0.90233	.	.	1
6818	0	1.29	1	0.90233	.	.	1
6819	0	1.29	1	0.90233	.	.	1
6820	0	1.29	1	0.90233	.	.	1
6821	0	1.29	1	0.90233	.	.	1
6822	0	1.29	1	0.90233	.	.	1
6823	0	1.29	1	0.90233	.	.	1
6824	0	1.29	1	0.90233	.	.	1
6825	0	1.29	1	0.90233	.	.	1

Obs	Sub_group	fuptimey	_CENSOR_	SURVIVAL	SDF_LCL	SDF_UCL	STRATUM
6826	0	1.29	1	0.90233	.	.	1
6827	0	1.29	1	0.90233	.	.	1
6828	0	1.29	1	0.90233	.	.	1
6829	0	1.29	1	0.90233	.	.	1
6830	0	1.29	1	0.90233	.	.	1
6831	0	1.29	1	0.90233	.	.	1
6832	0	1.29	1	0.90233	.	.	1
6833	0	1.29	1	0.90233	.	.	1
6834	0	1.29	1	0.90233	.	.	1
6835	0	1.29	1	0.90233	.	.	1
6836	0	1.29	1	0.90233	.	.	1
6837	0	1.29	1	0.90233	.	.	1
6838	0	1.29	1	0.90233	.	.	1
6839	0	1.29	1	0.90233	.	.	1
6840	0	1.29	1	0.90233	.	.	1
6841	0	1.29	1	0.90233	.	.	1
6842	0	1.29	1	0.90233	.	.	1
6843	0	1.29	1	0.90233	.	.	1
6844	0	1.29	1	0.90233	.	.	1
6845	0	1.29	1	0.90233	.	.	1
6846	0	1.29	1	0.90233	.	.	1
6847	0	1.29	1	0.90233	.	.	1
6848	0	1.29	1	0.90233	.	.	1
6849	0	1.29	1	0.90233	.	.	1
6850	0	1.29	1	0.90233	.	.	1
6851	0	1.29	1	0.90233	.	.	1
6852	0	1.29	1	0.90233	.	.	1
6853	0	1.29	1	0.90233	.	.	1
6854	0	1.29	1	0.90233	.	.	1
6855	0	1.29	1	0.90233	.	.	1
6856	0	1.29	1	0.90233	.	.	1
6857	0	1.29	1	0.90233	.	.	1
6858	0	1.29	1	0.90233	.	.	1
6859	0	1.29	1	0.90233	.	.	1
6860	0	1.29	1	0.90233	.	.	1

Obs	Sub_group	fuptimey	_CENSOR_	SURVIVAL	SDF_LCL	SDF_UCL	STRATUM
**6861**	0	1.29	1	0.90233	.	.	1
**6862**	0	1.29	1	0.90233	.	.	1
**6863**	0	1.29	1	0.90233	.	.	1
**6864**	0	1.29	1	0.90233	.	.	1
**6865**	0	1.29	1	0.90233	.	.	1
**6866**	0	1.29	1	0.90233	.	.	1
**6867**	0	1.29	1	0.90233	.	.	1
**6868**	0	1.29	1	0.90233	.	.	1
**6869**	0	1.29	1	0.90233	.	.	1
**6870**	0	1.29	1	0.90233	.	.	1
**6871**	0	1.29	1	0.90233	.	.	1
**6872**	0	1.29	1	0.90233	.	.	1
**6873**	0	1.29	1	0.90233	.	.	1
**6874**	0	1.29	1	0.90233	.	.	1
**6875**	0	1.29	1	0.90233	.	.	1
**6876**	0	1.29	1	0.90233	.	.	1
**6877**	0	1.29	1	0.90233	.	.	1
**6878**	0	1.29	1	0.90233	.	.	1
**6879**	0	1.29	1	0.90233	.	.	1
**6880**	0	1.29	1	0.90233	.	.	1
**6881**	0	1.29	1	0.90233	.	.	1
**6882**	0	1.29	1	0.90233	.	.	1
**6883**	0	1.29	1	0.90233	.	.	1
**6884**	0	1.29	1	0.90233	.	.	1
**6885**	0	1.29	1	0.90233	.	.	1
**6886**	0	1.29	1	0.90233	.	.	1
**6887**	0	1.29	1	0.90233	.	.	1
**6888**	0	1.29	1	0.90233	.	.	1
**6889**	0	1.29	1	0.90233	.	.	1
**6890**	0	1.30	0	0.90187	0.89906	0.90461	1
**6891**	0	1.30	1	0.90187	.	.	1
**6892**	0	1.30	1	0.90187	.	.	1
**6893**	0	1.30	1	0.90187	.	.	1
**6894**	0	1.30	1	0.90187	.	.	1
**6895**	0	1.30	1	0.90187	.	.	1

Obs	Sub_group	fuptimey	_CENSOR_	SURVIVAL	SDF_LCL	SDF_UCL	STRATUM
6896	0	1.30	1	0.90187	.	.	1
6897	0	1.30	1	0.90187	.	.	1
6898	0	1.30	1	0.90187	.	.	1
6899	0	1.30	1	0.90187	.	.	1
6900	0	1.30	1	0.90187	.	.	1
6901	0	1.30	1	0.90187	.	.	1
6902	0	1.30	1	0.90187	.	.	1
6903	0	1.30	1	0.90187	.	.	1
6904	0	1.30	1	0.90187	.	.	1
6905	0	1.30	1	0.90187	.	.	1
6906	0	1.30	1	0.90187	.	.	1
6907	0	1.30	1	0.90187	.	.	1
6908	0	1.30	1	0.90187	.	.	1
6909	0	1.30	1	0.90187	.	.	1
6910	0	1.30	1	0.90187	.	.	1
6911	0	1.30	1	0.90187	.	.	1
6912	0	1.30	1	0.90187	.	.	1
6913	0	1.30	1	0.90187	.	.	1
6914	0	1.30	1	0.90187	.	.	1
6915	0	1.30	1	0.90187	.	.	1
6916	0	1.30	1	0.90187	.	.	1
6917	0	1.30	1	0.90187	.	.	1
6918	0	1.30	1	0.90187	.	.	1
6919	0	1.30	1	0.90187	.	.	1
6920	0	1.30	1	0.90187	.	.	1
6921	0	1.30	1	0.90187	.	.	1
6922	0	1.30	1	0.90187	.	.	1
6923	0	1.30	1	0.90187	.	.	1
6924	0	1.30	1	0.90187	.	.	1
6925	0	1.30	1	0.90187	.	.	1
6926	0	1.30	1	0.90187	.	.	1
6927	0	1.30	1	0.90187	.	.	1
6928	0	1.30	1	0.90187	.	.	1
6929	0	1.30	1	0.90187	.	.	1
6930	0	1.30	1	0.90187	.	.	1

Obs	Sub_group	fuptimey	_CENSOR_	SURVIVAL	SDF_LCL	SDF_UCL	STRATUM
6931	0	1.30	1	0.90187	.	.	1
6932	0	1.30	1	0.90187	.	.	1
6933	0	1.30	1	0.90187	.	.	1
6934	0	1.30	1	0.90187	.	.	1
6935	0	1.30	1	0.90187	.	.	1
6936	0	1.30	1	0.90187	.	.	1
6937	0	1.30	1	0.90187	.	.	1
6938	0	1.30	1	0.90187	.	.	1
6939	0	1.30	1	0.90187	.	.	1
6940	0	1.31	0	0.90152	0.89870	0.90426	1
6941	0	1.31	1	0.90152	.	.	1
6942	0	1.31	1	0.90152	.	.	1
6943	0	1.31	1	0.90152	.	.	1
6944	0	1.31	1	0.90152	.	.	1
6945	0	1.31	1	0.90152	.	.	1
6946	0	1.31	1	0.90152	.	.	1
6947	0	1.31	1	0.90152	.	.	1
6948	0	1.31	1	0.90152	.	.	1
6949	0	1.31	1	0.90152	.	.	1
6950	0	1.31	1	0.90152	.	.	1
6951	0	1.31	1	0.90152	.	.	1
6952	0	1.31	1	0.90152	.	.	1
6953	0	1.31	1	0.90152	.	.	1
6954	0	1.31	1	0.90152	.	.	1
6955	0	1.31	1	0.90152	.	.	1
6956	0	1.31	1	0.90152	.	.	1
6957	0	1.31	1	0.90152	.	.	1
6958	0	1.31	1	0.90152	.	.	1
6959	0	1.31	1	0.90152	.	.	1
6960	0	1.31	1	0.90152	.	.	1
6961	0	1.31	1	0.90152	.	.	1
6962	0	1.31	1	0.90152	.	.	1
6963	0	1.31	1	0.90152	.	.	1
6964	0	1.31	1	0.90152	.	.	1
6965	0	1.31	1	0.90152	.	.	1

Obs	Sub_group	fuptimey	_CENSOR_	SURVIVAL	SDF_LCL	SDF_UCL	STRATUM
6966	0	1.31	1	0.90152	.	.	1
6967	0	1.31	1	0.90152	.	.	1
6968	0	1.31	1	0.90152	.	.	1
6969	0	1.31	1	0.90152	.	.	1
6970	0	1.31	1	0.90152	.	.	1
6971	0	1.31	1	0.90152	.	.	1
6972	0	1.31	1	0.90152	.	.	1
6973	0	1.31	1	0.90152	.	.	1
6974	0	1.31	1	0.90152	.	.	1
6975	0	1.31	1	0.90152	.	.	1
6976	0	1.31	1	0.90152	.	.	1
6977	0	1.31	1	0.90152	.	.	1
6978	0	1.31	1	0.90152	.	.	1
6979	0	1.31	1	0.90152	.	.	1
6980	0	1.31	1	0.90152	.	.	1
6981	0	1.31	1	0.90152	.	.	1
6982	0	1.31	1	0.90152	.	.	1
6983	0	1.31	1	0.90152	.	.	1
6984	0	1.31	1	0.90152	.	.	1
6985	0	1.31	1	0.90152	.	.	1
6986	0	1.31	1	0.90152	.	.	1
6987	0	1.31	1	0.90152	.	.	1
6988	0	1.31	1	0.90152	.	.	1
6989	0	1.31	1	0.90152	.	.	1
6990	0	1.31	1	0.90152	.	.	1
6991	0	1.31	1	0.90152	.	.	1
6992	0	1.31	1	0.90152	.	.	1
6993	0	1.32	0	0.90129	0.89847	0.90403	1
6994	0	1.32	1	0.90129	.	.	1
6995	0	1.32	1	0.90129	.	.	1
6996	0	1.32	1	0.90129	.	.	1
6997	0	1.32	1	0.90129	.	.	1
6998	0	1.32	1	0.90129	.	.	1
6999	0	1.32	1	0.90129	.	.	1
7000	0	1.32	1	0.90129	.	.	1

Obs	Sub_group	fuptimey	_CENSOR_	SURVIVAL	SDF_LCL	SDF_UCL	STRATUM
7001	0	1.32	1	0.90129	.	.	1
7002	0	1.32	1	0.90129	.	.	1
7003	0	1.32	1	0.90129	.	.	1
7004	0	1.32	1	0.90129	.	.	1
7005	0	1.32	1	0.90129	.	.	1
7006	0	1.32	1	0.90129	.	.	1
7007	0	1.32	1	0.90129	.	.	1
7008	0	1.32	1	0.90129	.	.	1
7009	0	1.32	1	0.90129	.	.	1
7010	0	1.32	1	0.90129	.	.	1
7011	0	1.32	1	0.90129	.	.	1
7012	0	1.32	1	0.90129	.	.	1
7013	0	1.32	1	0.90129	.	.	1
7014	0	1.32	1	0.90129	.	.	1
7015	0	1.32	1	0.90129	.	.	1
7016	0	1.32	1	0.90129	.	.	1
7017	0	1.32	1	0.90129	.	.	1
7018	0	1.32	1	0.90129	.	.	1
7019	0	1.32	1	0.90129	.	.	1
7020	0	1.32	1	0.90129	.	.	1
7021	0	1.32	1	0.90129	.	.	1
7022	0	1.32	1	0.90129	.	.	1
7023	0	1.32	1	0.90129	.	.	1
7024	0	1.32	1	0.90129	.	.	1
7025	0	1.32	1	0.90129	.	.	1
7026	0	1.32	1	0.90129	.	.	1
7027	0	1.32	1	0.90129	.	.	1
7028	0	1.32	1	0.90129	.	.	1
7029	0	1.32	1	0.90129	.	.	1
7030	0	1.32	1	0.90129	.	.	1
7031	0	1.32	1	0.90129	.	.	1
7032	0	1.32	1	0.90129	.	.	1
7033	0	1.32	1	0.90129	.	.	1
7034	0	1.32	1	0.90129	.	.	1
7035	0	1.32	1	0.90129	.	.	1

Obs	Sub_group	fuptimey	_CENSOR_	SURVIVAL	SDF_LCL	SDF_UCL	STRATUM
**7036**	0	1.32	1	0.90129	.	.	1
**7037**	0	1.32	1	0.90129	.	.	1
**7038**	0	1.33	0	0.90096	0.89813	0.90371	1
**7039**	0	1.33	1	0.90096	.	.	1
**7040**	0	1.33	1	0.90096	.	.	1
**7041**	0	1.33	1	0.90096	.	.	1
**7042**	0	1.33	1	0.90096	.	.	1
**7043**	0	1.33	1	0.90096	.	.	1
**7044**	0	1.33	1	0.90096	.	.	1
**7045**	0	1.33	1	0.90096	.	.	1
**7046**	0	1.33	1	0.90096	.	.	1
**7047**	0	1.33	1	0.90096	.	.	1
**7048**	0	1.33	1	0.90096	.	.	1
**7049**	0	1.33	1	0.90096	.	.	1
**7050**	0	1.33	1	0.90096	.	.	1
**7051**	0	1.33	1	0.90096	.	.	1
**7052**	0	1.33	1	0.90096	.	.	1
**7053**	0	1.33	1	0.90096	.	.	1
**7054**	0	1.33	1	0.90096	.	.	1
**7055**	0	1.33	1	0.90096	.	.	1
**7056**	0	1.33	1	0.90096	.	.	1
**7057**	0	1.33	1	0.90096	.	.	1
**7058**	0	1.33	1	0.90096	.	.	1
**7059**	0	1.33	1	0.90096	.	.	1
**7060**	0	1.33	1	0.90096	.	.	1
**7061**	0	1.33	1	0.90096	.	.	1
**7062**	0	1.33	1	0.90096	.	.	1
**7063**	0	1.33	1	0.90096	.	.	1
**7064**	0	1.33	1	0.90096	.	.	1
**7065**	0	1.33	1	0.90096	.	.	1
**7066**	0	1.33	1	0.90096	.	.	1
**7067**	0	1.33	1	0.90096	.	.	1
**7068**	0	1.33	1	0.90096	.	.	1
**7069**	0	1.33	1	0.90096	.	.	1
**7070**	0	1.33	1	0.90096	.	.	1

Obs	Sub_group	fuptimey	_CENSOR_	SURVIVAL	SDF_LCL	SDF_UCL	STRATUM
7071	0	1.33	1	0.90096	.	.	1
7072	0	1.33	1	0.90096	.	.	1
7073	0	1.33	1	0.90096	.	.	1
7074	0	1.33	1	0.90096	.	.	1
7075	0	1.33	1	0.90096	.	.	1
7076	0	1.33	1	0.90096	.	.	1
7077	0	1.33	1	0.90096	.	.	1
7078	0	1.33	1	0.90096	.	.	1
7079	0	1.33	1	0.90096	.	.	1
7080	0	1.33	1	0.90096	.	.	1
7081	0	1.33	1	0.90096	.	.	1
7082	0	1.33	1	0.90096	.	.	1
7083	0	1.33	1	0.90096	.	.	1
7084	0	1.33	1	0.90096	.	.	1
7085	0	1.33	1	0.90096	.	.	1
7086	0	1.33	1	0.90096	.	.	1
7087	0	1.33	1	0.90096	.	.	1
7088	0	1.33	1	0.90096	.	.	1
7089	0	1.33	1	0.90096	.	.	1
7090	0	1.33	1	0.90096	.	.	1
7091	0	1.33	1	0.90096	.	.	1
7092	0	1.33	1	0.90096	.	.	1
7093	0	1.33	1	0.90096	.	.	1
7094	0	1.33	1	0.90096	.	.	1
7095	0	1.33	1	0.90096	.	.	1
7096	0	1.33	1	0.90096	.	.	1
7097	0	1.33	1	0.90096	.	.	1
7098	0	1.33	1	0.90096	.	.	1
7099	0	1.33	1	0.90096	.	.	1
7100	0	1.33	1	0.90096	.	.	1
7101	0	1.33	1	0.90096	.	.	1
7102	0	1.33	1	0.90096	.	.	1
7103	0	1.33	1	0.90096	.	.	1
7104	0	1.33	1	0.90096	.	.	1
7105	0	1.33	1	0.90096	.	.	1

Obs	Sub_group	fuptimey	_CENSOR_	SURVIVAL	SDF_LCL	SDF_UCL	STRATUM
**7106**	0	1.34	0	0.90063	0.89780	0.90338	1
**7107**	0	1.34	1	0.90063	.	.	1
**7108**	0	1.34	1	0.90063	.	.	1
**7109**	0	1.34	1	0.90063	.	.	1
**7110**	0	1.34	1	0.90063	.	.	1
**7111**	0	1.34	1	0.90063	.	.	1
**7112**	0	1.34	1	0.90063	.	.	1
**7113**	0	1.34	1	0.90063	.	.	1
**7114**	0	1.34	1	0.90063	.	.	1
**7115**	0	1.34	1	0.90063	.	.	1
**7116**	0	1.34	1	0.90063	.	.	1
**7117**	0	1.34	1	0.90063	.	.	1
**7118**	0	1.34	1	0.90063	.	.	1
**7119**	0	1.34	1	0.90063	.	.	1
**7120**	0	1.34	1	0.90063	.	.	1
**7121**	0	1.34	1	0.90063	.	.	1
**7122**	0	1.34	1	0.90063	.	.	1
**7123**	0	1.34	1	0.90063	.	.	1
**7124**	0	1.34	1	0.90063	.	.	1
**7125**	0	1.34	1	0.90063	.	.	1
**7126**	0	1.34	1	0.90063	.	.	1
**7127**	0	1.34	1	0.90063	.	.	1
**7128**	0	1.34	1	0.90063	.	.	1
**7129**	0	1.34	1	0.90063	.	.	1
**7130**	0	1.34	1	0.90063	.	.	1
**7131**	0	1.34	1	0.90063	.	.	1
**7132**	0	1.34	1	0.90063	.	.	1
**7133**	0	1.34	1	0.90063	.	.	1
**7134**	0	1.34	1	0.90063	.	.	1
**7135**	0	1.34	1	0.90063	.	.	1
**7136**	0	1.34	1	0.90063	.	.	1
**7137**	0	1.34	1	0.90063	.	.	1
**7138**	0	1.34	1	0.90063	.	.	1
**7139**	0	1.34	1	0.90063	.	.	1
**7140**	0	1.34	1	0.90063	.	.	1

Obs	Sub_group	fuptimey	_CENSOR_	SURVIVAL	SDF_LCL	SDF_UCL	STRATUM
7141	0	1.34	1	0.90063	.	.	1
7142	0	1.34	1	0.90063	.	.	1
7143	0	1.34	1	0.90063	.	.	1
7144	0	1.34	1	0.90063	.	.	1
7145	0	1.34	1	0.90063	.	.	1
7146	0	1.34	1	0.90063	.	.	1
7147	0	1.34	1	0.90063	.	.	1
7148	0	1.34	1	0.90063	.	.	1
7149	0	1.34	1	0.90063	.	.	1
7150	0	1.35	0	0.90014	0.89730	0.90290	1
7151	0	1.35	1	0.90014	.	.	1
7152	0	1.35	1	0.90014	.	.	1
7153	0	1.35	1	0.90014	.	.	1
7154	0	1.35	1	0.90014	.	.	1
7155	0	1.35	1	0.90014	.	.	1
7156	0	1.35	1	0.90014	.	.	1
7157	0	1.35	1	0.90014	.	.	1
7158	0	1.35	1	0.90014	.	.	1
7159	0	1.35	1	0.90014	.	.	1
7160	0	1.35	1	0.90014	.	.	1
7161	0	1.35	1	0.90014	.	.	1
7162	0	1.35	1	0.90014	.	.	1
7163	0	1.35	1	0.90014	.	.	1
7164	0	1.35	1	0.90014	.	.	1
7165	0	1.35	1	0.90014	.	.	1
7166	0	1.35	1	0.90014	.	.	1
7167	0	1.35	1	0.90014	.	.	1
7168	0	1.35	1	0.90014	.	.	1
7169	0	1.35	1	0.90014	.	.	1
7170	0	1.35	1	0.90014	.	.	1
7171	0	1.35	1	0.90014	.	.	1
7172	0	1.35	1	0.90014	.	.	1
7173	0	1.35	1	0.90014	.	.	1
7174	0	1.35	1	0.90014	.	.	1
7175	0	1.35	1	0.90014	.	.	1

Obs	Sub_group	fuptimey	_CENSOR_	SURVIVAL	SDF_LCL	SDF_UCL	STRATUM
7176	0	1.35	1	0.90014	.	.	1
7177	0	1.35	1	0.90014	.	.	1
7178	0	1.35	1	0.90014	.	.	1
7179	0	1.35	1	0.90014	.	.	1
7180	0	1.35	1	0.90014	.	.	1
7181	0	1.35	1	0.90014	.	.	1
7182	0	1.35	1	0.90014	.	.	1
7183	0	1.35	1	0.90014	.	.	1
7184	0	1.35	1	0.90014	.	.	1
7185	0	1.35	1	0.90014	.	.	1
7186	0	1.35	1	0.90014	.	.	1
7187	0	1.35	1	0.90014	.	.	1
7188	0	1.35	1	0.90014	.	.	1
7189	0	1.35	1	0.90014	.	.	1
7190	0	1.35	1	0.90014	.	.	1
7191	0	1.35	1	0.90014	.	.	1
7192	0	1.35	1	0.90014	.	.	1
7193	0	1.35	1	0.90014	.	.	1
7194	0	1.35	1	0.90014	.	.	1
7195	0	1.35	1	0.90014	.	.	1
7196	0	1.35	1	0.90014	.	.	1
7197	0	1.35	1	0.90014	.	.	1
7198	0	1.35	1	0.90014	.	.	1
7199	0	1.35	1	0.90014	.	.	1
7200	0	1.35	1	0.90014	.	.	1
7201	0	1.35	1	0.90014	.	.	1
7202	0	1.35	1	0.90014	.	.	1
7203	0	1.35	1	0.90014	.	.	1
7204	0	1.35	1	0.90014	.	.	1
7205	0	1.35	1	0.90014	.	.	1
7206	0	1.35	1	0.90014	.	.	1
7207	0	1.35	1	0.90014	.	.	1
7208	0	1.35	1	0.90014	.	.	1
7209	0	1.35	1	0.90014	.	.	1
7210	0	1.35	1	0.90014	.	.	1

Obs	Sub_group	fuptimey	_CENSOR_	SURVIVAL	SDF_LCL	SDF_UCL	STRATUM
7211	0	1.35	1	0.90014	.	.	1
7212	0	1.35	1	0.90014	.	.	1
7213	0	1.35	1	0.90014	.	.	1
7214	0	1.35	1	0.90014	.	.	1
7215	0	1.35	1	0.90014	.	.	1
7216	0	1.35	1	0.90014	.	.	1
7217	0	1.35	1	0.90014	.	.	1
7218	0	1.35	1	0.90014	.	.	1
7219	0	1.35	1	0.90014	.	.	1
7220	0	1.35	1	0.90014	.	.	1
7221	0	1.35	1	0.90014	.	.	1
7222	0	1.35	1	0.90014	.	.	1
7223	0	1.36	0	0.89988	0.89704	0.90265	1
7224	0	1.36	1	0.89988	.	.	1
7225	0	1.36	1	0.89988	.	.	1
7226	0	1.36	1	0.89988	.	.	1
7227	0	1.36	1	0.89988	.	.	1
7228	0	1.36	1	0.89988	.	.	1
7229	0	1.36	1	0.89988	.	.	1
7230	0	1.36	1	0.89988	.	.	1
7231	0	1.36	1	0.89988	.	.	1
7232	0	1.36	1	0.89988	.	.	1
7233	0	1.36	1	0.89988	.	.	1
7234	0	1.36	1	0.89988	.	.	1
7235	0	1.36	1	0.89988	.	.	1
7236	0	1.36	1	0.89988	.	.	1
7237	0	1.36	1	0.89988	.	.	1
7238	0	1.36	1	0.89988	.	.	1
7239	0	1.36	1	0.89988	.	.	1
7240	0	1.36	1	0.89988	.	.	1
7241	0	1.36	1	0.89988	.	.	1
7242	0	1.36	1	0.89988	.	.	1
7243	0	1.36	1	0.89988	.	.	1
7244	0	1.36	1	0.89988	.	.	1
7245	0	1.36	1	0.89988	.	.	1

Obs	Sub_group	fuptimey	_CENSOR_	SURVIVAL	SDF_LCL	SDF_UCL	STRATUM
7246	0	1.36	1	0.89988	.	.	1
7247	0	1.36	1	0.89988	.	.	1
7248	0	1.36	1	0.89988	.	.	1
7249	0	1.36	1	0.89988	.	.	1
7250	0	1.36	1	0.89988	.	.	1
7251	0	1.36	1	0.89988	.	.	1
7252	0	1.36	1	0.89988	.	.	1
7253	0	1.36	1	0.89988	.	.	1
7254	0	1.36	1	0.89988	.	.	1
7255	0	1.36	1	0.89988	.	.	1
7256	0	1.36	1	0.89988	.	.	1
7257	0	1.36	1	0.89988	.	.	1
7258	0	1.36	1	0.89988	.	.	1
7259	0	1.36	1	0.89988	.	.	1
7260	0	1.36	1	0.89988	.	.	1
7261	0	1.36	1	0.89988	.	.	1
7262	0	1.36	1	0.89988	.	.	1
7263	0	1.36	1	0.89988	.	.	1
7264	0	1.36	1	0.89988	.	.	1
7265	0	1.36	1	0.89988	.	.	1
7266	0	1.37	0	0.89958	0.89673	0.90235	1
7267	0	1.37	1	0.89958	.	.	1
7268	0	1.37	1	0.89958	.	.	1
7269	0	1.37	1	0.89958	.	.	1
7270	0	1.37	1	0.89958	.	.	1
7271	0	1.37	1	0.89958	.	.	1
7272	0	1.37	1	0.89958	.	.	1
7273	0	1.37	1	0.89958	.	.	1
7274	0	1.37	1	0.89958	.	.	1
7275	0	1.37	1	0.89958	.	.	1
7276	0	1.37	1	0.89958	.	.	1
7277	0	1.37	1	0.89958	.	.	1
7278	0	1.37	1	0.89958	.	.	1
7279	0	1.37	1	0.89958	.	.	1
7280	0	1.37	1	0.89958	.	.	1

Obs	Sub_group	fuptimey	_CENSOR_	SURVIVAL	SDF_LCL	SDF_UCL	STRATUM
7281	0	1.37	1	0.89958	.	.	1
7282	0	1.37	1	0.89958	.	.	1
7283	0	1.37	1	0.89958	.	.	1
7284	0	1.37	1	0.89958	.	.	1
7285	0	1.37	1	0.89958	.	.	1
7286	0	1.37	1	0.89958	.	.	1
7287	0	1.37	1	0.89958	.	.	1
7288	0	1.37	1	0.89958	.	.	1
7289	0	1.37	1	0.89958	.	.	1
7290	0	1.37	1	0.89958	.	.	1
7291	0	1.37	1	0.89958	.	.	1
7292	0	1.37	1	0.89958	.	.	1
7293	0	1.37	1	0.89958	.	.	1
7294	0	1.37	1	0.89958	.	.	1
7295	0	1.37	1	0.89958	.	.	1
7296	0	1.37	1	0.89958	.	.	1
7297	0	1.37	1	0.89958	.	.	1
7298	0	1.37	1	0.89958	.	.	1
7299	0	1.37	1	0.89958	.	.	1
7300	0	1.37	1	0.89958	.	.	1
7301	0	1.37	1	0.89958	.	.	1
7302	0	1.37	1	0.89958	.	.	1
7303	0	1.37	1	0.89958	.	.	1
7304	0	1.37	1	0.89958	.	.	1
7305	0	1.37	1	0.89958	.	.	1
7306	0	1.37	1	0.89958	.	.	1
7307	0	1.37	1	0.89958	.	.	1
7308	0	1.37	1	0.89958	.	.	1
7309	0	1.37	1	0.89958	.	.	1
7310	0	1.37	1	0.89958	.	.	1
7311	0	1.37	1	0.89958	.	.	1
7312	0	1.37	1	0.89958	.	.	1
7313	0	1.37	1	0.89958	.	.	1
7314	0	1.37	1	0.89958	.	.	1
7315	0	1.37	1	0.89958	.	.	1

Obs	Sub_group	fuptimey	_CENSOR_	SURVIVAL	SDF_LCL	SDF_UCL	STRATUM
7316	0	1.37	1	0.89958	.	.	1
7317	0	1.37	1	0.89958	.	.	1
7318	0	1.37	1	0.89958	.	.	1
7319	0	1.37	1	0.89958	.	.	1
7320	0	1.37	1	0.89958	.	.	1
7321	0	1.37	1	0.89958	.	.	1
7322	0	1.37	1	0.89958	.	.	1
7323	0	1.37	1	0.89958	.	.	1
7324	0	1.37	1	0.89958	.	.	1
7325	0	1.37	1	0.89958	.	.	1
7326	0	1.37	1	0.89958	.	.	1
7327	0	1.37	1	0.89958	.	.	1
7328	0	1.38	0	0.89922	0.89637	0.90199	1
7329	0	1.38	1	0.89922	.	.	1
7330	0	1.38	1	0.89922	.	.	1
7331	0	1.38	1	0.89922	.	.	1
7332	0	1.38	1	0.89922	.	.	1
7333	0	1.38	1	0.89922	.	.	1
7334	0	1.38	1	0.89922	.	.	1
7335	0	1.38	1	0.89922	.	.	1
7336	0	1.38	1	0.89922	.	.	1
7337	0	1.38	1	0.89922	.	.	1
7338	0	1.38	1	0.89922	.	.	1
7339	0	1.38	1	0.89922	.	.	1
7340	0	1.38	1	0.89922	.	.	1
7341	0	1.38	1	0.89922	.	.	1
7342	0	1.38	1	0.89922	.	.	1
7343	0	1.38	1	0.89922	.	.	1
7344	0	1.38	1	0.89922	.	.	1
7345	0	1.38	1	0.89922	.	.	1
7346	0	1.38	1	0.89922	.	.	1
7347	0	1.38	1	0.89922	.	.	1
7348	0	1.38	1	0.89922	.	.	1
7349	0	1.38	1	0.89922	.	.	1
7350	0	1.38	1	0.89922	.	.	1

Obs	Sub_group	fuptimey	_CENSOR_	SURVIVAL	SDF_LCL	SDF_UCL	STRATUM
7351	0	1.38	1	0.89922	.	.	1
7352	0	1.38	1	0.89922	.	.	1
7353	0	1.38	1	0.89922	.	.	1
7354	0	1.38	1	0.89922	.	.	1
7355	0	1.38	1	0.89922	.	.	1
7356	0	1.38	1	0.89922	.	.	1
7357	0	1.38	1	0.89922	.	.	1
7358	0	1.38	1	0.89922	.	.	1
7359	0	1.38	1	0.89922	.	.	1
7360	0	1.38	1	0.89922	.	.	1
7361	0	1.38	1	0.89922	.	.	1
7362	0	1.38	1	0.89922	.	.	1
7363	0	1.38	1	0.89922	.	.	1
7364	0	1.38	1	0.89922	.	.	1
7365	0	1.38	1	0.89922	.	.	1
7366	0	1.38	1	0.89922	.	.	1
7367	0	1.38	1	0.89922	.	.	1
7368	0	1.38	1	0.89922	.	.	1
7369	0	1.38	1	0.89922	.	.	1
7370	0	1.38	1	0.89922	.	.	1
7371	0	1.38	1	0.89922	.	.	1
7372	0	1.38	1	0.89922	.	.	1
7373	0	1.38	1	0.89922	.	.	1
7374	0	1.38	1	0.89922	.	.	1
7375	0	1.38	1	0.89922	.	.	1
7376	0	1.38	1	0.89922	.	.	1
7377	0	1.38	1	0.89922	.	.	1
7378	0	1.38	1	0.89922	.	.	1
7379	0	1.38	1	0.89922	.	.	1
7380	0	1.38	1	0.89922	.	.	1
7381	0	1.38	1	0.89922	.	.	1
7382	0	1.38	1	0.89922	.	.	1
7383	0	1.39	0	0.89901	0.89616	0.90179	1
7384	0	1.39	1	0.89901	.	.	1
7385	0	1.39	1	0.89901	.	.	1

Obs	Sub_group	fuptimey	_CENSOR_	SURVIVAL	SDF_LCL	SDF_UCL	STRATUM
7386	0	1.39	1	0.89901	.	.	1
7387	0	1.39	1	0.89901	.	.	1
7388	0	1.39	1	0.89901	.	.	1
7389	0	1.39	1	0.89901	.	.	1
7390	0	1.39	1	0.89901	.	.	1
7391	0	1.39	1	0.89901	.	.	1
7392	0	1.39	1	0.89901	.	.	1
7393	0	1.39	1	0.89901	.	.	1
7394	0	1.39	1	0.89901	.	.	1
7395	0	1.39	1	0.89901	.	.	1
7396	0	1.39	1	0.89901	.	.	1
7397	0	1.39	1	0.89901	.	.	1
7398	0	1.39	1	0.89901	.	.	1
7399	0	1.39	1	0.89901	.	.	1
7400	0	1.39	1	0.89901	.	.	1
7401	0	1.39	1	0.89901	.	.	1
7402	0	1.39	1	0.89901	.	.	1
7403	0	1.39	1	0.89901	.	.	1
7404	0	1.39	1	0.89901	.	.	1
7405	0	1.39	1	0.89901	.	.	1
7406	0	1.39	1	0.89901	.	.	1
7407	0	1.39	1	0.89901	.	.	1
7408	0	1.39	1	0.89901	.	.	1
7409	0	1.39	1	0.89901	.	.	1
7410	0	1.39	1	0.89901	.	.	1
7411	0	1.39	1	0.89901	.	.	1
7412	0	1.39	1	0.89901	.	.	1
7413	0	1.39	1	0.89901	.	.	1
7414	0	1.39	1	0.89901	.	.	1
7415	0	1.39	1	0.89901	.	.	1
7416	0	1.39	1	0.89901	.	.	1
7417	0	1.39	1	0.89901	.	.	1
7418	0	1.39	1	0.89901	.	.	1
7419	0	1.39	1	0.89901	.	.	1
7420	0	1.39	1	0.89901	.	.	1

Obs	Sub_group	fuptimey	_CENSOR_	SURVIVAL	SDF_LCL	SDF_UCL	STRATUM
7421	0	1.39	1	0.89901	.	.	1
7422	0	1.39	1	0.89901	.	.	1
7423	0	1.39	1	0.89901	.	.	1
7424	0	1.39	1	0.89901	.	.	1
7425	0	1.39	1	0.89901	.	.	1
7426	0	1.39	1	0.89901	.	.	1
7427	0	1.39	1	0.89901	.	.	1
7428	0	1.39	1	0.89901	.	.	1
7429	0	1.39	1	0.89901	.	.	1
7430	0	1.39	1	0.89901	.	.	1
7431	0	1.39	1	0.89901	.	.	1
7432	0	1.39	1	0.89901	.	.	1
7433	0	1.39	1	0.89901	.	.	1
7434	0	1.39	1	0.89901	.	.	1
7435	0	1.39	1	0.89901	.	.	1
7436	0	1.39	1	0.89901	.	.	1
7437	0	1.39	1	0.89901	.	.	1
7438	0	1.39	1	0.89901	.	.	1
7439	0	1.39	1	0.89901	.	.	1
7440	0	1.39	1	0.89901	.	.	1
7441	0	1.39	1	0.89901	.	.	1
7442	0	1.39	1	0.89901	.	.	1
7443	0	1.40	0	0.89855	0.89569	0.90133	1
7444	0	1.40	1	0.89855	.	.	1
7445	0	1.40	1	0.89855	.	.	1
7446	0	1.40	1	0.89855	.	.	1
7447	0	1.40	1	0.89855	.	.	1
7448	0	1.40	1	0.89855	.	.	1
7449	0	1.40	1	0.89855	.	.	1
7450	0	1.40	1	0.89855	.	.	1
7451	0	1.40	1	0.89855	.	.	1
7452	0	1.40	1	0.89855	.	.	1
7453	0	1.40	1	0.89855	.	.	1
7454	0	1.40	1	0.89855	.	.	1
7455	0	1.40	1	0.89855	.	.	1

Obs	Sub_group	fuptimey	_CENSOR_	SURVIVAL	SDF_LCL	SDF_UCL	STRATUM
7456	0	1.40	1	0.89855	.	.	1
7457	0	1.40	1	0.89855	.	.	1
7458	0	1.40	1	0.89855	.	.	1
7459	0	1.40	1	0.89855	.	.	1
7460	0	1.40	1	0.89855	.	.	1
7461	0	1.40	1	0.89855	.	.	1
7462	0	1.40	1	0.89855	.	.	1
7463	0	1.40	1	0.89855	.	.	1
7464	0	1.40	1	0.89855	.	.	1
7465	0	1.40	1	0.89855	.	.	1
7466	0	1.40	1	0.89855	.	.	1
7467	0	1.40	1	0.89855	.	.	1
7468	0	1.40	1	0.89855	.	.	1
7469	0	1.40	1	0.89855	.	.	1
7470	0	1.40	1	0.89855	.	.	1
7471	0	1.40	1	0.89855	.	.	1
7472	0	1.40	1	0.89855	.	.	1
7473	0	1.40	1	0.89855	.	.	1
7474	0	1.40	1	0.89855	.	.	1
7475	0	1.40	1	0.89855	.	.	1
7476	0	1.40	1	0.89855	.	.	1
7477	0	1.40	1	0.89855	.	.	1
7478	0	1.40	1	0.89855	.	.	1
7479	0	1.40	1	0.89855	.	.	1
7480	0	1.40	1	0.89855	.	.	1
7481	0	1.40	1	0.89855	.	.	1
7482	0	1.40	1	0.89855	.	.	1
7483	0	1.40	1	0.89855	.	.	1
7484	0	1.40	1	0.89855	.	.	1
7485	0	1.40	1	0.89855	.	.	1
7486	0	1.40	1	0.89855	.	.	1
7487	0	1.40	1	0.89855	.	.	1
7488	0	1.40	1	0.89855	.	.	1
7489	0	1.40	1	0.89855	.	.	1
7490	0	1.40	1	0.89855	.	.	1

Obs	Sub_group	fuptimey	_CENSOR_	SURVIVAL	SDF_LCL	SDF_UCL	STRATUM
7491	0	1.41	0	0.89834	0.89548	0.90113	1
7492	0	1.41	1	0.89834	.	.	1
7493	0	1.41	1	0.89834	.	.	1
7494	0	1.41	1	0.89834	.	.	1
7495	0	1.41	1	0.89834	.	.	1
7496	0	1.41	1	0.89834	.	.	1
7497	0	1.41	1	0.89834	.	.	1
7498	0	1.41	1	0.89834	.	.	1
7499	0	1.41	1	0.89834	.	.	1
7500	0	1.41	1	0.89834	.	.	1
7501	0	1.41	1	0.89834	.	.	1
7502	0	1.41	1	0.89834	.	.	1
7503	0	1.41	1	0.89834	.	.	1
7504	0	1.41	1	0.89834	.	.	1
7505	0	1.41	1	0.89834	.	.	1
7506	0	1.41	1	0.89834	.	.	1
7507	0	1.41	1	0.89834	.	.	1
7508	0	1.41	1	0.89834	.	.	1
7509	0	1.41	1	0.89834	.	.	1
7510	0	1.41	1	0.89834	.	.	1
7511	0	1.41	1	0.89834	.	.	1
7512	0	1.41	1	0.89834	.	.	1
7513	0	1.41	1	0.89834	.	.	1
7514	0	1.41	1	0.89834	.	.	1
7515	0	1.41	1	0.89834	.	.	1
7516	0	1.41	1	0.89834	.	.	1
7517	0	1.41	1	0.89834	.	.	1
7518	0	1.41	1	0.89834	.	.	1
7519	0	1.41	1	0.89834	.	.	1
7520	0	1.41	1	0.89834	.	.	1
7521	0	1.41	1	0.89834	.	.	1
7522	0	1.41	1	0.89834	.	.	1
7523	0	1.41	1	0.89834	.	.	1
7524	0	1.41	1	0.89834	.	.	1
7525	0	1.41	1	0.89834	.	.	1

Obs	Sub_group	fuptimey	_CENSOR_	SURVIVAL	SDF_LCL	SDF_UCL	STRATUM
7526	0	1.41	1	0.89834	.	.	1
7527	0	1.41	1	0.89834	.	.	1
7528	0	1.41	1	0.89834	.	.	1
7529	0	1.41	1	0.89834	.	.	1
7530	0	1.41	1	0.89834	.	.	1
7531	0	1.41	1	0.89834	.	.	1
7532	0	1.41	1	0.89834	.	.	1
7533	0	1.41	1	0.89834	.	.	1
7534	0	1.41	1	0.89834	.	.	1
7535	0	1.41	1	0.89834	.	.	1
7536	0	1.41	1	0.89834	.	.	1
7537	0	1.41	1	0.89834	.	.	1
7538	0	1.41	1	0.89834	.	.	1
7539	0	1.41	1	0.89834	.	.	1
7540	0	1.41	1	0.89834	.	.	1
7541	0	1.41	1	0.89834	.	.	1
7542	0	1.41	1	0.89834	.	.	1
7543	0	1.41	1	0.89834	.	.	1
7544	0	1.41	1	0.89834	.	.	1
7545	0	1.41	1	0.89834	.	.	1
7546	0	1.41	1	0.89834	.	.	1
7547	0	1.41	1	0.89834	.	.	1
7548	0	1.41	1	0.89834	.	.	1
7549	0	1.41	1	0.89834	.	.	1
7550	0	1.41	1	0.89834	.	.	1
7551	0	1.41	1	0.89834	.	.	1
7552	0	1.41	1	0.89834	.	.	1
7553	0	1.41	1	0.89834	.	.	1
7554	0	1.42	0	0.89816	0.89529	0.90095	1
7555	0	1.42	1	0.89816	.	.	1
7556	0	1.42	1	0.89816	.	.	1
7557	0	1.42	1	0.89816	.	.	1
7558	0	1.42	1	0.89816	.	.	1
7559	0	1.42	1	0.89816	.	.	1
7560	0	1.42	1	0.89816	.	.	1

Obs	Sub_group	fuptimey	_CENSOR_	SURVIVAL	SDF_LCL	SDF_UCL	STRATUM
7561	0	1.42	1	0.89816	.	.	1
7562	0	1.42	1	0.89816	.	.	1
7563	0	1.42	1	0.89816	.	.	1
7564	0	1.42	1	0.89816	.	.	1
7565	0	1.42	1	0.89816	.	.	1
7566	0	1.42	1	0.89816	.	.	1
7567	0	1.42	1	0.89816	.	.	1
7568	0	1.42	1	0.89816	.	.	1
7569	0	1.42	1	0.89816	.	.	1
7570	0	1.42	1	0.89816	.	.	1
7571	0	1.42	1	0.89816	.	.	1
7572	0	1.42	1	0.89816	.	.	1
7573	0	1.42	1	0.89816	.	.	1
7574	0	1.42	1	0.89816	.	.	1
7575	0	1.42	1	0.89816	.	.	1
7576	0	1.42	1	0.89816	.	.	1
7577	0	1.42	1	0.89816	.	.	1
7578	0	1.42	1	0.89816	.	.	1
7579	0	1.42	1	0.89816	.	.	1
7580	0	1.42	1	0.89816	.	.	1
7581	0	1.42	1	0.89816	.	.	1
7582	0	1.42	1	0.89816	.	.	1
7583	0	1.42	1	0.89816	.	.	1
7584	0	1.42	1	0.89816	.	.	1
7585	0	1.42	1	0.89816	.	.	1
7586	0	1.42	1	0.89816	.	.	1
7587	0	1.42	1	0.89816	.	.	1
7588	0	1.42	1	0.89816	.	.	1
7589	0	1.42	1	0.89816	.	.	1
7590	0	1.42	1	0.89816	.	.	1
7591	0	1.42	1	0.89816	.	.	1
7592	0	1.42	1	0.89816	.	.	1
7593	0	1.42	1	0.89816	.	.	1
7594	0	1.42	1	0.89816	.	.	1
7595	0	1.42	1	0.89816	.	.	1

Obs	Sub_group	fuptimey	_CENSOR_	SURVIVAL	SDF_LCL	SDF_UCL	STRATUM
7596	0	1.42	1	0.89816	.	.	1
7597	0	1.42	1	0.89816	.	.	1
7598	0	1.42	1	0.89816	.	.	1
7599	0	1.42	1	0.89816	.	.	1
7600	0	1.42	1	0.89816	.	.	1
7601	0	1.42	1	0.89816	.	.	1
7602	0	1.42	1	0.89816	.	.	1
7603	0	1.42	1	0.89816	.	.	1
7604	0	1.42	1	0.89816	.	.	1
7605	0	1.42	1	0.89816	.	.	1
7606	0	1.42	1	0.89816	.	.	1
7607	0	1.42	1	0.89816	.	.	1
7608	0	1.42	1	0.89816	.	.	1
7609	0	1.42	1	0.89816	.	.	1
7610	0	1.42	1	0.89816	.	.	1
7611	0	1.42	1	0.89816	.	.	1
7612	0	1.42	1	0.89816	.	.	1
7613	0	1.42	1	0.89816	.	.	1
7614	0	1.43	0	0.89759	0.89472	0.90039	1
7615	0	1.43	1	0.89759	.	.	1
7616	0	1.43	1	0.89759	.	.	1
7617	0	1.43	1	0.89759	.	.	1
7618	0	1.43	1	0.89759	.	.	1
7619	0	1.43	1	0.89759	.	.	1
7620	0	1.43	1	0.89759	.	.	1
7621	0	1.43	1	0.89759	.	.	1
7622	0	1.43	1	0.89759	.	.	1
7623	0	1.43	1	0.89759	.	.	1
7624	0	1.43	1	0.89759	.	.	1
7625	0	1.43	1	0.89759	.	.	1
7626	0	1.43	1	0.89759	.	.	1
7627	0	1.43	1	0.89759	.	.	1
7628	0	1.43	1	0.89759	.	.	1
7629	0	1.43	1	0.89759	.	.	1
7630	0	1.43	1	0.89759	.	.	1

Obs	Sub_group	fuptimey	_CENSOR_	SURVIVAL	SDF_LCL	SDF_UCL	STRATUM
7631	0	1.43	1	0.89759	.	.	1
7632	0	1.43	1	0.89759	.	.	1
7633	0	1.43	1	0.89759	.	.	1
7634	0	1.43	1	0.89759	.	.	1
7635	0	1.43	1	0.89759	.	.	1
7636	0	1.43	1	0.89759	.	.	1
7637	0	1.43	1	0.89759	.	.	1
7638	0	1.43	1	0.89759	.	.	1
7639	0	1.43	1	0.89759	.	.	1
7640	0	1.43	1	0.89759	.	.	1
7641	0	1.43	1	0.89759	.	.	1
7642	0	1.43	1	0.89759	.	.	1
7643	0	1.43	1	0.89759	.	.	1
7644	0	1.43	1	0.89759	.	.	1
7645	0	1.43	1	0.89759	.	.	1
7646	0	1.43	1	0.89759	.	.	1
7647	0	1.43	1	0.89759	.	.	1
7648	0	1.43	1	0.89759	.	.	1
7649	0	1.43	1	0.89759	.	.	1
7650	0	1.43	1	0.89759	.	.	1
7651	0	1.43	1	0.89759	.	.	1
7652	0	1.43	1	0.89759	.	.	1
7653	0	1.43	1	0.89759	.	.	1
7654	0	1.43	1	0.89759	.	.	1
7655	0	1.43	1	0.89759	.	.	1
7656	0	1.43	1	0.89759	.	.	1
7657	0	1.43	1	0.89759	.	.	1
7658	0	1.43	1	0.89759	.	.	1
7659	0	1.43	1	0.89759	.	.	1
7660	0	1.43	1	0.89759	.	.	1
7661	0	1.43	1	0.89759	.	.	1
7662	0	1.43	1	0.89759	.	.	1
7663	0	1.43	1	0.89759	.	.	1
7664	0	1.43	1	0.89759	.	.	1
7665	0	1.43	1	0.89759	.	.	1

Obs	Sub_group	fuptimey	_CENSOR_	SURVIVAL	SDF_LCL	SDF_UCL	STRATUM
7666	0	1.43	1	0.89759	.	.	1
7667	0	1.43	1	0.89759	.	.	1
7668	0	1.43	1	0.89759	.	.	1
7669	0	1.43	1	0.89759	.	.	1
7670	0	1.43	1	0.89759	.	.	1
7671	0	1.43	1	0.89759	.	.	1
7672	0	1.43	1	0.89759	.	.	1
7673	0	1.43	1	0.89759	.	.	1
7674	0	1.43	1	0.89759	.	.	1
7675	0	1.43	1	0.89759	.	.	1
7676	0	1.44	0	0.89738	0.89451	0.90018	1
7677	0	1.44	1	0.89738	.	.	1
7678	0	1.44	1	0.89738	.	.	1
7679	0	1.44	1	0.89738	.	.	1
7680	0	1.44	1	0.89738	.	.	1
7681	0	1.44	1	0.89738	.	.	1
7682	0	1.44	1	0.89738	.	.	1
7683	0	1.44	1	0.89738	.	.	1
7684	0	1.44	1	0.89738	.	.	1
7685	0	1.44	1	0.89738	.	.	1
7686	0	1.44	1	0.89738	.	.	1
7687	0	1.44	1	0.89738	.	.	1
7688	0	1.44	1	0.89738	.	.	1
7689	0	1.44	1	0.89738	.	.	1
7690	0	1.44	1	0.89738	.	.	1
7691	0	1.44	1	0.89738	.	.	1
7692	0	1.44	1	0.89738	.	.	1
7693	0	1.44	1	0.89738	.	.	1
7694	0	1.44	1	0.89738	.	.	1
7695	0	1.44	1	0.89738	.	.	1
7696	0	1.44	1	0.89738	.	.	1
7697	0	1.44	1	0.89738	.	.	1
7698	0	1.44	1	0.89738	.	.	1
7699	0	1.44	1	0.89738	.	.	1
7700	0	1.44	1	0.89738	.	.	1

Obs	Sub_group	fuptimey	_CENSOR_	SURVIVAL	SDF_LCL	SDF_UCL	STRATUM
7701	0	1.44	1	0.89738	.	.	1
7702	0	1.44	1	0.89738	.	.	1
7703	0	1.44	1	0.89738	.	.	1
7704	0	1.44	1	0.89738	.	.	1
7705	0	1.44	1	0.89738	.	.	1
7706	0	1.44	1	0.89738	.	.	1
7707	0	1.44	1	0.89738	.	.	1
7708	0	1.44	1	0.89738	.	.	1
7709	0	1.44	1	0.89738	.	.	1
7710	0	1.44	1	0.89738	.	.	1
7711	0	1.44	1	0.89738	.	.	1
7712	0	1.44	1	0.89738	.	.	1
7713	0	1.44	1	0.89738	.	.	1
7714	0	1.44	1	0.89738	.	.	1
7715	0	1.44	1	0.89738	.	.	1
7716	0	1.44	1	0.89738	.	.	1
7717	0	1.44	1	0.89738	.	.	1
7718	0	1.44	1	0.89738	.	.	1
7719	0	1.44	1	0.89738	.	.	1
7720	0	1.44	1	0.89738	.	.	1
7721	0	1.44	1	0.89738	.	.	1
7722	0	1.44	1	0.89738	.	.	1
7723	0	1.44	1	0.89738	.	.	1
7724	0	1.44	1	0.89738	.	.	1
7725	0	1.44	1	0.89738	.	.	1
7726	0	1.44	1	0.89738	.	.	1
7727	0	1.44	1	0.89738	.	.	1
7728	0	1.44	1	0.89738	.	.	1
7729	0	1.44	1	0.89738	.	.	1
7730	0	1.44	1	0.89738	.	.	1
7731	0	1.44	1	0.89738	.	.	1
7732	0	1.44	1	0.89738	.	.	1
7733	0	1.44	1	0.89738	.	.	1
7734	0	1.45	0	0.89699	0.89411	0.89980	1
7735	0	1.45	1	0.89699	.	.	1

Obs	Sub_group	fuptimey	_CENSOR_	SURVIVAL	SDF_LCL	SDF_UCL	STRATUM
7736	0	1.45	1	0.89699	.	.	1
7737	0	1.45	1	0.89699	.	.	1
7738	0	1.45	1	0.89699	.	.	1
7739	0	1.45	1	0.89699	.	.	1
7740	0	1.45	1	0.89699	.	.	1
7741	0	1.45	1	0.89699	.	.	1
7742	0	1.45	1	0.89699	.	.	1
7743	0	1.45	1	0.89699	.	.	1
7744	0	1.45	1	0.89699	.	.	1
7745	0	1.45	1	0.89699	.	.	1
7746	0	1.45	1	0.89699	.	.	1
7747	0	1.45	1	0.89699	.	.	1
7748	0	1.45	1	0.89699	.	.	1
7749	0	1.45	1	0.89699	.	.	1
7750	0	1.45	1	0.89699	.	.	1
7751	0	1.45	1	0.89699	.	.	1
7752	0	1.45	1	0.89699	.	.	1
7753	0	1.45	1	0.89699	.	.	1
7754	0	1.45	1	0.89699	.	.	1
7755	0	1.45	1	0.89699	.	.	1
7756	0	1.45	1	0.89699	.	.	1
7757	0	1.45	1	0.89699	.	.	1
7758	0	1.45	1	0.89699	.	.	1
7759	0	1.45	1	0.89699	.	.	1
7760	0	1.45	1	0.89699	.	.	1
7761	0	1.45	1	0.89699	.	.	1
7762	0	1.45	1	0.89699	.	.	1
7763	0	1.45	1	0.89699	.	.	1
7764	0	1.45	1	0.89699	.	.	1
7765	0	1.45	1	0.89699	.	.	1
7766	0	1.45	1	0.89699	.	.	1
7767	0	1.45	1	0.89699	.	.	1
7768	0	1.45	1	0.89699	.	.	1
7769	0	1.45	1	0.89699	.	.	1
7770	0	1.45	1	0.89699	.	.	1

Obs	Sub_group	fuptimey	_CENSOR_	SURVIVAL	SDF_LCL	SDF_UCL	STRATUM
7771	0	1.45	1	0.89699	.	.	1
7772	0	1.45	1	0.89699	.	.	1
7773	0	1.45	1	0.89699	.	.	1
7774	0	1.45	1	0.89699	.	.	1
7775	0	1.45	1	0.89699	.	.	1
7776	0	1.46	0	0.89673	0.89385	0.89954	1
7777	0	1.46	1	0.89673	.	.	1
7778	0	1.46	1	0.89673	.	.	1
7779	0	1.46	1	0.89673	.	.	1
7780	0	1.46	1	0.89673	.	.	1
7781	0	1.46	1	0.89673	.	.	1
7782	0	1.46	1	0.89673	.	.	1
7783	0	1.46	1	0.89673	.	.	1
7784	0	1.46	1	0.89673	.	.	1
7785	0	1.46	1	0.89673	.	.	1
7786	0	1.46	1	0.89673	.	.	1
7787	0	1.46	1	0.89673	.	.	1
7788	0	1.46	1	0.89673	.	.	1
7789	0	1.46	1	0.89673	.	.	1
7790	0	1.46	1	0.89673	.	.	1
7791	0	1.46	1	0.89673	.	.	1
7792	0	1.46	1	0.89673	.	.	1
7793	0	1.46	1	0.89673	.	.	1
7794	0	1.46	1	0.89673	.	.	1
7795	0	1.46	1	0.89673	.	.	1
7796	0	1.46	1	0.89673	.	.	1
7797	0	1.46	1	0.89673	.	.	1
7798	0	1.46	1	0.89673	.	.	1
7799	0	1.46	1	0.89673	.	.	1
7800	0	1.46	1	0.89673	.	.	1
7801	0	1.46	1	0.89673	.	.	1
7802	0	1.46	1	0.89673	.	.	1
7803	0	1.46	1	0.89673	.	.	1
7804	0	1.46	1	0.89673	.	.	1
7805	0	1.46	1	0.89673	.	.	1

Obs	Sub_group	fuptimey	_CENSOR_	SURVIVAL	SDF_LCL	SDF_UCL	STRATUM
7806	0	1.46	1	0.89673	.	.	1
7807	0	1.46	1	0.89673	.	.	1
7808	0	1.46	1	0.89673	.	.	1
7809	0	1.46	1	0.89673	.	.	1
7810	0	1.46	1	0.89673	.	.	1
7811	0	1.46	1	0.89673	.	.	1
7812	0	1.46	1	0.89673	.	.	1
7813	0	1.46	1	0.89673	.	.	1
7814	0	1.46	1	0.89673	.	.	1
7815	0	1.46	1	0.89673	.	.	1
7816	0	1.46	1	0.89673	.	.	1
7817	0	1.46	1	0.89673	.	.	1
7818	0	1.46	1	0.89673	.	.	1
7819	0	1.46	1	0.89673	.	.	1
7820	0	1.46	1	0.89673	.	.	1
7821	0	1.46	1	0.89673	.	.	1
7822	0	1.46	1	0.89673	.	.	1
7823	0	1.46	1	0.89673	.	.	1
7824	0	1.46	1	0.89673	.	.	1
7825	0	1.46	1	0.89673	.	.	1
7826	0	1.46	1	0.89673	.	.	1
7827	0	1.46	1	0.89673	.	.	1
7828	0	1.47	0	0.89637	0.89348	0.89918	1
7829	0	1.47	1	0.89637	.	.	1
7830	0	1.47	1	0.89637	.	.	1
7831	0	1.47	1	0.89637	.	.	1
7832	0	1.47	1	0.89637	.	.	1
7833	0	1.47	1	0.89637	.	.	1
7834	0	1.47	1	0.89637	.	.	1
7835	0	1.47	1	0.89637	.	.	1
7836	0	1.47	1	0.89637	.	.	1
7837	0	1.47	1	0.89637	.	.	1
7838	0	1.47	1	0.89637	.	.	1
7839	0	1.47	1	0.89637	.	.	1
7840	0	1.47	1	0.89637	.	.	1

Obs	Sub_group	fuptimey	_CENSOR_	SURVIVAL	SDF_LCL	SDF_UCL	STRATUM
7841	0	1.47	1	0.89637	.	.	1
7842	0	1.47	1	0.89637	.	.	1
7843	0	1.47	1	0.89637	.	.	1
7844	0	1.47	1	0.89637	.	.	1
7845	0	1.47	1	0.89637	.	.	1
7846	0	1.47	1	0.89637	.	.	1
7847	0	1.47	1	0.89637	.	.	1
7848	0	1.47	1	0.89637	.	.	1
7849	0	1.47	1	0.89637	.	.	1
7850	0	1.47	1	0.89637	.	.	1
7851	0	1.47	1	0.89637	.	.	1
7852	0	1.47	1	0.89637	.	.	1
7853	0	1.47	1	0.89637	.	.	1
7854	0	1.47	1	0.89637	.	.	1
7855	0	1.47	1	0.89637	.	.	1
7856	0	1.47	1	0.89637	.	.	1
7857	0	1.47	1	0.89637	.	.	1
7858	0	1.47	1	0.89637	.	.	1
7859	0	1.47	1	0.89637	.	.	1
7860	0	1.47	1	0.89637	.	.	1
7861	0	1.47	1	0.89637	.	.	1
7862	0	1.47	1	0.89637	.	.	1
7863	0	1.47	1	0.89637	.	.	1
7864	0	1.47	1	0.89637	.	.	1
7865	0	1.47	1	0.89637	.	.	1
7866	0	1.47	1	0.89637	.	.	1
7867	0	1.47	1	0.89637	.	.	1
7868	0	1.47	1	0.89637	.	.	1
7869	0	1.47	1	0.89637	.	.	1
7870	0	1.47	1	0.89637	.	.	1
7871	0	1.48	0	0.89598	0.89308	0.89880	1
7872	0	1.48	1	0.89598	.	.	1
7873	0	1.48	1	0.89598	.	.	1
7874	0	1.48	1	0.89598	.	.	1
7875	0	1.48	1	0.89598	.	.	1

Obs	Sub_group	fuptimey	_CENSOR_	SURVIVAL	SDF_LCL	SDF_UCL	STRATUM
7876	0	1.48	1	0.89598	.	.	1
7877	0	1.48	1	0.89598	.	.	1
7878	0	1.48	1	0.89598	.	.	1
7879	0	1.48	1	0.89598	.	.	1
7880	0	1.48	1	0.89598	.	.	1
7881	0	1.48	1	0.89598	.	.	1
7882	0	1.48	1	0.89598	.	.	1
7883	0	1.48	1	0.89598	.	.	1
7884	0	1.48	1	0.89598	.	.	1
7885	0	1.48	1	0.89598	.	.	1
7886	0	1.48	1	0.89598	.	.	1
7887	0	1.48	1	0.89598	.	.	1
7888	0	1.48	1	0.89598	.	.	1
7889	0	1.48	1	0.89598	.	.	1
7890	0	1.48	1	0.89598	.	.	1
7891	0	1.48	1	0.89598	.	.	1
7892	0	1.48	1	0.89598	.	.	1
7893	0	1.48	1	0.89598	.	.	1
7894	0	1.48	1	0.89598	.	.	1
7895	0	1.48	1	0.89598	.	.	1
7896	0	1.48	1	0.89598	.	.	1
7897	0	1.48	1	0.89598	.	.	1
7898	0	1.48	1	0.89598	.	.	1
7899	0	1.48	1	0.89598	.	.	1
7900	0	1.48	1	0.89598	.	.	1
7901	0	1.48	1	0.89598	.	.	1
7902	0	1.48	1	0.89598	.	.	1
7903	0	1.48	1	0.89598	.	.	1
7904	0	1.48	1	0.89598	.	.	1
7905	0	1.48	1	0.89598	.	.	1
7906	0	1.48	1	0.89598	.	.	1
7907	0	1.48	1	0.89598	.	.	1
7908	0	1.48	1	0.89598	.	.	1
7909	0	1.48	1	0.89598	.	.	1
7910	0	1.48	1	0.89598	.	.	1

Obs	Sub_group	fuptimey	_CENSOR_	SURVIVAL	SDF_LCL	SDF_UCL	STRATUM
7911	0	1.48	1	0.89598	.	.	1
7912	0	1.48	1	0.89598	.	.	1
7913	0	1.48	1	0.89598	.	.	1
7914	0	1.48	1	0.89598	.	.	1
7915	0	1.48	1	0.89598	.	.	1
7916	0	1.48	1	0.89598	.	.	1
7917	0	1.48	1	0.89598	.	.	1
7918	0	1.48	1	0.89598	.	.	1
7919	0	1.48	1	0.89598	.	.	1
7920	0	1.49	0	0.89553	0.89263	0.89836	1
7921	0	1.49	1	0.89553	.	.	1
7922	0	1.49	1	0.89553	.	.	1
7923	0	1.49	1	0.89553	.	.	1
7924	0	1.49	1	0.89553	.	.	1
7925	0	1.49	1	0.89553	.	.	1
7926	0	1.49	1	0.89553	.	.	1
7927	0	1.49	1	0.89553	.	.	1
7928	0	1.49	1	0.89553	.	.	1
7929	0	1.49	1	0.89553	.	.	1
7930	0	1.49	1	0.89553	.	.	1
7931	0	1.49	1	0.89553	.	.	1
7932	0	1.49	1	0.89553	.	.	1
7933	0	1.49	1	0.89553	.	.	1
7934	0	1.49	1	0.89553	.	.	1
7935	0	1.49	1	0.89553	.	.	1
7936	0	1.49	1	0.89553	.	.	1
7937	0	1.49	1	0.89553	.	.	1
7938	0	1.49	1	0.89553	.	.	1
7939	0	1.49	1	0.89553	.	.	1
7940	0	1.49	1	0.89553	.	.	1
7941	0	1.49	1	0.89553	.	.	1
7942	0	1.49	1	0.89553	.	.	1
7943	0	1.49	1	0.89553	.	.	1
7944	0	1.49	1	0.89553	.	.	1
7945	0	1.49	1	0.89553	.	.	1

Obs	Sub_group	fuptimey	_CENSOR_	SURVIVAL	SDF_LCL	SDF_UCL	STRATUM
7946	0	1.49	1	0.89553	.	.	1
7947	0	1.49	1	0.89553	.	.	1
7948	0	1.49	1	0.89553	.	.	1
7949	0	1.49	1	0.89553	.	.	1
7950	0	1.49	1	0.89553	.	.	1
7951	0	1.49	1	0.89553	.	.	1
7952	0	1.49	1	0.89553	.	.	1
7953	0	1.49	1	0.89553	.	.	1
7954	0	1.49	1	0.89553	.	.	1
7955	0	1.49	1	0.89553	.	.	1
7956	0	1.49	1	0.89553	.	.	1
7957	0	1.49	1	0.89553	.	.	1
7958	0	1.49	1	0.89553	.	.	1
7959	0	1.49	1	0.89553	.	.	1
7960	0	1.49	1	0.89553	.	.	1
7961	0	1.49	1	0.89553	.	.	1
7962	0	1.49	1	0.89553	.	.	1
7963	0	1.49	1	0.89553	.	.	1
7964	0	1.49	1	0.89553	.	.	1
7965	0	1.49	1	0.89553	.	.	1
7966	0	1.49	1	0.89553	.	.	1
7967	0	1.49	1	0.89553	.	.	1
7968	0	1.49	1	0.89553	.	.	1
7969	0	1.49	1	0.89553	.	.	1
7970	0	1.49	1	0.89553	.	.	1
7971	0	1.49	1	0.89553	.	.	1
7972	0	1.49	1	0.89553	.	.	1
7973	0	1.49	1	0.89553	.	.	1
7974	0	1.49	1	0.89553	.	.	1
7975	0	1.49	1	0.89553	.	.	1
7976	0	1.49	1	0.89553	.	.	1
7977	0	1.49	1	0.89553	.	.	1
7978	0	1.49	1	0.89553	.	.	1
7979	0	1.50	0	0.89517	0.89226	0.89800	1
7980	0	1.50	1	0.89517	.	.	1

Obs	Sub_group	fuptimey	_CENSOR_	SURVIVAL	SDF_LCL	SDF_UCL	STRATUM
**7981**	0	1.50	1	0.89517	.	.	1
**7982**	0	1.50	1	0.89517	.	.	1
**7983**	0	1.50	1	0.89517	.	.	1
**7984**	0	1.50	1	0.89517	.	.	1
**7985**	0	1.50	1	0.89517	.	.	1
**7986**	0	1.50	1	0.89517	.	.	1
**7987**	0	1.50	1	0.89517	.	.	1
**7988**	0	1.50	1	0.89517	.	.	1
**7989**	0	1.50	1	0.89517	.	.	1
**7990**	0	1.50	1	0.89517	.	.	1
**7991**	0	1.50	1	0.89517	.	.	1
**7992**	0	1.50	1	0.89517	.	.	1
**7993**	0	1.50	1	0.89517	.	.	1
**7994**	0	1.50	1	0.89517	.	.	1
**7995**	0	1.50	1	0.89517	.	.	1
**7996**	0	1.50	1	0.89517	.	.	1
**7997**	0	1.50	1	0.89517	.	.	1
**7998**	0	1.50	1	0.89517	.	.	1
**7999**	0	1.50	1	0.89517	.	.	1
**8000**	0	1.50	1	0.89517	.	.	1
**8001**	0	1.50	1	0.89517	.	.	1
**8002**	0	1.50	1	0.89517	.	.	1
**8003**	0	1.50	1	0.89517	.	.	1
**8004**	0	1.50	1	0.89517	.	.	1
**8005**	0	1.50	1	0.89517	.	.	1
**8006**	0	1.50	1	0.89517	.	.	1
**8007**	0	1.50	1	0.89517	.	.	1
**8008**	0	1.50	1	0.89517	.	.	1
**8009**	0	1.50	1	0.89517	.	.	1
**8010**	0	1.50	1	0.89517	.	.	1
**8011**	0	1.50	1	0.89517	.	.	1
**8012**	0	1.50	1	0.89517	.	.	1
**8013**	0	1.50	1	0.89517	.	.	1
**8014**	0	1.50	1	0.89517	.	.	1
**8015**	0	1.50	1	0.89517	.	.	1

Obs	Sub_group	fuptimey	_CENSOR_	SURVIVAL	SDF_LCL	SDF_UCL	STRATUM
8016	0	1.50	1	0.89517	.	.	1
8017	0	1.50	1	0.89517	.	.	1
8018	0	1.50	1	0.89517	.	.	1
8019	0	1.50	1	0.89517	.	.	1
8020	0	1.50	1	0.89517	.	.	1
8021	0	1.50	1	0.89517	.	.	1
8022	0	1.50	1	0.89517	.	.	1
8023	0	1.50	1	0.89517	.	.	1
8024	0	1.50	1	0.89517	.	.	1
8025	0	1.50	1	0.89517	.	.	1
8026	0	1.50	1	0.89517	.	.	1
8027	0	1.50	1	0.89517	.	.	1
8028	0	1.50	1	0.89517	.	.	1
8029	0	1.50	1	0.89517	.	.	1
8030	0	1.50	1	0.89517	.	.	1
8031	0	1.51	0	0.89482	0.89191	0.89766	1
8032	0	1.51	1	0.89482	.	.	1
8033	0	1.51	1	0.89482	.	.	1
8034	0	1.51	1	0.89482	.	.	1
8035	0	1.51	1	0.89482	.	.	1
8036	0	1.51	1	0.89482	.	.	1
8037	0	1.51	1	0.89482	.	.	1
8038	0	1.51	1	0.89482	.	.	1
8039	0	1.51	1	0.89482	.	.	1
8040	0	1.51	1	0.89482	.	.	1
8041	0	1.51	1	0.89482	.	.	1
8042	0	1.51	1	0.89482	.	.	1
8043	0	1.51	1	0.89482	.	.	1
8044	0	1.51	1	0.89482	.	.	1
8045	0	1.51	1	0.89482	.	.	1
8046	0	1.51	1	0.89482	.	.	1
8047	0	1.51	1	0.89482	.	.	1
8048	0	1.51	1	0.89482	.	.	1
8049	0	1.51	1	0.89482	.	.	1
8050	0	1.51	1	0.89482	.	.	1

Obs	Sub_group	fuptimey	_CENSOR_	SURVIVAL	SDF_LCL	SDF_UCL	STRATUM
8051	0	1.51	1	0.89482	.	.	1
8052	0	1.51	1	0.89482	.	.	1
8053	0	1.51	1	0.89482	.	.	1
8054	0	1.51	1	0.89482	.	.	1
8055	0	1.51	1	0.89482	.	.	1
8056	0	1.51	1	0.89482	.	.	1
8057	0	1.51	1	0.89482	.	.	1
8058	0	1.51	1	0.89482	.	.	1
8059	0	1.51	1	0.89482	.	.	1
8060	0	1.51	1	0.89482	.	.	1
8061	0	1.51	1	0.89482	.	.	1
8062	0	1.51	1	0.89482	.	.	1
8063	0	1.51	1	0.89482	.	.	1
8064	0	1.51	1	0.89482	.	.	1
8065	0	1.51	1	0.89482	.	.	1
8066	0	1.51	1	0.89482	.	.	1
8067	0	1.51	1	0.89482	.	.	1
8068	0	1.51	1	0.89482	.	.	1
8069	0	1.51	1	0.89482	.	.	1
8070	0	1.51	1	0.89482	.	.	1
8071	0	1.51	1	0.89482	.	.	1
8072	0	1.51	1	0.89482	.	.	1
8073	0	1.51	1	0.89482	.	.	1
8074	0	1.52	0	0.89448	0.89157	0.89733	1
8075	0	1.52	1	0.89448	.	.	1
8076	0	1.52	1	0.89448	.	.	1
8077	0	1.52	1	0.89448	.	.	1
8078	0	1.52	1	0.89448	.	.	1
8079	0	1.52	1	0.89448	.	.	1
8080	0	1.52	1	0.89448	.	.	1
8081	0	1.52	1	0.89448	.	.	1
8082	0	1.52	1	0.89448	.	.	1
8083	0	1.52	1	0.89448	.	.	1
8084	0	1.52	1	0.89448	.	.	1
8085	0	1.52	1	0.89448	.	.	1

Obs	Sub_group	fuptimey	_CENSOR_	SURVIVAL	SDF_LCL	SDF_UCL	STRATUM
8086	0	1.52	1	0.89448	.	.	1
8087	0	1.52	1	0.89448	.	.	1
8088	0	1.52	1	0.89448	.	.	1
8089	0	1.52	1	0.89448	.	.	1
8090	0	1.52	1	0.89448	.	.	1
8091	0	1.52	1	0.89448	.	.	1
8092	0	1.52	1	0.89448	.	.	1
8093	0	1.52	1	0.89448	.	.	1
8094	0	1.52	1	0.89448	.	.	1
8095	0	1.52	1	0.89448	.	.	1
8096	0	1.52	1	0.89448	.	.	1
8097	0	1.52	1	0.89448	.	.	1
8098	0	1.52	1	0.89448	.	.	1
8099	0	1.52	1	0.89448	.	.	1
8100	0	1.52	1	0.89448	.	.	1
8101	0	1.52	1	0.89448	.	.	1
8102	0	1.52	1	0.89448	.	.	1
8103	0	1.52	1	0.89448	.	.	1
8104	0	1.52	1	0.89448	.	.	1
8105	0	1.52	1	0.89448	.	.	1
8106	0	1.52	1	0.89448	.	.	1
8107	0	1.52	1	0.89448	.	.	1
8108	0	1.52	1	0.89448	.	.	1
8109	0	1.52	1	0.89448	.	.	1
8110	0	1.52	1	0.89448	.	.	1
8111	0	1.52	1	0.89448	.	.	1
8112	0	1.52	1	0.89448	.	.	1
8113	0	1.52	1	0.89448	.	.	1
8114	0	1.52	1	0.89448	.	.	1
8115	0	1.52	1	0.89448	.	.	1
8116	0	1.52	1	0.89448	.	.	1
8117	0	1.52	1	0.89448	.	.	1
8118	0	1.52	1	0.89448	.	.	1
8119	0	1.52	1	0.89448	.	.	1
8120	0	1.52	1	0.89448	.	.	1

Obs	Sub_group	fuptimey	_CENSOR_	SURVIVAL	SDF_LCL	SDF_UCL	STRATUM
**8121**	0	1.52	1	0.89448	.	.	1
**8122**	0	1.52	1	0.89448	.	.	1
**8123**	0	1.52	1	0.89448	.	.	1
**8124**	0	1.52	1	0.89448	.	.	1
**8125**	0	1.52	1	0.89448	.	.	1
**8126**	0	1.52	1	0.89448	.	.	1
**8127**	0	1.52	1	0.89448	.	.	1
**8128**	0	1.52	1	0.89448	.	.	1
**8129**	0	1.52	1	0.89448	.	.	1
**8130**	0	1.52	1	0.89448	.	.	1
**8131**	0	1.52	1	0.89448	.	.	1
**8132**	0	1.52	1	0.89448	.	.	1
**8133**	0	1.52	1	0.89448	.	.	1
**8134**	0	1.52	1	0.89448	.	.	1
**8135**	0	1.52	1	0.89448	.	.	1
**8136**	0	1.52	1	0.89448	.	.	1
**8137**	0	1.53	0	0.89419	0.89127	0.89704	1
**8138**	0	1.53	1	0.89419	.	.	1
**8139**	0	1.53	1	0.89419	.	.	1
**8140**	0	1.53	1	0.89419	.	.	1
**8141**	0	1.53	1	0.89419	.	.	1
**8142**	0	1.53	1	0.89419	.	.	1
**8143**	0	1.53	1	0.89419	.	.	1
**8144**	0	1.53	1	0.89419	.	.	1
**8145**	0	1.53	1	0.89419	.	.	1
**8146**	0	1.53	1	0.89419	.	.	1
**8147**	0	1.53	1	0.89419	.	.	1
**8148**	0	1.53	1	0.89419	.	.	1
**8149**	0	1.53	1	0.89419	.	.	1
**8150**	0	1.53	1	0.89419	.	.	1
**8151**	0	1.53	1	0.89419	.	.	1
**8152**	0	1.53	1	0.89419	.	.	1
**8153**	0	1.53	1	0.89419	.	.	1
**8154**	0	1.53	1	0.89419	.	.	1
**8155**	0	1.53	1	0.89419	.	.	1

Obs	Sub_group	fuptimey	_CENSOR_	SURVIVAL	SDF_LCL	SDF_UCL	STRATUM
8156	0	1.53	1	0.89419	.	.	1
8157	0	1.53	1	0.89419	.	.	1
8158	0	1.53	1	0.89419	.	.	1
8159	0	1.53	1	0.89419	.	.	1
8160	0	1.53	1	0.89419	.	.	1
8161	0	1.53	1	0.89419	.	.	1
8162	0	1.53	1	0.89419	.	.	1
8163	0	1.53	1	0.89419	.	.	1
8164	0	1.53	1	0.89419	.	.	1
8165	0	1.53	1	0.89419	.	.	1
8166	0	1.53	1	0.89419	.	.	1
8167	0	1.53	1	0.89419	.	.	1
8168	0	1.53	1	0.89419	.	.	1
8169	0	1.53	1	0.89419	.	.	1
8170	0	1.53	1	0.89419	.	.	1
8171	0	1.53	1	0.89419	.	.	1
8172	0	1.53	1	0.89419	.	.	1
8173	0	1.53	1	0.89419	.	.	1
8174	0	1.53	1	0.89419	.	.	1
8175	0	1.53	1	0.89419	.	.	1
8176	0	1.53	1	0.89419	.	.	1
8177	0	1.53	1	0.89419	.	.	1
8178	0	1.53	1	0.89419	.	.	1
8179	0	1.53	1	0.89419	.	.	1
8180	0	1.53	1	0.89419	.	.	1
8181	0	1.53	1	0.89419	.	.	1
8182	0	1.53	1	0.89419	.	.	1
8183	0	1.53	1	0.89419	.	.	1
8184	0	1.53	1	0.89419	.	.	1
8185	0	1.53	1	0.89419	.	.	1
8186	0	1.53	1	0.89419	.	.	1
8187	0	1.54	0	0.89409	0.89117	0.89694	1
8188	0	1.54	1	0.89409	.	.	1
8189	0	1.54	1	0.89409	.	.	1
8190	0	1.54	1	0.89409	.	.	1

Obs	Sub_group	fuptimey	_CENSOR_	SURVIVAL	SDF_LCL	SDF_UCL	STRATUM
8191	0	1.54	1	0.89409	.	.	1
8192	0	1.54	1	0.89409	.	.	1
8193	0	1.54	1	0.89409	.	.	1
8194	0	1.54	1	0.89409	.	.	1
8195	0	1.54	1	0.89409	.	.	1
8196	0	1.54	1	0.89409	.	.	1
8197	0	1.54	1	0.89409	.	.	1
8198	0	1.54	1	0.89409	.	.	1
8199	0	1.54	1	0.89409	.	.	1
8200	0	1.54	1	0.89409	.	.	1
8201	0	1.54	1	0.89409	.	.	1
8202	0	1.54	1	0.89409	.	.	1
8203	0	1.54	1	0.89409	.	.	1
8204	0	1.54	1	0.89409	.	.	1
8205	0	1.54	1	0.89409	.	.	1
8206	0	1.54	1	0.89409	.	.	1
8207	0	1.54	1	0.89409	.	.	1
8208	0	1.54	1	0.89409	.	.	1
8209	0	1.54	1	0.89409	.	.	1
8210	0	1.54	1	0.89409	.	.	1
8211	0	1.54	1	0.89409	.	.	1
8212	0	1.54	1	0.89409	.	.	1
8213	0	1.54	1	0.89409	.	.	1
8214	0	1.54	1	0.89409	.	.	1
8215	0	1.54	1	0.89409	.	.	1
8216	0	1.54	1	0.89409	.	.	1
8217	0	1.54	1	0.89409	.	.	1
8218	0	1.54	1	0.89409	.	.	1
8219	0	1.54	1	0.89409	.	.	1
8220	0	1.54	1	0.89409	.	.	1
8221	0	1.54	1	0.89409	.	.	1
8222	0	1.54	1	0.89409	.	.	1
8223	0	1.54	1	0.89409	.	.	1
8224	0	1.54	1	0.89409	.	.	1
8225	0	1.54	1	0.89409	.	.	1

Obs	Sub_group	fuptimey	_CENSOR_	SURVIVAL	SDF_LCL	SDF_UCL	STRATUM
8226	0	1.54	1	0.89409	.	.	1
8227	0	1.54	1	0.89409	.	.	1
8228	0	1.54	1	0.89409	.	.	1
8229	0	1.54	1	0.89409	.	.	1
8230	0	1.54	1	0.89409	.	.	1
8231	0	1.54	1	0.89409	.	.	1
8232	0	1.54	1	0.89409	.	.	1
8233	0	1.54	1	0.89409	.	.	1
8234	0	1.54	1	0.89409	.	.	1
8235	0	1.54	1	0.89409	.	.	1
8236	0	1.54	1	0.89409	.	.	1
8237	0	1.54	1	0.89409	.	.	1
8238	0	1.55	0	0.89383	0.89090	0.89668	1
8239	0	1.55	1	0.89383	.	.	1
8240	0	1.55	1	0.89383	.	.	1
8241	0	1.55	1	0.89383	.	.	1
8242	0	1.55	1	0.89383	.	.	1
8243	0	1.55	1	0.89383	.	.	1
8244	0	1.55	1	0.89383	.	.	1
8245	0	1.55	1	0.89383	.	.	1
8246	0	1.55	1	0.89383	.	.	1
8247	0	1.55	1	0.89383	.	.	1
8248	0	1.55	1	0.89383	.	.	1
8249	0	1.55	1	0.89383	.	.	1
8250	0	1.55	1	0.89383	.	.	1
8251	0	1.55	1	0.89383	.	.	1
8252	0	1.55	1	0.89383	.	.	1
8253	0	1.55	1	0.89383	.	.	1
8254	0	1.55	1	0.89383	.	.	1
8255	0	1.55	1	0.89383	.	.	1
8256	0	1.55	1	0.89383	.	.	1
8257	0	1.55	1	0.89383	.	.	1
8258	0	1.55	1	0.89383	.	.	1
8259	0	1.55	1	0.89383	.	.	1
8260	0	1.55	1	0.89383	.	.	1

Obs	Sub_group	fuptimey	_CENSOR_	SURVIVAL	SDF_LCL	SDF_UCL	STRATUM
8261	0	1.55	1	0.89383	.	.	1
8262	0	1.55	1	0.89383	.	.	1
8263	0	1.55	1	0.89383	.	.	1
8264	0	1.55	1	0.89383	.	.	1
8265	0	1.55	1	0.89383	.	.	1
8266	0	1.55	1	0.89383	.	.	1
8267	0	1.55	1	0.89383	.	.	1
8268	0	1.55	1	0.89383	.	.	1
8269	0	1.55	1	0.89383	.	.	1
8270	0	1.55	1	0.89383	.	.	1
8271	0	1.55	1	0.89383	.	.	1
8272	0	1.55	1	0.89383	.	.	1
8273	0	1.55	1	0.89383	.	.	1
8274	0	1.55	1	0.89383	.	.	1
8275	0	1.55	1	0.89383	.	.	1
8276	0	1.55	1	0.89383	.	.	1
8277	0	1.55	1	0.89383	.	.	1
8278	0	1.55	1	0.89383	.	.	1
8279	0	1.55	1	0.89383	.	.	1
8280	0	1.55	1	0.89383	.	.	1
8281	0	1.55	1	0.89383	.	.	1
8282	0	1.55	1	0.89383	.	.	1
8283	0	1.55	1	0.89383	.	.	1
8284	0	1.55	1	0.89383	.	.	1
8285	0	1.55	1	0.89383	.	.	1
8286	0	1.55	1	0.89383	.	.	1
8287	0	1.55	1	0.89383	.	.	1
8288	0	1.55	1	0.89383	.	.	1
8289	0	1.55	1	0.89383	.	.	1
8290	0	1.56	0	0.89346	0.89052	0.89631	1
8291	0	1.56	1	0.89346	.	.	1
8292	0	1.56	1	0.89346	.	.	1
8293	0	1.56	1	0.89346	.	.	1
8294	0	1.56	1	0.89346	.	.	1
8295	0	1.56	1	0.89346	.	.	1

Obs	Sub_group	fuptimey	_CENSOR_	SURVIVAL	SDF_LCL	SDF_UCL	STRATUM
8296	0	1.56	1	0.89346	.	.	1
8297	0	1.56	1	0.89346	.	.	1
8298	0	1.56	1	0.89346	.	.	1
8299	0	1.56	1	0.89346	.	.	1
8300	0	1.56	1	0.89346	.	.	1
8301	0	1.56	1	0.89346	.	.	1
8302	0	1.56	1	0.89346	.	.	1
8303	0	1.56	1	0.89346	.	.	1
8304	0	1.56	1	0.89346	.	.	1
8305	0	1.56	1	0.89346	.	.	1
8306	0	1.56	1	0.89346	.	.	1
8307	0	1.56	1	0.89346	.	.	1
8308	0	1.56	1	0.89346	.	.	1
8309	0	1.56	1	0.89346	.	.	1
8310	0	1.56	1	0.89346	.	.	1
8311	0	1.56	1	0.89346	.	.	1
8312	0	1.56	1	0.89346	.	.	1
8313	0	1.56	1	0.89346	.	.	1
8314	0	1.56	1	0.89346	.	.	1
8315	0	1.56	1	0.89346	.	.	1
8316	0	1.56	1	0.89346	.	.	1
8317	0	1.56	1	0.89346	.	.	1
8318	0	1.56	1	0.89346	.	.	1
8319	0	1.56	1	0.89346	.	.	1
8320	0	1.56	1	0.89346	.	.	1
8321	0	1.56	1	0.89346	.	.	1
8322	0	1.56	1	0.89346	.	.	1
8323	0	1.56	1	0.89346	.	.	1
8324	0	1.56	1	0.89346	.	.	1
8325	0	1.56	1	0.89346	.	.	1
8326	0	1.56	1	0.89346	.	.	1
8327	0	1.56	1	0.89346	.	.	1
8328	0	1.56	1	0.89346	.	.	1
8329	0	1.56	1	0.89346	.	.	1
8330	0	1.56	1	0.89346	.	.	1

Obs	Sub_group	fuptimey	_CENSOR_	SURVIVAL	SDF_LCL	SDF_UCL	STRATUM
**8331**	0	1.56	1	0.89346	.	.	1
**8332**	0	1.56	1	0.89346	.	.	1
**8333**	0	1.56	1	0.89346	.	.	1
**8334**	0	1.56	1	0.89346	.	.	1
**8335**	0	1.56	1	0.89346	.	.	1
**8336**	0	1.56	1	0.89346	.	.	1
**8337**	0	1.56	1	0.89346	.	.	1
**8338**	0	1.56	1	0.89346	.	.	1
**8339**	0	1.56	1	0.89346	.	.	1
**8340**	0	1.56	1	0.89346	.	.	1
**8341**	0	1.56	1	0.89346	.	.	1
**8342**	0	1.56	1	0.89346	.	.	1
**8343**	0	1.56	1	0.89346	.	.	1
**8344**	0	1.56	1	0.89346	.	.	1
**8345**	0	1.56	1	0.89346	.	.	1
**8346**	0	1.56	1	0.89346	.	.	1
**8347**	0	1.56	1	0.89346	.	.	1
**8348**	0	1.56	1	0.89346	.	.	1
**8349**	0	1.56	1	0.89346	.	.	1
**8350**	0	1.56	1	0.89346	.	.	1
**8351**	0	1.56	1	0.89346	.	.	1
**8352**	0	1.56	1	0.89346	.	.	1
**8353**	0	1.56	1	0.89346	.	.	1
**8354**	0	1.56	1	0.89346	.	.	1
**8355**	0	1.56	1	0.89346	.	.	1
**8356**	0	1.56	1	0.89346	.	.	1
**8357**	0	1.56	1	0.89346	.	.	1
**8358**	0	1.56	1	0.89346	.	.	1
**8359**	0	1.56	1	0.89346	.	.	1
**8360**	0	1.56	1	0.89346	.	.	1
**8361**	0	1.56	1	0.89346	.	.	1
**8362**	0	1.57	0	0.89314	0.89020	0.89600	1
**8363**	0	1.57	1	0.89314	.	.	1
**8364**	0	1.57	1	0.89314	.	.	1
**8365**	0	1.57	1	0.89314	.	.	1

Obs	Sub_group	fuptimey	_CENSOR_	SURVIVAL	SDF_LCL	SDF_UCL	STRATUM
8366	0	1.57	1	0.89314	.	.	1
8367	0	1.57	1	0.89314	.	.	1
8368	0	1.57	1	0.89314	.	.	1
8369	0	1.57	1	0.89314	.	.	1
8370	0	1.57	1	0.89314	.	.	1
8371	0	1.57	1	0.89314	.	.	1
8372	0	1.57	1	0.89314	.	.	1
8373	0	1.57	1	0.89314	.	.	1
8374	0	1.57	1	0.89314	.	.	1
8375	0	1.57	1	0.89314	.	.	1
8376	0	1.57	1	0.89314	.	.	1
8377	0	1.57	1	0.89314	.	.	1
8378	0	1.57	1	0.89314	.	.	1
8379	0	1.57	1	0.89314	.	.	1
8380	0	1.57	1	0.89314	.	.	1
8381	0	1.57	1	0.89314	.	.	1
8382	0	1.57	1	0.89314	.	.	1
8383	0	1.57	1	0.89314	.	.	1
8384	0	1.57	1	0.89314	.	.	1
8385	0	1.57	1	0.89314	.	.	1
8386	0	1.57	1	0.89314	.	.	1
8387	0	1.57	1	0.89314	.	.	1
8388	0	1.57	1	0.89314	.	.	1
8389	0	1.57	1	0.89314	.	.	1
8390	0	1.57	1	0.89314	.	.	1
8391	0	1.57	1	0.89314	.	.	1
8392	0	1.57	1	0.89314	.	.	1
8393	0	1.57	1	0.89314	.	.	1
8394	0	1.57	1	0.89314	.	.	1
8395	0	1.57	1	0.89314	.	.	1
8396	0	1.57	1	0.89314	.	.	1
8397	0	1.57	1	0.89314	.	.	1
8398	0	1.57	1	0.89314	.	.	1
8399	0	1.58	0	0.89279	0.88985	0.89566	1
8400	0	1.58	1	0.89279	.	.	1

Obs	Sub_group	fuptimey	_CENSOR_	SURVIVAL	SDF_LCL	SDF_UCL	STRATUM
8401	0	1.58	1	0.89279	.	.	1
8402	0	1.58	1	0.89279	.	.	1
8403	0	1.58	1	0.89279	.	.	1
8404	0	1.58	1	0.89279	.	.	1
8405	0	1.58	1	0.89279	.	.	1
8406	0	1.58	1	0.89279	.	.	1
8407	0	1.58	1	0.89279	.	.	1
8408	0	1.58	1	0.89279	.	.	1
8409	0	1.58	1	0.89279	.	.	1
8410	0	1.58	1	0.89279	.	.	1
8411	0	1.58	1	0.89279	.	.	1
8412	0	1.58	1	0.89279	.	.	1
8413	0	1.58	1	0.89279	.	.	1
8414	0	1.58	1	0.89279	.	.	1
8415	0	1.58	1	0.89279	.	.	1
8416	0	1.58	1	0.89279	.	.	1
8417	0	1.58	1	0.89279	.	.	1
8418	0	1.58	1	0.89279	.	.	1
8419	0	1.58	1	0.89279	.	.	1
8420	0	1.58	1	0.89279	.	.	1
8421	0	1.58	1	0.89279	.	.	1
8422	0	1.58	1	0.89279	.	.	1
8423	0	1.58	1	0.89279	.	.	1
8424	0	1.58	1	0.89279	.	.	1
8425	0	1.58	1	0.89279	.	.	1
8426	0	1.58	1	0.89279	.	.	1
8427	0	1.58	1	0.89279	.	.	1
8428	0	1.58	1	0.89279	.	.	1
8429	0	1.58	1	0.89279	.	.	1
8430	0	1.58	1	0.89279	.	.	1
8431	0	1.58	1	0.89279	.	.	1
8432	0	1.58	1	0.89279	.	.	1
8433	0	1.58	1	0.89279	.	.	1
8434	0	1.58	1	0.89279	.	.	1
8435	0	1.58	1	0.89279	.	.	1

Obs	Sub_group	fuptimey	_CENSOR_	SURVIVAL	SDF_LCL	SDF_UCL	STRATUM
**8436**	0	1.58	1	0.89279	.	.	1
**8437**	0	1.58	1	0.89279	.	.	1
**8438**	0	1.58	1	0.89279	.	.	1
**8439**	0	1.58	1	0.89279	.	.	1
**8440**	0	1.58	1	0.89279	.	.	1
**8441**	0	1.58	1	0.89279	.	.	1
**8442**	0	1.58	1	0.89279	.	.	1
**8443**	0	1.58	1	0.89279	.	.	1
**8444**	0	1.58	1	0.89279	.	.	1
**8445**	0	1.58	1	0.89279	.	.	1
**8446**	0	1.58	1	0.89279	.	.	1
**8447**	0	1.58	1	0.89279	.	.	1
**8448**	0	1.58	1	0.89279	.	.	1
**8449**	0	1.58	1	0.89279	.	.	1
**8450**	0	1.58	1	0.89279	.	.	1
**8451**	0	1.58	1	0.89279	.	.	1
**8452**	0	1.58	1	0.89279	.	.	1
**8453**	0	1.58	1	0.89279	.	.	1
**8454**	0	1.58	1	0.89279	.	.	1
**8455**	0	1.58	1	0.89279	.	.	1
**8456**	0	1.58	1	0.89279	.	.	1
**8457**	0	1.58	1	0.89279	.	.	1
**8458**	0	1.58	1	0.89279	.	.	1
**8459**	0	1.58	1	0.89279	.	.	1
**8460**	0	1.58	1	0.89279	.	.	1
**8461**	0	1.59	0	0.89248	0.88953	0.89535	1
**8462**	0	1.59	1	0.89248	.	.	1
**8463**	0	1.59	1	0.89248	.	.	1
**8464**	0	1.59	1	0.89248	.	.	1
**8465**	0	1.59	1	0.89248	.	.	1
**8466**	0	1.59	1	0.89248	.	.	1
**8467**	0	1.59	1	0.89248	.	.	1
**8468**	0	1.59	1	0.89248	.	.	1
**8469**	0	1.59	1	0.89248	.	.	1
**8470**	0	1.59	1	0.89248	.	.	1

Obs	Sub_group	fuptimey	_CENSOR_	SURVIVAL	SDF_LCL	SDF_UCL	STRATUM
**8471**	0	1.59	1	0.89248	.	.	1
**8472**	0	1.59	1	0.89248	.	.	1
**8473**	0	1.59	1	0.89248	.	.	1
**8474**	0	1.59	1	0.89248	.	.	1
**8475**	0	1.59	1	0.89248	.	.	1
**8476**	0	1.59	1	0.89248	.	.	1
**8477**	0	1.59	1	0.89248	.	.	1
**8478**	0	1.59	1	0.89248	.	.	1
**8479**	0	1.59	1	0.89248	.	.	1
**8480**	0	1.59	1	0.89248	.	.	1
**8481**	0	1.59	1	0.89248	.	.	1
**8482**	0	1.59	1	0.89248	.	.	1
**8483**	0	1.59	1	0.89248	.	.	1
**8484**	0	1.59	1	0.89248	.	.	1
**8485**	0	1.59	1	0.89248	.	.	1
**8486**	0	1.59	1	0.89248	.	.	1
**8487**	0	1.59	1	0.89248	.	.	1
**8488**	0	1.59	1	0.89248	.	.	1
**8489**	0	1.59	1	0.89248	.	.	1
**8490**	0	1.59	1	0.89248	.	.	1
**8491**	0	1.59	1	0.89248	.	.	1
**8492**	0	1.59	1	0.89248	.	.	1
**8493**	0	1.59	1	0.89248	.	.	1
**8494**	0	1.59	1	0.89248	.	.	1
**8495**	0	1.59	1	0.89248	.	.	1
**8496**	0	1.59	1	0.89248	.	.	1
**8497**	0	1.59	1	0.89248	.	.	1
**8498**	0	1.59	1	0.89248	.	.	1
**8499**	0	1.59	1	0.89248	.	.	1
**8500**	0	1.59	1	0.89248	.	.	1
**8501**	0	1.59	1	0.89248	.	.	1
**8502**	0	1.60	0	0.89218	0.88923	0.89506	1
**8503**	0	1.60	1	0.89218	.	.	1
**8504**	0	1.60	1	0.89218	.	.	1
**8505**	0	1.60	1	0.89218	.	.	1

Obs	Sub_group	fuptimey	_CENSOR_	SURVIVAL	SDF_LCL	SDF_UCL	STRATUM
**8506**	0	1.60	1	0.89218	.	.	1
**8507**	0	1.60	1	0.89218	.	.	1
**8508**	0	1.60	1	0.89218	.	.	1
**8509**	0	1.60	1	0.89218	.	.	1
**8510**	0	1.60	1	0.89218	.	.	1
**8511**	0	1.60	1	0.89218	.	.	1
**8512**	0	1.60	1	0.89218	.	.	1
**8513**	0	1.60	1	0.89218	.	.	1
**8514**	0	1.60	1	0.89218	.	.	1
**8515**	0	1.60	1	0.89218	.	.	1
**8516**	0	1.60	1	0.89218	.	.	1
**8517**	0	1.60	1	0.89218	.	.	1
**8518**	0	1.60	1	0.89218	.	.	1
**8519**	0	1.60	1	0.89218	.	.	1
**8520**	0	1.60	1	0.89218	.	.	1
**8521**	0	1.60	1	0.89218	.	.	1
**8522**	0	1.60	1	0.89218	.	.	1
**8523**	0	1.60	1	0.89218	.	.	1
**8524**	0	1.60	1	0.89218	.	.	1
**8525**	0	1.60	1	0.89218	.	.	1
**8526**	0	1.60	1	0.89218	.	.	1
**8527**	0	1.60	1	0.89218	.	.	1
**8528**	0	1.60	1	0.89218	.	.	1
**8529**	0	1.60	1	0.89218	.	.	1
**8530**	0	1.60	1	0.89218	.	.	1
**8531**	0	1.60	1	0.89218	.	.	1
**8532**	0	1.60	1	0.89218	.	.	1
**8533**	0	1.60	1	0.89218	.	.	1
**8534**	0	1.60	1	0.89218	.	.	1
**8535**	0	1.60	1	0.89218	.	.	1
**8536**	0	1.60	1	0.89218	.	.	1
**8537**	0	1.60	1	0.89218	.	.	1
**8538**	0	1.60	1	0.89218	.	.	1
**8539**	0	1.60	1	0.89218	.	.	1
**8540**	0	1.60	1	0.89218	.	.	1

Obs	Sub_group	fuptimey	_CENSOR_	SURVIVAL	SDF_LCL	SDF_UCL	STRATUM
8541	0	1.60	1	0.89218	.	.	1
8542	0	1.60	1	0.89218	.	.	1
8543	0	1.60	1	0.89218	.	.	1
8544	0	1.60	1	0.89218	.	.	1
8545	0	1.60	1	0.89218	.	.	1
8546	0	1.60	1	0.89218	.	.	1
8547	0	1.60	1	0.89218	.	.	1
8548	0	1.60	1	0.89218	.	.	1
8549	0	1.60	1	0.89218	.	.	1
8550	0	1.60	1	0.89218	.	.	1
8551	0	1.60	1	0.89218	.	.	1
8552	0	1.60	1	0.89218	.	.	1
8553	0	1.60	1	0.89218	.	.	1
8554	0	1.60	1	0.89218	.	.	1
8555	0	1.60	1	0.89218	.	.	1
8556	0	1.60	1	0.89218	.	.	1
8557	0	1.60	1	0.89218	.	.	1
8558	0	1.60	1	0.89218	.	.	1
8559	0	1.60	1	0.89218	.	.	1
8560	0	1.60	1	0.89218	.	.	1
8561	0	1.60	1	0.89218	.	.	1
8562	0	1.60	1	0.89218	.	.	1
8563	0	1.60	1	0.89218	.	.	1
8564	0	1.60	1	0.89218	.	.	1
8565	0	1.60	1	0.89218	.	.	1
8566	0	1.60	1	0.89218	.	.	1
8567	0	1.60	1	0.89218	.	.	1
8568	0	1.60	1	0.89218	.	.	1
8569	0	1.60	1	0.89218	.	.	1
8570	0	1.60	1	0.89218	.	.	1
8571	0	1.60	1	0.89218	.	.	1
8572	0	1.61	0	0.89160	0.88864	0.89448	1
8573	0	1.61	1	0.89160	.	.	1
8574	0	1.61	1	0.89160	.	.	1
8575	0	1.61	1	0.89160	.	.	1

Obs	Sub_group	fuptimey	_CENSOR_	SURVIVAL	SDF_LCL	SDF_UCL	STRATUM
**8576**	0	1.61	1	0.89160	.	.	1
**8577**	0	1.61	1	0.89160	.	.	1
**8578**	0	1.61	1	0.89160	.	.	1
**8579**	0	1.61	1	0.89160	.	.	1
**8580**	0	1.61	1	0.89160	.	.	1
**8581**	0	1.61	1	0.89160	.	.	1
**8582**	0	1.61	1	0.89160	.	.	1
**8583**	0	1.61	1	0.89160	.	.	1
**8584**	0	1.61	1	0.89160	.	.	1
**8585**	0	1.61	1	0.89160	.	.	1
**8586**	0	1.61	1	0.89160	.	.	1
**8587**	0	1.61	1	0.89160	.	.	1
**8588**	0	1.61	1	0.89160	.	.	1
**8589**	0	1.61	1	0.89160	.	.	1
**8590**	0	1.61	1	0.89160	.	.	1
**8591**	0	1.61	1	0.89160	.	.	1
**8592**	0	1.61	1	0.89160	.	.	1
**8593**	0	1.61	1	0.89160	.	.	1
**8594**	0	1.61	1	0.89160	.	.	1
**8595**	0	1.61	1	0.89160	.	.	1
**8596**	0	1.61	1	0.89160	.	.	1
**8597**	0	1.61	1	0.89160	.	.	1
**8598**	0	1.61	1	0.89160	.	.	1
**8599**	0	1.61	1	0.89160	.	.	1
**8600**	0	1.61	1	0.89160	.	.	1
**8601**	0	1.61	1	0.89160	.	.	1
**8602**	0	1.61	1	0.89160	.	.	1
**8603**	0	1.61	1	0.89160	.	.	1
**8604**	0	1.61	1	0.89160	.	.	1
**8605**	0	1.61	1	0.89160	.	.	1
**8606**	0	1.61	1	0.89160	.	.	1
**8607**	0	1.61	1	0.89160	.	.	1
**8608**	0	1.61	1	0.89160	.	.	1
**8609**	0	1.61	1	0.89160	.	.	1
**8610**	0	1.61	1	0.89160	.	.	1

Obs	Sub_group	fuptimey	_CENSOR_	SURVIVAL	SDF_LCL	SDF_UCL	STRATUM
8611	0	1.61	1	0.89160	.	.	1
8612	0	1.62	0	0.89125	0.88829	0.89414	1
8613	0	1.62	1	0.89125	.	.	1
8614	0	1.62	1	0.89125	.	.	1
8615	0	1.62	1	0.89125	.	.	1
8616	0	1.62	1	0.89125	.	.	1
8617	0	1.62	1	0.89125	.	.	1
8618	0	1.62	1	0.89125	.	.	1
8619	0	1.62	1	0.89125	.	.	1
8620	0	1.62	1	0.89125	.	.	1
8621	0	1.62	1	0.89125	.	.	1
8622	0	1.62	1	0.89125	.	.	1
8623	0	1.62	1	0.89125	.	.	1
8624	0	1.62	1	0.89125	.	.	1
8625	0	1.62	1	0.89125	.	.	1
8626	0	1.62	1	0.89125	.	.	1
8627	0	1.62	1	0.89125	.	.	1
8628	0	1.62	1	0.89125	.	.	1
8629	0	1.62	1	0.89125	.	.	1
8630	0	1.62	1	0.89125	.	.	1
8631	0	1.62	1	0.89125	.	.	1
8632	0	1.62	1	0.89125	.	.	1
8633	0	1.62	1	0.89125	.	.	1
8634	0	1.62	1	0.89125	.	.	1
8635	0	1.62	1	0.89125	.	.	1
8636	0	1.62	1	0.89125	.	.	1
8637	0	1.62	1	0.89125	.	.	1
8638	0	1.62	1	0.89125	.	.	1
8639	0	1.62	1	0.89125	.	.	1
8640	0	1.62	1	0.89125	.	.	1
8641	0	1.62	1	0.89125	.	.	1
8642	0	1.62	1	0.89125	.	.	1
8643	0	1.62	1	0.89125	.	.	1
8644	0	1.62	1	0.89125	.	.	1
8645	0	1.62	1	0.89125	.	.	1

Obs	Sub_group	fuptimey	_CENSOR_	SURVIVAL	SDF_LCL	SDF_UCL	STRATUM
8646	0	1.62	1	0.89125	.	.	1
8647	0	1.62	1	0.89125	.	.	1
8648	0	1.62	1	0.89125	.	.	1
8649	0	1.62	1	0.89125	.	.	1
8650	0	1.62	1	0.89125	.	.	1
8651	0	1.62	1	0.89125	.	.	1
8652	0	1.62	1	0.89125	.	.	1
8653	0	1.62	1	0.89125	.	.	1
8654	0	1.62	1	0.89125	.	.	1
8655	0	1.62	1	0.89125	.	.	1
8656	0	1.62	1	0.89125	.	.	1
8657	0	1.62	1	0.89125	.	.	1
8658	0	1.62	1	0.89125	.	.	1
8659	0	1.62	1	0.89125	.	.	1
8660	0	1.62	1	0.89125	.	.	1
8661	0	1.62	1	0.89125	.	.	1
8662	0	1.62	1	0.89125	.	.	1
8663	0	1.62	1	0.89125	.	.	1
8664	0	1.62	1	0.89125	.	.	1
8665	0	1.62	1	0.89125	.	.	1
8666	0	1.62	1	0.89125	.	.	1
8667	0	1.62	1	0.89125	.	.	1
8668	0	1.62	1	0.89125	.	.	1
8669	0	1.62	1	0.89125	.	.	1
8670	0	1.62	1	0.89125	.	.	1
8671	0	1.62	1	0.89125	.	.	1
8672	0	1.63	0	0.89098	0.88802	0.89388	1
8673	0	1.63	1	0.89098	.	.	1
8674	0	1.63	1	0.89098	.	.	1
8675	0	1.63	1	0.89098	.	.	1
8676	0	1.63	1	0.89098	.	.	1
8677	0	1.63	1	0.89098	.	.	1
8678	0	1.63	1	0.89098	.	.	1
8679	0	1.63	1	0.89098	.	.	1
8680	0	1.63	1	0.89098	.	.	1

Obs	Sub_group	fuptimey	_CENSOR_	SURVIVAL	SDF_LCL	SDF_UCL	STRATUM
8681	0	1.63	1	0.89098	.	.	1
8682	0	1.63	1	0.89098	.	.	1
8683	0	1.63	1	0.89098	.	.	1
8684	0	1.63	1	0.89098	.	.	1
8685	0	1.63	1	0.89098	.	.	1
8686	0	1.63	1	0.89098	.	.	1
8687	0	1.63	1	0.89098	.	.	1
8688	0	1.63	1	0.89098	.	.	1
8689	0	1.63	1	0.89098	.	.	1
8690	0	1.63	1	0.89098	.	.	1
8691	0	1.63	1	0.89098	.	.	1
8692	0	1.63	1	0.89098	.	.	1
8693	0	1.63	1	0.89098	.	.	1
8694	0	1.63	1	0.89098	.	.	1
8695	0	1.63	1	0.89098	.	.	1
8696	0	1.63	1	0.89098	.	.	1
8697	0	1.63	1	0.89098	.	.	1
8698	0	1.63	1	0.89098	.	.	1
8699	0	1.63	1	0.89098	.	.	1
8700	0	1.63	1	0.89098	.	.	1
8701	0	1.63	1	0.89098	.	.	1
8702	0	1.63	1	0.89098	.	.	1
8703	0	1.63	1	0.89098	.	.	1
8704	0	1.63	1	0.89098	.	.	1
8705	0	1.63	1	0.89098	.	.	1
8706	0	1.63	1	0.89098	.	.	1
8707	0	1.63	1	0.89098	.	.	1
8708	0	1.63	1	0.89098	.	.	1
8709	0	1.63	1	0.89098	.	.	1
8710	0	1.63	1	0.89098	.	.	1
8711	0	1.63	1	0.89098	.	.	1
8712	0	1.63	1	0.89098	.	.	1
8713	0	1.63	1	0.89098	.	.	1
8714	0	1.63	1	0.89098	.	.	1
8715	0	1.64	0	0.89053	0.88756	0.89343	1

Obs	Sub_group	fuptimey	_CENSOR_	SURVIVAL	SDF_LCL	SDF_UCL	STRATUM
**8716**	0	1.64	1	0.89053	.	.	1
**8717**	0	1.64	1	0.89053	.	.	1
**8718**	0	1.64	1	0.89053	.	.	1
**8719**	0	1.64	1	0.89053	.	.	1
**8720**	0	1.64	1	0.89053	.	.	1
**8721**	0	1.64	1	0.89053	.	.	1
**8722**	0	1.64	1	0.89053	.	.	1
**8723**	0	1.64	1	0.89053	.	.	1
**8724**	0	1.64	1	0.89053	.	.	1
**8725**	0	1.64	1	0.89053	.	.	1
**8726**	0	1.64	1	0.89053	.	.	1
**8727**	0	1.64	1	0.89053	.	.	1
**8728**	0	1.64	1	0.89053	.	.	1
**8729**	0	1.64	1	0.89053	.	.	1
**8730**	0	1.64	1	0.89053	.	.	1
**8731**	0	1.64	1	0.89053	.	.	1
**8732**	0	1.64	1	0.89053	.	.	1
**8733**	0	1.64	1	0.89053	.	.	1
**8734**	0	1.64	1	0.89053	.	.	1
**8735**	0	1.64	1	0.89053	.	.	1
**8736**	0	1.64	1	0.89053	.	.	1
**8737**	0	1.64	1	0.89053	.	.	1
**8738**	0	1.64	1	0.89053	.	.	1
**8739**	0	1.64	1	0.89053	.	.	1
**8740**	0	1.64	1	0.89053	.	.	1
**8741**	0	1.64	1	0.89053	.	.	1
**8742**	0	1.64	1	0.89053	.	.	1
**8743**	0	1.64	1	0.89053	.	.	1
**8744**	0	1.64	1	0.89053	.	.	1
**8745**	0	1.64	1	0.89053	.	.	1
**8746**	0	1.64	1	0.89053	.	.	1
**8747**	0	1.64	1	0.89053	.	.	1
**8748**	0	1.64	1	0.89053	.	.	1
**8749**	0	1.64	1	0.89053	.	.	1
**8750**	0	1.64	1	0.89053	.	.	1

Obs	Sub_group	fuptimey	_CENSOR_	SURVIVAL	SDF_LCL	SDF_UCL	STRATUM
**8751**	0	1.64	1	0.89053	.	.	1
**8752**	0	1.64	1	0.89053	.	.	1
**8753**	0	1.64	1	0.89053	.	.	1
**8754**	0	1.64	1	0.89053	.	.	1
**8755**	0	1.64	1	0.89053	.	.	1
**8756**	0	1.64	1	0.89053	.	.	1
**8757**	0	1.64	1	0.89053	.	.	1
**8758**	0	1.64	1	0.89053	.	.	1
**8759**	0	1.64	1	0.89053	.	.	1
**8760**	0	1.64	1	0.89053	.	.	1
**8761**	0	1.64	1	0.89053	.	.	1
**8762**	0	1.64	1	0.89053	.	.	1
**8763**	0	1.64	1	0.89053	.	.	1
**8764**	0	1.64	1	0.89053	.	.	1
**8765**	0	1.64	1	0.89053	.	.	1
**8766**	0	1.64	1	0.89053	.	.	1
**8767**	0	1.64	1	0.89053	.	.	1
**8768**	0	1.64	1	0.89053	.	.	1
**8769**	0	1.64	1	0.89053	.	.	1
**8770**	0	1.64	1	0.89053	.	.	1
**8771**	0	1.64	1	0.89053	.	.	1
**8772**	0	1.64	1	0.89053	.	.	1
**8773**	0	1.64	1	0.89053	.	.	1
**8774**	0	1.64	1	0.89053	.	.	1
**8775**	0	1.64	1	0.89053	.	.	1
**8776**	0	1.64	1	0.89053	.	.	1
**8777**	0	1.64	1	0.89053	.	.	1
**8778**	0	1.64	1	0.89053	.	.	1
**8779**	0	1.64	1	0.89053	.	.	1
**8780**	0	1.64	1	0.89053	.	.	1
**8781**	0	1.64	1	0.89053	.	.	1
**8782**	0	1.64	1	0.89053	.	.	1
**8783**	0	1.64	1	0.89053	.	.	1
**8784**	0	1.64	1	0.89053	.	.	1
**8785**	0	1.64	1	0.89053	.	.	1

Obs	Sub_group	fuptimey	_CENSOR_	SURVIVAL	SDF_LCL	SDF_UCL	STRATUM
8786	0	1.65	0	0.89029	0.88731	0.89319	1
8787	0	1.65	1	0.89029	.	.	1
8788	0	1.65	1	0.89029	.	.	1
8789	0	1.65	1	0.89029	.	.	1
8790	0	1.65	1	0.89029	.	.	1
8791	0	1.65	1	0.89029	.	.	1
8792	0	1.65	1	0.89029	.	.	1
8793	0	1.65	1	0.89029	.	.	1
8794	0	1.65	1	0.89029	.	.	1
8795	0	1.65	1	0.89029	.	.	1
8796	0	1.65	1	0.89029	.	.	1
8797	0	1.65	1	0.89029	.	.	1
8798	0	1.65	1	0.89029	.	.	1
8799	0	1.65	1	0.89029	.	.	1
8800	0	1.65	1	0.89029	.	.	1
8801	0	1.65	1	0.89029	.	.	1
8802	0	1.65	1	0.89029	.	.	1
8803	0	1.65	1	0.89029	.	.	1
8804	0	1.65	1	0.89029	.	.	1
8805	0	1.65	1	0.89029	.	.	1
8806	0	1.65	1	0.89029	.	.	1
8807	0	1.65	1	0.89029	.	.	1
8808	0	1.65	1	0.89029	.	.	1
8809	0	1.65	1	0.89029	.	.	1
8810	0	1.65	1	0.89029	.	.	1
8811	0	1.65	1	0.89029	.	.	1
8812	0	1.65	1	0.89029	.	.	1
8813	0	1.65	1	0.89029	.	.	1
8814	0	1.65	1	0.89029	.	.	1
8815	0	1.65	1	0.89029	.	.	1
8816	0	1.65	1	0.89029	.	.	1
8817	0	1.65	1	0.89029	.	.	1
8818	0	1.65	1	0.89029	.	.	1
8819	0	1.65	1	0.89029	.	.	1
8820	0	1.65	1	0.89029	.	.	1

Obs	Sub_group	fuptimey	_CENSOR_	SURVIVAL	SDF_LCL	SDF_UCL	STRATUM
8821	0	1.65	1	0.89029	.	.	1
8822	0	1.65	1	0.89029	.	.	1
8823	0	1.65	1	0.89029	.	.	1
8824	0	1.65	1	0.89029	.	.	1
8825	0	1.65	1	0.89029	.	.	1
8826	0	1.65	1	0.89029	.	.	1
8827	0	1.65	1	0.89029	.	.	1
8828	0	1.65	1	0.89029	.	.	1
8829	0	1.65	1	0.89029	.	.	1
8830	0	1.66	0	0.89013	0.88715	0.89303	1
8831	0	1.66	1	0.89013	.	.	1
8832	0	1.66	1	0.89013	.	.	1
8833	0	1.66	1	0.89013	.	.	1
8834	0	1.66	1	0.89013	.	.	1
8835	0	1.66	1	0.89013	.	.	1
8836	0	1.66	1	0.89013	.	.	1
8837	0	1.66	1	0.89013	.	.	1
8838	0	1.66	1	0.89013	.	.	1
8839	0	1.66	1	0.89013	.	.	1
8840	0	1.66	1	0.89013	.	.	1
8841	0	1.66	1	0.89013	.	.	1
8842	0	1.66	1	0.89013	.	.	1
8843	0	1.66	1	0.89013	.	.	1
8844	0	1.66	1	0.89013	.	.	1
8845	0	1.66	1	0.89013	.	.	1
8846	0	1.66	1	0.89013	.	.	1
8847	0	1.66	1	0.89013	.	.	1
8848	0	1.66	1	0.89013	.	.	1
8849	0	1.66	1	0.89013	.	.	1
8850	0	1.66	1	0.89013	.	.	1
8851	0	1.66	1	0.89013	.	.	1
8852	0	1.66	1	0.89013	.	.	1
8853	0	1.66	1	0.89013	.	.	1
8854	0	1.66	1	0.89013	.	.	1
8855	0	1.66	1	0.89013	.	.	1

Obs	Sub_group	fuptimey	_CENSOR_	SURVIVAL	SDF_LCL	SDF_UCL	STRATUM
8856	0	1.66	1	0.89013	.	.	1
8857	0	1.66	1	0.89013	.	.	1
8858	0	1.66	1	0.89013	.	.	1
8859	0	1.66	1	0.89013	.	.	1
8860	0	1.66	1	0.89013	.	.	1
8861	0	1.66	1	0.89013	.	.	1
8862	0	1.66	1	0.89013	.	.	1
8863	0	1.66	1	0.89013	.	.	1
8864	0	1.66	1	0.89013	.	.	1
8865	0	1.66	1	0.89013	.	.	1
8866	0	1.66	1	0.89013	.	.	1
8867	0	1.66	1	0.89013	.	.	1
8868	0	1.66	1	0.89013	.	.	1
8869	0	1.66	1	0.89013	.	.	1
8870	0	1.66	1	0.89013	.	.	1
8871	0	1.66	1	0.89013	.	.	1
8872	0	1.66	1	0.89013	.	.	1
8873	0	1.66	1	0.89013	.	.	1
8874	0	1.66	1	0.89013	.	.	1
8875	0	1.66	1	0.89013	.	.	1
8876	0	1.66	1	0.89013	.	.	1
8877	0	1.67	0	0.88986	0.88688	0.89277	1
8878	0	1.67	1	0.88986	.	.	1
8879	0	1.67	1	0.88986	.	.	1
8880	0	1.67	1	0.88986	.	.	1
8881	0	1.67	1	0.88986	.	.	1
8882	0	1.67	1	0.88986	.	.	1
8883	0	1.67	1	0.88986	.	.	1
8884	0	1.67	1	0.88986	.	.	1
8885	0	1.67	1	0.88986	.	.	1
8886	0	1.67	1	0.88986	.	.	1
8887	0	1.67	1	0.88986	.	.	1
8888	0	1.67	1	0.88986	.	.	1
8889	0	1.67	1	0.88986	.	.	1
8890	0	1.67	1	0.88986	.	.	1

Obs	Sub_group	fuptimey	_CENSOR_	SURVIVAL	SDF_LCL	SDF_UCL	STRATUM
8891	0	1.67	1	0.88986	.	.	1
8892	0	1.67	1	0.88986	.	.	1
8893	0	1.67	1	0.88986	.	.	1
8894	0	1.67	1	0.88986	.	.	1
8895	0	1.67	1	0.88986	.	.	1
8896	0	1.67	1	0.88986	.	.	1
8897	0	1.67	1	0.88986	.	.	1
8898	0	1.67	1	0.88986	.	.	1
8899	0	1.67	1	0.88986	.	.	1
8900	0	1.67	1	0.88986	.	.	1
8901	0	1.67	1	0.88986	.	.	1
8902	0	1.67	1	0.88986	.	.	1
8903	0	1.67	1	0.88986	.	.	1
8904	0	1.67	1	0.88986	.	.	1
8905	0	1.67	1	0.88986	.	.	1
8906	0	1.67	1	0.88986	.	.	1
8907	0	1.67	1	0.88986	.	.	1
8908	0	1.67	1	0.88986	.	.	1
8909	0	1.67	1	0.88986	.	.	1
8910	0	1.67	1	0.88986	.	.	1
8911	0	1.67	1	0.88986	.	.	1
8912	0	1.67	1	0.88986	.	.	1
8913	0	1.67	1	0.88986	.	.	1
8914	0	1.67	1	0.88986	.	.	1
8915	0	1.67	1	0.88986	.	.	1
8916	0	1.67	1	0.88986	.	.	1
8917	0	1.67	1	0.88986	.	.	1
8918	0	1.67	1	0.88986	.	.	1
8919	0	1.67	1	0.88986	.	.	1
8920	0	1.68	0	0.88964	0.88666	0.89256	1
8921	0	1.68	1	0.88964	.	.	1
8922	0	1.68	1	0.88964	.	.	1
8923	0	1.68	1	0.88964	.	.	1
8924	0	1.68	1	0.88964	.	.	1
8925	0	1.68	1	0.88964	.	.	1

Obs	Sub_group	fuptimey	_CENSOR_	SURVIVAL	SDF_LCL	SDF_UCL	STRATUM
8926	0	1.68	1	0.88964	.	.	1
8927	0	1.68	1	0.88964	.	.	1
8928	0	1.68	1	0.88964	.	.	1
8929	0	1.68	1	0.88964	.	.	1
8930	0	1.68	1	0.88964	.	.	1
8931	0	1.68	1	0.88964	.	.	1
8932	0	1.68	1	0.88964	.	.	1
8933	0	1.68	1	0.88964	.	.	1
8934	0	1.68	1	0.88964	.	.	1
8935	0	1.68	1	0.88964	.	.	1
8936	0	1.68	1	0.88964	.	.	1
8937	0	1.68	1	0.88964	.	.	1
8938	0	1.68	1	0.88964	.	.	1
8939	0	1.68	1	0.88964	.	.	1
8940	0	1.68	1	0.88964	.	.	1
8941	0	1.68	1	0.88964	.	.	1
8942	0	1.68	1	0.88964	.	.	1
8943	0	1.68	1	0.88964	.	.	1
8944	0	1.68	1	0.88964	.	.	1
8945	0	1.68	1	0.88964	.	.	1
8946	0	1.68	1	0.88964	.	.	1
8947	0	1.68	1	0.88964	.	.	1
8948	0	1.68	1	0.88964	.	.	1
8949	0	1.68	1	0.88964	.	.	1
8950	0	1.68	1	0.88964	.	.	1
8951	0	1.68	1	0.88964	.	.	1
8952	0	1.68	1	0.88964	.	.	1
8953	0	1.68	1	0.88964	.	.	1
8954	0	1.68	1	0.88964	.	.	1
8955	0	1.68	1	0.88964	.	.	1
8956	0	1.68	1	0.88964	.	.	1
8957	0	1.68	1	0.88964	.	.	1
8958	0	1.68	1	0.88964	.	.	1
8959	0	1.68	1	0.88964	.	.	1
8960	0	1.68	1	0.88964	.	.	1

Obs	Sub_group	fuptimey	_CENSOR_	SURVIVAL	SDF_LCL	SDF_UCL	STRATUM
8961	0	1.68	1	0.88964	.	.	1
8962	0	1.68	1	0.88964	.	.	1
8963	0	1.68	1	0.88964	.	.	1
8964	0	1.68	1	0.88964	.	.	1
8965	0	1.68	1	0.88964	.	.	1
8966	0	1.68	1	0.88964	.	.	1
8967	0	1.69	0	0.88946	0.88647	0.89237	1
8968	0	1.69	1	0.88946	.	.	1
8969	0	1.69	1	0.88946	.	.	1
8970	0	1.69	1	0.88946	.	.	1
8971	0	1.69	1	0.88946	.	.	1
8972	0	1.69	1	0.88946	.	.	1
8973	0	1.69	1	0.88946	.	.	1
8974	0	1.69	1	0.88946	.	.	1
8975	0	1.69	1	0.88946	.	.	1
8976	0	1.69	1	0.88946	.	.	1
8977	0	1.69	1	0.88946	.	.	1
8978	0	1.69	1	0.88946	.	.	1
8979	0	1.69	1	0.88946	.	.	1
8980	0	1.69	1	0.88946	.	.	1
8981	0	1.69	1	0.88946	.	.	1
8982	0	1.69	1	0.88946	.	.	1
8983	0	1.69	1	0.88946	.	.	1
8984	0	1.69	1	0.88946	.	.	1
8985	0	1.69	1	0.88946	.	.	1
8986	0	1.69	1	0.88946	.	.	1
8987	0	1.69	1	0.88946	.	.	1
8988	0	1.69	1	0.88946	.	.	1
8989	0	1.69	1	0.88946	.	.	1
8990	0	1.69	1	0.88946	.	.	1
8991	0	1.69	1	0.88946	.	.	1
8992	0	1.69	1	0.88946	.	.	1
8993	0	1.69	1	0.88946	.	.	1
8994	0	1.69	1	0.88946	.	.	1
8995	0	1.69	1	0.88946	.	.	1

Obs	Sub_group	fuptimey	_CENSOR_	SURVIVAL	SDF_LCL	SDF_UCL	STRATUM
8996	0	1.69	1	0.88946	.	.	1
8997	0	1.69	1	0.88946	.	.	1
8998	0	1.69	1	0.88946	.	.	1
8999	0	1.69	1	0.88946	.	.	1
9000	0	1.69	1	0.88946	.	.	1
9001	0	1.69	1	0.88946	.	.	1
9002	0	1.69	1	0.88946	.	.	1
9003	0	1.69	1	0.88946	.	.	1
9004	0	1.69	1	0.88946	.	.	1
9005	0	1.69	1	0.88946	.	.	1
9006	0	1.69	1	0.88946	.	.	1
9007	0	1.69	1	0.88946	.	.	1
9008	0	1.69	1	0.88946	.	.	1
9009	0	1.69	1	0.88946	.	.	1
9010	0	1.69	1	0.88946	.	.	1
9011	0	1.69	1	0.88946	.	.	1
9012	0	1.69	1	0.88946	.	.	1
9013	0	1.69	1	0.88946	.	.	1
9014	0	1.69	1	0.88946	.	.	1
9015	0	1.69	1	0.88946	.	.	1
9016	0	1.69	1	0.88946	.	.	1
9017	0	1.69	1	0.88946	.	.	1
9018	0	1.69	1	0.88946	.	.	1
9019	0	1.69	1	0.88946	.	.	1
9020	0	1.69	1	0.88946	.	.	1
9021	0	1.69	1	0.88946	.	.	1
9022	0	1.69	1	0.88946	.	.	1
9023	0	1.69	1	0.88946	.	.	1
9024	0	1.69	1	0.88946	.	.	1
9025	0	1.69	1	0.88946	.	.	1
9026	0	1.69	1	0.88946	.	.	1
9027	0	1.69	1	0.88946	.	.	1
9028	0	1.69	1	0.88946	.	.	1
9029	0	1.69	1	0.88946	.	.	1
9030	0	1.70	0	0.88919	0.88619	0.89211	1

Obs	Sub_group	fuptimey	_CENSOR_	SURVIVAL	SDF_LCL	SDF_UCL	STRATUM
9031	0	1.70	1	0.88919	.	.	1
9032	0	1.70	1	0.88919	.	.	1
9033	0	1.70	1	0.88919	.	.	1
9034	0	1.70	1	0.88919	.	.	1
9035	0	1.70	1	0.88919	.	.	1
9036	0	1.70	1	0.88919	.	.	1
9037	0	1.70	1	0.88919	.	.	1
9038	0	1.70	1	0.88919	.	.	1
9039	0	1.70	1	0.88919	.	.	1
9040	0	1.70	1	0.88919	.	.	1
9041	0	1.70	1	0.88919	.	.	1
9042	0	1.70	1	0.88919	.	.	1
9043	0	1.70	1	0.88919	.	.	1
9044	0	1.70	1	0.88919	.	.	1
9045	0	1.70	1	0.88919	.	.	1
9046	0	1.70	1	0.88919	.	.	1
9047	0	1.70	1	0.88919	.	.	1
9048	0	1.70	1	0.88919	.	.	1
9049	0	1.70	1	0.88919	.	.	1
9050	0	1.70	1	0.88919	.	.	1
9051	0	1.70	1	0.88919	.	.	1
9052	0	1.70	1	0.88919	.	.	1
9053	0	1.70	1	0.88919	.	.	1
9054	0	1.70	1	0.88919	.	.	1
9055	0	1.70	1	0.88919	.	.	1
9056	0	1.70	1	0.88919	.	.	1
9057	0	1.70	1	0.88919	.	.	1
9058	0	1.70	1	0.88919	.	.	1
9059	0	1.70	1	0.88919	.	.	1
9060	0	1.70	1	0.88919	.	.	1
9061	0	1.70	1	0.88919	.	.	1
9062	0	1.70	1	0.88919	.	.	1
9063	0	1.70	1	0.88919	.	.	1
9064	0	1.70	1	0.88919	.	.	1
9065	0	1.70	1	0.88919	.	.	1

Obs	Sub_group	fuptimey	_CENSOR_	SURVIVAL	SDF_LCL	SDF_UCL	STRATUM
9066	0	1.70	1	0.88919	.	.	1
9067	0	1.70	1	0.88919	.	.	1
9068	0	1.70	1	0.88919	.	.	1
9069	0	1.70	1	0.88919	.	.	1
9070	0	1.70	1	0.88919	.	.	1
9071	0	1.70	1	0.88919	.	.	1
9072	0	1.70	1	0.88919	.	.	1
9073	0	1.70	1	0.88919	.	.	1
9074	0	1.70	1	0.88919	.	.	1
9075	0	1.71	0	0.88892	0.88592	0.89184	1
9076	0	1.71	1	0.88892	.	.	1
9077	0	1.71	1	0.88892	.	.	1
9078	0	1.71	1	0.88892	.	.	1
9079	0	1.71	1	0.88892	.	.	1
9080	0	1.71	1	0.88892	.	.	1
9081	0	1.71	1	0.88892	.	.	1
9082	0	1.71	1	0.88892	.	.	1
9083	0	1.71	1	0.88892	.	.	1
9084	0	1.71	1	0.88892	.	.	1
9085	0	1.71	1	0.88892	.	.	1
9086	0	1.71	1	0.88892	.	.	1
9087	0	1.71	1	0.88892	.	.	1
9088	0	1.71	1	0.88892	.	.	1
9089	0	1.71	1	0.88892	.	.	1
9090	0	1.71	1	0.88892	.	.	1
9091	0	1.71	1	0.88892	.	.	1
9092	0	1.71	1	0.88892	.	.	1
9093	0	1.71	1	0.88892	.	.	1
9094	0	1.71	1	0.88892	.	.	1
9095	0	1.71	1	0.88892	.	.	1
9096	0	1.71	1	0.88892	.	.	1
9097	0	1.71	1	0.88892	.	.	1
9098	0	1.71	1	0.88892	.	.	1
9099	0	1.71	1	0.88892	.	.	1
9100	0	1.71	1	0.88892	.	.	1

Obs	Sub_group	fuptimey	_CENSOR_	SURVIVAL	SDF_LCL	SDF_UCL	STRATUM
9101	0	1.71	1	0.88892	.	.	1
9102	0	1.71	1	0.88892	.	.	1
9103	0	1.71	1	0.88892	.	.	1
9104	0	1.71	1	0.88892	.	.	1
9105	0	1.71	1	0.88892	.	.	1
9106	0	1.71	1	0.88892	.	.	1
9107	0	1.71	1	0.88892	.	.	1
9108	0	1.71	1	0.88892	.	.	1
9109	0	1.71	1	0.88892	.	.	1
9110	0	1.71	1	0.88892	.	.	1
9111	0	1.71	1	0.88892	.	.	1
9112	0	1.71	1	0.88892	.	.	1
9113	0	1.71	1	0.88892	.	.	1
9114	0	1.71	1	0.88892	.	.	1
9115	0	1.71	1	0.88892	.	.	1
9116	0	1.71	1	0.88892	.	.	1
9117	0	1.71	1	0.88892	.	.	1
9118	0	1.71	1	0.88892	.	.	1
9119	0	1.71	1	0.88892	.	.	1
9120	0	1.71	1	0.88892	.	.	1
9121	0	1.71	1	0.88892	.	.	1
9122	0	1.71	1	0.88892	.	.	1
9123	0	1.71	1	0.88892	.	.	1
9124	0	1.71	1	0.88892	.	.	1
9125	0	1.71	1	0.88892	.	.	1
9126	0	1.71	1	0.88892	.	.	1
9127	0	1.71	1	0.88892	.	.	1
9128	0	1.71	1	0.88892	.	.	1
9129	0	1.71	1	0.88892	.	.	1
9130	0	1.71	1	0.88892	.	.	1
9131	0	1.71	1	0.88892	.	.	1
9132	0	1.71	1	0.88892	.	.	1
9133	0	1.71	1	0.88892	.	.	1
9134	0	1.71	1	0.88892	.	.	1
9135	0	1.71	1	0.88892	.	.	1

Obs	Sub_group	fuptimey	_CENSOR_	SURVIVAL	SDF_LCL	SDF_UCL	STRATUM
9136	0	1.71	1	0.88892	.	.	1
9137	0	1.71	1	0.88892	.	.	1
9138	0	1.71	1	0.88892	.	.	1
9139	0	1.71	1	0.88892	.	.	1
9140	0	1.71	1	0.88892	.	.	1
9141	0	1.71	1	0.88892	.	.	1
9142	0	1.71	1	0.88892	.	.	1
9143	0	1.72	0	0.88870	0.88570	0.89163	1
9144	0	1.72	1	0.88870	.	.	1
9145	0	1.72	1	0.88870	.	.	1
9146	0	1.72	1	0.88870	.	.	1
9147	0	1.72	1	0.88870	.	.	1
9148	0	1.72	1	0.88870	.	.	1
9149	0	1.72	1	0.88870	.	.	1
9150	0	1.72	1	0.88870	.	.	1
9151	0	1.72	1	0.88870	.	.	1
9152	0	1.72	1	0.88870	.	.	1
9153	0	1.72	1	0.88870	.	.	1
9154	0	1.72	1	0.88870	.	.	1
9155	0	1.72	1	0.88870	.	.	1
9156	0	1.72	1	0.88870	.	.	1
9157	0	1.72	1	0.88870	.	.	1
9158	0	1.72	1	0.88870	.	.	1
9159	0	1.72	1	0.88870	.	.	1
9160	0	1.72	1	0.88870	.	.	1
9161	0	1.72	1	0.88870	.	.	1
9162	0	1.72	1	0.88870	.	.	1
9163	0	1.72	1	0.88870	.	.	1
9164	0	1.72	1	0.88870	.	.	1
9165	0	1.72	1	0.88870	.	.	1
9166	0	1.72	1	0.88870	.	.	1
9167	0	1.72	1	0.88870	.	.	1
9168	0	1.72	1	0.88870	.	.	1
9169	0	1.72	1	0.88870	.	.	1
9170	0	1.72	1	0.88870	.	.	1

Obs	Sub_group	fuptimey	_CENSOR_	SURVIVAL	SDF_LCL	SDF_UCL	STRATUM
**9171**	0	1.72	1	0.88870	.	.	1
**9172**	0	1.72	1	0.88870	.	.	1
**9173**	0	1.72	1	0.88870	.	.	1
**9174**	0	1.72	1	0.88870	.	.	1
**9175**	0	1.72	1	0.88870	.	.	1
**9176**	0	1.72	1	0.88870	.	.	1
**9177**	0	1.72	1	0.88870	.	.	1
**9178**	0	1.72	1	0.88870	.	.	1
**9179**	0	1.72	1	0.88870	.	.	1
**9180**	0	1.72	1	0.88870	.	.	1
**9181**	0	1.72	1	0.88870	.	.	1
**9182**	0	1.72	1	0.88870	.	.	1
**9183**	0	1.72	1	0.88870	.	.	1
**9184**	0	1.73	0	0.88854	0.88553	0.89147	1
**9185**	0	1.73	1	0.88854	.	.	1
**9186**	0	1.73	1	0.88854	.	.	1
**9187**	0	1.73	1	0.88854	.	.	1
**9188**	0	1.73	1	0.88854	.	.	1
**9189**	0	1.73	1	0.88854	.	.	1
**9190**	0	1.73	1	0.88854	.	.	1
**9191**	0	1.73	1	0.88854	.	.	1
**9192**	0	1.73	1	0.88854	.	.	1
**9193**	0	1.73	1	0.88854	.	.	1
**9194**	0	1.73	1	0.88854	.	.	1
**9195**	0	1.73	1	0.88854	.	.	1
**9196**	0	1.73	1	0.88854	.	.	1
**9197**	0	1.73	1	0.88854	.	.	1
**9198**	0	1.73	1	0.88854	.	.	1
**9199**	0	1.73	1	0.88854	.	.	1
**9200**	0	1.73	1	0.88854	.	.	1
**9201**	0	1.73	1	0.88854	.	.	1
**9202**	0	1.73	1	0.88854	.	.	1
**9203**	0	1.73	1	0.88854	.	.	1
**9204**	0	1.73	1	0.88854	.	.	1
**9205**	0	1.73	1	0.88854	.	.	1

Obs	Sub_group	fuptimey	_CENSOR_	SURVIVAL	SDF_LCL	SDF_UCL	STRATUM
9206	0	1.73	1	0.88854	.	.	1
9207	0	1.73	1	0.88854	.	.	1
9208	0	1.73	1	0.88854	.	.	1
9209	0	1.73	1	0.88854	.	.	1
9210	0	1.73	1	0.88854	.	.	1
9211	0	1.73	1	0.88854	.	.	1
9212	0	1.73	1	0.88854	.	.	1
9213	0	1.73	1	0.88854	.	.	1
9214	0	1.73	1	0.88854	.	.	1
9215	0	1.73	1	0.88854	.	.	1
9216	0	1.73	1	0.88854	.	.	1
9217	0	1.73	1	0.88854	.	.	1
9218	0	1.73	1	0.88854	.	.	1
9219	0	1.73	1	0.88854	.	.	1
9220	0	1.73	1	0.88854	.	.	1
9221	0	1.73	1	0.88854	.	.	1
9222	0	1.73	1	0.88854	.	.	1
9223	0	1.73	1	0.88854	.	.	1
9224	0	1.73	1	0.88854	.	.	1
9225	0	1.73	1	0.88854	.	.	1
9226	0	1.73	1	0.88854	.	.	1
9227	0	1.73	1	0.88854	.	.	1
9228	0	1.73	1	0.88854	.	.	1
9229	0	1.73	1	0.88854	.	.	1
9230	0	1.73	1	0.88854	.	.	1
9231	0	1.73	1	0.88854	.	.	1
9232	0	1.73	1	0.88854	.	.	1
9233	0	1.73	1	0.88854	.	.	1
9234	0	1.73	1	0.88854	.	.	1
9235	0	1.73	1	0.88854	.	.	1
9236	0	1.73	1	0.88854	.	.	1
9237	0	1.73	1	0.88854	.	.	1
9238	0	1.73	1	0.88854	.	.	1
9239	0	1.73	1	0.88854	.	.	1
9240	0	1.73	1	0.88854	.	.	1

Obs	Sub_group	fuptimey	_CENSOR_	SURVIVAL	SDF_LCL	SDF_UCL	STRATUM
**9241**	0	1.73	1	0.88854	.	.	1
**9242**	0	1.73	1	0.88854	.	.	1
**9243**	0	1.73	1	0.88854	.	.	1
**9244**	0	1.73	1	0.88854	.	.	1
**9245**	0	1.73	1	0.88854	.	.	1
**9246**	0	1.73	1	0.88854	.	.	1
**9247**	0	1.73	1	0.88854	.	.	1
**9248**	0	1.74	0	0.88813	0.88512	0.89106	1
**9249**	0	1.74	1	0.88813	.	.	1
**9250**	0	1.74	1	0.88813	.	.	1
**9251**	0	1.74	1	0.88813	.	.	1
**9252**	0	1.74	1	0.88813	.	.	1
**9253**	0	1.74	1	0.88813	.	.	1
**9254**	0	1.74	1	0.88813	.	.	1
**9255**	0	1.74	1	0.88813	.	.	1
**9256**	0	1.74	1	0.88813	.	.	1
**9257**	0	1.74	1	0.88813	.	.	1
**9258**	0	1.74	1	0.88813	.	.	1
**9259**	0	1.74	1	0.88813	.	.	1
**9260**	0	1.74	1	0.88813	.	.	1
**9261**	0	1.74	1	0.88813	.	.	1
**9262**	0	1.74	1	0.88813	.	.	1
**9263**	0	1.74	1	0.88813	.	.	1
**9264**	0	1.74	1	0.88813	.	.	1
**9265**	0	1.74	1	0.88813	.	.	1
**9266**	0	1.74	1	0.88813	.	.	1
**9267**	0	1.74	1	0.88813	.	.	1
**9268**	0	1.74	1	0.88813	.	.	1
**9269**	0	1.74	1	0.88813	.	.	1
**9270**	0	1.74	1	0.88813	.	.	1
**9271**	0	1.74	1	0.88813	.	.	1
**9272**	0	1.74	1	0.88813	.	.	1
**9273**	0	1.74	1	0.88813	.	.	1
**9274**	0	1.74	1	0.88813	.	.	1
**9275**	0	1.74	1	0.88813	.	.	1

Obs	Sub_group	fuptimey	_CENSOR_	SURVIVAL	SDF_LCL	SDF_UCL	STRATUM
9276	0	1.74	1	0.88813	.	.	1
9277	0	1.74	1	0.88813	.	.	1
9278	0	1.74	1	0.88813	.	.	1
9279	0	1.74	1	0.88813	.	.	1
9280	0	1.74	1	0.88813	.	.	1
9281	0	1.74	1	0.88813	.	.	1
9282	0	1.74	1	0.88813	.	.	1
9283	0	1.74	1	0.88813	.	.	1
9284	0	1.74	1	0.88813	.	.	1
9285	0	1.74	1	0.88813	.	.	1
9286	0	1.74	1	0.88813	.	.	1
9287	0	1.74	1	0.88813	.	.	1
9288	0	1.74	1	0.88813	.	.	1
9289	0	1.74	1	0.88813	.	.	1
9290	0	1.74	1	0.88813	.	.	1
9291	0	1.74	1	0.88813	.	.	1
9292	0	1.74	1	0.88813	.	.	1
9293	0	1.74	1	0.88813	.	.	1
9294	0	1.74	1	0.88813	.	.	1
9295	0	1.75	0	0.88794	0.88493	0.89088	1
9296	0	1.75	1	0.88794	.	.	1
9297	0	1.75	1	0.88794	.	.	1
9298	0	1.75	1	0.88794	.	.	1
9299	0	1.75	1	0.88794	.	.	1
9300	0	1.75	1	0.88794	.	.	1
9301	0	1.75	1	0.88794	.	.	1
9302	0	1.75	1	0.88794	.	.	1
9303	0	1.75	1	0.88794	.	.	1
9304	0	1.75	1	0.88794	.	.	1
9305	0	1.75	1	0.88794	.	.	1
9306	0	1.75	1	0.88794	.	.	1
9307	0	1.75	1	0.88794	.	.	1
9308	0	1.75	1	0.88794	.	.	1
9309	0	1.75	1	0.88794	.	.	1
9310	0	1.75	1	0.88794	.	.	1

Obs	Sub_group	fuptimey	_CENSOR_	SURVIVAL	SDF_LCL	SDF_UCL	STRATUM
**9311**	0	1.75	1	0.88794	.	.	1
**9312**	0	1.75	1	0.88794	.	.	1
**9313**	0	1.75	1	0.88794	.	.	1
**9314**	0	1.75	1	0.88794	.	.	1
**9315**	0	1.75	1	0.88794	.	.	1
**9316**	0	1.75	1	0.88794	.	.	1
**9317**	0	1.75	1	0.88794	.	.	1
**9318**	0	1.75	1	0.88794	.	.	1
**9319**	0	1.75	1	0.88794	.	.	1
**9320**	0	1.75	1	0.88794	.	.	1
**9321**	0	1.75	1	0.88794	.	.	1
**9322**	0	1.75	1	0.88794	.	.	1
**9323**	0	1.75	1	0.88794	.	.	1
**9324**	0	1.75	1	0.88794	.	.	1
**9325**	0	1.75	1	0.88794	.	.	1
**9326**	0	1.75	1	0.88794	.	.	1
**9327**	0	1.75	1	0.88794	.	.	1
**9328**	0	1.75	1	0.88794	.	.	1
**9329**	0	1.75	1	0.88794	.	.	1
**9330**	0	1.75	1	0.88794	.	.	1
**9331**	0	1.75	1	0.88794	.	.	1
**9332**	0	1.75	1	0.88794	.	.	1
**9333**	0	1.75	1	0.88794	.	.	1
**9334**	0	1.75	1	0.88794	.	.	1
**9335**	0	1.75	1	0.88794	.	.	1
**9336**	0	1.75	1	0.88794	.	.	1
**9337**	0	1.75	1	0.88794	.	.	1
**9338**	0	1.75	1	0.88794	.	.	1
**9339**	0	1.75	1	0.88794	.	.	1
**9340**	0	1.75	1	0.88794	.	.	1
**9341**	0	1.75	1	0.88794	.	.	1
**9342**	0	1.75	1	0.88794	.	.	1
**9343**	0	1.75	1	0.88794	.	.	1
**9344**	0	1.75	1	0.88794	.	.	1
**9345**	0	1.76	0	0.88761	0.88460	0.89055	1

Obs	Sub_group	fuptimey	_CENSOR_	SURVIVAL	SDF_LCL	SDF_UCL	STRATUM
9346	0	1.76	1	0.88761	.	.	1
9347	0	1.76	1	0.88761	.	.	1
9348	0	1.76	1	0.88761	.	.	1
9349	0	1.76	1	0.88761	.	.	1
9350	0	1.76	1	0.88761	.	.	1
9351	0	1.76	1	0.88761	.	.	1
9352	0	1.76	1	0.88761	.	.	1
9353	0	1.76	1	0.88761	.	.	1
9354	0	1.76	1	0.88761	.	.	1
9355	0	1.76	1	0.88761	.	.	1
9356	0	1.76	1	0.88761	.	.	1
9357	0	1.76	1	0.88761	.	.	1
9358	0	1.76	1	0.88761	.	.	1
9359	0	1.76	1	0.88761	.	.	1
9360	0	1.76	1	0.88761	.	.	1
9361	0	1.76	1	0.88761	.	.	1
9362	0	1.76	1	0.88761	.	.	1
9363	0	1.76	1	0.88761	.	.	1
9364	0	1.76	1	0.88761	.	.	1
9365	0	1.76	1	0.88761	.	.	1
9366	0	1.76	1	0.88761	.	.	1
9367	0	1.76	1	0.88761	.	.	1
9368	0	1.76	1	0.88761	.	.	1
9369	0	1.76	1	0.88761	.	.	1
9370	0	1.76	1	0.88761	.	.	1
9371	0	1.76	1	0.88761	.	.	1
9372	0	1.76	1	0.88761	.	.	1
9373	0	1.76	1	0.88761	.	.	1
9374	0	1.76	1	0.88761	.	.	1
9375	0	1.76	1	0.88761	.	.	1
9376	0	1.76	1	0.88761	.	.	1
9377	0	1.77	0	0.88745	0.88443	0.89039	1
9378	0	1.77	1	0.88745	.	.	1
9379	0	1.77	1	0.88745	.	.	1
9380	0	1.77	1	0.88745	.	.	1

Obs	Sub_group	fuptimey	_CENSOR_	SURVIVAL	SDF_LCL	SDF_UCL	STRATUM
9381	0	1.77	1	0.88745	.	.	1
9382	0	1.77	1	0.88745	.	.	1
9383	0	1.77	1	0.88745	.	.	1
9384	0	1.77	1	0.88745	.	.	1
9385	0	1.77	1	0.88745	.	.	1
9386	0	1.77	1	0.88745	.	.	1
9387	0	1.77	1	0.88745	.	.	1
9388	0	1.77	1	0.88745	.	.	1
9389	0	1.77	1	0.88745	.	.	1
9390	0	1.77	1	0.88745	.	.	1
9391	0	1.77	1	0.88745	.	.	1
9392	0	1.77	1	0.88745	.	.	1
9393	0	1.77	1	0.88745	.	.	1
9394	0	1.77	1	0.88745	.	.	1
9395	0	1.77	1	0.88745	.	.	1
9396	0	1.77	1	0.88745	.	.	1
9397	0	1.77	1	0.88745	.	.	1
9398	0	1.77	1	0.88745	.	.	1
9399	0	1.77	1	0.88745	.	.	1
9400	0	1.77	1	0.88745	.	.	1
9401	0	1.77	1	0.88745	.	.	1
9402	0	1.77	1	0.88745	.	.	1
9403	0	1.77	1	0.88745	.	.	1
9404	0	1.77	1	0.88745	.	.	1
9405	0	1.77	1	0.88745	.	.	1
9406	0	1.77	1	0.88745	.	.	1
9407	0	1.77	1	0.88745	.	.	1
9408	0	1.77	1	0.88745	.	.	1
9409	0	1.77	1	0.88745	.	.	1
9410	0	1.77	1	0.88745	.	.	1
9411	0	1.77	1	0.88745	.	.	1
9412	0	1.77	1	0.88745	.	.	1
9413	0	1.77	1	0.88745	.	.	1
9414	0	1.77	1	0.88745	.	.	1
9415	0	1.77	1	0.88745	.	.	1

Obs	Sub_group	fuptimey	_CENSOR_	SURVIVAL	SDF_LCL	SDF_UCL	STRATUM
**9416**	0	1.77	1	0.88745	.	.	1
**9417**	0	1.77	1	0.88745	.	.	1
**9418**	0	1.77	1	0.88745	.	.	1
**9419**	0	1.77	1	0.88745	.	.	1
**9420**	0	1.77	1	0.88745	.	.	1
**9421**	0	1.77	1	0.88745	.	.	1
**9422**	0	1.77	1	0.88745	.	.	1
**9423**	0	1.77	1	0.88745	.	.	1
**9424**	0	1.77	1	0.88745	.	.	1
**9425**	0	1.77	1	0.88745	.	.	1
**9426**	0	1.77	1	0.88745	.	.	1
**9427**	0	1.77	1	0.88745	.	.	1
**9428**	0	1.77	1	0.88745	.	.	1
**9429**	0	1.77	1	0.88745	.	.	1
**9430**	0	1.77	1	0.88745	.	.	1
**9431**	0	1.77	1	0.88745	.	.	1
**9432**	0	1.77	1	0.88745	.	.	1
**9433**	0	1.77	1	0.88745	.	.	1
**9434**	0	1.77	1	0.88745	.	.	1
**9435**	0	1.78	0	0.88704	0.88401	0.88999	1
**9436**	0	1.78	1	0.88704	.	.	1
**9437**	0	1.78	1	0.88704	.	.	1
**9438**	0	1.78	1	0.88704	.	.	1
**9439**	0	1.78	1	0.88704	.	.	1
**9440**	0	1.78	1	0.88704	.	.	1
**9441**	0	1.78	1	0.88704	.	.	1
**9442**	0	1.78	1	0.88704	.	.	1
**9443**	0	1.78	1	0.88704	.	.	1
**9444**	0	1.78	1	0.88704	.	.	1
**9445**	0	1.78	1	0.88704	.	.	1
**9446**	0	1.78	1	0.88704	.	.	1
**9447**	0	1.78	1	0.88704	.	.	1
**9448**	0	1.78	1	0.88704	.	.	1
**9449**	0	1.78	1	0.88704	.	.	1
**9450**	0	1.78	1	0.88704	.	.	1

Obs	Sub_group	fuptimey	_CENSOR_	SURVIVAL	SDF_LCL	SDF_UCL	STRATUM
9451	0	1.78	1	0.88704	.	.	1
9452	0	1.78	1	0.88704	.	.	1
9453	0	1.78	1	0.88704	.	.	1
9454	0	1.78	1	0.88704	.	.	1
9455	0	1.78	1	0.88704	.	.	1
9456	0	1.78	1	0.88704	.	.	1
9457	0	1.78	1	0.88704	.	.	1
9458	0	1.78	1	0.88704	.	.	1
9459	0	1.78	1	0.88704	.	.	1
9460	0	1.78	1	0.88704	.	.	1
9461	0	1.78	1	0.88704	.	.	1
9462	0	1.78	1	0.88704	.	.	1
9463	0	1.78	1	0.88704	.	.	1
9464	0	1.78	1	0.88704	.	.	1
9465	0	1.78	1	0.88704	.	.	1
9466	0	1.78	1	0.88704	.	.	1
9467	0	1.78	1	0.88704	.	.	1
9468	0	1.78	1	0.88704	.	.	1
9469	0	1.78	1	0.88704	.	.	1
9470	0	1.78	1	0.88704	.	.	1
9471	0	1.78	1	0.88704	.	.	1
9472	0	1.78	1	0.88704	.	.	1
9473	0	1.78	1	0.88704	.	.	1
9474	0	1.78	1	0.88704	.	.	1
9475	0	1.78	1	0.88704	.	.	1
9476	0	1.78	1	0.88704	.	.	1
9477	0	1.78	1	0.88704	.	.	1
9478	0	1.78	1	0.88704	.	.	1
9479	0	1.78	1	0.88704	.	.	1
9480	0	1.78	1	0.88704	.	.	1
9481	0	1.78	1	0.88704	.	.	1
9482	0	1.78	1	0.88704	.	.	1
9483	0	1.78	1	0.88704	.	.	1
9484	0	1.78	1	0.88704	.	.	1
9485	0	1.78	1	0.88704	.	.	1

Obs	Sub_group	fuptimey	_CENSOR_	SURVIVAL	SDF_LCL	SDF_UCL	STRATUM
**9486**	0	1.78	1	0.88704	.	.	1
**9487**	0	1.78	1	0.88704	.	.	1
**9488**	0	1.78	1	0.88704	.	.	1
**9489**	0	1.78	1	0.88704	.	.	1
**9490**	0	1.78	1	0.88704	.	.	1
**9491**	0	1.78	1	0.88704	.	.	1
**9492**	0	1.78	1	0.88704	.	.	1
**9493**	0	1.78	1	0.88704	.	.	1
**9494**	0	1.78	1	0.88704	.	.	1
**9495**	0	1.78	1	0.88704	.	.	1
**9496**	0	1.78	1	0.88704	.	.	1
**9497**	0	1.78	1	0.88704	.	.	1
**9498**	0	1.78	1	0.88704	.	.	1
**9499**	0	1.78	1	0.88704	.	.	1
**9500**	0	1.78	1	0.88704	.	.	1
**9501**	0	1.79	0	0.88687	0.88385	0.88983	1
**9502**	0	1.79	1	0.88687	.	.	1
**9503**	0	1.79	1	0.88687	.	.	1
**9504**	0	1.79	1	0.88687	.	.	1
**9505**	0	1.79	1	0.88687	.	.	1
**9506**	0	1.79	1	0.88687	.	.	1
**9507**	0	1.79	1	0.88687	.	.	1
**9508**	0	1.79	1	0.88687	.	.	1
**9509**	0	1.79	1	0.88687	.	.	1
**9510**	0	1.79	1	0.88687	.	.	1
**9511**	0	1.79	1	0.88687	.	.	1
**9512**	0	1.79	1	0.88687	.	.	1
**9513**	0	1.79	1	0.88687	.	.	1
**9514**	0	1.79	1	0.88687	.	.	1
**9515**	0	1.79	1	0.88687	.	.	1
**9516**	0	1.79	1	0.88687	.	.	1
**9517**	0	1.79	1	0.88687	.	.	1
**9518**	0	1.79	1	0.88687	.	.	1
**9519**	0	1.79	1	0.88687	.	.	1
**9520**	0	1.79	1	0.88687	.	.	1

Obs	Sub_group	fuptimey	_CENSOR_	SURVIVAL	SDF_LCL	SDF_UCL	STRATUM
9521	0	1.79	1	0.88687	.	.	1
9522	0	1.79	1	0.88687	.	.	1
9523	0	1.79	1	0.88687	.	.	1
9524	0	1.79	1	0.88687	.	.	1
9525	0	1.79	1	0.88687	.	.	1
9526	0	1.79	1	0.88687	.	.	1
9527	0	1.79	1	0.88687	.	.	1
9528	0	1.79	1	0.88687	.	.	1
9529	0	1.79	1	0.88687	.	.	1
9530	0	1.79	1	0.88687	.	.	1
9531	0	1.79	1	0.88687	.	.	1
9532	0	1.79	1	0.88687	.	.	1
9533	0	1.79	1	0.88687	.	.	1
9534	0	1.79	1	0.88687	.	.	1
9535	0	1.79	1	0.88687	.	.	1
9536	0	1.79	1	0.88687	.	.	1
9537	0	1.79	1	0.88687	.	.	1
9538	0	1.79	1	0.88687	.	.	1
9539	0	1.79	1	0.88687	.	.	1
9540	0	1.79	1	0.88687	.	.	1
9541	0	1.79	1	0.88687	.	.	1
9542	0	1.79	1	0.88687	.	.	1
9543	0	1.79	1	0.88687	.	.	1
9544	0	1.79	1	0.88687	.	.	1
9545	0	1.79	1	0.88687	.	.	1
9546	0	1.79	1	0.88687	.	.	1
9547	0	1.79	1	0.88687	.	.	1
9548	0	1.79	1	0.88687	.	.	1
9549	0	1.79	1	0.88687	.	.	1
9550	0	1.79	1	0.88687	.	.	1
9551	0	1.79	1	0.88687	.	.	1
9552	0	1.79	1	0.88687	.	.	1
9553	0	1.79	1	0.88687	.	.	1
9554	0	1.79	1	0.88687	.	.	1
9555	0	1.79	1	0.88687	.	.	1

Obs	Sub_group	fuptimey	_CENSOR_	SURVIVAL	SDF_LCL	SDF_UCL	STRATUM
**9556**	0	1.80	0	0.88657	0.88354	0.88953	1
**9557**	0	1.80	1	0.88657	.	.	1
**9558**	0	1.80	1	0.88657	.	.	1
**9559**	0	1.80	1	0.88657	.	.	1
**9560**	0	1.80	1	0.88657	.	.	1
**9561**	0	1.80	1	0.88657	.	.	1
**9562**	0	1.80	1	0.88657	.	.	1
**9563**	0	1.80	1	0.88657	.	.	1
**9564**	0	1.80	1	0.88657	.	.	1
**9565**	0	1.80	1	0.88657	.	.	1
**9566**	0	1.80	1	0.88657	.	.	1
**9567**	0	1.80	1	0.88657	.	.	1
**9568**	0	1.80	1	0.88657	.	.	1
**9569**	0	1.80	1	0.88657	.	.	1
**9570**	0	1.80	1	0.88657	.	.	1
**9571**	0	1.80	1	0.88657	.	.	1
**9572**	0	1.80	1	0.88657	.	.	1
**9573**	0	1.80	1	0.88657	.	.	1
**9574**	0	1.80	1	0.88657	.	.	1
**9575**	0	1.80	1	0.88657	.	.	1
**9576**	0	1.80	1	0.88657	.	.	1
**9577**	0	1.80	1	0.88657	.	.	1
**9578**	0	1.80	1	0.88657	.	.	1
**9579**	0	1.80	1	0.88657	.	.	1
**9580**	0	1.80	1	0.88657	.	.	1
**9581**	0	1.80	1	0.88657	.	.	1
**9582**	0	1.80	1	0.88657	.	.	1
**9583**	0	1.80	1	0.88657	.	.	1
**9584**	0	1.81	0	0.88641	0.88338	0.88937	1
**9585**	0	1.81	1	0.88641	.	.	1
**9586**	0	1.81	1	0.88641	.	.	1
**9587**	0	1.81	1	0.88641	.	.	1
**9588**	0	1.81	1	0.88641	.	.	1
**9589**	0	1.81	1	0.88641	.	.	1
**9590**	0	1.81	1	0.88641	.	.	1

Obs	Sub_group	fuptimey	_CENSOR_	SURVIVAL	SDF_LCL	SDF_UCL	STRATUM
**9591**	0	1.81	1	0.88641	.	.	1
**9592**	0	1.81	1	0.88641	.	.	1
**9593**	0	1.81	1	0.88641	.	.	1
**9594**	0	1.81	1	0.88641	.	.	1
**9595**	0	1.81	1	0.88641	.	.	1
**9596**	0	1.81	1	0.88641	.	.	1
**9597**	0	1.81	1	0.88641	.	.	1
**9598**	0	1.81	1	0.88641	.	.	1
**9599**	0	1.81	1	0.88641	.	.	1
**9600**	0	1.81	1	0.88641	.	.	1
**9601**	0	1.81	1	0.88641	.	.	1
**9602**	0	1.81	1	0.88641	.	.	1
**9603**	0	1.81	1	0.88641	.	.	1
**9604**	0	1.81	1	0.88641	.	.	1
**9605**	0	1.81	1	0.88641	.	.	1
**9606**	0	1.81	1	0.88641	.	.	1
**9607**	0	1.81	1	0.88641	.	.	1
**9608**	0	1.81	1	0.88641	.	.	1
**9609**	0	1.81	1	0.88641	.	.	1
**9610**	0	1.81	1	0.88641	.	.	1
**9611**	0	1.81	1	0.88641	.	.	1
**9612**	0	1.81	1	0.88641	.	.	1
**9613**	0	1.81	1	0.88641	.	.	1
**9614**	0	1.81	1	0.88641	.	.	1
**9615**	0	1.81	1	0.88641	.	.	1
**9616**	0	1.81	1	0.88641	.	.	1
**9617**	0	1.81	1	0.88641	.	.	1
**9618**	0	1.81	1	0.88641	.	.	1
**9619**	0	1.81	1	0.88641	.	.	1
**9620**	0	1.81	1	0.88641	.	.	1
**9621**	0	1.81	1	0.88641	.	.	1
**9622**	0	1.81	1	0.88641	.	.	1
**9623**	0	1.81	1	0.88641	.	.	1
**9624**	0	1.81	1	0.88641	.	.	1
**9625**	0	1.81	1	0.88641	.	.	1

Obs	Sub_group	fuptimey	_CENSOR_	SURVIVAL	SDF_LCL	SDF_UCL	STRATUM
9626	0	1.81	1	0.88641	.	.	1
9627	0	1.81	1	0.88641	.	.	1
9628	0	1.81	1	0.88641	.	.	1
9629	0	1.81	1	0.88641	.	.	1
9630	0	1.81	1	0.88641	.	.	1
9631	0	1.81	1	0.88641	.	.	1
9632	0	1.81	1	0.88641	.	.	1
9633	0	1.81	1	0.88641	.	.	1
9634	0	1.81	1	0.88641	.	.	1
9635	0	1.81	1	0.88641	.	.	1
9636	0	1.81	1	0.88641	.	.	1
9637	0	1.81	1	0.88641	.	.	1
9638	0	1.81	1	0.88641	.	.	1
9639	0	1.81	1	0.88641	.	.	1
9640	0	1.81	1	0.88641	.	.	1
9641	0	1.81	1	0.88641	.	.	1
9642	0	1.81	1	0.88641	.	.	1
9643	0	1.81	1	0.88641	.	.	1
9644	0	1.81	1	0.88641	.	.	1
9645	0	1.81	1	0.88641	.	.	1
9646	0	1.81	1	0.88641	.	.	1
9647	0	1.81	1	0.88641	.	.	1
9648	0	1.82	0	0.88613	0.88310	0.88910	1
9649	0	1.82	1	0.88613	.	.	1
9650	0	1.82	1	0.88613	.	.	1
9651	0	1.82	1	0.88613	.	.	1
9652	0	1.82	1	0.88613	.	.	1
9653	0	1.82	1	0.88613	.	.	1
9654	0	1.82	1	0.88613	.	.	1
9655	0	1.82	1	0.88613	.	.	1
9656	0	1.82	1	0.88613	.	.	1
9657	0	1.82	1	0.88613	.	.	1
9658	0	1.82	1	0.88613	.	.	1
9659	0	1.82	1	0.88613	.	.	1
9660	0	1.82	1	0.88613	.	.	1

Obs	Sub_group	fuptimey	_CENSOR_	SURVIVAL	SDF_LCL	SDF_UCL	STRATUM
**9661**	0	1.82	1	0.88613	.	.	1
**9662**	0	1.82	1	0.88613	.	.	1
**9663**	0	1.82	1	0.88613	.	.	1
**9664**	0	1.82	1	0.88613	.	.	1
**9665**	0	1.82	1	0.88613	.	.	1
**9666**	0	1.82	1	0.88613	.	.	1
**9667**	0	1.82	1	0.88613	.	.	1
**9668**	0	1.82	1	0.88613	.	.	1
**9669**	0	1.82	1	0.88613	.	.	1
**9670**	0	1.82	1	0.88613	.	.	1
**9671**	0	1.82	1	0.88613	.	.	1
**9672**	0	1.82	1	0.88613	.	.	1
**9673**	0	1.82	1	0.88613	.	.	1
**9674**	0	1.82	1	0.88613	.	.	1
**9675**	0	1.82	1	0.88613	.	.	1
**9676**	0	1.82	1	0.88613	.	.	1
**9677**	0	1.82	1	0.88613	.	.	1
**9678**	0	1.82	1	0.88613	.	.	1
**9679**	0	1.82	1	0.88613	.	.	1
**9680**	0	1.82	1	0.88613	.	.	1
**9681**	0	1.82	1	0.88613	.	.	1
**9682**	0	1.82	1	0.88613	.	.	1
**9683**	0	1.82	1	0.88613	.	.	1
**9684**	0	1.82	1	0.88613	.	.	1
**9685**	0	1.82	1	0.88613	.	.	1
**9686**	0	1.82	1	0.88613	.	.	1
**9687**	0	1.82	1	0.88613	.	.	1
**9688**	0	1.82	1	0.88613	.	.	1
**9689**	0	1.82	1	0.88613	.	.	1
**9690**	0	1.82	1	0.88613	.	.	1
**9691**	0	1.82	1	0.88613	.	.	1
**9692**	0	1.83	0	0.88583	0.88279	0.88880	1
**9693**	0	1.83	1	0.88583	.	.	1
**9694**	0	1.83	1	0.88583	.	.	1
**9695**	0	1.83	1	0.88583	.	.	1

Obs	Sub_group	fuptimey	_CENSOR_	SURVIVAL	SDF_LCL	SDF_UCL	STRATUM
9696	0	1.83	1	0.88583	.	.	1
9697	0	1.83	1	0.88583	.	.	1
9698	0	1.83	1	0.88583	.	.	1
9699	0	1.83	1	0.88583	.	.	1
9700	0	1.83	1	0.88583	.	.	1
9701	0	1.83	1	0.88583	.	.	1
9702	0	1.83	1	0.88583	.	.	1
9703	0	1.83	1	0.88583	.	.	1
9704	0	1.83	1	0.88583	.	.	1
9705	0	1.83	1	0.88583	.	.	1
9706	0	1.83	1	0.88583	.	.	1
9707	0	1.83	1	0.88583	.	.	1
9708	0	1.83	1	0.88583	.	.	1
9709	0	1.83	1	0.88583	.	.	1
9710	0	1.83	1	0.88583	.	.	1
9711	0	1.83	1	0.88583	.	.	1
9712	0	1.83	1	0.88583	.	.	1
9713	0	1.83	1	0.88583	.	.	1
9714	0	1.83	1	0.88583	.	.	1
9715	0	1.83	1	0.88583	.	.	1
9716	0	1.83	1	0.88583	.	.	1
9717	0	1.83	1	0.88583	.	.	1
9718	0	1.83	1	0.88583	.	.	1
9719	0	1.83	1	0.88583	.	.	1
9720	0	1.83	1	0.88583	.	.	1
9721	0	1.83	1	0.88583	.	.	1
9722	0	1.83	1	0.88583	.	.	1
9723	0	1.83	1	0.88583	.	.	1
9724	0	1.83	1	0.88583	.	.	1
9725	0	1.83	1	0.88583	.	.	1
9726	0	1.83	1	0.88583	.	.	1
9727	0	1.83	1	0.88583	.	.	1
9728	0	1.83	1	0.88583	.	.	1
9729	0	1.83	1	0.88583	.	.	1
9730	0	1.83	1	0.88583	.	.	1

Obs	Sub_group	fuptimey	_CENSOR_	SURVIVAL	SDF_LCL	SDF_UCL	STRATUM
9731	0	1.83	1	0.88583	.	.	1
9732	0	1.83	1	0.88583	.	.	1
9733	0	1.83	1	0.88583	.	.	1
9734	0	1.83	1	0.88583	.	.	1
9735	0	1.83	1	0.88583	.	.	1
9736	0	1.83	1	0.88583	.	.	1
9737	0	1.83	1	0.88583	.	.	1
9738	0	1.83	1	0.88583	.	.	1
9739	0	1.83	1	0.88583	.	.	1
9740	0	1.83	1	0.88583	.	.	1
9741	0	1.83	1	0.88583	.	.	1
9742	0	1.83	1	0.88583	.	.	1
9743	0	1.83	1	0.88583	.	.	1
9744	0	1.83	1	0.88583	.	.	1
9745	0	1.83	1	0.88583	.	.	1
9746	0	1.83	1	0.88583	.	.	1
9747	0	1.83	1	0.88583	.	.	1
9748	0	1.83	1	0.88583	.	.	1
9749	0	1.83	1	0.88583	.	.	1
9750	0	1.83	1	0.88583	.	.	1
9751	0	1.83	1	0.88583	.	.	1
9752	0	1.83	1	0.88583	.	.	1
9753	0	1.83	1	0.88583	.	.	1
9754	0	1.83	1	0.88583	.	.	1
9755	0	1.83	1	0.88583	.	.	1
9756	0	1.83	1	0.88583	.	.	1
9757	0	1.83	1	0.88583	.	.	1
9758	0	1.83	1	0.88583	.	.	1
9759	0	1.83	1	0.88583	.	.	1
9760	0	1.83	1	0.88583	.	.	1
9761	0	1.83	1	0.88583	.	.	1
9762	0	1.83	1	0.88583	.	.	1
9763	0	1.83	1	0.88583	.	.	1
9764	0	1.84	0	0.88547	0.88243	0.88844	1
9765	0	1.84	1	0.88547	.	.	1

Obs	Sub_group	fuptimey	_CENSOR_	SURVIVAL	SDF_LCL	SDF_UCL	STRATUM
9766	0	1.84	1	0.88547	.	.	1
9767	0	1.84	1	0.88547	.	.	1
9768	0	1.84	1	0.88547	.	.	1
9769	0	1.84	1	0.88547	.	.	1
9770	0	1.84	1	0.88547	.	.	1
9771	0	1.84	1	0.88547	.	.	1
9772	0	1.84	1	0.88547	.	.	1
9773	0	1.84	1	0.88547	.	.	1
9774	0	1.84	1	0.88547	.	.	1
9775	0	1.84	1	0.88547	.	.	1
9776	0	1.84	1	0.88547	.	.	1
9777	0	1.84	1	0.88547	.	.	1
9778	0	1.84	1	0.88547	.	.	1
9779	0	1.84	1	0.88547	.	.	1
9780	0	1.84	1	0.88547	.	.	1
9781	0	1.84	1	0.88547	.	.	1
9782	0	1.84	1	0.88547	.	.	1
9783	0	1.84	1	0.88547	.	.	1
9784	0	1.84	1	0.88547	.	.	1
9785	0	1.84	1	0.88547	.	.	1
9786	0	1.84	1	0.88547	.	.	1
9787	0	1.84	1	0.88547	.	.	1
9788	0	1.84	1	0.88547	.	.	1
9789	0	1.84	1	0.88547	.	.	1
9790	0	1.84	1	0.88547	.	.	1
9791	0	1.84	1	0.88547	.	.	1
9792	0	1.84	1	0.88547	.	.	1
9793	0	1.84	1	0.88547	.	.	1
9794	0	1.84	1	0.88547	.	.	1
9795	0	1.84	1	0.88547	.	.	1
9796	0	1.84	1	0.88547	.	.	1
9797	0	1.84	1	0.88547	.	.	1
9798	0	1.84	1	0.88547	.	.	1
9799	0	1.84	1	0.88547	.	.	1
9800	0	1.84	1	0.88547	.	.	1

Obs	Sub_group	fuptimey	_CENSOR_	SURVIVAL	SDF_LCL	SDF_UCL	STRATUM
9801	0	1.84	1	0.88547	.	.	1
9802	0	1.84	1	0.88547	.	.	1
9803	0	1.84	1	0.88547	.	.	1
9804	0	1.84	1	0.88547	.	.	1
9805	0	1.84	1	0.88547	.	.	1
9806	0	1.84	1	0.88547	.	.	1
9807	0	1.84	1	0.88547	.	.	1
9808	0	1.84	1	0.88547	.	.	1
9809	0	1.84	1	0.88547	.	.	1
9810	0	1.84	1	0.88547	.	.	1
9811	0	1.85	0	0.88517	0.88212	0.88814	1
9812	0	1.85	1	0.88517	.	.	1
9813	0	1.85	1	0.88517	.	.	1
9814	0	1.85	1	0.88517	.	.	1
9815	0	1.85	1	0.88517	.	.	1
9816	0	1.85	1	0.88517	.	.	1
9817	0	1.85	1	0.88517	.	.	1
9818	0	1.85	1	0.88517	.	.	1
9819	0	1.85	1	0.88517	.	.	1
9820	0	1.85	1	0.88517	.	.	1
9821	0	1.85	1	0.88517	.	.	1
9822	0	1.85	1	0.88517	.	.	1
9823	0	1.85	1	0.88517	.	.	1
9824	0	1.85	1	0.88517	.	.	1
9825	0	1.85	1	0.88517	.	.	1
9826	0	1.85	1	0.88517	.	.	1
9827	0	1.85	1	0.88517	.	.	1
9828	0	1.85	1	0.88517	.	.	1
9829	0	1.85	1	0.88517	.	.	1
9830	0	1.85	1	0.88517	.	.	1
9831	0	1.85	1	0.88517	.	.	1
9832	0	1.85	1	0.88517	.	.	1
9833	0	1.85	1	0.88517	.	.	1
9834	0	1.85	1	0.88517	.	.	1
9835	0	1.85	1	0.88517	.	.	1

Obs	Sub_group	fuptimey	_CENSOR_	SURVIVAL	SDF_LCL	SDF_UCL	STRATUM
**9836**	0	1.85	1	0.88517	.	.	1
**9837**	0	1.85	1	0.88517	.	.	1
**9838**	0	1.85	1	0.88517	.	.	1
**9839**	0	1.85	1	0.88517	.	.	1
**9840**	0	1.85	1	0.88517	.	.	1
**9841**	0	1.85	1	0.88517	.	.	1
**9842**	0	1.85	1	0.88517	.	.	1
**9843**	0	1.85	1	0.88517	.	.	1
**9844**	0	1.85	1	0.88517	.	.	1
**9845**	0	1.85	1	0.88517	.	.	1
**9846**	0	1.85	1	0.88517	.	.	1
**9847**	0	1.85	1	0.88517	.	.	1
**9848**	0	1.85	1	0.88517	.	.	1
**9849**	0	1.85	1	0.88517	.	.	1
**9850**	0	1.85	1	0.88517	.	.	1
**9851**	0	1.85	1	0.88517	.	.	1
**9852**	0	1.85	1	0.88517	.	.	1
**9853**	0	1.85	1	0.88517	.	.	1
**9854**	0	1.85	1	0.88517	.	.	1
**9855**	0	1.85	1	0.88517	.	.	1
**9856**	0	1.85	1	0.88517	.	.	1
**9857**	0	1.85	1	0.88517	.	.	1
**9858**	0	1.85	1	0.88517	.	.	1
**9859**	0	1.85	1	0.88517	.	.	1
**9860**	0	1.85	1	0.88517	.	.	1
**9861**	0	1.85	1	0.88517	.	.	1
**9862**	0	1.85	1	0.88517	.	.	1
**9863**	0	1.85	1	0.88517	.	.	1
**9864**	0	1.85	1	0.88517	.	.	1
**9865**	0	1.85	1	0.88517	.	.	1
**9866**	0	1.85	1	0.88517	.	.	1
**9867**	0	1.85	1	0.88517	.	.	1
**9868**	0	1.85	1	0.88517	.	.	1
**9869**	0	1.85	1	0.88517	.	.	1
**9870**	0	1.85	1	0.88517	.	.	1

Obs	Sub_group	fuptimey	_CENSOR_	SURVIVAL	SDF_LCL	SDF_UCL	STRATUM
9871	0	1.86	0	0.88489	0.88184	0.88787	1
9872	0	1.86	1	0.88489	.	.	1
9873	0	1.86	1	0.88489	.	.	1
9874	0	1.86	1	0.88489	.	.	1
9875	0	1.86	1	0.88489	.	.	1
9876	0	1.86	1	0.88489	.	.	1
9877	0	1.86	1	0.88489	.	.	1
9878	0	1.86	1	0.88489	.	.	1
9879	0	1.86	1	0.88489	.	.	1
9880	0	1.86	1	0.88489	.	.	1
9881	0	1.86	1	0.88489	.	.	1
9882	0	1.86	1	0.88489	.	.	1
9883	0	1.86	1	0.88489	.	.	1
9884	0	1.86	1	0.88489	.	.	1
9885	0	1.86	1	0.88489	.	.	1
9886	0	1.86	1	0.88489	.	.	1
9887	0	1.86	1	0.88489	.	.	1
9888	0	1.86	1	0.88489	.	.	1
9889	0	1.86	1	0.88489	.	.	1
9890	0	1.86	1	0.88489	.	.	1
9891	0	1.86	1	0.88489	.	.	1
9892	0	1.86	1	0.88489	.	.	1
9893	0	1.86	1	0.88489	.	.	1
9894	0	1.86	1	0.88489	.	.	1
9895	0	1.86	1	0.88489	.	.	1
9896	0	1.86	1	0.88489	.	.	1
9897	0	1.86	1	0.88489	.	.	1
9898	0	1.86	1	0.88489	.	.	1
9899	0	1.86	1	0.88489	.	.	1
9900	0	1.86	1	0.88489	.	.	1
9901	0	1.86	1	0.88489	.	.	1
9902	0	1.86	1	0.88489	.	.	1
9903	0	1.86	1	0.88489	.	.	1
9904	0	1.86	1	0.88489	.	.	1
9905	0	1.86	1	0.88489	.	.	1

Obs	Sub_group	fuptimey	_CENSOR_	SURVIVAL	SDF_LCL	SDF_UCL	STRATUM
**9906**	0	1.86	1	0.88489	.	.	1
**9907**	0	1.86	1	0.88489	.	.	1
**9908**	0	1.86	1	0.88489	.	.	1
**9909**	0	1.86	1	0.88489	.	.	1
**9910**	0	1.86	1	0.88489	.	.	1
**9911**	0	1.86	1	0.88489	.	.	1
**9912**	0	1.86	1	0.88489	.	.	1
**9913**	0	1.86	1	0.88489	.	.	1
**9914**	0	1.86	1	0.88489	.	.	1
**9915**	0	1.86	1	0.88489	.	.	1
**9916**	0	1.86	1	0.88489	.	.	1
**9917**	0	1.86	1	0.88489	.	.	1
**9918**	0	1.87	0	0.88464	0.88158	0.88763	1
**9919**	0	1.87	1	0.88464	.	.	1
**9920**	0	1.87	1	0.88464	.	.	1
**9921**	0	1.87	1	0.88464	.	.	1
**9922**	0	1.87	1	0.88464	.	.	1
**9923**	0	1.87	1	0.88464	.	.	1
**9924**	0	1.87	1	0.88464	.	.	1
**9925**	0	1.87	1	0.88464	.	.	1
**9926**	0	1.87	1	0.88464	.	.	1
**9927**	0	1.87	1	0.88464	.	.	1
**9928**	0	1.87	1	0.88464	.	.	1
**9929**	0	1.87	1	0.88464	.	.	1
**9930**	0	1.87	1	0.88464	.	.	1
**9931**	0	1.87	1	0.88464	.	.	1
**9932**	0	1.87	1	0.88464	.	.	1
**9933**	0	1.87	1	0.88464	.	.	1
**9934**	0	1.87	1	0.88464	.	.	1
**9935**	0	1.87	1	0.88464	.	.	1
**9936**	0	1.87	1	0.88464	.	.	1
**9937**	0	1.87	1	0.88464	.	.	1
**9938**	0	1.87	1	0.88464	.	.	1
**9939**	0	1.87	1	0.88464	.	.	1
**9940**	0	1.87	1	0.88464	.	.	1

Obs	Sub_group	fuptimey	_CENSOR_	SURVIVAL	SDF_LCL	SDF_UCL	STRATUM
**9941**	0	1.87	1	0.88464	.	.	1
**9942**	0	1.87	1	0.88464	.	.	1
**9943**	0	1.87	1	0.88464	.	.	1
**9944**	0	1.87	1	0.88464	.	.	1
**9945**	0	1.87	1	0.88464	.	.	1
**9946**	0	1.87	1	0.88464	.	.	1
**9947**	0	1.87	1	0.88464	.	.	1
**9948**	0	1.87	1	0.88464	.	.	1
**9949**	0	1.87	1	0.88464	.	.	1
**9950**	0	1.87	1	0.88464	.	.	1
**9951**	0	1.87	1	0.88464	.	.	1
**9952**	0	1.87	1	0.88464	.	.	1
**9953**	0	1.87	1	0.88464	.	.	1
**9954**	0	1.87	1	0.88464	.	.	1
**9955**	0	1.87	1	0.88464	.	.	1
**9956**	0	1.87	1	0.88464	.	.	1
**9957**	0	1.88	0	0.88434	0.88127	0.88733	1
**9958**	0	1.88	1	0.88434	.	.	1
**9959**	0	1.88	1	0.88434	.	.	1
**9960**	0	1.88	1	0.88434	.	.	1
**9961**	0	1.88	1	0.88434	.	.	1
**9962**	0	1.88	1	0.88434	.	.	1
**9963**	0	1.88	1	0.88434	.	.	1
**9964**	0	1.88	1	0.88434	.	.	1
**9965**	0	1.88	1	0.88434	.	.	1
**9966**	0	1.88	1	0.88434	.	.	1
**9967**	0	1.88	1	0.88434	.	.	1
**9968**	0	1.88	1	0.88434	.	.	1
**9969**	0	1.88	1	0.88434	.	.	1
**9970**	0	1.88	1	0.88434	.	.	1
**9971**	0	1.88	1	0.88434	.	.	1
**9972**	0	1.88	1	0.88434	.	.	1
**9973**	0	1.88	1	0.88434	.	.	1
**9974**	0	1.88	1	0.88434	.	.	1
**9975**	0	1.88	1	0.88434	.	.	1

Obs	Sub_group	fuptimey	_CENSOR_	SURVIVAL	SDF_LCL	SDF_UCL	STRATUM
9976	0	1.88	1	0.88434	.	.	1
9977	0	1.88	1	0.88434	.	.	1
9978	0	1.88	1	0.88434	.	.	1
9979	0	1.88	1	0.88434	.	.	1
9980	0	1.88	1	0.88434	.	.	1
9981	0	1.88	1	0.88434	.	.	1
9982	0	1.88	1	0.88434	.	.	1
9983	0	1.88	1	0.88434	.	.	1
9984	0	1.88	1	0.88434	.	.	1
9985	0	1.88	1	0.88434	.	.	1
9986	0	1.88	1	0.88434	.	.	1
9987	0	1.88	1	0.88434	.	.	1
9988	0	1.88	1	0.88434	.	.	1
9989	0	1.88	1	0.88434	.	.	1
9990	0	1.88	1	0.88434	.	.	1
9991	0	1.88	1	0.88434	.	.	1
9992	0	1.88	1	0.88434	.	.	1
9993	0	1.88	1	0.88434	.	.	1
9994	0	1.88	1	0.88434	.	.	1
9995	0	1.88	1	0.88434	.	.	1
9996	0	1.88	1	0.88434	.	.	1
9997	0	1.88	1	0.88434	.	.	1
9998	0	1.88	1	0.88434	.	.	1
9999	0	1.88	1	0.88434	.	.	1
10000	0	1.88	1	0.88434	.	.	1
10001	0	1.88	1	0.88434	.	.	1
10002	0	1.88	1	0.88434	.	.	1
10003	0	1.89	0	0.88409	0.88102	0.88708	1
10004	0	1.89	1	0.88409	.	.	1
10005	0	1.89	1	0.88409	.	.	1
10006	0	1.89	1	0.88409	.	.	1
10007	0	1.89	1	0.88409	.	.	1
10008	0	1.89	1	0.88409	.	.	1
10009	0	1.89	1	0.88409	.	.	1
10010	0	1.89	1	0.88409	.	.	1

Obs	Sub_group	fuptimey	_CENSOR_	SURVIVAL	SDF_LCL	SDF_UCL	STRATUM
10011	0	1.89	1	0.88409	.	.	1
10012	0	1.89	1	0.88409	.	.	1
10013	0	1.89	1	0.88409	.	.	1
10014	0	1.89	1	0.88409	.	.	1
10015	0	1.89	1	0.88409	.	.	1
10016	0	1.89	1	0.88409	.	.	1
10017	0	1.89	1	0.88409	.	.	1
10018	0	1.89	1	0.88409	.	.	1
10019	0	1.89	1	0.88409	.	.	1
10020	0	1.89	1	0.88409	.	.	1
10021	0	1.89	1	0.88409	.	.	1
10022	0	1.89	1	0.88409	.	.	1
10023	0	1.89	1	0.88409	.	.	1
10024	0	1.89	1	0.88409	.	.	1
10025	0	1.89	1	0.88409	.	.	1
10026	0	1.89	1	0.88409	.	.	1
10027	0	1.89	1	0.88409	.	.	1
10028	0	1.89	1	0.88409	.	.	1
10029	0	1.89	1	0.88409	.	.	1
10030	0	1.89	1	0.88409	.	.	1
10031	0	1.89	1	0.88409	.	.	1
10032	0	1.89	1	0.88409	.	.	1
10033	0	1.89	1	0.88409	.	.	1
10034	0	1.89	1	0.88409	.	.	1
10035	0	1.89	1	0.88409	.	.	1
10036	0	1.89	1	0.88409	.	.	1
10037	0	1.89	1	0.88409	.	.	1
10038	0	1.89	1	0.88409	.	.	1
10039	0	1.89	1	0.88409	.	.	1
10040	0	1.89	1	0.88409	.	.	1
10041	0	1.89	1	0.88409	.	.	1
10042	0	1.89	1	0.88409	.	.	1
10043	0	1.89	1	0.88409	.	.	1
10044	0	1.89	1	0.88409	.	.	1
10045	0	1.89	1	0.88409	.	.	1

Obs	Sub_group	fuptimey	_CENSOR_	SURVIVAL	SDF_LCL	SDF_UCL	STRATUM
10046	0	1.89	1	0.88409	.	.	1
10047	0	1.89	1	0.88409	.	.	1
10048	0	1.90	0	0.88378	0.88071	0.88678	1
10049	0	1.90	1	0.88378	.	.	1
10050	0	1.90	1	0.88378	.	.	1
10051	0	1.90	1	0.88378	.	.	1
10052	0	1.90	1	0.88378	.	.	1
10053	0	1.90	1	0.88378	.	.	1
10054	0	1.90	1	0.88378	.	.	1
10055	0	1.90	1	0.88378	.	.	1
10056	0	1.90	1	0.88378	.	.	1
10057	0	1.90	1	0.88378	.	.	1
10058	0	1.90	1	0.88378	.	.	1
10059	0	1.90	1	0.88378	.	.	1
10060	0	1.90	1	0.88378	.	.	1
10061	0	1.90	1	0.88378	.	.	1
10062	0	1.90	1	0.88378	.	.	1
10063	0	1.90	1	0.88378	.	.	1
10064	0	1.90	1	0.88378	.	.	1
10065	0	1.90	1	0.88378	.	.	1
10066	0	1.90	1	0.88378	.	.	1
10067	0	1.90	1	0.88378	.	.	1
10068	0	1.90	1	0.88378	.	.	1
10069	0	1.90	1	0.88378	.	.	1
10070	0	1.90	1	0.88378	.	.	1
10071	0	1.90	1	0.88378	.	.	1
10072	0	1.90	1	0.88378	.	.	1
10073	0	1.90	1	0.88378	.	.	1
10074	0	1.90	1	0.88378	.	.	1
10075	0	1.90	1	0.88378	.	.	1
10076	0	1.90	1	0.88378	.	.	1
10077	0	1.90	1	0.88378	.	.	1
10078	0	1.90	1	0.88378	.	.	1
10079	0	1.90	1	0.88378	.	.	1
10080	0	1.90	1	0.88378	.	.	1

Obs	Sub_group	fuptimey	_CENSOR_	SURVIVAL	SDF_LCL	SDF_UCL	STRATUM
**10081**	0	1.90	1	0.88378	.	.	1
**10082**	0	1.90	1	0.88378	.	.	1
**10083**	0	1.90	1	0.88378	.	.	1
**10084**	0	1.90	1	0.88378	.	.	1
**10085**	0	1.90	1	0.88378	.	.	1
**10086**	0	1.90	1	0.88378	.	.	1
**10087**	0	1.90	1	0.88378	.	.	1
**10088**	0	1.90	1	0.88378	.	.	1
**10089**	0	1.90	1	0.88378	.	.	1
**10090**	0	1.90	1	0.88378	.	.	1
**10091**	0	1.90	1	0.88378	.	.	1
**10092**	0	1.91	0	0.88359	0.88051	0.88658	1
**10093**	0	1.91	1	0.88359	.	.	1
**10094**	0	1.91	1	0.88359	.	.	1
**10095**	0	1.91	1	0.88359	.	.	1
**10096**	0	1.91	1	0.88359	.	.	1
**10097**	0	1.91	1	0.88359	.	.	1
**10098**	0	1.91	1	0.88359	.	.	1
**10099**	0	1.91	1	0.88359	.	.	1
**10100**	0	1.91	1	0.88359	.	.	1
**10101**	0	1.91	1	0.88359	.	.	1
**10102**	0	1.91	1	0.88359	.	.	1
**10103**	0	1.91	1	0.88359	.	.	1
**10104**	0	1.91	1	0.88359	.	.	1
**10105**	0	1.91	1	0.88359	.	.	1
**10106**	0	1.91	1	0.88359	.	.	1
**10107**	0	1.91	1	0.88359	.	.	1
**10108**	0	1.91	1	0.88359	.	.	1
**10109**	0	1.91	1	0.88359	.	.	1
**10110**	0	1.91	1	0.88359	.	.	1
**10111**	0	1.91	1	0.88359	.	.	1
**10112**	0	1.91	1	0.88359	.	.	1
**10113**	0	1.91	1	0.88359	.	.	1
**10114**	0	1.91	1	0.88359	.	.	1
**10115**	0	1.91	1	0.88359	.	.	1

Obs	Sub_group	fuptimey	_CENSOR_	SURVIVAL	SDF_LCL	SDF_UCL	STRATUM
10116	0	1.91	1	0.88359	.	.	1
10117	0	1.91	1	0.88359	.	.	1
10118	0	1.91	1	0.88359	.	.	1
10119	0	1.91	1	0.88359	.	.	1
10120	0	1.91	1	0.88359	.	.	1
10121	0	1.91	1	0.88359	.	.	1
10122	0	1.91	1	0.88359	.	.	1
10123	0	1.91	1	0.88359	.	.	1
10124	0	1.91	1	0.88359	.	.	1
10125	0	1.91	1	0.88359	.	.	1
10126	0	1.91	1	0.88359	.	.	1
10127	0	1.91	1	0.88359	.	.	1
10128	0	1.91	1	0.88359	.	.	1
10129	0	1.92	0	0.88328	0.88020	0.88628	1
10130	0	1.92	1	0.88328	.	.	1
10131	0	1.92	1	0.88328	.	.	1
10132	0	1.92	1	0.88328	.	.	1
10133	0	1.92	1	0.88328	.	.	1
10134	0	1.92	1	0.88328	.	.	1
10135	0	1.92	1	0.88328	.	.	1
10136	0	1.92	1	0.88328	.	.	1
10137	0	1.92	1	0.88328	.	.	1
10138	0	1.92	1	0.88328	.	.	1
10139	0	1.92	1	0.88328	.	.	1
10140	0	1.92	1	0.88328	.	.	1
10141	0	1.92	1	0.88328	.	.	1
10142	0	1.92	1	0.88328	.	.	1
10143	0	1.92	1	0.88328	.	.	1
10144	0	1.92	1	0.88328	.	.	1
10145	0	1.92	1	0.88328	.	.	1
10146	0	1.92	1	0.88328	.	.	1
10147	0	1.92	1	0.88328	.	.	1
10148	0	1.92	1	0.88328	.	.	1
10149	0	1.92	1	0.88328	.	.	1
10150	0	1.92	1	0.88328	.	.	1

Obs	Sub_group	fuptimey	_CENSOR_	SURVIVAL	SDF_LCL	SDF_UCL	STRATUM
**10151**	0	1.92	1	0.88328	.	.	1
**10152**	0	1.92	1	0.88328	.	.	1
**10153**	0	1.92	1	0.88328	.	.	1
**10154**	0	1.92	1	0.88328	.	.	1
**10155**	0	1.92	1	0.88328	.	.	1
**10156**	0	1.92	1	0.88328	.	.	1
**10157**	0	1.92	1	0.88328	.	.	1
**10158**	0	1.92	1	0.88328	.	.	1
**10159**	0	1.92	1	0.88328	.	.	1
**10160**	0	1.92	1	0.88328	.	.	1
**10161**	0	1.92	1	0.88328	.	.	1
**10162**	0	1.92	1	0.88328	.	.	1
**10163**	0	1.92	1	0.88328	.	.	1
**10164**	0	1.92	1	0.88328	.	.	1
**10165**	0	1.92	1	0.88328	.	.	1
**10166**	0	1.92	1	0.88328	.	.	1
**10167**	0	1.92	1	0.88328	.	.	1
**10168**	0	1.92	1	0.88328	.	.	1
**10169**	0	1.92	1	0.88328	.	.	1
**10170**	0	1.92	1	0.88328	.	.	1
**10171**	0	1.92	1	0.88328	.	.	1
**10172**	0	1.92	1	0.88328	.	.	1
**10173**	0	1.92	1	0.88328	.	.	1
**10174**	0	1.92	1	0.88328	.	.	1
**10175**	0	1.92	1	0.88328	.	.	1
**10176**	0	1.92	1	0.88328	.	.	1
**10177**	0	1.92	1	0.88328	.	.	1
**10178**	0	1.92	1	0.88328	.	.	1
**10179**	0	1.92	1	0.88328	.	.	1
**10180**	0	1.92	1	0.88328	.	.	1
**10181**	0	1.93	0	0.88294	0.87986	0.88595	1
**10182**	0	1.93	1	0.88294	.	.	1
**10183**	0	1.93	1	0.88294	.	.	1
**10184**	0	1.93	1	0.88294	.	.	1
**10185**	0	1.93	1	0.88294	.	.	1

Obs	Sub_group	fuptimey	_CENSOR_	SURVIVAL	SDF_LCL	SDF_UCL	STRATUM
10186	0	1.93	1	0.88294	.	.	1
10187	0	1.93	1	0.88294	.	.	1
10188	0	1.93	1	0.88294	.	.	1
10189	0	1.93	1	0.88294	.	.	1
10190	0	1.93	1	0.88294	.	.	1
10191	0	1.93	1	0.88294	.	.	1
10192	0	1.93	1	0.88294	.	.	1
10193	0	1.93	1	0.88294	.	.	1
10194	0	1.93	1	0.88294	.	.	1
10195	0	1.93	1	0.88294	.	.	1
10196	0	1.93	1	0.88294	.	.	1
10197	0	1.93	1	0.88294	.	.	1
10198	0	1.93	1	0.88294	.	.	1
10199	0	1.93	1	0.88294	.	.	1
10200	0	1.93	1	0.88294	.	.	1
10201	0	1.93	1	0.88294	.	.	1
10202	0	1.93	1	0.88294	.	.	1
10203	0	1.93	1	0.88294	.	.	1
10204	0	1.93	1	0.88294	.	.	1
10205	0	1.93	1	0.88294	.	.	1
10206	0	1.93	1	0.88294	.	.	1
10207	0	1.93	1	0.88294	.	.	1
10208	0	1.93	1	0.88294	.	.	1
10209	0	1.93	1	0.88294	.	.	1
10210	0	1.93	1	0.88294	.	.	1
10211	0	1.93	1	0.88294	.	.	1
10212	0	1.93	1	0.88294	.	.	1
10213	0	1.93	1	0.88294	.	.	1
10214	0	1.93	1	0.88294	.	.	1
10215	0	1.94	0	0.88261	0.87952	0.88562	1
10216	0	1.94	1	0.88261	.	.	1
10217	0	1.94	1	0.88261	.	.	1
10218	0	1.94	1	0.88261	.	.	1
10219	0	1.94	1	0.88261	.	.	1
10220	0	1.94	1	0.88261	.	.	1

Obs	Sub_group	fuptimey	_CENSOR_	SURVIVAL	SDF_LCL	SDF_UCL	STRATUM
10221	0	1.94	1	0.88261	.	.	1
10222	0	1.94	1	0.88261	.	.	1
10223	0	1.94	1	0.88261	.	.	1
10224	0	1.94	1	0.88261	.	.	1
10225	0	1.94	1	0.88261	.	.	1
10226	0	1.94	1	0.88261	.	.	1
10227	0	1.94	1	0.88261	.	.	1
10228	0	1.94	1	0.88261	.	.	1
10229	0	1.94	1	0.88261	.	.	1
10230	0	1.94	1	0.88261	.	.	1
10231	0	1.94	1	0.88261	.	.	1
10232	0	1.94	1	0.88261	.	.	1
10233	0	1.94	1	0.88261	.	.	1
10234	0	1.94	1	0.88261	.	.	1
10235	0	1.94	1	0.88261	.	.	1
10236	0	1.94	1	0.88261	.	.	1
10237	0	1.94	1	0.88261	.	.	1
10238	0	1.94	1	0.88261	.	.	1
10239	0	1.94	1	0.88261	.	.	1
10240	0	1.94	1	0.88261	.	.	1
10241	0	1.94	1	0.88261	.	.	1
10242	0	1.94	1	0.88261	.	.	1
10243	0	1.94	1	0.88261	.	.	1
10244	0	1.94	1	0.88261	.	.	1
10245	0	1.94	1	0.88261	.	.	1
10246	0	1.94	1	0.88261	.	.	1
10247	0	1.94	1	0.88261	.	.	1
10248	0	1.94	1	0.88261	.	.	1
10249	0	1.94	1	0.88261	.	.	1
10250	0	1.94	1	0.88261	.	.	1
10251	0	1.94	1	0.88261	.	.	1
10252	0	1.94	1	0.88261	.	.	1
10253	0	1.94	1	0.88261	.	.	1
10254	0	1.94	1	0.88261	.	.	1
10255	0	1.94	1	0.88261	.	.	1

Obs	Sub_group	fuptimey	_CENSOR_	SURVIVAL	SDF_LCL	SDF_UCL	STRATUM
10256	0	1.94	1	0.88261	.	.	1
10257	0	1.94	1	0.88261	.	.	1
10258	0	1.94	1	0.88261	.	.	1
10259	0	1.94	1	0.88261	.	.	1
10260	0	1.94	1	0.88261	.	.	1
10261	0	1.94	1	0.88261	.	.	1
10262	0	1.94	1	0.88261	.	.	1
10263	0	1.94	1	0.88261	.	.	1
10264	0	1.95	0	0.88233	0.87924	0.88534	1
10265	0	1.95	1	0.88233	.	.	1
10266	0	1.95	1	0.88233	.	.	1
10267	0	1.95	1	0.88233	.	.	1
10268	0	1.95	1	0.88233	.	.	1
10269	0	1.95	1	0.88233	.	.	1
10270	0	1.95	1	0.88233	.	.	1
10271	0	1.95	1	0.88233	.	.	1
10272	0	1.95	1	0.88233	.	.	1
10273	0	1.95	1	0.88233	.	.	1
10274	0	1.95	1	0.88233	.	.	1
10275	0	1.95	1	0.88233	.	.	1
10276	0	1.95	1	0.88233	.	.	1
10277	0	1.95	1	0.88233	.	.	1
10278	0	1.95	1	0.88233	.	.	1
10279	0	1.95	1	0.88233	.	.	1
10280	0	1.95	1	0.88233	.	.	1
10281	0	1.95	1	0.88233	.	.	1
10282	0	1.95	1	0.88233	.	.	1
10283	0	1.95	1	0.88233	.	.	1
10284	0	1.95	1	0.88233	.	.	1
10285	0	1.95	1	0.88233	.	.	1
10286	0	1.95	1	0.88233	.	.	1
10287	0	1.95	1	0.88233	.	.	1
10288	0	1.95	1	0.88233	.	.	1
10289	0	1.95	1	0.88233	.	.	1
10290	0	1.95	1	0.88233	.	.	1

Obs	Sub_group	fuptimey	_CENSOR_	SURVIVAL	SDF_LCL	SDF_UCL	STRATUM
**10291**	0	1.95	1	0.88233	.	.	1
**10292**	0	1.95	1	0.88233	.	.	1
**10293**	0	1.95	1	0.88233	.	.	1
**10294**	0	1.95	1	0.88233	.	.	1
**10295**	0	1.95	1	0.88233	.	.	1
**10296**	0	1.95	1	0.88233	.	.	1
**10297**	0	1.95	1	0.88233	.	.	1
**10298**	0	1.95	1	0.88233	.	.	1
**10299**	0	1.95	1	0.88233	.	.	1
**10300**	0	1.95	1	0.88233	.	.	1
**10301**	0	1.96	0	0.88207	0.87898	0.88510	1
**10302**	0	1.96	1	0.88207	.	.	1
**10303**	0	1.96	1	0.88207	.	.	1
**10304**	0	1.96	1	0.88207	.	.	1
**10305**	0	1.96	1	0.88207	.	.	1
**10306**	0	1.96	1	0.88207	.	.	1
**10307**	0	1.96	1	0.88207	.	.	1
**10308**	0	1.96	1	0.88207	.	.	1
**10309**	0	1.96	1	0.88207	.	.	1
**10310**	0	1.96	1	0.88207	.	.	1
**10311**	0	1.96	1	0.88207	.	.	1
**10312**	0	1.96	1	0.88207	.	.	1
**10313**	0	1.96	1	0.88207	.	.	1
**10314**	0	1.96	1	0.88207	.	.	1
**10315**	0	1.96	1	0.88207	.	.	1
**10316**	0	1.96	1	0.88207	.	.	1
**10317**	0	1.96	1	0.88207	.	.	1
**10318**	0	1.96	1	0.88207	.	.	1
**10319**	0	1.96	1	0.88207	.	.	1
**10320**	0	1.96	1	0.88207	.	.	1
**10321**	0	1.96	1	0.88207	.	.	1
**10322**	0	1.96	1	0.88207	.	.	1
**10323**	0	1.96	1	0.88207	.	.	1
**10324**	0	1.96	1	0.88207	.	.	1
**10325**	0	1.96	1	0.88207	.	.	1

Obs	Sub_group	fuptimey	_CENSOR_	SURVIVAL	SDF_LCL	SDF_UCL	STRATUM
10326	0	1.96	1	0.88207	.	.	1
10327	0	1.96	1	0.88207	.	.	1
10328	0	1.96	1	0.88207	.	.	1
10329	0	1.96	1	0.88207	.	.	1
10330	0	1.96	1	0.88207	.	.	1
10331	0	1.96	1	0.88207	.	.	1
10332	0	1.96	1	0.88207	.	.	1
10333	0	1.96	1	0.88207	.	.	1
10334	0	1.96	1	0.88207	.	.	1
10335	0	1.96	1	0.88207	.	.	1
10336	0	1.96	1	0.88207	.	.	1
10337	0	1.96	1	0.88207	.	.	1
10338	0	1.96	1	0.88207	.	.	1
10339	0	1.96	1	0.88207	.	.	1
10340	0	1.96	1	0.88207	.	.	1
10341	0	1.96	1	0.88207	.	.	1
10342	0	1.96	1	0.88207	.	.	1
10343	0	1.96	1	0.88207	.	.	1
10344	0	1.96	1	0.88207	.	.	1
10345	0	1.96	1	0.88207	.	.	1
10346	0	1.96	1	0.88207	.	.	1
10347	0	1.96	1	0.88207	.	.	1
10348	0	1.96	1	0.88207	.	.	1
10349	0	1.96	1	0.88207	.	.	1
10350	0	1.96	1	0.88207	.	.	1
10351	0	1.96	1	0.88207	.	.	1
10352	0	1.96	1	0.88207	.	.	1
10353	0	1.96	1	0.88207	.	.	1
10354	0	1.96	1	0.88207	.	.	1
10355	0	1.96	1	0.88207	.	.	1
10356	0	1.96	1	0.88207	.	.	1
10357	0	1.96	1	0.88207	.	.	1
10358	0	1.96	1	0.88207	.	.	1
10359	0	1.96	1	0.88207	.	.	1
10360	0	1.96	1	0.88207	.	.	1

Obs	Sub_group	fuptimey	_CENSOR_	SURVIVAL	SDF_LCL	SDF_UCL	STRATUM
10361	0	1.96	1	0.88207	.	.	1
10362	0	1.96	1	0.88207	.	.	1
10363	0	1.96	1	0.88207	.	.	1
10364	0	1.96	1	0.88207	.	.	1
10365	0	1.96	1	0.88207	.	.	1
10366	0	1.96	1	0.88207	.	.	1
10367	0	1.96	1	0.88207	.	.	1
10368	0	1.96	1	0.88207	.	.	1
10369	0	1.96	1	0.88207	.	.	1
10370	0	1.97	0	0.88191	0.87881	0.88493	1
10371	0	1.97	1	0.88191	.	.	1
10372	0	1.97	1	0.88191	.	.	1
10373	0	1.97	1	0.88191	.	.	1
10374	0	1.97	1	0.88191	.	.	1
10375	0	1.97	1	0.88191	.	.	1
10376	0	1.97	1	0.88191	.	.	1
10377	0	1.97	1	0.88191	.	.	1
10378	0	1.97	1	0.88191	.	.	1
10379	0	1.97	1	0.88191	.	.	1
10380	0	1.97	1	0.88191	.	.	1
10381	0	1.97	1	0.88191	.	.	1
10382	0	1.97	1	0.88191	.	.	1
10383	0	1.97	1	0.88191	.	.	1
10384	0	1.97	1	0.88191	.	.	1
10385	0	1.97	1	0.88191	.	.	1
10386	0	1.97	1	0.88191	.	.	1
10387	0	1.97	1	0.88191	.	.	1
10388	0	1.97	1	0.88191	.	.	1
10389	0	1.97	1	0.88191	.	.	1
10390	0	1.97	1	0.88191	.	.	1
10391	0	1.97	1	0.88191	.	.	1
10392	0	1.97	1	0.88191	.	.	1
10393	0	1.97	1	0.88191	.	.	1
10394	0	1.97	1	0.88191	.	.	1
10395	0	1.97	1	0.88191	.	.	1

Obs	Sub_group	fuptimey	_CENSOR_	SURVIVAL	SDF_LCL	SDF_UCL	STRATUM
10396	0	1.97	1	0.88191	.	.	1
10397	0	1.97	1	0.88191	.	.	1
10398	0	1.97	1	0.88191	.	.	1
10399	0	1.97	1	0.88191	.	.	1
10400	0	1.97	1	0.88191	.	.	1
10401	0	1.97	1	0.88191	.	.	1
10402	0	1.97	1	0.88191	.	.	1
10403	0	1.97	1	0.88191	.	.	1
10404	0	1.97	1	0.88191	.	.	1
10405	0	1.97	1	0.88191	.	.	1
10406	0	1.97	1	0.88191	.	.	1
10407	0	1.97	1	0.88191	.	.	1
10408	0	1.97	1	0.88191	.	.	1
10409	0	1.97	1	0.88191	.	.	1
10410	0	1.97	1	0.88191	.	.	1
10411	0	1.97	1	0.88191	.	.	1
10412	0	1.97	1	0.88191	.	.	1
10413	0	1.98	0	0.88162	0.87852	0.88465	1
10414	0	1.98	1	0.88162	.	.	1
10415	0	1.98	1	0.88162	.	.	1
10416	0	1.98	1	0.88162	.	.	1
10417	0	1.98	1	0.88162	.	.	1
10418	0	1.98	1	0.88162	.	.	1
10419	0	1.98	1	0.88162	.	.	1
10420	0	1.98	1	0.88162	.	.	1
10421	0	1.98	1	0.88162	.	.	1
10422	0	1.98	1	0.88162	.	.	1
10423	0	1.98	1	0.88162	.	.	1
10424	0	1.98	1	0.88162	.	.	1
10425	0	1.98	1	0.88162	.	.	1
10426	0	1.98	1	0.88162	.	.	1
10427	0	1.98	1	0.88162	.	.	1
10428	0	1.98	1	0.88162	.	.	1
10429	0	1.98	1	0.88162	.	.	1
10430	0	1.98	1	0.88162	.	.	1

Obs	Sub_group	fuptimey	_CENSOR_	SURVIVAL	SDF_LCL	SDF_UCL	STRATUM
10431	0	1.98	1	0.88162	.	.	1
10432	0	1.98	1	0.88162	.	.	1
10433	0	1.98	1	0.88162	.	.	1
10434	0	1.98	1	0.88162	.	.	1
10435	0	1.98	1	0.88162	.	.	1
10436	0	1.98	1	0.88162	.	.	1
10437	0	1.98	1	0.88162	.	.	1
10438	0	1.98	1	0.88162	.	.	1
10439	0	1.98	1	0.88162	.	.	1
10440	0	1.98	1	0.88162	.	.	1
10441	0	1.98	1	0.88162	.	.	1
10442	0	1.98	1	0.88162	.	.	1
10443	0	1.98	1	0.88162	.	.	1
10444	0	1.98	1	0.88162	.	.	1
10445	0	1.98	1	0.88162	.	.	1
10446	0	1.98	1	0.88162	.	.	1
10447	0	1.98	1	0.88162	.	.	1
10448	0	1.98	1	0.88162	.	.	1
10449	0	1.98	1	0.88162	.	.	1
10450	0	1.98	1	0.88162	.	.	1
10451	0	1.98	1	0.88162	.	.	1
10452	0	1.98	1	0.88162	.	.	1
10453	0	1.98	1	0.88162	.	.	1
10454	0	1.98	1	0.88162	.	.	1
10455	0	1.98	1	0.88162	.	.	1
10456	0	1.98	1	0.88162	.	.	1
10457	0	1.98	1	0.88162	.	.	1
10458	0	1.98	1	0.88162	.	.	1
10459	0	1.98	1	0.88162	.	.	1
10460	0	1.98	1	0.88162	.	.	1
10461	0	1.98	1	0.88162	.	.	1
10462	0	1.98	1	0.88162	.	.	1
10463	0	1.98	1	0.88162	.	.	1
10464	0	1.98	1	0.88162	.	.	1
10465	0	1.99	0	0.88134	0.87824	0.88437	1

Obs	Sub_group	fuptimey	_CENSOR_	SURVIVAL	SDF_LCL	SDF_UCL	STRATUM
10466	0	1.99	1	0.88134	.	.	1
10467	0	1.99	1	0.88134	.	.	1
10468	0	1.99	1	0.88134	.	.	1
10469	0	1.99	1	0.88134	.	.	1
10470	0	1.99	1	0.88134	.	.	1
10471	0	1.99	1	0.88134	.	.	1
10472	0	1.99	1	0.88134	.	.	1
10473	0	1.99	1	0.88134	.	.	1
10474	0	1.99	1	0.88134	.	.	1
10475	0	1.99	1	0.88134	.	.	1
10476	0	1.99	1	0.88134	.	.	1
10477	0	1.99	1	0.88134	.	.	1
10478	0	1.99	1	0.88134	.	.	1
10479	0	1.99	1	0.88134	.	.	1
10480	0	1.99	1	0.88134	.	.	1
10481	0	1.99	1	0.88134	.	.	1
10482	0	1.99	1	0.88134	.	.	1
10483	0	1.99	1	0.88134	.	.	1
10484	0	1.99	1	0.88134	.	.	1
10485	0	1.99	1	0.88134	.	.	1
10486	0	1.99	1	0.88134	.	.	1
10487	0	1.99	1	0.88134	.	.	1
10488	0	1.99	1	0.88134	.	.	1
10489	0	1.99	1	0.88134	.	.	1
10490	0	1.99	1	0.88134	.	.	1
10491	0	1.99	1	0.88134	.	.	1
10492	0	1.99	1	0.88134	.	.	1
10493	0	1.99	1	0.88134	.	.	1
10494	0	1.99	1	0.88134	.	.	1
10495	0	1.99	1	0.88134	.	.	1
10496	0	1.99	1	0.88134	.	.	1
10497	0	1.99	1	0.88134	.	.	1
10498	0	1.99	1	0.88134	.	.	1
10499	0	1.99	1	0.88134	.	.	1
10500	0	1.99	1	0.88134	.	.	1

Obs	Sub_group	fuptimey	_CENSOR_	SURVIVAL	SDF_LCL	SDF_UCL	STRATUM
10501	0	1.99	1	0.88134	.	.	1
10502	0	1.99	1	0.88134	.	.	1
10503	0	1.99	1	0.88134	.	.	1
10504	0	1.99	1	0.88134	.	.	1
10505	0	1.99	1	0.88134	.	.	1
10506	0	1.99	1	0.88134	.	.	1
10507	0	1.99	1	0.88134	.	.	1
10508	0	1.99	1	0.88134	.	.	1
10509	0	1.99	1	0.88134	.	.	1
10510	0	1.99	1	0.88134	.	.	1
10511	0	1.99	1	0.88134	.	.	1
10512	0	1.99	1	0.88134	.	.	1
10513	0	1.99	1	0.88134	.	.	1
10514	0	2.00	0	0.88126	0.87815	0.88429	1
10515	0	2.00	1	0.88126	.	.	1
10516	0	2.00	1	0.88126	.	.	1
10517	0	2.00	1	0.88126	.	.	1
10518	0	2.00	1	0.88126	.	.	1
10519	0	2.00	1	0.88126	.	.	1
10520	0	2.00	1	0.88126	.	.	1
10521	0	2.00	1	0.88126	.	.	1
10522	0	2.00	1	0.88126	.	.	1
10523	0	2.00	1	0.88126	.	.	1
10524	0	2.00	1	0.88126	.	.	1
10525	0	2.00	1	0.88126	.	.	1
10526	0	2.00	1	0.88126	.	.	1
10527	0	2.00	1	0.88126	.	.	1
10528	0	2.00	1	0.88126	.	.	1
10529	0	2.00	1	0.88126	.	.	1
10530	0	2.00	1	0.88126	.	.	1
10531	0	2.00	1	0.88126	.	.	1
10532	0	2.00	1	0.88126	.	.	1
10533	0	2.00	1	0.88126	.	.	1
10534	0	2.00	1	0.88126	.	.	1
10535	0	2.00	1	0.88126	.	.	1

Obs	Sub_group	fuptimey	_CENSOR_	SURVIVAL	SDF_LCL	SDF_UCL	STRATUM
**10536**	0	2.00	1	0.88126	.	.	1
**10537**	0	2.00	1	0.88126	.	.	1
**10538**	0	2.00	1	0.88126	.	.	1
**10539**	0	2.00	1	0.88126	.	.	1
**10540**	0	2.00	1	0.88126	.	.	1
**10541**	0	2.00	1	0.88126	.	.	1
**10542**	0	2.00	1	0.88126	.	.	1
**10543**	0	2.00	1	0.88126	.	.	1
**10544**	0	2.00	1	0.88126	.	.	1
**10545**	0	2.00	1	0.88126	.	.	1
**10546**	0	2.00	1	0.88126	.	.	1
**10547**	0	2.00	1	0.88126	.	.	1
**10548**	0	2.00	1	0.88126	.	.	1
**10549**	0	2.00	1	0.88126	.	.	1
**10550**	0	2.00	1	0.88126	.	.	1
**10551**	0	2.00	1	0.88126	.	.	1
**10552**	0	2.00	1	0.88126	.	.	1
**10553**	0	2.00	1	0.88126	.	.	1
**10554**	0	2.00	1	0.88126	.	.	1
**10555**	0	2.00	1	0.88126	.	.	1
**10556**	0	2.00	1	0.88126	.	.	1
**10557**	0	2.00	1	0.88126	.	.	1
**10558**	0	2.00	1	0.88126	.	.	1
**10559**	0	2.00	1	0.88126	.	.	1
**10560**	0	2.00	1	0.88126	.	.	1
**10561**	0	2.00	1	0.88126	.	.	1
**10562**	0	2.00	1	0.88126	.	.	1
**10563**	0	2.00	1	0.88126	.	.	1
**10564**	0	2.01	0	0.88098	0.87787	0.88401	1
**10565**	0	2.01	1	0.88098	.	.	1
**10566**	0	2.01	1	0.88098	.	.	1
**10567**	0	2.01	1	0.88098	.	.	1
**10568**	0	2.01	1	0.88098	.	.	1
**10569**	0	2.01	1	0.88098	.	.	1
**10570**	0	2.01	1	0.88098	.	.	1

Obs	Sub_group	fuptimey	_CENSOR_	SURVIVAL	SDF_LCL	SDF_UCL	STRATUM
**10571**	0	2.01	1	0.88098	.	.	1
**10572**	0	2.01	1	0.88098	.	.	1
**10573**	0	2.01	1	0.88098	.	.	1
**10574**	0	2.01	1	0.88098	.	.	1
**10575**	0	2.01	1	0.88098	.	.	1
**10576**	0	2.01	1	0.88098	.	.	1
**10577**	0	2.01	1	0.88098	.	.	1
**10578**	0	2.01	1	0.88098	.	.	1
**10579**	0	2.01	1	0.88098	.	.	1
**10580**	0	2.01	1	0.88098	.	.	1
**10581**	0	2.01	1	0.88098	.	.	1
**10582**	0	2.01	1	0.88098	.	.	1
**10583**	0	2.01	1	0.88098	.	.	1
**10584**	0	2.01	1	0.88098	.	.	1
**10585**	0	2.01	1	0.88098	.	.	1
**10586**	0	2.01	1	0.88098	.	.	1
**10587**	0	2.01	1	0.88098	.	.	1
**10588**	0	2.01	1	0.88098	.	.	1
**10589**	0	2.01	1	0.88098	.	.	1
**10590**	0	2.01	1	0.88098	.	.	1
**10591**	0	2.01	1	0.88098	.	.	1
**10592**	0	2.01	1	0.88098	.	.	1
**10593**	0	2.01	1	0.88098	.	.	1
**10594**	0	2.01	1	0.88098	.	.	1
**10595**	0	2.01	1	0.88098	.	.	1
**10596**	0	2.01	1	0.88098	.	.	1
**10597**	0	2.01	1	0.88098	.	.	1
**10598**	0	2.01	1	0.88098	.	.	1
**10599**	0	2.01	1	0.88098	.	.	1
**10600**	0	2.01	1	0.88098	.	.	1
**10601**	0	2.01	1	0.88098	.	.	1
**10602**	0	2.01	1	0.88098	.	.	1
**10603**	0	2.02	0	0.88083	0.87772	0.88387	1
**10604**	0	2.02	1	0.88083	.	.	1
**10605**	0	2.02	1	0.88083	.	.	1

Obs	Sub_group	fuptimey	_CENSOR_	SURVIVAL	SDF_LCL	SDF_UCL	STRATUM
10606	0	2.02	1	0.88083	.	.	1
10607	0	2.02	1	0.88083	.	.	1
10608	0	2.02	1	0.88083	.	.	1
10609	0	2.02	1	0.88083	.	.	1
10610	0	2.02	1	0.88083	.	.	1
10611	0	2.02	1	0.88083	.	.	1
10612	0	2.02	1	0.88083	.	.	1
10613	0	2.02	1	0.88083	.	.	1
10614	0	2.02	1	0.88083	.	.	1
10615	0	2.02	1	0.88083	.	.	1
10616	0	2.02	1	0.88083	.	.	1
10617	0	2.02	1	0.88083	.	.	1
10618	0	2.02	1	0.88083	.	.	1
10619	0	2.02	1	0.88083	.	.	1
10620	0	2.02	1	0.88083	.	.	1
10621	0	2.02	1	0.88083	.	.	1
10622	0	2.02	1	0.88083	.	.	1
10623	0	2.02	1	0.88083	.	.	1
10624	0	2.02	1	0.88083	.	.	1
10625	0	2.02	1	0.88083	.	.	1
10626	0	2.02	1	0.88083	.	.	1
10627	0	2.02	1	0.88083	.	.	1
10628	0	2.02	1	0.88083	.	.	1
10629	0	2.02	1	0.88083	.	.	1
10630	0	2.02	1	0.88083	.	.	1
10631	0	2.02	1	0.88083	.	.	1
10632	0	2.02	1	0.88083	.	.	1
10633	0	2.02	1	0.88083	.	.	1
10634	0	2.02	1	0.88083	.	.	1
10635	0	2.02	1	0.88083	.	.	1
10636	0	2.02	1	0.88083	.	.	1
10637	0	2.02	1	0.88083	.	.	1
10638	0	2.02	1	0.88083	.	.	1
10639	0	2.02	1	0.88083	.	.	1
10640	0	2.02	1	0.88083	.	.	1

Obs	Sub_group	fuptimey	_CENSOR_	SURVIVAL	SDF_LCL	SDF_UCL	STRATUM
10641	0	2.02	1	0.88083	.	.	1
10642	0	2.02	1	0.88083	.	.	1
10643	0	2.02	1	0.88083	.	.	1
10644	0	2.02	1	0.88083	.	.	1
10645	0	2.02	1	0.88083	.	.	1
10646	0	2.02	1	0.88083	.	.	1
10647	0	2.02	1	0.88083	.	.	1
10648	0	2.02	1	0.88083	.	.	1
10649	0	2.02	1	0.88083	.	.	1
10650	0	2.02	1	0.88083	.	.	1
10651	0	2.02	1	0.88083	.	.	1
10652	0	2.02	1	0.88083	.	.	1
10653	0	2.02	1	0.88083	.	.	1
10654	0	2.02	1	0.88083	.	.	1
10655	0	2.02	1	0.88083	.	.	1
10656	0	2.02	1	0.88083	.	.	1
10657	0	2.02	1	0.88083	.	.	1
10658	0	2.02	1	0.88083	.	.	1
10659	0	2.02	1	0.88083	.	.	1
10660	0	2.02	1	0.88083	.	.	1
10661	0	2.02	1	0.88083	.	.	1
10662	0	2.02	1	0.88083	.	.	1
10663	0	2.02	1	0.88083	.	.	1
10664	0	2.02	1	0.88083	.	.	1
10665	0	2.02	1	0.88083	.	.	1
10666	0	2.02	1	0.88083	.	.	1
10667	0	2.02	1	0.88083	.	.	1
10668	0	2.03	0	0.88064	0.87752	0.88368	1
10669	0	2.03	1	0.88064	.	.	1
10670	0	2.03	1	0.88064	.	.	1
10671	0	2.03	1	0.88064	.	.	1
10672	0	2.03	1	0.88064	.	.	1
10673	0	2.03	1	0.88064	.	.	1
10674	0	2.03	1	0.88064	.	.	1
10675	0	2.03	1	0.88064	.	.	1

Obs	Sub_group	fuptimey	_CENSOR_	SURVIVAL	SDF_LCL	SDF_UCL	STRATUM
10676	0	2.03	1	0.88064	.	.	1
10677	0	2.03	1	0.88064	.	.	1
10678	0	2.03	1	0.88064	.	.	1
10679	0	2.03	1	0.88064	.	.	1
10680	0	2.03	1	0.88064	.	.	1
10681	0	2.03	1	0.88064	.	.	1
10682	0	2.03	1	0.88064	.	.	1
10683	0	2.03	1	0.88064	.	.	1
10684	0	2.03	1	0.88064	.	.	1
10685	0	2.03	1	0.88064	.	.	1
10686	0	2.03	1	0.88064	.	.	1
10687	0	2.03	1	0.88064	.	.	1
10688	0	2.03	1	0.88064	.	.	1
10689	0	2.03	1	0.88064	.	.	1
10690	0	2.03	1	0.88064	.	.	1
10691	0	2.04	0	0.88049	0.87738	0.88354	1
10692	0	2.04	1	0.88049	.	.	1
10693	0	2.04	1	0.88049	.	.	1
10694	0	2.04	1	0.88049	.	.	1
10695	0	2.04	1	0.88049	.	.	1
10696	0	2.04	1	0.88049	.	.	1
10697	0	2.04	1	0.88049	.	.	1
10698	0	2.04	1	0.88049	.	.	1
10699	0	2.04	1	0.88049	.	.	1
10700	0	2.04	1	0.88049	.	.	1
10701	0	2.04	1	0.88049	.	.	1
10702	0	2.04	1	0.88049	.	.	1
10703	0	2.04	1	0.88049	.	.	1
10704	0	2.04	1	0.88049	.	.	1
10705	0	2.04	1	0.88049	.	.	1
10706	0	2.04	1	0.88049	.	.	1
10707	0	2.04	1	0.88049	.	.	1
10708	0	2.04	1	0.88049	.	.	1
10709	0	2.04	1	0.88049	.	.	1
10710	0	2.04	1	0.88049	.	.	1

Obs	Sub_group	fuptimey	_CENSOR_	SURVIVAL	SDF_LCL	SDF_UCL	STRATUM
10711	0	2.04	1	0.88049	.	.	1
10712	0	2.04	1	0.88049	.	.	1
10713	0	2.04	1	0.88049	.	.	1
10714	0	2.04	1	0.88049	.	.	1
10715	0	2.04	1	0.88049	.	.	1
10716	0	2.04	1	0.88049	.	.	1
10717	0	2.04	1	0.88049	.	.	1
10718	0	2.04	1	0.88049	.	.	1
10719	0	2.04	1	0.88049	.	.	1
10720	0	2.04	1	0.88049	.	.	1
10721	0	2.04	1	0.88049	.	.	1
10722	0	2.04	1	0.88049	.	.	1
10723	0	2.04	1	0.88049	.	.	1
10724	0	2.04	1	0.88049	.	.	1
10725	0	2.04	1	0.88049	.	.	1
10726	0	2.04	1	0.88049	.	.	1
10727	0	2.04	1	0.88049	.	.	1
10728	0	2.04	1	0.88049	.	.	1
10729	0	2.04	1	0.88049	.	.	1
10730	0	2.04	1	0.88049	.	.	1
10731	0	2.04	1	0.88049	.	.	1
10732	0	2.04	1	0.88049	.	.	1
10733	0	2.04	1	0.88049	.	.	1
10734	0	2.04	1	0.88049	.	.	1
10735	0	2.04	1	0.88049	.	.	1
10736	0	2.04	1	0.88049	.	.	1
10737	0	2.04	1	0.88049	.	.	1
10738	0	2.04	1	0.88049	.	.	1
10739	0	2.04	1	0.88049	.	.	1
10740	0	2.04	1	0.88049	.	.	1
10741	0	2.04	1	0.88049	.	.	1
10742	0	2.04	1	0.88049	.	.	1
10743	0	2.04	1	0.88049	.	.	1
10744	0	2.04	1	0.88049	.	.	1
10745	0	2.04	1	0.88049	.	.	1

Obs	Sub_group	fuptimey	_CENSOR_	SURVIVAL	SDF_LCL	SDF_UCL	STRATUM
10746	0	2.04	1	0.88049	.	.	1
10747	0	2.04	1	0.88049	.	.	1
10748	0	2.04	1	0.88049	.	.	1
10749	0	2.04	1	0.88049	.	.	1
10750	0	2.04	1	0.88049	.	.	1
10751	0	2.04	1	0.88049	.	.	1
10752	0	2.05	0	0.88007	0.87694	0.88312	1
10753	0	2.05	1	0.88007	.	.	1
10754	0	2.05	1	0.88007	.	.	1
10755	0	2.05	1	0.88007	.	.	1
10756	0	2.05	1	0.88007	.	.	1
10757	0	2.05	1	0.88007	.	.	1
10758	0	2.05	1	0.88007	.	.	1
10759	0	2.05	1	0.88007	.	.	1
10760	0	2.05	1	0.88007	.	.	1
10761	0	2.05	1	0.88007	.	.	1
10762	0	2.05	1	0.88007	.	.	1
10763	0	2.05	1	0.88007	.	.	1
10764	0	2.05	1	0.88007	.	.	1
10765	0	2.05	1	0.88007	.	.	1
10766	0	2.05	1	0.88007	.	.	1
10767	0	2.05	1	0.88007	.	.	1
10768	0	2.05	1	0.88007	.	.	1
10769	0	2.05	1	0.88007	.	.	1
10770	0	2.05	1	0.88007	.	.	1
10771	0	2.05	1	0.88007	.	.	1
10772	0	2.05	1	0.88007	.	.	1
10773	0	2.05	1	0.88007	.	.	1
10774	0	2.05	1	0.88007	.	.	1
10775	0	2.05	1	0.88007	.	.	1
10776	0	2.05	1	0.88007	.	.	1
10777	0	2.05	1	0.88007	.	.	1
10778	0	2.05	1	0.88007	.	.	1
10779	0	2.05	1	0.88007	.	.	1
10780	0	2.05	1	0.88007	.	.	1

Obs	Sub_group	fuptimey	_CENSOR_	SURVIVAL	SDF_LCL	SDF_UCL	STRATUM
10781	0	2.05	1	0.88007	.	.	1
10782	0	2.05	1	0.88007	.	.	1
10783	0	2.05	1	0.88007	.	.	1
10784	0	2.05	1	0.88007	.	.	1
10785	0	2.05	1	0.88007	.	.	1
10786	0	2.05	1	0.88007	.	.	1
10787	0	2.05	1	0.88007	.	.	1
10788	0	2.05	1	0.88007	.	.	1
10789	0	2.05	1	0.88007	.	.	1
10790	0	2.05	1	0.88007	.	.	1
10791	0	2.06	0	0.87990	0.87677	0.88295	1
10792	0	2.06	1	0.87990	.	.	1
10793	0	2.06	1	0.87990	.	.	1
10794	0	2.06	1	0.87990	.	.	1
10795	0	2.06	1	0.87990	.	.	1
10796	0	2.06	1	0.87990	.	.	1
10797	0	2.06	1	0.87990	.	.	1
10798	0	2.06	1	0.87990	.	.	1
10799	0	2.06	1	0.87990	.	.	1
10800	0	2.06	1	0.87990	.	.	1
10801	0	2.06	1	0.87990	.	.	1
10802	0	2.06	1	0.87990	.	.	1
10803	0	2.06	1	0.87990	.	.	1
10804	0	2.06	1	0.87990	.	.	1
10805	0	2.06	1	0.87990	.	.	1
10806	0	2.06	1	0.87990	.	.	1
10807	0	2.06	1	0.87990	.	.	1
10808	0	2.06	1	0.87990	.	.	1
10809	0	2.06	1	0.87990	.	.	1
10810	0	2.06	1	0.87990	.	.	1
10811	0	2.06	1	0.87990	.	.	1
10812	0	2.06	1	0.87990	.	.	1
10813	0	2.06	1	0.87990	.	.	1
10814	0	2.06	1	0.87990	.	.	1
10815	0	2.06	1	0.87990	.	.	1

Obs	Sub_group	fuptimey	_CENSOR_	SURVIVAL	SDF_LCL	SDF_UCL	STRATUM
10816	0	2.06	1	0.87990	.	.	1
10817	0	2.06	1	0.87990	.	.	1
10818	0	2.06	1	0.87990	.	.	1
10819	0	2.06	1	0.87990	.	.	1
10820	0	2.06	1	0.87990	.	.	1
10821	0	2.06	1	0.87990	.	.	1
10822	0	2.06	1	0.87990	.	.	1
10823	0	2.06	1	0.87990	.	.	1
10824	0	2.06	1	0.87990	.	.	1
10825	0	2.06	1	0.87990	.	.	1
10826	0	2.06	1	0.87990	.	.	1
10827	0	2.06	1	0.87990	.	.	1
10828	0	2.06	1	0.87990	.	.	1
10829	0	2.06	1	0.87990	.	.	1
10830	0	2.06	1	0.87990	.	.	1
10831	0	2.06	1	0.87990	.	.	1
10832	0	2.06	1	0.87990	.	.	1
10833	0	2.06	1	0.87990	.	.	1
10834	0	2.06	1	0.87990	.	.	1
10835	0	2.06	1	0.87990	.	.	1
10836	0	2.06	1	0.87990	.	.	1
10837	0	2.06	1	0.87990	.	.	1
10838	0	2.06	1	0.87990	.	.	1
10839	0	2.06	1	0.87990	.	.	1
10840	0	2.06	1	0.87990	.	.	1
10841	0	2.06	1	0.87990	.	.	1
10842	0	2.06	1	0.87990	.	.	1
10843	0	2.06	1	0.87990	.	.	1
10844	0	2.06	1	0.87990	.	.	1
10845	0	2.06	1	0.87990	.	.	1
10846	0	2.06	1	0.87990	.	.	1
10847	0	2.06	1	0.87990	.	.	1
10848	0	2.06	1	0.87990	.	.	1
10849	0	2.06	1	0.87990	.	.	1
10850	0	2.06	1	0.87990	.	.	1

Obs	Sub_group	fuptimey	_CENSOR_	SURVIVAL	SDF_LCL	SDF_UCL	STRATUM
**10851**	0	2.07	0	0.87952	0.87639	0.88258	1
**10852**	0	2.07	1	0.87952	.	.	1
**10853**	0	2.07	1	0.87952	.	.	1
**10854**	0	2.07	1	0.87952	.	.	1
**10855**	0	2.07	1	0.87952	.	.	1
**10856**	0	2.07	1	0.87952	.	.	1
**10857**	0	2.07	1	0.87952	.	.	1
**10858**	0	2.07	1	0.87952	.	.	1
**10859**	0	2.07	1	0.87952	.	.	1
**10860**	0	2.07	1	0.87952	.	.	1
**10861**	0	2.07	1	0.87952	.	.	1
**10862**	0	2.07	1	0.87952	.	.	1
**10863**	0	2.07	1	0.87952	.	.	1
**10864**	0	2.07	1	0.87952	.	.	1
**10865**	0	2.07	1	0.87952	.	.	1
**10866**	0	2.07	1	0.87952	.	.	1
**10867**	0	2.07	1	0.87952	.	.	1
**10868**	0	2.07	1	0.87952	.	.	1
**10869**	0	2.07	1	0.87952	.	.	1
**10870**	0	2.07	1	0.87952	.	.	1
**10871**	0	2.07	1	0.87952	.	.	1
**10872**	0	2.07	1	0.87952	.	.	1
**10873**	0	2.07	1	0.87952	.	.	1
**10874**	0	2.07	1	0.87952	.	.	1
**10875**	0	2.07	1	0.87952	.	.	1
**10876**	0	2.07	1	0.87952	.	.	1
**10877**	0	2.07	1	0.87952	.	.	1
**10878**	0	2.07	1	0.87952	.	.	1
**10879**	0	2.07	1	0.87952	.	.	1
**10880**	0	2.07	1	0.87952	.	.	1
**10881**	0	2.07	1	0.87952	.	.	1
**10882**	0	2.07	1	0.87952	.	.	1
**10883**	0	2.07	1	0.87952	.	.	1
**10884**	0	2.07	1	0.87952	.	.	1
**10885**	0	2.07	1	0.87952	.	.	1

Obs	Sub_group	fuptimey	_CENSOR_	SURVIVAL	SDF_LCL	SDF_UCL	STRATUM
10886	0	2.07	1	0.87952	.	.	1
10887	0	2.07	1	0.87952	.	.	1
10888	0	2.07	1	0.87952	.	.	1
10889	0	2.07	1	0.87952	.	.	1
10890	0	2.07	1	0.87952	.	.	1
10891	0	2.07	1	0.87952	.	.	1
10892	0	2.07	1	0.87952	.	.	1
10893	0	2.07	1	0.87952	.	.	1
10894	0	2.08	0	0.87941	0.87628	0.88247	1
10895	0	2.08	1	0.87941	.	.	1
10896	0	2.08	1	0.87941	.	.	1
10897	0	2.08	1	0.87941	.	.	1
10898	0	2.08	1	0.87941	.	.	1
10899	0	2.08	1	0.87941	.	.	1
10900	0	2.08	1	0.87941	.	.	1
10901	0	2.08	1	0.87941	.	.	1
10902	0	2.08	1	0.87941	.	.	1
10903	0	2.08	1	0.87941	.	.	1
10904	0	2.08	1	0.87941	.	.	1
10905	0	2.08	1	0.87941	.	.	1
10906	0	2.08	1	0.87941	.	.	1
10907	0	2.08	1	0.87941	.	.	1
10908	0	2.08	1	0.87941	.	.	1
10909	0	2.08	1	0.87941	.	.	1
10910	0	2.08	1	0.87941	.	.	1
10911	0	2.08	1	0.87941	.	.	1
10912	0	2.08	1	0.87941	.	.	1
10913	0	2.08	1	0.87941	.	.	1
10914	0	2.08	1	0.87941	.	.	1
10915	0	2.08	1	0.87941	.	.	1
10916	0	2.08	1	0.87941	.	.	1
10917	0	2.08	1	0.87941	.	.	1
10918	0	2.08	1	0.87941	.	.	1
10919	0	2.08	1	0.87941	.	.	1
10920	0	2.08	1	0.87941	.	.	1

Obs	Sub_group	fuptimey	_CENSOR_	SURVIVAL	SDF_LCL	SDF_UCL	STRATUM
10921	0	2.08	1	0.87941	.	.	1
10922	0	2.08	1	0.87941	.	.	1
10923	0	2.08	1	0.87941	.	.	1
10924	0	2.08	1	0.87941	.	.	1
10925	0	2.08	1	0.87941	.	.	1
10926	0	2.08	1	0.87941	.	.	1
10927	0	2.08	1	0.87941	.	.	1
10928	0	2.08	1	0.87941	.	.	1
10929	0	2.08	1	0.87941	.	.	1
10930	0	2.08	1	0.87941	.	.	1
10931	0	2.08	1	0.87941	.	.	1
10932	0	2.08	1	0.87941	.	.	1
10933	0	2.08	1	0.87941	.	.	1
10934	0	2.08	1	0.87941	.	.	1
10935	0	2.08	1	0.87941	.	.	1
10936	0	2.08	1	0.87941	.	.	1
10937	0	2.08	1	0.87941	.	.	1
10938	0	2.08	1	0.87941	.	.	1
10939	0	2.08	1	0.87941	.	.	1
10940	0	2.08	1	0.87941	.	.	1
10941	0	2.09	0	0.87921	0.87607	0.88227	1
10942	0	2.09	1	0.87921	.	.	1
10943	0	2.09	1	0.87921	.	.	1
10944	0	2.09	1	0.87921	.	.	1
10945	0	2.09	1	0.87921	.	.	1
10946	0	2.09	1	0.87921	.	.	1
10947	0	2.09	1	0.87921	.	.	1
10948	0	2.09	1	0.87921	.	.	1
10949	0	2.09	1	0.87921	.	.	1
10950	0	2.09	1	0.87921	.	.	1
10951	0	2.09	1	0.87921	.	.	1
10952	0	2.09	1	0.87921	.	.	1
10953	0	2.09	1	0.87921	.	.	1
10954	0	2.09	1	0.87921	.	.	1
10955	0	2.09	1	0.87921	.	.	1

Obs	Sub_group	fuptimey	_CENSOR_	SURVIVAL	SDF_LCL	SDF_UCL	STRATUM
**10956**	0	2.09	1	0.87921	.	.	1
**10957**	0	2.09	1	0.87921	.	.	1
**10958**	0	2.09	1	0.87921	.	.	1
**10959**	0	2.09	1	0.87921	.	.	1
**10960**	0	2.09	1	0.87921	.	.	1
**10961**	0	2.09	1	0.87921	.	.	1
**10962**	0	2.09	1	0.87921	.	.	1
**10963**	0	2.09	1	0.87921	.	.	1
**10964**	0	2.09	1	0.87921	.	.	1
**10965**	0	2.09	1	0.87921	.	.	1
**10966**	0	2.09	1	0.87921	.	.	1
**10967**	0	2.09	1	0.87921	.	.	1
**10968**	0	2.09	1	0.87921	.	.	1
**10969**	0	2.09	1	0.87921	.	.	1
**10970**	0	2.09	1	0.87921	.	.	1
**10971**	0	2.09	1	0.87921	.	.	1
**10972**	0	2.09	1	0.87921	.	.	1
**10973**	0	2.09	1	0.87921	.	.	1
**10974**	0	2.09	1	0.87921	.	.	1
**10975**	0	2.09	1	0.87921	.	.	1
**10976**	0	2.09	1	0.87921	.	.	1
**10977**	0	2.09	1	0.87921	.	.	1
**10978**	0	2.09	1	0.87921	.	.	1
**10979**	0	2.09	1	0.87921	.	.	1
**10980**	0	2.09	1	0.87921	.	.	1
**10981**	0	2.09	1	0.87921	.	.	1
**10982**	0	2.09	1	0.87921	.	.	1
**10983**	0	2.09	1	0.87921	.	.	1
**10984**	0	2.09	1	0.87921	.	.	1
**10985**	0	2.10	0	0.87898	0.87584	0.88205	1
**10986**	0	2.10	1	0.87898	.	.	1
**10987**	0	2.10	1	0.87898	.	.	1
**10988**	0	2.10	1	0.87898	.	.	1
**10989**	0	2.10	1	0.87898	.	.	1
**10990**	0	2.10	1	0.87898	.	.	1

Obs	Sub_group	fuptimey	_CENSOR_	SURVIVAL	SDF_LCL	SDF_UCL	STRATUM
10991	0	2.10	1	0.87898	.	.	1
10992	0	2.10	1	0.87898	.	.	1
10993	0	2.10	1	0.87898	.	.	1
10994	0	2.10	1	0.87898	.	.	1
10995	0	2.10	1	0.87898	.	.	1
10996	0	2.10	1	0.87898	.	.	1
10997	0	2.10	1	0.87898	.	.	1
10998	0	2.10	1	0.87898	.	.	1
10999	0	2.10	1	0.87898	.	.	1
11000	0	2.10	1	0.87898	.	.	1
11001	0	2.10	1	0.87898	.	.	1
11002	0	2.10	1	0.87898	.	.	1
11003	0	2.10	1	0.87898	.	.	1
11004	0	2.10	1	0.87898	.	.	1
11005	0	2.10	1	0.87898	.	.	1
11006	0	2.10	1	0.87898	.	.	1
11007	0	2.10	1	0.87898	.	.	1
11008	0	2.10	1	0.87898	.	.	1
11009	0	2.10	1	0.87898	.	.	1
11010	0	2.10	1	0.87898	.	.	1
11011	0	2.10	1	0.87898	.	.	1
11012	0	2.10	1	0.87898	.	.	1
11013	0	2.10	1	0.87898	.	.	1
11014	0	2.10	1	0.87898	.	.	1
11015	0	2.10	1	0.87898	.	.	1
11016	0	2.10	1	0.87898	.	.	1
11017	0	2.10	1	0.87898	.	.	1
11018	0	2.10	1	0.87898	.	.	1
11019	0	2.10	1	0.87898	.	.	1
11020	0	2.10	1	0.87898	.	.	1
11021	0	2.10	1	0.87898	.	.	1
11022	0	2.10	1	0.87898	.	.	1
11023	0	2.10	1	0.87898	.	.	1
11024	0	2.10	1	0.87898	.	.	1
11025	0	2.10	1	0.87898	.	.	1

Obs	Sub_group	fuptimey	_CENSOR_	SURVIVAL	SDF_LCL	SDF_UCL	STRATUM
11026	0	2.10	1	0.87898	.	.	1
11027	0	2.10	1	0.87898	.	.	1
11028	0	2.10	1	0.87898	.	.	1
11029	0	2.10	1	0.87898	.	.	1
11030	0	2.10	1	0.87898	.	.	1
11031	0	2.10	1	0.87898	.	.	1
11032	0	2.10	1	0.87898	.	.	1
11033	0	2.10	1	0.87898	.	.	1
11034	0	2.10	1	0.87898	.	.	1
11035	0	2.10	1	0.87898	.	.	1
11036	0	2.11	0	0.87869	0.87555	0.88176	1
11037	0	2.11	1	0.87869	.	.	1
11038	0	2.11	1	0.87869	.	.	1
11039	0	2.11	1	0.87869	.	.	1
11040	0	2.11	1	0.87869	.	.	1
11041	0	2.11	1	0.87869	.	.	1
11042	0	2.11	1	0.87869	.	.	1
11043	0	2.11	1	0.87869	.	.	1
11044	0	2.11	1	0.87869	.	.	1
11045	0	2.11	1	0.87869	.	.	1
11046	0	2.11	1	0.87869	.	.	1
11047	0	2.11	1	0.87869	.	.	1
11048	0	2.11	1	0.87869	.	.	1
11049	0	2.11	1	0.87869	.	.	1
11050	0	2.11	1	0.87869	.	.	1
11051	0	2.11	1	0.87869	.	.	1
11052	0	2.11	1	0.87869	.	.	1
11053	0	2.11	1	0.87869	.	.	1
11054	0	2.11	1	0.87869	.	.	1
11055	0	2.11	1	0.87869	.	.	1
11056	0	2.11	1	0.87869	.	.	1
11057	0	2.11	1	0.87869	.	.	1
11058	0	2.11	1	0.87869	.	.	1
11059	0	2.11	1	0.87869	.	.	1
11060	0	2.11	1	0.87869	.	.	1

Obs	Sub_group	fuptimey	_CENSOR_	SURVIVAL	SDF_LCL	SDF_UCL	STRATUM
**11061**	0	2.11	1	0.87869	.	.	1
**11062**	0	2.11	1	0.87869	.	.	1
**11063**	0	2.11	1	0.87869	.	.	1
**11064**	0	2.11	1	0.87869	.	.	1
**11065**	0	2.11	1	0.87869	.	.	1
**11066**	0	2.11	1	0.87869	.	.	1
**11067**	0	2.11	1	0.87869	.	.	1
**11068**	0	2.11	1	0.87869	.	.	1
**11069**	0	2.11	1	0.87869	.	.	1
**11070**	0	2.11	1	0.87869	.	.	1
**11071**	0	2.11	1	0.87869	.	.	1
**11072**	0	2.11	1	0.87869	.	.	1
**11073**	0	2.11	1	0.87869	.	.	1
**11074**	0	2.11	1	0.87869	.	.	1
**11075**	0	2.11	1	0.87869	.	.	1
**11076**	0	2.11	1	0.87869	.	.	1
**11077**	0	2.11	1	0.87869	.	.	1
**11078**	0	2.11	1	0.87869	.	.	1
**11079**	0	2.12	0	0.87838	0.87523	0.88145	1
**11080**	0	2.12	1	0.87838	.	.	1
**11081**	0	2.12	1	0.87838	.	.	1
**11082**	0	2.12	1	0.87838	.	.	1
**11083**	0	2.12	1	0.87838	.	.	1
**11084**	0	2.12	1	0.87838	.	.	1
**11085**	0	2.12	1	0.87838	.	.	1
**11086**	0	2.12	1	0.87838	.	.	1
**11087**	0	2.12	1	0.87838	.	.	1
**11088**	0	2.12	1	0.87838	.	.	1
**11089**	0	2.12	1	0.87838	.	.	1
**11090**	0	2.12	1	0.87838	.	.	1
**11091**	0	2.12	1	0.87838	.	.	1
**11092**	0	2.12	1	0.87838	.	.	1
**11093**	0	2.12	1	0.87838	.	.	1
**11094**	0	2.12	1	0.87838	.	.	1
**11095**	0	2.12	1	0.87838	.	.	1

Obs	Sub_group	fuptimey	_CENSOR_	SURVIVAL	SDF_LCL	SDF_UCL	STRATUM
**11096**	0	2.12	1	0.87838	.	.	1
**11097**	0	2.12	1	0.87838	.	.	1
**11098**	0	2.12	1	0.87838	.	.	1
**11099**	0	2.12	1	0.87838	.	.	1
**11100**	0	2.12	1	0.87838	.	.	1
**11101**	0	2.12	1	0.87838	.	.	1
**11102**	0	2.12	1	0.87838	.	.	1
**11103**	0	2.12	1	0.87838	.	.	1
**11104**	0	2.12	1	0.87838	.	.	1
**11105**	0	2.12	1	0.87838	.	.	1
**11106**	0	2.12	1	0.87838	.	.	1
**11107**	0	2.12	1	0.87838	.	.	1
**11108**	0	2.12	1	0.87838	.	.	1
**11109**	0	2.12	1	0.87838	.	.	1
**11110**	0	2.12	1	0.87838	.	.	1
**11111**	0	2.12	1	0.87838	.	.	1
**11112**	0	2.12	1	0.87838	.	.	1
**11113**	0	2.12	1	0.87838	.	.	1
**11114**	0	2.12	1	0.87838	.	.	1
**11115**	0	2.12	1	0.87838	.	.	1
**11116**	0	2.12	1	0.87838	.	.	1
**11117**	0	2.12	1	0.87838	.	.	1
**11118**	0	2.12	1	0.87838	.	.	1
**11119**	0	2.12	1	0.87838	.	.	1
**11120**	0	2.13	0	0.87820	0.87506	0.88128	1
**11121**	0	2.13	1	0.87820	.	.	1
**11122**	0	2.13	1	0.87820	.	.	1
**11123**	0	2.13	1	0.87820	.	.	1
**11124**	0	2.13	1	0.87820	.	.	1
**11125**	0	2.13	1	0.87820	.	.	1
**11126**	0	2.13	1	0.87820	.	.	1
**11127**	0	2.13	1	0.87820	.	.	1
**11128**	0	2.13	1	0.87820	.	.	1
**11129**	0	2.13	1	0.87820	.	.	1
**11130**	0	2.13	1	0.87820	.	.	1

Obs	Sub_group	fuptimey	_CENSOR_	SURVIVAL	SDF_LCL	SDF_UCL	STRATUM
11131	0	2.13	1	0.87820	.	.	1
11132	0	2.13	1	0.87820	.	.	1
11133	0	2.13	1	0.87820	.	.	1
11134	0	2.13	1	0.87820	.	.	1
11135	0	2.13	1	0.87820	.	.	1
11136	0	2.13	1	0.87820	.	.	1
11137	0	2.13	1	0.87820	.	.	1
11138	0	2.13	1	0.87820	.	.	1
11139	0	2.13	1	0.87820	.	.	1
11140	0	2.13	1	0.87820	.	.	1
11141	0	2.13	1	0.87820	.	.	1
11142	0	2.13	1	0.87820	.	.	1
11143	0	2.13	1	0.87820	.	.	1
11144	0	2.13	1	0.87820	.	.	1
11145	0	2.13	1	0.87820	.	.	1
11146	0	2.13	1	0.87820	.	.	1
11147	0	2.13	1	0.87820	.	.	1
11148	0	2.13	1	0.87820	.	.	1
11149	0	2.13	1	0.87820	.	.	1
11150	0	2.13	1	0.87820	.	.	1
11151	0	2.13	1	0.87820	.	.	1
11152	0	2.13	1	0.87820	.	.	1
11153	0	2.13	1	0.87820	.	.	1
11154	0	2.13	1	0.87820	.	.	1
11155	0	2.13	1	0.87820	.	.	1
11156	0	2.13	1	0.87820	.	.	1
11157	0	2.13	1	0.87820	.	.	1
11158	0	2.13	1	0.87820	.	.	1
11159	0	2.13	1	0.87820	.	.	1
11160	0	2.13	1	0.87820	.	.	1
11161	0	2.13	1	0.87820	.	.	1
11162	0	2.13	1	0.87820	.	.	1
11163	0	2.13	1	0.87820	.	.	1
11164	0	2.13	1	0.87820	.	.	1
11165	0	2.13	1	0.87820	.	.	1

Obs	Sub_group	fuptimey	_CENSOR_	SURVIVAL	SDF_LCL	SDF_UCL	STRATUM
**11166**	0	2.13	1	0.87820	.	.	1
**11167**	0	2.13	1	0.87820	.	.	1
**11168**	0	2.14	0	0.87800	0.87485	0.88108	1
**11169**	0	2.14	1	0.87800	.	.	1
**11170**	0	2.14	1	0.87800	.	.	1
**11171**	0	2.14	1	0.87800	.	.	1
**11172**	0	2.14	1	0.87800	.	.	1
**11173**	0	2.14	1	0.87800	.	.	1
**11174**	0	2.14	1	0.87800	.	.	1
**11175**	0	2.14	1	0.87800	.	.	1
**11176**	0	2.14	1	0.87800	.	.	1
**11177**	0	2.14	1	0.87800	.	.	1
**11178**	0	2.14	1	0.87800	.	.	1
**11179**	0	2.14	1	0.87800	.	.	1
**11180**	0	2.14	1	0.87800	.	.	1
**11181**	0	2.14	1	0.87800	.	.	1
**11182**	0	2.14	1	0.87800	.	.	1
**11183**	0	2.14	1	0.87800	.	.	1
**11184**	0	2.14	1	0.87800	.	.	1
**11185**	0	2.14	1	0.87800	.	.	1
**11186**	0	2.14	1	0.87800	.	.	1
**11187**	0	2.14	1	0.87800	.	.	1
**11188**	0	2.14	1	0.87800	.	.	1
**11189**	0	2.14	1	0.87800	.	.	1
**11190**	0	2.14	1	0.87800	.	.	1
**11191**	0	2.14	1	0.87800	.	.	1
**11192**	0	2.14	1	0.87800	.	.	1
**11193**	0	2.14	1	0.87800	.	.	1
**11194**	0	2.14	1	0.87800	.	.	1
**11195**	0	2.14	1	0.87800	.	.	1
**11196**	0	2.14	1	0.87800	.	.	1
**11197**	0	2.14	1	0.87800	.	.	1
**11198**	0	2.14	1	0.87800	.	.	1
**11199**	0	2.14	1	0.87800	.	.	1
**11200**	0	2.14	1	0.87800	.	.	1

Obs	Sub_group	fuptimey	_CENSOR_	SURVIVAL	SDF_LCL	SDF_UCL	STRATUM
**11201**	0	2.14	1	0.87800	.	.	1
**11202**	0	2.14	1	0.87800	.	.	1
**11203**	0	2.14	1	0.87800	.	.	1
**11204**	0	2.14	1	0.87800	.	.	1
**11205**	0	2.14	1	0.87800	.	.	1
**11206**	0	2.14	1	0.87800	.	.	1
**11207**	0	2.14	1	0.87800	.	.	1
**11208**	0	2.14	1	0.87800	.	.	1
**11209**	0	2.14	1	0.87800	.	.	1
**11210**	0	2.14	1	0.87800	.	.	1
**11211**	0	2.14	1	0.87800	.	.	1
**11212**	0	2.14	1	0.87800	.	.	1
**11213**	0	2.14	1	0.87800	.	.	1
**11214**	0	2.15	0	0.87760	0.87444	0.88068	1
**11215**	0	2.15	1	0.87760	.	.	1
**11216**	0	2.15	1	0.87760	.	.	1
**11217**	0	2.15	1	0.87760	.	.	1
**11218**	0	2.15	1	0.87760	.	.	1
**11219**	0	2.15	1	0.87760	.	.	1
**11220**	0	2.15	1	0.87760	.	.	1
**11221**	0	2.15	1	0.87760	.	.	1
**11222**	0	2.15	1	0.87760	.	.	1
**11223**	0	2.15	1	0.87760	.	.	1
**11224**	0	2.15	1	0.87760	.	.	1
**11225**	0	2.15	1	0.87760	.	.	1
**11226**	0	2.15	1	0.87760	.	.	1
**11227**	0	2.15	1	0.87760	.	.	1
**11228**	0	2.15	1	0.87760	.	.	1
**11229**	0	2.15	1	0.87760	.	.	1
**11230**	0	2.15	1	0.87760	.	.	1
**11231**	0	2.15	1	0.87760	.	.	1
**11232**	0	2.15	1	0.87760	.	.	1
**11233**	0	2.15	1	0.87760	.	.	1
**11234**	0	2.15	1	0.87760	.	.	1
**11235**	0	2.15	1	0.87760	.	.	1

Obs	Sub_group	fuptimey	_CENSOR_	SURVIVAL	SDF_LCL	SDF_UCL	STRATUM
11236	0	2.15	1	0.87760	.	.	1
11237	0	2.15	1	0.87760	.	.	1
11238	0	2.15	1	0.87760	.	.	1
11239	0	2.15	1	0.87760	.	.	1
11240	0	2.15	1	0.87760	.	.	1
11241	0	2.15	1	0.87760	.	.	1
11242	0	2.15	1	0.87760	.	.	1
11243	0	2.15	1	0.87760	.	.	1
11244	0	2.15	1	0.87760	.	.	1
11245	0	2.15	1	0.87760	.	.	1
11246	0	2.15	1	0.87760	.	.	1
11247	0	2.15	1	0.87760	.	.	1
11248	0	2.15	1	0.87760	.	.	1
11249	0	2.15	1	0.87760	.	.	1
11250	0	2.15	1	0.87760	.	.	1
11251	0	2.15	1	0.87760	.	.	1
11252	0	2.15	1	0.87760	.	.	1
11253	0	2.15	1	0.87760	.	.	1
11254	0	2.15	1	0.87760	.	.	1
11255	0	2.15	1	0.87760	.	.	1
11256	0	2.15	1	0.87760	.	.	1
11257	0	2.15	1	0.87760	.	.	1
11258	0	2.15	1	0.87760	.	.	1
11259	0	2.15	1	0.87760	.	.	1
11260	0	2.16	0	0.87737	0.87421	0.88046	1
11261	0	2.16	1	0.87737	.	.	1
11262	0	2.16	1	0.87737	.	.	1
11263	0	2.16	1	0.87737	.	.	1
11264	0	2.16	1	0.87737	.	.	1
11265	0	2.16	1	0.87737	.	.	1
11266	0	2.16	1	0.87737	.	.	1
11267	0	2.16	1	0.87737	.	.	1
11268	0	2.16	1	0.87737	.	.	1
11269	0	2.16	1	0.87737	.	.	1
11270	0	2.16	1	0.87737	.	.	1

Obs	Sub_group	fuptimey	_CENSOR_	SURVIVAL	SDF_LCL	SDF_UCL	STRATUM
11271	0	2.16	1	0.87737	.	.	1
11272	0	2.16	1	0.87737	.	.	1
11273	0	2.16	1	0.87737	.	.	1
11274	0	2.16	1	0.87737	.	.	1
11275	0	2.16	1	0.87737	.	.	1
11276	0	2.16	1	0.87737	.	.	1
11277	0	2.16	1	0.87737	.	.	1
11278	0	2.16	1	0.87737	.	.	1
11279	0	2.16	1	0.87737	.	.	1
11280	0	2.16	1	0.87737	.	.	1
11281	0	2.16	1	0.87737	.	.	1
11282	0	2.16	1	0.87737	.	.	1
11283	0	2.16	1	0.87737	.	.	1
11284	0	2.16	1	0.87737	.	.	1
11285	0	2.16	1	0.87737	.	.	1
11286	0	2.16	1	0.87737	.	.	1
11287	0	2.16	1	0.87737	.	.	1
11288	0	2.16	1	0.87737	.	.	1
11289	0	2.16	1	0.87737	.	.	1
11290	0	2.16	1	0.87737	.	.	1
11291	0	2.16	1	0.87737	.	.	1
11292	0	2.16	1	0.87737	.	.	1
11293	0	2.16	1	0.87737	.	.	1
11294	0	2.16	1	0.87737	.	.	1
11295	0	2.16	1	0.87737	.	.	1
11296	0	2.16	1	0.87737	.	.	1
11297	0	2.16	1	0.87737	.	.	1
11298	0	2.16	1	0.87737	.	.	1
11299	0	2.16	1	0.87737	.	.	1
11300	0	2.16	1	0.87737	.	.	1
11301	0	2.16	1	0.87737	.	.	1
11302	0	2.16	1	0.87737	.	.	1
11303	0	2.16	1	0.87737	.	.	1
11304	0	2.16	1	0.87737	.	.	1
11305	0	2.16	1	0.87737	.	.	1

Obs	Sub_group	fuptimey	_CENSOR_	SURVIVAL	SDF_LCL	SDF_UCL	STRATUM
11306	0	2.16	1	0.87737	.	.	1
11307	0	2.17	0	0.87711	0.87394	0.88020	1
11308	0	2.17	1	0.87711	.	.	1
11309	0	2.17	1	0.87711	.	.	1
11310	0	2.17	1	0.87711	.	.	1
11311	0	2.17	1	0.87711	.	.	1
11312	0	2.17	1	0.87711	.	.	1
11313	0	2.17	1	0.87711	.	.	1
11314	0	2.17	1	0.87711	.	.	1
11315	0	2.17	1	0.87711	.	.	1
11316	0	2.17	1	0.87711	.	.	1
11317	0	2.17	1	0.87711	.	.	1
11318	0	2.17	1	0.87711	.	.	1
11319	0	2.17	1	0.87711	.	.	1
11320	0	2.17	1	0.87711	.	.	1
11321	0	2.17	1	0.87711	.	.	1
11322	0	2.17	1	0.87711	.	.	1
11323	0	2.17	1	0.87711	.	.	1
11324	0	2.17	1	0.87711	.	.	1
11325	0	2.17	1	0.87711	.	.	1
11326	0	2.17	1	0.87711	.	.	1
11327	0	2.17	1	0.87711	.	.	1
11328	0	2.17	1	0.87711	.	.	1
11329	0	2.17	1	0.87711	.	.	1
11330	0	2.17	1	0.87711	.	.	1
11331	0	2.17	1	0.87711	.	.	1
11332	0	2.17	1	0.87711	.	.	1
11333	0	2.17	1	0.87711	.	.	1
11334	0	2.17	1	0.87711	.	.	1
11335	0	2.17	1	0.87711	.	.	1
11336	0	2.17	1	0.87711	.	.	1
11337	0	2.17	1	0.87711	.	.	1
11338	0	2.17	1	0.87711	.	.	1
11339	0	2.17	1	0.87711	.	.	1
11340	0	2.17	1	0.87711	.	.	1

Obs	Sub_group	fuptimey	_CENSOR_	SURVIVAL	SDF_LCL	SDF_UCL	STRATUM
11341	0	2.17	1	0.87711	.	.	1
11342	0	2.17	1	0.87711	.	.	1
11343	0	2.17	1	0.87711	.	.	1
11344	0	2.17	1	0.87711	.	.	1
11345	0	2.17	1	0.87711	.	.	1
11346	0	2.17	1	0.87711	.	.	1
11347	0	2.17	1	0.87711	.	.	1
11348	0	2.17	1	0.87711	.	.	1
11349	0	2.17	1	0.87711	.	.	1
11350	0	2.17	1	0.87711	.	.	1
11351	0	2.17	1	0.87711	.	.	1
11352	0	2.17	1	0.87711	.	.	1
11353	0	2.17	1	0.87711	.	.	1
11354	0	2.17	1	0.87711	.	.	1
11355	0	2.17	1	0.87711	.	.	1
11356	0	2.17	1	0.87711	.	.	1
11357	0	2.18	0	0.87682	0.87365	0.87991	1
11358	0	2.18	1	0.87682	.	.	1
11359	0	2.18	1	0.87682	.	.	1
11360	0	2.18	1	0.87682	.	.	1
11361	0	2.18	1	0.87682	.	.	1
11362	0	2.18	1	0.87682	.	.	1
11363	0	2.18	1	0.87682	.	.	1
11364	0	2.18	1	0.87682	.	.	1
11365	0	2.18	1	0.87682	.	.	1
11366	0	2.18	1	0.87682	.	.	1
11367	0	2.18	1	0.87682	.	.	1
11368	0	2.18	1	0.87682	.	.	1
11369	0	2.18	1	0.87682	.	.	1
11370	0	2.18	1	0.87682	.	.	1
11371	0	2.18	1	0.87682	.	.	1
11372	0	2.18	1	0.87682	.	.	1
11373	0	2.18	1	0.87682	.	.	1
11374	0	2.18	1	0.87682	.	.	1
11375	0	2.18	1	0.87682	.	.	1

Obs	Sub_group	fuptimey	_CENSOR_	SURVIVAL	SDF_LCL	SDF_UCL	STRATUM
**11376**	0	2.18	1	0.87682	.	.	1
**11377**	0	2.18	1	0.87682	.	.	1
**11378**	0	2.18	1	0.87682	.	.	1
**11379**	0	2.18	1	0.87682	.	.	1
**11380**	0	2.18	1	0.87682	.	.	1
**11381**	0	2.18	1	0.87682	.	.	1
**11382**	0	2.18	1	0.87682	.	.	1
**11383**	0	2.18	1	0.87682	.	.	1
**11384**	0	2.18	1	0.87682	.	.	1
**11385**	0	2.18	1	0.87682	.	.	1
**11386**	0	2.18	1	0.87682	.	.	1
**11387**	0	2.18	1	0.87682	.	.	1
**11388**	0	2.18	1	0.87682	.	.	1
**11389**	0	2.18	1	0.87682	.	.	1
**11390**	0	2.18	1	0.87682	.	.	1
**11391**	0	2.18	1	0.87682	.	.	1
**11392**	0	2.18	1	0.87682	.	.	1
**11393**	0	2.18	1	0.87682	.	.	1
**11394**	0	2.18	1	0.87682	.	.	1
**11395**	0	2.18	1	0.87682	.	.	1
**11396**	0	2.18	1	0.87682	.	.	1
**11397**	0	2.18	1	0.87682	.	.	1
**11398**	0	2.18	1	0.87682	.	.	1
**11399**	0	2.18	1	0.87682	.	.	1
**11400**	0	2.18	1	0.87682	.	.	1
**11401**	0	2.18	1	0.87682	.	.	1
**11402**	0	2.18	1	0.87682	.	.	1
**11403**	0	2.18	1	0.87682	.	.	1
**11404**	0	2.18	1	0.87682	.	.	1
**11405**	0	2.18	1	0.87682	.	.	1
**11406**	0	2.18	1	0.87682	.	.	1
**11407**	0	2.18	1	0.87682	.	.	1
**11408**	0	2.19	0	0.87656	0.87338	0.87965	1
**11409**	0	2.19	1	0.87656	.	.	1
**11410**	0	2.19	1	0.87656	.	.	1

Obs	Sub_group	fuptimey	_CENSOR_	SURVIVAL	SDF_LCL	SDF_UCL	STRATUM
11411	0	2.19	1	0.87656	.	.	1
11412	0	2.19	1	0.87656	.	.	1
11413	0	2.19	1	0.87656	.	.	1
11414	0	2.19	1	0.87656	.	.	1
11415	0	2.19	1	0.87656	.	.	1
11416	0	2.19	1	0.87656	.	.	1
11417	0	2.19	1	0.87656	.	.	1
11418	0	2.19	1	0.87656	.	.	1
11419	0	2.19	1	0.87656	.	.	1
11420	0	2.19	1	0.87656	.	.	1
11421	0	2.19	1	0.87656	.	.	1
11422	0	2.19	1	0.87656	.	.	1
11423	0	2.19	1	0.87656	.	.	1
11424	0	2.19	1	0.87656	.	.	1
11425	0	2.19	1	0.87656	.	.	1
11426	0	2.19	1	0.87656	.	.	1
11427	0	2.19	1	0.87656	.	.	1
11428	0	2.19	1	0.87656	.	.	1
11429	0	2.19	1	0.87656	.	.	1
11430	0	2.19	1	0.87656	.	.	1
11431	0	2.19	1	0.87656	.	.	1
11432	0	2.19	1	0.87656	.	.	1
11433	0	2.19	1	0.87656	.	.	1
11434	0	2.19	1	0.87656	.	.	1
11435	0	2.19	1	0.87656	.	.	1
11436	0	2.19	1	0.87656	.	.	1
11437	0	2.19	1	0.87656	.	.	1
11438	0	2.19	1	0.87656	.	.	1
11439	0	2.19	1	0.87656	.	.	1
11440	0	2.19	1	0.87656	.	.	1
11441	0	2.19	1	0.87656	.	.	1
11442	0	2.19	1	0.87656	.	.	1
11443	0	2.19	1	0.87656	.	.	1
11444	0	2.19	1	0.87656	.	.	1
11445	0	2.19	1	0.87656	.	.	1

Obs	Sub_group	fuptimey	_CENSOR_	SURVIVAL	SDF_LCL	SDF_UCL	STRATUM
11446	0	2.19	1	0.87656	.	.	1
11447	0	2.19	1	0.87656	.	.	1
11448	0	2.19	1	0.87656	.	.	1
11449	0	2.19	1	0.87656	.	.	1
11450	0	2.19	1	0.87656	.	.	1
11451	0	2.19	1	0.87656	.	.	1
11452	0	2.20	0	0.87638	0.87321	0.87948	1
11453	0	2.20	1	0.87638	.	.	1
11454	0	2.20	1	0.87638	.	.	1
11455	0	2.20	1	0.87638	.	.	1
11456	0	2.20	1	0.87638	.	.	1
11457	0	2.20	1	0.87638	.	.	1
11458	0	2.20	1	0.87638	.	.	1
11459	0	2.20	1	0.87638	.	.	1
11460	0	2.20	1	0.87638	.	.	1
11461	0	2.20	1	0.87638	.	.	1
11462	0	2.20	1	0.87638	.	.	1
11463	0	2.20	1	0.87638	.	.	1
11464	0	2.20	1	0.87638	.	.	1
11465	0	2.20	1	0.87638	.	.	1
11466	0	2.20	1	0.87638	.	.	1
11467	0	2.20	1	0.87638	.	.	1
11468	0	2.20	1	0.87638	.	.	1
11469	0	2.20	1	0.87638	.	.	1
11470	0	2.20	1	0.87638	.	.	1
11471	0	2.20	1	0.87638	.	.	1
11472	0	2.20	1	0.87638	.	.	1
11473	0	2.20	1	0.87638	.	.	1
11474	0	2.20	1	0.87638	.	.	1
11475	0	2.20	1	0.87638	.	.	1
11476	0	2.20	1	0.87638	.	.	1
11477	0	2.20	1	0.87638	.	.	1
11478	0	2.20	1	0.87638	.	.	1
11479	0	2.20	1	0.87638	.	.	1
11480	0	2.20	1	0.87638	.	.	1

Obs	Sub_group	fuptimey	_CENSOR_	SURVIVAL	SDF_LCL	SDF_UCL	STRATUM
11481	0	2.20	1	0.87638	.	.	1
11482	0	2.20	1	0.87638	.	.	1
11483	0	2.20	1	0.87638	.	.	1
11484	0	2.20	1	0.87638	.	.	1
11485	0	2.20	1	0.87638	.	.	1
11486	0	2.20	1	0.87638	.	.	1
11487	0	2.20	1	0.87638	.	.	1
11488	0	2.20	1	0.87638	.	.	1
11489	0	2.20	1	0.87638	.	.	1
11490	0	2.21	0	0.87624	0.87306	0.87934	1
11491	0	2.21	1	0.87624	.	.	1
11492	0	2.21	1	0.87624	.	.	1
11493	0	2.21	1	0.87624	.	.	1
11494	0	2.21	1	0.87624	.	.	1
11495	0	2.21	1	0.87624	.	.	1
11496	0	2.21	1	0.87624	.	.	1
11497	0	2.21	1	0.87624	.	.	1
11498	0	2.21	1	0.87624	.	.	1
11499	0	2.21	1	0.87624	.	.	1
11500	0	2.21	1	0.87624	.	.	1
11501	0	2.21	1	0.87624	.	.	1
11502	0	2.21	1	0.87624	.	.	1
11503	0	2.21	1	0.87624	.	.	1
11504	0	2.21	1	0.87624	.	.	1
11505	0	2.21	1	0.87624	.	.	1
11506	0	2.21	1	0.87624	.	.	1
11507	0	2.21	1	0.87624	.	.	1
11508	0	2.21	1	0.87624	.	.	1
11509	0	2.21	1	0.87624	.	.	1
11510	0	2.21	1	0.87624	.	.	1
11511	0	2.21	1	0.87624	.	.	1
11512	0	2.21	1	0.87624	.	.	1
11513	0	2.21	1	0.87624	.	.	1
11514	0	2.21	1	0.87624	.	.	1
11515	0	2.21	1	0.87624	.	.	1

Obs	Sub_group	fuptimey	_CENSOR_	SURVIVAL	SDF_LCL	SDF_UCL	STRATUM
11516	0	2.21	1	0.87624	.	.	1
11517	0	2.21	1	0.87624	.	.	1
11518	0	2.21	1	0.87624	.	.	1
11519	0	2.21	1	0.87624	.	.	1
11520	0	2.21	1	0.87624	.	.	1
11521	0	2.21	1	0.87624	.	.	1
11522	0	2.21	1	0.87624	.	.	1
11523	0	2.21	1	0.87624	.	.	1
11524	0	2.21	1	0.87624	.	.	1
11525	0	2.21	1	0.87624	.	.	1
11526	0	2.21	1	0.87624	.	.	1
11527	0	2.21	1	0.87624	.	.	1
11528	0	2.21	1	0.87624	.	.	1
11529	0	2.21	1	0.87624	.	.	1
11530	0	2.21	1	0.87624	.	.	1
11531	0	2.21	1	0.87624	.	.	1
11532	0	2.21	1	0.87624	.	.	1
11533	0	2.21	1	0.87624	.	.	1
11534	0	2.21	1	0.87624	.	.	1
11535	0	2.21	1	0.87624	.	.	1
11536	0	2.21	1	0.87624	.	.	1
11537	0	2.21	1	0.87624	.	.	1
11538	0	2.21	1	0.87624	.	.	1
11539	0	2.21	1	0.87624	.	.	1
11540	0	2.22	0	0.87603	0.87285	0.87914	1
11541	0	2.22	1	0.87603	.	.	1
11542	0	2.22	1	0.87603	.	.	1
11543	0	2.22	1	0.87603	.	.	1
11544	0	2.22	1	0.87603	.	.	1
11545	0	2.22	1	0.87603	.	.	1
11546	0	2.22	1	0.87603	.	.	1
11547	0	2.22	1	0.87603	.	.	1
11548	0	2.22	1	0.87603	.	.	1
11549	0	2.22	1	0.87603	.	.	1
11550	0	2.22	1	0.87603	.	.	1

Obs	Sub_group	fuptimey	_CENSOR_	SURVIVAL	SDF_LCL	SDF_UCL	STRATUM
**11551**	0	2.22	1	0.87603	.	.	1
**11552**	0	2.22	1	0.87603	.	.	1
**11553**	0	2.22	1	0.87603	.	.	1
**11554**	0	2.22	1	0.87603	.	.	1
**11555**	0	2.22	1	0.87603	.	.	1
**11556**	0	2.22	1	0.87603	.	.	1
**11557**	0	2.22	1	0.87603	.	.	1
**11558**	0	2.22	1	0.87603	.	.	1
**11559**	0	2.22	1	0.87603	.	.	1
**11560**	0	2.22	1	0.87603	.	.	1
**11561**	0	2.22	1	0.87603	.	.	1
**11562**	0	2.22	1	0.87603	.	.	1
**11563**	0	2.22	1	0.87603	.	.	1
**11564**	0	2.22	1	0.87603	.	.	1
**11565**	0	2.22	1	0.87603	.	.	1
**11566**	0	2.22	1	0.87603	.	.	1
**11567**	0	2.22	1	0.87603	.	.	1
**11568**	0	2.22	1	0.87603	.	.	1
**11569**	0	2.22	1	0.87603	.	.	1
**11570**	0	2.22	1	0.87603	.	.	1
**11571**	0	2.22	1	0.87603	.	.	1
**11572**	0	2.22	1	0.87603	.	.	1
**11573**	0	2.22	1	0.87603	.	.	1
**11574**	0	2.22	1	0.87603	.	.	1
**11575**	0	2.22	1	0.87603	.	.	1
**11576**	0	2.22	1	0.87603	.	.	1
**11577**	0	2.22	1	0.87603	.	.	1
**11578**	0	2.23	0	0.87574	0.87255	0.87885	1
**11579**	0	2.23	1	0.87574	.	.	1
**11580**	0	2.23	1	0.87574	.	.	1
**11581**	0	2.23	1	0.87574	.	.	1
**11582**	0	2.23	1	0.87574	.	.	1
**11583**	0	2.23	1	0.87574	.	.	1
**11584**	0	2.23	1	0.87574	.	.	1
**11585**	0	2.23	1	0.87574	.	.	1

Obs	Sub_group	fuptimey	_CENSOR_	SURVIVAL	SDF_LCL	SDF_UCL	STRATUM
11586	0	2.23	1	0.87574	.	.	1
11587	0	2.23	1	0.87574	.	.	1
11588	0	2.23	1	0.87574	.	.	1
11589	0	2.23	1	0.87574	.	.	1
11590	0	2.23	1	0.87574	.	.	1
11591	0	2.23	1	0.87574	.	.	1
11592	0	2.23	1	0.87574	.	.	1
11593	0	2.23	1	0.87574	.	.	1
11594	0	2.23	1	0.87574	.	.	1
11595	0	2.23	1	0.87574	.	.	1
11596	0	2.23	1	0.87574	.	.	1
11597	0	2.23	1	0.87574	.	.	1
11598	0	2.23	1	0.87574	.	.	1
11599	0	2.23	1	0.87574	.	.	1
11600	0	2.23	1	0.87574	.	.	1
11601	0	2.23	1	0.87574	.	.	1
11602	0	2.23	1	0.87574	.	.	1
11603	0	2.23	1	0.87574	.	.	1
11604	0	2.23	1	0.87574	.	.	1
11605	0	2.23	1	0.87574	.	.	1
11606	0	2.23	1	0.87574	.	.	1
11607	0	2.23	1	0.87574	.	.	1
11608	0	2.23	1	0.87574	.	.	1
11609	0	2.23	1	0.87574	.	.	1
11610	0	2.23	1	0.87574	.	.	1
11611	0	2.23	1	0.87574	.	.	1
11612	0	2.23	1	0.87574	.	.	1
11613	0	2.23	1	0.87574	.	.	1
11614	0	2.23	1	0.87574	.	.	1
11615	0	2.23	1	0.87574	.	.	1
11616	0	2.23	1	0.87574	.	.	1
11617	0	2.23	1	0.87574	.	.	1
11618	0	2.23	1	0.87574	.	.	1
11619	0	2.23	1	0.87574	.	.	1
11620	0	2.23	1	0.87574	.	.	1

Obs	Sub_group	fuptimey	_CENSOR_	SURVIVAL	SDF_LCL	SDF_UCL	STRATUM
**11621**	0	2.23	1	0.87574	.	.	1
**11622**	0	2.23	1	0.87574	.	.	1
**11623**	0	2.24	0	0.87556	0.87238	0.87868	1
**11624**	0	2.24	1	0.87556	.	.	1
**11625**	0	2.24	1	0.87556	.	.	1
**11626**	0	2.24	1	0.87556	.	.	1
**11627**	0	2.24	1	0.87556	.	.	1
**11628**	0	2.24	1	0.87556	.	.	1
**11629**	0	2.24	1	0.87556	.	.	1
**11630**	0	2.24	1	0.87556	.	.	1
**11631**	0	2.24	1	0.87556	.	.	1
**11632**	0	2.24	1	0.87556	.	.	1
**11633**	0	2.24	1	0.87556	.	.	1
**11634**	0	2.24	1	0.87556	.	.	1
**11635**	0	2.24	1	0.87556	.	.	1
**11636**	0	2.24	1	0.87556	.	.	1
**11637**	0	2.24	1	0.87556	.	.	1
**11638**	0	2.24	1	0.87556	.	.	1
**11639**	0	2.24	1	0.87556	.	.	1
**11640**	0	2.24	1	0.87556	.	.	1
**11641**	0	2.24	1	0.87556	.	.	1
**11642**	0	2.24	1	0.87556	.	.	1
**11643**	0	2.24	1	0.87556	.	.	1
**11644**	0	2.24	1	0.87556	.	.	1
**11645**	0	2.24	1	0.87556	.	.	1
**11646**	0	2.24	1	0.87556	.	.	1
**11647**	0	2.24	1	0.87556	.	.	1
**11648**	0	2.24	1	0.87556	.	.	1
**11649**	0	2.24	1	0.87556	.	.	1
**11650**	0	2.24	1	0.87556	.	.	1
**11651**	0	2.24	1	0.87556	.	.	1
**11652**	0	2.24	1	0.87556	.	.	1
**11653**	0	2.24	1	0.87556	.	.	1
**11654**	0	2.24	1	0.87556	.	.	1
**11655**	0	2.24	1	0.87556	.	.	1

Obs	Sub_group	fuptimey	_CENSOR_	SURVIVAL	SDF_LCL	SDF_UCL	STRATUM
11656	0	2.24	1	0.87556	.	.	1
11657	0	2.24	1	0.87556	.	.	1
11658	0	2.24	1	0.87556	.	.	1
11659	0	2.25	0	0.87524	0.87205	0.87836	1
11660	0	2.25	1	0.87524	.	.	1
11661	0	2.25	1	0.87524	.	.	1
11662	0	2.25	1	0.87524	.	.	1
11663	0	2.25	1	0.87524	.	.	1
11664	0	2.25	1	0.87524	.	.	1
11665	0	2.25	1	0.87524	.	.	1
11666	0	2.25	1	0.87524	.	.	1
11667	0	2.25	1	0.87524	.	.	1
11668	0	2.25	1	0.87524	.	.	1
11669	0	2.25	1	0.87524	.	.	1
11670	0	2.25	1	0.87524	.	.	1
11671	0	2.25	1	0.87524	.	.	1
11672	0	2.25	1	0.87524	.	.	1
11673	0	2.25	1	0.87524	.	.	1
11674	0	2.25	1	0.87524	.	.	1
11675	0	2.25	1	0.87524	.	.	1
11676	0	2.25	1	0.87524	.	.	1
11677	0	2.25	1	0.87524	.	.	1
11678	0	2.25	1	0.87524	.	.	1
11679	0	2.25	1	0.87524	.	.	1
11680	0	2.25	1	0.87524	.	.	1
11681	0	2.25	1	0.87524	.	.	1
11682	0	2.25	1	0.87524	.	.	1
11683	0	2.25	1	0.87524	.	.	1
11684	0	2.25	1	0.87524	.	.	1
11685	0	2.25	1	0.87524	.	.	1
11686	0	2.25	1	0.87524	.	.	1
11687	0	2.25	1	0.87524	.	.	1
11688	0	2.25	1	0.87524	.	.	1
11689	0	2.25	1	0.87524	.	.	1
11690	0	2.25	1	0.87524	.	.	1

Obs	Sub_group	fuptimey	_CENSOR_	SURVIVAL	SDF_LCL	SDF_UCL	STRATUM
11691	0	2.25	1	0.87524	.	.	1
11692	0	2.25	1	0.87524	.	.	1
11693	0	2.25	1	0.87524	.	.	1
11694	0	2.25	1	0.87524	.	.	1
11695	0	2.25	1	0.87524	.	.	1
11696	0	2.25	1	0.87524	.	.	1
11697	0	2.25	1	0.87524	.	.	1
11698	0	2.25	1	0.87524	.	.	1
11699	0	2.25	1	0.87524	.	.	1
11700	0	2.25	1	0.87524	.	.	1
11701	0	2.25	1	0.87524	.	.	1
11702	0	2.25	1	0.87524	.	.	1
11703	0	2.25	1	0.87524	.	.	1
11704	0	2.25	1	0.87524	.	.	1
11705	0	2.25	1	0.87524	.	.	1
11706	0	2.25	1	0.87524	.	.	1
11707	0	2.25	1	0.87524	.	.	1
11708	0	2.25	1	0.87524	.	.	1
11709	0	2.25	1	0.87524	.	.	1
11710	0	2.25	1	0.87524	.	.	1
11711	0	2.25	1	0.87524	.	.	1
11712	0	2.25	1	0.87524	.	.	1
11713	0	2.25	1	0.87524	.	.	1
11714	0	2.25	1	0.87524	.	.	1
11715	0	2.25	1	0.87524	.	.	1
11716	0	2.25	1	0.87524	.	.	1
11717	0	2.25	1	0.87524	.	.	1
11718	0	2.26	0	0.87495	0.87175	0.87807	1
11719	0	2.26	1	0.87495	.	.	1
11720	0	2.26	1	0.87495	.	.	1
11721	0	2.26	1	0.87495	.	.	1
11722	0	2.26	1	0.87495	.	.	1
11723	0	2.26	1	0.87495	.	.	1
11724	0	2.26	1	0.87495	.	.	1
11725	0	2.26	1	0.87495	.	.	1

Obs	Sub_group	fuptimey	_CENSOR_	SURVIVAL	SDF_LCL	SDF_UCL	STRATUM
**11726**	0	2.26	1	0.87495	.	.	1
**11727**	0	2.26	1	0.87495	.	.	1
**11728**	0	2.26	1	0.87495	.	.	1
**11729**	0	2.26	1	0.87495	.	.	1
**11730**	0	2.26	1	0.87495	.	.	1
**11731**	0	2.26	1	0.87495	.	.	1
**11732**	0	2.26	1	0.87495	.	.	1
**11733**	0	2.26	1	0.87495	.	.	1
**11734**	0	2.26	1	0.87495	.	.	1
**11735**	0	2.26	1	0.87495	.	.	1
**11736**	0	2.26	1	0.87495	.	.	1
**11737**	0	2.26	1	0.87495	.	.	1
**11738**	0	2.26	1	0.87495	.	.	1
**11739**	0	2.26	1	0.87495	.	.	1
**11740**	0	2.26	1	0.87495	.	.	1
**11741**	0	2.26	1	0.87495	.	.	1
**11742**	0	2.26	1	0.87495	.	.	1
**11743**	0	2.26	1	0.87495	.	.	1
**11744**	0	2.26	1	0.87495	.	.	1
**11745**	0	2.26	1	0.87495	.	.	1
**11746**	0	2.26	1	0.87495	.	.	1
**11747**	0	2.26	1	0.87495	.	.	1
**11748**	0	2.26	1	0.87495	.	.	1
**11749**	0	2.26	1	0.87495	.	.	1
**11750**	0	2.26	1	0.87495	.	.	1
**11751**	0	2.26	1	0.87495	.	.	1
**11752**	0	2.26	1	0.87495	.	.	1
**11753**	0	2.26	1	0.87495	.	.	1
**11754**	0	2.26	1	0.87495	.	.	1
**11755**	0	2.26	1	0.87495	.	.	1
**11756**	0	2.26	1	0.87495	.	.	1
**11757**	0	2.26	1	0.87495	.	.	1
**11758**	0	2.27	0	0.87468	0.87148	0.87781	1
**11759**	0	2.27	1	0.87468	.	.	1
**11760**	0	2.27	1	0.87468	.	.	1

Obs	Sub_group	fuptimey	_CENSOR_	SURVIVAL	SDF_LCL	SDF_UCL	STRATUM
11761	0	2.27	1	0.87468	.	.	1
11762	0	2.27	1	0.87468	.	.	1
11763	0	2.27	1	0.87468	.	.	1
11764	0	2.27	1	0.87468	.	.	1
11765	0	2.27	1	0.87468	.	.	1
11766	0	2.27	1	0.87468	.	.	1
11767	0	2.27	1	0.87468	.	.	1
11768	0	2.27	1	0.87468	.	.	1
11769	0	2.27	1	0.87468	.	.	1
11770	0	2.27	1	0.87468	.	.	1
11771	0	2.27	1	0.87468	.	.	1
11772	0	2.27	1	0.87468	.	.	1
11773	0	2.27	1	0.87468	.	.	1
11774	0	2.27	1	0.87468	.	.	1
11775	0	2.27	1	0.87468	.	.	1
11776	0	2.27	1	0.87468	.	.	1
11777	0	2.27	1	0.87468	.	.	1
11778	0	2.27	1	0.87468	.	.	1
11779	0	2.27	1	0.87468	.	.	1
11780	0	2.27	1	0.87468	.	.	1
11781	0	2.27	1	0.87468	.	.	1
11782	0	2.27	1	0.87468	.	.	1
11783	0	2.27	1	0.87468	.	.	1
11784	0	2.27	1	0.87468	.	.	1
11785	0	2.27	1	0.87468	.	.	1
11786	0	2.27	1	0.87468	.	.	1
11787	0	2.27	1	0.87468	.	.	1
11788	0	2.27	1	0.87468	.	.	1
11789	0	2.27	1	0.87468	.	.	1
11790	0	2.27	1	0.87468	.	.	1
11791	0	2.27	1	0.87468	.	.	1
11792	0	2.27	1	0.87468	.	.	1
11793	0	2.27	1	0.87468	.	.	1
11794	0	2.27	1	0.87468	.	.	1
11795	0	2.27	1	0.87468	.	.	1

Obs	Sub_group	fuptimey	_CENSOR_	SURVIVAL	SDF_LCL	SDF_UCL	STRATUM
11796	0	2.27	1	0.87468	.	.	1
11797	0	2.27	1	0.87468	.	.	1
11798	0	2.27	1	0.87468	.	.	1
11799	0	2.27	1	0.87468	.	.	1
11800	0	2.27	1	0.87468	.	.	1
11801	0	2.27	1	0.87468	.	.	1
11802	0	2.27	1	0.87468	.	.	1
11803	0	2.27	1	0.87468	.	.	1
11804	0	2.27	1	0.87468	.	.	1
11805	0	2.27	1	0.87468	.	.	1
11806	0	2.27	1	0.87468	.	.	1
11807	0	2.27	1	0.87468	.	.	1
11808	0	2.28	0	0.87454	0.87133	0.87766	1
11809	0	2.28	1	0.87454	.	.	1
11810	0	2.28	1	0.87454	.	.	1
11811	0	2.28	1	0.87454	.	.	1
11812	0	2.28	1	0.87454	.	.	1
11813	0	2.28	1	0.87454	.	.	1
11814	0	2.28	1	0.87454	.	.	1
11815	0	2.28	1	0.87454	.	.	1
11816	0	2.28	1	0.87454	.	.	1
11817	0	2.28	1	0.87454	.	.	1
11818	0	2.28	1	0.87454	.	.	1
11819	0	2.28	1	0.87454	.	.	1
11820	0	2.28	1	0.87454	.	.	1
11821	0	2.28	1	0.87454	.	.	1
11822	0	2.28	1	0.87454	.	.	1
11823	0	2.28	1	0.87454	.	.	1
11824	0	2.28	1	0.87454	.	.	1
11825	0	2.28	1	0.87454	.	.	1
11826	0	2.28	1	0.87454	.	.	1
11827	0	2.28	1	0.87454	.	.	1
11828	0	2.28	1	0.87454	.	.	1
11829	0	2.28	1	0.87454	.	.	1
11830	0	2.28	1	0.87454	.	.	1

Obs	Sub_group	fuptimey	_CENSOR_	SURVIVAL	SDF_LCL	SDF_UCL	STRATUM
11831	0	2.28	1	0.87454	.	.	1
11832	0	2.28	1	0.87454	.	.	1
11833	0	2.28	1	0.87454	.	.	1
11834	0	2.28	1	0.87454	.	.	1
11835	0	2.28	1	0.87454	.	.	1
11836	0	2.28	1	0.87454	.	.	1
11837	0	2.28	1	0.87454	.	.	1
11838	0	2.28	1	0.87454	.	.	1
11839	0	2.28	1	0.87454	.	.	1
11840	0	2.28	1	0.87454	.	.	1
11841	0	2.28	1	0.87454	.	.	1
11842	0	2.28	1	0.87454	.	.	1
11843	0	2.28	1	0.87454	.	.	1
11844	0	2.28	1	0.87454	.	.	1
11845	0	2.28	1	0.87454	.	.	1
11846	0	2.28	1	0.87454	.	.	1
11847	0	2.28	1	0.87454	.	.	1
11848	0	2.28	1	0.87454	.	.	1
11849	0	2.29	0	0.87421	0.87101	0.87734	1
11850	0	2.29	1	0.87421	.	.	1
11851	0	2.29	1	0.87421	.	.	1
11852	0	2.29	1	0.87421	.	.	1
11853	0	2.29	1	0.87421	.	.	1
11854	0	2.29	1	0.87421	.	.	1
11855	0	2.29	1	0.87421	.	.	1
11856	0	2.29	1	0.87421	.	.	1
11857	0	2.29	1	0.87421	.	.	1
11858	0	2.29	1	0.87421	.	.	1
11859	0	2.29	1	0.87421	.	.	1
11860	0	2.29	1	0.87421	.	.	1
11861	0	2.29	1	0.87421	.	.	1
11862	0	2.29	1	0.87421	.	.	1
11863	0	2.29	1	0.87421	.	.	1
11864	0	2.29	1	0.87421	.	.	1
11865	0	2.29	1	0.87421	.	.	1

Obs	Sub_group	fuptimey	_CENSOR_	SURVIVAL	SDF_LCL	SDF_UCL	STRATUM
11866	0	2.29	1	0.87421	.	.	1
11867	0	2.29	1	0.87421	.	.	1
11868	0	2.29	1	0.87421	.	.	1
11869	0	2.29	1	0.87421	.	.	1
11870	0	2.29	1	0.87421	.	.	1
11871	0	2.29	1	0.87421	.	.	1
11872	0	2.29	1	0.87421	.	.	1
11873	0	2.29	1	0.87421	.	.	1
11874	0	2.29	1	0.87421	.	.	1
11875	0	2.29	1	0.87421	.	.	1
11876	0	2.29	1	0.87421	.	.	1
11877	0	2.29	1	0.87421	.	.	1
11878	0	2.29	1	0.87421	.	.	1
11879	0	2.29	1	0.87421	.	.	1
11880	0	2.29	1	0.87421	.	.	1
11881	0	2.29	1	0.87421	.	.	1
11882	0	2.29	1	0.87421	.	.	1
11883	0	2.29	1	0.87421	.	.	1
11884	0	2.29	1	0.87421	.	.	1
11885	0	2.29	1	0.87421	.	.	1
11886	0	2.29	1	0.87421	.	.	1
11887	0	2.29	1	0.87421	.	.	1
11888	0	2.29	1	0.87421	.	.	1
11889	0	2.29	1	0.87421	.	.	1
11890	0	2.29	1	0.87421	.	.	1
11891	0	2.29	1	0.87421	.	.	1
11892	0	2.29	1	0.87421	.	.	1
11893	0	2.29	1	0.87421	.	.	1
11894	0	2.29	1	0.87421	.	.	1
11895	0	2.29	1	0.87421	.	.	1
11896	0	2.29	1	0.87421	.	.	1
11897	0	2.29	1	0.87421	.	.	1
11898	0	2.30	0	0.87395	0.87074	0.87708	1
11899	0	2.30	1	0.87395	.	.	1
11900	0	2.30	1	0.87395	.	.	1

Obs	Sub_group	fuptimey	_CENSOR_	SURVIVAL	SDF_LCL	SDF_UCL	STRATUM
**11901**	0	2.30	1	0.87395	.	.	1
**11902**	0	2.30	1	0.87395	.	.	1
**11903**	0	2.30	1	0.87395	.	.	1
**11904**	0	2.30	1	0.87395	.	.	1
**11905**	0	2.30	1	0.87395	.	.	1
**11906**	0	2.30	1	0.87395	.	.	1
**11907**	0	2.30	1	0.87395	.	.	1
**11908**	0	2.30	1	0.87395	.	.	1
**11909**	0	2.30	1	0.87395	.	.	1
**11910**	0	2.30	1	0.87395	.	.	1
**11911**	0	2.30	1	0.87395	.	.	1
**11912**	0	2.30	1	0.87395	.	.	1
**11913**	0	2.30	1	0.87395	.	.	1
**11914**	0	2.30	1	0.87395	.	.	1
**11915**	0	2.30	1	0.87395	.	.	1
**11916**	0	2.30	1	0.87395	.	.	1
**11917**	0	2.30	1	0.87395	.	.	1
**11918**	0	2.30	1	0.87395	.	.	1
**11919**	0	2.30	1	0.87395	.	.	1
**11920**	0	2.30	1	0.87395	.	.	1
**11921**	0	2.30	1	0.87395	.	.	1
**11922**	0	2.30	1	0.87395	.	.	1
**11923**	0	2.30	1	0.87395	.	.	1
**11924**	0	2.30	1	0.87395	.	.	1
**11925**	0	2.30	1	0.87395	.	.	1
**11926**	0	2.30	1	0.87395	.	.	1
**11927**	0	2.30	1	0.87395	.	.	1
**11928**	0	2.30	1	0.87395	.	.	1
**11929**	0	2.30	1	0.87395	.	.	1
**11930**	0	2.30	1	0.87395	.	.	1
**11931**	0	2.30	1	0.87395	.	.	1
**11932**	0	2.30	1	0.87395	.	.	1
**11933**	0	2.30	1	0.87395	.	.	1
**11934**	0	2.30	1	0.87395	.	.	1
**11935**	0	2.30	1	0.87395	.	.	1

Obs	Sub_group	fuptimey	_CENSOR_	SURVIVAL	SDF_LCL	SDF_UCL	STRATUM
11936	0	2.30	1	0.87395	.	.	1
11937	0	2.30	1	0.87395	.	.	1
11938	0	2.30	1	0.87395	.	.	1
11939	0	2.30	1	0.87395	.	.	1
11940	0	2.30	1	0.87395	.	.	1
11941	0	2.30	1	0.87395	.	.	1
11942	0	2.30	1	0.87395	.	.	1
11943	0	2.31	0	0.87380	0.87059	0.87694	1
11944	0	2.31	1	0.87380	.	.	1
11945	0	2.31	1	0.87380	.	.	1
11946	0	2.31	1	0.87380	.	.	1
11947	0	2.31	1	0.87380	.	.	1
11948	0	2.31	1	0.87380	.	.	1
11949	0	2.31	1	0.87380	.	.	1
11950	0	2.31	1	0.87380	.	.	1
11951	0	2.31	1	0.87380	.	.	1
11952	0	2.31	1	0.87380	.	.	1
11953	0	2.31	1	0.87380	.	.	1
11954	0	2.31	1	0.87380	.	.	1
11955	0	2.31	1	0.87380	.	.	1
11956	0	2.31	1	0.87380	.	.	1
11957	0	2.31	1	0.87380	.	.	1
11958	0	2.31	1	0.87380	.	.	1
11959	0	2.31	1	0.87380	.	.	1
11960	0	2.31	1	0.87380	.	.	1
11961	0	2.31	1	0.87380	.	.	1
11962	0	2.31	1	0.87380	.	.	1
11963	0	2.31	1	0.87380	.	.	1
11964	0	2.31	1	0.87380	.	.	1
11965	0	2.31	1	0.87380	.	.	1
11966	0	2.31	1	0.87380	.	.	1
11967	0	2.31	1	0.87380	.	.	1
11968	0	2.31	1	0.87380	.	.	1
11969	0	2.31	1	0.87380	.	.	1
11970	0	2.31	1	0.87380	.	.	1

Obs	Sub_group	fuptimey	_CENSOR_	SURVIVAL	SDF_LCL	SDF_UCL	STRATUM
11971	0	2.31	1	0.87380	.	.	1
11972	0	2.31	1	0.87380	.	.	1
11973	0	2.31	1	0.87380	.	.	1
11974	0	2.31	1	0.87380	.	.	1
11975	0	2.31	1	0.87380	.	.	1
11976	0	2.31	1	0.87380	.	.	1
11977	0	2.31	1	0.87380	.	.	1
11978	0	2.31	1	0.87380	.	.	1
11979	0	2.31	1	0.87380	.	.	1
11980	0	2.31	1	0.87380	.	.	1
11981	0	2.31	1	0.87380	.	.	1
11982	0	2.31	1	0.87380	.	.	1
11983	0	2.31	1	0.87380	.	.	1
11984	0	2.31	1	0.87380	.	.	1
11985	0	2.31	1	0.87380	.	.	1
11986	0	2.31	1	0.87380	.	.	1
11987	0	2.31	1	0.87380	.	.	1
11988	0	2.31	1	0.87380	.	.	1
11989	0	2.31	1	0.87380	.	.	1
11990	0	2.31	1	0.87380	.	.	1
11991	0	2.31	1	0.87380	.	.	1
11992	0	2.31	1	0.87380	.	.	1
11993	0	2.32	0	0.87353	0.87032	0.87667	1
11994	0	2.32	1	0.87353	.	.	1
11995	0	2.32	1	0.87353	.	.	1
11996	0	2.32	1	0.87353	.	.	1
11997	0	2.32	1	0.87353	.	.	1
11998	0	2.32	1	0.87353	.	.	1
11999	0	2.32	1	0.87353	.	.	1
12000	0	2.32	1	0.87353	.	.	1
12001	0	2.32	1	0.87353	.	.	1
12002	0	2.32	1	0.87353	.	.	1
12003	0	2.32	1	0.87353	.	.	1
12004	0	2.32	1	0.87353	.	.	1
12005	0	2.32	1	0.87353	.	.	1

Obs	Sub_group	fuptimey	_CENSOR_	SURVIVAL	SDF_LCL	SDF_UCL	STRATUM
12006	0	2.32	1	0.87353	.	.	1
12007	0	2.32	1	0.87353	.	.	1
12008	0	2.32	1	0.87353	.	.	1
12009	0	2.32	1	0.87353	.	.	1
12010	0	2.32	1	0.87353	.	.	1
12011	0	2.32	1	0.87353	.	.	1
12012	0	2.32	1	0.87353	.	.	1
12013	0	2.32	1	0.87353	.	.	1
12014	0	2.32	1	0.87353	.	.	1
12015	0	2.32	1	0.87353	.	.	1
12016	0	2.32	1	0.87353	.	.	1
12017	0	2.32	1	0.87353	.	.	1
12018	0	2.32	1	0.87353	.	.	1
12019	0	2.32	1	0.87353	.	.	1
12020	0	2.32	1	0.87353	.	.	1
12021	0	2.32	1	0.87353	.	.	1
12022	0	2.32	1	0.87353	.	.	1
12023	0	2.32	1	0.87353	.	.	1
12024	0	2.32	1	0.87353	.	.	1
12025	0	2.32	1	0.87353	.	.	1
12026	0	2.32	1	0.87353	.	.	1
12027	0	2.32	1	0.87353	.	.	1
12028	0	2.32	1	0.87353	.	.	1
12029	0	2.32	1	0.87353	.	.	1
12030	0	2.32	1	0.87353	.	.	1
12031	0	2.32	1	0.87353	.	.	1
12032	0	2.32	1	0.87353	.	.	1
12033	0	2.32	1	0.87353	.	.	1
12034	0	2.33	0	0.87315	0.86992	0.87629	1
12035	0	2.33	1	0.87315	.	.	1
12036	0	2.33	1	0.87315	.	.	1
12037	0	2.33	1	0.87315	.	.	1
12038	0	2.33	1	0.87315	.	.	1
12039	0	2.33	1	0.87315	.	.	1
12040	0	2.33	1	0.87315	.	.	1

Obs	Sub_group	fuptimey	_CENSOR_	SURVIVAL	SDF_LCL	SDF_UCL	STRATUM
12041	0	2.33	1	0.87315	.	.	1
12042	0	2.33	1	0.87315	.	.	1
12043	0	2.33	1	0.87315	.	.	1
12044	0	2.33	1	0.87315	.	.	1
12045	0	2.33	1	0.87315	.	.	1
12046	0	2.33	1	0.87315	.	.	1
12047	0	2.33	1	0.87315	.	.	1
12048	0	2.33	1	0.87315	.	.	1
12049	0	2.33	1	0.87315	.	.	1
12050	0	2.33	1	0.87315	.	.	1
12051	0	2.33	1	0.87315	.	.	1
12052	0	2.33	1	0.87315	.	.	1
12053	0	2.33	1	0.87315	.	.	1
12054	0	2.33	1	0.87315	.	.	1
12055	0	2.33	1	0.87315	.	.	1
12056	0	2.33	1	0.87315	.	.	1
12057	0	2.33	1	0.87315	.	.	1
12058	0	2.33	1	0.87315	.	.	1
12059	0	2.33	1	0.87315	.	.	1
12060	0	2.33	1	0.87315	.	.	1
12061	0	2.33	1	0.87315	.	.	1
12062	0	2.33	1	0.87315	.	.	1
12063	0	2.33	1	0.87315	.	.	1
12064	0	2.33	1	0.87315	.	.	1
12065	0	2.33	1	0.87315	.	.	1
12066	0	2.33	1	0.87315	.	.	1
12067	0	2.33	1	0.87315	.	.	1
12068	0	2.33	1	0.87315	.	.	1
12069	0	2.34	0	0.87288	0.86965	0.87603	1
12070	0	2.34	1	0.87288	.	.	1
12071	0	2.34	1	0.87288	.	.	1
12072	0	2.34	1	0.87288	.	.	1
12073	0	2.34	1	0.87288	.	.	1
12074	0	2.34	1	0.87288	.	.	1
12075	0	2.34	1	0.87288	.	.	1

Obs	Sub_group	fuptimey	_CENSOR_	SURVIVAL	SDF_LCL	SDF_UCL	STRATUM
12076	0	2.34	1	0.87288	.	.	1
12077	0	2.34	1	0.87288	.	.	1
12078	0	2.34	1	0.87288	.	.	1
12079	0	2.34	1	0.87288	.	.	1
12080	0	2.34	1	0.87288	.	.	1
12081	0	2.34	1	0.87288	.	.	1
12082	0	2.34	1	0.87288	.	.	1
12083	0	2.34	1	0.87288	.	.	1
12084	0	2.34	1	0.87288	.	.	1
12085	0	2.34	1	0.87288	.	.	1
12086	0	2.34	1	0.87288	.	.	1
12087	0	2.34	1	0.87288	.	.	1
12088	0	2.34	1	0.87288	.	.	1
12089	0	2.34	1	0.87288	.	.	1
12090	0	2.34	1	0.87288	.	.	1
12091	0	2.34	1	0.87288	.	.	1
12092	0	2.34	1	0.87288	.	.	1
12093	0	2.34	1	0.87288	.	.	1
12094	0	2.34	1	0.87288	.	.	1
12095	0	2.34	1	0.87288	.	.	1
12096	0	2.34	1	0.87288	.	.	1
12097	0	2.34	1	0.87288	.	.	1
12098	0	2.34	1	0.87288	.	.	1
12099	0	2.34	1	0.87288	.	.	1
12100	0	2.34	1	0.87288	.	.	1
12101	0	2.34	1	0.87288	.	.	1
12102	0	2.34	1	0.87288	.	.	1
12103	0	2.34	1	0.87288	.	.	1
12104	0	2.34	1	0.87288	.	.	1
12105	0	2.34	1	0.87288	.	.	1
12106	0	2.34	1	0.87288	.	.	1
12107	0	2.34	1	0.87288	.	.	1
12108	0	2.34	1	0.87288	.	.	1
12109	0	2.34	1	0.87288	.	.	1
12110	0	2.34	1	0.87288	.	.	1

Obs	Sub_group	fuptimey	_CENSOR_	SURVIVAL	SDF_LCL	SDF_UCL	STRATUM
**12111**	0	2.34	1	0.87288	.	.	1
**12112**	0	2.34	1	0.87288	.	.	1
**12113**	0	2.34	1	0.87288	.	.	1
**12114**	0	2.34	1	0.87288	.	.	1
**12115**	0	2.34	1	0.87288	.	.	1
**12116**	0	2.34	1	0.87288	.	.	1
**12117**	0	2.34	1	0.87288	.	.	1
**12118**	0	2.34	1	0.87288	.	.	1
**12119**	0	2.34	1	0.87288	.	.	1
**12120**	0	2.34	1	0.87288	.	.	1
**12121**	0	2.34	1	0.87288	.	.	1
**12122**	0	2.34	1	0.87288	.	.	1
**12123**	0	2.34	1	0.87288	.	.	1
**12124**	0	2.34	1	0.87288	.	.	1
**12125**	0	2.34	1	0.87288	.	.	1
**12126**	0	2.34	1	0.87288	.	.	1
**12127**	0	2.34	1	0.87288	.	.	1
**12128**	0	2.34	1	0.87288	.	.	1
**12129**	0	2.34	1	0.87288	.	.	1
**12130**	0	2.34	1	0.87288	.	.	1
**12131**	0	2.35	0	0.87261	0.86938	0.87577	1
**12132**	0	2.35	1	0.87261	.	.	1
**12133**	0	2.35	1	0.87261	.	.	1
**12134**	0	2.35	1	0.87261	.	.	1
**12135**	0	2.35	1	0.87261	.	.	1
**12136**	0	2.35	1	0.87261	.	.	1
**12137**	0	2.35	1	0.87261	.	.	1
**12138**	0	2.35	1	0.87261	.	.	1
**12139**	0	2.35	1	0.87261	.	.	1
**12140**	0	2.35	1	0.87261	.	.	1
**12141**	0	2.35	1	0.87261	.	.	1
**12142**	0	2.35	1	0.87261	.	.	1
**12143**	0	2.35	1	0.87261	.	.	1
**12144**	0	2.35	1	0.87261	.	.	1
**12145**	0	2.35	1	0.87261	.	.	1

Obs	Sub_group	fuptimey	_CENSOR_	SURVIVAL	SDF_LCL	SDF_UCL	STRATUM
12146	0	2.35	1	0.87261	.	.	1
12147	0	2.35	1	0.87261	.	.	1
12148	0	2.35	1	0.87261	.	.	1
12149	0	2.35	1	0.87261	.	.	1
12150	0	2.35	1	0.87261	.	.	1
12151	0	2.35	1	0.87261	.	.	1
12152	0	2.35	1	0.87261	.	.	1
12153	0	2.35	1	0.87261	.	.	1
12154	0	2.35	1	0.87261	.	.	1
12155	0	2.35	1	0.87261	.	.	1
12156	0	2.35	1	0.87261	.	.	1
12157	0	2.35	1	0.87261	.	.	1
12158	0	2.35	1	0.87261	.	.	1
12159	0	2.35	1	0.87261	.	.	1
12160	0	2.35	1	0.87261	.	.	1
12161	0	2.35	1	0.87261	.	.	1
12162	0	2.35	1	0.87261	.	.	1
12163	0	2.35	1	0.87261	.	.	1
12164	0	2.35	1	0.87261	.	.	1
12165	0	2.35	1	0.87261	.	.	1
12166	0	2.35	1	0.87261	.	.	1
12167	0	2.35	1	0.87261	.	.	1
12168	0	2.35	1	0.87261	.	.	1
12169	0	2.35	1	0.87261	.	.	1
12170	0	2.35	1	0.87261	.	.	1
12171	0	2.35	1	0.87261	.	.	1
12172	0	2.35	1	0.87261	.	.	1
12173	0	2.35	1	0.87261	.	.	1
12174	0	2.36	0	0.87243	0.86920	0.87559	1
12175	0	2.36	1	0.87243	.	.	1
12176	0	2.36	1	0.87243	.	.	1
12177	0	2.36	1	0.87243	.	.	1
12178	0	2.36	1	0.87243	.	.	1
12179	0	2.36	1	0.87243	.	.	1
12180	0	2.36	1	0.87243	.	.	1

Obs	Sub_group	fuptimey	_CENSOR_	SURVIVAL	SDF_LCL	SDF_UCL	STRATUM
12181	0	2.36	1	0.87243	.	.	1
12182	0	2.36	1	0.87243	.	.	1
12183	0	2.36	1	0.87243	.	.	1
12184	0	2.36	1	0.87243	.	.	1
12185	0	2.36	1	0.87243	.	.	1
12186	0	2.36	1	0.87243	.	.	1
12187	0	2.36	1	0.87243	.	.	1
12188	0	2.36	1	0.87243	.	.	1
12189	0	2.36	1	0.87243	.	.	1
12190	0	2.36	1	0.87243	.	.	1
12191	0	2.36	1	0.87243	.	.	1
12192	0	2.36	1	0.87243	.	.	1
12193	0	2.36	1	0.87243	.	.	1
12194	0	2.36	1	0.87243	.	.	1
12195	0	2.36	1	0.87243	.	.	1
12196	0	2.36	1	0.87243	.	.	1
12197	0	2.36	1	0.87243	.	.	1
12198	0	2.36	1	0.87243	.	.	1
12199	0	2.36	1	0.87243	.	.	1
12200	0	2.36	1	0.87243	.	.	1
12201	0	2.36	1	0.87243	.	.	1
12202	0	2.36	1	0.87243	.	.	1
12203	0	2.36	1	0.87243	.	.	1
12204	0	2.36	1	0.87243	.	.	1
12205	0	2.36	1	0.87243	.	.	1
12206	0	2.36	1	0.87243	.	.	1
12207	0	2.36	1	0.87243	.	.	1
12208	0	2.36	1	0.87243	.	.	1
12209	0	2.36	1	0.87243	.	.	1
12210	0	2.36	1	0.87243	.	.	1
12211	0	2.36	1	0.87243	.	.	1
12212	0	2.36	1	0.87243	.	.	1
12213	0	2.36	1	0.87243	.	.	1
12214	0	2.36	1	0.87243	.	.	1
12215	0	2.36	1	0.87243	.	.	1

Obs	Sub_group	fuptimey	_CENSOR_	SURVIVAL	SDF_LCL	SDF_UCL	STRATUM
12216	0	2.36	1	0.87243	.	.	1
12217	0	2.36	1	0.87243	.	.	1
12218	0	2.36	1	0.87243	.	.	1
12219	0	2.36	1	0.87243	.	.	1
12220	0	2.36	1	0.87243	.	.	1
12221	0	2.37	0	0.87193	0.86869	0.87509	1
12222	0	2.37	1	0.87193	.	.	1
12223	0	2.37	1	0.87193	.	.	1
12224	0	2.37	1	0.87193	.	.	1
12225	0	2.37	1	0.87193	.	.	1
12226	0	2.37	1	0.87193	.	.	1
12227	0	2.37	1	0.87193	.	.	1
12228	0	2.37	1	0.87193	.	.	1
12229	0	2.37	1	0.87193	.	.	1
12230	0	2.37	1	0.87193	.	.	1
12231	0	2.37	1	0.87193	.	.	1
12232	0	2.37	1	0.87193	.	.	1
12233	0	2.37	1	0.87193	.	.	1
12234	0	2.37	1	0.87193	.	.	1
12235	0	2.37	1	0.87193	.	.	1
12236	0	2.37	1	0.87193	.	.	1
12237	0	2.37	1	0.87193	.	.	1
12238	0	2.37	1	0.87193	.	.	1
12239	0	2.37	1	0.87193	.	.	1
12240	0	2.37	1	0.87193	.	.	1
12241	0	2.37	1	0.87193	.	.	1
12242	0	2.37	1	0.87193	.	.	1
12243	0	2.37	1	0.87193	.	.	1
12244	0	2.37	1	0.87193	.	.	1
12245	0	2.37	1	0.87193	.	.	1
12246	0	2.37	1	0.87193	.	.	1
12247	0	2.37	1	0.87193	.	.	1
12248	0	2.37	1	0.87193	.	.	1
12249	0	2.37	1	0.87193	.	.	1
12250	0	2.37	1	0.87193	.	.	1

Obs	Sub_group	fuptimey	_CENSOR_	SURVIVAL	SDF_LCL	SDF_UCL	STRATUM
12251	0	2.37	1	0.87193	.	.	1
12252	0	2.37	1	0.87193	.	.	1
12253	0	2.37	1	0.87193	.	.	1
12254	0	2.37	1	0.87193	.	.	1
12255	0	2.37	1	0.87193	.	.	1
12256	0	2.37	1	0.87193	.	.	1
12257	0	2.37	1	0.87193	.	.	1
12258	0	2.37	1	0.87193	.	.	1
12259	0	2.38	0	0.87154	0.86829	0.87471	1
12260	0	2.38	1	0.87154	.	.	1
12261	0	2.38	1	0.87154	.	.	1
12262	0	2.38	1	0.87154	.	.	1
12263	0	2.38	1	0.87154	.	.	1
12264	0	2.38	1	0.87154	.	.	1
12265	0	2.38	1	0.87154	.	.	1
12266	0	2.38	1	0.87154	.	.	1
12267	0	2.38	1	0.87154	.	.	1
12268	0	2.38	1	0.87154	.	.	1
12269	0	2.38	1	0.87154	.	.	1
12270	0	2.38	1	0.87154	.	.	1
12271	0	2.38	1	0.87154	.	.	1
12272	0	2.38	1	0.87154	.	.	1
12273	0	2.38	1	0.87154	.	.	1
12274	0	2.38	1	0.87154	.	.	1
12275	0	2.38	1	0.87154	.	.	1
12276	0	2.38	1	0.87154	.	.	1
12277	0	2.38	1	0.87154	.	.	1
12278	0	2.38	1	0.87154	.	.	1
12279	0	2.38	1	0.87154	.	.	1
12280	0	2.38	1	0.87154	.	.	1
12281	0	2.38	1	0.87154	.	.	1
12282	0	2.38	1	0.87154	.	.	1
12283	0	2.38	1	0.87154	.	.	1
12284	0	2.38	1	0.87154	.	.	1
12285	0	2.38	1	0.87154	.	.	1

Obs	Sub_group	fuptimey	_CENSOR_	SURVIVAL	SDF_LCL	SDF_UCL	STRATUM
12286	0	2.38	1	0.87154	.	.	1
12287	0	2.38	1	0.87154	.	.	1
12288	0	2.38	1	0.87154	.	.	1
12289	0	2.38	1	0.87154	.	.	1
12290	0	2.38	1	0.87154	.	.	1
12291	0	2.38	1	0.87154	.	.	1
12292	0	2.38	1	0.87154	.	.	1
12293	0	2.38	1	0.87154	.	.	1
12294	0	2.38	1	0.87154	.	.	1
12295	0	2.38	1	0.87154	.	.	1
12296	0	2.38	1	0.87154	.	.	1
12297	0	2.38	1	0.87154	.	.	1
12298	0	2.38	1	0.87154	.	.	1
12299	0	2.38	1	0.87154	.	.	1
12300	0	2.38	1	0.87154	.	.	1
12301	0	2.38	1	0.87154	.	.	1
12302	0	2.38	1	0.87154	.	.	1
12303	0	2.38	1	0.87154	.	.	1
12304	0	2.38	1	0.87154	.	.	1
12305	0	2.38	1	0.87154	.	.	1
12306	0	2.38	1	0.87154	.	.	1
12307	0	2.38	1	0.87154	.	.	1
12308	0	2.38	1	0.87154	.	.	1
12309	0	2.38	1	0.87154	.	.	1
12310	0	2.38	1	0.87154	.	.	1
12311	0	2.38	1	0.87154	.	.	1
12312	0	2.38	1	0.87154	.	.	1
12313	0	2.38	1	0.87154	.	.	1
12314	0	2.39	0	0.87121	0.86796	0.87438	1
12315	0	2.39	1	0.87121	.	.	1
12316	0	2.39	1	0.87121	.	.	1
12317	0	2.39	1	0.87121	.	.	1
12318	0	2.39	1	0.87121	.	.	1
12319	0	2.39	1	0.87121	.	.	1
12320	0	2.39	1	0.87121	.	.	1

Obs	Sub_group	fuptimey	_CENSOR_	SURVIVAL	SDF_LCL	SDF_UCL	STRATUM
12321	0	2.39	1	0.87121	.	.	1
12322	0	2.39	1	0.87121	.	.	1
12323	0	2.39	1	0.87121	.	.	1
12324	0	2.39	1	0.87121	.	.	1
12325	0	2.39	1	0.87121	.	.	1
12326	0	2.39	1	0.87121	.	.	1
12327	0	2.39	1	0.87121	.	.	1
12328	0	2.39	1	0.87121	.	.	1
12329	0	2.39	1	0.87121	.	.	1
12330	0	2.39	1	0.87121	.	.	1
12331	0	2.39	1	0.87121	.	.	1
12332	0	2.39	1	0.87121	.	.	1
12333	0	2.39	1	0.87121	.	.	1
12334	0	2.39	1	0.87121	.	.	1
12335	0	2.39	1	0.87121	.	.	1
12336	0	2.39	1	0.87121	.	.	1
12337	0	2.39	1	0.87121	.	.	1
12338	0	2.39	1	0.87121	.	.	1
12339	0	2.39	1	0.87121	.	.	1
12340	0	2.39	1	0.87121	.	.	1
12341	0	2.39	1	0.87121	.	.	1
12342	0	2.39	1	0.87121	.	.	1
12343	0	2.39	1	0.87121	.	.	1
12344	0	2.39	1	0.87121	.	.	1
12345	0	2.39	1	0.87121	.	.	1
12346	0	2.39	1	0.87121	.	.	1
12347	0	2.39	1	0.87121	.	.	1
12348	0	2.39	1	0.87121	.	.	1
12349	0	2.39	1	0.87121	.	.	1
12350	0	2.39	1	0.87121	.	.	1
12351	0	2.39	1	0.87121	.	.	1
12352	0	2.39	1	0.87121	.	.	1
12353	0	2.40	0	0.87088	0.86762	0.87406	1
12354	0	2.40	1	0.87088	.	.	1
12355	0	2.40	1	0.87088	.	.	1

Obs	Sub_group	fuptimey	_CENSOR_	SURVIVAL	SDF_LCL	SDF_UCL	STRATUM
12356	0	2.40	1	0.87088	.	.	1
12357	0	2.40	1	0.87088	.	.	1
12358	0	2.40	1	0.87088	.	.	1
12359	0	2.40	1	0.87088	.	.	1
12360	0	2.40	1	0.87088	.	.	1
12361	0	2.40	1	0.87088	.	.	1
12362	0	2.40	1	0.87088	.	.	1
12363	0	2.40	1	0.87088	.	.	1
12364	0	2.40	1	0.87088	.	.	1
12365	0	2.40	1	0.87088	.	.	1
12366	0	2.40	1	0.87088	.	.	1
12367	0	2.40	1	0.87088	.	.	1
12368	0	2.40	1	0.87088	.	.	1
12369	0	2.40	1	0.87088	.	.	1
12370	0	2.40	1	0.87088	.	.	1
12371	0	2.40	1	0.87088	.	.	1
12372	0	2.40	1	0.87088	.	.	1
12373	0	2.40	1	0.87088	.	.	1
12374	0	2.40	1	0.87088	.	.	1
12375	0	2.40	1	0.87088	.	.	1
12376	0	2.40	1	0.87088	.	.	1
12377	0	2.40	1	0.87088	.	.	1
12378	0	2.40	1	0.87088	.	.	1
12379	0	2.40	1	0.87088	.	.	1
12380	0	2.40	1	0.87088	.	.	1
12381	0	2.40	1	0.87088	.	.	1
12382	0	2.40	1	0.87088	.	.	1
12383	0	2.40	1	0.87088	.	.	1
12384	0	2.40	1	0.87088	.	.	1
12385	0	2.40	1	0.87088	.	.	1
12386	0	2.40	1	0.87088	.	.	1
12387	0	2.40	1	0.87088	.	.	1
12388	0	2.40	1	0.87088	.	.	1
12389	0	2.40	1	0.87088	.	.	1
12390	0	2.40	1	0.87088	.	.	1

Obs	Sub_group	fuptimey	_CENSOR_	SURVIVAL	SDF_LCL	SDF_UCL	STRATUM
12391	0	2.40	1	0.87088	.	.	1
12392	0	2.40	1	0.87088	.	.	1
12393	0	2.40	1	0.87088	.	.	1
12394	0	2.40	1	0.87088	.	.	1
12395	0	2.40	1	0.87088	.	.	1
12396	0	2.40	1	0.87088	.	.	1
12397	0	2.40	1	0.87088	.	.	1
12398	0	2.40	1	0.87088	.	.	1
12399	0	2.40	1	0.87088	.	.	1
12400	0	2.40	1	0.87088	.	.	1
12401	0	2.40	1	0.87088	.	.	1
12402	0	2.40	1	0.87088	.	.	1
12403	0	2.40	1	0.87088	.	.	1
12404	0	2.40	1	0.87088	.	.	1
12405	0	2.40	1	0.87088	.	.	1
12406	0	2.40	1	0.87088	.	.	1
12407	0	2.40	1	0.87088	.	.	1
12408	0	2.40	1	0.87088	.	.	1
12409	0	2.40	1	0.87088	.	.	1
12410	0	2.40	1	0.87088	.	.	1
12411	0	2.40	1	0.87088	.	.	1
12412	0	2.40	1	0.87088	.	.	1
12413	0	2.41	0	0.87055	0.86729	0.87373	1
12414	0	2.41	1	0.87055	.	.	1
12415	0	2.41	1	0.87055	.	.	1
12416	0	2.41	1	0.87055	.	.	1
12417	0	2.41	1	0.87055	.	.	1
12418	0	2.41	1	0.87055	.	.	1
12419	0	2.41	1	0.87055	.	.	1
12420	0	2.41	1	0.87055	.	.	1
12421	0	2.41	1	0.87055	.	.	1
12422	0	2.41	1	0.87055	.	.	1
12423	0	2.41	1	0.87055	.	.	1
12424	0	2.41	1	0.87055	.	.	1
12425	0	2.41	1	0.87055	.	.	1

Obs	Sub_group	fuptimey	_CENSOR_	SURVIVAL	SDF_LCL	SDF_UCL	STRATUM
12426	0	2.41	1	0.87055	.	.	1
12427	0	2.41	1	0.87055	.	.	1
12428	0	2.41	1	0.87055	.	.	1
12429	0	2.41	1	0.87055	.	.	1
12430	0	2.41	1	0.87055	.	.	1
12431	0	2.41	1	0.87055	.	.	1
12432	0	2.41	1	0.87055	.	.	1
12433	0	2.41	1	0.87055	.	.	1
12434	0	2.41	1	0.87055	.	.	1
12435	0	2.41	1	0.87055	.	.	1
12436	0	2.41	1	0.87055	.	.	1
12437	0	2.41	1	0.87055	.	.	1
12438	0	2.41	1	0.87055	.	.	1
12439	0	2.41	1	0.87055	.	.	1
12440	0	2.41	1	0.87055	.	.	1
12441	0	2.41	1	0.87055	.	.	1
12442	0	2.41	1	0.87055	.	.	1
12443	0	2.41	1	0.87055	.	.	1
12444	0	2.41	1	0.87055	.	.	1
12445	0	2.41	1	0.87055	.	.	1
12446	0	2.41	1	0.87055	.	.	1
12447	0	2.41	1	0.87055	.	.	1
12448	0	2.41	1	0.87055	.	.	1
12449	0	2.41	1	0.87055	.	.	1
12450	0	2.41	1	0.87055	.	.	1
12451	0	2.41	1	0.87055	.	.	1
12452	0	2.41	1	0.87055	.	.	1
12453	0	2.41	1	0.87055	.	.	1
12454	0	2.41	1	0.87055	.	.	1
12455	0	2.41	1	0.87055	.	.	1
12456	0	2.42	0	0.87025	0.86698	0.87344	1
12457	0	2.42	1	0.87025	.	.	1
12458	0	2.42	1	0.87025	.	.	1
12459	0	2.42	1	0.87025	.	.	1
12460	0	2.42	1	0.87025	.	.	1

Obs	Sub_group	fuptimey	_CENSOR_	SURVIVAL	SDF_LCL	SDF_UCL	STRATUM
12461	0	2.42	1	0.87025	.	.	1
12462	0	2.42	1	0.87025	.	.	1
12463	0	2.42	1	0.87025	.	.	1
12464	0	2.42	1	0.87025	.	.	1
12465	0	2.42	1	0.87025	.	.	1
12466	0	2.42	1	0.87025	.	.	1
12467	0	2.42	1	0.87025	.	.	1
12468	0	2.42	1	0.87025	.	.	1
12469	0	2.42	1	0.87025	.	.	1
12470	0	2.42	1	0.87025	.	.	1
12471	0	2.42	1	0.87025	.	.	1
12472	0	2.42	1	0.87025	.	.	1
12473	0	2.42	1	0.87025	.	.	1
12474	0	2.42	1	0.87025	.	.	1
12475	0	2.42	1	0.87025	.	.	1
12476	0	2.42	1	0.87025	.	.	1
12477	0	2.42	1	0.87025	.	.	1
12478	0	2.42	1	0.87025	.	.	1
12479	0	2.42	1	0.87025	.	.	1
12480	0	2.42	1	0.87025	.	.	1
12481	0	2.42	1	0.87025	.	.	1
12482	0	2.42	1	0.87025	.	.	1
12483	0	2.42	1	0.87025	.	.	1
12484	0	2.42	1	0.87025	.	.	1
12485	0	2.42	1	0.87025	.	.	1
12486	0	2.42	1	0.87025	.	.	1
12487	0	2.42	1	0.87025	.	.	1
12488	0	2.42	1	0.87025	.	.	1
12489	0	2.42	1	0.87025	.	.	1
12490	0	2.42	1	0.87025	.	.	1
12491	0	2.42	1	0.87025	.	.	1
12492	0	2.42	1	0.87025	.	.	1
12493	0	2.42	1	0.87025	.	.	1
12494	0	2.42	1	0.87025	.	.	1
12495	0	2.42	1	0.87025	.	.	1

Obs	Sub_group	fuptimey	_CENSOR_	SURVIVAL	SDF_LCL	SDF_UCL	STRATUM
12496	0	2.42	1	0.87025	.	.	1
12497	0	2.43	0	0.87010	0.86683	0.87329	1
12498	0	2.43	1	0.87010	.	.	1
12499	0	2.43	1	0.87010	.	.	1
12500	0	2.43	1	0.87010	.	.	1
12501	0	2.43	1	0.87010	.	.	1
12502	0	2.43	1	0.87010	.	.	1
12503	0	2.43	1	0.87010	.	.	1
12504	0	2.43	1	0.87010	.	.	1
12505	0	2.43	1	0.87010	.	.	1
12506	0	2.43	1	0.87010	.	.	1
12507	0	2.43	1	0.87010	.	.	1
12508	0	2.43	1	0.87010	.	.	1
12509	0	2.43	1	0.87010	.	.	1
12510	0	2.43	1	0.87010	.	.	1
12511	0	2.43	1	0.87010	.	.	1
12512	0	2.43	1	0.87010	.	.	1
12513	0	2.43	1	0.87010	.	.	1
12514	0	2.43	1	0.87010	.	.	1
12515	0	2.43	1	0.87010	.	.	1
12516	0	2.43	1	0.87010	.	.	1
12517	0	2.43	1	0.87010	.	.	1
12518	0	2.43	1	0.87010	.	.	1
12519	0	2.43	1	0.87010	.	.	1
12520	0	2.43	1	0.87010	.	.	1
12521	0	2.43	1	0.87010	.	.	1
12522	0	2.43	1	0.87010	.	.	1
12523	0	2.43	1	0.87010	.	.	1
12524	0	2.43	1	0.87010	.	.	1
12525	0	2.43	1	0.87010	.	.	1
12526	0	2.43	1	0.87010	.	.	1
12527	0	2.43	1	0.87010	.	.	1
12528	0	2.43	1	0.87010	.	.	1
12529	0	2.43	1	0.87010	.	.	1
12530	0	2.43	1	0.87010	.	.	1

Obs	Sub_group	fuptimey	_CENSOR_	SURVIVAL	SDF_LCL	SDF_UCL	STRATUM
12531	0	2.43	1	0.87010	.	.	1
12532	0	2.43	1	0.87010	.	.	1
12533	0	2.43	1	0.87010	.	.	1
12534	0	2.43	1	0.87010	.	.	1
12535	0	2.44	0	0.87004	0.86677	0.87323	1
12536	0	2.44	1	0.87004	.	.	1
12537	0	2.44	1	0.87004	.	.	1
12538	0	2.44	1	0.87004	.	.	1
12539	0	2.44	1	0.87004	.	.	1
12540	0	2.44	1	0.87004	.	.	1
12541	0	2.44	1	0.87004	.	.	1
12542	0	2.44	1	0.87004	.	.	1
12543	0	2.44	1	0.87004	.	.	1
12544	0	2.44	1	0.87004	.	.	1
12545	0	2.44	1	0.87004	.	.	1
12546	0	2.44	1	0.87004	.	.	1
12547	0	2.44	1	0.87004	.	.	1
12548	0	2.44	1	0.87004	.	.	1
12549	0	2.44	1	0.87004	.	.	1
12550	0	2.44	1	0.87004	.	.	1
12551	0	2.44	1	0.87004	.	.	1
12552	0	2.44	1	0.87004	.	.	1
12553	0	2.44	1	0.87004	.	.	1
12554	0	2.44	1	0.87004	.	.	1
12555	0	2.44	1	0.87004	.	.	1
12556	0	2.44	1	0.87004	.	.	1
12557	0	2.44	1	0.87004	.	.	1
12558	0	2.44	1	0.87004	.	.	1
12559	0	2.44	1	0.87004	.	.	1
12560	0	2.44	1	0.87004	.	.	1
12561	0	2.44	1	0.87004	.	.	1
12562	0	2.44	1	0.87004	.	.	1
12563	0	2.44	1	0.87004	.	.	1
12564	0	2.44	1	0.87004	.	.	1
12565	0	2.44	1	0.87004	.	.	1

Obs	Sub_group	fuptimey	_CENSOR_	SURVIVAL	SDF_LCL	SDF_UCL	STRATUM
12566	0	2.44	1	0.87004	.	.	1
12567	0	2.44	1	0.87004	.	.	1
12568	0	2.44	1	0.87004	.	.	1
12569	0	2.44	1	0.87004	.	.	1
12570	0	2.44	1	0.87004	.	.	1
12571	0	2.44	1	0.87004	.	.	1
12572	0	2.44	1	0.87004	.	.	1
12573	0	2.44	1	0.87004	.	.	1
12574	0	2.44	1	0.87004	.	.	1
12575	0	2.44	1	0.87004	.	.	1
12576	0	2.44	1	0.87004	.	.	1
12577	0	2.44	1	0.87004	.	.	1
12578	0	2.44	1	0.87004	.	.	1
12579	0	2.44	1	0.87004	.	.	1
12580	0	2.44	1	0.87004	.	.	1
12581	0	2.44	1	0.87004	.	.	1
12582	0	2.44	1	0.87004	.	.	1
12583	0	2.44	1	0.87004	.	.	1
12584	0	2.44	1	0.87004	.	.	1
12585	0	2.44	1	0.87004	.	.	1
12586	0	2.44	1	0.87004	.	.	1
12587	0	2.44	1	0.87004	.	.	1
12588	0	2.44	1	0.87004	.	.	1
12589	0	2.44	1	0.87004	.	.	1
12590	0	2.44	1	0.87004	.	.	1
12591	0	2.44	1	0.87004	.	.	1
12592	0	2.44	1	0.87004	.	.	1
12593	0	2.44	1	0.87004	.	.	1
12594	0	2.44	1	0.87004	.	.	1
12595	0	2.44	1	0.87004	.	.	1
12596	0	2.44	1	0.87004	.	.	1
12597	0	2.44	1	0.87004	.	.	1
12598	0	2.45	0	0.86992	0.86665	0.87311	1
12599	0	2.45	1	0.86992	.	.	1
12600	0	2.45	1	0.86992	.	.	1

Obs	Sub_group	fuptimey	_CENSOR_	SURVIVAL	SDF_LCL	SDF_UCL	STRATUM
12601	0	2.45	1	0.86992	.	.	1
12602	0	2.45	1	0.86992	.	.	1
12603	0	2.45	1	0.86992	.	.	1
12604	0	2.45	1	0.86992	.	.	1
12605	0	2.45	1	0.86992	.	.	1
12606	0	2.45	1	0.86992	.	.	1
12607	0	2.45	1	0.86992	.	.	1
12608	0	2.45	1	0.86992	.	.	1
12609	0	2.45	1	0.86992	.	.	1
12610	0	2.45	1	0.86992	.	.	1
12611	0	2.45	1	0.86992	.	.	1
12612	0	2.45	1	0.86992	.	.	1
12613	0	2.45	1	0.86992	.	.	1
12614	0	2.45	1	0.86992	.	.	1
12615	0	2.45	1	0.86992	.	.	1
12616	0	2.45	1	0.86992	.	.	1
12617	0	2.45	1	0.86992	.	.	1
12618	0	2.45	1	0.86992	.	.	1
12619	0	2.45	1	0.86992	.	.	1
12620	0	2.45	1	0.86992	.	.	1
12621	0	2.45	1	0.86992	.	.	1
12622	0	2.45	1	0.86992	.	.	1
12623	0	2.45	1	0.86992	.	.	1
12624	0	2.45	1	0.86992	.	.	1
12625	0	2.45	1	0.86992	.	.	1
12626	0	2.45	1	0.86992	.	.	1
12627	0	2.45	1	0.86992	.	.	1
12628	0	2.45	1	0.86992	.	.	1
12629	0	2.45	1	0.86992	.	.	1
12630	0	2.45	1	0.86992	.	.	1
12631	0	2.45	1	0.86992	.	.	1
12632	0	2.45	1	0.86992	.	.	1
12633	0	2.45	1	0.86992	.	.	1
12634	0	2.45	1	0.86992	.	.	1
12635	0	2.45	1	0.86992	.	.	1

Obs	Sub_group	fuptimey	_CENSOR_	SURVIVAL	SDF_LCL	SDF_UCL	STRATUM
12636	0	2.45	1	0.86992	.	.	1
12637	0	2.45	1	0.86992	.	.	1
12638	0	2.45	1	0.86992	.	.	1
12639	0	2.45	1	0.86992	.	.	1
12640	0	2.45	1	0.86992	.	.	1
12641	0	2.45	1	0.86992	.	.	1
12642	0	2.45	1	0.86992	.	.	1
12643	0	2.45	1	0.86992	.	.	1
12644	0	2.45	1	0.86992	.	.	1
12645	0	2.45	1	0.86992	.	.	1
12646	0	2.46	0	0.86961	0.86634	0.87281	1
12647	0	2.46	1	0.86961	.	.	1
12648	0	2.46	1	0.86961	.	.	1
12649	0	2.46	1	0.86961	.	.	1
12650	0	2.46	1	0.86961	.	.	1
12651	0	2.46	1	0.86961	.	.	1
12652	0	2.46	1	0.86961	.	.	1
12653	0	2.46	1	0.86961	.	.	1
12654	0	2.46	1	0.86961	.	.	1
12655	0	2.46	1	0.86961	.	.	1
12656	0	2.46	1	0.86961	.	.	1
12657	0	2.46	1	0.86961	.	.	1
12658	0	2.46	1	0.86961	.	.	1
12659	0	2.46	1	0.86961	.	.	1
12660	0	2.46	1	0.86961	.	.	1
12661	0	2.46	1	0.86961	.	.	1
12662	0	2.46	1	0.86961	.	.	1
12663	0	2.46	1	0.86961	.	.	1
12664	0	2.46	1	0.86961	.	.	1
12665	0	2.46	1	0.86961	.	.	1
12666	0	2.46	1	0.86961	.	.	1
12667	0	2.46	1	0.86961	.	.	1
12668	0	2.46	1	0.86961	.	.	1
12669	0	2.46	1	0.86961	.	.	1
12670	0	2.46	1	0.86961	.	.	1

Obs	Sub_group	fuptimey	_CENSOR_	SURVIVAL	SDF_LCL	SDF_UCL	STRATUM
12671	0	2.46	1	0.86961	.	.	1
12672	0	2.46	1	0.86961	.	.	1
12673	0	2.46	1	0.86961	.	.	1
12674	0	2.46	1	0.86961	.	.	1
12675	0	2.46	1	0.86961	.	.	1
12676	0	2.46	1	0.86961	.	.	1
12677	0	2.46	1	0.86961	.	.	1
12678	0	2.46	1	0.86961	.	.	1
12679	0	2.46	1	0.86961	.	.	1
12680	0	2.46	1	0.86961	.	.	1
12681	0	2.46	1	0.86961	.	.	1
12682	0	2.46	1	0.86961	.	.	1
12683	0	2.46	1	0.86961	.	.	1
12684	0	2.46	1	0.86961	.	.	1
12685	0	2.46	1	0.86961	.	.	1
12686	0	2.46	1	0.86961	.	.	1
12687	0	2.46	1	0.86961	.	.	1
12688	0	2.46	1	0.86961	.	.	1
12689	0	2.46	1	0.86961	.	.	1
12690	0	2.46	1	0.86961	.	.	1
12691	0	2.46	1	0.86961	.	.	1
12692	0	2.46	1	0.86961	.	.	1
12693	0	2.46	1	0.86961	.	.	1
12694	0	2.46	1	0.86961	.	.	1
12695	0	2.46	1	0.86961	.	.	1
12696	0	2.46	1	0.86961	.	.	1
12697	0	2.47	0	0.86946	0.86619	0.87266	1
12698	0	2.47	1	0.86946	.	.	1
12699	0	2.47	1	0.86946	.	.	1
12700	0	2.47	1	0.86946	.	.	1
12701	0	2.47	1	0.86946	.	.	1
12702	0	2.47	1	0.86946	.	.	1
12703	0	2.47	1	0.86946	.	.	1
12704	0	2.47	1	0.86946	.	.	1
12705	0	2.47	1	0.86946	.	.	1

Obs	Sub_group	fuptimey	_CENSOR_	SURVIVAL	SDF_LCL	SDF_UCL	STRATUM
12706	0	2.47	1	0.86946	.	.	1
12707	0	2.47	1	0.86946	.	.	1
12708	0	2.47	1	0.86946	.	.	1
12709	0	2.47	1	0.86946	.	.	1
12710	0	2.47	1	0.86946	.	.	1
12711	0	2.47	1	0.86946	.	.	1
12712	0	2.47	1	0.86946	.	.	1
12713	0	2.47	1	0.86946	.	.	1
12714	0	2.47	1	0.86946	.	.	1
12715	0	2.47	1	0.86946	.	.	1
12716	0	2.47	1	0.86946	.	.	1
12717	0	2.47	1	0.86946	.	.	1
12718	0	2.47	1	0.86946	.	.	1
12719	0	2.47	1	0.86946	.	.	1
12720	0	2.47	1	0.86946	.	.	1
12721	0	2.47	1	0.86946	.	.	1
12722	0	2.47	1	0.86946	.	.	1
12723	0	2.47	1	0.86946	.	.	1
12724	0	2.47	1	0.86946	.	.	1
12725	0	2.48	0	0.86928	0.86600	0.87248	1
12726	0	2.48	1	0.86928	.	.	1
12727	0	2.48	1	0.86928	.	.	1
12728	0	2.48	1	0.86928	.	.	1
12729	0	2.48	1	0.86928	.	.	1
12730	0	2.48	1	0.86928	.	.	1
12731	0	2.48	1	0.86928	.	.	1
12732	0	2.48	1	0.86928	.	.	1
12733	0	2.48	1	0.86928	.	.	1
12734	0	2.48	1	0.86928	.	.	1
12735	0	2.48	1	0.86928	.	.	1
12736	0	2.48	1	0.86928	.	.	1
12737	0	2.48	1	0.86928	.	.	1
12738	0	2.48	1	0.86928	.	.	1
12739	0	2.48	1	0.86928	.	.	1
12740	0	2.48	1	0.86928	.	.	1

Obs	Sub_group	fuptimey	_CENSOR_	SURVIVAL	SDF_LCL	SDF_UCL	STRATUM
12741	0	2.48	1	0.86928	.	.	1
12742	0	2.48	1	0.86928	.	.	1
12743	0	2.48	1	0.86928	.	.	1
12744	0	2.48	1	0.86928	.	.	1
12745	0	2.48	1	0.86928	.	.	1
12746	0	2.48	1	0.86928	.	.	1
12747	0	2.48	1	0.86928	.	.	1
12748	0	2.48	1	0.86928	.	.	1
12749	0	2.48	1	0.86928	.	.	1
12750	0	2.48	1	0.86928	.	.	1
12751	0	2.48	1	0.86928	.	.	1
12752	0	2.48	1	0.86928	.	.	1
12753	0	2.48	1	0.86928	.	.	1
12754	0	2.48	1	0.86928	.	.	1
12755	0	2.48	1	0.86928	.	.	1
12756	0	2.48	1	0.86928	.	.	1
12757	0	2.48	1	0.86928	.	.	1
12758	0	2.48	1	0.86928	.	.	1
12759	0	2.48	1	0.86928	.	.	1
12760	0	2.48	1	0.86928	.	.	1
12761	0	2.48	1	0.86928	.	.	1
12762	0	2.48	1	0.86928	.	.	1
12763	0	2.48	1	0.86928	.	.	1
12764	0	2.48	1	0.86928	.	.	1
12765	0	2.48	1	0.86928	.	.	1
12766	0	2.48	1	0.86928	.	.	1
12767	0	2.48	1	0.86928	.	.	1
12768	0	2.48	1	0.86928	.	.	1
12769	0	2.48	1	0.86928	.	.	1
12770	0	2.48	1	0.86928	.	.	1
12771	0	2.48	1	0.86928	.	.	1
12772	0	2.48	1	0.86928	.	.	1
12773	0	2.48	1	0.86928	.	.	1
12774	0	2.48	1	0.86928	.	.	1
12775	0	2.48	1	0.86928	.	.	1

Obs	Sub_group	fuptimey	_CENSOR_	SURVIVAL	SDF_LCL	SDF_UCL	STRATUM
12776	0	2.48	1	0.86928	.	.	1
12777	0	2.48	1	0.86928	.	.	1
12778	0	2.48	1	0.86928	.	.	1
12779	0	2.48	1	0.86928	.	.	1
12780	0	2.48	1	0.86928	.	.	1
12781	0	2.48	1	0.86928	.	.	1
12782	0	2.48	1	0.86928	.	.	1
12783	0	2.48	1	0.86928	.	.	1
12784	0	2.49	0	0.86901	0.86572	0.87221	1
12785	0	2.49	1	0.86901	.	.	1
12786	0	2.49	1	0.86901	.	.	1
12787	0	2.49	1	0.86901	.	.	1
12788	0	2.49	1	0.86901	.	.	1
12789	0	2.49	1	0.86901	.	.	1
12790	0	2.49	1	0.86901	.	.	1
12791	0	2.49	1	0.86901	.	.	1
12792	0	2.49	1	0.86901	.	.	1
12793	0	2.49	1	0.86901	.	.	1
12794	0	2.49	1	0.86901	.	.	1
12795	0	2.49	1	0.86901	.	.	1
12796	0	2.49	1	0.86901	.	.	1
12797	0	2.49	1	0.86901	.	.	1
12798	0	2.49	1	0.86901	.	.	1
12799	0	2.49	1	0.86901	.	.	1
12800	0	2.49	1	0.86901	.	.	1
12801	0	2.49	1	0.86901	.	.	1
12802	0	2.49	1	0.86901	.	.	1
12803	0	2.49	1	0.86901	.	.	1
12804	0	2.49	1	0.86901	.	.	1
12805	0	2.49	1	0.86901	.	.	1
12806	0	2.49	1	0.86901	.	.	1
12807	0	2.49	1	0.86901	.	.	1
12808	0	2.49	1	0.86901	.	.	1
12809	0	2.49	1	0.86901	.	.	1
12810	0	2.49	1	0.86901	.	.	1

Obs	Sub_group	fuptimey	_CENSOR_	SURVIVAL	SDF_LCL	SDF_UCL	STRATUM
**12811**	0	2.49	1	0.86901	.	.	1
**12812**	0	2.49	1	0.86901	.	.	1
**12813**	0	2.49	1	0.86901	.	.	1
**12814**	0	2.49	1	0.86901	.	.	1
**12815**	0	2.49	1	0.86901	.	.	1
**12816**	0	2.49	1	0.86901	.	.	1
**12817**	0	2.49	1	0.86901	.	.	1
**12818**	0	2.49	1	0.86901	.	.	1
**12819**	0	2.49	1	0.86901	.	.	1
**12820**	0	2.49	1	0.86901	.	.	1
**12821**	0	2.49	1	0.86901	.	.	1
**12822**	0	2.49	1	0.86901	.	.	1
**12823**	0	2.49	1	0.86901	.	.	1
**12824**	0	2.49	1	0.86901	.	.	1
**12825**	0	2.50	0	0.86864	0.86535	0.87185	1
**12826**	0	2.50	1	0.86864	.	.	1
**12827**	0	2.50	1	0.86864	.	.	1
**12828**	0	2.50	1	0.86864	.	.	1
**12829**	0	2.50	1	0.86864	.	.	1
**12830**	0	2.50	1	0.86864	.	.	1
**12831**	0	2.50	1	0.86864	.	.	1
**12832**	0	2.50	1	0.86864	.	.	1
**12833**	0	2.50	1	0.86864	.	.	1
**12834**	0	2.50	1	0.86864	.	.	1
**12835**	0	2.50	1	0.86864	.	.	1
**12836**	0	2.50	1	0.86864	.	.	1
**12837**	0	2.50	1	0.86864	.	.	1
**12838**	0	2.50	1	0.86864	.	.	1
**12839**	0	2.50	1	0.86864	.	.	1
**12840**	0	2.50	1	0.86864	.	.	1
**12841**	0	2.50	1	0.86864	.	.	1
**12842**	0	2.50	1	0.86864	.	.	1
**12843**	0	2.50	1	0.86864	.	.	1
**12844**	0	2.50	1	0.86864	.	.	1
**12845**	0	2.50	1	0.86864	.	.	1

Obs	Sub_group	fuptimey	_CENSOR_	SURVIVAL	SDF_LCL	SDF_UCL	STRATUM
12846	0	2.50	1	0.86864	.	.	1
12847	0	2.50	1	0.86864	.	.	1
12848	0	2.50	1	0.86864	.	.	1
12849	0	2.50	1	0.86864	.	.	1
12850	0	2.50	1	0.86864	.	.	1
12851	0	2.50	1	0.86864	.	.	1
12852	0	2.50	1	0.86864	.	.	1
12853	0	2.50	1	0.86864	.	.	1
12854	0	2.50	1	0.86864	.	.	1
12855	0	2.50	1	0.86864	.	.	1
12856	0	2.50	1	0.86864	.	.	1
12857	0	2.50	1	0.86864	.	.	1
12858	0	2.50	1	0.86864	.	.	1
12859	0	2.50	1	0.86864	.	.	1
12860	0	2.50	1	0.86864	.	.	1
12861	0	2.50	1	0.86864	.	.	1
12862	0	2.50	1	0.86864	.	.	1
12863	0	2.50	1	0.86864	.	.	1
12864	0	2.50	1	0.86864	.	.	1
12865	0	2.50	1	0.86864	.	.	1
12866	0	2.50	1	0.86864	.	.	1
12867	0	2.51	0	0.86846	0.86517	0.87167	1
12868	0	2.51	1	0.86846	.	.	1
12869	0	2.51	1	0.86846	.	.	1
12870	0	2.51	1	0.86846	.	.	1
12871	0	2.51	1	0.86846	.	.	1
12872	0	2.51	1	0.86846	.	.	1
12873	0	2.51	1	0.86846	.	.	1
12874	0	2.51	1	0.86846	.	.	1
12875	0	2.51	1	0.86846	.	.	1
12876	0	2.51	1	0.86846	.	.	1
12877	0	2.51	1	0.86846	.	.	1
12878	0	2.51	1	0.86846	.	.	1
12879	0	2.51	1	0.86846	.	.	1
12880	0	2.51	1	0.86846	.	.	1

Obs	Sub_group	fuptimey	_CENSOR_	SURVIVAL	SDF_LCL	SDF_UCL	STRATUM
12881	0	2.51	1	0.86846	.	.	1
12882	0	2.51	1	0.86846	.	.	1
12883	0	2.51	1	0.86846	.	.	1
12884	0	2.51	1	0.86846	.	.	1
12885	0	2.51	1	0.86846	.	.	1
12886	0	2.51	1	0.86846	.	.	1
12887	0	2.51	1	0.86846	.	.	1
12888	0	2.51	1	0.86846	.	.	1
12889	0	2.51	1	0.86846	.	.	1
12890	0	2.51	1	0.86846	.	.	1
12891	0	2.51	1	0.86846	.	.	1
12892	0	2.51	1	0.86846	.	.	1
12893	0	2.51	1	0.86846	.	.	1
12894	0	2.51	1	0.86846	.	.	1
12895	0	2.51	1	0.86846	.	.	1
12896	0	2.51	1	0.86846	.	.	1
12897	0	2.51	1	0.86846	.	.	1
12898	0	2.51	1	0.86846	.	.	1
12899	0	2.51	1	0.86846	.	.	1
12900	0	2.51	1	0.86846	.	.	1
12901	0	2.51	1	0.86846	.	.	1
12902	0	2.51	1	0.86846	.	.	1
12903	0	2.51	1	0.86846	.	.	1
12904	0	2.51	1	0.86846	.	.	1
12905	0	2.51	1	0.86846	.	.	1
12906	0	2.51	1	0.86846	.	.	1
12907	0	2.51	1	0.86846	.	.	1
12908	0	2.51	1	0.86846	.	.	1
12909	0	2.51	1	0.86846	.	.	1
12910	0	2.51	1	0.86846	.	.	1
12911	0	2.51	1	0.86846	.	.	1
12912	0	2.51	1	0.86846	.	.	1
12913	0	2.51	1	0.86846	.	.	1
12914	0	2.51	1	0.86846	.	.	1
12915	0	2.51	1	0.86846	.	.	1

Obs	Sub_group	fuptimey	_CENSOR_	SURVIVAL	SDF_LCL	SDF_UCL	STRATUM
12916	0	2.52	0	0.86812	0.86483	0.87134	1
12917	0	2.52	1	0.86812	.	.	1
12918	0	2.52	1	0.86812	.	.	1
12919	0	2.52	1	0.86812	.	.	1
12920	0	2.52	1	0.86812	.	.	1
12921	0	2.52	1	0.86812	.	.	1
12922	0	2.52	1	0.86812	.	.	1
12923	0	2.52	1	0.86812	.	.	1
12924	0	2.52	1	0.86812	.	.	1
12925	0	2.52	1	0.86812	.	.	1
12926	0	2.52	1	0.86812	.	.	1
12927	0	2.52	1	0.86812	.	.	1
12928	0	2.52	1	0.86812	.	.	1
12929	0	2.52	1	0.86812	.	.	1
12930	0	2.52	1	0.86812	.	.	1
12931	0	2.52	1	0.86812	.	.	1
12932	0	2.52	1	0.86812	.	.	1
12933	0	2.52	1	0.86812	.	.	1
12934	0	2.52	1	0.86812	.	.	1
12935	0	2.52	1	0.86812	.	.	1
12936	0	2.52	1	0.86812	.	.	1
12937	0	2.52	1	0.86812	.	.	1
12938	0	2.52	1	0.86812	.	.	1
12939	0	2.52	1	0.86812	.	.	1
12940	0	2.52	1	0.86812	.	.	1
12941	0	2.52	1	0.86812	.	.	1
12942	0	2.52	1	0.86812	.	.	1
12943	0	2.52	1	0.86812	.	.	1
12944	0	2.52	1	0.86812	.	.	1
12945	0	2.52	1	0.86812	.	.	1
12946	0	2.52	1	0.86812	.	.	1
12947	0	2.52	1	0.86812	.	.	1
12948	0	2.52	1	0.86812	.	.	1
12949	0	2.52	1	0.86812	.	.	1
12950	0	2.52	1	0.86812	.	.	1

Obs	Sub_group	fuptimey	_CENSOR_	SURVIVAL	SDF_LCL	SDF_UCL	STRATUM
12951	0	2.52	1	0.86812	.	.	1
12952	0	2.52	1	0.86812	.	.	1
12953	0	2.52	1	0.86812	.	.	1
12954	0	2.52	1	0.86812	.	.	1
12955	0	2.52	1	0.86812	.	.	1
12956	0	2.52	1	0.86812	.	.	1
12957	0	2.52	1	0.86812	.	.	1
12958	0	2.52	1	0.86812	.	.	1
12959	0	2.52	1	0.86812	.	.	1
12960	0	2.52	1	0.86812	.	.	1
12961	0	2.52	1	0.86812	.	.	1
12962	0	2.52	1	0.86812	.	.	1
12963	0	2.52	1	0.86812	.	.	1
12964	0	2.52	1	0.86812	.	.	1
12965	0	2.52	1	0.86812	.	.	1
12966	0	2.52	1	0.86812	.	.	1
12967	0	2.52	1	0.86812	.	.	1
12968	0	2.52	1	0.86812	.	.	1
12969	0	2.52	1	0.86812	.	.	1
12970	0	2.52	1	0.86812	.	.	1
12971	0	2.52	1	0.86812	.	.	1
12972	0	2.52	1	0.86812	.	.	1
12973	0	2.53	0	0.86778	0.86449	0.87101	1
12974	0	2.53	1	0.86778	.	.	1
12975	0	2.53	1	0.86778	.	.	1
12976	0	2.53	1	0.86778	.	.	1
12977	0	2.53	1	0.86778	.	.	1
12978	0	2.53	1	0.86778	.	.	1
12979	0	2.53	1	0.86778	.	.	1
12980	0	2.53	1	0.86778	.	.	1
12981	0	2.53	1	0.86778	.	.	1
12982	0	2.53	1	0.86778	.	.	1
12983	0	2.53	1	0.86778	.	.	1
12984	0	2.53	1	0.86778	.	.	1
12985	0	2.53	1	0.86778	.	.	1

Obs	Sub_group	fuptimey	_CENSOR_	SURVIVAL	SDF_LCL	SDF_UCL	STRATUM
12986	0	2.53	1	0.86778	.	.	1
12987	0	2.53	1	0.86778	.	.	1
12988	0	2.53	1	0.86778	.	.	1
12989	0	2.53	1	0.86778	.	.	1
12990	0	2.53	1	0.86778	.	.	1
12991	0	2.53	1	0.86778	.	.	1
12992	0	2.53	1	0.86778	.	.	1
12993	0	2.53	1	0.86778	.	.	1
12994	0	2.53	1	0.86778	.	.	1
12995	0	2.53	1	0.86778	.	.	1
12996	0	2.53	1	0.86778	.	.	1
12997	0	2.53	1	0.86778	.	.	1
12998	0	2.53	1	0.86778	.	.	1
12999	0	2.53	1	0.86778	.	.	1
13000	0	2.53	1	0.86778	.	.	1
13001	0	2.53	1	0.86778	.	.	1
13002	0	2.53	1	0.86778	.	.	1
13003	0	2.53	1	0.86778	.	.	1
13004	0	2.53	1	0.86778	.	.	1
13005	0	2.53	1	0.86778	.	.	1
13006	0	2.53	1	0.86778	.	.	1
13007	0	2.53	1	0.86778	.	.	1
13008	0	2.53	1	0.86778	.	.	1
13009	0	2.54	0	0.86754	0.86424	0.87077	1
13010	0	2.54	1	0.86754	.	.	1
13011	0	2.54	1	0.86754	.	.	1
13012	0	2.54	1	0.86754	.	.	1
13013	0	2.54	1	0.86754	.	.	1
13014	0	2.54	1	0.86754	.	.	1
13015	0	2.54	1	0.86754	.	.	1
13016	0	2.54	1	0.86754	.	.	1
13017	0	2.54	1	0.86754	.	.	1
13018	0	2.54	1	0.86754	.	.	1
13019	0	2.54	1	0.86754	.	.	1
13020	0	2.54	1	0.86754	.	.	1

Obs	Sub_group	fuptimey	_CENSOR_	SURVIVAL	SDF_LCL	SDF_UCL	STRATUM
13021	0	2.54	1	0.86754	.	.	1
13022	0	2.54	1	0.86754	.	.	1
13023	0	2.54	1	0.86754	.	.	1
13024	0	2.54	1	0.86754	.	.	1
13025	0	2.54	1	0.86754	.	.	1
13026	0	2.54	1	0.86754	.	.	1
13027	0	2.54	1	0.86754	.	.	1
13028	0	2.54	1	0.86754	.	.	1
13029	0	2.54	1	0.86754	.	.	1
13030	0	2.54	1	0.86754	.	.	1
13031	0	2.54	1	0.86754	.	.	1
13032	0	2.54	1	0.86754	.	.	1
13033	0	2.54	1	0.86754	.	.	1
13034	0	2.54	1	0.86754	.	.	1
13035	0	2.54	1	0.86754	.	.	1
13036	0	2.54	1	0.86754	.	.	1
13037	0	2.54	1	0.86754	.	.	1
13038	0	2.54	1	0.86754	.	.	1
13039	0	2.54	1	0.86754	.	.	1
13040	0	2.54	1	0.86754	.	.	1
13041	0	2.54	1	0.86754	.	.	1
13042	0	2.54	1	0.86754	.	.	1
13043	0	2.54	1	0.86754	.	.	1
13044	0	2.54	1	0.86754	.	.	1
13045	0	2.54	1	0.86754	.	.	1
13046	0	2.54	1	0.86754	.	.	1
13047	0	2.54	1	0.86754	.	.	1
13048	0	2.54	1	0.86754	.	.	1
13049	0	2.54	1	0.86754	.	.	1
13050	0	2.54	1	0.86754	.	.	1
13051	0	2.54	1	0.86754	.	.	1
13052	0	2.54	1	0.86754	.	.	1
13053	0	2.54	1	0.86754	.	.	1
13054	0	2.54	1	0.86754	.	.	1
13055	0	2.54	1	0.86754	.	.	1

Obs	Sub_group	fuptimey	_CENSOR_	SURVIVAL	SDF_LCL	SDF_UCL	STRATUM
13056	0	2.54	1	0.86754	.	.	1
13057	0	2.54	1	0.86754	.	.	1
13058	0	2.55	0	0.86745	0.86414	0.87068	1
13059	0	2.55	1	0.86745	.	.	1
13060	0	2.55	1	0.86745	.	.	1
13061	0	2.55	1	0.86745	.	.	1
13062	0	2.55	1	0.86745	.	.	1
13063	0	2.55	1	0.86745	.	.	1
13064	0	2.55	1	0.86745	.	.	1
13065	0	2.55	1	0.86745	.	.	1
13066	0	2.55	1	0.86745	.	.	1
13067	0	2.55	1	0.86745	.	.	1
13068	0	2.55	1	0.86745	.	.	1
13069	0	2.55	1	0.86745	.	.	1
13070	0	2.55	1	0.86745	.	.	1
13071	0	2.55	1	0.86745	.	.	1
13072	0	2.55	1	0.86745	.	.	1
13073	0	2.55	1	0.86745	.	.	1
13074	0	2.55	1	0.86745	.	.	1
13075	0	2.55	1	0.86745	.	.	1
13076	0	2.55	1	0.86745	.	.	1
13077	0	2.55	1	0.86745	.	.	1
13078	0	2.55	1	0.86745	.	.	1
13079	0	2.55	1	0.86745	.	.	1
13080	0	2.55	1	0.86745	.	.	1
13081	0	2.55	1	0.86745	.	.	1
13082	0	2.55	1	0.86745	.	.	1
13083	0	2.55	1	0.86745	.	.	1
13084	0	2.55	1	0.86745	.	.	1
13085	0	2.55	1	0.86745	.	.	1
13086	0	2.55	1	0.86745	.	.	1
13087	0	2.55	1	0.86745	.	.	1
13088	0	2.55	1	0.86745	.	.	1
13089	0	2.55	1	0.86745	.	.	1
13090	0	2.55	1	0.86745	.	.	1

Obs	Sub_group	fuptimey	_CENSOR_	SURVIVAL	SDF_LCL	SDF_UCL	STRATUM
13091	0	2.55	1	0.86745	.	.	1
13092	0	2.55	1	0.86745	.	.	1
13093	0	2.55	1	0.86745	.	.	1
13094	0	2.55	1	0.86745	.	.	1
13095	0	2.55	1	0.86745	.	.	1
13096	0	2.55	1	0.86745	.	.	1
13097	0	2.55	1	0.86745	.	.	1
13098	0	2.55	1	0.86745	.	.	1
13099	0	2.55	1	0.86745	.	.	1
13100	0	2.56	0	0.86729	0.86399	0.87052	1
13101	0	2.56	1	0.86729	.	.	1
13102	0	2.56	1	0.86729	.	.	1
13103	0	2.56	1	0.86729	.	.	1
13104	0	2.56	1	0.86729	.	.	1
13105	0	2.56	1	0.86729	.	.	1
13106	0	2.56	1	0.86729	.	.	1
13107	0	2.56	1	0.86729	.	.	1
13108	0	2.56	1	0.86729	.	.	1
13109	0	2.56	1	0.86729	.	.	1
13110	0	2.56	1	0.86729	.	.	1
13111	0	2.56	1	0.86729	.	.	1
13112	0	2.56	1	0.86729	.	.	1
13113	0	2.56	1	0.86729	.	.	1
13114	0	2.56	1	0.86729	.	.	1
13115	0	2.56	1	0.86729	.	.	1
13116	0	2.56	1	0.86729	.	.	1
13117	0	2.56	1	0.86729	.	.	1
13118	0	2.56	1	0.86729	.	.	1
13119	0	2.56	1	0.86729	.	.	1
13120	0	2.56	1	0.86729	.	.	1
13121	0	2.56	1	0.86729	.	.	1
13122	0	2.56	1	0.86729	.	.	1
13123	0	2.56	1	0.86729	.	.	1
13124	0	2.56	1	0.86729	.	.	1
13125	0	2.56	1	0.86729	.	.	1

Obs	Sub_group	fuptimey	_CENSOR_	SURVIVAL	SDF_LCL	SDF_UCL	STRATUM
13126	0	2.56	1	0.86729	.	.	1
13127	0	2.56	1	0.86729	.	.	1
13128	0	2.56	1	0.86729	.	.	1
13129	0	2.56	1	0.86729	.	.	1
13130	0	2.56	1	0.86729	.	.	1
13131	0	2.56	1	0.86729	.	.	1
13132	0	2.56	1	0.86729	.	.	1
13133	0	2.56	1	0.86729	.	.	1
13134	0	2.56	1	0.86729	.	.	1
13135	0	2.56	1	0.86729	.	.	1
13136	0	2.56	1	0.86729	.	.	1
13137	0	2.56	1	0.86729	.	.	1
13138	0	2.56	1	0.86729	.	.	1
13139	0	2.56	1	0.86729	.	.	1
13140	0	2.56	1	0.86729	.	.	1
13141	0	2.56	1	0.86729	.	.	1
13142	0	2.56	1	0.86729	.	.	1
13143	0	2.56	1	0.86729	.	.	1
13144	0	2.57	0	0.86699	0.86368	0.87022	1
13145	0	2.57	1	0.86699	.	.	1
13146	0	2.57	1	0.86699	.	.	1
13147	0	2.57	1	0.86699	.	.	1
13148	0	2.57	1	0.86699	.	.	1
13149	0	2.57	1	0.86699	.	.	1
13150	0	2.57	1	0.86699	.	.	1
13151	0	2.57	1	0.86699	.	.	1
13152	0	2.57	1	0.86699	.	.	1
13153	0	2.57	1	0.86699	.	.	1
13154	0	2.57	1	0.86699	.	.	1
13155	0	2.57	1	0.86699	.	.	1
13156	0	2.57	1	0.86699	.	.	1
13157	0	2.57	1	0.86699	.	.	1
13158	0	2.57	1	0.86699	.	.	1
13159	0	2.57	1	0.86699	.	.	1
13160	0	2.57	1	0.86699	.	.	1

Obs	Sub_group	fuptimey	_CENSOR_	SURVIVAL	SDF_LCL	SDF_UCL	STRATUM
13161	0	2.57	1	0.86699	.	.	1
13162	0	2.57	1	0.86699	.	.	1
13163	0	2.57	1	0.86699	.	.	1
13164	0	2.57	1	0.86699	.	.	1
13165	0	2.57	1	0.86699	.	.	1
13166	0	2.57	1	0.86699	.	.	1
13167	0	2.57	1	0.86699	.	.	1
13168	0	2.57	1	0.86699	.	.	1
13169	0	2.57	1	0.86699	.	.	1
13170	0	2.57	1	0.86699	.	.	1
13171	0	2.57	1	0.86699	.	.	1
13172	0	2.57	1	0.86699	.	.	1
13173	0	2.57	1	0.86699	.	.	1
13174	0	2.57	1	0.86699	.	.	1
13175	0	2.57	1	0.86699	.	.	1
13176	0	2.57	1	0.86699	.	.	1
13177	0	2.57	1	0.86699	.	.	1
13178	0	2.57	1	0.86699	.	.	1
13179	0	2.57	1	0.86699	.	.	1
13180	0	2.57	1	0.86699	.	.	1
13181	0	2.57	1	0.86699	.	.	1
13182	0	2.58	0	0.86655	0.86324	0.86980	1
13183	0	2.58	1	0.86655	.	.	1
13184	0	2.58	1	0.86655	.	.	1
13185	0	2.58	1	0.86655	.	.	1
13186	0	2.58	1	0.86655	.	.	1
13187	0	2.58	1	0.86655	.	.	1
13188	0	2.58	1	0.86655	.	.	1
13189	0	2.58	1	0.86655	.	.	1
13190	0	2.58	1	0.86655	.	.	1
13191	0	2.58	1	0.86655	.	.	1
13192	0	2.58	1	0.86655	.	.	1
13193	0	2.58	1	0.86655	.	.	1
13194	0	2.58	1	0.86655	.	.	1
13195	0	2.58	1	0.86655	.	.	1

Obs	Sub_group	fuptimey	_CENSOR_	SURVIVAL	SDF_LCL	SDF_UCL	STRATUM
13196	0	2.58	1	0.86655	.	.	1
13197	0	2.58	1	0.86655	.	.	1
13198	0	2.58	1	0.86655	.	.	1
13199	0	2.58	1	0.86655	.	.	1
13200	0	2.58	1	0.86655	.	.	1
13201	0	2.58	1	0.86655	.	.	1
13202	0	2.58	1	0.86655	.	.	1
13203	0	2.58	1	0.86655	.	.	1
13204	0	2.58	1	0.86655	.	.	1
13205	0	2.58	1	0.86655	.	.	1
13206	0	2.58	1	0.86655	.	.	1
13207	0	2.58	1	0.86655	.	.	1
13208	0	2.58	1	0.86655	.	.	1
13209	0	2.58	1	0.86655	.	.	1
13210	0	2.58	1	0.86655	.	.	1
13211	0	2.58	1	0.86655	.	.	1
13212	0	2.58	1	0.86655	.	.	1
13213	0	2.58	1	0.86655	.	.	1
13214	0	2.58	1	0.86655	.	.	1
13215	0	2.58	1	0.86655	.	.	1
13216	0	2.58	1	0.86655	.	.	1
13217	0	2.58	1	0.86655	.	.	1
13218	0	2.58	1	0.86655	.	.	1
13219	0	2.58	1	0.86655	.	.	1
13220	0	2.59	0	0.86624	0.86292	0.86949	1
13221	0	2.59	1	0.86624	.	.	1
13222	0	2.59	1	0.86624	.	.	1
13223	0	2.59	1	0.86624	.	.	1
13224	0	2.59	1	0.86624	.	.	1
13225	0	2.59	1	0.86624	.	.	1
13226	0	2.59	1	0.86624	.	.	1
13227	0	2.59	1	0.86624	.	.	1
13228	0	2.59	1	0.86624	.	.	1
13229	0	2.59	1	0.86624	.	.	1
13230	0	2.59	1	0.86624	.	.	1

Obs	Sub_group	fuptimey	_CENSOR_	SURVIVAL	SDF_LCL	SDF_UCL	STRATUM
13231	0	2.59	1	0.86624	.	.	1
13232	0	2.59	1	0.86624	.	.	1
13233	0	2.59	1	0.86624	.	.	1
13234	0	2.59	1	0.86624	.	.	1
13235	0	2.59	1	0.86624	.	.	1
13236	0	2.59	1	0.86624	.	.	1
13237	0	2.59	1	0.86624	.	.	1
13238	0	2.59	1	0.86624	.	.	1
13239	0	2.59	1	0.86624	.	.	1
13240	0	2.59	1	0.86624	.	.	1
13241	0	2.59	1	0.86624	.	.	1
13242	0	2.59	1	0.86624	.	.	1
13243	0	2.59	1	0.86624	.	.	1
13244	0	2.59	1	0.86624	.	.	1
13245	0	2.59	1	0.86624	.	.	1
13246	0	2.59	1	0.86624	.	.	1
13247	0	2.59	1	0.86624	.	.	1
13248	0	2.59	1	0.86624	.	.	1
13249	0	2.59	1	0.86624	.	.	1
13250	0	2.59	1	0.86624	.	.	1
13251	0	2.59	1	0.86624	.	.	1
13252	0	2.59	1	0.86624	.	.	1
13253	0	2.59	1	0.86624	.	.	1
13254	0	2.59	1	0.86624	.	.	1
13255	0	2.59	1	0.86624	.	.	1
13256	0	2.59	1	0.86624	.	.	1
13257	0	2.59	1	0.86624	.	.	1
13258	0	2.59	1	0.86624	.	.	1
13259	0	2.59	1	0.86624	.	.	1
13260	0	2.59	1	0.86624	.	.	1
13261	0	2.59	1	0.86624	.	.	1
13262	0	2.59	1	0.86624	.	.	1
13263	0	2.59	1	0.86624	.	.	1
13264	0	2.59	1	0.86624	.	.	1
13265	0	2.59	1	0.86624	.	.	1

Obs	Sub_group	fuptimey	_CENSOR_	SURVIVAL	SDF_LCL	SDF_UCL	STRATUM
13266	0	2.59	1	0.86624	.	.	1
13267	0	2.59	1	0.86624	.	.	1
13268	0	2.59	1	0.86624	.	.	1
13269	0	2.59	1	0.86624	.	.	1
13270	0	2.59	1	0.86624	.	.	1
13271	0	2.59	1	0.86624	.	.	1
13272	0	2.59	1	0.86624	.	.	1
13273	0	2.59	1	0.86624	.	.	1
13274	0	2.59	1	0.86624	.	.	1
13275	0	2.59	1	0.86624	.	.	1
13276	0	2.59	1	0.86624	.	.	1
13277	0	2.60	0	0.86603	0.86270	0.86928	1
13278	0	2.60	1	0.86603	.	.	1
13279	0	2.60	1	0.86603	.	.	1
13280	0	2.60	1	0.86603	.	.	1
13281	0	2.60	1	0.86603	.	.	1
13282	0	2.60	1	0.86603	.	.	1
13283	0	2.60	1	0.86603	.	.	1
13284	0	2.60	1	0.86603	.	.	1
13285	0	2.60	1	0.86603	.	.	1
13286	0	2.60	1	0.86603	.	.	1
13287	0	2.60	1	0.86603	.	.	1
13288	0	2.60	1	0.86603	.	.	1
13289	0	2.60	1	0.86603	.	.	1
13290	0	2.60	1	0.86603	.	.	1
13291	0	2.60	1	0.86603	.	.	1
13292	0	2.60	1	0.86603	.	.	1
13293	0	2.60	1	0.86603	.	.	1
13294	0	2.60	1	0.86603	.	.	1
13295	0	2.60	1	0.86603	.	.	1
13296	0	2.60	1	0.86603	.	.	1
13297	0	2.60	1	0.86603	.	.	1
13298	0	2.60	1	0.86603	.	.	1
13299	0	2.60	1	0.86603	.	.	1
13300	0	2.60	1	0.86603	.	.	1

Obs	Sub_group	fuptimey	_CENSOR_	SURVIVAL	SDF_LCL	SDF_UCL	STRATUM
13301	0	2.60	1	0.86603	.	.	1
13302	0	2.60	1	0.86603	.	.	1
13303	0	2.60	1	0.86603	.	.	1
13304	0	2.60	1	0.86603	.	.	1
13305	0	2.60	1	0.86603	.	.	1
13306	0	2.60	1	0.86603	.	.	1
13307	0	2.60	1	0.86603	.	.	1
13308	0	2.60	1	0.86603	.	.	1
13309	0	2.60	1	0.86603	.	.	1
13310	0	2.60	1	0.86603	.	.	1
13311	0	2.61	0	0.86572	0.86239	0.86897	1
13312	0	2.61	1	0.86572	.	.	1
13313	0	2.61	1	0.86572	.	.	1
13314	0	2.61	1	0.86572	.	.	1
13315	0	2.61	1	0.86572	.	.	1
13316	0	2.61	1	0.86572	.	.	1
13317	0	2.61	1	0.86572	.	.	1
13318	0	2.61	1	0.86572	.	.	1
13319	0	2.61	1	0.86572	.	.	1
13320	0	2.61	1	0.86572	.	.	1
13321	0	2.61	1	0.86572	.	.	1
13322	0	2.61	1	0.86572	.	.	1
13323	0	2.61	1	0.86572	.	.	1
13324	0	2.61	1	0.86572	.	.	1
13325	0	2.61	1	0.86572	.	.	1
13326	0	2.61	1	0.86572	.	.	1
13327	0	2.61	1	0.86572	.	.	1
13328	0	2.61	1	0.86572	.	.	1
13329	0	2.61	1	0.86572	.	.	1
13330	0	2.61	1	0.86572	.	.	1
13331	0	2.61	1	0.86572	.	.	1
13332	0	2.61	1	0.86572	.	.	1
13333	0	2.61	1	0.86572	.	.	1
13334	0	2.61	1	0.86572	.	.	1
13335	0	2.61	1	0.86572	.	.	1

Obs	Sub_group	fuptimey	_CENSOR_	SURVIVAL	SDF_LCL	SDF_UCL	STRATUM
13336	0	2.61	1	0.86572	.	.	1
13337	0	2.61	1	0.86572	.	.	1
13338	0	2.61	1	0.86572	.	.	1
13339	0	2.61	1	0.86572	.	.	1
13340	0	2.61	1	0.86572	.	.	1
13341	0	2.61	1	0.86572	.	.	1
13342	0	2.61	1	0.86572	.	.	1
13343	0	2.61	1	0.86572	.	.	1
13344	0	2.61	1	0.86572	.	.	1
13345	0	2.61	1	0.86572	.	.	1
13346	0	2.61	1	0.86572	.	.	1
13347	0	2.61	1	0.86572	.	.	1
13348	0	2.61	1	0.86572	.	.	1
13349	0	2.61	1	0.86572	.	.	1
13350	0	2.61	1	0.86572	.	.	1
13351	0	2.61	1	0.86572	.	.	1
13352	0	2.61	1	0.86572	.	.	1
13353	0	2.61	1	0.86572	.	.	1
13354	0	2.61	1	0.86572	.	.	1
13355	0	2.62	0	0.86538	0.86204	0.86864	1
13356	0	2.62	1	0.86538	.	.	1
13357	0	2.62	1	0.86538	.	.	1
13358	0	2.62	1	0.86538	.	.	1
13359	0	2.62	1	0.86538	.	.	1
13360	0	2.62	1	0.86538	.	.	1
13361	0	2.62	1	0.86538	.	.	1
13362	0	2.62	1	0.86538	.	.	1
13363	0	2.62	1	0.86538	.	.	1
13364	0	2.62	1	0.86538	.	.	1
13365	0	2.62	1	0.86538	.	.	1
13366	0	2.62	1	0.86538	.	.	1
13367	0	2.62	1	0.86538	.	.	1
13368	0	2.62	1	0.86538	.	.	1
13369	0	2.62	1	0.86538	.	.	1
13370	0	2.62	1	0.86538	.	.	1

Obs	Sub_group	fuptimey	_CENSOR_	SURVIVAL	SDF_LCL	SDF_UCL	STRATUM
13371	0	2.62	1	0.86538	.	.	1
13372	0	2.62	1	0.86538	.	.	1
13373	0	2.62	1	0.86538	.	.	1
13374	0	2.62	1	0.86538	.	.	1
13375	0	2.62	1	0.86538	.	.	1
13376	0	2.62	1	0.86538	.	.	1
13377	0	2.62	1	0.86538	.	.	1
13378	0	2.62	1	0.86538	.	.	1
13379	0	2.62	1	0.86538	.	.	1
13380	0	2.62	1	0.86538	.	.	1
13381	0	2.62	1	0.86538	.	.	1
13382	0	2.62	1	0.86538	.	.	1
13383	0	2.62	1	0.86538	.	.	1
13384	0	2.62	1	0.86538	.	.	1
13385	0	2.62	1	0.86538	.	.	1
13386	0	2.62	1	0.86538	.	.	1
13387	0	2.62	1	0.86538	.	.	1
13388	0	2.62	1	0.86538	.	.	1
13389	0	2.62	1	0.86538	.	.	1
13390	0	2.63	0	0.86504	0.86170	0.86830	1
13391	0	2.63	1	0.86504	.	.	1
13392	0	2.63	1	0.86504	.	.	1
13393	0	2.63	1	0.86504	.	.	1
13394	0	2.63	1	0.86504	.	.	1
13395	0	2.63	1	0.86504	.	.	1
13396	0	2.63	1	0.86504	.	.	1
13397	0	2.63	1	0.86504	.	.	1
13398	0	2.63	1	0.86504	.	.	1
13399	0	2.63	1	0.86504	.	.	1
13400	0	2.63	1	0.86504	.	.	1
13401	0	2.63	1	0.86504	.	.	1
13402	0	2.63	1	0.86504	.	.	1
13403	0	2.63	1	0.86504	.	.	1
13404	0	2.63	1	0.86504	.	.	1
13405	0	2.63	1	0.86504	.	.	1

Obs	Sub_group	fuptimey	_CENSOR_	SURVIVAL	SDF_LCL	SDF_UCL	STRATUM
13406	0	2.63	1	0.86504	.	.	1
13407	0	2.63	1	0.86504	.	.	1
13408	0	2.63	1	0.86504	.	.	1
13409	0	2.63	1	0.86504	.	.	1
13410	0	2.63	1	0.86504	.	.	1
13411	0	2.63	1	0.86504	.	.	1
13412	0	2.63	1	0.86504	.	.	1
13413	0	2.63	1	0.86504	.	.	1
13414	0	2.63	1	0.86504	.	.	1
13415	0	2.63	1	0.86504	.	.	1
13416	0	2.63	1	0.86504	.	.	1
13417	0	2.63	1	0.86504	.	.	1
13418	0	2.63	1	0.86504	.	.	1
13419	0	2.63	1	0.86504	.	.	1
13420	0	2.63	1	0.86504	.	.	1
13421	0	2.63	1	0.86504	.	.	1
13422	0	2.63	1	0.86504	.	.	1
13423	0	2.63	1	0.86504	.	.	1
13424	0	2.63	1	0.86504	.	.	1
13425	0	2.63	1	0.86504	.	.	1
13426	0	2.63	1	0.86504	.	.	1
13427	0	2.63	1	0.86504	.	.	1
13428	0	2.63	1	0.86504	.	.	1
13429	0	2.63	1	0.86504	.	.	1
13430	0	2.63	1	0.86504	.	.	1
13431	0	2.63	1	0.86504	.	.	1
13432	0	2.63	1	0.86504	.	.	1
13433	0	2.63	1	0.86504	.	.	1
13434	0	2.63	1	0.86504	.	.	1
13435	0	2.63	1	0.86504	.	.	1
13436	0	2.63	1	0.86504	.	.	1
13437	0	2.63	1	0.86504	.	.	1
13438	0	2.64	0	0.86485	0.86151	0.86812	1
13439	0	2.64	1	0.86485	.	.	1
13440	0	2.64	1	0.86485	.	.	1

Obs	Sub_group	fuptimey	_CENSOR_	SURVIVAL	SDF_LCL	SDF_UCL	STRATUM
13441	0	2.64	1	0.86485	.	.	1
13442	0	2.64	1	0.86485	.	.	1
13443	0	2.64	1	0.86485	.	.	1
13444	0	2.64	1	0.86485	.	.	1
13445	0	2.64	1	0.86485	.	.	1
13446	0	2.64	1	0.86485	.	.	1
13447	0	2.64	1	0.86485	.	.	1
13448	0	2.64	1	0.86485	.	.	1
13449	0	2.64	1	0.86485	.	.	1
13450	0	2.64	1	0.86485	.	.	1
13451	0	2.64	1	0.86485	.	.	1
13452	0	2.64	1	0.86485	.	.	1
13453	0	2.64	1	0.86485	.	.	1
13454	0	2.64	1	0.86485	.	.	1
13455	0	2.64	1	0.86485	.	.	1
13456	0	2.64	1	0.86485	.	.	1
13457	0	2.64	1	0.86485	.	.	1
13458	0	2.64	1	0.86485	.	.	1
13459	0	2.64	1	0.86485	.	.	1
13460	0	2.64	1	0.86485	.	.	1
13461	0	2.64	1	0.86485	.	.	1
13462	0	2.64	1	0.86485	.	.	1
13463	0	2.64	1	0.86485	.	.	1
13464	0	2.64	1	0.86485	.	.	1
13465	0	2.64	1	0.86485	.	.	1
13466	0	2.64	1	0.86485	.	.	1
13467	0	2.64	1	0.86485	.	.	1
13468	0	2.64	1	0.86485	.	.	1
13469	0	2.64	1	0.86485	.	.	1
13470	0	2.64	1	0.86485	.	.	1
13471	0	2.64	1	0.86485	.	.	1
13472	0	2.64	1	0.86485	.	.	1
13473	0	2.64	1	0.86485	.	.	1
13474	0	2.64	1	0.86485	.	.	1
13475	0	2.64	1	0.86485	.	.	1

Obs	Sub_group	fuptimey	_CENSOR_	SURVIVAL	SDF_LCL	SDF_UCL	STRATUM
13476	0	2.64	1	0.86485	.	.	1
13477	0	2.64	1	0.86485	.	.	1
13478	0	2.64	1	0.86485	.	.	1
13479	0	2.64	1	0.86485	.	.	1
13480	0	2.64	1	0.86485	.	.	1
13481	0	2.64	1	0.86485	.	.	1
13482	0	2.64	1	0.86485	.	.	1
13483	0	2.65	0	0.86469	0.86135	0.86796	1
13484	0	2.65	1	0.86469	.	.	1
13485	0	2.65	1	0.86469	.	.	1
13486	0	2.65	1	0.86469	.	.	1
13487	0	2.65	1	0.86469	.	.	1
13488	0	2.65	1	0.86469	.	.	1
13489	0	2.65	1	0.86469	.	.	1
13490	0	2.65	1	0.86469	.	.	1
13491	0	2.65	1	0.86469	.	.	1
13492	0	2.65	1	0.86469	.	.	1
13493	0	2.65	1	0.86469	.	.	1
13494	0	2.65	1	0.86469	.	.	1
13495	0	2.65	1	0.86469	.	.	1
13496	0	2.65	1	0.86469	.	.	1
13497	0	2.65	1	0.86469	.	.	1
13498	0	2.65	1	0.86469	.	.	1
13499	0	2.65	1	0.86469	.	.	1
13500	0	2.65	1	0.86469	.	.	1
13501	0	2.65	1	0.86469	.	.	1
13502	0	2.65	1	0.86469	.	.	1
13503	0	2.65	1	0.86469	.	.	1
13504	0	2.65	1	0.86469	.	.	1
13505	0	2.65	1	0.86469	.	.	1
13506	0	2.65	1	0.86469	.	.	1
13507	0	2.65	1	0.86469	.	.	1
13508	0	2.65	1	0.86469	.	.	1
13509	0	2.65	1	0.86469	.	.	1
13510	0	2.65	1	0.86469	.	.	1

Obs	Sub_group	fuptimey	_CENSOR_	SURVIVAL	SDF_LCL	SDF_UCL	STRATUM
**13511**	0	2.65	1	0.86469	.	.	1
**13512**	0	2.65	1	0.86469	.	.	1
**13513**	0	2.65	1	0.86469	.	.	1
**13514**	0	2.65	1	0.86469	.	.	1
**13515**	0	2.65	1	0.86469	.	.	1
**13516**	0	2.65	1	0.86469	.	.	1
**13517**	0	2.65	1	0.86469	.	.	1
**13518**	0	2.65	1	0.86469	.	.	1
**13519**	0	2.65	1	0.86469	.	.	1
**13520**	0	2.65	1	0.86469	.	.	1
**13521**	0	2.65	1	0.86469	.	.	1
**13522**	0	2.65	1	0.86469	.	.	1
**13523**	0	2.65	1	0.86469	.	.	1
**13524**	0	2.65	1	0.86469	.	.	1
**13525**	0	2.65	1	0.86469	.	.	1
**13526**	0	2.65	1	0.86469	.	.	1
**13527**	0	2.65	1	0.86469	.	.	1
**13528**	0	2.65	1	0.86469	.	.	1
**13529**	0	2.65	1	0.86469	.	.	1
**13530**	0	2.65	1	0.86469	.	.	1
**13531**	0	2.65	1	0.86469	.	.	1
**13532**	0	2.65	1	0.86469	.	.	1
**13533**	0	2.65	1	0.86469	.	.	1
**13534**	0	2.65	1	0.86469	.	.	1
**13535**	0	2.65	1	0.86469	.	.	1
**13536**	0	2.65	1	0.86469	.	.	1
**13537**	0	2.66	0	0.86454	0.86119	0.86781	1
**13538**	0	2.66	1	0.86454	.	.	1
**13539**	0	2.66	1	0.86454	.	.	1
**13540**	0	2.66	1	0.86454	.	.	1
**13541**	0	2.66	1	0.86454	.	.	1
**13542**	0	2.66	1	0.86454	.	.	1
**13543**	0	2.66	1	0.86454	.	.	1
**13544**	0	2.66	1	0.86454	.	.	1
**13545**	0	2.66	1	0.86454	.	.	1

Obs	Sub_group	fuptimey	_CENSOR_	SURVIVAL	SDF_LCL	SDF_UCL	STRATUM
13546	0	2.66	1	0.86454	.	.	1
13547	0	2.66	1	0.86454	.	.	1
13548	0	2.66	1	0.86454	.	.	1
13549	0	2.66	1	0.86454	.	.	1
13550	0	2.66	1	0.86454	.	.	1
13551	0	2.66	1	0.86454	.	.	1
13552	0	2.66	1	0.86454	.	.	1
13553	0	2.66	1	0.86454	.	.	1
13554	0	2.66	1	0.86454	.	.	1
13555	0	2.66	1	0.86454	.	.	1
13556	0	2.66	1	0.86454	.	.	1
13557	0	2.66	1	0.86454	.	.	1
13558	0	2.66	1	0.86454	.	.	1
13559	0	2.66	1	0.86454	.	.	1
13560	0	2.66	1	0.86454	.	.	1
13561	0	2.66	1	0.86454	.	.	1
13562	0	2.66	1	0.86454	.	.	1
13563	0	2.66	1	0.86454	.	.	1
13564	0	2.66	1	0.86454	.	.	1
13565	0	2.66	1	0.86454	.	.	1
13566	0	2.66	1	0.86454	.	.	1
13567	0	2.66	1	0.86454	.	.	1
13568	0	2.66	1	0.86454	.	.	1
13569	0	2.67	0	0.86441	0.86107	0.86768	1
13570	0	2.67	1	0.86441	.	.	1
13571	0	2.67	1	0.86441	.	.	1
13572	0	2.67	1	0.86441	.	.	1
13573	0	2.67	1	0.86441	.	.	1
13574	0	2.67	1	0.86441	.	.	1
13575	0	2.67	1	0.86441	.	.	1
13576	0	2.67	1	0.86441	.	.	1
13577	0	2.67	1	0.86441	.	.	1
13578	0	2.67	1	0.86441	.	.	1
13579	0	2.67	1	0.86441	.	.	1
13580	0	2.67	1	0.86441	.	.	1

Obs	Sub_group	fuptimey	_CENSOR_	SURVIVAL	SDF_LCL	SDF_UCL	STRATUM
13581	0	2.67	1	0.86441	.	.	1
13582	0	2.67	1	0.86441	.	.	1
13583	0	2.67	1	0.86441	.	.	1
13584	0	2.67	1	0.86441	.	.	1
13585	0	2.67	1	0.86441	.	.	1
13586	0	2.67	1	0.86441	.	.	1
13587	0	2.67	1	0.86441	.	.	1
13588	0	2.67	1	0.86441	.	.	1
13589	0	2.67	1	0.86441	.	.	1
13590	0	2.67	1	0.86441	.	.	1
13591	0	2.67	1	0.86441	.	.	1
13592	0	2.67	1	0.86441	.	.	1
13593	0	2.67	1	0.86441	.	.	1
13594	0	2.67	1	0.86441	.	.	1
13595	0	2.67	1	0.86441	.	.	1
13596	0	2.67	1	0.86441	.	.	1
13597	0	2.67	1	0.86441	.	.	1
13598	0	2.67	1	0.86441	.	.	1
13599	0	2.67	1	0.86441	.	.	1
13600	0	2.67	1	0.86441	.	.	1
13601	0	2.67	1	0.86441	.	.	1
13602	0	2.67	1	0.86441	.	.	1
13603	0	2.67	1	0.86441	.	.	1
13604	0	2.67	1	0.86441	.	.	1
13605	0	2.67	1	0.86441	.	.	1
13606	0	2.67	1	0.86441	.	.	1
13607	0	2.67	1	0.86441	.	.	1
13608	0	2.67	1	0.86441	.	.	1
13609	0	2.67	1	0.86441	.	.	1
13610	0	2.67	1	0.86441	.	.	1
13611	0	2.67	1	0.86441	.	.	1
13612	0	2.67	1	0.86441	.	.	1
13613	0	2.67	1	0.86441	.	.	1
13614	0	2.67	1	0.86441	.	.	1
13615	0	2.67	1	0.86441	.	.	1

Obs	Sub_group	fuptimey	_CENSOR_	SURVIVAL	SDF_LCL	SDF_UCL	STRATUM
13616	0	2.67	1	0.86441	.	.	1
13617	0	2.67	1	0.86441	.	.	1
13618	0	2.67	1	0.86441	.	.	1
13619	0	2.67	1	0.86441	.	.	1
13620	0	2.67	1	0.86441	.	.	1
13621	0	2.67	1	0.86441	.	.	1
13622	0	2.68	0	0.86419	0.86084	0.86747	1
13623	0	2.68	1	0.86419	.	.	1
13624	0	2.68	1	0.86419	.	.	1
13625	0	2.68	1	0.86419	.	.	1
13626	0	2.68	1	0.86419	.	.	1
13627	0	2.68	1	0.86419	.	.	1
13628	0	2.68	1	0.86419	.	.	1
13629	0	2.68	1	0.86419	.	.	1
13630	0	2.68	1	0.86419	.	.	1
13631	0	2.68	1	0.86419	.	.	1
13632	0	2.68	1	0.86419	.	.	1
13633	0	2.68	1	0.86419	.	.	1
13634	0	2.68	1	0.86419	.	.	1
13635	0	2.68	1	0.86419	.	.	1
13636	0	2.68	1	0.86419	.	.	1
13637	0	2.68	1	0.86419	.	.	1
13638	0	2.68	1	0.86419	.	.	1
13639	0	2.68	1	0.86419	.	.	1
13640	0	2.68	1	0.86419	.	.	1
13641	0	2.68	1	0.86419	.	.	1
13642	0	2.68	1	0.86419	.	.	1
13643	0	2.68	1	0.86419	.	.	1
13644	0	2.68	1	0.86419	.	.	1
13645	0	2.68	1	0.86419	.	.	1
13646	0	2.68	1	0.86419	.	.	1
13647	0	2.68	1	0.86419	.	.	1
13648	0	2.68	1	0.86419	.	.	1
13649	0	2.68	1	0.86419	.	.	1
13650	0	2.68	1	0.86419	.	.	1

Obs	Sub_group	fuptimey	_CENSOR_	SURVIVAL	SDF_LCL	SDF_UCL	STRATUM
13651	0	2.69	0	0.86394	0.86059	0.86722	1
13652	0	2.69	1	0.86394	.	.	1
13653	0	2.69	1	0.86394	.	.	1
13654	0	2.69	1	0.86394	.	.	1
13655	0	2.69	1	0.86394	.	.	1
13656	0	2.69	1	0.86394	.	.	1
13657	0	2.69	1	0.86394	.	.	1
13658	0	2.69	1	0.86394	.	.	1
13659	0	2.69	1	0.86394	.	.	1
13660	0	2.69	1	0.86394	.	.	1
13661	0	2.69	1	0.86394	.	.	1
13662	0	2.69	1	0.86394	.	.	1
13663	0	2.69	1	0.86394	.	.	1
13664	0	2.69	1	0.86394	.	.	1
13665	0	2.69	1	0.86394	.	.	1
13666	0	2.69	1	0.86394	.	.	1
13667	0	2.69	1	0.86394	.	.	1
13668	0	2.69	1	0.86394	.	.	1
13669	0	2.69	1	0.86394	.	.	1
13670	0	2.69	1	0.86394	.	.	1
13671	0	2.69	1	0.86394	.	.	1
13672	0	2.69	1	0.86394	.	.	1
13673	0	2.69	1	0.86394	.	.	1
13674	0	2.69	1	0.86394	.	.	1
13675	0	2.69	1	0.86394	.	.	1
13676	0	2.69	1	0.86394	.	.	1
13677	0	2.69	1	0.86394	.	.	1
13678	0	2.69	1	0.86394	.	.	1
13679	0	2.69	1	0.86394	.	.	1
13680	0	2.69	1	0.86394	.	.	1
13681	0	2.69	1	0.86394	.	.	1
13682	0	2.69	1	0.86394	.	.	1
13683	0	2.69	1	0.86394	.	.	1
13684	0	2.69	1	0.86394	.	.	1
13685	0	2.69	1	0.86394	.	.	1

Obs	Sub_group	fuptimey	_CENSOR_	SURVIVAL	SDF_LCL	SDF_UCL	STRATUM
13686	0	2.69	1	0.86394	.	.	1
13687	0	2.69	1	0.86394	.	.	1
13688	0	2.69	1	0.86394	.	.	1
13689	0	2.69	1	0.86394	.	.	1
13690	0	2.69	1	0.86394	.	.	1
13691	0	2.69	1	0.86394	.	.	1
13692	0	2.69	1	0.86394	.	.	1
13693	0	2.69	1	0.86394	.	.	1
13694	0	2.69	1	0.86394	.	.	1
13695	0	2.69	1	0.86394	.	.	1
13696	0	2.69	1	0.86394	.	.	1
13697	0	2.69	1	0.86394	.	.	1
13698	0	2.69	1	0.86394	.	.	1
13699	0	2.69	1	0.86394	.	.	1
13700	0	2.69	1	0.86394	.	.	1
13701	0	2.69	1	0.86394	.	.	1
13702	0	2.69	1	0.86394	.	.	1
13703	0	2.69	1	0.86394	.	.	1
13704	0	2.69	1	0.86394	.	.	1
13705	0	2.69	1	0.86394	.	.	1
13706	0	2.69	1	0.86394	.	.	1
13707	0	2.70	0	0.86366	0.86030	0.86694	1
13708	0	2.70	1	0.86366	.	.	1
13709	0	2.70	1	0.86366	.	.	1
13710	0	2.70	1	0.86366	.	.	1
13711	0	2.70	1	0.86366	.	.	1
13712	0	2.70	1	0.86366	.	.	1
13713	0	2.70	1	0.86366	.	.	1
13714	0	2.70	1	0.86366	.	.	1
13715	0	2.70	1	0.86366	.	.	1
13716	0	2.70	1	0.86366	.	.	1
13717	0	2.70	1	0.86366	.	.	1
13718	0	2.70	1	0.86366	.	.	1
13719	0	2.70	1	0.86366	.	.	1
13720	0	2.70	1	0.86366	.	.	1

Obs	Sub_group	fuptimey	_CENSOR_	SURVIVAL	SDF_LCL	SDF_UCL	STRATUM
13721	0	2.70	1	0.86366	.	.	1
13722	0	2.70	1	0.86366	.	.	1
13723	0	2.70	1	0.86366	.	.	1
13724	0	2.70	1	0.86366	.	.	1
13725	0	2.70	1	0.86366	.	.	1
13726	0	2.70	1	0.86366	.	.	1
13727	0	2.70	1	0.86366	.	.	1
13728	0	2.70	1	0.86366	.	.	1
13729	0	2.70	1	0.86366	.	.	1
13730	0	2.70	1	0.86366	.	.	1
13731	0	2.70	1	0.86366	.	.	1
13732	0	2.71	0	0.86338	0.86002	0.86666	1
13733	0	2.71	1	0.86338	.	.	1
13734	0	2.71	1	0.86338	.	.	1
13735	0	2.71	1	0.86338	.	.	1
13736	0	2.71	1	0.86338	.	.	1
13737	0	2.71	1	0.86338	.	.	1
13738	0	2.71	1	0.86338	.	.	1
13739	0	2.71	1	0.86338	.	.	1
13740	0	2.71	1	0.86338	.	.	1
13741	0	2.71	1	0.86338	.	.	1
13742	0	2.71	1	0.86338	.	.	1
13743	0	2.71	1	0.86338	.	.	1
13744	0	2.71	1	0.86338	.	.	1
13745	0	2.71	1	0.86338	.	.	1
13746	0	2.71	1	0.86338	.	.	1
13747	0	2.71	1	0.86338	.	.	1
13748	0	2.71	1	0.86338	.	.	1
13749	0	2.71	1	0.86338	.	.	1
13750	0	2.71	1	0.86338	.	.	1
13751	0	2.71	1	0.86338	.	.	1
13752	0	2.71	1	0.86338	.	.	1
13753	0	2.71	1	0.86338	.	.	1
13754	0	2.71	1	0.86338	.	.	1
13755	0	2.71	1	0.86338	.	.	1

Obs	Sub_group	fuptimey	_CENSOR_	SURVIVAL	SDF_LCL	SDF_UCL	STRATUM
13756	0	2.71	1	0.86338	.	.	1
13757	0	2.71	1	0.86338	.	.	1
13758	0	2.71	1	0.86338	.	.	1
13759	0	2.71	1	0.86338	.	.	1
13760	0	2.71	1	0.86338	.	.	1
13761	0	2.71	1	0.86338	.	.	1
13762	0	2.71	1	0.86338	.	.	1
13763	0	2.71	1	0.86338	.	.	1
13764	0	2.71	1	0.86338	.	.	1
13765	0	2.71	1	0.86338	.	.	1
13766	0	2.71	1	0.86338	.	.	1
13767	0	2.71	1	0.86338	.	.	1
13768	0	2.71	1	0.86338	.	.	1
13769	0	2.71	1	0.86338	.	.	1
13770	0	2.71	1	0.86338	.	.	1
13771	0	2.71	1	0.86338	.	.	1
13772	0	2.71	1	0.86338	.	.	1
13773	0	2.71	1	0.86338	.	.	1
13774	0	2.71	1	0.86338	.	.	1
13775	0	2.71	1	0.86338	.	.	1
13776	0	2.71	1	0.86338	.	.	1
13777	0	2.71	1	0.86338	.	.	1
13778	0	2.71	1	0.86338	.	.	1
13779	0	2.71	1	0.86338	.	.	1
13780	0	2.71	1	0.86338	.	.	1
13781	0	2.71	1	0.86338	.	.	1
13782	0	2.71	1	0.86338	.	.	1
13783	0	2.71	1	0.86338	.	.	1
13784	0	2.71	1	0.86338	.	.	1
13785	0	2.71	1	0.86338	.	.	1
13786	0	2.71	1	0.86338	.	.	1
13787	0	2.71	1	0.86338	.	.	1
13788	0	2.71	1	0.86338	.	.	1
13789	0	2.71	1	0.86338	.	.	1
13790	0	2.72	0	0.86300	0.85963	0.86629	1

Obs	Sub_group	fuptimey	_CENSOR_	SURVIVAL	SDF_LCL	SDF_UCL	STRATUM
13791	0	2.72	1	0.86300	.	.	1
13792	0	2.72	1	0.86300	.	.	1
13793	0	2.72	1	0.86300	.	.	1
13794	0	2.72	1	0.86300	.	.	1
13795	0	2.72	1	0.86300	.	.	1
13796	0	2.72	1	0.86300	.	.	1
13797	0	2.72	1	0.86300	.	.	1
13798	0	2.72	1	0.86300	.	.	1
13799	0	2.72	1	0.86300	.	.	1
13800	0	2.72	1	0.86300	.	.	1
13801	0	2.72	1	0.86300	.	.	1
13802	0	2.72	1	0.86300	.	.	1
13803	0	2.72	1	0.86300	.	.	1
13804	0	2.72	1	0.86300	.	.	1
13805	0	2.72	1	0.86300	.	.	1
13806	0	2.72	1	0.86300	.	.	1
13807	0	2.72	1	0.86300	.	.	1
13808	0	2.72	1	0.86300	.	.	1
13809	0	2.72	1	0.86300	.	.	1
13810	0	2.72	1	0.86300	.	.	1
13811	0	2.72	1	0.86300	.	.	1
13812	0	2.72	1	0.86300	.	.	1
13813	0	2.72	1	0.86300	.	.	1
13814	0	2.72	1	0.86300	.	.	1
13815	0	2.72	1	0.86300	.	.	1
13816	0	2.72	1	0.86300	.	.	1
13817	0	2.73	0	0.86278	0.85941	0.86607	1
13818	0	2.73	1	0.86278	.	.	1
13819	0	2.73	1	0.86278	.	.	1
13820	0	2.73	1	0.86278	.	.	1
13821	0	2.73	1	0.86278	.	.	1
13822	0	2.73	1	0.86278	.	.	1
13823	0	2.73	1	0.86278	.	.	1
13824	0	2.73	1	0.86278	.	.	1
13825	0	2.73	1	0.86278	.	.	1

Obs	Sub_group	fuptimey	_CENSOR_	SURVIVAL	SDF_LCL	SDF_UCL	STRATUM
13826	0	2.73	1	0.86278	.	.	1
13827	0	2.73	1	0.86278	.	.	1
13828	0	2.73	1	0.86278	.	.	1
13829	0	2.73	1	0.86278	.	.	1
13830	0	2.73	1	0.86278	.	.	1
13831	0	2.73	1	0.86278	.	.	1
13832	0	2.73	1	0.86278	.	.	1
13833	0	2.73	1	0.86278	.	.	1
13834	0	2.73	1	0.86278	.	.	1
13835	0	2.73	1	0.86278	.	.	1
13836	0	2.73	1	0.86278	.	.	1
13837	0	2.73	1	0.86278	.	.	1
13838	0	2.73	1	0.86278	.	.	1
13839	0	2.73	1	0.86278	.	.	1
13840	0	2.73	1	0.86278	.	.	1
13841	0	2.73	1	0.86278	.	.	1
13842	0	2.73	1	0.86278	.	.	1
13843	0	2.73	1	0.86278	.	.	1
13844	0	2.73	1	0.86278	.	.	1
13845	0	2.73	1	0.86278	.	.	1
13846	0	2.73	1	0.86278	.	.	1
13847	0	2.73	1	0.86278	.	.	1
13848	0	2.73	1	0.86278	.	.	1
13849	0	2.73	1	0.86278	.	.	1
13850	0	2.73	1	0.86278	.	.	1
13851	0	2.73	1	0.86278	.	.	1
13852	0	2.73	1	0.86278	.	.	1
13853	0	2.73	1	0.86278	.	.	1
13854	0	2.73	1	0.86278	.	.	1
13855	0	2.73	1	0.86278	.	.	1
13856	0	2.73	1	0.86278	.	.	1
13857	0	2.73	1	0.86278	.	.	1
13858	0	2.73	1	0.86278	.	.	1
13859	0	2.73	1	0.86278	.	.	1
13860	0	2.73	1	0.86278	.	.	1

Obs	Sub_group	fuptimey	_CENSOR_	SURVIVAL	SDF_LCL	SDF_UCL	STRATUM
13861	0	2.73	1	0.86278	.	.	1
13862	0	2.73	1	0.86278	.	.	1
13863	0	2.73	1	0.86278	.	.	1
13864	0	2.73	1	0.86278	.	.	1
13865	0	2.73	1	0.86278	.	.	1
13866	0	2.73	1	0.86278	.	.	1
13867	0	2.73	1	0.86278	.	.	1
13868	0	2.73	1	0.86278	.	.	1
13869	0	2.73	1	0.86278	.	.	1
13870	0	2.73	1	0.86278	.	.	1
13871	0	2.73	1	0.86278	.	.	1
13872	0	2.73	1	0.86278	.	.	1
13873	0	2.73	1	0.86278	.	.	1
13874	0	2.73	1	0.86278	.	.	1
13875	0	2.73	1	0.86278	.	.	1
13876	0	2.73	1	0.86278	.	.	1
13877	0	2.73	1	0.86278	.	.	1
13878	0	2.73	1	0.86278	.	.	1
13879	0	2.73	1	0.86278	.	.	1
13880	0	2.74	0	0.86237	0.85899	0.86567	1
13881	0	2.74	1	0.86237	.	.	1
13882	0	2.74	1	0.86237	.	.	1
13883	0	2.74	1	0.86237	.	.	1
13884	0	2.74	1	0.86237	.	.	1
13885	0	2.74	1	0.86237	.	.	1
13886	0	2.74	1	0.86237	.	.	1
13887	0	2.74	1	0.86237	.	.	1
13888	0	2.74	1	0.86237	.	.	1
13889	0	2.74	1	0.86237	.	.	1
13890	0	2.74	1	0.86237	.	.	1
13891	0	2.74	1	0.86237	.	.	1
13892	0	2.74	1	0.86237	.	.	1
13893	0	2.74	1	0.86237	.	.	1
13894	0	2.74	1	0.86237	.	.	1
13895	0	2.74	1	0.86237	.	.	1

Obs	Sub_group	fuptimey	_CENSOR_	SURVIVAL	SDF_LCL	SDF_UCL	STRATUM
13896	0	2.74	1	0.86237	.	.	1
13897	0	2.74	1	0.86237	.	.	1
13898	0	2.74	1	0.86237	.	.	1
13899	0	2.74	1	0.86237	.	.	1
13900	0	2.74	1	0.86237	.	.	1
13901	0	2.74	1	0.86237	.	.	1
13902	0	2.74	1	0.86237	.	.	1
13903	0	2.74	1	0.86237	.	.	1
13904	0	2.74	1	0.86237	.	.	1
13905	0	2.74	1	0.86237	.	.	1
13906	0	2.74	1	0.86237	.	.	1
13907	0	2.74	1	0.86237	.	.	1
13908	0	2.74	1	0.86237	.	.	1
13909	0	2.74	1	0.86237	.	.	1
13910	0	2.74	1	0.86237	.	.	1
13911	0	2.74	1	0.86237	.	.	1
13912	0	2.74	1	0.86237	.	.	1
13913	0	2.75	0	0.86215	0.85877	0.86545	1
13914	0	2.75	1	0.86215	.	.	1
13915	0	2.75	1	0.86215	.	.	1
13916	0	2.75	1	0.86215	.	.	1
13917	0	2.75	1	0.86215	.	.	1
13918	0	2.75	1	0.86215	.	.	1
13919	0	2.75	1	0.86215	.	.	1
13920	0	2.75	1	0.86215	.	.	1
13921	0	2.75	1	0.86215	.	.	1
13922	0	2.75	1	0.86215	.	.	1
13923	0	2.75	1	0.86215	.	.	1
13924	0	2.75	1	0.86215	.	.	1
13925	0	2.75	1	0.86215	.	.	1
13926	0	2.75	1	0.86215	.	.	1
13927	0	2.75	1	0.86215	.	.	1
13928	0	2.75	1	0.86215	.	.	1
13929	0	2.75	1	0.86215	.	.	1
13930	0	2.75	1	0.86215	.	.	1

Obs	Sub_group	fuptimey	_CENSOR_	SURVIVAL	SDF_LCL	SDF_UCL	STRATUM
13931	0	2.75	1	0.86215	.	.	1
13932	0	2.75	1	0.86215	.	.	1
13933	0	2.75	1	0.86215	.	.	1
13934	0	2.75	1	0.86215	.	.	1
13935	0	2.75	1	0.86215	.	.	1
13936	0	2.75	1	0.86215	.	.	1
13937	0	2.75	1	0.86215	.	.	1
13938	0	2.75	1	0.86215	.	.	1
13939	0	2.75	1	0.86215	.	.	1
13940	0	2.75	1	0.86215	.	.	1
13941	0	2.75	1	0.86215	.	.	1
13942	0	2.75	1	0.86215	.	.	1
13943	0	2.75	1	0.86215	.	.	1
13944	0	2.75	1	0.86215	.	.	1
13945	0	2.75	1	0.86215	.	.	1
13946	0	2.75	1	0.86215	.	.	1
13947	0	2.75	1	0.86215	.	.	1
13948	0	2.75	1	0.86215	.	.	1
13949	0	2.75	1	0.86215	.	.	1
13950	0	2.75	1	0.86215	.	.	1
13951	0	2.75	1	0.86215	.	.	1
13952	0	2.75	1	0.86215	.	.	1
13953	0	2.75	1	0.86215	.	.	1
13954	0	2.75	1	0.86215	.	.	1
13955	0	2.75	1	0.86215	.	.	1
13956	0	2.75	1	0.86215	.	.	1
13957	0	2.75	1	0.86215	.	.	1
13958	0	2.75	1	0.86215	.	.	1
13959	0	2.75	1	0.86215	.	.	1
13960	0	2.75	1	0.86215	.	.	1
13961	0	2.75	1	0.86215	.	.	1
13962	0	2.75	1	0.86215	.	.	1
13963	0	2.76	0	0.86199	0.85861	0.86529	1
13964	0	2.76	1	0.86199	.	.	1
13965	0	2.76	1	0.86199	.	.	1

Obs	Sub_group	fuptimey	_CENSOR_	SURVIVAL	SDF_LCL	SDF_UCL	STRATUM
**13966**	0	2.76	1	0.86199	.	.	1
**13967**	0	2.76	1	0.86199	.	.	1
**13968**	0	2.76	1	0.86199	.	.	1
**13969**	0	2.76	1	0.86199	.	.	1
**13970**	0	2.76	1	0.86199	.	.	1
**13971**	0	2.76	1	0.86199	.	.	1
**13972**	0	2.76	1	0.86199	.	.	1
**13973**	0	2.76	1	0.86199	.	.	1
**13974**	0	2.76	1	0.86199	.	.	1
**13975**	0	2.76	1	0.86199	.	.	1
**13976**	0	2.76	1	0.86199	.	.	1
**13977**	0	2.76	1	0.86199	.	.	1
**13978**	0	2.76	1	0.86199	.	.	1
**13979**	0	2.76	1	0.86199	.	.	1
**13980**	0	2.76	1	0.86199	.	.	1
**13981**	0	2.76	1	0.86199	.	.	1
**13982**	0	2.76	1	0.86199	.	.	1
**13983**	0	2.76	1	0.86199	.	.	1
**13984**	0	2.76	1	0.86199	.	.	1
**13985**	0	2.76	1	0.86199	.	.	1
**13986**	0	2.76	1	0.86199	.	.	1
**13987**	0	2.76	1	0.86199	.	.	1
**13988**	0	2.76	1	0.86199	.	.	1
**13989**	0	2.76	1	0.86199	.	.	1
**13990**	0	2.76	1	0.86199	.	.	1
**13991**	0	2.77	0	0.86183	0.85845	0.86514	1
**13992**	0	2.77	1	0.86183	.	.	1
**13993**	0	2.77	1	0.86183	.	.	1
**13994**	0	2.77	1	0.86183	.	.	1
**13995**	0	2.77	1	0.86183	.	.	1
**13996**	0	2.77	1	0.86183	.	.	1
**13997**	0	2.77	1	0.86183	.	.	1
**13998**	0	2.77	1	0.86183	.	.	1
**13999**	0	2.77	1	0.86183	.	.	1
**14000**	0	2.77	1	0.86183	.	.	1

Obs	Sub_group	fuptimey	_CENSOR_	SURVIVAL	SDF_LCL	SDF_UCL	STRATUM
14001	0	2.77	1	0.86183	.	.	1
14002	0	2.77	1	0.86183	.	.	1
14003	0	2.77	1	0.86183	.	.	1
14004	0	2.77	1	0.86183	.	.	1
14005	0	2.77	1	0.86183	.	.	1
14006	0	2.77	1	0.86183	.	.	1
14007	0	2.77	1	0.86183	.	.	1
14008	0	2.77	1	0.86183	.	.	1
14009	0	2.77	1	0.86183	.	.	1
14010	0	2.77	1	0.86183	.	.	1
14011	0	2.77	1	0.86183	.	.	1
14012	0	2.77	1	0.86183	.	.	1
14013	0	2.77	1	0.86183	.	.	1
14014	0	2.77	1	0.86183	.	.	1
14015	0	2.77	1	0.86183	.	.	1
14016	0	2.77	1	0.86183	.	.	1
14017	0	2.77	1	0.86183	.	.	1
14018	0	2.77	1	0.86183	.	.	1
14019	0	2.77	1	0.86183	.	.	1
14020	0	2.77	1	0.86183	.	.	1
14021	0	2.77	1	0.86183	.	.	1
14022	0	2.77	1	0.86183	.	.	1
14023	0	2.77	1	0.86183	.	.	1
14024	0	2.77	1	0.86183	.	.	1
14025	0	2.77	1	0.86183	.	.	1
14026	0	2.77	1	0.86183	.	.	1
14027	0	2.77	1	0.86183	.	.	1
14028	0	2.77	1	0.86183	.	.	1
14029	0	2.77	1	0.86183	.	.	1
14030	0	2.77	1	0.86183	.	.	1
14031	0	2.77	1	0.86183	.	.	1
14032	0	2.78	0	0.86157	0.85819	0.86489	1
14033	0	2.78	1	0.86157	.	.	1
14034	0	2.78	1	0.86157	.	.	1
14035	0	2.78	1	0.86157	.	.	1

Obs	Sub_group	fuptimey	_CENSOR_	SURVIVAL	SDF_LCL	SDF_UCL	STRATUM
14036	0	2.78	1	0.86157	.	.	1
14037	0	2.78	1	0.86157	.	.	1
14038	0	2.78	1	0.86157	.	.	1
14039	0	2.78	1	0.86157	.	.	1
14040	0	2.78	1	0.86157	.	.	1
14041	0	2.78	1	0.86157	.	.	1
14042	0	2.78	1	0.86157	.	.	1
14043	0	2.78	1	0.86157	.	.	1
14044	0	2.78	1	0.86157	.	.	1
14045	0	2.78	1	0.86157	.	.	1
14046	0	2.78	1	0.86157	.	.	1
14047	0	2.78	1	0.86157	.	.	1
14048	0	2.78	1	0.86157	.	.	1
14049	0	2.78	1	0.86157	.	.	1
14050	0	2.78	1	0.86157	.	.	1
14051	0	2.78	1	0.86157	.	.	1
14052	0	2.78	1	0.86157	.	.	1
14053	0	2.78	1	0.86157	.	.	1
14054	0	2.78	1	0.86157	.	.	1
14055	0	2.78	1	0.86157	.	.	1
14056	0	2.78	1	0.86157	.	.	1
14057	0	2.78	1	0.86157	.	.	1
14058	0	2.78	1	0.86157	.	.	1
14059	0	2.78	1	0.86157	.	.	1
14060	0	2.78	1	0.86157	.	.	1
14061	0	2.78	1	0.86157	.	.	1
14062	0	2.78	1	0.86157	.	.	1
14063	0	2.78	1	0.86157	.	.	1
14064	0	2.78	1	0.86157	.	.	1
14065	0	2.78	1	0.86157	.	.	1
14066	0	2.78	1	0.86157	.	.	1
14067	0	2.78	1	0.86157	.	.	1
14068	0	2.78	1	0.86157	.	.	1
14069	0	2.78	1	0.86157	.	.	1
14070	0	2.78	1	0.86157	.	.	1

Obs	Sub_group	fuptimey	_CENSOR_	SURVIVAL	SDF_LCL	SDF_UCL	STRATUM
**14071**	0	2.78	1	0.86157	.	.	1
**14072**	0	2.79	0	0.86122	0.85783	0.86454	1
**14073**	0	2.79	1	0.86122	.	.	1
**14074**	0	2.79	1	0.86122	.	.	1
**14075**	0	2.79	1	0.86122	.	.	1
**14076**	0	2.79	1	0.86122	.	.	1
**14077**	0	2.79	1	0.86122	.	.	1
**14078**	0	2.79	1	0.86122	.	.	1
**14079**	0	2.79	1	0.86122	.	.	1
**14080**	0	2.79	1	0.86122	.	.	1
**14081**	0	2.79	1	0.86122	.	.	1
**14082**	0	2.79	1	0.86122	.	.	1
**14083**	0	2.79	1	0.86122	.	.	1
**14084**	0	2.79	1	0.86122	.	.	1
**14085**	0	2.79	1	0.86122	.	.	1
**14086**	0	2.79	1	0.86122	.	.	1
**14087**	0	2.79	1	0.86122	.	.	1
**14088**	0	2.79	1	0.86122	.	.	1
**14089**	0	2.79	1	0.86122	.	.	1
**14090**	0	2.79	1	0.86122	.	.	1
**14091**	0	2.79	1	0.86122	.	.	1
**14092**	0	2.79	1	0.86122	.	.	1
**14093**	0	2.79	1	0.86122	.	.	1
**14094**	0	2.79	1	0.86122	.	.	1
**14095**	0	2.79	1	0.86122	.	.	1
**14096**	0	2.79	1	0.86122	.	.	1
**14097**	0	2.79	1	0.86122	.	.	1
**14098**	0	2.79	1	0.86122	.	.	1
**14099**	0	2.79	1	0.86122	.	.	1
**14100**	0	2.79	1	0.86122	.	.	1
**14101**	0	2.79	1	0.86122	.	.	1
**14102**	0	2.79	1	0.86122	.	.	1
**14103**	0	2.79	1	0.86122	.	.	1
**14104**	0	2.79	1	0.86122	.	.	1
**14105**	0	2.79	1	0.86122	.	.	1

Obs	Sub_group	fuptimey	_CENSOR_	SURVIVAL	SDF_LCL	SDF_UCL	STRATUM
**14106**	0	2.79	1	0.86122	.	.	1
**14107**	0	2.79	1	0.86122	.	.	1
**14108**	0	2.79	1	0.86122	.	.	1
**14109**	0	2.79	1	0.86122	.	.	1
**14110**	0	2.79	1	0.86122	.	.	1
**14111**	0	2.79	1	0.86122	.	.	1
**14112**	0	2.79	1	0.86122	.	.	1
**14113**	0	2.79	1	0.86122	.	.	1
**14114**	0	2.79	1	0.86122	.	.	1
**14115**	0	2.79	1	0.86122	.	.	1
**14116**	0	2.79	1	0.86122	.	.	1
**14117**	0	2.79	1	0.86122	.	.	1
**14118**	0	2.79	1	0.86122	.	.	1
**14119**	0	2.79	1	0.86122	.	.	1
**14120**	0	2.79	1	0.86122	.	.	1
**14121**	0	2.80	0	0.86097	0.85757	0.86429	1
**14122**	0	2.80	1	0.86097	.	.	1
**14123**	0	2.80	1	0.86097	.	.	1
**14124**	0	2.80	1	0.86097	.	.	1
**14125**	0	2.80	1	0.86097	.	.	1
**14126**	0	2.80	1	0.86097	.	.	1
**14127**	0	2.80	1	0.86097	.	.	1
**14128**	0	2.80	1	0.86097	.	.	1
**14129**	0	2.80	1	0.86097	.	.	1
**14130**	0	2.80	1	0.86097	.	.	1
**14131**	0	2.80	1	0.86097	.	.	1
**14132**	0	2.80	1	0.86097	.	.	1
**14133**	0	2.80	1	0.86097	.	.	1
**14134**	0	2.80	1	0.86097	.	.	1
**14135**	0	2.80	1	0.86097	.	.	1
**14136**	0	2.80	1	0.86097	.	.	1
**14137**	0	2.80	1	0.86097	.	.	1
**14138**	0	2.80	1	0.86097	.	.	1
**14139**	0	2.80	1	0.86097	.	.	1
**14140**	0	2.80	1	0.86097	.	.	1

Obs	Sub_group	fuptimey	_CENSOR_	SURVIVAL	SDF_LCL	SDF_UCL	STRATUM
14141	0	2.80	1	0.86097	.	.	1
14142	0	2.80	1	0.86097	.	.	1
14143	0	2.80	1	0.86097	.	.	1
14144	0	2.80	1	0.86097	.	.	1
14145	0	2.80	1	0.86097	.	.	1
14146	0	2.80	1	0.86097	.	.	1
14147	0	2.80	1	0.86097	.	.	1
14148	0	2.80	1	0.86097	.	.	1
14149	0	2.80	1	0.86097	.	.	1
14150	0	2.81	0	0.86075	0.85735	0.86407	1
14151	0	2.81	1	0.86075	.	.	1
14152	0	2.81	1	0.86075	.	.	1
14153	0	2.81	1	0.86075	.	.	1
14154	0	2.81	1	0.86075	.	.	1
14155	0	2.81	1	0.86075	.	.	1
14156	0	2.81	1	0.86075	.	.	1
14157	0	2.81	1	0.86075	.	.	1
14158	0	2.81	1	0.86075	.	.	1
14159	0	2.81	1	0.86075	.	.	1
14160	0	2.81	1	0.86075	.	.	1
14161	0	2.81	1	0.86075	.	.	1
14162	0	2.81	1	0.86075	.	.	1
14163	0	2.81	1	0.86075	.	.	1
14164	0	2.81	1	0.86075	.	.	1
14165	0	2.81	1	0.86075	.	.	1
14166	0	2.81	1	0.86075	.	.	1
14167	0	2.81	1	0.86075	.	.	1
14168	0	2.81	1	0.86075	.	.	1
14169	0	2.81	1	0.86075	.	.	1
14170	0	2.81	1	0.86075	.	.	1
14171	0	2.81	1	0.86075	.	.	1
14172	0	2.81	1	0.86075	.	.	1
14173	0	2.81	1	0.86075	.	.	1
14174	0	2.81	1	0.86075	.	.	1
14175	0	2.81	1	0.86075	.	.	1

Obs	Sub_group	fuptimey	_CENSOR_	SURVIVAL	SDF_LCL	SDF_UCL	STRATUM
14176	0	2.81	1	0.86075	.	.	1
14177	0	2.81	1	0.86075	.	.	1
14178	0	2.81	1	0.86075	.	.	1
14179	0	2.81	1	0.86075	.	.	1
14180	0	2.81	1	0.86075	.	.	1
14181	0	2.81	1	0.86075	.	.	1
14182	0	2.81	1	0.86075	.	.	1
14183	0	2.81	1	0.86075	.	.	1
14184	0	2.81	1	0.86075	.	.	1
14185	0	2.81	1	0.86075	.	.	1
14186	0	2.82	0	0.86052	0.85712	0.86385	1
14187	0	2.82	1	0.86052	.	.	1
14188	0	2.82	1	0.86052	.	.	1
14189	0	2.82	1	0.86052	.	.	1
14190	0	2.82	1	0.86052	.	.	1
14191	0	2.82	1	0.86052	.	.	1
14192	0	2.82	1	0.86052	.	.	1
14193	0	2.82	1	0.86052	.	.	1
14194	0	2.82	1	0.86052	.	.	1
14195	0	2.82	1	0.86052	.	.	1
14196	0	2.82	1	0.86052	.	.	1
14197	0	2.82	1	0.86052	.	.	1
14198	0	2.82	1	0.86052	.	.	1
14199	0	2.82	1	0.86052	.	.	1
14200	0	2.82	1	0.86052	.	.	1
14201	0	2.82	1	0.86052	.	.	1
14202	0	2.82	1	0.86052	.	.	1
14203	0	2.82	1	0.86052	.	.	1
14204	0	2.82	1	0.86052	.	.	1
14205	0	2.82	1	0.86052	.	.	1
14206	0	2.82	1	0.86052	.	.	1
14207	0	2.82	1	0.86052	.	.	1
14208	0	2.82	1	0.86052	.	.	1
14209	0	2.82	1	0.86052	.	.	1
14210	0	2.82	1	0.86052	.	.	1

Obs	Sub_group	fuptimey	_CENSOR_	SURVIVAL	SDF_LCL	SDF_UCL	STRATUM
**14211**	0	2.82	1	0.86052	.	.	1
**14212**	0	2.82	1	0.86052	.	.	1
**14213**	0	2.82	1	0.86052	.	.	1
**14214**	0	2.82	1	0.86052	.	.	1
**14215**	0	2.82	1	0.86052	.	.	1
**14216**	0	2.82	1	0.86052	.	.	1
**14217**	0	2.82	1	0.86052	.	.	1
**14218**	0	2.82	1	0.86052	.	.	1
**14219**	0	2.82	1	0.86052	.	.	1
**14220**	0	2.82	1	0.86052	.	.	1
**14221**	0	2.82	1	0.86052	.	.	1
**14222**	0	2.82	1	0.86052	.	.	1
**14223**	0	2.82	1	0.86052	.	.	1
**14224**	0	2.82	1	0.86052	.	.	1
**14225**	0	2.82	1	0.86052	.	.	1
**14226**	0	2.82	1	0.86052	.	.	1
**14227**	0	2.82	1	0.86052	.	.	1
**14228**	0	2.82	1	0.86052	.	.	1
**14229**	0	2.82	1	0.86052	.	.	1
**14230**	0	2.82	1	0.86052	.	.	1
**14231**	0	2.82	1	0.86052	.	.	1
**14232**	0	2.82	1	0.86052	.	.	1
**14233**	0	2.83	0	0.86023	0.85683	0.86357	1
**14234**	0	2.83	1	0.86023	.	.	1
**14235**	0	2.83	1	0.86023	.	.	1
**14236**	0	2.83	1	0.86023	.	.	1
**14237**	0	2.83	1	0.86023	.	.	1
**14238**	0	2.83	1	0.86023	.	.	1
**14239**	0	2.83	1	0.86023	.	.	1
**14240**	0	2.83	1	0.86023	.	.	1
**14241**	0	2.83	1	0.86023	.	.	1
**14242**	0	2.83	1	0.86023	.	.	1
**14243**	0	2.83	1	0.86023	.	.	1
**14244**	0	2.83	1	0.86023	.	.	1
**14245**	0	2.83	1	0.86023	.	.	1

Obs	Sub_group	fuptimey	_CENSOR_	SURVIVAL	SDF_LCL	SDF_UCL	STRATUM
14246	0	2.83	1	0.86023	.	.	1
14247	0	2.83	1	0.86023	.	.	1
14248	0	2.83	1	0.86023	.	.	1
14249	0	2.83	1	0.86023	.	.	1
14250	0	2.83	1	0.86023	.	.	1
14251	0	2.83	1	0.86023	.	.	1
14252	0	2.83	1	0.86023	.	.	1
14253	0	2.83	1	0.86023	.	.	1
14254	0	2.83	1	0.86023	.	.	1
14255	0	2.83	1	0.86023	.	.	1
14256	0	2.83	1	0.86023	.	.	1
14257	0	2.83	1	0.86023	.	.	1
14258	0	2.83	1	0.86023	.	.	1
14259	0	2.83	1	0.86023	.	.	1
14260	0	2.83	1	0.86023	.	.	1
14261	0	2.83	1	0.86023	.	.	1
14262	0	2.83	1	0.86023	.	.	1
14263	0	2.83	1	0.86023	.	.	1
14264	0	2.83	1	0.86023	.	.	1
14265	0	2.83	1	0.86023	.	.	1
14266	0	2.84	0	0.85988	0.85647	0.86322	1
14267	0	2.84	1	0.85988	.	.	1
14268	0	2.84	1	0.85988	.	.	1
14269	0	2.84	1	0.85988	.	.	1
14270	0	2.84	1	0.85988	.	.	1
14271	0	2.84	1	0.85988	.	.	1
14272	0	2.84	1	0.85988	.	.	1
14273	0	2.84	1	0.85988	.	.	1
14274	0	2.84	1	0.85988	.	.	1
14275	0	2.84	1	0.85988	.	.	1
14276	0	2.84	1	0.85988	.	.	1
14277	0	2.84	1	0.85988	.	.	1
14278	0	2.84	1	0.85988	.	.	1
14279	0	2.84	1	0.85988	.	.	1
14280	0	2.84	1	0.85988	.	.	1

Obs	Sub_group	fuptimey	_CENSOR_	SURVIVAL	SDF_LCL	SDF_UCL	STRATUM
**14281**	0	2.84	1	0.85988	.	.	1
**14282**	0	2.84	1	0.85988	.	.	1
**14283**	0	2.84	1	0.85988	.	.	1
**14284**	0	2.84	1	0.85988	.	.	1
**14285**	0	2.84	1	0.85988	.	.	1
**14286**	0	2.84	1	0.85988	.	.	1
**14287**	0	2.84	1	0.85988	.	.	1
**14288**	0	2.84	1	0.85988	.	.	1
**14289**	0	2.84	1	0.85988	.	.	1
**14290**	0	2.84	1	0.85988	.	.	1
**14291**	0	2.84	1	0.85988	.	.	1
**14292**	0	2.84	1	0.85988	.	.	1
**14293**	0	2.84	1	0.85988	.	.	1
**14294**	0	2.84	1	0.85988	.	.	1
**14295**	0	2.84	1	0.85988	.	.	1
**14296**	0	2.84	1	0.85988	.	.	1
**14297**	0	2.84	1	0.85988	.	.	1
**14298**	0	2.84	1	0.85988	.	.	1
**14299**	0	2.84	1	0.85988	.	.	1
**14300**	0	2.84	1	0.85988	.	.	1
**14301**	0	2.84	1	0.85988	.	.	1
**14302**	0	2.84	1	0.85988	.	.	1
**14303**	0	2.84	1	0.85988	.	.	1
**14304**	0	2.84	1	0.85988	.	.	1
**14305**	0	2.84	1	0.85988	.	.	1
**14306**	0	2.84	1	0.85988	.	.	1
**14307**	0	2.84	1	0.85988	.	.	1
**14308**	0	2.84	1	0.85988	.	.	1
**14309**	0	2.84	1	0.85988	.	.	1
**14310**	0	2.85	0	0.85953	0.85611	0.86287	1
**14311**	0	2.85	1	0.85953	.	.	1
**14312**	0	2.85	1	0.85953	.	.	1
**14313**	0	2.85	1	0.85953	.	.	1
**14314**	0	2.85	1	0.85953	.	.	1
**14315**	0	2.85	1	0.85953	.	.	1

Obs	Sub_group	fuptimey	_CENSOR_	SURVIVAL	SDF_LCL	SDF_UCL	STRATUM
14316	0	2.85	1	0.85953	.	.	1
14317	0	2.85	1	0.85953	.	.	1
14318	0	2.85	1	0.85953	.	.	1
14319	0	2.85	1	0.85953	.	.	1
14320	0	2.85	1	0.85953	.	.	1
14321	0	2.85	1	0.85953	.	.	1
14322	0	2.85	1	0.85953	.	.	1
14323	0	2.85	1	0.85953	.	.	1
14324	0	2.85	1	0.85953	.	.	1
14325	0	2.85	1	0.85953	.	.	1
14326	0	2.85	1	0.85953	.	.	1
14327	0	2.85	1	0.85953	.	.	1
14328	0	2.85	1	0.85953	.	.	1
14329	0	2.85	1	0.85953	.	.	1
14330	0	2.85	1	0.85953	.	.	1
14331	0	2.85	1	0.85953	.	.	1
14332	0	2.85	1	0.85953	.	.	1
14333	0	2.85	1	0.85953	.	.	1
14334	0	2.85	1	0.85953	.	.	1
14335	0	2.85	1	0.85953	.	.	1
14336	0	2.85	1	0.85953	.	.	1
14337	0	2.85	1	0.85953	.	.	1
14338	0	2.85	1	0.85953	.	.	1
14339	0	2.85	1	0.85953	.	.	1
14340	0	2.85	1	0.85953	.	.	1
14341	0	2.85	1	0.85953	.	.	1
14342	0	2.85	1	0.85953	.	.	1
14343	0	2.85	1	0.85953	.	.	1
14344	0	2.85	1	0.85953	.	.	1
14345	0	2.85	1	0.85953	.	.	1
14346	0	2.85	1	0.85953	.	.	1
14347	0	2.85	1	0.85953	.	.	1
14348	0	2.85	1	0.85953	.	.	1
14349	0	2.85	1	0.85953	.	.	1
14350	0	2.85	1	0.85953	.	.	1

Obs	Sub_group	fuptimey	_CENSOR_	SURVIVAL	SDF_LCL	SDF_UCL	STRATUM
14351	0	2.86	0	0.85921	0.85579	0.86255	1
14352	0	2.86	1	0.85921	.	.	1
14353	0	2.86	1	0.85921	.	.	1
14354	0	2.86	1	0.85921	.	.	1
14355	0	2.86	1	0.85921	.	.	1
14356	0	2.86	1	0.85921	.	.	1
14357	0	2.86	1	0.85921	.	.	1
14358	0	2.86	1	0.85921	.	.	1
14359	0	2.86	1	0.85921	.	.	1
14360	0	2.86	1	0.85921	.	.	1
14361	0	2.86	1	0.85921	.	.	1
14362	0	2.86	1	0.85921	.	.	1
14363	0	2.86	1	0.85921	.	.	1
14364	0	2.86	1	0.85921	.	.	1
14365	0	2.86	1	0.85921	.	.	1
14366	0	2.86	1	0.85921	.	.	1
14367	0	2.86	1	0.85921	.	.	1
14368	0	2.86	1	0.85921	.	.	1
14369	0	2.86	1	0.85921	.	.	1
14370	0	2.86	1	0.85921	.	.	1
14371	0	2.86	1	0.85921	.	.	1
14372	0	2.86	1	0.85921	.	.	1
14373	0	2.86	1	0.85921	.	.	1
14374	0	2.86	1	0.85921	.	.	1
14375	0	2.86	1	0.85921	.	.	1
14376	0	2.86	1	0.85921	.	.	1
14377	0	2.86	1	0.85921	.	.	1
14378	0	2.86	1	0.85921	.	.	1
14379	0	2.86	1	0.85921	.	.	1
14380	0	2.86	1	0.85921	.	.	1
14381	0	2.86	1	0.85921	.	.	1
14382	0	2.86	1	0.85921	.	.	1
14383	0	2.86	1	0.85921	.	.	1
14384	0	2.86	1	0.85921	.	.	1
14385	0	2.86	1	0.85921	.	.	1

Obs	Sub_group	fuptimey	_CENSOR_	SURVIVAL	SDF_LCL	SDF_UCL	STRATUM
**14386**	0	2.86	1	0.85921	.	.	1
**14387**	0	2.86	1	0.85921	.	.	1
**14388**	0	2.86	1	0.85921	.	.	1
**14389**	0	2.86	1	0.85921	.	.	1
**14390**	0	2.86	1	0.85921	.	.	1
**14391**	0	2.86	1	0.85921	.	.	1
**14392**	0	2.87	0	0.85901	0.85559	0.86236	1
**14393**	0	2.87	1	0.85901	.	.	1
**14394**	0	2.87	1	0.85901	.	.	1
**14395**	0	2.87	1	0.85901	.	.	1
**14396**	0	2.87	1	0.85901	.	.	1
**14397**	0	2.87	1	0.85901	.	.	1
**14398**	0	2.87	1	0.85901	.	.	1
**14399**	0	2.87	1	0.85901	.	.	1
**14400**	0	2.87	1	0.85901	.	.	1
**14401**	0	2.87	1	0.85901	.	.	1
**14402**	0	2.87	1	0.85901	.	.	1
**14403**	0	2.87	1	0.85901	.	.	1
**14404**	0	2.87	1	0.85901	.	.	1
**14405**	0	2.87	1	0.85901	.	.	1
**14406**	0	2.87	1	0.85901	.	.	1
**14407**	0	2.87	1	0.85901	.	.	1
**14408**	0	2.87	1	0.85901	.	.	1
**14409**	0	2.87	1	0.85901	.	.	1
**14410**	0	2.87	1	0.85901	.	.	1
**14411**	0	2.87	1	0.85901	.	.	1
**14412**	0	2.87	1	0.85901	.	.	1
**14413**	0	2.87	1	0.85901	.	.	1
**14414**	0	2.87	1	0.85901	.	.	1
**14415**	0	2.87	1	0.85901	.	.	1
**14416**	0	2.87	1	0.85901	.	.	1
**14417**	0	2.87	1	0.85901	.	.	1
**14418**	0	2.87	1	0.85901	.	.	1
**14419**	0	2.87	1	0.85901	.	.	1
**14420**	0	2.87	1	0.85901	.	.	1

Obs	Sub_group	fuptimey	_CENSOR_	SURVIVAL	SDF_LCL	SDF_UCL	STRATUM
**14421**	0	2.88	0	0.85879	0.85536	0.86214	1
**14422**	0	2.88	1	0.85879	.	.	1
**14423**	0	2.88	1	0.85879	.	.	1
**14424**	0	2.88	1	0.85879	.	.	1
**14425**	0	2.88	1	0.85879	.	.	1
**14426**	0	2.88	1	0.85879	.	.	1
**14427**	0	2.88	1	0.85879	.	.	1
**14428**	0	2.88	1	0.85879	.	.	1
**14429**	0	2.88	1	0.85879	.	.	1
**14430**	0	2.88	1	0.85879	.	.	1
**14431**	0	2.88	1	0.85879	.	.	1
**14432**	0	2.88	1	0.85879	.	.	1
**14433**	0	2.88	1	0.85879	.	.	1
**14434**	0	2.88	1	0.85879	.	.	1
**14435**	0	2.88	1	0.85879	.	.	1
**14436**	0	2.88	1	0.85879	.	.	1
**14437**	0	2.88	1	0.85879	.	.	1
**14438**	0	2.88	1	0.85879	.	.	1
**14439**	0	2.88	1	0.85879	.	.	1
**14440**	0	2.88	1	0.85879	.	.	1
**14441**	0	2.88	1	0.85879	.	.	1
**14442**	0	2.88	1	0.85879	.	.	1
**14443**	0	2.88	1	0.85879	.	.	1
**14444**	0	2.88	1	0.85879	.	.	1
**14445**	0	2.88	1	0.85879	.	.	1
**14446**	0	2.88	1	0.85879	.	.	1
**14447**	0	2.88	1	0.85879	.	.	1
**14448**	0	2.88	1	0.85879	.	.	1
**14449**	0	2.88	1	0.85879	.	.	1
**14450**	0	2.88	1	0.85879	.	.	1
**14451**	0	2.88	1	0.85879	.	.	1
**14452**	0	2.88	1	0.85879	.	.	1
**14453**	0	2.88	1	0.85879	.	.	1
**14454**	0	2.88	1	0.85879	.	.	1
**14455**	0	2.88	1	0.85879	.	.	1

Obs	Sub_group	fuptimey	_CENSOR_	SURVIVAL	SDF_LCL	SDF_UCL	STRATUM
14456	0	2.88	1	0.85879	.	.	1
14457	0	2.88	1	0.85879	.	.	1
14458	0	2.88	1	0.85879	.	.	1
14459	0	2.88	1	0.85879	.	.	1
14460	0	2.88	1	0.85879	.	.	1
14461	0	2.88	1	0.85879	.	.	1
14462	0	2.88	1	0.85879	.	.	1
14463	0	2.88	1	0.85879	.	.	1
14464	0	2.88	1	0.85879	.	.	1
14465	0	2.88	1	0.85879	.	.	1
14466	0	2.88	1	0.85879	.	.	1
14467	0	2.88	1	0.85879	.	.	1
14468	0	2.88	1	0.85879	.	.	1
14469	0	2.88	1	0.85879	.	.	1
14470	0	2.88	1	0.85879	.	.	1
14471	0	2.89	0	0.85866	0.85523	0.86201	1
14472	0	2.89	1	0.85866	.	.	1
14473	0	2.89	1	0.85866	.	.	1
14474	0	2.89	1	0.85866	.	.	1
14475	0	2.89	1	0.85866	.	.	1
14476	0	2.89	1	0.85866	.	.	1
14477	0	2.89	1	0.85866	.	.	1
14478	0	2.89	1	0.85866	.	.	1
14479	0	2.89	1	0.85866	.	.	1
14480	0	2.89	1	0.85866	.	.	1
14481	0	2.89	1	0.85866	.	.	1
14482	0	2.89	1	0.85866	.	.	1
14483	0	2.89	1	0.85866	.	.	1
14484	0	2.89	1	0.85866	.	.	1
14485	0	2.89	1	0.85866	.	.	1
14486	0	2.89	1	0.85866	.	.	1
14487	0	2.89	1	0.85866	.	.	1
14488	0	2.89	1	0.85866	.	.	1
14489	0	2.89	1	0.85866	.	.	1
14490	0	2.89	1	0.85866	.	.	1

Obs	Sub_group	fuptimey	_CENSOR_	SURVIVAL	SDF_LCL	SDF_UCL	STRATUM
14491	0	2.89	1	0.85866	.	.	1
14492	0	2.89	1	0.85866	.	.	1
14493	0	2.89	1	0.85866	.	.	1
14494	0	2.89	1	0.85866	.	.	1
14495	0	2.89	1	0.85866	.	.	1
14496	0	2.89	1	0.85866	.	.	1
14497	0	2.89	1	0.85866	.	.	1
14498	0	2.89	1	0.85866	.	.	1
14499	0	2.89	1	0.85866	.	.	1
14500	0	2.89	1	0.85866	.	.	1
14501	0	2.89	1	0.85866	.	.	1
14502	0	2.89	1	0.85866	.	.	1
14503	0	2.89	1	0.85866	.	.	1
14504	0	2.89	1	0.85866	.	.	1
14505	0	2.89	1	0.85866	.	.	1
14506	0	2.89	1	0.85866	.	.	1
14507	0	2.89	1	0.85866	.	.	1
14508	0	2.89	1	0.85866	.	.	1
14509	0	2.89	1	0.85866	.	.	1
14510	0	2.90	0	0.85843	0.85500	0.86179	1
14511	0	2.90	1	0.85843	.	.	1
14512	0	2.90	1	0.85843	.	.	1
14513	0	2.90	1	0.85843	.	.	1
14514	0	2.90	1	0.85843	.	.	1
14515	0	2.90	1	0.85843	.	.	1
14516	0	2.90	1	0.85843	.	.	1
14517	0	2.90	1	0.85843	.	.	1
14518	0	2.90	1	0.85843	.	.	1
14519	0	2.90	1	0.85843	.	.	1
14520	0	2.90	1	0.85843	.	.	1
14521	0	2.90	1	0.85843	.	.	1
14522	0	2.90	1	0.85843	.	.	1
14523	0	2.90	1	0.85843	.	.	1
14524	0	2.90	1	0.85843	.	.	1
14525	0	2.90	1	0.85843	.	.	1

Obs	Sub_group	fuptimey	_CENSOR_	SURVIVAL	SDF_LCL	SDF_UCL	STRATUM
14526	0	2.90	1	0.85843	.	.	1
14527	0	2.90	1	0.85843	.	.	1
14528	0	2.90	1	0.85843	.	.	1
14529	0	2.90	1	0.85843	.	.	1
14530	0	2.90	1	0.85843	.	.	1
14531	0	2.90	1	0.85843	.	.	1
14532	0	2.90	1	0.85843	.	.	1
14533	0	2.90	1	0.85843	.	.	1
14534	0	2.90	1	0.85843	.	.	1
14535	0	2.90	1	0.85843	.	.	1
14536	0	2.90	1	0.85843	.	.	1
14537	0	2.90	1	0.85843	.	.	1
14538	0	2.90	1	0.85843	.	.	1
14539	0	2.90	1	0.85843	.	.	1
14540	0	2.90	1	0.85843	.	.	1
14541	0	2.90	1	0.85843	.	.	1
14542	0	2.90	1	0.85843	.	.	1
14543	0	2.90	1	0.85843	.	.	1
14544	0	2.90	1	0.85843	.	.	1
14545	0	2.90	1	0.85843	.	.	1
14546	0	2.90	1	0.85843	.	.	1
14547	0	2.90	1	0.85843	.	.	1
14548	0	2.90	1	0.85843	.	.	1
14549	0	2.90	1	0.85843	.	.	1
14550	0	2.90	1	0.85843	.	.	1
14551	0	2.91	0	0.85808	0.85464	0.86144	1
14552	0	2.91	1	0.85808	.	.	1
14553	0	2.91	1	0.85808	.	.	1
14554	0	2.91	1	0.85808	.	.	1
14555	0	2.91	1	0.85808	.	.	1
14556	0	2.91	1	0.85808	.	.	1
14557	0	2.91	1	0.85808	.	.	1
14558	0	2.91	1	0.85808	.	.	1
14559	0	2.91	1	0.85808	.	.	1
14560	0	2.91	1	0.85808	.	.	1

Obs	Sub_group	fuptimey	_CENSOR_	SURVIVAL	SDF_LCL	SDF_UCL	STRATUM
14561	0	2.91	1	0.85808	.	.	1
14562	0	2.91	1	0.85808	.	.	1
14563	0	2.91	1	0.85808	.	.	1
14564	0	2.91	1	0.85808	.	.	1
14565	0	2.91	1	0.85808	.	.	1
14566	0	2.91	1	0.85808	.	.	1
14567	0	2.91	1	0.85808	.	.	1
14568	0	2.91	1	0.85808	.	.	1
14569	0	2.91	1	0.85808	.	.	1
14570	0	2.91	1	0.85808	.	.	1
14571	0	2.91	1	0.85808	.	.	1
14572	0	2.91	1	0.85808	.	.	1
14573	0	2.91	1	0.85808	.	.	1
14574	0	2.91	1	0.85808	.	.	1
14575	0	2.91	1	0.85808	.	.	1
14576	0	2.91	1	0.85808	.	.	1
14577	0	2.91	1	0.85808	.	.	1
14578	0	2.91	1	0.85808	.	.	1
14579	0	2.91	1	0.85808	.	.	1
14580	0	2.92	0	0.85779	0.85434	0.86115	1
14581	0	2.92	1	0.85779	.	.	1
14582	0	2.92	1	0.85779	.	.	1
14583	0	2.92	1	0.85779	.	.	1
14584	0	2.92	1	0.85779	.	.	1
14585	0	2.92	1	0.85779	.	.	1
14586	0	2.92	1	0.85779	.	.	1
14587	0	2.92	1	0.85779	.	.	1
14588	0	2.92	1	0.85779	.	.	1
14589	0	2.92	1	0.85779	.	.	1
14590	0	2.92	1	0.85779	.	.	1
14591	0	2.92	1	0.85779	.	.	1
14592	0	2.92	1	0.85779	.	.	1
14593	0	2.92	1	0.85779	.	.	1
14594	0	2.92	1	0.85779	.	.	1
14595	0	2.92	1	0.85779	.	.	1

Obs	Sub_group	fuptimey	_CENSOR_	SURVIVAL	SDF_LCL	SDF_UCL	STRATUM
14596	0	2.92	1	0.85779	.	.	1
14597	0	2.92	1	0.85779	.	.	1
14598	0	2.92	1	0.85779	.	.	1
14599	0	2.92	1	0.85779	.	.	1
14600	0	2.92	1	0.85779	.	.	1
14601	0	2.92	1	0.85779	.	.	1
14602	0	2.92	1	0.85779	.	.	1
14603	0	2.92	1	0.85779	.	.	1
14604	0	2.92	1	0.85779	.	.	1
14605	0	2.92	1	0.85779	.	.	1
14606	0	2.92	1	0.85779	.	.	1
14607	0	2.92	1	0.85779	.	.	1
14608	0	2.92	1	0.85779	.	.	1
14609	0	2.92	1	0.85779	.	.	1
14610	0	2.92	1	0.85779	.	.	1
14611	0	2.92	1	0.85779	.	.	1
14612	0	2.92	1	0.85779	.	.	1
14613	0	2.92	1	0.85779	.	.	1
14614	0	2.92	1	0.85779	.	.	1
14615	0	2.92	1	0.85779	.	.	1
14616	0	2.92	1	0.85779	.	.	1
14617	0	2.92	1	0.85779	.	.	1
14618	0	2.92	1	0.85779	.	.	1
14619	0	2.92	1	0.85779	.	.	1
14620	0	2.92	1	0.85779	.	.	1
14621	0	2.92	1	0.85779	.	.	1
14622	0	2.92	1	0.85779	.	.	1
14623	0	2.92	1	0.85779	.	.	1
14624	0	2.92	1	0.85779	.	.	1
14625	0	2.92	1	0.85779	.	.	1
14626	0	2.92	1	0.85779	.	.	1
14627	0	2.92	1	0.85779	.	.	1
14628	0	2.92	1	0.85779	.	.	1
14629	0	2.92	1	0.85779	.	.	1
14630	0	2.92	1	0.85779	.	.	1

Obs	Sub_group	fuptimey	_CENSOR_	SURVIVAL	SDF_LCL	SDF_UCL	STRATUM
14631	0	2.92	1	0.85779	.	.	1
14632	0	2.93	0	0.85756	0.85411	0.86093	1
14633	0	2.93	1	0.85756	.	.	1
14634	0	2.93	1	0.85756	.	.	1
14635	0	2.93	1	0.85756	.	.	1
14636	0	2.93	1	0.85756	.	.	1
14637	0	2.93	1	0.85756	.	.	1
14638	0	2.93	1	0.85756	.	.	1
14639	0	2.93	1	0.85756	.	.	1
14640	0	2.93	1	0.85756	.	.	1
14641	0	2.93	1	0.85756	.	.	1
14642	0	2.93	1	0.85756	.	.	1
14643	0	2.93	1	0.85756	.	.	1
14644	0	2.93	1	0.85756	.	.	1
14645	0	2.93	1	0.85756	.	.	1
14646	0	2.93	1	0.85756	.	.	1
14647	0	2.93	1	0.85756	.	.	1
14648	0	2.93	1	0.85756	.	.	1
14649	0	2.93	1	0.85756	.	.	1
14650	0	2.93	1	0.85756	.	.	1
14651	0	2.93	1	0.85756	.	.	1
14652	0	2.93	1	0.85756	.	.	1
14653	0	2.93	1	0.85756	.	.	1
14654	0	2.93	1	0.85756	.	.	1
14655	0	2.93	1	0.85756	.	.	1
14656	0	2.93	1	0.85756	.	.	1
14657	0	2.93	1	0.85756	.	.	1
14658	0	2.93	1	0.85756	.	.	1
14659	0	2.93	1	0.85756	.	.	1
14660	0	2.93	1	0.85756	.	.	1
14661	0	2.93	1	0.85756	.	.	1
14662	0	2.93	1	0.85756	.	.	1
14663	0	2.94	0	0.85740	0.85395	0.86077	1
14664	0	2.94	1	0.85740	.	.	1
14665	0	2.94	1	0.85740	.	.	1

Obs	Sub_group	fuptimey	_CENSOR_	SURVIVAL	SDF_LCL	SDF_UCL	STRATUM
14666	0	2.94	1	0.85740	.	.	1
14667	0	2.94	1	0.85740	.	.	1
14668	0	2.94	1	0.85740	.	.	1
14669	0	2.94	1	0.85740	.	.	1
14670	0	2.94	1	0.85740	.	.	1
14671	0	2.94	1	0.85740	.	.	1
14672	0	2.94	1	0.85740	.	.	1
14673	0	2.94	1	0.85740	.	.	1
14674	0	2.94	1	0.85740	.	.	1
14675	0	2.94	1	0.85740	.	.	1
14676	0	2.94	1	0.85740	.	.	1
14677	0	2.94	1	0.85740	.	.	1
14678	0	2.94	1	0.85740	.	.	1
14679	0	2.94	1	0.85740	.	.	1
14680	0	2.94	1	0.85740	.	.	1
14681	0	2.94	1	0.85740	.	.	1
14682	0	2.94	1	0.85740	.	.	1
14683	0	2.94	1	0.85740	.	.	1
14684	0	2.94	1	0.85740	.	.	1
14685	0	2.94	1	0.85740	.	.	1
14686	0	2.94	1	0.85740	.	.	1
14687	0	2.94	1	0.85740	.	.	1
14688	0	2.94	1	0.85740	.	.	1
14689	0	2.94	1	0.85740	.	.	1
14690	0	2.94	1	0.85740	.	.	1
14691	0	2.94	1	0.85740	.	.	1
14692	0	2.94	1	0.85740	.	.	1
14693	0	2.94	1	0.85740	.	.	1
14694	0	2.94	1	0.85740	.	.	1
14695	0	2.94	1	0.85740	.	.	1
14696	0	2.94	1	0.85740	.	.	1
14697	0	2.94	1	0.85740	.	.	1
14698	0	2.94	1	0.85740	.	.	1
14699	0	2.94	1	0.85740	.	.	1
14700	0	2.94	1	0.85740	.	.	1

Obs	Sub_group	fuptimey	_CENSOR_	SURVIVAL	SDF_LCL	SDF_UCL	STRATUM
14701	0	2.94	1	0.85740	.	.	1
14702	0	2.94	1	0.85740	.	.	1
14703	0	2.94	1	0.85740	.	.	1
14704	0	2.94	1	0.85740	.	.	1
14705	0	2.94	1	0.85740	.	.	1
14706	0	2.94	1	0.85740	.	.	1
14707	0	2.94	1	0.85740	.	.	1
14708	0	2.94	1	0.85740	.	.	1
14709	0	2.94	1	0.85740	.	.	1
14710	0	2.94	1	0.85740	.	.	1
14711	0	2.94	1	0.85740	.	.	1
14712	0	2.95	0	0.85720	0.85375	0.86058	1
14713	0	2.95	1	0.85720	.	.	1
14714	0	2.95	1	0.85720	.	.	1
14715	0	2.95	1	0.85720	.	.	1
14716	0	2.95	1	0.85720	.	.	1
14717	0	2.95	1	0.85720	.	.	1
14718	0	2.95	1	0.85720	.	.	1
14719	0	2.95	1	0.85720	.	.	1
14720	0	2.95	1	0.85720	.	.	1
14721	0	2.95	1	0.85720	.	.	1
14722	0	2.95	1	0.85720	.	.	1
14723	0	2.95	1	0.85720	.	.	1
14724	0	2.95	1	0.85720	.	.	1
14725	0	2.95	1	0.85720	.	.	1
14726	0	2.95	1	0.85720	.	.	1
14727	0	2.95	1	0.85720	.	.	1
14728	0	2.95	1	0.85720	.	.	1
14729	0	2.95	1	0.85720	.	.	1
14730	0	2.95	1	0.85720	.	.	1
14731	0	2.95	1	0.85720	.	.	1
14732	0	2.95	1	0.85720	.	.	1
14733	0	2.95	1	0.85720	.	.	1
14734	0	2.95	1	0.85720	.	.	1
14735	0	2.95	1	0.85720	.	.	1

Obs	Sub_group	fuptimey	_CENSOR_	SURVIVAL	SDF_LCL	SDF_UCL	STRATUM
14736	0	2.95	1	0.85720	.	.	1
14737	0	2.95	1	0.85720	.	.	1
14738	0	2.95	1	0.85720	.	.	1
14739	0	2.95	1	0.85720	.	.	1
14740	0	2.95	1	0.85720	.	.	1
14741	0	2.95	1	0.85720	.	.	1
14742	0	2.96	0	0.85691	0.85345	0.86029	1
14743	0	2.96	1	0.85691	.	.	1
14744	0	2.96	1	0.85691	.	.	1
14745	0	2.96	1	0.85691	.	.	1
14746	0	2.96	1	0.85691	.	.	1
14747	0	2.96	1	0.85691	.	.	1
14748	0	2.96	1	0.85691	.	.	1
14749	0	2.96	1	0.85691	.	.	1
14750	0	2.96	1	0.85691	.	.	1
14751	0	2.96	1	0.85691	.	.	1
14752	0	2.96	1	0.85691	.	.	1
14753	0	2.96	1	0.85691	.	.	1
14754	0	2.96	1	0.85691	.	.	1
14755	0	2.96	1	0.85691	.	.	1
14756	0	2.96	1	0.85691	.	.	1
14757	0	2.96	1	0.85691	.	.	1
14758	0	2.96	1	0.85691	.	.	1
14759	0	2.96	1	0.85691	.	.	1
14760	0	2.96	1	0.85691	.	.	1
14761	0	2.96	1	0.85691	.	.	1
14762	0	2.96	1	0.85691	.	.	1
14763	0	2.96	1	0.85691	.	.	1
14764	0	2.96	1	0.85691	.	.	1
14765	0	2.96	1	0.85691	.	.	1
14766	0	2.96	1	0.85691	.	.	1
14767	0	2.96	1	0.85691	.	.	1
14768	0	2.96	1	0.85691	.	.	1
14769	0	2.96	1	0.85691	.	.	1
14770	0	2.96	1	0.85691	.	.	1

Obs	Sub_group	fuptimey	_CENSOR_	SURVIVAL	SDF_LCL	SDF_UCL	STRATUM
**14771**	0	2.96	1	0.85691	.	.	1
**14772**	0	2.96	1	0.85691	.	.	1
**14773**	0	2.96	1	0.85691	.	.	1
**14774**	0	2.96	1	0.85691	.	.	1
**14775**	0	2.96	1	0.85691	.	.	1
**14776**	0	2.96	1	0.85691	.	.	1
**14777**	0	2.96	1	0.85691	.	.	1
**14778**	0	2.96	1	0.85691	.	.	1
**14779**	0	2.96	1	0.85691	.	.	1
**14780**	0	2.96	1	0.85691	.	.	1
**14781**	0	2.96	1	0.85691	.	.	1
**14782**	0	2.96	1	0.85691	.	.	1
**14783**	0	2.96	1	0.85691	.	.	1
**14784**	0	2.96	1	0.85691	.	.	1
**14785**	0	2.96	1	0.85691	.	.	1
**14786**	0	2.96	1	0.85691	.	.	1
**14787**	0	2.96	1	0.85691	.	.	1
**14788**	0	2.96	1	0.85691	.	.	1
**14789**	0	2.96	1	0.85691	.	.	1
**14790**	0	2.96	1	0.85691	.	.	1
**14791**	0	2.96	1	0.85691	.	.	1
**14792**	0	2.96	1	0.85691	.	.	1
**14793**	0	2.96	1	0.85691	.	.	1
**14794**	0	2.96	1	0.85691	.	.	1
**14795**	0	2.96	1	0.85691	.	.	1
**14796**	0	2.96	1	0.85691	.	.	1
**14797**	0	2.96	1	0.85691	.	.	1
**14798**	0	2.96	1	0.85691	.	.	1
**14799**	0	2.96	1	0.85691	.	.	1
**14800**	0	2.96	1	0.85691	.	.	1
**14801**	0	2.96	1	0.85691	.	.	1
**14802**	0	2.97	0	0.85668	0.85322	0.86006	1
**14803**	0	2.97	1	0.85668	.	.	1
**14804**	0	2.97	1	0.85668	.	.	1
**14805**	0	2.97	1	0.85668	.	.	1

Obs	Sub_group	fuptimey	_CENSOR_	SURVIVAL	SDF_LCL	SDF_UCL	STRATUM
**14806**	0	2.97	1	0.85668	.	.	1
**14807**	0	2.97	1	0.85668	.	.	1
**14808**	0	2.97	1	0.85668	.	.	1
**14809**	0	2.97	1	0.85668	.	.	1
**14810**	0	2.97	1	0.85668	.	.	1
**14811**	0	2.97	1	0.85668	.	.	1
**14812**	0	2.97	1	0.85668	.	.	1
**14813**	0	2.97	1	0.85668	.	.	1
**14814**	0	2.97	1	0.85668	.	.	1
**14815**	0	2.97	1	0.85668	.	.	1
**14816**	0	2.97	1	0.85668	.	.	1
**14817**	0	2.97	1	0.85668	.	.	1
**14818**	0	2.97	1	0.85668	.	.	1
**14819**	0	2.97	1	0.85668	.	.	1
**14820**	0	2.97	1	0.85668	.	.	1
**14821**	0	2.97	1	0.85668	.	.	1
**14822**	0	2.97	1	0.85668	.	.	1
**14823**	0	2.97	1	0.85668	.	.	1
**14824**	0	2.97	1	0.85668	.	.	1
**14825**	0	2.97	1	0.85668	.	.	1
**14826**	0	2.97	1	0.85668	.	.	1
**14827**	0	2.97	1	0.85668	.	.	1
**14828**	0	2.97	1	0.85668	.	.	1
**14829**	0	2.97	1	0.85668	.	.	1
**14830**	0	2.97	1	0.85668	.	.	1
**14831**	0	2.97	1	0.85668	.	.	1
**14832**	0	2.97	1	0.85668	.	.	1
**14833**	0	2.97	1	0.85668	.	.	1
**14834**	0	2.97	1	0.85668	.	.	1
**14835**	0	2.97	1	0.85668	.	.	1
**14836**	0	2.98	0	0.85645	0.85299	0.85984	1
**14837**	0	2.98	1	0.85645	.	.	1
**14838**	0	2.98	1	0.85645	.	.	1
**14839**	0	2.98	1	0.85645	.	.	1
**14840**	0	2.98	1	0.85645	.	.	1

Obs	Sub_group	fuptimey	_CENSOR_	SURVIVAL	SDF_LCL	SDF_UCL	STRATUM
**14841**	0	2.98	1	0.85645	.	.	1
**14842**	0	2.98	1	0.85645	.	.	1
**14843**	0	2.98	1	0.85645	.	.	1
**14844**	0	2.98	1	0.85645	.	.	1
**14845**	0	2.98	1	0.85645	.	.	1
**14846**	0	2.98	1	0.85645	.	.	1
**14847**	0	2.98	1	0.85645	.	.	1
**14848**	0	2.98	1	0.85645	.	.	1
**14849**	0	2.98	1	0.85645	.	.	1
**14850**	0	2.98	1	0.85645	.	.	1
**14851**	0	2.98	1	0.85645	.	.	1
**14852**	0	2.98	1	0.85645	.	.	1
**14853**	0	2.98	1	0.85645	.	.	1
**14854**	0	2.98	1	0.85645	.	.	1
**14855**	0	2.98	1	0.85645	.	.	1
**14856**	0	2.98	1	0.85645	.	.	1
**14857**	0	2.98	1	0.85645	.	.	1
**14858**	0	2.98	1	0.85645	.	.	1
**14859**	0	2.98	1	0.85645	.	.	1
**14860**	0	2.98	1	0.85645	.	.	1
**14861**	0	2.98	1	0.85645	.	.	1
**14862**	0	2.98	1	0.85645	.	.	1
**14863**	0	2.98	1	0.85645	.	.	1
**14864**	0	2.98	1	0.85645	.	.	1
**14865**	0	2.98	1	0.85645	.	.	1
**14866**	0	2.98	1	0.85645	.	.	1
**14867**	0	2.98	1	0.85645	.	.	1
**14868**	0	2.98	1	0.85645	.	.	1
**14869**	0	2.98	1	0.85645	.	.	1
**14870**	0	2.98	1	0.85645	.	.	1
**14871**	0	2.98	1	0.85645	.	.	1
**14872**	0	2.98	1	0.85645	.	.	1
**14873**	0	2.98	1	0.85645	.	.	1
**14874**	0	2.98	1	0.85645	.	.	1
**14875**	0	2.98	1	0.85645	.	.	1

Obs	Sub_group	fuptimey	_CENSOR_	SURVIVAL	SDF_LCL	SDF_UCL	STRATUM
**14876**	0	2.98	1	0.85645	.	.	1
**14877**	0	2.98	1	0.85645	.	.	1
**14878**	0	2.98	1	0.85645	.	.	1
**14879**	0	2.98	1	0.85645	.	.	1
**14880**	0	2.98	1	0.85645	.	.	1
**14881**	0	2.98	1	0.85645	.	.	1
**14882**	0	2.98	1	0.85645	.	.	1
**14883**	0	2.98	1	0.85645	.	.	1
**14884**	0	2.98	1	0.85645	.	.	1
**14885**	0	2.98	1	0.85645	.	.	1
**14886**	0	2.98	1	0.85645	.	.	1
**14887**	0	2.98	1	0.85645	.	.	1
**14888**	0	2.98	1	0.85645	.	.	1
**14889**	0	2.98	1	0.85645	.	.	1
**14890**	0	2.99	0	0.85606	0.85259	0.85945	1
**14891**	0	2.99	1	0.85606	.	.	1
**14892**	0	2.99	1	0.85606	.	.	1
**14893**	0	2.99	1	0.85606	.	.	1
**14894**	0	2.99	1	0.85606	.	.	1
**14895**	0	2.99	1	0.85606	.	.	1
**14896**	0	2.99	1	0.85606	.	.	1
**14897**	0	2.99	1	0.85606	.	.	1
**14898**	0	2.99	1	0.85606	.	.	1
**14899**	0	2.99	1	0.85606	.	.	1
**14900**	0	2.99	1	0.85606	.	.	1
**14901**	0	2.99	1	0.85606	.	.	1
**14902**	0	2.99	1	0.85606	.	.	1
**14903**	0	2.99	1	0.85606	.	.	1
**14904**	0	2.99	1	0.85606	.	.	1
**14905**	0	2.99	1	0.85606	.	.	1
**14906**	0	2.99	1	0.85606	.	.	1
**14907**	0	2.99	1	0.85606	.	.	1
**14908**	0	2.99	1	0.85606	.	.	1
**14909**	0	2.99	1	0.85606	.	.	1
**14910**	0	2.99	1	0.85606	.	.	1

Obs	Sub_group	fuptimey	_CENSOR_	SURVIVAL	SDF_LCL	SDF_UCL	STRATUM
**14911**	0	2.99	1	0.85606	.	.	1
**14912**	0	2.99	1	0.85606	.	.	1
**14913**	0	2.99	1	0.85606	.	.	1
**14914**	0	2.99	1	0.85606	.	.	1
**14915**	0	2.99	1	0.85606	.	.	1
**14916**	0	2.99	1	0.85606	.	.	1
**14917**	0	2.99	1	0.85606	.	.	1
**14918**	0	3.00	0	0.85586	0.85239	0.85925	1
**14919**	0	3.00	1	0.85586	.	.	1
**14920**	0	3.00	1	0.85586	.	.	1
**14921**	0	3.00	1	0.85586	.	.	1
**14922**	0	3.00	1	0.85586	.	.	1
**14923**	0	3.00	1	0.85586	.	.	1
**14924**	0	3.00	1	0.85586	.	.	1
**14925**	0	3.00	1	0.85586	.	.	1
**14926**	0	3.00	1	0.85586	.	.	1
**14927**	0	3.00	1	0.85586	.	.	1
**14928**	0	3.00	1	0.85586	.	.	1
**14929**	0	3.00	1	0.85586	.	.	1
**14930**	0	3.00	1	0.85586	.	.	1
**14931**	0	3.00	1	0.85586	.	.	1
**14932**	0	3.00	1	0.85586	.	.	1
**14933**	0	3.00	1	0.85586	.	.	1
**14934**	0	3.00	1	0.85586	.	.	1
**14935**	0	3.00	1	0.85586	.	.	1
**14936**	0	3.00	1	0.85586	.	.	1
**14937**	0	3.00	1	0.85586	.	.	1
**14938**	0	3.00	1	0.85586	.	.	1
**14939**	0	3.00	1	0.85586	.	.	1
**14940**	0	3.00	1	0.85586	.	.	1
**14941**	0	3.00	1	0.85586	.	.	1
**14942**	0	3.00	1	0.85586	.	.	1
**14943**	0	3.00	1	0.85586	.	.	1
**14944**	0	3.00	1	0.85586	.	.	1
**14945**	0	3.00	1	0.85586	.	.	1

Obs	Sub_group	fuptimey	_CENSOR_	SURVIVAL	SDF_LCL	SDF_UCL	STRATUM
**14946**	0	3.00	1	0.85586	.	.	1
**14947**	0	3.00	1	0.85586	.	.	1
**14948**	0	3.00	1	0.85586	.	.	1
**14949**	0	3.00	1	0.85586	.	.	1
**14950**	0	3.00	1	0.85586	.	.	1
**14951**	0	3.00	1	0.85586	.	.	1
**14952**	0	3.00	1	0.85586	.	.	1
**14953**	0	3.00	1	0.85586	.	.	1
**14954**	0	3.00	1	0.85586	.	.	1
**14955**	0	3.00	1	0.85586	.	.	1
**14956**	0	3.00	1	0.85586	.	.	1
**14957**	0	3.00	1	0.85586	.	.	1
**14958**	0	3.00	1	0.85586	.	.	1
**14959**	0	3.00	1	0.85586	.	.	1
**14960**	0	3.00	1	0.85586	.	.	1
**14961**	0	3.00	1	0.85586	.	.	1
**14962**	0	3.00	1	0.85586	.	.	1
**14963**	0	3.00	1	0.85586	.	.	1
**14964**	0	3.00	1	0.85586	.	.	1
**14965**	0	3.00	1	0.85586	.	.	1
**14966**	0	3.00	1	0.85586	.	.	1
**14967**	0	3.00	1	0.85586	.	.	1
**14968**	0	3.00	1	0.85586	.	.	1
**14969**	0	3.00	1	0.85586	.	.	1
**14970**	0	3.00	1	0.85586	.	.	1
**14971**	0	3.00	1	0.85586	.	.	1
**14972**	0	3.01	0	0.85566	0.85219	0.85906	1
**14973**	0	3.01	1	0.85566	.	.	1
**14974**	0	3.01	1	0.85566	.	.	1
**14975**	0	3.01	1	0.85566	.	.	1
**14976**	0	3.01	1	0.85566	.	.	1
**14977**	0	3.01	1	0.85566	.	.	1
**14978**	0	3.01	1	0.85566	.	.	1
**14979**	0	3.01	1	0.85566	.	.	1
**14980**	0	3.01	1	0.85566	.	.	1

Obs	Sub_group	fuptimey	_CENSOR_	SURVIVAL	SDF_LCL	SDF_UCL	STRATUM
**14981**	0	3.01	1	0.85566	.	.	1
**14982**	0	3.01	1	0.85566	.	.	1
**14983**	0	3.01	1	0.85566	.	.	1
**14984**	0	3.01	1	0.85566	.	.	1
**14985**	0	3.01	1	0.85566	.	.	1
**14986**	0	3.01	1	0.85566	.	.	1
**14987**	0	3.01	1	0.85566	.	.	1
**14988**	0	3.01	1	0.85566	.	.	1
**14989**	0	3.01	1	0.85566	.	.	1
**14990**	0	3.01	1	0.85566	.	.	1
**14991**	0	3.01	1	0.85566	.	.	1
**14992**	0	3.01	1	0.85566	.	.	1
**14993**	0	3.01	1	0.85566	.	.	1
**14994**	0	3.01	1	0.85566	.	.	1
**14995**	0	3.01	1	0.85566	.	.	1
**14996**	0	3.01	1	0.85566	.	.	1
**14997**	0	3.01	1	0.85566	.	.	1
**14998**	0	3.01	1	0.85566	.	.	1
**14999**	0	3.01	1	0.85566	.	.	1
**15000**	0	3.01	1	0.85566	.	.	1
**15001**	0	3.01	1	0.85566	.	.	1
**15002**	0	3.01	1	0.85566	.	.	1
**15003**	0	3.01	1	0.85566	.	.	1
**15004**	0	3.01	1	0.85566	.	.	1
**15005**	0	3.01	1	0.85566	.	.	1
**15006**	0	3.02	0	0.85556	0.85209	0.85896	1
**15007**	0	3.02	1	0.85556	.	.	1
**15008**	0	3.02	1	0.85556	.	.	1
**15009**	0	3.02	1	0.85556	.	.	1
**15010**	0	3.02	1	0.85556	.	.	1
**15011**	0	3.02	1	0.85556	.	.	1
**15012**	0	3.02	1	0.85556	.	.	1
**15013**	0	3.02	1	0.85556	.	.	1
**15014**	0	3.02	1	0.85556	.	.	1
**15015**	0	3.02	1	0.85556	.	.	1

Obs	Sub_group	fuptimey	_CENSOR_	SURVIVAL	SDF_LCL	SDF_UCL	STRATUM
15016	0	3.02	1	0.85556	.	.	1
15017	0	3.02	1	0.85556	.	.	1
15018	0	3.02	1	0.85556	.	.	1
15019	0	3.02	1	0.85556	.	.	1
15020	0	3.02	1	0.85556	.	.	1
15021	0	3.02	1	0.85556	.	.	1
15022	0	3.02	1	0.85556	.	.	1
15023	0	3.02	1	0.85556	.	.	1
15024	0	3.02	1	0.85556	.	.	1
15025	0	3.02	1	0.85556	.	.	1
15026	0	3.02	1	0.85556	.	.	1
15027	0	3.02	1	0.85556	.	.	1
15028	0	3.02	1	0.85556	.	.	1
15029	0	3.02	1	0.85556	.	.	1
15030	0	3.02	1	0.85556	.	.	1
15031	0	3.02	1	0.85556	.	.	1
15032	0	3.02	1	0.85556	.	.	1
15033	0	3.02	1	0.85556	.	.	1
15034	0	3.02	1	0.85556	.	.	1
15035	0	3.02	1	0.85556	.	.	1
15036	0	3.02	1	0.85556	.	.	1
15037	0	3.02	1	0.85556	.	.	1
15038	0	3.02	1	0.85556	.	.	1
15039	0	3.02	1	0.85556	.	.	1
15040	0	3.02	1	0.85556	.	.	1
15041	0	3.02	1	0.85556	.	.	1
15042	0	3.02	1	0.85556	.	.	1
15043	0	3.02	1	0.85556	.	.	1
15044	0	3.02	1	0.85556	.	.	1
15045	0	3.02	1	0.85556	.	.	1
15046	0	3.02	1	0.85556	.	.	1
15047	0	3.02	1	0.85556	.	.	1
15048	0	3.03	0	0.85540	0.85192	0.85880	1
15049	0	3.03	1	0.85540	.	.	1
15050	0	3.03	1	0.85540	.	.	1

Obs	Sub_group	fuptimey	_CENSOR_	SURVIVAL	SDF_LCL	SDF_UCL	STRATUM
15051	0	3.03	1	0.85540	.	.	1
15052	0	3.03	1	0.85540	.	.	1
15053	0	3.03	1	0.85540	.	.	1
15054	0	3.03	1	0.85540	.	.	1
15055	0	3.03	1	0.85540	.	.	1
15056	0	3.03	1	0.85540	.	.	1
15057	0	3.03	1	0.85540	.	.	1
15058	0	3.03	1	0.85540	.	.	1
15059	0	3.03	1	0.85540	.	.	1
15060	0	3.03	1	0.85540	.	.	1
15061	0	3.03	1	0.85540	.	.	1
15062	0	3.03	1	0.85540	.	.	1
15063	0	3.03	1	0.85540	.	.	1
15064	0	3.03	1	0.85540	.	.	1
15065	0	3.03	1	0.85540	.	.	1
15066	0	3.03	1	0.85540	.	.	1
15067	0	3.03	1	0.85540	.	.	1
15068	0	3.03	1	0.85540	.	.	1
15069	0	3.03	1	0.85540	.	.	1
15070	0	3.03	1	0.85540	.	.	1
15071	0	3.03	1	0.85540	.	.	1
15072	0	3.03	1	0.85540	.	.	1
15073	0	3.03	1	0.85540	.	.	1
15074	0	3.03	1	0.85540	.	.	1
15075	0	3.03	1	0.85540	.	.	1
15076	0	3.03	1	0.85540	.	.	1
15077	0	3.03	1	0.85540	.	.	1
15078	0	3.03	1	0.85540	.	.	1
15079	0	3.03	1	0.85540	.	.	1
15080	0	3.03	1	0.85540	.	.	1
15081	0	3.03	1	0.85540	.	.	1
15082	0	3.03	1	0.85540	.	.	1
15083	0	3.03	1	0.85540	.	.	1
15084	0	3.03	1	0.85540	.	.	1
15085	0	3.03	1	0.85540	.	.	1

Obs	Sub_group	fuptimey	_CENSOR_	SURVIVAL	SDF_LCL	SDF_UCL	STRATUM
**15086**	0	3.03	1	0.85540	.	.	1
**15087**	0	3.03	1	0.85540	.	.	1
**15088**	0	3.03	1	0.85540	.	.	1
**15089**	0	3.03	1	0.85540	.	.	1
**15090**	0	3.04	0	0.85527	0.85179	0.85867	1
**15091**	0	3.04	1	0.85527	.	.	1
**15092**	0	3.04	1	0.85527	.	.	1
**15093**	0	3.04	1	0.85527	.	.	1
**15094**	0	3.04	1	0.85527	.	.	1
**15095**	0	3.04	1	0.85527	.	.	1
**15096**	0	3.04	1	0.85527	.	.	1
**15097**	0	3.04	1	0.85527	.	.	1
**15098**	0	3.04	1	0.85527	.	.	1
**15099**	0	3.04	1	0.85527	.	.	1
**15100**	0	3.04	1	0.85527	.	.	1
**15101**	0	3.04	1	0.85527	.	.	1
**15102**	0	3.04	1	0.85527	.	.	1
**15103**	0	3.04	1	0.85527	.	.	1
**15104**	0	3.04	1	0.85527	.	.	1
**15105**	0	3.04	1	0.85527	.	.	1
**15106**	0	3.04	1	0.85527	.	.	1
**15107**	0	3.04	1	0.85527	.	.	1
**15108**	0	3.04	1	0.85527	.	.	1
**15109**	0	3.04	1	0.85527	.	.	1
**15110**	0	3.04	1	0.85527	.	.	1
**15111**	0	3.04	1	0.85527	.	.	1
**15112**	0	3.04	1	0.85527	.	.	1
**15113**	0	3.04	1	0.85527	.	.	1
**15114**	0	3.04	1	0.85527	.	.	1
**15115**	0	3.04	1	0.85527	.	.	1
**15116**	0	3.04	1	0.85527	.	.	1
**15117**	0	3.04	1	0.85527	.	.	1
**15118**	0	3.04	1	0.85527	.	.	1
**15119**	0	3.04	1	0.85527	.	.	1
**15120**	0	3.04	1	0.85527	.	.	1

Obs	Sub_group	fuptimey	_CENSOR_	SURVIVAL	SDF_LCL	SDF_UCL	STRATUM
15121	0	3.04	1	0.85527	.	.	1
15122	0	3.04	1	0.85527	.	.	1
15123	0	3.04	1	0.85527	.	.	1
15124	0	3.04	1	0.85527	.	.	1
15125	0	3.04	1	0.85527	.	.	1
15126	0	3.04	1	0.85527	.	.	1
15127	0	3.04	1	0.85527	.	.	1
15128	0	3.04	1	0.85527	.	.	1
15129	0	3.04	1	0.85527	.	.	1
15130	0	3.04	1	0.85527	.	.	1
15131	0	3.04	1	0.85527	.	.	1
15132	0	3.04	1	0.85527	.	.	1
15133	0	3.04	1	0.85527	.	.	1
15134	0	3.04	1	0.85527	.	.	1
15135	0	3.04	1	0.85527	.	.	1
15136	0	3.04	1	0.85527	.	.	1
15137	0	3.05	0	0.85503	0.85155	0.85844	1
15138	0	3.05	1	0.85503	.	.	1
15139	0	3.05	1	0.85503	.	.	1
15140	0	3.05	1	0.85503	.	.	1
15141	0	3.05	1	0.85503	.	.	1
15142	0	3.05	1	0.85503	.	.	1
15143	0	3.05	1	0.85503	.	.	1
15144	0	3.05	1	0.85503	.	.	1
15145	0	3.05	1	0.85503	.	.	1
15146	0	3.05	1	0.85503	.	.	1
15147	0	3.05	1	0.85503	.	.	1
15148	0	3.05	1	0.85503	.	.	1
15149	0	3.05	1	0.85503	.	.	1
15150	0	3.05	1	0.85503	.	.	1
15151	0	3.05	1	0.85503	.	.	1
15152	0	3.05	1	0.85503	.	.	1
15153	0	3.05	1	0.85503	.	.	1
15154	0	3.05	1	0.85503	.	.	1
15155	0	3.05	1	0.85503	.	.	1

Obs	Sub_group	fuptimey	_CENSOR_	SURVIVAL	SDF_LCL	SDF_UCL	STRATUM
15156	0	3.05	1	0.85503	.	.	1
15157	0	3.05	1	0.85503	.	.	1
15158	0	3.05	1	0.85503	.	.	1
15159	0	3.05	1	0.85503	.	.	1
15160	0	3.05	1	0.85503	.	.	1
15161	0	3.05	1	0.85503	.	.	1
15162	0	3.05	1	0.85503	.	.	1
15163	0	3.05	1	0.85503	.	.	1
15164	0	3.05	1	0.85503	.	.	1
15165	0	3.05	1	0.85503	.	.	1
15166	0	3.05	1	0.85503	.	.	1
15167	0	3.05	1	0.85503	.	.	1
15168	0	3.05	1	0.85503	.	.	1
15169	0	3.05	1	0.85503	.	.	1
15170	0	3.05	1	0.85503	.	.	1
15171	0	3.05	1	0.85503	.	.	1
15172	0	3.05	1	0.85503	.	.	1
15173	0	3.05	1	0.85503	.	.	1
15174	0	3.05	1	0.85503	.	.	1
15175	0	3.05	1	0.85503	.	.	1
15176	0	3.05	1	0.85503	.	.	1
15177	0	3.05	1	0.85503	.	.	1
15178	0	3.05	1	0.85503	.	.	1
15179	0	3.05	1	0.85503	.	.	1
15180	0	3.05	1	0.85503	.	.	1
15181	0	3.05	1	0.85503	.	.	1
15182	0	3.05	1	0.85503	.	.	1
15183	0	3.06	0	0.85487	0.85138	0.85828	1
15184	0	3.06	1	0.85487	.	.	1
15185	0	3.06	1	0.85487	.	.	1
15186	0	3.06	1	0.85487	.	.	1
15187	0	3.06	1	0.85487	.	.	1
15188	0	3.06	1	0.85487	.	.	1
15189	0	3.06	1	0.85487	.	.	1
15190	0	3.06	1	0.85487	.	.	1

Obs	Sub_group	fuptimey	_CENSOR_	SURVIVAL	SDF_LCL	SDF_UCL	STRATUM
15191	0	3.06	1	0.85487	.	.	1
15192	0	3.06	1	0.85487	.	.	1
15193	0	3.06	1	0.85487	.	.	1
15194	0	3.06	1	0.85487	.	.	1
15195	0	3.06	1	0.85487	.	.	1
15196	0	3.06	1	0.85487	.	.	1
15197	0	3.06	1	0.85487	.	.	1
15198	0	3.06	1	0.85487	.	.	1
15199	0	3.06	1	0.85487	.	.	1
15200	0	3.06	1	0.85487	.	.	1
15201	0	3.06	1	0.85487	.	.	1
15202	0	3.06	1	0.85487	.	.	1
15203	0	3.06	1	0.85487	.	.	1
15204	0	3.06	1	0.85487	.	.	1
15205	0	3.06	1	0.85487	.	.	1
15206	0	3.06	1	0.85487	.	.	1
15207	0	3.06	1	0.85487	.	.	1
15208	0	3.06	1	0.85487	.	.	1
15209	0	3.06	1	0.85487	.	.	1
15210	0	3.06	1	0.85487	.	.	1
15211	0	3.06	1	0.85487	.	.	1
15212	0	3.06	1	0.85487	.	.	1
15213	0	3.06	1	0.85487	.	.	1
15214	0	3.06	1	0.85487	.	.	1
15215	0	3.06	1	0.85487	.	.	1
15216	0	3.06	1	0.85487	.	.	1
15217	0	3.06	1	0.85487	.	.	1
15218	0	3.07	0	0.85473	0.85125	0.85815	1
15219	0	3.07	1	0.85473	.	.	1
15220	0	3.07	1	0.85473	.	.	1
15221	0	3.07	1	0.85473	.	.	1
15222	0	3.07	1	0.85473	.	.	1
15223	0	3.07	1	0.85473	.	.	1
15224	0	3.07	1	0.85473	.	.	1
15225	0	3.07	1	0.85473	.	.	1

Obs	Sub_group	fuptimey	_CENSOR_	SURVIVAL	SDF_LCL	SDF_UCL	STRATUM
15226	0	3.07	1	0.85473	.	.	1
15227	0	3.07	1	0.85473	.	.	1
15228	0	3.07	1	0.85473	.	.	1
15229	0	3.07	1	0.85473	.	.	1
15230	0	3.07	1	0.85473	.	.	1
15231	0	3.07	1	0.85473	.	.	1
15232	0	3.07	1	0.85473	.	.	1
15233	0	3.07	1	0.85473	.	.	1
15234	0	3.07	1	0.85473	.	.	1
15235	0	3.07	1	0.85473	.	.	1
15236	0	3.07	1	0.85473	.	.	1
15237	0	3.07	1	0.85473	.	.	1
15238	0	3.07	1	0.85473	.	.	1
15239	0	3.07	1	0.85473	.	.	1
15240	0	3.07	1	0.85473	.	.	1
15241	0	3.07	1	0.85473	.	.	1
15242	0	3.07	1	0.85473	.	.	1
15243	0	3.07	1	0.85473	.	.	1
15244	0	3.07	1	0.85473	.	.	1
15245	0	3.07	1	0.85473	.	.	1
15246	0	3.07	1	0.85473	.	.	1
15247	0	3.07	1	0.85473	.	.	1
15248	0	3.07	1	0.85473	.	.	1
15249	0	3.07	1	0.85473	.	.	1
15250	0	3.07	1	0.85473	.	.	1
15251	0	3.07	1	0.85473	.	.	1
15252	0	3.07	1	0.85473	.	.	1
15253	0	3.07	1	0.85473	.	.	1
15254	0	3.07	1	0.85473	.	.	1
15255	0	3.07	1	0.85473	.	.	1
15256	0	3.08	0	0.85457	0.85108	0.85798	1
15257	0	3.08	1	0.85457	.	.	1
15258	0	3.08	1	0.85457	.	.	1
15259	0	3.08	1	0.85457	.	.	1
15260	0	3.08	1	0.85457	.	.	1

Obs	Sub_group	fuptimey	_CENSOR_	SURVIVAL	SDF_LCL	SDF_UCL	STRATUM
15261	0	3.08	1	0.85457	.	.	1
15262	0	3.08	1	0.85457	.	.	1
15263	0	3.08	1	0.85457	.	.	1
15264	0	3.08	1	0.85457	.	.	1
15265	0	3.08	1	0.85457	.	.	1
15266	0	3.08	1	0.85457	.	.	1
15267	0	3.08	1	0.85457	.	.	1
15268	0	3.08	1	0.85457	.	.	1
15269	0	3.08	1	0.85457	.	.	1
15270	0	3.08	1	0.85457	.	.	1
15271	0	3.08	1	0.85457	.	.	1
15272	0	3.08	1	0.85457	.	.	1
15273	0	3.08	1	0.85457	.	.	1
15274	0	3.08	1	0.85457	.	.	1
15275	0	3.08	1	0.85457	.	.	1
15276	0	3.08	1	0.85457	.	.	1
15277	0	3.08	1	0.85457	.	.	1
15278	0	3.08	1	0.85457	.	.	1
15279	0	3.08	1	0.85457	.	.	1
15280	0	3.08	1	0.85457	.	.	1
15281	0	3.08	1	0.85457	.	.	1
15282	0	3.08	1	0.85457	.	.	1
15283	0	3.08	1	0.85457	.	.	1
15284	0	3.08	1	0.85457	.	.	1
15285	0	3.08	1	0.85457	.	.	1
15286	0	3.08	1	0.85457	.	.	1
15287	0	3.08	1	0.85457	.	.	1
15288	0	3.08	1	0.85457	.	.	1
15289	0	3.08	1	0.85457	.	.	1
15290	0	3.08	1	0.85457	.	.	1
15291	0	3.08	1	0.85457	.	.	1
15292	0	3.08	1	0.85457	.	.	1
15293	0	3.08	1	0.85457	.	.	1
15294	0	3.09	0	0.85424	0.85074	0.85765	1
15295	0	3.09	1	0.85424	.	.	1

Obs	Sub_group	fuptimey	_CENSOR_	SURVIVAL	SDF_LCL	SDF_UCL	STRATUM
15296	0	3.09	1	0.85424	.	.	1
15297	0	3.09	1	0.85424	.	.	1
15298	0	3.09	1	0.85424	.	.	1
15299	0	3.09	1	0.85424	.	.	1
15300	0	3.09	1	0.85424	.	.	1
15301	0	3.09	1	0.85424	.	.	1
15302	0	3.09	1	0.85424	.	.	1
15303	0	3.09	1	0.85424	.	.	1
15304	0	3.09	1	0.85424	.	.	1
15305	0	3.09	1	0.85424	.	.	1
15306	0	3.09	1	0.85424	.	.	1
15307	0	3.09	1	0.85424	.	.	1
15308	0	3.09	1	0.85424	.	.	1
15309	0	3.09	1	0.85424	.	.	1
15310	0	3.09	1	0.85424	.	.	1
15311	0	3.09	1	0.85424	.	.	1
15312	0	3.09	1	0.85424	.	.	1
15313	0	3.09	1	0.85424	.	.	1
15314	0	3.09	1	0.85424	.	.	1
15315	0	3.09	1	0.85424	.	.	1
15316	0	3.09	1	0.85424	.	.	1
15317	0	3.09	1	0.85424	.	.	1
15318	0	3.09	1	0.85424	.	.	1
15319	0	3.09	1	0.85424	.	.	1
15320	0	3.09	1	0.85424	.	.	1
15321	0	3.09	1	0.85424	.	.	1
15322	0	3.09	1	0.85424	.	.	1
15323	0	3.09	1	0.85424	.	.	1
15324	0	3.09	1	0.85424	.	.	1
15325	0	3.09	1	0.85424	.	.	1
15326	0	3.09	1	0.85424	.	.	1
15327	0	3.09	1	0.85424	.	.	1
15328	0	3.09	1	0.85424	.	.	1
15329	0	3.09	1	0.85424	.	.	1
15330	0	3.09	1	0.85424	.	.	1

Obs	Sub_group	fuptimey	_CENSOR_	SURVIVAL	SDF_LCL	SDF_UCL	STRATUM
15331	0	3.09	1	0.85424	.	.	1
15332	0	3.09	1	0.85424	.	.	1
15333	0	3.09	1	0.85424	.	.	1
15334	0	3.09	1	0.85424	.	.	1
15335	0	3.09	1	0.85424	.	.	1
15336	0	3.09	1	0.85424	.	.	1
15337	0	3.09	1	0.85424	.	.	1
15338	0	3.09	1	0.85424	.	.	1
15339	0	3.09	1	0.85424	.	.	1
15340	0	3.09	1	0.85424	.	.	1
15341	0	3.09	1	0.85424	.	.	1
15342	0	3.09	1	0.85424	.	.	1
15343	0	3.09	1	0.85424	.	.	1
15344	0	3.09	1	0.85424	.	.	1
15345	0	3.09	1	0.85424	.	.	1
15346	0	3.09	1	0.85424	.	.	1
15347	0	3.09	1	0.85424	.	.	1
15348	0	3.09	1	0.85424	.	.	1
15349	0	3.09	1	0.85424	.	.	1
15350	0	3.09	1	0.85424	.	.	1
15351	0	3.09	1	0.85424	.	.	1
15352	0	3.10	0	0.85394	0.85044	0.85736	1
15353	0	3.10	1	0.85394	.	.	1
15354	0	3.10	1	0.85394	.	.	1
15355	0	3.10	1	0.85394	.	.	1
15356	0	3.10	1	0.85394	.	.	1
15357	0	3.10	1	0.85394	.	.	1
15358	0	3.10	1	0.85394	.	.	1
15359	0	3.10	1	0.85394	.	.	1
15360	0	3.10	1	0.85394	.	.	1
15361	0	3.10	1	0.85394	.	.	1
15362	0	3.10	1	0.85394	.	.	1
15363	0	3.10	1	0.85394	.	.	1
15364	0	3.10	1	0.85394	.	.	1
15365	0	3.10	1	0.85394	.	.	1

Obs	Sub_group	fuptimey	_CENSOR_	SURVIVAL	SDF_LCL	SDF_UCL	STRATUM
15366	0	3.10	1	0.85394	.	.	1
15367	0	3.10	1	0.85394	.	.	1
15368	0	3.10	1	0.85394	.	.	1
15369	0	3.10	1	0.85394	.	.	1
15370	0	3.10	1	0.85394	.	.	1
15371	0	3.10	1	0.85394	.	.	1
15372	0	3.10	1	0.85394	.	.	1
15373	0	3.10	1	0.85394	.	.	1
15374	0	3.10	1	0.85394	.	.	1
15375	0	3.10	1	0.85394	.	.	1
15376	0	3.10	1	0.85394	.	.	1
15377	0	3.10	1	0.85394	.	.	1
15378	0	3.10	1	0.85394	.	.	1
15379	0	3.10	1	0.85394	.	.	1
15380	0	3.10	1	0.85394	.	.	1
15381	0	3.10	1	0.85394	.	.	1
15382	0	3.10	1	0.85394	.	.	1
15383	0	3.10	1	0.85394	.	.	1
15384	0	3.10	1	0.85394	.	.	1
15385	0	3.10	1	0.85394	.	.	1
15386	0	3.10	1	0.85394	.	.	1
15387	0	3.11	0	0.85370	0.85020	0.85713	1
15388	0	3.11	1	0.85370	.	.	1
15389	0	3.11	1	0.85370	.	.	1
15390	0	3.11	1	0.85370	.	.	1
15391	0	3.11	1	0.85370	.	.	1
15392	0	3.11	1	0.85370	.	.	1
15393	0	3.11	1	0.85370	.	.	1
15394	0	3.11	1	0.85370	.	.	1
15395	0	3.11	1	0.85370	.	.	1
15396	0	3.11	1	0.85370	.	.	1
15397	0	3.11	1	0.85370	.	.	1
15398	0	3.11	1	0.85370	.	.	1
15399	0	3.11	1	0.85370	.	.	1
15400	0	3.11	1	0.85370	.	.	1

Obs	Sub_group	fuptimey	_CENSOR_	SURVIVAL	SDF_LCL	SDF_UCL	STRATUM
15401	0	3.11	1	0.85370	.	.	1
15402	0	3.11	1	0.85370	.	.	1
15403	0	3.11	1	0.85370	.	.	1
15404	0	3.11	1	0.85370	.	.	1
15405	0	3.11	1	0.85370	.	.	1
15406	0	3.11	1	0.85370	.	.	1
15407	0	3.11	1	0.85370	.	.	1
15408	0	3.11	1	0.85370	.	.	1
15409	0	3.11	1	0.85370	.	.	1
15410	0	3.11	1	0.85370	.	.	1
15411	0	3.11	1	0.85370	.	.	1
15412	0	3.11	1	0.85370	.	.	1
15413	0	3.11	1	0.85370	.	.	1
15414	0	3.11	1	0.85370	.	.	1
15415	0	3.11	1	0.85370	.	.	1
15416	0	3.11	1	0.85370	.	.	1
15417	0	3.11	1	0.85370	.	.	1
15418	0	3.11	1	0.85370	.	.	1
15419	0	3.11	1	0.85370	.	.	1
15420	0	3.11	1	0.85370	.	.	1
15421	0	3.11	1	0.85370	.	.	1
15422	0	3.11	1	0.85370	.	.	1
15423	0	3.11	1	0.85370	.	.	1
15424	0	3.11	1	0.85370	.	.	1
15425	0	3.11	1	0.85370	.	.	1
15426	0	3.11	1	0.85370	.	.	1
15427	0	3.11	1	0.85370	.	.	1
15428	0	3.11	1	0.85370	.	.	1
15429	0	3.11	1	0.85370	.	.	1
15430	0	3.11	1	0.85370	.	.	1
15431	0	3.11	1	0.85370	.	.	1
15432	0	3.11	1	0.85370	.	.	1
15433	0	3.11	1	0.85370	.	.	1
15434	0	3.12	0	0.85360	0.85010	0.85703	1
15435	0	3.12	1	0.85360	.	.	1

Obs	Sub_group	fuptimey	_CENSOR_	SURVIVAL	SDF_LCL	SDF_UCL	STRATUM
15436	0	3.12	1	0.85360	.	.	1
15437	0	3.12	1	0.85360	.	.	1
15438	0	3.12	1	0.85360	.	.	1
15439	0	3.12	1	0.85360	.	.	1
15440	0	3.12	1	0.85360	.	.	1
15441	0	3.12	1	0.85360	.	.	1
15442	0	3.12	1	0.85360	.	.	1
15443	0	3.12	1	0.85360	.	.	1
15444	0	3.12	1	0.85360	.	.	1
15445	0	3.12	1	0.85360	.	.	1
15446	0	3.12	1	0.85360	.	.	1
15447	0	3.12	1	0.85360	.	.	1
15448	0	3.12	1	0.85360	.	.	1
15449	0	3.12	1	0.85360	.	.	1
15450	0	3.12	1	0.85360	.	.	1
15451	0	3.12	1	0.85360	.	.	1
15452	0	3.12	1	0.85360	.	.	1
15453	0	3.12	1	0.85360	.	.	1
15454	0	3.12	1	0.85360	.	.	1
15455	0	3.12	1	0.85360	.	.	1
15456	0	3.12	1	0.85360	.	.	1
15457	0	3.12	1	0.85360	.	.	1
15458	0	3.12	1	0.85360	.	.	1
15459	0	3.12	1	0.85360	.	.	1
15460	0	3.12	1	0.85360	.	.	1
15461	0	3.12	1	0.85360	.	.	1
15462	0	3.12	1	0.85360	.	.	1
15463	0	3.12	1	0.85360	.	.	1
15464	0	3.12	1	0.85360	.	.	1
15465	0	3.12	1	0.85360	.	.	1
15466	0	3.12	1	0.85360	.	.	1
15467	0	3.13	0	0.85347	0.84996	0.85690	1
15468	0	3.13	1	0.85347	.	.	1
15469	0	3.13	1	0.85347	.	.	1
15470	0	3.13	1	0.85347	.	.	1

Obs	Sub_group	fuptimey	_CENSOR_	SURVIVAL	SDF_LCL	SDF_UCL	STRATUM
15471	0	3.13	1	0.85347	.	.	1
15472	0	3.13	1	0.85347	.	.	1
15473	0	3.13	1	0.85347	.	.	1
15474	0	3.13	1	0.85347	.	.	1
15475	0	3.13	1	0.85347	.	.	1
15476	0	3.13	1	0.85347	.	.	1
15477	0	3.13	1	0.85347	.	.	1
15478	0	3.13	1	0.85347	.	.	1
15479	0	3.13	1	0.85347	.	.	1
15480	0	3.13	1	0.85347	.	.	1
15481	0	3.13	1	0.85347	.	.	1
15482	0	3.13	1	0.85347	.	.	1
15483	0	3.13	1	0.85347	.	.	1
15484	0	3.13	1	0.85347	.	.	1
15485	0	3.13	1	0.85347	.	.	1
15486	0	3.13	1	0.85347	.	.	1
15487	0	3.13	1	0.85347	.	.	1
15488	0	3.13	1	0.85347	.	.	1
15489	0	3.13	1	0.85347	.	.	1
15490	0	3.13	1	0.85347	.	.	1
15491	0	3.13	1	0.85347	.	.	1
15492	0	3.13	1	0.85347	.	.	1
15493	0	3.13	1	0.85347	.	.	1
15494	0	3.13	1	0.85347	.	.	1
15495	0	3.13	1	0.85347	.	.	1
15496	0	3.13	1	0.85347	.	.	1
15497	0	3.13	1	0.85347	.	.	1
15498	0	3.13	1	0.85347	.	.	1
15499	0	3.13	1	0.85347	.	.	1
15500	0	3.13	1	0.85347	.	.	1
15501	0	3.13	1	0.85347	.	.	1
15502	0	3.13	1	0.85347	.	.	1
15503	0	3.13	1	0.85347	.	.	1
15504	0	3.13	1	0.85347	.	.	1
15505	0	3.13	1	0.85347	.	.	1

Obs	Sub_group	fuptimey	_CENSOR_	SURVIVAL	SDF_LCL	SDF_UCL	STRATUM
15506	0	3.13	1	0.85347	.	.	1
15507	0	3.13	1	0.85347	.	.	1
15508	0	3.13	1	0.85347	.	.	1
15509	0	3.13	1	0.85347	.	.	1
15510	0	3.13	1	0.85347	.	.	1
15511	0	3.13	1	0.85347	.	.	1
15512	0	3.13	1	0.85347	.	.	1
15513	0	3.13	1	0.85347	.	.	1
15514	0	3.13	1	0.85347	.	.	1
15515	0	3.13	1	0.85347	.	.	1
15516	0	3.13	1	0.85347	.	.	1
15517	0	3.13	1	0.85347	.	.	1
15518	0	3.13	1	0.85347	.	.	1
15519	0	3.13	1	0.85347	.	.	1
15520	0	3.13	1	0.85347	.	.	1
15521	0	3.13	1	0.85347	.	.	1
15522	0	3.14	0	0.85320	0.84969	0.85663	1
15523	0	3.14	1	0.85320	.	.	1
15524	0	3.14	1	0.85320	.	.	1
15525	0	3.14	1	0.85320	.	.	1
15526	0	3.14	1	0.85320	.	.	1
15527	0	3.14	1	0.85320	.	.	1
15528	0	3.14	1	0.85320	.	.	1
15529	0	3.14	1	0.85320	.	.	1
15530	0	3.14	1	0.85320	.	.	1
15531	0	3.14	1	0.85320	.	.	1
15532	0	3.14	1	0.85320	.	.	1
15533	0	3.14	1	0.85320	.	.	1
15534	0	3.14	1	0.85320	.	.	1
15535	0	3.14	1	0.85320	.	.	1
15536	0	3.14	1	0.85320	.	.	1
15537	0	3.14	1	0.85320	.	.	1
15538	0	3.14	1	0.85320	.	.	1
15539	0	3.14	1	0.85320	.	.	1
15540	0	3.14	1	0.85320	.	.	1

Obs	Sub_group	fuptimey	_CENSOR_	SURVIVAL	SDF_LCL	SDF_UCL	STRATUM
15541	0	3.14	1	0.85320	.	.	1
15542	0	3.14	1	0.85320	.	.	1
15543	0	3.14	1	0.85320	.	.	1
15544	0	3.14	1	0.85320	.	.	1
15545	0	3.14	1	0.85320	.	.	1
15546	0	3.14	1	0.85320	.	.	1
15547	0	3.14	1	0.85320	.	.	1
15548	0	3.14	1	0.85320	.	.	1
15549	0	3.14	1	0.85320	.	.	1
15550	0	3.14	1	0.85320	.	.	1
15551	0	3.14	1	0.85320	.	.	1
15552	0	3.14	1	0.85320	.	.	1
15553	0	3.14	1	0.85320	.	.	1
15554	0	3.14	1	0.85320	.	.	1
15555	0	3.14	1	0.85320	.	.	1
15556	0	3.14	1	0.85320	.	.	1
15557	0	3.14	1	0.85320	.	.	1
15558	0	3.14	1	0.85320	.	.	1
15559	0	3.14	1	0.85320	.	.	1
15560	0	3.14	1	0.85320	.	.	1
15561	0	3.14	1	0.85320	.	.	1
15562	0	3.14	1	0.85320	.	.	1
15563	0	3.14	1	0.85320	.	.	1
15564	0	3.15	0	0.85286	0.84935	0.85630	1
15565	0	3.15	1	0.85286	.	.	1
15566	0	3.15	1	0.85286	.	.	1
15567	0	3.15	1	0.85286	.	.	1
15568	0	3.15	1	0.85286	.	.	1
15569	0	3.15	1	0.85286	.	.	1
15570	0	3.15	1	0.85286	.	.	1
15571	0	3.15	1	0.85286	.	.	1
15572	0	3.15	1	0.85286	.	.	1
15573	0	3.15	1	0.85286	.	.	1
15574	0	3.15	1	0.85286	.	.	1
15575	0	3.15	1	0.85286	.	.	1

Obs	Sub_group	fuptimey	_CENSOR_	SURVIVAL	SDF_LCL	SDF_UCL	STRATUM
15576	0	3.15	1	0.85286	.	.	1
15577	0	3.15	1	0.85286	.	.	1
15578	0	3.15	1	0.85286	.	.	1
15579	0	3.15	1	0.85286	.	.	1
15580	0	3.15	1	0.85286	.	.	1
15581	0	3.15	1	0.85286	.	.	1
15582	0	3.15	1	0.85286	.	.	1
15583	0	3.15	1	0.85286	.	.	1
15584	0	3.15	1	0.85286	.	.	1
15585	0	3.15	1	0.85286	.	.	1
15586	0	3.15	1	0.85286	.	.	1
15587	0	3.15	1	0.85286	.	.	1
15588	0	3.15	1	0.85286	.	.	1
15589	0	3.15	1	0.85286	.	.	1
15590	0	3.15	1	0.85286	.	.	1
15591	0	3.15	1	0.85286	.	.	1
15592	0	3.15	1	0.85286	.	.	1
15593	0	3.15	1	0.85286	.	.	1
15594	0	3.15	1	0.85286	.	.	1
15595	0	3.15	1	0.85286	.	.	1
15596	0	3.15	1	0.85286	.	.	1
15597	0	3.15	1	0.85286	.	.	1
15598	0	3.15	1	0.85286	.	.	1
15599	0	3.15	1	0.85286	.	.	1
15600	0	3.15	1	0.85286	.	.	1
15601	0	3.15	1	0.85286	.	.	1
15602	0	3.15	1	0.85286	.	.	1
15603	0	3.15	1	0.85286	.	.	1
15604	0	3.15	1	0.85286	.	.	1
15605	0	3.15	1	0.85286	.	.	1
15606	0	3.15	1	0.85286	.	.	1
15607	0	3.15	1	0.85286	.	.	1
15608	0	3.15	1	0.85286	.	.	1
15609	0	3.15	1	0.85286	.	.	1
15610	0	3.15	1	0.85286	.	.	1

Obs	Sub_group	fuptimey	_CENSOR_	SURVIVAL	SDF_LCL	SDF_UCL	STRATUM
15611	0	3.15	1	0.85286	.	.	1
15612	0	3.15	1	0.85286	.	.	1
15613	0	3.15	1	0.85286	.	.	1
15614	0	3.16	0	0.85259	0.84908	0.85603	1
15615	0	3.16	1	0.85259	.	.	1
15616	0	3.16	1	0.85259	.	.	1
15617	0	3.16	1	0.85259	.	.	1
15618	0	3.16	1	0.85259	.	.	1
15619	0	3.16	1	0.85259	.	.	1
15620	0	3.16	1	0.85259	.	.	1
15621	0	3.16	1	0.85259	.	.	1
15622	0	3.16	1	0.85259	.	.	1
15623	0	3.16	1	0.85259	.	.	1
15624	0	3.16	1	0.85259	.	.	1
15625	0	3.16	1	0.85259	.	.	1
15626	0	3.16	1	0.85259	.	.	1
15627	0	3.16	1	0.85259	.	.	1
15628	0	3.16	1	0.85259	.	.	1
15629	0	3.16	1	0.85259	.	.	1
15630	0	3.16	1	0.85259	.	.	1
15631	0	3.16	1	0.85259	.	.	1
15632	0	3.16	1	0.85259	.	.	1
15633	0	3.16	1	0.85259	.	.	1
15634	0	3.16	1	0.85259	.	.	1
15635	0	3.16	1	0.85259	.	.	1
15636	0	3.16	1	0.85259	.	.	1
15637	0	3.16	1	0.85259	.	.	1
15638	0	3.16	1	0.85259	.	.	1
15639	0	3.16	1	0.85259	.	.	1
15640	0	3.16	1	0.85259	.	.	1
15641	0	3.16	1	0.85259	.	.	1
15642	0	3.16	1	0.85259	.	.	1
15643	0	3.16	1	0.85259	.	.	1
15644	0	3.16	1	0.85259	.	.	1
15645	0	3.16	1	0.85259	.	.	1

Obs	Sub_group	fuptimey	_CENSOR_	SURVIVAL	SDF_LCL	SDF_UCL	STRATUM
15646	0	3.16	1	0.85259	.	.	1
15647	0	3.16	1	0.85259	.	.	1
15648	0	3.16	1	0.85259	.	.	1
15649	0	3.16	1	0.85259	.	.	1
15650	0	3.17	0	0.85229	0.84877	0.85574	1
15651	0	3.17	1	0.85229	.	.	1
15652	0	3.17	1	0.85229	.	.	1
15653	0	3.17	1	0.85229	.	.	1
15654	0	3.17	1	0.85229	.	.	1
15655	0	3.17	1	0.85229	.	.	1
15656	0	3.17	1	0.85229	.	.	1
15657	0	3.17	1	0.85229	.	.	1
15658	0	3.17	1	0.85229	.	.	1
15659	0	3.17	1	0.85229	.	.	1
15660	0	3.17	1	0.85229	.	.	1
15661	0	3.17	1	0.85229	.	.	1
15662	0	3.17	1	0.85229	.	.	1
15663	0	3.17	1	0.85229	.	.	1
15664	0	3.17	1	0.85229	.	.	1
15665	0	3.17	1	0.85229	.	.	1
15666	0	3.17	1	0.85229	.	.	1
15667	0	3.17	1	0.85229	.	.	1
15668	0	3.17	1	0.85229	.	.	1
15669	0	3.17	1	0.85229	.	.	1
15670	0	3.17	1	0.85229	.	.	1
15671	0	3.17	1	0.85229	.	.	1
15672	0	3.17	1	0.85229	.	.	1
15673	0	3.17	1	0.85229	.	.	1
15674	0	3.17	1	0.85229	.	.	1
15675	0	3.17	1	0.85229	.	.	1
15676	0	3.17	1	0.85229	.	.	1
15677	0	3.17	1	0.85229	.	.	1
15678	0	3.17	1	0.85229	.	.	1
15679	0	3.17	1	0.85229	.	.	1
15680	0	3.17	1	0.85229	.	.	1

Obs	Sub_group	fuptimey	_CENSOR_	SURVIVAL	SDF_LCL	SDF_UCL	STRATUM
15681	0	3.17	1	0.85229	.	.	1
15682	0	3.17	1	0.85229	.	.	1
15683	0	3.17	1	0.85229	.	.	1
15684	0	3.17	1	0.85229	.	.	1
15685	0	3.17	1	0.85229	.	.	1
15686	0	3.17	1	0.85229	.	.	1
15687	0	3.17	1	0.85229	.	.	1
15688	0	3.17	1	0.85229	.	.	1
15689	0	3.17	1	0.85229	.	.	1
15690	0	3.17	1	0.85229	.	.	1
15691	0	3.17	1	0.85229	.	.	1
15692	0	3.17	1	0.85229	.	.	1
15693	0	3.17	1	0.85229	.	.	1
15694	0	3.17	1	0.85229	.	.	1
15695	0	3.17	1	0.85229	.	.	1
15696	0	3.17	1	0.85229	.	.	1
15697	0	3.17	1	0.85229	.	.	1
15698	0	3.17	1	0.85229	.	.	1
15699	0	3.17	1	0.85229	.	.	1
15700	0	3.17	1	0.85229	.	.	1
15701	0	3.17	1	0.85229	.	.	1
15702	0	3.17	1	0.85229	.	.	1
15703	0	3.17	1	0.85229	.	.	1
15704	0	3.17	1	0.85229	.	.	1
15705	0	3.17	1	0.85229	.	.	1
15706	0	3.17	1	0.85229	.	.	1
15707	0	3.17	1	0.85229	.	.	1
15708	0	3.17	1	0.85229	.	.	1
15709	0	3.17	1	0.85229	.	.	1
15710	0	3.17	1	0.85229	.	.	1
15711	0	3.17	1	0.85229	.	.	1
15712	0	3.17	1	0.85229	.	.	1
15713	0	3.17	1	0.85229	.	.	1
15714	0	3.17	1	0.85229	.	.	1
15715	0	3.17	1	0.85229	.	.	1

Obs	Sub_group	fuptimey	_CENSOR_	SURVIVAL	SDF_LCL	SDF_UCL	STRATUM
15716	0	3.17	1	0.85229	.	.	1
15717	0	3.18	0	0.85209	0.84856	0.85554	1
15718	0	3.18	1	0.85209	.	.	1
15719	0	3.18	1	0.85209	.	.	1
15720	0	3.18	1	0.85209	.	.	1
15721	0	3.18	1	0.85209	.	.	1
15722	0	3.18	1	0.85209	.	.	1
15723	0	3.18	1	0.85209	.	.	1
15724	0	3.18	1	0.85209	.	.	1
15725	0	3.18	1	0.85209	.	.	1
15726	0	3.18	1	0.85209	.	.	1
15727	0	3.18	1	0.85209	.	.	1
15728	0	3.18	1	0.85209	.	.	1
15729	0	3.18	1	0.85209	.	.	1
15730	0	3.18	1	0.85209	.	.	1
15731	0	3.18	1	0.85209	.	.	1
15732	0	3.18	1	0.85209	.	.	1
15733	0	3.18	1	0.85209	.	.	1
15734	0	3.18	1	0.85209	.	.	1
15735	0	3.18	1	0.85209	.	.	1
15736	0	3.18	1	0.85209	.	.	1
15737	0	3.18	1	0.85209	.	.	1
15738	0	3.18	1	0.85209	.	.	1
15739	0	3.18	1	0.85209	.	.	1
15740	0	3.18	1	0.85209	.	.	1
15741	0	3.18	1	0.85209	.	.	1
15742	0	3.18	1	0.85209	.	.	1
15743	0	3.18	1	0.85209	.	.	1
15744	0	3.19	0	0.85195	0.84842	0.85540	1
15745	0	3.19	1	0.85195	.	.	1
15746	0	3.19	1	0.85195	.	.	1
15747	0	3.19	1	0.85195	.	.	1
15748	0	3.19	1	0.85195	.	.	1
15749	0	3.19	1	0.85195	.	.	1
15750	0	3.19	1	0.85195	.	.	1

Obs	Sub_group	fuptimey	_CENSOR_	SURVIVAL	SDF_LCL	SDF_UCL	STRATUM
15751	0	3.19	1	0.85195	.	.	1
15752	0	3.19	1	0.85195	.	.	1
15753	0	3.19	1	0.85195	.	.	1
15754	0	3.19	1	0.85195	.	.	1
15755	0	3.19	1	0.85195	.	.	1
15756	0	3.19	1	0.85195	.	.	1
15757	0	3.19	1	0.85195	.	.	1
15758	0	3.19	1	0.85195	.	.	1
15759	0	3.19	1	0.85195	.	.	1
15760	0	3.19	1	0.85195	.	.	1
15761	0	3.19	1	0.85195	.	.	1
15762	0	3.19	1	0.85195	.	.	1
15763	0	3.19	1	0.85195	.	.	1
15764	0	3.19	1	0.85195	.	.	1
15765	0	3.19	1	0.85195	.	.	1
15766	0	3.19	1	0.85195	.	.	1
15767	0	3.19	1	0.85195	.	.	1
15768	0	3.19	1	0.85195	.	.	1
15769	0	3.19	1	0.85195	.	.	1
15770	0	3.19	1	0.85195	.	.	1
15771	0	3.19	1	0.85195	.	.	1
15772	0	3.19	1	0.85195	.	.	1
15773	0	3.19	1	0.85195	.	.	1
15774	0	3.19	1	0.85195	.	.	1
15775	0	3.19	1	0.85195	.	.	1
15776	0	3.19	1	0.85195	.	.	1
15777	0	3.19	1	0.85195	.	.	1
15778	0	3.19	1	0.85195	.	.	1
15779	0	3.19	1	0.85195	.	.	1
15780	0	3.19	1	0.85195	.	.	1
15781	0	3.19	1	0.85195	.	.	1
15782	0	3.19	1	0.85195	.	.	1
15783	0	3.19	1	0.85195	.	.	1
15784	0	3.19	1	0.85195	.	.	1
15785	0	3.19	1	0.85195	.	.	1

Obs	Sub_group	fuptimey	_CENSOR_	SURVIVAL	SDF_LCL	SDF_UCL	STRATUM
15786	0	3.19	1	0.85195	.	.	1
15787	0	3.19	1	0.85195	.	.	1
15788	0	3.19	1	0.85195	.	.	1
15789	0	3.19	1	0.85195	.	.	1
15790	0	3.19	1	0.85195	.	.	1
15791	0	3.19	1	0.85195	.	.	1
15792	0	3.19	1	0.85195	.	.	1
15793	0	3.19	1	0.85195	.	.	1
15794	0	3.19	1	0.85195	.	.	1
15795	0	3.19	1	0.85195	.	.	1
15796	0	3.19	1	0.85195	.	.	1
15797	0	3.19	1	0.85195	.	.	1
15798	0	3.19	1	0.85195	.	.	1
15799	0	3.19	1	0.85195	.	.	1
15800	0	3.19	1	0.85195	.	.	1
15801	0	3.19	1	0.85195	.	.	1
15802	0	3.20	0	0.85175	0.84822	0.85520	1
15803	0	3.20	1	0.85175	.	.	1
15804	0	3.20	1	0.85175	.	.	1
15805	0	3.20	1	0.85175	.	.	1
15806	0	3.20	1	0.85175	.	.	1
15807	0	3.20	1	0.85175	.	.	1
15808	0	3.20	1	0.85175	.	.	1
15809	0	3.20	1	0.85175	.	.	1
15810	0	3.20	1	0.85175	.	.	1
15811	0	3.20	1	0.85175	.	.	1
15812	0	3.20	1	0.85175	.	.	1
15813	0	3.20	1	0.85175	.	.	1
15814	0	3.20	1	0.85175	.	.	1
15815	0	3.20	1	0.85175	.	.	1
15816	0	3.20	1	0.85175	.	.	1
15817	0	3.20	1	0.85175	.	.	1
15818	0	3.20	1	0.85175	.	.	1
15819	0	3.20	1	0.85175	.	.	1
15820	0	3.20	1	0.85175	.	.	1

Obs	Sub_group	fuptimey	_CENSOR_	SURVIVAL	SDF_LCL	SDF_UCL	STRATUM
15821	0	3.20	1	0.85175	.	.	1
15822	0	3.20	1	0.85175	.	.	1
15823	0	3.20	1	0.85175	.	.	1
15824	0	3.20	1	0.85175	.	.	1
15825	0	3.20	1	0.85175	.	.	1
15826	0	3.20	1	0.85175	.	.	1
15827	0	3.20	1	0.85175	.	.	1
15828	0	3.20	1	0.85175	.	.	1
15829	0	3.20	1	0.85175	.	.	1
15830	0	3.20	1	0.85175	.	.	1
15831	0	3.20	1	0.85175	.	.	1
15832	0	3.20	1	0.85175	.	.	1
15833	0	3.20	1	0.85175	.	.	1
15834	0	3.20	1	0.85175	.	.	1
15835	0	3.21	0	0.85158	0.84804	0.85503	1
15836	0	3.21	1	0.85158	.	.	1
15837	0	3.21	1	0.85158	.	.	1
15838	0	3.21	1	0.85158	.	.	1
15839	0	3.21	1	0.85158	.	.	1
15840	0	3.21	1	0.85158	.	.	1
15841	0	3.21	1	0.85158	.	.	1
15842	0	3.21	1	0.85158	.	.	1
15843	0	3.21	1	0.85158	.	.	1
15844	0	3.21	1	0.85158	.	.	1
15845	0	3.21	1	0.85158	.	.	1
15846	0	3.21	1	0.85158	.	.	1
15847	0	3.21	1	0.85158	.	.	1
15848	0	3.21	1	0.85158	.	.	1
15849	0	3.21	1	0.85158	.	.	1
15850	0	3.21	1	0.85158	.	.	1
15851	0	3.21	1	0.85158	.	.	1
15852	0	3.21	1	0.85158	.	.	1
15853	0	3.21	1	0.85158	.	.	1
15854	0	3.21	1	0.85158	.	.	1
15855	0	3.21	1	0.85158	.	.	1

Obs	Sub_group	fuptimey	_CENSOR_	SURVIVAL	SDF_LCL	SDF_UCL	STRATUM
15856	0	3.21	1	0.85158	.	.	1
15857	0	3.21	1	0.85158	.	.	1
15858	0	3.21	1	0.85158	.	.	1
15859	0	3.21	1	0.85158	.	.	1
15860	0	3.21	1	0.85158	.	.	1
15861	0	3.21	1	0.85158	.	.	1
15862	0	3.21	1	0.85158	.	.	1
15863	0	3.21	1	0.85158	.	.	1
15864	0	3.21	1	0.85158	.	.	1
15865	0	3.21	1	0.85158	.	.	1
15866	0	3.21	1	0.85158	.	.	1
15867	0	3.21	1	0.85158	.	.	1
15868	0	3.21	1	0.85158	.	.	1
15869	0	3.21	1	0.85158	.	.	1
15870	0	3.21	1	0.85158	.	.	1
15871	0	3.21	1	0.85158	.	.	1
15872	0	3.21	1	0.85158	.	.	1
15873	0	3.21	1	0.85158	.	.	1
15874	0	3.21	1	0.85158	.	.	1
15875	0	3.21	1	0.85158	.	.	1
15876	0	3.21	1	0.85158	.	.	1
15877	0	3.21	1	0.85158	.	.	1
15878	0	3.21	1	0.85158	.	.	1
15879	0	3.21	1	0.85158	.	.	1
15880	0	3.21	1	0.85158	.	.	1
15881	0	3.22	0	0.85120	0.84766	0.85466	1
15882	0	3.22	1	0.85120	.	.	1
15883	0	3.22	1	0.85120	.	.	1
15884	0	3.22	1	0.85120	.	.	1
15885	0	3.22	1	0.85120	.	.	1
15886	0	3.22	1	0.85120	.	.	1
15887	0	3.22	1	0.85120	.	.	1
15888	0	3.22	1	0.85120	.	.	1
15889	0	3.22	1	0.85120	.	.	1
15890	0	3.22	1	0.85120	.	.	1

Obs	Sub_group	fuptimey	_CENSOR_	SURVIVAL	SDF_LCL	SDF_UCL	STRATUM
15891	0	3.22	1	0.85120	.	.	1
15892	0	3.22	1	0.85120	.	.	1
15893	0	3.22	1	0.85120	.	.	1
15894	0	3.22	1	0.85120	.	.	1
15895	0	3.22	1	0.85120	.	.	1
15896	0	3.22	1	0.85120	.	.	1
15897	0	3.22	1	0.85120	.	.	1
15898	0	3.22	1	0.85120	.	.	1
15899	0	3.22	1	0.85120	.	.	1
15900	0	3.22	1	0.85120	.	.	1
15901	0	3.22	1	0.85120	.	.	1
15902	0	3.22	1	0.85120	.	.	1
15903	0	3.22	1	0.85120	.	.	1
15904	0	3.22	1	0.85120	.	.	1
15905	0	3.22	1	0.85120	.	.	1
15906	0	3.22	1	0.85120	.	.	1
15907	0	3.22	1	0.85120	.	.	1
15908	0	3.22	1	0.85120	.	.	1
15909	0	3.22	1	0.85120	.	.	1
15910	0	3.22	1	0.85120	.	.	1
15911	0	3.22	1	0.85120	.	.	1
15912	0	3.22	1	0.85120	.	.	1
15913	0	3.22	1	0.85120	.	.	1
15914	0	3.22	1	0.85120	.	.	1
15915	0	3.22	1	0.85120	.	.	1
15916	0	3.22	1	0.85120	.	.	1
15917	0	3.22	1	0.85120	.	.	1
15918	0	3.22	1	0.85120	.	.	1
15919	0	3.22	1	0.85120	.	.	1
15920	0	3.22	1	0.85120	.	.	1
15921	0	3.23	0	0.85093	0.84739	0.85440	1
15922	0	3.23	1	0.85093	.	.	1
15923	0	3.23	1	0.85093	.	.	1
15924	0	3.23	1	0.85093	.	.	1
15925	0	3.23	1	0.85093	.	.	1

Obs	Sub_group	fuptimey	_CENSOR_	SURVIVAL	SDF_LCL	SDF_UCL	STRATUM
15926	0	3.23	1	0.85093	.	.	1
15927	0	3.23	1	0.85093	.	.	1
15928	0	3.23	1	0.85093	.	.	1
15929	0	3.23	1	0.85093	.	.	1
15930	0	3.23	1	0.85093	.	.	1
15931	0	3.23	1	0.85093	.	.	1
15932	0	3.23	1	0.85093	.	.	1
15933	0	3.23	1	0.85093	.	.	1
15934	0	3.23	1	0.85093	.	.	1
15935	0	3.23	1	0.85093	.	.	1
15936	0	3.23	1	0.85093	.	.	1
15937	0	3.23	1	0.85093	.	.	1
15938	0	3.23	1	0.85093	.	.	1
15939	0	3.23	1	0.85093	.	.	1
15940	0	3.23	1	0.85093	.	.	1
15941	0	3.23	1	0.85093	.	.	1
15942	0	3.23	1	0.85093	.	.	1
15943	0	3.23	1	0.85093	.	.	1
15944	0	3.23	1	0.85093	.	.	1
15945	0	3.23	1	0.85093	.	.	1
15946	0	3.23	1	0.85093	.	.	1
15947	0	3.23	1	0.85093	.	.	1
15948	0	3.23	1	0.85093	.	.	1
15949	0	3.23	1	0.85093	.	.	1
15950	0	3.23	1	0.85093	.	.	1
15951	0	3.23	1	0.85093	.	.	1
15952	0	3.23	1	0.85093	.	.	1
15953	0	3.23	1	0.85093	.	.	1
15954	0	3.23	1	0.85093	.	.	1
15955	0	3.23	1	0.85093	.	.	1
15956	0	3.23	1	0.85093	.	.	1
15957	0	3.23	1	0.85093	.	.	1
15958	0	3.23	1	0.85093	.	.	1
15959	0	3.23	1	0.85093	.	.	1
15960	0	3.23	1	0.85093	.	.	1

Obs	Sub_group	fuptimey	_CENSOR_	SURVIVAL	SDF_LCL	SDF_UCL	STRATUM
15961	0	3.23	1	0.85093	.	.	1
15962	0	3.23	1	0.85093	.	.	1
15963	0	3.23	1	0.85093	.	.	1
15964	0	3.23	1	0.85093	.	.	1
15965	0	3.23	1	0.85093	.	.	1
15966	0	3.24	0	0.85076	0.84721	0.85423	1
15967	0	3.24	1	0.85076	.	.	1
15968	0	3.24	1	0.85076	.	.	1
15969	0	3.24	1	0.85076	.	.	1
15970	0	3.24	1	0.85076	.	.	1
15971	0	3.24	1	0.85076	.	.	1
15972	0	3.24	1	0.85076	.	.	1
15973	0	3.24	1	0.85076	.	.	1
15974	0	3.24	1	0.85076	.	.	1
15975	0	3.24	1	0.85076	.	.	1
15976	0	3.24	1	0.85076	.	.	1
15977	0	3.24	1	0.85076	.	.	1
15978	0	3.24	1	0.85076	.	.	1
15979	0	3.24	1	0.85076	.	.	1
15980	0	3.24	1	0.85076	.	.	1
15981	0	3.24	1	0.85076	.	.	1
15982	0	3.24	1	0.85076	.	.	1
15983	0	3.24	1	0.85076	.	.	1
15984	0	3.24	1	0.85076	.	.	1
15985	0	3.24	1	0.85076	.	.	1
15986	0	3.24	1	0.85076	.	.	1
15987	0	3.24	1	0.85076	.	.	1
15988	0	3.24	1	0.85076	.	.	1
15989	0	3.24	1	0.85076	.	.	1
15990	0	3.24	1	0.85076	.	.	1
15991	0	3.24	1	0.85076	.	.	1
15992	0	3.24	1	0.85076	.	.	1
15993	0	3.24	1	0.85076	.	.	1
15994	0	3.24	1	0.85076	.	.	1
15995	0	3.24	1	0.85076	.	.	1

Obs	Sub_group	fuptimey	_CENSOR_	SURVIVAL	SDF_LCL	SDF_UCL	STRATUM
15996	0	3.24	1	0.85076	.	.	1
15997	0	3.24	1	0.85076	.	.	1
15998	0	3.24	1	0.85076	.	.	1
15999	0	3.24	1	0.85076	.	.	1
16000	0	3.24	1	0.85076	.	.	1
16001	0	3.24	1	0.85076	.	.	1
16002	0	3.24	1	0.85076	.	.	1
16003	0	3.24	1	0.85076	.	.	1
16004	0	3.24	1	0.85076	.	.	1
16005	0	3.24	1	0.85076	.	.	1
16006	0	3.24	1	0.85076	.	.	1
16007	0	3.25	0	0.85042	0.84687	0.85389	1
16008	0	3.25	1	0.85042	.	.	1
16009	0	3.25	1	0.85042	.	.	1
16010	0	3.25	1	0.85042	.	.	1
16011	0	3.25	1	0.85042	.	.	1
16012	0	3.25	1	0.85042	.	.	1
16013	0	3.25	1	0.85042	.	.	1
16014	0	3.25	1	0.85042	.	.	1
16015	0	3.25	1	0.85042	.	.	1
16016	0	3.25	1	0.85042	.	.	1
16017	0	3.25	1	0.85042	.	.	1
16018	0	3.25	1	0.85042	.	.	1
16019	0	3.25	1	0.85042	.	.	1
16020	0	3.25	1	0.85042	.	.	1
16021	0	3.25	1	0.85042	.	.	1
16022	0	3.25	1	0.85042	.	.	1
16023	0	3.25	1	0.85042	.	.	1
16024	0	3.25	1	0.85042	.	.	1
16025	0	3.25	1	0.85042	.	.	1
16026	0	3.25	1	0.85042	.	.	1
16027	0	3.25	1	0.85042	.	.	1
16028	0	3.25	1	0.85042	.	.	1
16029	0	3.25	1	0.85042	.	.	1
16030	0	3.25	1	0.85042	.	.	1

Obs	Sub_group	fuptimey	_CENSOR_	SURVIVAL	SDF_LCL	SDF_UCL	STRATUM
16031	0	3.25	1	0.85042	.	.	1
16032	0	3.25	1	0.85042	.	.	1
16033	0	3.25	1	0.85042	.	.	1
16034	0	3.25	1	0.85042	.	.	1
16035	0	3.25	1	0.85042	.	.	1
16036	0	3.25	1	0.85042	.	.	1
16037	0	3.25	1	0.85042	.	.	1
16038	0	3.25	1	0.85042	.	.	1
16039	0	3.25	1	0.85042	.	.	1
16040	0	3.25	1	0.85042	.	.	1
16041	0	3.25	1	0.85042	.	.	1
16042	0	3.26	0	0.85021	0.84666	0.85369	1
16043	0	3.26	1	0.85021	.	.	1
16044	0	3.26	1	0.85021	.	.	1
16045	0	3.26	1	0.85021	.	.	1
16046	0	3.26	1	0.85021	.	.	1
16047	0	3.26	1	0.85021	.	.	1
16048	0	3.26	1	0.85021	.	.	1
16049	0	3.26	1	0.85021	.	.	1
16050	0	3.26	1	0.85021	.	.	1
16051	0	3.26	1	0.85021	.	.	1
16052	0	3.26	1	0.85021	.	.	1
16053	0	3.26	1	0.85021	.	.	1
16054	0	3.26	1	0.85021	.	.	1
16055	0	3.26	1	0.85021	.	.	1
16056	0	3.26	1	0.85021	.	.	1
16057	0	3.26	1	0.85021	.	.	1
16058	0	3.26	1	0.85021	.	.	1
16059	0	3.26	1	0.85021	.	.	1
16060	0	3.26	1	0.85021	.	.	1
16061	0	3.26	1	0.85021	.	.	1
16062	0	3.26	1	0.85021	.	.	1
16063	0	3.26	1	0.85021	.	.	1
16064	0	3.26	1	0.85021	.	.	1
16065	0	3.26	1	0.85021	.	.	1

Obs	Sub_group	fuptimey	_CENSOR_	SURVIVAL	SDF_LCL	SDF_UCL	STRATUM
16066	0	3.26	1	0.85021	.	.	1
16067	0	3.26	1	0.85021	.	.	1
16068	0	3.26	1	0.85021	.	.	1
16069	0	3.26	1	0.85021	.	.	1
16070	0	3.26	1	0.85021	.	.	1
16071	0	3.26	1	0.85021	.	.	1
16072	0	3.26	1	0.85021	.	.	1
16073	0	3.26	1	0.85021	.	.	1
16074	0	3.26	1	0.85021	.	.	1
16075	0	3.26	1	0.85021	.	.	1
16076	0	3.26	1	0.85021	.	.	1
16077	0	3.26	1	0.85021	.	.	1
16078	0	3.26	1	0.85021	.	.	1
16079	0	3.26	1	0.85021	.	.	1
16080	0	3.26	1	0.85021	.	.	1
16081	0	3.26	1	0.85021	.	.	1
16082	0	3.27	0	0.85007	0.84652	0.85355	1
16083	0	3.27	1	0.85007	.	.	1
16084	0	3.27	1	0.85007	.	.	1
16085	0	3.27	1	0.85007	.	.	1
16086	0	3.27	1	0.85007	.	.	1
16087	0	3.27	1	0.85007	.	.	1
16088	0	3.27	1	0.85007	.	.	1
16089	0	3.27	1	0.85007	.	.	1
16090	0	3.27	1	0.85007	.	.	1
16091	0	3.27	1	0.85007	.	.	1
16092	0	3.27	1	0.85007	.	.	1
16093	0	3.27	1	0.85007	.	.	1
16094	0	3.27	1	0.85007	.	.	1
16095	0	3.27	1	0.85007	.	.	1
16096	0	3.27	1	0.85007	.	.	1
16097	0	3.27	1	0.85007	.	.	1
16098	0	3.27	1	0.85007	.	.	1
16099	0	3.27	1	0.85007	.	.	1
16100	0	3.27	1	0.85007	.	.	1

Obs	Sub_group	fuptimey	_CENSOR_	SURVIVAL	SDF_LCL	SDF_UCL	STRATUM
16101	0	3.27	1	0.85007	.	.	1
16102	0	3.27	1	0.85007	.	.	1
16103	0	3.27	1	0.85007	.	.	1
16104	0	3.27	1	0.85007	.	.	1
16105	0	3.27	1	0.85007	.	.	1
16106	0	3.27	1	0.85007	.	.	1
16107	0	3.27	1	0.85007	.	.	1
16108	0	3.27	1	0.85007	.	.	1
16109	0	3.27	1	0.85007	.	.	1
16110	0	3.27	1	0.85007	.	.	1
16111	0	3.27	1	0.85007	.	.	1
16112	0	3.27	1	0.85007	.	.	1
16113	0	3.27	1	0.85007	.	.	1
16114	0	3.27	1	0.85007	.	.	1
16115	0	3.27	1	0.85007	.	.	1
16116	0	3.27	1	0.85007	.	.	1
16117	0	3.27	1	0.85007	.	.	1
16118	0	3.27	1	0.85007	.	.	1
16119	0	3.27	1	0.85007	.	.	1
16120	0	3.27	1	0.85007	.	.	1
16121	0	3.27	1	0.85007	.	.	1
16122	0	3.27	1	0.85007	.	.	1
16123	0	3.27	1	0.85007	.	.	1
16124	0	3.27	1	0.85007	.	.	1
16125	0	3.27	1	0.85007	.	.	1
16126	0	3.27	1	0.85007	.	.	1
16127	0	3.28	0	0.84994	0.84638	0.85342	1
16128	0	3.28	1	0.84994	.	.	1
16129	0	3.28	1	0.84994	.	.	1
16130	0	3.28	1	0.84994	.	.	1
16131	0	3.28	1	0.84994	.	.	1
16132	0	3.28	1	0.84994	.	.	1
16133	0	3.28	1	0.84994	.	.	1
16134	0	3.28	1	0.84994	.	.	1
16135	0	3.28	1	0.84994	.	.	1

Obs	Sub_group	fuptimey	_CENSOR_	SURVIVAL	SDF_LCL	SDF_UCL	STRATUM
16136	0	3.28	1	0.84994	.	.	1
16137	0	3.28	1	0.84994	.	.	1
16138	0	3.28	1	0.84994	.	.	1
16139	0	3.28	1	0.84994	.	.	1
16140	0	3.28	1	0.84994	.	.	1
16141	0	3.28	1	0.84994	.	.	1
16142	0	3.28	1	0.84994	.	.	1
16143	0	3.28	1	0.84994	.	.	1
16144	0	3.28	1	0.84994	.	.	1
16145	0	3.28	1	0.84994	.	.	1
16146	0	3.28	1	0.84994	.	.	1
16147	0	3.28	1	0.84994	.	.	1
16148	0	3.28	1	0.84994	.	.	1
16149	0	3.28	1	0.84994	.	.	1
16150	0	3.28	1	0.84994	.	.	1
16151	0	3.28	1	0.84994	.	.	1
16152	0	3.28	1	0.84994	.	.	1
16153	0	3.28	1	0.84994	.	.	1
16154	0	3.28	1	0.84994	.	.	1
16155	0	3.28	1	0.84994	.	.	1
16156	0	3.28	1	0.84994	.	.	1
16157	0	3.28	1	0.84994	.	.	1
16158	0	3.28	1	0.84994	.	.	1
16159	0	3.28	1	0.84994	.	.	1
16160	0	3.28	1	0.84994	.	.	1
16161	0	3.28	1	0.84994	.	.	1
16162	0	3.28	1	0.84994	.	.	1
16163	0	3.28	1	0.84994	.	.	1
16164	0	3.28	1	0.84994	.	.	1
16165	0	3.28	1	0.84994	.	.	1
16166	0	3.28	1	0.84994	.	.	1
16167	0	3.28	1	0.84994	.	.	1
16168	0	3.28	1	0.84994	.	.	1
16169	0	3.28	1	0.84994	.	.	1
16170	0	3.28	1	0.84994	.	.	1

Obs	Sub_group	fuptimey	_CENSOR_	SURVIVAL	SDF_LCL	SDF_UCL	STRATUM
16171	0	3.28	1	0.84994	.	.	1
16172	0	3.28	1	0.84994	.	.	1
16173	0	3.28	1	0.84994	.	.	1
16174	0	3.28	1	0.84994	.	.	1
16175	0	3.28	1	0.84994	.	.	1
16176	0	3.29	0	0.84963	0.84607	0.85311	1
16177	0	3.29	1	0.84963	.	.	1
16178	0	3.29	1	0.84963	.	.	1
16179	0	3.29	1	0.84963	.	.	1
16180	0	3.29	1	0.84963	.	.	1
16181	0	3.29	1	0.84963	.	.	1
16182	0	3.29	1	0.84963	.	.	1
16183	0	3.29	1	0.84963	.	.	1
16184	0	3.29	1	0.84963	.	.	1
16185	0	3.29	1	0.84963	.	.	1
16186	0	3.29	1	0.84963	.	.	1
16187	0	3.29	1	0.84963	.	.	1
16188	0	3.29	1	0.84963	.	.	1
16189	0	3.29	1	0.84963	.	.	1
16190	0	3.29	1	0.84963	.	.	1
16191	0	3.29	1	0.84963	.	.	1
16192	0	3.29	1	0.84963	.	.	1
16193	0	3.29	1	0.84963	.	.	1
16194	0	3.29	1	0.84963	.	.	1
16195	0	3.29	1	0.84963	.	.	1
16196	0	3.29	1	0.84963	.	.	1
16197	0	3.29	1	0.84963	.	.	1
16198	0	3.29	1	0.84963	.	.	1
16199	0	3.29	1	0.84963	.	.	1
16200	0	3.29	1	0.84963	.	.	1
16201	0	3.29	1	0.84963	.	.	1
16202	0	3.29	1	0.84963	.	.	1
16203	0	3.29	1	0.84963	.	.	1
16204	0	3.29	1	0.84963	.	.	1
16205	0	3.29	1	0.84963	.	.	1

Obs	Sub_group	fuptimey	_CENSOR_	SURVIVAL	SDF_LCL	SDF_UCL	STRATUM
16206	0	3.29	1	0.84963	.	.	1
16207	0	3.29	1	0.84963	.	.	1
16208	0	3.29	1	0.84963	.	.	1
16209	0	3.29	1	0.84963	.	.	1
16210	0	3.29	1	0.84963	.	.	1
16211	0	3.29	1	0.84963	.	.	1
16212	0	3.29	1	0.84963	.	.	1
16213	0	3.29	1	0.84963	.	.	1
16214	0	3.29	1	0.84963	.	.	1
16215	0	3.29	1	0.84963	.	.	1
16216	0	3.29	1	0.84963	.	.	1
16217	0	3.30	0	0.84952	0.84596	0.85301	1
16218	0	3.30	1	0.84952	.	.	1
16219	0	3.30	1	0.84952	.	.	1
16220	0	3.30	1	0.84952	.	.	1
16221	0	3.30	1	0.84952	.	.	1
16222	0	3.30	1	0.84952	.	.	1
16223	0	3.30	1	0.84952	.	.	1
16224	0	3.30	1	0.84952	.	.	1
16225	0	3.30	1	0.84952	.	.	1
16226	0	3.30	1	0.84952	.	.	1
16227	0	3.30	1	0.84952	.	.	1
16228	0	3.30	1	0.84952	.	.	1
16229	0	3.30	1	0.84952	.	.	1
16230	0	3.30	1	0.84952	.	.	1
16231	0	3.30	1	0.84952	.	.	1
16232	0	3.30	1	0.84952	.	.	1
16233	0	3.30	1	0.84952	.	.	1
16234	0	3.30	1	0.84952	.	.	1
16235	0	3.30	1	0.84952	.	.	1
16236	0	3.30	1	0.84952	.	.	1
16237	0	3.30	1	0.84952	.	.	1
16238	0	3.30	1	0.84952	.	.	1
16239	0	3.30	1	0.84952	.	.	1
16240	0	3.30	1	0.84952	.	.	1

Obs	Sub_group	fuptimey	_CENSOR_	SURVIVAL	SDF_LCL	SDF_UCL	STRATUM
16241	0	3.30	1	0.84952	.	.	1
16242	0	3.30	1	0.84952	.	.	1
16243	0	3.30	1	0.84952	.	.	1
16244	0	3.30	1	0.84952	.	.	1
16245	0	3.30	1	0.84952	.	.	1
16246	0	3.30	1	0.84952	.	.	1
16247	0	3.30	1	0.84952	.	.	1
16248	0	3.30	1	0.84952	.	.	1
16249	0	3.30	1	0.84952	.	.	1
16250	0	3.30	1	0.84952	.	.	1
16251	0	3.30	1	0.84952	.	.	1
16252	0	3.30	1	0.84952	.	.	1
16253	0	3.30	1	0.84952	.	.	1
16254	0	3.30	1	0.84952	.	.	1
16255	0	3.30	1	0.84952	.	.	1
16256	0	3.30	1	0.84952	.	.	1
16257	0	3.30	1	0.84952	.	.	1
16258	0	3.30	1	0.84952	.	.	1
16259	0	3.30	1	0.84952	.	.	1
16260	0	3.31	0	0.84928	0.84571	0.85277	1
16261	0	3.31	1	0.84928	.	.	1
16262	0	3.31	1	0.84928	.	.	1
16263	0	3.31	1	0.84928	.	.	1
16264	0	3.31	1	0.84928	.	.	1
16265	0	3.31	1	0.84928	.	.	1
16266	0	3.31	1	0.84928	.	.	1
16267	0	3.31	1	0.84928	.	.	1
16268	0	3.31	1	0.84928	.	.	1
16269	0	3.31	1	0.84928	.	.	1
16270	0	3.31	1	0.84928	.	.	1
16271	0	3.31	1	0.84928	.	.	1
16272	0	3.31	1	0.84928	.	.	1
16273	0	3.31	1	0.84928	.	.	1
16274	0	3.31	1	0.84928	.	.	1
16275	0	3.31	1	0.84928	.	.	1

Obs	Sub_group	fuptimey	_CENSOR_	SURVIVAL	SDF_LCL	SDF_UCL	STRATUM
16276	0	3.31	1	0.84928	.	.	1
16277	0	3.31	1	0.84928	.	.	1
16278	0	3.31	1	0.84928	.	.	1
16279	0	3.31	1	0.84928	.	.	1
16280	0	3.31	1	0.84928	.	.	1
16281	0	3.31	1	0.84928	.	.	1
16282	0	3.31	1	0.84928	.	.	1
16283	0	3.31	1	0.84928	.	.	1
16284	0	3.31	1	0.84928	.	.	1
16285	0	3.31	1	0.84928	.	.	1
16286	0	3.31	1	0.84928	.	.	1
16287	0	3.31	1	0.84928	.	.	1
16288	0	3.31	1	0.84928	.	.	1
16289	0	3.31	1	0.84928	.	.	1
16290	0	3.31	1	0.84928	.	.	1
16291	0	3.31	1	0.84928	.	.	1
16292	0	3.31	1	0.84928	.	.	1
16293	0	3.31	1	0.84928	.	.	1
16294	0	3.32	0	0.84907	0.84550	0.85257	1
16295	0	3.32	1	0.84907	.	.	1
16296	0	3.32	1	0.84907	.	.	1
16297	0	3.32	1	0.84907	.	.	1
16298	0	3.32	1	0.84907	.	.	1
16299	0	3.32	1	0.84907	.	.	1
16300	0	3.32	1	0.84907	.	.	1
16301	0	3.32	1	0.84907	.	.	1
16302	0	3.32	1	0.84907	.	.	1
16303	0	3.32	1	0.84907	.	.	1
16304	0	3.32	1	0.84907	.	.	1
16305	0	3.32	1	0.84907	.	.	1
16306	0	3.32	1	0.84907	.	.	1
16307	0	3.32	1	0.84907	.	.	1
16308	0	3.32	1	0.84907	.	.	1
16309	0	3.32	1	0.84907	.	.	1
16310	0	3.32	1	0.84907	.	.	1

Obs	Sub_group	fuptimey	_CENSOR_	SURVIVAL	SDF_LCL	SDF_UCL	STRATUM
16311	0	3.32	1	0.84907	.	.	1
16312	0	3.32	1	0.84907	.	.	1
16313	0	3.32	1	0.84907	.	.	1
16314	0	3.32	1	0.84907	.	.	1
16315	0	3.32	1	0.84907	.	.	1
16316	0	3.32	1	0.84907	.	.	1
16317	0	3.32	1	0.84907	.	.	1
16318	0	3.32	1	0.84907	.	.	1
16319	0	3.32	1	0.84907	.	.	1
16320	0	3.32	1	0.84907	.	.	1
16321	0	3.32	1	0.84907	.	.	1
16322	0	3.32	1	0.84907	.	.	1
16323	0	3.32	1	0.84907	.	.	1
16324	0	3.32	1	0.84907	.	.	1
16325	0	3.32	1	0.84907	.	.	1
16326	0	3.32	1	0.84907	.	.	1
16327	0	3.32	1	0.84907	.	.	1
16328	0	3.32	1	0.84907	.	.	1
16329	0	3.32	1	0.84907	.	.	1
16330	0	3.32	1	0.84907	.	.	1
16331	0	3.32	1	0.84907	.	.	1
16332	0	3.32	1	0.84907	.	.	1
16333	0	3.32	1	0.84907	.	.	1
16334	0	3.32	1	0.84907	.	.	1
16335	0	3.32	1	0.84907	.	.	1
16336	0	3.32	1	0.84907	.	.	1
16337	0	3.32	1	0.84907	.	.	1
16338	0	3.32	1	0.84907	.	.	1
16339	0	3.32	1	0.84907	.	.	1
16340	0	3.32	1	0.84907	.	.	1
16341	0	3.32	1	0.84907	.	.	1
16342	0	3.32	1	0.84907	.	.	1
16343	0	3.32	1	0.84907	.	.	1
16344	0	3.32	1	0.84907	.	.	1
16345	0	3.33	0	0.84890	0.84533	0.85239	1

Obs	Sub_group	fuptimey	_CENSOR_	SURVIVAL	SDF_LCL	SDF_UCL	STRATUM
16346	0	3.33	1	0.84890	.	.	1
16347	0	3.33	1	0.84890	.	.	1
16348	0	3.33	1	0.84890	.	.	1
16349	0	3.33	1	0.84890	.	.	1
16350	0	3.33	1	0.84890	.	.	1
16351	0	3.33	1	0.84890	.	.	1
16352	0	3.33	1	0.84890	.	.	1
16353	0	3.33	1	0.84890	.	.	1
16354	0	3.33	1	0.84890	.	.	1
16355	0	3.33	1	0.84890	.	.	1
16356	0	3.33	1	0.84890	.	.	1
16357	0	3.33	1	0.84890	.	.	1
16358	0	3.33	1	0.84890	.	.	1
16359	0	3.33	1	0.84890	.	.	1
16360	0	3.33	1	0.84890	.	.	1
16361	0	3.33	1	0.84890	.	.	1
16362	0	3.33	1	0.84890	.	.	1
16363	0	3.33	1	0.84890	.	.	1
16364	0	3.33	1	0.84890	.	.	1
16365	0	3.33	1	0.84890	.	.	1
16366	0	3.33	1	0.84890	.	.	1
16367	0	3.33	1	0.84890	.	.	1
16368	0	3.33	1	0.84890	.	.	1
16369	0	3.33	1	0.84890	.	.	1
16370	0	3.33	1	0.84890	.	.	1
16371	0	3.33	1	0.84890	.	.	1
16372	0	3.33	1	0.84890	.	.	1
16373	0	3.33	1	0.84890	.	.	1
16374	0	3.33	1	0.84890	.	.	1
16375	0	3.33	1	0.84890	.	.	1
16376	0	3.33	1	0.84890	.	.	1
16377	0	3.33	1	0.84890	.	.	1
16378	0	3.33	1	0.84890	.	.	1
16379	0	3.33	1	0.84890	.	.	1
16380	0	3.33	1	0.84890	.	.	1

Obs	Sub_group	fuptimey	_CENSOR_	SURVIVAL	SDF_LCL	SDF_UCL	STRATUM
16381	0	3.33	1	0.84890	.	.	1
16382	0	3.33	1	0.84890	.	.	1
16383	0	3.33	1	0.84890	.	.	1
16384	0	3.33	1	0.84890	.	.	1
16385	0	3.34	0	0.84852	0.84494	0.85202	1
16386	0	3.34	1	0.84852	.	.	1
16387	0	3.34	1	0.84852	.	.	1
16388	0	3.34	1	0.84852	.	.	1
16389	0	3.34	1	0.84852	.	.	1
16390	0	3.34	1	0.84852	.	.	1
16391	0	3.34	1	0.84852	.	.	1
16392	0	3.34	1	0.84852	.	.	1
16393	0	3.34	1	0.84852	.	.	1
16394	0	3.34	1	0.84852	.	.	1
16395	0	3.34	1	0.84852	.	.	1
16396	0	3.34	1	0.84852	.	.	1
16397	0	3.34	1	0.84852	.	.	1
16398	0	3.34	1	0.84852	.	.	1
16399	0	3.34	1	0.84852	.	.	1
16400	0	3.34	1	0.84852	.	.	1
16401	0	3.34	1	0.84852	.	.	1
16402	0	3.34	1	0.84852	.	.	1
16403	0	3.34	1	0.84852	.	.	1
16404	0	3.34	1	0.84852	.	.	1
16405	0	3.34	1	0.84852	.	.	1
16406	0	3.34	1	0.84852	.	.	1
16407	0	3.34	1	0.84852	.	.	1
16408	0	3.34	1	0.84852	.	.	1
16409	0	3.34	1	0.84852	.	.	1
16410	0	3.34	1	0.84852	.	.	1
16411	0	3.34	1	0.84852	.	.	1
16412	0	3.34	1	0.84852	.	.	1
16413	0	3.34	1	0.84852	.	.	1
16414	0	3.34	1	0.84852	.	.	1
16415	0	3.34	1	0.84852	.	.	1

Obs	Sub_group	fuptimey	_CENSOR_	SURVIVAL	SDF_LCL	SDF_UCL	STRATUM
16416	0	3.34	1	0.84852	.	.	1
16417	0	3.34	1	0.84852	.	.	1
16418	0	3.34	1	0.84852	.	.	1
16419	0	3.34	1	0.84852	.	.	1
16420	0	3.34	1	0.84852	.	.	1
16421	0	3.34	1	0.84852	.	.	1
16422	0	3.34	1	0.84852	.	.	1
16423	0	3.34	1	0.84852	.	.	1
16424	0	3.34	1	0.84852	.	.	1
16425	0	3.34	1	0.84852	.	.	1
16426	0	3.34	1	0.84852	.	.	1
16427	0	3.34	1	0.84852	.	.	1
16428	0	3.34	1	0.84852	.	.	1
16429	0	3.34	1	0.84852	.	.	1
16430	0	3.34	1	0.84852	.	.	1
16431	0	3.34	1	0.84852	.	.	1
16432	0	3.34	1	0.84852	.	.	1
16433	0	3.34	1	0.84852	.	.	1
16434	0	3.34	1	0.84852	.	.	1
16435	0	3.34	1	0.84852	.	.	1
16436	0	3.34	1	0.84852	.	.	1
16437	0	3.34	1	0.84852	.	.	1
16438	0	3.34	1	0.84852	.	.	1
16439	0	3.34	1	0.84852	.	.	1
16440	0	3.34	1	0.84852	.	.	1
16441	0	3.34	1	0.84852	.	.	1
16442	0	3.35	0	0.84827	0.84469	0.85178	1
16443	0	3.35	1	0.84827	.	.	1
16444	0	3.35	1	0.84827	.	.	1
16445	0	3.35	1	0.84827	.	.	1
16446	0	3.35	1	0.84827	.	.	1
16447	0	3.35	1	0.84827	.	.	1
16448	0	3.35	1	0.84827	.	.	1
16449	0	3.35	1	0.84827	.	.	1
16450	0	3.35	1	0.84827	.	.	1

Obs	Sub_group	fuptimey	_CENSOR_	SURVIVAL	SDF_LCL	SDF_UCL	STRATUM
16451	0	3.35	1	0.84827	.	.	1
16452	0	3.35	1	0.84827	.	.	1
16453	0	3.35	1	0.84827	.	.	1
16454	0	3.35	1	0.84827	.	.	1
16455	0	3.35	1	0.84827	.	.	1
16456	0	3.35	1	0.84827	.	.	1
16457	0	3.35	1	0.84827	.	.	1
16458	0	3.35	1	0.84827	.	.	1
16459	0	3.35	1	0.84827	.	.	1
16460	0	3.35	1	0.84827	.	.	1
16461	0	3.35	1	0.84827	.	.	1
16462	0	3.35	1	0.84827	.	.	1
16463	0	3.35	1	0.84827	.	.	1
16464	0	3.35	1	0.84827	.	.	1
16465	0	3.35	1	0.84827	.	.	1
16466	0	3.35	1	0.84827	.	.	1
16467	0	3.35	1	0.84827	.	.	1
16468	0	3.35	1	0.84827	.	.	1
16469	0	3.35	1	0.84827	.	.	1
16470	0	3.35	1	0.84827	.	.	1
16471	0	3.35	1	0.84827	.	.	1
16472	0	3.35	1	0.84827	.	.	1
16473	0	3.36	0	0.84803	0.84445	0.85154	1
16474	0	3.36	1	0.84803	.	.	1
16475	0	3.36	1	0.84803	.	.	1
16476	0	3.36	1	0.84803	.	.	1
16477	0	3.36	1	0.84803	.	.	1
16478	0	3.36	1	0.84803	.	.	1
16479	0	3.36	1	0.84803	.	.	1
16480	0	3.36	1	0.84803	.	.	1
16481	0	3.36	1	0.84803	.	.	1
16482	0	3.36	1	0.84803	.	.	1
16483	0	3.36	1	0.84803	.	.	1
16484	0	3.36	1	0.84803	.	.	1
16485	0	3.36	1	0.84803	.	.	1

Obs	Sub_group	fuptimey	_CENSOR_	SURVIVAL	SDF_LCL	SDF_UCL	STRATUM
16486	0	3.36	1	0.84803	.	.	1
16487	0	3.36	1	0.84803	.	.	1
16488	0	3.36	1	0.84803	.	.	1
16489	0	3.36	1	0.84803	.	.	1
16490	0	3.36	1	0.84803	.	.	1
16491	0	3.36	1	0.84803	.	.	1
16492	0	3.36	1	0.84803	.	.	1
16493	0	3.36	1	0.84803	.	.	1
16494	0	3.36	1	0.84803	.	.	1
16495	0	3.36	1	0.84803	.	.	1
16496	0	3.36	1	0.84803	.	.	1
16497	0	3.36	1	0.84803	.	.	1
16498	0	3.36	1	0.84803	.	.	1
16499	0	3.36	1	0.84803	.	.	1
16500	0	3.36	1	0.84803	.	.	1
16501	0	3.36	1	0.84803	.	.	1
16502	0	3.36	1	0.84803	.	.	1
16503	0	3.36	1	0.84803	.	.	1
16504	0	3.36	1	0.84803	.	.	1
16505	0	3.36	1	0.84803	.	.	1
16506	0	3.36	1	0.84803	.	.	1
16507	0	3.36	1	0.84803	.	.	1
16508	0	3.36	1	0.84803	.	.	1
16509	0	3.36	1	0.84803	.	.	1
16510	0	3.36	1	0.84803	.	.	1
16511	0	3.36	1	0.84803	.	.	1
16512	0	3.36	1	0.84803	.	.	1
16513	0	3.36	1	0.84803	.	.	1
16514	0	3.36	1	0.84803	.	.	1
16515	0	3.36	1	0.84803	.	.	1
16516	0	3.36	1	0.84803	.	.	1
16517	0	3.36	1	0.84803	.	.	1
16518	0	3.37	0	0.84771	0.84413	0.85123	1
16519	0	3.37	1	0.84771	.	.	1
16520	0	3.37	1	0.84771	.	.	1

Obs	Sub_group	fuptimey	_CENSOR_	SURVIVAL	SDF_LCL	SDF_UCL	STRATUM
16521	0	3.37	1	0.84771	.	.	1
16522	0	3.37	1	0.84771	.	.	1
16523	0	3.37	1	0.84771	.	.	1
16524	0	3.37	1	0.84771	.	.	1
16525	0	3.37	1	0.84771	.	.	1
16526	0	3.37	1	0.84771	.	.	1
16527	0	3.37	1	0.84771	.	.	1
16528	0	3.37	1	0.84771	.	.	1
16529	0	3.37	1	0.84771	.	.	1
16530	0	3.37	1	0.84771	.	.	1
16531	0	3.37	1	0.84771	.	.	1
16532	0	3.37	1	0.84771	.	.	1
16533	0	3.37	1	0.84771	.	.	1
16534	0	3.37	1	0.84771	.	.	1
16535	0	3.37	1	0.84771	.	.	1
16536	0	3.37	1	0.84771	.	.	1
16537	0	3.37	1	0.84771	.	.	1
16538	0	3.37	1	0.84771	.	.	1
16539	0	3.37	1	0.84771	.	.	1
16540	0	3.37	1	0.84771	.	.	1
16541	0	3.37	1	0.84771	.	.	1
16542	0	3.37	1	0.84771	.	.	1
16543	0	3.37	1	0.84771	.	.	1
16544	0	3.37	1	0.84771	.	.	1
16545	0	3.37	1	0.84771	.	.	1
16546	0	3.37	1	0.84771	.	.	1
16547	0	3.37	1	0.84771	.	.	1
16548	0	3.37	1	0.84771	.	.	1
16549	0	3.38	0	0.84754	0.84395	0.85106	1
16550	0	3.38	1	0.84754	.	.	1
16551	0	3.38	1	0.84754	.	.	1
16552	0	3.38	1	0.84754	.	.	1
16553	0	3.38	1	0.84754	.	.	1
16554	0	3.38	1	0.84754	.	.	1
16555	0	3.38	1	0.84754	.	.	1

Obs	Sub_group	fuptimey	_CENSOR_	SURVIVAL	SDF_LCL	SDF_UCL	STRATUM
16556	0	3.38	1	0.84754	.	.	1
16557	0	3.38	1	0.84754	.	.	1
16558	0	3.38	1	0.84754	.	.	1
16559	0	3.38	1	0.84754	.	.	1
16560	0	3.38	1	0.84754	.	.	1
16561	0	3.38	1	0.84754	.	.	1
16562	0	3.38	1	0.84754	.	.	1
16563	0	3.38	1	0.84754	.	.	1
16564	0	3.38	1	0.84754	.	.	1
16565	0	3.38	1	0.84754	.	.	1
16566	0	3.38	1	0.84754	.	.	1
16567	0	3.38	1	0.84754	.	.	1
16568	0	3.38	1	0.84754	.	.	1
16569	0	3.38	1	0.84754	.	.	1
16570	0	3.38	1	0.84754	.	.	1
16571	0	3.38	1	0.84754	.	.	1
16572	0	3.38	1	0.84754	.	.	1
16573	0	3.38	1	0.84754	.	.	1
16574	0	3.38	1	0.84754	.	.	1
16575	0	3.38	1	0.84754	.	.	1
16576	0	3.38	1	0.84754	.	.	1
16577	0	3.38	1	0.84754	.	.	1
16578	0	3.38	1	0.84754	.	.	1
16579	0	3.38	1	0.84754	.	.	1
16580	0	3.38	1	0.84754	.	.	1
16581	0	3.38	1	0.84754	.	.	1
16582	0	3.38	1	0.84754	.	.	1
16583	0	3.38	1	0.84754	.	.	1
16584	0	3.38	1	0.84754	.	.	1
16585	0	3.38	1	0.84754	.	.	1
16586	0	3.38	1	0.84754	.	.	1
16587	0	3.38	1	0.84754	.	.	1
16588	0	3.38	1	0.84754	.	.	1
16589	0	3.38	1	0.84754	.	.	1
16590	0	3.39	0	0.84715	0.84356	0.85068	1

Obs	Sub_group	fuptimey	_CENSOR_	SURVIVAL	SDF_LCL	SDF_UCL	STRATUM
16591	0	3.39	1	0.84715	.	.	1
16592	0	3.39	1	0.84715	.	.	1
16593	0	3.39	1	0.84715	.	.	1
16594	0	3.39	1	0.84715	.	.	1
16595	0	3.39	1	0.84715	.	.	1
16596	0	3.39	1	0.84715	.	.	1
16597	0	3.39	1	0.84715	.	.	1
16598	0	3.39	1	0.84715	.	.	1
16599	0	3.39	1	0.84715	.	.	1
16600	0	3.39	1	0.84715	.	.	1
16601	0	3.39	1	0.84715	.	.	1
16602	0	3.39	1	0.84715	.	.	1
16603	0	3.39	1	0.84715	.	.	1
16604	0	3.39	1	0.84715	.	.	1
16605	0	3.39	1	0.84715	.	.	1
16606	0	3.39	1	0.84715	.	.	1
16607	0	3.39	1	0.84715	.	.	1
16608	0	3.39	1	0.84715	.	.	1
16609	0	3.39	1	0.84715	.	.	1
16610	0	3.39	1	0.84715	.	.	1
16611	0	3.39	1	0.84715	.	.	1
16612	0	3.39	1	0.84715	.	.	1
16613	0	3.39	1	0.84715	.	.	1
16614	0	3.39	1	0.84715	.	.	1
16615	0	3.39	1	0.84715	.	.	1
16616	0	3.39	1	0.84715	.	.	1
16617	0	3.39	1	0.84715	.	.	1
16618	0	3.39	1	0.84715	.	.	1
16619	0	3.39	1	0.84715	.	.	1
16620	0	3.39	1	0.84715	.	.	1
16621	0	3.39	1	0.84715	.	.	1
16622	0	3.39	1	0.84715	.	.	1
16623	0	3.39	1	0.84715	.	.	1
16624	0	3.39	1	0.84715	.	.	1
16625	0	3.39	1	0.84715	.	.	1

Obs	Sub_group	fuptimey	_CENSOR_	SURVIVAL	SDF_LCL	SDF_UCL	STRATUM
16626	0	3.39	1	0.84715	.	.	1
16627	0	3.39	1	0.84715	.	.	1
16628	0	3.39	1	0.84715	.	.	1
16629	0	3.39	1	0.84715	.	.	1
16630	0	3.40	0	0.84701	0.84342	0.85054	1
16631	0	3.40	1	0.84701	.	.	1
16632	0	3.40	1	0.84701	.	.	1
16633	0	3.40	1	0.84701	.	.	1
16634	0	3.40	1	0.84701	.	.	1
16635	0	3.40	1	0.84701	.	.	1
16636	0	3.40	1	0.84701	.	.	1
16637	0	3.40	1	0.84701	.	.	1
16638	0	3.40	1	0.84701	.	.	1
16639	0	3.40	1	0.84701	.	.	1
16640	0	3.40	1	0.84701	.	.	1
16641	0	3.40	1	0.84701	.	.	1
16642	0	3.40	1	0.84701	.	.	1
16643	0	3.40	1	0.84701	.	.	1
16644	0	3.40	1	0.84701	.	.	1
16645	0	3.40	1	0.84701	.	.	1
16646	0	3.40	1	0.84701	.	.	1
16647	0	3.40	1	0.84701	.	.	1
16648	0	3.40	1	0.84701	.	.	1
16649	0	3.40	1	0.84701	.	.	1
16650	0	3.40	1	0.84701	.	.	1
16651	0	3.40	1	0.84701	.	.	1
16652	0	3.40	1	0.84701	.	.	1
16653	0	3.40	1	0.84701	.	.	1
16654	0	3.40	1	0.84701	.	.	1
16655	0	3.40	1	0.84701	.	.	1
16656	0	3.40	1	0.84701	.	.	1
16657	0	3.40	1	0.84701	.	.	1
16658	0	3.40	1	0.84701	.	.	1
16659	0	3.40	1	0.84701	.	.	1
16660	0	3.40	1	0.84701	.	.	1

Obs	Sub_group	fuptimey	_CENSOR_	SURVIVAL	SDF_LCL	SDF_UCL	STRATUM
16661	0	3.40	1	0.84701	.	.	1
16662	0	3.40	1	0.84701	.	.	1
16663	0	3.40	1	0.84701	.	.	1
16664	0	3.40	1	0.84701	.	.	1
16665	0	3.40	1	0.84701	.	.	1
16666	0	3.40	1	0.84701	.	.	1
16667	0	3.40	1	0.84701	.	.	1
16668	0	3.40	1	0.84701	.	.	1
16669	0	3.41	0	0.84666	0.84306	0.85019	1
16670	0	3.41	1	0.84666	.	.	1
16671	0	3.41	1	0.84666	.	.	1
16672	0	3.41	1	0.84666	.	.	1
16673	0	3.41	1	0.84666	.	.	1
16674	0	3.41	1	0.84666	.	.	1
16675	0	3.41	1	0.84666	.	.	1
16676	0	3.41	1	0.84666	.	.	1
16677	0	3.41	1	0.84666	.	.	1
16678	0	3.41	1	0.84666	.	.	1
16679	0	3.41	1	0.84666	.	.	1
16680	0	3.41	1	0.84666	.	.	1
16681	0	3.41	1	0.84666	.	.	1
16682	0	3.41	1	0.84666	.	.	1
16683	0	3.41	1	0.84666	.	.	1
16684	0	3.41	1	0.84666	.	.	1
16685	0	3.41	1	0.84666	.	.	1
16686	0	3.41	1	0.84666	.	.	1
16687	0	3.41	1	0.84666	.	.	1
16688	0	3.41	1	0.84666	.	.	1
16689	0	3.41	1	0.84666	.	.	1
16690	0	3.41	1	0.84666	.	.	1
16691	0	3.41	1	0.84666	.	.	1
16692	0	3.41	1	0.84666	.	.	1
16693	0	3.41	1	0.84666	.	.	1
16694	0	3.41	1	0.84666	.	.	1
16695	0	3.41	1	0.84666	.	.	1

Obs	Sub_group	fuptimey	_CENSOR_	SURVIVAL	SDF_LCL	SDF_UCL	STRATUM
16696	0	3.41	1	0.84666	.	.	1
16697	0	3.41	1	0.84666	.	.	1
16698	0	3.41	1	0.84666	.	.	1
16699	0	3.41	1	0.84666	.	.	1
16700	0	3.41	1	0.84666	.	.	1
16701	0	3.41	1	0.84666	.	.	1
16702	0	3.41	1	0.84666	.	.	1
16703	0	3.41	1	0.84666	.	.	1
16704	0	3.41	1	0.84666	.	.	1
16705	0	3.41	1	0.84666	.	.	1
16706	0	3.41	1	0.84666	.	.	1
16707	0	3.42	0	0.84659	0.84299	0.85012	1
16708	0	3.42	1	0.84659	.	.	1
16709	0	3.42	1	0.84659	.	.	1
16710	0	3.42	1	0.84659	.	.	1
16711	0	3.42	1	0.84659	.	.	1
16712	0	3.42	1	0.84659	.	.	1
16713	0	3.42	1	0.84659	.	.	1
16714	0	3.42	1	0.84659	.	.	1
16715	0	3.42	1	0.84659	.	.	1
16716	0	3.42	1	0.84659	.	.	1
16717	0	3.42	1	0.84659	.	.	1
16718	0	3.42	1	0.84659	.	.	1
16719	0	3.42	1	0.84659	.	.	1
16720	0	3.42	1	0.84659	.	.	1
16721	0	3.42	1	0.84659	.	.	1
16722	0	3.42	1	0.84659	.	.	1
16723	0	3.42	1	0.84659	.	.	1
16724	0	3.42	1	0.84659	.	.	1
16725	0	3.42	1	0.84659	.	.	1
16726	0	3.42	1	0.84659	.	.	1
16727	0	3.42	1	0.84659	.	.	1
16728	0	3.42	1	0.84659	.	.	1
16729	0	3.42	1	0.84659	.	.	1
16730	0	3.42	1	0.84659	.	.	1

Obs	Sub_group	fuptimey	_CENSOR_	SURVIVAL	SDF_LCL	SDF_UCL	STRATUM
**16731**	0	3.42	1	0.84659	.	.	1
**16732**	0	3.42	1	0.84659	.	.	1
**16733**	0	3.42	1	0.84659	.	.	1
**16734**	0	3.42	1	0.84659	.	.	1
**16735**	0	3.42	1	0.84659	.	.	1
**16736**	0	3.42	1	0.84659	.	.	1
**16737**	0	3.42	1	0.84659	.	.	1
**16738**	0	3.42	1	0.84659	.	.	1
**16739**	0	3.42	1	0.84659	.	.	1
**16740**	0	3.42	1	0.84659	.	.	1
**16741**	0	3.42	1	0.84659	.	.	1
**16742**	0	3.42	1	0.84659	.	.	1
**16743**	0	3.42	1	0.84659	.	.	1
**16744**	0	3.43	0	0.84642	0.84281	0.84995	1
**16745**	0	3.43	1	0.84642	.	.	1
**16746**	0	3.43	1	0.84642	.	.	1
**16747**	0	3.43	1	0.84642	.	.	1
**16748**	0	3.43	1	0.84642	.	.	1
**16749**	0	3.43	1	0.84642	.	.	1
**16750**	0	3.43	1	0.84642	.	.	1
**16751**	0	3.43	1	0.84642	.	.	1
**16752**	0	3.43	1	0.84642	.	.	1
**16753**	0	3.43	1	0.84642	.	.	1
**16754**	0	3.43	1	0.84642	.	.	1
**16755**	0	3.43	1	0.84642	.	.	1
**16756**	0	3.43	1	0.84642	.	.	1
**16757**	0	3.43	1	0.84642	.	.	1
**16758**	0	3.43	1	0.84642	.	.	1
**16759**	0	3.43	1	0.84642	.	.	1
**16760**	0	3.43	1	0.84642	.	.	1
**16761**	0	3.43	1	0.84642	.	.	1
**16762**	0	3.43	1	0.84642	.	.	1
**16763**	0	3.43	1	0.84642	.	.	1
**16764**	0	3.43	1	0.84642	.	.	1
**16765**	0	3.43	1	0.84642	.	.	1

Obs	Sub_group	fuptimey	_CENSOR_	SURVIVAL	SDF_LCL	SDF_UCL	STRATUM
16766	0	3.43	1	0.84642	.	.	1
16767	0	3.43	1	0.84642	.	.	1
16768	0	3.43	1	0.84642	.	.	1
16769	0	3.43	1	0.84642	.	.	1
16770	0	3.43	1	0.84642	.	.	1
16771	0	3.43	1	0.84642	.	.	1
16772	0	3.43	1	0.84642	.	.	1
16773	0	3.43	1	0.84642	.	.	1
16774	0	3.43	1	0.84642	.	.	1
16775	0	3.43	1	0.84642	.	.	1
16776	0	3.43	1	0.84642	.	.	1
16777	0	3.43	1	0.84642	.	.	1
16778	0	3.43	1	0.84642	.	.	1
16779	0	3.43	1	0.84642	.	.	1
16780	0	3.43	1	0.84642	.	.	1
16781	0	3.43	1	0.84642	.	.	1
16782	0	3.43	1	0.84642	.	.	1
16783	0	3.43	1	0.84642	.	.	1
16784	0	3.44	0	0.84613	0.84252	0.84967	1
16785	0	3.44	1	0.84613	.	.	1
16786	0	3.44	1	0.84613	.	.	1
16787	0	3.44	1	0.84613	.	.	1
16788	0	3.44	1	0.84613	.	.	1
16789	0	3.44	1	0.84613	.	.	1
16790	0	3.44	1	0.84613	.	.	1
16791	0	3.44	1	0.84613	.	.	1
16792	0	3.44	1	0.84613	.	.	1
16793	0	3.44	1	0.84613	.	.	1
16794	0	3.44	1	0.84613	.	.	1
16795	0	3.44	1	0.84613	.	.	1
16796	0	3.44	1	0.84613	.	.	1
16797	0	3.44	1	0.84613	.	.	1
16798	0	3.44	1	0.84613	.	.	1
16799	0	3.44	1	0.84613	.	.	1
16800	0	3.44	1	0.84613	.	.	1

Obs	Sub_group	fuptimey	_CENSOR_	SURVIVAL	SDF_LCL	SDF_UCL	STRATUM
16801	0	3.44	1	0.84613	.	.	1
16802	0	3.44	1	0.84613	.	.	1
16803	0	3.44	1	0.84613	.	.	1
16804	0	3.44	1	0.84613	.	.	1
16805	0	3.44	1	0.84613	.	.	1
16806	0	3.44	1	0.84613	.	.	1
16807	0	3.44	1	0.84613	.	.	1
16808	0	3.44	1	0.84613	.	.	1
16809	0	3.44	1	0.84613	.	.	1
16810	0	3.44	1	0.84613	.	.	1
16811	0	3.44	1	0.84613	.	.	1
16812	0	3.44	1	0.84613	.	.	1
16813	0	3.44	1	0.84613	.	.	1
16814	0	3.44	1	0.84613	.	.	1
16815	0	3.44	1	0.84613	.	.	1
16816	0	3.44	1	0.84613	.	.	1
16817	0	3.44	1	0.84613	.	.	1
16818	0	3.44	1	0.84613	.	.	1
16819	0	3.44	1	0.84613	.	.	1
16820	0	3.44	1	0.84613	.	.	1
16821	0	3.44	1	0.84613	.	.	1
16822	0	3.44	1	0.84613	.	.	1
16823	0	3.44	1	0.84613	.	.	1
16824	0	3.44	1	0.84613	.	.	1
16825	0	3.44	1	0.84613	.	.	1
16826	0	3.44	1	0.84613	.	.	1
16827	0	3.44	1	0.84613	.	.	1
16828	0	3.44	1	0.84613	.	.	1
16829	0	3.44	1	0.84613	.	.	1
16830	0	3.44	1	0.84613	.	.	1
16831	0	3.44	1	0.84613	.	.	1
16832	0	3.44	1	0.84613	.	.	1
16833	0	3.44	1	0.84613	.	.	1
16834	0	3.44	1	0.84613	.	.	1
16835	0	3.44	1	0.84613	.	.	1

Obs	Sub_group	fuptimey	_CENSOR_	SURVIVAL	SDF_LCL	SDF_UCL	STRATUM
16836	0	3.44	1	0.84613	.	.	1
16837	0	3.44	1	0.84613	.	.	1
16838	0	3.44	1	0.84613	.	.	1
16839	0	3.44	1	0.84613	.	.	1
16840	0	3.44	1	0.84613	.	.	1
16841	0	3.44	1	0.84613	.	.	1
16842	0	3.44	1	0.84613	.	.	1
16843	0	3.44	1	0.84613	.	.	1
16844	0	3.44	1	0.84613	.	.	1
16845	0	3.44	1	0.84613	.	.	1
16846	0	3.44	1	0.84613	.	.	1
16847	0	3.45	0	0.84606	0.84245	0.84960	1
16848	0	3.45	1	0.84606	.	.	1
16849	0	3.45	1	0.84606	.	.	1
16850	0	3.45	1	0.84606	.	.	1
16851	0	3.45	1	0.84606	.	.	1
16852	0	3.45	1	0.84606	.	.	1
16853	0	3.45	1	0.84606	.	.	1
16854	0	3.45	1	0.84606	.	.	1
16855	0	3.45	1	0.84606	.	.	1
16856	0	3.45	1	0.84606	.	.	1
16857	0	3.45	1	0.84606	.	.	1
16858	0	3.45	1	0.84606	.	.	1
16859	0	3.45	1	0.84606	.	.	1
16860	0	3.45	1	0.84606	.	.	1
16861	0	3.45	1	0.84606	.	.	1
16862	0	3.45	1	0.84606	.	.	1
16863	0	3.45	1	0.84606	.	.	1
16864	0	3.45	1	0.84606	.	.	1
16865	0	3.45	1	0.84606	.	.	1
16866	0	3.45	1	0.84606	.	.	1
16867	0	3.45	1	0.84606	.	.	1
16868	0	3.45	1	0.84606	.	.	1
16869	0	3.45	1	0.84606	.	.	1
16870	0	3.45	1	0.84606	.	.	1

Obs	Sub_group	fuptimey	_CENSOR_	SURVIVAL	SDF_LCL	SDF_UCL	STRATUM
16871	0	3.45	1	0.84606	.	.	1
16872	0	3.45	1	0.84606	.	.	1
16873	0	3.45	1	0.84606	.	.	1
16874	0	3.45	1	0.84606	.	.	1
16875	0	3.45	1	0.84606	.	.	1
16876	0	3.45	1	0.84606	.	.	1
16877	0	3.45	1	0.84606	.	.	1
16878	0	3.45	1	0.84606	.	.	1
16879	0	3.45	1	0.84606	.	.	1
16880	0	3.45	1	0.84606	.	.	1
16881	0	3.45	1	0.84606	.	.	1
16882	0	3.45	1	0.84606	.	.	1
16883	0	3.45	1	0.84606	.	.	1
16884	0	3.45	1	0.84606	.	.	1
16885	0	3.45	1	0.84606	.	.	1
16886	0	3.45	1	0.84606	.	.	1
16887	0	3.45	1	0.84606	.	.	1
16888	0	3.45	1	0.84606	.	.	1
16889	0	3.45	1	0.84606	.	.	1
16890	0	3.45	1	0.84606	.	.	1
16891	0	3.45	1	0.84606	.	.	1
16892	0	3.45	1	0.84606	.	.	1
16893	0	3.45	1	0.84606	.	.	1
16894	0	3.45	1	0.84606	.	.	1
16895	0	3.45	1	0.84606	.	.	1
16896	0	3.45	1	0.84606	.	.	1
16897	0	3.45	1	0.84606	.	.	1
16898	0	3.46	0	0.84592	0.84231	0.84946	1
16899	0	3.46	1	0.84592	.	.	1
16900	0	3.46	1	0.84592	.	.	1
16901	0	3.46	1	0.84592	.	.	1
16902	0	3.46	1	0.84592	.	.	1
16903	0	3.46	1	0.84592	.	.	1
16904	0	3.46	1	0.84592	.	.	1
16905	0	3.46	1	0.84592	.	.	1

Obs	Sub_group	fuptimey	_CENSOR_	SURVIVAL	SDF_LCL	SDF_UCL	STRATUM
16906	0	3.46	1	0.84592	.	.	1
16907	0	3.46	1	0.84592	.	.	1
16908	0	3.46	1	0.84592	.	.	1
16909	0	3.46	1	0.84592	.	.	1
16910	0	3.46	1	0.84592	.	.	1
16911	0	3.46	1	0.84592	.	.	1
16912	0	3.46	1	0.84592	.	.	1
16913	0	3.46	1	0.84592	.	.	1
16914	0	3.46	1	0.84592	.	.	1
16915	0	3.46	1	0.84592	.	.	1
16916	0	3.46	1	0.84592	.	.	1
16917	0	3.46	1	0.84592	.	.	1
16918	0	3.46	1	0.84592	.	.	1
16919	0	3.46	1	0.84592	.	.	1
16920	0	3.46	1	0.84592	.	.	1
16921	0	3.46	1	0.84592	.	.	1
16922	0	3.46	1	0.84592	.	.	1
16923	0	3.46	1	0.84592	.	.	1
16924	0	3.46	1	0.84592	.	.	1
16925	0	3.46	1	0.84592	.	.	1
16926	0	3.46	1	0.84592	.	.	1
16927	0	3.46	1	0.84592	.	.	1
16928	0	3.46	1	0.84592	.	.	1
16929	0	3.46	1	0.84592	.	.	1
16930	0	3.46	1	0.84592	.	.	1
16931	0	3.46	1	0.84592	.	.	1
16932	0	3.46	1	0.84592	.	.	1
16933	0	3.46	1	0.84592	.	.	1
16934	0	3.46	1	0.84592	.	.	1
16935	0	3.46	1	0.84592	.	.	1
16936	0	3.46	1	0.84592	.	.	1
16937	0	3.46	1	0.84592	.	.	1
16938	0	3.46	1	0.84592	.	.	1
16939	0	3.46	1	0.84592	.	.	1
16940	0	3.46	1	0.84592	.	.	1

Obs	Sub_group	fuptimey	_CENSOR_	SURVIVAL	SDF_LCL	SDF_UCL	STRATUM
16941	0	3.46	1	0.84592	.	.	1
16942	0	3.46	1	0.84592	.	.	1
16943	0	3.46	1	0.84592	.	.	1
16944	0	3.46	1	0.84592	.	.	1
16945	0	3.46	1	0.84592	.	.	1
16946	0	3.46	1	0.84592	.	.	1
16947	0	3.46	1	0.84592	.	.	1
16948	0	3.46	1	0.84592	.	.	1
16949	0	3.46	1	0.84592	.	.	1
16950	0	3.46	1	0.84592	.	.	1
16951	0	3.46	1	0.84592	.	.	1
16952	0	3.46	1	0.84592	.	.	1
16953	0	3.46	1	0.84592	.	.	1
16954	0	3.46	1	0.84592	.	.	1
16955	0	3.46	1	0.84592	.	.	1
16956	0	3.46	1	0.84592	.	.	1
16957	0	3.47	0	0.84557	0.84195	0.84911	1
16958	0	3.47	1	0.84557	.	.	1
16959	0	3.47	1	0.84557	.	.	1
16960	0	3.47	1	0.84557	.	.	1
16961	0	3.47	1	0.84557	.	.	1
16962	0	3.47	1	0.84557	.	.	1
16963	0	3.47	1	0.84557	.	.	1
16964	0	3.47	1	0.84557	.	.	1
16965	0	3.47	1	0.84557	.	.	1
16966	0	3.47	1	0.84557	.	.	1
16967	0	3.47	1	0.84557	.	.	1
16968	0	3.47	1	0.84557	.	.	1
16969	0	3.47	1	0.84557	.	.	1
16970	0	3.47	1	0.84557	.	.	1
16971	0	3.47	1	0.84557	.	.	1
16972	0	3.47	1	0.84557	.	.	1
16973	0	3.47	1	0.84557	.	.	1
16974	0	3.47	1	0.84557	.	.	1
16975	0	3.47	1	0.84557	.	.	1

Obs	Sub_group	fuptimey	_CENSOR_	SURVIVAL	SDF_LCL	SDF_UCL	STRATUM
16976	0	3.47	1	0.84557	.	.	1
16977	0	3.47	1	0.84557	.	.	1
16978	0	3.47	1	0.84557	.	.	1
16979	0	3.47	1	0.84557	.	.	1
16980	0	3.47	1	0.84557	.	.	1
16981	0	3.47	1	0.84557	.	.	1
16982	0	3.47	1	0.84557	.	.	1
16983	0	3.47	1	0.84557	.	.	1
16984	0	3.47	1	0.84557	.	.	1
16985	0	3.47	1	0.84557	.	.	1
16986	0	3.47	1	0.84557	.	.	1
16987	0	3.47	1	0.84557	.	.	1
16988	0	3.47	1	0.84557	.	.	1
16989	0	3.47	1	0.84557	.	.	1
16990	0	3.47	1	0.84557	.	.	1
16991	0	3.47	1	0.84557	.	.	1
16992	0	3.48	0	0.84542	0.84180	0.84897	1
16993	0	3.48	1	0.84542	.	.	1
16994	0	3.48	1	0.84542	.	.	1
16995	0	3.48	1	0.84542	.	.	1
16996	0	3.48	1	0.84542	.	.	1
16997	0	3.48	1	0.84542	.	.	1
16998	0	3.48	1	0.84542	.	.	1
16999	0	3.48	1	0.84542	.	.	1
17000	0	3.48	1	0.84542	.	.	1
17001	0	3.48	1	0.84542	.	.	1
17002	0	3.48	1	0.84542	.	.	1
17003	0	3.48	1	0.84542	.	.	1
17004	0	3.48	1	0.84542	.	.	1
17005	0	3.48	1	0.84542	.	.	1
17006	0	3.48	1	0.84542	.	.	1
17007	0	3.48	1	0.84542	.	.	1
17008	0	3.48	1	0.84542	.	.	1
17009	0	3.48	1	0.84542	.	.	1
17010	0	3.48	1	0.84542	.	.	1

Obs	Sub_group	fuptimey	_CENSOR_	SURVIVAL	SDF_LCL	SDF_UCL	STRATUM
**17011**	0	3.48	1	0.84542	.	.	1
**17012**	0	3.48	1	0.84542	.	.	1
**17013**	0	3.48	1	0.84542	.	.	1
**17014**	0	3.48	1	0.84542	.	.	1
**17015**	0	3.48	1	0.84542	.	.	1
**17016**	0	3.48	1	0.84542	.	.	1
**17017**	0	3.48	1	0.84542	.	.	1
**17018**	0	3.48	1	0.84542	.	.	1
**17019**	0	3.48	1	0.84542	.	.	1
**17020**	0	3.48	1	0.84542	.	.	1
**17021**	0	3.48	1	0.84542	.	.	1
**17022**	0	3.48	1	0.84542	.	.	1
**17023**	0	3.48	1	0.84542	.	.	1
**17024**	0	3.48	1	0.84542	.	.	1
**17025**	0	3.48	1	0.84542	.	.	1
**17026**	0	3.48	1	0.84542	.	.	1
**17027**	0	3.48	1	0.84542	.	.	1
**17028**	0	3.48	1	0.84542	.	.	1
**17029**	0	3.48	1	0.84542	.	.	1
**17030**	0	3.48	1	0.84542	.	.	1
**17031**	0	3.48	1	0.84542	.	.	1
**17032**	0	3.48	1	0.84542	.	.	1
**17033**	0	3.48	1	0.84542	.	.	1
**17034**	0	3.48	1	0.84542	.	.	1
**17035**	0	3.49	0	0.84517	0.84155	0.84872	1
**17036**	0	3.49	1	0.84517	.	.	1
**17037**	0	3.49	1	0.84517	.	.	1
**17038**	0	3.49	1	0.84517	.	.	1
**17039**	0	3.49	1	0.84517	.	.	1
**17040**	0	3.49	1	0.84517	.	.	1
**17041**	0	3.49	1	0.84517	.	.	1
**17042**	0	3.49	1	0.84517	.	.	1
**17043**	0	3.49	1	0.84517	.	.	1
**17044**	0	3.49	1	0.84517	.	.	1
**17045**	0	3.49	1	0.84517	.	.	1

Obs	Sub_group	fuptimey	_CENSOR_	SURVIVAL	SDF_LCL	SDF_UCL	STRATUM
17046	0	3.49	1	0.84517	.	.	1
17047	0	3.49	1	0.84517	.	.	1
17048	0	3.49	1	0.84517	.	.	1
17049	0	3.49	1	0.84517	.	.	1
17050	0	3.49	1	0.84517	.	.	1
17051	0	3.49	1	0.84517	.	.	1
17052	0	3.49	1	0.84517	.	.	1
17053	0	3.49	1	0.84517	.	.	1
17054	0	3.49	1	0.84517	.	.	1
17055	0	3.49	1	0.84517	.	.	1
17056	0	3.49	1	0.84517	.	.	1
17057	0	3.49	1	0.84517	.	.	1
17058	0	3.49	1	0.84517	.	.	1
17059	0	3.49	1	0.84517	.	.	1
17060	0	3.49	1	0.84517	.	.	1
17061	0	3.49	1	0.84517	.	.	1
17062	0	3.49	1	0.84517	.	.	1
17063	0	3.49	1	0.84517	.	.	1
17064	0	3.49	1	0.84517	.	.	1
17065	0	3.49	1	0.84517	.	.	1
17066	0	3.49	1	0.84517	.	.	1
17067	0	3.49	1	0.84517	.	.	1
17068	0	3.50	0	0.84507	0.84144	0.84862	1
17069	0	3.50	1	0.84507	.	.	1
17070	0	3.50	1	0.84507	.	.	1
17071	0	3.50	1	0.84507	.	.	1
17072	0	3.50	1	0.84507	.	.	1
17073	0	3.50	1	0.84507	.	.	1
17074	0	3.50	1	0.84507	.	.	1
17075	0	3.50	1	0.84507	.	.	1
17076	0	3.50	1	0.84507	.	.	1
17077	0	3.50	1	0.84507	.	.	1
17078	0	3.50	1	0.84507	.	.	1
17079	0	3.50	1	0.84507	.	.	1
17080	0	3.50	1	0.84507	.	.	1

Obs	Sub_group	fuptimey	_CENSOR_	SURVIVAL	SDF_LCL	SDF_UCL	STRATUM
**17081**	0	3.50	1	0.84507	.	.	1
**17082**	0	3.50	1	0.84507	.	.	1
**17083**	0	3.50	1	0.84507	.	.	1
**17084**	0	3.50	1	0.84507	.	.	1
**17085**	0	3.50	1	0.84507	.	.	1
**17086**	0	3.50	1	0.84507	.	.	1
**17087**	0	3.50	1	0.84507	.	.	1
**17088**	0	3.50	1	0.84507	.	.	1
**17089**	0	3.50	1	0.84507	.	.	1
**17090**	0	3.50	1	0.84507	.	.	1
**17091**	0	3.50	1	0.84507	.	.	1
**17092**	0	3.50	1	0.84507	.	.	1
**17093**	0	3.50	1	0.84507	.	.	1
**17094**	0	3.50	1	0.84507	.	.	1
**17095**	0	3.50	1	0.84507	.	.	1
**17096**	0	3.50	1	0.84507	.	.	1
**17097**	0	3.50	1	0.84507	.	.	1
**17098**	0	3.51	0	0.84496	0.84133	0.84851	1
**17099**	0	3.51	1	0.84496	.	.	1
**17100**	0	3.51	1	0.84496	.	.	1
**17101**	0	3.51	1	0.84496	.	.	1
**17102**	0	3.51	1	0.84496	.	.	1
**17103**	0	3.51	1	0.84496	.	.	1
**17104**	0	3.51	1	0.84496	.	.	1
**17105**	0	3.51	1	0.84496	.	.	1
**17106**	0	3.51	1	0.84496	.	.	1
**17107**	0	3.51	1	0.84496	.	.	1
**17108**	0	3.51	1	0.84496	.	.	1
**17109**	0	3.51	1	0.84496	.	.	1
**17110**	0	3.51	1	0.84496	.	.	1
**17111**	0	3.51	1	0.84496	.	.	1
**17112**	0	3.51	1	0.84496	.	.	1
**17113**	0	3.51	1	0.84496	.	.	1
**17114**	0	3.51	1	0.84496	.	.	1
**17115**	0	3.51	1	0.84496	.	.	1

Obs	Sub_group	fuptimey	_CENSOR_	SURVIVAL	SDF_LCL	SDF_UCL	STRATUM
**17116**	0	3.51	1	0.84496	.	.	1
**17117**	0	3.51	1	0.84496	.	.	1
**17118**	0	3.51	1	0.84496	.	.	1
**17119**	0	3.51	1	0.84496	.	.	1
**17120**	0	3.51	1	0.84496	.	.	1
**17121**	0	3.51	1	0.84496	.	.	1
**17122**	0	3.51	1	0.84496	.	.	1
**17123**	0	3.51	1	0.84496	.	.	1
**17124**	0	3.51	1	0.84496	.	.	1
**17125**	0	3.51	1	0.84496	.	.	1
**17126**	0	3.51	1	0.84496	.	.	1
**17127**	0	3.51	1	0.84496	.	.	1
**17128**	0	3.51	1	0.84496	.	.	1
**17129**	0	3.51	1	0.84496	.	.	1
**17130**	0	3.51	1	0.84496	.	.	1
**17131**	0	3.51	1	0.84496	.	.	1
**17132**	0	3.51	1	0.84496	.	.	1
**17133**	0	3.51	1	0.84496	.	.	1
**17134**	0	3.51	1	0.84496	.	.	1
**17135**	0	3.51	1	0.84496	.	.	1
**17136**	0	3.51	1	0.84496	.	.	1
**17137**	0	3.51	1	0.84496	.	.	1
**17138**	0	3.51	1	0.84496	.	.	1
**17139**	0	3.51	1	0.84496	.	.	1
**17140**	0	3.51	1	0.84496	.	.	1
**17141**	0	3.51	1	0.84496	.	.	1
**17142**	0	3.51	1	0.84496	.	.	1
**17143**	0	3.51	1	0.84496	.	.	1
**17144**	0	3.51	1	0.84496	.	.	1
**17145**	0	3.51	1	0.84496	.	.	1
**17146**	0	3.51	1	0.84496	.	.	1
**17147**	0	3.52	0	0.84478	0.84115	0.84834	1
**17148**	0	3.52	1	0.84478	.	.	1
**17149**	0	3.52	1	0.84478	.	.	1
**17150**	0	3.52	1	0.84478	.	.	1

Obs	Sub_group	fuptimey	_CENSOR_	SURVIVAL	SDF_LCL	SDF_UCL	STRATUM
**17151**	0	3.52	1	0.84478	.	.	1
**17152**	0	3.52	1	0.84478	.	.	1
**17153**	0	3.52	1	0.84478	.	.	1
**17154**	0	3.52	1	0.84478	.	.	1
**17155**	0	3.52	1	0.84478	.	.	1
**17156**	0	3.52	1	0.84478	.	.	1
**17157**	0	3.52	1	0.84478	.	.	1
**17158**	0	3.52	1	0.84478	.	.	1
**17159**	0	3.52	1	0.84478	.	.	1
**17160**	0	3.52	1	0.84478	.	.	1
**17161**	0	3.52	1	0.84478	.	.	1
**17162**	0	3.52	1	0.84478	.	.	1
**17163**	0	3.52	1	0.84478	.	.	1
**17164**	0	3.52	1	0.84478	.	.	1
**17165**	0	3.52	1	0.84478	.	.	1
**17166**	0	3.52	1	0.84478	.	.	1
**17167**	0	3.52	1	0.84478	.	.	1
**17168**	0	3.52	1	0.84478	.	.	1
**17169**	0	3.52	1	0.84478	.	.	1
**17170**	0	3.52	1	0.84478	.	.	1
**17171**	0	3.52	1	0.84478	.	.	1
**17172**	0	3.52	1	0.84478	.	.	1
**17173**	0	3.52	1	0.84478	.	.	1
**17174**	0	3.52	1	0.84478	.	.	1
**17175**	0	3.52	1	0.84478	.	.	1
**17176**	0	3.52	1	0.84478	.	.	1
**17177**	0	3.52	1	0.84478	.	.	1
**17178**	0	3.52	1	0.84478	.	.	1
**17179**	0	3.52	1	0.84478	.	.	1
**17180**	0	3.52	1	0.84478	.	.	1
**17181**	0	3.52	1	0.84478	.	.	1
**17182**	0	3.52	1	0.84478	.	.	1
**17183**	0	3.52	1	0.84478	.	.	1
**17184**	0	3.52	1	0.84478	.	.	1
**17185**	0	3.52	1	0.84478	.	.	1

Obs	Sub_group	fuptimey	_CENSOR_	SURVIVAL	SDF_LCL	SDF_UCL	STRATUM
17186	0	3.52	1	0.84478	.	.	1
17187	0	3.52	1	0.84478	.	.	1
17188	0	3.52	1	0.84478	.	.	1
17189	0	3.52	1	0.84478	.	.	1
17190	0	3.52	1	0.84478	.	.	1
17191	0	3.52	1	0.84478	.	.	1
17192	0	3.53	0	0.84464	0.84100	0.84819	1
17193	0	3.53	1	0.84464	.	.	1
17194	0	3.53	1	0.84464	.	.	1
17195	0	3.53	1	0.84464	.	.	1
17196	0	3.53	1	0.84464	.	.	1
17197	0	3.53	1	0.84464	.	.	1
17198	0	3.53	1	0.84464	.	.	1
17199	0	3.53	1	0.84464	.	.	1
17200	0	3.53	1	0.84464	.	.	1
17201	0	3.53	1	0.84464	.	.	1
17202	0	3.53	1	0.84464	.	.	1
17203	0	3.53	1	0.84464	.	.	1
17204	0	3.53	1	0.84464	.	.	1
17205	0	3.53	1	0.84464	.	.	1
17206	0	3.53	1	0.84464	.	.	1
17207	0	3.53	1	0.84464	.	.	1
17208	0	3.53	1	0.84464	.	.	1
17209	0	3.53	1	0.84464	.	.	1
17210	0	3.53	1	0.84464	.	.	1
17211	0	3.53	1	0.84464	.	.	1
17212	0	3.53	1	0.84464	.	.	1
17213	0	3.53	1	0.84464	.	.	1
17214	0	3.53	1	0.84464	.	.	1
17215	0	3.53	1	0.84464	.	.	1
17216	0	3.53	1	0.84464	.	.	1
17217	0	3.53	1	0.84464	.	.	1
17218	0	3.53	1	0.84464	.	.	1
17219	0	3.53	1	0.84464	.	.	1
17220	0	3.53	1	0.84464	.	.	1

Obs	Sub_group	fuptimey	_CENSOR_	SURVIVAL	SDF_LCL	SDF_UCL	STRATUM
**17221**	0	3.53	1	0.84464	.	.	1
**17222**	0	3.53	1	0.84464	.	.	1
**17223**	0	3.53	1	0.84464	.	.	1
**17224**	0	3.53	1	0.84464	.	.	1
**17225**	0	3.53	1	0.84464	.	.	1
**17226**	0	3.53	1	0.84464	.	.	1
**17227**	0	3.53	1	0.84464	.	.	1
**17228**	0	3.53	1	0.84464	.	.	1
**17229**	0	3.53	1	0.84464	.	.	1
**17230**	0	3.53	1	0.84464	.	.	1
**17231**	0	3.53	1	0.84464	.	.	1
**17232**	0	3.53	1	0.84464	.	.	1
**17233**	0	3.53	1	0.84464	.	.	1
**17234**	0	3.53	1	0.84464	.	.	1
**17235**	0	3.53	1	0.84464	.	.	1
**17236**	0	3.53	1	0.84464	.	.	1
**17237**	0	3.53	1	0.84464	.	.	1
**17238**	0	3.53	1	0.84464	.	.	1
**17239**	0	3.53	1	0.84464	.	.	1
**17240**	0	3.54	0	0.84442	0.84078	0.84798	1
**17241**	0	3.54	1	0.84442	.	.	1
**17242**	0	3.54	1	0.84442	.	.	1
**17243**	0	3.54	1	0.84442	.	.	1
**17244**	0	3.54	1	0.84442	.	.	1
**17245**	0	3.54	1	0.84442	.	.	1
**17246**	0	3.54	1	0.84442	.	.	1
**17247**	0	3.54	1	0.84442	.	.	1
**17248**	0	3.54	1	0.84442	.	.	1
**17249**	0	3.54	1	0.84442	.	.	1
**17250**	0	3.54	1	0.84442	.	.	1
**17251**	0	3.54	1	0.84442	.	.	1
**17252**	0	3.54	1	0.84442	.	.	1
**17253**	0	3.54	1	0.84442	.	.	1
**17254**	0	3.54	1	0.84442	.	.	1
**17255**	0	3.54	1	0.84442	.	.	1

Obs	Sub_group	fuptimey	_CENSOR_	SURVIVAL	SDF_LCL	SDF_UCL	STRATUM
17256	0	3.54	1	0.84442	.	.	1
17257	0	3.54	1	0.84442	.	.	1
17258	0	3.54	1	0.84442	.	.	1
17259	0	3.54	1	0.84442	.	.	1
17260	0	3.54	1	0.84442	.	.	1
17261	0	3.54	1	0.84442	.	.	1
17262	0	3.54	1	0.84442	.	.	1
17263	0	3.54	1	0.84442	.	.	1
17264	0	3.54	1	0.84442	.	.	1
17265	0	3.54	1	0.84442	.	.	1
17266	0	3.54	1	0.84442	.	.	1
17267	0	3.54	1	0.84442	.	.	1
17268	0	3.54	1	0.84442	.	.	1
17269	0	3.54	1	0.84442	.	.	1
17270	0	3.54	1	0.84442	.	.	1
17271	0	3.54	1	0.84442	.	.	1
17272	0	3.54	1	0.84442	.	.	1
17273	0	3.54	1	0.84442	.	.	1
17274	0	3.54	1	0.84442	.	.	1
17275	0	3.54	1	0.84442	.	.	1
17276	0	3.54	1	0.84442	.	.	1
17277	0	3.54	1	0.84442	.	.	1
17278	0	3.54	1	0.84442	.	.	1
17279	0	3.54	1	0.84442	.	.	1
17280	0	3.54	1	0.84442	.	.	1
17281	0	3.54	1	0.84442	.	.	1
17282	0	3.54	1	0.84442	.	.	1
17283	0	3.54	1	0.84442	.	.	1
17284	0	3.54	1	0.84442	.	.	1
17285	0	3.54	1	0.84442	.	.	1
17286	0	3.54	1	0.84442	.	.	1
17287	0	3.54	1	0.84442	.	.	1
17288	0	3.54	1	0.84442	.	.	1
17289	0	3.55	0	0.84417	0.84053	0.84773	1
17290	0	3.55	1	0.84417	.	.	1

Obs	Sub_group	fuptimey	_CENSOR_	SURVIVAL	SDF_LCL	SDF_UCL	STRATUM
**17291**	0	3.55	1	0.84417	.	.	1
**17292**	0	3.55	1	0.84417	.	.	1
**17293**	0	3.55	1	0.84417	.	.	1
**17294**	0	3.55	1	0.84417	.	.	1
**17295**	0	3.55	1	0.84417	.	.	1
**17296**	0	3.55	1	0.84417	.	.	1
**17297**	0	3.55	1	0.84417	.	.	1
**17298**	0	3.55	1	0.84417	.	.	1
**17299**	0	3.55	1	0.84417	.	.	1
**17300**	0	3.55	1	0.84417	.	.	1
**17301**	0	3.55	1	0.84417	.	.	1
**17302**	0	3.55	1	0.84417	.	.	1
**17303**	0	3.55	1	0.84417	.	.	1
**17304**	0	3.55	1	0.84417	.	.	1
**17305**	0	3.55	1	0.84417	.	.	1
**17306**	0	3.55	1	0.84417	.	.	1
**17307**	0	3.55	1	0.84417	.	.	1
**17308**	0	3.55	1	0.84417	.	.	1
**17309**	0	3.55	1	0.84417	.	.	1
**17310**	0	3.55	1	0.84417	.	.	1
**17311**	0	3.55	1	0.84417	.	.	1
**17312**	0	3.55	1	0.84417	.	.	1
**17313**	0	3.55	1	0.84417	.	.	1
**17314**	0	3.55	1	0.84417	.	.	1
**17315**	0	3.55	1	0.84417	.	.	1
**17316**	0	3.55	1	0.84417	.	.	1
**17317**	0	3.55	1	0.84417	.	.	1
**17318**	0	3.55	1	0.84417	.	.	1
**17319**	0	3.55	1	0.84417	.	.	1
**17320**	0	3.55	1	0.84417	.	.	1
**17321**	0	3.55	1	0.84417	.	.	1
**17322**	0	3.55	1	0.84417	.	.	1
**17323**	0	3.55	1	0.84417	.	.	1
**17324**	0	3.55	1	0.84417	.	.	1
**17325**	0	3.55	1	0.84417	.	.	1

Obs	Sub_group	fuptimey	_CENSOR_	SURVIVAL	SDF_LCL	SDF_UCL	STRATUM
**17326**	0	3.55	1	0.84417	.	.	1
**17327**	0	3.55	1	0.84417	.	.	1
**17328**	0	3.55	1	0.84417	.	.	1
**17329**	0	3.55	1	0.84417	.	.	1
**17330**	0	3.56	0	0.84399	0.84034	0.84756	1
**17331**	0	3.56	1	0.84399	.	.	1
**17332**	0	3.56	1	0.84399	.	.	1
**17333**	0	3.56	1	0.84399	.	.	1
**17334**	0	3.56	1	0.84399	.	.	1
**17335**	0	3.56	1	0.84399	.	.	1
**17336**	0	3.56	1	0.84399	.	.	1
**17337**	0	3.56	1	0.84399	.	.	1
**17338**	0	3.56	1	0.84399	.	.	1
**17339**	0	3.56	1	0.84399	.	.	1
**17340**	0	3.56	1	0.84399	.	.	1
**17341**	0	3.56	1	0.84399	.	.	1
**17342**	0	3.56	1	0.84399	.	.	1
**17343**	0	3.56	1	0.84399	.	.	1
**17344**	0	3.56	1	0.84399	.	.	1
**17345**	0	3.56	1	0.84399	.	.	1
**17346**	0	3.56	1	0.84399	.	.	1
**17347**	0	3.56	1	0.84399	.	.	1
**17348**	0	3.56	1	0.84399	.	.	1
**17349**	0	3.56	1	0.84399	.	.	1
**17350**	0	3.56	1	0.84399	.	.	1
**17351**	0	3.56	1	0.84399	.	.	1
**17352**	0	3.56	1	0.84399	.	.	1
**17353**	0	3.56	1	0.84399	.	.	1
**17354**	0	3.56	1	0.84399	.	.	1
**17355**	0	3.56	1	0.84399	.	.	1
**17356**	0	3.56	1	0.84399	.	.	1
**17357**	0	3.56	1	0.84399	.	.	1
**17358**	0	3.56	1	0.84399	.	.	1
**17359**	0	3.57	0	0.84373	0.84009	0.84731	1
**17360**	0	3.57	1	0.84373	.	.	1

Obs	Sub_group	fuptimey	_CENSOR_	SURVIVAL	SDF_LCL	SDF_UCL	STRATUM
17361	0	3.57	1	0.84373	.	.	1
17362	0	3.57	1	0.84373	.	.	1
17363	0	3.57	1	0.84373	.	.	1
17364	0	3.57	1	0.84373	.	.	1
17365	0	3.57	1	0.84373	.	.	1
17366	0	3.57	1	0.84373	.	.	1
17367	0	3.57	1	0.84373	.	.	1
17368	0	3.57	1	0.84373	.	.	1
17369	0	3.57	1	0.84373	.	.	1
17370	0	3.57	1	0.84373	.	.	1
17371	0	3.57	1	0.84373	.	.	1
17372	0	3.57	1	0.84373	.	.	1
17373	0	3.57	1	0.84373	.	.	1
17374	0	3.57	1	0.84373	.	.	1
17375	0	3.57	1	0.84373	.	.	1
17376	0	3.57	1	0.84373	.	.	1
17377	0	3.57	1	0.84373	.	.	1
17378	0	3.57	1	0.84373	.	.	1
17379	0	3.57	1	0.84373	.	.	1
17380	0	3.57	1	0.84373	.	.	1
17381	0	3.57	1	0.84373	.	.	1
17382	0	3.57	1	0.84373	.	.	1
17383	0	3.57	1	0.84373	.	.	1
17384	0	3.57	1	0.84373	.	.	1
17385	0	3.57	1	0.84373	.	.	1
17386	0	3.57	1	0.84373	.	.	1
17387	0	3.57	1	0.84373	.	.	1
17388	0	3.57	1	0.84373	.	.	1
17389	0	3.57	1	0.84373	.	.	1
17390	0	3.57	1	0.84373	.	.	1
17391	0	3.57	1	0.84373	.	.	1
17392	0	3.57	1	0.84373	.	.	1
17393	0	3.57	1	0.84373	.	.	1
17394	0	3.57	1	0.84373	.	.	1
17395	0	3.57	1	0.84373	.	.	1

Obs	Sub_group	fuptimey	_CENSOR_	SURVIVAL	SDF_LCL	SDF_UCL	STRATUM
**17396**	0	3.57	1	0.84373	.	.	1
**17397**	0	3.57	1	0.84373	.	.	1
**17398**	0	3.57	1	0.84373	.	.	1
**17399**	0	3.57	1	0.84373	.	.	1
**17400**	0	3.57	1	0.84373	.	.	1
**17401**	0	3.57	1	0.84373	.	.	1
**17402**	0	3.57	1	0.84373	.	.	1
**17403**	0	3.57	1	0.84373	.	.	1
**17404**	0	3.57	1	0.84373	.	.	1
**17405**	0	3.57	1	0.84373	.	.	1
**17406**	0	3.57	1	0.84373	.	.	1
**17407**	0	3.58	0	0.84348	0.83983	0.84706	1
**17408**	0	3.58	1	0.84348	.	.	1
**17409**	0	3.58	1	0.84348	.	.	1
**17410**	0	3.58	1	0.84348	.	.	1
**17411**	0	3.58	1	0.84348	.	.	1
**17412**	0	3.58	1	0.84348	.	.	1
**17413**	0	3.58	1	0.84348	.	.	1
**17414**	0	3.58	1	0.84348	.	.	1
**17415**	0	3.58	1	0.84348	.	.	1
**17416**	0	3.58	1	0.84348	.	.	1
**17417**	0	3.58	1	0.84348	.	.	1
**17418**	0	3.58	1	0.84348	.	.	1
**17419**	0	3.58	1	0.84348	.	.	1
**17420**	0	3.58	1	0.84348	.	.	1
**17421**	0	3.58	1	0.84348	.	.	1
**17422**	0	3.58	1	0.84348	.	.	1
**17423**	0	3.58	1	0.84348	.	.	1
**17424**	0	3.58	1	0.84348	.	.	1
**17425**	0	3.58	1	0.84348	.	.	1
**17426**	0	3.58	1	0.84348	.	.	1
**17427**	0	3.58	1	0.84348	.	.	1
**17428**	0	3.58	1	0.84348	.	.	1
**17429**	0	3.58	1	0.84348	.	.	1
**17430**	0	3.58	1	0.84348	.	.	1

Obs	Sub_group	fuptimey	_CENSOR_	SURVIVAL	SDF_LCL	SDF_UCL	STRATUM
17431	0	3.58	1	0.84348	.	.	1
17432	0	3.58	1	0.84348	.	.	1
17433	0	3.58	1	0.84348	.	.	1
17434	0	3.58	1	0.84348	.	.	1
17435	0	3.58	1	0.84348	.	.	1
17436	0	3.58	1	0.84348	.	.	1
17437	0	3.58	1	0.84348	.	.	1
17438	0	3.58	1	0.84348	.	.	1
17439	0	3.58	1	0.84348	.	.	1
17440	0	3.58	1	0.84348	.	.	1
17441	0	3.58	1	0.84348	.	.	1
17442	0	3.58	1	0.84348	.	.	1
17443	0	3.58	1	0.84348	.	.	1
17444	0	3.58	1	0.84348	.	.	1
17445	0	3.58	1	0.84348	.	.	1
17446	0	3.58	1	0.84348	.	.	1
17447	0	3.58	1	0.84348	.	.	1
17448	0	3.58	1	0.84348	.	.	1
17449	0	3.59	0	0.84323	0.83957	0.84681	1
17450	0	3.59	1	0.84323	.	.	1
17451	0	3.59	1	0.84323	.	.	1
17452	0	3.59	1	0.84323	.	.	1
17453	0	3.59	1	0.84323	.	.	1
17454	0	3.59	1	0.84323	.	.	1
17455	0	3.59	1	0.84323	.	.	1
17456	0	3.59	1	0.84323	.	.	1
17457	0	3.59	1	0.84323	.	.	1
17458	0	3.59	1	0.84323	.	.	1
17459	0	3.59	1	0.84323	.	.	1
17460	0	3.59	1	0.84323	.	.	1
17461	0	3.59	1	0.84323	.	.	1
17462	0	3.59	1	0.84323	.	.	1
17463	0	3.59	1	0.84323	.	.	1
17464	0	3.59	1	0.84323	.	.	1
17465	0	3.59	1	0.84323	.	.	1

Obs	Sub_group	fuptimey	_CENSOR_	SURVIVAL	SDF_LCL	SDF_UCL	STRATUM
17466	0	3.59	1	0.84323	.	.	1
17467	0	3.59	1	0.84323	.	.	1
17468	0	3.59	1	0.84323	.	.	1
17469	0	3.59	1	0.84323	.	.	1
17470	0	3.59	1	0.84323	.	.	1
17471	0	3.59	1	0.84323	.	.	1
17472	0	3.59	1	0.84323	.	.	1
17473	0	3.59	1	0.84323	.	.	1
17474	0	3.59	1	0.84323	.	.	1
17475	0	3.59	1	0.84323	.	.	1
17476	0	3.59	1	0.84323	.	.	1
17477	0	3.59	1	0.84323	.	.	1
17478	0	3.59	1	0.84323	.	.	1
17479	0	3.59	1	0.84323	.	.	1
17480	0	3.59	1	0.84323	.	.	1
17481	0	3.59	1	0.84323	.	.	1
17482	0	3.59	1	0.84323	.	.	1
17483	0	3.59	1	0.84323	.	.	1
17484	0	3.59	1	0.84323	.	.	1
17485	0	3.59	1	0.84323	.	.	1
17486	0	3.59	1	0.84323	.	.	1
17487	0	3.59	1	0.84323	.	.	1
17488	0	3.59	1	0.84323	.	.	1
17489	0	3.59	1	0.84323	.	.	1
17490	0	3.59	1	0.84323	.	.	1
17491	0	3.59	1	0.84323	.	.	1
17492	0	3.59	1	0.84323	.	.	1
17493	0	3.59	1	0.84323	.	.	1
17494	0	3.59	1	0.84323	.	.	1
17495	0	3.59	1	0.84323	.	.	1
17496	0	3.59	1	0.84323	.	.	1
17497	0	3.59	1	0.84323	.	.	1
17498	0	3.59	1	0.84323	.	.	1
17499	0	3.59	1	0.84323	.	.	1
17500	0	3.59	1	0.84323	.	.	1

Obs	Sub_group	fuptimey	_CENSOR_	SURVIVAL	SDF_LCL	SDF_UCL	STRATUM
**17501**	0	3.59	1	0.84323	.	.	1
**17502**	0	3.59	1	0.84323	.	.	1
**17503**	0	3.59	1	0.84323	.	.	1
**17504**	0	3.59	1	0.84323	.	.	1
**17505**	0	3.60	0	0.84297	0.83931	0.84656	1
**17506**	0	3.60	1	0.84297	.	.	1
**17507**	0	3.60	1	0.84297	.	.	1
**17508**	0	3.60	1	0.84297	.	.	1
**17509**	0	3.60	1	0.84297	.	.	1
**17510**	0	3.60	1	0.84297	.	.	1
**17511**	0	3.60	1	0.84297	.	.	1
**17512**	0	3.60	1	0.84297	.	.	1
**17513**	0	3.60	1	0.84297	.	.	1
**17514**	0	3.60	1	0.84297	.	.	1
**17515**	0	3.60	1	0.84297	.	.	1
**17516**	0	3.60	1	0.84297	.	.	1
**17517**	0	3.60	1	0.84297	.	.	1
**17518**	0	3.60	1	0.84297	.	.	1
**17519**	0	3.60	1	0.84297	.	.	1
**17520**	0	3.60	1	0.84297	.	.	1
**17521**	0	3.60	1	0.84297	.	.	1
**17522**	0	3.60	1	0.84297	.	.	1
**17523**	0	3.60	1	0.84297	.	.	1
**17524**	0	3.60	1	0.84297	.	.	1
**17525**	0	3.60	1	0.84297	.	.	1
**17526**	0	3.60	1	0.84297	.	.	1
**17527**	0	3.60	1	0.84297	.	.	1
**17528**	0	3.60	1	0.84297	.	.	1
**17529**	0	3.60	1	0.84297	.	.	1
**17530**	0	3.60	1	0.84297	.	.	1
**17531**	0	3.60	1	0.84297	.	.	1
**17532**	0	3.60	1	0.84297	.	.	1
**17533**	0	3.60	1	0.84297	.	.	1
**17534**	0	3.60	1	0.84297	.	.	1
**17535**	0	3.60	1	0.84297	.	.	1

Obs	Sub_group	fuptimey	_CENSOR_	SURVIVAL	SDF_LCL	SDF_UCL	STRATUM
**17536**	0	3.60	1	0.84297	.	.	1
**17537**	0	3.60	1	0.84297	.	.	1
**17538**	0	3.60	1	0.84297	.	.	1
**17539**	0	3.60	1	0.84297	.	.	1
**17540**	0	3.60	1	0.84297	.	.	1
**17541**	0	3.60	1	0.84297	.	.	1
**17542**	0	3.60	1	0.84297	.	.	1
**17543**	0	3.61	0	0.84279	0.83913	0.84638	1
**17544**	0	3.61	1	0.84279	.	.	1
**17545**	0	3.61	1	0.84279	.	.	1
**17546**	0	3.61	1	0.84279	.	.	1
**17547**	0	3.61	1	0.84279	.	.	1
**17548**	0	3.61	1	0.84279	.	.	1
**17549**	0	3.61	1	0.84279	.	.	1
**17550**	0	3.61	1	0.84279	.	.	1
**17551**	0	3.61	1	0.84279	.	.	1
**17552**	0	3.61	1	0.84279	.	.	1
**17553**	0	3.61	1	0.84279	.	.	1
**17554**	0	3.61	1	0.84279	.	.	1
**17555**	0	3.61	1	0.84279	.	.	1
**17556**	0	3.61	1	0.84279	.	.	1
**17557**	0	3.61	1	0.84279	.	.	1
**17558**	0	3.61	1	0.84279	.	.	1
**17559**	0	3.61	1	0.84279	.	.	1
**17560**	0	3.61	1	0.84279	.	.	1
**17561**	0	3.61	1	0.84279	.	.	1
**17562**	0	3.61	1	0.84279	.	.	1
**17563**	0	3.61	1	0.84279	.	.	1
**17564**	0	3.61	1	0.84279	.	.	1
**17565**	0	3.61	1	0.84279	.	.	1
**17566**	0	3.61	1	0.84279	.	.	1
**17567**	0	3.61	1	0.84279	.	.	1
**17568**	0	3.61	1	0.84279	.	.	1
**17569**	0	3.61	1	0.84279	.	.	1
**17570**	0	3.61	1	0.84279	.	.	1

Obs	Sub_group	fuptimey	_CENSOR_	SURVIVAL	SDF_LCL	SDF_UCL	STRATUM
17571	0	3.61	1	0.84279	.	.	1
17572	0	3.61	1	0.84279	.	.	1
17573	0	3.61	1	0.84279	.	.	1
17574	0	3.61	1	0.84279	.	.	1
17575	0	3.61	1	0.84279	.	.	1
17576	0	3.61	1	0.84279	.	.	1
17577	0	3.61	1	0.84279	.	.	1
17578	0	3.61	1	0.84279	.	.	1
17579	0	3.61	1	0.84279	.	.	1
17580	0	3.61	1	0.84279	.	.	1
17581	0	3.61	1	0.84279	.	.	1
17582	0	3.61	1	0.84279	.	.	1
17583	0	3.61	1	0.84279	.	.	1
17584	0	3.61	1	0.84279	.	.	1
17585	0	3.62	0	0.84257	0.83891	0.84616	1
17586	0	3.62	1	0.84257	.	.	1
17587	0	3.62	1	0.84257	.	.	1
17588	0	3.62	1	0.84257	.	.	1
17589	0	3.62	1	0.84257	.	.	1
17590	0	3.62	1	0.84257	.	.	1
17591	0	3.62	1	0.84257	.	.	1
17592	0	3.62	1	0.84257	.	.	1
17593	0	3.62	1	0.84257	.	.	1
17594	0	3.62	1	0.84257	.	.	1
17595	0	3.62	1	0.84257	.	.	1
17596	0	3.62	1	0.84257	.	.	1
17597	0	3.62	1	0.84257	.	.	1
17598	0	3.62	1	0.84257	.	.	1
17599	0	3.62	1	0.84257	.	.	1
17600	0	3.62	1	0.84257	.	.	1
17601	0	3.62	1	0.84257	.	.	1
17602	0	3.62	1	0.84257	.	.	1
17603	0	3.62	1	0.84257	.	.	1
17604	0	3.62	1	0.84257	.	.	1
17605	0	3.62	1	0.84257	.	.	1

Obs	Sub_group	fuptimey	_CENSOR_	SURVIVAL	SDF_LCL	SDF_UCL	STRATUM
17606	0	3.62	1	0.84257	.	.	1
17607	0	3.62	1	0.84257	.	.	1
17608	0	3.62	1	0.84257	.	.	1
17609	0	3.62	1	0.84257	.	.	1
17610	0	3.62	1	0.84257	.	.	1
17611	0	3.62	1	0.84257	.	.	1
17612	0	3.62	1	0.84257	.	.	1
17613	0	3.62	1	0.84257	.	.	1
17614	0	3.62	1	0.84257	.	.	1
17615	0	3.62	1	0.84257	.	.	1
17616	0	3.62	1	0.84257	.	.	1
17617	0	3.62	1	0.84257	.	.	1
17618	0	3.62	1	0.84257	.	.	1
17619	0	3.62	1	0.84257	.	.	1
17620	0	3.62	1	0.84257	.	.	1
17621	0	3.62	1	0.84257	.	.	1
17622	0	3.62	1	0.84257	.	.	1
17623	0	3.62	1	0.84257	.	.	1
17624	0	3.62	1	0.84257	.	.	1
17625	0	3.62	1	0.84257	.	.	1
17626	0	3.63	0	0.84250	0.83883	0.84609	1
17627	0	3.63	1	0.84250	.	.	1
17628	0	3.63	1	0.84250	.	.	1
17629	0	3.63	1	0.84250	.	.	1
17630	0	3.63	1	0.84250	.	.	1
17631	0	3.63	1	0.84250	.	.	1
17632	0	3.63	1	0.84250	.	.	1
17633	0	3.63	1	0.84250	.	.	1
17634	0	3.63	1	0.84250	.	.	1
17635	0	3.63	1	0.84250	.	.	1
17636	0	3.63	1	0.84250	.	.	1
17637	0	3.63	1	0.84250	.	.	1
17638	0	3.63	1	0.84250	.	.	1
17639	0	3.63	1	0.84250	.	.	1
17640	0	3.63	1	0.84250	.	.	1

Obs	Sub_group	fuptimey	_CENSOR_	SURVIVAL	SDF_LCL	SDF_UCL	STRATUM
**17641**	0	3.63	1	0.84250	.	.	1
**17642**	0	3.63	1	0.84250	.	.	1
**17643**	0	3.63	1	0.84250	.	.	1
**17644**	0	3.63	1	0.84250	.	.	1
**17645**	0	3.63	1	0.84250	.	.	1
**17646**	0	3.63	1	0.84250	.	.	1
**17647**	0	3.63	1	0.84250	.	.	1
**17648**	0	3.63	1	0.84250	.	.	1
**17649**	0	3.63	1	0.84250	.	.	1
**17650**	0	3.63	1	0.84250	.	.	1
**17651**	0	3.63	1	0.84250	.	.	1
**17652**	0	3.63	1	0.84250	.	.	1
**17653**	0	3.63	1	0.84250	.	.	1
**17654**	0	3.63	1	0.84250	.	.	1
**17655**	0	3.63	1	0.84250	.	.	1
**17656**	0	3.63	1	0.84250	.	.	1
**17657**	0	3.63	1	0.84250	.	.	1
**17658**	0	3.63	1	0.84250	.	.	1
**17659**	0	3.63	1	0.84250	.	.	1
**17660**	0	3.63	1	0.84250	.	.	1
**17661**	0	3.63	1	0.84250	.	.	1
**17662**	0	3.63	1	0.84250	.	.	1
**17663**	0	3.63	1	0.84250	.	.	1
**17664**	0	3.63	1	0.84250	.	.	1
**17665**	0	3.63	1	0.84250	.	.	1
**17666**	0	3.63	1	0.84250	.	.	1
**17667**	0	3.63	1	0.84250	.	.	1
**17668**	0	3.63	1	0.84250	.	.	1
**17669**	0	3.63	1	0.84250	.	.	1
**17670**	0	3.63	1	0.84250	.	.	1
**17671**	0	3.63	1	0.84250	.	.	1
**17672**	0	3.64	0	0.84231	0.83865	0.84591	1
**17673**	0	3.64	1	0.84231	.	.	1
**17674**	0	3.64	1	0.84231	.	.	1
**17675**	0	3.64	1	0.84231	.	.	1

Obs	Sub_group	fuptimey	_CENSOR_	SURVIVAL	SDF_LCL	SDF_UCL	STRATUM
17676	0	3.64	1	0.84231	.	.	1
17677	0	3.64	1	0.84231	.	.	1
17678	0	3.64	1	0.84231	.	.	1
17679	0	3.64	1	0.84231	.	.	1
17680	0	3.64	1	0.84231	.	.	1
17681	0	3.64	1	0.84231	.	.	1
17682	0	3.64	1	0.84231	.	.	1
17683	0	3.64	1	0.84231	.	.	1
17684	0	3.64	1	0.84231	.	.	1
17685	0	3.64	1	0.84231	.	.	1
17686	0	3.64	1	0.84231	.	.	1
17687	0	3.64	1	0.84231	.	.	1
17688	0	3.64	1	0.84231	.	.	1
17689	0	3.64	1	0.84231	.	.	1
17690	0	3.64	1	0.84231	.	.	1
17691	0	3.64	1	0.84231	.	.	1
17692	0	3.64	1	0.84231	.	.	1
17693	0	3.64	1	0.84231	.	.	1
17694	0	3.64	1	0.84231	.	.	1
17695	0	3.64	1	0.84231	.	.	1
17696	0	3.64	1	0.84231	.	.	1
17697	0	3.64	1	0.84231	.	.	1
17698	0	3.64	1	0.84231	.	.	1
17699	0	3.64	1	0.84231	.	.	1
17700	0	3.64	1	0.84231	.	.	1
17701	0	3.64	1	0.84231	.	.	1
17702	0	3.64	1	0.84231	.	.	1
17703	0	3.64	1	0.84231	.	.	1
17704	0	3.65	0	0.84206	0.83839	0.84566	1
17705	0	3.65	1	0.84206	.	.	1
17706	0	3.65	1	0.84206	.	.	1
17707	0	3.65	1	0.84206	.	.	1
17708	0	3.65	1	0.84206	.	.	1
17709	0	3.65	1	0.84206	.	.	1
17710	0	3.65	1	0.84206	.	.	1

Obs	Sub_group	fuptimey	_CENSOR_	SURVIVAL	SDF_LCL	SDF_UCL	STRATUM
**17711**	0	3.65	1	0.84206	.	.	1
**17712**	0	3.65	1	0.84206	.	.	1
**17713**	0	3.65	1	0.84206	.	.	1
**17714**	0	3.65	1	0.84206	.	.	1
**17715**	0	3.65	1	0.84206	.	.	1
**17716**	0	3.65	1	0.84206	.	.	1
**17717**	0	3.65	1	0.84206	.	.	1
**17718**	0	3.65	1	0.84206	.	.	1
**17719**	0	3.65	1	0.84206	.	.	1
**17720**	0	3.65	1	0.84206	.	.	1
**17721**	0	3.65	1	0.84206	.	.	1
**17722**	0	3.65	1	0.84206	.	.	1
**17723**	0	3.65	1	0.84206	.	.	1
**17724**	0	3.65	1	0.84206	.	.	1
**17725**	0	3.65	1	0.84206	.	.	1
**17726**	0	3.65	1	0.84206	.	.	1
**17727**	0	3.65	1	0.84206	.	.	1
**17728**	0	3.65	1	0.84206	.	.	1
**17729**	0	3.65	1	0.84206	.	.	1
**17730**	0	3.65	1	0.84206	.	.	1
**17731**	0	3.65	1	0.84206	.	.	1
**17732**	0	3.65	1	0.84206	.	.	1
**17733**	0	3.65	1	0.84206	.	.	1
**17734**	0	3.65	1	0.84206	.	.	1
**17735**	0	3.65	1	0.84206	.	.	1
**17736**	0	3.65	1	0.84206	.	.	1
**17737**	0	3.65	1	0.84206	.	.	1
**17738**	0	3.65	1	0.84206	.	.	1
**17739**	0	3.65	1	0.84206	.	.	1
**17740**	0	3.65	1	0.84206	.	.	1
**17741**	0	3.65	1	0.84206	.	.	1
**17742**	0	3.65	1	0.84206	.	.	1
**17743**	0	3.65	1	0.84206	.	.	1
**17744**	0	3.65	1	0.84206	.	.	1
**17745**	0	3.65	1	0.84206	.	.	1

Obs	Sub_group	fuptimey	_CENSOR_	SURVIVAL	SDF_LCL	SDF_UCL	STRATUM
**17746**	0	3.65	1	0.84206	.	.	1
**17747**	0	3.65	1	0.84206	.	.	1
**17748**	0	3.65	1	0.84206	.	.	1
**17749**	0	3.65	1	0.84206	.	.	1
**17750**	0	3.65	1	0.84206	.	.	1
**17751**	0	3.65	1	0.84206	.	.	1
**17752**	0	3.65	1	0.84206	.	.	1
**17753**	0	3.65	1	0.84206	.	.	1
**17754**	0	3.65	1	0.84206	.	.	1
**17755**	0	3.65	1	0.84206	.	.	1
**17756**	0	3.65	1	0.84206	.	.	1
**17757**	0	3.65	1	0.84206	.	.	1
**17758**	0	3.65	1	0.84206	.	.	1
**17759**	0	3.65	1	0.84206	.	.	1
**17760**	0	3.65	1	0.84206	.	.	1
**17761**	0	3.66	0	0.84198	0.83831	0.84558	1
**17762**	0	3.66	1	0.84198	.	.	1
**17763**	0	3.66	1	0.84198	.	.	1
**17764**	0	3.66	1	0.84198	.	.	1
**17765**	0	3.66	1	0.84198	.	.	1
**17766**	0	3.66	1	0.84198	.	.	1
**17767**	0	3.66	1	0.84198	.	.	1
**17768**	0	3.66	1	0.84198	.	.	1
**17769**	0	3.66	1	0.84198	.	.	1
**17770**	0	3.66	1	0.84198	.	.	1
**17771**	0	3.66	1	0.84198	.	.	1
**17772**	0	3.66	1	0.84198	.	.	1
**17773**	0	3.66	1	0.84198	.	.	1
**17774**	0	3.66	1	0.84198	.	.	1
**17775**	0	3.66	1	0.84198	.	.	1
**17776**	0	3.66	1	0.84198	.	.	1
**17777**	0	3.66	1	0.84198	.	.	1
**17778**	0	3.66	1	0.84198	.	.	1
**17779**	0	3.66	1	0.84198	.	.	1
**17780**	0	3.66	1	0.84198	.	.	1

Obs	Sub_group	fuptimey	_CENSOR_	SURVIVAL	SDF_LCL	SDF_UCL	STRATUM
**17781**	0	3.66	1	0.84198	.	.	1
**17782**	0	3.66	1	0.84198	.	.	1
**17783**	0	3.66	1	0.84198	.	.	1
**17784**	0	3.66	1	0.84198	.	.	1
**17785**	0	3.66	1	0.84198	.	.	1
**17786**	0	3.66	1	0.84198	.	.	1
**17787**	0	3.66	1	0.84198	.	.	1
**17788**	0	3.66	1	0.84198	.	.	1
**17789**	0	3.66	1	0.84198	.	.	1
**17790**	0	3.66	1	0.84198	.	.	1
**17791**	0	3.66	1	0.84198	.	.	1
**17792**	0	3.66	1	0.84198	.	.	1
**17793**	0	3.66	1	0.84198	.	.	1
**17794**	0	3.66	1	0.84198	.	.	1
**17795**	0	3.66	1	0.84198	.	.	1
**17796**	0	3.66	1	0.84198	.	.	1
**17797**	0	3.66	1	0.84198	.	.	1
**17798**	0	3.66	1	0.84198	.	.	1
**17799**	0	3.66	1	0.84198	.	.	1
**17800**	0	3.67	0	0.84165	0.83797	0.84526	1
**17801**	0	3.67	1	0.84165	.	.	1
**17802**	0	3.67	1	0.84165	.	.	1
**17803**	0	3.67	1	0.84165	.	.	1
**17804**	0	3.67	1	0.84165	.	.	1
**17805**	0	3.67	1	0.84165	.	.	1
**17806**	0	3.67	1	0.84165	.	.	1
**17807**	0	3.67	1	0.84165	.	.	1
**17808**	0	3.67	1	0.84165	.	.	1
**17809**	0	3.67	1	0.84165	.	.	1
**17810**	0	3.67	1	0.84165	.	.	1
**17811**	0	3.67	1	0.84165	.	.	1
**17812**	0	3.67	1	0.84165	.	.	1
**17813**	0	3.67	1	0.84165	.	.	1
**17814**	0	3.67	1	0.84165	.	.	1
**17815**	0	3.67	1	0.84165	.	.	1

Obs	Sub_group	fuptimey	_CENSOR_	SURVIVAL	SDF_LCL	SDF_UCL	STRATUM
**17816**	0	3.67	1	0.84165	.	.	1
**17817**	0	3.67	1	0.84165	.	.	1
**17818**	0	3.67	1	0.84165	.	.	1
**17819**	0	3.67	1	0.84165	.	.	1
**17820**	0	3.67	1	0.84165	.	.	1
**17821**	0	3.67	1	0.84165	.	.	1
**17822**	0	3.67	1	0.84165	.	.	1
**17823**	0	3.67	1	0.84165	.	.	1
**17824**	0	3.67	1	0.84165	.	.	1
**17825**	0	3.67	1	0.84165	.	.	1
**17826**	0	3.67	1	0.84165	.	.	1
**17827**	0	3.67	1	0.84165	.	.	1
**17828**	0	3.67	1	0.84165	.	.	1
**17829**	0	3.67	1	0.84165	.	.	1
**17830**	0	3.67	1	0.84165	.	.	1
**17831**	0	3.67	1	0.84165	.	.	1
**17832**	0	3.67	1	0.84165	.	.	1
**17833**	0	3.67	1	0.84165	.	.	1
**17834**	0	3.67	1	0.84165	.	.	1
**17835**	0	3.67	1	0.84165	.	.	1
**17836**	0	3.67	1	0.84165	.	.	1
**17837**	0	3.67	1	0.84165	.	.	1
**17838**	0	3.67	1	0.84165	.	.	1
**17839**	0	3.67	1	0.84165	.	.	1
**17840**	0	3.67	1	0.84165	.	.	1
**17841**	0	3.67	1	0.84165	.	.	1
**17842**	0	3.68	0	0.84158	0.83790	0.84518	1
**17843**	0	3.68	1	0.84158	.	.	1
**17844**	0	3.68	1	0.84158	.	.	1
**17845**	0	3.68	1	0.84158	.	.	1
**17846**	0	3.68	1	0.84158	.	.	1
**17847**	0	3.68	1	0.84158	.	.	1
**17848**	0	3.68	1	0.84158	.	.	1
**17849**	0	3.68	1	0.84158	.	.	1
**17850**	0	3.68	1	0.84158	.	.	1

Obs	Sub_group	fuptimey	_CENSOR_	SURVIVAL	SDF_LCL	SDF_UCL	STRATUM
17851	0	3.68	1	0.84158	.	.	1
17852	0	3.68	1	0.84158	.	.	1
17853	0	3.68	1	0.84158	.	.	1
17854	0	3.68	1	0.84158	.	.	1
17855	0	3.68	1	0.84158	.	.	1
17856	0	3.68	1	0.84158	.	.	1
17857	0	3.68	1	0.84158	.	.	1
17858	0	3.68	1	0.84158	.	.	1
17859	0	3.68	1	0.84158	.	.	1
17860	0	3.68	1	0.84158	.	.	1
17861	0	3.68	1	0.84158	.	.	1
17862	0	3.68	1	0.84158	.	.	1
17863	0	3.68	1	0.84158	.	.	1
17864	0	3.68	1	0.84158	.	.	1
17865	0	3.68	1	0.84158	.	.	1
17866	0	3.68	1	0.84158	.	.	1
17867	0	3.68	1	0.84158	.	.	1
17868	0	3.68	1	0.84158	.	.	1
17869	0	3.68	1	0.84158	.	.	1
17870	0	3.69	0	0.84117	0.83749	0.84478	1
17871	0	3.69	1	0.84117	.	.	1
17872	0	3.69	1	0.84117	.	.	1
17873	0	3.69	1	0.84117	.	.	1
17874	0	3.69	1	0.84117	.	.	1
17875	0	3.69	1	0.84117	.	.	1
17876	0	3.69	1	0.84117	.	.	1
17877	0	3.69	1	0.84117	.	.	1
17878	0	3.69	1	0.84117	.	.	1
17879	0	3.69	1	0.84117	.	.	1
17880	0	3.69	1	0.84117	.	.	1
17881	0	3.69	1	0.84117	.	.	1
17882	0	3.69	1	0.84117	.	.	1
17883	0	3.69	1	0.84117	.	.	1
17884	0	3.69	1	0.84117	.	.	1
17885	0	3.69	1	0.84117	.	.	1

Obs	Sub_group	fuptimey	_CENSOR_	SURVIVAL	SDF_LCL	SDF_UCL	STRATUM
**17886**	0	3.69	1	0.84117	.	.	1
**17887**	0	3.69	1	0.84117	.	.	1
**17888**	0	3.69	1	0.84117	.	.	1
**17889**	0	3.69	1	0.84117	.	.	1
**17890**	0	3.69	1	0.84117	.	.	1
**17891**	0	3.69	1	0.84117	.	.	1
**17892**	0	3.69	1	0.84117	.	.	1
**17893**	0	3.69	1	0.84117	.	.	1
**17894**	0	3.69	1	0.84117	.	.	1
**17895**	0	3.69	1	0.84117	.	.	1
**17896**	0	3.69	1	0.84117	.	.	1
**17897**	0	3.69	1	0.84117	.	.	1
**17898**	0	3.69	1	0.84117	.	.	1
**17899**	0	3.69	1	0.84117	.	.	1
**17900**	0	3.69	1	0.84117	.	.	1
**17901**	0	3.69	1	0.84117	.	.	1
**17902**	0	3.69	1	0.84117	.	.	1
**17903**	0	3.69	1	0.84117	.	.	1
**17904**	0	3.69	1	0.84117	.	.	1
**17905**	0	3.69	1	0.84117	.	.	1
**17906**	0	3.69	1	0.84117	.	.	1
**17907**	0	3.69	1	0.84117	.	.	1
**17908**	0	3.69	1	0.84117	.	.	1
**17909**	0	3.69	1	0.84117	.	.	1
**17910**	0	3.69	1	0.84117	.	.	1
**17911**	0	3.70	0	0.84077	0.83707	0.84438	1
**17912**	0	3.70	1	0.84077	.	.	1
**17913**	0	3.70	1	0.84077	.	.	1
**17914**	0	3.70	1	0.84077	.	.	1
**17915**	0	3.70	1	0.84077	.	.	1
**17916**	0	3.70	1	0.84077	.	.	1
**17917**	0	3.70	1	0.84077	.	.	1
**17918**	0	3.70	1	0.84077	.	.	1
**17919**	0	3.70	1	0.84077	.	.	1
**17920**	0	3.70	1	0.84077	.	.	1

Obs	Sub_group	fuptimey	_CENSOR_	SURVIVAL	SDF_LCL	SDF_UCL	STRATUM
**17921**	0	3.70	1	0.84077	.	.	1
**17922**	0	3.70	1	0.84077	.	.	1
**17923**	0	3.70	1	0.84077	.	.	1
**17924**	0	3.70	1	0.84077	.	.	1
**17925**	0	3.70	1	0.84077	.	.	1
**17926**	0	3.70	1	0.84077	.	.	1
**17927**	0	3.70	1	0.84077	.	.	1
**17928**	0	3.70	1	0.84077	.	.	1
**17929**	0	3.70	1	0.84077	.	.	1
**17930**	0	3.70	1	0.84077	.	.	1
**17931**	0	3.70	1	0.84077	.	.	1
**17932**	0	3.70	1	0.84077	.	.	1
**17933**	0	3.70	1	0.84077	.	.	1
**17934**	0	3.70	1	0.84077	.	.	1
**17935**	0	3.70	1	0.84077	.	.	1
**17936**	0	3.70	1	0.84077	.	.	1
**17937**	0	3.70	1	0.84077	.	.	1
**17938**	0	3.70	1	0.84077	.	.	1
**17939**	0	3.70	1	0.84077	.	.	1
**17940**	0	3.70	1	0.84077	.	.	1
**17941**	0	3.70	1	0.84077	.	.	1
**17942**	0	3.70	1	0.84077	.	.	1
**17943**	0	3.70	1	0.84077	.	.	1
**17944**	0	3.70	1	0.84077	.	.	1
**17945**	0	3.70	1	0.84077	.	.	1
**17946**	0	3.70	1	0.84077	.	.	1
**17947**	0	3.70	1	0.84077	.	.	1
**17948**	0	3.71	0	0.84062	0.83692	0.84424	1
**17949**	0	3.71	1	0.84062	.	.	1
**17950**	0	3.71	1	0.84062	.	.	1
**17951**	0	3.71	1	0.84062	.	.	1
**17952**	0	3.71	1	0.84062	.	.	1
**17953**	0	3.71	1	0.84062	.	.	1
**17954**	0	3.71	1	0.84062	.	.	1
**17955**	0	3.71	1	0.84062	.	.	1

Obs	Sub_group	fuptimey	_CENSOR_	SURVIVAL	SDF_LCL	SDF_UCL	STRATUM
**17956**	0	3.71	1	0.84062	.	.	1
**17957**	0	3.71	1	0.84062	.	.	1
**17958**	0	3.71	1	0.84062	.	.	1
**17959**	0	3.71	1	0.84062	.	.	1
**17960**	0	3.71	1	0.84062	.	.	1
**17961**	0	3.71	1	0.84062	.	.	1
**17962**	0	3.71	1	0.84062	.	.	1
**17963**	0	3.71	1	0.84062	.	.	1
**17964**	0	3.71	1	0.84062	.	.	1
**17965**	0	3.71	1	0.84062	.	.	1
**17966**	0	3.71	1	0.84062	.	.	1
**17967**	0	3.71	1	0.84062	.	.	1
**17968**	0	3.71	1	0.84062	.	.	1
**17969**	0	3.71	1	0.84062	.	.	1
**17970**	0	3.71	1	0.84062	.	.	1
**17971**	0	3.71	1	0.84062	.	.	1
**17972**	0	3.71	1	0.84062	.	.	1
**17973**	0	3.71	1	0.84062	.	.	1
**17974**	0	3.71	1	0.84062	.	.	1
**17975**	0	3.71	1	0.84062	.	.	1
**17976**	0	3.71	1	0.84062	.	.	1
**17977**	0	3.71	1	0.84062	.	.	1
**17978**	0	3.71	1	0.84062	.	.	1
**17979**	0	3.71	1	0.84062	.	.	1
**17980**	0	3.71	1	0.84062	.	.	1
**17981**	0	3.71	1	0.84062	.	.	1
**17982**	0	3.71	1	0.84062	.	.	1
**17983**	0	3.71	1	0.84062	.	.	1
**17984**	0	3.71	1	0.84062	.	.	1
**17985**	0	3.71	1	0.84062	.	.	1
**17986**	0	3.71	1	0.84062	.	.	1
**17987**	0	3.71	1	0.84062	.	.	1
**17988**	0	3.72	0	0.84032	0.83662	0.84394	1
**17989**	0	3.72	1	0.84032	.	.	1
**17990**	0	3.72	1	0.84032	.	.	1

Obs	Sub_group	fuptimey	_CENSOR_	SURVIVAL	SDF_LCL	SDF_UCL	STRATUM
**17991**	0	3.72	1	0.84032	.	.	1
**17992**	0	3.72	1	0.84032	.	.	1
**17993**	0	3.72	1	0.84032	.	.	1
**17994**	0	3.72	1	0.84032	.	.	1
**17995**	0	3.72	1	0.84032	.	.	1
**17996**	0	3.72	1	0.84032	.	.	1
**17997**	0	3.72	1	0.84032	.	.	1
**17998**	0	3.72	1	0.84032	.	.	1
**17999**	0	3.72	1	0.84032	.	.	1
**18000**	0	3.72	1	0.84032	.	.	1
**18001**	0	3.72	1	0.84032	.	.	1
**18002**	0	3.72	1	0.84032	.	.	1
**18003**	0	3.72	1	0.84032	.	.	1
**18004**	0	3.72	1	0.84032	.	.	1
**18005**	0	3.72	1	0.84032	.	.	1
**18006**	0	3.72	1	0.84032	.	.	1
**18007**	0	3.72	1	0.84032	.	.	1
**18008**	0	3.72	1	0.84032	.	.	1
**18009**	0	3.72	1	0.84032	.	.	1
**18010**	0	3.72	1	0.84032	.	.	1
**18011**	0	3.72	1	0.84032	.	.	1
**18012**	0	3.72	1	0.84032	.	.	1
**18013**	0	3.72	1	0.84032	.	.	1
**18014**	0	3.72	1	0.84032	.	.	1
**18015**	0	3.72	1	0.84032	.	.	1
**18016**	0	3.72	1	0.84032	.	.	1
**18017**	0	3.72	1	0.84032	.	.	1
**18018**	0	3.72	1	0.84032	.	.	1
**18019**	0	3.72	1	0.84032	.	.	1
**18020**	0	3.72	1	0.84032	.	.	1
**18021**	0	3.72	1	0.84032	.	.	1
**18022**	0	3.72	1	0.84032	.	.	1
**18023**	0	3.72	1	0.84032	.	.	1
**18024**	0	3.72	1	0.84032	.	.	1
**18025**	0	3.72	1	0.84032	.	.	1

Obs	Sub_group	fuptimey	_CENSOR_	SURVIVAL	SDF_LCL	SDF_UCL	STRATUM
**18026**	0	3.72	1	0.84032	.	.	1
**18027**	0	3.72	1	0.84032	.	.	1
**18028**	0	3.72	1	0.84032	.	.	1
**18029**	0	3.72	1	0.84032	.	.	1
**18030**	0	3.72	1	0.84032	.	.	1
**18031**	0	3.72	1	0.84032	.	.	1
**18032**	0	3.72	1	0.84032	.	.	1
**18033**	0	3.72	1	0.84032	.	.	1
**18034**	0	3.72	1	0.84032	.	.	1
**18035**	0	3.73	0	0.83999	0.83628	0.84361	1
**18036**	0	3.73	1	0.83999	.	.	1
**18037**	0	3.73	1	0.83999	.	.	1
**18038**	0	3.73	1	0.83999	.	.	1
**18039**	0	3.73	1	0.83999	.	.	1
**18040**	0	3.73	1	0.83999	.	.	1
**18041**	0	3.73	1	0.83999	.	.	1
**18042**	0	3.73	1	0.83999	.	.	1
**18043**	0	3.73	1	0.83999	.	.	1
**18044**	0	3.73	1	0.83999	.	.	1
**18045**	0	3.73	1	0.83999	.	.	1
**18046**	0	3.73	1	0.83999	.	.	1
**18047**	0	3.73	1	0.83999	.	.	1
**18048**	0	3.73	1	0.83999	.	.	1
**18049**	0	3.73	1	0.83999	.	.	1
**18050**	0	3.73	1	0.83999	.	.	1
**18051**	0	3.73	1	0.83999	.	.	1
**18052**	0	3.73	1	0.83999	.	.	1
**18053**	0	3.73	1	0.83999	.	.	1
**18054**	0	3.73	1	0.83999	.	.	1
**18055**	0	3.73	1	0.83999	.	.	1
**18056**	0	3.73	1	0.83999	.	.	1
**18057**	0	3.73	1	0.83999	.	.	1
**18058**	0	3.73	1	0.83999	.	.	1
**18059**	0	3.73	1	0.83999	.	.	1
**18060**	0	3.73	1	0.83999	.	.	1

Obs	Sub_group	fuptimey	_CENSOR_	SURVIVAL	SDF_LCL	SDF_UCL	STRATUM
18061	0	3.73	1	0.83999	.	.	1
18062	0	3.73	1	0.83999	.	.	1
18063	0	3.73	1	0.83999	.	.	1
18064	0	3.73	1	0.83999	.	.	1
18065	0	3.73	1	0.83999	.	.	1
18066	0	3.73	1	0.83999	.	.	1
18067	0	3.73	1	0.83999	.	.	1
18068	0	3.73	1	0.83999	.	.	1
18069	0	3.73	1	0.83999	.	.	1
18070	0	3.73	1	0.83999	.	.	1
18071	0	3.73	1	0.83999	.	.	1
18072	0	3.74	0	0.83976	0.83605	0.84339	1
18073	0	3.74	1	0.83976	.	.	1
18074	0	3.74	1	0.83976	.	.	1
18075	0	3.74	1	0.83976	.	.	1
18076	0	3.74	1	0.83976	.	.	1
18077	0	3.74	1	0.83976	.	.	1
18078	0	3.74	1	0.83976	.	.	1
18079	0	3.74	1	0.83976	.	.	1
18080	0	3.74	1	0.83976	.	.	1
18081	0	3.74	1	0.83976	.	.	1
18082	0	3.74	1	0.83976	.	.	1
18083	0	3.74	1	0.83976	.	.	1
18084	0	3.74	1	0.83976	.	.	1
18085	0	3.74	1	0.83976	.	.	1
18086	0	3.74	1	0.83976	.	.	1
18087	0	3.74	1	0.83976	.	.	1
18088	0	3.74	1	0.83976	.	.	1
18089	0	3.74	1	0.83976	.	.	1
18090	0	3.74	1	0.83976	.	.	1
18091	0	3.74	1	0.83976	.	.	1
18092	0	3.74	1	0.83976	.	.	1
18093	0	3.74	1	0.83976	.	.	1
18094	0	3.74	1	0.83976	.	.	1
18095	0	3.74	1	0.83976	.	.	1

Obs	Sub_group	fuptimey	_CENSOR_	SURVIVAL	SDF_LCL	SDF_UCL	STRATUM
18096	0	3.74	1	0.83976	.	.	1
18097	0	3.74	1	0.83976	.	.	1
18098	0	3.74	1	0.83976	.	.	1
18099	0	3.74	1	0.83976	.	.	1
18100	0	3.74	1	0.83976	.	.	1
18101	0	3.74	1	0.83976	.	.	1
18102	0	3.74	1	0.83976	.	.	1
18103	0	3.74	1	0.83976	.	.	1
18104	0	3.74	1	0.83976	.	.	1
18105	0	3.74	1	0.83976	.	.	1
18106	0	3.74	1	0.83976	.	.	1
18107	0	3.74	1	0.83976	.	.	1
18108	0	3.74	1	0.83976	.	.	1
18109	0	3.74	1	0.83976	.	.	1
18110	0	3.75	0	0.83965	0.83594	0.84328	1
18111	0	3.75	1	0.83965	.	.	1
18112	0	3.75	1	0.83965	.	.	1
18113	0	3.75	1	0.83965	.	.	1
18114	0	3.75	1	0.83965	.	.	1
18115	0	3.75	1	0.83965	.	.	1
18116	0	3.75	1	0.83965	.	.	1
18117	0	3.75	1	0.83965	.	.	1
18118	0	3.75	1	0.83965	.	.	1
18119	0	3.75	1	0.83965	.	.	1
18120	0	3.75	1	0.83965	.	.	1
18121	0	3.75	1	0.83965	.	.	1
18122	0	3.75	1	0.83965	.	.	1
18123	0	3.75	1	0.83965	.	.	1
18124	0	3.75	1	0.83965	.	.	1
18125	0	3.75	1	0.83965	.	.	1
18126	0	3.75	1	0.83965	.	.	1
18127	0	3.75	1	0.83965	.	.	1
18128	0	3.75	1	0.83965	.	.	1
18129	0	3.75	1	0.83965	.	.	1
18130	0	3.75	1	0.83965	.	.	1

Obs	Sub_group	fuptimey	_CENSOR_	SURVIVAL	SDF_LCL	SDF_UCL	STRATUM
**18131**	0	3.75	1	0.83965	.	.	1
**18132**	0	3.75	1	0.83965	.	.	1
**18133**	0	3.75	1	0.83965	.	.	1
**18134**	0	3.75	1	0.83965	.	.	1
**18135**	0	3.75	1	0.83965	.	.	1
**18136**	0	3.76	0	0.83961	0.83590	0.84325	1
**18137**	0	3.76	1	0.83961	.	.	1
**18138**	0	3.76	1	0.83961	.	.	1
**18139**	0	3.76	1	0.83961	.	.	1
**18140**	0	3.76	1	0.83961	.	.	1
**18141**	0	3.76	1	0.83961	.	.	1
**18142**	0	3.76	1	0.83961	.	.	1
**18143**	0	3.76	1	0.83961	.	.	1
**18144**	0	3.76	1	0.83961	.	.	1
**18145**	0	3.76	1	0.83961	.	.	1
**18146**	0	3.76	1	0.83961	.	.	1
**18147**	0	3.76	1	0.83961	.	.	1
**18148**	0	3.76	1	0.83961	.	.	1
**18149**	0	3.76	1	0.83961	.	.	1
**18150**	0	3.76	1	0.83961	.	.	1
**18151**	0	3.76	1	0.83961	.	.	1
**18152**	0	3.76	1	0.83961	.	.	1
**18153**	0	3.76	1	0.83961	.	.	1
**18154**	0	3.76	1	0.83961	.	.	1
**18155**	0	3.76	1	0.83961	.	.	1
**18156**	0	3.76	1	0.83961	.	.	1
**18157**	0	3.76	1	0.83961	.	.	1
**18158**	0	3.76	1	0.83961	.	.	1
**18159**	0	3.76	1	0.83961	.	.	1
**18160**	0	3.76	1	0.83961	.	.	1
**18161**	0	3.76	1	0.83961	.	.	1
**18162**	0	3.76	1	0.83961	.	.	1
**18163**	0	3.76	1	0.83961	.	.	1
**18164**	0	3.76	1	0.83961	.	.	1
**18165**	0	3.76	1	0.83961	.	.	1

Obs	Sub_group	fuptimey	_CENSOR_	SURVIVAL	SDF_LCL	SDF_UCL	STRATUM
**18166**	0	3.77	0	0.83943	0.83571	0.84306	1
**18167**	0	3.77	1	0.83943	.	.	1
**18168**	0	3.77	1	0.83943	.	.	1
**18169**	0	3.77	1	0.83943	.	.	1
**18170**	0	3.77	1	0.83943	.	.	1
**18171**	0	3.77	1	0.83943	.	.	1
**18172**	0	3.77	1	0.83943	.	.	1
**18173**	0	3.77	1	0.83943	.	.	1
**18174**	0	3.77	1	0.83943	.	.	1
**18175**	0	3.77	1	0.83943	.	.	1
**18176**	0	3.77	1	0.83943	.	.	1
**18177**	0	3.77	1	0.83943	.	.	1
**18178**	0	3.77	1	0.83943	.	.	1
**18179**	0	3.77	1	0.83943	.	.	1
**18180**	0	3.77	1	0.83943	.	.	1
**18181**	0	3.77	1	0.83943	.	.	1
**18182**	0	3.77	1	0.83943	.	.	1
**18183**	0	3.77	1	0.83943	.	.	1
**18184**	0	3.77	1	0.83943	.	.	1
**18185**	0	3.77	1	0.83943	.	.	1
**18186**	0	3.77	1	0.83943	.	.	1
**18187**	0	3.77	1	0.83943	.	.	1
**18188**	0	3.77	1	0.83943	.	.	1
**18189**	0	3.77	1	0.83943	.	.	1
**18190**	0	3.77	1	0.83943	.	.	1
**18191**	0	3.77	1	0.83943	.	.	1
**18192**	0	3.77	1	0.83943	.	.	1
**18193**	0	3.77	1	0.83943	.	.	1
**18194**	0	3.77	1	0.83943	.	.	1
**18195**	0	3.77	1	0.83943	.	.	1
**18196**	0	3.77	1	0.83943	.	.	1
**18197**	0	3.77	1	0.83943	.	.	1
**18198**	0	3.77	1	0.83943	.	.	1
**18199**	0	3.77	1	0.83943	.	.	1
**18200**	0	3.77	1	0.83943	.	.	1

Obs	Sub_group	fuptimey	_CENSOR_	SURVIVAL	SDF_LCL	SDF_UCL	STRATUM
18201	0	3.77	1	0.83943	.	.	1
18202	0	3.77	1	0.83943	.	.	1
18203	0	3.77	1	0.83943	.	.	1
18204	0	3.77	1	0.83943	.	.	1
18205	0	3.78	0	0.83935	0.83564	0.84299	1
18206	0	3.78	1	0.83935	.	.	1
18207	0	3.78	1	0.83935	.	.	1
18208	0	3.78	1	0.83935	.	.	1
18209	0	3.78	1	0.83935	.	.	1
18210	0	3.78	1	0.83935	.	.	1
18211	0	3.78	1	0.83935	.	.	1
18212	0	3.78	1	0.83935	.	.	1
18213	0	3.78	1	0.83935	.	.	1
18214	0	3.78	1	0.83935	.	.	1
18215	0	3.78	1	0.83935	.	.	1
18216	0	3.78	1	0.83935	.	.	1
18217	0	3.78	1	0.83935	.	.	1
18218	0	3.78	1	0.83935	.	.	1
18219	0	3.78	1	0.83935	.	.	1
18220	0	3.78	1	0.83935	.	.	1
18221	0	3.78	1	0.83935	.	.	1
18222	0	3.78	1	0.83935	.	.	1
18223	0	3.78	1	0.83935	.	.	1
18224	0	3.78	1	0.83935	.	.	1
18225	0	3.78	1	0.83935	.	.	1
18226	0	3.78	1	0.83935	.	.	1
18227	0	3.78	1	0.83935	.	.	1
18228	0	3.78	1	0.83935	.	.	1
18229	0	3.78	1	0.83935	.	.	1
18230	0	3.78	1	0.83935	.	.	1
18231	0	3.78	1	0.83935	.	.	1
18232	0	3.78	1	0.83935	.	.	1
18233	0	3.78	1	0.83935	.	.	1
18234	0	3.78	1	0.83935	.	.	1
18235	0	3.78	1	0.83935	.	.	1

Obs	Sub_group	fuptimey	_CENSOR_	SURVIVAL	SDF_LCL	SDF_UCL	STRATUM
18236	0	3.78	1	0.83935	.	.	1
18237	0	3.78	1	0.83935	.	.	1
18238	0	3.78	1	0.83935	.	.	1
18239	0	3.78	1	0.83935	.	.	1
18240	0	3.79	0	0.83924	0.83552	0.84288	1
18241	0	3.79	1	0.83924	.	.	1
18242	0	3.79	1	0.83924	.	.	1
18243	0	3.79	1	0.83924	.	.	1
18244	0	3.79	1	0.83924	.	.	1
18245	0	3.79	1	0.83924	.	.	1
18246	0	3.79	1	0.83924	.	.	1
18247	0	3.79	1	0.83924	.	.	1
18248	0	3.79	1	0.83924	.	.	1
18249	0	3.79	1	0.83924	.	.	1
18250	0	3.79	1	0.83924	.	.	1
18251	0	3.79	1	0.83924	.	.	1
18252	0	3.79	1	0.83924	.	.	1
18253	0	3.79	1	0.83924	.	.	1
18254	0	3.79	1	0.83924	.	.	1
18255	0	3.79	1	0.83924	.	.	1
18256	0	3.79	1	0.83924	.	.	1
18257	0	3.79	1	0.83924	.	.	1
18258	0	3.79	1	0.83924	.	.	1
18259	0	3.79	1	0.83924	.	.	1
18260	0	3.79	1	0.83924	.	.	1
18261	0	3.79	1	0.83924	.	.	1
18262	0	3.79	1	0.83924	.	.	1
18263	0	3.79	1	0.83924	.	.	1
18264	0	3.79	1	0.83924	.	.	1
18265	0	3.79	1	0.83924	.	.	1
18266	0	3.79	1	0.83924	.	.	1
18267	0	3.79	1	0.83924	.	.	1
18268	0	3.79	1	0.83924	.	.	1
18269	0	3.79	1	0.83924	.	.	1
18270	0	3.79	1	0.83924	.	.	1

Obs	Sub_group	fuptimey	_CENSOR_	SURVIVAL	SDF_LCL	SDF_UCL	STRATUM
18271	0	3.79	1	0.83924	.	.	1
18272	0	3.79	1	0.83924	.	.	1
18273	0	3.79	1	0.83924	.	.	1
18274	0	3.79	1	0.83924	.	.	1
18275	0	3.79	1	0.83924	.	.	1
18276	0	3.79	1	0.83924	.	.	1
18277	0	3.79	1	0.83924	.	.	1
18278	0	3.79	1	0.83924	.	.	1
18279	0	3.79	1	0.83924	.	.	1
18280	0	3.79	1	0.83924	.	.	1
18281	0	3.79	1	0.83924	.	.	1
18282	0	3.79	1	0.83924	.	.	1
18283	0	3.79	1	0.83924	.	.	1
18284	0	3.80	0	0.83897	0.83526	0.84262	1
18285	0	3.80	1	0.83897	.	.	1
18286	0	3.80	1	0.83897	.	.	1
18287	0	3.80	1	0.83897	.	.	1
18288	0	3.80	1	0.83897	.	.	1
18289	0	3.80	1	0.83897	.	.	1
18290	0	3.80	1	0.83897	.	.	1
18291	0	3.80	1	0.83897	.	.	1
18292	0	3.80	1	0.83897	.	.	1
18293	0	3.80	1	0.83897	.	.	1
18294	0	3.80	1	0.83897	.	.	1
18295	0	3.80	1	0.83897	.	.	1
18296	0	3.80	1	0.83897	.	.	1
18297	0	3.80	1	0.83897	.	.	1
18298	0	3.80	1	0.83897	.	.	1
18299	0	3.80	1	0.83897	.	.	1
18300	0	3.80	1	0.83897	.	.	1
18301	0	3.80	1	0.83897	.	.	1
18302	0	3.80	1	0.83897	.	.	1
18303	0	3.80	1	0.83897	.	.	1
18304	0	3.80	1	0.83897	.	.	1
18305	0	3.80	1	0.83897	.	.	1

Obs	Sub_group	fuptimey	_CENSOR_	SURVIVAL	SDF_LCL	SDF_UCL	STRATUM
18306	0	3.80	1	0.83897	.	.	1
18307	0	3.80	1	0.83897	.	.	1
18308	0	3.80	1	0.83897	.	.	1
18309	0	3.80	1	0.83897	.	.	1
18310	0	3.80	1	0.83897	.	.	1
18311	0	3.80	1	0.83897	.	.	1
18312	0	3.80	1	0.83897	.	.	1
18313	0	3.80	1	0.83897	.	.	1
18314	0	3.80	1	0.83897	.	.	1
18315	0	3.80	1	0.83897	.	.	1
18316	0	3.80	1	0.83897	.	.	1
18317	0	3.80	1	0.83897	.	.	1
18318	0	3.80	1	0.83897	.	.	1
18319	0	3.80	1	0.83897	.	.	1
18320	0	3.80	1	0.83897	.	.	1
18321	0	3.80	1	0.83897	.	.	1
18322	0	3.80	1	0.83897	.	.	1
18323	0	3.80	1	0.83897	.	.	1
18324	0	3.80	1	0.83897	.	.	1
18325	0	3.80	1	0.83897	.	.	1
18326	0	3.80	1	0.83897	.	.	1
18327	0	3.80	1	0.83897	.	.	1
18328	0	3.80	1	0.83897	.	.	1
18329	0	3.80	1	0.83897	.	.	1
18330	0	3.80	1	0.83897	.	.	1
18331	0	3.81	0	0.83882	0.83510	0.84247	1
18332	0	3.81	1	0.83882	.	.	1
18333	0	3.81	1	0.83882	.	.	1
18334	0	3.81	1	0.83882	.	.	1
18335	0	3.81	1	0.83882	.	.	1
18336	0	3.81	1	0.83882	.	.	1
18337	0	3.81	1	0.83882	.	.	1
18338	0	3.81	1	0.83882	.	.	1
18339	0	3.81	1	0.83882	.	.	1
18340	0	3.81	1	0.83882	.	.	1

Obs	Sub_group	fuptimey	_CENSOR_	SURVIVAL	SDF_LCL	SDF_UCL	STRATUM
18341	0	3.81	1	0.83882	.	.	1
18342	0	3.81	1	0.83882	.	.	1
18343	0	3.81	1	0.83882	.	.	1
18344	0	3.81	1	0.83882	.	.	1
18345	0	3.81	1	0.83882	.	.	1
18346	0	3.81	1	0.83882	.	.	1
18347	0	3.81	1	0.83882	.	.	1
18348	0	3.81	1	0.83882	.	.	1
18349	0	3.81	1	0.83882	.	.	1
18350	0	3.81	1	0.83882	.	.	1
18351	0	3.81	1	0.83882	.	.	1
18352	0	3.81	1	0.83882	.	.	1
18353	0	3.82	0	0.83841	0.83468	0.84206	1
18354	0	3.82	1	0.83841	.	.	1
18355	0	3.82	1	0.83841	.	.	1
18356	0	3.82	1	0.83841	.	.	1
18357	0	3.82	1	0.83841	.	.	1
18358	0	3.82	1	0.83841	.	.	1
18359	0	3.82	1	0.83841	.	.	1
18360	0	3.82	1	0.83841	.	.	1
18361	0	3.82	1	0.83841	.	.	1
18362	0	3.82	1	0.83841	.	.	1
18363	0	3.82	1	0.83841	.	.	1
18364	0	3.82	1	0.83841	.	.	1
18365	0	3.82	1	0.83841	.	.	1
18366	0	3.82	1	0.83841	.	.	1
18367	0	3.82	1	0.83841	.	.	1
18368	0	3.82	1	0.83841	.	.	1
18369	0	3.82	1	0.83841	.	.	1
18370	0	3.82	1	0.83841	.	.	1
18371	0	3.82	1	0.83841	.	.	1
18372	0	3.82	1	0.83841	.	.	1
18373	0	3.82	1	0.83841	.	.	1
18374	0	3.82	1	0.83841	.	.	1
18375	0	3.82	1	0.83841	.	.	1

Obs	Sub_group	fuptimey	_CENSOR_	SURVIVAL	SDF_LCL	SDF_UCL	STRATUM
**18376**	0	3.82	1	0.83841	.	.	1
**18377**	0	3.82	1	0.83841	.	.	1
**18378**	0	3.82	1	0.83841	.	.	1
**18379**	0	3.82	1	0.83841	.	.	1
**18380**	0	3.82	1	0.83841	.	.	1
**18381**	0	3.82	1	0.83841	.	.	1
**18382**	0	3.82	1	0.83841	.	.	1
**18383**	0	3.82	1	0.83841	.	.	1
**18384**	0	3.82	1	0.83841	.	.	1
**18385**	0	3.82	1	0.83841	.	.	1
**18386**	0	3.82	1	0.83841	.	.	1
**18387**	0	3.82	1	0.83841	.	.	1
**18388**	0	3.82	1	0.83841	.	.	1
**18389**	0	3.82	1	0.83841	.	.	1
**18390**	0	3.82	1	0.83841	.	.	1
**18391**	0	3.82	1	0.83841	.	.	1
**18392**	0	3.82	1	0.83841	.	.	1
**18393**	0	3.82	1	0.83841	.	.	1
**18394**	0	3.82	1	0.83841	.	.	1
**18395**	0	3.82	1	0.83841	.	.	1
**18396**	0	3.82	1	0.83841	.	.	1
**18397**	0	3.82	1	0.83841	.	.	1
**18398**	0	3.82	1	0.83841	.	.	1
**18399**	0	3.82	1	0.83841	.	.	1
**18400**	0	3.82	1	0.83841	.	.	1
**18401**	0	3.82	1	0.83841	.	.	1
**18402**	0	3.82	1	0.83841	.	.	1
**18403**	0	3.82	1	0.83841	.	.	1
**18404**	0	3.82	1	0.83841	.	.	1
**18405**	0	3.82	1	0.83841	.	.	1
**18406**	0	3.82	1	0.83841	.	.	1
**18407**	0	3.82	1	0.83841	.	.	1
**18408**	0	3.83	0	0.83818	0.83445	0.84184	1
**18409**	0	3.83	1	0.83818	.	.	1
**18410**	0	3.83	1	0.83818	.	.	1

Obs	Sub_group	fuptimey	_CENSOR_	SURVIVAL	SDF_LCL	SDF_UCL	STRATUM
**18411**	0	3.83	1	0.83818	.	.	1
**18412**	0	3.83	1	0.83818	.	.	1
**18413**	0	3.83	1	0.83818	.	.	1
**18414**	0	3.83	1	0.83818	.	.	1
**18415**	0	3.83	1	0.83818	.	.	1
**18416**	0	3.83	1	0.83818	.	.	1
**18417**	0	3.83	1	0.83818	.	.	1
**18418**	0	3.83	1	0.83818	.	.	1
**18419**	0	3.83	1	0.83818	.	.	1
**18420**	0	3.83	1	0.83818	.	.	1
**18421**	0	3.83	1	0.83818	.	.	1
**18422**	0	3.83	1	0.83818	.	.	1
**18423**	0	3.83	1	0.83818	.	.	1
**18424**	0	3.83	1	0.83818	.	.	1
**18425**	0	3.83	1	0.83818	.	.	1
**18426**	0	3.83	1	0.83818	.	.	1
**18427**	0	3.83	1	0.83818	.	.	1
**18428**	0	3.83	1	0.83818	.	.	1
**18429**	0	3.83	1	0.83818	.	.	1
**18430**	0	3.83	1	0.83818	.	.	1
**18431**	0	3.83	1	0.83818	.	.	1
**18432**	0	3.83	1	0.83818	.	.	1
**18433**	0	3.83	1	0.83818	.	.	1
**18434**	0	3.83	1	0.83818	.	.	1
**18435**	0	3.83	1	0.83818	.	.	1
**18436**	0	3.83	1	0.83818	.	.	1
**18437**	0	3.83	1	0.83818	.	.	1
**18438**	0	3.84	0	0.83811	0.83437	0.84176	1
**18439**	0	3.84	1	0.83811	.	.	1
**18440**	0	3.84	1	0.83811	.	.	1
**18441**	0	3.84	1	0.83811	.	.	1
**18442**	0	3.84	1	0.83811	.	.	1
**18443**	0	3.84	1	0.83811	.	.	1
**18444**	0	3.84	1	0.83811	.	.	1
**18445**	0	3.84	1	0.83811	.	.	1

Obs	Sub_group	fuptimey	_CENSOR_	SURVIVAL	SDF_LCL	SDF_UCL	STRATUM
18446	0	3.84	1	0.83811	.	.	1
18447	0	3.84	1	0.83811	.	.	1
18448	0	3.84	1	0.83811	.	.	1
18449	0	3.84	1	0.83811	.	.	1
18450	0	3.84	1	0.83811	.	.	1
18451	0	3.84	1	0.83811	.	.	1
18452	0	3.84	1	0.83811	.	.	1
18453	0	3.84	1	0.83811	.	.	1
18454	0	3.84	1	0.83811	.	.	1
18455	0	3.84	1	0.83811	.	.	1
18456	0	3.84	1	0.83811	.	.	1
18457	0	3.84	1	0.83811	.	.	1
18458	0	3.84	1	0.83811	.	.	1
18459	0	3.84	1	0.83811	.	.	1
18460	0	3.84	1	0.83811	.	.	1
18461	0	3.84	1	0.83811	.	.	1
18462	0	3.84	1	0.83811	.	.	1
18463	0	3.84	1	0.83811	.	.	1
18464	0	3.84	1	0.83811	.	.	1
18465	0	3.84	1	0.83811	.	.	1
18466	0	3.84	1	0.83811	.	.	1
18467	0	3.84	1	0.83811	.	.	1
18468	0	3.84	1	0.83811	.	.	1
18469	0	3.84	1	0.83811	.	.	1
18470	0	3.84	1	0.83811	.	.	1
18471	0	3.84	1	0.83811	.	.	1
18472	0	3.84	1	0.83811	.	.	1
18473	0	3.84	1	0.83811	.	.	1
18474	0	3.84	1	0.83811	.	.	1
18475	0	3.84	1	0.83811	.	.	1
18476	0	3.84	1	0.83811	.	.	1
18477	0	3.84	1	0.83811	.	.	1
18478	0	3.84	1	0.83811	.	.	1
18479	0	3.84	1	0.83811	.	.	1
18480	0	3.84	1	0.83811	.	.	1

Obs	Sub_group	fuptimey	_CENSOR_	SURVIVAL	SDF_LCL	SDF_UCL	STRATUM
18481	0	3.84	1	0.83811	.	.	1
18482	0	3.84	1	0.83811	.	.	1
18483	0	3.84	1	0.83811	.	.	1
18484	0	3.84	1	0.83811	.	.	1
18485	0	3.85	0	0.83795	0.83422	0.84161	1
18486	0	3.85	1	0.83795	.	.	1
18487	0	3.85	1	0.83795	.	.	1
18488	0	3.85	1	0.83795	.	.	1
18489	0	3.85	1	0.83795	.	.	1
18490	0	3.85	1	0.83795	.	.	1
18491	0	3.85	1	0.83795	.	.	1
18492	0	3.85	1	0.83795	.	.	1
18493	0	3.85	1	0.83795	.	.	1
18494	0	3.85	1	0.83795	.	.	1
18495	0	3.85	1	0.83795	.	.	1
18496	0	3.85	1	0.83795	.	.	1
18497	0	3.85	1	0.83795	.	.	1
18498	0	3.85	1	0.83795	.	.	1
18499	0	3.85	1	0.83795	.	.	1
18500	0	3.85	1	0.83795	.	.	1
18501	0	3.85	1	0.83795	.	.	1
18502	0	3.85	1	0.83795	.	.	1
18503	0	3.85	1	0.83795	.	.	1
18504	0	3.85	1	0.83795	.	.	1
18505	0	3.85	1	0.83795	.	.	1
18506	0	3.85	1	0.83795	.	.	1
18507	0	3.85	1	0.83795	.	.	1
18508	0	3.85	1	0.83795	.	.	1
18509	0	3.85	1	0.83795	.	.	1
18510	0	3.85	1	0.83795	.	.	1
18511	0	3.85	1	0.83795	.	.	1
18512	0	3.85	1	0.83795	.	.	1
18513	0	3.85	1	0.83795	.	.	1
18514	0	3.85	1	0.83795	.	.	1
18515	0	3.85	1	0.83795	.	.	1

Obs	Sub_group	fuptimey	_CENSOR_	SURVIVAL	SDF_LCL	SDF_UCL	STRATUM
**18516**	0	3.85	1	0.83795	.	.	1
**18517**	0	3.85	1	0.83795	.	.	1
**18518**	0	3.85	1	0.83795	.	.	1
**18519**	0	3.85	1	0.83795	.	.	1
**18520**	0	3.85	1	0.83795	.	.	1
**18521**	0	3.85	1	0.83795	.	.	1
**18522**	0	3.85	1	0.83795	.	.	1
**18523**	0	3.85	1	0.83795	.	.	1
**18524**	0	3.85	1	0.83795	.	.	1
**18525**	0	3.85	1	0.83795	.	.	1
**18526**	0	3.85	1	0.83795	.	.	1
**18527**	0	3.85	1	0.83795	.	.	1
**18528**	0	3.86	0	0.83773	0.83399	0.84139	1
**18529**	0	3.86	1	0.83773	.	.	1
**18530**	0	3.86	1	0.83773	.	.	1
**18531**	0	3.86	1	0.83773	.	.	1
**18532**	0	3.86	1	0.83773	.	.	1
**18533**	0	3.86	1	0.83773	.	.	1
**18534**	0	3.86	1	0.83773	.	.	1
**18535**	0	3.86	1	0.83773	.	.	1
**18536**	0	3.86	1	0.83773	.	.	1
**18537**	0	3.86	1	0.83773	.	.	1
**18538**	0	3.86	1	0.83773	.	.	1
**18539**	0	3.86	1	0.83773	.	.	1
**18540**	0	3.86	1	0.83773	.	.	1
**18541**	0	3.86	1	0.83773	.	.	1
**18542**	0	3.86	1	0.83773	.	.	1
**18543**	0	3.86	1	0.83773	.	.	1
**18544**	0	3.86	1	0.83773	.	.	1
**18545**	0	3.86	1	0.83773	.	.	1
**18546**	0	3.86	1	0.83773	.	.	1
**18547**	0	3.86	1	0.83773	.	.	1
**18548**	0	3.86	1	0.83773	.	.	1
**18549**	0	3.86	1	0.83773	.	.	1
**18550**	0	3.86	1	0.83773	.	.	1

Obs	Sub_group	fuptimey	_CENSOR_	SURVIVAL	SDF_LCL	SDF_UCL	STRATUM
**18551**	0	3.86	1	0.83773	.	.	1
**18552**	0	3.86	1	0.83773	.	.	1
**18553**	0	3.86	1	0.83773	.	.	1
**18554**	0	3.86	1	0.83773	.	.	1
**18555**	0	3.86	1	0.83773	.	.	1
**18556**	0	3.86	1	0.83773	.	.	1
**18557**	0	3.86	1	0.83773	.	.	1
**18558**	0	3.86	1	0.83773	.	.	1
**18559**	0	3.86	1	0.83773	.	.	1
**18560**	0	3.86	1	0.83773	.	.	1
**18561**	0	3.86	1	0.83773	.	.	1
**18562**	0	3.86	1	0.83773	.	.	1
**18563**	0	3.86	1	0.83773	.	.	1
**18564**	0	3.86	1	0.83773	.	.	1
**18565**	0	3.86	1	0.83773	.	.	1
**18566**	0	3.86	1	0.83773	.	.	1
**18567**	0	3.86	1	0.83773	.	.	1
**18568**	0	3.86	1	0.83773	.	.	1
**18569**	0	3.86	1	0.83773	.	.	1
**18570**	0	3.86	1	0.83773	.	.	1
**18571**	0	3.86	1	0.83773	.	.	1
**18572**	0	3.86	1	0.83773	.	.	1
**18573**	0	3.86	1	0.83773	.	.	1
**18574**	0	3.86	1	0.83773	.	.	1
**18575**	0	3.86	1	0.83773	.	.	1
**18576**	0	3.86	1	0.83773	.	.	1
**18577**	0	3.86	1	0.83773	.	.	1
**18578**	0	3.86	1	0.83773	.	.	1
**18579**	0	3.86	1	0.83773	.	.	1
**18580**	0	3.86	1	0.83773	.	.	1
**18581**	0	3.87	0	0.83765	0.83391	0.84131	1
**18582**	0	3.87	1	0.83765	.	.	1
**18583**	0	3.87	1	0.83765	.	.	1
**18584**	0	3.87	1	0.83765	.	.	1
**18585**	0	3.87	1	0.83765	.	.	1

Obs	Sub_group	fuptimey	_CENSOR_	SURVIVAL	SDF_LCL	SDF_UCL	STRATUM
**18586**	0	3.87	1	0.83765	.	.	1
**18587**	0	3.87	1	0.83765	.	.	1
**18588**	0	3.87	1	0.83765	.	.	1
**18589**	0	3.87	1	0.83765	.	.	1
**18590**	0	3.87	1	0.83765	.	.	1
**18591**	0	3.87	1	0.83765	.	.	1
**18592**	0	3.87	1	0.83765	.	.	1
**18593**	0	3.87	1	0.83765	.	.	1
**18594**	0	3.87	1	0.83765	.	.	1
**18595**	0	3.87	1	0.83765	.	.	1
**18596**	0	3.87	1	0.83765	.	.	1
**18597**	0	3.87	1	0.83765	.	.	1
**18598**	0	3.87	1	0.83765	.	.	1
**18599**	0	3.87	1	0.83765	.	.	1
**18600**	0	3.87	1	0.83765	.	.	1
**18601**	0	3.87	1	0.83765	.	.	1
**18602**	0	3.87	1	0.83765	.	.	1
**18603**	0	3.87	1	0.83765	.	.	1
**18604**	0	3.87	1	0.83765	.	.	1
**18605**	0	3.87	1	0.83765	.	.	1
**18606**	0	3.87	1	0.83765	.	.	1
**18607**	0	3.87	1	0.83765	.	.	1
**18608**	0	3.87	1	0.83765	.	.	1
**18609**	0	3.87	1	0.83765	.	.	1
**18610**	0	3.88	0	0.83731	0.83356	0.84098	1
**18611**	0	3.88	1	0.83731	.	.	1
**18612**	0	3.88	1	0.83731	.	.	1
**18613**	0	3.88	1	0.83731	.	.	1
**18614**	0	3.88	1	0.83731	.	.	1
**18615**	0	3.88	1	0.83731	.	.	1
**18616**	0	3.88	1	0.83731	.	.	1
**18617**	0	3.88	1	0.83731	.	.	1
**18618**	0	3.88	1	0.83731	.	.	1
**18619**	0	3.88	1	0.83731	.	.	1
**18620**	0	3.88	1	0.83731	.	.	1

Obs	Sub_group	fuptimey	_CENSOR_	SURVIVAL	SDF_LCL	SDF_UCL	STRATUM
18621	0	3.88	1	0.83731	.	.	1
18622	0	3.88	1	0.83731	.	.	1
18623	0	3.88	1	0.83731	.	.	1
18624	0	3.88	1	0.83731	.	.	1
18625	0	3.88	1	0.83731	.	.	1
18626	0	3.88	1	0.83731	.	.	1
18627	0	3.88	1	0.83731	.	.	1
18628	0	3.88	1	0.83731	.	.	1
18629	0	3.88	1	0.83731	.	.	1
18630	0	3.88	1	0.83731	.	.	1
18631	0	3.88	1	0.83731	.	.	1
18632	0	3.88	1	0.83731	.	.	1
18633	0	3.88	1	0.83731	.	.	1
18634	0	3.88	1	0.83731	.	.	1
18635	0	3.88	1	0.83731	.	.	1
18636	0	3.88	1	0.83731	.	.	1
18637	0	3.88	1	0.83731	.	.	1
18638	0	3.88	1	0.83731	.	.	1
18639	0	3.88	1	0.83731	.	.	1
18640	0	3.88	1	0.83731	.	.	1
18641	0	3.88	1	0.83731	.	.	1
18642	0	3.88	1	0.83731	.	.	1
18643	0	3.88	1	0.83731	.	.	1
18644	0	3.88	1	0.83731	.	.	1
18645	0	3.88	1	0.83731	.	.	1
18646	0	3.88	1	0.83731	.	.	1
18647	0	3.88	1	0.83731	.	.	1
18648	0	3.88	1	0.83731	.	.	1
18649	0	3.88	1	0.83731	.	.	1
18650	0	3.88	1	0.83731	.	.	1
18651	0	3.88	1	0.83731	.	.	1
18652	0	3.88	1	0.83731	.	.	1
18653	0	3.88	1	0.83731	.	.	1
18654	0	3.88	1	0.83731	.	.	1
18655	0	3.88	1	0.83731	.	.	1

Obs	Sub_group	fuptimey	_CENSOR_	SURVIVAL	SDF_LCL	SDF_UCL	STRATUM
18656	0	3.88	1	0.83731	.	.	1
18657	0	3.88	1	0.83731	.	.	1
18658	0	3.89	0	0.83712	0.83337	0.84079	1
18659	0	3.89	1	0.83712	.	.	1
18660	0	3.89	1	0.83712	.	.	1
18661	0	3.89	1	0.83712	.	.	1
18662	0	3.89	1	0.83712	.	.	1
18663	0	3.89	1	0.83712	.	.	1
18664	0	3.89	1	0.83712	.	.	1
18665	0	3.89	1	0.83712	.	.	1
18666	0	3.89	1	0.83712	.	.	1
18667	0	3.89	1	0.83712	.	.	1
18668	0	3.89	1	0.83712	.	.	1
18669	0	3.89	1	0.83712	.	.	1
18670	0	3.89	1	0.83712	.	.	1
18671	0	3.89	1	0.83712	.	.	1
18672	0	3.89	1	0.83712	.	.	1
18673	0	3.89	1	0.83712	.	.	1
18674	0	3.89	1	0.83712	.	.	1
18675	0	3.89	1	0.83712	.	.	1
18676	0	3.89	1	0.83712	.	.	1
18677	0	3.89	1	0.83712	.	.	1
18678	0	3.89	1	0.83712	.	.	1
18679	0	3.89	1	0.83712	.	.	1
18680	0	3.89	1	0.83712	.	.	1
18681	0	3.89	1	0.83712	.	.	1
18682	0	3.89	1	0.83712	.	.	1
18683	0	3.89	1	0.83712	.	.	1
18684	0	3.89	1	0.83712	.	.	1
18685	0	3.89	1	0.83712	.	.	1
18686	0	3.89	1	0.83712	.	.	1
18687	0	3.89	1	0.83712	.	.	1
18688	0	3.89	1	0.83712	.	.	1
18689	0	3.89	1	0.83712	.	.	1
18690	0	3.89	1	0.83712	.	.	1

Obs	Sub_group	fuptimey	_CENSOR_	SURVIVAL	SDF_LCL	SDF_UCL	STRATUM
18691	0	3.89	1	0.83712	.	.	1
18692	0	3.89	1	0.83712	.	.	1
18693	0	3.89	1	0.83712	.	.	1
18694	0	3.89	1	0.83712	.	.	1
18695	0	3.89	1	0.83712	.	.	1
18696	0	3.89	1	0.83712	.	.	1
18697	0	3.89	1	0.83712	.	.	1
18698	0	3.90	0	0.83704	0.83329	0.84071	1
18699	0	3.90	1	0.83704	.	.	1
18700	0	3.90	1	0.83704	.	.	1
18701	0	3.90	1	0.83704	.	.	1
18702	0	3.90	1	0.83704	.	.	1
18703	0	3.90	1	0.83704	.	.	1
18704	0	3.90	1	0.83704	.	.	1
18705	0	3.90	1	0.83704	.	.	1
18706	0	3.90	1	0.83704	.	.	1
18707	0	3.90	1	0.83704	.	.	1
18708	0	3.90	1	0.83704	.	.	1
18709	0	3.90	1	0.83704	.	.	1
18710	0	3.90	1	0.83704	.	.	1
18711	0	3.90	1	0.83704	.	.	1
18712	0	3.90	1	0.83704	.	.	1
18713	0	3.90	1	0.83704	.	.	1
18714	0	3.90	1	0.83704	.	.	1
18715	0	3.90	1	0.83704	.	.	1
18716	0	3.90	1	0.83704	.	.	1
18717	0	3.90	1	0.83704	.	.	1
18718	0	3.90	1	0.83704	.	.	1
18719	0	3.90	1	0.83704	.	.	1
18720	0	3.90	1	0.83704	.	.	1
18721	0	3.90	1	0.83704	.	.	1
18722	0	3.90	1	0.83704	.	.	1
18723	0	3.90	1	0.83704	.	.	1
18724	0	3.90	1	0.83704	.	.	1
18725	0	3.90	1	0.83704	.	.	1

Obs	Sub_group	fuptimey	_CENSOR_	SURVIVAL	SDF_LCL	SDF_UCL	STRATUM
**18726**	0	3.90	1	0.83704	.	.	1
**18727**	0	3.90	1	0.83704	.	.	1
**18728**	0	3.90	1	0.83704	.	.	1
**18729**	0	3.90	1	0.83704	.	.	1
**18730**	0	3.90	1	0.83704	.	.	1
**18731**	0	3.90	1	0.83704	.	.	1
**18732**	0	3.90	1	0.83704	.	.	1
**18733**	0	3.90	1	0.83704	.	.	1
**18734**	0	3.90	1	0.83704	.	.	1
**18735**	0	3.90	1	0.83704	.	.	1
**18736**	0	3.90	1	0.83704	.	.	1
**18737**	0	3.90	1	0.83704	.	.	1
**18738**	0	3.90	1	0.83704	.	.	1
**18739**	0	3.90	1	0.83704	.	.	1
**18740**	0	3.90	1	0.83704	.	.	1
**18741**	0	3.90	1	0.83704	.	.	1
**18742**	0	3.90	1	0.83704	.	.	1
**18743**	0	3.90	1	0.83704	.	.	1
**18744**	0	3.91	0	0.83677	0.83302	0.84045	1
**18745**	0	3.91	1	0.83677	.	.	1
**18746**	0	3.91	1	0.83677	.	.	1
**18747**	0	3.91	1	0.83677	.	.	1
**18748**	0	3.91	1	0.83677	.	.	1
**18749**	0	3.91	1	0.83677	.	.	1
**18750**	0	3.91	1	0.83677	.	.	1
**18751**	0	3.91	1	0.83677	.	.	1
**18752**	0	3.91	1	0.83677	.	.	1
**18753**	0	3.91	1	0.83677	.	.	1
**18754**	0	3.91	1	0.83677	.	.	1
**18755**	0	3.91	1	0.83677	.	.	1
**18756**	0	3.91	1	0.83677	.	.	1
**18757**	0	3.91	1	0.83677	.	.	1
**18758**	0	3.91	1	0.83677	.	.	1
**18759**	0	3.91	1	0.83677	.	.	1
**18760**	0	3.91	1	0.83677	.	.	1

Obs	Sub_group	fuptimey	_CENSOR_	SURVIVAL	SDF_LCL	SDF_UCL	STRATUM
18761	0	3.91	1	0.83677	.	.	1
18762	0	3.91	1	0.83677	.	.	1
18763	0	3.91	1	0.83677	.	.	1
18764	0	3.91	1	0.83677	.	.	1
18765	0	3.91	1	0.83677	.	.	1
18766	0	3.91	1	0.83677	.	.	1
18767	0	3.91	1	0.83677	.	.	1
18768	0	3.91	1	0.83677	.	.	1
18769	0	3.91	1	0.83677	.	.	1
18770	0	3.91	1	0.83677	.	.	1
18771	0	3.92	0	0.83650	0.83274	0.84018	1
18772	0	3.92	1	0.83650	.	.	1
18773	0	3.92	1	0.83650	.	.	1
18774	0	3.92	1	0.83650	.	.	1
18775	0	3.92	1	0.83650	.	.	1
18776	0	3.92	1	0.83650	.	.	1
18777	0	3.92	1	0.83650	.	.	1
18778	0	3.92	1	0.83650	.	.	1
18779	0	3.92	1	0.83650	.	.	1
18780	0	3.92	1	0.83650	.	.	1
18781	0	3.92	1	0.83650	.	.	1
18782	0	3.92	1	0.83650	.	.	1
18783	0	3.92	1	0.83650	.	.	1
18784	0	3.92	1	0.83650	.	.	1
18785	0	3.92	1	0.83650	.	.	1
18786	0	3.92	1	0.83650	.	.	1
18787	0	3.92	1	0.83650	.	.	1
18788	0	3.92	1	0.83650	.	.	1
18789	0	3.92	1	0.83650	.	.	1
18790	0	3.92	1	0.83650	.	.	1
18791	0	3.92	1	0.83650	.	.	1
18792	0	3.92	1	0.83650	.	.	1
18793	0	3.92	1	0.83650	.	.	1
18794	0	3.92	1	0.83650	.	.	1
18795	0	3.92	1	0.83650	.	.	1

Obs	Sub_group	fuptimey	_CENSOR_	SURVIVAL	SDF_LCL	SDF_UCL	STRATUM
18796	0	3.92	1	0.83650	.	.	1
18797	0	3.92	1	0.83650	.	.	1
18798	0	3.92	1	0.83650	.	.	1
18799	0	3.92	1	0.83650	.	.	1
18800	0	3.93	0	0.83635	0.83259	0.84003	1
18801	0	3.93	1	0.83635	.	.	1
18802	0	3.93	1	0.83635	.	.	1
18803	0	3.93	1	0.83635	.	.	1
18804	0	3.93	1	0.83635	.	.	1
18805	0	3.93	1	0.83635	.	.	1
18806	0	3.93	1	0.83635	.	.	1
18807	0	3.93	1	0.83635	.	.	1
18808	0	3.93	1	0.83635	.	.	1
18809	0	3.93	1	0.83635	.	.	1
18810	0	3.93	1	0.83635	.	.	1
18811	0	3.93	1	0.83635	.	.	1
18812	0	3.93	1	0.83635	.	.	1
18813	0	3.93	1	0.83635	.	.	1
18814	0	3.93	1	0.83635	.	.	1
18815	0	3.93	1	0.83635	.	.	1
18816	0	3.93	1	0.83635	.	.	1
18817	0	3.93	1	0.83635	.	.	1
18818	0	3.93	1	0.83635	.	.	1
18819	0	3.93	1	0.83635	.	.	1
18820	0	3.93	1	0.83635	.	.	1
18821	0	3.93	1	0.83635	.	.	1
18822	0	3.93	1	0.83635	.	.	1
18823	0	3.93	1	0.83635	.	.	1
18824	0	3.93	1	0.83635	.	.	1
18825	0	3.93	1	0.83635	.	.	1
18826	0	3.93	1	0.83635	.	.	1
18827	0	3.93	1	0.83635	.	.	1
18828	0	3.93	1	0.83635	.	.	1
18829	0	3.93	1	0.83635	.	.	1
18830	0	3.93	1	0.83635	.	.	1

Obs	Sub_group	fuptimey	_CENSOR_	SURVIVAL	SDF_LCL	SDF_UCL	STRATUM
**18831**	0	3.93	1	0.83635	.	.	1
**18832**	0	3.93	1	0.83635	.	.	1
**18833**	0	3.93	1	0.83635	.	.	1
**18834**	0	3.93	1	0.83635	.	.	1
**18835**	0	3.93	1	0.83635	.	.	1
**18836**	0	3.93	1	0.83635	.	.	1
**18837**	0	3.94	0	0.83608	0.83231	0.83977	1
**18838**	0	3.94	1	0.83608	.	.	1
**18839**	0	3.94	1	0.83608	.	.	1
**18840**	0	3.94	1	0.83608	.	.	1
**18841**	0	3.94	1	0.83608	.	.	1
**18842**	0	3.94	1	0.83608	.	.	1
**18843**	0	3.94	1	0.83608	.	.	1
**18844**	0	3.94	1	0.83608	.	.	1
**18845**	0	3.94	1	0.83608	.	.	1
**18846**	0	3.94	1	0.83608	.	.	1
**18847**	0	3.94	1	0.83608	.	.	1
**18848**	0	3.94	1	0.83608	.	.	1
**18849**	0	3.94	1	0.83608	.	.	1
**18850**	0	3.94	1	0.83608	.	.	1
**18851**	0	3.94	1	0.83608	.	.	1
**18852**	0	3.94	1	0.83608	.	.	1
**18853**	0	3.94	1	0.83608	.	.	1
**18854**	0	3.94	1	0.83608	.	.	1
**18855**	0	3.94	1	0.83608	.	.	1
**18856**	0	3.94	1	0.83608	.	.	1
**18857**	0	3.94	1	0.83608	.	.	1
**18858**	0	3.94	1	0.83608	.	.	1
**18859**	0	3.94	1	0.83608	.	.	1
**18860**	0	3.94	1	0.83608	.	.	1
**18861**	0	3.94	1	0.83608	.	.	1
**18862**	0	3.94	1	0.83608	.	.	1
**18863**	0	3.94	1	0.83608	.	.	1
**18864**	0	3.94	1	0.83608	.	.	1
**18865**	0	3.94	1	0.83608	.	.	1

Obs	Sub_group	fuptimey	_CENSOR_	SURVIVAL	SDF_LCL	SDF_UCL	STRATUM
**18866**	0	3.94	1	0.83608	.	.	1
**18867**	0	3.94	1	0.83608	.	.	1
**18868**	0	3.94	1	0.83608	.	.	1
**18869**	0	3.94	1	0.83608	.	.	1
**18870**	0	3.94	1	0.83608	.	.	1
**18871**	0	3.94	1	0.83608	.	.	1
**18872**	0	3.94	1	0.83608	.	.	1
**18873**	0	3.95	0	0.83592	0.83216	0.83961	1
**18874**	0	3.95	1	0.83592	.	.	1
**18875**	0	3.95	1	0.83592	.	.	1
**18876**	0	3.95	1	0.83592	.	.	1
**18877**	0	3.95	1	0.83592	.	.	1
**18878**	0	3.95	1	0.83592	.	.	1
**18879**	0	3.95	1	0.83592	.	.	1
**18880**	0	3.95	1	0.83592	.	.	1
**18881**	0	3.95	1	0.83592	.	.	1
**18882**	0	3.95	1	0.83592	.	.	1
**18883**	0	3.95	1	0.83592	.	.	1
**18884**	0	3.95	1	0.83592	.	.	1
**18885**	0	3.95	1	0.83592	.	.	1
**18886**	0	3.95	1	0.83592	.	.	1
**18887**	0	3.95	1	0.83592	.	.	1
**18888**	0	3.95	1	0.83592	.	.	1
**18889**	0	3.95	1	0.83592	.	.	1
**18890**	0	3.95	1	0.83592	.	.	1
**18891**	0	3.95	1	0.83592	.	.	1
**18892**	0	3.95	1	0.83592	.	.	1
**18893**	0	3.95	1	0.83592	.	.	1
**18894**	0	3.95	1	0.83592	.	.	1
**18895**	0	3.95	1	0.83592	.	.	1
**18896**	0	3.95	1	0.83592	.	.	1
**18897**	0	3.95	1	0.83592	.	.	1
**18898**	0	3.95	1	0.83592	.	.	1
**18899**	0	3.95	1	0.83592	.	.	1
**18900**	0	3.95	1	0.83592	.	.	1

Obs	Sub_group	fuptimey	_CENSOR_	SURVIVAL	SDF_LCL	SDF_UCL	STRATUM
18901	0	3.95	1	0.83592	.	.	1
18902	0	3.95	1	0.83592	.	.	1
18903	0	3.95	1	0.83592	.	.	1
18904	0	3.95	1	0.83592	.	.	1
18905	0	3.95	1	0.83592	.	.	1
18906	0	3.95	1	0.83592	.	.	1
18907	0	3.95	1	0.83592	.	.	1
18908	0	3.95	1	0.83592	.	.	1
18909	0	3.95	1	0.83592	.	.	1
18910	0	3.95	1	0.83592	.	.	1
18911	0	3.95	1	0.83592	.	.	1
18912	0	3.95	1	0.83592	.	.	1
18913	0	3.95	1	0.83592	.	.	1
18914	0	3.95	1	0.83592	.	.	1
18915	0	3.95	1	0.83592	.	.	1
18916	0	3.96	0	0.83554	0.83176	0.83923	1
18917	0	3.96	1	0.83554	.	.	1
18918	0	3.96	1	0.83554	.	.	1
18919	0	3.96	1	0.83554	.	.	1
18920	0	3.96	1	0.83554	.	.	1
18921	0	3.96	1	0.83554	.	.	1
18922	0	3.96	1	0.83554	.	.	1
18923	0	3.96	1	0.83554	.	.	1
18924	0	3.96	1	0.83554	.	.	1
18925	0	3.96	1	0.83554	.	.	1
18926	0	3.96	1	0.83554	.	.	1
18927	0	3.96	1	0.83554	.	.	1
18928	0	3.96	1	0.83554	.	.	1
18929	0	3.96	1	0.83554	.	.	1
18930	0	3.96	1	0.83554	.	.	1
18931	0	3.96	1	0.83554	.	.	1
18932	0	3.96	1	0.83554	.	.	1
18933	0	3.96	1	0.83554	.	.	1
18934	0	3.96	1	0.83554	.	.	1
18935	0	3.96	1	0.83554	.	.	1

Obs	Sub_group	fuptimey	_CENSOR_	SURVIVAL	SDF_LCL	SDF_UCL	STRATUM
18936	0	3.96	1	0.83554	.	.	1
18937	0	3.96	1	0.83554	.	.	1
18938	0	3.96	1	0.83554	.	.	1
18939	0	3.96	1	0.83554	.	.	1
18940	0	3.96	1	0.83554	.	.	1
18941	0	3.96	1	0.83554	.	.	1
18942	0	3.96	1	0.83554	.	.	1
18943	0	3.96	1	0.83554	.	.	1
18944	0	3.96	1	0.83554	.	.	1
18945	0	3.96	1	0.83554	.	.	1
18946	0	3.96	1	0.83554	.	.	1
18947	0	3.96	1	0.83554	.	.	1
18948	0	3.96	1	0.83554	.	.	1
18949	0	3.96	1	0.83554	.	.	1
18950	0	3.96	1	0.83554	.	.	1
18951	0	3.96	1	0.83554	.	.	1
18952	0	3.96	1	0.83554	.	.	1
18953	0	3.97	0	0.83523	0.83145	0.83893	1
18954	0	3.97	1	0.83523	.	.	1
18955	0	3.97	1	0.83523	.	.	1
18956	0	3.97	1	0.83523	.	.	1
18957	0	3.97	1	0.83523	.	.	1
18958	0	3.97	1	0.83523	.	.	1
18959	0	3.97	1	0.83523	.	.	1
18960	0	3.97	1	0.83523	.	.	1
18961	0	3.97	1	0.83523	.	.	1
18962	0	3.97	1	0.83523	.	.	1
18963	0	3.97	1	0.83523	.	.	1
18964	0	3.97	1	0.83523	.	.	1
18965	0	3.97	1	0.83523	.	.	1
18966	0	3.97	1	0.83523	.	.	1
18967	0	3.97	1	0.83523	.	.	1
18968	0	3.97	1	0.83523	.	.	1
18969	0	3.97	1	0.83523	.	.	1
18970	0	3.97	1	0.83523	.	.	1

Obs	Sub_group	fuptimey	_CENSOR_	SURVIVAL	SDF_LCL	SDF_UCL	STRATUM
**18971**	0	3.97	1	0.83523	.	.	1
**18972**	0	3.97	1	0.83523	.	.	1
**18973**	0	3.97	1	0.83523	.	.	1
**18974**	0	3.97	1	0.83523	.	.	1
**18975**	0	3.97	1	0.83523	.	.	1
**18976**	0	3.97	1	0.83523	.	.	1
**18977**	0	3.97	1	0.83523	.	.	1
**18978**	0	3.97	1	0.83523	.	.	1
**18979**	0	3.97	1	0.83523	.	.	1
**18980**	0	3.97	1	0.83523	.	.	1
**18981**	0	3.97	1	0.83523	.	.	1
**18982**	0	3.97	1	0.83523	.	.	1
**18983**	0	3.97	1	0.83523	.	.	1
**18984**	0	3.97	1	0.83523	.	.	1
**18985**	0	3.97	1	0.83523	.	.	1
**18986**	0	3.98	0	0.83499	0.83121	0.83870	1
**18987**	0	3.98	1	0.83499	.	.	1
**18988**	0	3.98	1	0.83499	.	.	1
**18989**	0	3.98	1	0.83499	.	.	1
**18990**	0	3.98	1	0.83499	.	.	1
**18991**	0	3.98	1	0.83499	.	.	1
**18992**	0	3.98	1	0.83499	.	.	1
**18993**	0	3.98	1	0.83499	.	.	1
**18994**	0	3.98	1	0.83499	.	.	1
**18995**	0	3.98	1	0.83499	.	.	1
**18996**	0	3.98	1	0.83499	.	.	1
**18997**	0	3.98	1	0.83499	.	.	1
**18998**	0	3.98	1	0.83499	.	.	1
**18999**	0	3.98	1	0.83499	.	.	1
**19000**	0	3.98	1	0.83499	.	.	1
**19001**	0	3.98	1	0.83499	.	.	1
**19002**	0	3.98	1	0.83499	.	.	1
**19003**	0	3.98	1	0.83499	.	.	1
**19004**	0	3.98	1	0.83499	.	.	1
**19005**	0	3.98	1	0.83499	.	.	1

Obs	Sub_group	fuptimey	_CENSOR_	SURVIVAL	SDF_LCL	SDF_UCL	STRATUM
19006	0	3.98	1	0.83499	.	.	1
19007	0	3.98	1	0.83499	.	.	1
19008	0	3.98	1	0.83499	.	.	1
19009	0	3.98	1	0.83499	.	.	1
19010	0	3.98	1	0.83499	.	.	1
19011	0	3.98	1	0.83499	.	.	1
19012	0	3.99	0	0.83484	0.83106	0.83855	1
19013	0	3.99	1	0.83484	.	.	1
19014	0	3.99	1	0.83484	.	.	1
19015	0	3.99	1	0.83484	.	.	1
19016	0	3.99	1	0.83484	.	.	1
19017	0	3.99	1	0.83484	.	.	1
19018	0	3.99	1	0.83484	.	.	1
19019	0	3.99	1	0.83484	.	.	1
19020	0	3.99	1	0.83484	.	.	1
19021	0	3.99	1	0.83484	.	.	1
19022	0	3.99	1	0.83484	.	.	1
19023	0	3.99	1	0.83484	.	.	1
19024	0	3.99	1	0.83484	.	.	1
19025	0	3.99	1	0.83484	.	.	1
19026	0	3.99	1	0.83484	.	.	1
19027	0	3.99	1	0.83484	.	.	1
19028	0	3.99	1	0.83484	.	.	1
19029	0	3.99	1	0.83484	.	.	1
19030	0	3.99	1	0.83484	.	.	1
19031	0	3.99	1	0.83484	.	.	1
19032	0	3.99	1	0.83484	.	.	1
19033	0	3.99	1	0.83484	.	.	1
19034	0	3.99	1	0.83484	.	.	1
19035	0	3.99	1	0.83484	.	.	1
19036	0	3.99	1	0.83484	.	.	1
19037	0	3.99	1	0.83484	.	.	1
19038	0	3.99	1	0.83484	.	.	1
19039	0	3.99	1	0.83484	.	.	1
19040	0	3.99	1	0.83484	.	.	1

Obs	Sub_group	fuptimey	_CENSOR_	SURVIVAL	SDF_LCL	SDF_UCL	STRATUM
**19041**	0	3.99	1	0.83484	.	.	1
**19042**	0	3.99	1	0.83484	.	.	1
**19043**	0	3.99	1	0.83484	.	.	1
**19044**	0	3.99	1	0.83484	.	.	1
**19045**	0	3.99	1	0.83484	.	.	1
**19046**	0	3.99	1	0.83484	.	.	1
**19047**	0	3.99	1	0.83484	.	.	1
**19048**	0	3.99	1	0.83484	.	.	1
**19049**	0	3.99	1	0.83484	.	.	1
**19050**	0	3.99	1	0.83484	.	.	1
**19051**	0	3.99	1	0.83484	.	.	1
**19052**	0	3.99	1	0.83484	.	.	1
**19053**	0	4.00	0	0.83445	0.83066	0.83816	1
**19054**	0	4.00	1	0.83445	.	.	1
**19055**	0	4.00	1	0.83445	.	.	1
**19056**	0	4.00	1	0.83445	.	.	1
**19057**	0	4.00	1	0.83445	.	.	1
**19058**	0	4.00	1	0.83445	.	.	1
**19059**	0	4.00	1	0.83445	.	.	1
**19060**	0	4.00	1	0.83445	.	.	1
**19061**	0	4.00	1	0.83445	.	.	1
**19062**	0	4.00	1	0.83445	.	.	1
**19063**	0	4.00	1	0.83445	.	.	1
**19064**	0	4.00	1	0.83445	.	.	1
**19065**	0	4.00	1	0.83445	.	.	1
**19066**	0	4.00	1	0.83445	.	.	1
**19067**	0	4.00	1	0.83445	.	.	1
**19068**	0	4.00	1	0.83445	.	.	1
**19069**	0	4.00	1	0.83445	.	.	1
**19070**	0	4.00	1	0.83445	.	.	1
**19071**	0	4.00	1	0.83445	.	.	1
**19072**	0	4.00	1	0.83445	.	.	1
**19073**	0	4.00	1	0.83445	.	.	1
**19074**	0	4.00	1	0.83445	.	.	1
**19075**	0	4.00	1	0.83445	.	.	1

Obs	Sub_group	fuptimey	_CENSOR_	SURVIVAL	SDF_LCL	SDF_UCL	STRATUM
19076	0	4.00	1	0.83445	.	.	1
19077	0	4.00	1	0.83445	.	.	1
19078	0	4.00	1	0.83445	.	.	1
19079	0	4.00	1	0.83445	.	.	1
19080	0	4.00	1	0.83445	.	.	1
19081	0	4.00	1	0.83445	.	.	1
19082	0	4.00	1	0.83445	.	.	1
19083	0	4.00	1	0.83445	.	.	1
19084	0	4.00	1	0.83445	.	.	1
19085	0	4.00	1	0.83445	.	.	1
19086	0	4.00	1	0.83445	.	.	1
19087	0	4.01	0	0.83437	0.83058	0.83809	1
19088	0	4.01	1	0.83437	.	.	1
19089	0	4.01	1	0.83437	.	.	1
19090	0	4.01	1	0.83437	.	.	1
19091	0	4.01	1	0.83437	.	.	1
19092	0	4.01	1	0.83437	.	.	1
19093	0	4.01	1	0.83437	.	.	1
19094	0	4.01	1	0.83437	.	.	1
19095	0	4.01	1	0.83437	.	.	1
19096	0	4.01	1	0.83437	.	.	1
19097	0	4.01	1	0.83437	.	.	1
19098	0	4.01	1	0.83437	.	.	1
19099	0	4.01	1	0.83437	.	.	1
19100	0	4.01	1	0.83437	.	.	1
19101	0	4.01	1	0.83437	.	.	1
19102	0	4.01	1	0.83437	.	.	1
19103	0	4.01	1	0.83437	.	.	1
19104	0	4.01	1	0.83437	.	.	1
19105	0	4.01	1	0.83437	.	.	1
19106	0	4.01	1	0.83437	.	.	1
19107	0	4.01	1	0.83437	.	.	1
19108	0	4.01	1	0.83437	.	.	1
19109	0	4.01	1	0.83437	.	.	1
19110	0	4.01	1	0.83437	.	.	1

Obs	Sub_group	fuptimey	_CENSOR_	SURVIVAL	SDF_LCL	SDF_UCL	STRATUM
**19111**	0	4.01	1	0.83437	.	.	1
**19112**	0	4.01	1	0.83437	.	.	1
**19113**	0	4.01	1	0.83437	.	.	1
**19114**	0	4.01	1	0.83437	.	.	1
**19115**	0	4.01	1	0.83437	.	.	1
**19116**	0	4.01	1	0.83437	.	.	1
**19117**	0	4.01	1	0.83437	.	.	1
**19118**	0	4.01	1	0.83437	.	.	1
**19119**	0	4.01	1	0.83437	.	.	1
**19120**	0	4.01	1	0.83437	.	.	1
**19121**	0	4.01	1	0.83437	.	.	1
**19122**	0	4.01	1	0.83437	.	.	1
**19123**	0	4.01	1	0.83437	.	.	1
**19124**	0	4.01	1	0.83437	.	.	1
**19125**	0	4.01	1	0.83437	.	.	1
**19126**	0	4.01	1	0.83437	.	.	1
**19127**	0	4.01	1	0.83437	.	.	1
**19128**	0	4.01	1	0.83437	.	.	1
**19129**	0	4.01	1	0.83437	.	.	1
**19130**	0	4.01	1	0.83437	.	.	1
**19131**	0	4.01	1	0.83437	.	.	1
**19132**	0	4.01	1	0.83437	.	.	1
**19133**	0	4.01	1	0.83437	.	.	1
**19134**	0	4.01	1	0.83437	.	.	1
**19135**	0	4.01	1	0.83437	.	.	1
**19136**	0	4.01	1	0.83437	.	.	1
**19137**	0	4.02	0	0.83418	0.83038	0.83789	1
**19138**	0	4.02	1	0.83418	.	.	1
**19139**	0	4.02	1	0.83418	.	.	1
**19140**	0	4.02	1	0.83418	.	.	1
**19141**	0	4.02	1	0.83418	.	.	1
**19142**	0	4.02	1	0.83418	.	.	1
**19143**	0	4.02	1	0.83418	.	.	1
**19144**	0	4.02	1	0.83418	.	.	1
**19145**	0	4.02	1	0.83418	.	.	1

Obs	Sub_group	fuptimey	_CENSOR_	SURVIVAL	SDF_LCL	SDF_UCL	STRATUM
**19146**	0	4.02	1	0.83418	.	.	1
**19147**	0	4.02	1	0.83418	.	.	1
**19148**	0	4.02	1	0.83418	.	.	1
**19149**	0	4.02	1	0.83418	.	.	1
**19150**	0	4.02	1	0.83418	.	.	1
**19151**	0	4.02	1	0.83418	.	.	1
**19152**	0	4.02	1	0.83418	.	.	1
**19153**	0	4.02	1	0.83418	.	.	1
**19154**	0	4.02	1	0.83418	.	.	1
**19155**	0	4.02	1	0.83418	.	.	1
**19156**	0	4.02	1	0.83418	.	.	1
**19157**	0	4.02	1	0.83418	.	.	1
**19158**	0	4.02	1	0.83418	.	.	1
**19159**	0	4.02	1	0.83418	.	.	1
**19160**	0	4.02	1	0.83418	.	.	1
**19161**	0	4.03	0	0.83390	0.83010	0.83762	1
**19162**	0	4.03	1	0.83390	.	.	1
**19163**	0	4.03	1	0.83390	.	.	1
**19164**	0	4.03	1	0.83390	.	.	1
**19165**	0	4.03	1	0.83390	.	.	1
**19166**	0	4.03	1	0.83390	.	.	1
**19167**	0	4.03	1	0.83390	.	.	1
**19168**	0	4.03	1	0.83390	.	.	1
**19169**	0	4.03	1	0.83390	.	.	1
**19170**	0	4.03	1	0.83390	.	.	1
**19171**	0	4.03	1	0.83390	.	.	1
**19172**	0	4.03	1	0.83390	.	.	1
**19173**	0	4.03	1	0.83390	.	.	1
**19174**	0	4.03	1	0.83390	.	.	1
**19175**	0	4.03	1	0.83390	.	.	1
**19176**	0	4.03	1	0.83390	.	.	1
**19177**	0	4.03	1	0.83390	.	.	1
**19178**	0	4.03	1	0.83390	.	.	1
**19179**	0	4.03	1	0.83390	.	.	1
**19180**	0	4.03	1	0.83390	.	.	1

Obs	Sub_group	fuptimey	_CENSOR_	SURVIVAL	SDF_LCL	SDF_UCL	STRATUM
19181	0	4.03	1	0.83390	.	.	1
19182	0	4.03	1	0.83390	.	.	1
19183	0	4.03	1	0.83390	.	.	1
19184	0	4.03	1	0.83390	.	.	1
19185	0	4.03	1	0.83390	.	.	1
19186	0	4.03	1	0.83390	.	.	1
19187	0	4.03	1	0.83390	.	.	1
19188	0	4.03	1	0.83390	.	.	1
19189	0	4.03	1	0.83390	.	.	1
19190	0	4.03	1	0.83390	.	.	1
19191	0	4.03	1	0.83390	.	.	1
19192	0	4.03	1	0.83390	.	.	1
19193	0	4.03	1	0.83390	.	.	1
19194	0	4.03	1	0.83390	.	.	1
19195	0	4.03	1	0.83390	.	.	1
19196	0	4.03	1	0.83390	.	.	1
19197	0	4.03	1	0.83390	.	.	1
19198	0	4.03	1	0.83390	.	.	1
19199	0	4.03	1	0.83390	.	.	1
19200	0	4.03	1	0.83390	.	.	1
19201	0	4.03	1	0.83390	.	.	1
19202	0	4.03	1	0.83390	.	.	1
19203	0	4.04	0	0.83367	0.82987	0.83739	1
19204	0	4.04	1	0.83367	.	.	1
19205	0	4.04	1	0.83367	.	.	1
19206	0	4.04	1	0.83367	.	.	1
19207	0	4.04	1	0.83367	.	.	1
19208	0	4.04	1	0.83367	.	.	1
19209	0	4.04	1	0.83367	.	.	1
19210	0	4.04	1	0.83367	.	.	1
19211	0	4.04	1	0.83367	.	.	1
19212	0	4.04	1	0.83367	.	.	1
19213	0	4.04	1	0.83367	.	.	1
19214	0	4.04	1	0.83367	.	.	1
19215	0	4.04	1	0.83367	.	.	1

Obs	Sub_group	fuptimey	_CENSOR_	SURVIVAL	SDF_LCL	SDF_UCL	STRATUM
**19216**	0	4.04	1	0.83367	.	.	1
**19217**	0	4.04	1	0.83367	.	.	1
**19218**	0	4.04	1	0.83367	.	.	1
**19219**	0	4.04	1	0.83367	.	.	1
**19220**	0	4.04	1	0.83367	.	.	1
**19221**	0	4.04	1	0.83367	.	.	1
**19222**	0	4.04	1	0.83367	.	.	1
**19223**	0	4.04	1	0.83367	.	.	1
**19224**	0	4.04	1	0.83367	.	.	1
**19225**	0	4.04	1	0.83367	.	.	1
**19226**	0	4.04	1	0.83367	.	.	1
**19227**	0	4.04	1	0.83367	.	.	1
**19228**	0	4.04	1	0.83367	.	.	1
**19229**	0	4.04	1	0.83367	.	.	1
**19230**	0	4.04	1	0.83367	.	.	1
**19231**	0	4.04	1	0.83367	.	.	1
**19232**	0	4.04	1	0.83367	.	.	1
**19233**	0	4.05	0	0.83355	0.82975	0.83728	1
**19234**	0	4.05	1	0.83355	.	.	1
**19235**	0	4.05	1	0.83355	.	.	1
**19236**	0	4.05	1	0.83355	.	.	1
**19237**	0	4.05	1	0.83355	.	.	1
**19238**	0	4.05	1	0.83355	.	.	1
**19239**	0	4.05	1	0.83355	.	.	1
**19240**	0	4.05	1	0.83355	.	.	1
**19241**	0	4.05	1	0.83355	.	.	1
**19242**	0	4.05	1	0.83355	.	.	1
**19243**	0	4.05	1	0.83355	.	.	1
**19244**	0	4.05	1	0.83355	.	.	1
**19245**	0	4.05	1	0.83355	.	.	1
**19246**	0	4.05	1	0.83355	.	.	1
**19247**	0	4.05	1	0.83355	.	.	1
**19248**	0	4.05	1	0.83355	.	.	1
**19249**	0	4.05	1	0.83355	.	.	1
**19250**	0	4.05	1	0.83355	.	.	1

Obs	Sub_group	fuptimey	_CENSOR_	SURVIVAL	SDF_LCL	SDF_UCL	STRATUM
**19251**	0	4.05	1	0.83355	.	.	1
**19252**	0	4.05	1	0.83355	.	.	1
**19253**	0	4.05	1	0.83355	.	.	1
**19254**	0	4.05	1	0.83355	.	.	1
**19255**	0	4.05	1	0.83355	.	.	1
**19256**	0	4.05	1	0.83355	.	.	1
**19257**	0	4.05	1	0.83355	.	.	1
**19258**	0	4.05	1	0.83355	.	.	1
**19259**	0	4.05	1	0.83355	.	.	1
**19260**	0	4.05	1	0.83355	.	.	1
**19261**	0	4.05	1	0.83355	.	.	1
**19262**	0	4.05	1	0.83355	.	.	1
**19263**	0	4.05	1	0.83355	.	.	1
**19264**	0	4.05	1	0.83355	.	.	1
**19265**	0	4.05	1	0.83355	.	.	1
**19266**	0	4.05	1	0.83355	.	.	1
**19267**	0	4.05	1	0.83355	.	.	1
**19268**	0	4.05	1	0.83355	.	.	1
**19269**	0	4.05	1	0.83355	.	.	1
**19270**	0	4.05	1	0.83355	.	.	1
**19271**	0	4.05	1	0.83355	.	.	1
**19272**	0	4.05	1	0.83355	.	.	1
**19273**	0	4.05	1	0.83355	.	.	1
**19274**	0	4.05	1	0.83355	.	.	1
**19275**	0	4.05	1	0.83355	.	.	1
**19276**	0	4.06	0	0.83343	0.82963	0.83716	1
**19277**	0	4.06	1	0.83343	.	.	1
**19278**	0	4.06	1	0.83343	.	.	1
**19279**	0	4.06	1	0.83343	.	.	1
**19280**	0	4.06	1	0.83343	.	.	1
**19281**	0	4.06	1	0.83343	.	.	1
**19282**	0	4.06	1	0.83343	.	.	1
**19283**	0	4.06	1	0.83343	.	.	1
**19284**	0	4.06	1	0.83343	.	.	1
**19285**	0	4.06	1	0.83343	.	.	1

Obs	Sub_group	fuptimey	_CENSOR_	SURVIVAL	SDF_LCL	SDF_UCL	STRATUM
**19286**	0	4.06	1	0.83343	.	.	1
**19287**	0	4.06	1	0.83343	.	.	1
**19288**	0	4.06	1	0.83343	.	.	1
**19289**	0	4.06	1	0.83343	.	.	1
**19290**	0	4.06	1	0.83343	.	.	1
**19291**	0	4.06	1	0.83343	.	.	1
**19292**	0	4.06	1	0.83343	.	.	1
**19293**	0	4.06	1	0.83343	.	.	1
**19294**	0	4.06	1	0.83343	.	.	1
**19295**	0	4.06	1	0.83343	.	.	1
**19296**	0	4.06	1	0.83343	.	.	1
**19297**	0	4.06	1	0.83343	.	.	1
**19298**	0	4.06	1	0.83343	.	.	1
**19299**	0	4.06	1	0.83343	.	.	1
**19300**	0	4.06	1	0.83343	.	.	1
**19301**	0	4.06	1	0.83343	.	.	1
**19302**	0	4.06	1	0.83343	.	.	1
**19303**	0	4.06	1	0.83343	.	.	1
**19304**	0	4.06	1	0.83343	.	.	1
**19305**	0	4.06	1	0.83343	.	.	1
**19306**	0	4.06	1	0.83343	.	.	1
**19307**	0	4.06	1	0.83343	.	.	1
**19308**	0	4.06	1	0.83343	.	.	1
**19309**	0	4.06	1	0.83343	.	.	1
**19310**	0	4.06	1	0.83343	.	.	1
**19311**	0	4.06	1	0.83343	.	.	1
**19312**	0	4.06	1	0.83343	.	.	1
**19313**	0	4.06	1	0.83343	.	.	1
**19314**	0	4.06	1	0.83343	.	.	1
**19315**	0	4.06	1	0.83343	.	.	1
**19316**	0	4.06	1	0.83343	.	.	1
**19317**	0	4.06	1	0.83343	.	.	1
**19318**	0	4.06	1	0.83343	.	.	1
**19319**	0	4.06	1	0.83343	.	.	1
**19320**	0	4.06	1	0.83343	.	.	1

Obs	Sub_group	fuptimey	_CENSOR_	SURVIVAL	SDF_LCL	SDF_UCL	STRATUM
19321	0	4.06	1	0.83343	.	.	1
19322	0	4.06	1	0.83343	.	.	1
19323	0	4.06	1	0.83343	.	.	1
19324	0	4.06	1	0.83343	.	.	1
19325	0	4.06	1	0.83343	.	.	1
19326	0	4.07	0	0.83316	0.82935	0.83689	1
19327	0	4.07	1	0.83316	.	.	1
19328	0	4.07	1	0.83316	.	.	1
19329	0	4.07	1	0.83316	.	.	1
19330	0	4.07	1	0.83316	.	.	1
19331	0	4.07	1	0.83316	.	.	1
19332	0	4.07	1	0.83316	.	.	1
19333	0	4.07	1	0.83316	.	.	1
19334	0	4.07	1	0.83316	.	.	1
19335	0	4.07	1	0.83316	.	.	1
19336	0	4.07	1	0.83316	.	.	1
19337	0	4.07	1	0.83316	.	.	1
19338	0	4.07	1	0.83316	.	.	1
19339	0	4.07	1	0.83316	.	.	1
19340	0	4.07	1	0.83316	.	.	1
19341	0	4.07	1	0.83316	.	.	1
19342	0	4.07	1	0.83316	.	.	1
19343	0	4.07	1	0.83316	.	.	1
19344	0	4.07	1	0.83316	.	.	1
19345	0	4.07	1	0.83316	.	.	1
19346	0	4.07	1	0.83316	.	.	1
19347	0	4.07	1	0.83316	.	.	1
19348	0	4.07	1	0.83316	.	.	1
19349	0	4.07	1	0.83316	.	.	1
19350	0	4.07	1	0.83316	.	.	1
19351	0	4.07	1	0.83316	.	.	1
19352	0	4.07	1	0.83316	.	.	1
19353	0	4.07	1	0.83316	.	.	1
19354	0	4.07	1	0.83316	.	.	1
19355	0	4.07	1	0.83316	.	.	1

Obs	Sub_group	fuptimey	_CENSOR_	SURVIVAL	SDF_LCL	SDF_UCL	STRATUM
19356	0	4.07	1	0.83316	.	.	1
19357	0	4.07	1	0.83316	.	.	1
19358	0	4.07	1	0.83316	.	.	1
19359	0	4.07	1	0.83316	.	.	1
19360	0	4.07	1	0.83316	.	.	1
19361	0	4.07	1	0.83316	.	.	1
19362	0	4.07	1	0.83316	.	.	1
19363	0	4.07	1	0.83316	.	.	1
19364	0	4.07	1	0.83316	.	.	1
19365	0	4.07	1	0.83316	.	.	1
19366	0	4.07	1	0.83316	.	.	1
19367	0	4.07	1	0.83316	.	.	1
19368	0	4.08	0	0.83296	0.82915	0.83669	1
19369	0	4.08	1	0.83296	.	.	1
19370	0	4.08	1	0.83296	.	.	1
19371	0	4.08	1	0.83296	.	.	1
19372	0	4.08	1	0.83296	.	.	1
19373	0	4.08	1	0.83296	.	.	1
19374	0	4.08	1	0.83296	.	.	1
19375	0	4.08	1	0.83296	.	.	1
19376	0	4.08	1	0.83296	.	.	1
19377	0	4.08	1	0.83296	.	.	1
19378	0	4.08	1	0.83296	.	.	1
19379	0	4.08	1	0.83296	.	.	1
19380	0	4.08	1	0.83296	.	.	1
19381	0	4.08	1	0.83296	.	.	1
19382	0	4.08	1	0.83296	.	.	1
19383	0	4.08	1	0.83296	.	.	1
19384	0	4.08	1	0.83296	.	.	1
19385	0	4.08	1	0.83296	.	.	1
19386	0	4.08	1	0.83296	.	.	1
19387	0	4.08	1	0.83296	.	.	1
19388	0	4.08	1	0.83296	.	.	1
19389	0	4.08	1	0.83296	.	.	1
19390	0	4.08	1	0.83296	.	.	1

Obs	Sub_group	fuptimey	_CENSOR_	SURVIVAL	SDF_LCL	SDF_UCL	STRATUM
19391	0	4.08	1	0.83296	.	.	1
19392	0	4.08	1	0.83296	.	.	1
19393	0	4.08	1	0.83296	.	.	1
19394	0	4.08	1	0.83296	.	.	1
19395	0	4.08	1	0.83296	.	.	1
19396	0	4.08	1	0.83296	.	.	1
19397	0	4.08	1	0.83296	.	.	1
19398	0	4.08	1	0.83296	.	.	1
19399	0	4.08	1	0.83296	.	.	1
19400	0	4.08	1	0.83296	.	.	1
19401	0	4.09	0	0.83272	0.82890	0.83646	1
19402	0	4.09	1	0.83272	.	.	1
19403	0	4.09	1	0.83272	.	.	1
19404	0	4.09	1	0.83272	.	.	1
19405	0	4.09	1	0.83272	.	.	1
19406	0	4.09	1	0.83272	.	.	1
19407	0	4.09	1	0.83272	.	.	1
19408	0	4.09	1	0.83272	.	.	1
19409	0	4.09	1	0.83272	.	.	1
19410	0	4.09	1	0.83272	.	.	1
19411	0	4.09	1	0.83272	.	.	1
19412	0	4.09	1	0.83272	.	.	1
19413	0	4.09	1	0.83272	.	.	1
19414	0	4.09	1	0.83272	.	.	1
19415	0	4.09	1	0.83272	.	.	1
19416	0	4.09	1	0.83272	.	.	1
19417	0	4.09	1	0.83272	.	.	1
19418	0	4.09	1	0.83272	.	.	1
19419	0	4.09	1	0.83272	.	.	1
19420	0	4.09	1	0.83272	.	.	1
19421	0	4.09	1	0.83272	.	.	1
19422	0	4.09	1	0.83272	.	.	1
19423	0	4.09	1	0.83272	.	.	1
19424	0	4.09	1	0.83272	.	.	1
19425	0	4.09	1	0.83272	.	.	1

Obs	Sub_group	fuptimey	_CENSOR_	SURVIVAL	SDF_LCL	SDF_UCL	STRATUM
**19426**	0	4.09	1	0.83272	.	.	1
**19427**	0	4.09	1	0.83272	.	.	1
**19428**	0	4.09	1	0.83272	.	.	1
**19429**	0	4.09	1	0.83272	.	.	1
**19430**	0	4.09	1	0.83272	.	.	1
**19431**	0	4.09	1	0.83272	.	.	1
**19432**	0	4.09	1	0.83272	.	.	1
**19433**	0	4.09	1	0.83272	.	.	1
**19434**	0	4.09	1	0.83272	.	.	1
**19435**	0	4.09	1	0.83272	.	.	1
**19436**	0	4.09	1	0.83272	.	.	1
**19437**	0	4.09	1	0.83272	.	.	1
**19438**	0	4.09	1	0.83272	.	.	1
**19439**	0	4.09	1	0.83272	.	.	1
**19440**	0	4.09	1	0.83272	.	.	1
**19441**	0	4.09	1	0.83272	.	.	1
**19442**	0	4.09	1	0.83272	.	.	1
**19443**	0	4.09	1	0.83272	.	.	1
**19444**	0	4.10	0	0.83252	0.82870	0.83627	1
**19445**	0	4.10	1	0.83252	.	.	1
**19446**	0	4.10	1	0.83252	.	.	1
**19447**	0	4.10	1	0.83252	.	.	1
**19448**	0	4.10	1	0.83252	.	.	1
**19449**	0	4.10	1	0.83252	.	.	1
**19450**	0	4.10	1	0.83252	.	.	1
**19451**	0	4.10	1	0.83252	.	.	1
**19452**	0	4.10	1	0.83252	.	.	1
**19453**	0	4.10	1	0.83252	.	.	1
**19454**	0	4.10	1	0.83252	.	.	1
**19455**	0	4.10	1	0.83252	.	.	1
**19456**	0	4.10	1	0.83252	.	.	1
**19457**	0	4.10	1	0.83252	.	.	1
**19458**	0	4.10	1	0.83252	.	.	1
**19459**	0	4.10	1	0.83252	.	.	1
**19460**	0	4.10	1	0.83252	.	.	1

Obs	Sub_group	fuptimey	_CENSOR_	SURVIVAL	SDF_LCL	SDF_UCL	STRATUM
19461	0	4.10	1	0.83252	.	.	1
19462	0	4.10	1	0.83252	.	.	1
19463	0	4.10	1	0.83252	.	.	1
19464	0	4.10	1	0.83252	.	.	1
19465	0	4.10	1	0.83252	.	.	1
19466	0	4.10	1	0.83252	.	.	1
19467	0	4.10	1	0.83252	.	.	1
19468	0	4.10	1	0.83252	.	.	1
19469	0	4.10	1	0.83252	.	.	1
19470	0	4.10	1	0.83252	.	.	1
19471	0	4.10	1	0.83252	.	.	1
19472	0	4.10	1	0.83252	.	.	1
19473	0	4.10	1	0.83252	.	.	1
19474	0	4.10	1	0.83252	.	.	1
19475	0	4.10	1	0.83252	.	.	1
19476	0	4.10	1	0.83252	.	.	1
19477	0	4.10	1	0.83252	.	.	1
19478	0	4.10	1	0.83252	.	.	1
19479	0	4.11	0	0.83232	0.82850	0.83607	1
19480	0	4.11	1	0.83232	.	.	1
19481	0	4.11	1	0.83232	.	.	1
19482	0	4.11	1	0.83232	.	.	1
19483	0	4.11	1	0.83232	.	.	1
19484	0	4.11	1	0.83232	.	.	1
19485	0	4.11	1	0.83232	.	.	1
19486	0	4.11	1	0.83232	.	.	1
19487	0	4.11	1	0.83232	.	.	1
19488	0	4.11	1	0.83232	.	.	1
19489	0	4.11	1	0.83232	.	.	1
19490	0	4.11	1	0.83232	.	.	1
19491	0	4.11	1	0.83232	.	.	1
19492	0	4.11	1	0.83232	.	.	1
19493	0	4.11	1	0.83232	.	.	1
19494	0	4.11	1	0.83232	.	.	1
19495	0	4.11	1	0.83232	.	.	1

Obs	Sub_group	fuptimey	_CENSOR_	SURVIVAL	SDF_LCL	SDF_UCL	STRATUM
**19496**	0	4.11	1	0.83232	.	.	1
**19497**	0	4.11	1	0.83232	.	.	1
**19498**	0	4.11	1	0.83232	.	.	1
**19499**	0	4.11	1	0.83232	.	.	1
**19500**	0	4.11	1	0.83232	.	.	1
**19501**	0	4.11	1	0.83232	.	.	1
**19502**	0	4.11	1	0.83232	.	.	1
**19503**	0	4.11	1	0.83232	.	.	1
**19504**	0	4.11	1	0.83232	.	.	1
**19505**	0	4.11	1	0.83232	.	.	1
**19506**	0	4.11	1	0.83232	.	.	1
**19507**	0	4.11	1	0.83232	.	.	1
**19508**	0	4.11	1	0.83232	.	.	1
**19509**	0	4.11	1	0.83232	.	.	1
**19510**	0	4.11	1	0.83232	.	.	1
**19511**	0	4.11	1	0.83232	.	.	1
**19512**	0	4.11	1	0.83232	.	.	1
**19513**	0	4.11	1	0.83232	.	.	1
**19514**	0	4.11	1	0.83232	.	.	1
**19515**	0	4.11	1	0.83232	.	.	1
**19516**	0	4.11	1	0.83232	.	.	1
**19517**	0	4.11	1	0.83232	.	.	1
**19518**	0	4.11	1	0.83232	.	.	1
**19519**	0	4.11	1	0.83232	.	.	1
**19520**	0	4.11	1	0.83232	.	.	1
**19521**	0	4.11	1	0.83232	.	.	1
**19522**	0	4.11	1	0.83232	.	.	1
**19523**	0	4.12	0	0.83217	0.82834	0.83591	1
**19524**	0	4.12	1	0.83217	.	.	1
**19525**	0	4.12	1	0.83217	.	.	1
**19526**	0	4.12	1	0.83217	.	.	1
**19527**	0	4.12	1	0.83217	.	.	1
**19528**	0	4.12	1	0.83217	.	.	1
**19529**	0	4.12	1	0.83217	.	.	1
**19530**	0	4.12	1	0.83217	.	.	1

Obs	Sub_group	fuptimey	_CENSOR_	SURVIVAL	SDF_LCL	SDF_UCL	STRATUM
19531	0	4.12	1	0.83217	.	.	1
19532	0	4.12	1	0.83217	.	.	1
19533	0	4.12	1	0.83217	.	.	1
19534	0	4.12	1	0.83217	.	.	1
19535	0	4.12	1	0.83217	.	.	1
19536	0	4.12	1	0.83217	.	.	1
19537	0	4.12	1	0.83217	.	.	1
19538	0	4.12	1	0.83217	.	.	1
19539	0	4.12	1	0.83217	.	.	1
19540	0	4.12	1	0.83217	.	.	1
19541	0	4.12	1	0.83217	.	.	1
19542	0	4.12	1	0.83217	.	.	1
19543	0	4.12	1	0.83217	.	.	1
19544	0	4.12	1	0.83217	.	.	1
19545	0	4.12	1	0.83217	.	.	1
19546	0	4.12	1	0.83217	.	.	1
19547	0	4.12	1	0.83217	.	.	1
19548	0	4.12	1	0.83217	.	.	1
19549	0	4.12	1	0.83217	.	.	1
19550	0	4.12	1	0.83217	.	.	1
19551	0	4.12	1	0.83217	.	.	1
19552	0	4.12	1	0.83217	.	.	1
19553	0	4.12	1	0.83217	.	.	1
19554	0	4.12	1	0.83217	.	.	1
19555	0	4.12	1	0.83217	.	.	1
19556	0	4.12	1	0.83217	.	.	1
19557	0	4.12	1	0.83217	.	.	1
19558	0	4.13	0	0.83197	0.82814	0.83572	1
19559	0	4.13	1	0.83197	.	.	1
19560	0	4.13	1	0.83197	.	.	1
19561	0	4.13	1	0.83197	.	.	1
19562	0	4.13	1	0.83197	.	.	1
19563	0	4.13	1	0.83197	.	.	1
19564	0	4.13	1	0.83197	.	.	1
19565	0	4.13	1	0.83197	.	.	1

Obs	Sub_group	fuptimey	_CENSOR_	SURVIVAL	SDF_LCL	SDF_UCL	STRATUM
19566	0	4.13	1	0.83197	.	.	1
19567	0	4.13	1	0.83197	.	.	1
19568	0	4.13	1	0.83197	.	.	1
19569	0	4.13	1	0.83197	.	.	1
19570	0	4.13	1	0.83197	.	.	1
19571	0	4.13	1	0.83197	.	.	1
19572	0	4.13	1	0.83197	.	.	1
19573	0	4.13	1	0.83197	.	.	1
19574	0	4.13	1	0.83197	.	.	1
19575	0	4.13	1	0.83197	.	.	1
19576	0	4.13	1	0.83197	.	.	1
19577	0	4.13	1	0.83197	.	.	1
19578	0	4.13	1	0.83197	.	.	1
19579	0	4.13	1	0.83197	.	.	1
19580	0	4.13	1	0.83197	.	.	1
19581	0	4.13	1	0.83197	.	.	1
19582	0	4.13	1	0.83197	.	.	1
19583	0	4.13	1	0.83197	.	.	1
19584	0	4.13	1	0.83197	.	.	1
19585	0	4.13	1	0.83197	.	.	1
19586	0	4.13	1	0.83197	.	.	1
19587	0	4.13	1	0.83197	.	.	1
19588	0	4.13	1	0.83197	.	.	1
19589	0	4.13	1	0.83197	.	.	1
19590	0	4.13	1	0.83197	.	.	1
19591	0	4.13	1	0.83197	.	.	1
19592	0	4.13	1	0.83197	.	.	1
19593	0	4.13	1	0.83197	.	.	1
19594	0	4.13	1	0.83197	.	.	1
19595	0	4.13	1	0.83197	.	.	1
19596	0	4.13	1	0.83197	.	.	1
19597	0	4.13	1	0.83197	.	.	1
19598	0	4.13	1	0.83197	.	.	1
19599	0	4.13	1	0.83197	.	.	1
19600	0	4.13	1	0.83197	.	.	1

Obs	Sub_group	fuptimey	_CENSOR_	SURVIVAL	SDF_LCL	SDF_UCL	STRATUM
19601	0	4.13	1	0.83197	.	.	1
19602	0	4.13	1	0.83197	.	.	1
19603	0	4.13	1	0.83197	.	.	1
19604	0	4.13	1	0.83197	.	.	1
19605	0	4.13	1	0.83197	.	.	1
19606	0	4.13	1	0.83197	.	.	1
19607	0	4.13	1	0.83197	.	.	1
19608	0	4.14	0	0.83189	0.82806	0.83564	1
19609	0	4.14	1	0.83189	.	.	1
19610	0	4.14	1	0.83189	.	.	1
19611	0	4.14	1	0.83189	.	.	1
19612	0	4.14	1	0.83189	.	.	1
19613	0	4.14	1	0.83189	.	.	1
19614	0	4.14	1	0.83189	.	.	1
19615	0	4.14	1	0.83189	.	.	1
19616	0	4.14	1	0.83189	.	.	1
19617	0	4.14	1	0.83189	.	.	1
19618	0	4.14	1	0.83189	.	.	1
19619	0	4.14	1	0.83189	.	.	1
19620	0	4.14	1	0.83189	.	.	1
19621	0	4.14	1	0.83189	.	.	1
19622	0	4.14	1	0.83189	.	.	1
19623	0	4.14	1	0.83189	.	.	1
19624	0	4.14	1	0.83189	.	.	1
19625	0	4.14	1	0.83189	.	.	1
19626	0	4.14	1	0.83189	.	.	1
19627	0	4.14	1	0.83189	.	.	1
19628	0	4.14	1	0.83189	.	.	1
19629	0	4.14	1	0.83189	.	.	1
19630	0	4.14	1	0.83189	.	.	1
19631	0	4.14	1	0.83189	.	.	1
19632	0	4.14	1	0.83189	.	.	1
19633	0	4.14	1	0.83189	.	.	1
19634	0	4.14	1	0.83189	.	.	1
19635	0	4.14	1	0.83189	.	.	1

Obs	Sub_group	fuptimey	_CENSOR_	SURVIVAL	SDF_LCL	SDF_UCL	STRATUM
19636	0	4.14	1	0.83189	.	.	1
19637	0	4.14	1	0.83189	.	.	1
19638	0	4.14	1	0.83189	.	.	1
19639	0	4.14	1	0.83189	.	.	1
19640	0	4.15	0	0.83185	0.82802	0.83560	1
19641	0	4.15	1	0.83185	.	.	1
19642	0	4.15	1	0.83185	.	.	1
19643	0	4.15	1	0.83185	.	.	1
19644	0	4.15	1	0.83185	.	.	1
19645	0	4.15	1	0.83185	.	.	1
19646	0	4.15	1	0.83185	.	.	1
19647	0	4.15	1	0.83185	.	.	1
19648	0	4.15	1	0.83185	.	.	1
19649	0	4.15	1	0.83185	.	.	1
19650	0	4.15	1	0.83185	.	.	1
19651	0	4.15	1	0.83185	.	.	1
19652	0	4.15	1	0.83185	.	.	1
19653	0	4.15	1	0.83185	.	.	1
19654	0	4.15	1	0.83185	.	.	1
19655	0	4.15	1	0.83185	.	.	1
19656	0	4.15	1	0.83185	.	.	1
19657	0	4.15	1	0.83185	.	.	1
19658	0	4.15	1	0.83185	.	.	1
19659	0	4.15	1	0.83185	.	.	1
19660	0	4.15	1	0.83185	.	.	1
19661	0	4.15	1	0.83185	.	.	1
19662	0	4.15	1	0.83185	.	.	1
19663	0	4.15	1	0.83185	.	.	1
19664	0	4.15	1	0.83185	.	.	1
19665	0	4.15	1	0.83185	.	.	1
19666	0	4.15	1	0.83185	.	.	1
19667	0	4.15	1	0.83185	.	.	1
19668	0	4.15	1	0.83185	.	.	1
19669	0	4.15	1	0.83185	.	.	1
19670	0	4.15	1	0.83185	.	.	1

Obs	Sub_group	fuptimey	_CENSOR_	SURVIVAL	SDF_LCL	SDF_UCL	STRATUM
**19671**	0	4.15	1	0.83185	.	.	1
**19672**	0	4.15	1	0.83185	.	.	1
**19673**	0	4.15	1	0.83185	.	.	1
**19674**	0	4.15	1	0.83185	.	.	1
**19675**	0	4.15	1	0.83185	.	.	1
**19676**	0	4.15	1	0.83185	.	.	1
**19677**	0	4.15	1	0.83185	.	.	1
**19678**	0	4.15	1	0.83185	.	.	1
**19679**	0	4.15	1	0.83185	.	.	1
**19680**	0	4.15	1	0.83185	.	.	1
**19681**	0	4.15	1	0.83185	.	.	1
**19682**	0	4.15	1	0.83185	.	.	1
**19683**	0	4.16	0	0.83153	0.82769	0.83528	1
**19684**	0	4.16	1	0.83153	.	.	1
**19685**	0	4.16	1	0.83153	.	.	1
**19686**	0	4.16	1	0.83153	.	.	1
**19687**	0	4.16	1	0.83153	.	.	1
**19688**	0	4.16	1	0.83153	.	.	1
**19689**	0	4.16	1	0.83153	.	.	1
**19690**	0	4.16	1	0.83153	.	.	1
**19691**	0	4.16	1	0.83153	.	.	1
**19692**	0	4.16	1	0.83153	.	.	1
**19693**	0	4.16	1	0.83153	.	.	1
**19694**	0	4.16	1	0.83153	.	.	1
**19695**	0	4.16	1	0.83153	.	.	1
**19696**	0	4.16	1	0.83153	.	.	1
**19697**	0	4.16	1	0.83153	.	.	1
**19698**	0	4.16	1	0.83153	.	.	1
**19699**	0	4.16	1	0.83153	.	.	1
**19700**	0	4.16	1	0.83153	.	.	1
**19701**	0	4.16	1	0.83153	.	.	1
**19702**	0	4.16	1	0.83153	.	.	1
**19703**	0	4.16	1	0.83153	.	.	1
**19704**	0	4.16	1	0.83153	.	.	1
**19705**	0	4.16	1	0.83153	.	.	1

Obs	Sub_group	fuptimey	_CENSOR_	SURVIVAL	SDF_LCL	SDF_UCL	STRATUM
19706	0	4.16	1	0.83153	.	.	1
19707	0	4.16	1	0.83153	.	.	1
19708	0	4.16	1	0.83153	.	.	1
19709	0	4.16	1	0.83153	.	.	1
19710	0	4.16	1	0.83153	.	.	1
19711	0	4.17	0	0.83133	0.82749	0.83509	1
19712	0	4.17	1	0.83133	.	.	1
19713	0	4.17	1	0.83133	.	.	1
19714	0	4.17	1	0.83133	.	.	1
19715	0	4.17	1	0.83133	.	.	1
19716	0	4.17	1	0.83133	.	.	1
19717	0	4.17	1	0.83133	.	.	1
19718	0	4.17	1	0.83133	.	.	1
19719	0	4.17	1	0.83133	.	.	1
19720	0	4.17	1	0.83133	.	.	1
19721	0	4.17	1	0.83133	.	.	1
19722	0	4.17	1	0.83133	.	.	1
19723	0	4.17	1	0.83133	.	.	1
19724	0	4.17	1	0.83133	.	.	1
19725	0	4.17	1	0.83133	.	.	1
19726	0	4.17	1	0.83133	.	.	1
19727	0	4.17	1	0.83133	.	.	1
19728	0	4.17	1	0.83133	.	.	1
19729	0	4.17	1	0.83133	.	.	1
19730	0	4.17	1	0.83133	.	.	1
19731	0	4.17	1	0.83133	.	.	1
19732	0	4.17	1	0.83133	.	.	1
19733	0	4.17	1	0.83133	.	.	1
19734	0	4.17	1	0.83133	.	.	1
19735	0	4.17	1	0.83133	.	.	1
19736	0	4.17	1	0.83133	.	.	1
19737	0	4.17	1	0.83133	.	.	1
19738	0	4.17	1	0.83133	.	.	1
19739	0	4.17	1	0.83133	.	.	1
19740	0	4.17	1	0.83133	.	.	1

Obs	Sub_group	fuptimey	_CENSOR_	SURVIVAL	SDF_LCL	SDF_UCL	STRATUM
**19741**	0	4.17	1	0.83133	.	.	1
**19742**	0	4.17	1	0.83133	.	.	1
**19743**	0	4.17	1	0.83133	.	.	1
**19744**	0	4.17	1	0.83133	.	.	1
**19745**	0	4.17	1	0.83133	.	.	1
**19746**	0	4.17	1	0.83133	.	.	1
**19747**	0	4.17	1	0.83133	.	.	1
**19748**	0	4.17	1	0.83133	.	.	1
**19749**	0	4.17	1	0.83133	.	.	1
**19750**	0	4.17	1	0.83133	.	.	1
**19751**	0	4.17	1	0.83133	.	.	1
**19752**	0	4.17	1	0.83133	.	.	1
**19753**	0	4.17	1	0.83133	.	.	1
**19754**	0	4.17	1	0.83133	.	.	1
**19755**	0	4.17	1	0.83133	.	.	1
**19756**	0	4.17	1	0.83133	.	.	1
**19757**	0	4.18	0	0.83120	0.82736	0.83497	1
**19758**	0	4.18	1	0.83120	.	.	1
**19759**	0	4.18	1	0.83120	.	.	1
**19760**	0	4.18	1	0.83120	.	.	1
**19761**	0	4.18	1	0.83120	.	.	1
**19762**	0	4.18	1	0.83120	.	.	1
**19763**	0	4.18	1	0.83120	.	.	1
**19764**	0	4.18	1	0.83120	.	.	1
**19765**	0	4.18	1	0.83120	.	.	1
**19766**	0	4.18	1	0.83120	.	.	1
**19767**	0	4.18	1	0.83120	.	.	1
**19768**	0	4.18	1	0.83120	.	.	1
**19769**	0	4.18	1	0.83120	.	.	1
**19770**	0	4.18	1	0.83120	.	.	1
**19771**	0	4.18	1	0.83120	.	.	1
**19772**	0	4.18	1	0.83120	.	.	1
**19773**	0	4.18	1	0.83120	.	.	1
**19774**	0	4.18	1	0.83120	.	.	1
**19775**	0	4.18	1	0.83120	.	.	1

Obs	Sub_group	fuptimey	_CENSOR_	SURVIVAL	SDF_LCL	SDF_UCL	STRATUM
**19776**	0	4.18	1	0.83120	.	.	1
**19777**	0	4.18	1	0.83120	.	.	1
**19778**	0	4.18	1	0.83120	.	.	1
**19779**	0	4.18	1	0.83120	.	.	1
**19780**	0	4.18	1	0.83120	.	.	1
**19781**	0	4.19	0	0.83100	0.82716	0.83477	1
**19782**	0	4.19	1	0.83100	.	.	1
**19783**	0	4.19	1	0.83100	.	.	1
**19784**	0	4.19	1	0.83100	.	.	1
**19785**	0	4.19	1	0.83100	.	.	1
**19786**	0	4.19	1	0.83100	.	.	1
**19787**	0	4.19	1	0.83100	.	.	1
**19788**	0	4.19	1	0.83100	.	.	1
**19789**	0	4.19	1	0.83100	.	.	1
**19790**	0	4.19	1	0.83100	.	.	1
**19791**	0	4.19	1	0.83100	.	.	1
**19792**	0	4.19	1	0.83100	.	.	1
**19793**	0	4.19	1	0.83100	.	.	1
**19794**	0	4.19	1	0.83100	.	.	1
**19795**	0	4.19	1	0.83100	.	.	1
**19796**	0	4.19	1	0.83100	.	.	1
**19797**	0	4.19	1	0.83100	.	.	1
**19798**	0	4.19	1	0.83100	.	.	1
**19799**	0	4.19	1	0.83100	.	.	1
**19800**	0	4.19	1	0.83100	.	.	1
**19801**	0	4.19	1	0.83100	.	.	1
**19802**	0	4.19	1	0.83100	.	.	1
**19803**	0	4.19	1	0.83100	.	.	1
**19804**	0	4.19	1	0.83100	.	.	1
**19805**	0	4.19	1	0.83100	.	.	1
**19806**	0	4.19	1	0.83100	.	.	1
**19807**	0	4.19	1	0.83100	.	.	1
**19808**	0	4.19	1	0.83100	.	.	1
**19809**	0	4.19	1	0.83100	.	.	1
**19810**	0	4.19	1	0.83100	.	.	1

Obs	Sub_group	fuptimey	_CENSOR_	SURVIVAL	SDF_LCL	SDF_UCL	STRATUM
**19811**	0	4.19	1	0.83100	.	.	1
**19812**	0	4.19	1	0.83100	.	.	1
**19813**	0	4.19	1	0.83100	.	.	1
**19814**	0	4.19	1	0.83100	.	.	1
**19815**	0	4.19	1	0.83100	.	.	1
**19816**	0	4.19	1	0.83100	.	.	1
**19817**	0	4.19	1	0.83100	.	.	1
**19818**	0	4.19	1	0.83100	.	.	1
**19819**	0	4.19	1	0.83100	.	.	1
**19820**	0	4.19	1	0.83100	.	.	1
**19821**	0	4.19	1	0.83100	.	.	1
**19822**	0	4.19	1	0.83100	.	.	1
**19823**	0	4.19	1	0.83100	.	.	1
**19824**	0	4.19	1	0.83100	.	.	1
**19825**	0	4.19	1	0.83100	.	.	1
**19826**	0	4.20	0	0.83092	0.82708	0.83469	1
**19827**	0	4.20	1	0.83092	.	.	1
**19828**	0	4.20	1	0.83092	.	.	1
**19829**	0	4.20	1	0.83092	.	.	1
**19830**	0	4.20	1	0.83092	.	.	1
**19831**	0	4.20	1	0.83092	.	.	1
**19832**	0	4.20	1	0.83092	.	.	1
**19833**	0	4.20	1	0.83092	.	.	1
**19834**	0	4.20	1	0.83092	.	.	1
**19835**	0	4.20	1	0.83092	.	.	1
**19836**	0	4.20	1	0.83092	.	.	1
**19837**	0	4.20	1	0.83092	.	.	1
**19838**	0	4.20	1	0.83092	.	.	1
**19839**	0	4.20	1	0.83092	.	.	1
**19840**	0	4.20	1	0.83092	.	.	1
**19841**	0	4.20	1	0.83092	.	.	1
**19842**	0	4.20	1	0.83092	.	.	1
**19843**	0	4.20	1	0.83092	.	.	1
**19844**	0	4.20	1	0.83092	.	.	1
**19845**	0	4.20	1	0.83092	.	.	1

Obs	Sub_group	fuptimey	_CENSOR_	SURVIVAL	SDF_LCL	SDF_UCL	STRATUM
19846	0	4.20	1	0.83092	.	.	1
19847	0	4.20	1	0.83092	.	.	1
19848	0	4.20	1	0.83092	.	.	1
19849	0	4.20	1	0.83092	.	.	1
19850	0	4.20	1	0.83092	.	.	1
19851	0	4.20	1	0.83092	.	.	1
19852	0	4.20	1	0.83092	.	.	1
19853	0	4.20	1	0.83092	.	.	1
19854	0	4.20	1	0.83092	.	.	1
19855	0	4.20	1	0.83092	.	.	1
19856	0	4.20	1	0.83092	.	.	1
19857	0	4.20	1	0.83092	.	.	1
19858	0	4.20	1	0.83092	.	.	1
19859	0	4.20	1	0.83092	.	.	1
19860	0	4.20	1	0.83092	.	.	1
19861	0	4.20	1	0.83092	.	.	1
19862	0	4.20	1	0.83092	.	.	1
19863	0	4.20	1	0.83092	.	.	1
19864	0	4.20	1	0.83092	.	.	1
19865	0	4.20	1	0.83092	.	.	1
19866	0	4.20	1	0.83092	.	.	1
19867	0	4.20	1	0.83092	.	.	1
19868	0	4.20	1	0.83092	.	.	1
19869	0	4.20	1	0.83092	.	.	1
19870	0	4.20	1	0.83092	.	.	1
19871	0	4.20	1	0.83092	.	.	1
19872	0	4.20	1	0.83092	.	.	1
19873	0	4.20	1	0.83092	.	.	1
19874	0	4.20	1	0.83092	.	.	1
19875	0	4.20	1	0.83092	.	.	1
19876	0	4.21	0	0.83060	0.82675	0.83437	1
19877	0	4.21	1	0.83060	.	.	1
19878	0	4.21	1	0.83060	.	.	1
19879	0	4.21	1	0.83060	.	.	1
19880	0	4.21	1	0.83060	.	.	1

Obs	Sub_group	fuptimey	_CENSOR_	SURVIVAL	SDF_LCL	SDF_UCL	STRATUM
**19881**	0	4.21	1	0.83060	.	.	1
**19882**	0	4.21	1	0.83060	.	.	1
**19883**	0	4.21	1	0.83060	.	.	1
**19884**	0	4.21	1	0.83060	.	.	1
**19885**	0	4.21	1	0.83060	.	.	1
**19886**	0	4.21	1	0.83060	.	.	1
**19887**	0	4.21	1	0.83060	.	.	1
**19888**	0	4.21	1	0.83060	.	.	1
**19889**	0	4.21	1	0.83060	.	.	1
**19890**	0	4.21	1	0.83060	.	.	1
**19891**	0	4.21	1	0.83060	.	.	1
**19892**	0	4.21	1	0.83060	.	.	1
**19893**	0	4.21	1	0.83060	.	.	1
**19894**	0	4.21	1	0.83060	.	.	1
**19895**	0	4.21	1	0.83060	.	.	1
**19896**	0	4.21	1	0.83060	.	.	1
**19897**	0	4.21	1	0.83060	.	.	1
**19898**	0	4.21	1	0.83060	.	.	1
**19899**	0	4.21	1	0.83060	.	.	1
**19900**	0	4.21	1	0.83060	.	.	1
**19901**	0	4.21	1	0.83060	.	.	1
**19902**	0	4.21	1	0.83060	.	.	1
**19903**	0	4.21	1	0.83060	.	.	1
**19904**	0	4.21	1	0.83060	.	.	1
**19905**	0	4.21	1	0.83060	.	.	1
**19906**	0	4.21	1	0.83060	.	.	1
**19907**	0	4.22	0	0.83028	0.82642	0.83405	1
**19908**	0	4.22	1	0.83028	.	.	1
**19909**	0	4.22	1	0.83028	.	.	1
**19910**	0	4.22	1	0.83028	.	.	1
**19911**	0	4.22	1	0.83028	.	.	1
**19912**	0	4.22	1	0.83028	.	.	1
**19913**	0	4.22	1	0.83028	.	.	1
**19914**	0	4.22	1	0.83028	.	.	1
**19915**	0	4.22	1	0.83028	.	.	1

Obs	Sub_group	fuptimey	_CENSOR_	SURVIVAL	SDF_LCL	SDF_UCL	STRATUM
19916	0	4.22	1	0.83028	.	.	1
19917	0	4.22	1	0.83028	.	.	1
19918	0	4.22	1	0.83028	.	.	1
19919	0	4.22	1	0.83028	.	.	1
19920	0	4.22	1	0.83028	.	.	1
19921	0	4.22	1	0.83028	.	.	1
19922	0	4.22	1	0.83028	.	.	1
19923	0	4.22	1	0.83028	.	.	1
19924	0	4.22	1	0.83028	.	.	1
19925	0	4.22	1	0.83028	.	.	1
19926	0	4.22	1	0.83028	.	.	1
19927	0	4.22	1	0.83028	.	.	1
19928	0	4.22	1	0.83028	.	.	1
19929	0	4.22	1	0.83028	.	.	1
19930	0	4.22	1	0.83028	.	.	1
19931	0	4.22	1	0.83028	.	.	1
19932	0	4.22	1	0.83028	.	.	1
19933	0	4.22	1	0.83028	.	.	1
19934	0	4.22	1	0.83028	.	.	1
19935	0	4.22	1	0.83028	.	.	1
19936	0	4.22	1	0.83028	.	.	1
19937	0	4.22	1	0.83028	.	.	1
19938	0	4.22	1	0.83028	.	.	1
19939	0	4.22	1	0.83028	.	.	1
19940	0	4.22	1	0.83028	.	.	1
19941	0	4.22	1	0.83028	.	.	1
19942	0	4.22	1	0.83028	.	.	1
19943	0	4.22	1	0.83028	.	.	1
19944	0	4.22	1	0.83028	.	.	1
19945	0	4.22	1	0.83028	.	.	1
19946	0	4.23	0	0.83003	0.82617	0.83381	1
19947	0	4.23	1	0.83003	.	.	1
19948	0	4.23	1	0.83003	.	.	1
19949	0	4.23	1	0.83003	.	.	1
19950	0	4.23	1	0.83003	.	.	1

Obs	Sub_group	fuptimey	_CENSOR_	SURVIVAL	SDF_LCL	SDF_UCL	STRATUM
19951	0	4.23	1	0.83003	.	.	1
19952	0	4.23	1	0.83003	.	.	1
19953	0	4.23	1	0.83003	.	.	1
19954	0	4.23	1	0.83003	.	.	1
19955	0	4.23	1	0.83003	.	.	1
19956	0	4.23	1	0.83003	.	.	1
19957	0	4.23	1	0.83003	.	.	1
19958	0	4.23	1	0.83003	.	.	1
19959	0	4.23	1	0.83003	.	.	1
19960	0	4.23	1	0.83003	.	.	1
19961	0	4.23	1	0.83003	.	.	1
19962	0	4.23	1	0.83003	.	.	1
19963	0	4.23	1	0.83003	.	.	1
19964	0	4.23	1	0.83003	.	.	1
19965	0	4.23	1	0.83003	.	.	1
19966	0	4.23	1	0.83003	.	.	1
19967	0	4.23	1	0.83003	.	.	1
19968	0	4.23	1	0.83003	.	.	1
19969	0	4.23	1	0.83003	.	.	1
19970	0	4.23	1	0.83003	.	.	1
19971	0	4.23	1	0.83003	.	.	1
19972	0	4.23	1	0.83003	.	.	1
19973	0	4.23	1	0.83003	.	.	1
19974	0	4.24	0	0.82987	0.82601	0.83365	1
19975	0	4.24	1	0.82987	.	.	1
19976	0	4.24	1	0.82987	.	.	1
19977	0	4.24	1	0.82987	.	.	1
19978	0	4.24	1	0.82987	.	.	1
19979	0	4.24	1	0.82987	.	.	1
19980	0	4.24	1	0.82987	.	.	1
19981	0	4.24	1	0.82987	.	.	1
19982	0	4.24	1	0.82987	.	.	1
19983	0	4.24	1	0.82987	.	.	1
19984	0	4.24	1	0.82987	.	.	1
19985	0	4.24	1	0.82987	.	.	1

Obs	Sub_group	fuptimey	_CENSOR_	SURVIVAL	SDF_LCL	SDF_UCL	STRATUM
19986	0	4.24	1	0.82987	.	.	1
19987	0	4.24	1	0.82987	.	.	1
19988	0	4.24	1	0.82987	.	.	1
19989	0	4.24	1	0.82987	.	.	1
19990	0	4.24	1	0.82987	.	.	1
19991	0	4.24	1	0.82987	.	.	1
19992	0	4.24	1	0.82987	.	.	1
19993	0	4.24	1	0.82987	.	.	1
19994	0	4.24	1	0.82987	.	.	1
19995	0	4.24	1	0.82987	.	.	1
19996	0	4.24	1	0.82987	.	.	1
19997	0	4.24	1	0.82987	.	.	1
19998	0	4.24	1	0.82987	.	.	1
19999	0	4.24	1	0.82987	.	.	1
20000	0	4.24	1	0.82987	.	.	1
20001	0	4.24	1	0.82987	.	.	1
20002	0	4.24	1	0.82987	.	.	1
20003	0	4.24	1	0.82987	.	.	1
20004	0	4.24	1	0.82987	.	.	1
20005	0	4.24	1	0.82987	.	.	1
20006	0	4.24	1	0.82987	.	.	1
20007	0	4.24	1	0.82987	.	.	1
20008	0	4.24	1	0.82987	.	.	1
20009	0	4.24	1	0.82987	.	.	1
20010	0	4.24	1	0.82987	.	.	1
20011	0	4.24	1	0.82987	.	.	1
20012	0	4.24	1	0.82987	.	.	1
20013	0	4.24	1	0.82987	.	.	1
20014	0	4.25	0	0.82971	0.82584	0.83349	1
20015	0	4.25	1	0.82971	.	.	1
20016	0	4.25	1	0.82971	.	.	1
20017	0	4.25	1	0.82971	.	.	1
20018	0	4.25	1	0.82971	.	.	1
20019	0	4.25	1	0.82971	.	.	1
20020	0	4.25	1	0.82971	.	.	1

Obs	Sub_group	fuptimey	_CENSOR_	SURVIVAL	SDF_LCL	SDF_UCL	STRATUM
20021	0	4.25	1	0.82971	.	.	1
20022	0	4.25	1	0.82971	.	.	1
20023	0	4.25	1	0.82971	.	.	1
20024	0	4.25	1	0.82971	.	.	1
20025	0	4.25	1	0.82971	.	.	1
20026	0	4.25	1	0.82971	.	.	1
20027	0	4.25	1	0.82971	.	.	1
20028	0	4.25	1	0.82971	.	.	1
20029	0	4.25	1	0.82971	.	.	1
20030	0	4.25	1	0.82971	.	.	1
20031	0	4.25	1	0.82971	.	.	1
20032	0	4.25	1	0.82971	.	.	1
20033	0	4.25	1	0.82971	.	.	1
20034	0	4.25	1	0.82971	.	.	1
20035	0	4.25	1	0.82971	.	.	1
20036	0	4.25	1	0.82971	.	.	1
20037	0	4.25	1	0.82971	.	.	1
20038	0	4.25	1	0.82971	.	.	1
20039	0	4.25	1	0.82971	.	.	1
20040	0	4.25	1	0.82971	.	.	1
20041	0	4.25	1	0.82971	.	.	1
20042	0	4.25	1	0.82971	.	.	1
20043	0	4.25	1	0.82971	.	.	1
20044	0	4.25	1	0.82971	.	.	1
20045	0	4.25	1	0.82971	.	.	1
20046	0	4.25	1	0.82971	.	.	1
20047	0	4.25	1	0.82971	.	.	1
20048	0	4.25	1	0.82971	.	.	1
20049	0	4.25	1	0.82971	.	.	1
20050	0	4.25	1	0.82971	.	.	1
20051	0	4.25	1	0.82971	.	.	1
20052	0	4.25	1	0.82971	.	.	1
20053	0	4.26	0	0.82954	0.82568	0.83333	1
20054	0	4.26	1	0.82954	.	.	1
20055	0	4.26	1	0.82954	.	.	1

Obs	Sub_group	fuptimey	_CENSOR_	SURVIVAL	SDF_LCL	SDF_UCL	STRATUM
20056	0	4.26	1	0.82954	.	.	1
20057	0	4.26	1	0.82954	.	.	1
20058	0	4.26	1	0.82954	.	.	1
20059	0	4.26	1	0.82954	.	.	1
20060	0	4.26	1	0.82954	.	.	1
20061	0	4.26	1	0.82954	.	.	1
20062	0	4.26	1	0.82954	.	.	1
20063	0	4.26	1	0.82954	.	.	1
20064	0	4.26	1	0.82954	.	.	1
20065	0	4.26	1	0.82954	.	.	1
20066	0	4.26	1	0.82954	.	.	1
20067	0	4.26	1	0.82954	.	.	1
20068	0	4.26	1	0.82954	.	.	1
20069	0	4.26	1	0.82954	.	.	1
20070	0	4.26	1	0.82954	.	.	1
20071	0	4.26	1	0.82954	.	.	1
20072	0	4.26	1	0.82954	.	.	1
20073	0	4.26	1	0.82954	.	.	1
20074	0	4.26	1	0.82954	.	.	1
20075	0	4.26	1	0.82954	.	.	1
20076	0	4.26	1	0.82954	.	.	1
20077	0	4.26	1	0.82954	.	.	1
20078	0	4.26	1	0.82954	.	.	1
20079	0	4.26	1	0.82954	.	.	1
20080	0	4.26	1	0.82954	.	.	1
20081	0	4.26	1	0.82954	.	.	1
20082	0	4.26	1	0.82954	.	.	1
20083	0	4.26	1	0.82954	.	.	1
20084	0	4.26	1	0.82954	.	.	1
20085	0	4.26	1	0.82954	.	.	1
20086	0	4.26	1	0.82954	.	.	1
20087	0	4.26	1	0.82954	.	.	1
20088	0	4.26	1	0.82954	.	.	1
20089	0	4.26	1	0.82954	.	.	1
20090	0	4.26	1	0.82954	.	.	1

Obs	Sub_group	fuptimey	_CENSOR_	SURVIVAL	SDF_LCL	SDF_UCL	STRATUM
**20091**	0	4.26	1	0.82954	.	.	1
**20092**	0	4.26	1	0.82954	.	.	1
**20093**	0	4.26	1	0.82954	.	.	1
**20094**	0	4.27	0	0.82926	0.82539	0.83305	1
**20095**	0	4.27	1	0.82926	.	.	1
**20096**	0	4.27	1	0.82926	.	.	1
**20097**	0	4.27	1	0.82926	.	.	1
**20098**	0	4.27	1	0.82926	.	.	1
**20099**	0	4.27	1	0.82926	.	.	1
**20100**	0	4.27	1	0.82926	.	.	1
**20101**	0	4.27	1	0.82926	.	.	1
**20102**	0	4.27	1	0.82926	.	.	1
**20103**	0	4.27	1	0.82926	.	.	1
**20104**	0	4.27	1	0.82926	.	.	1
**20105**	0	4.27	1	0.82926	.	.	1
**20106**	0	4.27	1	0.82926	.	.	1
**20107**	0	4.27	1	0.82926	.	.	1
**20108**	0	4.27	1	0.82926	.	.	1
**20109**	0	4.27	1	0.82926	.	.	1
**20110**	0	4.27	1	0.82926	.	.	1
**20111**	0	4.27	1	0.82926	.	.	1
**20112**	0	4.27	1	0.82926	.	.	1
**20113**	0	4.27	1	0.82926	.	.	1
**20114**	0	4.27	1	0.82926	.	.	1
**20115**	0	4.27	1	0.82926	.	.	1
**20116**	0	4.27	1	0.82926	.	.	1
**20117**	0	4.27	1	0.82926	.	.	1
**20118**	0	4.27	1	0.82926	.	.	1
**20119**	0	4.27	1	0.82926	.	.	1
**20120**	0	4.28	0	0.82905	0.82518	0.83285	1
**20121**	0	4.28	1	0.82905	.	.	1
**20122**	0	4.28	1	0.82905	.	.	1
**20123**	0	4.28	1	0.82905	.	.	1
**20124**	0	4.28	1	0.82905	.	.	1
**20125**	0	4.28	1	0.82905	.	.	1

Obs	Sub_group	fuptimey	_CENSOR_	SURVIVAL	SDF_LCL	SDF_UCL	STRATUM
20126	0	4.28	1	0.82905	.	.	1
20127	0	4.28	1	0.82905	.	.	1
20128	0	4.28	1	0.82905	.	.	1
20129	0	4.28	1	0.82905	.	.	1
20130	0	4.28	1	0.82905	.	.	1
20131	0	4.28	1	0.82905	.	.	1
20132	0	4.28	1	0.82905	.	.	1
20133	0	4.28	1	0.82905	.	.	1
20134	0	4.28	1	0.82905	.	.	1
20135	0	4.28	1	0.82905	.	.	1
20136	0	4.28	1	0.82905	.	.	1
20137	0	4.28	1	0.82905	.	.	1
20138	0	4.28	1	0.82905	.	.	1
20139	0	4.28	1	0.82905	.	.	1
20140	0	4.28	1	0.82905	.	.	1
20141	0	4.28	1	0.82905	.	.	1
20142	0	4.28	1	0.82905	.	.	1
20143	0	4.28	1	0.82905	.	.	1
20144	0	4.28	1	0.82905	.	.	1
20145	0	4.28	1	0.82905	.	.	1
20146	0	4.28	1	0.82905	.	.	1
20147	0	4.28	1	0.82905	.	.	1
20148	0	4.28	1	0.82905	.	.	1
20149	0	4.28	1	0.82905	.	.	1
20150	0	4.28	1	0.82905	.	.	1
20151	0	4.28	1	0.82905	.	.	1
20152	0	4.28	1	0.82905	.	.	1
20153	0	4.28	1	0.82905	.	.	1
20154	0	4.28	1	0.82905	.	.	1
20155	0	4.28	1	0.82905	.	.	1
20156	0	4.28	1	0.82905	.	.	1
20157	0	4.28	1	0.82905	.	.	1
20158	0	4.28	1	0.82905	.	.	1
20159	0	4.28	1	0.82905	.	.	1
20160	0	4.29	0	0.82873	0.82485	0.83253	1

Obs	Sub_group	fuptimey	_CENSOR_	SURVIVAL	SDF_LCL	SDF_UCL	STRATUM
20161	0	4.29	1	0.82873	.	.	1
20162	0	4.29	1	0.82873	.	.	1
20163	0	4.29	1	0.82873	.	.	1
20164	0	4.29	1	0.82873	.	.	1
20165	0	4.29	1	0.82873	.	.	1
20166	0	4.29	1	0.82873	.	.	1
20167	0	4.29	1	0.82873	.	.	1
20168	0	4.29	1	0.82873	.	.	1
20169	0	4.29	1	0.82873	.	.	1
20170	0	4.29	1	0.82873	.	.	1
20171	0	4.29	1	0.82873	.	.	1
20172	0	4.29	1	0.82873	.	.	1
20173	0	4.29	1	0.82873	.	.	1
20174	0	4.29	1	0.82873	.	.	1
20175	0	4.29	1	0.82873	.	.	1
20176	0	4.29	1	0.82873	.	.	1
20177	0	4.29	1	0.82873	.	.	1
20178	0	4.29	1	0.82873	.	.	1
20179	0	4.29	1	0.82873	.	.	1
20180	0	4.29	1	0.82873	.	.	1
20181	0	4.29	1	0.82873	.	.	1
20182	0	4.29	1	0.82873	.	.	1
20183	0	4.29	1	0.82873	.	.	1
20184	0	4.29	1	0.82873	.	.	1
20185	0	4.29	1	0.82873	.	.	1
20186	0	4.29	1	0.82873	.	.	1
20187	0	4.29	1	0.82873	.	.	1
20188	0	4.30	0	0.82844	0.82455	0.83225	1
20189	0	4.30	1	0.82844	.	.	1
20190	0	4.30	1	0.82844	.	.	1
20191	0	4.30	1	0.82844	.	.	1
20192	0	4.30	1	0.82844	.	.	1
20193	0	4.30	1	0.82844	.	.	1
20194	0	4.30	1	0.82844	.	.	1
20195	0	4.30	1	0.82844	.	.	1

Obs	Sub_group	fuptimey	_CENSOR_	SURVIVAL	SDF_LCL	SDF_UCL	STRATUM
20196	0	4.30	1	0.82844	.	.	1
20197	0	4.30	1	0.82844	.	.	1
20198	0	4.30	1	0.82844	.	.	1
20199	0	4.30	1	0.82844	.	.	1
20200	0	4.30	1	0.82844	.	.	1
20201	0	4.30	1	0.82844	.	.	1
20202	0	4.30	1	0.82844	.	.	1
20203	0	4.30	1	0.82844	.	.	1
20204	0	4.30	1	0.82844	.	.	1
20205	0	4.30	1	0.82844	.	.	1
20206	0	4.30	1	0.82844	.	.	1
20207	0	4.30	1	0.82844	.	.	1
20208	0	4.30	1	0.82844	.	.	1
20209	0	4.30	1	0.82844	.	.	1
20210	0	4.30	1	0.82844	.	.	1
20211	0	4.30	1	0.82844	.	.	1
20212	0	4.30	1	0.82844	.	.	1
20213	0	4.30	1	0.82844	.	.	1
20214	0	4.30	1	0.82844	.	.	1
20215	0	4.30	1	0.82844	.	.	1
20216	0	4.30	1	0.82844	.	.	1
20217	0	4.30	1	0.82844	.	.	1
20218	0	4.30	1	0.82844	.	.	1
20219	0	4.30	1	0.82844	.	.	1
20220	0	4.30	1	0.82844	.	.	1
20221	0	4.30	1	0.82844	.	.	1
20222	0	4.30	1	0.82844	.	.	1
20223	0	4.30	1	0.82844	.	.	1
20224	0	4.30	1	0.82844	.	.	1
20225	0	4.30	1	0.82844	.	.	1
20226	0	4.30	1	0.82844	.	.	1
20227	0	4.30	1	0.82844	.	.	1
20228	0	4.30	1	0.82844	.	.	1
20229	0	4.30	1	0.82844	.	.	1
20230	0	4.30	1	0.82844	.	.	1

Obs	Sub_group	fuptimey	_CENSOR_	SURVIVAL	SDF_LCL	SDF_UCL	STRATUM
20231	0	4.30	1	0.82844	.	.	1
20232	0	4.31	0	0.82831	0.82443	0.83212	1
20233	0	4.31	1	0.82831	.	.	1
20234	0	4.31	1	0.82831	.	.	1
20235	0	4.31	1	0.82831	.	.	1
20236	0	4.31	1	0.82831	.	.	1
20237	0	4.31	1	0.82831	.	.	1
20238	0	4.31	1	0.82831	.	.	1
20239	0	4.31	1	0.82831	.	.	1
20240	0	4.31	1	0.82831	.	.	1
20241	0	4.31	1	0.82831	.	.	1
20242	0	4.31	1	0.82831	.	.	1
20243	0	4.31	1	0.82831	.	.	1
20244	0	4.31	1	0.82831	.	.	1
20245	0	4.31	1	0.82831	.	.	1
20246	0	4.31	1	0.82831	.	.	1
20247	0	4.31	1	0.82831	.	.	1
20248	0	4.31	1	0.82831	.	.	1
20249	0	4.31	1	0.82831	.	.	1
20250	0	4.31	1	0.82831	.	.	1
20251	0	4.31	1	0.82831	.	.	1
20252	0	4.31	1	0.82831	.	.	1
20253	0	4.31	1	0.82831	.	.	1
20254	0	4.31	1	0.82831	.	.	1
20255	0	4.31	1	0.82831	.	.	1
20256	0	4.31	1	0.82831	.	.	1
20257	0	4.31	1	0.82831	.	.	1
20258	0	4.31	1	0.82831	.	.	1
20259	0	4.31	1	0.82831	.	.	1
20260	0	4.31	1	0.82831	.	.	1
20261	0	4.31	1	0.82831	.	.	1
20262	0	4.31	1	0.82831	.	.	1
20263	0	4.31	1	0.82831	.	.	1
20264	0	4.31	1	0.82831	.	.	1
20265	0	4.31	1	0.82831	.	.	1

Obs	Sub_group	fuptimey	_CENSOR_	SURVIVAL	SDF_LCL	SDF_UCL	STRATUM
20266	0	4.31	1	0.82831	.	.	1
20267	0	4.31	1	0.82831	.	.	1
20268	0	4.31	1	0.82831	.	.	1
20269	0	4.31	1	0.82831	.	.	1
20270	0	4.31	1	0.82831	.	.	1
20271	0	4.31	1	0.82831	.	.	1
20272	0	4.31	1	0.82831	.	.	1
20273	0	4.32	0	0.82823	0.82434	0.83204	1
20274	0	4.32	1	0.82823	.	.	1
20275	0	4.32	1	0.82823	.	.	1
20276	0	4.32	1	0.82823	.	.	1
20277	0	4.32	1	0.82823	.	.	1
20278	0	4.32	1	0.82823	.	.	1
20279	0	4.32	1	0.82823	.	.	1
20280	0	4.32	1	0.82823	.	.	1
20281	0	4.32	1	0.82823	.	.	1
20282	0	4.32	1	0.82823	.	.	1
20283	0	4.32	1	0.82823	.	.	1
20284	0	4.32	1	0.82823	.	.	1
20285	0	4.32	1	0.82823	.	.	1
20286	0	4.32	1	0.82823	.	.	1
20287	0	4.32	1	0.82823	.	.	1
20288	0	4.32	1	0.82823	.	.	1
20289	0	4.32	1	0.82823	.	.	1
20290	0	4.32	1	0.82823	.	.	1
20291	0	4.32	1	0.82823	.	.	1
20292	0	4.32	1	0.82823	.	.	1
20293	0	4.32	1	0.82823	.	.	1
20294	0	4.32	1	0.82823	.	.	1
20295	0	4.32	1	0.82823	.	.	1
20296	0	4.32	1	0.82823	.	.	1
20297	0	4.32	1	0.82823	.	.	1
20298	0	4.32	1	0.82823	.	.	1
20299	0	4.32	1	0.82823	.	.	1
20300	0	4.32	1	0.82823	.	.	1

Obs	Sub_group	fuptimey	_CENSOR_	SURVIVAL	SDF_LCL	SDF_UCL	STRATUM
20301	0	4.32	1	0.82823	.	.	1
20302	0	4.32	1	0.82823	.	.	1
20303	0	4.32	1	0.82823	.	.	1
20304	0	4.32	1	0.82823	.	.	1
20305	0	4.32	1	0.82823	.	.	1
20306	0	4.32	1	0.82823	.	.	1
20307	0	4.32	1	0.82823	.	.	1
20308	0	4.32	1	0.82823	.	.	1
20309	0	4.32	1	0.82823	.	.	1
20310	0	4.32	1	0.82823	.	.	1
20311	0	4.32	1	0.82823	.	.	1
20312	0	4.32	1	0.82823	.	.	1
20313	0	4.32	1	0.82823	.	.	1
20314	0	4.32	1	0.82823	.	.	1
20315	0	4.32	1	0.82823	.	.	1
20316	0	4.33	0	0.82794	0.82405	0.83176	1
20317	0	4.33	1	0.82794	.	.	1
20318	0	4.33	1	0.82794	.	.	1
20319	0	4.33	1	0.82794	.	.	1
20320	0	4.33	1	0.82794	.	.	1
20321	0	4.33	1	0.82794	.	.	1
20322	0	4.33	1	0.82794	.	.	1
20323	0	4.33	1	0.82794	.	.	1
20324	0	4.33	1	0.82794	.	.	1
20325	0	4.33	1	0.82794	.	.	1
20326	0	4.33	1	0.82794	.	.	1
20327	0	4.33	1	0.82794	.	.	1
20328	0	4.33	1	0.82794	.	.	1
20329	0	4.33	1	0.82794	.	.	1
20330	0	4.33	1	0.82794	.	.	1
20331	0	4.33	1	0.82794	.	.	1
20332	0	4.33	1	0.82794	.	.	1
20333	0	4.33	1	0.82794	.	.	1
20334	0	4.33	1	0.82794	.	.	1
20335	0	4.33	1	0.82794	.	.	1

Obs	Sub_group	fuptimey	_CENSOR_	SURVIVAL	SDF_LCL	SDF_UCL	STRATUM
20336	0	4.33	1	0.82794	.	.	1
20337	0	4.33	1	0.82794	.	.	1
20338	0	4.33	1	0.82794	.	.	1
20339	0	4.33	1	0.82794	.	.	1
20340	0	4.34	0	0.82782	0.82392	0.83164	1
20341	0	4.34	1	0.82782	.	.	1
20342	0	4.34	1	0.82782	.	.	1
20343	0	4.34	1	0.82782	.	.	1
20344	0	4.34	1	0.82782	.	.	1
20345	0	4.34	1	0.82782	.	.	1
20346	0	4.34	1	0.82782	.	.	1
20347	0	4.34	1	0.82782	.	.	1
20348	0	4.34	1	0.82782	.	.	1
20349	0	4.34	1	0.82782	.	.	1
20350	0	4.34	1	0.82782	.	.	1
20351	0	4.34	1	0.82782	.	.	1
20352	0	4.34	1	0.82782	.	.	1
20353	0	4.34	1	0.82782	.	.	1
20354	0	4.34	1	0.82782	.	.	1
20355	0	4.34	1	0.82782	.	.	1
20356	0	4.34	1	0.82782	.	.	1
20357	0	4.34	1	0.82782	.	.	1
20358	0	4.34	1	0.82782	.	.	1
20359	0	4.34	1	0.82782	.	.	1
20360	0	4.34	1	0.82782	.	.	1
20361	0	4.34	1	0.82782	.	.	1
20362	0	4.34	1	0.82782	.	.	1
20363	0	4.34	1	0.82782	.	.	1
20364	0	4.34	1	0.82782	.	.	1
20365	0	4.34	1	0.82782	.	.	1
20366	0	4.34	1	0.82782	.	.	1
20367	0	4.34	1	0.82782	.	.	1
20368	0	4.34	1	0.82782	.	.	1
20369	0	4.34	1	0.82782	.	.	1
20370	0	4.34	1	0.82782	.	.	1

Obs	Sub_group	fuptimey	_CENSOR_	SURVIVAL	SDF_LCL	SDF_UCL	STRATUM
**20371**	0	4.34	1	0.82782	.	.	1
**20372**	0	4.34	1	0.82782	.	.	1
**20373**	0	4.34	1	0.82782	.	.	1
**20374**	0	4.34	1	0.82782	.	.	1
**20375**	0	4.34	1	0.82782	.	.	1
**20376**	0	4.34	1	0.82782	.	.	1
**20377**	0	4.34	1	0.82782	.	.	1
**20378**	0	4.34	1	0.82782	.	.	1
**20379**	0	4.34	1	0.82782	.	.	1
**20380**	0	4.34	1	0.82782	.	.	1
**20381**	0	4.34	1	0.82782	.	.	1
**20382**	0	4.34	1	0.82782	.	.	1
**20383**	0	4.35	0	0.82774	0.82384	0.83155	1
**20384**	0	4.35	1	0.82774	.	.	1
**20385**	0	4.35	1	0.82774	.	.	1
**20386**	0	4.35	1	0.82774	.	.	1
**20387**	0	4.35	1	0.82774	.	.	1
**20388**	0	4.35	1	0.82774	.	.	1
**20389**	0	4.35	1	0.82774	.	.	1
**20390**	0	4.35	1	0.82774	.	.	1
**20391**	0	4.35	1	0.82774	.	.	1
**20392**	0	4.35	1	0.82774	.	.	1
**20393**	0	4.35	1	0.82774	.	.	1
**20394**	0	4.35	1	0.82774	.	.	1
**20395**	0	4.35	1	0.82774	.	.	1
**20396**	0	4.35	1	0.82774	.	.	1
**20397**	0	4.35	1	0.82774	.	.	1
**20398**	0	4.35	1	0.82774	.	.	1
**20399**	0	4.35	1	0.82774	.	.	1
**20400**	0	4.35	1	0.82774	.	.	1
**20401**	0	4.35	1	0.82774	.	.	1
**20402**	0	4.35	1	0.82774	.	.	1
**20403**	0	4.35	1	0.82774	.	.	1
**20404**	0	4.35	1	0.82774	.	.	1
**20405**	0	4.35	1	0.82774	.	.	1

Obs	Sub_group	fuptimey	_CENSOR_	SURVIVAL	SDF_LCL	SDF_UCL	STRATUM
20406	0	4.35	1	0.82774	.	.	1
20407	0	4.35	1	0.82774	.	.	1
20408	0	4.35	1	0.82774	.	.	1
20409	0	4.35	1	0.82774	.	.	1
20410	0	4.35	1	0.82774	.	.	1
20411	0	4.35	1	0.82774	.	.	1
20412	0	4.35	1	0.82774	.	.	1
20413	0	4.35	1	0.82774	.	.	1
20414	0	4.35	1	0.82774	.	.	1
20415	0	4.35	1	0.82774	.	.	1
20416	0	4.35	1	0.82774	.	.	1
20417	0	4.35	1	0.82774	.	.	1
20418	0	4.35	1	0.82774	.	.	1
20419	0	4.36	0	0.82749	0.82359	0.83131	1
20420	0	4.36	1	0.82749	.	.	1
20421	0	4.36	1	0.82749	.	.	1
20422	0	4.36	1	0.82749	.	.	1
20423	0	4.36	1	0.82749	.	.	1
20424	0	4.36	1	0.82749	.	.	1
20425	0	4.36	1	0.82749	.	.	1
20426	0	4.36	1	0.82749	.	.	1
20427	0	4.36	1	0.82749	.	.	1
20428	0	4.36	1	0.82749	.	.	1
20429	0	4.36	1	0.82749	.	.	1
20430	0	4.36	1	0.82749	.	.	1
20431	0	4.36	1	0.82749	.	.	1
20432	0	4.36	1	0.82749	.	.	1
20433	0	4.36	1	0.82749	.	.	1
20434	0	4.36	1	0.82749	.	.	1
20435	0	4.36	1	0.82749	.	.	1
20436	0	4.36	1	0.82749	.	.	1
20437	0	4.36	1	0.82749	.	.	1
20438	0	4.36	1	0.82749	.	.	1
20439	0	4.36	1	0.82749	.	.	1
20440	0	4.36	1	0.82749	.	.	1

Obs	Sub_group	fuptimey	_CENSOR_	SURVIVAL	SDF_LCL	SDF_UCL	STRATUM
20441	0	4.36	1	0.82749	.	.	1
20442	0	4.36	1	0.82749	.	.	1
20443	0	4.36	1	0.82749	.	.	1
20444	0	4.36	1	0.82749	.	.	1
20445	0	4.36	1	0.82749	.	.	1
20446	0	4.36	1	0.82749	.	.	1
20447	0	4.36	1	0.82749	.	.	1
20448	0	4.36	1	0.82749	.	.	1
20449	0	4.36	1	0.82749	.	.	1
20450	0	4.36	1	0.82749	.	.	1
20451	0	4.36	1	0.82749	.	.	1
20452	0	4.36	1	0.82749	.	.	1
20453	0	4.36	1	0.82749	.	.	1
20454	0	4.36	1	0.82749	.	.	1
20455	0	4.36	1	0.82749	.	.	1
20456	0	4.36	1	0.82749	.	.	1
20457	0	4.36	1	0.82749	.	.	1
20458	0	4.36	1	0.82749	.	.	1
20459	0	4.36	1	0.82749	.	.	1
20460	0	4.36	1	0.82749	.	.	1
20461	0	4.37	0	0.82740	0.82350	0.83123	1
20462	0	4.37	1	0.82740	.	.	1
20463	0	4.37	1	0.82740	.	.	1
20464	0	4.37	1	0.82740	.	.	1
20465	0	4.37	1	0.82740	.	.	1
20466	0	4.37	1	0.82740	.	.	1
20467	0	4.37	1	0.82740	.	.	1
20468	0	4.37	1	0.82740	.	.	1
20469	0	4.37	1	0.82740	.	.	1
20470	0	4.37	1	0.82740	.	.	1
20471	0	4.37	1	0.82740	.	.	1
20472	0	4.37	1	0.82740	.	.	1
20473	0	4.37	1	0.82740	.	.	1
20474	0	4.37	1	0.82740	.	.	1
20475	0	4.37	1	0.82740	.	.	1

Obs	Sub_group	fuptimey	_CENSOR_	SURVIVAL	SDF_LCL	SDF_UCL	STRATUM
20476	0	4.37	1	0.82740	.	.	1
20477	0	4.37	1	0.82740	.	.	1
20478	0	4.37	1	0.82740	.	.	1
20479	0	4.37	1	0.82740	.	.	1
20480	0	4.37	1	0.82740	.	.	1
20481	0	4.37	1	0.82740	.	.	1
20482	0	4.37	1	0.82740	.	.	1
20483	0	4.37	1	0.82740	.	.	1
20484	0	4.37	1	0.82740	.	.	1
20485	0	4.37	1	0.82740	.	.	1
20486	0	4.37	1	0.82740	.	.	1
20487	0	4.37	1	0.82740	.	.	1
20488	0	4.37	1	0.82740	.	.	1
20489	0	4.37	1	0.82740	.	.	1
20490	0	4.37	1	0.82740	.	.	1
20491	0	4.37	1	0.82740	.	.	1
20492	0	4.37	1	0.82740	.	.	1
20493	0	4.37	1	0.82740	.	.	1
20494	0	4.38	0	0.82724	0.82333	0.83106	1
20495	0	4.38	1	0.82724	.	.	1
20496	0	4.38	1	0.82724	.	.	1
20497	0	4.38	1	0.82724	.	.	1
20498	0	4.38	1	0.82724	.	.	1
20499	0	4.38	1	0.82724	.	.	1
20500	0	4.38	1	0.82724	.	.	1
20501	0	4.38	1	0.82724	.	.	1
20502	0	4.38	1	0.82724	.	.	1
20503	0	4.38	1	0.82724	.	.	1
20504	0	4.38	1	0.82724	.	.	1
20505	0	4.38	1	0.82724	.	.	1
20506	0	4.38	1	0.82724	.	.	1
20507	0	4.38	1	0.82724	.	.	1
20508	0	4.38	1	0.82724	.	.	1
20509	0	4.38	1	0.82724	.	.	1
20510	0	4.38	1	0.82724	.	.	1

Obs	Sub_group	fuptimey	_CENSOR_	SURVIVAL	SDF_LCL	SDF_UCL	STRATUM
20511	0	4.38	1	0.82724	.	.	1
20512	0	4.38	1	0.82724	.	.	1
20513	0	4.38	1	0.82724	.	.	1
20514	0	4.38	1	0.82724	.	.	1
20515	0	4.38	1	0.82724	.	.	1
20516	0	4.38	1	0.82724	.	.	1
20517	0	4.38	1	0.82724	.	.	1
20518	0	4.38	1	0.82724	.	.	1
20519	0	4.38	1	0.82724	.	.	1
20520	0	4.38	1	0.82724	.	.	1
20521	0	4.38	1	0.82724	.	.	1
20522	0	4.38	1	0.82724	.	.	1
20523	0	4.38	1	0.82724	.	.	1
20524	0	4.38	1	0.82724	.	.	1
20525	0	4.38	1	0.82724	.	.	1
20526	0	4.38	1	0.82724	.	.	1
20527	0	4.38	1	0.82724	.	.	1
20528	0	4.38	1	0.82724	.	.	1
20529	0	4.38	1	0.82724	.	.	1
20530	0	4.38	1	0.82724	.	.	1
20531	0	4.38	1	0.82724	.	.	1
20532	0	4.38	1	0.82724	.	.	1
20533	0	4.38	1	0.82724	.	.	1
20534	0	4.38	1	0.82724	.	.	1
20535	0	4.38	1	0.82724	.	.	1
20536	0	4.38	1	0.82724	.	.	1
20537	0	4.38	1	0.82724	.	.	1
20538	0	4.38	1	0.82724	.	.	1
20539	0	4.38	1	0.82724	.	.	1
20540	0	4.38	1	0.82724	.	.	1
20541	0	4.38	1	0.82724	.	.	1
20542	0	4.38	1	0.82724	.	.	1
20543	0	4.38	1	0.82724	.	.	1
20544	0	4.38	1	0.82724	.	.	1
20545	0	4.38	1	0.82724	.	.	1

Obs	Sub_group	fuptimey	_CENSOR_	SURVIVAL	SDF_LCL	SDF_UCL	STRATUM
20546	0	4.38	1	0.82724	.	.	1
20547	0	4.38	1	0.82724	.	.	1
20548	0	4.38	1	0.82724	.	.	1
20549	0	4.38	1	0.82724	.	.	1
20550	0	4.38	1	0.82724	.	.	1
20551	0	4.38	1	0.82724	.	.	1
20552	0	4.39	0	0.82711	0.82321	0.83094	1
20553	0	4.39	1	0.82711	.	.	1
20554	0	4.39	1	0.82711	.	.	1
20555	0	4.39	1	0.82711	.	.	1
20556	0	4.39	1	0.82711	.	.	1
20557	0	4.39	1	0.82711	.	.	1
20558	0	4.39	1	0.82711	.	.	1
20559	0	4.39	1	0.82711	.	.	1
20560	0	4.39	1	0.82711	.	.	1
20561	0	4.39	1	0.82711	.	.	1
20562	0	4.39	1	0.82711	.	.	1
20563	0	4.39	1	0.82711	.	.	1
20564	0	4.39	1	0.82711	.	.	1
20565	0	4.39	1	0.82711	.	.	1
20566	0	4.39	1	0.82711	.	.	1
20567	0	4.39	1	0.82711	.	.	1
20568	0	4.39	1	0.82711	.	.	1
20569	0	4.39	1	0.82711	.	.	1
20570	0	4.39	1	0.82711	.	.	1
20571	0	4.39	1	0.82711	.	.	1
20572	0	4.39	1	0.82711	.	.	1
20573	0	4.39	1	0.82711	.	.	1
20574	0	4.39	1	0.82711	.	.	1
20575	0	4.39	1	0.82711	.	.	1
20576	0	4.39	1	0.82711	.	.	1
20577	0	4.40	0	0.82686	0.82295	0.83069	1
20578	0	4.40	1	0.82686	.	.	1
20579	0	4.40	1	0.82686	.	.	1
20580	0	4.40	1	0.82686	.	.	1

Obs	Sub_group	fuptimey	_CENSOR_	SURVIVAL	SDF_LCL	SDF_UCL	STRATUM
20581	0	4.40	1	0.82686	.	.	1
20582	0	4.40	1	0.82686	.	.	1
20583	0	4.40	1	0.82686	.	.	1
20584	0	4.40	1	0.82686	.	.	1
20585	0	4.40	1	0.82686	.	.	1
20586	0	4.40	1	0.82686	.	.	1
20587	0	4.40	1	0.82686	.	.	1
20588	0	4.40	1	0.82686	.	.	1
20589	0	4.40	1	0.82686	.	.	1
20590	0	4.40	1	0.82686	.	.	1
20591	0	4.40	1	0.82686	.	.	1
20592	0	4.40	1	0.82686	.	.	1
20593	0	4.40	1	0.82686	.	.	1
20594	0	4.40	1	0.82686	.	.	1
20595	0	4.40	1	0.82686	.	.	1
20596	0	4.40	1	0.82686	.	.	1
20597	0	4.40	1	0.82686	.	.	1
20598	0	4.40	1	0.82686	.	.	1
20599	0	4.40	1	0.82686	.	.	1
20600	0	4.40	1	0.82686	.	.	1
20601	0	4.40	1	0.82686	.	.	1
20602	0	4.40	1	0.82686	.	.	1
20603	0	4.40	1	0.82686	.	.	1
20604	0	4.40	1	0.82686	.	.	1
20605	0	4.40	1	0.82686	.	.	1
20606	0	4.40	1	0.82686	.	.	1
20607	0	4.40	1	0.82686	.	.	1
20608	0	4.40	1	0.82686	.	.	1
20609	0	4.40	1	0.82686	.	.	1
20610	0	4.40	1	0.82686	.	.	1
20611	0	4.40	1	0.82686	.	.	1
20612	0	4.40	1	0.82686	.	.	1
20613	0	4.40	1	0.82686	.	.	1
20614	0	4.40	1	0.82686	.	.	1
20615	0	4.40	1	0.82686	.	.	1

Obs	Sub_group	fuptimey	_CENSOR_	SURVIVAL	SDF_LCL	SDF_UCL	STRATUM
20616	0	4.40	1	0.82686	.	.	1
20617	0	4.40	1	0.82686	.	.	1
20618	0	4.40	1	0.82686	.	.	1
20619	0	4.40	1	0.82686	.	.	1
20620	0	4.40	1	0.82686	.	.	1
20621	0	4.41	0	0.82669	0.82278	0.83053	1
20622	0	4.41	1	0.82669	.	.	1
20623	0	4.41	1	0.82669	.	.	1
20624	0	4.41	1	0.82669	.	.	1
20625	0	4.41	1	0.82669	.	.	1
20626	0	4.41	1	0.82669	.	.	1
20627	0	4.41	1	0.82669	.	.	1
20628	0	4.41	1	0.82669	.	.	1
20629	0	4.41	1	0.82669	.	.	1
20630	0	4.41	1	0.82669	.	.	1
20631	0	4.41	1	0.82669	.	.	1
20632	0	4.41	1	0.82669	.	.	1
20633	0	4.41	1	0.82669	.	.	1
20634	0	4.41	1	0.82669	.	.	1
20635	0	4.41	1	0.82669	.	.	1
20636	0	4.41	1	0.82669	.	.	1
20637	0	4.41	1	0.82669	.	.	1
20638	0	4.41	1	0.82669	.	.	1
20639	0	4.41	1	0.82669	.	.	1
20640	0	4.41	1	0.82669	.	.	1
20641	0	4.41	1	0.82669	.	.	1
20642	0	4.41	1	0.82669	.	.	1
20643	0	4.41	1	0.82669	.	.	1
20644	0	4.41	1	0.82669	.	.	1
20645	0	4.41	1	0.82669	.	.	1
20646	0	4.41	1	0.82669	.	.	1
20647	0	4.41	1	0.82669	.	.	1
20648	0	4.41	1	0.82669	.	.	1
20649	0	4.41	1	0.82669	.	.	1
20650	0	4.41	1	0.82669	.	.	1

Obs	Sub_group	fuptimey	_CENSOR_	SURVIVAL	SDF_LCL	SDF_UCL	STRATUM
20651	0	4.41	1	0.82669	.	.	1
20652	0	4.41	1	0.82669	.	.	1
20653	0	4.41	1	0.82669	.	.	1
20654	0	4.41	1	0.82669	.	.	1
20655	0	4.41	1	0.82669	.	.	1
20656	0	4.41	1	0.82669	.	.	1
20657	0	4.41	1	0.82669	.	.	1
20658	0	4.41	1	0.82669	.	.	1
20659	0	4.41	1	0.82669	.	.	1
20660	0	4.41	1	0.82669	.	.	1
20661	0	4.41	1	0.82669	.	.	1
20662	0	4.41	1	0.82669	.	.	1
20663	0	4.41	1	0.82669	.	.	1
20664	0	4.41	1	0.82669	.	.	1
20665	0	4.42	0	0.82636	0.82244	0.83020	1
20666	0	4.42	1	0.82636	.	.	1
20667	0	4.42	1	0.82636	.	.	1
20668	0	4.42	1	0.82636	.	.	1
20669	0	4.42	1	0.82636	.	.	1
20670	0	4.42	1	0.82636	.	.	1
20671	0	4.42	1	0.82636	.	.	1
20672	0	4.42	1	0.82636	.	.	1
20673	0	4.42	1	0.82636	.	.	1
20674	0	4.42	1	0.82636	.	.	1
20675	0	4.42	1	0.82636	.	.	1
20676	0	4.42	1	0.82636	.	.	1
20677	0	4.42	1	0.82636	.	.	1
20678	0	4.42	1	0.82636	.	.	1
20679	0	4.42	1	0.82636	.	.	1
20680	0	4.42	1	0.82636	.	.	1
20681	0	4.42	1	0.82636	.	.	1
20682	0	4.42	1	0.82636	.	.	1
20683	0	4.42	1	0.82636	.	.	1
20684	0	4.42	1	0.82636	.	.	1
20685	0	4.42	1	0.82636	.	.	1

Obs	Sub_group	fuptimey	_CENSOR_	SURVIVAL	SDF_LCL	SDF_UCL	STRATUM
20686	0	4.42	1	0.82636	.	.	1
20687	0	4.42	1	0.82636	.	.	1
20688	0	4.42	1	0.82636	.	.	1
20689	0	4.42	1	0.82636	.	.	1
20690	0	4.42	1	0.82636	.	.	1
20691	0	4.42	1	0.82636	.	.	1
20692	0	4.42	1	0.82636	.	.	1
20693	0	4.42	1	0.82636	.	.	1
20694	0	4.42	1	0.82636	.	.	1
20695	0	4.42	1	0.82636	.	.	1
20696	0	4.42	1	0.82636	.	.	1
20697	0	4.42	1	0.82636	.	.	1
20698	0	4.42	1	0.82636	.	.	1
20699	0	4.42	1	0.82636	.	.	1
20700	0	4.42	1	0.82636	.	.	1
20701	0	4.42	1	0.82636	.	.	1
20702	0	4.43	0	0.82598	0.82205	0.82982	1
20703	0	4.43	1	0.82598	.	.	1
20704	0	4.43	1	0.82598	.	.	1
20705	0	4.43	1	0.82598	.	.	1
20706	0	4.43	1	0.82598	.	.	1
20707	0	4.43	1	0.82598	.	.	1
20708	0	4.43	1	0.82598	.	.	1
20709	0	4.43	1	0.82598	.	.	1
20710	0	4.43	1	0.82598	.	.	1
20711	0	4.43	1	0.82598	.	.	1
20712	0	4.43	1	0.82598	.	.	1
20713	0	4.43	1	0.82598	.	.	1
20714	0	4.43	1	0.82598	.	.	1
20715	0	4.43	1	0.82598	.	.	1
20716	0	4.43	1	0.82598	.	.	1
20717	0	4.43	1	0.82598	.	.	1
20718	0	4.43	1	0.82598	.	.	1
20719	0	4.43	1	0.82598	.	.	1
20720	0	4.43	1	0.82598	.	.	1

Obs	Sub_group	fuptimey	_CENSOR_	SURVIVAL	SDF_LCL	SDF_UCL	STRATUM
20721	0	4.43	1	0.82598	.	.	1
20722	0	4.43	1	0.82598	.	.	1
20723	0	4.43	1	0.82598	.	.	1
20724	0	4.43	1	0.82598	.	.	1
20725	0	4.43	1	0.82598	.	.	1
20726	0	4.43	1	0.82598	.	.	1
20727	0	4.43	1	0.82598	.	.	1
20728	0	4.43	1	0.82598	.	.	1
20729	0	4.43	1	0.82598	.	.	1
20730	0	4.43	1	0.82598	.	.	1
20731	0	4.43	1	0.82598	.	.	1
20732	0	4.43	1	0.82598	.	.	1
20733	0	4.43	1	0.82598	.	.	1
20734	0	4.43	1	0.82598	.	.	1
20735	0	4.43	1	0.82598	.	.	1
20736	0	4.43	1	0.82598	.	.	1
20737	0	4.43	1	0.82598	.	.	1
20738	0	4.43	1	0.82598	.	.	1
20739	0	4.43	1	0.82598	.	.	1
20740	0	4.43	1	0.82598	.	.	1
20741	0	4.43	1	0.82598	.	.	1
20742	0	4.43	1	0.82598	.	.	1
20743	0	4.43	1	0.82598	.	.	1
20744	0	4.43	1	0.82598	.	.	1
20745	0	4.43	1	0.82598	.	.	1
20746	0	4.43	1	0.82598	.	.	1
20747	0	4.44	0	0.82556	0.82163	0.82941	1
20748	0	4.44	1	0.82556	.	.	1
20749	0	4.44	1	0.82556	.	.	1
20750	0	4.44	1	0.82556	.	.	1
20751	0	4.44	1	0.82556	.	.	1
20752	0	4.44	1	0.82556	.	.	1
20753	0	4.44	1	0.82556	.	.	1
20754	0	4.44	1	0.82556	.	.	1
20755	0	4.44	1	0.82556	.	.	1

Obs	Sub_group	fuptimey	_CENSOR_	SURVIVAL	SDF_LCL	SDF_UCL	STRATUM
20756	0	4.44	1	0.82556	.	.	1
20757	0	4.44	1	0.82556	.	.	1
20758	0	4.44	1	0.82556	.	.	1
20759	0	4.44	1	0.82556	.	.	1
20760	0	4.44	1	0.82556	.	.	1
20761	0	4.44	1	0.82556	.	.	1
20762	0	4.44	1	0.82556	.	.	1
20763	0	4.44	1	0.82556	.	.	1
20764	0	4.44	1	0.82556	.	.	1
20765	0	4.44	1	0.82556	.	.	1
20766	0	4.44	1	0.82556	.	.	1
20767	0	4.44	1	0.82556	.	.	1
20768	0	4.44	1	0.82556	.	.	1
20769	0	4.44	1	0.82556	.	.	1
20770	0	4.44	1	0.82556	.	.	1
20771	0	4.44	1	0.82556	.	.	1
20772	0	4.44	1	0.82556	.	.	1
20773	0	4.44	1	0.82556	.	.	1
20774	0	4.44	1	0.82556	.	.	1
20775	0	4.44	1	0.82556	.	.	1
20776	0	4.44	1	0.82556	.	.	1
20777	0	4.44	1	0.82556	.	.	1
20778	0	4.44	1	0.82556	.	.	1
20779	0	4.44	1	0.82556	.	.	1
20780	0	4.44	1	0.82556	.	.	1
20781	0	4.44	1	0.82556	.	.	1
20782	0	4.44	1	0.82556	.	.	1
20783	0	4.44	1	0.82556	.	.	1
20784	0	4.44	1	0.82556	.	.	1
20785	0	4.45	0	0.82535	0.82141	0.82920	1
20786	0	4.45	1	0.82535	.	.	1
20787	0	4.45	1	0.82535	.	.	1
20788	0	4.45	1	0.82535	.	.	1
20789	0	4.45	1	0.82535	.	.	1
20790	0	4.45	1	0.82535	.	.	1

Obs	Sub_group	fuptimey	_CENSOR_	SURVIVAL	SDF_LCL	SDF_UCL	STRATUM
**20791**	0	4.45	1	0.82535	.	.	1
**20792**	0	4.45	1	0.82535	.	.	1
**20793**	0	4.45	1	0.82535	.	.	1
**20794**	0	4.45	1	0.82535	.	.	1
**20795**	0	4.45	1	0.82535	.	.	1
**20796**	0	4.45	1	0.82535	.	.	1
**20797**	0	4.45	1	0.82535	.	.	1
**20798**	0	4.45	1	0.82535	.	.	1
**20799**	0	4.45	1	0.82535	.	.	1
**20800**	0	4.45	1	0.82535	.	.	1
**20801**	0	4.45	1	0.82535	.	.	1
**20802**	0	4.45	1	0.82535	.	.	1
**20803**	0	4.45	1	0.82535	.	.	1
**20804**	0	4.45	1	0.82535	.	.	1
**20805**	0	4.45	1	0.82535	.	.	1
**20806**	0	4.45	1	0.82535	.	.	1
**20807**	0	4.45	1	0.82535	.	.	1
**20808**	0	4.45	1	0.82535	.	.	1
**20809**	0	4.45	1	0.82535	.	.	1
**20810**	0	4.45	1	0.82535	.	.	1
**20811**	0	4.45	1	0.82535	.	.	1
**20812**	0	4.45	1	0.82535	.	.	1
**20813**	0	4.45	1	0.82535	.	.	1
**20814**	0	4.45	1	0.82535	.	.	1
**20815**	0	4.45	1	0.82535	.	.	1
**20816**	0	4.45	1	0.82535	.	.	1
**20817**	0	4.45	1	0.82535	.	.	1
**20818**	0	4.46	0	0.82509	0.82115	0.82895	1
**20819**	0	4.46	1	0.82509	.	.	1
**20820**	0	4.46	1	0.82509	.	.	1
**20821**	0	4.46	1	0.82509	.	.	1
**20822**	0	4.46	1	0.82509	.	.	1
**20823**	0	4.46	1	0.82509	.	.	1
**20824**	0	4.46	1	0.82509	.	.	1
**20825**	0	4.46	1	0.82509	.	.	1

Obs	Sub_group	fuptimey	_CENSOR_	SURVIVAL	SDF_LCL	SDF_UCL	STRATUM
**20826**	0	4.46	1	0.82509	.	.	1
**20827**	0	4.46	1	0.82509	.	.	1
**20828**	0	4.46	1	0.82509	.	.	1
**20829**	0	4.46	1	0.82509	.	.	1
**20830**	0	4.46	1	0.82509	.	.	1
**20831**	0	4.46	1	0.82509	.	.	1
**20832**	0	4.46	1	0.82509	.	.	1
**20833**	0	4.46	1	0.82509	.	.	1
**20834**	0	4.46	1	0.82509	.	.	1
**20835**	0	4.46	1	0.82509	.	.	1
**20836**	0	4.46	1	0.82509	.	.	1
**20837**	0	4.46	1	0.82509	.	.	1
**20838**	0	4.46	1	0.82509	.	.	1
**20839**	0	4.46	1	0.82509	.	.	1
**20840**	0	4.46	1	0.82509	.	.	1
**20841**	0	4.46	1	0.82509	.	.	1
**20842**	0	4.46	1	0.82509	.	.	1
**20843**	0	4.46	1	0.82509	.	.	1
**20844**	0	4.46	1	0.82509	.	.	1
**20845**	0	4.46	1	0.82509	.	.	1
**20846**	0	4.46	1	0.82509	.	.	1
**20847**	0	4.46	1	0.82509	.	.	1
**20848**	0	4.46	1	0.82509	.	.	1
**20849**	0	4.46	1	0.82509	.	.	1
**20850**	0	4.47	0	0.82492	0.82098	0.82879	1
**20851**	0	4.47	1	0.82492	.	.	1
**20852**	0	4.47	1	0.82492	.	.	1
**20853**	0	4.47	1	0.82492	.	.	1
**20854**	0	4.47	1	0.82492	.	.	1
**20855**	0	4.47	1	0.82492	.	.	1
**20856**	0	4.47	1	0.82492	.	.	1
**20857**	0	4.47	1	0.82492	.	.	1
**20858**	0	4.47	1	0.82492	.	.	1
**20859**	0	4.47	1	0.82492	.	.	1
**20860**	0	4.47	1	0.82492	.	.	1

Obs	Sub_group	fuptimey	_CENSOR_	SURVIVAL	SDF_LCL	SDF_UCL	STRATUM
20861	0	4.47	1	0.82492	.	.	1
20862	0	4.47	1	0.82492	.	.	1
20863	0	4.47	1	0.82492	.	.	1
20864	0	4.47	1	0.82492	.	.	1
20865	0	4.47	1	0.82492	.	.	1
20866	0	4.47	1	0.82492	.	.	1
20867	0	4.47	1	0.82492	.	.	1
20868	0	4.47	1	0.82492	.	.	1
20869	0	4.47	1	0.82492	.	.	1
20870	0	4.47	1	0.82492	.	.	1
20871	0	4.47	1	0.82492	.	.	1
20872	0	4.47	1	0.82492	.	.	1
20873	0	4.47	1	0.82492	.	.	1
20874	0	4.47	1	0.82492	.	.	1
20875	0	4.47	1	0.82492	.	.	1
20876	0	4.47	1	0.82492	.	.	1
20877	0	4.47	1	0.82492	.	.	1
20878	0	4.47	1	0.82492	.	.	1
20879	0	4.47	1	0.82492	.	.	1
20880	0	4.47	1	0.82492	.	.	1
20881	0	4.47	1	0.82492	.	.	1
20882	0	4.47	1	0.82492	.	.	1
20883	0	4.47	1	0.82492	.	.	1
20884	0	4.47	1	0.82492	.	.	1
20885	0	4.47	1	0.82492	.	.	1
20886	0	4.47	1	0.82492	.	.	1
20887	0	4.47	1	0.82492	.	.	1
20888	0	4.47	1	0.82492	.	.	1
20889	0	4.47	1	0.82492	.	.	1
20890	0	4.47	1	0.82492	.	.	1
20891	0	4.47	1	0.82492	.	.	1
20892	0	4.47	1	0.82492	.	.	1
20893	0	4.47	1	0.82492	.	.	1
20894	0	4.47	1	0.82492	.	.	1
20895	0	4.47	1	0.82492	.	.	1

Obs	Sub_group	fuptimey	_CENSOR_	SURVIVAL	SDF_LCL	SDF_UCL	STRATUM
20896	0	4.48	0	0.82458	0.82064	0.82845	1
20897	0	4.48	1	0.82458	.	.	1
20898	0	4.48	1	0.82458	.	.	1
20899	0	4.48	1	0.82458	.	.	1
20900	0	4.48	1	0.82458	.	.	1
20901	0	4.48	1	0.82458	.	.	1
20902	0	4.48	1	0.82458	.	.	1
20903	0	4.48	1	0.82458	.	.	1
20904	0	4.48	1	0.82458	.	.	1
20905	0	4.48	1	0.82458	.	.	1
20906	0	4.48	1	0.82458	.	.	1
20907	0	4.48	1	0.82458	.	.	1
20908	0	4.48	1	0.82458	.	.	1
20909	0	4.48	1	0.82458	.	.	1
20910	0	4.48	1	0.82458	.	.	1
20911	0	4.48	1	0.82458	.	.	1
20912	0	4.48	1	0.82458	.	.	1
20913	0	4.48	1	0.82458	.	.	1
20914	0	4.48	1	0.82458	.	.	1
20915	0	4.48	1	0.82458	.	.	1
20916	0	4.48	1	0.82458	.	.	1
20917	0	4.48	1	0.82458	.	.	1
20918	0	4.48	1	0.82458	.	.	1
20919	0	4.48	1	0.82458	.	.	1
20920	0	4.48	1	0.82458	.	.	1
20921	0	4.48	1	0.82458	.	.	1
20922	0	4.48	1	0.82458	.	.	1
20923	0	4.48	1	0.82458	.	.	1
20924	0	4.48	1	0.82458	.	.	1
20925	0	4.49	0	0.82433	0.82038	0.82820	1
20926	0	4.49	1	0.82433	.	.	1
20927	0	4.49	1	0.82433	.	.	1
20928	0	4.49	1	0.82433	.	.	1
20929	0	4.49	1	0.82433	.	.	1
20930	0	4.49	1	0.82433	.	.	1

Obs	Sub_group	fuptimey	_CENSOR_	SURVIVAL	SDF_LCL	SDF_UCL	STRATUM
20931	0	4.49	1	0.82433	.	.	1
20932	0	4.49	1	0.82433	.	.	1
20933	0	4.49	1	0.82433	.	.	1
20934	0	4.49	1	0.82433	.	.	1
20935	0	4.49	1	0.82433	.	.	1
20936	0	4.49	1	0.82433	.	.	1
20937	0	4.49	1	0.82433	.	.	1
20938	0	4.49	1	0.82433	.	.	1
20939	0	4.49	1	0.82433	.	.	1
20940	0	4.49	1	0.82433	.	.	1
20941	0	4.49	1	0.82433	.	.	1
20942	0	4.49	1	0.82433	.	.	1
20943	0	4.49	1	0.82433	.	.	1
20944	0	4.49	1	0.82433	.	.	1
20945	0	4.49	1	0.82433	.	.	1
20946	0	4.49	1	0.82433	.	.	1
20947	0	4.49	1	0.82433	.	.	1
20948	0	4.49	1	0.82433	.	.	1
20949	0	4.49	1	0.82433	.	.	1
20950	0	4.49	1	0.82433	.	.	1
20951	0	4.49	1	0.82433	.	.	1
20952	0	4.49	1	0.82433	.	.	1
20953	0	4.49	1	0.82433	.	.	1
20954	0	4.49	1	0.82433	.	.	1
20955	0	4.49	1	0.82433	.	.	1
20956	0	4.49	1	0.82433	.	.	1
20957	0	4.49	1	0.82433	.	.	1
20958	0	4.49	1	0.82433	.	.	1
20959	0	4.49	1	0.82433	.	.	1
20960	0	4.49	1	0.82433	.	.	1
20961	0	4.49	1	0.82433	.	.	1
20962	0	4.49	1	0.82433	.	.	1
20963	0	4.49	1	0.82433	.	.	1
20964	0	4.49	1	0.82433	.	.	1
20965	0	4.49	1	0.82433	.	.	1

Obs	Sub_group	fuptimey	_CENSOR_	SURVIVAL	SDF_LCL	SDF_UCL	STRATUM
20966	0	4.49	1	0.82433	.	.	1
20967	0	4.49	1	0.82433	.	.	1
20968	0	4.49	1	0.82433	.	.	1
20969	0	4.49	1	0.82433	.	.	1
20970	0	4.49	1	0.82433	.	.	1
20971	0	4.49	1	0.82433	.	.	1
20972	0	4.49	1	0.82433	.	.	1
20973	0	4.49	1	0.82433	.	.	1
20974	0	4.49	1	0.82433	.	.	1
20975	0	4.49	1	0.82433	.	.	1
20976	0	4.49	1	0.82433	.	.	1
20977	0	4.49	1	0.82433	.	.	1
20978	0	4.50	0	0.82416	0.82020	0.82803	1
20979	0	4.50	1	0.82416	.	.	1
20980	0	4.50	1	0.82416	.	.	1
20981	0	4.50	1	0.82416	.	.	1
20982	0	4.50	1	0.82416	.	.	1
20983	0	4.50	1	0.82416	.	.	1
20984	0	4.50	1	0.82416	.	.	1
20985	0	4.50	1	0.82416	.	.	1
20986	0	4.50	1	0.82416	.	.	1
20987	0	4.50	1	0.82416	.	.	1
20988	0	4.50	1	0.82416	.	.	1
20989	0	4.50	1	0.82416	.	.	1
20990	0	4.50	1	0.82416	.	.	1
20991	0	4.50	1	0.82416	.	.	1
20992	0	4.50	1	0.82416	.	.	1
20993	0	4.50	1	0.82416	.	.	1
20994	0	4.50	1	0.82416	.	.	1
20995	0	4.50	1	0.82416	.	.	1
20996	0	4.50	1	0.82416	.	.	1
20997	0	4.50	1	0.82416	.	.	1
20998	0	4.50	1	0.82416	.	.	1
20999	0	4.50	1	0.82416	.	.	1
21000	0	4.50	1	0.82416	.	.	1

Obs	Sub_group	fuptimey	_CENSOR_	SURVIVAL	SDF_LCL	SDF_UCL	STRATUM
21001	0	4.50	1	0.82416	.	.	1
21002	0	4.50	1	0.82416	.	.	1
21003	0	4.50	1	0.82416	.	.	1
21004	0	4.50	1	0.82416	.	.	1
21005	0	4.50	1	0.82416	.	.	1
21006	0	4.50	1	0.82416	.	.	1
21007	0	4.50	1	0.82416	.	.	1
21008	0	4.50	1	0.82416	.	.	1
21009	0	4.50	1	0.82416	.	.	1
21010	0	4.50	1	0.82416	.	.	1
21011	0	4.50	1	0.82416	.	.	1
21012	0	4.50	1	0.82416	.	.	1
21013	0	4.50	1	0.82416	.	.	1
21014	0	4.50	1	0.82416	.	.	1
21015	0	4.51	0	0.82403	0.82007	0.82791	1
21016	0	4.51	1	0.82403	.	.	1
21017	0	4.51	1	0.82403	.	.	1
21018	0	4.51	1	0.82403	.	.	1
21019	0	4.51	1	0.82403	.	.	1
21020	0	4.51	1	0.82403	.	.	1
21021	0	4.51	1	0.82403	.	.	1
21022	0	4.51	1	0.82403	.	.	1
21023	0	4.51	1	0.82403	.	.	1
21024	0	4.51	1	0.82403	.	.	1
21025	0	4.51	1	0.82403	.	.	1
21026	0	4.51	1	0.82403	.	.	1
21027	0	4.51	1	0.82403	.	.	1
21028	0	4.51	1	0.82403	.	.	1
21029	0	4.51	1	0.82403	.	.	1
21030	0	4.51	1	0.82403	.	.	1
21031	0	4.51	1	0.82403	.	.	1
21032	0	4.51	1	0.82403	.	.	1
21033	0	4.51	1	0.82403	.	.	1
21034	0	4.51	1	0.82403	.	.	1
21035	0	4.51	1	0.82403	.	.	1

Obs	Sub_group	fuptimey	_CENSOR_	SURVIVAL	SDF_LCL	SDF_UCL	STRATUM
21036	0	4.51	1	0.82403	.	.	1
21037	0	4.51	1	0.82403	.	.	1
21038	0	4.51	1	0.82403	.	.	1
21039	0	4.51	1	0.82403	.	.	1
21040	0	4.51	1	0.82403	.	.	1
21041	0	4.51	1	0.82403	.	.	1
21042	0	4.51	1	0.82403	.	.	1
21043	0	4.51	1	0.82403	.	.	1
21044	0	4.51	1	0.82403	.	.	1
21045	0	4.51	1	0.82403	.	.	1
21046	0	4.51	1	0.82403	.	.	1
21047	0	4.51	1	0.82403	.	.	1
21048	0	4.51	1	0.82403	.	.	1
21049	0	4.51	1	0.82403	.	.	1
21050	0	4.51	1	0.82403	.	.	1
21051	0	4.51	1	0.82403	.	.	1
21052	0	4.51	1	0.82403	.	.	1
21053	0	4.51	1	0.82403	.	.	1
21054	0	4.51	1	0.82403	.	.	1
21055	0	4.51	1	0.82403	.	.	1
21056	0	4.51	1	0.82403	.	.	1
21057	0	4.51	1	0.82403	.	.	1
21058	0	4.51	1	0.82403	.	.	1
21059	0	4.51	1	0.82403	.	.	1
21060	0	4.51	1	0.82403	.	.	1
21061	0	4.51	1	0.82403	.	.	1
21062	0	4.51	1	0.82403	.	.	1
21063	0	4.51	1	0.82403	.	.	1
21064	0	4.51	1	0.82403	.	.	1
21065	0	4.51	1	0.82403	.	.	1
21066	0	4.52	0	0.82381	0.81985	0.82769	1
21067	0	4.52	1	0.82381	.	.	1
21068	0	4.52	1	0.82381	.	.	1
21069	0	4.52	1	0.82381	.	.	1
21070	0	4.52	1	0.82381	.	.	1

Obs	Sub_group	fuptimey	_CENSOR_	SURVIVAL	SDF_LCL	SDF_UCL	STRATUM
21071	0	4.52	1	0.82381	.	.	1
21072	0	4.52	1	0.82381	.	.	1
21073	0	4.52	1	0.82381	.	.	1
21074	0	4.52	1	0.82381	.	.	1
21075	0	4.52	1	0.82381	.	.	1
21076	0	4.52	1	0.82381	.	.	1
21077	0	4.52	1	0.82381	.	.	1
21078	0	4.52	1	0.82381	.	.	1
21079	0	4.52	1	0.82381	.	.	1
21080	0	4.52	1	0.82381	.	.	1
21081	0	4.52	1	0.82381	.	.	1
21082	0	4.52	1	0.82381	.	.	1
21083	0	4.52	1	0.82381	.	.	1
21084	0	4.52	1	0.82381	.	.	1
21085	0	4.52	1	0.82381	.	.	1
21086	0	4.52	1	0.82381	.	.	1
21087	0	4.52	1	0.82381	.	.	1
21088	0	4.53	0	0.82351	0.81955	0.82740	1
21089	0	4.53	1	0.82351	.	.	1
21090	0	4.53	1	0.82351	.	.	1
21091	0	4.53	1	0.82351	.	.	1
21092	0	4.53	1	0.82351	.	.	1
21093	0	4.53	1	0.82351	.	.	1
21094	0	4.53	1	0.82351	.	.	1
21095	0	4.53	1	0.82351	.	.	1
21096	0	4.53	1	0.82351	.	.	1
21097	0	4.53	1	0.82351	.	.	1
21098	0	4.53	1	0.82351	.	.	1
21099	0	4.53	1	0.82351	.	.	1
21100	0	4.53	1	0.82351	.	.	1
21101	0	4.53	1	0.82351	.	.	1
21102	0	4.53	1	0.82351	.	.	1
21103	0	4.53	1	0.82351	.	.	1
21104	0	4.53	1	0.82351	.	.	1
21105	0	4.53	1	0.82351	.	.	1

Obs	Sub_group	fuptimey	_CENSOR_	SURVIVAL	SDF_LCL	SDF_UCL	STRATUM
21106	0	4.53	1	0.82351	.	.	1
21107	0	4.53	1	0.82351	.	.	1
21108	0	4.53	1	0.82351	.	.	1
21109	0	4.53	1	0.82351	.	.	1
21110	0	4.53	1	0.82351	.	.	1
21111	0	4.53	1	0.82351	.	.	1
21112	0	4.53	1	0.82351	.	.	1
21113	0	4.53	1	0.82351	.	.	1
21114	0	4.53	1	0.82351	.	.	1
21115	0	4.53	1	0.82351	.	.	1
21116	0	4.53	1	0.82351	.	.	1
21117	0	4.53	1	0.82351	.	.	1
21118	0	4.53	1	0.82351	.	.	1
21119	0	4.53	1	0.82351	.	.	1
21120	0	4.53	1	0.82351	.	.	1
21121	0	4.53	1	0.82351	.	.	1
21122	0	4.53	1	0.82351	.	.	1
21123	0	4.53	1	0.82351	.	.	1
21124	0	4.53	1	0.82351	.	.	1
21125	0	4.53	1	0.82351	.	.	1
21126	0	4.53	1	0.82351	.	.	1
21127	0	4.53	1	0.82351	.	.	1
21128	0	4.54	0	0.82325	0.81929	0.82715	1
21129	0	4.54	1	0.82325	.	.	1
21130	0	4.54	1	0.82325	.	.	1
21131	0	4.54	1	0.82325	.	.	1
21132	0	4.54	1	0.82325	.	.	1
21133	0	4.54	1	0.82325	.	.	1
21134	0	4.54	1	0.82325	.	.	1
21135	0	4.54	1	0.82325	.	.	1
21136	0	4.54	1	0.82325	.	.	1
21137	0	4.54	1	0.82325	.	.	1
21138	0	4.54	1	0.82325	.	.	1
21139	0	4.54	1	0.82325	.	.	1
21140	0	4.54	1	0.82325	.	.	1

Obs	Sub_group	fuptimey	_CENSOR_	SURVIVAL	SDF_LCL	SDF_UCL	STRATUM
21141	0	4.54	1	0.82325	.	.	1
21142	0	4.54	1	0.82325	.	.	1
21143	0	4.54	1	0.82325	.	.	1
21144	0	4.54	1	0.82325	.	.	1
21145	0	4.54	1	0.82325	.	.	1
21146	0	4.54	1	0.82325	.	.	1
21147	0	4.54	1	0.82325	.	.	1
21148	0	4.54	1	0.82325	.	.	1
21149	0	4.54	1	0.82325	.	.	1
21150	0	4.54	1	0.82325	.	.	1
21151	0	4.54	1	0.82325	.	.	1
21152	0	4.54	1	0.82325	.	.	1
21153	0	4.54	1	0.82325	.	.	1
21154	0	4.54	1	0.82325	.	.	1
21155	0	4.54	1	0.82325	.	.	1
21156	0	4.54	1	0.82325	.	.	1
21157	0	4.54	1	0.82325	.	.	1
21158	0	4.55	0	0.82282	0.81885	0.82672	1
21159	0	4.55	1	0.82282	.	.	1
21160	0	4.55	1	0.82282	.	.	1
21161	0	4.55	1	0.82282	.	.	1
21162	0	4.55	1	0.82282	.	.	1
21163	0	4.55	1	0.82282	.	.	1
21164	0	4.55	1	0.82282	.	.	1
21165	0	4.55	1	0.82282	.	.	1
21166	0	4.55	1	0.82282	.	.	1
21167	0	4.55	1	0.82282	.	.	1
21168	0	4.55	1	0.82282	.	.	1
21169	0	4.55	1	0.82282	.	.	1
21170	0	4.55	1	0.82282	.	.	1
21171	0	4.55	1	0.82282	.	.	1
21172	0	4.55	1	0.82282	.	.	1
21173	0	4.55	1	0.82282	.	.	1
21174	0	4.55	1	0.82282	.	.	1
21175	0	4.55	1	0.82282	.	.	1

Obs	Sub_group	fuptimey	_CENSOR_	SURVIVAL	SDF_LCL	SDF_UCL	STRATUM
21176	0	4.55	1	0.82282	.	.	1
21177	0	4.55	1	0.82282	.	.	1
21178	0	4.55	1	0.82282	.	.	1
21179	0	4.55	1	0.82282	.	.	1
21180	0	4.55	1	0.82282	.	.	1
21181	0	4.55	1	0.82282	.	.	1
21182	0	4.55	1	0.82282	.	.	1
21183	0	4.55	1	0.82282	.	.	1
21184	0	4.55	1	0.82282	.	.	1
21185	0	4.55	1	0.82282	.	.	1
21186	0	4.55	1	0.82282	.	.	1
21187	0	4.55	1	0.82282	.	.	1
21188	0	4.55	1	0.82282	.	.	1
21189	0	4.55	1	0.82282	.	.	1
21190	0	4.55	1	0.82282	.	.	1
21191	0	4.55	1	0.82282	.	.	1
21192	0	4.55	1	0.82282	.	.	1
21193	0	4.55	1	0.82282	.	.	1
21194	0	4.55	1	0.82282	.	.	1
21195	0	4.55	1	0.82282	.	.	1
21196	0	4.55	1	0.82282	.	.	1
21197	0	4.55	1	0.82282	.	.	1
21198	0	4.55	1	0.82282	.	.	1
21199	0	4.55	1	0.82282	.	.	1
21200	0	4.55	1	0.82282	.	.	1
21201	0	4.55	1	0.82282	.	.	1
21202	0	4.55	1	0.82282	.	.	1
21203	0	4.55	1	0.82282	.	.	1
21204	0	4.55	1	0.82282	.	.	1
21205	0	4.55	1	0.82282	.	.	1
21206	0	4.55	1	0.82282	.	.	1
21207	0	4.55	1	0.82282	.	.	1
21208	0	4.55	1	0.82282	.	.	1
21209	0	4.55	1	0.82282	.	.	1
21210	0	4.56	0	0.82269	0.81872	0.82659	1

Obs	Sub_group	fuptimey	_CENSOR_	SURVIVAL	SDF_LCL	SDF_UCL	STRATUM
21211	0	4.56	1	0.82269	.	.	1
21212	0	4.56	1	0.82269	.	.	1
21213	0	4.56	1	0.82269	.	.	1
21214	0	4.56	1	0.82269	.	.	1
21215	0	4.56	1	0.82269	.	.	1
21216	0	4.56	1	0.82269	.	.	1
21217	0	4.56	1	0.82269	.	.	1
21218	0	4.56	1	0.82269	.	.	1
21219	0	4.56	1	0.82269	.	.	1
21220	0	4.56	1	0.82269	.	.	1
21221	0	4.56	1	0.82269	.	.	1
21222	0	4.56	1	0.82269	.	.	1
21223	0	4.56	1	0.82269	.	.	1
21224	0	4.56	1	0.82269	.	.	1
21225	0	4.56	1	0.82269	.	.	1
21226	0	4.56	1	0.82269	.	.	1
21227	0	4.56	1	0.82269	.	.	1
21228	0	4.56	1	0.82269	.	.	1
21229	0	4.56	1	0.82269	.	.	1
21230	0	4.56	1	0.82269	.	.	1
21231	0	4.56	1	0.82269	.	.	1
21232	0	4.56	1	0.82269	.	.	1
21233	0	4.56	1	0.82269	.	.	1
21234	0	4.56	1	0.82269	.	.	1
21235	0	4.56	1	0.82269	.	.	1
21236	0	4.56	1	0.82269	.	.	1
21237	0	4.56	1	0.82269	.	.	1
21238	0	4.56	1	0.82269	.	.	1
21239	0	4.56	1	0.82269	.	.	1
21240	0	4.56	1	0.82269	.	.	1
21241	0	4.56	1	0.82269	.	.	1
21242	0	4.56	1	0.82269	.	.	1
21243	0	4.56	1	0.82269	.	.	1
21244	0	4.57	1	0.82269	.	.	1
21245	0	4.57	1	0.82269	.	.	1

Obs	Sub_group	fuptimey	_CENSOR_	SURVIVAL	SDF_LCL	SDF_UCL	STRATUM
21246	0	4.57	1	0.82269	.	.	1
21247	0	4.57	1	0.82269	.	.	1
21248	0	4.57	1	0.82269	.	.	1
21249	0	4.57	1	0.82269	.	.	1
21250	0	4.57	1	0.82269	.	.	1
21251	0	4.57	1	0.82269	.	.	1
21252	0	4.57	1	0.82269	.	.	1
21253	0	4.57	1	0.82269	.	.	1
21254	0	4.57	1	0.82269	.	.	1
21255	0	4.57	1	0.82269	.	.	1
21256	0	4.57	1	0.82269	.	.	1
21257	0	4.57	1	0.82269	.	.	1
21258	0	4.57	1	0.82269	.	.	1
21259	0	4.57	1	0.82269	.	.	1
21260	0	4.57	1	0.82269	.	.	1
21261	0	4.57	1	0.82269	.	.	1
21262	0	4.57	1	0.82269	.	.	1
21263	0	4.57	1	0.82269	.	.	1
21264	0	4.57	1	0.82269	.	.	1
21265	0	4.57	1	0.82269	.	.	1
21266	0	4.57	1	0.82269	.	.	1
21267	0	4.57	1	0.82269	.	.	1
21268	0	4.57	1	0.82269	.	.	1
21269	0	4.57	1	0.82269	.	.	1
21270	0	4.57	1	0.82269	.	.	1
21271	0	4.57	1	0.82269	.	.	1
21272	0	4.57	1	0.82269	.	.	1
21273	0	4.57	1	0.82269	.	.	1
21274	0	4.57	1	0.82269	.	.	1
21275	0	4.57	1	0.82269	.	.	1
21276	0	4.57	1	0.82269	.	.	1
21277	0	4.57	1	0.82269	.	.	1
21278	0	4.57	1	0.82269	.	.	1
21279	0	4.57	1	0.82269	.	.	1
21280	0	4.57	1	0.82269	.	.	1

Obs	Sub_group	fuptimey	_CENSOR_	SURVIVAL	SDF_LCL	SDF_UCL	STRATUM
**21281**	0	4.57	1	0.82269	.	.	1
**21282**	0	4.57	1	0.82269	.	.	1
**21283**	0	4.57	1	0.82269	.	.	1
**21284**	0	4.57	1	0.82269	.	.	1
**21285**	0	4.57	1	0.82269	.	.	1
**21286**	0	4.57	1	0.82269	.	.	1
**21287**	0	4.57	1	0.82269	.	.	1
**21288**	0	4.58	0	0.82252	0.81854	0.82642	1
**21289**	0	4.58	1	0.82252	.	.	1
**21290**	0	4.58	1	0.82252	.	.	1
**21291**	0	4.58	1	0.82252	.	.	1
**21292**	0	4.58	1	0.82252	.	.	1
**21293**	0	4.58	1	0.82252	.	.	1
**21294**	0	4.58	1	0.82252	.	.	1
**21295**	0	4.58	1	0.82252	.	.	1
**21296**	0	4.58	1	0.82252	.	.	1
**21297**	0	4.58	1	0.82252	.	.	1
**21298**	0	4.58	1	0.82252	.	.	1
**21299**	0	4.58	1	0.82252	.	.	1
**21300**	0	4.58	1	0.82252	.	.	1
**21301**	0	4.58	1	0.82252	.	.	1
**21302**	0	4.58	1	0.82252	.	.	1
**21303**	0	4.58	1	0.82252	.	.	1
**21304**	0	4.58	1	0.82252	.	.	1
**21305**	0	4.58	1	0.82252	.	.	1
**21306**	0	4.58	1	0.82252	.	.	1
**21307**	0	4.58	1	0.82252	.	.	1
**21308**	0	4.58	1	0.82252	.	.	1
**21309**	0	4.58	1	0.82252	.	.	1
**21310**	0	4.58	1	0.82252	.	.	1
**21311**	0	4.58	1	0.82252	.	.	1
**21312**	0	4.58	1	0.82252	.	.	1
**21313**	0	4.58	1	0.82252	.	.	1
**21314**	0	4.58	1	0.82252	.	.	1
**21315**	0	4.58	1	0.82252	.	.	1

Obs	Sub_group	fuptimey	_CENSOR_	SURVIVAL	SDF_LCL	SDF_UCL	STRATUM
21316	0	4.58	1	0.82252	.	.	1
21317	0	4.58	1	0.82252	.	.	1
21318	0	4.58	1	0.82252	.	.	1
21319	0	4.58	1	0.82252	.	.	1
21320	0	4.58	1	0.82252	.	.	1
21321	0	4.58	1	0.82252	.	.	1
21322	0	4.58	1	0.82252	.	.	1
21323	0	4.59	0	0.82239	0.81841	0.82630	1
21324	0	4.59	1	0.82239	.	.	1
21325	0	4.59	1	0.82239	.	.	1
21326	0	4.59	1	0.82239	.	.	1
21327	0	4.59	1	0.82239	.	.	1
21328	0	4.59	1	0.82239	.	.	1
21329	0	4.59	1	0.82239	.	.	1
21330	0	4.59	1	0.82239	.	.	1
21331	0	4.59	1	0.82239	.	.	1
21332	0	4.59	1	0.82239	.	.	1
21333	0	4.59	1	0.82239	.	.	1
21334	0	4.59	1	0.82239	.	.	1
21335	0	4.59	1	0.82239	.	.	1
21336	0	4.59	1	0.82239	.	.	1
21337	0	4.59	1	0.82239	.	.	1
21338	0	4.59	1	0.82239	.	.	1
21339	0	4.59	1	0.82239	.	.	1
21340	0	4.59	1	0.82239	.	.	1
21341	0	4.59	1	0.82239	.	.	1
21342	0	4.59	1	0.82239	.	.	1
21343	0	4.59	1	0.82239	.	.	1
21344	0	4.59	1	0.82239	.	.	1
21345	0	4.59	1	0.82239	.	.	1
21346	0	4.59	1	0.82239	.	.	1
21347	0	4.59	1	0.82239	.	.	1
21348	0	4.59	1	0.82239	.	.	1
21349	0	4.59	1	0.82239	.	.	1
21350	0	4.59	1	0.82239	.	.	1

Obs	Sub_group	fuptimey	_CENSOR_	SURVIVAL	SDF_LCL	SDF_UCL	STRATUM
**21351**	0	4.59	1	0.82239	.	.	1
**21352**	0	4.59	1	0.82239	.	.	1
**21353**	0	4.59	1	0.82239	.	.	1
**21354**	0	4.59	1	0.82239	.	.	1
**21355**	0	4.59	1	0.82239	.	.	1
**21356**	0	4.59	1	0.82239	.	.	1
**21357**	0	4.59	1	0.82239	.	.	1
**21358**	0	4.59	1	0.82239	.	.	1
**21359**	0	4.59	1	0.82239	.	.	1
**21360**	0	4.59	1	0.82239	.	.	1
**21361**	0	4.60	0	0.82222	0.81823	0.82612	1
**21362**	0	4.60	1	0.82222	.	.	1
**21363**	0	4.60	1	0.82222	.	.	1
**21364**	0	4.60	1	0.82222	.	.	1
**21365**	0	4.60	1	0.82222	.	.	1
**21366**	0	4.60	1	0.82222	.	.	1
**21367**	0	4.60	1	0.82222	.	.	1
**21368**	0	4.60	1	0.82222	.	.	1
**21369**	0	4.60	1	0.82222	.	.	1
**21370**	0	4.60	1	0.82222	.	.	1
**21371**	0	4.60	1	0.82222	.	.	1
**21372**	0	4.60	1	0.82222	.	.	1
**21373**	0	4.60	1	0.82222	.	.	1
**21374**	0	4.60	1	0.82222	.	.	1
**21375**	0	4.60	1	0.82222	.	.	1
**21376**	0	4.60	1	0.82222	.	.	1
**21377**	0	4.60	1	0.82222	.	.	1
**21378**	0	4.60	1	0.82222	.	.	1
**21379**	0	4.60	1	0.82222	.	.	1
**21380**	0	4.60	1	0.82222	.	.	1
**21381**	0	4.60	1	0.82222	.	.	1
**21382**	0	4.60	1	0.82222	.	.	1
**21383**	0	4.60	1	0.82222	.	.	1
**21384**	0	4.60	1	0.82222	.	.	1
**21385**	0	4.60	1	0.82222	.	.	1

Obs	Sub_group	fuptimey	_CENSOR_	SURVIVAL	SDF_LCL	SDF_UCL	STRATUM
21386	0	4.60	1	0.82222	.	.	1
21387	0	4.60	1	0.82222	.	.	1
21388	0	4.60	1	0.82222	.	.	1
21389	0	4.60	1	0.82222	.	.	1
21390	0	4.60	1	0.82222	.	.	1
21391	0	4.60	1	0.82222	.	.	1
21392	0	4.60	1	0.82222	.	.	1
21393	0	4.60	1	0.82222	.	.	1
21394	0	4.61	0	0.82191	0.81792	0.82582	1
21395	0	4.61	1	0.82191	.	.	1
21396	0	4.61	1	0.82191	.	.	1
21397	0	4.61	1	0.82191	.	.	1
21398	0	4.61	1	0.82191	.	.	1
21399	0	4.61	1	0.82191	.	.	1
21400	0	4.61	1	0.82191	.	.	1
21401	0	4.61	1	0.82191	.	.	1
21402	0	4.61	1	0.82191	.	.	1
21403	0	4.61	1	0.82191	.	.	1
21404	0	4.61	1	0.82191	.	.	1
21405	0	4.61	1	0.82191	.	.	1
21406	0	4.61	1	0.82191	.	.	1
21407	0	4.61	1	0.82191	.	.	1
21408	0	4.61	1	0.82191	.	.	1
21409	0	4.61	1	0.82191	.	.	1
21410	0	4.61	1	0.82191	.	.	1
21411	0	4.61	1	0.82191	.	.	1
21412	0	4.61	1	0.82191	.	.	1
21413	0	4.61	1	0.82191	.	.	1
21414	0	4.61	1	0.82191	.	.	1
21415	0	4.61	1	0.82191	.	.	1
21416	0	4.61	1	0.82191	.	.	1
21417	0	4.61	1	0.82191	.	.	1
21418	0	4.61	1	0.82191	.	.	1
21419	0	4.61	1	0.82191	.	.	1
21420	0	4.61	1	0.82191	.	.	1

Obs	Sub_group	fuptimey	_CENSOR_	SURVIVAL	SDF_LCL	SDF_UCL	STRATUM
21421	0	4.61	1	0.82191	.	.	1
21422	0	4.61	1	0.82191	.	.	1
21423	0	4.62	0	0.82165	0.81766	0.82557	1
21424	0	4.62	1	0.82165	.	.	1
21425	0	4.62	1	0.82165	.	.	1
21426	0	4.62	1	0.82165	.	.	1
21427	0	4.62	1	0.82165	.	.	1
21428	0	4.62	1	0.82165	.	.	1
21429	0	4.62	1	0.82165	.	.	1
21430	0	4.62	1	0.82165	.	.	1
21431	0	4.62	1	0.82165	.	.	1
21432	0	4.62	1	0.82165	.	.	1
21433	0	4.62	1	0.82165	.	.	1
21434	0	4.62	1	0.82165	.	.	1
21435	0	4.62	1	0.82165	.	.	1
21436	0	4.62	1	0.82165	.	.	1
21437	0	4.62	1	0.82165	.	.	1
21438	0	4.62	1	0.82165	.	.	1
21439	0	4.62	1	0.82165	.	.	1
21440	0	4.62	1	0.82165	.	.	1
21441	0	4.62	1	0.82165	.	.	1
21442	0	4.62	1	0.82165	.	.	1
21443	0	4.62	1	0.82165	.	.	1
21444	0	4.62	1	0.82165	.	.	1
21445	0	4.62	1	0.82165	.	.	1
21446	0	4.62	1	0.82165	.	.	1
21447	0	4.62	1	0.82165	.	.	1
21448	0	4.62	1	0.82165	.	.	1
21449	0	4.63	0	0.82152	0.81752	0.82544	1
21450	0	4.63	1	0.82152	.	.	1
21451	0	4.63	1	0.82152	.	.	1
21452	0	4.63	1	0.82152	.	.	1
21453	0	4.63	1	0.82152	.	.	1
21454	0	4.63	1	0.82152	.	.	1
21455	0	4.63	1	0.82152	.	.	1

Obs	Sub_group	fuptimey	_CENSOR_	SURVIVAL	SDF_LCL	SDF_UCL	STRATUM
21456	0	4.63	1	0.82152	.	.	1
21457	0	4.63	1	0.82152	.	.	1
21458	0	4.63	1	0.82152	.	.	1
21459	0	4.63	1	0.82152	.	.	1
21460	0	4.63	1	0.82152	.	.	1
21461	0	4.63	1	0.82152	.	.	1
21462	0	4.63	1	0.82152	.	.	1
21463	0	4.63	1	0.82152	.	.	1
21464	0	4.63	1	0.82152	.	.	1
21465	0	4.63	1	0.82152	.	.	1
21466	0	4.63	1	0.82152	.	.	1
21467	0	4.63	1	0.82152	.	.	1
21468	0	4.63	1	0.82152	.	.	1
21469	0	4.63	1	0.82152	.	.	1
21470	0	4.63	1	0.82152	.	.	1
21471	0	4.63	1	0.82152	.	.	1
21472	0	4.63	1	0.82152	.	.	1
21473	0	4.63	1	0.82152	.	.	1
21474	0	4.63	1	0.82152	.	.	1
21475	0	4.63	1	0.82152	.	.	1
21476	0	4.63	1	0.82152	.	.	1
21477	0	4.63	1	0.82152	.	.	1
21478	0	4.63	1	0.82152	.	.	1
21479	0	4.63	1	0.82152	.	.	1
21480	0	4.63	1	0.82152	.	.	1
21481	0	4.63	1	0.82152	.	.	1
21482	0	4.64	0	0.82134	0.81734	0.82527	1
21483	0	4.64	1	0.82134	.	.	1
21484	0	4.64	1	0.82134	.	.	1
21485	0	4.64	1	0.82134	.	.	1
21486	0	4.64	1	0.82134	.	.	1
21487	0	4.64	1	0.82134	.	.	1
21488	0	4.64	1	0.82134	.	.	1
21489	0	4.64	1	0.82134	.	.	1
21490	0	4.64	1	0.82134	.	.	1

Obs	Sub_group	fuptimey	_CENSOR_	SURVIVAL	SDF_LCL	SDF_UCL	STRATUM
21491	0	4.64	1	0.82134	.	.	1
21492	0	4.64	1	0.82134	.	.	1
21493	0	4.64	1	0.82134	.	.	1
21494	0	4.64	1	0.82134	.	.	1
21495	0	4.64	1	0.82134	.	.	1
21496	0	4.64	1	0.82134	.	.	1
21497	0	4.64	1	0.82134	.	.	1
21498	0	4.64	1	0.82134	.	.	1
21499	0	4.64	1	0.82134	.	.	1
21500	0	4.64	1	0.82134	.	.	1
21501	0	4.64	1	0.82134	.	.	1
21502	0	4.64	1	0.82134	.	.	1
21503	0	4.64	1	0.82134	.	.	1
21504	0	4.64	1	0.82134	.	.	1
21505	0	4.64	1	0.82134	.	.	1
21506	0	4.64	1	0.82134	.	.	1
21507	0	4.64	1	0.82134	.	.	1
21508	0	4.64	1	0.82134	.	.	1
21509	0	4.65	0	0.82104	0.81703	0.82496	1
21510	0	4.65	1	0.82104	.	.	1
21511	0	4.65	1	0.82104	.	.	1
21512	0	4.65	1	0.82104	.	.	1
21513	0	4.65	1	0.82104	.	.	1
21514	0	4.65	1	0.82104	.	.	1
21515	0	4.65	1	0.82104	.	.	1
21516	0	4.65	1	0.82104	.	.	1
21517	0	4.65	1	0.82104	.	.	1
21518	0	4.65	1	0.82104	.	.	1
21519	0	4.65	1	0.82104	.	.	1
21520	0	4.65	1	0.82104	.	.	1
21521	0	4.65	1	0.82104	.	.	1
21522	0	4.65	1	0.82104	.	.	1
21523	0	4.65	1	0.82104	.	.	1
21524	0	4.65	1	0.82104	.	.	1
21525	0	4.65	1	0.82104	.	.	1

Obs	Sub_group	fuptimey	_CENSOR_	SURVIVAL	SDF_LCL	SDF_UCL	STRATUM
21526	0	4.65	1	0.82104	.	.	1
21527	0	4.65	1	0.82104	.	.	1
21528	0	4.65	1	0.82104	.	.	1
21529	0	4.65	1	0.82104	.	.	1
21530	0	4.65	1	0.82104	.	.	1
21531	0	4.65	1	0.82104	.	.	1
21532	0	4.65	1	0.82104	.	.	1
21533	0	4.65	1	0.82104	.	.	1
21534	0	4.65	1	0.82104	.	.	1
21535	0	4.65	1	0.82104	.	.	1
21536	0	4.65	1	0.82104	.	.	1
21537	0	4.65	1	0.82104	.	.	1
21538	0	4.65	1	0.82104	.	.	1
21539	0	4.65	1	0.82104	.	.	1
21540	0	4.65	1	0.82104	.	.	1
21541	0	4.65	1	0.82104	.	.	1
21542	0	4.65	1	0.82104	.	.	1
21543	0	4.65	1	0.82104	.	.	1
21544	0	4.65	1	0.82104	.	.	1
21545	0	4.65	1	0.82104	.	.	1
21546	0	4.65	1	0.82104	.	.	1
21547	0	4.65	1	0.82104	.	.	1
21548	0	4.65	1	0.82104	.	.	1
21549	0	4.66	0	0.82073	0.81672	0.82466	1
21550	0	4.66	1	0.82073	.	.	1
21551	0	4.66	1	0.82073	.	.	1
21552	0	4.66	1	0.82073	.	.	1
21553	0	4.66	1	0.82073	.	.	1
21554	0	4.66	1	0.82073	.	.	1
21555	0	4.66	1	0.82073	.	.	1
21556	0	4.66	1	0.82073	.	.	1
21557	0	4.66	1	0.82073	.	.	1
21558	0	4.66	1	0.82073	.	.	1
21559	0	4.66	1	0.82073	.	.	1
21560	0	4.66	1	0.82073	.	.	1

Obs	Sub_group	fuptimey	_CENSOR_	SURVIVAL	SDF_LCL	SDF_UCL	STRATUM
21561	0	4.66	1	0.82073	.	.	1
21562	0	4.66	1	0.82073	.	.	1
21563	0	4.66	1	0.82073	.	.	1
21564	0	4.66	1	0.82073	.	.	1
21565	0	4.66	1	0.82073	.	.	1
21566	0	4.66	1	0.82073	.	.	1
21567	0	4.66	1	0.82073	.	.	1
21568	0	4.66	1	0.82073	.	.	1
21569	0	4.66	1	0.82073	.	.	1
21570	0	4.66	1	0.82073	.	.	1
21571	0	4.66	1	0.82073	.	.	1
21572	0	4.66	1	0.82073	.	.	1
21573	0	4.66	1	0.82073	.	.	1
21574	0	4.66	1	0.82073	.	.	1
21575	0	4.66	1	0.82073	.	.	1
21576	0	4.66	1	0.82073	.	.	1
21577	0	4.66	1	0.82073	.	.	1
21578	0	4.66	1	0.82073	.	.	1
21579	0	4.66	1	0.82073	.	.	1
21580	0	4.66	1	0.82073	.	.	1
21581	0	4.66	1	0.82073	.	.	1
21582	0	4.66	1	0.82073	.	.	1
21583	0	4.66	1	0.82073	.	.	1
21584	0	4.66	1	0.82073	.	.	1
21585	0	4.67	0	0.82055	0.81654	0.82449	1
21586	0	4.67	1	0.82055	.	.	1
21587	0	4.67	1	0.82055	.	.	1
21588	0	4.67	1	0.82055	.	.	1
21589	0	4.67	1	0.82055	.	.	1
21590	0	4.67	1	0.82055	.	.	1
21591	0	4.67	1	0.82055	.	.	1
21592	0	4.67	1	0.82055	.	.	1
21593	0	4.67	1	0.82055	.	.	1
21594	0	4.67	1	0.82055	.	.	1
21595	0	4.67	1	0.82055	.	.	1

Obs	Sub_group	fuptimey	_CENSOR_	SURVIVAL	SDF_LCL	SDF_UCL	STRATUM
21596	0	4.67	1	0.82055	.	.	1
21597	0	4.67	1	0.82055	.	.	1
21598	0	4.67	1	0.82055	.	.	1
21599	0	4.67	1	0.82055	.	.	1
21600	0	4.67	1	0.82055	.	.	1
21601	0	4.67	1	0.82055	.	.	1
21602	0	4.67	1	0.82055	.	.	1
21603	0	4.67	1	0.82055	.	.	1
21604	0	4.67	1	0.82055	.	.	1
21605	0	4.67	1	0.82055	.	.	1
21606	0	4.67	1	0.82055	.	.	1
21607	0	4.67	1	0.82055	.	.	1
21608	0	4.67	1	0.82055	.	.	1
21609	0	4.67	1	0.82055	.	.	1
21610	0	4.67	1	0.82055	.	.	1
21611	0	4.67	1	0.82055	.	.	1
21612	0	4.67	1	0.82055	.	.	1
21613	0	4.67	1	0.82055	.	.	1
21614	0	4.67	1	0.82055	.	.	1
21615	0	4.67	1	0.82055	.	.	1
21616	0	4.67	1	0.82055	.	.	1
21617	0	4.67	1	0.82055	.	.	1
21618	0	4.67	1	0.82055	.	.	1
21619	0	4.67	1	0.82055	.	.	1
21620	0	4.67	1	0.82055	.	.	1
21621	0	4.67	1	0.82055	.	.	1
21622	0	4.68	0	0.82038	0.81636	0.82431	1
21623	0	4.68	1	0.82038	.	.	1
21624	0	4.68	1	0.82038	.	.	1
21625	0	4.68	1	0.82038	.	.	1
21626	0	4.68	1	0.82038	.	.	1
21627	0	4.68	1	0.82038	.	.	1
21628	0	4.68	1	0.82038	.	.	1
21629	0	4.68	1	0.82038	.	.	1
21630	0	4.68	1	0.82038	.	.	1

Obs	Sub_group	fuptimey	_CENSOR_	SURVIVAL	SDF_LCL	SDF_UCL	STRATUM
21631	0	4.68	1	0.82038	.	.	1
21632	0	4.68	1	0.82038	.	.	1
21633	0	4.68	1	0.82038	.	.	1
21634	0	4.68	1	0.82038	.	.	1
21635	0	4.68	1	0.82038	.	.	1
21636	0	4.68	1	0.82038	.	.	1
21637	0	4.68	1	0.82038	.	.	1
21638	0	4.68	1	0.82038	.	.	1
21639	0	4.68	1	0.82038	.	.	1
21640	0	4.68	1	0.82038	.	.	1
21641	0	4.68	1	0.82038	.	.	1
21642	0	4.68	1	0.82038	.	.	1
21643	0	4.68	1	0.82038	.	.	1
21644	0	4.68	1	0.82038	.	.	1
21645	0	4.68	1	0.82038	.	.	1
21646	0	4.68	1	0.82038	.	.	1
21647	0	4.68	1	0.82038	.	.	1
21648	0	4.68	1	0.82038	.	.	1
21649	0	4.68	1	0.82038	.	.	1
21650	0	4.68	1	0.82038	.	.	1
21651	0	4.68	1	0.82038	.	.	1
21652	0	4.68	1	0.82038	.	.	1
21653	0	4.68	1	0.82038	.	.	1
21654	0	4.68	1	0.82038	.	.	1
21655	0	4.68	1	0.82038	.	.	1
21656	0	4.68	1	0.82038	.	.	1
21657	0	4.68	1	0.82038	.	.	1
21658	0	4.68	1	0.82038	.	.	1
21659	0	4.69	0	0.82016	0.81614	0.82410	1
21660	0	4.69	1	0.82016	.	.	1
21661	0	4.69	1	0.82016	.	.	1
21662	0	4.69	1	0.82016	.	.	1
21663	0	4.69	1	0.82016	.	.	1
21664	0	4.69	1	0.82016	.	.	1
21665	0	4.69	1	0.82016	.	.	1

Obs	Sub_group	fuptimey	_CENSOR_	SURVIVAL	SDF_LCL	SDF_UCL	STRATUM
21666	0	4.69	1	0.82016	.	.	1
21667	0	4.69	1	0.82016	.	.	1
21668	0	4.69	1	0.82016	.	.	1
21669	0	4.69	1	0.82016	.	.	1
21670	0	4.69	1	0.82016	.	.	1
21671	0	4.69	1	0.82016	.	.	1
21672	0	4.69	1	0.82016	.	.	1
21673	0	4.69	1	0.82016	.	.	1
21674	0	4.69	1	0.82016	.	.	1
21675	0	4.69	1	0.82016	.	.	1
21676	0	4.69	1	0.82016	.	.	1
21677	0	4.69	1	0.82016	.	.	1
21678	0	4.69	1	0.82016	.	.	1
21679	0	4.69	1	0.82016	.	.	1
21680	0	4.69	1	0.82016	.	.	1
21681	0	4.69	1	0.82016	.	.	1
21682	0	4.69	1	0.82016	.	.	1
21683	0	4.69	1	0.82016	.	.	1
21684	0	4.69	1	0.82016	.	.	1
21685	0	4.69	1	0.82016	.	.	1
21686	0	4.69	1	0.82016	.	.	1
21687	0	4.69	1	0.82016	.	.	1
21688	0	4.69	1	0.82016	.	.	1
21689	0	4.69	1	0.82016	.	.	1
21690	0	4.69	1	0.82016	.	.	1
21691	0	4.69	1	0.82016	.	.	1
21692	0	4.69	1	0.82016	.	.	1
21693	0	4.70	0	0.81985	0.81582	0.82379	1
21694	0	4.70	1	0.81985	.	.	1
21695	0	4.70	1	0.81985	.	.	1
21696	0	4.70	1	0.81985	.	.	1
21697	0	4.70	1	0.81985	.	.	1
21698	0	4.70	1	0.81985	.	.	1
21699	0	4.70	1	0.81985	.	.	1
21700	0	4.70	1	0.81985	.	.	1

Obs	Sub_group	fuptimey	_CENSOR_	SURVIVAL	SDF_LCL	SDF_UCL	STRATUM
**21701**	0	4.70	1	0.81985	.	.	1
**21702**	0	4.70	1	0.81985	.	.	1
**21703**	0	4.70	1	0.81985	.	.	1
**21704**	0	4.70	1	0.81985	.	.	1
**21705**	0	4.70	1	0.81985	.	.	1
**21706**	0	4.70	1	0.81985	.	.	1
**21707**	0	4.70	1	0.81985	.	.	1
**21708**	0	4.70	1	0.81985	.	.	1
**21709**	0	4.70	1	0.81985	.	.	1
**21710**	0	4.70	1	0.81985	.	.	1
**21711**	0	4.70	1	0.81985	.	.	1
**21712**	0	4.70	1	0.81985	.	.	1
**21713**	0	4.70	1	0.81985	.	.	1
**21714**	0	4.70	1	0.81985	.	.	1
**21715**	0	4.70	1	0.81985	.	.	1
**21716**	0	4.70	1	0.81985	.	.	1
**21717**	0	4.70	1	0.81985	.	.	1
**21718**	0	4.70	1	0.81985	.	.	1
**21719**	0	4.70	1	0.81985	.	.	1
**21720**	0	4.70	1	0.81985	.	.	1
**21721**	0	4.70	1	0.81985	.	.	1
**21722**	0	4.70	1	0.81985	.	.	1
**21723**	0	4.70	1	0.81985	.	.	1
**21724**	0	4.70	1	0.81985	.	.	1
**21725**	0	4.70	1	0.81985	.	.	1
**21726**	0	4.70	1	0.81985	.	.	1
**21727**	0	4.70	1	0.81985	.	.	1
**21728**	0	4.71	0	0.81967	0.81564	0.82362	1
**21729**	0	4.71	1	0.81967	.	.	1
**21730**	0	4.71	1	0.81967	.	.	1
**21731**	0	4.71	1	0.81967	.	.	1
**21732**	0	4.71	1	0.81967	.	.	1
**21733**	0	4.71	1	0.81967	.	.	1
**21734**	0	4.71	1	0.81967	.	.	1
**21735**	0	4.71	1	0.81967	.	.	1

Obs	Sub_group	fuptimey	_CENSOR_	SURVIVAL	SDF_LCL	SDF_UCL	STRATUM
21736	0	4.71	1	0.81967	.	.	1
21737	0	4.71	1	0.81967	.	.	1
21738	0	4.71	1	0.81967	.	.	1
21739	0	4.71	1	0.81967	.	.	1
21740	0	4.71	1	0.81967	.	.	1
21741	0	4.71	1	0.81967	.	.	1
21742	0	4.71	1	0.81967	.	.	1
21743	0	4.71	1	0.81967	.	.	1
21744	0	4.71	1	0.81967	.	.	1
21745	0	4.71	1	0.81967	.	.	1
21746	0	4.71	1	0.81967	.	.	1
21747	0	4.71	1	0.81967	.	.	1
21748	0	4.71	1	0.81967	.	.	1
21749	0	4.71	1	0.81967	.	.	1
21750	0	4.71	1	0.81967	.	.	1
21751	0	4.71	1	0.81967	.	.	1
21752	0	4.71	1	0.81967	.	.	1
21753	0	4.71	1	0.81967	.	.	1
21754	0	4.71	1	0.81967	.	.	1
21755	0	4.71	1	0.81967	.	.	1
21756	0	4.71	1	0.81967	.	.	1
21757	0	4.71	1	0.81967	.	.	1
21758	0	4.71	1	0.81967	.	.	1
21759	0	4.72	0	0.81945	0.81542	0.82340	1
21760	0	4.72	1	0.81945	.	.	1
21761	0	4.72	1	0.81945	.	.	1
21762	0	4.72	1	0.81945	.	.	1
21763	0	4.72	1	0.81945	.	.	1
21764	0	4.72	1	0.81945	.	.	1
21765	0	4.72	1	0.81945	.	.	1
21766	0	4.72	1	0.81945	.	.	1
21767	0	4.72	1	0.81945	.	.	1
21768	0	4.72	1	0.81945	.	.	1
21769	0	4.72	1	0.81945	.	.	1
21770	0	4.72	1	0.81945	.	.	1

Obs	Sub_group	fuptimey	_CENSOR_	SURVIVAL	SDF_LCL	SDF_UCL	STRATUM
**21771**	0	4.72	1	0.81945	.	.	1
**21772**	0	4.72	1	0.81945	.	.	1
**21773**	0	4.72	1	0.81945	.	.	1
**21774**	0	4.72	1	0.81945	.	.	1
**21775**	0	4.72	1	0.81945	.	.	1
**21776**	0	4.72	1	0.81945	.	.	1
**21777**	0	4.72	1	0.81945	.	.	1
**21778**	0	4.72	1	0.81945	.	.	1
**21779**	0	4.72	1	0.81945	.	.	1
**21780**	0	4.72	1	0.81945	.	.	1
**21781**	0	4.72	1	0.81945	.	.	1
**21782**	0	4.72	1	0.81945	.	.	1
**21783**	0	4.72	1	0.81945	.	.	1
**21784**	0	4.72	1	0.81945	.	.	1
**21785**	0	4.72	1	0.81945	.	.	1
**21786**	0	4.72	1	0.81945	.	.	1
**21787**	0	4.72	1	0.81945	.	.	1
**21788**	0	4.72	1	0.81945	.	.	1
**21789**	0	4.72	1	0.81945	.	.	1
**21790**	0	4.72	1	0.81945	.	.	1
**21791**	0	4.72	1	0.81945	.	.	1
**21792**	0	4.72	1	0.81945	.	.	1
**21793**	0	4.72	1	0.81945	.	.	1
**21794**	0	4.72	1	0.81945	.	.	1
**21795**	0	4.72	1	0.81945	.	.	1
**21796**	0	4.72	1	0.81945	.	.	1
**21797**	0	4.72	1	0.81945	.	.	1
**21798**	0	4.72	1	0.81945	.	.	1
**21799**	0	4.73	0	0.81940	0.81537	0.82336	1
**21800**	0	4.73	1	0.81940	.	.	1
**21801**	0	4.73	1	0.81940	.	.	1
**21802**	0	4.73	1	0.81940	.	.	1
**21803**	0	4.73	1	0.81940	.	.	1
**21804**	0	4.73	1	0.81940	.	.	1
**21805**	0	4.73	1	0.81940	.	.	1

Obs	Sub_group	fuptimey	_CENSOR_	SURVIVAL	SDF_LCL	SDF_UCL	STRATUM
21806	0	4.73	1	0.81940	.	.	1
21807	0	4.73	1	0.81940	.	.	1
21808	0	4.73	1	0.81940	.	.	1
21809	0	4.73	1	0.81940	.	.	1
21810	0	4.73	1	0.81940	.	.	1
21811	0	4.73	1	0.81940	.	.	1
21812	0	4.73	1	0.81940	.	.	1
21813	0	4.73	1	0.81940	.	.	1
21814	0	4.73	1	0.81940	.	.	1
21815	0	4.73	1	0.81940	.	.	1
21816	0	4.73	1	0.81940	.	.	1
21817	0	4.73	1	0.81940	.	.	1
21818	0	4.73	1	0.81940	.	.	1
21819	0	4.73	1	0.81940	.	.	1
21820	0	4.73	1	0.81940	.	.	1
21821	0	4.73	1	0.81940	.	.	1
21822	0	4.74	0	0.81931	0.81528	0.82327	1
21823	0	4.74	1	0.81931	.	.	1
21824	0	4.74	1	0.81931	.	.	1
21825	0	4.74	1	0.81931	.	.	1
21826	0	4.74	1	0.81931	.	.	1
21827	0	4.74	1	0.81931	.	.	1
21828	0	4.74	1	0.81931	.	.	1
21829	0	4.74	1	0.81931	.	.	1
21830	0	4.74	1	0.81931	.	.	1
21831	0	4.74	1	0.81931	.	.	1
21832	0	4.74	1	0.81931	.	.	1
21833	0	4.74	1	0.81931	.	.	1
21834	0	4.74	1	0.81931	.	.	1
21835	0	4.74	1	0.81931	.	.	1
21836	0	4.74	1	0.81931	.	.	1
21837	0	4.74	1	0.81931	.	.	1
21838	0	4.74	1	0.81931	.	.	1
21839	0	4.74	1	0.81931	.	.	1
21840	0	4.74	1	0.81931	.	.	1

Obs	Sub_group	fuptimey	_CENSOR_	SURVIVAL	SDF_LCL	SDF_UCL	STRATUM
21841	0	4.74	1	0.81931	.	.	1
21842	0	4.74	1	0.81931	.	.	1
21843	0	4.74	1	0.81931	.	.	1
21844	0	4.74	1	0.81931	.	.	1
21845	0	4.74	1	0.81931	.	.	1
21846	0	4.74	1	0.81931	.	.	1
21847	0	4.74	1	0.81931	.	.	1
21848	0	4.74	1	0.81931	.	.	1
21849	0	4.74	1	0.81931	.	.	1
21850	0	4.74	1	0.81931	.	.	1
21851	0	4.74	1	0.81931	.	.	1
21852	0	4.74	1	0.81931	.	.	1
21853	0	4.74	1	0.81931	.	.	1
21854	0	4.74	1	0.81931	.	.	1
21855	0	4.74	1	0.81931	.	.	1
21856	0	4.74	1	0.81931	.	.	1
21857	0	4.74	1	0.81931	.	.	1
21858	0	4.74	1	0.81931	.	.	1
21859	0	4.74	1	0.81931	.	.	1
21860	0	4.74	1	0.81931	.	.	1
21861	0	4.74	1	0.81931	.	.	1
21862	0	4.75	0	0.81922	0.81519	0.82318	1
21863	0	4.75	1	0.81922	.	.	1
21864	0	4.75	1	0.81922	.	.	1
21865	0	4.75	1	0.81922	.	.	1
21866	0	4.75	1	0.81922	.	.	1
21867	0	4.75	1	0.81922	.	.	1
21868	0	4.75	1	0.81922	.	.	1
21869	0	4.75	1	0.81922	.	.	1
21870	0	4.75	1	0.81922	.	.	1
21871	0	4.75	1	0.81922	.	.	1
21872	0	4.75	1	0.81922	.	.	1
21873	0	4.75	1	0.81922	.	.	1
21874	0	4.75	1	0.81922	.	.	1
21875	0	4.75	1	0.81922	.	.	1

Obs	Sub_group	fuptimey	_CENSOR_	SURVIVAL	SDF_LCL	SDF_UCL	STRATUM
21876	0	4.75	1	0.81922	.	.	1
21877	0	4.75	1	0.81922	.	.	1
21878	0	4.75	1	0.81922	.	.	1
21879	0	4.75	1	0.81922	.	.	1
21880	0	4.75	1	0.81922	.	.	1
21881	0	4.75	1	0.81922	.	.	1
21882	0	4.75	1	0.81922	.	.	1
21883	0	4.75	1	0.81922	.	.	1
21884	0	4.75	1	0.81922	.	.	1
21885	0	4.75	1	0.81922	.	.	1
21886	0	4.75	1	0.81922	.	.	1
21887	0	4.75	1	0.81922	.	.	1
21888	0	4.75	1	0.81922	.	.	1
21889	0	4.76	0	0.81887	0.81482	0.82283	1
21890	0	4.76	1	0.81887	.	.	1
21891	0	4.76	1	0.81887	.	.	1
21892	0	4.76	1	0.81887	.	.	1
21893	0	4.76	1	0.81887	.	.	1
21894	0	4.76	1	0.81887	.	.	1
21895	0	4.76	1	0.81887	.	.	1
21896	0	4.76	1	0.81887	.	.	1
21897	0	4.76	1	0.81887	.	.	1
21898	0	4.76	1	0.81887	.	.	1
21899	0	4.76	1	0.81887	.	.	1
21900	0	4.76	1	0.81887	.	.	1
21901	0	4.76	1	0.81887	.	.	1
21902	0	4.76	1	0.81887	.	.	1
21903	0	4.76	1	0.81887	.	.	1
21904	0	4.76	1	0.81887	.	.	1
21905	0	4.76	1	0.81887	.	.	1
21906	0	4.76	1	0.81887	.	.	1
21907	0	4.76	1	0.81887	.	.	1
21908	0	4.76	1	0.81887	.	.	1
21909	0	4.76	1	0.81887	.	.	1
21910	0	4.76	1	0.81887	.	.	1

Obs	Sub_group	fuptimey	_CENSOR_	SURVIVAL	SDF_LCL	SDF_UCL	STRATUM
21911	0	4.76	1	0.81887	.	.	1
21912	0	4.76	1	0.81887	.	.	1
21913	0	4.76	1	0.81887	.	.	1
21914	0	4.76	1	0.81887	.	.	1
21915	0	4.76	1	0.81887	.	.	1
21916	0	4.76	1	0.81887	.	.	1
21917	0	4.76	1	0.81887	.	.	1
21918	0	4.76	1	0.81887	.	.	1
21919	0	4.76	1	0.81887	.	.	1
21920	0	4.77	0	0.81864	0.81460	0.82261	1
21921	0	4.77	1	0.81864	.	.	1
21922	0	4.77	1	0.81864	.	.	1
21923	0	4.77	1	0.81864	.	.	1
21924	0	4.77	1	0.81864	.	.	1
21925	0	4.77	1	0.81864	.	.	1
21926	0	4.77	1	0.81864	.	.	1
21927	0	4.77	1	0.81864	.	.	1
21928	0	4.77	1	0.81864	.	.	1
21929	0	4.77	1	0.81864	.	.	1
21930	0	4.77	1	0.81864	.	.	1
21931	0	4.77	1	0.81864	.	.	1
21932	0	4.77	1	0.81864	.	.	1
21933	0	4.77	1	0.81864	.	.	1
21934	0	4.77	1	0.81864	.	.	1
21935	0	4.77	1	0.81864	.	.	1
21936	0	4.77	1	0.81864	.	.	1
21937	0	4.77	1	0.81864	.	.	1
21938	0	4.77	1	0.81864	.	.	1
21939	0	4.77	1	0.81864	.	.	1
21940	0	4.77	1	0.81864	.	.	1
21941	0	4.77	1	0.81864	.	.	1
21942	0	4.77	1	0.81864	.	.	1
21943	0	4.77	1	0.81864	.	.	1
21944	0	4.77	1	0.81864	.	.	1
21945	0	4.77	1	0.81864	.	.	1

Obs	Sub_group	fuptimey	_CENSOR_	SURVIVAL	SDF_LCL	SDF_UCL	STRATUM
21946	0	4.77	1	0.81864	.	.	1
21947	0	4.77	1	0.81864	.	.	1
21948	0	4.77	1	0.81864	.	.	1
21949	0	4.77	1	0.81864	.	.	1
21950	0	4.77	1	0.81864	.	.	1
21951	0	4.77	1	0.81864	.	.	1
21952	0	4.77	1	0.81864	.	.	1
21953	0	4.77	1	0.81864	.	.	1
21954	0	4.78	0	0.81860	0.81455	0.82256	1
21955	0	4.78	1	0.81860	.	.	1
21956	0	4.78	1	0.81860	.	.	1
21957	0	4.78	1	0.81860	.	.	1
21958	0	4.78	1	0.81860	.	.	1
21959	0	4.78	1	0.81860	.	.	1
21960	0	4.78	1	0.81860	.	.	1
21961	0	4.78	1	0.81860	.	.	1
21962	0	4.78	1	0.81860	.	.	1
21963	0	4.78	1	0.81860	.	.	1
21964	0	4.78	1	0.81860	.	.	1
21965	0	4.78	1	0.81860	.	.	1
21966	0	4.78	1	0.81860	.	.	1
21967	0	4.78	1	0.81860	.	.	1
21968	0	4.78	1	0.81860	.	.	1
21969	0	4.78	1	0.81860	.	.	1
21970	0	4.78	1	0.81860	.	.	1
21971	0	4.78	1	0.81860	.	.	1
21972	0	4.78	1	0.81860	.	.	1
21973	0	4.78	1	0.81860	.	.	1
21974	0	4.78	1	0.81860	.	.	1
21975	0	4.78	1	0.81860	.	.	1
21976	0	4.78	1	0.81860	.	.	1
21977	0	4.78	1	0.81860	.	.	1
21978	0	4.78	1	0.81860	.	.	1
21979	0	4.78	1	0.81860	.	.	1
21980	0	4.78	1	0.81860	.	.	1

Obs	Sub_group	fuptimey	_CENSOR_	SURVIVAL	SDF_LCL	SDF_UCL	STRATUM
21981	0	4.78	1	0.81860	.	.	1
21982	0	4.78	1	0.81860	.	.	1
21983	0	4.78	1	0.81860	.	.	1
21984	0	4.78	1	0.81860	.	.	1
21985	0	4.78	1	0.81860	.	.	1
21986	0	4.78	1	0.81860	.	.	1
21987	0	4.78	1	0.81860	.	.	1
21988	0	4.78	1	0.81860	.	.	1
21989	0	4.78	1	0.81860	.	.	1
21990	0	4.78	1	0.81860	.	.	1
21991	0	4.78	1	0.81860	.	.	1
21992	0	4.78	1	0.81860	.	.	1
21993	0	4.78	1	0.81860	.	.	1
21994	0	4.78	1	0.81860	.	.	1
21995	0	4.78	1	0.81860	.	.	1
21996	0	4.79	0	0.81851	0.81446	0.82247	1
21997	0	4.79	1	0.81851	.	.	1
21998	0	4.79	1	0.81851	.	.	1
21999	0	4.79	1	0.81851	.	.	1
22000	0	4.79	1	0.81851	.	.	1
22001	0	4.79	1	0.81851	.	.	1
22002	0	4.79	1	0.81851	.	.	1
22003	0	4.79	1	0.81851	.	.	1
22004	0	4.79	1	0.81851	.	.	1
22005	0	4.79	1	0.81851	.	.	1
22006	0	4.79	1	0.81851	.	.	1
22007	0	4.79	1	0.81851	.	.	1
22008	0	4.79	1	0.81851	.	.	1
22009	0	4.79	1	0.81851	.	.	1
22010	0	4.79	1	0.81851	.	.	1
22011	0	4.79	1	0.81851	.	.	1
22012	0	4.79	1	0.81851	.	.	1
22013	0	4.79	1	0.81851	.	.	1
22014	0	4.79	1	0.81851	.	.	1
22015	0	4.79	1	0.81851	.	.	1

Obs	Sub_group	fuptimey	_CENSOR_	SURVIVAL	SDF_LCL	SDF_UCL	STRATUM
**22016**	0	4.79	1	0.81851	.	.	1
**22017**	0	4.79	1	0.81851	.	.	1
**22018**	0	4.79	1	0.81851	.	.	1
**22019**	0	4.79	1	0.81851	.	.	1
**22020**	0	4.79	1	0.81851	.	.	1
**22021**	0	4.79	1	0.81851	.	.	1
**22022**	0	4.79	1	0.81851	.	.	1
**22023**	0	4.79	1	0.81851	.	.	1
**22024**	0	4.80	0	0.81833	0.81428	0.82230	1
**22025**	0	4.80	1	0.81833	.	.	1
**22026**	0	4.80	1	0.81833	.	.	1
**22027**	0	4.80	1	0.81833	.	.	1
**22028**	0	4.80	1	0.81833	.	.	1
**22029**	0	4.80	1	0.81833	.	.	1
**22030**	0	4.80	1	0.81833	.	.	1
**22031**	0	4.80	1	0.81833	.	.	1
**22032**	0	4.80	1	0.81833	.	.	1
**22033**	0	4.80	1	0.81833	.	.	1
**22034**	0	4.80	1	0.81833	.	.	1
**22035**	0	4.80	1	0.81833	.	.	1
**22036**	0	4.80	1	0.81833	.	.	1
**22037**	0	4.80	1	0.81833	.	.	1
**22038**	0	4.80	1	0.81833	.	.	1
**22039**	0	4.80	1	0.81833	.	.	1
**22040**	0	4.80	1	0.81833	.	.	1
**22041**	0	4.80	1	0.81833	.	.	1
**22042**	0	4.80	1	0.81833	.	.	1
**22043**	0	4.80	1	0.81833	.	.	1
**22044**	0	4.80	1	0.81833	.	.	1
**22045**	0	4.80	1	0.81833	.	.	1
**22046**	0	4.80	1	0.81833	.	.	1
**22047**	0	4.80	1	0.81833	.	.	1
**22048**	0	4.80	1	0.81833	.	.	1
**22049**	0	4.80	1	0.81833	.	.	1
**22050**	0	4.80	1	0.81833	.	.	1

Obs	Sub_group	fuptimey	_CENSOR_	SURVIVAL	SDF_LCL	SDF_UCL	STRATUM
22051	0	4.80	1	0.81833	.	.	1
22052	0	4.80	1	0.81833	.	.	1
22053	0	4.80	1	0.81833	.	.	1
22054	0	4.80	1	0.81833	.	.	1
22055	0	4.80	1	0.81833	.	.	1
22056	0	4.80	1	0.81833	.	.	1
22057	0	4.80	1	0.81833	.	.	1
22058	0	4.80	1	0.81833	.	.	1
22059	0	4.80	1	0.81833	.	.	1
22060	0	4.80	1	0.81833	.	.	1
22061	0	4.80	1	0.81833	.	.	1
22062	0	4.80	1	0.81833	.	.	1
22063	0	4.80	1	0.81833	.	.	1
22064	0	4.80	1	0.81833	.	.	1
22065	0	4.80	1	0.81833	.	.	1
22066	0	4.80	1	0.81833	.	.	1
22067	0	4.80	1	0.81833	.	.	1
22068	0	4.80	1	0.81833	.	.	1
22069	0	4.80	1	0.81833	.	.	1
22070	0	4.80	1	0.81833	.	.	1
22071	0	4.80	1	0.81833	.	.	1
22072	0	4.80	1	0.81833	.	.	1
22073	0	4.80	1	0.81833	.	.	1
22074	0	4.80	1	0.81833	.	.	1
22075	0	4.80	1	0.81833	.	.	1
22076	0	4.80	1	0.81833	.	.	1
22077	0	4.80	1	0.81833	.	.	1
22078	0	4.80	1	0.81833	.	.	1
22079	0	4.80	1	0.81833	.	.	1
22080	0	4.80	1	0.81833	.	.	1
22081	0	4.80	1	0.81833	.	.	1
22082	0	4.80	1	0.81833	.	.	1
22083	0	4.81	0	0.81819	0.81414	0.82216	1
22084	0	4.81	1	0.81819	.	.	1
22085	0	4.81	1	0.81819	.	.	1

Obs	Sub_group	fuptimey	_CENSOR_	SURVIVAL	SDF_LCL	SDF_UCL	STRATUM
22086	0	4.81	1	0.81819	.	.	1
22087	0	4.81	1	0.81819	.	.	1
22088	0	4.81	1	0.81819	.	.	1
22089	0	4.81	1	0.81819	.	.	1
22090	0	4.81	1	0.81819	.	.	1
22091	0	4.81	1	0.81819	.	.	1
22092	0	4.81	1	0.81819	.	.	1
22093	0	4.81	1	0.81819	.	.	1
22094	0	4.81	1	0.81819	.	.	1
22095	0	4.81	1	0.81819	.	.	1
22096	0	4.81	1	0.81819	.	.	1
22097	0	4.81	1	0.81819	.	.	1
22098	0	4.81	1	0.81819	.	.	1
22099	0	4.81	1	0.81819	.	.	1
22100	0	4.81	1	0.81819	.	.	1
22101	0	4.81	1	0.81819	.	.	1
22102	0	4.81	1	0.81819	.	.	1
22103	0	4.81	1	0.81819	.	.	1
22104	0	4.81	1	0.81819	.	.	1
22105	0	4.81	1	0.81819	.	.	1
22106	0	4.81	1	0.81819	.	.	1
22107	0	4.81	1	0.81819	.	.	1
22108	0	4.81	1	0.81819	.	.	1
22109	0	4.81	1	0.81819	.	.	1
22110	0	4.81	1	0.81819	.	.	1
22111	0	4.81	1	0.81819	.	.	1
22112	0	4.81	1	0.81819	.	.	1
22113	0	4.82	0	0.81792	0.81386	0.82190	1
22114	0	4.82	1	0.81792	.	.	1
22115	0	4.82	1	0.81792	.	.	1
22116	0	4.82	1	0.81792	.	.	1
22117	0	4.82	1	0.81792	.	.	1
22118	0	4.82	1	0.81792	.	.	1
22119	0	4.82	1	0.81792	.	.	1
22120	0	4.82	1	0.81792	.	.	1

Obs	Sub_group	fuptimey	_CENSOR_	SURVIVAL	SDF_LCL	SDF_UCL	STRATUM
22121	0	4.82	1	0.81792	.	.	1
22122	0	4.82	1	0.81792	.	.	1
22123	0	4.82	1	0.81792	.	.	1
22124	0	4.82	1	0.81792	.	.	1
22125	0	4.82	1	0.81792	.	.	1
22126	0	4.82	1	0.81792	.	.	1
22127	0	4.82	1	0.81792	.	.	1
22128	0	4.82	1	0.81792	.	.	1
22129	0	4.82	1	0.81792	.	.	1
22130	0	4.82	1	0.81792	.	.	1
22131	0	4.82	1	0.81792	.	.	1
22132	0	4.82	1	0.81792	.	.	1
22133	0	4.82	1	0.81792	.	.	1
22134	0	4.82	1	0.81792	.	.	1
22135	0	4.82	1	0.81792	.	.	1
22136	0	4.82	1	0.81792	.	.	1
22137	0	4.82	1	0.81792	.	.	1
22138	0	4.82	1	0.81792	.	.	1
22139	0	4.82	1	0.81792	.	.	1
22140	0	4.82	1	0.81792	.	.	1
22141	0	4.82	1	0.81792	.	.	1
22142	0	4.82	1	0.81792	.	.	1
22143	0	4.82	1	0.81792	.	.	1
22144	0	4.82	1	0.81792	.	.	1
22145	0	4.82	1	0.81792	.	.	1
22146	0	4.83	0	0.81769	0.81363	0.82167	1
22147	0	4.83	1	0.81769	.	.	1
22148	0	4.83	1	0.81769	.	.	1
22149	0	4.83	1	0.81769	.	.	1
22150	0	4.83	1	0.81769	.	.	1
22151	0	4.83	1	0.81769	.	.	1
22152	0	4.83	1	0.81769	.	.	1
22153	0	4.83	1	0.81769	.	.	1
22154	0	4.83	1	0.81769	.	.	1
22155	0	4.83	1	0.81769	.	.	1

Obs	Sub_group	fuptimey	_CENSOR_	SURVIVAL	SDF_LCL	SDF_UCL	STRATUM
22156	0	4.83	1	0.81769	.	.	1
22157	0	4.83	1	0.81769	.	.	1
22158	0	4.83	1	0.81769	.	.	1
22159	0	4.83	1	0.81769	.	.	1
22160	0	4.83	1	0.81769	.	.	1
22161	0	4.83	1	0.81769	.	.	1
22162	0	4.83	1	0.81769	.	.	1
22163	0	4.83	1	0.81769	.	.	1
22164	0	4.83	1	0.81769	.	.	1
22165	0	4.83	1	0.81769	.	.	1
22166	0	4.83	1	0.81769	.	.	1
22167	0	4.83	1	0.81769	.	.	1
22168	0	4.83	1	0.81769	.	.	1
22169	0	4.83	1	0.81769	.	.	1
22170	0	4.83	1	0.81769	.	.	1
22171	0	4.83	1	0.81769	.	.	1
22172	0	4.83	1	0.81769	.	.	1
22173	0	4.83	1	0.81769	.	.	1
22174	0	4.83	1	0.81769	.	.	1
22175	0	4.83	1	0.81769	.	.	1
22176	0	4.83	1	0.81769	.	.	1
22177	0	4.83	1	0.81769	.	.	1
22178	0	4.83	1	0.81769	.	.	1
22179	0	4.83	1	0.81769	.	.	1
22180	0	4.83	1	0.81769	.	.	1
22181	0	4.83	1	0.81769	.	.	1
22182	0	4.83	1	0.81769	.	.	1
22183	0	4.83	1	0.81769	.	.	1
22184	0	4.83	1	0.81769	.	.	1
22185	0	4.83	1	0.81769	.	.	1
22186	0	4.84	0	0.81756	0.81349	0.82154	1
22187	0	4.84	1	0.81756	.	.	1
22188	0	4.84	1	0.81756	.	.	1
22189	0	4.84	1	0.81756	.	.	1
22190	0	4.84	1	0.81756	.	.	1

Obs	Sub_group	fuptimey	_CENSOR_	SURVIVAL	SDF_LCL	SDF_UCL	STRATUM
22191	0	4.84	1	0.81756	.	.	1
22192	0	4.84	1	0.81756	.	.	1
22193	0	4.84	1	0.81756	.	.	1
22194	0	4.84	1	0.81756	.	.	1
22195	0	4.84	1	0.81756	.	.	1
22196	0	4.84	1	0.81756	.	.	1
22197	0	4.84	1	0.81756	.	.	1
22198	0	4.84	1	0.81756	.	.	1
22199	0	4.84	1	0.81756	.	.	1
22200	0	4.84	1	0.81756	.	.	1
22201	0	4.84	1	0.81756	.	.	1
22202	0	4.84	1	0.81756	.	.	1
22203	0	4.84	1	0.81756	.	.	1
22204	0	4.84	1	0.81756	.	.	1
22205	0	4.84	1	0.81756	.	.	1
22206	0	4.84	1	0.81756	.	.	1
22207	0	4.84	1	0.81756	.	.	1
22208	0	4.84	1	0.81756	.	.	1
22209	0	4.84	1	0.81756	.	.	1
22210	0	4.84	1	0.81756	.	.	1
22211	0	4.84	1	0.81756	.	.	1
22212	0	4.84	1	0.81756	.	.	1
22213	0	4.84	1	0.81756	.	.	1
22214	0	4.84	1	0.81756	.	.	1
22215	0	4.84	1	0.81756	.	.	1
22216	0	4.84	1	0.81756	.	.	1
22217	0	4.84	1	0.81756	.	.	1
22218	0	4.84	1	0.81756	.	.	1
22219	0	4.84	1	0.81756	.	.	1
22220	0	4.84	1	0.81756	.	.	1
22221	0	4.84	1	0.81756	.	.	1
22222	0	4.84	1	0.81756	.	.	1
22223	0	4.84	1	0.81756	.	.	1
22224	0	4.84	1	0.81756	.	.	1
22225	0	4.84	1	0.81756	.	.	1

Obs	Sub_group	fuptimey	_CENSOR_	SURVIVAL	SDF_LCL	SDF_UCL	STRATUM
22226	0	4.84	1	0.81756	.	.	1
22227	0	4.84	1	0.81756	.	.	1
22228	0	4.85	0	0.81742	0.81335	0.82141	1
22229	0	4.85	1	0.81742	.	.	1
22230	0	4.85	1	0.81742	.	.	1
22231	0	4.85	1	0.81742	.	.	1
22232	0	4.85	1	0.81742	.	.	1
22233	0	4.85	1	0.81742	.	.	1
22234	0	4.85	1	0.81742	.	.	1
22235	0	4.85	1	0.81742	.	.	1
22236	0	4.85	1	0.81742	.	.	1
22237	0	4.85	1	0.81742	.	.	1
22238	0	4.85	1	0.81742	.	.	1
22239	0	4.85	1	0.81742	.	.	1
22240	0	4.85	1	0.81742	.	.	1
22241	0	4.85	1	0.81742	.	.	1
22242	0	4.85	1	0.81742	.	.	1
22243	0	4.85	1	0.81742	.	.	1
22244	0	4.85	1	0.81742	.	.	1
22245	0	4.85	1	0.81742	.	.	1
22246	0	4.85	1	0.81742	.	.	1
22247	0	4.85	1	0.81742	.	.	1
22248	0	4.85	1	0.81742	.	.	1
22249	0	4.85	1	0.81742	.	.	1
22250	0	4.85	1	0.81742	.	.	1
22251	0	4.85	1	0.81742	.	.	1
22252	0	4.85	1	0.81742	.	.	1
22253	0	4.85	1	0.81742	.	.	1
22254	0	4.85	1	0.81742	.	.	1
22255	0	4.85	1	0.81742	.	.	1
22256	0	4.85	1	0.81742	.	.	1
22257	0	4.85	1	0.81742	.	.	1
22258	0	4.85	1	0.81742	.	.	1
22259	0	4.85	1	0.81742	.	.	1
22260	0	4.85	1	0.81742	.	.	1

Obs	Sub_group	fuptimey	_CENSOR_	SURVIVAL	SDF_LCL	SDF_UCL	STRATUM
**22261**	0	4.86	0	0.81715	0.81308	0.82114	1
**22262**	0	4.86	1	0.81715	.	.	1
**22263**	0	4.86	1	0.81715	.	.	1
**22264**	0	4.86	1	0.81715	.	.	1
**22265**	0	4.86	1	0.81715	.	.	1
**22266**	0	4.86	1	0.81715	.	.	1
**22267**	0	4.86	1	0.81715	.	.	1
**22268**	0	4.86	1	0.81715	.	.	1
**22269**	0	4.86	1	0.81715	.	.	1
**22270**	0	4.86	1	0.81715	.	.	1
**22271**	0	4.86	1	0.81715	.	.	1
**22272**	0	4.86	1	0.81715	.	.	1
**22273**	0	4.86	1	0.81715	.	.	1
**22274**	0	4.86	1	0.81715	.	.	1
**22275**	0	4.86	1	0.81715	.	.	1
**22276**	0	4.86	1	0.81715	.	.	1
**22277**	0	4.86	1	0.81715	.	.	1
**22278**	0	4.86	1	0.81715	.	.	1
**22279**	0	4.86	1	0.81715	.	.	1
**22280**	0	4.86	1	0.81715	.	.	1
**22281**	0	4.86	1	0.81715	.	.	1
**22282**	0	4.86	1	0.81715	.	.	1
**22283**	0	4.86	1	0.81715	.	.	1
**22284**	0	4.86	1	0.81715	.	.	1
**22285**	0	4.86	1	0.81715	.	.	1
**22286**	0	4.86	1	0.81715	.	.	1
**22287**	0	4.86	1	0.81715	.	.	1
**22288**	0	4.87	0	0.81687	0.81280	0.82087	1
**22289**	0	4.87	1	0.81687	.	.	1
**22290**	0	4.87	1	0.81687	.	.	1
**22291**	0	4.87	1	0.81687	.	.	1
**22292**	0	4.87	1	0.81687	.	.	1
**22293**	0	4.87	1	0.81687	.	.	1
**22294**	0	4.87	1	0.81687	.	.	1
**22295**	0	4.87	1	0.81687	.	.	1

Obs	Sub_group	fuptimey	_CENSOR_	SURVIVAL	SDF_LCL	SDF_UCL	STRATUM
22296	0	4.87	1	0.81687	.	.	1
22297	0	4.87	1	0.81687	.	.	1
22298	0	4.87	1	0.81687	.	.	1
22299	0	4.87	1	0.81687	.	.	1
22300	0	4.87	1	0.81687	.	.	1
22301	0	4.87	1	0.81687	.	.	1
22302	0	4.87	1	0.81687	.	.	1
22303	0	4.87	1	0.81687	.	.	1
22304	0	4.87	1	0.81687	.	.	1
22305	0	4.87	1	0.81687	.	.	1
22306	0	4.87	1	0.81687	.	.	1
22307	0	4.87	1	0.81687	.	.	1
22308	0	4.87	1	0.81687	.	.	1
22309	0	4.87	1	0.81687	.	.	1
22310	0	4.87	1	0.81687	.	.	1
22311	0	4.87	1	0.81687	.	.	1
22312	0	4.87	1	0.81687	.	.	1
22313	0	4.87	1	0.81687	.	.	1
22314	0	4.87	1	0.81687	.	.	1
22315	0	4.87	1	0.81687	.	.	1
22316	0	4.87	1	0.81687	.	.	1
22317	0	4.87	1	0.81687	.	.	1
22318	0	4.87	1	0.81687	.	.	1
22319	0	4.87	1	0.81687	.	.	1
22320	0	4.87	1	0.81687	.	.	1
22321	0	4.87	1	0.81687	.	.	1
22322	0	4.87	1	0.81687	.	.	1
22323	0	4.87	1	0.81687	.	.	1
22324	0	4.87	1	0.81687	.	.	1
22325	0	4.87	1	0.81687	.	.	1
22326	0	4.87	1	0.81687	.	.	1
22327	0	4.87	1	0.81687	.	.	1
22328	0	4.87	1	0.81687	.	.	1
22329	0	4.88	0	0.81669	0.81261	0.82069	1
22330	0	4.88	1	0.81669	.	.	1

Obs	Sub_group	fuptimey	_CENSOR_	SURVIVAL	SDF_LCL	SDF_UCL	STRATUM
22331	0	4.88	1	0.81669	.	.	1
22332	0	4.88	1	0.81669	.	.	1
22333	0	4.88	1	0.81669	.	.	1
22334	0	4.88	1	0.81669	.	.	1
22335	0	4.88	1	0.81669	.	.	1
22336	0	4.88	1	0.81669	.	.	1
22337	0	4.88	1	0.81669	.	.	1
22338	0	4.88	1	0.81669	.	.	1
22339	0	4.88	1	0.81669	.	.	1
22340	0	4.88	1	0.81669	.	.	1
22341	0	4.88	1	0.81669	.	.	1
22342	0	4.88	1	0.81669	.	.	1
22343	0	4.88	1	0.81669	.	.	1
22344	0	4.88	1	0.81669	.	.	1
22345	0	4.88	1	0.81669	.	.	1
22346	0	4.88	1	0.81669	.	.	1
22347	0	4.88	1	0.81669	.	.	1
22348	0	4.88	1	0.81669	.	.	1
22349	0	4.88	1	0.81669	.	.	1
22350	0	4.88	1	0.81669	.	.	1
22351	0	4.88	1	0.81669	.	.	1
22352	0	4.88	1	0.81669	.	.	1
22353	0	4.88	1	0.81669	.	.	1
22354	0	4.88	1	0.81669	.	.	1
22355	0	4.88	1	0.81669	.	.	1
22356	0	4.88	1	0.81669	.	.	1
22357	0	4.88	1	0.81669	.	.	1
22358	0	4.88	1	0.81669	.	.	1
22359	0	4.88	1	0.81669	.	.	1
22360	0	4.88	1	0.81669	.	.	1
22361	0	4.88	1	0.81669	.	.	1
22362	0	4.89	1	0.81669	.	.	1
22363	0	4.89	1	0.81669	.	.	1
22364	0	4.89	1	0.81669	.	.	1
22365	0	4.89	1	0.81669	.	.	1

Obs	Sub_group	fuptimey	_CENSOR_	SURVIVAL	SDF_LCL	SDF_UCL	STRATUM
22366	0	4.89	1	0.81669	.	.	1
22367	0	4.89	1	0.81669	.	.	1
22368	0	4.89	1	0.81669	.	.	1
22369	0	4.89	1	0.81669	.	.	1
22370	0	4.89	1	0.81669	.	.	1
22371	0	4.89	1	0.81669	.	.	1
22372	0	4.89	1	0.81669	.	.	1
22373	0	4.89	1	0.81669	.	.	1
22374	0	4.89	1	0.81669	.	.	1
22375	0	4.89	1	0.81669	.	.	1
22376	0	4.89	1	0.81669	.	.	1
22377	0	4.89	1	0.81669	.	.	1
22378	0	4.89	1	0.81669	.	.	1
22379	0	4.89	1	0.81669	.	.	1
22380	0	4.89	1	0.81669	.	.	1
22381	0	4.89	1	0.81669	.	.	1
22382	0	4.89	1	0.81669	.	.	1
22383	0	4.89	1	0.81669	.	.	1
22384	0	4.89	1	0.81669	.	.	1
22385	0	4.89	1	0.81669	.	.	1
22386	0	4.89	1	0.81669	.	.	1
22387	0	4.89	1	0.81669	.	.	1
22388	0	4.89	1	0.81669	.	.	1
22389	0	4.89	1	0.81669	.	.	1
22390	0	4.89	1	0.81669	.	.	1
22391	0	4.89	1	0.81669	.	.	1
22392	0	4.89	1	0.81669	.	.	1
22393	0	4.89	1	0.81669	.	.	1
22394	0	4.89	1	0.81669	.	.	1
22395	0	4.89	1	0.81669	.	.	1
22396	0	4.90	0	0.81660	0.81252	0.82060	1
22397	0	4.90	1	0.81660	.	.	1
22398	0	4.90	1	0.81660	.	.	1
22399	0	4.90	1	0.81660	.	.	1
22400	0	4.90	1	0.81660	.	.	1

Obs	Sub_group	fuptimey	_CENSOR_	SURVIVAL	SDF_LCL	SDF_UCL	STRATUM
22401	0	4.90	1	0.81660	.	.	1
22402	0	4.90	1	0.81660	.	.	1
22403	0	4.90	1	0.81660	.	.	1
22404	0	4.90	1	0.81660	.	.	1
22405	0	4.90	1	0.81660	.	.	1
22406	0	4.90	1	0.81660	.	.	1
22407	0	4.90	1	0.81660	.	.	1
22408	0	4.90	1	0.81660	.	.	1
22409	0	4.90	1	0.81660	.	.	1
22410	0	4.90	1	0.81660	.	.	1
22411	0	4.90	1	0.81660	.	.	1
22412	0	4.90	1	0.81660	.	.	1
22413	0	4.90	1	0.81660	.	.	1
22414	0	4.90	1	0.81660	.	.	1
22415	0	4.90	1	0.81660	.	.	1
22416	0	4.90	1	0.81660	.	.	1
22417	0	4.90	1	0.81660	.	.	1
22418	0	4.90	1	0.81660	.	.	1
22419	0	4.90	1	0.81660	.	.	1
22420	0	4.90	1	0.81660	.	.	1
22421	0	4.90	1	0.81660	.	.	1
22422	0	4.90	1	0.81660	.	.	1
22423	0	4.90	1	0.81660	.	.	1
22424	0	4.90	1	0.81660	.	.	1
22425	0	4.90	1	0.81660	.	.	1
22426	0	4.90	1	0.81660	.	.	1
22427	0	4.90	1	0.81660	.	.	1
22428	0	4.91	0	0.81646	0.81238	0.82046	1
22429	0	4.91	1	0.81646	.	.	1
22430	0	4.91	1	0.81646	.	.	1
22431	0	4.91	1	0.81646	.	.	1
22432	0	4.91	1	0.81646	.	.	1
22433	0	4.91	1	0.81646	.	.	1
22434	0	4.91	1	0.81646	.	.	1
22435	0	4.91	1	0.81646	.	.	1

Obs	Sub_group	fuptimey	_CENSOR_	SURVIVAL	SDF_LCL	SDF_UCL	STRATUM
22436	0	4.91	1	0.81646	.	.	1
22437	0	4.91	1	0.81646	.	.	1
22438	0	4.91	1	0.81646	.	.	1
22439	0	4.91	1	0.81646	.	.	1
22440	0	4.91	1	0.81646	.	.	1
22441	0	4.91	1	0.81646	.	.	1
22442	0	4.91	1	0.81646	.	.	1
22443	0	4.91	1	0.81646	.	.	1
22444	0	4.91	1	0.81646	.	.	1
22445	0	4.91	1	0.81646	.	.	1
22446	0	4.91	1	0.81646	.	.	1
22447	0	4.91	1	0.81646	.	.	1
22448	0	4.91	1	0.81646	.	.	1
22449	0	4.91	1	0.81646	.	.	1
22450	0	4.91	1	0.81646	.	.	1
22451	0	4.91	1	0.81646	.	.	1
22452	0	4.91	1	0.81646	.	.	1
22453	0	4.91	1	0.81646	.	.	1
22454	0	4.91	1	0.81646	.	.	1
22455	0	4.91	1	0.81646	.	.	1
22456	0	4.91	1	0.81646	.	.	1
22457	0	4.91	1	0.81646	.	.	1
22458	0	4.91	1	0.81646	.	.	1
22459	0	4.91	1	0.81646	.	.	1
22460	0	4.91	1	0.81646	.	.	1
22461	0	4.91	1	0.81646	.	.	1
22462	0	4.91	1	0.81646	.	.	1
22463	0	4.91	1	0.81646	.	.	1
22464	0	4.91	1	0.81646	.	.	1
22465	0	4.91	1	0.81646	.	.	1
22466	0	4.91	1	0.81646	.	.	1
22467	0	4.91	1	0.81646	.	.	1
22468	0	4.91	1	0.81646	.	.	1
22469	0	4.91	1	0.81646	.	.	1
22470	0	4.92	0	0.81641	0.81233	0.82042	1

Obs	Sub_group	fuptimey	_CENSOR_	SURVIVAL	SDF_LCL	SDF_UCL	STRATUM
22471	0	4.92	1	0.81641	.	.	1
22472	0	4.92	1	0.81641	.	.	1
22473	0	4.92	1	0.81641	.	.	1
22474	0	4.92	1	0.81641	.	.	1
22475	0	4.92	1	0.81641	.	.	1
22476	0	4.92	1	0.81641	.	.	1
22477	0	4.92	1	0.81641	.	.	1
22478	0	4.92	1	0.81641	.	.	1
22479	0	4.92	1	0.81641	.	.	1
22480	0	4.92	1	0.81641	.	.	1
22481	0	4.92	1	0.81641	.	.	1
22482	0	4.92	1	0.81641	.	.	1
22483	0	4.92	1	0.81641	.	.	1
22484	0	4.92	1	0.81641	.	.	1
22485	0	4.92	1	0.81641	.	.	1
22486	0	4.92	1	0.81641	.	.	1
22487	0	4.92	1	0.81641	.	.	1
22488	0	4.92	1	0.81641	.	.	1
22489	0	4.92	1	0.81641	.	.	1
22490	0	4.92	1	0.81641	.	.	1
22491	0	4.92	1	0.81641	.	.	1
22492	0	4.92	1	0.81641	.	.	1
22493	0	4.92	1	0.81641	.	.	1
22494	0	4.92	1	0.81641	.	.	1
22495	0	4.92	1	0.81641	.	.	1
22496	0	4.92	1	0.81641	.	.	1
22497	0	4.92	1	0.81641	.	.	1
22498	0	4.92	1	0.81641	.	.	1
22499	0	4.92	1	0.81641	.	.	1
22500	0	4.93	0	0.81604	0.81195	0.82005	1
22501	0	4.93	1	0.81604	.	.	1
22502	0	4.93	1	0.81604	.	.	1
22503	0	4.93	1	0.81604	.	.	1
22504	0	4.93	1	0.81604	.	.	1
22505	0	4.93	1	0.81604	.	.	1

Obs	Sub_group	fuptimey	_CENSOR_	SURVIVAL	SDF_LCL	SDF_UCL	STRATUM
22506	0	4.93	1	0.81604	.	.	1
22507	0	4.93	1	0.81604	.	.	1
22508	0	4.93	1	0.81604	.	.	1
22509	0	4.93	1	0.81604	.	.	1
22510	0	4.93	1	0.81604	.	.	1
22511	0	4.93	1	0.81604	.	.	1
22512	0	4.93	1	0.81604	.	.	1
22513	0	4.93	1	0.81604	.	.	1
22514	0	4.93	1	0.81604	.	.	1
22515	0	4.93	1	0.81604	.	.	1
22516	0	4.93	1	0.81604	.	.	1
22517	0	4.93	1	0.81604	.	.	1
22518	0	4.93	1	0.81604	.	.	1
22519	0	4.93	1	0.81604	.	.	1
22520	0	4.93	1	0.81604	.	.	1
22521	0	4.93	1	0.81604	.	.	1
22522	0	4.93	1	0.81604	.	.	1
22523	0	4.93	1	0.81604	.	.	1
22524	0	4.93	1	0.81604	.	.	1
22525	0	4.93	1	0.81604	.	.	1
22526	0	4.93	1	0.81604	.	.	1
22527	0	4.93	1	0.81604	.	.	1
22528	0	4.93	1	0.81604	.	.	1
22529	0	4.93	1	0.81604	.	.	1
22530	0	4.93	1	0.81604	.	.	1
22531	0	4.93	1	0.81604	.	.	1
22532	0	4.93	1	0.81604	.	.	1
22533	0	4.93	1	0.81604	.	.	1
22534	0	4.93	1	0.81604	.	.	1
22535	0	4.93	1	0.81604	.	.	1
22536	0	4.93	1	0.81604	.	.	1
22537	0	4.93	1	0.81604	.	.	1
22538	0	4.94	0	0.81576	0.81167	0.81978	1
22539	0	4.94	1	0.81576	.	.	1
22540	0	4.94	1	0.81576	.	.	1

Obs	Sub_group	fuptimey	_CENSOR_	SURVIVAL	SDF_LCL	SDF_UCL	STRATUM
22541	0	4.94	1	0.81576	.	.	1
22542	0	4.94	1	0.81576	.	.	1
22543	0	4.94	1	0.81576	.	.	1
22544	0	4.94	1	0.81576	.	.	1
22545	0	4.94	1	0.81576	.	.	1
22546	0	4.94	1	0.81576	.	.	1
22547	0	4.94	1	0.81576	.	.	1
22548	0	4.94	1	0.81576	.	.	1
22549	0	4.94	1	0.81576	.	.	1
22550	0	4.94	1	0.81576	.	.	1
22551	0	4.94	1	0.81576	.	.	1
22552	0	4.94	1	0.81576	.	.	1
22553	0	4.94	1	0.81576	.	.	1
22554	0	4.94	1	0.81576	.	.	1
22555	0	4.94	1	0.81576	.	.	1
22556	0	4.94	1	0.81576	.	.	1
22557	0	4.94	1	0.81576	.	.	1
22558	0	4.94	1	0.81576	.	.	1
22559	0	4.94	1	0.81576	.	.	1
22560	0	4.94	1	0.81576	.	.	1
22561	0	4.94	1	0.81576	.	.	1
22562	0	4.94	1	0.81576	.	.	1
22563	0	4.94	1	0.81576	.	.	1
22564	0	4.94	1	0.81576	.	.	1
22565	0	4.94	1	0.81576	.	.	1
22566	0	4.94	1	0.81576	.	.	1
22567	0	4.94	1	0.81576	.	.	1
22568	0	4.94	1	0.81576	.	.	1
22569	0	4.94	1	0.81576	.	.	1
22570	0	4.94	1	0.81576	.	.	1
22571	0	4.94	1	0.81576	.	.	1
22572	0	4.94	1	0.81576	.	.	1
22573	0	4.94	1	0.81576	.	.	1
22574	0	4.94	1	0.81576	.	.	1
22575	0	4.94	1	0.81576	.	.	1

Obs	Sub_group	fuptimey	_CENSOR_	SURVIVAL	SDF_LCL	SDF_UCL	STRATUM
22576	0	4.94	1	0.81576	.	.	1
22577	0	4.94	1	0.81576	.	.	1
22578	0	4.94	1	0.81576	.	.	1
22579	0	4.94	1	0.81576	.	.	1
22580	0	4.94	1	0.81576	.	.	1
22581	0	4.94	1	0.81576	.	.	1
22582	0	4.94	1	0.81576	.	.	1
22583	0	4.94	1	0.81576	.	.	1
22584	0	4.94	1	0.81576	.	.	1
22585	0	4.95	0	0.81521	0.81110	0.81923	1
22586	0	4.95	1	0.81521	.	.	1
22587	0	4.95	1	0.81521	.	.	1
22588	0	4.95	1	0.81521	.	.	1
22589	0	4.95	1	0.81521	.	.	1
22590	0	4.95	1	0.81521	.	.	1
22591	0	4.95	1	0.81521	.	.	1
22592	0	4.95	1	0.81521	.	.	1
22593	0	4.95	1	0.81521	.	.	1
22594	0	4.95	1	0.81521	.	.	1
22595	0	4.95	1	0.81521	.	.	1
22596	0	4.95	1	0.81521	.	.	1
22597	0	4.95	1	0.81521	.	.	1
22598	0	4.95	1	0.81521	.	.	1
22599	0	4.95	1	0.81521	.	.	1
22600	0	4.95	1	0.81521	.	.	1
22601	0	4.95	1	0.81521	.	.	1
22602	0	4.95	1	0.81521	.	.	1
22603	0	4.95	1	0.81521	.	.	1
22604	0	4.95	1	0.81521	.	.	1
22605	0	4.95	1	0.81521	.	.	1
22606	0	4.95	1	0.81521	.	.	1
22607	0	4.95	1	0.81521	.	.	1
22608	0	4.95	1	0.81521	.	.	1
22609	0	4.95	1	0.81521	.	.	1
22610	0	4.95	1	0.81521	.	.	1

Obs	Sub_group	fuptimey	_CENSOR_	SURVIVAL	SDF_LCL	SDF_UCL	STRATUM
22611	0	4.95	1	0.81521	.	.	1
22612	0	4.95	1	0.81521	.	.	1
22613	0	4.95	1	0.81521	.	.	1
22614	0	4.95	1	0.81521	.	.	1
22615	0	4.95	1	0.81521	.	.	1
22616	0	4.95	1	0.81521	.	.	1
22617	0	4.95	1	0.81521	.	.	1
22618	0	4.95	1	0.81521	.	.	1
22619	0	4.95	1	0.81521	.	.	1
22620	0	4.95	1	0.81521	.	.	1
22621	0	4.95	1	0.81521	.	.	1
22622	0	4.95	1	0.81521	.	.	1
22623	0	4.95	1	0.81521	.	.	1
22624	0	4.96	0	0.81507	0.81096	0.81909	1
22625	0	4.96	1	0.81507	.	.	1
22626	0	4.96	1	0.81507	.	.	1
22627	0	4.96	1	0.81507	.	.	1
22628	0	4.96	1	0.81507	.	.	1
22629	0	4.96	1	0.81507	.	.	1
22630	0	4.96	1	0.81507	.	.	1
22631	0	4.96	1	0.81507	.	.	1
22632	0	4.96	1	0.81507	.	.	1
22633	0	4.96	1	0.81507	.	.	1
22634	0	4.96	1	0.81507	.	.	1
22635	0	4.96	1	0.81507	.	.	1
22636	0	4.96	1	0.81507	.	.	1
22637	0	4.96	1	0.81507	.	.	1
22638	0	4.96	1	0.81507	.	.	1
22639	0	4.96	1	0.81507	.	.	1
22640	0	4.96	1	0.81507	.	.	1
22641	0	4.96	1	0.81507	.	.	1
22642	0	4.96	1	0.81507	.	.	1
22643	0	4.96	1	0.81507	.	.	1
22644	0	4.96	1	0.81507	.	.	1
22645	0	4.96	1	0.81507	.	.	1

Obs	Sub_group	fuptimey	_CENSOR_	SURVIVAL	SDF_LCL	SDF_UCL	STRATUM
22646	0	4.96	1	0.81507	.	.	1
22647	0	4.96	1	0.81507	.	.	1
22648	0	4.96	1	0.81507	.	.	1
22649	0	4.96	1	0.81507	.	.	1
22650	0	4.96	1	0.81507	.	.	1
22651	0	4.96	1	0.81507	.	.	1
22652	0	4.96	1	0.81507	.	.	1
22653	0	4.97	0	0.81493	0.81082	0.81896	1
22654	0	4.97	1	0.81493	.	.	1
22655	0	4.97	1	0.81493	.	.	1
22656	0	4.97	1	0.81493	.	.	1
22657	0	4.97	1	0.81493	.	.	1
22658	0	4.97	1	0.81493	.	.	1
22659	0	4.97	1	0.81493	.	.	1
22660	0	4.97	1	0.81493	.	.	1
22661	0	4.97	1	0.81493	.	.	1
22662	0	4.97	1	0.81493	.	.	1
22663	0	4.97	1	0.81493	.	.	1
22664	0	4.97	1	0.81493	.	.	1
22665	0	4.97	1	0.81493	.	.	1
22666	0	4.97	1	0.81493	.	.	1
22667	0	4.97	1	0.81493	.	.	1
22668	0	4.97	1	0.81493	.	.	1
22669	0	4.97	1	0.81493	.	.	1
22670	0	4.97	1	0.81493	.	.	1
22671	0	4.97	1	0.81493	.	.	1
22672	0	4.97	1	0.81493	.	.	1
22673	0	4.97	1	0.81493	.	.	1
22674	0	4.97	1	0.81493	.	.	1
22675	0	4.97	1	0.81493	.	.	1
22676	0	4.97	1	0.81493	.	.	1
22677	0	4.97	1	0.81493	.	.	1
22678	0	4.97	1	0.81493	.	.	1
22679	0	4.97	1	0.81493	.	.	1
22680	0	4.97	1	0.81493	.	.	1

Obs	Sub_group	fuptimey	_CENSOR_	SURVIVAL	SDF_LCL	SDF_UCL	STRATUM
22681	0	4.97	1	0.81493	.	.	1
22682	0	4.97	1	0.81493	.	.	1
22683	0	4.97	1	0.81493	.	.	1
22684	0	4.97	1	0.81493	.	.	1
22685	0	4.97	1	0.81493	.	.	1
22686	0	4.97	1	0.81493	.	.	1
22687	0	4.97	1	0.81493	.	.	1
22688	0	4.97	1	0.81493	.	.	1
22689	0	4.97	1	0.81493	.	.	1
22690	0	4.97	1	0.81493	.	.	1
22691	0	4.97	1	0.81493	.	.	1
22692	0	4.97	1	0.81493	.	.	1
22693	0	4.97	1	0.81493	.	.	1
22694	0	4.97	1	0.81493	.	.	1
22695	0	4.98	0	0.81479	0.81068	0.81882	1
22696	0	4.98	1	0.81479	.	.	1
22697	0	4.98	1	0.81479	.	.	1
22698	0	4.98	1	0.81479	.	.	1
22699	0	4.98	1	0.81479	.	.	1
22700	0	4.98	1	0.81479	.	.	1
22701	0	4.98	1	0.81479	.	.	1
22702	0	4.98	1	0.81479	.	.	1
22703	0	4.98	1	0.81479	.	.	1
22704	0	4.98	1	0.81479	.	.	1
22705	0	4.98	1	0.81479	.	.	1
22706	0	4.98	1	0.81479	.	.	1
22707	0	4.98	1	0.81479	.	.	1
22708	0	4.98	1	0.81479	.	.	1
22709	0	4.98	1	0.81479	.	.	1
22710	0	4.98	1	0.81479	.	.	1
22711	0	4.98	1	0.81479	.	.	1
22712	0	4.98	1	0.81479	.	.	1
22713	0	4.98	1	0.81479	.	.	1
22714	0	4.98	1	0.81479	.	.	1
22715	0	4.98	1	0.81479	.	.	1

Obs	Sub_group	fuptimey	_CENSOR_	SURVIVAL	SDF_LCL	SDF_UCL	STRATUM
22716	0	4.98	1	0.81479	.	.	1
22717	0	4.98	1	0.81479	.	.	1
22718	0	4.98	1	0.81479	.	.	1
22719	0	4.98	1	0.81479	.	.	1
22720	0	4.98	1	0.81479	.	.	1
22721	0	4.98	1	0.81479	.	.	1
22722	0	4.98	1	0.81479	.	.	1
22723	0	4.98	1	0.81479	.	.	1
22724	0	4.98	1	0.81479	.	.	1
22725	0	4.98	1	0.81479	.	.	1
22726	0	4.98	1	0.81479	.	.	1
22727	0	4.98	1	0.81479	.	.	1
22728	0	4.99	0	0.81465	0.81053	0.81868	1
22729	0	4.99	1	0.81465	.	.	1
22730	0	4.99	1	0.81465	.	.	1
22731	0	4.99	1	0.81465	.	.	1
22732	0	4.99	1	0.81465	.	.	1
22733	0	4.99	1	0.81465	.	.	1
22734	0	4.99	1	0.81465	.	.	1
22735	0	4.99	1	0.81465	.	.	1
22736	0	4.99	1	0.81465	.	.	1
22737	0	4.99	1	0.81465	.	.	1
22738	0	4.99	1	0.81465	.	.	1
22739	0	4.99	1	0.81465	.	.	1
22740	0	4.99	1	0.81465	.	.	1
22741	0	4.99	1	0.81465	.	.	1
22742	0	4.99	1	0.81465	.	.	1
22743	0	4.99	1	0.81465	.	.	1
22744	0	4.99	1	0.81465	.	.	1
22745	0	4.99	1	0.81465	.	.	1
22746	0	4.99	1	0.81465	.	.	1
22747	0	4.99	1	0.81465	.	.	1
22748	0	4.99	1	0.81465	.	.	1
22749	0	4.99	1	0.81465	.	.	1
22750	0	4.99	1	0.81465	.	.	1

Obs	Sub_group	fuptimey	_CENSOR_	SURVIVAL	SDF_LCL	SDF_UCL	STRATUM
22751	0	4.99	1	0.81465	.	.	1
22752	0	4.99	1	0.81465	.	.	1
22753	0	4.99	1	0.81465	.	.	1
22754	0	4.99	1	0.81465	.	.	1
22755	0	4.99	1	0.81465	.	.	1
22756	0	4.99	1	0.81465	.	.	1
22757	0	4.99	1	0.81465	.	.	1
22758	0	4.99	1	0.81465	.	.	1
22759	0	4.99	1	0.81465	.	.	1
22760	0	4.99	1	0.81465	.	.	1
22761	0	4.99	1	0.81465	.	.	1
22762	0	4.99	1	0.81465	.	.	1
22763	0	4.99	1	0.81465	.	.	1
22764	0	4.99	1	0.81465	.	.	1
22765	0	4.99	1	0.81465	.	.	1
22766	0	4.99	1	0.81465	.	.	1
22767	0	4.99	1	0.81465	.	.	1
22768	0	5.00	0	0.81441	0.81030	0.81845	1
22769	0	5.00	1	0.81441	.	.	1
22770	0	5.00	1	0.81441	.	.	1
22771	0	5.00	1	0.81441	.	.	1
22772	0	5.00	1	0.81441	.	.	1
22773	0	5.00	1	0.81441	.	.	1
22774	0	5.00	1	0.81441	.	.	1
22775	0	5.00	1	0.81441	.	.	1
22776	0	5.00	1	0.81441	.	.	1
22777	0	5.00	1	0.81441	.	.	1
22778	0	5.00	1	0.81441	.	.	1
22779	0	5.00	1	0.81441	.	.	1
22780	0	5.00	1	0.81441	.	.	1
22781	0	5.00	1	0.81441	.	.	1
22782	0	5.00	1	0.81441	.	.	1
22783	0	5.00	1	0.81441	.	.	1
22784	0	5.00	1	0.81441	.	.	1
22785	0	5.00	1	0.81441	.	.	1

Obs	Sub_group	fuptimey	_CENSOR_	SURVIVAL	SDF_LCL	SDF_UCL	STRATUM
22786	0	5.00	1	0.81441	.	.	1
22787	0	5.00	1	0.81441	.	.	1
22788	0	5.00	1	0.81441	.	.	1
22789	0	5.00	1	0.81441	.	.	1
22790	0	5.00	1	0.81441	.	.	1
22791	0	5.00	1	0.81441	.	.	1
22792	0	5.00	1	0.81441	.	.	1
22793	0	5.00	1	0.81441	.	.	1
22794	0	5.00	1	0.81441	.	.	1
22795	0	5.01	1	0.81441	.	.	1
22796	0	5.01	1	0.81441	.	.	1
22797	0	5.01	1	0.81441	.	.	1
22798	0	5.01	1	0.81441	.	.	1
22799	0	5.01	1	0.81441	.	.	1
22800	0	5.01	1	0.81441	.	.	1
22801	0	5.01	1	0.81441	.	.	1
22802	0	5.01	1	0.81441	.	.	1
22803	0	5.01	1	0.81441	.	.	1
22804	0	5.01	1	0.81441	.	.	1
22805	0	5.01	1	0.81441	.	.	1
22806	0	5.01	1	0.81441	.	.	1
22807	0	5.01	1	0.81441	.	.	1
22808	0	5.01	1	0.81441	.	.	1
22809	0	5.01	1	0.81441	.	.	1
22810	0	5.01	1	0.81441	.	.	1
22811	0	5.01	1	0.81441	.	.	1
22812	0	5.01	1	0.81441	.	.	1
22813	0	5.01	1	0.81441	.	.	1
22814	0	5.01	1	0.81441	.	.	1
22815	0	5.01	1	0.81441	.	.	1
22816	0	5.01	1	0.81441	.	.	1
22817	0	5.01	1	0.81441	.	.	1
22818	0	5.01	1	0.81441	.	.	1
22819	0	5.01	1	0.81441	.	.	1
22820	0	5.01	1	0.81441	.	.	1

Obs	Sub_group	fuptimey	_CENSOR_	SURVIVAL	SDF_LCL	SDF_UCL	STRATUM
22821	0	5.01	1	0.81441	.	.	1
22822	0	5.01	1	0.81441	.	.	1
22823	0	5.01	1	0.81441	.	.	1
22824	0	5.01	1	0.81441	.	.	1
22825	0	5.01	1	0.81441	.	.	1
22826	0	5.01	1	0.81441	.	.	1
22827	0	5.01	1	0.81441	.	.	1
22828	0	5.01	1	0.81441	.	.	1
22829	0	5.01	1	0.81441	.	.	1
22830	0	5.01	1	0.81441	.	.	1
22831	0	5.01	1	0.81441	.	.	1
22832	0	5.01	1	0.81441	.	.	1
22833	0	5.01	1	0.81441	.	.	1
22834	0	5.01	1	0.81441	.	.	1
22835	0	5.01	1	0.81441	.	.	1
22836	0	5.01	1	0.81441	.	.	1
22837	0	5.01	1	0.81441	.	.	1
22838	0	5.02	0	0.81413	0.81001	0.81817	1
22839	0	5.02	1	0.81413	.	.	1
22840	0	5.02	1	0.81413	.	.	1
22841	0	5.02	1	0.81413	.	.	1
22842	0	5.02	1	0.81413	.	.	1
22843	0	5.02	1	0.81413	.	.	1
22844	0	5.02	1	0.81413	.	.	1
22845	0	5.02	1	0.81413	.	.	1
22846	0	5.02	1	0.81413	.	.	1
22847	0	5.02	1	0.81413	.	.	1
22848	0	5.02	1	0.81413	.	.	1
22849	0	5.02	1	0.81413	.	.	1
22850	0	5.02	1	0.81413	.	.	1
22851	0	5.02	1	0.81413	.	.	1
22852	0	5.02	1	0.81413	.	.	1
22853	0	5.02	1	0.81413	.	.	1
22854	0	5.02	1	0.81413	.	.	1
22855	0	5.02	1	0.81413	.	.	1

Obs	Sub_group	fuptimey	_CENSOR_	SURVIVAL	SDF_LCL	SDF_UCL	STRATUM
22856	0	5.02	1	0.81413	.	.	1
22857	0	5.02	1	0.81413	.	.	1
22858	0	5.02	1	0.81413	.	.	1
22859	0	5.02	1	0.81413	.	.	1
22860	0	5.02	1	0.81413	.	.	1
22861	0	5.02	1	0.81413	.	.	1
22862	0	5.02	1	0.81413	.	.	1
22863	0	5.02	1	0.81413	.	.	1
22864	0	5.02	1	0.81413	.	.	1
22865	0	5.02	1	0.81413	.	.	1
22866	0	5.02	1	0.81413	.	.	1
22867	0	5.02	1	0.81413	.	.	1
22868	0	5.02	1	0.81413	.	.	1
22869	0	5.02	1	0.81413	.	.	1
22870	0	5.02	1	0.81413	.	.	1
22871	0	5.02	1	0.81413	.	.	1
22872	0	5.02	1	0.81413	.	.	1
22873	0	5.02	1	0.81413	.	.	1
22874	0	5.02	1	0.81413	.	.	1
22875	0	5.02	1	0.81413	.	.	1
22876	0	5.02	1	0.81413	.	.	1
22877	0	5.03	0	0.81403	0.80991	0.81808	1
22878	0	5.03	1	0.81403	.	.	1
22879	0	5.03	1	0.81403	.	.	1
22880	0	5.03	1	0.81403	.	.	1
22881	0	5.03	1	0.81403	.	.	1
22882	0	5.03	1	0.81403	.	.	1
22883	0	5.03	1	0.81403	.	.	1
22884	0	5.03	1	0.81403	.	.	1
22885	0	5.03	1	0.81403	.	.	1
22886	0	5.03	1	0.81403	.	.	1
22887	0	5.03	1	0.81403	.	.	1
22888	0	5.03	1	0.81403	.	.	1
22889	0	5.03	1	0.81403	.	.	1
22890	0	5.03	1	0.81403	.	.	1

Obs	Sub_group	fuptimey	_CENSOR_	SURVIVAL	SDF_LCL	SDF_UCL	STRATUM
**22891**	0	5.03	1	0.81403	.	.	1
**22892**	0	5.03	1	0.81403	.	.	1
**22893**	0	5.03	1	0.81403	.	.	1
**22894**	0	5.03	1	0.81403	.	.	1
**22895**	0	5.03	1	0.81403	.	.	1
**22896**	0	5.03	1	0.81403	.	.	1
**22897**	0	5.03	1	0.81403	.	.	1
**22898**	0	5.03	1	0.81403	.	.	1
**22899**	0	5.03	1	0.81403	.	.	1
**22900**	0	5.03	1	0.81403	.	.	1
**22901**	0	5.03	1	0.81403	.	.	1
**22902**	0	5.03	1	0.81403	.	.	1
**22903**	0	5.03	1	0.81403	.	.	1
**22904**	0	5.03	1	0.81403	.	.	1
**22905**	0	5.03	1	0.81403	.	.	1
**22906**	0	5.03	1	0.81403	.	.	1
**22907**	0	5.03	1	0.81403	.	.	1
**22908**	0	5.03	1	0.81403	.	.	1
**22909**	0	5.03	1	0.81403	.	.	1
**22910**	0	5.03	1	0.81403	.	.	1
**22911**	0	5.03	1	0.81403	.	.	1
**22912**	0	5.03	1	0.81403	.	.	1
**22913**	0	5.03	1	0.81403	.	.	1
**22914**	0	5.03	1	0.81403	.	.	1
**22915**	0	5.03	1	0.81403	.	.	1
**22916**	0	5.04	0	0.81375	0.80962	0.81780	1
**22917**	0	5.04	1	0.81375	.	.	1
**22918**	0	5.04	1	0.81375	.	.	1
**22919**	0	5.04	1	0.81375	.	.	1
**22920**	0	5.04	1	0.81375	.	.	1
**22921**	0	5.04	1	0.81375	.	.	1
**22922**	0	5.04	1	0.81375	.	.	1
**22923**	0	5.04	1	0.81375	.	.	1
**22924**	0	5.04	1	0.81375	.	.	1
**22925**	0	5.04	1	0.81375	.	.	1

Obs	Sub_group	fuptimey	_CENSOR_	SURVIVAL	SDF_LCL	SDF_UCL	STRATUM
22926	0	5.04	1	0.81375	.	.	1
22927	0	5.04	1	0.81375	.	.	1
22928	0	5.04	1	0.81375	.	.	1
22929	0	5.04	1	0.81375	.	.	1
22930	0	5.04	1	0.81375	.	.	1
22931	0	5.04	1	0.81375	.	.	1
22932	0	5.04	1	0.81375	.	.	1
22933	0	5.04	1	0.81375	.	.	1
22934	0	5.04	1	0.81375	.	.	1
22935	0	5.04	1	0.81375	.	.	1
22936	0	5.04	1	0.81375	.	.	1
22937	0	5.04	1	0.81375	.	.	1
22938	0	5.04	1	0.81375	.	.	1
22939	0	5.04	1	0.81375	.	.	1
22940	0	5.04	1	0.81375	.	.	1
22941	0	5.04	1	0.81375	.	.	1
22942	0	5.04	1	0.81375	.	.	1
22943	0	5.04	1	0.81375	.	.	1
22944	0	5.05	0	0.81370	0.80957	0.81775	1
22945	0	5.05	1	0.81370	.	.	1
22946	0	5.05	1	0.81370	.	.	1
22947	0	5.05	1	0.81370	.	.	1
22948	0	5.05	1	0.81370	.	.	1
22949	0	5.05	1	0.81370	.	.	1
22950	0	5.05	1	0.81370	.	.	1
22951	0	5.05	1	0.81370	.	.	1
22952	0	5.05	1	0.81370	.	.	1
22953	0	5.05	1	0.81370	.	.	1
22954	0	5.05	1	0.81370	.	.	1
22955	0	5.05	1	0.81370	.	.	1
22956	0	5.05	1	0.81370	.	.	1
22957	0	5.05	1	0.81370	.	.	1
22958	0	5.05	1	0.81370	.	.	1
22959	0	5.05	1	0.81370	.	.	1
22960	0	5.05	1	0.81370	.	.	1

Obs	Sub_group	fuptimey	_CENSOR_	SURVIVAL	SDF_LCL	SDF_UCL	STRATUM
22961	0	5.05	1	0.81370	.	.	1
22962	0	5.05	1	0.81370	.	.	1
22963	0	5.05	1	0.81370	.	.	1
22964	0	5.05	1	0.81370	.	.	1
22965	0	5.05	1	0.81370	.	.	1
22966	0	5.05	1	0.81370	.	.	1
22967	0	5.05	1	0.81370	.	.	1
22968	0	5.05	1	0.81370	.	.	1
22969	0	5.05	1	0.81370	.	.	1
22970	0	5.05	1	0.81370	.	.	1
22971	0	5.05	1	0.81370	.	.	1
22972	0	5.05	1	0.81370	.	.	1
22973	0	5.05	1	0.81370	.	.	1
22974	0	5.05	1	0.81370	.	.	1
22975	0	5.05	1	0.81370	.	.	1
22976	0	5.05	1	0.81370	.	.	1
22977	0	5.05	1	0.81370	.	.	1
22978	0	5.05	1	0.81370	.	.	1
22979	0	5.05	1	0.81370	.	.	1
22980	0	5.05	1	0.81370	.	.	1
22981	0	5.05	1	0.81370	.	.	1
22982	0	5.05	1	0.81370	.	.	1
22983	0	5.05	1	0.81370	.	.	1
22984	0	5.05	1	0.81370	.	.	1
22985	0	5.05	1	0.81370	.	.	1
22986	0	5.05	1	0.81370	.	.	1
22987	0	5.06	0	0.81366	0.80953	0.81771	1
22988	0	5.06	1	0.81366	.	.	1
22989	0	5.06	1	0.81366	.	.	1
22990	0	5.06	1	0.81366	.	.	1
22991	0	5.06	1	0.81366	.	.	1
22992	0	5.06	1	0.81366	.	.	1
22993	0	5.06	1	0.81366	.	.	1
22994	0	5.06	1	0.81366	.	.	1
22995	0	5.06	1	0.81366	.	.	1

Obs	Sub_group	fuptimey	_CENSOR_	SURVIVAL	SDF_LCL	SDF_UCL	STRATUM
22996	0	5.06	1	0.81366	.	.	1
22997	0	5.06	1	0.81366	.	.	1
22998	0	5.06	1	0.81366	.	.	1
22999	0	5.06	1	0.81366	.	.	1
23000	0	5.06	1	0.81366	.	.	1
23001	0	5.06	1	0.81366	.	.	1
23002	0	5.06	1	0.81366	.	.	1
23003	0	5.06	1	0.81366	.	.	1
23004	0	5.06	1	0.81366	.	.	1
23005	0	5.06	1	0.81366	.	.	1
23006	0	5.06	1	0.81366	.	.	1
23007	0	5.06	1	0.81366	.	.	1
23008	0	5.06	1	0.81366	.	.	1
23009	0	5.06	1	0.81366	.	.	1
23010	0	5.06	1	0.81366	.	.	1
23011	0	5.06	1	0.81366	.	.	1
23012	0	5.06	1	0.81366	.	.	1
23013	0	5.06	1	0.81366	.	.	1
23014	0	5.07	0	0.81337	0.80924	0.81743	1
23015	0	5.07	1	0.81337	.	.	1
23016	0	5.07	1	0.81337	.	.	1
23017	0	5.07	1	0.81337	.	.	1
23018	0	5.07	1	0.81337	.	.	1
23019	0	5.07	1	0.81337	.	.	1
23020	0	5.07	1	0.81337	.	.	1
23021	0	5.07	1	0.81337	.	.	1
23022	0	5.07	1	0.81337	.	.	1
23023	0	5.07	1	0.81337	.	.	1
23024	0	5.07	1	0.81337	.	.	1
23025	0	5.07	1	0.81337	.	.	1
23026	0	5.07	1	0.81337	.	.	1
23027	0	5.07	1	0.81337	.	.	1
23028	0	5.07	1	0.81337	.	.	1
23029	0	5.07	1	0.81337	.	.	1
23030	0	5.07	1	0.81337	.	.	1

Obs	Sub_group	fuptimey	_CENSOR_	SURVIVAL	SDF_LCL	SDF_UCL	STRATUM
**23031**	0	5.07	1	0.81337	.	.	1
**23032**	0	5.07	1	0.81337	.	.	1
**23033**	0	5.07	1	0.81337	.	.	1
**23034**	0	5.07	1	0.81337	.	.	1
**23035**	0	5.07	1	0.81337	.	.	1
**23036**	0	5.07	1	0.81337	.	.	1
**23037**	0	5.07	1	0.81337	.	.	1
**23038**	0	5.07	1	0.81337	.	.	1
**23039**	0	5.07	1	0.81337	.	.	1
**23040**	0	5.07	1	0.81337	.	.	1
**23041**	0	5.07	1	0.81337	.	.	1
**23042**	0	5.07	1	0.81337	.	.	1
**23043**	0	5.07	1	0.81337	.	.	1
**23044**	0	5.07	1	0.81337	.	.	1
**23045**	0	5.07	1	0.81337	.	.	1
**23046**	0	5.07	1	0.81337	.	.	1
**23047**	0	5.07	1	0.81337	.	.	1
**23048**	0	5.07	1	0.81337	.	.	1
**23049**	0	5.07	1	0.81337	.	.	1
**23050**	0	5.07	1	0.81337	.	.	1
**23051**	0	5.07	1	0.81337	.	.	1
**23052**	0	5.07	1	0.81337	.	.	1
**23053**	0	5.07	1	0.81337	.	.	1
**23054**	0	5.07	1	0.81337	.	.	1
**23055**	0	5.07	1	0.81337	.	.	1
**23056**	0	5.07	1	0.81337	.	.	1
**23057**	0	5.07	1	0.81337	.	.	1
**23058**	0	5.08	0	0.81323	0.80909	0.81728	1
**23059**	0	5.08	1	0.81323	.	.	1
**23060**	0	5.08	1	0.81323	.	.	1
**23061**	0	5.08	1	0.81323	.	.	1
**23062**	0	5.08	1	0.81323	.	.	1
**23063**	0	5.08	1	0.81323	.	.	1
**23064**	0	5.08	1	0.81323	.	.	1
**23065**	0	5.08	1	0.81323	.	.	1

Obs	Sub_group	fuptimey	_CENSOR_	SURVIVAL	SDF_LCL	SDF_UCL	STRATUM
23066	0	5.08	1	0.81323	.	.	1
23067	0	5.08	1	0.81323	.	.	1
23068	0	5.08	1	0.81323	.	.	1
23069	0	5.08	1	0.81323	.	.	1
23070	0	5.08	1	0.81323	.	.	1
23071	0	5.08	1	0.81323	.	.	1
23072	0	5.08	1	0.81323	.	.	1
23073	0	5.08	1	0.81323	.	.	1
23074	0	5.08	1	0.81323	.	.	1
23075	0	5.08	1	0.81323	.	.	1
23076	0	5.08	1	0.81323	.	.	1
23077	0	5.08	1	0.81323	.	.	1
23078	0	5.08	1	0.81323	.	.	1
23079	0	5.08	1	0.81323	.	.	1
23080	0	5.08	1	0.81323	.	.	1
23081	0	5.08	1	0.81323	.	.	1
23082	0	5.08	1	0.81323	.	.	1
23083	0	5.08	1	0.81323	.	.	1
23084	0	5.08	1	0.81323	.	.	1
23085	0	5.08	1	0.81323	.	.	1
23086	0	5.08	1	0.81323	.	.	1
23087	0	5.08	1	0.81323	.	.	1
23088	0	5.08	1	0.81323	.	.	1
23089	0	5.08	1	0.81323	.	.	1
23090	0	5.09	0	0.81284	0.80870	0.81691	1
23091	0	5.09	1	0.81284	.	.	1
23092	0	5.09	1	0.81284	.	.	1
23093	0	5.09	1	0.81284	.	.	1
23094	0	5.09	1	0.81284	.	.	1
23095	0	5.09	1	0.81284	.	.	1
23096	0	5.09	1	0.81284	.	.	1
23097	0	5.09	1	0.81284	.	.	1
23098	0	5.09	1	0.81284	.	.	1
23099	0	5.09	1	0.81284	.	.	1
23100	0	5.09	1	0.81284	.	.	1

Obs	Sub_group	fuptimey	_CENSOR_	SURVIVAL	SDF_LCL	SDF_UCL	STRATUM
23101	0	5.09	1	0.81284	.	.	1
23102	0	5.09	1	0.81284	.	.	1
23103	0	5.09	1	0.81284	.	.	1
23104	0	5.09	1	0.81284	.	.	1
23105	0	5.09	1	0.81284	.	.	1
23106	0	5.09	1	0.81284	.	.	1
23107	0	5.09	1	0.81284	.	.	1
23108	0	5.09	1	0.81284	.	.	1
23109	0	5.09	1	0.81284	.	.	1
23110	0	5.09	1	0.81284	.	.	1
23111	0	5.09	1	0.81284	.	.	1
23112	0	5.09	1	0.81284	.	.	1
23113	0	5.09	1	0.81284	.	.	1
23114	0	5.09	1	0.81284	.	.	1
23115	0	5.09	1	0.81284	.	.	1
23116	0	5.09	1	0.81284	.	.	1
23117	0	5.09	1	0.81284	.	.	1
23118	0	5.09	1	0.81284	.	.	1
23119	0	5.09	1	0.81284	.	.	1
23120	0	5.09	1	0.81284	.	.	1
23121	0	5.09	1	0.81284	.	.	1
23122	0	5.09	1	0.81284	.	.	1
23123	0	5.09	1	0.81284	.	.	1
23124	0	5.10	0	0.81256	0.80841	0.81663	1
23125	0	5.10	1	0.81256	.	.	1
23126	0	5.10	1	0.81256	.	.	1
23127	0	5.10	1	0.81256	.	.	1
23128	0	5.10	1	0.81256	.	.	1
23129	0	5.10	1	0.81256	.	.	1
23130	0	5.10	1	0.81256	.	.	1
23131	0	5.10	1	0.81256	.	.	1
23132	0	5.10	1	0.81256	.	.	1
23133	0	5.10	1	0.81256	.	.	1
23134	0	5.10	1	0.81256	.	.	1
23135	0	5.10	1	0.81256	.	.	1

Obs	Sub_group	fuptimey	_CENSOR_	SURVIVAL	SDF_LCL	SDF_UCL	STRATUM
23136	0	5.10	1	0.81256	.	.	1
23137	0	5.10	1	0.81256	.	.	1
23138	0	5.10	1	0.81256	.	.	1
23139	0	5.10	1	0.81256	.	.	1
23140	0	5.10	1	0.81256	.	.	1
23141	0	5.10	1	0.81256	.	.	1
23142	0	5.10	1	0.81256	.	.	1
23143	0	5.10	1	0.81256	.	.	1
23144	0	5.10	1	0.81256	.	.	1
23145	0	5.10	1	0.81256	.	.	1
23146	0	5.10	1	0.81256	.	.	1
23147	0	5.10	1	0.81256	.	.	1
23148	0	5.10	1	0.81256	.	.	1
23149	0	5.10	1	0.81256	.	.	1
23150	0	5.10	1	0.81256	.	.	1
23151	0	5.10	1	0.81256	.	.	1
23152	0	5.10	1	0.81256	.	.	1
23153	0	5.10	1	0.81256	.	.	1
23154	0	5.10	1	0.81256	.	.	1
23155	0	5.10	1	0.81256	.	.	1
23156	0	5.10	1	0.81256	.	.	1
23157	0	5.10	1	0.81256	.	.	1
23158	0	5.10	1	0.81256	.	.	1
23159	0	5.10	1	0.81256	.	.	1
23160	0	5.10	1	0.81256	.	.	1
23161	0	5.10	1	0.81256	.	.	1
23162	0	5.10	1	0.81256	.	.	1
23163	0	5.10	1	0.81256	.	.	1
23164	0	5.10	1	0.81256	.	.	1
23165	0	5.10	1	0.81256	.	.	1
23166	0	5.11	0	0.81246	0.80831	0.81653	1
23167	0	5.11	1	0.81246	.	.	1
23168	0	5.11	1	0.81246	.	.	1
23169	0	5.11	1	0.81246	.	.	1
23170	0	5.11	1	0.81246	.	.	1

Obs	Sub_group	fuptimey	_CENSOR_	SURVIVAL	SDF_LCL	SDF_UCL	STRATUM
23171	0	5.11	1	0.81246	.	.	1
23172	0	5.11	1	0.81246	.	.	1
23173	0	5.11	1	0.81246	.	.	1
23174	0	5.11	1	0.81246	.	.	1
23175	0	5.11	1	0.81246	.	.	1
23176	0	5.11	1	0.81246	.	.	1
23177	0	5.11	1	0.81246	.	.	1
23178	0	5.11	1	0.81246	.	.	1
23179	0	5.11	1	0.81246	.	.	1
23180	0	5.11	1	0.81246	.	.	1
23181	0	5.11	1	0.81246	.	.	1
23182	0	5.11	1	0.81246	.	.	1
23183	0	5.11	1	0.81246	.	.	1
23184	0	5.11	1	0.81246	.	.	1
23185	0	5.11	1	0.81246	.	.	1
23186	0	5.11	1	0.81246	.	.	1
23187	0	5.11	1	0.81246	.	.	1
23188	0	5.11	1	0.81246	.	.	1
23189	0	5.11	1	0.81246	.	.	1
23190	0	5.11	1	0.81246	.	.	1
23191	0	5.11	1	0.81246	.	.	1
23192	0	5.11	1	0.81246	.	.	1
23193	0	5.11	1	0.81246	.	.	1
23194	0	5.11	1	0.81246	.	.	1
23195	0	5.11	1	0.81246	.	.	1
23196	0	5.11	1	0.81246	.	.	1
23197	0	5.11	1	0.81246	.	.	1
23198	0	5.12	0	0.81217	0.80802	0.81625	1
23199	0	5.12	1	0.81217	.	.	1
23200	0	5.12	1	0.81217	.	.	1
23201	0	5.12	1	0.81217	.	.	1
23202	0	5.12	1	0.81217	.	.	1
23203	0	5.12	1	0.81217	.	.	1
23204	0	5.12	1	0.81217	.	.	1
23205	0	5.12	1	0.81217	.	.	1

Obs	Sub_group	fuptimey	_CENSOR_	SURVIVAL	SDF_LCL	SDF_UCL	STRATUM
23206	0	5.12	1	0.81217	.	.	1
23207	0	5.12	1	0.81217	.	.	1
23208	0	5.12	1	0.81217	.	.	1
23209	0	5.12	1	0.81217	.	.	1
23210	0	5.12	1	0.81217	.	.	1
23211	0	5.12	1	0.81217	.	.	1
23212	0	5.12	1	0.81217	.	.	1
23213	0	5.12	1	0.81217	.	.	1
23214	0	5.12	1	0.81217	.	.	1
23215	0	5.12	1	0.81217	.	.	1
23216	0	5.12	1	0.81217	.	.	1
23217	0	5.12	1	0.81217	.	.	1
23218	0	5.12	1	0.81217	.	.	1
23219	0	5.12	1	0.81217	.	.	1
23220	0	5.12	1	0.81217	.	.	1
23221	0	5.12	1	0.81217	.	.	1
23222	0	5.12	1	0.81217	.	.	1
23223	0	5.12	1	0.81217	.	.	1
23224	0	5.12	1	0.81217	.	.	1
23225	0	5.12	1	0.81217	.	.	1
23226	0	5.12	1	0.81217	.	.	1
23227	0	5.12	1	0.81217	.	.	1
23228	0	5.12	1	0.81217	.	.	1
23229	0	5.12	1	0.81217	.	.	1
23230	0	5.12	1	0.81217	.	.	1
23231	0	5.12	1	0.81217	.	.	1
23232	0	5.12	1	0.81217	.	.	1
23233	0	5.12	1	0.81217	.	.	1
23234	0	5.12	1	0.81217	.	.	1
23235	0	5.12	1	0.81217	.	.	1
23236	0	5.12	1	0.81217	.	.	1
23237	0	5.12	1	0.81217	.	.	1
23238	0	5.12	1	0.81217	.	.	1
23239	0	5.12	1	0.81217	.	.	1
23240	0	5.12	1	0.81217	.	.	1

Obs	Sub_group	fuptimey	_CENSOR_	SURVIVAL	SDF_LCL	SDF_UCL	STRATUM
23241	0	5.12	1	0.81217	.	.	1
23242	0	5.12	1	0.81217	.	.	1
23243	0	5.12	1	0.81217	.	.	1
23244	0	5.12	1	0.81217	.	.	1
23245	0	5.12	1	0.81217	.	.	1
23246	0	5.13	0	0.81198	0.80782	0.81606	1
23247	0	5.13	1	0.81198	.	.	1
23248	0	5.13	1	0.81198	.	.	1
23249	0	5.13	1	0.81198	.	.	1
23250	0	5.13	1	0.81198	.	.	1
23251	0	5.13	1	0.81198	.	.	1
23252	0	5.13	1	0.81198	.	.	1
23253	0	5.13	1	0.81198	.	.	1
23254	0	5.13	1	0.81198	.	.	1
23255	0	5.13	1	0.81198	.	.	1
23256	0	5.13	1	0.81198	.	.	1
23257	0	5.13	1	0.81198	.	.	1
23258	0	5.13	1	0.81198	.	.	1
23259	0	5.13	1	0.81198	.	.	1
23260	0	5.13	1	0.81198	.	.	1
23261	0	5.13	1	0.81198	.	.	1
23262	0	5.13	1	0.81198	.	.	1
23263	0	5.13	1	0.81198	.	.	1
23264	0	5.13	1	0.81198	.	.	1
23265	0	5.13	1	0.81198	.	.	1
23266	0	5.13	1	0.81198	.	.	1
23267	0	5.13	1	0.81198	.	.	1
23268	0	5.13	1	0.81198	.	.	1
23269	0	5.13	1	0.81198	.	.	1
23270	0	5.13	1	0.81198	.	.	1
23271	0	5.13	1	0.81198	.	.	1
23272	0	5.13	1	0.81198	.	.	1
23273	0	5.13	1	0.81198	.	.	1
23274	0	5.13	1	0.81198	.	.	1
23275	0	5.13	1	0.81198	.	.	1

Obs	Sub_group	fuptimey	_CENSOR_	SURVIVAL	SDF_LCL	SDF_UCL	STRATUM
**23276**	0	5.13	1	0.81198	.	.	1
**23277**	0	5.13	1	0.81198	.	.	1
**23278**	0	5.13	1	0.81198	.	.	1
**23279**	0	5.13	1	0.81198	.	.	1
**23280**	0	5.13	1	0.81198	.	.	1
**23281**	0	5.13	1	0.81198	.	.	1
**23282**	0	5.13	1	0.81198	.	.	1
**23283**	0	5.13	1	0.81198	.	.	1
**23284**	0	5.13	1	0.81198	.	.	1
**23285**	0	5.13	1	0.81198	.	.	1
**23286**	0	5.13	1	0.81198	.	.	1
**23287**	0	5.14	0	0.81164	0.80748	0.81573	1
**23288**	0	5.14	1	0.81164	.	.	1
**23289**	0	5.14	1	0.81164	.	.	1
**23290**	0	5.14	1	0.81164	.	.	1
**23291**	0	5.14	1	0.81164	.	.	1
**23292**	0	5.14	1	0.81164	.	.	1
**23293**	0	5.14	1	0.81164	.	.	1
**23294**	0	5.14	1	0.81164	.	.	1
**23295**	0	5.14	1	0.81164	.	.	1
**23296**	0	5.14	1	0.81164	.	.	1
**23297**	0	5.14	1	0.81164	.	.	1
**23298**	0	5.14	1	0.81164	.	.	1
**23299**	0	5.14	1	0.81164	.	.	1
**23300**	0	5.14	1	0.81164	.	.	1
**23301**	0	5.14	1	0.81164	.	.	1
**23302**	0	5.14	1	0.81164	.	.	1
**23303**	0	5.14	1	0.81164	.	.	1
**23304**	0	5.14	1	0.81164	.	.	1
**23305**	0	5.14	1	0.81164	.	.	1
**23306**	0	5.14	1	0.81164	.	.	1
**23307**	0	5.14	1	0.81164	.	.	1
**23308**	0	5.14	1	0.81164	.	.	1
**23309**	0	5.14	1	0.81164	.	.	1
**23310**	0	5.14	1	0.81164	.	.	1

Obs	Sub_group	fuptimey	_CENSOR_	SURVIVAL	SDF_LCL	SDF_UCL	STRATUM
**23311**	0	5.14	1	0.81164	.	.	1
**23312**	0	5.14	1	0.81164	.	.	1
**23313**	0	5.14	1	0.81164	.	.	1
**23314**	0	5.14	1	0.81164	.	.	1
**23315**	0	5.14	1	0.81164	.	.	1
**23316**	0	5.14	1	0.81164	.	.	1
**23317**	0	5.14	1	0.81164	.	.	1
**23318**	0	5.14	1	0.81164	.	.	1
**23319**	0	5.14	1	0.81164	.	.	1
**23320**	0	5.14	1	0.81164	.	.	1
**23321**	0	5.14	1	0.81164	.	.	1
**23322**	0	5.14	1	0.81164	.	.	1
**23323**	0	5.14	1	0.81164	.	.	1
**23324**	0	5.14	1	0.81164	.	.	1
**23325**	0	5.14	1	0.81164	.	.	1
**23326**	0	5.14	1	0.81164	.	.	1
**23327**	0	5.14	1	0.81164	.	.	1
**23328**	0	5.14	1	0.81164	.	.	1
**23329**	0	5.15	0	0.81150	0.80733	0.81558	1
**23330**	0	5.15	1	0.81150	.	.	1
**23331**	0	5.15	1	0.81150	.	.	1
**23332**	0	5.15	1	0.81150	.	.	1
**23333**	0	5.15	1	0.81150	.	.	1
**23334**	0	5.15	1	0.81150	.	.	1
**23335**	0	5.15	1	0.81150	.	.	1
**23336**	0	5.15	1	0.81150	.	.	1
**23337**	0	5.15	1	0.81150	.	.	1
**23338**	0	5.15	1	0.81150	.	.	1
**23339**	0	5.15	1	0.81150	.	.	1
**23340**	0	5.15	1	0.81150	.	.	1
**23341**	0	5.15	1	0.81150	.	.	1
**23342**	0	5.15	1	0.81150	.	.	1
**23343**	0	5.15	1	0.81150	.	.	1
**23344**	0	5.15	1	0.81150	.	.	1
**23345**	0	5.15	1	0.81150	.	.	1

Obs	Sub_group	fuptimey	_CENSOR_	SURVIVAL	SDF_LCL	SDF_UCL	STRATUM
23346	0	5.15	1	0.81150	.	.	1
23347	0	5.15	1	0.81150	.	.	1
23348	0	5.15	1	0.81150	.	.	1
23349	0	5.15	1	0.81150	.	.	1
23350	0	5.15	1	0.81150	.	.	1
23351	0	5.15	1	0.81150	.	.	1
23352	0	5.15	1	0.81150	.	.	1
23353	0	5.15	1	0.81150	.	.	1
23354	0	5.15	1	0.81150	.	.	1
23355	0	5.15	1	0.81150	.	.	1
23356	0	5.15	1	0.81150	.	.	1
23357	0	5.15	1	0.81150	.	.	1
23358	0	5.15	1	0.81150	.	.	1
23359	0	5.15	1	0.81150	.	.	1
23360	0	5.15	1	0.81150	.	.	1
23361	0	5.15	1	0.81150	.	.	1
23362	0	5.15	1	0.81150	.	.	1
23363	0	5.15	1	0.81150	.	.	1
23364	0	5.15	1	0.81150	.	.	1
23365	0	5.15	1	0.81150	.	.	1
23366	0	5.15	1	0.81150	.	.	1
23367	0	5.15	1	0.81150	.	.	1
23368	0	5.15	1	0.81150	.	.	1
23369	0	5.15	1	0.81150	.	.	1
23370	0	5.16	0	0.81125	0.80708	0.81534	1
23371	0	5.16	1	0.81125	.	.	1
23372	0	5.16	1	0.81125	.	.	1
23373	0	5.16	1	0.81125	.	.	1
23374	0	5.16	1	0.81125	.	.	1
23375	0	5.16	1	0.81125	.	.	1
23376	0	5.16	1	0.81125	.	.	1
23377	0	5.16	1	0.81125	.	.	1
23378	0	5.16	1	0.81125	.	.	1
23379	0	5.16	1	0.81125	.	.	1
23380	0	5.16	1	0.81125	.	.	1

Obs	Sub_group	fuptimey	_CENSOR_	SURVIVAL	SDF_LCL	SDF_UCL	STRATUM
23381	0	5.16	1	0.81125	.	.	1
23382	0	5.16	1	0.81125	.	.	1
23383	0	5.16	1	0.81125	.	.	1
23384	0	5.16	1	0.81125	.	.	1
23385	0	5.16	1	0.81125	.	.	1
23386	0	5.16	1	0.81125	.	.	1
23387	0	5.16	1	0.81125	.	.	1
23388	0	5.16	1	0.81125	.	.	1
23389	0	5.16	1	0.81125	.	.	1
23390	0	5.16	1	0.81125	.	.	1
23391	0	5.16	1	0.81125	.	.	1
23392	0	5.16	1	0.81125	.	.	1
23393	0	5.16	1	0.81125	.	.	1
23394	0	5.16	1	0.81125	.	.	1
23395	0	5.16	1	0.81125	.	.	1
23396	0	5.16	1	0.81125	.	.	1
23397	0	5.16	1	0.81125	.	.	1
23398	0	5.16	1	0.81125	.	.	1
23399	0	5.16	1	0.81125	.	.	1
23400	0	5.17	0	0.81111	0.80693	0.81520	1
23401	0	5.17	1	0.81111	.	.	1
23402	0	5.17	1	0.81111	.	.	1
23403	0	5.17	1	0.81111	.	.	1
23404	0	5.17	1	0.81111	.	.	1
23405	0	5.17	1	0.81111	.	.	1
23406	0	5.17	1	0.81111	.	.	1
23407	0	5.17	1	0.81111	.	.	1
23408	0	5.17	1	0.81111	.	.	1
23409	0	5.17	1	0.81111	.	.	1
23410	0	5.17	1	0.81111	.	.	1
23411	0	5.17	1	0.81111	.	.	1
23412	0	5.17	1	0.81111	.	.	1
23413	0	5.17	1	0.81111	.	.	1
23414	0	5.17	1	0.81111	.	.	1
23415	0	5.17	1	0.81111	.	.	1

Obs	Sub_group	fuptimey	_CENSOR_	SURVIVAL	SDF_LCL	SDF_UCL	STRATUM
23416	0	5.17	1	0.81111	.	.	1
23417	0	5.17	1	0.81111	.	.	1
23418	0	5.17	1	0.81111	.	.	1
23419	0	5.17	1	0.81111	.	.	1
23420	0	5.17	1	0.81111	.	.	1
23421	0	5.17	1	0.81111	.	.	1
23422	0	5.17	1	0.81111	.	.	1
23423	0	5.17	1	0.81111	.	.	1
23424	0	5.17	1	0.81111	.	.	1
23425	0	5.17	1	0.81111	.	.	1
23426	0	5.17	1	0.81111	.	.	1
23427	0	5.17	1	0.81111	.	.	1
23428	0	5.17	1	0.81111	.	.	1
23429	0	5.17	1	0.81111	.	.	1
23430	0	5.17	1	0.81111	.	.	1
23431	0	5.17	1	0.81111	.	.	1
23432	0	5.18	0	0.81091	0.80674	0.81501	1
23433	0	5.18	1	0.81091	.	.	1
23434	0	5.18	1	0.81091	.	.	1
23435	0	5.18	1	0.81091	.	.	1
23436	0	5.18	1	0.81091	.	.	1
23437	0	5.18	1	0.81091	.	.	1
23438	0	5.18	1	0.81091	.	.	1
23439	0	5.18	1	0.81091	.	.	1
23440	0	5.18	1	0.81091	.	.	1
23441	0	5.18	1	0.81091	.	.	1
23442	0	5.18	1	0.81091	.	.	1
23443	0	5.18	1	0.81091	.	.	1
23444	0	5.18	1	0.81091	.	.	1
23445	0	5.18	1	0.81091	.	.	1
23446	0	5.18	1	0.81091	.	.	1
23447	0	5.18	1	0.81091	.	.	1
23448	0	5.18	1	0.81091	.	.	1
23449	0	5.18	1	0.81091	.	.	1
23450	0	5.18	1	0.81091	.	.	1

Obs	Sub_group	fuptimey	_CENSOR_	SURVIVAL	SDF_LCL	SDF_UCL	STRATUM
23451	0	5.18	1	0.81091	.	.	1
23452	0	5.18	1	0.81091	.	.	1
23453	0	5.18	1	0.81091	.	.	1
23454	0	5.18	1	0.81091	.	.	1
23455	0	5.18	1	0.81091	.	.	1
23456	0	5.18	1	0.81091	.	.	1
23457	0	5.18	1	0.81091	.	.	1
23458	0	5.18	1	0.81091	.	.	1
23459	0	5.18	1	0.81091	.	.	1
23460	0	5.18	1	0.81091	.	.	1
23461	0	5.18	1	0.81091	.	.	1
23462	0	5.18	1	0.81091	.	.	1
23463	0	5.18	1	0.81091	.	.	1
23464	0	5.18	1	0.81091	.	.	1
23465	0	5.18	1	0.81091	.	.	1
23466	0	5.18	1	0.81091	.	.	1
23467	0	5.18	1	0.81091	.	.	1
23468	0	5.18	1	0.81091	.	.	1
23469	0	5.18	1	0.81091	.	.	1
23470	0	5.18	1	0.81091	.	.	1
23471	0	5.18	1	0.81091	.	.	1
23472	0	5.18	1	0.81091	.	.	1
23473	0	5.18	1	0.81091	.	.	1
23474	0	5.19	0	0.81057	0.80639	0.81467	1
23475	0	5.19	1	0.81057	.	.	1
23476	0	5.19	1	0.81057	.	.	1
23477	0	5.19	1	0.81057	.	.	1
23478	0	5.19	1	0.81057	.	.	1
23479	0	5.19	1	0.81057	.	.	1
23480	0	5.19	1	0.81057	.	.	1
23481	0	5.19	1	0.81057	.	.	1
23482	0	5.19	1	0.81057	.	.	1
23483	0	5.19	1	0.81057	.	.	1
23484	0	5.19	1	0.81057	.	.	1
23485	0	5.19	1	0.81057	.	.	1

Obs	Sub_group	fuptimey	_CENSOR_	SURVIVAL	SDF_LCL	SDF_UCL	STRATUM
23486	0	5.19	1	0.81057	.	.	1
23487	0	5.19	1	0.81057	.	.	1
23488	0	5.19	1	0.81057	.	.	1
23489	0	5.19	1	0.81057	.	.	1
23490	0	5.19	1	0.81057	.	.	1
23491	0	5.20	0	0.81047	0.80629	0.81458	1
23492	0	5.20	1	0.81047	.	.	1
23493	0	5.20	1	0.81047	.	.	1
23494	0	5.20	1	0.81047	.	.	1
23495	0	5.20	1	0.81047	.	.	1
23496	0	5.20	1	0.81047	.	.	1
23497	0	5.20	1	0.81047	.	.	1
23498	0	5.20	1	0.81047	.	.	1
23499	0	5.20	1	0.81047	.	.	1
23500	0	5.20	1	0.81047	.	.	1
23501	0	5.20	1	0.81047	.	.	1
23502	0	5.20	1	0.81047	.	.	1
23503	0	5.20	1	0.81047	.	.	1
23504	0	5.20	1	0.81047	.	.	1
23505	0	5.20	1	0.81047	.	.	1
23506	0	5.20	1	0.81047	.	.	1
23507	0	5.20	1	0.81047	.	.	1
23508	0	5.20	1	0.81047	.	.	1
23509	0	5.20	1	0.81047	.	.	1
23510	0	5.20	1	0.81047	.	.	1
23511	0	5.20	1	0.81047	.	.	1
23512	0	5.20	1	0.81047	.	.	1
23513	0	5.20	1	0.81047	.	.	1
23514	0	5.20	1	0.81047	.	.	1
23515	0	5.20	1	0.81047	.	.	1
23516	0	5.20	1	0.81047	.	.	1
23517	0	5.20	1	0.81047	.	.	1
23518	0	5.20	1	0.81047	.	.	1
23519	0	5.20	1	0.81047	.	.	1
23520	0	5.20	1	0.81047	.	.	1

Obs	Sub_group	fuptimey	_CENSOR_	SURVIVAL	SDF_LCL	SDF_UCL	STRATUM
23521	0	5.20	1	0.81047	.	.	1
23522	0	5.20	1	0.81047	.	.	1
23523	0	5.20	1	0.81047	.	.	1
23524	0	5.21	0	0.81028	0.80609	0.81439	1
23525	0	5.21	1	0.81028	.	.	1
23526	0	5.21	1	0.81028	.	.	1
23527	0	5.21	1	0.81028	.	.	1
23528	0	5.21	1	0.81028	.	.	1
23529	0	5.21	1	0.81028	.	.	1
23530	0	5.21	1	0.81028	.	.	1
23531	0	5.21	1	0.81028	.	.	1
23532	0	5.21	1	0.81028	.	.	1
23533	0	5.21	1	0.81028	.	.	1
23534	0	5.21	1	0.81028	.	.	1
23535	0	5.21	1	0.81028	.	.	1
23536	0	5.21	1	0.81028	.	.	1
23537	0	5.21	1	0.81028	.	.	1
23538	0	5.21	1	0.81028	.	.	1
23539	0	5.21	1	0.81028	.	.	1
23540	0	5.21	1	0.81028	.	.	1
23541	0	5.21	1	0.81028	.	.	1
23542	0	5.21	1	0.81028	.	.	1
23543	0	5.21	1	0.81028	.	.	1
23544	0	5.21	1	0.81028	.	.	1
23545	0	5.21	1	0.81028	.	.	1
23546	0	5.21	1	0.81028	.	.	1
23547	0	5.21	1	0.81028	.	.	1
23548	0	5.21	1	0.81028	.	.	1
23549	0	5.21	1	0.81028	.	.	1
23550	0	5.21	1	0.81028	.	.	1
23551	0	5.21	1	0.81028	.	.	1
23552	0	5.21	1	0.81028	.	.	1
23553	0	5.21	1	0.81028	.	.	1
23554	0	5.22	0	0.81003	0.80584	0.81414	1
23555	0	5.22	1	0.81003	.	.	1

Obs	Sub_group	fuptimey	_CENSOR_	SURVIVAL	SDF_LCL	SDF_UCL	STRATUM
23556	0	5.22	1	0.81003	.	.	1
23557	0	5.22	1	0.81003	.	.	1
23558	0	5.22	1	0.81003	.	.	1
23559	0	5.22	1	0.81003	.	.	1
23560	0	5.22	1	0.81003	.	.	1
23561	0	5.22	1	0.81003	.	.	1
23562	0	5.22	1	0.81003	.	.	1
23563	0	5.22	1	0.81003	.	.	1
23564	0	5.22	1	0.81003	.	.	1
23565	0	5.22	1	0.81003	.	.	1
23566	0	5.22	1	0.81003	.	.	1
23567	0	5.22	1	0.81003	.	.	1
23568	0	5.22	1	0.81003	.	.	1
23569	0	5.22	1	0.81003	.	.	1
23570	0	5.22	1	0.81003	.	.	1
23571	0	5.22	1	0.81003	.	.	1
23572	0	5.22	1	0.81003	.	.	1
23573	0	5.22	1	0.81003	.	.	1
23574	0	5.22	1	0.81003	.	.	1
23575	0	5.22	1	0.81003	.	.	1
23576	0	5.22	1	0.81003	.	.	1
23577	0	5.22	1	0.81003	.	.	1
23578	0	5.22	1	0.81003	.	.	1
23579	0	5.22	1	0.81003	.	.	1
23580	0	5.22	1	0.81003	.	.	1
23581	0	5.22	1	0.81003	.	.	1
23582	0	5.22	1	0.81003	.	.	1
23583	0	5.22	1	0.81003	.	.	1
23584	0	5.22	1	0.81003	.	.	1
23585	0	5.23	0	0.80983	0.80564	0.81395	1
23586	0	5.23	1	0.80983	.	.	1
23587	0	5.23	1	0.80983	.	.	1
23588	0	5.23	1	0.80983	.	.	1
23589	0	5.23	1	0.80983	.	.	1
23590	0	5.23	1	0.80983	.	.	1

Obs	Sub_group	fuptimey	_CENSOR_	SURVIVAL	SDF_LCL	SDF_UCL	STRATUM
23591	0	5.23	1	0.80983	.	.	1
23592	0	5.23	1	0.80983	.	.	1
23593	0	5.23	1	0.80983	.	.	1
23594	0	5.23	1	0.80983	.	.	1
23595	0	5.23	1	0.80983	.	.	1
23596	0	5.23	1	0.80983	.	.	1
23597	0	5.23	1	0.80983	.	.	1
23598	0	5.23	1	0.80983	.	.	1
23599	0	5.23	1	0.80983	.	.	1
23600	0	5.23	1	0.80983	.	.	1
23601	0	5.23	1	0.80983	.	.	1
23602	0	5.23	1	0.80983	.	.	1
23603	0	5.23	1	0.80983	.	.	1
23604	0	5.23	1	0.80983	.	.	1
23605	0	5.23	1	0.80983	.	.	1
23606	0	5.23	1	0.80983	.	.	1
23607	0	5.23	1	0.80983	.	.	1
23608	0	5.23	1	0.80983	.	.	1
23609	0	5.23	1	0.80983	.	.	1
23610	0	5.23	1	0.80983	.	.	1
23611	0	5.23	1	0.80983	.	.	1
23612	0	5.23	1	0.80983	.	.	1
23613	0	5.24	0	0.80969	0.80549	0.81381	1
23614	0	5.24	1	0.80969	.	.	1
23615	0	5.24	1	0.80969	.	.	1
23616	0	5.24	1	0.80969	.	.	1
23617	0	5.24	1	0.80969	.	.	1
23618	0	5.24	1	0.80969	.	.	1
23619	0	5.24	1	0.80969	.	.	1
23620	0	5.24	1	0.80969	.	.	1
23621	0	5.24	1	0.80969	.	.	1
23622	0	5.24	1	0.80969	.	.	1
23623	0	5.24	1	0.80969	.	.	1
23624	0	5.24	1	0.80969	.	.	1
23625	0	5.24	1	0.80969	.	.	1

Obs	Sub_group	fuptimey	_CENSOR_	SURVIVAL	SDF_LCL	SDF_UCL	STRATUM
23626	0	5.24	1	0.80969	.	.	1
23627	0	5.24	1	0.80969	.	.	1
23628	0	5.24	1	0.80969	.	.	1
23629	0	5.24	1	0.80969	.	.	1
23630	0	5.24	1	0.80969	.	.	1
23631	0	5.24	1	0.80969	.	.	1
23632	0	5.24	1	0.80969	.	.	1
23633	0	5.24	1	0.80969	.	.	1
23634	0	5.24	1	0.80969	.	.	1
23635	0	5.24	1	0.80969	.	.	1
23636	0	5.24	1	0.80969	.	.	1
23637	0	5.24	1	0.80969	.	.	1
23638	0	5.24	1	0.80969	.	.	1
23639	0	5.24	1	0.80969	.	.	1
23640	0	5.24	1	0.80969	.	.	1
23641	0	5.24	1	0.80969	.	.	1
23642	0	5.24	1	0.80969	.	.	1
23643	0	5.24	1	0.80969	.	.	1
23644	0	5.24	1	0.80969	.	.	1
23645	0	5.24	1	0.80969	.	.	1
23646	0	5.24	1	0.80969	.	.	1
23647	0	5.24	1	0.80969	.	.	1
23648	0	5.24	1	0.80969	.	.	1
23649	0	5.24	1	0.80969	.	.	1
23650	0	5.24	1	0.80969	.	.	1
23651	0	5.24	1	0.80969	.	.	1
23652	0	5.24	1	0.80969	.	.	1
23653	0	5.25	0	0.80939	0.80519	0.81351	1
23654	0	5.25	1	0.80939	.	.	1
23655	0	5.25	1	0.80939	.	.	1
23656	0	5.25	1	0.80939	.	.	1
23657	0	5.25	1	0.80939	.	.	1
23658	0	5.25	1	0.80939	.	.	1
23659	0	5.25	1	0.80939	.	.	1
23660	0	5.25	1	0.80939	.	.	1

Obs	Sub_group	fuptimey	_CENSOR_	SURVIVAL	SDF_LCL	SDF_UCL	STRATUM
23661	0	5.25	1	0.80939	.	.	1
23662	0	5.25	1	0.80939	.	.	1
23663	0	5.25	1	0.80939	.	.	1
23664	0	5.25	1	0.80939	.	.	1
23665	0	5.25	1	0.80939	.	.	1
23666	0	5.25	1	0.80939	.	.	1
23667	0	5.25	1	0.80939	.	.	1
23668	0	5.25	1	0.80939	.	.	1
23669	0	5.25	1	0.80939	.	.	1
23670	0	5.25	1	0.80939	.	.	1
23671	0	5.25	1	0.80939	.	.	1
23672	0	5.26	0	0.80924	0.80503	0.81337	1
23673	0	5.26	1	0.80924	.	.	1
23674	0	5.26	1	0.80924	.	.	1
23675	0	5.26	1	0.80924	.	.	1
23676	0	5.26	1	0.80924	.	.	1
23677	0	5.26	1	0.80924	.	.	1
23678	0	5.26	1	0.80924	.	.	1
23679	0	5.26	1	0.80924	.	.	1
23680	0	5.26	1	0.80924	.	.	1
23681	0	5.26	1	0.80924	.	.	1
23682	0	5.26	1	0.80924	.	.	1
23683	0	5.26	1	0.80924	.	.	1
23684	0	5.26	1	0.80924	.	.	1
23685	0	5.26	1	0.80924	.	.	1
23686	0	5.26	1	0.80924	.	.	1
23687	0	5.26	1	0.80924	.	.	1
23688	0	5.26	1	0.80924	.	.	1
23689	0	5.26	1	0.80924	.	.	1
23690	0	5.26	1	0.80924	.	.	1
23691	0	5.26	1	0.80924	.	.	1
23692	0	5.26	1	0.80924	.	.	1
23693	0	5.26	1	0.80924	.	.	1
23694	0	5.26	1	0.80924	.	.	1
23695	0	5.26	1	0.80924	.	.	1

Obs	Sub_group	fuptimey	_CENSOR_	SURVIVAL	SDF_LCL	SDF_UCL	STRATUM
23696	0	5.26	1	0.80924	.	.	1
23697	0	5.26	1	0.80924	.	.	1
23698	0	5.26	1	0.80924	.	.	1
23699	0	5.26	1	0.80924	.	.	1
23700	0	5.26	1	0.80924	.	.	1
23701	0	5.26	1	0.80924	.	.	1
23702	0	5.26	1	0.80924	.	.	1
23703	0	5.26	1	0.80924	.	.	1
23704	0	5.26	1	0.80924	.	.	1
23705	0	5.26	1	0.80924	.	.	1
23706	0	5.26	1	0.80924	.	.	1
23707	0	5.26	1	0.80924	.	.	1
23708	0	5.26	1	0.80924	.	.	1
23709	0	5.26	1	0.80924	.	.	1
23710	0	5.26	1	0.80924	.	.	1
23711	0	5.26	1	0.80924	.	.	1
23712	0	5.26	1	0.80924	.	.	1
23713	0	5.26	1	0.80924	.	.	1
23714	0	5.26	1	0.80924	.	.	1
23715	0	5.27	0	0.80914	0.80493	0.81327	1
23716	0	5.27	1	0.80914	.	.	1
23717	0	5.27	1	0.80914	.	.	1
23718	0	5.27	1	0.80914	.	.	1
23719	0	5.27	1	0.80914	.	.	1
23720	0	5.27	1	0.80914	.	.	1
23721	0	5.27	1	0.80914	.	.	1
23722	0	5.27	1	0.80914	.	.	1
23723	0	5.27	1	0.80914	.	.	1
23724	0	5.27	1	0.80914	.	.	1
23725	0	5.27	1	0.80914	.	.	1
23726	0	5.27	1	0.80914	.	.	1
23727	0	5.27	1	0.80914	.	.	1
23728	0	5.27	1	0.80914	.	.	1
23729	0	5.27	1	0.80914	.	.	1
23730	0	5.27	1	0.80914	.	.	1

Obs	Sub_group	fuptimey	_CENSOR_	SURVIVAL	SDF_LCL	SDF_UCL	STRATUM
23731	0	5.27	1	0.80914	.	.	1
23732	0	5.27	1	0.80914	.	.	1
23733	0	5.27	1	0.80914	.	.	1
23734	0	5.27	1	0.80914	.	.	1
23735	0	5.27	1	0.80914	.	.	1
23736	0	5.27	1	0.80914	.	.	1
23737	0	5.27	1	0.80914	.	.	1
23738	0	5.27	1	0.80914	.	.	1
23739	0	5.27	1	0.80914	.	.	1
23740	0	5.27	1	0.80914	.	.	1
23741	0	5.27	1	0.80914	.	.	1
23742	0	5.28	0	0.80879	0.80458	0.81293	1
23743	0	5.28	1	0.80879	.	.	1
23744	0	5.28	1	0.80879	.	.	1
23745	0	5.28	1	0.80879	.	.	1
23746	0	5.28	1	0.80879	.	.	1
23747	0	5.28	1	0.80879	.	.	1
23748	0	5.28	1	0.80879	.	.	1
23749	0	5.28	1	0.80879	.	.	1
23750	0	5.28	1	0.80879	.	.	1
23751	0	5.28	1	0.80879	.	.	1
23752	0	5.28	1	0.80879	.	.	1
23753	0	5.28	1	0.80879	.	.	1
23754	0	5.28	1	0.80879	.	.	1
23755	0	5.28	1	0.80879	.	.	1
23756	0	5.28	1	0.80879	.	.	1
23757	0	5.28	1	0.80879	.	.	1
23758	0	5.28	1	0.80879	.	.	1
23759	0	5.28	1	0.80879	.	.	1
23760	0	5.28	1	0.80879	.	.	1
23761	0	5.28	1	0.80879	.	.	1
23762	0	5.28	1	0.80879	.	.	1
23763	0	5.28	1	0.80879	.	.	1
23764	0	5.28	1	0.80879	.	.	1
23765	0	5.28	1	0.80879	.	.	1

Obs	Sub_group	fuptimey	_CENSOR_	SURVIVAL	SDF_LCL	SDF_UCL	STRATUM
**23766**	0	5.28	1	0.80879	.	.	1
**23767**	0	5.28	1	0.80879	.	.	1
**23768**	0	5.28	1	0.80879	.	.	1
**23769**	0	5.28	1	0.80879	.	.	1
**23770**	0	5.28	1	0.80879	.	.	1
**23771**	0	5.28	1	0.80879	.	.	1
**23772**	0	5.28	1	0.80879	.	.	1
**23773**	0	5.28	1	0.80879	.	.	1
**23774**	0	5.28	1	0.80879	.	.	1
**23775**	0	5.28	1	0.80879	.	.	1
**23776**	0	5.28	1	0.80879	.	.	1
**23777**	0	5.29	0	0.80865	0.80443	0.81278	1
**23778**	0	5.29	1	0.80865	.	.	1
**23779**	0	5.29	1	0.80865	.	.	1
**23780**	0	5.29	1	0.80865	.	.	1
**23781**	0	5.29	1	0.80865	.	.	1
**23782**	0	5.29	1	0.80865	.	.	1
**23783**	0	5.29	1	0.80865	.	.	1
**23784**	0	5.29	1	0.80865	.	.	1
**23785**	0	5.29	1	0.80865	.	.	1
**23786**	0	5.29	1	0.80865	.	.	1
**23787**	0	5.29	1	0.80865	.	.	1
**23788**	0	5.29	1	0.80865	.	.	1
**23789**	0	5.29	1	0.80865	.	.	1
**23790**	0	5.29	1	0.80865	.	.	1
**23791**	0	5.29	1	0.80865	.	.	1
**23792**	0	5.29	1	0.80865	.	.	1
**23793**	0	5.29	1	0.80865	.	.	1
**23794**	0	5.29	1	0.80865	.	.	1
**23795**	0	5.29	1	0.80865	.	.	1
**23796**	0	5.29	1	0.80865	.	.	1
**23797**	0	5.29	1	0.80865	.	.	1
**23798**	0	5.29	1	0.80865	.	.	1
**23799**	0	5.29	1	0.80865	.	.	1
**23800**	0	5.29	1	0.80865	.	.	1

Obs	Sub_group	fuptimey	_CENSOR_	SURVIVAL	SDF_LCL	SDF_UCL	STRATUM
23801	0	5.29	1	0.80865	.	.	1
23802	0	5.29	1	0.80865	.	.	1
23803	0	5.30	0	0.80835	0.80412	0.81249	1
23804	0	5.30	1	0.80835	.	.	1
23805	0	5.30	1	0.80835	.	.	1
23806	0	5.30	1	0.80835	.	.	1
23807	0	5.30	1	0.80835	.	.	1
23808	0	5.30	1	0.80835	.	.	1
23809	0	5.30	1	0.80835	.	.	1
23810	0	5.30	1	0.80835	.	.	1
23811	0	5.30	1	0.80835	.	.	1
23812	0	5.30	1	0.80835	.	.	1
23813	0	5.30	1	0.80835	.	.	1
23814	0	5.30	1	0.80835	.	.	1
23815	0	5.30	1	0.80835	.	.	1
23816	0	5.30	1	0.80835	.	.	1
23817	0	5.30	1	0.80835	.	.	1
23818	0	5.30	1	0.80835	.	.	1
23819	0	5.30	1	0.80835	.	.	1
23820	0	5.30	1	0.80835	.	.	1
23821	0	5.30	1	0.80835	.	.	1
23822	0	5.30	1	0.80835	.	.	1
23823	0	5.30	1	0.80835	.	.	1
23824	0	5.30	1	0.80835	.	.	1
23825	0	5.30	1	0.80835	.	.	1
23826	0	5.30	1	0.80835	.	.	1
23827	0	5.30	1	0.80835	.	.	1
23828	0	5.30	1	0.80835	.	.	1
23829	0	5.30	1	0.80835	.	.	1
23830	0	5.30	1	0.80835	.	.	1
23831	0	5.30	1	0.80835	.	.	1
23832	0	5.30	1	0.80835	.	.	1
23833	0	5.30	1	0.80835	.	.	1
23834	0	5.30	1	0.80835	.	.	1
23835	0	5.30	1	0.80835	.	.	1

Obs	Sub_group	fuptimey	_CENSOR_	SURVIVAL	SDF_LCL	SDF_UCL	STRATUM
23836	0	5.30	1	0.80835	.	.	1
23837	0	5.30	1	0.80835	.	.	1
23838	0	5.30	1	0.80835	.	.	1
23839	0	5.30	1	0.80835	.	.	1
23840	0	5.30	1	0.80835	.	.	1
23841	0	5.31	0	0.80805	0.80382	0.81220	1
23842	0	5.31	1	0.80805	.	.	1
23843	0	5.31	1	0.80805	.	.	1
23844	0	5.31	1	0.80805	.	.	1
23845	0	5.31	1	0.80805	.	.	1
23846	0	5.31	1	0.80805	.	.	1
23847	0	5.31	1	0.80805	.	.	1
23848	0	5.31	1	0.80805	.	.	1
23849	0	5.31	1	0.80805	.	.	1
23850	0	5.31	1	0.80805	.	.	1
23851	0	5.31	1	0.80805	.	.	1
23852	0	5.31	1	0.80805	.	.	1
23853	0	5.31	1	0.80805	.	.	1
23854	0	5.31	1	0.80805	.	.	1
23855	0	5.31	1	0.80805	.	.	1
23856	0	5.31	1	0.80805	.	.	1
23857	0	5.31	1	0.80805	.	.	1
23858	0	5.31	1	0.80805	.	.	1
23859	0	5.31	1	0.80805	.	.	1
23860	0	5.31	1	0.80805	.	.	1
23861	0	5.31	1	0.80805	.	.	1
23862	0	5.31	1	0.80805	.	.	1
23863	0	5.31	1	0.80805	.	.	1
23864	0	5.31	1	0.80805	.	.	1
23865	0	5.31	1	0.80805	.	.	1
23866	0	5.31	1	0.80805	.	.	1
23867	0	5.31	1	0.80805	.	.	1
23868	0	5.31	1	0.80805	.	.	1
23869	0	5.31	1	0.80805	.	.	1
23870	0	5.31	1	0.80805	.	.	1

Obs	Sub_group	fuptimey	_CENSOR_	SURVIVAL	SDF_LCL	SDF_UCL	STRATUM
23871	0	5.31	1	0.80805	.	.	1
23872	0	5.31	1	0.80805	.	.	1
23873	0	5.31	1	0.80805	.	.	1
23874	0	5.32	0	0.80795	0.80372	0.81210	1
23875	0	5.32	1	0.80795	.	.	1
23876	0	5.32	1	0.80795	.	.	1
23877	0	5.32	1	0.80795	.	.	1
23878	0	5.32	1	0.80795	.	.	1
23879	0	5.32	1	0.80795	.	.	1
23880	0	5.32	1	0.80795	.	.	1
23881	0	5.32	1	0.80795	.	.	1
23882	0	5.32	1	0.80795	.	.	1
23883	0	5.32	1	0.80795	.	.	1
23884	0	5.32	1	0.80795	.	.	1
23885	0	5.32	1	0.80795	.	.	1
23886	0	5.32	1	0.80795	.	.	1
23887	0	5.32	1	0.80795	.	.	1
23888	0	5.32	1	0.80795	.	.	1
23889	0	5.32	1	0.80795	.	.	1
23890	0	5.32	1	0.80795	.	.	1
23891	0	5.32	1	0.80795	.	.	1
23892	0	5.32	1	0.80795	.	.	1
23893	0	5.32	1	0.80795	.	.	1
23894	0	5.32	1	0.80795	.	.	1
23895	0	5.32	1	0.80795	.	.	1
23896	0	5.32	1	0.80795	.	.	1
23897	0	5.32	1	0.80795	.	.	1
23898	0	5.32	1	0.80795	.	.	1
23899	0	5.33	1	0.80795	.	.	1
23900	0	5.33	1	0.80795	.	.	1
23901	0	5.33	1	0.80795	.	.	1
23902	0	5.33	1	0.80795	.	.	1
23903	0	5.33	1	0.80795	.	.	1
23904	0	5.33	1	0.80795	.	.	1
23905	0	5.33	1	0.80795	.	.	1

Obs	Sub_group	fuptimey	_CENSOR_	SURVIVAL	SDF_LCL	SDF_UCL	STRATUM
23906	0	5.33	1	0.80795	.	.	1
23907	0	5.33	1	0.80795	.	.	1
23908	0	5.33	1	0.80795	.	.	1
23909	0	5.33	1	0.80795	.	.	1
23910	0	5.33	1	0.80795	.	.	1
23911	0	5.33	1	0.80795	.	.	1
23912	0	5.33	1	0.80795	.	.	1
23913	0	5.33	1	0.80795	.	.	1
23914	0	5.33	1	0.80795	.	.	1
23915	0	5.33	1	0.80795	.	.	1
23916	0	5.33	1	0.80795	.	.	1
23917	0	5.33	1	0.80795	.	.	1
23918	0	5.33	1	0.80795	.	.	1
23919	0	5.33	1	0.80795	.	.	1
23920	0	5.33	1	0.80795	.	.	1
23921	0	5.33	1	0.80795	.	.	1
23922	0	5.33	1	0.80795	.	.	1
23923	0	5.33	1	0.80795	.	.	1
23924	0	5.33	1	0.80795	.	.	1
23925	0	5.33	1	0.80795	.	.	1
23926	0	5.33	1	0.80795	.	.	1
23927	0	5.33	1	0.80795	.	.	1
23928	0	5.33	1	0.80795	.	.	1
23929	0	5.33	1	0.80795	.	.	1
23930	0	5.33	1	0.80795	.	.	1
23931	0	5.33	1	0.80795	.	.	1
23932	0	5.33	1	0.80795	.	.	1
23933	0	5.33	1	0.80795	.	.	1
23934	0	5.33	1	0.80795	.	.	1
23935	0	5.33	1	0.80795	.	.	1
23936	0	5.33	1	0.80795	.	.	1
23937	0	5.34	0	0.80790	0.80367	0.81205	1
23938	0	5.34	1	0.80790	.	.	1
23939	0	5.34	1	0.80790	.	.	1
23940	0	5.34	1	0.80790	.	.	1

Obs	Sub_group	fuptimey	_CENSOR_	SURVIVAL	SDF_LCL	SDF_UCL	STRATUM
23941	0	5.34	1	0.80790	.	.	1
23942	0	5.34	1	0.80790	.	.	1
23943	0	5.34	1	0.80790	.	.	1
23944	0	5.34	1	0.80790	.	.	1
23945	0	5.34	1	0.80790	.	.	1
23946	0	5.34	1	0.80790	.	.	1
23947	0	5.34	1	0.80790	.	.	1
23948	0	5.34	1	0.80790	.	.	1
23949	0	5.34	1	0.80790	.	.	1
23950	0	5.34	1	0.80790	.	.	1
23951	0	5.34	1	0.80790	.	.	1
23952	0	5.34	1	0.80790	.	.	1
23953	0	5.34	1	0.80790	.	.	1
23954	0	5.34	1	0.80790	.	.	1
23955	0	5.34	1	0.80790	.	.	1
23956	0	5.34	1	0.80790	.	.	1
23957	0	5.34	1	0.80790	.	.	1
23958	0	5.34	1	0.80790	.	.	1
23959	0	5.34	1	0.80790	.	.	1
23960	0	5.34	1	0.80790	.	.	1
23961	0	5.34	1	0.80790	.	.	1
23962	0	5.34	1	0.80790	.	.	1
23963	0	5.34	1	0.80790	.	.	1
23964	0	5.34	1	0.80790	.	.	1
23965	0	5.34	1	0.80790	.	.	1
23966	0	5.34	1	0.80790	.	.	1
23967	0	5.34	1	0.80790	.	.	1
23968	0	5.34	1	0.80790	.	.	1
23969	0	5.34	1	0.80790	.	.	1
23970	0	5.34	1	0.80790	.	.	1
23971	0	5.34	1	0.80790	.	.	1
23972	0	5.34	1	0.80790	.	.	1
23973	0	5.35	0	0.80765	0.80341	0.81180	1
23974	0	5.35	1	0.80765	.	.	1
23975	0	5.35	1	0.80765	.	.	1

Obs	Sub_group	fuptimey	_CENSOR_	SURVIVAL	SDF_LCL	SDF_UCL	STRATUM
23976	0	5.35	1	0.80765	.	.	1
23977	0	5.35	1	0.80765	.	.	1
23978	0	5.35	1	0.80765	.	.	1
23979	0	5.35	1	0.80765	.	.	1
23980	0	5.35	1	0.80765	.	.	1
23981	0	5.35	1	0.80765	.	.	1
23982	0	5.35	1	0.80765	.	.	1
23983	0	5.35	1	0.80765	.	.	1
23984	0	5.35	1	0.80765	.	.	1
23985	0	5.35	1	0.80765	.	.	1
23986	0	5.35	1	0.80765	.	.	1
23987	0	5.35	1	0.80765	.	.	1
23988	0	5.35	1	0.80765	.	.	1
23989	0	5.35	1	0.80765	.	.	1
23990	0	5.35	1	0.80765	.	.	1
23991	0	5.35	1	0.80765	.	.	1
23992	0	5.35	1	0.80765	.	.	1
23993	0	5.35	1	0.80765	.	.	1
23994	0	5.35	1	0.80765	.	.	1
23995	0	5.35	1	0.80765	.	.	1
23996	0	5.35	1	0.80765	.	.	1
23997	0	5.35	1	0.80765	.	.	1
23998	0	5.35	1	0.80765	.	.	1
23999	0	5.35	1	0.80765	.	.	1
24000	0	5.35	1	0.80765	.	.	1
24001	0	5.35	1	0.80765	.	.	1
24002	0	5.35	1	0.80765	.	.	1
24003	0	5.35	1	0.80765	.	.	1
24004	0	5.35	1	0.80765	.	.	1
24005	0	5.35	1	0.80765	.	.	1
24006	0	5.35	1	0.80765	.	.	1
24007	0	5.36	0	0.80739	0.80315	0.81155	1
24008	0	5.36	1	0.80739	.	.	1
24009	0	5.36	1	0.80739	.	.	1
24010	0	5.36	1	0.80739	.	.	1

Obs	Sub_group	fuptimey	_CENSOR_	SURVIVAL	SDF_LCL	SDF_UCL	STRATUM
24011	0	5.36	1	0.80739	.	.	1
24012	0	5.36	1	0.80739	.	.	1
24013	0	5.36	1	0.80739	.	.	1
24014	0	5.36	1	0.80739	.	.	1
24015	0	5.36	1	0.80739	.	.	1
24016	0	5.36	1	0.80739	.	.	1
24017	0	5.36	1	0.80739	.	.	1
24018	0	5.36	1	0.80739	.	.	1
24019	0	5.36	1	0.80739	.	.	1
24020	0	5.36	1	0.80739	.	.	1
24021	0	5.36	1	0.80739	.	.	1
24022	0	5.36	1	0.80739	.	.	1
24023	0	5.36	1	0.80739	.	.	1
24024	0	5.36	1	0.80739	.	.	1
24025	0	5.36	1	0.80739	.	.	1
24026	0	5.36	1	0.80739	.	.	1
24027	0	5.36	1	0.80739	.	.	1
24028	0	5.36	1	0.80739	.	.	1
24029	0	5.36	1	0.80739	.	.	1
24030	0	5.36	1	0.80739	.	.	1
24031	0	5.36	1	0.80739	.	.	1
24032	0	5.36	1	0.80739	.	.	1
24033	0	5.36	1	0.80739	.	.	1
24034	0	5.36	1	0.80739	.	.	1
24035	0	5.36	1	0.80739	.	.	1
24036	0	5.36	1	0.80739	.	.	1
24037	0	5.36	1	0.80739	.	.	1
24038	0	5.36	1	0.80739	.	.	1
24039	0	5.36	1	0.80739	.	.	1
24040	0	5.36	1	0.80739	.	.	1
24041	0	5.36	1	0.80739	.	.	1
24042	0	5.36	1	0.80739	.	.	1
24043	0	5.36	1	0.80739	.	.	1
24044	0	5.36	1	0.80739	.	.	1
24045	0	5.36	1	0.80739	.	.	1

Obs	Sub_group	fuptimey	_CENSOR_	SURVIVAL	SDF_LCL	SDF_UCL	STRATUM
24046	0	5.37	0	0.80704	0.80279	0.81120	1
24047	0	5.37	1	0.80704	.	.	1
24048	0	5.37	1	0.80704	.	.	1
24049	0	5.37	1	0.80704	.	.	1
24050	0	5.37	1	0.80704	.	.	1
24051	0	5.37	1	0.80704	.	.	1
24052	0	5.37	1	0.80704	.	.	1
24053	0	5.37	1	0.80704	.	.	1
24054	0	5.37	1	0.80704	.	.	1
24055	0	5.37	1	0.80704	.	.	1
24056	0	5.37	1	0.80704	.	.	1
24057	0	5.37	1	0.80704	.	.	1
24058	0	5.37	1	0.80704	.	.	1
24059	0	5.37	1	0.80704	.	.	1
24060	0	5.37	1	0.80704	.	.	1
24061	0	5.37	1	0.80704	.	.	1
24062	0	5.37	1	0.80704	.	.	1
24063	0	5.37	1	0.80704	.	.	1
24064	0	5.37	1	0.80704	.	.	1
24065	0	5.37	1	0.80704	.	.	1
24066	0	5.37	1	0.80704	.	.	1
24067	0	5.37	1	0.80704	.	.	1
24068	0	5.37	1	0.80704	.	.	1
24069	0	5.37	1	0.80704	.	.	1
24070	0	5.37	1	0.80704	.	.	1
24071	0	5.37	1	0.80704	.	.	1
24072	0	5.37	1	0.80704	.	.	1
24073	0	5.37	1	0.80704	.	.	1
24074	0	5.37	1	0.80704	.	.	1
24075	0	5.37	1	0.80704	.	.	1
24076	0	5.37	1	0.80704	.	.	1
24077	0	5.37	1	0.80704	.	.	1
24078	0	5.37	1	0.80704	.	.	1
24079	0	5.37	1	0.80704	.	.	1
24080	0	5.37	1	0.80704	.	.	1

Obs	Sub_group	fuptimey	_CENSOR_	SURVIVAL	SDF_LCL	SDF_UCL	STRATUM
24081	0	5.37	1	0.80704	.	.	1
24082	0	5.37	1	0.80704	.	.	1
24083	0	5.37	1	0.80704	.	.	1
24084	0	5.37	1	0.80704	.	.	1
24085	0	5.37	1	0.80704	.	.	1
24086	0	5.37	1	0.80704	.	.	1
24087	0	5.37	1	0.80704	.	.	1
24088	0	5.38	0	0.80689	0.80264	0.81106	1
24089	0	5.38	1	0.80689	.	.	1
24090	0	5.38	1	0.80689	.	.	1
24091	0	5.38	1	0.80689	.	.	1
24092	0	5.38	1	0.80689	.	.	1
24093	0	5.38	1	0.80689	.	.	1
24094	0	5.38	1	0.80689	.	.	1
24095	0	5.38	1	0.80689	.	.	1
24096	0	5.38	1	0.80689	.	.	1
24097	0	5.38	1	0.80689	.	.	1
24098	0	5.38	1	0.80689	.	.	1
24099	0	5.38	1	0.80689	.	.	1
24100	0	5.38	1	0.80689	.	.	1
24101	0	5.38	1	0.80689	.	.	1
24102	0	5.38	1	0.80689	.	.	1
24103	0	5.38	1	0.80689	.	.	1
24104	0	5.38	1	0.80689	.	.	1
24105	0	5.38	1	0.80689	.	.	1
24106	0	5.38	1	0.80689	.	.	1
24107	0	5.38	1	0.80689	.	.	1
24108	0	5.38	1	0.80689	.	.	1
24109	0	5.38	1	0.80689	.	.	1
24110	0	5.38	1	0.80689	.	.	1
24111	0	5.38	1	0.80689	.	.	1
24112	0	5.38	1	0.80689	.	.	1
24113	0	5.38	1	0.80689	.	.	1
24114	0	5.38	1	0.80689	.	.	1
24115	0	5.38	1	0.80689	.	.	1

Obs	Sub_group	fuptimey	_CENSOR_	SURVIVAL	SDF_LCL	SDF_UCL	STRATUM
24116	0	5.38	1	0.80689	.	.	1
24117	0	5.38	1	0.80689	.	.	1
24118	0	5.38	1	0.80689	.	.	1
24119	0	5.38	1	0.80689	.	.	1
24120	0	5.38	1	0.80689	.	.	1
24121	0	5.38	1	0.80689	.	.	1
24122	0	5.38	1	0.80689	.	.	1
24123	0	5.38	1	0.80689	.	.	1
24124	0	5.38	1	0.80689	.	.	1
24125	0	5.38	1	0.80689	.	.	1
24126	0	5.38	1	0.80689	.	.	1
24127	0	5.38	1	0.80689	.	.	1
24128	0	5.38	1	0.80689	.	.	1
24129	0	5.38	1	0.80689	.	.	1
24130	0	5.38	1	0.80689	.	.	1
24131	0	5.38	1	0.80689	.	.	1
24132	0	5.39	0	0.80684	0.80259	0.81101	1
24133	0	5.39	1	0.80684	.	.	1
24134	0	5.39	1	0.80684	.	.	1
24135	0	5.39	1	0.80684	.	.	1
24136	0	5.39	1	0.80684	.	.	1
24137	0	5.39	1	0.80684	.	.	1
24138	0	5.39	1	0.80684	.	.	1
24139	0	5.39	1	0.80684	.	.	1
24140	0	5.39	1	0.80684	.	.	1
24141	0	5.39	1	0.80684	.	.	1
24142	0	5.39	1	0.80684	.	.	1
24143	0	5.39	1	0.80684	.	.	1
24144	0	5.39	1	0.80684	.	.	1
24145	0	5.39	1	0.80684	.	.	1
24146	0	5.39	1	0.80684	.	.	1
24147	0	5.39	1	0.80684	.	.	1
24148	0	5.39	1	0.80684	.	.	1
24149	0	5.39	1	0.80684	.	.	1
24150	0	5.39	1	0.80684	.	.	1

Obs	Sub_group	fuptimey	_CENSOR_	SURVIVAL	SDF_LCL	SDF_UCL	STRATUM
24151	0	5.39	1	0.80684	.	.	1
24152	0	5.39	1	0.80684	.	.	1
24153	0	5.39	1	0.80684	.	.	1
24154	0	5.39	1	0.80684	.	.	1
24155	0	5.39	1	0.80684	.	.	1
24156	0	5.39	1	0.80684	.	.	1
24157	0	5.39	1	0.80684	.	.	1
24158	0	5.39	1	0.80684	.	.	1
24159	0	5.39	1	0.80684	.	.	1
24160	0	5.39	1	0.80684	.	.	1
24161	0	5.39	1	0.80684	.	.	1
24162	0	5.39	1	0.80684	.	.	1
24163	0	5.39	1	0.80684	.	.	1
24164	0	5.39	1	0.80684	.	.	1
24165	0	5.39	1	0.80684	.	.	1
24166	0	5.39	1	0.80684	.	.	1
24167	0	5.39	1	0.80684	.	.	1
24168	0	5.39	1	0.80684	.	.	1
24169	0	5.39	1	0.80684	.	.	1
24170	0	5.39	1	0.80684	.	.	1
24171	0	5.39	1	0.80684	.	.	1
24172	0	5.40	0	0.80663	0.80238	0.81081	1
24173	0	5.40	1	0.80663	.	.	1
24174	0	5.40	1	0.80663	.	.	1
24175	0	5.40	1	0.80663	.	.	1
24176	0	5.40	1	0.80663	.	.	1
24177	0	5.40	1	0.80663	.	.	1
24178	0	5.40	1	0.80663	.	.	1
24179	0	5.40	1	0.80663	.	.	1
24180	0	5.40	1	0.80663	.	.	1
24181	0	5.40	1	0.80663	.	.	1
24182	0	5.40	1	0.80663	.	.	1
24183	0	5.40	1	0.80663	.	.	1
24184	0	5.40	1	0.80663	.	.	1
24185	0	5.40	1	0.80663	.	.	1

Obs	Sub_group	fuptimey	_CENSOR_	SURVIVAL	SDF_LCL	SDF_UCL	STRATUM
24186	0	5.40	1	0.80663	.	.	1
24187	0	5.40	1	0.80663	.	.	1
24188	0	5.40	1	0.80663	.	.	1
24189	0	5.40	1	0.80663	.	.	1
24190	0	5.40	1	0.80663	.	.	1
24191	0	5.40	1	0.80663	.	.	1
24192	0	5.40	1	0.80663	.	.	1
24193	0	5.40	1	0.80663	.	.	1
24194	0	5.40	1	0.80663	.	.	1
24195	0	5.40	1	0.80663	.	.	1
24196	0	5.40	1	0.80663	.	.	1
24197	0	5.40	1	0.80663	.	.	1
24198	0	5.40	1	0.80663	.	.	1
24199	0	5.40	1	0.80663	.	.	1
24200	0	5.40	1	0.80663	.	.	1
24201	0	5.40	1	0.80663	.	.	1
24202	0	5.40	1	0.80663	.	.	1
24203	0	5.40	1	0.80663	.	.	1
24204	0	5.41	0	0.80638	0.80212	0.81055	1
24205	0	5.41	1	0.80638	.	.	1
24206	0	5.41	1	0.80638	.	.	1
24207	0	5.41	1	0.80638	.	.	1
24208	0	5.41	1	0.80638	.	.	1
24209	0	5.41	1	0.80638	.	.	1
24210	0	5.41	1	0.80638	.	.	1
24211	0	5.41	1	0.80638	.	.	1
24212	0	5.41	1	0.80638	.	.	1
24213	0	5.41	1	0.80638	.	.	1
24214	0	5.41	1	0.80638	.	.	1
24215	0	5.41	1	0.80638	.	.	1
24216	0	5.41	1	0.80638	.	.	1
24217	0	5.41	1	0.80638	.	.	1
24218	0	5.41	1	0.80638	.	.	1
24219	0	5.41	1	0.80638	.	.	1
24220	0	5.41	1	0.80638	.	.	1

Obs	Sub_group	fuptimey	_CENSOR_	SURVIVAL	SDF_LCL	SDF_UCL	STRATUM
**24221**	0	5.41	1	0.80638	.	.	1
**24222**	0	5.41	1	0.80638	.	.	1
**24223**	0	5.41	1	0.80638	.	.	1
**24224**	0	5.41	1	0.80638	.	.	1
**24225**	0	5.41	1	0.80638	.	.	1
**24226**	0	5.41	1	0.80638	.	.	1
**24227**	0	5.41	1	0.80638	.	.	1
**24228**	0	5.41	1	0.80638	.	.	1
**24229**	0	5.41	1	0.80638	.	.	1
**24230**	0	5.41	1	0.80638	.	.	1
**24231**	0	5.41	1	0.80638	.	.	1
**24232**	0	5.41	1	0.80638	.	.	1
**24233**	0	5.41	1	0.80638	.	.	1
**24234**	0	5.41	1	0.80638	.	.	1
**24235**	0	5.41	1	0.80638	.	.	1
**24236**	0	5.41	1	0.80638	.	.	1
**24237**	0	5.41	1	0.80638	.	.	1
**24238**	0	5.41	1	0.80638	.	.	1
**24239**	0	5.41	1	0.80638	.	.	1
**24240**	0	5.41	1	0.80638	.	.	1
**24241**	0	5.41	1	0.80638	.	.	1
**24242**	0	5.41	1	0.80638	.	.	1
**24243**	0	5.41	1	0.80638	.	.	1
**24244**	0	5.41	1	0.80638	.	.	1
**24245**	0	5.41	1	0.80638	.	.	1
**24246**	0	5.41	1	0.80638	.	.	1
**24247**	0	5.41	1	0.80638	.	.	1
**24248**	0	5.41	1	0.80638	.	.	1
**24249**	0	5.41	1	0.80638	.	.	1
**24250**	0	5.41	1	0.80638	.	.	1
**24251**	0	5.42	0	0.80633	0.80207	0.81050	1
**24252**	0	5.42	1	0.80633	.	.	1
**24253**	0	5.42	1	0.80633	.	.	1
**24254**	0	5.42	1	0.80633	.	.	1
**24255**	0	5.42	1	0.80633	.	.	1

Obs	Sub_group	fuptimey	_CENSOR_	SURVIVAL	SDF_LCL	SDF_UCL	STRATUM
**24256**	0	5.42	1	0.80633	.	.	1
**24257**	0	5.42	1	0.80633	.	.	1
**24258**	0	5.42	1	0.80633	.	.	1
**24259**	0	5.42	1	0.80633	.	.	1
**24260**	0	5.42	1	0.80633	.	.	1
**24261**	0	5.42	1	0.80633	.	.	1
**24262**	0	5.42	1	0.80633	.	.	1
**24263**	0	5.42	1	0.80633	.	.	1
**24264**	0	5.42	1	0.80633	.	.	1
**24265**	0	5.42	1	0.80633	.	.	1
**24266**	0	5.42	1	0.80633	.	.	1
**24267**	0	5.42	1	0.80633	.	.	1
**24268**	0	5.42	1	0.80633	.	.	1
**24269**	0	5.42	1	0.80633	.	.	1
**24270**	0	5.42	1	0.80633	.	.	1
**24271**	0	5.42	1	0.80633	.	.	1
**24272**	0	5.42	1	0.80633	.	.	1
**24273**	0	5.42	1	0.80633	.	.	1
**24274**	0	5.42	1	0.80633	.	.	1
**24275**	0	5.42	1	0.80633	.	.	1
**24276**	0	5.43	0	0.80607	0.80181	0.81025	1
**24277**	0	5.43	1	0.80607	.	.	1
**24278**	0	5.43	1	0.80607	.	.	1
**24279**	0	5.43	1	0.80607	.	.	1
**24280**	0	5.43	1	0.80607	.	.	1
**24281**	0	5.43	1	0.80607	.	.	1
**24282**	0	5.43	1	0.80607	.	.	1
**24283**	0	5.43	1	0.80607	.	.	1
**24284**	0	5.43	1	0.80607	.	.	1
**24285**	0	5.43	1	0.80607	.	.	1
**24286**	0	5.43	1	0.80607	.	.	1
**24287**	0	5.43	1	0.80607	.	.	1
**24288**	0	5.43	1	0.80607	.	.	1
**24289**	0	5.43	1	0.80607	.	.	1
**24290**	0	5.43	1	0.80607	.	.	1

Obs	Sub_group	fuptimey	_CENSOR_	SURVIVAL	SDF_LCL	SDF_UCL	STRATUM
24291	0	5.43	1	0.80607	.	.	1
24292	0	5.43	1	0.80607	.	.	1
24293	0	5.43	1	0.80607	.	.	1
24294	0	5.43	1	0.80607	.	.	1
24295	0	5.43	1	0.80607	.	.	1
24296	0	5.43	1	0.80607	.	.	1
24297	0	5.43	1	0.80607	.	.	1
24298	0	5.43	1	0.80607	.	.	1
24299	0	5.43	1	0.80607	.	.	1
24300	0	5.43	1	0.80607	.	.	1
24301	0	5.43	1	0.80607	.	.	1
24302	0	5.43	1	0.80607	.	.	1
24303	0	5.43	1	0.80607	.	.	1
24304	0	5.43	1	0.80607	.	.	1
24305	0	5.43	1	0.80607	.	.	1
24306	0	5.43	1	0.80607	.	.	1
24307	0	5.43	1	0.80607	.	.	1
24308	0	5.43	1	0.80607	.	.	1
24309	0	5.43	1	0.80607	.	.	1
24310	0	5.43	1	0.80607	.	.	1
24311	0	5.43	1	0.80607	.	.	1
24312	0	5.43	1	0.80607	.	.	1
24313	0	5.43	1	0.80607	.	.	1
24314	0	5.43	1	0.80607	.	.	1
24315	0	5.43	1	0.80607	.	.	1
24316	0	5.43	1	0.80607	.	.	1
24317	0	5.43	1	0.80607	.	.	1
24318	0	5.43	1	0.80607	.	.	1
24319	0	5.43	1	0.80607	.	.	1
24320	0	5.43	1	0.80607	.	.	1
24321	0	5.44	1	0.80607	.	.	1
24322	0	5.44	1	0.80607	.	.	1
24323	0	5.44	1	0.80607	.	.	1
24324	0	5.44	1	0.80607	.	.	1
24325	0	5.44	1	0.80607	.	.	1

Obs	Sub_group	fuptimey	_CENSOR_	SURVIVAL	SDF_LCL	SDF_UCL	STRATUM
24326	0	5.44	1	0.80607	.	.	1
24327	0	5.44	1	0.80607	.	.	1
24328	0	5.44	1	0.80607	.	.	1
24329	0	5.44	1	0.80607	.	.	1
24330	0	5.44	1	0.80607	.	.	1
24331	0	5.44	1	0.80607	.	.	1
24332	0	5.44	1	0.80607	.	.	1
24333	0	5.44	1	0.80607	.	.	1
24334	0	5.44	1	0.80607	.	.	1
24335	0	5.44	1	0.80607	.	.	1
24336	0	5.44	1	0.80607	.	.	1
24337	0	5.44	1	0.80607	.	.	1
24338	0	5.44	1	0.80607	.	.	1
24339	0	5.44	1	0.80607	.	.	1
24340	0	5.44	1	0.80607	.	.	1
24341	0	5.44	1	0.80607	.	.	1
24342	0	5.44	1	0.80607	.	.	1
24343	0	5.44	1	0.80607	.	.	1
24344	0	5.44	1	0.80607	.	.	1
24345	0	5.44	1	0.80607	.	.	1
24346	0	5.44	1	0.80607	.	.	1
24347	0	5.44	1	0.80607	.	.	1
24348	0	5.45	0	0.80602	0.80175	0.81020	1
24349	0	5.45	1	0.80602	.	.	1
24350	0	5.45	1	0.80602	.	.	1
24351	0	5.45	1	0.80602	.	.	1
24352	0	5.45	1	0.80602	.	.	1
24353	0	5.45	1	0.80602	.	.	1
24354	0	5.45	1	0.80602	.	.	1
24355	0	5.45	1	0.80602	.	.	1
24356	0	5.45	1	0.80602	.	.	1
24357	0	5.45	1	0.80602	.	.	1
24358	0	5.45	1	0.80602	.	.	1
24359	0	5.45	1	0.80602	.	.	1
24360	0	5.45	1	0.80602	.	.	1

Obs	Sub_group	fuptimey	_CENSOR_	SURVIVAL	SDF_LCL	SDF_UCL	STRATUM
24361	0	5.45	1	0.80602	.	.	1
24362	0	5.45	1	0.80602	.	.	1
24363	0	5.45	1	0.80602	.	.	1
24364	0	5.45	1	0.80602	.	.	1
24365	0	5.45	1	0.80602	.	.	1
24366	0	5.45	1	0.80602	.	.	1
24367	0	5.45	1	0.80602	.	.	1
24368	0	5.45	1	0.80602	.	.	1
24369	0	5.45	1	0.80602	.	.	1
24370	0	5.45	1	0.80602	.	.	1
24371	0	5.45	1	0.80602	.	.	1
24372	0	5.45	1	0.80602	.	.	1
24373	0	5.45	1	0.80602	.	.	1
24374	0	5.45	1	0.80602	.	.	1
24375	0	5.45	1	0.80602	.	.	1
24376	0	5.45	1	0.80602	.	.	1
24377	0	5.45	1	0.80602	.	.	1
24378	0	5.45	1	0.80602	.	.	1
24379	0	5.45	1	0.80602	.	.	1
24380	0	5.45	1	0.80602	.	.	1
24381	0	5.45	1	0.80602	.	.	1
24382	0	5.45	1	0.80602	.	.	1
24383	0	5.45	1	0.80602	.	.	1
24384	0	5.45	1	0.80602	.	.	1
24385	0	5.45	1	0.80602	.	.	1
24386	0	5.45	1	0.80602	.	.	1
24387	0	5.45	1	0.80602	.	.	1
24388	0	5.45	1	0.80602	.	.	1
24389	0	5.46	0	0.80586	0.80160	0.81005	1
24390	0	5.46	1	0.80586	.	.	1
24391	0	5.46	1	0.80586	.	.	1
24392	0	5.46	1	0.80586	.	.	1
24393	0	5.46	1	0.80586	.	.	1
24394	0	5.46	1	0.80586	.	.	1
24395	0	5.46	1	0.80586	.	.	1

Obs	Sub_group	fuptimey	_CENSOR_	SURVIVAL	SDF_LCL	SDF_UCL	STRATUM
24396	0	5.46	1	0.80586	.	.	1
24397	0	5.46	1	0.80586	.	.	1
24398	0	5.46	1	0.80586	.	.	1
24399	0	5.46	1	0.80586	.	.	1
24400	0	5.46	1	0.80586	.	.	1
24401	0	5.46	1	0.80586	.	.	1
24402	0	5.46	1	0.80586	.	.	1
24403	0	5.46	1	0.80586	.	.	1
24404	0	5.46	1	0.80586	.	.	1
24405	0	5.46	1	0.80586	.	.	1
24406	0	5.46	1	0.80586	.	.	1
24407	0	5.46	1	0.80586	.	.	1
24408	0	5.46	1	0.80586	.	.	1
24409	0	5.46	1	0.80586	.	.	1
24410	0	5.46	1	0.80586	.	.	1
24411	0	5.46	1	0.80586	.	.	1
24412	0	5.46	1	0.80586	.	.	1
24413	0	5.46	1	0.80586	.	.	1
24414	0	5.46	1	0.80586	.	.	1
24415	0	5.46	1	0.80586	.	.	1
24416	0	5.46	1	0.80586	.	.	1
24417	0	5.46	1	0.80586	.	.	1
24418	0	5.46	1	0.80586	.	.	1
24419	0	5.46	1	0.80586	.	.	1
24420	0	5.46	1	0.80586	.	.	1
24421	0	5.46	1	0.80586	.	.	1
24422	0	5.46	1	0.80586	.	.	1
24423	0	5.46	1	0.80586	.	.	1
24424	0	5.46	1	0.80586	.	.	1
24425	0	5.47	0	0.80576	0.80149	0.80995	1
24426	0	5.47	1	0.80576	.	.	1
24427	0	5.47	1	0.80576	.	.	1
24428	0	5.47	1	0.80576	.	.	1
24429	0	5.47	1	0.80576	.	.	1
24430	0	5.47	1	0.80576	.	.	1

Obs	Sub_group	fuptimey	_CENSOR_	SURVIVAL	SDF_LCL	SDF_UCL	STRATUM
24431	0	5.47	1	0.80576	.	.	1
24432	0	5.47	1	0.80576	.	.	1
24433	0	5.47	1	0.80576	.	.	1
24434	0	5.47	1	0.80576	.	.	1
24435	0	5.47	1	0.80576	.	.	1
24436	0	5.47	1	0.80576	.	.	1
24437	0	5.47	1	0.80576	.	.	1
24438	0	5.47	1	0.80576	.	.	1
24439	0	5.47	1	0.80576	.	.	1
24440	0	5.47	1	0.80576	.	.	1
24441	0	5.47	1	0.80576	.	.	1
24442	0	5.47	1	0.80576	.	.	1
24443	0	5.47	1	0.80576	.	.	1
24444	0	5.47	1	0.80576	.	.	1
24445	0	5.47	1	0.80576	.	.	1
24446	0	5.47	1	0.80576	.	.	1
24447	0	5.47	1	0.80576	.	.	1
24448	0	5.47	1	0.80576	.	.	1
24449	0	5.47	1	0.80576	.	.	1
24450	0	5.47	1	0.80576	.	.	1
24451	0	5.47	1	0.80576	.	.	1
24452	0	5.47	1	0.80576	.	.	1
24453	0	5.47	1	0.80576	.	.	1
24454	0	5.47	1	0.80576	.	.	1
24455	0	5.47	1	0.80576	.	.	1
24456	0	5.47	1	0.80576	.	.	1
24457	0	5.47	1	0.80576	.	.	1
24458	0	5.47	1	0.80576	.	.	1
24459	0	5.47	1	0.80576	.	.	1
24460	0	5.47	1	0.80576	.	.	1
24461	0	5.47	1	0.80576	.	.	1
24462	0	5.47	1	0.80576	.	.	1
24463	0	5.47	1	0.80576	.	.	1
24464	0	5.47	1	0.80576	.	.	1
24465	0	5.47	1	0.80576	.	.	1

Obs	Sub_group	fuptimey	_CENSOR_	SURVIVAL	SDF_LCL	SDF_UCL	STRATUM
**24466**	0	5.48	0	0.80555	0.80128	0.80974	1
**24467**	0	5.48	1	0.80555	.	.	1
**24468**	0	5.48	1	0.80555	.	.	1
**24469**	0	5.48	1	0.80555	.	.	1
**24470**	0	5.48	1	0.80555	.	.	1
**24471**	0	5.48	1	0.80555	.	.	1
**24472**	0	5.48	1	0.80555	.	.	1
**24473**	0	5.48	1	0.80555	.	.	1
**24474**	0	5.48	1	0.80555	.	.	1
**24475**	0	5.48	1	0.80555	.	.	1
**24476**	0	5.48	1	0.80555	.	.	1
**24477**	0	5.48	1	0.80555	.	.	1
**24478**	0	5.48	1	0.80555	.	.	1
**24479**	0	5.48	1	0.80555	.	.	1
**24480**	0	5.48	1	0.80555	.	.	1
**24481**	0	5.48	1	0.80555	.	.	1
**24482**	0	5.48	1	0.80555	.	.	1
**24483**	0	5.48	1	0.80555	.	.	1
**24484**	0	5.48	1	0.80555	.	.	1
**24485**	0	5.48	1	0.80555	.	.	1
**24486**	0	5.48	1	0.80555	.	.	1
**24487**	0	5.48	1	0.80555	.	.	1
**24488**	0	5.48	1	0.80555	.	.	1
**24489**	0	5.48	1	0.80555	.	.	1
**24490**	0	5.48	1	0.80555	.	.	1
**24491**	0	5.48	1	0.80555	.	.	1
**24492**	0	5.48	1	0.80555	.	.	1
**24493**	0	5.48	1	0.80555	.	.	1
**24494**	0	5.48	1	0.80555	.	.	1
**24495**	0	5.48	1	0.80555	.	.	1
**24496**	0	5.48	1	0.80555	.	.	1
**24497**	0	5.48	1	0.80555	.	.	1
**24498**	0	5.48	1	0.80555	.	.	1
**24499**	0	5.48	1	0.80555	.	.	1
**24500**	0	5.49	0	0.80534	0.80107	0.80954	1

Obs	Sub_group	fuptimey	_CENSOR_	SURVIVAL	SDF_LCL	SDF_UCL	STRATUM
24501	0	5.49	1	0.80534	.	.	1
24502	0	5.49	1	0.80534	.	.	1
24503	0	5.49	1	0.80534	.	.	1
24504	0	5.49	1	0.80534	.	.	1
24505	0	5.49	1	0.80534	.	.	1
24506	0	5.49	1	0.80534	.	.	1
24507	0	5.49	1	0.80534	.	.	1
24508	0	5.49	1	0.80534	.	.	1
24509	0	5.49	1	0.80534	.	.	1
24510	0	5.49	1	0.80534	.	.	1
24511	0	5.49	1	0.80534	.	.	1
24512	0	5.49	1	0.80534	.	.	1
24513	0	5.49	1	0.80534	.	.	1
24514	0	5.49	1	0.80534	.	.	1
24515	0	5.49	1	0.80534	.	.	1
24516	0	5.49	1	0.80534	.	.	1
24517	0	5.49	1	0.80534	.	.	1
24518	0	5.49	1	0.80534	.	.	1
24519	0	5.49	1	0.80534	.	.	1
24520	0	5.49	1	0.80534	.	.	1
24521	0	5.49	1	0.80534	.	.	1
24522	0	5.49	1	0.80534	.	.	1
24523	0	5.49	1	0.80534	.	.	1
24524	0	5.49	1	0.80534	.	.	1
24525	0	5.49	1	0.80534	.	.	1
24526	0	5.49	1	0.80534	.	.	1
24527	0	5.49	1	0.80534	.	.	1
24528	0	5.49	1	0.80534	.	.	1
24529	0	5.49	1	0.80534	.	.	1
24530	0	5.49	1	0.80534	.	.	1
24531	0	5.49	1	0.80534	.	.	1
24532	0	5.49	1	0.80534	.	.	1
24533	0	5.49	1	0.80534	.	.	1
24534	0	5.49	1	0.80534	.	.	1
24535	0	5.49	1	0.80534	.	.	1

Obs	Sub_group	fuptimey	_CENSOR_	SURVIVAL	SDF_LCL	SDF_UCL	STRATUM
24536	0	5.49	1	0.80534	.	.	1
24537	0	5.50	0	0.80508	0.80080	0.80928	1
24538	0	5.50	1	0.80508	.	.	1
24539	0	5.50	1	0.80508	.	.	1
24540	0	5.50	1	0.80508	.	.	1
24541	0	5.50	1	0.80508	.	.	1
24542	0	5.50	1	0.80508	.	.	1
24543	0	5.50	1	0.80508	.	.	1
24544	0	5.50	1	0.80508	.	.	1
24545	0	5.50	1	0.80508	.	.	1
24546	0	5.50	1	0.80508	.	.	1
24547	0	5.50	1	0.80508	.	.	1
24548	0	5.50	1	0.80508	.	.	1
24549	0	5.50	1	0.80508	.	.	1
24550	0	5.50	1	0.80508	.	.	1
24551	0	5.50	1	0.80508	.	.	1
24552	0	5.50	1	0.80508	.	.	1
24553	0	5.50	1	0.80508	.	.	1
24554	0	5.50	1	0.80508	.	.	1
24555	0	5.50	1	0.80508	.	.	1
24556	0	5.50	1	0.80508	.	.	1
24557	0	5.50	1	0.80508	.	.	1
24558	0	5.50	1	0.80508	.	.	1
24559	0	5.50	1	0.80508	.	.	1
24560	0	5.50	1	0.80508	.	.	1
24561	0	5.50	1	0.80508	.	.	1
24562	0	5.50	1	0.80508	.	.	1
24563	0	5.50	1	0.80508	.	.	1
24564	0	5.50	1	0.80508	.	.	1
24565	0	5.50	1	0.80508	.	.	1
24566	0	5.50	1	0.80508	.	.	1
24567	0	5.50	1	0.80508	.	.	1
24568	0	5.50	1	0.80508	.	.	1
24569	0	5.50	1	0.80508	.	.	1
24570	0	5.50	1	0.80508	.	.	1

Obs	Sub_group	fuptimey	_CENSOR_	SURVIVAL	SDF_LCL	SDF_UCL	STRATUM
24571	0	5.50	1	0.80508	.	.	1
24572	0	5.51	0	0.80503	0.80075	0.80923	1
24573	0	5.51	1	0.80503	.	.	1
24574	0	5.51	1	0.80503	.	.	1
24575	0	5.51	1	0.80503	.	.	1
24576	0	5.51	1	0.80503	.	.	1
24577	0	5.51	1	0.80503	.	.	1
24578	0	5.51	1	0.80503	.	.	1
24579	0	5.51	1	0.80503	.	.	1
24580	0	5.51	1	0.80503	.	.	1
24581	0	5.51	1	0.80503	.	.	1
24582	0	5.51	1	0.80503	.	.	1
24583	0	5.51	1	0.80503	.	.	1
24584	0	5.51	1	0.80503	.	.	1
24585	0	5.51	1	0.80503	.	.	1
24586	0	5.51	1	0.80503	.	.	1
24587	0	5.51	1	0.80503	.	.	1
24588	0	5.51	1	0.80503	.	.	1
24589	0	5.51	1	0.80503	.	.	1
24590	0	5.51	1	0.80503	.	.	1
24591	0	5.51	1	0.80503	.	.	1
24592	0	5.51	1	0.80503	.	.	1
24593	0	5.51	1	0.80503	.	.	1
24594	0	5.51	1	0.80503	.	.	1
24595	0	5.51	1	0.80503	.	.	1
24596	0	5.51	1	0.80503	.	.	1
24597	0	5.51	1	0.80503	.	.	1
24598	0	5.51	1	0.80503	.	.	1
24599	0	5.51	1	0.80503	.	.	1
24600	0	5.51	1	0.80503	.	.	1
24601	0	5.51	1	0.80503	.	.	1
24602	0	5.51	1	0.80503	.	.	1
24603	0	5.51	1	0.80503	.	.	1
24604	0	5.51	1	0.80503	.	.	1
24605	0	5.51	1	0.80503	.	.	1

Obs	Sub_group	fuptimey	_CENSOR_	SURVIVAL	SDF_LCL	SDF_UCL	STRATUM
**24606**	0	5.51	1	0.80503	.	.	1
**24607**	0	5.51	1	0.80503	.	.	1
**24608**	0	5.52	0	0.80493	0.80064	0.80913	1
**24609**	0	5.52	1	0.80493	.	.	1
**24610**	0	5.52	1	0.80493	.	.	1
**24611**	0	5.52	1	0.80493	.	.	1
**24612**	0	5.52	1	0.80493	.	.	1
**24613**	0	5.52	1	0.80493	.	.	1
**24614**	0	5.52	1	0.80493	.	.	1
**24615**	0	5.52	1	0.80493	.	.	1
**24616**	0	5.52	1	0.80493	.	.	1
**24617**	0	5.52	1	0.80493	.	.	1
**24618**	0	5.52	1	0.80493	.	.	1
**24619**	0	5.52	1	0.80493	.	.	1
**24620**	0	5.52	1	0.80493	.	.	1
**24621**	0	5.52	1	0.80493	.	.	1
**24622**	0	5.52	1	0.80493	.	.	1
**24623**	0	5.52	1	0.80493	.	.	1
**24624**	0	5.52	1	0.80493	.	.	1
**24625**	0	5.52	1	0.80493	.	.	1
**24626**	0	5.52	1	0.80493	.	.	1
**24627**	0	5.52	1	0.80493	.	.	1
**24628**	0	5.52	1	0.80493	.	.	1
**24629**	0	5.52	1	0.80493	.	.	1
**24630**	0	5.52	1	0.80493	.	.	1
**24631**	0	5.52	1	0.80493	.	.	1
**24632**	0	5.52	1	0.80493	.	.	1
**24633**	0	5.52	1	0.80493	.	.	1
**24634**	0	5.52	1	0.80493	.	.	1
**24635**	0	5.53	0	0.80477	0.80048	0.80898	1
**24636**	0	5.53	1	0.80477	.	.	1
**24637**	0	5.53	1	0.80477	.	.	1
**24638**	0	5.53	1	0.80477	.	.	1
**24639**	0	5.53	1	0.80477	.	.	1
**24640**	0	5.53	1	0.80477	.	.	1

Obs	Sub_group	fuptimey	_CENSOR_	SURVIVAL	SDF_LCL	SDF_UCL	STRATUM
24641	0	5.53	1	0.80477	.	.	1
24642	0	5.53	1	0.80477	.	.	1
24643	0	5.53	1	0.80477	.	.	1
24644	0	5.53	1	0.80477	.	.	1
24645	0	5.53	1	0.80477	.	.	1
24646	0	5.53	1	0.80477	.	.	1
24647	0	5.53	1	0.80477	.	.	1
24648	0	5.53	1	0.80477	.	.	1
24649	0	5.53	1	0.80477	.	.	1
24650	0	5.53	1	0.80477	.	.	1
24651	0	5.53	1	0.80477	.	.	1
24652	0	5.53	1	0.80477	.	.	1
24653	0	5.53	1	0.80477	.	.	1
24654	0	5.53	1	0.80477	.	.	1
24655	0	5.53	1	0.80477	.	.	1
24656	0	5.53	1	0.80477	.	.	1
24657	0	5.53	1	0.80477	.	.	1
24658	0	5.53	1	0.80477	.	.	1
24659	0	5.53	1	0.80477	.	.	1
24660	0	5.53	1	0.80477	.	.	1
24661	0	5.53	1	0.80477	.	.	1
24662	0	5.53	1	0.80477	.	.	1
24663	0	5.53	1	0.80477	.	.	1
24664	0	5.53	1	0.80477	.	.	1
24665	0	5.54	0	0.80445	0.80016	0.80867	1
24666	0	5.54	1	0.80445	.	.	1
24667	0	5.54	1	0.80445	.	.	1
24668	0	5.54	1	0.80445	.	.	1
24669	0	5.54	1	0.80445	.	.	1
24670	0	5.54	1	0.80445	.	.	1
24671	0	5.54	1	0.80445	.	.	1
24672	0	5.54	1	0.80445	.	.	1
24673	0	5.54	1	0.80445	.	.	1
24674	0	5.54	1	0.80445	.	.	1
24675	0	5.54	1	0.80445	.	.	1

Obs	Sub_group	fuptimey	_CENSOR_	SURVIVAL	SDF_LCL	SDF_UCL	STRATUM
24676	0	5.54	1	0.80445	.	.	1
24677	0	5.54	1	0.80445	.	.	1
24678	0	5.54	1	0.80445	.	.	1
24679	0	5.54	1	0.80445	.	.	1
24680	0	5.54	1	0.80445	.	.	1
24681	0	5.54	1	0.80445	.	.	1
24682	0	5.54	1	0.80445	.	.	1
24683	0	5.54	1	0.80445	.	.	1
24684	0	5.54	1	0.80445	.	.	1
24685	0	5.54	1	0.80445	.	.	1
24686	0	5.54	1	0.80445	.	.	1
24687	0	5.54	1	0.80445	.	.	1
24688	0	5.54	1	0.80445	.	.	1
24689	0	5.54	1	0.80445	.	.	1
24690	0	5.54	1	0.80445	.	.	1
24691	0	5.54	1	0.80445	.	.	1
24692	0	5.54	1	0.80445	.	.	1
24693	0	5.54	1	0.80445	.	.	1
24694	0	5.55	0	0.80430	0.80000	0.80851	1
24695	0	5.55	1	0.80430	.	.	1
24696	0	5.55	1	0.80430	.	.	1
24697	0	5.55	1	0.80430	.	.	1
24698	0	5.55	1	0.80430	.	.	1
24699	0	5.55	1	0.80430	.	.	1
24700	0	5.55	1	0.80430	.	.	1
24701	0	5.55	1	0.80430	.	.	1
24702	0	5.55	1	0.80430	.	.	1
24703	0	5.55	1	0.80430	.	.	1
24704	0	5.55	1	0.80430	.	.	1
24705	0	5.55	1	0.80430	.	.	1
24706	0	5.55	1	0.80430	.	.	1
24707	0	5.55	1	0.80430	.	.	1
24708	0	5.55	1	0.80430	.	.	1
24709	0	5.55	1	0.80430	.	.	1
24710	0	5.55	1	0.80430	.	.	1

Obs	Sub_group	fuptimey	_CENSOR_	SURVIVAL	SDF_LCL	SDF_UCL	STRATUM
24711	0	5.55	1	0.80430	.	.	1
24712	0	5.55	1	0.80430	.	.	1
24713	0	5.55	1	0.80430	.	.	1
24714	0	5.55	1	0.80430	.	.	1
24715	0	5.55	1	0.80430	.	.	1
24716	0	5.55	1	0.80430	.	.	1
24717	0	5.55	1	0.80430	.	.	1
24718	0	5.55	1	0.80430	.	.	1
24719	0	5.55	1	0.80430	.	.	1
24720	0	5.55	1	0.80430	.	.	1
24721	0	5.55	1	0.80430	.	.	1
24722	0	5.55	1	0.80430	.	.	1
24723	0	5.55	1	0.80430	.	.	1
24724	0	5.55	1	0.80430	.	.	1
24725	0	5.55	1	0.80430	.	.	1
24726	0	5.55	1	0.80430	.	.	1
24727	0	5.55	1	0.80430	.	.	1
24728	0	5.55	1	0.80430	.	.	1
24729	0	5.55	1	0.80430	.	.	1
24730	0	5.55	1	0.80430	.	.	1
24731	0	5.55	1	0.80430	.	.	1
24732	0	5.55	1	0.80430	.	.	1
24733	0	5.55	1	0.80430	.	.	1
24734	0	5.56	0	0.80408	0.79978	0.80830	1
24735	0	5.56	1	0.80408	.	.	1
24736	0	5.56	1	0.80408	.	.	1
24737	0	5.56	1	0.80408	.	.	1
24738	0	5.56	1	0.80408	.	.	1
24739	0	5.56	1	0.80408	.	.	1
24740	0	5.56	1	0.80408	.	.	1
24741	0	5.56	1	0.80408	.	.	1
24742	0	5.56	1	0.80408	.	.	1
24743	0	5.56	1	0.80408	.	.	1
24744	0	5.56	1	0.80408	.	.	1
24745	0	5.56	1	0.80408	.	.	1

Obs	Sub_group	fuptimey	_CENSOR_	SURVIVAL	SDF_LCL	SDF_UCL	STRATUM
24746	0	5.56	1	0.80408	.	.	1
24747	0	5.56	1	0.80408	.	.	1
24748	0	5.56	1	0.80408	.	.	1
24749	0	5.56	1	0.80408	.	.	1
24750	0	5.56	1	0.80408	.	.	1
24751	0	5.56	1	0.80408	.	.	1
24752	0	5.56	1	0.80408	.	.	1
24753	0	5.56	1	0.80408	.	.	1
24754	0	5.56	1	0.80408	.	.	1
24755	0	5.56	1	0.80408	.	.	1
24756	0	5.56	1	0.80408	.	.	1
24757	0	5.56	1	0.80408	.	.	1
24758	0	5.56	1	0.80408	.	.	1
24759	0	5.56	1	0.80408	.	.	1
24760	0	5.56	1	0.80408	.	.	1
24761	0	5.56	1	0.80408	.	.	1
24762	0	5.56	1	0.80408	.	.	1
24763	0	5.56	1	0.80408	.	.	1
24764	0	5.56	1	0.80408	.	.	1
24765	0	5.56	1	0.80408	.	.	1
24766	0	5.57	0	0.80403	0.79973	0.80825	1
24767	0	5.57	1	0.80403	.	.	1
24768	0	5.57	1	0.80403	.	.	1
24769	0	5.57	1	0.80403	.	.	1
24770	0	5.57	1	0.80403	.	.	1
24771	0	5.57	1	0.80403	.	.	1
24772	0	5.57	1	0.80403	.	.	1
24773	0	5.57	1	0.80403	.	.	1
24774	0	5.57	1	0.80403	.	.	1
24775	0	5.57	1	0.80403	.	.	1
24776	0	5.57	1	0.80403	.	.	1
24777	0	5.57	1	0.80403	.	.	1
24778	0	5.57	1	0.80403	.	.	1
24779	0	5.57	1	0.80403	.	.	1
24780	0	5.57	1	0.80403	.	.	1

Obs	Sub_group	fuptimey	_CENSOR_	SURVIVAL	SDF_LCL	SDF_UCL	STRATUM
24781	0	5.57	1	0.80403	.	.	1
24782	0	5.57	1	0.80403	.	.	1
24783	0	5.57	1	0.80403	.	.	1
24784	0	5.57	1	0.80403	.	.	1
24785	0	5.57	1	0.80403	.	.	1
24786	0	5.57	1	0.80403	.	.	1
24787	0	5.57	1	0.80403	.	.	1
24788	0	5.57	1	0.80403	.	.	1
24789	0	5.57	1	0.80403	.	.	1
24790	0	5.57	1	0.80403	.	.	1
24791	0	5.57	1	0.80403	.	.	1
24792	0	5.57	1	0.80403	.	.	1
24793	0	5.57	1	0.80403	.	.	1
24794	0	5.57	1	0.80403	.	.	1
24795	0	5.57	1	0.80403	.	.	1
24796	0	5.57	1	0.80403	.	.	1
24797	0	5.57	1	0.80403	.	.	1
24798	0	5.57	1	0.80403	.	.	1
24799	0	5.57	1	0.80403	.	.	1
24800	0	5.57	1	0.80403	.	.	1
24801	0	5.57	1	0.80403	.	.	1
24802	0	5.57	1	0.80403	.	.	1
24803	0	5.57	1	0.80403	.	.	1
24804	0	5.57	1	0.80403	.	.	1
24805	0	5.58	0	0.80387	0.79957	0.80810	1
24806	0	5.58	1	0.80387	.	.	1
24807	0	5.58	1	0.80387	.	.	1
24808	0	5.58	1	0.80387	.	.	1
24809	0	5.58	1	0.80387	.	.	1
24810	0	5.58	1	0.80387	.	.	1
24811	0	5.58	1	0.80387	.	.	1
24812	0	5.58	1	0.80387	.	.	1
24813	0	5.58	1	0.80387	.	.	1
24814	0	5.58	1	0.80387	.	.	1
24815	0	5.58	1	0.80387	.	.	1

Obs	Sub_group	fuptimey	_CENSOR_	SURVIVAL	SDF_LCL	SDF_UCL	STRATUM
24816	0	5.58	1	0.80387	.	.	1
24817	0	5.58	1	0.80387	.	.	1
24818	0	5.58	1	0.80387	.	.	1
24819	0	5.58	1	0.80387	.	.	1
24820	0	5.58	1	0.80387	.	.	1
24821	0	5.58	1	0.80387	.	.	1
24822	0	5.58	1	0.80387	.	.	1
24823	0	5.58	1	0.80387	.	.	1
24824	0	5.58	1	0.80387	.	.	1
24825	0	5.58	1	0.80387	.	.	1
24826	0	5.58	1	0.80387	.	.	1
24827	0	5.58	1	0.80387	.	.	1
24828	0	5.58	1	0.80387	.	.	1
24829	0	5.58	1	0.80387	.	.	1
24830	0	5.58	1	0.80387	.	.	1
24831	0	5.58	1	0.80387	.	.	1
24832	0	5.58	1	0.80387	.	.	1
24833	0	5.58	1	0.80387	.	.	1
24834	0	5.58	1	0.80387	.	.	1
24835	0	5.58	1	0.80387	.	.	1
24836	0	5.58	1	0.80387	.	.	1
24837	0	5.58	1	0.80387	.	.	1
24838	0	5.58	1	0.80387	.	.	1
24839	0	5.58	1	0.80387	.	.	1
24840	0	5.58	1	0.80387	.	.	1
24841	0	5.58	1	0.80387	.	.	1
24842	0	5.58	1	0.80387	.	.	1
24843	0	5.58	1	0.80387	.	.	1
24844	0	5.58	1	0.80387	.	.	1
24845	0	5.58	1	0.80387	.	.	1
24846	0	5.58	1	0.80387	.	.	1
24847	0	5.58	1	0.80387	.	.	1
24848	0	5.58	1	0.80387	.	.	1
24849	0	5.58	1	0.80387	.	.	1
24850	0	5.58	1	0.80387	.	.	1

Obs	Sub_group	fuptimey	_CENSOR_	SURVIVAL	SDF_LCL	SDF_UCL	STRATUM
24851	0	5.58	1	0.80387	.	.	1
24852	0	5.58	1	0.80387	.	.	1
24853	0	5.58	1	0.80387	.	.	1
24854	0	5.58	1	0.80387	.	.	1
24855	0	5.58	1	0.80387	.	.	1
24856	0	5.58	1	0.80387	.	.	1
24857	0	5.58	1	0.80387	.	.	1
24858	0	5.59	0	0.80366	0.79935	0.80789	1
24859	0	5.59	1	0.80366	.	.	1
24860	0	5.59	1	0.80366	.	.	1
24861	0	5.59	1	0.80366	.	.	1
24862	0	5.59	1	0.80366	.	.	1
24863	0	5.59	1	0.80366	.	.	1
24864	0	5.59	1	0.80366	.	.	1
24865	0	5.59	1	0.80366	.	.	1
24866	0	5.59	1	0.80366	.	.	1
24867	0	5.59	1	0.80366	.	.	1
24868	0	5.59	1	0.80366	.	.	1
24869	0	5.59	1	0.80366	.	.	1
24870	0	5.59	1	0.80366	.	.	1
24871	0	5.59	1	0.80366	.	.	1
24872	0	5.59	1	0.80366	.	.	1
24873	0	5.59	1	0.80366	.	.	1
24874	0	5.59	1	0.80366	.	.	1
24875	0	5.59	1	0.80366	.	.	1
24876	0	5.59	1	0.80366	.	.	1
24877	0	5.59	1	0.80366	.	.	1
24878	0	5.59	1	0.80366	.	.	1
24879	0	5.59	1	0.80366	.	.	1
24880	0	5.59	1	0.80366	.	.	1
24881	0	5.59	1	0.80366	.	.	1
24882	0	5.59	1	0.80366	.	.	1
24883	0	5.59	1	0.80366	.	.	1
24884	0	5.59	1	0.80366	.	.	1
24885	0	5.59	1	0.80366	.	.	1

Obs	Sub_group	fuptimey	_CENSOR_	SURVIVAL	SDF_LCL	SDF_UCL	STRATUM
24886	0	5.59	1	0.80366	.	.	1
24887	0	5.59	1	0.80366	.	.	1
24888	0	5.59	1	0.80366	.	.	1
24889	0	5.60	0	0.80345	0.79913	0.80768	1
24890	0	5.60	1	0.80345	.	.	1
24891	0	5.60	1	0.80345	.	.	1
24892	0	5.60	1	0.80345	.	.	1
24893	0	5.60	1	0.80345	.	.	1
24894	0	5.60	1	0.80345	.	.	1
24895	0	5.60	1	0.80345	.	.	1
24896	0	5.60	1	0.80345	.	.	1
24897	0	5.60	1	0.80345	.	.	1
24898	0	5.60	1	0.80345	.	.	1
24899	0	5.60	1	0.80345	.	.	1
24900	0	5.60	1	0.80345	.	.	1
24901	0	5.60	1	0.80345	.	.	1
24902	0	5.60	1	0.80345	.	.	1
24903	0	5.60	1	0.80345	.	.	1
24904	0	5.60	1	0.80345	.	.	1
24905	0	5.60	1	0.80345	.	.	1
24906	0	5.60	1	0.80345	.	.	1
24907	0	5.60	1	0.80345	.	.	1
24908	0	5.60	1	0.80345	.	.	1
24909	0	5.60	1	0.80345	.	.	1
24910	0	5.60	1	0.80345	.	.	1
24911	0	5.60	1	0.80345	.	.	1
24912	0	5.60	1	0.80345	.	.	1
24913	0	5.60	1	0.80345	.	.	1
24914	0	5.60	1	0.80345	.	.	1
24915	0	5.60	1	0.80345	.	.	1
24916	0	5.60	1	0.80345	.	.	1
24917	0	5.60	1	0.80345	.	.	1
24918	0	5.60	1	0.80345	.	.	1
24919	0	5.60	1	0.80345	.	.	1
24920	0	5.60	1	0.80345	.	.	1

Obs	Sub_group	fuptimey	_CENSOR_	SURVIVAL	SDF_LCL	SDF_UCL	STRATUM
24921	0	5.60	1	0.80345	.	.	1
24922	0	5.60	1	0.80345	.	.	1
24923	0	5.60	1	0.80345	.	.	1
24924	0	5.60	1	0.80345	.	.	1
24925	0	5.60	1	0.80345	.	.	1
24926	0	5.60	1	0.80345	.	.	1
24927	0	5.60	1	0.80345	.	.	1
24928	0	5.60	1	0.80345	.	.	1
24929	0	5.60	1	0.80345	.	.	1
24930	0	5.60	1	0.80345	.	.	1
24931	0	5.61	0	0.80313	0.79881	0.80736	1
24932	0	5.61	1	0.80313	.	.	1
24933	0	5.61	1	0.80313	.	.	1
24934	0	5.61	1	0.80313	.	.	1
24935	0	5.61	1	0.80313	.	.	1
24936	0	5.61	1	0.80313	.	.	1
24937	0	5.61	1	0.80313	.	.	1
24938	0	5.61	1	0.80313	.	.	1
24939	0	5.61	1	0.80313	.	.	1
24940	0	5.61	1	0.80313	.	.	1
24941	0	5.61	1	0.80313	.	.	1
24942	0	5.61	1	0.80313	.	.	1
24943	0	5.61	1	0.80313	.	.	1
24944	0	5.61	1	0.80313	.	.	1
24945	0	5.61	1	0.80313	.	.	1
24946	0	5.61	1	0.80313	.	.	1
24947	0	5.61	1	0.80313	.	.	1
24948	0	5.61	1	0.80313	.	.	1
24949	0	5.61	1	0.80313	.	.	1
24950	0	5.61	1	0.80313	.	.	1
24951	0	5.61	1	0.80313	.	.	1
24952	0	5.61	1	0.80313	.	.	1
24953	0	5.61	1	0.80313	.	.	1
24954	0	5.61	1	0.80313	.	.	1
24955	0	5.61	1	0.80313	.	.	1

Obs	Sub_group	fuptimey	_CENSOR_	SURVIVAL	SDF_LCL	SDF_UCL	STRATUM
24956	0	5.61	1	0.80313	.	.	1
24957	0	5.61	1	0.80313	.	.	1
24958	0	5.61	1	0.80313	.	.	1
24959	0	5.62	0	0.80296	0.79864	0.80720	1
24960	0	5.62	1	0.80296	.	.	1
24961	0	5.62	1	0.80296	.	.	1
24962	0	5.62	1	0.80296	.	.	1
24963	0	5.62	1	0.80296	.	.	1
24964	0	5.62	1	0.80296	.	.	1
24965	0	5.62	1	0.80296	.	.	1
24966	0	5.62	1	0.80296	.	.	1
24967	0	5.62	1	0.80296	.	.	1
24968	0	5.62	1	0.80296	.	.	1
24969	0	5.62	1	0.80296	.	.	1
24970	0	5.62	1	0.80296	.	.	1
24971	0	5.62	1	0.80296	.	.	1
24972	0	5.62	1	0.80296	.	.	1
24973	0	5.62	1	0.80296	.	.	1
24974	0	5.62	1	0.80296	.	.	1
24975	0	5.62	1	0.80296	.	.	1
24976	0	5.62	1	0.80296	.	.	1
24977	0	5.62	1	0.80296	.	.	1
24978	0	5.62	1	0.80296	.	.	1
24979	0	5.62	1	0.80296	.	.	1
24980	0	5.62	1	0.80296	.	.	1
24981	0	5.62	1	0.80296	.	.	1
24982	0	5.62	1	0.80296	.	.	1
24983	0	5.62	1	0.80296	.	.	1
24984	0	5.62	1	0.80296	.	.	1
24985	0	5.62	1	0.80296	.	.	1
24986	0	5.62	1	0.80296	.	.	1
24987	0	5.62	1	0.80296	.	.	1
24988	0	5.62	1	0.80296	.	.	1
24989	0	5.62	1	0.80296	.	.	1
24990	0	5.62	1	0.80296	.	.	1

Obs	Sub_group	fuptimey	_CENSOR_	SURVIVAL	SDF_LCL	SDF_UCL	STRATUM
**24991**	0	5.62	1	0.80296	.	.	1
**24992**	0	5.62	1	0.80296	.	.	1
**24993**	0	5.62	1	0.80296	.	.	1
**24994**	0	5.62	1	0.80296	.	.	1
**24995**	0	5.62	1	0.80296	.	.	1
**24996**	0	5.62	1	0.80296	.	.	1
**24997**	0	5.62	1	0.80296	.	.	1
**24998**	0	5.62	1	0.80296	.	.	1
**24999**	0	5.63	0	0.80280	0.79848	0.80705	1
**25000**	0	5.63	1	0.80280	.	.	1
**25001**	0	5.63	1	0.80280	.	.	1
**25002**	0	5.63	1	0.80280	.	.	1
**25003**	0	5.63	1	0.80280	.	.	1
**25004**	0	5.63	1	0.80280	.	.	1
**25005**	0	5.63	1	0.80280	.	.	1
**25006**	0	5.63	1	0.80280	.	.	1
**25007**	0	5.63	1	0.80280	.	.	1
**25008**	0	5.63	1	0.80280	.	.	1
**25009**	0	5.63	1	0.80280	.	.	1
**25010**	0	5.63	1	0.80280	.	.	1
**25011**	0	5.63	1	0.80280	.	.	1
**25012**	0	5.63	1	0.80280	.	.	1
**25013**	0	5.63	1	0.80280	.	.	1
**25014**	0	5.63	1	0.80280	.	.	1
**25015**	0	5.63	1	0.80280	.	.	1
**25016**	0	5.63	1	0.80280	.	.	1
**25017**	0	5.63	1	0.80280	.	.	1
**25018**	0	5.63	1	0.80280	.	.	1
**25019**	0	5.63	1	0.80280	.	.	1
**25020**	0	5.63	1	0.80280	.	.	1
**25021**	0	5.63	1	0.80280	.	.	1
**25022**	0	5.63	1	0.80280	.	.	1
**25023**	0	5.63	1	0.80280	.	.	1
**25024**	0	5.63	1	0.80280	.	.	1
**25025**	0	5.63	1	0.80280	.	.	1

Obs	Sub_group	fuptimey	_CENSOR_	SURVIVAL	SDF_LCL	SDF_UCL	STRATUM
25026	0	5.63	1	0.80280	.	.	1
25027	0	5.63	1	0.80280	.	.	1
25028	0	5.63	1	0.80280	.	.	1
25029	0	5.63	1	0.80280	.	.	1
25030	0	5.63	1	0.80280	.	.	1
25031	0	5.63	1	0.80280	.	.	1
25032	0	5.64	0	0.80264	0.79831	0.80689	1
25033	0	5.64	1	0.80264	.	.	1
25034	0	5.64	1	0.80264	.	.	1
25035	0	5.64	1	0.80264	.	.	1
25036	0	5.64	1	0.80264	.	.	1
25037	0	5.64	1	0.80264	.	.	1
25038	0	5.64	1	0.80264	.	.	1
25039	0	5.64	1	0.80264	.	.	1
25040	0	5.64	1	0.80264	.	.	1
25041	0	5.64	1	0.80264	.	.	1
25042	0	5.64	1	0.80264	.	.	1
25043	0	5.64	1	0.80264	.	.	1
25044	0	5.64	1	0.80264	.	.	1
25045	0	5.64	1	0.80264	.	.	1
25046	0	5.64	1	0.80264	.	.	1
25047	0	5.64	1	0.80264	.	.	1
25048	0	5.64	1	0.80264	.	.	1
25049	0	5.64	1	0.80264	.	.	1
25050	0	5.64	1	0.80264	.	.	1
25051	0	5.64	1	0.80264	.	.	1
25052	0	5.64	1	0.80264	.	.	1
25053	0	5.64	1	0.80264	.	.	1
25054	0	5.64	1	0.80264	.	.	1
25055	0	5.64	1	0.80264	.	.	1
25056	0	5.64	1	0.80264	.	.	1
25057	0	5.64	1	0.80264	.	.	1
25058	0	5.64	1	0.80264	.	.	1
25059	0	5.64	1	0.80264	.	.	1
25060	0	5.64	1	0.80264	.	.	1

Obs	Sub_group	fuptimey	_CENSOR_	SURVIVAL	SDF_LCL	SDF_UCL	STRATUM
25061	0	5.64	1	0.80264	.	.	1
25062	0	5.64	1	0.80264	.	.	1
25063	0	5.64	1	0.80264	.	.	1
25064	0	5.64	1	0.80264	.	.	1
25065	0	5.64	1	0.80264	.	.	1
25066	0	5.64	1	0.80264	.	.	1
25067	0	5.64	1	0.80264	.	.	1
25068	0	5.64	1	0.80264	.	.	1
25069	0	5.64	1	0.80264	.	.	1
25070	0	5.64	1	0.80264	.	.	1
25071	0	5.64	1	0.80264	.	.	1
25072	0	5.64	1	0.80264	.	.	1
25073	0	5.64	1	0.80264	.	.	1
25074	0	5.64	1	0.80264	.	.	1
25075	0	5.65	0	0.80248	0.79815	0.80673	1
25076	0	5.65	1	0.80248	.	.	1
25077	0	5.65	1	0.80248	.	.	1
25078	0	5.65	1	0.80248	.	.	1
25079	0	5.65	1	0.80248	.	.	1
25080	0	5.65	1	0.80248	.	.	1
25081	0	5.65	1	0.80248	.	.	1
25082	0	5.65	1	0.80248	.	.	1
25083	0	5.65	1	0.80248	.	.	1
25084	0	5.65	1	0.80248	.	.	1
25085	0	5.65	1	0.80248	.	.	1
25086	0	5.65	1	0.80248	.	.	1
25087	0	5.65	1	0.80248	.	.	1
25088	0	5.65	1	0.80248	.	.	1
25089	0	5.65	1	0.80248	.	.	1
25090	0	5.65	1	0.80248	.	.	1
25091	0	5.65	1	0.80248	.	.	1
25092	0	5.65	1	0.80248	.	.	1
25093	0	5.65	1	0.80248	.	.	1
25094	0	5.65	1	0.80248	.	.	1
25095	0	5.65	1	0.80248	.	.	1

Obs	Sub_group	fuptimey	_CENSOR_	SURVIVAL	SDF_LCL	SDF_UCL	STRATUM
**25096**	0	5.65	1	0.80248	.	.	1
**25097**	0	5.65	1	0.80248	.	.	1
**25098**	0	5.65	1	0.80248	.	.	1
**25099**	0	5.65	1	0.80248	.	.	1
**25100**	0	5.65	1	0.80248	.	.	1
**25101**	0	5.65	1	0.80248	.	.	1
**25102**	0	5.65	1	0.80248	.	.	1
**25103**	0	5.66	0	0.80232	0.79798	0.80657	1
**25104**	0	5.66	1	0.80232	.	.	1
**25105**	0	5.66	1	0.80232	.	.	1
**25106**	0	5.66	1	0.80232	.	.	1
**25107**	0	5.66	1	0.80232	.	.	1
**25108**	0	5.66	1	0.80232	.	.	1
**25109**	0	5.66	1	0.80232	.	.	1
**25110**	0	5.66	1	0.80232	.	.	1
**25111**	0	5.66	1	0.80232	.	.	1
**25112**	0	5.66	1	0.80232	.	.	1
**25113**	0	5.66	1	0.80232	.	.	1
**25114**	0	5.66	1	0.80232	.	.	1
**25115**	0	5.66	1	0.80232	.	.	1
**25116**	0	5.66	1	0.80232	.	.	1
**25117**	0	5.66	1	0.80232	.	.	1
**25118**	0	5.66	1	0.80232	.	.	1
**25119**	0	5.66	1	0.80232	.	.	1
**25120**	0	5.66	1	0.80232	.	.	1
**25121**	0	5.66	1	0.80232	.	.	1
**25122**	0	5.66	1	0.80232	.	.	1
**25123**	0	5.66	1	0.80232	.	.	1
**25124**	0	5.66	1	0.80232	.	.	1
**25125**	0	5.66	1	0.80232	.	.	1
**25126**	0	5.66	1	0.80232	.	.	1
**25127**	0	5.66	1	0.80232	.	.	1
**25128**	0	5.66	1	0.80232	.	.	1
**25129**	0	5.66	1	0.80232	.	.	1
**25130**	0	5.66	1	0.80232	.	.	1

Obs	Sub_group	fuptimey	_CENSOR_	SURVIVAL	SDF_LCL	SDF_UCL	STRATUM
25131	0	5.66	1	0.80232	.	.	1
25132	0	5.66	1	0.80232	.	.	1
25133	0	5.66	1	0.80232	.	.	1
25134	0	5.66	1	0.80232	.	.	1
25135	0	5.66	1	0.80232	.	.	1
25136	0	5.66	1	0.80232	.	.	1
25137	0	5.66	1	0.80232	.	.	1
25138	0	5.66	1	0.80232	.	.	1
25139	0	5.66	1	0.80232	.	.	1
25140	0	5.66	1	0.80232	.	.	1
25141	0	5.66	1	0.80232	.	.	1
25142	0	5.66	1	0.80232	.	.	1
25143	0	5.66	1	0.80232	.	.	1
25144	0	5.66	1	0.80232	.	.	1
25145	0	5.66	1	0.80232	.	.	1
25146	0	5.66	1	0.80232	.	.	1
25147	0	5.66	1	0.80232	.	.	1
25148	0	5.67	0	0.80221	0.79787	0.80646	1
25149	0	5.67	1	0.80221	.	.	1
25150	0	5.67	1	0.80221	.	.	1
25151	0	5.67	1	0.80221	.	.	1
25152	0	5.67	1	0.80221	.	.	1
25153	0	5.67	1	0.80221	.	.	1
25154	0	5.67	1	0.80221	.	.	1
25155	0	5.67	1	0.80221	.	.	1
25156	0	5.67	1	0.80221	.	.	1
25157	0	5.67	1	0.80221	.	.	1
25158	0	5.67	1	0.80221	.	.	1
25159	0	5.67	1	0.80221	.	.	1
25160	0	5.67	1	0.80221	.	.	1
25161	0	5.67	1	0.80221	.	.	1
25162	0	5.67	1	0.80221	.	.	1
25163	0	5.67	1	0.80221	.	.	1
25164	0	5.67	1	0.80221	.	.	1
25165	0	5.67	1	0.80221	.	.	1

Obs	Sub_group	fuptimey	_CENSOR_	SURVIVAL	SDF_LCL	SDF_UCL	STRATUM
25166	0	5.67	1	0.80221	.	.	1
25167	0	5.67	1	0.80221	.	.	1
25168	0	5.67	1	0.80221	.	.	1
25169	0	5.67	1	0.80221	.	.	1
25170	0	5.67	1	0.80221	.	.	1
25171	0	5.67	1	0.80221	.	.	1
25172	0	5.67	1	0.80221	.	.	1
25173	0	5.67	1	0.80221	.	.	1
25174	0	5.67	1	0.80221	.	.	1
25175	0	5.67	1	0.80221	.	.	1
25176	0	5.67	1	0.80221	.	.	1
25177	0	5.67	1	0.80221	.	.	1
25178	0	5.68	0	0.80216	0.79782	0.80641	1
25179	0	5.68	1	0.80216	.	.	1
25180	0	5.68	1	0.80216	.	.	1
25181	0	5.68	1	0.80216	.	.	1
25182	0	5.68	1	0.80216	.	.	1
25183	0	5.68	1	0.80216	.	.	1
25184	0	5.68	1	0.80216	.	.	1
25185	0	5.68	1	0.80216	.	.	1
25186	0	5.68	1	0.80216	.	.	1
25187	0	5.68	1	0.80216	.	.	1
25188	0	5.68	1	0.80216	.	.	1
25189	0	5.68	1	0.80216	.	.	1
25190	0	5.68	1	0.80216	.	.	1
25191	0	5.68	1	0.80216	.	.	1
25192	0	5.68	1	0.80216	.	.	1
25193	0	5.68	1	0.80216	.	.	1
25194	0	5.68	1	0.80216	.	.	1
25195	0	5.68	1	0.80216	.	.	1
25196	0	5.68	1	0.80216	.	.	1
25197	0	5.68	1	0.80216	.	.	1
25198	0	5.68	1	0.80216	.	.	1
25199	0	5.68	1	0.80216	.	.	1
25200	0	5.68	1	0.80216	.	.	1

Obs	Sub_group	fuptimey	_CENSOR_	SURVIVAL	SDF_LCL	SDF_UCL	STRATUM
25201	0	5.68	1	0.80216	.	.	1
25202	0	5.68	1	0.80216	.	.	1
25203	0	5.68	1	0.80216	.	.	1
25204	0	5.68	1	0.80216	.	.	1
25205	0	5.68	1	0.80216	.	.	1
25206	0	5.68	1	0.80216	.	.	1
25207	0	5.68	1	0.80216	.	.	1
25208	0	5.68	1	0.80216	.	.	1
25209	0	5.68	1	0.80216	.	.	1
25210	0	5.68	1	0.80216	.	.	1
25211	0	5.68	1	0.80216	.	.	1
25212	0	5.68	1	0.80216	.	.	1
25213	0	5.68	1	0.80216	.	.	1
25214	0	5.68	1	0.80216	.	.	1
25215	0	5.69	0	0.80205	0.79771	0.80630	1
25216	0	5.69	1	0.80205	.	.	1
25217	0	5.69	1	0.80205	.	.	1
25218	0	5.69	1	0.80205	.	.	1
25219	0	5.69	1	0.80205	.	.	1
25220	0	5.69	1	0.80205	.	.	1
25221	0	5.69	1	0.80205	.	.	1
25222	0	5.69	1	0.80205	.	.	1
25223	0	5.69	1	0.80205	.	.	1
25224	0	5.69	1	0.80205	.	.	1
25225	0	5.69	1	0.80205	.	.	1
25226	0	5.69	1	0.80205	.	.	1
25227	0	5.69	1	0.80205	.	.	1
25228	0	5.69	1	0.80205	.	.	1
25229	0	5.69	1	0.80205	.	.	1
25230	0	5.69	1	0.80205	.	.	1
25231	0	5.69	1	0.80205	.	.	1
25232	0	5.69	1	0.80205	.	.	1
25233	0	5.69	1	0.80205	.	.	1
25234	0	5.69	1	0.80205	.	.	1
25235	0	5.69	1	0.80205	.	.	1

Obs	Sub_group	fuptimey	_CENSOR_	SURVIVAL	SDF_LCL	SDF_UCL	STRATUM
**25236**	0	5.69	1	0.80205	.	.	1
**25237**	0	5.69	1	0.80205	.	.	1
**25238**	0	5.69	1	0.80205	.	.	1
**25239**	0	5.69	1	0.80205	.	.	1
**25240**	0	5.69	1	0.80205	.	.	1
**25241**	0	5.69	1	0.80205	.	.	1
**25242**	0	5.69	1	0.80205	.	.	1
**25243**	0	5.69	1	0.80205	.	.	1
**25244**	0	5.70	0	0.80183	0.79749	0.80609	1
**25245**	0	5.70	1	0.80183	.	.	1
**25246**	0	5.70	1	0.80183	.	.	1
**25247**	0	5.70	1	0.80183	.	.	1
**25248**	0	5.70	1	0.80183	.	.	1
**25249**	0	5.70	1	0.80183	.	.	1
**25250**	0	5.70	1	0.80183	.	.	1
**25251**	0	5.70	1	0.80183	.	.	1
**25252**	0	5.70	1	0.80183	.	.	1
**25253**	0	5.70	1	0.80183	.	.	1
**25254**	0	5.70	1	0.80183	.	.	1
**25255**	0	5.70	1	0.80183	.	.	1
**25256**	0	5.70	1	0.80183	.	.	1
**25257**	0	5.70	1	0.80183	.	.	1
**25258**	0	5.70	1	0.80183	.	.	1
**25259**	0	5.70	1	0.80183	.	.	1
**25260**	0	5.70	1	0.80183	.	.	1
**25261**	0	5.70	1	0.80183	.	.	1
**25262**	0	5.70	1	0.80183	.	.	1
**25263**	0	5.70	1	0.80183	.	.	1
**25264**	0	5.70	1	0.80183	.	.	1
**25265**	0	5.70	1	0.80183	.	.	1
**25266**	0	5.70	1	0.80183	.	.	1
**25267**	0	5.70	1	0.80183	.	.	1
**25268**	0	5.70	1	0.80183	.	.	1
**25269**	0	5.70	1	0.80183	.	.	1
**25270**	0	5.70	1	0.80183	.	.	1

Obs	Sub_group	fuptimey	_CENSOR_	SURVIVAL	SDF_LCL	SDF_UCL	STRATUM
25271	0	5.70	1	0.80183	.	.	1
25272	0	5.70	1	0.80183	.	.	1
25273	0	5.70	1	0.80183	.	.	1
25274	0	5.70	1	0.80183	.	.	1
25275	0	5.70	1	0.80183	.	.	1
25276	0	5.70	1	0.80183	.	.	1
25277	0	5.70	1	0.80183	.	.	1
25278	0	5.70	1	0.80183	.	.	1
25279	0	5.70	1	0.80183	.	.	1
25280	0	5.70	1	0.80183	.	.	1
25281	0	5.70	1	0.80183	.	.	1
25282	0	5.70	1	0.80183	.	.	1
25283	0	5.70	1	0.80183	.	.	1
25284	0	5.70	1	0.80183	.	.	1
25285	0	5.71	0	0.80177	0.79743	0.80604	1
25286	0	5.71	1	0.80177	.	.	1
25287	0	5.71	1	0.80177	.	.	1
25288	0	5.71	1	0.80177	.	.	1
25289	0	5.71	1	0.80177	.	.	1
25290	0	5.71	1	0.80177	.	.	1
25291	0	5.71	1	0.80177	.	.	1
25292	0	5.71	1	0.80177	.	.	1
25293	0	5.71	1	0.80177	.	.	1
25294	0	5.71	1	0.80177	.	.	1
25295	0	5.71	1	0.80177	.	.	1
25296	0	5.71	1	0.80177	.	.	1
25297	0	5.71	1	0.80177	.	.	1
25298	0	5.71	1	0.80177	.	.	1
25299	0	5.71	1	0.80177	.	.	1
25300	0	5.71	1	0.80177	.	.	1
25301	0	5.71	1	0.80177	.	.	1
25302	0	5.71	1	0.80177	.	.	1
25303	0	5.71	1	0.80177	.	.	1
25304	0	5.71	1	0.80177	.	.	1
25305	0	5.71	1	0.80177	.	.	1

Obs	Sub_group	fuptimey	_CENSOR_	SURVIVAL	SDF_LCL	SDF_UCL	STRATUM
25306	0	5.71	1	0.80177	.	.	1
25307	0	5.71	1	0.80177	.	.	1
25308	0	5.71	1	0.80177	.	.	1
25309	0	5.71	1	0.80177	.	.	1
25310	0	5.72	0	0.80161	0.79726	0.80587	1
25311	0	5.72	1	0.80161	.	.	1
25312	0	5.72	1	0.80161	.	.	1
25313	0	5.72	1	0.80161	.	.	1
25314	0	5.72	1	0.80161	.	.	1
25315	0	5.72	1	0.80161	.	.	1
25316	0	5.72	1	0.80161	.	.	1
25317	0	5.72	1	0.80161	.	.	1
25318	0	5.72	1	0.80161	.	.	1
25319	0	5.72	1	0.80161	.	.	1
25320	0	5.72	1	0.80161	.	.	1
25321	0	5.72	1	0.80161	.	.	1
25322	0	5.72	1	0.80161	.	.	1
25323	0	5.72	1	0.80161	.	.	1
25324	0	5.72	1	0.80161	.	.	1
25325	0	5.72	1	0.80161	.	.	1
25326	0	5.72	1	0.80161	.	.	1
25327	0	5.72	1	0.80161	.	.	1
25328	0	5.72	1	0.80161	.	.	1
25329	0	5.72	1	0.80161	.	.	1
25330	0	5.72	1	0.80161	.	.	1
25331	0	5.72	1	0.80161	.	.	1
25332	0	5.72	1	0.80161	.	.	1
25333	0	5.72	1	0.80161	.	.	1
25334	0	5.72	1	0.80161	.	.	1
25335	0	5.72	1	0.80161	.	.	1
25336	0	5.72	1	0.80161	.	.	1
25337	0	5.72	1	0.80161	.	.	1
25338	0	5.72	1	0.80161	.	.	1
25339	0	5.72	1	0.80161	.	.	1
25340	0	5.72	1	0.80161	.	.	1

Obs	Sub_group	fuptimey	_CENSOR_	SURVIVAL	SDF_LCL	SDF_UCL	STRATUM
25341	0	5.72	1	0.80161	.	.	1
25342	0	5.72	1	0.80161	.	.	1
25343	0	5.72	1	0.80161	.	.	1
25344	0	5.72	1	0.80161	.	.	1
25345	0	5.72	1	0.80161	.	.	1
25346	0	5.72	1	0.80161	.	.	1
25347	0	5.72	1	0.80161	.	.	1
25348	0	5.72	1	0.80161	.	.	1
25349	0	5.72	1	0.80161	.	.	1
25350	0	5.72	1	0.80161	.	.	1
25351	0	5.72	1	0.80161	.	.	1
25352	0	5.72	1	0.80161	.	.	1
25353	0	5.72	1	0.80161	.	.	1
25354	0	5.72	1	0.80161	.	.	1
25355	0	5.72	1	0.80161	.	.	1
25356	0	5.72	1	0.80161	.	.	1
25357	0	5.72	1	0.80161	.	.	1
25358	0	5.73	0	0.80128	0.79693	0.80555	1
25359	0	5.73	1	0.80128	.	.	1
25360	0	5.73	1	0.80128	.	.	1
25361	0	5.73	1	0.80128	.	.	1
25362	0	5.73	1	0.80128	.	.	1
25363	0	5.73	1	0.80128	.	.	1
25364	0	5.73	1	0.80128	.	.	1
25365	0	5.73	1	0.80128	.	.	1
25366	0	5.73	1	0.80128	.	.	1
25367	0	5.73	1	0.80128	.	.	1
25368	0	5.73	1	0.80128	.	.	1
25369	0	5.73	1	0.80128	.	.	1
25370	0	5.73	1	0.80128	.	.	1
25371	0	5.73	1	0.80128	.	.	1
25372	0	5.73	1	0.80128	.	.	1
25373	0	5.73	1	0.80128	.	.	1
25374	0	5.73	1	0.80128	.	.	1
25375	0	5.73	1	0.80128	.	.	1

Obs	Sub_group	fuptimey	_CENSOR_	SURVIVAL	SDF_LCL	SDF_UCL	STRATUM
25376	0	5.73	1	0.80128	.	.	1
25377	0	5.73	1	0.80128	.	.	1
25378	0	5.73	1	0.80128	.	.	1
25379	0	5.73	1	0.80128	.	.	1
25380	0	5.73	1	0.80128	.	.	1
25381	0	5.73	1	0.80128	.	.	1
25382	0	5.73	1	0.80128	.	.	1
25383	0	5.73	1	0.80128	.	.	1
25384	0	5.73	1	0.80128	.	.	1
25385	0	5.73	1	0.80128	.	.	1
25386	0	5.73	1	0.80128	.	.	1
25387	0	5.73	1	0.80128	.	.	1
25388	0	5.73	1	0.80128	.	.	1
25389	0	5.73	1	0.80128	.	.	1
25390	0	5.73	1	0.80128	.	.	1
25391	0	5.73	1	0.80128	.	.	1
25392	0	5.74	0	0.80089	0.79653	0.80517	1
25393	0	5.74	1	0.80089	.	.	1
25394	0	5.74	1	0.80089	.	.	1
25395	0	5.74	1	0.80089	.	.	1
25396	0	5.74	1	0.80089	.	.	1
25397	0	5.74	1	0.80089	.	.	1
25398	0	5.74	1	0.80089	.	.	1
25399	0	5.74	1	0.80089	.	.	1
25400	0	5.74	1	0.80089	.	.	1
25401	0	5.74	1	0.80089	.	.	1
25402	0	5.74	1	0.80089	.	.	1
25403	0	5.74	1	0.80089	.	.	1
25404	0	5.74	1	0.80089	.	.	1
25405	0	5.74	1	0.80089	.	.	1
25406	0	5.74	1	0.80089	.	.	1
25407	0	5.74	1	0.80089	.	.	1
25408	0	5.74	1	0.80089	.	.	1
25409	0	5.74	1	0.80089	.	.	1
25410	0	5.74	1	0.80089	.	.	1

Obs	Sub_group	fuptimey	_CENSOR_	SURVIVAL	SDF_LCL	SDF_UCL	STRATUM
**25411**	0	5.74	1	0.80089	.	.	1
**25412**	0	5.74	1	0.80089	.	.	1
**25413**	0	5.74	1	0.80089	.	.	1
**25414**	0	5.74	1	0.80089	.	.	1
**25415**	0	5.74	1	0.80089	.	.	1
**25416**	0	5.74	1	0.80089	.	.	1
**25417**	0	5.74	1	0.80089	.	.	1
**25418**	0	5.74	1	0.80089	.	.	1
**25419**	0	5.74	1	0.80089	.	.	1
**25420**	0	5.74	1	0.80089	.	.	1
**25421**	0	5.74	1	0.80089	.	.	1
**25422**	0	5.74	1	0.80089	.	.	1
**25423**	0	5.74	1	0.80089	.	.	1
**25424**	0	5.74	1	0.80089	.	.	1
**25425**	0	5.74	1	0.80089	.	.	1
**25426**	0	5.74	1	0.80089	.	.	1
**25427**	0	5.74	1	0.80089	.	.	1
**25428**	0	5.74	1	0.80089	.	.	1
**25429**	0	5.74	1	0.80089	.	.	1
**25430**	0	5.74	1	0.80089	.	.	1
**25431**	0	5.74	1	0.80089	.	.	1
**25432**	0	5.74	1	0.80089	.	.	1
**25433**	0	5.75	0	0.80067	0.79631	0.80496	1
**25434**	0	5.75	1	0.80067	.	.	1
**25435**	0	5.75	1	0.80067	.	.	1
**25436**	0	5.75	1	0.80067	.	.	1
**25437**	0	5.75	1	0.80067	.	.	1
**25438**	0	5.75	1	0.80067	.	.	1
**25439**	0	5.75	1	0.80067	.	.	1
**25440**	0	5.75	1	0.80067	.	.	1
**25441**	0	5.75	1	0.80067	.	.	1
**25442**	0	5.75	1	0.80067	.	.	1
**25443**	0	5.75	1	0.80067	.	.	1
**25444**	0	5.75	1	0.80067	.	.	1
**25445**	0	5.75	1	0.80067	.	.	1

Obs	Sub_group	fuptimey	_CENSOR_	SURVIVAL	SDF_LCL	SDF_UCL	STRATUM
**25446**	0	5.75	1	0.80067	.	.	1
**25447**	0	5.75	1	0.80067	.	.	1
**25448**	0	5.75	1	0.80067	.	.	1
**25449**	0	5.75	1	0.80067	.	.	1
**25450**	0	5.75	1	0.80067	.	.	1
**25451**	0	5.75	1	0.80067	.	.	1
**25452**	0	5.75	1	0.80067	.	.	1
**25453**	0	5.75	1	0.80067	.	.	1
**25454**	0	5.75	1	0.80067	.	.	1
**25455**	0	5.76	0	0.80056	0.79619	0.80485	1
**25456**	0	5.76	1	0.80056	.	.	1
**25457**	0	5.76	1	0.80056	.	.	1
**25458**	0	5.76	1	0.80056	.	.	1
**25459**	0	5.76	1	0.80056	.	.	1
**25460**	0	5.76	1	0.80056	.	.	1
**25461**	0	5.76	1	0.80056	.	.	1
**25462**	0	5.76	1	0.80056	.	.	1
**25463**	0	5.76	1	0.80056	.	.	1
**25464**	0	5.76	1	0.80056	.	.	1
**25465**	0	5.76	1	0.80056	.	.	1
**25466**	0	5.76	1	0.80056	.	.	1
**25467**	0	5.76	1	0.80056	.	.	1
**25468**	0	5.76	1	0.80056	.	.	1
**25469**	0	5.76	1	0.80056	.	.	1
**25470**	0	5.76	1	0.80056	.	.	1
**25471**	0	5.76	1	0.80056	.	.	1
**25472**	0	5.76	1	0.80056	.	.	1
**25473**	0	5.76	1	0.80056	.	.	1
**25474**	0	5.76	1	0.80056	.	.	1
**25475**	0	5.76	1	0.80056	.	.	1
**25476**	0	5.76	1	0.80056	.	.	1
**25477**	0	5.76	1	0.80056	.	.	1
**25478**	0	5.76	1	0.80056	.	.	1
**25479**	0	5.76	1	0.80056	.	.	1
**25480**	0	5.76	1	0.80056	.	.	1

Obs	Sub_group	fuptimey	_CENSOR_	SURVIVAL	SDF_LCL	SDF_UCL	STRATUM
25481	0	5.76	1	0.80056	.	.	1
25482	0	5.76	1	0.80056	.	.	1
25483	0	5.76	1	0.80056	.	.	1
25484	0	5.76	1	0.80056	.	.	1
25485	0	5.76	1	0.80056	.	.	1
25486	0	5.76	1	0.80056	.	.	1
25487	0	5.76	1	0.80056	.	.	1
25488	0	5.76	1	0.80056	.	.	1
25489	0	5.76	1	0.80056	.	.	1
25490	0	5.76	1	0.80056	.	.	1
25491	0	5.76	1	0.80056	.	.	1
25492	0	5.76	1	0.80056	.	.	1
25493	0	5.76	1	0.80056	.	.	1
25494	0	5.76	1	0.80056	.	.	1
25495	0	5.76	1	0.80056	.	.	1
25496	0	5.77	0	0.80028	0.79591	0.80458	1
25497	0	5.77	1	0.80028	.	.	1
25498	0	5.77	1	0.80028	.	.	1
25499	0	5.77	1	0.80028	.	.	1
25500	0	5.77	1	0.80028	.	.	1
25501	0	5.77	1	0.80028	.	.	1
25502	0	5.77	1	0.80028	.	.	1
25503	0	5.77	1	0.80028	.	.	1
25504	0	5.77	1	0.80028	.	.	1
25505	0	5.77	1	0.80028	.	.	1
25506	0	5.77	1	0.80028	.	.	1
25507	0	5.77	1	0.80028	.	.	1
25508	0	5.77	1	0.80028	.	.	1
25509	0	5.77	1	0.80028	.	.	1
25510	0	5.77	1	0.80028	.	.	1
25511	0	5.77	1	0.80028	.	.	1
25512	0	5.77	1	0.80028	.	.	1
25513	0	5.77	1	0.80028	.	.	1
25514	0	5.77	1	0.80028	.	.	1
25515	0	5.77	1	0.80028	.	.	1

Obs	Sub_group	fuptimey	_CENSOR_	SURVIVAL	SDF_LCL	SDF_UCL	STRATUM
25516	0	5.77	1	0.80028	.	.	1
25517	0	5.77	1	0.80028	.	.	1
25518	0	5.77	1	0.80028	.	.	1
25519	0	5.77	1	0.80028	.	.	1
25520	0	5.77	1	0.80028	.	.	1
25521	0	5.77	1	0.80028	.	.	1
25522	0	5.77	1	0.80028	.	.	1
25523	0	5.77	1	0.80028	.	.	1
25524	0	5.77	1	0.80028	.	.	1
25525	0	5.77	1	0.80028	.	.	1
25526	0	5.78	0	0.79995	0.79557	0.80425	1
25527	0	5.78	1	0.79995	.	.	1
25528	0	5.78	1	0.79995	.	.	1
25529	0	5.78	1	0.79995	.	.	1
25530	0	5.78	1	0.79995	.	.	1
25531	0	5.78	1	0.79995	.	.	1
25532	0	5.78	1	0.79995	.	.	1
25533	0	5.78	1	0.79995	.	.	1
25534	0	5.78	1	0.79995	.	.	1
25535	0	5.78	1	0.79995	.	.	1
25536	0	5.78	1	0.79995	.	.	1
25537	0	5.78	1	0.79995	.	.	1
25538	0	5.78	1	0.79995	.	.	1
25539	0	5.78	1	0.79995	.	.	1
25540	0	5.78	1	0.79995	.	.	1
25541	0	5.78	1	0.79995	.	.	1
25542	0	5.78	1	0.79995	.	.	1
25543	0	5.78	1	0.79995	.	.	1
25544	0	5.78	1	0.79995	.	.	1
25545	0	5.78	1	0.79995	.	.	1
25546	0	5.78	1	0.79995	.	.	1
25547	0	5.78	1	0.79995	.	.	1
25548	0	5.78	1	0.79995	.	.	1
25549	0	5.78	1	0.79995	.	.	1
25550	0	5.78	1	0.79995	.	.	1

Obs	Sub_group	fuptimey	_CENSOR_	SURVIVAL	SDF_LCL	SDF_UCL	STRATUM
25551	0	5.78	1	0.79995	.	.	1
25552	0	5.78	1	0.79995	.	.	1
25553	0	5.78	1	0.79995	.	.	1
25554	0	5.78	1	0.79995	.	.	1
25555	0	5.78	1	0.79995	.	.	1
25556	0	5.78	1	0.79995	.	.	1
25557	0	5.78	1	0.79995	.	.	1
25558	0	5.78	1	0.79995	.	.	1
25559	0	5.78	1	0.79995	.	.	1
25560	0	5.78	1	0.79995	.	.	1
25561	0	5.78	1	0.79995	.	.	1
25562	0	5.78	1	0.79995	.	.	1
25563	0	5.79	0	0.79984	0.79546	0.80414	1
25564	0	5.79	1	0.79984	.	.	1
25565	0	5.79	1	0.79984	.	.	1
25566	0	5.79	1	0.79984	.	.	1
25567	0	5.79	1	0.79984	.	.	1
25568	0	5.79	1	0.79984	.	.	1
25569	0	5.79	1	0.79984	.	.	1
25570	0	5.79	1	0.79984	.	.	1
25571	0	5.79	1	0.79984	.	.	1
25572	0	5.79	1	0.79984	.	.	1
25573	0	5.79	1	0.79984	.	.	1
25574	0	5.79	1	0.79984	.	.	1
25575	0	5.79	1	0.79984	.	.	1
25576	0	5.79	1	0.79984	.	.	1
25577	0	5.79	1	0.79984	.	.	1
25578	0	5.79	1	0.79984	.	.	1
25579	0	5.79	1	0.79984	.	.	1
25580	0	5.79	1	0.79984	.	.	1
25581	0	5.79	1	0.79984	.	.	1
25582	0	5.79	1	0.79984	.	.	1
25583	0	5.79	1	0.79984	.	.	1
25584	0	5.79	1	0.79984	.	.	1
25585	0	5.79	1	0.79984	.	.	1

Obs	Sub_group	fuptimey	_CENSOR_	SURVIVAL	SDF_LCL	SDF_UCL	STRATUM
25586	0	5.79	1	0.79984	.	.	1
25587	0	5.79	1	0.79984	.	.	1
25588	0	5.80	0	0.79950	0.79512	0.80381	1
25589	0	5.80	1	0.79950	.	.	1
25590	0	5.80	1	0.79950	.	.	1
25591	0	5.80	1	0.79950	.	.	1
25592	0	5.80	1	0.79950	.	.	1
25593	0	5.80	1	0.79950	.	.	1
25594	0	5.80	1	0.79950	.	.	1
25595	0	5.80	1	0.79950	.	.	1
25596	0	5.80	1	0.79950	.	.	1
25597	0	5.80	1	0.79950	.	.	1
25598	0	5.80	1	0.79950	.	.	1
25599	0	5.80	1	0.79950	.	.	1
25600	0	5.80	1	0.79950	.	.	1
25601	0	5.80	1	0.79950	.	.	1
25602	0	5.80	1	0.79950	.	.	1
25603	0	5.80	1	0.79950	.	.	1
25604	0	5.80	1	0.79950	.	.	1
25605	0	5.80	1	0.79950	.	.	1
25606	0	5.80	1	0.79950	.	.	1
25607	0	5.81	0	0.79922	0.79483	0.80354	1
25608	0	5.81	1	0.79922	.	.	1
25609	0	5.81	1	0.79922	.	.	1
25610	0	5.81	1	0.79922	.	.	1
25611	0	5.81	1	0.79922	.	.	1
25612	0	5.81	1	0.79922	.	.	1
25613	0	5.81	1	0.79922	.	.	1
25614	0	5.81	1	0.79922	.	.	1
25615	0	5.81	1	0.79922	.	.	1
25616	0	5.81	1	0.79922	.	.	1
25617	0	5.81	1	0.79922	.	.	1
25618	0	5.81	1	0.79922	.	.	1
25619	0	5.81	1	0.79922	.	.	1
25620	0	5.81	1	0.79922	.	.	1

Obs	Sub_group	fuptimey	_CENSOR_	SURVIVAL	SDF_LCL	SDF_UCL	STRATUM
25621	0	5.81	1	0.79922	.	.	1
25622	0	5.81	1	0.79922	.	.	1
25623	0	5.81	1	0.79922	.	.	1
25624	0	5.81	1	0.79922	.	.	1
25625	0	5.81	1	0.79922	.	.	1
25626	0	5.81	1	0.79922	.	.	1
25627	0	5.81	1	0.79922	.	.	1
25628	0	5.81	1	0.79922	.	.	1
25629	0	5.81	1	0.79922	.	.	1
25630	0	5.81	1	0.79922	.	.	1
25631	0	5.81	1	0.79922	.	.	1
25632	0	5.81	1	0.79922	.	.	1
25633	0	5.81	1	0.79922	.	.	1
25634	0	5.81	1	0.79922	.	.	1
25635	0	5.82	0	0.79906	0.79466	0.80337	1
25636	0	5.82	1	0.79906	.	.	1
25637	0	5.82	1	0.79906	.	.	1
25638	0	5.82	1	0.79906	.	.	1
25639	0	5.82	1	0.79906	.	.	1
25640	0	5.82	1	0.79906	.	.	1
25641	0	5.82	1	0.79906	.	.	1
25642	0	5.82	1	0.79906	.	.	1
25643	0	5.82	1	0.79906	.	.	1
25644	0	5.82	1	0.79906	.	.	1
25645	0	5.82	1	0.79906	.	.	1
25646	0	5.82	1	0.79906	.	.	1
25647	0	5.82	1	0.79906	.	.	1
25648	0	5.82	1	0.79906	.	.	1
25649	0	5.82	1	0.79906	.	.	1
25650	0	5.82	1	0.79906	.	.	1
25651	0	5.82	1	0.79906	.	.	1
25652	0	5.82	1	0.79906	.	.	1
25653	0	5.82	1	0.79906	.	.	1
25654	0	5.82	1	0.79906	.	.	1
25655	0	5.82	1	0.79906	.	.	1

Obs	Sub_group	fuptimey	_CENSOR_	SURVIVAL	SDF_LCL	SDF_UCL	STRATUM
25656	0	5.82	1	0.79906	.	.	1
25657	0	5.82	1	0.79906	.	.	1
25658	0	5.82	1	0.79906	.	.	1
25659	0	5.82	1	0.79906	.	.	1
25660	0	5.82	1	0.79906	.	.	1
25661	0	5.83	0	0.79872	0.79432	0.80304	1
25662	0	5.83	1	0.79872	.	.	1
25663	0	5.83	1	0.79872	.	.	1
25664	0	5.83	1	0.79872	.	.	1
25665	0	5.83	1	0.79872	.	.	1
25666	0	5.83	1	0.79872	.	.	1
25667	0	5.83	1	0.79872	.	.	1
25668	0	5.83	1	0.79872	.	.	1
25669	0	5.83	1	0.79872	.	.	1
25670	0	5.83	1	0.79872	.	.	1
25671	0	5.83	1	0.79872	.	.	1
25672	0	5.83	1	0.79872	.	.	1
25673	0	5.83	1	0.79872	.	.	1
25674	0	5.83	1	0.79872	.	.	1
25675	0	5.83	1	0.79872	.	.	1
25676	0	5.83	1	0.79872	.	.	1
25677	0	5.83	1	0.79872	.	.	1
25678	0	5.83	1	0.79872	.	.	1
25679	0	5.83	1	0.79872	.	.	1
25680	0	5.83	1	0.79872	.	.	1
25681	0	5.83	1	0.79872	.	.	1
25682	0	5.83	1	0.79872	.	.	1
25683	0	5.83	1	0.79872	.	.	1
25684	0	5.83	1	0.79872	.	.	1
25685	0	5.83	1	0.79872	.	.	1
25686	0	5.83	1	0.79872	.	.	1
25687	0	5.83	1	0.79872	.	.	1
25688	0	5.83	1	0.79872	.	.	1
25689	0	5.83	1	0.79872	.	.	1
25690	0	5.83	1	0.79872	.	.	1

Obs	Sub_group	fuptimey	_CENSOR_	SURVIVAL	SDF_LCL	SDF_UCL	STRATUM
25691	0	5.83	1	0.79872	.	.	1
25692	0	5.83	1	0.79872	.	.	1
25693	0	5.83	1	0.79872	.	.	1
25694	0	5.83	1	0.79872	.	.	1
25695	0	5.83	1	0.79872	.	.	1
25696	0	5.83	1	0.79872	.	.	1
25697	0	5.83	1	0.79872	.	.	1
25698	0	5.83	1	0.79872	.	.	1
25699	0	5.83	1	0.79872	.	.	1
25700	0	5.83	1	0.79872	.	.	1
25701	0	5.83	1	0.79872	.	.	1
25702	0	5.83	1	0.79872	.	.	1
25703	0	5.83	1	0.79872	.	.	1
25704	0	5.84	0	0.79855	0.79414	0.80288	1
25705	0	5.84	1	0.79855	.	.	1
25706	0	5.84	1	0.79855	.	.	1
25707	0	5.84	1	0.79855	.	.	1
25708	0	5.84	1	0.79855	.	.	1
25709	0	5.84	1	0.79855	.	.	1
25710	0	5.84	1	0.79855	.	.	1
25711	0	5.84	1	0.79855	.	.	1
25712	0	5.84	1	0.79855	.	.	1
25713	0	5.84	1	0.79855	.	.	1
25714	0	5.84	1	0.79855	.	.	1
25715	0	5.84	1	0.79855	.	.	1
25716	0	5.84	1	0.79855	.	.	1
25717	0	5.84	1	0.79855	.	.	1
25718	0	5.84	1	0.79855	.	.	1
25719	0	5.84	1	0.79855	.	.	1
25720	0	5.84	1	0.79855	.	.	1
25721	0	5.84	1	0.79855	.	.	1
25722	0	5.84	1	0.79855	.	.	1
25723	0	5.84	1	0.79855	.	.	1
25724	0	5.84	1	0.79855	.	.	1
25725	0	5.84	1	0.79855	.	.	1

Obs	Sub_group	fuptimey	_CENSOR_	SURVIVAL	SDF_LCL	SDF_UCL	STRATUM
25726	0	5.84	1	0.79855	.	.	1
25727	0	5.84	1	0.79855	.	.	1
25728	0	5.84	1	0.79855	.	.	1
25729	0	5.84	1	0.79855	.	.	1
25730	0	5.84	1	0.79855	.	.	1
25731	0	5.84	1	0.79855	.	.	1
25732	0	5.84	1	0.79855	.	.	1
25733	0	5.84	1	0.79855	.	.	1
25734	0	5.84	1	0.79855	.	.	1
25735	0	5.84	1	0.79855	.	.	1
25736	0	5.84	1	0.79855	.	.	1
25737	0	5.84	1	0.79855	.	.	1
25738	0	5.85	0	0.79838	0.79397	0.80271	1
25739	0	5.85	1	0.79838	.	.	1
25740	0	5.85	1	0.79838	.	.	1
25741	0	5.85	1	0.79838	.	.	1
25742	0	5.85	1	0.79838	.	.	1
25743	0	5.85	1	0.79838	.	.	1
25744	0	5.85	1	0.79838	.	.	1
25745	0	5.85	1	0.79838	.	.	1
25746	0	5.85	1	0.79838	.	.	1
25747	0	5.85	1	0.79838	.	.	1
25748	0	5.85	1	0.79838	.	.	1
25749	0	5.85	1	0.79838	.	.	1
25750	0	5.85	1	0.79838	.	.	1
25751	0	5.85	1	0.79838	.	.	1
25752	0	5.85	1	0.79838	.	.	1
25753	0	5.85	1	0.79838	.	.	1
25754	0	5.85	1	0.79838	.	.	1
25755	0	5.85	1	0.79838	.	.	1
25756	0	5.85	1	0.79838	.	.	1
25757	0	5.85	1	0.79838	.	.	1
25758	0	5.85	1	0.79838	.	.	1
25759	0	5.85	1	0.79838	.	.	1
25760	0	5.85	1	0.79838	.	.	1

Obs	Sub_group	fuptimey	_CENSOR_	SURVIVAL	SDF_LCL	SDF_UCL	STRATUM
25761	0	5.85	1	0.79838	.	.	1
25762	0	5.85	1	0.79838	.	.	1
25763	0	5.85	1	0.79838	.	.	1
25764	0	5.85	1	0.79838	.	.	1
25765	0	5.85	1	0.79838	.	.	1
25766	0	5.85	1	0.79838	.	.	1
25767	0	5.85	1	0.79838	.	.	1
25768	0	5.85	1	0.79838	.	.	1
25769	0	5.85	1	0.79838	.	.	1
25770	0	5.85	1	0.79838	.	.	1
25771	0	5.85	1	0.79838	.	.	1
25772	0	5.85	1	0.79838	.	.	1
25773	0	5.85	1	0.79838	.	.	1
25774	0	5.85	1	0.79838	.	.	1
25775	0	5.85	1	0.79838	.	.	1
25776	0	5.85	1	0.79838	.	.	1
25777	0	5.85	1	0.79838	.	.	1
25778	0	5.85	1	0.79838	.	.	1
25779	0	5.85	1	0.79838	.	.	1
25780	0	5.85	1	0.79838	.	.	1
25781	0	5.85	1	0.79838	.	.	1
25782	0	5.85	1	0.79838	.	.	1
25783	0	5.85	1	0.79838	.	.	1
25784	0	5.86	0	0.79793	0.79351	0.80227	1
25785	0	5.86	1	0.79793	.	.	1
25786	0	5.86	1	0.79793	.	.	1
25787	0	5.86	1	0.79793	.	.	1
25788	0	5.86	1	0.79793	.	.	1
25789	0	5.86	1	0.79793	.	.	1
25790	0	5.86	1	0.79793	.	.	1
25791	0	5.86	1	0.79793	.	.	1
25792	0	5.86	1	0.79793	.	.	1
25793	0	5.86	1	0.79793	.	.	1
25794	0	5.86	1	0.79793	.	.	1
25795	0	5.86	1	0.79793	.	.	1

Obs	Sub_group	fuptimey	_CENSOR_	SURVIVAL	SDF_LCL	SDF_UCL	STRATUM
25796	0	5.86	1	0.79793	.	.	1
25797	0	5.86	1	0.79793	.	.	1
25798	0	5.86	1	0.79793	.	.	1
25799	0	5.86	1	0.79793	.	.	1
25800	0	5.86	1	0.79793	.	.	1
25801	0	5.86	1	0.79793	.	.	1
25802	0	5.86	1	0.79793	.	.	1
25803	0	5.86	1	0.79793	.	.	1
25804	0	5.86	1	0.79793	.	.	1
25805	0	5.86	1	0.79793	.	.	1
25806	0	5.86	1	0.79793	.	.	1
25807	0	5.86	1	0.79793	.	.	1
25808	0	5.86	1	0.79793	.	.	1
25809	0	5.86	1	0.79793	.	.	1
25810	0	5.86	1	0.79793	.	.	1
25811	0	5.86	1	0.79793	.	.	1
25812	0	5.87	0	0.79776	0.79334	0.80210	1
25813	0	5.87	1	0.79776	.	.	1
25814	0	5.87	1	0.79776	.	.	1
25815	0	5.87	1	0.79776	.	.	1
25816	0	5.87	1	0.79776	.	.	1
25817	0	5.87	1	0.79776	.	.	1
25818	0	5.87	1	0.79776	.	.	1
25819	0	5.87	1	0.79776	.	.	1
25820	0	5.87	1	0.79776	.	.	1
25821	0	5.87	1	0.79776	.	.	1
25822	0	5.87	1	0.79776	.	.	1
25823	0	5.87	1	0.79776	.	.	1
25824	0	5.87	1	0.79776	.	.	1
25825	0	5.87	1	0.79776	.	.	1
25826	0	5.87	1	0.79776	.	.	1
25827	0	5.87	1	0.79776	.	.	1
25828	0	5.87	1	0.79776	.	.	1
25829	0	5.87	1	0.79776	.	.	1
25830	0	5.87	1	0.79776	.	.	1

Obs	Sub_group	fuptimey	_CENSOR_	SURVIVAL	SDF_LCL	SDF_UCL	STRATUM
25831	0	5.87	1	0.79776	.	.	1
25832	0	5.87	1	0.79776	.	.	1
25833	0	5.87	1	0.79776	.	.	1
25834	0	5.87	1	0.79776	.	.	1
25835	0	5.87	1	0.79776	.	.	1
25836	0	5.87	1	0.79776	.	.	1
25837	0	5.87	1	0.79776	.	.	1
25838	0	5.87	1	0.79776	.	.	1
25839	0	5.87	1	0.79776	.	.	1
25840	0	5.87	1	0.79776	.	.	1
25841	0	5.87	1	0.79776	.	.	1
25842	0	5.87	1	0.79776	.	.	1
25843	0	5.87	1	0.79776	.	.	1
25844	0	5.87	1	0.79776	.	.	1
25845	0	5.87	1	0.79776	.	.	1
25846	0	5.87	1	0.79776	.	.	1
25847	0	5.87	1	0.79776	.	.	1
25848	0	5.87	1	0.79776	.	.	1
25849	0	5.87	1	0.79776	.	.	1
25850	0	5.88	0	0.79759	0.79316	0.80193	1
25851	0	5.88	1	0.79759	.	.	1
25852	0	5.88	1	0.79759	.	.	1
25853	0	5.88	1	0.79759	.	.	1
25854	0	5.88	1	0.79759	.	.	1
25855	0	5.88	1	0.79759	.	.	1
25856	0	5.88	1	0.79759	.	.	1
25857	0	5.88	1	0.79759	.	.	1
25858	0	5.88	1	0.79759	.	.	1
25859	0	5.88	1	0.79759	.	.	1
25860	0	5.88	1	0.79759	.	.	1
25861	0	5.88	1	0.79759	.	.	1
25862	0	5.88	1	0.79759	.	.	1
25863	0	5.88	1	0.79759	.	.	1
25864	0	5.88	1	0.79759	.	.	1
25865	0	5.88	1	0.79759	.	.	1

Obs	Sub_group	fuptimey	_CENSOR_	SURVIVAL	SDF_LCL	SDF_UCL	STRATUM
**25866**	0	5.88	1	0.79759	.	.	1
**25867**	0	5.88	1	0.79759	.	.	1
**25868**	0	5.88	1	0.79759	.	.	1
**25869**	0	5.88	1	0.79759	.	.	1
**25870**	0	5.88	1	0.79759	.	.	1
**25871**	0	5.88	1	0.79759	.	.	1
**25872**	0	5.88	1	0.79759	.	.	1
**25873**	0	5.89	0	0.79736	0.79293	0.80171	1
**25874**	0	5.89	1	0.79736	.	.	1
**25875**	0	5.89	1	0.79736	.	.	1
**25876**	0	5.89	1	0.79736	.	.	1
**25877**	0	5.89	1	0.79736	.	.	1
**25878**	0	5.89	1	0.79736	.	.	1
**25879**	0	5.89	1	0.79736	.	.	1
**25880**	0	5.89	1	0.79736	.	.	1
**25881**	0	5.89	1	0.79736	.	.	1
**25882**	0	5.89	1	0.79736	.	.	1
**25883**	0	5.89	1	0.79736	.	.	1
**25884**	0	5.89	1	0.79736	.	.	1
**25885**	0	5.89	1	0.79736	.	.	1
**25886**	0	5.89	1	0.79736	.	.	1
**25887**	0	5.89	1	0.79736	.	.	1
**25888**	0	5.89	1	0.79736	.	.	1
**25889**	0	5.89	1	0.79736	.	.	1
**25890**	0	5.89	1	0.79736	.	.	1
**25891**	0	5.89	1	0.79736	.	.	1
**25892**	0	5.89	1	0.79736	.	.	1
**25893**	0	5.89	1	0.79736	.	.	1
**25894**	0	5.89	1	0.79736	.	.	1
**25895**	0	5.89	1	0.79736	.	.	1
**25896**	0	5.89	1	0.79736	.	.	1
**25897**	0	5.89	1	0.79736	.	.	1
**25898**	0	5.89	1	0.79736	.	.	1
**25899**	0	5.89	1	0.79736	.	.	1
**25900**	0	5.89	1	0.79736	.	.	1

Obs	Sub_group	fuptimey	_CENSOR_	SURVIVAL	SDF_LCL	SDF_UCL	STRATUM
25901	0	5.89	1	0.79736	.	.	1
25902	0	5.89	1	0.79736	.	.	1
25903	0	5.89	1	0.79736	.	.	1
25904	0	5.89	1	0.79736	.	.	1
25905	0	5.89	1	0.79736	.	.	1
25906	0	5.89	1	0.79736	.	.	1
25907	0	5.89	1	0.79736	.	.	1
25908	0	5.89	1	0.79736	.	.	1
25909	0	5.90	0	0.79725	0.79281	0.80160	1
25910	0	5.90	1	0.79725	.	.	1
25911	0	5.90	1	0.79725	.	.	1
25912	0	5.90	1	0.79725	.	.	1
25913	0	5.90	1	0.79725	.	.	1
25914	0	5.90	1	0.79725	.	.	1
25915	0	5.90	1	0.79725	.	.	1
25916	0	5.90	1	0.79725	.	.	1
25917	0	5.90	1	0.79725	.	.	1
25918	0	5.90	1	0.79725	.	.	1
25919	0	5.90	1	0.79725	.	.	1
25920	0	5.90	1	0.79725	.	.	1
25921	0	5.90	1	0.79725	.	.	1
25922	0	5.90	1	0.79725	.	.	1
25923	0	5.90	1	0.79725	.	.	1
25924	0	5.90	1	0.79725	.	.	1
25925	0	5.90	1	0.79725	.	.	1
25926	0	5.90	1	0.79725	.	.	1
25927	0	5.90	1	0.79725	.	.	1
25928	0	5.90	1	0.79725	.	.	1
25929	0	5.90	1	0.79725	.	.	1
25930	0	5.90	1	0.79725	.	.	1
25931	0	5.90	1	0.79725	.	.	1
25932	0	5.90	1	0.79725	.	.	1
25933	0	5.90	1	0.79725	.	.	1
25934	0	5.90	1	0.79725	.	.	1
25935	0	5.90	1	0.79725	.	.	1

Obs	Sub_group	fuptimey	_CENSOR_	SURVIVAL	SDF_LCL	SDF_UCL	STRATUM
25936	0	5.90	1	0.79725	.	.	1
25937	0	5.90	1	0.79725	.	.	1
25938	0	5.90	1	0.79725	.	.	1
25939	0	5.90	1	0.79725	.	.	1
25940	0	5.90	1	0.79725	.	.	1
25941	0	5.90	1	0.79725	.	.	1
25942	0	5.90	1	0.79725	.	.	1
25943	0	5.90	1	0.79725	.	.	1
25944	0	5.90	1	0.79725	.	.	1
25945	0	5.90	1	0.79725	.	.	1
25946	0	5.90	1	0.79725	.	.	1
25947	0	5.90	1	0.79725	.	.	1
25948	0	5.90	1	0.79725	.	.	1
25949	0	5.90	1	0.79725	.	.	1
25950	0	5.91	0	0.79707	0.79264	0.80143	1
25951	0	5.91	1	0.79707	.	.	1
25952	0	5.91	1	0.79707	.	.	1
25953	0	5.91	1	0.79707	.	.	1
25954	0	5.91	1	0.79707	.	.	1
25955	0	5.91	1	0.79707	.	.	1
25956	0	5.91	1	0.79707	.	.	1
25957	0	5.91	1	0.79707	.	.	1
25958	0	5.91	1	0.79707	.	.	1
25959	0	5.91	1	0.79707	.	.	1
25960	0	5.91	1	0.79707	.	.	1
25961	0	5.91	1	0.79707	.	.	1
25962	0	5.91	1	0.79707	.	.	1
25963	0	5.91	1	0.79707	.	.	1
25964	0	5.91	1	0.79707	.	.	1
25965	0	5.91	1	0.79707	.	.	1
25966	0	5.91	1	0.79707	.	.	1
25967	0	5.91	1	0.79707	.	.	1
25968	0	5.91	1	0.79707	.	.	1
25969	0	5.91	1	0.79707	.	.	1
25970	0	5.91	1	0.79707	.	.	1

Obs	Sub_group	fuptimey	_CENSOR_	SURVIVAL	SDF_LCL	SDF_UCL	STRATUM
25971	0	5.91	1	0.79707	.	.	1
25972	0	5.91	1	0.79707	.	.	1
25973	0	5.91	1	0.79707	.	.	1
25974	0	5.91	1	0.79707	.	.	1
25975	0	5.91	1	0.79707	.	.	1
25976	0	5.91	1	0.79707	.	.	1
25977	0	5.91	1	0.79707	.	.	1
25978	0	5.91	1	0.79707	.	.	1
25979	0	5.91	1	0.79707	.	.	1
25980	0	5.91	1	0.79707	.	.	1
25981	0	5.91	1	0.79707	.	.	1
25982	0	5.91	1	0.79707	.	.	1
25983	0	5.91	1	0.79707	.	.	1
25984	0	5.92	0	0.79633	0.79188	0.80070	1
25985	0	5.92	1	0.79633	.	.	1
25986	0	5.92	1	0.79633	.	.	1
25987	0	5.92	1	0.79633	.	.	1
25988	0	5.92	1	0.79633	.	.	1
25989	0	5.92	1	0.79633	.	.	1
25990	0	5.92	1	0.79633	.	.	1
25991	0	5.92	1	0.79633	.	.	1
25992	0	5.92	1	0.79633	.	.	1
25993	0	5.92	1	0.79633	.	.	1
25994	0	5.92	1	0.79633	.	.	1
25995	0	5.92	1	0.79633	.	.	1
25996	0	5.92	1	0.79633	.	.	1
25997	0	5.92	1	0.79633	.	.	1
25998	0	5.92	1	0.79633	.	.	1
25999	0	5.92	1	0.79633	.	.	1
26000	0	5.92	1	0.79633	.	.	1
26001	0	5.92	1	0.79633	.	.	1
26002	0	5.92	1	0.79633	.	.	1
26003	0	5.92	1	0.79633	.	.	1
26004	0	5.92	1	0.79633	.	.	1
26005	0	5.92	1	0.79633	.	.	1

Obs	Sub_group	fuptimey	_CENSOR_	SURVIVAL	SDF_LCL	SDF_UCL	STRATUM
26006	0	5.92	1	0.79633	.	.	1
26007	0	5.92	1	0.79633	.	.	1
26008	0	5.92	1	0.79633	.	.	1
26009	0	5.92	1	0.79633	.	.	1
26010	0	5.92	1	0.79633	.	.	1
26011	0	5.92	1	0.79633	.	.	1
26012	0	5.92	1	0.79633	.	.	1
26013	0	5.92	1	0.79633	.	.	1
26014	0	5.93	0	0.79593	0.79147	0.80030	1
26015	0	5.93	1	0.79593	.	.	1
26016	0	5.93	1	0.79593	.	.	1
26017	0	5.93	1	0.79593	.	.	1
26018	0	5.93	1	0.79593	.	.	1
26019	0	5.93	1	0.79593	.	.	1
26020	0	5.93	1	0.79593	.	.	1
26021	0	5.93	1	0.79593	.	.	1
26022	0	5.93	1	0.79593	.	.	1
26023	0	5.93	1	0.79593	.	.	1
26024	0	5.93	1	0.79593	.	.	1
26025	0	5.93	1	0.79593	.	.	1
26026	0	5.93	1	0.79593	.	.	1
26027	0	5.93	1	0.79593	.	.	1
26028	0	5.93	1	0.79593	.	.	1
26029	0	5.93	1	0.79593	.	.	1
26030	0	5.93	1	0.79593	.	.	1
26031	0	5.93	1	0.79593	.	.	1
26032	0	5.93	1	0.79593	.	.	1
26033	0	5.93	1	0.79593	.	.	1
26034	0	5.93	1	0.79593	.	.	1
26035	0	5.93	1	0.79593	.	.	1
26036	0	5.93	1	0.79593	.	.	1
26037	0	5.93	1	0.79593	.	.	1
26038	0	5.93	1	0.79593	.	.	1
26039	0	5.93	1	0.79593	.	.	1
26040	0	5.93	1	0.79593	.	.	1

Obs	Sub_group	fuptimey	_CENSOR_	SURVIVAL	SDF_LCL	SDF_UCL	STRATUM
26041	0	5.93	1	0.79593	.	.	1
26042	0	5.93	1	0.79593	.	.	1
26043	0	5.93	1	0.79593	.	.	1
26044	0	5.93	1	0.79593	.	.	1
26045	0	5.93	1	0.79593	.	.	1
26046	0	5.93	1	0.79593	.	.	1
26047	0	5.93	1	0.79593	.	.	1
26048	0	5.93	1	0.79593	.	.	1
26049	0	5.93	1	0.79593	.	.	1
26050	0	5.93	1	0.79593	.	.	1
26051	0	5.93	1	0.79593	.	.	1
26052	0	5.93	1	0.79593	.	.	1
26053	0	5.93	1	0.79593	.	.	1
26054	0	5.93	1	0.79593	.	.	1
26055	0	5.93	1	0.79593	.	.	1
26056	0	5.93	1	0.79593	.	.	1
26057	0	5.94	0	0.79552	0.79106	0.79991	1
26058	0	5.94	1	0.79552	.	.	1
26059	0	5.94	1	0.79552	.	.	1
26060	0	5.94	1	0.79552	.	.	1
26061	0	5.94	1	0.79552	.	.	1
26062	0	5.94	1	0.79552	.	.	1
26063	0	5.94	1	0.79552	.	.	1
26064	0	5.94	1	0.79552	.	.	1
26065	0	5.94	1	0.79552	.	.	1
26066	0	5.94	1	0.79552	.	.	1
26067	0	5.94	1	0.79552	.	.	1
26068	0	5.94	1	0.79552	.	.	1
26069	0	5.94	1	0.79552	.	.	1
26070	0	5.94	1	0.79552	.	.	1
26071	0	5.94	1	0.79552	.	.	1
26072	0	5.94	1	0.79552	.	.	1
26073	0	5.94	1	0.79552	.	.	1
26074	0	5.94	1	0.79552	.	.	1
26075	0	5.94	1	0.79552	.	.	1

Obs	Sub_group	fuptimey	_CENSOR_	SURVIVAL	SDF_LCL	SDF_UCL	STRATUM
**26076**	0	5.94	1	0.79552	.	.	1
**26077**	0	5.94	1	0.79552	.	.	1
**26078**	0	5.95	0	0.79506	0.79059	0.79945	1
**26079**	0	5.95	1	0.79506	.	.	1
**26080**	0	5.95	1	0.79506	.	.	1
**26081**	0	5.95	1	0.79506	.	.	1
**26082**	0	5.95	1	0.79506	.	.	1
**26083**	0	5.95	1	0.79506	.	.	1
**26084**	0	5.95	1	0.79506	.	.	1
**26085**	0	5.95	1	0.79506	.	.	1
**26086**	0	5.95	1	0.79506	.	.	1
**26087**	0	5.95	1	0.79506	.	.	1
**26088**	0	5.95	1	0.79506	.	.	1
**26089**	0	5.95	1	0.79506	.	.	1
**26090**	0	5.95	1	0.79506	.	.	1
**26091**	0	5.95	1	0.79506	.	.	1
**26092**	0	5.95	1	0.79506	.	.	1
**26093**	0	5.95	1	0.79506	.	.	1
**26094**	0	5.95	1	0.79506	.	.	1
**26095**	0	5.95	1	0.79506	.	.	1
**26096**	0	5.95	1	0.79506	.	.	1
**26097**	0	5.95	1	0.79506	.	.	1
**26098**	0	5.95	1	0.79506	.	.	1
**26099**	0	5.95	1	0.79506	.	.	1
**26100**	0	5.95	1	0.79506	.	.	1
**26101**	0	5.95	1	0.79506	.	.	1
**26102**	0	5.95	1	0.79506	.	.	1
**26103**	0	5.95	1	0.79506	.	.	1
**26104**	0	5.95	1	0.79506	.	.	1
**26105**	0	5.95	1	0.79506	.	.	1
**26106**	0	5.95	1	0.79506	.	.	1
**26107**	0	5.95	1	0.79506	.	.	1
**26108**	0	5.95	1	0.79506	.	.	1
**26109**	0	5.96	0	0.79466	0.79017	0.79906	1
**26110**	0	5.96	1	0.79466	.	.	1

Obs	Sub_group	fuptimey	_CENSOR_	SURVIVAL	SDF_LCL	SDF_UCL	STRATUM
26111	0	5.96	1	0.79466	.	.	1
26112	0	5.96	1	0.79466	.	.	1
26113	0	5.96	1	0.79466	.	.	1
26114	0	5.96	1	0.79466	.	.	1
26115	0	5.96	1	0.79466	.	.	1
26116	0	5.96	1	0.79466	.	.	1
26117	0	5.96	1	0.79466	.	.	1
26118	0	5.96	1	0.79466	.	.	1
26119	0	5.96	1	0.79466	.	.	1
26120	0	5.96	1	0.79466	.	.	1
26121	0	5.96	1	0.79466	.	.	1
26122	0	5.96	1	0.79466	.	.	1
26123	0	5.96	1	0.79466	.	.	1
26124	0	5.96	1	0.79466	.	.	1
26125	0	5.96	1	0.79466	.	.	1
26126	0	5.96	1	0.79466	.	.	1
26127	0	5.96	1	0.79466	.	.	1
26128	0	5.96	1	0.79466	.	.	1
26129	0	5.96	1	0.79466	.	.	1
26130	0	5.96	1	0.79466	.	.	1
26131	0	5.96	1	0.79466	.	.	1
26132	0	5.96	1	0.79466	.	.	1
26133	0	5.96	1	0.79466	.	.	1
26134	0	5.96	1	0.79466	.	.	1
26135	0	5.96	1	0.79466	.	.	1
26136	0	5.96	1	0.79466	.	.	1
26137	0	5.96	1	0.79466	.	.	1
26138	0	5.96	1	0.79466	.	.	1
26139	0	5.96	1	0.79466	.	.	1
26140	0	5.97	0	0.79460	0.79011	0.79900	1
26141	0	5.97	1	0.79460	.	.	1
26142	0	5.97	1	0.79460	.	.	1
26143	0	5.97	1	0.79460	.	.	1
26144	0	5.97	1	0.79460	.	.	1
26145	0	5.97	1	0.79460	.	.	1

Obs	Sub_group	fuptimey	_CENSOR_	SURVIVAL	SDF_LCL	SDF_UCL	STRATUM
26146	0	5.97	1	0.79460	.	.	1
26147	0	5.97	1	0.79460	.	.	1
26148	0	5.97	1	0.79460	.	.	1
26149	0	5.97	1	0.79460	.	.	1
26150	0	5.97	1	0.79460	.	.	1
26151	0	5.97	1	0.79460	.	.	1
26152	0	5.97	1	0.79460	.	.	1
26153	0	5.97	1	0.79460	.	.	1
26154	0	5.97	1	0.79460	.	.	1
26155	0	5.97	1	0.79460	.	.	1
26156	0	5.97	1	0.79460	.	.	1
26157	0	5.97	1	0.79460	.	.	1
26158	0	5.97	1	0.79460	.	.	1
26159	0	5.97	1	0.79460	.	.	1
26160	0	5.97	1	0.79460	.	.	1
26161	0	5.97	1	0.79460	.	.	1
26162	0	5.97	1	0.79460	.	.	1
26163	0	5.97	1	0.79460	.	.	1
26164	0	5.97	1	0.79460	.	.	1
26165	0	5.97	1	0.79460	.	.	1
26166	0	5.97	1	0.79460	.	.	1
26167	0	5.97	1	0.79460	.	.	1
26168	0	5.97	1	0.79460	.	.	1
26169	0	5.97	1	0.79460	.	.	1
26170	0	5.97	1	0.79460	.	.	1
26171	0	5.97	1	0.79460	.	.	1
26172	0	5.97	1	0.79460	.	.	1
26173	0	5.97	1	0.79460	.	.	1
26174	0	5.97	1	0.79460	.	.	1
26175	0	5.97	1	0.79460	.	.	1
26176	0	5.97	1	0.79460	.	.	1
26177	0	5.97	1	0.79460	.	.	1
26178	0	5.97	1	0.79460	.	.	1
26179	0	5.97	1	0.79460	.	.	1
26180	0	5.97	1	0.79460	.	.	1

Obs	Sub_group	fuptimey	_CENSOR_	SURVIVAL	SDF_LCL	SDF_UCL	STRATUM
26181	0	5.97	1	0.79460	.	.	1
26182	0	5.97	1	0.79460	.	.	1
26183	0	5.97	1	0.79460	.	.	1
26184	0	5.97	1	0.79460	.	.	1
26185	0	5.97	1	0.79460	.	.	1
26186	0	5.98	0	0.79436	0.78988	0.79877	1
26187	0	5.98	1	0.79436	.	.	1
26188	0	5.98	1	0.79436	.	.	1
26189	0	5.98	1	0.79436	.	.	1
26190	0	5.98	1	0.79436	.	.	1
26191	0	5.98	1	0.79436	.	.	1
26192	0	5.98	1	0.79436	.	.	1
26193	0	5.98	1	0.79436	.	.	1
26194	0	5.98	1	0.79436	.	.	1
26195	0	5.98	1	0.79436	.	.	1
26196	0	5.98	1	0.79436	.	.	1
26197	0	5.98	1	0.79436	.	.	1
26198	0	5.98	1	0.79436	.	.	1
26199	0	5.98	1	0.79436	.	.	1
26200	0	5.98	1	0.79436	.	.	1
26201	0	5.98	1	0.79436	.	.	1
26202	0	5.98	1	0.79436	.	.	1
26203	0	5.98	1	0.79436	.	.	1
26204	0	5.98	1	0.79436	.	.	1
26205	0	5.98	1	0.79436	.	.	1
26206	0	5.98	1	0.79436	.	.	1
26207	0	5.98	1	0.79436	.	.	1
26208	0	5.98	1	0.79436	.	.	1
26209	0	5.98	1	0.79436	.	.	1
26210	0	5.98	1	0.79436	.	.	1
26211	0	5.98	1	0.79436	.	.	1
26212	0	5.98	1	0.79436	.	.	1
26213	0	5.98	1	0.79436	.	.	1
26214	0	5.98	1	0.79436	.	.	1
26215	0	5.98	1	0.79436	.	.	1

Obs	Sub_group	fuptimey	_CENSOR_	SURVIVAL	SDF_LCL	SDF_UCL	STRATUM
26216	0	5.98	1	0.79436	.	.	1
26217	0	5.98	1	0.79436	.	.	1
26218	0	5.98	1	0.79436	.	.	1
26219	0	5.98	1	0.79436	.	.	1
26220	0	5.98	1	0.79436	.	.	1
26221	0	5.98	1	0.79436	.	.	1
26222	0	5.98	1	0.79436	.	.	1
26223	0	5.98	1	0.79436	.	.	1
26224	0	5.98	1	0.79436	.	.	1
26225	0	5.98	1	0.79436	.	.	1
26226	0	5.99	0	0.79425	0.78976	0.79866	1
26227	0	5.99	1	0.79425	.	.	1
26228	0	5.99	1	0.79425	.	.	1
26229	0	5.99	1	0.79425	.	.	1
26230	0	5.99	1	0.79425	.	.	1
26231	0	5.99	1	0.79425	.	.	1
26232	0	5.99	1	0.79425	.	.	1
26233	0	5.99	1	0.79425	.	.	1
26234	0	5.99	1	0.79425	.	.	1
26235	0	5.99	1	0.79425	.	.	1
26236	0	5.99	1	0.79425	.	.	1
26237	0	5.99	1	0.79425	.	.	1
26238	0	5.99	1	0.79425	.	.	1
26239	0	5.99	1	0.79425	.	.	1
26240	0	5.99	1	0.79425	.	.	1
26241	0	5.99	1	0.79425	.	.	1
26242	0	5.99	1	0.79425	.	.	1
26243	0	5.99	1	0.79425	.	.	1
26244	0	5.99	1	0.79425	.	.	1
26245	0	5.99	1	0.79425	.	.	1
26246	0	5.99	1	0.79425	.	.	1
26247	0	5.99	1	0.79425	.	.	1
26248	0	5.99	1	0.79425	.	.	1
26249	0	5.99	1	0.79425	.	.	1
26250	0	5.99	1	0.79425	.	.	1

Obs	Sub_group	fuptimey	_CENSOR_	SURVIVAL	SDF_LCL	SDF_UCL	STRATUM
26251	0	5.99	1	0.79425	.	.	1
26252	0	5.99	1	0.79425	.	.	1
26253	0	5.99	1	0.79425	.	.	1
26254	0	5.99	1	0.79425	.	.	1
26255	0	5.99	1	0.79425	.	.	1
26256	0	5.99	1	0.79425	.	.	1
26257	0	5.99	1	0.79425	.	.	1
26258	0	5.99	1	0.79425	.	.	1
26259	0	5.99	1	0.79425	.	.	1
26260	0	5.99	1	0.79425	.	.	1
26261	0	5.99	1	0.79425	.	.	1
26262	0	5.99	1	0.79425	.	.	1
26263	0	5.99	1	0.79425	.	.	1
26264	0	5.99	1	0.79425	.	.	1
26265	0	6.00	0	0.79407	0.78958	0.79848	1
26266	0	6.00	1	0.79407	.	.	1
26267	0	6.00	1	0.79407	.	.	1
26268	0	6.00	1	0.79407	.	.	1
26269	0	6.00	1	0.79407	.	.	1
26270	0	6.00	1	0.79407	.	.	1
26271	0	6.00	1	0.79407	.	.	1
26272	0	6.00	1	0.79407	.	.	1
26273	0	6.00	1	0.79407	.	.	1
26274	0	6.00	1	0.79407	.	.	1
26275	0	6.00	1	0.79407	.	.	1
26276	0	6.00	1	0.79407	.	.	1
26277	0	6.00	1	0.79407	.	.	1
26278	0	6.00	1	0.79407	.	.	1
26279	0	6.00	1	0.79407	.	.	1
26280	0	6.00	1	0.79407	.	.	1
26281	0	6.00	1	0.79407	.	.	1
26282	0	6.00	1	0.79407	.	.	1
26283	0	6.00	1	0.79407	.	.	1
26284	0	6.00	1	0.79407	.	.	1
26285	0	6.00	1	0.79407	.	.	1

Obs	Sub_group	fuptimey	_CENSOR_	SURVIVAL	SDF_LCL	SDF_UCL	STRATUM
26286	0	6.00	1	0.79407	.	.	1
26287	0	6.00	1	0.79407	.	.	1
26288	0	6.00	1	0.79407	.	.	1
26289	0	6.00	1	0.79407	.	.	1
26290	0	6.00	1	0.79407	.	.	1
26291	0	6.00	1	0.79407	.	.	1
26292	0	6.00	1	0.79407	.	.	1
26293	0	6.00	1	0.79407	.	.	1
26294	0	6.00	1	0.79407	.	.	1
26295	0	6.00	1	0.79407	.	.	1
26296	0	6.00	1	0.79407	.	.	1
26297	0	6.00	1	0.79407	.	.	1
26298	0	6.00	1	0.79407	.	.	1
26299	0	6.00	1	0.79407	.	.	1
26300	0	6.00	1	0.79407	.	.	1
26301	0	6.01	0	0.79384	0.78934	0.79825	1
26302	0	6.01	1	0.79384	.	.	1
26303	0	6.01	1	0.79384	.	.	1
26304	0	6.01	1	0.79384	.	.	1
26305	0	6.01	1	0.79384	.	.	1
26306	0	6.01	1	0.79384	.	.	1
26307	0	6.01	1	0.79384	.	.	1
26308	0	6.01	1	0.79384	.	.	1
26309	0	6.01	1	0.79384	.	.	1
26310	0	6.01	1	0.79384	.	.	1
26311	0	6.01	1	0.79384	.	.	1
26312	0	6.01	1	0.79384	.	.	1
26313	0	6.01	1	0.79384	.	.	1
26314	0	6.01	1	0.79384	.	.	1
26315	0	6.01	1	0.79384	.	.	1
26316	0	6.01	1	0.79384	.	.	1
26317	0	6.01	1	0.79384	.	.	1
26318	0	6.01	1	0.79384	.	.	1
26319	0	6.01	1	0.79384	.	.	1
26320	0	6.01	1	0.79384	.	.	1

Obs	Sub_group	fuptimey	_CENSOR_	SURVIVAL	SDF_LCL	SDF_UCL	STRATUM
26321	0	6.01	1	0.79384	.	.	1
26322	0	6.01	1	0.79384	.	.	1
26323	0	6.01	1	0.79384	.	.	1
26324	0	6.01	1	0.79384	.	.	1
26325	0	6.01	1	0.79384	.	.	1
26326	0	6.01	1	0.79384	.	.	1
26327	0	6.01	1	0.79384	.	.	1
26328	0	6.01	1	0.79384	.	.	1
26329	0	6.01	1	0.79384	.	.	1
26330	0	6.02	0	0.79372	0.78922	0.79814	1
26331	0	6.02	1	0.79372	.	.	1
26332	0	6.02	1	0.79372	.	.	1
26333	0	6.02	1	0.79372	.	.	1
26334	0	6.02	1	0.79372	.	.	1
26335	0	6.02	1	0.79372	.	.	1
26336	0	6.02	1	0.79372	.	.	1
26337	0	6.02	1	0.79372	.	.	1
26338	0	6.02	1	0.79372	.	.	1
26339	0	6.02	1	0.79372	.	.	1
26340	0	6.02	1	0.79372	.	.	1
26341	0	6.02	1	0.79372	.	.	1
26342	0	6.02	1	0.79372	.	.	1
26343	0	6.02	1	0.79372	.	.	1
26344	0	6.02	1	0.79372	.	.	1
26345	0	6.02	1	0.79372	.	.	1
26346	0	6.02	1	0.79372	.	.	1
26347	0	6.02	1	0.79372	.	.	1
26348	0	6.02	1	0.79372	.	.	1
26349	0	6.02	1	0.79372	.	.	1
26350	0	6.02	1	0.79372	.	.	1
26351	0	6.02	1	0.79372	.	.	1
26352	0	6.02	1	0.79372	.	.	1
26353	0	6.02	1	0.79372	.	.	1
26354	0	6.02	1	0.79372	.	.	1
26355	0	6.02	1	0.79372	.	.	1

Obs	Sub_group	fuptimey	_CENSOR_	SURVIVAL	SDF_LCL	SDF_UCL	STRATUM
26356	0	6.02	1	0.79372	.	.	1
26357	0	6.02	1	0.79372	.	.	1
26358	0	6.02	1	0.79372	.	.	1
26359	0	6.03	0	0.79354	0.78904	0.79796	1
26360	0	6.03	1	0.79354	.	.	1
26361	0	6.03	1	0.79354	.	.	1
26362	0	6.03	1	0.79354	.	.	1
26363	0	6.03	1	0.79354	.	.	1
26364	0	6.03	1	0.79354	.	.	1
26365	0	6.03	1	0.79354	.	.	1
26366	0	6.03	1	0.79354	.	.	1
26367	0	6.03	1	0.79354	.	.	1
26368	0	6.03	1	0.79354	.	.	1
26369	0	6.03	1	0.79354	.	.	1
26370	0	6.03	1	0.79354	.	.	1
26371	0	6.03	1	0.79354	.	.	1
26372	0	6.03	1	0.79354	.	.	1
26373	0	6.03	1	0.79354	.	.	1
26374	0	6.03	1	0.79354	.	.	1
26375	0	6.03	1	0.79354	.	.	1
26376	0	6.03	1	0.79354	.	.	1
26377	0	6.03	1	0.79354	.	.	1
26378	0	6.03	1	0.79354	.	.	1
26379	0	6.03	1	0.79354	.	.	1
26380	0	6.03	1	0.79354	.	.	1
26381	0	6.03	1	0.79354	.	.	1
26382	0	6.03	1	0.79354	.	.	1
26383	0	6.03	1	0.79354	.	.	1
26384	0	6.04	0	0.79337	0.78886	0.79779	1
26385	0	6.04	1	0.79337	.	.	1
26386	0	6.04	1	0.79337	.	.	1
26387	0	6.04	1	0.79337	.	.	1
26388	0	6.04	1	0.79337	.	.	1
26389	0	6.04	1	0.79337	.	.	1
26390	0	6.04	1	0.79337	.	.	1

Obs	Sub_group	fuptimey	_CENSOR_	SURVIVAL	SDF_LCL	SDF_UCL	STRATUM
26391	0	6.04	1	0.79337	.	.	1
26392	0	6.04	1	0.79337	.	.	1
26393	0	6.04	1	0.79337	.	.	1
26394	0	6.04	1	0.79337	.	.	1
26395	0	6.04	1	0.79337	.	.	1
26396	0	6.04	1	0.79337	.	.	1
26397	0	6.04	1	0.79337	.	.	1
26398	0	6.04	1	0.79337	.	.	1
26399	0	6.04	1	0.79337	.	.	1
26400	0	6.04	1	0.79337	.	.	1
26401	0	6.04	1	0.79337	.	.	1
26402	0	6.04	1	0.79337	.	.	1
26403	0	6.04	1	0.79337	.	.	1
26404	0	6.04	1	0.79337	.	.	1
26405	0	6.04	1	0.79337	.	.	1
26406	0	6.04	1	0.79337	.	.	1
26407	0	6.04	1	0.79337	.	.	1
26408	0	6.04	1	0.79337	.	.	1
26409	0	6.04	1	0.79337	.	.	1
26410	0	6.04	1	0.79337	.	.	1
26411	0	6.04	1	0.79337	.	.	1
26412	0	6.05	0	0.79307	0.78856	0.79750	1
26413	0	6.05	1	0.79307	.	.	1
26414	0	6.05	1	0.79307	.	.	1
26415	0	6.05	1	0.79307	.	.	1
26416	0	6.05	1	0.79307	.	.	1
26417	0	6.05	1	0.79307	.	.	1
26418	0	6.05	1	0.79307	.	.	1
26419	0	6.05	1	0.79307	.	.	1
26420	0	6.05	1	0.79307	.	.	1
26421	0	6.05	1	0.79307	.	.	1
26422	0	6.05	1	0.79307	.	.	1
26423	0	6.05	1	0.79307	.	.	1
26424	0	6.05	1	0.79307	.	.	1
26425	0	6.05	1	0.79307	.	.	1

Obs	Sub_group	fuptimey	_CENSOR_	SURVIVAL	SDF_LCL	SDF_UCL	STRATUM
26426	0	6.05	1	0.79307	.	.	1
26427	0	6.05	1	0.79307	.	.	1
26428	0	6.05	1	0.79307	.	.	1
26429	0	6.05	1	0.79307	.	.	1
26430	0	6.05	1	0.79307	.	.	1
26431	0	6.05	1	0.79307	.	.	1
26432	0	6.05	1	0.79307	.	.	1
26433	0	6.05	1	0.79307	.	.	1
26434	0	6.05	1	0.79307	.	.	1
26435	0	6.05	1	0.79307	.	.	1
26436	0	6.05	1	0.79307	.	.	1
26437	0	6.05	1	0.79307	.	.	1
26438	0	6.05	1	0.79307	.	.	1
26439	0	6.05	1	0.79307	.	.	1
26440	0	6.05	1	0.79307	.	.	1
26441	0	6.05	1	0.79307	.	.	1
26442	0	6.05	1	0.79307	.	.	1
26443	0	6.05	1	0.79307	.	.	1
26444	0	6.05	1	0.79307	.	.	1
26445	0	6.05	1	0.79307	.	.	1
26446	0	6.05	1	0.79307	.	.	1
26447	0	6.06	0	0.79283	0.78831	0.79727	1
26448	0	6.06	1	0.79283	.	.	1
26449	0	6.06	1	0.79283	.	.	1
26450	0	6.06	1	0.79283	.	.	1
26451	0	6.06	1	0.79283	.	.	1
26452	0	6.06	1	0.79283	.	.	1
26453	0	6.06	1	0.79283	.	.	1
26454	0	6.06	1	0.79283	.	.	1
26455	0	6.06	1	0.79283	.	.	1
26456	0	6.06	1	0.79283	.	.	1
26457	0	6.06	1	0.79283	.	.	1
26458	0	6.06	1	0.79283	.	.	1
26459	0	6.06	1	0.79283	.	.	1
26460	0	6.06	1	0.79283	.	.	1

Obs	Sub_group	fuptimey	_CENSOR_	SURVIVAL	SDF_LCL	SDF_UCL	STRATUM
26461	0	6.06	1	0.79283	.	.	1
26462	0	6.06	1	0.79283	.	.	1
26463	0	6.06	1	0.79283	.	.	1
26464	0	6.06	1	0.79283	.	.	1
26465	0	6.06	1	0.79283	.	.	1
26466	0	6.06	1	0.79283	.	.	1
26467	0	6.06	1	0.79283	.	.	1
26468	0	6.06	1	0.79283	.	.	1
26469	0	6.06	1	0.79283	.	.	1
26470	0	6.06	1	0.79283	.	.	1
26471	0	6.06	1	0.79283	.	.	1
26472	0	6.06	1	0.79283	.	.	1
26473	0	6.06	1	0.79283	.	.	1
26474	0	6.06	1	0.79283	.	.	1
26475	0	6.07	0	0.79260	0.78807	0.79704	1
26476	0	6.07	1	0.79260	.	.	1
26477	0	6.07	1	0.79260	.	.	1
26478	0	6.07	1	0.79260	.	.	1
26479	0	6.07	1	0.79260	.	.	1
26480	0	6.07	1	0.79260	.	.	1
26481	0	6.07	1	0.79260	.	.	1
26482	0	6.07	1	0.79260	.	.	1
26483	0	6.07	1	0.79260	.	.	1
26484	0	6.07	1	0.79260	.	.	1
26485	0	6.07	1	0.79260	.	.	1
26486	0	6.07	1	0.79260	.	.	1
26487	0	6.07	1	0.79260	.	.	1
26488	0	6.07	1	0.79260	.	.	1
26489	0	6.07	1	0.79260	.	.	1
26490	0	6.07	1	0.79260	.	.	1
26491	0	6.07	1	0.79260	.	.	1
26492	0	6.07	1	0.79260	.	.	1
26493	0	6.07	1	0.79260	.	.	1
26494	0	6.07	1	0.79260	.	.	1
26495	0	6.07	1	0.79260	.	.	1

Obs	Sub_group	fuptimey	_CENSOR_	SURVIVAL	SDF_LCL	SDF_UCL	STRATUM
26496	0	6.07	1	0.79260	.	.	1
26497	0	6.07	1	0.79260	.	.	1
26498	0	6.07	1	0.79260	.	.	1
26499	0	6.07	1	0.79260	.	.	1
26500	0	6.07	1	0.79260	.	.	1
26501	0	6.07	1	0.79260	.	.	1
26502	0	6.07	1	0.79260	.	.	1
26503	0	6.08	1	0.79260	.	.	1
26504	0	6.08	1	0.79260	.	.	1
26505	0	6.08	1	0.79260	.	.	1
26506	0	6.08	1	0.79260	.	.	1
26507	0	6.08	1	0.79260	.	.	1
26508	0	6.08	1	0.79260	.	.	1
26509	0	6.08	1	0.79260	.	.	1
26510	0	6.08	1	0.79260	.	.	1
26511	0	6.08	1	0.79260	.	.	1
26512	0	6.08	1	0.79260	.	.	1
26513	0	6.08	1	0.79260	.	.	1
26514	0	6.08	1	0.79260	.	.	1
26515	0	6.08	1	0.79260	.	.	1
26516	0	6.08	1	0.79260	.	.	1
26517	0	6.08	1	0.79260	.	.	1
26518	0	6.08	1	0.79260	.	.	1
26519	0	6.08	1	0.79260	.	.	1
26520	0	6.08	1	0.79260	.	.	1
26521	0	6.08	1	0.79260	.	.	1
26522	0	6.08	1	0.79260	.	.	1
26523	0	6.08	1	0.79260	.	.	1
26524	0	6.08	1	0.79260	.	.	1
26525	0	6.08	1	0.79260	.	.	1
26526	0	6.08	1	0.79260	.	.	1
26527	0	6.08	1	0.79260	.	.	1
26528	0	6.08	1	0.79260	.	.	1
26529	0	6.08	1	0.79260	.	.	1
26530	0	6.08	1	0.79260	.	.	1

Obs	Sub_group	fuptimey	_CENSOR_	SURVIVAL	SDF_LCL	SDF_UCL	STRATUM
26531	0	6.08	1	0.79260	.	.	1
26532	0	6.08	1	0.79260	.	.	1
26533	0	6.08	1	0.79260	.	.	1
26534	0	6.08	1	0.79260	.	.	1
26535	0	6.08	1	0.79260	.	.	1
26536	0	6.08	1	0.79260	.	.	1
26537	0	6.08	1	0.79260	.	.	1
26538	0	6.08	1	0.79260	.	.	1
26539	0	6.08	1	0.79260	.	.	1
26540	0	6.08	1	0.79260	.	.	1
26541	0	6.08	1	0.79260	.	.	1
26542	0	6.08	1	0.79260	.	.	1
26543	0	6.09	0	0.79230	0.78777	0.79674	1
26544	0	6.09	1	0.79230	.	.	1
26545	0	6.09	1	0.79230	.	.	1
26546	0	6.09	1	0.79230	.	.	1
26547	0	6.09	1	0.79230	.	.	1
26548	0	6.09	1	0.79230	.	.	1
26549	0	6.09	1	0.79230	.	.	1
26550	0	6.09	1	0.79230	.	.	1
26551	0	6.09	1	0.79230	.	.	1
26552	0	6.09	1	0.79230	.	.	1
26553	0	6.09	1	0.79230	.	.	1
26554	0	6.09	1	0.79230	.	.	1
26555	0	6.09	1	0.79230	.	.	1
26556	0	6.09	1	0.79230	.	.	1
26557	0	6.09	1	0.79230	.	.	1
26558	0	6.09	1	0.79230	.	.	1
26559	0	6.09	1	0.79230	.	.	1
26560	0	6.09	1	0.79230	.	.	1
26561	0	6.09	1	0.79230	.	.	1
26562	0	6.09	1	0.79230	.	.	1
26563	0	6.09	1	0.79230	.	.	1
26564	0	6.09	1	0.79230	.	.	1
26565	0	6.09	1	0.79230	.	.	1

Obs	Sub_group	fuptimey	_CENSOR_	SURVIVAL	SDF_LCL	SDF_UCL	STRATUM
26566	0	6.09	1	0.79230	.	.	1
26567	0	6.09	1	0.79230	.	.	1
26568	0	6.09	1	0.79230	.	.	1
26569	0	6.09	1	0.79230	.	.	1
26570	0	6.09	1	0.79230	.	.	1
26571	0	6.09	1	0.79230	.	.	1
26572	0	6.09	1	0.79230	.	.	1
26573	0	6.09	1	0.79230	.	.	1
26574	0	6.09	1	0.79230	.	.	1
26575	0	6.09	1	0.79230	.	.	1
26576	0	6.09	1	0.79230	.	.	1
26577	0	6.09	1	0.79230	.	.	1
26578	0	6.09	1	0.79230	.	.	1
26579	0	6.09	1	0.79230	.	.	1
26580	0	6.09	1	0.79230	.	.	1
26581	0	6.09	1	0.79230	.	.	1
26582	0	6.09	1	0.79230	.	.	1
26583	0	6.09	1	0.79230	.	.	1
26584	0	6.09	1	0.79230	.	.	1
26585	0	6.10	0	0.79224	0.78771	0.79668	1
26586	0	6.10	1	0.79224	.	.	1
26587	0	6.10	1	0.79224	.	.	1
26588	0	6.10	1	0.79224	.	.	1
26589	0	6.10	1	0.79224	.	.	1
26590	0	6.10	1	0.79224	.	.	1
26591	0	6.10	1	0.79224	.	.	1
26592	0	6.10	1	0.79224	.	.	1
26593	0	6.10	1	0.79224	.	.	1
26594	0	6.10	1	0.79224	.	.	1
26595	0	6.10	1	0.79224	.	.	1
26596	0	6.10	1	0.79224	.	.	1
26597	0	6.10	1	0.79224	.	.	1
26598	0	6.10	1	0.79224	.	.	1
26599	0	6.10	1	0.79224	.	.	1
26600	0	6.10	1	0.79224	.	.	1

Obs	Sub_group	fuptimey	_CENSOR_	SURVIVAL	SDF_LCL	SDF_UCL	STRATUM
26601	0	6.10	1	0.79224	.	.	1
26602	0	6.10	1	0.79224	.	.	1
26603	0	6.10	1	0.79224	.	.	1
26604	0	6.10	1	0.79224	.	.	1
26605	0	6.10	1	0.79224	.	.	1
26606	0	6.10	1	0.79224	.	.	1
26607	0	6.10	1	0.79224	.	.	1
26608	0	6.10	1	0.79224	.	.	1
26609	0	6.10	1	0.79224	.	.	1
26610	0	6.10	1	0.79224	.	.	1
26611	0	6.10	1	0.79224	.	.	1
26612	0	6.10	1	0.79224	.	.	1
26613	0	6.10	1	0.79224	.	.	1
26614	0	6.10	1	0.79224	.	.	1
26615	0	6.10	1	0.79224	.	.	1
26616	0	6.10	1	0.79224	.	.	1
26617	0	6.10	1	0.79224	.	.	1
26618	0	6.10	1	0.79224	.	.	1
26619	0	6.11	0	0.79206	0.78752	0.79651	1
26620	0	6.11	1	0.79206	.	.	1
26621	0	6.11	1	0.79206	.	.	1
26622	0	6.11	1	0.79206	.	.	1
26623	0	6.11	1	0.79206	.	.	1
26624	0	6.11	1	0.79206	.	.	1
26625	0	6.11	1	0.79206	.	.	1
26626	0	6.11	1	0.79206	.	.	1
26627	0	6.11	1	0.79206	.	.	1
26628	0	6.11	1	0.79206	.	.	1
26629	0	6.11	1	0.79206	.	.	1
26630	0	6.11	1	0.79206	.	.	1
26631	0	6.11	1	0.79206	.	.	1
26632	0	6.11	1	0.79206	.	.	1
26633	0	6.11	1	0.79206	.	.	1
26634	0	6.11	1	0.79206	.	.	1
26635	0	6.11	1	0.79206	.	.	1

Obs	Sub_group	fuptimey	_CENSOR_	SURVIVAL	SDF_LCL	SDF_UCL	STRATUM
26636	0	6.11	1	0.79206	.	.	1
26637	0	6.11	1	0.79206	.	.	1
26638	0	6.11	1	0.79206	.	.	1
26639	0	6.11	1	0.79206	.	.	1
26640	0	6.11	1	0.79206	.	.	1
26641	0	6.11	1	0.79206	.	.	1
26642	0	6.11	1	0.79206	.	.	1
26643	0	6.11	1	0.79206	.	.	1
26644	0	6.11	1	0.79206	.	.	1
26645	0	6.11	1	0.79206	.	.	1
26646	0	6.11	1	0.79206	.	.	1
26647	0	6.12	1	0.79206	.	.	1
26648	0	6.12	1	0.79206	.	.	1
26649	0	6.12	1	0.79206	.	.	1
26650	0	6.12	1	0.79206	.	.	1
26651	0	6.12	1	0.79206	.	.	1
26652	0	6.12	1	0.79206	.	.	1
26653	0	6.12	1	0.79206	.	.	1
26654	0	6.12	1	0.79206	.	.	1
26655	0	6.12	1	0.79206	.	.	1
26656	0	6.12	1	0.79206	.	.	1
26657	0	6.12	1	0.79206	.	.	1
26658	0	6.12	1	0.79206	.	.	1
26659	0	6.12	1	0.79206	.	.	1
26660	0	6.12	1	0.79206	.	.	1
26661	0	6.12	1	0.79206	.	.	1
26662	0	6.12	1	0.79206	.	.	1
26663	0	6.12	1	0.79206	.	.	1
26664	0	6.12	1	0.79206	.	.	1
26665	0	6.12	1	0.79206	.	.	1
26666	0	6.12	1	0.79206	.	.	1
26667	0	6.12	1	0.79206	.	.	1
26668	0	6.12	1	0.79206	.	.	1
26669	0	6.12	1	0.79206	.	.	1
26670	0	6.12	1	0.79206	.	.	1

Obs	Sub_group	fuptimey	_CENSOR_	SURVIVAL	SDF_LCL	SDF_UCL	STRATUM
26671	0	6.12	1	0.79206	.	.	1
26672	0	6.12	1	0.79206	.	.	1
26673	0	6.12	1	0.79206	.	.	1
26674	0	6.12	1	0.79206	.	.	1
26675	0	6.12	1	0.79206	.	.	1
26676	0	6.12	1	0.79206	.	.	1
26677	0	6.12	1	0.79206	.	.	1
26678	0	6.12	1	0.79206	.	.	1
26679	0	6.12	1	0.79206	.	.	1
26680	0	6.12	1	0.79206	.	.	1
26681	0	6.12	1	0.79206	.	.	1
26682	0	6.12	1	0.79206	.	.	1
26683	0	6.12	1	0.79206	.	.	1
26684	0	6.12	1	0.79206	.	.	1
26685	0	6.12	1	0.79206	.	.	1
26686	0	6.12	1	0.79206	.	.	1
26687	0	6.12	1	0.79206	.	.	1
26688	0	6.12	1	0.79206	.	.	1
26689	0	6.12	1	0.79206	.	.	1
26690	0	6.12	1	0.79206	.	.	1
26691	0	6.12	1	0.79206	.	.	1
26692	0	6.13	0	0.79169	0.78715	0.79615	1
26693	0	6.13	1	0.79169	.	.	1
26694	0	6.13	1	0.79169	.	.	1
26695	0	6.13	1	0.79169	.	.	1
26696	0	6.13	1	0.79169	.	.	1
26697	0	6.13	1	0.79169	.	.	1
26698	0	6.13	1	0.79169	.	.	1
26699	0	6.13	1	0.79169	.	.	1
26700	0	6.13	1	0.79169	.	.	1
26701	0	6.13	1	0.79169	.	.	1
26702	0	6.13	1	0.79169	.	.	1
26703	0	6.13	1	0.79169	.	.	1
26704	0	6.13	1	0.79169	.	.	1
26705	0	6.13	1	0.79169	.	.	1

Obs	Sub_group	fuptimey	_CENSOR_	SURVIVAL	SDF_LCL	SDF_UCL	STRATUM
26706	0	6.13	1	0.79169	.	.	1
26707	0	6.13	1	0.79169	.	.	1
26708	0	6.13	1	0.79169	.	.	1
26709	0	6.13	1	0.79169	.	.	1
26710	0	6.13	1	0.79169	.	.	1
26711	0	6.13	1	0.79169	.	.	1
26712	0	6.13	1	0.79169	.	.	1
26713	0	6.13	1	0.79169	.	.	1
26714	0	6.13	1	0.79169	.	.	1
26715	0	6.13	1	0.79169	.	.	1
26716	0	6.13	1	0.79169	.	.	1
26717	0	6.13	1	0.79169	.	.	1
26718	0	6.13	1	0.79169	.	.	1
26719	0	6.13	1	0.79169	.	.	1
26720	0	6.13	1	0.79169	.	.	1
26721	0	6.13	1	0.79169	.	.	1
26722	0	6.13	1	0.79169	.	.	1
26723	0	6.14	0	0.79145	0.78691	0.79592	1
26724	0	6.14	1	0.79145	.	.	1
26725	0	6.14	1	0.79145	.	.	1
26726	0	6.14	1	0.79145	.	.	1
26727	0	6.14	1	0.79145	.	.	1
26728	0	6.14	1	0.79145	.	.	1
26729	0	6.14	1	0.79145	.	.	1
26730	0	6.14	1	0.79145	.	.	1
26731	0	6.14	1	0.79145	.	.	1
26732	0	6.14	1	0.79145	.	.	1
26733	0	6.14	1	0.79145	.	.	1
26734	0	6.14	1	0.79145	.	.	1
26735	0	6.14	1	0.79145	.	.	1
26736	0	6.14	1	0.79145	.	.	1
26737	0	6.14	1	0.79145	.	.	1
26738	0	6.14	1	0.79145	.	.	1
26739	0	6.14	1	0.79145	.	.	1
26740	0	6.14	1	0.79145	.	.	1

Obs	Sub_group	fuptimey	_CENSOR_	SURVIVAL	SDF_LCL	SDF_UCL	STRATUM
26741	0	6.14	1	0.79145	.	.	1
26742	0	6.14	1	0.79145	.	.	1
26743	0	6.14	1	0.79145	.	.	1
26744	0	6.14	1	0.79145	.	.	1
26745	0	6.14	1	0.79145	.	.	1
26746	0	6.14	1	0.79145	.	.	1
26747	0	6.14	1	0.79145	.	.	1
26748	0	6.14	1	0.79145	.	.	1
26749	0	6.14	1	0.79145	.	.	1
26750	0	6.14	1	0.79145	.	.	1
26751	0	6.14	1	0.79145	.	.	1
26752	0	6.14	1	0.79145	.	.	1
26753	0	6.14	1	0.79145	.	.	1
26754	0	6.14	1	0.79145	.	.	1
26755	0	6.14	1	0.79145	.	.	1
26756	0	6.14	1	0.79145	.	.	1
26757	0	6.14	1	0.79145	.	.	1
26758	0	6.14	1	0.79145	.	.	1
26759	0	6.14	1	0.79145	.	.	1
26760	0	6.14	1	0.79145	.	.	1
26761	0	6.14	1	0.79145	.	.	1
26762	0	6.14	1	0.79145	.	.	1
26763	0	6.14	1	0.79145	.	.	1
26764	0	6.14	1	0.79145	.	.	1
26765	0	6.15	0	0.79127	0.78672	0.79574	1
26766	0	6.15	1	0.79127	.	.	1
26767	0	6.15	1	0.79127	.	.	1
26768	0	6.15	1	0.79127	.	.	1
26769	0	6.15	1	0.79127	.	.	1
26770	0	6.15	1	0.79127	.	.	1
26771	0	6.15	1	0.79127	.	.	1
26772	0	6.15	1	0.79127	.	.	1
26773	0	6.15	1	0.79127	.	.	1
26774	0	6.15	1	0.79127	.	.	1
26775	0	6.15	1	0.79127	.	.	1

Obs	Sub_group	fuptimey	_CENSOR_	SURVIVAL	SDF_LCL	SDF_UCL	STRATUM
26776	0	6.15	1	0.79127	.	.	1
26777	0	6.15	1	0.79127	.	.	1
26778	0	6.15	1	0.79127	.	.	1
26779	0	6.15	1	0.79127	.	.	1
26780	0	6.15	1	0.79127	.	.	1
26781	0	6.15	1	0.79127	.	.	1
26782	0	6.15	1	0.79127	.	.	1
26783	0	6.15	1	0.79127	.	.	1
26784	0	6.15	1	0.79127	.	.	1
26785	0	6.15	1	0.79127	.	.	1
26786	0	6.15	1	0.79127	.	.	1
26787	0	6.15	1	0.79127	.	.	1
26788	0	6.15	1	0.79127	.	.	1
26789	0	6.15	1	0.79127	.	.	1
26790	0	6.15	1	0.79127	.	.	1
26791	0	6.15	1	0.79127	.	.	1
26792	0	6.15	1	0.79127	.	.	1
26793	0	6.15	1	0.79127	.	.	1
26794	0	6.16	1	0.79127	.	.	1
26795	0	6.16	1	0.79127	.	.	1
26796	0	6.16	1	0.79127	.	.	1
26797	0	6.16	1	0.79127	.	.	1
26798	0	6.16	1	0.79127	.	.	1
26799	0	6.16	1	0.79127	.	.	1
26800	0	6.16	1	0.79127	.	.	1
26801	0	6.16	1	0.79127	.	.	1
26802	0	6.16	1	0.79127	.	.	1
26803	0	6.16	1	0.79127	.	.	1
26804	0	6.16	1	0.79127	.	.	1
26805	0	6.16	1	0.79127	.	.	1
26806	0	6.16	1	0.79127	.	.	1
26807	0	6.16	1	0.79127	.	.	1
26808	0	6.16	1	0.79127	.	.	1
26809	0	6.16	1	0.79127	.	.	1
26810	0	6.16	1	0.79127	.	.	1

Obs	Sub_group	fuptimey	_CENSOR_	SURVIVAL	SDF_LCL	SDF_UCL	STRATUM
26811	0	6.16	1	0.79127	.	.	1
26812	0	6.16	1	0.79127	.	.	1
26813	0	6.16	1	0.79127	.	.	1
26814	0	6.16	1	0.79127	.	.	1
26815	0	6.16	1	0.79127	.	.	1
26816	0	6.16	1	0.79127	.	.	1
26817	0	6.16	1	0.79127	.	.	1
26818	0	6.16	1	0.79127	.	.	1
26819	0	6.16	1	0.79127	.	.	1
26820	0	6.16	1	0.79127	.	.	1
26821	0	6.16	1	0.79127	.	.	1
26822	0	6.16	1	0.79127	.	.	1
26823	0	6.16	1	0.79127	.	.	1
26824	0	6.16	1	0.79127	.	.	1
26825	0	6.16	1	0.79127	.	.	1
26826	0	6.16	1	0.79127	.	.	1
26827	0	6.16	1	0.79127	.	.	1
26828	0	6.16	1	0.79127	.	.	1
26829	0	6.16	1	0.79127	.	.	1
26830	0	6.16	1	0.79127	.	.	1
26831	0	6.16	1	0.79127	.	.	1
26832	0	6.16	1	0.79127	.	.	1
26833	0	6.17	0	0.79097	0.78641	0.79544	1
26834	0	6.17	1	0.79097	.	.	1
26835	0	6.17	1	0.79097	.	.	1
26836	0	6.17	1	0.79097	.	.	1
26837	0	6.17	1	0.79097	.	.	1
26838	0	6.17	1	0.79097	.	.	1
26839	0	6.17	1	0.79097	.	.	1
26840	0	6.17	1	0.79097	.	.	1
26841	0	6.17	1	0.79097	.	.	1
26842	0	6.17	1	0.79097	.	.	1
26843	0	6.17	1	0.79097	.	.	1
26844	0	6.17	1	0.79097	.	.	1
26845	0	6.17	1	0.79097	.	.	1

Obs	Sub_group	fuptimey	_CENSOR_	SURVIVAL	SDF_LCL	SDF_UCL	STRATUM
**26846**	0	6.17	1	0.79097	.	.	1
**26847**	0	6.17	1	0.79097	.	.	1
**26848**	0	6.17	1	0.79097	.	.	1
**26849**	0	6.17	1	0.79097	.	.	1
**26850**	0	6.17	1	0.79097	.	.	1
**26851**	0	6.17	1	0.79097	.	.	1
**26852**	0	6.17	1	0.79097	.	.	1
**26853**	0	6.17	1	0.79097	.	.	1
**26854**	0	6.17	1	0.79097	.	.	1
**26855**	0	6.17	1	0.79097	.	.	1
**26856**	0	6.17	1	0.79097	.	.	1
**26857**	0	6.17	1	0.79097	.	.	1
**26858**	0	6.17	1	0.79097	.	.	1
**26859**	0	6.17	1	0.79097	.	.	1
**26860**	0	6.17	1	0.79097	.	.	1
**26861**	0	6.17	1	0.79097	.	.	1
**26862**	0	6.17	1	0.79097	.	.	1
**26863**	0	6.17	1	0.79097	.	.	1
**26864**	0	6.17	1	0.79097	.	.	1
**26865**	0	6.17	1	0.79097	.	.	1
**26866**	0	6.17	1	0.79097	.	.	1
**26867**	0	6.17	1	0.79097	.	.	1
**26868**	0	6.18	0	0.79078	0.78622	0.79526	1
**26869**	0	6.18	1	0.79078	.	.	1
**26870**	0	6.18	1	0.79078	.	.	1
**26871**	0	6.18	1	0.79078	.	.	1
**26872**	0	6.18	1	0.79078	.	.	1
**26873**	0	6.18	1	0.79078	.	.	1
**26874**	0	6.18	1	0.79078	.	.	1
**26875**	0	6.18	1	0.79078	.	.	1
**26876**	0	6.18	1	0.79078	.	.	1
**26877**	0	6.18	1	0.79078	.	.	1
**26878**	0	6.18	1	0.79078	.	.	1
**26879**	0	6.18	1	0.79078	.	.	1
**26880**	0	6.18	1	0.79078	.	.	1

Obs	Sub_group	fuptimey	_CENSOR_	SURVIVAL	SDF_LCL	SDF_UCL	STRATUM
26881	0	6.18	1	0.79078	.	.	1
26882	0	6.18	1	0.79078	.	.	1
26883	0	6.18	1	0.79078	.	.	1
26884	0	6.18	1	0.79078	.	.	1
26885	0	6.18	1	0.79078	.	.	1
26886	0	6.18	1	0.79078	.	.	1
26887	0	6.18	1	0.79078	.	.	1
26888	0	6.18	1	0.79078	.	.	1
26889	0	6.18	1	0.79078	.	.	1
26890	0	6.18	1	0.79078	.	.	1
26891	0	6.18	1	0.79078	.	.	1
26892	0	6.18	1	0.79078	.	.	1
26893	0	6.18	1	0.79078	.	.	1
26894	0	6.18	1	0.79078	.	.	1
26895	0	6.18	1	0.79078	.	.	1
26896	0	6.18	1	0.79078	.	.	1
26897	0	6.18	1	0.79078	.	.	1
26898	0	6.18	1	0.79078	.	.	1
26899	0	6.18	1	0.79078	.	.	1
26900	0	6.18	1	0.79078	.	.	1
26901	0	6.19	0	0.79072	0.78616	0.79520	1
26902	0	6.19	1	0.79072	.	.	1
26903	0	6.19	1	0.79072	.	.	1
26904	0	6.19	1	0.79072	.	.	1
26905	0	6.19	1	0.79072	.	.	1
26906	0	6.19	1	0.79072	.	.	1
26907	0	6.19	1	0.79072	.	.	1
26908	0	6.19	1	0.79072	.	.	1
26909	0	6.19	1	0.79072	.	.	1
26910	0	6.19	1	0.79072	.	.	1
26911	0	6.19	1	0.79072	.	.	1
26912	0	6.19	1	0.79072	.	.	1
26913	0	6.19	1	0.79072	.	.	1
26914	0	6.19	1	0.79072	.	.	1
26915	0	6.19	1	0.79072	.	.	1

Obs	Sub_group	fuptimey	_CENSOR_	SURVIVAL	SDF_LCL	SDF_UCL	STRATUM
**26916**	0	6.19	1	0.79072	.	.	1
**26917**	0	6.19	1	0.79072	.	.	1
**26918**	0	6.19	1	0.79072	.	.	1
**26919**	0	6.19	1	0.79072	.	.	1
**26920**	0	6.19	1	0.79072	.	.	1
**26921**	0	6.19	1	0.79072	.	.	1
**26922**	0	6.19	1	0.79072	.	.	1
**26923**	0	6.19	1	0.79072	.	.	1
**26924**	0	6.19	1	0.79072	.	.	1
**26925**	0	6.19	1	0.79072	.	.	1
**26926**	0	6.19	1	0.79072	.	.	1
**26927**	0	6.19	1	0.79072	.	.	1
**26928**	0	6.19	1	0.79072	.	.	1
**26929**	0	6.19	1	0.79072	.	.	1
**26930**	0	6.19	1	0.79072	.	.	1
**26931**	0	6.20	0	0.79060	0.78603	0.79508	1
**26932**	0	6.20	1	0.79060	.	.	1
**26933**	0	6.20	1	0.79060	.	.	1
**26934**	0	6.20	1	0.79060	.	.	1
**26935**	0	6.20	1	0.79060	.	.	1
**26936**	0	6.20	1	0.79060	.	.	1
**26937**	0	6.20	1	0.79060	.	.	1
**26938**	0	6.20	1	0.79060	.	.	1
**26939**	0	6.20	1	0.79060	.	.	1
**26940**	0	6.20	1	0.79060	.	.	1
**26941**	0	6.20	1	0.79060	.	.	1
**26942**	0	6.20	1	0.79060	.	.	1
**26943**	0	6.20	1	0.79060	.	.	1
**26944**	0	6.20	1	0.79060	.	.	1
**26945**	0	6.20	1	0.79060	.	.	1
**26946**	0	6.20	1	0.79060	.	.	1
**26947**	0	6.20	1	0.79060	.	.	1
**26948**	0	6.20	1	0.79060	.	.	1
**26949**	0	6.20	1	0.79060	.	.	1
**26950**	0	6.20	1	0.79060	.	.	1

Obs	Sub_group	fuptimey	_CENSOR_	SURVIVAL	SDF_LCL	SDF_UCL	STRATUM
26951	0	6.20	1	0.79060	.	.	1
26952	0	6.20	1	0.79060	.	.	1
26953	0	6.20	1	0.79060	.	.	1
26954	0	6.20	1	0.79060	.	.	1
26955	0	6.20	1	0.79060	.	.	1
26956	0	6.20	1	0.79060	.	.	1
26957	0	6.20	1	0.79060	.	.	1
26958	0	6.20	1	0.79060	.	.	1
26959	0	6.20	1	0.79060	.	.	1
26960	0	6.20	1	0.79060	.	.	1
26961	0	6.20	1	0.79060	.	.	1
26962	0	6.20	1	0.79060	.	.	1
26963	0	6.20	1	0.79060	.	.	1
26964	0	6.20	1	0.79060	.	.	1
26965	0	6.20	1	0.79060	.	.	1
26966	0	6.20	1	0.79060	.	.	1
26967	0	6.20	1	0.79060	.	.	1
26968	0	6.20	1	0.79060	.	.	1
26969	0	6.20	1	0.79060	.	.	1
26970	0	6.20	1	0.79060	.	.	1
26971	0	6.20	1	0.79060	.	.	1
26972	0	6.20	1	0.79060	.	.	1
26973	0	6.20	1	0.79060	.	.	1
26974	0	6.20	1	0.79060	.	.	1
26975	0	6.20	1	0.79060	.	.	1
26976	0	6.20	1	0.79060	.	.	1
26977	0	6.20	1	0.79060	.	.	1
26978	0	6.21	0	0.79029	0.78572	0.79478	1
26979	0	6.21	1	0.79029	.	.	1
26980	0	6.21	1	0.79029	.	.	1
26981	0	6.21	1	0.79029	.	.	1
26982	0	6.21	1	0.79029	.	.	1
26983	0	6.21	1	0.79029	.	.	1
26984	0	6.21	1	0.79029	.	.	1
26985	0	6.21	1	0.79029	.	.	1

Obs	Sub_group	fuptimey	_CENSOR_	SURVIVAL	SDF_LCL	SDF_UCL	STRATUM
26986	0	6.21	1	0.79029	.	.	1
26987	0	6.21	1	0.79029	.	.	1
26988	0	6.21	1	0.79029	.	.	1
26989	0	6.21	1	0.79029	.	.	1
26990	0	6.21	1	0.79029	.	.	1
26991	0	6.21	1	0.79029	.	.	1
26992	0	6.21	1	0.79029	.	.	1
26993	0	6.21	1	0.79029	.	.	1
26994	0	6.21	1	0.79029	.	.	1
26995	0	6.21	1	0.79029	.	.	1
26996	0	6.21	1	0.79029	.	.	1
26997	0	6.21	1	0.79029	.	.	1
26998	0	6.21	1	0.79029	.	.	1
26999	0	6.21	1	0.79029	.	.	1
27000	0	6.21	1	0.79029	.	.	1
27001	0	6.21	1	0.79029	.	.	1
27002	0	6.21	1	0.79029	.	.	1
27003	0	6.21	1	0.79029	.	.	1
27004	0	6.21	1	0.79029	.	.	1
27005	0	6.21	1	0.79029	.	.	1
27006	0	6.21	1	0.79029	.	.	1
27007	0	6.21	1	0.79029	.	.	1
27008	0	6.21	1	0.79029	.	.	1
27009	0	6.21	1	0.79029	.	.	1
27010	0	6.21	1	0.79029	.	.	1
27011	0	6.22	0	0.79010	0.78553	0.79459	1
27012	0	6.22	1	0.79010	.	.	1
27013	0	6.22	1	0.79010	.	.	1
27014	0	6.22	1	0.79010	.	.	1
27015	0	6.22	1	0.79010	.	.	1
27016	0	6.22	1	0.79010	.	.	1
27017	0	6.22	1	0.79010	.	.	1
27018	0	6.22	1	0.79010	.	.	1
27019	0	6.22	1	0.79010	.	.	1
27020	0	6.22	1	0.79010	.	.	1

Obs	Sub_group	fuptimey	_CENSOR_	SURVIVAL	SDF_LCL	SDF_UCL	STRATUM
**27021**	0	6.22	1	0.79010	.	.	1
**27022**	0	6.22	1	0.79010	.	.	1
**27023**	0	6.22	1	0.79010	.	.	1
**27024**	0	6.22	1	0.79010	.	.	1
**27025**	0	6.22	1	0.79010	.	.	1
**27026**	0	6.22	1	0.79010	.	.	1
**27027**	0	6.22	1	0.79010	.	.	1
**27028**	0	6.22	1	0.79010	.	.	1
**27029**	0	6.22	1	0.79010	.	.	1
**27030**	0	6.22	1	0.79010	.	.	1
**27031**	0	6.22	1	0.79010	.	.	1
**27032**	0	6.22	1	0.79010	.	.	1
**27033**	0	6.22	1	0.79010	.	.	1
**27034**	0	6.22	1	0.79010	.	.	1
**27035**	0	6.22	1	0.79010	.	.	1
**27036**	0	6.22	1	0.79010	.	.	1
**27037**	0	6.22	1	0.79010	.	.	1
**27038**	0	6.22	1	0.79010	.	.	1
**27039**	0	6.22	1	0.79010	.	.	1
**27040**	0	6.22	1	0.79010	.	.	1
**27041**	0	6.22	1	0.79010	.	.	1
**27042**	0	6.22	1	0.79010	.	.	1
**27043**	0	6.22	1	0.79010	.	.	1
**27044**	0	6.22	1	0.79010	.	.	1
**27045**	0	6.22	1	0.79010	.	.	1
**27046**	0	6.22	1	0.79010	.	.	1
**27047**	0	6.22	1	0.79010	.	.	1
**27048**	0	6.22	1	0.79010	.	.	1
**27049**	0	6.22	1	0.79010	.	.	1
**27050**	0	6.22	1	0.79010	.	.	1
**27051**	0	6.22	1	0.79010	.	.	1
**27052**	0	6.22	1	0.79010	.	.	1
**27053**	0	6.22	1	0.79010	.	.	1
**27054**	0	6.22	1	0.79010	.	.	1
**27055**	0	6.22	1	0.79010	.	.	1

Obs	Sub_group	fuptimey	_CENSOR_	SURVIVAL	SDF_LCL	SDF_UCL	STRATUM
**27056**	0	6.22	1	0.79010	.	.	1
**27057**	0	6.22	1	0.79010	.	.	1
**27058**	0	6.22	1	0.79010	.	.	1
**27059**	0	6.22	1	0.79010	.	.	1
**27060**	0	6.22	1	0.79010	.	.	1
**27061**	0	6.22	1	0.79010	.	.	1
**27062**	0	6.22	1	0.79010	.	.	1
**27063**	0	6.23	0	0.78998	0.78540	0.79447	1
**27064**	0	6.23	1	0.78998	.	.	1
**27065**	0	6.23	1	0.78998	.	.	1
**27066**	0	6.23	1	0.78998	.	.	1
**27067**	0	6.23	1	0.78998	.	.	1
**27068**	0	6.23	1	0.78998	.	.	1
**27069**	0	6.23	1	0.78998	.	.	1
**27070**	0	6.23	1	0.78998	.	.	1
**27071**	0	6.23	1	0.78998	.	.	1
**27072**	0	6.23	1	0.78998	.	.	1
**27073**	0	6.23	1	0.78998	.	.	1
**27074**	0	6.23	1	0.78998	.	.	1
**27075**	0	6.23	1	0.78998	.	.	1
**27076**	0	6.23	1	0.78998	.	.	1
**27077**	0	6.23	1	0.78998	.	.	1
**27078**	0	6.23	1	0.78998	.	.	1
**27079**	0	6.23	1	0.78998	.	.	1
**27080**	0	6.23	1	0.78998	.	.	1
**27081**	0	6.23	1	0.78998	.	.	1
**27082**	0	6.23	1	0.78998	.	.	1
**27083**	0	6.23	1	0.78998	.	.	1
**27084**	0	6.23	1	0.78998	.	.	1
**27085**	0	6.23	1	0.78998	.	.	1
**27086**	0	6.23	1	0.78998	.	.	1
**27087**	0	6.23	1	0.78998	.	.	1
**27088**	0	6.23	1	0.78998	.	.	1
**27089**	0	6.23	1	0.78998	.	.	1
**27090**	0	6.23	1	0.78998	.	.	1

Obs	Sub_group	fuptimey	_CENSOR_	SURVIVAL	SDF_LCL	SDF_UCL	STRATUM
**27091**	0	6.23	1	0.78998	.	.	1
**27092**	0	6.23	1	0.78998	.	.	1
**27093**	0	6.23	1	0.78998	.	.	1
**27094**	0	6.23	1	0.78998	.	.	1
**27095**	0	6.23	1	0.78998	.	.	1
**27096**	0	6.23	1	0.78998	.	.	1
**27097**	0	6.23	1	0.78998	.	.	1
**27098**	0	6.23	1	0.78998	.	.	1
**27099**	0	6.23	1	0.78998	.	.	1
**27100**	0	6.23	1	0.78998	.	.	1
**27101**	0	6.23	1	0.78998	.	.	1
**27102**	0	6.23	1	0.78998	.	.	1
**27103**	0	6.23	1	0.78998	.	.	1
**27104**	0	6.23	1	0.78998	.	.	1
**27105**	0	6.23	1	0.78998	.	.	1
**27106**	0	6.23	1	0.78998	.	.	1
**27107**	0	6.24	0	0.78985	0.78528	0.79435	1
**27108**	0	6.24	1	0.78985	.	.	1
**27109**	0	6.24	1	0.78985	.	.	1
**27110**	0	6.24	1	0.78985	.	.	1
**27111**	0	6.24	1	0.78985	.	.	1
**27112**	0	6.24	1	0.78985	.	.	1
**27113**	0	6.24	1	0.78985	.	.	1
**27114**	0	6.24	1	0.78985	.	.	1
**27115**	0	6.24	1	0.78985	.	.	1
**27116**	0	6.24	1	0.78985	.	.	1
**27117**	0	6.24	1	0.78985	.	.	1
**27118**	0	6.24	1	0.78985	.	.	1
**27119**	0	6.24	1	0.78985	.	.	1
**27120**	0	6.24	1	0.78985	.	.	1
**27121**	0	6.24	1	0.78985	.	.	1
**27122**	0	6.24	1	0.78985	.	.	1
**27123**	0	6.24	1	0.78985	.	.	1
**27124**	0	6.24	1	0.78985	.	.	1
**27125**	0	6.24	1	0.78985	.	.	1

Obs	Sub_group	fuptimey	_CENSOR_	SURVIVAL	SDF_LCL	SDF_UCL	STRATUM
**27126**	0	6.24	1	0.78985	.	.	1
**27127**	0	6.24	1	0.78985	.	.	1
**27128**	0	6.24	1	0.78985	.	.	1
**27129**	0	6.24	1	0.78985	.	.	1
**27130**	0	6.24	1	0.78985	.	.	1
**27131**	0	6.24	1	0.78985	.	.	1
**27132**	0	6.24	1	0.78985	.	.	1
**27133**	0	6.24	1	0.78985	.	.	1
**27134**	0	6.24	1	0.78985	.	.	1
**27135**	0	6.24	1	0.78985	.	.	1
**27136**	0	6.24	1	0.78985	.	.	1
**27137**	0	6.24	1	0.78985	.	.	1
**27138**	0	6.24	1	0.78985	.	.	1
**27139**	0	6.24	1	0.78985	.	.	1
**27140**	0	6.24	1	0.78985	.	.	1
**27141**	0	6.24	1	0.78985	.	.	1
**27142**	0	6.24	1	0.78985	.	.	1
**27143**	0	6.24	1	0.78985	.	.	1
**27144**	0	6.24	1	0.78985	.	.	1
**27145**	0	6.24	1	0.78985	.	.	1
**27146**	0	6.24	1	0.78985	.	.	1
**27147**	0	6.24	1	0.78985	.	.	1
**27148**	0	6.24	1	0.78985	.	.	1
**27149**	0	6.25	0	0.78973	0.78515	0.79423	1
**27150**	0	6.25	1	0.78973	.	.	1
**27151**	0	6.25	1	0.78973	.	.	1
**27152**	0	6.25	1	0.78973	.	.	1
**27153**	0	6.25	1	0.78973	.	.	1
**27154**	0	6.25	1	0.78973	.	.	1
**27155**	0	6.25	1	0.78973	.	.	1
**27156**	0	6.25	1	0.78973	.	.	1
**27157**	0	6.25	1	0.78973	.	.	1
**27158**	0	6.25	1	0.78973	.	.	1
**27159**	0	6.25	1	0.78973	.	.	1
**27160**	0	6.25	1	0.78973	.	.	1

Obs	Sub_group	fuptimey	_CENSOR_	SURVIVAL	SDF_LCL	SDF_UCL	STRATUM
27161	0	6.25	1	0.78973	.	.	1
27162	0	6.25	1	0.78973	.	.	1
27163	0	6.25	1	0.78973	.	.	1
27164	0	6.25	1	0.78973	.	.	1
27165	0	6.25	1	0.78973	.	.	1
27166	0	6.25	1	0.78973	.	.	1
27167	0	6.25	1	0.78973	.	.	1
27168	0	6.25	1	0.78973	.	.	1
27169	0	6.25	1	0.78973	.	.	1
27170	0	6.25	1	0.78973	.	.	1
27171	0	6.25	1	0.78973	.	.	1
27172	0	6.25	1	0.78973	.	.	1
27173	0	6.25	1	0.78973	.	.	1
27174	0	6.25	1	0.78973	.	.	1
27175	0	6.25	1	0.78973	.	.	1
27176	0	6.25	1	0.78973	.	.	1
27177	0	6.25	1	0.78973	.	.	1
27178	0	6.25	1	0.78973	.	.	1
27179	0	6.25	1	0.78973	.	.	1
27180	0	6.26	0	0.78960	0.78502	0.79410	1
27181	0	6.26	1	0.78960	.	.	1
27182	0	6.26	1	0.78960	.	.	1
27183	0	6.26	1	0.78960	.	.	1
27184	0	6.26	1	0.78960	.	.	1
27185	0	6.26	1	0.78960	.	.	1
27186	0	6.26	1	0.78960	.	.	1
27187	0	6.26	1	0.78960	.	.	1
27188	0	6.26	1	0.78960	.	.	1
27189	0	6.26	1	0.78960	.	.	1
27190	0	6.26	1	0.78960	.	.	1
27191	0	6.26	1	0.78960	.	.	1
27192	0	6.26	1	0.78960	.	.	1
27193	0	6.26	1	0.78960	.	.	1
27194	0	6.26	1	0.78960	.	.	1
27195	0	6.26	1	0.78960	.	.	1

Obs	Sub_group	fuptimey	_CENSOR_	SURVIVAL	SDF_LCL	SDF_UCL	STRATUM
27196	0	6.26	1	0.78960	.	.	1
27197	0	6.26	1	0.78960	.	.	1
27198	0	6.26	1	0.78960	.	.	1
27199	0	6.26	1	0.78960	.	.	1
27200	0	6.26	1	0.78960	.	.	1
27201	0	6.26	1	0.78960	.	.	1
27202	0	6.26	1	0.78960	.	.	1
27203	0	6.26	1	0.78960	.	.	1
27204	0	6.26	1	0.78960	.	.	1
27205	0	6.26	1	0.78960	.	.	1
27206	0	6.26	1	0.78960	.	.	1
27207	0	6.26	1	0.78960	.	.	1
27208	0	6.26	1	0.78960	.	.	1
27209	0	6.26	1	0.78960	.	.	1
27210	0	6.26	1	0.78960	.	.	1
27211	0	6.26	1	0.78960	.	.	1
27212	0	6.26	1	0.78960	.	.	1
27213	0	6.26	1	0.78960	.	.	1
27214	0	6.26	1	0.78960	.	.	1
27215	0	6.26	1	0.78960	.	.	1
27216	0	6.26	1	0.78960	.	.	1
27217	0	6.26	1	0.78960	.	.	1
27218	0	6.26	1	0.78960	.	.	1
27219	0	6.26	1	0.78960	.	.	1
27220	0	6.26	1	0.78960	.	.	1
27221	0	6.26	1	0.78960	.	.	1
27222	0	6.26	1	0.78960	.	.	1
27223	0	6.26	1	0.78960	.	.	1
27224	0	6.27	0	0.78954	0.78496	0.79404	1
27225	0	6.27	1	0.78954	.	.	1
27226	0	6.27	1	0.78954	.	.	1
27227	0	6.27	1	0.78954	.	.	1
27228	0	6.27	1	0.78954	.	.	1
27229	0	6.27	1	0.78954	.	.	1
27230	0	6.27	1	0.78954	.	.	1

Obs	Sub_group	fuptimey	_CENSOR_	SURVIVAL	SDF_LCL	SDF_UCL	STRATUM
**27231**	0	6.27	1	0.78954	.	.	1
**27232**	0	6.27	1	0.78954	.	.	1
**27233**	0	6.27	1	0.78954	.	.	1
**27234**	0	6.27	1	0.78954	.	.	1
**27235**	0	6.27	1	0.78954	.	.	1
**27236**	0	6.27	1	0.78954	.	.	1
**27237**	0	6.27	1	0.78954	.	.	1
**27238**	0	6.27	1	0.78954	.	.	1
**27239**	0	6.27	1	0.78954	.	.	1
**27240**	0	6.27	1	0.78954	.	.	1
**27241**	0	6.27	1	0.78954	.	.	1
**27242**	0	6.27	1	0.78954	.	.	1
**27243**	0	6.27	1	0.78954	.	.	1
**27244**	0	6.27	1	0.78954	.	.	1
**27245**	0	6.27	1	0.78954	.	.	1
**27246**	0	6.27	1	0.78954	.	.	1
**27247**	0	6.27	1	0.78954	.	.	1
**27248**	0	6.27	1	0.78954	.	.	1
**27249**	0	6.27	1	0.78954	.	.	1
**27250**	0	6.27	1	0.78954	.	.	1
**27251**	0	6.27	1	0.78954	.	.	1
**27252**	0	6.27	1	0.78954	.	.	1
**27253**	0	6.27	1	0.78954	.	.	1
**27254**	0	6.27	1	0.78954	.	.	1
**27255**	0	6.27	1	0.78954	.	.	1
**27256**	0	6.27	1	0.78954	.	.	1
**27257**	0	6.27	1	0.78954	.	.	1
**27258**	0	6.27	1	0.78954	.	.	1
**27259**	0	6.27	1	0.78954	.	.	1
**27260**	0	6.27	1	0.78954	.	.	1
**27261**	0	6.27	1	0.78954	.	.	1
**27262**	0	6.27	1	0.78954	.	.	1
**27263**	0	6.28	0	0.78923	0.78463	0.79373	1
**27264**	0	6.28	1	0.78923	.	.	1
**27265**	0	6.28	1	0.78923	.	.	1

Obs	Sub_group	fuptimey	_CENSOR_	SURVIVAL	SDF_LCL	SDF_UCL	STRATUM
27266	0	6.28	1	0.78923	.	.	1
27267	0	6.28	1	0.78923	.	.	1
27268	0	6.28	1	0.78923	.	.	1
27269	0	6.28	1	0.78923	.	.	1
27270	0	6.28	1	0.78923	.	.	1
27271	0	6.28	1	0.78923	.	.	1
27272	0	6.28	1	0.78923	.	.	1
27273	0	6.28	1	0.78923	.	.	1
27274	0	6.28	1	0.78923	.	.	1
27275	0	6.28	1	0.78923	.	.	1
27276	0	6.28	1	0.78923	.	.	1
27277	0	6.28	1	0.78923	.	.	1
27278	0	6.28	1	0.78923	.	.	1
27279	0	6.28	1	0.78923	.	.	1
27280	0	6.28	1	0.78923	.	.	1
27281	0	6.28	1	0.78923	.	.	1
27282	0	6.28	1	0.78923	.	.	1
27283	0	6.28	1	0.78923	.	.	1
27284	0	6.28	1	0.78923	.	.	1
27285	0	6.28	1	0.78923	.	.	1
27286	0	6.28	1	0.78923	.	.	1
27287	0	6.28	1	0.78923	.	.	1
27288	0	6.28	1	0.78923	.	.	1
27289	0	6.28	1	0.78923	.	.	1
27290	0	6.28	1	0.78923	.	.	1
27291	0	6.28	1	0.78923	.	.	1
27292	0	6.28	1	0.78923	.	.	1
27293	0	6.28	1	0.78923	.	.	1
27294	0	6.28	1	0.78923	.	.	1
27295	0	6.28	1	0.78923	.	.	1
27296	0	6.28	1	0.78923	.	.	1
27297	0	6.28	1	0.78923	.	.	1
27298	0	6.28	1	0.78923	.	.	1
27299	0	6.28	1	0.78923	.	.	1
27300	0	6.28	1	0.78923	.	.	1

Obs	Sub_group	fuptimey	_CENSOR_	SURVIVAL	SDF_LCL	SDF_UCL	STRATUM
27301	0	6.29	0	0.78916	0.78457	0.79367	1
27302	0	6.29	1	0.78916	.	.	1
27303	0	6.29	1	0.78916	.	.	1
27304	0	6.29	1	0.78916	.	.	1
27305	0	6.29	1	0.78916	.	.	1
27306	0	6.29	1	0.78916	.	.	1
27307	0	6.29	1	0.78916	.	.	1
27308	0	6.29	1	0.78916	.	.	1
27309	0	6.29	1	0.78916	.	.	1
27310	0	6.29	1	0.78916	.	.	1
27311	0	6.29	1	0.78916	.	.	1
27312	0	6.29	1	0.78916	.	.	1
27313	0	6.29	1	0.78916	.	.	1
27314	0	6.29	1	0.78916	.	.	1
27315	0	6.29	1	0.78916	.	.	1
27316	0	6.29	1	0.78916	.	.	1
27317	0	6.29	1	0.78916	.	.	1
27318	0	6.29	1	0.78916	.	.	1
27319	0	6.29	1	0.78916	.	.	1
27320	0	6.29	1	0.78916	.	.	1
27321	0	6.29	1	0.78916	.	.	1
27322	0	6.29	1	0.78916	.	.	1
27323	0	6.29	1	0.78916	.	.	1
27324	0	6.29	1	0.78916	.	.	1
27325	0	6.29	1	0.78916	.	.	1
27326	0	6.29	1	0.78916	.	.	1
27327	0	6.29	1	0.78916	.	.	1
27328	0	6.29	1	0.78916	.	.	1
27329	0	6.29	1	0.78916	.	.	1
27330	0	6.29	1	0.78916	.	.	1
27331	0	6.29	1	0.78916	.	.	1
27332	0	6.29	1	0.78916	.	.	1
27333	0	6.30	0	0.78897	0.78437	0.79349	1
27334	0	6.30	1	0.78897	.	.	1
27335	0	6.30	1	0.78897	.	.	1

Obs	Sub_group	fuptimey	_CENSOR_	SURVIVAL	SDF_LCL	SDF_UCL	STRATUM
**27336**	0	6.30	1	0.78897	.	.	1
**27337**	0	6.30	1	0.78897	.	.	1
**27338**	0	6.30	1	0.78897	.	.	1
**27339**	0	6.30	1	0.78897	.	.	1
**27340**	0	6.30	1	0.78897	.	.	1
**27341**	0	6.30	1	0.78897	.	.	1
**27342**	0	6.30	1	0.78897	.	.	1
**27343**	0	6.30	1	0.78897	.	.	1
**27344**	0	6.30	1	0.78897	.	.	1
**27345**	0	6.30	1	0.78897	.	.	1
**27346**	0	6.30	1	0.78897	.	.	1
**27347**	0	6.30	1	0.78897	.	.	1
**27348**	0	6.30	1	0.78897	.	.	1
**27349**	0	6.30	1	0.78897	.	.	1
**27350**	0	6.30	1	0.78897	.	.	1
**27351**	0	6.30	1	0.78897	.	.	1
**27352**	0	6.30	1	0.78897	.	.	1
**27353**	0	6.30	1	0.78897	.	.	1
**27354**	0	6.30	1	0.78897	.	.	1
**27355**	0	6.30	1	0.78897	.	.	1
**27356**	0	6.30	1	0.78897	.	.	1
**27357**	0	6.30	1	0.78897	.	.	1
**27358**	0	6.30	1	0.78897	.	.	1
**27359**	0	6.30	1	0.78897	.	.	1
**27360**	0	6.30	1	0.78897	.	.	1
**27361**	0	6.30	1	0.78897	.	.	1
**27362**	0	6.30	1	0.78897	.	.	1
**27363**	0	6.30	1	0.78897	.	.	1
**27364**	0	6.31	0	0.78885	0.78424	0.79336	1
**27365**	0	6.31	1	0.78885	.	.	1
**27366**	0	6.31	1	0.78885	.	.	1
**27367**	0	6.31	1	0.78885	.	.	1
**27368**	0	6.31	1	0.78885	.	.	1
**27369**	0	6.31	1	0.78885	.	.	1
**27370**	0	6.31	1	0.78885	.	.	1

Obs	Sub_group	fuptimey	_CENSOR_	SURVIVAL	SDF_LCL	SDF_UCL	STRATUM
**27371**	0	6.31	1	0.78885	.	.	1
**27372**	0	6.31	1	0.78885	.	.	1
**27373**	0	6.31	1	0.78885	.	.	1
**27374**	0	6.31	1	0.78885	.	.	1
**27375**	0	6.31	1	0.78885	.	.	1
**27376**	0	6.31	1	0.78885	.	.	1
**27377**	0	6.31	1	0.78885	.	.	1
**27378**	0	6.31	1	0.78885	.	.	1
**27379**	0	6.31	1	0.78885	.	.	1
**27380**	0	6.31	1	0.78885	.	.	1
**27381**	0	6.31	1	0.78885	.	.	1
**27382**	0	6.31	1	0.78885	.	.	1
**27383**	0	6.31	1	0.78885	.	.	1
**27384**	0	6.31	1	0.78885	.	.	1
**27385**	0	6.31	1	0.78885	.	.	1
**27386**	0	6.31	1	0.78885	.	.	1
**27387**	0	6.31	1	0.78885	.	.	1
**27388**	0	6.31	1	0.78885	.	.	1
**27389**	0	6.31	1	0.78885	.	.	1
**27390**	0	6.31	1	0.78885	.	.	1
**27391**	0	6.31	1	0.78885	.	.	1
**27392**	0	6.31	1	0.78885	.	.	1
**27393**	0	6.31	1	0.78885	.	.	1
**27394**	0	6.31	1	0.78885	.	.	1
**27395**	0	6.31	1	0.78885	.	.	1
**27396**	0	6.31	1	0.78885	.	.	1
**27397**	0	6.31	1	0.78885	.	.	1
**27398**	0	6.31	1	0.78885	.	.	1
**27399**	0	6.31	1	0.78885	.	.	1
**27400**	0	6.31	1	0.78885	.	.	1
**27401**	0	6.31	1	0.78885	.	.	1
**27402**	0	6.31	1	0.78885	.	.	1
**27403**	0	6.32	0	0.78872	0.78411	0.79324	1
**27404**	0	6.32	1	0.78872	.	.	1
**27405**	0	6.32	1	0.78872	.	.	1

Obs	Sub_group	fuptimey	_CENSOR_	SURVIVAL	SDF_LCL	SDF_UCL	STRATUM
27406	0	6.32	1	0.78872	.	.	1
27407	0	6.32	1	0.78872	.	.	1
27408	0	6.32	1	0.78872	.	.	1
27409	0	6.32	1	0.78872	.	.	1
27410	0	6.32	1	0.78872	.	.	1
27411	0	6.32	1	0.78872	.	.	1
27412	0	6.32	1	0.78872	.	.	1
27413	0	6.32	1	0.78872	.	.	1
27414	0	6.32	1	0.78872	.	.	1
27415	0	6.32	1	0.78872	.	.	1
27416	0	6.32	1	0.78872	.	.	1
27417	0	6.32	1	0.78872	.	.	1
27418	0	6.32	1	0.78872	.	.	1
27419	0	6.32	1	0.78872	.	.	1
27420	0	6.32	1	0.78872	.	.	1
27421	0	6.32	1	0.78872	.	.	1
27422	0	6.32	1	0.78872	.	.	1
27423	0	6.32	1	0.78872	.	.	1
27424	0	6.32	1	0.78872	.	.	1
27425	0	6.32	1	0.78872	.	.	1
27426	0	6.32	1	0.78872	.	.	1
27427	0	6.32	1	0.78872	.	.	1
27428	0	6.32	1	0.78872	.	.	1
27429	0	6.32	1	0.78872	.	.	1
27430	0	6.33	0	0.78859	0.78398	0.79311	1
27431	0	6.33	1	0.78859	.	.	1
27432	0	6.33	1	0.78859	.	.	1
27433	0	6.33	1	0.78859	.	.	1
27434	0	6.33	1	0.78859	.	.	1
27435	0	6.33	1	0.78859	.	.	1
27436	0	6.33	1	0.78859	.	.	1
27437	0	6.33	1	0.78859	.	.	1
27438	0	6.33	1	0.78859	.	.	1
27439	0	6.33	1	0.78859	.	.	1
27440	0	6.33	1	0.78859	.	.	1

Obs	Sub_group	fuptimey	_CENSOR_	SURVIVAL	SDF_LCL	SDF_UCL	STRATUM
**27441**	0	6.33	1	0.78859	.	.	1
**27442**	0	6.33	1	0.78859	.	.	1
**27443**	0	6.33	1	0.78859	.	.	1
**27444**	0	6.33	1	0.78859	.	.	1
**27445**	0	6.33	1	0.78859	.	.	1
**27446**	0	6.33	1	0.78859	.	.	1
**27447**	0	6.33	1	0.78859	.	.	1
**27448**	0	6.33	1	0.78859	.	.	1
**27449**	0	6.33	1	0.78859	.	.	1
**27450**	0	6.33	1	0.78859	.	.	1
**27451**	0	6.33	1	0.78859	.	.	1
**27452**	0	6.33	1	0.78859	.	.	1
**27453**	0	6.33	1	0.78859	.	.	1
**27454**	0	6.33	1	0.78859	.	.	1
**27455**	0	6.33	1	0.78859	.	.	1
**27456**	0	6.33	1	0.78859	.	.	1
**27457**	0	6.33	1	0.78859	.	.	1
**27458**	0	6.33	1	0.78859	.	.	1
**27459**	0	6.33	1	0.78859	.	.	1
**27460**	0	6.33	1	0.78859	.	.	1
**27461**	0	6.33	1	0.78859	.	.	1
**27462**	0	6.33	1	0.78859	.	.	1
**27463**	0	6.33	1	0.78859	.	.	1
**27464**	0	6.33	1	0.78859	.	.	1
**27465**	0	6.33	1	0.78859	.	.	1
**27466**	0	6.33	1	0.78859	.	.	1
**27467**	0	6.33	1	0.78859	.	.	1
**27468**	0	6.34	0	0.78846	0.78385	0.79299	1
**27469**	0	6.34	1	0.78846	.	.	1
**27470**	0	6.34	1	0.78846	.	.	1
**27471**	0	6.34	1	0.78846	.	.	1
**27472**	0	6.34	1	0.78846	.	.	1
**27473**	0	6.34	1	0.78846	.	.	1
**27474**	0	6.34	1	0.78846	.	.	1
**27475**	0	6.34	1	0.78846	.	.	1

Obs	Sub_group	fuptimey	_CENSOR_	SURVIVAL	SDF_LCL	SDF_UCL	STRATUM
27476	0	6.34	1	0.78846	.	.	1
27477	0	6.34	1	0.78846	.	.	1
27478	0	6.34	1	0.78846	.	.	1
27479	0	6.34	1	0.78846	.	.	1
27480	0	6.34	1	0.78846	.	.	1
27481	0	6.34	1	0.78846	.	.	1
27482	0	6.34	1	0.78846	.	.	1
27483	0	6.34	1	0.78846	.	.	1
27484	0	6.34	1	0.78846	.	.	1
27485	0	6.34	1	0.78846	.	.	1
27486	0	6.34	1	0.78846	.	.	1
27487	0	6.34	1	0.78846	.	.	1
27488	0	6.34	1	0.78846	.	.	1
27489	0	6.34	1	0.78846	.	.	1
27490	0	6.34	1	0.78846	.	.	1
27491	0	6.34	1	0.78846	.	.	1
27492	0	6.34	1	0.78846	.	.	1
27493	0	6.34	1	0.78846	.	.	1
27494	0	6.34	1	0.78846	.	.	1
27495	0	6.34	1	0.78846	.	.	1
27496	0	6.34	1	0.78846	.	.	1
27497	0	6.34	1	0.78846	.	.	1
27498	0	6.34	1	0.78846	.	.	1
27499	0	6.34	1	0.78846	.	.	1
27500	0	6.34	1	0.78846	.	.	1
27501	0	6.34	1	0.78846	.	.	1
27502	0	6.34	1	0.78846	.	.	1
27503	0	6.34	1	0.78846	.	.	1
27504	0	6.34	1	0.78846	.	.	1
27505	0	6.34	1	0.78846	.	.	1
27506	0	6.34	1	0.78846	.	.	1
27507	0	6.35	0	0.78827	0.78366	0.79280	1
27508	0	6.35	1	0.78827	.	.	1
27509	0	6.35	1	0.78827	.	.	1
27510	0	6.35	1	0.78827	.	.	1

Obs	Sub_group	fuptimey	_CENSOR_	SURVIVAL	SDF_LCL	SDF_UCL	STRATUM
27511	0	6.35	1	0.78827	.	.	1
27512	0	6.35	1	0.78827	.	.	1
27513	0	6.35	1	0.78827	.	.	1
27514	0	6.35	1	0.78827	.	.	1
27515	0	6.35	1	0.78827	.	.	1
27516	0	6.35	1	0.78827	.	.	1
27517	0	6.35	1	0.78827	.	.	1
27518	0	6.35	1	0.78827	.	.	1
27519	0	6.35	1	0.78827	.	.	1
27520	0	6.35	1	0.78827	.	.	1
27521	0	6.35	1	0.78827	.	.	1
27522	0	6.35	1	0.78827	.	.	1
27523	0	6.35	1	0.78827	.	.	1
27524	0	6.35	1	0.78827	.	.	1
27525	0	6.35	1	0.78827	.	.	1
27526	0	6.35	1	0.78827	.	.	1
27527	0	6.35	1	0.78827	.	.	1
27528	0	6.35	1	0.78827	.	.	1
27529	0	6.35	1	0.78827	.	.	1
27530	0	6.35	1	0.78827	.	.	1
27531	0	6.35	1	0.78827	.	.	1
27532	0	6.35	1	0.78827	.	.	1
27533	0	6.35	1	0.78827	.	.	1
27534	0	6.35	1	0.78827	.	.	1
27535	0	6.35	1	0.78827	.	.	1
27536	0	6.35	1	0.78827	.	.	1
27537	0	6.35	1	0.78827	.	.	1
27538	0	6.35	1	0.78827	.	.	1
27539	0	6.35	1	0.78827	.	.	1
27540	0	6.35	1	0.78827	.	.	1
27541	0	6.35	1	0.78827	.	.	1
27542	0	6.35	1	0.78827	.	.	1
27543	0	6.35	1	0.78827	.	.	1
27544	0	6.35	1	0.78827	.	.	1
27545	0	6.35	1	0.78827	.	.	1

Obs	Sub_group	fuptimey	_CENSOR_	SURVIVAL	SDF_LCL	SDF_UCL	STRATUM
27546	0	6.35	1	0.78827	.	.	1
27547	0	6.35	1	0.78827	.	.	1
27548	0	6.35	1	0.78827	.	.	1
27549	0	6.35	1	0.78827	.	.	1
27550	0	6.35	1	0.78827	.	.	1
27551	0	6.36	0	0.78820	0.78359	0.79273	1
27552	0	6.36	1	0.78820	.	.	1
27553	0	6.36	1	0.78820	.	.	1
27554	0	6.36	1	0.78820	.	.	1
27555	0	6.36	1	0.78820	.	.	1
27556	0	6.36	1	0.78820	.	.	1
27557	0	6.36	1	0.78820	.	.	1
27558	0	6.36	1	0.78820	.	.	1
27559	0	6.36	1	0.78820	.	.	1
27560	0	6.36	1	0.78820	.	.	1
27561	0	6.36	1	0.78820	.	.	1
27562	0	6.36	1	0.78820	.	.	1
27563	0	6.36	1	0.78820	.	.	1
27564	0	6.36	1	0.78820	.	.	1
27565	0	6.36	1	0.78820	.	.	1
27566	0	6.36	1	0.78820	.	.	1
27567	0	6.36	1	0.78820	.	.	1
27568	0	6.36	1	0.78820	.	.	1
27569	0	6.36	1	0.78820	.	.	1
27570	0	6.36	1	0.78820	.	.	1
27571	0	6.36	1	0.78820	.	.	1
27572	0	6.36	1	0.78820	.	.	1
27573	0	6.36	1	0.78820	.	.	1
27574	0	6.36	1	0.78820	.	.	1
27575	0	6.36	1	0.78820	.	.	1
27576	0	6.36	1	0.78820	.	.	1
27577	0	6.36	1	0.78820	.	.	1
27578	0	6.36	1	0.78820	.	.	1
27579	0	6.37	0	0.78808	0.78346	0.79261	1
27580	0	6.37	1	0.78808	.	.	1

Obs	Sub_group	fuptimey	_CENSOR_	SURVIVAL	SDF_LCL	SDF_UCL	STRATUM
27581	0	6.37	1	0.78808	.	.	1
27582	0	6.37	1	0.78808	.	.	1
27583	0	6.37	1	0.78808	.	.	1
27584	0	6.37	1	0.78808	.	.	1
27585	0	6.37	1	0.78808	.	.	1
27586	0	6.37	1	0.78808	.	.	1
27587	0	6.37	1	0.78808	.	.	1
27588	0	6.37	1	0.78808	.	.	1
27589	0	6.37	1	0.78808	.	.	1
27590	0	6.37	1	0.78808	.	.	1
27591	0	6.37	1	0.78808	.	.	1
27592	0	6.37	1	0.78808	.	.	1
27593	0	6.37	1	0.78808	.	.	1
27594	0	6.37	1	0.78808	.	.	1
27595	0	6.37	1	0.78808	.	.	1
27596	0	6.37	1	0.78808	.	.	1
27597	0	6.37	1	0.78808	.	.	1
27598	0	6.37	1	0.78808	.	.	1
27599	0	6.37	1	0.78808	.	.	1
27600	0	6.37	1	0.78808	.	.	1
27601	0	6.37	1	0.78808	.	.	1
27602	0	6.37	1	0.78808	.	.	1
27603	0	6.37	1	0.78808	.	.	1
27604	0	6.37	1	0.78808	.	.	1
27605	0	6.37	1	0.78808	.	.	1
27606	0	6.37	1	0.78808	.	.	1
27607	0	6.37	1	0.78808	.	.	1
27608	0	6.37	1	0.78808	.	.	1
27609	0	6.37	1	0.78808	.	.	1
27610	0	6.37	1	0.78808	.	.	1
27611	0	6.37	1	0.78808	.	.	1
27612	0	6.37	1	0.78808	.	.	1
27613	0	6.37	1	0.78808	.	.	1
27614	0	6.37	1	0.78808	.	.	1
27615	0	6.37	1	0.78808	.	.	1

Obs	Sub_group	fuptimey	_CENSOR_	SURVIVAL	SDF_LCL	SDF_UCL	STRATUM
27616	0	6.37	1	0.78808	.	.	1
27617	0	6.37	1	0.78808	.	.	1
27618	0	6.37	1	0.78808	.	.	1
27619	0	6.37	1	0.78808	.	.	1
27620	0	6.37	1	0.78808	.	.	1
27621	0	6.37	1	0.78808	.	.	1
27622	0	6.37	1	0.78808	.	.	1
27623	0	6.37	1	0.78808	.	.	1
27624	0	6.37	1	0.78808	.	.	1
27625	0	6.38	0	0.78775	0.78313	0.79229	1
27626	0	6.38	1	0.78775	.	.	1
27627	0	6.38	1	0.78775	.	.	1
27628	0	6.38	1	0.78775	.	.	1
27629	0	6.38	1	0.78775	.	.	1
27630	0	6.38	1	0.78775	.	.	1
27631	0	6.38	1	0.78775	.	.	1
27632	0	6.38	1	0.78775	.	.	1
27633	0	6.38	1	0.78775	.	.	1
27634	0	6.38	1	0.78775	.	.	1
27635	0	6.38	1	0.78775	.	.	1
27636	0	6.38	1	0.78775	.	.	1
27637	0	6.38	1	0.78775	.	.	1
27638	0	6.38	1	0.78775	.	.	1
27639	0	6.38	1	0.78775	.	.	1
27640	0	6.38	1	0.78775	.	.	1
27641	0	6.38	1	0.78775	.	.	1
27642	0	6.38	1	0.78775	.	.	1
27643	0	6.38	1	0.78775	.	.	1
27644	0	6.38	1	0.78775	.	.	1
27645	0	6.38	1	0.78775	.	.	1
27646	0	6.38	1	0.78775	.	.	1
27647	0	6.38	1	0.78775	.	.	1
27648	0	6.38	1	0.78775	.	.	1
27649	0	6.38	1	0.78775	.	.	1
27650	0	6.38	1	0.78775	.	.	1

Obs	Sub_group	fuptimey	_CENSOR_	SURVIVAL	SDF_LCL	SDF_UCL	STRATUM
27651	0	6.38	1	0.78775	.	.	1
27652	0	6.38	1	0.78775	.	.	1
27653	0	6.38	1	0.78775	.	.	1
27654	0	6.38	1	0.78775	.	.	1
27655	0	6.39	0	0.78762	0.78299	0.79216	1
27656	0	6.39	1	0.78762	.	.	1
27657	0	6.39	1	0.78762	.	.	1
27658	0	6.39	1	0.78762	.	.	1
27659	0	6.39	1	0.78762	.	.	1
27660	0	6.39	1	0.78762	.	.	1
27661	0	6.39	1	0.78762	.	.	1
27662	0	6.39	1	0.78762	.	.	1
27663	0	6.39	1	0.78762	.	.	1
27664	0	6.39	1	0.78762	.	.	1
27665	0	6.39	1	0.78762	.	.	1
27666	0	6.39	1	0.78762	.	.	1
27667	0	6.39	1	0.78762	.	.	1
27668	0	6.39	1	0.78762	.	.	1
27669	0	6.39	1	0.78762	.	.	1
27670	0	6.39	1	0.78762	.	.	1
27671	0	6.39	1	0.78762	.	.	1
27672	0	6.39	1	0.78762	.	.	1
27673	0	6.39	1	0.78762	.	.	1
27674	0	6.39	1	0.78762	.	.	1
27675	0	6.39	1	0.78762	.	.	1
27676	0	6.39	1	0.78762	.	.	1
27677	0	6.39	1	0.78762	.	.	1
27678	0	6.39	1	0.78762	.	.	1
27679	0	6.39	1	0.78762	.	.	1
27680	0	6.39	1	0.78762	.	.	1
27681	0	6.39	1	0.78762	.	.	1
27682	0	6.39	1	0.78762	.	.	1
27683	0	6.39	1	0.78762	.	.	1
27684	0	6.39	1	0.78762	.	.	1
27685	0	6.39	1	0.78762	.	.	1

Obs	Sub_group	fuptimey	_CENSOR_	SURVIVAL	SDF_LCL	SDF_UCL	STRATUM
27686	0	6.39	1	0.78762	.	.	1
27687	0	6.39	1	0.78762	.	.	1
27688	0	6.39	1	0.78762	.	.	1
27689	0	6.39	1	0.78762	.	.	1
27690	0	6.39	1	0.78762	.	.	1
27691	0	6.39	1	0.78762	.	.	1
27692	0	6.39	1	0.78762	.	.	1
27693	0	6.39	1	0.78762	.	.	1
27694	0	6.39	1	0.78762	.	.	1
27695	0	6.39	1	0.78762	.	.	1
27696	0	6.39	1	0.78762	.	.	1
27697	0	6.39	1	0.78762	.	.	1
27698	0	6.39	1	0.78762	.	.	1
27699	0	6.39	1	0.78762	.	.	1
27700	0	6.40	0	0.78749	0.78286	0.79203	1
27701	0	6.40	1	0.78749	.	.	1
27702	0	6.40	1	0.78749	.	.	1
27703	0	6.40	1	0.78749	.	.	1
27704	0	6.40	1	0.78749	.	.	1
27705	0	6.40	1	0.78749	.	.	1
27706	0	6.40	1	0.78749	.	.	1
27707	0	6.40	1	0.78749	.	.	1
27708	0	6.40	1	0.78749	.	.	1
27709	0	6.40	1	0.78749	.	.	1
27710	0	6.40	1	0.78749	.	.	1
27711	0	6.40	1	0.78749	.	.	1
27712	0	6.40	1	0.78749	.	.	1
27713	0	6.40	1	0.78749	.	.	1
27714	0	6.40	1	0.78749	.	.	1
27715	0	6.40	1	0.78749	.	.	1
27716	0	6.40	1	0.78749	.	.	1
27717	0	6.40	1	0.78749	.	.	1
27718	0	6.40	1	0.78749	.	.	1
27719	0	6.40	1	0.78749	.	.	1
27720	0	6.40	1	0.78749	.	.	1

Obs	Sub_group	fuptimey	_CENSOR_	SURVIVAL	SDF_LCL	SDF_UCL	STRATUM
27721	0	6.40	1	0.78749	.	.	1
27722	0	6.40	1	0.78749	.	.	1
27723	0	6.40	1	0.78749	.	.	1
27724	0	6.40	1	0.78749	.	.	1
27725	0	6.40	1	0.78749	.	.	1
27726	0	6.40	1	0.78749	.	.	1
27727	0	6.40	1	0.78749	.	.	1
27728	0	6.40	1	0.78749	.	.	1
27729	0	6.40	1	0.78749	.	.	1
27730	0	6.41	0	0.78723	0.78259	0.79178	1
27731	0	6.41	1	0.78723	.	.	1
27732	0	6.41	1	0.78723	.	.	1
27733	0	6.41	1	0.78723	.	.	1
27734	0	6.41	1	0.78723	.	.	1
27735	0	6.41	1	0.78723	.	.	1
27736	0	6.41	1	0.78723	.	.	1
27737	0	6.41	1	0.78723	.	.	1
27738	0	6.41	1	0.78723	.	.	1
27739	0	6.41	1	0.78723	.	.	1
27740	0	6.41	1	0.78723	.	.	1
27741	0	6.41	1	0.78723	.	.	1
27742	0	6.41	1	0.78723	.	.	1
27743	0	6.41	1	0.78723	.	.	1
27744	0	6.41	1	0.78723	.	.	1
27745	0	6.41	1	0.78723	.	.	1
27746	0	6.41	1	0.78723	.	.	1
27747	0	6.41	1	0.78723	.	.	1
27748	0	6.41	1	0.78723	.	.	1
27749	0	6.41	1	0.78723	.	.	1
27750	0	6.41	1	0.78723	.	.	1
27751	0	6.41	1	0.78723	.	.	1
27752	0	6.41	1	0.78723	.	.	1
27753	0	6.41	1	0.78723	.	.	1
27754	0	6.41	1	0.78723	.	.	1
27755	0	6.41	1	0.78723	.	.	1

Obs	Sub_group	fuptimey	_CENSOR_	SURVIVAL	SDF_LCL	SDF_UCL	STRATUM
27756	0	6.41	1	0.78723	.	.	1
27757	0	6.41	1	0.78723	.	.	1
27758	0	6.41	1	0.78723	.	.	1
27759	0	6.41	1	0.78723	.	.	1
27760	0	6.41	1	0.78723	.	.	1
27761	0	6.41	1	0.78723	.	.	1
27762	0	6.41	1	0.78723	.	.	1
27763	0	6.41	1	0.78723	.	.	1
27764	0	6.41	1	0.78723	.	.	1
27765	0	6.41	1	0.78723	.	.	1
27766	0	6.41	1	0.78723	.	.	1
27767	0	6.41	1	0.78723	.	.	1
27768	0	6.41	1	0.78723	.	.	1
27769	0	6.41	1	0.78723	.	.	1
27770	0	6.41	1	0.78723	.	.	1
27771	0	6.41	1	0.78723	.	.	1
27772	0	6.41	1	0.78723	.	.	1
27773	0	6.41	1	0.78723	.	.	1
27774	0	6.42	1	0.78723	.	.	1
27775	0	6.42	1	0.78723	.	.	1
27776	0	6.42	1	0.78723	.	.	1
27777	0	6.42	1	0.78723	.	.	1
27778	0	6.42	1	0.78723	.	.	1
27779	0	6.42	1	0.78723	.	.	1
27780	0	6.42	1	0.78723	.	.	1
27781	0	6.42	1	0.78723	.	.	1
27782	0	6.42	1	0.78723	.	.	1
27783	0	6.42	1	0.78723	.	.	1
27784	0	6.42	1	0.78723	.	.	1
27785	0	6.42	1	0.78723	.	.	1
27786	0	6.42	1	0.78723	.	.	1
27787	0	6.42	1	0.78723	.	.	1
27788	0	6.42	1	0.78723	.	.	1
27789	0	6.42	1	0.78723	.	.	1
27790	0	6.42	1	0.78723	.	.	1

Obs	Sub_group	fuptimey	_CENSOR_	SURVIVAL	SDF_LCL	SDF_UCL	STRATUM
27791	0	6.42	1	0.78723	.	.	1
27792	0	6.42	1	0.78723	.	.	1
27793	0	6.42	1	0.78723	.	.	1
27794	0	6.42	1	0.78723	.	.	1
27795	0	6.42	1	0.78723	.	.	1
27796	0	6.42	1	0.78723	.	.	1
27797	0	6.42	1	0.78723	.	.	1
27798	0	6.42	1	0.78723	.	.	1
27799	0	6.42	1	0.78723	.	.	1
27800	0	6.42	1	0.78723	.	.	1
27801	0	6.42	1	0.78723	.	.	1
27802	0	6.42	1	0.78723	.	.	1
27803	0	6.42	1	0.78723	.	.	1
27804	0	6.42	1	0.78723	.	.	1
27805	0	6.43	0	0.78703	0.78239	0.79159	1
27806	0	6.43	1	0.78703	.	.	1
27807	0	6.43	1	0.78703	.	.	1
27808	0	6.43	1	0.78703	.	.	1
27809	0	6.43	1	0.78703	.	.	1
27810	0	6.43	1	0.78703	.	.	1
27811	0	6.43	1	0.78703	.	.	1
27812	0	6.43	1	0.78703	.	.	1
27813	0	6.43	1	0.78703	.	.	1
27814	0	6.43	1	0.78703	.	.	1
27815	0	6.43	1	0.78703	.	.	1
27816	0	6.43	1	0.78703	.	.	1
27817	0	6.43	1	0.78703	.	.	1
27818	0	6.43	1	0.78703	.	.	1
27819	0	6.43	1	0.78703	.	.	1
27820	0	6.43	1	0.78703	.	.	1
27821	0	6.43	1	0.78703	.	.	1
27822	0	6.43	1	0.78703	.	.	1
27823	0	6.43	1	0.78703	.	.	1
27824	0	6.43	1	0.78703	.	.	1
27825	0	6.43	1	0.78703	.	.	1

Obs	Sub_group	fuptimey	_CENSOR_	SURVIVAL	SDF_LCL	SDF_UCL	STRATUM
27826	0	6.43	1	0.78703	.	.	1
27827	0	6.43	1	0.78703	.	.	1
27828	0	6.43	1	0.78703	.	.	1
27829	0	6.43	1	0.78703	.	.	1
27830	0	6.43	1	0.78703	.	.	1
27831	0	6.43	1	0.78703	.	.	1
27832	0	6.43	1	0.78703	.	.	1
27833	0	6.43	1	0.78703	.	.	1
27834	0	6.43	1	0.78703	.	.	1
27835	0	6.43	1	0.78703	.	.	1
27836	0	6.43	1	0.78703	.	.	1
27837	0	6.43	1	0.78703	.	.	1
27838	0	6.43	1	0.78703	.	.	1
27839	0	6.43	1	0.78703	.	.	1
27840	0	6.43	1	0.78703	.	.	1
27841	0	6.43	1	0.78703	.	.	1
27842	0	6.43	1	0.78703	.	.	1
27843	0	6.43	1	0.78703	.	.	1
27844	0	6.44	0	0.78683	0.78219	0.79139	1
27845	0	6.44	1	0.78683	.	.	1
27846	0	6.44	1	0.78683	.	.	1
27847	0	6.44	1	0.78683	.	.	1
27848	0	6.44	1	0.78683	.	.	1
27849	0	6.44	1	0.78683	.	.	1
27850	0	6.44	1	0.78683	.	.	1
27851	0	6.44	1	0.78683	.	.	1
27852	0	6.44	1	0.78683	.	.	1
27853	0	6.44	1	0.78683	.	.	1
27854	0	6.44	1	0.78683	.	.	1
27855	0	6.44	1	0.78683	.	.	1
27856	0	6.44	1	0.78683	.	.	1
27857	0	6.44	1	0.78683	.	.	1
27858	0	6.44	1	0.78683	.	.	1
27859	0	6.44	1	0.78683	.	.	1
27860	0	6.44	1	0.78683	.	.	1

Obs	Sub_group	fuptimey	_CENSOR_	SURVIVAL	SDF_LCL	SDF_UCL	STRATUM
27861	0	6.44	1	0.78683	.	.	1
27862	0	6.44	1	0.78683	.	.	1
27863	0	6.44	1	0.78683	.	.	1
27864	0	6.44	1	0.78683	.	.	1
27865	0	6.44	1	0.78683	.	.	1
27866	0	6.44	1	0.78683	.	.	1
27867	0	6.44	1	0.78683	.	.	1
27868	0	6.44	1	0.78683	.	.	1
27869	0	6.44	1	0.78683	.	.	1
27870	0	6.44	1	0.78683	.	.	1
27871	0	6.44	1	0.78683	.	.	1
27872	0	6.44	1	0.78683	.	.	1
27873	0	6.44	1	0.78683	.	.	1
27874	0	6.44	1	0.78683	.	.	1
27875	0	6.45	0	0.78670	0.78205	0.79126	1
27876	0	6.45	1	0.78670	.	.	1
27877	0	6.45	1	0.78670	.	.	1
27878	0	6.45	1	0.78670	.	.	1
27879	0	6.45	1	0.78670	.	.	1
27880	0	6.45	1	0.78670	.	.	1
27881	0	6.45	1	0.78670	.	.	1
27882	0	6.45	1	0.78670	.	.	1
27883	0	6.45	1	0.78670	.	.	1
27884	0	6.45	1	0.78670	.	.	1
27885	0	6.45	1	0.78670	.	.	1
27886	0	6.45	1	0.78670	.	.	1
27887	0	6.45	1	0.78670	.	.	1
27888	0	6.45	1	0.78670	.	.	1
27889	0	6.45	1	0.78670	.	.	1
27890	0	6.45	1	0.78670	.	.	1
27891	0	6.45	1	0.78670	.	.	1
27892	0	6.45	1	0.78670	.	.	1
27893	0	6.45	1	0.78670	.	.	1
27894	0	6.45	1	0.78670	.	.	1
27895	0	6.45	1	0.78670	.	.	1

Obs	Sub_group	fuptimey	_CENSOR_	SURVIVAL	SDF_LCL	SDF_UCL	STRATUM
27896	0	6.45	1	0.78670	.	.	1
27897	0	6.45	1	0.78670	.	.	1
27898	0	6.45	1	0.78670	.	.	1
27899	0	6.45	1	0.78670	.	.	1
27900	0	6.45	1	0.78670	.	.	1
27901	0	6.45	1	0.78670	.	.	1
27902	0	6.45	1	0.78670	.	.	1
27903	0	6.45	1	0.78670	.	.	1
27904	0	6.45	1	0.78670	.	.	1
27905	0	6.45	1	0.78670	.	.	1
27906	0	6.45	1	0.78670	.	.	1
27907	0	6.45	1	0.78670	.	.	1
27908	0	6.45	1	0.78670	.	.	1
27909	0	6.45	1	0.78670	.	.	1
27910	0	6.45	1	0.78670	.	.	1
27911	0	6.45	1	0.78670	.	.	1
27912	0	6.45	1	0.78670	.	.	1
27913	0	6.45	1	0.78670	.	.	1
27914	0	6.45	1	0.78670	.	.	1
27915	0	6.46	0	0.78617	0.78151	0.79074	1
27916	0	6.46	1	0.78617	.	.	1
27917	0	6.46	1	0.78617	.	.	1
27918	0	6.46	1	0.78617	.	.	1
27919	0	6.46	1	0.78617	.	.	1
27920	0	6.46	1	0.78617	.	.	1
27921	0	6.46	1	0.78617	.	.	1
27922	0	6.46	1	0.78617	.	.	1
27923	0	6.46	1	0.78617	.	.	1
27924	0	6.46	1	0.78617	.	.	1
27925	0	6.46	1	0.78617	.	.	1
27926	0	6.46	1	0.78617	.	.	1
27927	0	6.46	1	0.78617	.	.	1
27928	0	6.46	1	0.78617	.	.	1
27929	0	6.46	1	0.78617	.	.	1
27930	0	6.46	1	0.78617	.	.	1

Obs	Sub_group	fuptimey	_CENSOR_	SURVIVAL	SDF_LCL	SDF_UCL	STRATUM
27931	0	6.46	1	0.78617	.	.	1
27932	0	6.46	1	0.78617	.	.	1
27933	0	6.46	1	0.78617	.	.	1
27934	0	6.46	1	0.78617	.	.	1
27935	0	6.46	1	0.78617	.	.	1
27936	0	6.46	1	0.78617	.	.	1
27937	0	6.46	1	0.78617	.	.	1
27938	0	6.46	1	0.78617	.	.	1
27939	0	6.46	1	0.78617	.	.	1
27940	0	6.46	1	0.78617	.	.	1
27941	0	6.46	1	0.78617	.	.	1
27942	0	6.46	1	0.78617	.	.	1
27943	0	6.46	1	0.78617	.	.	1
27944	0	6.46	1	0.78617	.	.	1
27945	0	6.46	1	0.78617	.	.	1
27946	0	6.46	1	0.78617	.	.	1
27947	0	6.46	1	0.78617	.	.	1
27948	0	6.47	0	0.78604	0.78137	0.79061	1
27949	0	6.47	1	0.78604	.	.	1
27950	0	6.47	1	0.78604	.	.	1
27951	0	6.47	1	0.78604	.	.	1
27952	0	6.47	1	0.78604	.	.	1
27953	0	6.47	1	0.78604	.	.	1
27954	0	6.47	1	0.78604	.	.	1
27955	0	6.47	1	0.78604	.	.	1
27956	0	6.47	1	0.78604	.	.	1
27957	0	6.47	1	0.78604	.	.	1
27958	0	6.47	1	0.78604	.	.	1
27959	0	6.47	1	0.78604	.	.	1
27960	0	6.47	1	0.78604	.	.	1
27961	0	6.47	1	0.78604	.	.	1
27962	0	6.47	1	0.78604	.	.	1
27963	0	6.47	1	0.78604	.	.	1
27964	0	6.47	1	0.78604	.	.	1
27965	0	6.47	1	0.78604	.	.	1

Obs	Sub_group	fuptimey	_CENSOR_	SURVIVAL	SDF_LCL	SDF_UCL	STRATUM
27966	0	6.47	1	0.78604	.	.	1
27967	0	6.47	1	0.78604	.	.	1
27968	0	6.47	1	0.78604	.	.	1
27969	0	6.47	1	0.78604	.	.	1
27970	0	6.47	1	0.78604	.	.	1
27971	0	6.47	1	0.78604	.	.	1
27972	0	6.47	1	0.78604	.	.	1
27973	0	6.47	1	0.78604	.	.	1
27974	0	6.47	1	0.78604	.	.	1
27975	0	6.47	1	0.78604	.	.	1
27976	0	6.47	1	0.78604	.	.	1
27977	0	6.47	1	0.78604	.	.	1
27978	0	6.47	1	0.78604	.	.	1
27979	0	6.47	1	0.78604	.	.	1
27980	0	6.47	1	0.78604	.	.	1
27981	0	6.47	1	0.78604	.	.	1
27982	0	6.47	1	0.78604	.	.	1
27983	0	6.47	1	0.78604	.	.	1
27984	0	6.47	1	0.78604	.	.	1
27985	0	6.47	1	0.78604	.	.	1
27986	0	6.47	1	0.78604	.	.	1
27987	0	6.47	1	0.78604	.	.	1
27988	0	6.47	1	0.78604	.	.	1
27989	0	6.47	1	0.78604	.	.	1
27990	0	6.48	0	0.78590	0.78124	0.79048	1
27991	0	6.48	1	0.78590	.	.	1
27992	0	6.48	1	0.78590	.	.	1
27993	0	6.48	1	0.78590	.	.	1
27994	0	6.48	1	0.78590	.	.	1
27995	0	6.48	1	0.78590	.	.	1
27996	0	6.48	1	0.78590	.	.	1
27997	0	6.48	1	0.78590	.	.	1
27998	0	6.48	1	0.78590	.	.	1
27999	0	6.48	1	0.78590	.	.	1
28000	0	6.48	1	0.78590	.	.	1

Obs	Sub_group	fuptimey	_CENSOR_	SURVIVAL	SDF_LCL	SDF_UCL	STRATUM
28001	0	6.48	1	0.78590	.	.	1
28002	0	6.48	1	0.78590	.	.	1
28003	0	6.48	1	0.78590	.	.	1
28004	0	6.48	1	0.78590	.	.	1
28005	0	6.48	1	0.78590	.	.	1
28006	0	6.48	1	0.78590	.	.	1
28007	0	6.48	1	0.78590	.	.	1
28008	0	6.48	1	0.78590	.	.	1
28009	0	6.48	1	0.78590	.	.	1
28010	0	6.48	1	0.78590	.	.	1
28011	0	6.48	1	0.78590	.	.	1
28012	0	6.48	1	0.78590	.	.	1
28013	0	6.48	1	0.78590	.	.	1
28014	0	6.48	1	0.78590	.	.	1
28015	0	6.48	1	0.78590	.	.	1
28016	0	6.48	1	0.78590	.	.	1
28017	0	6.48	1	0.78590	.	.	1
28018	0	6.48	1	0.78590	.	.	1
28019	0	6.48	1	0.78590	.	.	1
28020	0	6.48	1	0.78590	.	.	1
28021	0	6.48	1	0.78590	.	.	1
28022	0	6.48	1	0.78590	.	.	1
28023	0	6.48	1	0.78590	.	.	1
28024	0	6.48	1	0.78590	.	.	1
28025	0	6.48	1	0.78590	.	.	1
28026	0	6.48	1	0.78590	.	.	1
28027	0	6.49	0	0.78577	0.78110	0.79035	1
28028	0	6.49	1	0.78577	.	.	1
28029	0	6.49	1	0.78577	.	.	1
28030	0	6.49	1	0.78577	.	.	1
28031	0	6.49	1	0.78577	.	.	1
28032	0	6.49	1	0.78577	.	.	1
28033	0	6.49	1	0.78577	.	.	1
28034	0	6.49	1	0.78577	.	.	1
28035	0	6.49	1	0.78577	.	.	1

Obs	Sub_group	fuptimey	_CENSOR_	SURVIVAL	SDF_LCL	SDF_UCL	STRATUM
28036	0	6.49	1	0.78577	.	.	1
28037	0	6.49	1	0.78577	.	.	1
28038	0	6.49	1	0.78577	.	.	1
28039	0	6.49	1	0.78577	.	.	1
28040	0	6.49	1	0.78577	.	.	1
28041	0	6.49	1	0.78577	.	.	1
28042	0	6.49	1	0.78577	.	.	1
28043	0	6.49	1	0.78577	.	.	1
28044	0	6.49	1	0.78577	.	.	1
28045	0	6.49	1	0.78577	.	.	1
28046	0	6.49	1	0.78577	.	.	1
28047	0	6.49	1	0.78577	.	.	1
28048	0	6.49	1	0.78577	.	.	1
28049	0	6.49	1	0.78577	.	.	1
28050	0	6.49	1	0.78577	.	.	1
28051	0	6.49	1	0.78577	.	.	1
28052	0	6.49	1	0.78577	.	.	1
28053	0	6.49	1	0.78577	.	.	1
28054	0	6.49	1	0.78577	.	.	1
28055	0	6.49	1	0.78577	.	.	1
28056	0	6.49	1	0.78577	.	.	1
28057	0	6.49	1	0.78577	.	.	1
28058	0	6.49	1	0.78577	.	.	1
28059	0	6.49	1	0.78577	.	.	1
28060	0	6.49	1	0.78577	.	.	1
28061	0	6.50	0	0.78570	0.78103	0.79028	1
28062	0	6.50	1	0.78570	.	.	1
28063	0	6.50	1	0.78570	.	.	1
28064	0	6.50	1	0.78570	.	.	1
28065	0	6.50	1	0.78570	.	.	1
28066	0	6.50	1	0.78570	.	.	1
28067	0	6.50	1	0.78570	.	.	1
28068	0	6.50	1	0.78570	.	.	1
28069	0	6.50	1	0.78570	.	.	1
28070	0	6.50	1	0.78570	.	.	1

Obs	Sub_group	fuptimey	_CENSOR_	SURVIVAL	SDF_LCL	SDF_UCL	STRATUM
28071	0	6.50	1	0.78570	.	.	1
28072	0	6.50	1	0.78570	.	.	1
28073	0	6.50	1	0.78570	.	.	1
28074	0	6.50	1	0.78570	.	.	1
28075	0	6.50	1	0.78570	.	.	1
28076	0	6.50	1	0.78570	.	.	1
28077	0	6.50	1	0.78570	.	.	1
28078	0	6.50	1	0.78570	.	.	1
28079	0	6.50	1	0.78570	.	.	1
28080	0	6.50	1	0.78570	.	.	1
28081	0	6.50	1	0.78570	.	.	1
28082	0	6.50	1	0.78570	.	.	1
28083	0	6.50	1	0.78570	.	.	1
28084	0	6.50	1	0.78570	.	.	1
28085	0	6.50	1	0.78570	.	.	1
28086	0	6.50	1	0.78570	.	.	1
28087	0	6.50	1	0.78570	.	.	1
28088	0	6.50	1	0.78570	.	.	1
28089	0	6.50	1	0.78570	.	.	1
28090	0	6.50	1	0.78570	.	.	1
28091	0	6.50	1	0.78570	.	.	1
28092	0	6.51	0	0.78550	0.78082	0.79009	1
28093	0	6.51	1	0.78550	.	.	1
28094	0	6.51	1	0.78550	.	.	1
28095	0	6.51	1	0.78550	.	.	1
28096	0	6.51	1	0.78550	.	.	1
28097	0	6.51	1	0.78550	.	.	1
28098	0	6.51	1	0.78550	.	.	1
28099	0	6.51	1	0.78550	.	.	1
28100	0	6.51	1	0.78550	.	.	1
28101	0	6.51	1	0.78550	.	.	1
28102	0	6.51	1	0.78550	.	.	1
28103	0	6.51	1	0.78550	.	.	1
28104	0	6.51	1	0.78550	.	.	1
28105	0	6.51	1	0.78550	.	.	1

Obs	Sub_group	fuptimey	_CENSOR_	SURVIVAL	SDF_LCL	SDF_UCL	STRATUM
28106	0	6.51	1	0.78550	.	.	1
28107	0	6.51	1	0.78550	.	.	1
28108	0	6.51	1	0.78550	.	.	1
28109	0	6.51	1	0.78550	.	.	1
28110	0	6.51	1	0.78550	.	.	1
28111	0	6.51	1	0.78550	.	.	1
28112	0	6.51	1	0.78550	.	.	1
28113	0	6.51	1	0.78550	.	.	1
28114	0	6.51	1	0.78550	.	.	1
28115	0	6.51	1	0.78550	.	.	1
28116	0	6.51	1	0.78550	.	.	1
28117	0	6.51	1	0.78550	.	.	1
28118	0	6.51	1	0.78550	.	.	1
28119	0	6.51	1	0.78550	.	.	1
28120	0	6.51	1	0.78550	.	.	1
28121	0	6.51	1	0.78550	.	.	1
28122	0	6.51	1	0.78550	.	.	1
28123	0	6.51	1	0.78550	.	.	1
28124	0	6.51	1	0.78550	.	.	1
28125	0	6.51	1	0.78550	.	.	1
28126	0	6.51	1	0.78550	.	.	1
28127	0	6.51	1	0.78550	.	.	1
28128	0	6.51	1	0.78550	.	.	1
28129	0	6.51	1	0.78550	.	.	1
28130	0	6.51	1	0.78550	.	.	1
28131	0	6.51	1	0.78550	.	.	1
28132	0	6.51	1	0.78550	.	.	1
28133	0	6.51	1	0.78550	.	.	1
28134	0	6.52	0	0.78530	0.78062	0.78989	1
28135	0	6.52	1	0.78530	.	.	1
28136	0	6.52	1	0.78530	.	.	1
28137	0	6.52	1	0.78530	.	.	1
28138	0	6.52	1	0.78530	.	.	1
28139	0	6.52	1	0.78530	.	.	1
28140	0	6.52	1	0.78530	.	.	1

Obs	Sub_group	fuptimey	_CENSOR_	SURVIVAL	SDF_LCL	SDF_UCL	STRATUM
28141	0	6.52	1	0.78530	.	.	1
28142	0	6.52	1	0.78530	.	.	1
28143	0	6.52	1	0.78530	.	.	1
28144	0	6.52	1	0.78530	.	.	1
28145	0	6.52	1	0.78530	.	.	1
28146	0	6.52	1	0.78530	.	.	1
28147	0	6.52	1	0.78530	.	.	1
28148	0	6.52	1	0.78530	.	.	1
28149	0	6.52	1	0.78530	.	.	1
28150	0	6.52	1	0.78530	.	.	1
28151	0	6.52	1	0.78530	.	.	1
28152	0	6.52	1	0.78530	.	.	1
28153	0	6.52	1	0.78530	.	.	1
28154	0	6.52	1	0.78530	.	.	1
28155	0	6.52	1	0.78530	.	.	1
28156	0	6.52	1	0.78530	.	.	1
28157	0	6.52	1	0.78530	.	.	1
28158	0	6.52	1	0.78530	.	.	1
28159	0	6.52	1	0.78530	.	.	1
28160	0	6.52	1	0.78530	.	.	1
28161	0	6.52	1	0.78530	.	.	1
28162	0	6.52	1	0.78530	.	.	1
28163	0	6.52	1	0.78530	.	.	1
28164	0	6.52	1	0.78530	.	.	1
28165	0	6.52	1	0.78530	.	.	1
28166	0	6.52	1	0.78530	.	.	1
28167	0	6.52	1	0.78530	.	.	1
28168	0	6.52	1	0.78530	.	.	1
28169	0	6.52	1	0.78530	.	.	1
28170	0	6.52	1	0.78530	.	.	1
28171	0	6.52	1	0.78530	.	.	1
28172	0	6.52	1	0.78530	.	.	1
28173	0	6.52	1	0.78530	.	.	1
28174	0	6.52	1	0.78530	.	.	1
28175	0	6.52	1	0.78530	.	.	1

Obs	Sub_group	fuptimey	_CENSOR_	SURVIVAL	SDF_LCL	SDF_UCL	STRATUM
28176	0	6.52	1	0.78530	.	.	1
28177	0	6.52	1	0.78530	.	.	1
28178	0	6.52	1	0.78530	.	.	1
28179	0	6.52	1	0.78530	.	.	1
28180	0	6.52	1	0.78530	.	.	1
28181	0	6.52	1	0.78530	.	.	1
28182	0	6.52	1	0.78530	.	.	1
28183	0	6.53	0	0.78523	0.78055	0.78982	1
28184	0	6.53	1	0.78523	.	.	1
28185	0	6.53	1	0.78523	.	.	1
28186	0	6.53	1	0.78523	.	.	1
28187	0	6.53	1	0.78523	.	.	1
28188	0	6.53	1	0.78523	.	.	1
28189	0	6.53	1	0.78523	.	.	1
28190	0	6.53	1	0.78523	.	.	1
28191	0	6.53	1	0.78523	.	.	1
28192	0	6.53	1	0.78523	.	.	1
28193	0	6.53	1	0.78523	.	.	1
28194	0	6.53	1	0.78523	.	.	1
28195	0	6.53	1	0.78523	.	.	1
28196	0	6.53	1	0.78523	.	.	1
28197	0	6.53	1	0.78523	.	.	1
28198	0	6.53	1	0.78523	.	.	1
28199	0	6.53	1	0.78523	.	.	1
28200	0	6.53	1	0.78523	.	.	1
28201	0	6.53	1	0.78523	.	.	1
28202	0	6.53	1	0.78523	.	.	1
28203	0	6.53	1	0.78523	.	.	1
28204	0	6.53	1	0.78523	.	.	1
28205	0	6.53	1	0.78523	.	.	1
28206	0	6.54	0	0.78502	0.78034	0.78962	1
28207	0	6.54	1	0.78502	.	.	1
28208	0	6.54	1	0.78502	.	.	1
28209	0	6.54	1	0.78502	.	.	1
28210	0	6.54	1	0.78502	.	.	1

Obs	Sub_group	fuptimey	_CENSOR_	SURVIVAL	SDF_LCL	SDF_UCL	STRATUM
**28211**	0	6.54	1	0.78502	.	.	1
**28212**	0	6.54	1	0.78502	.	.	1
**28213**	0	6.54	1	0.78502	.	.	1
**28214**	0	6.54	1	0.78502	.	.	1
**28215**	0	6.54	1	0.78502	.	.	1
**28216**	0	6.54	1	0.78502	.	.	1
**28217**	0	6.54	1	0.78502	.	.	1
**28218**	0	6.54	1	0.78502	.	.	1
**28219**	0	6.54	1	0.78502	.	.	1
**28220**	0	6.54	1	0.78502	.	.	1
**28221**	0	6.54	1	0.78502	.	.	1
**28222**	0	6.54	1	0.78502	.	.	1
**28223**	0	6.54	1	0.78502	.	.	1
**28224**	0	6.54	1	0.78502	.	.	1
**28225**	0	6.54	1	0.78502	.	.	1
**28226**	0	6.54	1	0.78502	.	.	1
**28227**	0	6.54	1	0.78502	.	.	1
**28228**	0	6.54	1	0.78502	.	.	1
**28229**	0	6.54	1	0.78502	.	.	1
**28230**	0	6.54	1	0.78502	.	.	1
**28231**	0	6.54	1	0.78502	.	.	1
**28232**	0	6.54	1	0.78502	.	.	1
**28233**	0	6.54	1	0.78502	.	.	1
**28234**	0	6.54	1	0.78502	.	.	1
**28235**	0	6.54	1	0.78502	.	.	1
**28236**	0	6.54	1	0.78502	.	.	1
**28237**	0	6.55	0	0.78482	0.78013	0.78942	1
**28238**	0	6.55	1	0.78482	.	.	1
**28239**	0	6.55	1	0.78482	.	.	1
**28240**	0	6.55	1	0.78482	.	.	1
**28241**	0	6.55	1	0.78482	.	.	1
**28242**	0	6.55	1	0.78482	.	.	1
**28243**	0	6.55	1	0.78482	.	.	1
**28244**	0	6.55	1	0.78482	.	.	1
**28245**	0	6.55	1	0.78482	.	.	1

Obs	Sub_group	fuptimey	_CENSOR_	SURVIVAL	SDF_LCL	SDF_UCL	STRATUM
28246	0	6.55	1	0.78482	.	.	1
28247	0	6.55	1	0.78482	.	.	1
28248	0	6.55	1	0.78482	.	.	1
28249	0	6.55	1	0.78482	.	.	1
28250	0	6.55	1	0.78482	.	.	1
28251	0	6.55	1	0.78482	.	.	1
28252	0	6.55	1	0.78482	.	.	1
28253	0	6.55	1	0.78482	.	.	1
28254	0	6.55	1	0.78482	.	.	1
28255	0	6.55	1	0.78482	.	.	1
28256	0	6.55	1	0.78482	.	.	1
28257	0	6.55	1	0.78482	.	.	1
28258	0	6.55	1	0.78482	.	.	1
28259	0	6.55	1	0.78482	.	.	1
28260	0	6.55	1	0.78482	.	.	1
28261	0	6.55	1	0.78482	.	.	1
28262	0	6.55	1	0.78482	.	.	1
28263	0	6.55	1	0.78482	.	.	1
28264	0	6.55	1	0.78482	.	.	1
28265	0	6.55	1	0.78482	.	.	1
28266	0	6.55	1	0.78482	.	.	1
28267	0	6.55	1	0.78482	.	.	1
28268	0	6.55	1	0.78482	.	.	1
28269	0	6.55	1	0.78482	.	.	1
28270	0	6.55	1	0.78482	.	.	1
28271	0	6.55	1	0.78482	.	.	1
28272	0	6.55	1	0.78482	.	.	1
28273	0	6.55	1	0.78482	.	.	1
28274	0	6.55	1	0.78482	.	.	1
28275	0	6.55	1	0.78482	.	.	1
28276	0	6.55	1	0.78482	.	.	1
28277	0	6.56	0	0.78475	0.78006	0.78935	1
28278	0	6.56	1	0.78475	.	.	1
28279	0	6.56	1	0.78475	.	.	1
28280	0	6.56	1	0.78475	.	.	1

Obs	Sub_group	fuptimey	_CENSOR_	SURVIVAL	SDF_LCL	SDF_UCL	STRATUM
28281	0	6.56	1	0.78475	.	.	1
28282	0	6.56	1	0.78475	.	.	1
28283	0	6.56	1	0.78475	.	.	1
28284	0	6.56	1	0.78475	.	.	1
28285	0	6.56	1	0.78475	.	.	1
28286	0	6.56	1	0.78475	.	.	1
28287	0	6.56	1	0.78475	.	.	1
28288	0	6.56	1	0.78475	.	.	1
28289	0	6.56	1	0.78475	.	.	1
28290	0	6.56	1	0.78475	.	.	1
28291	0	6.56	1	0.78475	.	.	1
28292	0	6.56	1	0.78475	.	.	1
28293	0	6.56	1	0.78475	.	.	1
28294	0	6.56	1	0.78475	.	.	1
28295	0	6.56	1	0.78475	.	.	1
28296	0	6.56	1	0.78475	.	.	1
28297	0	6.56	1	0.78475	.	.	1
28298	0	6.56	1	0.78475	.	.	1
28299	0	6.56	1	0.78475	.	.	1
28300	0	6.56	1	0.78475	.	.	1
28301	0	6.56	1	0.78475	.	.	1
28302	0	6.56	1	0.78475	.	.	1
28303	0	6.56	1	0.78475	.	.	1
28304	0	6.56	1	0.78475	.	.	1
28305	0	6.56	1	0.78475	.	.	1
28306	0	6.56	1	0.78475	.	.	1
28307	0	6.56	1	0.78475	.	.	1
28308	0	6.56	1	0.78475	.	.	1
28309	0	6.56	1	0.78475	.	.	1
28310	0	6.56	1	0.78475	.	.	1
28311	0	6.56	1	0.78475	.	.	1
28312	0	6.56	1	0.78475	.	.	1
28313	0	6.56	1	0.78475	.	.	1
28314	0	6.56	1	0.78475	.	.	1
28315	0	6.56	1	0.78475	.	.	1

Obs	Sub_group	fuptimey	_CENSOR_	SURVIVAL	SDF_LCL	SDF_UCL	STRATUM
28316	0	6.56	1	0.78475	.	.	1
28317	0	6.56	1	0.78475	.	.	1
28318	0	6.56	1	0.78475	.	.	1
28319	0	6.56	1	0.78475	.	.	1
28320	0	6.57	0	0.78447	0.77978	0.78909	1
28321	0	6.57	1	0.78447	.	.	1
28322	0	6.57	1	0.78447	.	.	1
28323	0	6.57	1	0.78447	.	.	1
28324	0	6.57	1	0.78447	.	.	1
28325	0	6.57	1	0.78447	.	.	1
28326	0	6.57	1	0.78447	.	.	1
28327	0	6.57	1	0.78447	.	.	1
28328	0	6.57	1	0.78447	.	.	1
28329	0	6.57	1	0.78447	.	.	1
28330	0	6.57	1	0.78447	.	.	1
28331	0	6.57	1	0.78447	.	.	1
28332	0	6.57	1	0.78447	.	.	1
28333	0	6.57	1	0.78447	.	.	1
28334	0	6.57	1	0.78447	.	.	1
28335	0	6.57	1	0.78447	.	.	1
28336	0	6.57	1	0.78447	.	.	1
28337	0	6.57	1	0.78447	.	.	1
28338	0	6.57	1	0.78447	.	.	1
28339	0	6.57	1	0.78447	.	.	1
28340	0	6.57	1	0.78447	.	.	1
28341	0	6.57	1	0.78447	.	.	1
28342	0	6.57	1	0.78447	.	.	1
28343	0	6.57	1	0.78447	.	.	1
28344	0	6.57	1	0.78447	.	.	1
28345	0	6.57	1	0.78447	.	.	1
28346	0	6.57	1	0.78447	.	.	1
28347	0	6.57	1	0.78447	.	.	1
28348	0	6.57	1	0.78447	.	.	1
28349	0	6.58	0	0.78413	0.77943	0.78875	1
28350	0	6.58	1	0.78413	.	.	1

Obs	Sub_group	fuptimey	_CENSOR_	SURVIVAL	SDF_LCL	SDF_UCL	STRATUM
28351	0	6.58	1	0.78413	.	.	1
28352	0	6.58	1	0.78413	.	.	1
28353	0	6.58	1	0.78413	.	.	1
28354	0	6.58	1	0.78413	.	.	1
28355	0	6.58	1	0.78413	.	.	1
28356	0	6.58	1	0.78413	.	.	1
28357	0	6.58	1	0.78413	.	.	1
28358	0	6.58	1	0.78413	.	.	1
28359	0	6.58	1	0.78413	.	.	1
28360	0	6.58	1	0.78413	.	.	1
28361	0	6.58	1	0.78413	.	.	1
28362	0	6.58	1	0.78413	.	.	1
28363	0	6.58	1	0.78413	.	.	1
28364	0	6.58	1	0.78413	.	.	1
28365	0	6.58	1	0.78413	.	.	1
28366	0	6.58	1	0.78413	.	.	1
28367	0	6.58	1	0.78413	.	.	1
28368	0	6.58	1	0.78413	.	.	1
28369	0	6.58	1	0.78413	.	.	1
28370	0	6.58	1	0.78413	.	.	1
28371	0	6.58	1	0.78413	.	.	1
28372	0	6.58	1	0.78413	.	.	1
28373	0	6.58	1	0.78413	.	.	1
28374	0	6.58	1	0.78413	.	.	1
28375	0	6.58	1	0.78413	.	.	1
28376	0	6.58	1	0.78413	.	.	1
28377	0	6.58	1	0.78413	.	.	1
28378	0	6.58	1	0.78413	.	.	1
28379	0	6.58	1	0.78413	.	.	1
28380	0	6.58	1	0.78413	.	.	1
28381	0	6.58	1	0.78413	.	.	1
28382	0	6.58	1	0.78413	.	.	1
28383	0	6.58	1	0.78413	.	.	1
28384	0	6.58	1	0.78413	.	.	1
28385	0	6.58	1	0.78413	.	.	1

Obs	Sub_group	fuptimey	_CENSOR_	SURVIVAL	SDF_LCL	SDF_UCL	STRATUM
28386	0	6.58	1	0.78413	.	.	1
28387	0	6.58	1	0.78413	.	.	1
28388	0	6.58	1	0.78413	.	.	1
28389	0	6.58	1	0.78413	.	.	1
28390	0	6.59	0	0.78372	0.77900	0.78834	1
28391	0	6.59	1	0.78372	.	.	1
28392	0	6.59	1	0.78372	.	.	1
28393	0	6.59	1	0.78372	.	.	1
28394	0	6.59	1	0.78372	.	.	1
28395	0	6.59	1	0.78372	.	.	1
28396	0	6.59	1	0.78372	.	.	1
28397	0	6.59	1	0.78372	.	.	1
28398	0	6.59	1	0.78372	.	.	1
28399	0	6.59	1	0.78372	.	.	1
28400	0	6.59	1	0.78372	.	.	1
28401	0	6.59	1	0.78372	.	.	1
28402	0	6.59	1	0.78372	.	.	1
28403	0	6.59	1	0.78372	.	.	1
28404	0	6.59	1	0.78372	.	.	1
28405	0	6.59	1	0.78372	.	.	1
28406	0	6.59	1	0.78372	.	.	1
28407	0	6.59	1	0.78372	.	.	1
28408	0	6.59	1	0.78372	.	.	1
28409	0	6.59	1	0.78372	.	.	1
28410	0	6.59	1	0.78372	.	.	1
28411	0	6.59	1	0.78372	.	.	1
28412	0	6.59	1	0.78372	.	.	1
28413	0	6.59	1	0.78372	.	.	1
28414	0	6.59	1	0.78372	.	.	1
28415	0	6.59	1	0.78372	.	.	1
28416	0	6.59	1	0.78372	.	.	1
28417	0	6.59	1	0.78372	.	.	1
28418	0	6.59	1	0.78372	.	.	1
28419	0	6.59	1	0.78372	.	.	1
28420	0	6.59	1	0.78372	.	.	1

Obs	Sub_group	fuptimey	_CENSOR_	SURVIVAL	SDF_LCL	SDF_UCL	STRATUM
**28421**	0	6.59	1	0.78372	.	.	1
**28422**	0	6.59	1	0.78372	.	.	1
**28423**	0	6.59	1	0.78372	.	.	1
**28424**	0	6.59	1	0.78372	.	.	1
**28425**	0	6.59	1	0.78372	.	.	1
**28426**	0	6.59	1	0.78372	.	.	1
**28427**	0	6.59	1	0.78372	.	.	1
**28428**	0	6.60	0	0.78330	0.77858	0.78794	1
**28429**	0	6.60	1	0.78330	.	.	1
**28430**	0	6.60	1	0.78330	.	.	1
**28431**	0	6.60	1	0.78330	.	.	1
**28432**	0	6.60	1	0.78330	.	.	1
**28433**	0	6.60	1	0.78330	.	.	1
**28434**	0	6.60	1	0.78330	.	.	1
**28435**	0	6.60	1	0.78330	.	.	1
**28436**	0	6.60	1	0.78330	.	.	1
**28437**	0	6.60	1	0.78330	.	.	1
**28438**	0	6.60	1	0.78330	.	.	1
**28439**	0	6.60	1	0.78330	.	.	1
**28440**	0	6.60	1	0.78330	.	.	1
**28441**	0	6.60	1	0.78330	.	.	1
**28442**	0	6.60	1	0.78330	.	.	1
**28443**	0	6.60	1	0.78330	.	.	1
**28444**	0	6.60	1	0.78330	.	.	1
**28445**	0	6.60	1	0.78330	.	.	1
**28446**	0	6.60	1	0.78330	.	.	1
**28447**	0	6.60	1	0.78330	.	.	1
**28448**	0	6.60	1	0.78330	.	.	1
**28449**	0	6.60	1	0.78330	.	.	1
**28450**	0	6.60	1	0.78330	.	.	1
**28451**	0	6.60	1	0.78330	.	.	1
**28452**	0	6.60	1	0.78330	.	.	1
**28453**	0	6.60	1	0.78330	.	.	1
**28454**	0	6.60	1	0.78330	.	.	1
**28455**	0	6.60	1	0.78330	.	.	1

Obs	Sub_group	fuptimey	_CENSOR_	SURVIVAL	SDF_LCL	SDF_UCL	STRATUM
28456	0	6.60	1	0.78330	.	.	1
28457	0	6.61	0	0.78295	0.77822	0.78760	1
28458	0	6.61	1	0.78295	.	.	1
28459	0	6.61	1	0.78295	.	.	1
28460	0	6.61	1	0.78295	.	.	1
28461	0	6.61	1	0.78295	.	.	1
28462	0	6.61	1	0.78295	.	.	1
28463	0	6.61	1	0.78295	.	.	1
28464	0	6.61	1	0.78295	.	.	1
28465	0	6.61	1	0.78295	.	.	1
28466	0	6.61	1	0.78295	.	.	1
28467	0	6.61	1	0.78295	.	.	1
28468	0	6.61	1	0.78295	.	.	1
28469	0	6.61	1	0.78295	.	.	1
28470	0	6.61	1	0.78295	.	.	1
28471	0	6.61	1	0.78295	.	.	1
28472	0	6.61	1	0.78295	.	.	1
28473	0	6.61	1	0.78295	.	.	1
28474	0	6.61	1	0.78295	.	.	1
28475	0	6.61	1	0.78295	.	.	1
28476	0	6.61	1	0.78295	.	.	1
28477	0	6.61	1	0.78295	.	.	1
28478	0	6.61	1	0.78295	.	.	1
28479	0	6.61	1	0.78295	.	.	1
28480	0	6.61	1	0.78295	.	.	1
28481	0	6.61	1	0.78295	.	.	1
28482	0	6.62	0	0.78288	0.77815	0.78753	1
28483	0	6.62	1	0.78288	.	.	1
28484	0	6.62	1	0.78288	.	.	1
28485	0	6.62	1	0.78288	.	.	1
28486	0	6.62	1	0.78288	.	.	1
28487	0	6.62	1	0.78288	.	.	1
28488	0	6.62	1	0.78288	.	.	1
28489	0	6.62	1	0.78288	.	.	1
28490	0	6.62	1	0.78288	.	.	1

Obs	Sub_group	fuptimey	_CENSOR_	SURVIVAL	SDF_LCL	SDF_UCL	STRATUM
28491	0	6.62	1	0.78288	.	.	1
28492	0	6.62	1	0.78288	.	.	1
28493	0	6.62	1	0.78288	.	.	1
28494	0	6.62	1	0.78288	.	.	1
28495	0	6.62	1	0.78288	.	.	1
28496	0	6.62	1	0.78288	.	.	1
28497	0	6.62	1	0.78288	.	.	1
28498	0	6.62	1	0.78288	.	.	1
28499	0	6.62	1	0.78288	.	.	1
28500	0	6.62	1	0.78288	.	.	1
28501	0	6.62	1	0.78288	.	.	1
28502	0	6.62	1	0.78288	.	.	1
28503	0	6.62	1	0.78288	.	.	1
28504	0	6.62	1	0.78288	.	.	1
28505	0	6.62	1	0.78288	.	.	1
28506	0	6.62	1	0.78288	.	.	1
28507	0	6.62	1	0.78288	.	.	1
28508	0	6.62	1	0.78288	.	.	1
28509	0	6.62	1	0.78288	.	.	1
28510	0	6.62	1	0.78288	.	.	1
28511	0	6.62	1	0.78288	.	.	1
28512	0	6.62	1	0.78288	.	.	1
28513	0	6.62	1	0.78288	.	.	1
28514	0	6.62	1	0.78288	.	.	1
28515	0	6.62	1	0.78288	.	.	1
28516	0	6.62	1	0.78288	.	.	1
28517	0	6.62	1	0.78288	.	.	1
28518	0	6.62	1	0.78288	.	.	1
28519	0	6.62	1	0.78288	.	.	1
28520	0	6.62	1	0.78288	.	.	1
28521	0	6.62	1	0.78288	.	.	1
28522	0	6.62	1	0.78288	.	.	1
28523	0	6.62	1	0.78288	.	.	1
28524	0	6.62	1	0.78288	.	.	1
28525	0	6.62	1	0.78288	.	.	1

Obs	Sub_group	fuptimey	_CENSOR_	SURVIVAL	SDF_LCL	SDF_UCL	STRATUM
28526	0	6.62	1	0.78288	.	.	1
28527	0	6.62	1	0.78288	.	.	1
28528	0	6.62	1	0.78288	.	.	1
28529	0	6.63	0	0.78274	0.77801	0.78739	1
28530	0	6.63	1	0.78274	.	.	1
28531	0	6.63	1	0.78274	.	.	1
28532	0	6.63	1	0.78274	.	.	1
28533	0	6.63	1	0.78274	.	.	1
28534	0	6.63	1	0.78274	.	.	1
28535	0	6.63	1	0.78274	.	.	1
28536	0	6.63	1	0.78274	.	.	1
28537	0	6.63	1	0.78274	.	.	1
28538	0	6.63	1	0.78274	.	.	1
28539	0	6.63	1	0.78274	.	.	1
28540	0	6.63	1	0.78274	.	.	1
28541	0	6.63	1	0.78274	.	.	1
28542	0	6.63	1	0.78274	.	.	1
28543	0	6.63	1	0.78274	.	.	1
28544	0	6.63	1	0.78274	.	.	1
28545	0	6.63	1	0.78274	.	.	1
28546	0	6.63	1	0.78274	.	.	1
28547	0	6.63	1	0.78274	.	.	1
28548	0	6.63	1	0.78274	.	.	1
28549	0	6.64	0	0.78246	0.77772	0.78712	1
28550	0	6.64	1	0.78246	.	.	1
28551	0	6.64	1	0.78246	.	.	1
28552	0	6.64	1	0.78246	.	.	1
28553	0	6.64	1	0.78246	.	.	1
28554	0	6.64	1	0.78246	.	.	1
28555	0	6.64	1	0.78246	.	.	1
28556	0	6.64	1	0.78246	.	.	1
28557	0	6.64	1	0.78246	.	.	1
28558	0	6.64	1	0.78246	.	.	1
28559	0	6.64	1	0.78246	.	.	1
28560	0	6.64	1	0.78246	.	.	1

Obs	Sub_group	fuptimey	_CENSOR_	SURVIVAL	SDF_LCL	SDF_UCL	STRATUM
28561	0	6.64	1	0.78246	.	.	1
28562	0	6.64	1	0.78246	.	.	1
28563	0	6.64	1	0.78246	.	.	1
28564	0	6.64	1	0.78246	.	.	1
28565	0	6.64	1	0.78246	.	.	1
28566	0	6.64	1	0.78246	.	.	1
28567	0	6.64	1	0.78246	.	.	1
28568	0	6.64	1	0.78246	.	.	1
28569	0	6.64	1	0.78246	.	.	1
28570	0	6.64	1	0.78246	.	.	1
28571	0	6.64	1	0.78246	.	.	1
28572	0	6.64	1	0.78246	.	.	1
28573	0	6.64	1	0.78246	.	.	1
28574	0	6.64	1	0.78246	.	.	1
28575	0	6.64	1	0.78246	.	.	1
28576	0	6.64	1	0.78246	.	.	1
28577	0	6.64	1	0.78246	.	.	1
28578	0	6.64	1	0.78246	.	.	1
28579	0	6.64	1	0.78246	.	.	1
28580	0	6.64	1	0.78246	.	.	1
28581	0	6.64	1	0.78246	.	.	1
28582	0	6.64	1	0.78246	.	.	1
28583	0	6.64	1	0.78246	.	.	1
28584	0	6.64	1	0.78246	.	.	1
28585	0	6.64	1	0.78246	.	.	1
28586	0	6.64	1	0.78246	.	.	1
28587	0	6.65	0	0.78190	0.77714	0.78657	1
28588	0	6.65	1	0.78190	.	.	1
28589	0	6.65	1	0.78190	.	.	1
28590	0	6.65	1	0.78190	.	.	1
28591	0	6.65	1	0.78190	.	.	1
28592	0	6.65	1	0.78190	.	.	1
28593	0	6.65	1	0.78190	.	.	1
28594	0	6.65	1	0.78190	.	.	1
28595	0	6.65	1	0.78190	.	.	1

Obs	Sub_group	fuptimey	_CENSOR_	SURVIVAL	SDF_LCL	SDF_UCL	STRATUM
**28596**	0	6.65	1	0.78190	.	.	1
**28597**	0	6.65	1	0.78190	.	.	1
**28598**	0	6.65	1	0.78190	.	.	1
**28599**	0	6.65	1	0.78190	.	.	1
**28600**	0	6.65	1	0.78190	.	.	1
**28601**	0	6.65	1	0.78190	.	.	1
**28602**	0	6.65	1	0.78190	.	.	1
**28603**	0	6.65	1	0.78190	.	.	1
**28604**	0	6.65	1	0.78190	.	.	1
**28605**	0	6.65	1	0.78190	.	.	1
**28606**	0	6.65	1	0.78190	.	.	1
**28607**	0	6.65	1	0.78190	.	.	1
**28608**	0	6.65	1	0.78190	.	.	1
**28609**	0	6.65	1	0.78190	.	.	1
**28610**	0	6.65	1	0.78190	.	.	1
**28611**	0	6.65	1	0.78190	.	.	1
**28612**	0	6.65	1	0.78190	.	.	1
**28613**	0	6.65	1	0.78190	.	.	1
**28614**	0	6.65	1	0.78190	.	.	1
**28615**	0	6.65	1	0.78190	.	.	1
**28616**	0	6.65	1	0.78190	.	.	1
**28617**	0	6.65	1	0.78190	.	.	1
**28618**	0	6.65	1	0.78190	.	.	1
**28619**	0	6.66	0	0.78155	0.77678	0.78622	1
**28620**	0	6.66	1	0.78155	.	.	1
**28621**	0	6.66	1	0.78155	.	.	1
**28622**	0	6.66	1	0.78155	.	.	1
**28623**	0	6.66	1	0.78155	.	.	1
**28624**	0	6.66	1	0.78155	.	.	1
**28625**	0	6.66	1	0.78155	.	.	1
**28626**	0	6.66	1	0.78155	.	.	1
**28627**	0	6.66	1	0.78155	.	.	1
**28628**	0	6.66	1	0.78155	.	.	1
**28629**	0	6.66	1	0.78155	.	.	1
**28630**	0	6.66	1	0.78155	.	.	1

Obs	Sub_group	fuptimey	_CENSOR_	SURVIVAL	SDF_LCL	SDF_UCL	STRATUM
28631	0	6.66	1	0.78155	.	.	1
28632	0	6.66	1	0.78155	.	.	1
28633	0	6.66	1	0.78155	.	.	1
28634	0	6.66	1	0.78155	.	.	1
28635	0	6.66	1	0.78155	.	.	1
28636	0	6.66	1	0.78155	.	.	1
28637	0	6.66	1	0.78155	.	.	1
28638	0	6.66	1	0.78155	.	.	1
28639	0	6.66	1	0.78155	.	.	1
28640	0	6.66	1	0.78155	.	.	1
28641	0	6.66	1	0.78155	.	.	1
28642	0	6.66	1	0.78155	.	.	1
28643	0	6.66	1	0.78155	.	.	1
28644	0	6.66	1	0.78155	.	.	1
28645	0	6.66	1	0.78155	.	.	1
28646	0	6.66	1	0.78155	.	.	1
28647	0	6.66	1	0.78155	.	.	1
28648	0	6.66	1	0.78155	.	.	1
28649	0	6.66	1	0.78155	.	.	1
28650	0	6.66	1	0.78155	.	.	1
28651	0	6.66	1	0.78155	.	.	1
28652	0	6.67	0	0.78148	0.77671	0.78615	1
28653	0	6.67	1	0.78148	.	.	1
28654	0	6.67	1	0.78148	.	.	1
28655	0	6.67	1	0.78148	.	.	1
28656	0	6.67	1	0.78148	.	.	1
28657	0	6.67	1	0.78148	.	.	1
28658	0	6.67	1	0.78148	.	.	1
28659	0	6.67	1	0.78148	.	.	1
28660	0	6.67	1	0.78148	.	.	1
28661	0	6.67	1	0.78148	.	.	1
28662	0	6.67	1	0.78148	.	.	1
28663	0	6.67	1	0.78148	.	.	1
28664	0	6.67	1	0.78148	.	.	1
28665	0	6.67	1	0.78148	.	.	1

Obs	Sub_group	fuptimey	_CENSOR_	SURVIVAL	SDF_LCL	SDF_UCL	STRATUM
28666	0	6.67	1	0.78148	.	.	1
28667	0	6.67	1	0.78148	.	.	1
28668	0	6.67	1	0.78148	.	.	1
28669	0	6.67	1	0.78148	.	.	1
28670	0	6.67	1	0.78148	.	.	1
28671	0	6.67	1	0.78148	.	.	1
28672	0	6.67	1	0.78148	.	.	1
28673	0	6.67	1	0.78148	.	.	1
28674	0	6.67	1	0.78148	.	.	1
28675	0	6.67	1	0.78148	.	.	1
28676	0	6.67	1	0.78148	.	.	1
28677	0	6.67	1	0.78148	.	.	1
28678	0	6.67	1	0.78148	.	.	1
28679	0	6.67	1	0.78148	.	.	1
28680	0	6.67	1	0.78148	.	.	1
28681	0	6.67	1	0.78148	.	.	1
28682	0	6.67	1	0.78148	.	.	1
28683	0	6.67	1	0.78148	.	.	1
28684	0	6.68	0	0.78140	0.77664	0.78608	1
28685	0	6.68	1	0.78140	.	.	1
28686	0	6.68	1	0.78140	.	.	1
28687	0	6.68	1	0.78140	.	.	1
28688	0	6.68	1	0.78140	.	.	1
28689	0	6.68	1	0.78140	.	.	1
28690	0	6.68	1	0.78140	.	.	1
28691	0	6.68	1	0.78140	.	.	1
28692	0	6.68	1	0.78140	.	.	1
28693	0	6.68	1	0.78140	.	.	1
28694	0	6.68	1	0.78140	.	.	1
28695	0	6.68	1	0.78140	.	.	1
28696	0	6.68	1	0.78140	.	.	1
28697	0	6.68	1	0.78140	.	.	1
28698	0	6.68	1	0.78140	.	.	1
28699	0	6.68	1	0.78140	.	.	1
28700	0	6.68	1	0.78140	.	.	1

Obs	Sub_group	fuptimey	_CENSOR_	SURVIVAL	SDF_LCL	SDF_UCL	STRATUM
**28701**	0	6.68	1	0.78140	.	.	1
**28702**	0	6.68	1	0.78140	.	.	1
**28703**	0	6.68	1	0.78140	.	.	1
**28704**	0	6.68	1	0.78140	.	.	1
**28705**	0	6.68	1	0.78140	.	.	1
**28706**	0	6.68	1	0.78140	.	.	1
**28707**	0	6.68	1	0.78140	.	.	1
**28708**	0	6.68	1	0.78140	.	.	1
**28709**	0	6.68	1	0.78140	.	.	1
**28710**	0	6.68	1	0.78140	.	.	1
**28711**	0	6.68	1	0.78140	.	.	1
**28712**	0	6.68	1	0.78140	.	.	1
**28713**	0	6.68	1	0.78140	.	.	1
**28714**	0	6.68	1	0.78140	.	.	1
**28715**	0	6.68	1	0.78140	.	.	1
**28716**	0	6.68	1	0.78140	.	.	1
**28717**	0	6.68	1	0.78140	.	.	1
**28718**	0	6.68	1	0.78140	.	.	1
**28719**	0	6.68	1	0.78140	.	.	1
**28720**	0	6.68	1	0.78140	.	.	1
**28721**	0	6.68	1	0.78140	.	.	1
**28722**	0	6.68	1	0.78140	.	.	1
**28723**	0	6.68	1	0.78140	.	.	1
**28724**	0	6.68	1	0.78140	.	.	1
**28725**	0	6.69	0	0.78112	0.77635	0.78581	1
**28726**	0	6.69	1	0.78112	.	.	1
**28727**	0	6.69	1	0.78112	.	.	1
**28728**	0	6.69	1	0.78112	.	.	1
**28729**	0	6.69	1	0.78112	.	.	1
**28730**	0	6.69	1	0.78112	.	.	1
**28731**	0	6.69	1	0.78112	.	.	1
**28732**	0	6.69	1	0.78112	.	.	1
**28733**	0	6.69	1	0.78112	.	.	1
**28734**	0	6.69	1	0.78112	.	.	1
**28735**	0	6.69	1	0.78112	.	.	1

Obs	Sub_group	fuptimey	_CENSOR_	SURVIVAL	SDF_LCL	SDF_UCL	STRATUM
28736	0	6.69	1	0.78112	.	.	1
28737	0	6.69	1	0.78112	.	.	1
28738	0	6.69	1	0.78112	.	.	1
28739	0	6.69	1	0.78112	.	.	1
28740	0	6.69	1	0.78112	.	.	1
28741	0	6.69	1	0.78112	.	.	1
28742	0	6.69	1	0.78112	.	.	1
28743	0	6.69	1	0.78112	.	.	1
28744	0	6.69	1	0.78112	.	.	1
28745	0	6.69	1	0.78112	.	.	1
28746	0	6.69	1	0.78112	.	.	1
28747	0	6.69	1	0.78112	.	.	1
28748	0	6.69	1	0.78112	.	.	1
28749	0	6.69	1	0.78112	.	.	1
28750	0	6.69	1	0.78112	.	.	1
28751	0	6.69	1	0.78112	.	.	1
28752	0	6.69	1	0.78112	.	.	1
28753	0	6.69	1	0.78112	.	.	1
28754	0	6.69	1	0.78112	.	.	1
28755	0	6.69	1	0.78112	.	.	1
28756	0	6.70	0	0.78091	0.77613	0.78560	1
28757	0	6.70	1	0.78091	.	.	1
28758	0	6.70	1	0.78091	.	.	1
28759	0	6.70	1	0.78091	.	.	1
28760	0	6.70	1	0.78091	.	.	1
28761	0	6.70	1	0.78091	.	.	1
28762	0	6.70	1	0.78091	.	.	1
28763	0	6.70	1	0.78091	.	.	1
28764	0	6.70	1	0.78091	.	.	1
28765	0	6.70	1	0.78091	.	.	1
28766	0	6.70	1	0.78091	.	.	1
28767	0	6.70	1	0.78091	.	.	1
28768	0	6.70	1	0.78091	.	.	1
28769	0	6.70	1	0.78091	.	.	1
28770	0	6.70	1	0.78091	.	.	1

Obs	Sub_group	fuptimey	_CENSOR_	SURVIVAL	SDF_LCL	SDF_UCL	STRATUM
**28771**	0	6.70	1	0.78091	.	.	1
**28772**	0	6.70	1	0.78091	.	.	1
**28773**	0	6.70	1	0.78091	.	.	1
**28774**	0	6.70	1	0.78091	.	.	1
**28775**	0	6.70	1	0.78091	.	.	1
**28776**	0	6.70	1	0.78091	.	.	1
**28777**	0	6.70	1	0.78091	.	.	1
**28778**	0	6.70	1	0.78091	.	.	1
**28779**	0	6.70	1	0.78091	.	.	1
**28780**	0	6.70	1	0.78091	.	.	1
**28781**	0	6.70	1	0.78091	.	.	1
**28782**	0	6.70	1	0.78091	.	.	1
**28783**	0	6.70	1	0.78091	.	.	1
**28784**	0	6.70	1	0.78091	.	.	1
**28785**	0	6.70	1	0.78091	.	.	1
**28786**	0	6.70	1	0.78091	.	.	1
**28787**	0	6.70	1	0.78091	.	.	1
**28788**	0	6.70	1	0.78091	.	.	1
**28789**	0	6.70	1	0.78091	.	.	1
**28790**	0	6.70	1	0.78091	.	.	1
**28791**	0	6.70	1	0.78091	.	.	1
**28792**	0	6.71	0	0.78062	0.77583	0.78532	1
**28793**	0	6.71	1	0.78062	.	.	1
**28794**	0	6.71	1	0.78062	.	.	1
**28795**	0	6.71	1	0.78062	.	.	1
**28796**	0	6.71	1	0.78062	.	.	1
**28797**	0	6.71	1	0.78062	.	.	1
**28798**	0	6.71	1	0.78062	.	.	1
**28799**	0	6.71	1	0.78062	.	.	1
**28800**	0	6.71	1	0.78062	.	.	1
**28801**	0	6.71	1	0.78062	.	.	1
**28802**	0	6.71	1	0.78062	.	.	1
**28803**	0	6.71	1	0.78062	.	.	1
**28804**	0	6.71	1	0.78062	.	.	1
**28805**	0	6.71	1	0.78062	.	.	1

Obs	Sub_group	fuptimey	_CENSOR_	SURVIVAL	SDF_LCL	SDF_UCL	STRATUM
28806	0	6.71	1	0.78062	.	.	1
28807	0	6.71	1	0.78062	.	.	1
28808	0	6.71	1	0.78062	.	.	1
28809	0	6.71	1	0.78062	.	.	1
28810	0	6.71	1	0.78062	.	.	1
28811	0	6.71	1	0.78062	.	.	1
28812	0	6.71	1	0.78062	.	.	1
28813	0	6.71	1	0.78062	.	.	1
28814	0	6.71	1	0.78062	.	.	1
28815	0	6.71	1	0.78062	.	.	1
28816	0	6.71	1	0.78062	.	.	1
28817	0	6.71	1	0.78062	.	.	1
28818	0	6.71	1	0.78062	.	.	1
28819	0	6.71	1	0.78062	.	.	1
28820	0	6.71	1	0.78062	.	.	1
28821	0	6.72	0	0.78033	0.77554	0.78504	1
28822	0	6.72	1	0.78033	.	.	1
28823	0	6.72	1	0.78033	.	.	1
28824	0	6.72	1	0.78033	.	.	1
28825	0	6.72	1	0.78033	.	.	1
28826	0	6.72	1	0.78033	.	.	1
28827	0	6.72	1	0.78033	.	.	1
28828	0	6.72	1	0.78033	.	.	1
28829	0	6.72	1	0.78033	.	.	1
28830	0	6.72	1	0.78033	.	.	1
28831	0	6.72	1	0.78033	.	.	1
28832	0	6.72	1	0.78033	.	.	1
28833	0	6.72	1	0.78033	.	.	1
28834	0	6.72	1	0.78033	.	.	1
28835	0	6.72	1	0.78033	.	.	1
28836	0	6.72	1	0.78033	.	.	1
28837	0	6.72	1	0.78033	.	.	1
28838	0	6.72	1	0.78033	.	.	1
28839	0	6.72	1	0.78033	.	.	1
28840	0	6.72	1	0.78033	.	.	1

Obs	Sub_group	fuptimey	_CENSOR_	SURVIVAL	SDF_LCL	SDF_UCL	STRATUM
28841	0	6.72	1	0.78033	.	.	1
28842	0	6.72	1	0.78033	.	.	1
28843	0	6.72	1	0.78033	.	.	1
28844	0	6.72	1	0.78033	.	.	1
28845	0	6.72	1	0.78033	.	.	1
28846	0	6.72	1	0.78033	.	.	1
28847	0	6.72	1	0.78033	.	.	1
28848	0	6.72	1	0.78033	.	.	1
28849	0	6.72	1	0.78033	.	.	1
28850	0	6.72	1	0.78033	.	.	1
28851	0	6.72	1	0.78033	.	.	1
28852	0	6.72	1	0.78033	.	.	1
28853	0	6.73	0	0.78026	0.77547	0.78496	1
28854	0	6.73	1	0.78026	.	.	1
28855	0	6.73	1	0.78026	.	.	1
28856	0	6.73	1	0.78026	.	.	1
28857	0	6.73	1	0.78026	.	.	1
28858	0	6.73	1	0.78026	.	.	1
28859	0	6.73	1	0.78026	.	.	1
28860	0	6.73	1	0.78026	.	.	1
28861	0	6.73	1	0.78026	.	.	1
28862	0	6.73	1	0.78026	.	.	1
28863	0	6.73	1	0.78026	.	.	1
28864	0	6.73	1	0.78026	.	.	1
28865	0	6.73	1	0.78026	.	.	1
28866	0	6.73	1	0.78026	.	.	1
28867	0	6.73	1	0.78026	.	.	1
28868	0	6.73	1	0.78026	.	.	1
28869	0	6.73	1	0.78026	.	.	1
28870	0	6.73	1	0.78026	.	.	1
28871	0	6.73	1	0.78026	.	.	1
28872	0	6.73	1	0.78026	.	.	1
28873	0	6.73	1	0.78026	.	.	1
28874	0	6.73	1	0.78026	.	.	1
28875	0	6.73	1	0.78026	.	.	1

Obs	Sub_group	fuptimey	_CENSOR_	SURVIVAL	SDF_LCL	SDF_UCL	STRATUM
28876	0	6.73	1	0.78026	.	.	1
28877	0	6.73	1	0.78026	.	.	1
28878	0	6.73	1	0.78026	.	.	1
28879	0	6.73	1	0.78026	.	.	1
28880	0	6.73	1	0.78026	.	.	1
28881	0	6.73	1	0.78026	.	.	1
28882	0	6.73	1	0.78026	.	.	1
28883	0	6.73	1	0.78026	.	.	1
28884	0	6.73	1	0.78026	.	.	1
28885	0	6.73	1	0.78026	.	.	1
28886	0	6.74	0	0.78004	0.77525	0.78475	1
28887	0	6.74	1	0.78004	.	.	1
28888	0	6.74	1	0.78004	.	.	1
28889	0	6.74	1	0.78004	.	.	1
28890	0	6.74	1	0.78004	.	.	1
28891	0	6.74	1	0.78004	.	.	1
28892	0	6.74	1	0.78004	.	.	1
28893	0	6.74	1	0.78004	.	.	1
28894	0	6.74	1	0.78004	.	.	1
28895	0	6.74	1	0.78004	.	.	1
28896	0	6.74	1	0.78004	.	.	1
28897	0	6.74	1	0.78004	.	.	1
28898	0	6.74	1	0.78004	.	.	1
28899	0	6.74	1	0.78004	.	.	1
28900	0	6.74	1	0.78004	.	.	1
28901	0	6.74	1	0.78004	.	.	1
28902	0	6.74	1	0.78004	.	.	1
28903	0	6.74	1	0.78004	.	.	1
28904	0	6.74	1	0.78004	.	.	1
28905	0	6.74	1	0.78004	.	.	1
28906	0	6.74	1	0.78004	.	.	1
28907	0	6.74	1	0.78004	.	.	1
28908	0	6.74	1	0.78004	.	.	1
28909	0	6.74	1	0.78004	.	.	1
28910	0	6.74	1	0.78004	.	.	1

Obs	Sub_group	fuptimey	_CENSOR_	SURVIVAL	SDF_LCL	SDF_UCL	STRATUM
28911	0	6.74	1	0.78004	.	.	1
28912	0	6.74	1	0.78004	.	.	1
28913	0	6.74	1	0.78004	.	.	1
28914	0	6.74	1	0.78004	.	.	1
28915	0	6.74	1	0.78004	.	.	1
28916	0	6.74	1	0.78004	.	.	1
28917	0	6.75	0	0.77990	0.77510	0.78461	1
28918	0	6.75	1	0.77990	.	.	1
28919	0	6.75	1	0.77990	.	.	1
28920	0	6.75	1	0.77990	.	.	1
28921	0	6.75	1	0.77990	.	.	1
28922	0	6.75	1	0.77990	.	.	1
28923	0	6.75	1	0.77990	.	.	1
28924	0	6.75	1	0.77990	.	.	1
28925	0	6.75	1	0.77990	.	.	1
28926	0	6.75	1	0.77990	.	.	1
28927	0	6.75	1	0.77990	.	.	1
28928	0	6.75	1	0.77990	.	.	1
28929	0	6.75	1	0.77990	.	.	1
28930	0	6.75	1	0.77990	.	.	1
28931	0	6.75	1	0.77990	.	.	1
28932	0	6.75	1	0.77990	.	.	1
28933	0	6.75	1	0.77990	.	.	1
28934	0	6.75	1	0.77990	.	.	1
28935	0	6.75	1	0.77990	.	.	1
28936	0	6.75	1	0.77990	.	.	1
28937	0	6.75	1	0.77990	.	.	1
28938	0	6.75	1	0.77990	.	.	1
28939	0	6.75	1	0.77990	.	.	1
28940	0	6.75	1	0.77990	.	.	1
28941	0	6.75	1	0.77990	.	.	1
28942	0	6.75	1	0.77990	.	.	1
28943	0	6.75	1	0.77990	.	.	1
28944	0	6.75	1	0.77990	.	.	1
28945	0	6.75	1	0.77990	.	.	1

Obs	Sub_group	fuptimey	_CENSOR_	SURVIVAL	SDF_LCL	SDF_UCL	STRATUM
28946	0	6.75	1	0.77990	.	.	1
28947	0	6.76	0	0.77983	0.77502	0.78454	1
28948	0	6.76	1	0.77983	.	.	1
28949	0	6.76	1	0.77983	.	.	1
28950	0	6.76	1	0.77983	.	.	1
28951	0	6.76	1	0.77983	.	.	1
28952	0	6.76	1	0.77983	.	.	1
28953	0	6.76	1	0.77983	.	.	1
28954	0	6.76	1	0.77983	.	.	1
28955	0	6.76	1	0.77983	.	.	1
28956	0	6.76	1	0.77983	.	.	1
28957	0	6.76	1	0.77983	.	.	1
28958	0	6.76	1	0.77983	.	.	1
28959	0	6.76	1	0.77983	.	.	1
28960	0	6.76	1	0.77983	.	.	1
28961	0	6.76	1	0.77983	.	.	1
28962	0	6.76	1	0.77983	.	.	1
28963	0	6.76	1	0.77983	.	.	1
28964	0	6.76	1	0.77983	.	.	1
28965	0	6.76	1	0.77983	.	.	1
28966	0	6.76	1	0.77983	.	.	1
28967	0	6.76	1	0.77983	.	.	1
28968	0	6.76	1	0.77983	.	.	1
28969	0	6.76	1	0.77983	.	.	1
28970	0	6.76	1	0.77983	.	.	1
28971	0	6.76	1	0.77983	.	.	1
28972	0	6.76	1	0.77983	.	.	1
28973	0	6.76	1	0.77983	.	.	1
28974	0	6.76	1	0.77983	.	.	1
28975	0	6.76	1	0.77983	.	.	1
28976	0	6.76	1	0.77983	.	.	1
28977	0	6.76	1	0.77983	.	.	1
28978	0	6.76	1	0.77983	.	.	1
28979	0	6.76	1	0.77983	.	.	1
28980	0	6.76	1	0.77983	.	.	1

Obs	Sub_group	fuptimey	_CENSOR_	SURVIVAL	SDF_LCL	SDF_UCL	STRATUM
28981	0	6.77	0	0.77961	0.77480	0.78433	1
28982	0	6.77	1	0.77961	.	.	1
28983	0	6.77	1	0.77961	.	.	1
28984	0	6.77	1	0.77961	.	.	1
28985	0	6.77	1	0.77961	.	.	1
28986	0	6.77	1	0.77961	.	.	1
28987	0	6.77	1	0.77961	.	.	1
28988	0	6.77	1	0.77961	.	.	1
28989	0	6.77	1	0.77961	.	.	1
28990	0	6.77	1	0.77961	.	.	1
28991	0	6.77	1	0.77961	.	.	1
28992	0	6.77	1	0.77961	.	.	1
28993	0	6.77	1	0.77961	.	.	1
28994	0	6.77	1	0.77961	.	.	1
28995	0	6.77	1	0.77961	.	.	1
28996	0	6.77	1	0.77961	.	.	1
28997	0	6.77	1	0.77961	.	.	1
28998	0	6.77	1	0.77961	.	.	1
28999	0	6.77	1	0.77961	.	.	1
29000	0	6.77	1	0.77961	.	.	1
29001	0	6.77	1	0.77961	.	.	1
29002	0	6.77	1	0.77961	.	.	1
29003	0	6.77	1	0.77961	.	.	1
29004	0	6.77	1	0.77961	.	.	1
29005	0	6.77	1	0.77961	.	.	1
29006	0	6.77	1	0.77961	.	.	1
29007	0	6.77	1	0.77961	.	.	1
29008	0	6.77	1	0.77961	.	.	1
29009	0	6.77	1	0.77961	.	.	1
29010	0	6.77	1	0.77961	.	.	1
29011	0	6.77	1	0.77961	.	.	1
29012	0	6.77	1	0.77961	.	.	1
29013	0	6.77	1	0.77961	.	.	1
29014	0	6.77	1	0.77961	.	.	1
29015	0	6.77	1	0.77961	.	.	1

Obs	Sub_group	fuptimey	_CENSOR_	SURVIVAL	SDF_LCL	SDF_UCL	STRATUM
29016	0	6.77	1	0.77961	.	.	1
29017	0	6.77	1	0.77961	.	.	1
29018	0	6.77	1	0.77961	.	.	1
29019	0	6.77	1	0.77961	.	.	1
29020	0	6.77	1	0.77961	.	.	1
29021	0	6.78	0	0.77953	0.77472	0.78426	1
29022	0	6.78	1	0.77953	.	.	1
29023	0	6.78	1	0.77953	.	.	1
29024	0	6.78	1	0.77953	.	.	1
29025	0	6.78	1	0.77953	.	.	1
29026	0	6.78	1	0.77953	.	.	1
29027	0	6.78	1	0.77953	.	.	1
29028	0	6.78	1	0.77953	.	.	1
29029	0	6.78	1	0.77953	.	.	1
29030	0	6.78	1	0.77953	.	.	1
29031	0	6.78	1	0.77953	.	.	1
29032	0	6.78	1	0.77953	.	.	1
29033	0	6.78	1	0.77953	.	.	1
29034	0	6.78	1	0.77953	.	.	1
29035	0	6.78	1	0.77953	.	.	1
29036	0	6.78	1	0.77953	.	.	1
29037	0	6.78	1	0.77953	.	.	1
29038	0	6.78	1	0.77953	.	.	1
29039	0	6.78	1	0.77953	.	.	1
29040	0	6.78	1	0.77953	.	.	1
29041	0	6.78	1	0.77953	.	.	1
29042	0	6.78	1	0.77953	.	.	1
29043	0	6.78	1	0.77953	.	.	1
29044	0	6.78	1	0.77953	.	.	1
29045	0	6.79	0	0.77917	0.77435	0.78390	1
29046	0	6.79	1	0.77917	.	.	1
29047	0	6.79	1	0.77917	.	.	1
29048	0	6.79	1	0.77917	.	.	1
29049	0	6.79	1	0.77917	.	.	1
29050	0	6.79	1	0.77917	.	.	1

Obs	Sub_group	fuptimey	_CENSOR_	SURVIVAL	SDF_LCL	SDF_UCL	STRATUM
29051	0	6.79	1	0.77917	.	.	1
29052	0	6.79	1	0.77917	.	.	1
29053	0	6.79	1	0.77917	.	.	1
29054	0	6.79	1	0.77917	.	.	1
29055	0	6.79	1	0.77917	.	.	1
29056	0	6.79	1	0.77917	.	.	1
29057	0	6.79	1	0.77917	.	.	1
29058	0	6.79	1	0.77917	.	.	1
29059	0	6.79	1	0.77917	.	.	1
29060	0	6.79	1	0.77917	.	.	1
29061	0	6.79	1	0.77917	.	.	1
29062	0	6.79	1	0.77917	.	.	1
29063	0	6.79	1	0.77917	.	.	1
29064	0	6.79	1	0.77917	.	.	1
29065	0	6.79	1	0.77917	.	.	1
29066	0	6.79	1	0.77917	.	.	1
29067	0	6.79	1	0.77917	.	.	1
29068	0	6.79	1	0.77917	.	.	1
29069	0	6.79	1	0.77917	.	.	1
29070	0	6.79	1	0.77917	.	.	1
29071	0	6.79	1	0.77917	.	.	1
29072	0	6.79	1	0.77917	.	.	1
29073	0	6.79	1	0.77917	.	.	1
29074	0	6.79	1	0.77917	.	.	1
29075	0	6.79	1	0.77917	.	.	1
29076	0	6.79	1	0.77917	.	.	1
29077	0	6.79	1	0.77917	.	.	1
29078	0	6.79	1	0.77917	.	.	1
29079	0	6.80	0	0.77887	0.77405	0.78361	1
29080	0	6.80	1	0.77887	.	.	1
29081	0	6.80	1	0.77887	.	.	1
29082	0	6.80	1	0.77887	.	.	1
29083	0	6.80	1	0.77887	.	.	1
29084	0	6.80	1	0.77887	.	.	1
29085	0	6.80	1	0.77887	.	.	1

Obs	Sub_group	fuptimey	_CENSOR_	SURVIVAL	SDF_LCL	SDF_UCL	STRATUM
**29086**	0	6.80	1	0.77887	.	.	1
**29087**	0	6.80	1	0.77887	.	.	1
**29088**	0	6.80	1	0.77887	.	.	1
**29089**	0	6.80	1	0.77887	.	.	1
**29090**	0	6.80	1	0.77887	.	.	1
**29091**	0	6.80	1	0.77887	.	.	1
**29092**	0	6.80	1	0.77887	.	.	1
**29093**	0	6.80	1	0.77887	.	.	1
**29094**	0	6.80	1	0.77887	.	.	1
**29095**	0	6.80	1	0.77887	.	.	1
**29096**	0	6.80	1	0.77887	.	.	1
**29097**	0	6.81	0	0.77865	0.77382	0.78340	1
**29098**	0	6.81	1	0.77865	.	.	1
**29099**	0	6.81	1	0.77865	.	.	1
**29100**	0	6.81	1	0.77865	.	.	1
**29101**	0	6.81	1	0.77865	.	.	1
**29102**	0	6.81	1	0.77865	.	.	1
**29103**	0	6.81	1	0.77865	.	.	1
**29104**	0	6.81	1	0.77865	.	.	1
**29105**	0	6.81	1	0.77865	.	.	1
**29106**	0	6.81	1	0.77865	.	.	1
**29107**	0	6.81	1	0.77865	.	.	1
**29108**	0	6.81	1	0.77865	.	.	1
**29109**	0	6.81	1	0.77865	.	.	1
**29110**	0	6.81	1	0.77865	.	.	1
**29111**	0	6.81	1	0.77865	.	.	1
**29112**	0	6.81	1	0.77865	.	.	1
**29113**	0	6.81	1	0.77865	.	.	1
**29114**	0	6.81	1	0.77865	.	.	1
**29115**	0	6.81	1	0.77865	.	.	1
**29116**	0	6.81	1	0.77865	.	.	1
**29117**	0	6.81	1	0.77865	.	.	1
**29118**	0	6.81	1	0.77865	.	.	1
**29119**	0	6.81	1	0.77865	.	.	1
**29120**	0	6.81	1	0.77865	.	.	1

Obs	Sub_group	fuptimey	_CENSOR_	SURVIVAL	SDF_LCL	SDF_UCL	STRATUM
29121	0	6.81	1	0.77865	.	.	1
29122	0	6.81	1	0.77865	.	.	1
29123	0	6.81	1	0.77865	.	.	1
29124	0	6.81	1	0.77865	.	.	1
29125	0	6.81	1	0.77865	.	.	1
29126	0	6.81	1	0.77865	.	.	1
29127	0	6.81	1	0.77865	.	.	1
29128	0	6.81	1	0.77865	.	.	1
29129	0	6.81	1	0.77865	.	.	1
29130	0	6.81	1	0.77865	.	.	1
29131	0	6.81	1	0.77865	.	.	1
29132	0	6.81	1	0.77865	.	.	1
29133	0	6.81	1	0.77865	.	.	1
29134	0	6.81	1	0.77865	.	.	1
29135	0	6.81	1	0.77865	.	.	1
29136	0	6.82	0	0.77843	0.77360	0.78318	1
29137	0	6.82	1	0.77843	.	.	1
29138	0	6.82	1	0.77843	.	.	1
29139	0	6.82	1	0.77843	.	.	1
29140	0	6.82	1	0.77843	.	.	1
29141	0	6.82	1	0.77843	.	.	1
29142	0	6.82	1	0.77843	.	.	1
29143	0	6.82	1	0.77843	.	.	1
29144	0	6.82	1	0.77843	.	.	1
29145	0	6.82	1	0.77843	.	.	1
29146	0	6.82	1	0.77843	.	.	1
29147	0	6.82	1	0.77843	.	.	1
29148	0	6.82	1	0.77843	.	.	1
29149	0	6.82	1	0.77843	.	.	1
29150	0	6.82	1	0.77843	.	.	1
29151	0	6.82	1	0.77843	.	.	1
29152	0	6.82	1	0.77843	.	.	1
29153	0	6.82	1	0.77843	.	.	1
29154	0	6.82	1	0.77843	.	.	1
29155	0	6.82	1	0.77843	.	.	1

Obs	Sub_group	fuptimey	_CENSOR_	SURVIVAL	SDF_LCL	SDF_UCL	STRATUM
29156	0	6.82	1	0.77843	.	.	1
29157	0	6.82	1	0.77843	.	.	1
29158	0	6.82	1	0.77843	.	.	1
29159	0	6.82	1	0.77843	.	.	1
29160	0	6.83	0	0.77821	0.77337	0.78296	1
29161	0	6.83	1	0.77821	.	.	1
29162	0	6.83	1	0.77821	.	.	1
29163	0	6.83	1	0.77821	.	.	1
29164	0	6.83	1	0.77821	.	.	1
29165	0	6.83	1	0.77821	.	.	1
29166	0	6.83	1	0.77821	.	.	1
29167	0	6.83	1	0.77821	.	.	1
29168	0	6.83	1	0.77821	.	.	1
29169	0	6.83	1	0.77821	.	.	1
29170	0	6.83	1	0.77821	.	.	1
29171	0	6.83	1	0.77821	.	.	1
29172	0	6.83	1	0.77821	.	.	1
29173	0	6.83	1	0.77821	.	.	1
29174	0	6.83	1	0.77821	.	.	1
29175	0	6.83	1	0.77821	.	.	1
29176	0	6.83	1	0.77821	.	.	1
29177	0	6.83	1	0.77821	.	.	1
29178	0	6.83	1	0.77821	.	.	1
29179	0	6.83	1	0.77821	.	.	1
29180	0	6.83	1	0.77821	.	.	1
29181	0	6.83	1	0.77821	.	.	1
29182	0	6.83	1	0.77821	.	.	1
29183	0	6.83	1	0.77821	.	.	1
29184	0	6.83	1	0.77821	.	.	1
29185	0	6.83	1	0.77821	.	.	1
29186	0	6.83	1	0.77821	.	.	1
29187	0	6.83	1	0.77821	.	.	1
29188	0	6.83	1	0.77821	.	.	1
29189	0	6.83	1	0.77821	.	.	1
29190	0	6.83	1	0.77821	.	.	1

Obs	Sub_group	fuptimey	_CENSOR_	SURVIVAL	SDF_LCL	SDF_UCL	STRATUM
**29191**	0	6.84	0	0.77813	0.77329	0.78289	1
**29192**	0	6.84	1	0.77813	.	.	1
**29193**	0	6.84	1	0.77813	.	.	1
**29194**	0	6.84	1	0.77813	.	.	1
**29195**	0	6.84	1	0.77813	.	.	1
**29196**	0	6.84	1	0.77813	.	.	1
**29197**	0	6.84	1	0.77813	.	.	1
**29198**	0	6.84	1	0.77813	.	.	1
**29199**	0	6.84	1	0.77813	.	.	1
**29200**	0	6.84	1	0.77813	.	.	1
**29201**	0	6.84	1	0.77813	.	.	1
**29202**	0	6.84	1	0.77813	.	.	1
**29203**	0	6.84	1	0.77813	.	.	1
**29204**	0	6.84	1	0.77813	.	.	1
**29205**	0	6.84	1	0.77813	.	.	1
**29206**	0	6.84	1	0.77813	.	.	1
**29207**	0	6.84	1	0.77813	.	.	1
**29208**	0	6.84	1	0.77813	.	.	1
**29209**	0	6.84	1	0.77813	.	.	1
**29210**	0	6.84	1	0.77813	.	.	1
**29211**	0	6.84	1	0.77813	.	.	1
**29212**	0	6.85	0	0.77806	0.77322	0.78282	1
**29213**	0	6.85	1	0.77806	.	.	1
**29214**	0	6.85	1	0.77806	.	.	1
**29215**	0	6.85	1	0.77806	.	.	1
**29216**	0	6.85	1	0.77806	.	.	1
**29217**	0	6.85	1	0.77806	.	.	1
**29218**	0	6.85	1	0.77806	.	.	1
**29219**	0	6.85	1	0.77806	.	.	1
**29220**	0	6.85	1	0.77806	.	.	1
**29221**	0	6.85	1	0.77806	.	.	1
**29222**	0	6.85	1	0.77806	.	.	1
**29223**	0	6.85	1	0.77806	.	.	1
**29224**	0	6.85	1	0.77806	.	.	1
**29225**	0	6.85	1	0.77806	.	.	1

Obs	Sub_group	fuptimey	_CENSOR_	SURVIVAL	SDF_LCL	SDF_UCL	STRATUM
29226	0	6.85	1	0.77806	.	.	1
29227	0	6.85	1	0.77806	.	.	1
29228	0	6.85	1	0.77806	.	.	1
29229	0	6.85	1	0.77806	.	.	1
29230	0	6.85	1	0.77806	.	.	1
29231	0	6.85	1	0.77806	.	.	1
29232	0	6.85	1	0.77806	.	.	1
29233	0	6.85	1	0.77806	.	.	1
29234	0	6.85	1	0.77806	.	.	1
29235	0	6.85	1	0.77806	.	.	1
29236	0	6.85	1	0.77806	.	.	1
29237	0	6.85	1	0.77806	.	.	1
29238	0	6.85	1	0.77806	.	.	1
29239	0	6.85	1	0.77806	.	.	1
29240	0	6.85	1	0.77806	.	.	1
29241	0	6.85	1	0.77806	.	.	1
29242	0	6.85	1	0.77806	.	.	1
29243	0	6.85	1	0.77806	.	.	1
29244	0	6.85	1	0.77806	.	.	1
29245	0	6.85	1	0.77806	.	.	1
29246	0	6.85	1	0.77806	.	.	1
29247	0	6.85	1	0.77806	.	.	1
29248	0	6.85	1	0.77806	.	.	1
29249	0	6.85	1	0.77806	.	.	1
29250	0	6.85	1	0.77806	.	.	1
29251	0	6.85	1	0.77806	.	.	1
29252	0	6.85	1	0.77806	.	.	1
29253	0	6.85	1	0.77806	.	.	1
29254	0	6.85	1	0.77806	.	.	1
29255	0	6.86	0	0.77799	0.77314	0.78274	1
29256	0	6.86	1	0.77799	.	.	1
29257	0	6.86	1	0.77799	.	.	1
29258	0	6.86	1	0.77799	.	.	1
29259	0	6.86	1	0.77799	.	.	1
29260	0	6.86	1	0.77799	.	.	1

Obs	Sub_group	fuptimey	_CENSOR_	SURVIVAL	SDF_LCL	SDF_UCL	STRATUM
29261	0	6.86	1	0.77799	.	.	1
29262	0	6.86	1	0.77799	.	.	1
29263	0	6.86	1	0.77799	.	.	1
29264	0	6.86	1	0.77799	.	.	1
29265	0	6.86	1	0.77799	.	.	1
29266	0	6.86	1	0.77799	.	.	1
29267	0	6.86	1	0.77799	.	.	1
29268	0	6.86	1	0.77799	.	.	1
29269	0	6.86	1	0.77799	.	.	1
29270	0	6.86	1	0.77799	.	.	1
29271	0	6.86	1	0.77799	.	.	1
29272	0	6.86	1	0.77799	.	.	1
29273	0	6.86	1	0.77799	.	.	1
29274	0	6.86	1	0.77799	.	.	1
29275	0	6.86	1	0.77799	.	.	1
29276	0	6.86	1	0.77799	.	.	1
29277	0	6.86	1	0.77799	.	.	1
29278	0	6.86	1	0.77799	.	.	1
29279	0	6.86	1	0.77799	.	.	1
29280	0	6.86	1	0.77799	.	.	1
29281	0	6.86	1	0.77799	.	.	1
29282	0	6.86	1	0.77799	.	.	1
29283	0	6.86	1	0.77799	.	.	1
29284	0	6.86	1	0.77799	.	.	1
29285	0	6.86	1	0.77799	.	.	1
29286	0	6.86	1	0.77799	.	.	1
29287	0	6.86	1	0.77799	.	.	1
29288	0	6.87	0	0.77784	0.77299	0.78260	1
29289	0	6.87	1	0.77784	.	.	1
29290	0	6.87	1	0.77784	.	.	1
29291	0	6.87	1	0.77784	.	.	1
29292	0	6.87	1	0.77784	.	.	1
29293	0	6.87	1	0.77784	.	.	1
29294	0	6.87	1	0.77784	.	.	1
29295	0	6.87	1	0.77784	.	.	1

Obs	Sub_group	fuptimey	_CENSOR_	SURVIVAL	SDF_LCL	SDF_UCL	STRATUM
29296	0	6.87	1	0.77784	.	.	1
29297	0	6.87	1	0.77784	.	.	1
29298	0	6.87	1	0.77784	.	.	1
29299	0	6.87	1	0.77784	.	.	1
29300	0	6.87	1	0.77784	.	.	1
29301	0	6.87	1	0.77784	.	.	1
29302	0	6.87	1	0.77784	.	.	1
29303	0	6.87	1	0.77784	.	.	1
29304	0	6.87	1	0.77784	.	.	1
29305	0	6.87	1	0.77784	.	.	1
29306	0	6.87	1	0.77784	.	.	1
29307	0	6.87	1	0.77784	.	.	1
29308	0	6.87	1	0.77784	.	.	1
29309	0	6.87	1	0.77784	.	.	1
29310	0	6.87	1	0.77784	.	.	1
29311	0	6.87	1	0.77784	.	.	1
29312	0	6.87	1	0.77784	.	.	1
29313	0	6.87	1	0.77784	.	.	1
29314	0	6.87	1	0.77784	.	.	1
29315	0	6.87	1	0.77784	.	.	1
29316	0	6.87	1	0.77784	.	.	1
29317	0	6.87	1	0.77784	.	.	1
29318	0	6.87	1	0.77784	.	.	1
29319	0	6.87	1	0.77784	.	.	1
29320	0	6.87	1	0.77784	.	.	1
29321	0	6.87	1	0.77784	.	.	1
29322	0	6.87	1	0.77784	.	.	1
29323	0	6.87	1	0.77784	.	.	1
29324	0	6.87	1	0.77784	.	.	1
29325	0	6.87	1	0.77784	.	.	1
29326	0	6.87	1	0.77784	.	.	1
29327	0	6.87	1	0.77784	.	.	1
29328	0	6.87	1	0.77784	.	.	1
29329	0	6.87	1	0.77784	.	.	1
29330	0	6.87	1	0.77784	.	.	1

Obs	Sub_group	fuptimey	_CENSOR_	SURVIVAL	SDF_LCL	SDF_UCL	STRATUM
29331	0	6.87	1	0.77784	.	.	1
29332	0	6.87	1	0.77784	.	.	1
29333	0	6.87	1	0.77784	.	.	1
29334	0	6.88	0	0.77769	0.77283	0.78245	1
29335	0	6.88	1	0.77769	.	.	1
29336	0	6.88	1	0.77769	.	.	1
29337	0	6.88	1	0.77769	.	.	1
29338	0	6.88	1	0.77769	.	.	1
29339	0	6.88	1	0.77769	.	.	1
29340	0	6.88	1	0.77769	.	.	1
29341	0	6.88	1	0.77769	.	.	1
29342	0	6.88	1	0.77769	.	.	1
29343	0	6.88	1	0.77769	.	.	1
29344	0	6.88	1	0.77769	.	.	1
29345	0	6.88	1	0.77769	.	.	1
29346	0	6.88	1	0.77769	.	.	1
29347	0	6.88	1	0.77769	.	.	1
29348	0	6.88	1	0.77769	.	.	1
29349	0	6.88	1	0.77769	.	.	1
29350	0	6.88	1	0.77769	.	.	1
29351	0	6.88	1	0.77769	.	.	1
29352	0	6.88	1	0.77769	.	.	1
29353	0	6.88	1	0.77769	.	.	1
29354	0	6.89	0	0.77746	0.77260	0.78223	1
29355	0	6.89	1	0.77746	.	.	1
29356	0	6.89	1	0.77746	.	.	1
29357	0	6.89	1	0.77746	.	.	1
29358	0	6.89	1	0.77746	.	.	1
29359	0	6.89	1	0.77746	.	.	1
29360	0	6.89	1	0.77746	.	.	1
29361	0	6.89	1	0.77746	.	.	1
29362	0	6.89	1	0.77746	.	.	1
29363	0	6.89	1	0.77746	.	.	1
29364	0	6.89	1	0.77746	.	.	1
29365	0	6.89	1	0.77746	.	.	1

Obs	Sub_group	fuptimey	_CENSOR_	SURVIVAL	SDF_LCL	SDF_UCL	STRATUM
29366	0	6.89	1	0.77746	.	.	1
29367	0	6.89	1	0.77746	.	.	1
29368	0	6.89	1	0.77746	.	.	1
29369	0	6.89	1	0.77746	.	.	1
29370	0	6.89	1	0.77746	.	.	1
29371	0	6.89	1	0.77746	.	.	1
29372	0	6.89	1	0.77746	.	.	1
29373	0	6.89	1	0.77746	.	.	1
29374	0	6.89	1	0.77746	.	.	1
29375	0	6.89	1	0.77746	.	.	1
29376	0	6.89	1	0.77746	.	.	1
29377	0	6.89	1	0.77746	.	.	1
29378	0	6.89	1	0.77746	.	.	1
29379	0	6.89	1	0.77746	.	.	1
29380	0	6.89	1	0.77746	.	.	1
29381	0	6.89	1	0.77746	.	.	1
29382	0	6.89	1	0.77746	.	.	1
29383	0	6.89	1	0.77746	.	.	1
29384	0	6.89	1	0.77746	.	.	1
29385	0	6.89	1	0.77746	.	.	1
29386	0	6.89	1	0.77746	.	.	1
29387	0	6.89	1	0.77746	.	.	1
29388	0	6.89	1	0.77746	.	.	1
29389	0	6.89	1	0.77746	.	.	1
29390	0	6.90	0	0.77731	0.77245	0.78208	1
29391	0	6.90	1	0.77731	.	.	1
29392	0	6.90	1	0.77731	.	.	1
29393	0	6.90	1	0.77731	.	.	1
29394	0	6.90	1	0.77731	.	.	1
29395	0	6.90	1	0.77731	.	.	1
29396	0	6.90	1	0.77731	.	.	1
29397	0	6.90	1	0.77731	.	.	1
29398	0	6.90	1	0.77731	.	.	1
29399	0	6.90	1	0.77731	.	.	1
29400	0	6.90	1	0.77731	.	.	1

Obs	Sub_group	fuptimey	_CENSOR_	SURVIVAL	SDF_LCL	SDF_UCL	STRATUM
29401	0	6.90	1	0.77731	.	.	1
29402	0	6.90	1	0.77731	.	.	1
29403	0	6.90	1	0.77731	.	.	1
29404	0	6.90	1	0.77731	.	.	1
29405	0	6.90	1	0.77731	.	.	1
29406	0	6.90	1	0.77731	.	.	1
29407	0	6.90	1	0.77731	.	.	1
29408	0	6.90	1	0.77731	.	.	1
29409	0	6.90	1	0.77731	.	.	1
29410	0	6.90	1	0.77731	.	.	1
29411	0	6.90	1	0.77731	.	.	1
29412	0	6.90	1	0.77731	.	.	1
29413	0	6.91	0	0.77708	0.77221	0.78186	1
29414	0	6.91	1	0.77708	.	.	1
29415	0	6.91	1	0.77708	.	.	1
29416	0	6.91	1	0.77708	.	.	1
29417	0	6.91	1	0.77708	.	.	1
29418	0	6.91	1	0.77708	.	.	1
29419	0	6.91	1	0.77708	.	.	1
29420	0	6.91	1	0.77708	.	.	1
29421	0	6.91	1	0.77708	.	.	1
29422	0	6.91	1	0.77708	.	.	1
29423	0	6.91	1	0.77708	.	.	1
29424	0	6.91	1	0.77708	.	.	1
29425	0	6.91	1	0.77708	.	.	1
29426	0	6.91	1	0.77708	.	.	1
29427	0	6.91	1	0.77708	.	.	1
29428	0	6.91	1	0.77708	.	.	1
29429	0	6.91	1	0.77708	.	.	1
29430	0	6.91	1	0.77708	.	.	1
29431	0	6.91	1	0.77708	.	.	1
29432	0	6.91	1	0.77708	.	.	1
29433	0	6.91	1	0.77708	.	.	1
29434	0	6.91	1	0.77708	.	.	1
29435	0	6.91	1	0.77708	.	.	1

Obs	Sub_group	fuptimey	_CENSOR_	SURVIVAL	SDF_LCL	SDF_UCL	STRATUM
29436	0	6.91	1	0.77708	.	.	1
29437	0	6.91	1	0.77708	.	.	1
29438	0	6.91	1	0.77708	.	.	1
29439	0	6.91	1	0.77708	.	.	1
29440	0	6.91	1	0.77708	.	.	1
29441	0	6.91	1	0.77708	.	.	1
29442	0	6.91	1	0.77708	.	.	1
29443	0	6.91	1	0.77708	.	.	1
29444	0	6.91	1	0.77708	.	.	1
29445	0	6.91	1	0.77708	.	.	1
29446	0	6.91	1	0.77708	.	.	1
29447	0	6.91	1	0.77708	.	.	1
29448	0	6.91	1	0.77708	.	.	1
29449	0	6.91	1	0.77708	.	.	1
29450	0	6.91	1	0.77708	.	.	1
29451	0	6.92	0	0.77685	0.77198	0.78164	1
29452	0	6.92	1	0.77685	.	.	1
29453	0	6.92	1	0.77685	.	.	1
29454	0	6.92	1	0.77685	.	.	1
29455	0	6.92	1	0.77685	.	.	1
29456	0	6.92	1	0.77685	.	.	1
29457	0	6.92	1	0.77685	.	.	1
29458	0	6.92	1	0.77685	.	.	1
29459	0	6.92	1	0.77685	.	.	1
29460	0	6.92	1	0.77685	.	.	1
29461	0	6.92	1	0.77685	.	.	1
29462	0	6.92	1	0.77685	.	.	1
29463	0	6.92	1	0.77685	.	.	1
29464	0	6.92	1	0.77685	.	.	1
29465	0	6.92	1	0.77685	.	.	1
29466	0	6.92	1	0.77685	.	.	1
29467	0	6.92	1	0.77685	.	.	1
29468	0	6.92	1	0.77685	.	.	1
29469	0	6.92	1	0.77685	.	.	1
29470	0	6.92	1	0.77685	.	.	1

Obs	Sub_group	fuptimey	_CENSOR_	SURVIVAL	SDF_LCL	SDF_UCL	STRATUM
**29471**	0	6.92	1	0.77685	.	.	1
**29472**	0	6.92	1	0.77685	.	.	1
**29473**	0	6.92	1	0.77685	.	.	1
**29474**	0	6.92	1	0.77685	.	.	1
**29475**	0	6.92	1	0.77685	.	.	1
**29476**	0	6.92	1	0.77685	.	.	1
**29477**	0	6.92	1	0.77685	.	.	1
**29478**	0	6.92	1	0.77685	.	.	1
**29479**	0	6.92	1	0.77685	.	.	1
**29480**	0	6.93	0	0.77662	0.77174	0.78141	1
**29481**	0	6.93	1	0.77662	.	.	1
**29482**	0	6.93	1	0.77662	.	.	1
**29483**	0	6.93	1	0.77662	.	.	1
**29484**	0	6.93	1	0.77662	.	.	1
**29485**	0	6.93	1	0.77662	.	.	1
**29486**	0	6.93	1	0.77662	.	.	1
**29487**	0	6.93	1	0.77662	.	.	1
**29488**	0	6.93	1	0.77662	.	.	1
**29489**	0	6.93	1	0.77662	.	.	1
**29490**	0	6.93	1	0.77662	.	.	1
**29491**	0	6.93	1	0.77662	.	.	1
**29492**	0	6.93	1	0.77662	.	.	1
**29493**	0	6.93	1	0.77662	.	.	1
**29494**	0	6.93	1	0.77662	.	.	1
**29495**	0	6.93	1	0.77662	.	.	1
**29496**	0	6.93	1	0.77662	.	.	1
**29497**	0	6.93	1	0.77662	.	.	1
**29498**	0	6.93	1	0.77662	.	.	1
**29499**	0	6.93	1	0.77662	.	.	1
**29500**	0	6.93	1	0.77662	.	.	1
**29501**	0	6.93	1	0.77662	.	.	1
**29502**	0	6.93	1	0.77662	.	.	1
**29503**	0	6.93	1	0.77662	.	.	1
**29504**	0	6.93	1	0.77662	.	.	1
**29505**	0	6.93	1	0.77662	.	.	1

Obs	Sub_group	fuptimey	_CENSOR_	SURVIVAL	SDF_LCL	SDF_UCL	STRATUM
29506	0	6.93	1	0.77662	.	.	1
29507	0	6.93	1	0.77662	.	.	1
29508	0	6.93	1	0.77662	.	.	1
29509	0	6.93	1	0.77662	.	.	1
29510	0	6.93	1	0.77662	.	.	1
29511	0	6.93	1	0.77662	.	.	1
29512	0	6.93	1	0.77662	.	.	1
29513	0	6.93	1	0.77662	.	.	1
29514	0	6.93	1	0.77662	.	.	1
29515	0	6.93	1	0.77662	.	.	1
29516	0	6.93	1	0.77662	.	.	1
29517	0	6.93	1	0.77662	.	.	1
29518	0	6.93	1	0.77662	.	.	1
29519	0	6.93	1	0.77662	.	.	1
29520	0	6.94	0	0.77647	0.77159	0.78126	1
29521	0	6.94	1	0.77647	.	.	1
29522	0	6.94	1	0.77647	.	.	1
29523	0	6.94	1	0.77647	.	.	1
29524	0	6.94	1	0.77647	.	.	1
29525	0	6.94	1	0.77647	.	.	1
29526	0	6.94	1	0.77647	.	.	1
29527	0	6.94	1	0.77647	.	.	1
29528	0	6.94	1	0.77647	.	.	1
29529	0	6.94	1	0.77647	.	.	1
29530	0	6.94	1	0.77647	.	.	1
29531	0	6.94	1	0.77647	.	.	1
29532	0	6.94	1	0.77647	.	.	1
29533	0	6.94	1	0.77647	.	.	1
29534	0	6.94	1	0.77647	.	.	1
29535	0	6.94	1	0.77647	.	.	1
29536	0	6.94	1	0.77647	.	.	1
29537	0	6.94	1	0.77647	.	.	1
29538	0	6.94	1	0.77647	.	.	1
29539	0	6.94	1	0.77647	.	.	1
29540	0	6.94	1	0.77647	.	.	1

Obs	Sub_group	fuptimey	_CENSOR_	SURVIVAL	SDF_LCL	SDF_UCL	STRATUM
29541	0	6.94	1	0.77647	.	.	1
29542	0	6.94	1	0.77647	.	.	1
29543	0	6.94	1	0.77647	.	.	1
29544	0	6.94	1	0.77647	.	.	1
29545	0	6.94	1	0.77647	.	.	1
29546	0	6.94	1	0.77647	.	.	1
29547	0	6.94	1	0.77647	.	.	1
29548	0	6.94	1	0.77647	.	.	1
29549	0	6.94	1	0.77647	.	.	1
29550	0	6.94	1	0.77647	.	.	1
29551	0	6.94	1	0.77647	.	.	1
29552	0	6.94	1	0.77647	.	.	1
29553	0	6.94	1	0.77647	.	.	1
29554	0	6.94	1	0.77647	.	.	1
29555	0	6.95	0	0.77639	0.77151	0.78119	1
29556	0	6.95	1	0.77639	.	.	1
29557	0	6.95	1	0.77639	.	.	1
29558	0	6.95	1	0.77639	.	.	1
29559	0	6.95	1	0.77639	.	.	1
29560	0	6.95	1	0.77639	.	.	1
29561	0	6.95	1	0.77639	.	.	1
29562	0	6.95	1	0.77639	.	.	1
29563	0	6.95	1	0.77639	.	.	1
29564	0	6.95	1	0.77639	.	.	1
29565	0	6.95	1	0.77639	.	.	1
29566	0	6.95	1	0.77639	.	.	1
29567	0	6.95	1	0.77639	.	.	1
29568	0	6.95	1	0.77639	.	.	1
29569	0	6.95	1	0.77639	.	.	1
29570	0	6.95	1	0.77639	.	.	1
29571	0	6.95	1	0.77639	.	.	1
29572	0	6.95	1	0.77639	.	.	1
29573	0	6.95	1	0.77639	.	.	1
29574	0	6.95	1	0.77639	.	.	1
29575	0	6.95	1	0.77639	.	.	1

Obs	Sub_group	fuptimey	_CENSOR_	SURVIVAL	SDF_LCL	SDF_UCL	STRATUM
29576	0	6.95	1	0.77639	.	.	1
29577	0	6.95	1	0.77639	.	.	1
29578	0	6.95	1	0.77639	.	.	1
29579	0	6.95	1	0.77639	.	.	1
29580	0	6.95	1	0.77639	.	.	1
29581	0	6.95	1	0.77639	.	.	1
29582	0	6.95	1	0.77639	.	.	1
29583	0	6.95	1	0.77639	.	.	1
29584	0	6.95	1	0.77639	.	.	1
29585	0	6.96	0	0.77624	0.77135	0.78104	1
29586	0	6.96	1	0.77624	.	.	1
29587	0	6.96	1	0.77624	.	.	1
29588	0	6.96	1	0.77624	.	.	1
29589	0	6.96	1	0.77624	.	.	1
29590	0	6.96	1	0.77624	.	.	1
29591	0	6.96	1	0.77624	.	.	1
29592	0	6.96	1	0.77624	.	.	1
29593	0	6.96	1	0.77624	.	.	1
29594	0	6.96	1	0.77624	.	.	1
29595	0	6.96	1	0.77624	.	.	1
29596	0	6.96	1	0.77624	.	.	1
29597	0	6.96	1	0.77624	.	.	1
29598	0	6.96	1	0.77624	.	.	1
29599	0	6.96	1	0.77624	.	.	1
29600	0	6.96	1	0.77624	.	.	1
29601	0	6.96	1	0.77624	.	.	1
29602	0	6.96	1	0.77624	.	.	1
29603	0	6.96	1	0.77624	.	.	1
29604	0	6.96	1	0.77624	.	.	1
29605	0	6.96	1	0.77624	.	.	1
29606	0	6.96	1	0.77624	.	.	1
29607	0	6.96	1	0.77624	.	.	1
29608	0	6.96	1	0.77624	.	.	1
29609	0	6.96	1	0.77624	.	.	1
29610	0	6.97	0	0.77601	0.77111	0.78081	1

Obs	Sub_group	fuptimey	_CENSOR_	SURVIVAL	SDF_LCL	SDF_UCL	STRATUM
29611	0	6.97	1	0.77601	.	.	1
29612	0	6.97	1	0.77601	.	.	1
29613	0	6.97	1	0.77601	.	.	1
29614	0	6.97	1	0.77601	.	.	1
29615	0	6.97	1	0.77601	.	.	1
29616	0	6.97	1	0.77601	.	.	1
29617	0	6.97	1	0.77601	.	.	1
29618	0	6.97	1	0.77601	.	.	1
29619	0	6.97	1	0.77601	.	.	1
29620	0	6.97	1	0.77601	.	.	1
29621	0	6.97	1	0.77601	.	.	1
29622	0	6.97	1	0.77601	.	.	1
29623	0	6.97	1	0.77601	.	.	1
29624	0	6.97	1	0.77601	.	.	1
29625	0	6.97	1	0.77601	.	.	1
29626	0	6.97	1	0.77601	.	.	1
29627	0	6.97	1	0.77601	.	.	1
29628	0	6.97	1	0.77601	.	.	1
29629	0	6.97	1	0.77601	.	.	1
29630	0	6.97	1	0.77601	.	.	1
29631	0	6.97	1	0.77601	.	.	1
29632	0	6.97	1	0.77601	.	.	1
29633	0	6.97	1	0.77601	.	.	1
29634	0	6.97	1	0.77601	.	.	1
29635	0	6.97	1	0.77601	.	.	1
29636	0	6.97	1	0.77601	.	.	1
29637	0	6.97	1	0.77601	.	.	1
29638	0	6.97	1	0.77601	.	.	1
29639	0	6.97	1	0.77601	.	.	1
29640	0	6.97	1	0.77601	.	.	1
29641	0	6.98	0	0.77570	0.77080	0.78051	1
29642	0	6.98	1	0.77570	.	.	1
29643	0	6.98	1	0.77570	.	.	1
29644	0	6.98	1	0.77570	.	.	1
29645	0	6.98	1	0.77570	.	.	1

Obs	Sub_group	fuptimey	_CENSOR_	SURVIVAL	SDF_LCL	SDF_UCL	STRATUM
29646	0	6.98	1	0.77570	.	.	1
29647	0	6.98	1	0.77570	.	.	1
29648	0	6.98	1	0.77570	.	.	1
29649	0	6.98	1	0.77570	.	.	1
29650	0	6.98	1	0.77570	.	.	1
29651	0	6.98	1	0.77570	.	.	1
29652	0	6.98	1	0.77570	.	.	1
29653	0	6.98	1	0.77570	.	.	1
29654	0	6.98	1	0.77570	.	.	1
29655	0	6.98	1	0.77570	.	.	1
29656	0	6.98	1	0.77570	.	.	1
29657	0	6.98	1	0.77570	.	.	1
29658	0	6.98	1	0.77570	.	.	1
29659	0	6.98	1	0.77570	.	.	1
29660	0	6.98	1	0.77570	.	.	1
29661	0	6.98	1	0.77570	.	.	1
29662	0	6.98	1	0.77570	.	.	1
29663	0	6.98	1	0.77570	.	.	1
29664	0	6.98	1	0.77570	.	.	1
29665	0	6.99	0	0.77539	0.77048	0.78021	1
29666	0	6.99	1	0.77539	.	.	1
29667	0	6.99	1	0.77539	.	.	1
29668	0	6.99	1	0.77539	.	.	1
29669	0	6.99	1	0.77539	.	.	1
29670	0	6.99	1	0.77539	.	.	1
29671	0	6.99	1	0.77539	.	.	1
29672	0	6.99	1	0.77539	.	.	1
29673	0	6.99	1	0.77539	.	.	1
29674	0	6.99	1	0.77539	.	.	1
29675	0	6.99	1	0.77539	.	.	1
29676	0	6.99	1	0.77539	.	.	1
29677	0	6.99	1	0.77539	.	.	1
29678	0	6.99	1	0.77539	.	.	1
29679	0	6.99	1	0.77539	.	.	1
29680	0	6.99	1	0.77539	.	.	1

Obs	Sub_group	fuptimey	_CENSOR_	SURVIVAL	SDF_LCL	SDF_UCL	STRATUM
29681	0	6.99	1	0.77539	.	.	1
29682	0	6.99	1	0.77539	.	.	1
29683	0	6.99	1	0.77539	.	.	1
29684	0	6.99	1	0.77539	.	.	1
29685	0	6.99	1	0.77539	.	.	1
29686	0	6.99	1	0.77539	.	.	1
29687	0	6.99	1	0.77539	.	.	1
29688	0	6.99	1	0.77539	.	.	1
29689	0	6.99	1	0.77539	.	.	1
29690	0	6.99	1	0.77539	.	.	1
29691	0	6.99	1	0.77539	.	.	1
29692	0	6.99	1	0.77539	.	.	1
29693	0	6.99	1	0.77539	.	.	1
29694	0	6.99	1	0.77539	.	.	1
29695	0	6.99	1	0.77539	.	.	1
29696	0	7.00	0	0.77523	0.77032	0.78006	1
29697	0	7.00	1	0.77523	.	.	1
29698	0	7.00	1	0.77523	.	.	1
29699	0	7.00	1	0.77523	.	.	1
29700	0	7.00	1	0.77523	.	.	1
29701	0	7.00	1	0.77523	.	.	1
29702	0	7.00	1	0.77523	.	.	1
29703	0	7.00	1	0.77523	.	.	1
29704	0	7.00	1	0.77523	.	.	1
29705	0	7.00	1	0.77523	.	.	1
29706	0	7.00	1	0.77523	.	.	1
29707	0	7.00	1	0.77523	.	.	1
29708	0	7.00	1	0.77523	.	.	1
29709	0	7.00	1	0.77523	.	.	1
29710	0	7.00	1	0.77523	.	.	1
29711	0	7.00	1	0.77523	.	.	1
29712	0	7.00	1	0.77523	.	.	1
29713	0	7.00	1	0.77523	.	.	1
29714	0	7.00	1	0.77523	.	.	1
29715	0	7.00	1	0.77523	.	.	1

Obs	Sub_group	fuptimey	_CENSOR_	SURVIVAL	SDF_LCL	SDF_UCL	STRATUM
**29716**	0	7.00	1	0.77523	.	.	1
**29717**	0	7.00	1	0.77523	.	.	1
**29718**	0	7.00	1	0.77523	.	.	1
**29719**	0	7.00	1	0.77523	.	.	1
**29720**	0	7.00	1	0.77523	.	.	1
**29721**	0	7.00	1	0.77523	.	.	1
**29722**	0	7.00	1	0.77523	.	.	1
**29723**	0	7.00	1	0.77523	.	.	1
**29724**	0	7.00	1	0.77523	.	.	1
**29725**	0	7.00	1	0.77523	.	.	1
**29726**	0	7.00	1	0.77523	.	.	1
**29727**	0	7.00	1	0.77523	.	.	1
**29728**	0	7.00	1	0.77523	.	.	1
**29729**	0	7.00	1	0.77523	.	.	1
**29730**	0	7.01	0	0.77515	0.77024	0.77998	1
**29731**	0	7.01	1	0.77515	.	.	1
**29732**	0	7.01	1	0.77515	.	.	1
**29733**	0	7.01	1	0.77515	.	.	1
**29734**	0	7.01	1	0.77515	.	.	1
**29735**	0	7.01	1	0.77515	.	.	1
**29736**	0	7.01	1	0.77515	.	.	1
**29737**	0	7.01	1	0.77515	.	.	1
**29738**	0	7.01	1	0.77515	.	.	1
**29739**	0	7.01	1	0.77515	.	.	1
**29740**	0	7.01	1	0.77515	.	.	1
**29741**	0	7.01	1	0.77515	.	.	1
**29742**	0	7.01	1	0.77515	.	.	1
**29743**	0	7.01	1	0.77515	.	.	1
**29744**	0	7.01	1	0.77515	.	.	1
**29745**	0	7.01	1	0.77515	.	.	1
**29746**	0	7.01	1	0.77515	.	.	1
**29747**	0	7.01	1	0.77515	.	.	1
**29748**	0	7.01	1	0.77515	.	.	1
**29749**	0	7.01	1	0.77515	.	.	1
**29750**	0	7.01	1	0.77515	.	.	1

Obs	Sub_group	fuptimey	_CENSOR_	SURVIVAL	SDF_LCL	SDF_UCL	STRATUM
29751	0	7.01	1	0.77515	.	.	1
29752	0	7.01	1	0.77515	.	.	1
29753	0	7.01	1	0.77515	.	.	1
29754	0	7.01	1	0.77515	.	.	1
29755	0	7.01	1	0.77515	.	.	1
29756	0	7.01	1	0.77515	.	.	1
29757	0	7.01	1	0.77515	.	.	1
29758	0	7.01	1	0.77515	.	.	1
29759	0	7.01	1	0.77515	.	.	1
29760	0	7.01	1	0.77515	.	.	1
29761	0	7.02	0	0.77500	0.77008	0.77983	1
29762	0	7.02	1	0.77500	.	.	1
29763	0	7.02	1	0.77500	.	.	1
29764	0	7.02	1	0.77500	.	.	1
29765	0	7.02	1	0.77500	.	.	1
29766	0	7.02	1	0.77500	.	.	1
29767	0	7.02	1	0.77500	.	.	1
29768	0	7.02	1	0.77500	.	.	1
29769	0	7.02	1	0.77500	.	.	1
29770	0	7.02	1	0.77500	.	.	1
29771	0	7.02	1	0.77500	.	.	1
29772	0	7.02	1	0.77500	.	.	1
29773	0	7.02	1	0.77500	.	.	1
29774	0	7.02	1	0.77500	.	.	1
29775	0	7.02	1	0.77500	.	.	1
29776	0	7.02	1	0.77500	.	.	1
29777	0	7.02	1	0.77500	.	.	1
29778	0	7.02	1	0.77500	.	.	1
29779	0	7.02	1	0.77500	.	.	1
29780	0	7.02	1	0.77500	.	.	1
29781	0	7.02	1	0.77500	.	.	1
29782	0	7.02	1	0.77500	.	.	1
29783	0	7.02	1	0.77500	.	.	1
29784	0	7.02	1	0.77500	.	.	1
29785	0	7.02	1	0.77500	.	.	1

Obs	Sub_group	fuptimey	_CENSOR_	SURVIVAL	SDF_LCL	SDF_UCL	STRATUM
**29786**	0	7.02	1	0.77500	.	.	1
**29787**	0	7.02	1	0.77500	.	.	1
**29788**	0	7.02	1	0.77500	.	.	1
**29789**	0	7.02	1	0.77500	.	.	1
**29790**	0	7.03	0	0.77468	0.76975	0.77952	1
**29791**	0	7.03	1	0.77468	.	.	1
**29792**	0	7.03	1	0.77468	.	.	1
**29793**	0	7.03	1	0.77468	.	.	1
**29794**	0	7.03	1	0.77468	.	.	1
**29795**	0	7.03	1	0.77468	.	.	1
**29796**	0	7.03	1	0.77468	.	.	1
**29797**	0	7.03	1	0.77468	.	.	1
**29798**	0	7.03	1	0.77468	.	.	1
**29799**	0	7.03	1	0.77468	.	.	1
**29800**	0	7.03	1	0.77468	.	.	1
**29801**	0	7.03	1	0.77468	.	.	1
**29802**	0	7.03	1	0.77468	.	.	1
**29803**	0	7.03	1	0.77468	.	.	1
**29804**	0	7.03	1	0.77468	.	.	1
**29805**	0	7.03	1	0.77468	.	.	1
**29806**	0	7.03	1	0.77468	.	.	1
**29807**	0	7.03	1	0.77468	.	.	1
**29808**	0	7.03	1	0.77468	.	.	1
**29809**	0	7.03	1	0.77468	.	.	1
**29810**	0	7.03	1	0.77468	.	.	1
**29811**	0	7.03	1	0.77468	.	.	1
**29812**	0	7.03	1	0.77468	.	.	1
**29813**	0	7.03	1	0.77468	.	.	1
**29814**	0	7.03	1	0.77468	.	.	1
**29815**	0	7.03	1	0.77468	.	.	1
**29816**	0	7.03	1	0.77468	.	.	1
**29817**	0	7.03	1	0.77468	.	.	1
**29818**	0	7.03	1	0.77468	.	.	1
**29819**	0	7.04	0	0.77413	0.76919	0.77898	1
**29820**	0	7.04	1	0.77413	.	.	1

Obs	Sub_group	fuptimey	_CENSOR_	SURVIVAL	SDF_LCL	SDF_UCL	STRATUM
29821	0	7.04	1	0.77413	.	.	1
29822	0	7.04	1	0.77413	.	.	1
29823	0	7.04	1	0.77413	.	.	1
29824	0	7.04	1	0.77413	.	.	1
29825	0	7.04	1	0.77413	.	.	1
29826	0	7.04	1	0.77413	.	.	1
29827	0	7.04	1	0.77413	.	.	1
29828	0	7.04	1	0.77413	.	.	1
29829	0	7.04	1	0.77413	.	.	1
29830	0	7.04	1	0.77413	.	.	1
29831	0	7.04	1	0.77413	.	.	1
29832	0	7.04	1	0.77413	.	.	1
29833	0	7.04	1	0.77413	.	.	1
29834	0	7.04	1	0.77413	.	.	1
29835	0	7.04	1	0.77413	.	.	1
29836	0	7.04	1	0.77413	.	.	1
29837	0	7.04	1	0.77413	.	.	1
29838	0	7.04	1	0.77413	.	.	1
29839	0	7.04	1	0.77413	.	.	1
29840	0	7.04	1	0.77413	.	.	1
29841	0	7.04	1	0.77413	.	.	1
29842	0	7.04	1	0.77413	.	.	1
29843	0	7.04	1	0.77413	.	.	1
29844	0	7.04	1	0.77413	.	.	1
29845	0	7.04	1	0.77413	.	.	1
29846	0	7.04	1	0.77413	.	.	1
29847	0	7.05	0	0.77373	0.76878	0.77860	1
29848	0	7.05	1	0.77373	.	.	1
29849	0	7.05	1	0.77373	.	.	1
29850	0	7.05	1	0.77373	.	.	1
29851	0	7.05	1	0.77373	.	.	1
29852	0	7.05	1	0.77373	.	.	1
29853	0	7.05	1	0.77373	.	.	1
29854	0	7.05	1	0.77373	.	.	1
29855	0	7.05	1	0.77373	.	.	1

Obs	Sub_group	fuptimey	_CENSOR_	SURVIVAL	SDF_LCL	SDF_UCL	STRATUM
29856	0	7.05	1	0.77373	.	.	1
29857	0	7.05	1	0.77373	.	.	1
29858	0	7.05	1	0.77373	.	.	1
29859	0	7.05	1	0.77373	.	.	1
29860	0	7.05	1	0.77373	.	.	1
29861	0	7.05	1	0.77373	.	.	1
29862	0	7.05	1	0.77373	.	.	1
29863	0	7.05	1	0.77373	.	.	1
29864	0	7.05	1	0.77373	.	.	1
29865	0	7.05	1	0.77373	.	.	1
29866	0	7.05	1	0.77373	.	.	1
29867	0	7.05	1	0.77373	.	.	1
29868	0	7.05	1	0.77373	.	.	1
29869	0	7.05	1	0.77373	.	.	1
29870	0	7.05	1	0.77373	.	.	1
29871	0	7.06	0	0.77350	0.76854	0.77836	1
29872	0	7.06	1	0.77350	.	.	1
29873	0	7.06	1	0.77350	.	.	1
29874	0	7.06	1	0.77350	.	.	1
29875	0	7.06	1	0.77350	.	.	1
29876	0	7.06	1	0.77350	.	.	1
29877	0	7.06	1	0.77350	.	.	1
29878	0	7.06	1	0.77350	.	.	1
29879	0	7.06	1	0.77350	.	.	1
29880	0	7.06	1	0.77350	.	.	1
29881	0	7.06	1	0.77350	.	.	1
29882	0	7.06	1	0.77350	.	.	1
29883	0	7.06	1	0.77350	.	.	1
29884	0	7.06	1	0.77350	.	.	1
29885	0	7.06	1	0.77350	.	.	1
29886	0	7.06	1	0.77350	.	.	1
29887	0	7.06	1	0.77350	.	.	1
29888	0	7.06	1	0.77350	.	.	1
29889	0	7.06	1	0.77350	.	.	1
29890	0	7.06	1	0.77350	.	.	1

Obs	Sub_group	fuptimey	_CENSOR_	SURVIVAL	SDF_LCL	SDF_UCL	STRATUM
29891	0	7.06	1	0.77350	.	.	1
29892	0	7.06	1	0.77350	.	.	1
29893	0	7.06	1	0.77350	.	.	1
29894	0	7.06	1	0.77350	.	.	1
29895	0	7.06	1	0.77350	.	.	1
29896	0	7.06	1	0.77350	.	.	1
29897	0	7.06	1	0.77350	.	.	1
29898	0	7.06	1	0.77350	.	.	1
29899	0	7.06	1	0.77350	.	.	1
29900	0	7.06	1	0.77350	.	.	1
29901	0	7.06	1	0.77350	.	.	1
29902	0	7.06	1	0.77350	.	.	1
29903	0	7.06	1	0.77350	.	.	1
29904	0	7.07	0	0.77318	0.76821	0.77805	1
29905	0	7.07	1	0.77318	.	.	1
29906	0	7.07	1	0.77318	.	.	1
29907	0	7.07	1	0.77318	.	.	1
29908	0	7.07	1	0.77318	.	.	1
29909	0	7.07	1	0.77318	.	.	1
29910	0	7.07	1	0.77318	.	.	1
29911	0	7.07	1	0.77318	.	.	1
29912	0	7.07	1	0.77318	.	.	1
29913	0	7.07	1	0.77318	.	.	1
29914	0	7.07	1	0.77318	.	.	1
29915	0	7.07	1	0.77318	.	.	1
29916	0	7.07	1	0.77318	.	.	1
29917	0	7.07	1	0.77318	.	.	1
29918	0	7.07	1	0.77318	.	.	1
29919	0	7.07	1	0.77318	.	.	1
29920	0	7.07	1	0.77318	.	.	1
29921	0	7.07	1	0.77318	.	.	1
29922	0	7.07	1	0.77318	.	.	1
29923	0	7.07	1	0.77318	.	.	1
29924	0	7.07	1	0.77318	.	.	1
29925	0	7.07	1	0.77318	.	.	1

Obs	Sub_group	fuptimey	_CENSOR_	SURVIVAL	SDF_LCL	SDF_UCL	STRATUM
29926	0	7.07	1	0.77318	.	.	1
29927	0	7.07	1	0.77318	.	.	1
29928	0	7.07	1	0.77318	.	.	1
29929	0	7.07	1	0.77318	.	.	1
29930	0	7.07	1	0.77318	.	.	1
29931	0	7.07	1	0.77318	.	.	1
29932	0	7.07	1	0.77318	.	.	1
29933	0	7.07	1	0.77318	.	.	1
29934	0	7.08	0	0.77302	0.76805	0.77790	1
29935	0	7.08	1	0.77302	.	.	1
29936	0	7.08	1	0.77302	.	.	1
29937	0	7.08	1	0.77302	.	.	1
29938	0	7.08	1	0.77302	.	.	1
29939	0	7.08	1	0.77302	.	.	1
29940	0	7.08	1	0.77302	.	.	1
29941	0	7.08	1	0.77302	.	.	1
29942	0	7.08	1	0.77302	.	.	1
29943	0	7.08	1	0.77302	.	.	1
29944	0	7.08	1	0.77302	.	.	1
29945	0	7.08	1	0.77302	.	.	1
29946	0	7.08	1	0.77302	.	.	1
29947	0	7.08	1	0.77302	.	.	1
29948	0	7.08	1	0.77302	.	.	1
29949	0	7.08	1	0.77302	.	.	1
29950	0	7.08	1	0.77302	.	.	1
29951	0	7.08	1	0.77302	.	.	1
29952	0	7.08	1	0.77302	.	.	1
29953	0	7.08	1	0.77302	.	.	1
29954	0	7.08	1	0.77302	.	.	1
29955	0	7.08	1	0.77302	.	.	1
29956	0	7.08	1	0.77302	.	.	1
29957	0	7.08	1	0.77302	.	.	1
29958	0	7.08	1	0.77302	.	.	1
29959	0	7.08	1	0.77302	.	.	1
29960	0	7.08	1	0.77302	.	.	1

Obs	Sub_group	fuptimey	_CENSOR_	SURVIVAL	SDF_LCL	SDF_UCL	STRATUM
29961	0	7.08	1	0.77302	.	.	1
29962	0	7.08	1	0.77302	.	.	1
29963	0	7.08	1	0.77302	.	.	1
29964	0	7.08	1	0.77302	.	.	1
29965	0	7.08	1	0.77302	.	.	1
29966	0	7.08	1	0.77302	.	.	1
29967	0	7.08	1	0.77302	.	.	1
29968	0	7.08	1	0.77302	.	.	1
29969	0	7.08	1	0.77302	.	.	1
29970	0	7.08	1	0.77302	.	.	1
29971	0	7.08	1	0.77302	.	.	1
29972	0	7.08	1	0.77302	.	.	1
29973	0	7.08	1	0.77302	.	.	1
29974	0	7.08	1	0.77302	.	.	1
29975	0	7.08	1	0.77302	.	.	1
29976	0	7.08	1	0.77302	.	.	1
29977	0	7.08	1	0.77302	.	.	1
29978	0	7.09	0	0.77270	0.76772	0.77759	1
29979	0	7.09	1	0.77270	.	.	1
29980	0	7.09	1	0.77270	.	.	1
29981	0	7.09	1	0.77270	.	.	1
29982	0	7.09	1	0.77270	.	.	1
29983	0	7.09	1	0.77270	.	.	1
29984	0	7.09	1	0.77270	.	.	1
29985	0	7.09	1	0.77270	.	.	1
29986	0	7.09	1	0.77270	.	.	1
29987	0	7.09	1	0.77270	.	.	1
29988	0	7.09	1	0.77270	.	.	1
29989	0	7.09	1	0.77270	.	.	1
29990	0	7.09	1	0.77270	.	.	1
29991	0	7.09	1	0.77270	.	.	1
29992	0	7.09	1	0.77270	.	.	1
29993	0	7.09	1	0.77270	.	.	1
29994	0	7.09	1	0.77270	.	.	1
29995	0	7.09	1	0.77270	.	.	1

Obs	Sub_group	fuptimey	_CENSOR_	SURVIVAL	SDF_LCL	SDF_UCL	STRATUM
29996	0	7.09	1	0.77270	.	.	1
29997	0	7.09	1	0.77270	.	.	1
29998	0	7.09	1	0.77270	.	.	1
29999	0	7.09	1	0.77270	.	.	1
30000	0	7.09	1	0.77270	.	.	1
30001	0	7.10	0	0.77246	0.76748	0.77735	1
30002	0	7.10	1	0.77246	.	.	1
30003	0	7.10	1	0.77246	.	.	1
30004	0	7.10	1	0.77246	.	.	1
30005	0	7.10	1	0.77246	.	.	1
30006	0	7.10	1	0.77246	.	.	1
30007	0	7.10	1	0.77246	.	.	1
30008	0	7.10	1	0.77246	.	.	1
30009	0	7.10	1	0.77246	.	.	1
30010	0	7.10	1	0.77246	.	.	1
30011	0	7.10	1	0.77246	.	.	1
30012	0	7.10	1	0.77246	.	.	1
30013	0	7.10	1	0.77246	.	.	1
30014	0	7.10	1	0.77246	.	.	1
30015	0	7.10	1	0.77246	.	.	1
30016	0	7.10	1	0.77246	.	.	1
30017	0	7.10	1	0.77246	.	.	1
30018	0	7.10	1	0.77246	.	.	1
30019	0	7.10	1	0.77246	.	.	1
30020	0	7.10	1	0.77246	.	.	1
30021	0	7.10	1	0.77246	.	.	1
30022	0	7.10	1	0.77246	.	.	1
30023	0	7.10	1	0.77246	.	.	1
30024	0	7.10	1	0.77246	.	.	1
30025	0	7.10	1	0.77246	.	.	1
30026	0	7.10	1	0.77246	.	.	1
30027	0	7.10	1	0.77246	.	.	1
30028	0	7.10	1	0.77246	.	.	1
30029	0	7.10	1	0.77246	.	.	1
30030	0	7.10	1	0.77246	.	.	1

Obs	Sub_group	fuptimey	_CENSOR_	SURVIVAL	SDF_LCL	SDF_UCL	STRATUM
30031	0	7.10	1	0.77246	.	.	1
30032	0	7.10	1	0.77246	.	.	1
30033	0	7.10	1	0.77246	.	.	1
30034	0	7.10	1	0.77246	.	.	1
30035	0	7.10	1	0.77246	.	.	1
30036	0	7.10	1	0.77246	.	.	1
30037	0	7.10	1	0.77246	.	.	1
30038	0	7.11	0	0.77238	0.76739	0.77727	1
30039	0	7.11	1	0.77238	.	.	1
30040	0	7.11	1	0.77238	.	.	1
30041	0	7.11	1	0.77238	.	.	1
30042	0	7.11	1	0.77238	.	.	1
30043	0	7.11	1	0.77238	.	.	1
30044	0	7.11	1	0.77238	.	.	1
30045	0	7.11	1	0.77238	.	.	1
30046	0	7.11	1	0.77238	.	.	1
30047	0	7.11	1	0.77238	.	.	1
30048	0	7.11	1	0.77238	.	.	1
30049	0	7.11	1	0.77238	.	.	1
30050	0	7.11	1	0.77238	.	.	1
30051	0	7.11	1	0.77238	.	.	1
30052	0	7.11	1	0.77238	.	.	1
30053	0	7.11	1	0.77238	.	.	1
30054	0	7.11	1	0.77238	.	.	1
30055	0	7.11	1	0.77238	.	.	1
30056	0	7.11	1	0.77238	.	.	1
30057	0	7.11	1	0.77238	.	.	1
30058	0	7.11	1	0.77238	.	.	1
30059	0	7.11	1	0.77238	.	.	1
30060	0	7.11	1	0.77238	.	.	1
30061	0	7.11	1	0.77238	.	.	1
30062	0	7.12	0	0.77222	0.76723	0.77711	1
30063	0	7.12	1	0.77222	.	.	1
30064	0	7.12	1	0.77222	.	.	1
30065	0	7.12	1	0.77222	.	.	1

Obs	Sub_group	fuptimey	_CENSOR_	SURVIVAL	SDF_LCL	SDF_UCL	STRATUM
30066	0	7.12	1	0.77222	.	.	1
30067	0	7.12	1	0.77222	.	.	1
30068	0	7.12	1	0.77222	.	.	1
30069	0	7.12	1	0.77222	.	.	1
30070	0	7.12	1	0.77222	.	.	1
30071	0	7.12	1	0.77222	.	.	1
30072	0	7.12	1	0.77222	.	.	1
30073	0	7.12	1	0.77222	.	.	1
30074	0	7.12	1	0.77222	.	.	1
30075	0	7.12	1	0.77222	.	.	1
30076	0	7.12	1	0.77222	.	.	1
30077	0	7.12	1	0.77222	.	.	1
30078	0	7.12	1	0.77222	.	.	1
30079	0	7.12	1	0.77222	.	.	1
30080	0	7.12	1	0.77222	.	.	1
30081	0	7.12	1	0.77222	.	.	1
30082	0	7.12	1	0.77222	.	.	1
30083	0	7.12	1	0.77222	.	.	1
30084	0	7.12	1	0.77222	.	.	1
30085	0	7.12	1	0.77222	.	.	1
30086	0	7.12	1	0.77222	.	.	1
30087	0	7.12	1	0.77222	.	.	1
30088	0	7.12	1	0.77222	.	.	1
30089	0	7.12	1	0.77222	.	.	1
30090	0	7.12	1	0.77222	.	.	1
30091	0	7.12	1	0.77222	.	.	1
30092	0	7.12	1	0.77222	.	.	1
30093	0	7.12	1	0.77222	.	.	1
30094	0	7.13	0	0.77205	0.76706	0.77696	1
30095	0	7.13	1	0.77205	.	.	1
30096	0	7.13	1	0.77205	.	.	1
30097	0	7.13	1	0.77205	.	.	1
30098	0	7.13	1	0.77205	.	.	1
30099	0	7.13	1	0.77205	.	.	1
30100	0	7.13	1	0.77205	.	.	1

Obs	Sub_group	fuptimey	_CENSOR_	SURVIVAL	SDF_LCL	SDF_UCL	STRATUM
30101	0	7.13	1	0.77205	.	.	1
30102	0	7.13	1	0.77205	.	.	1
30103	0	7.13	1	0.77205	.	.	1
30104	0	7.13	1	0.77205	.	.	1
30105	0	7.13	1	0.77205	.	.	1
30106	0	7.13	1	0.77205	.	.	1
30107	0	7.13	1	0.77205	.	.	1
30108	0	7.13	1	0.77205	.	.	1
30109	0	7.13	1	0.77205	.	.	1
30110	0	7.13	1	0.77205	.	.	1
30111	0	7.13	1	0.77205	.	.	1
30112	0	7.13	1	0.77205	.	.	1
30113	0	7.13	1	0.77205	.	.	1
30114	0	7.13	1	0.77205	.	.	1
30115	0	7.13	1	0.77205	.	.	1
30116	0	7.13	1	0.77205	.	.	1
30117	0	7.13	1	0.77205	.	.	1
30118	0	7.13	1	0.77205	.	.	1
30119	0	7.13	1	0.77205	.	.	1
30120	0	7.13	1	0.77205	.	.	1
30121	0	7.13	1	0.77205	.	.	1
30122	0	7.13	1	0.77205	.	.	1
30123	0	7.13	1	0.77205	.	.	1
30124	0	7.13	1	0.77205	.	.	1
30125	0	7.13	1	0.77205	.	.	1
30126	0	7.13	1	0.77205	.	.	1
30127	0	7.13	1	0.77205	.	.	1
30128	0	7.13	1	0.77205	.	.	1
30129	0	7.13	1	0.77205	.	.	1
30130	0	7.13	1	0.77205	.	.	1
30131	0	7.13	1	0.77205	.	.	1
30132	0	7.13	1	0.77205	.	.	1
30133	0	7.14	0	0.77173	0.76673	0.77664	1
30134	0	7.14	1	0.77173	.	.	1
30135	0	7.14	1	0.77173	.	.	1

Obs	Sub_group	fuptimey	_CENSOR_	SURVIVAL	SDF_LCL	SDF_UCL	STRATUM
30136	0	7.14	1	0.77173	.	.	1
30137	0	7.14	1	0.77173	.	.	1
30138	0	7.14	1	0.77173	.	.	1
30139	0	7.14	1	0.77173	.	.	1
30140	0	7.14	1	0.77173	.	.	1
30141	0	7.14	1	0.77173	.	.	1
30142	0	7.14	1	0.77173	.	.	1
30143	0	7.14	1	0.77173	.	.	1
30144	0	7.14	1	0.77173	.	.	1
30145	0	7.14	1	0.77173	.	.	1
30146	0	7.14	1	0.77173	.	.	1
30147	0	7.14	1	0.77173	.	.	1
30148	0	7.14	1	0.77173	.	.	1
30149	0	7.14	1	0.77173	.	.	1
30150	0	7.14	1	0.77173	.	.	1
30151	0	7.14	1	0.77173	.	.	1
30152	0	7.14	1	0.77173	.	.	1
30153	0	7.14	1	0.77173	.	.	1
30154	0	7.14	1	0.77173	.	.	1
30155	0	7.14	1	0.77173	.	.	1
30156	0	7.14	1	0.77173	.	.	1
30157	0	7.14	1	0.77173	.	.	1
30158	0	7.14	1	0.77173	.	.	1
30159	0	7.14	1	0.77173	.	.	1
30160	0	7.14	1	0.77173	.	.	1
30161	0	7.14	1	0.77173	.	.	1
30162	0	7.14	1	0.77173	.	.	1
30163	0	7.14	1	0.77173	.	.	1
30164	0	7.14	1	0.77173	.	.	1
30165	0	7.14	1	0.77173	.	.	1
30166	0	7.14	1	0.77173	.	.	1
30167	0	7.14	1	0.77173	.	.	1
30168	0	7.14	1	0.77173	.	.	1
30169	0	7.14	1	0.77173	.	.	1
30170	0	7.14	1	0.77173	.	.	1

Obs	Sub_group	fuptimey	_CENSOR_	SURVIVAL	SDF_LCL	SDF_UCL	STRATUM
30171	0	7.14	1	0.77173	.	.	1
30172	0	7.14	1	0.77173	.	.	1
30173	0	7.14	1	0.77173	.	.	1
30174	0	7.15	0	0.77165	0.76664	0.77656	1
30175	0	7.15	1	0.77165	.	.	1
30176	0	7.15	1	0.77165	.	.	1
30177	0	7.15	1	0.77165	.	.	1
30178	0	7.15	1	0.77165	.	.	1
30179	0	7.15	1	0.77165	.	.	1
30180	0	7.15	1	0.77165	.	.	1
30181	0	7.15	1	0.77165	.	.	1
30182	0	7.15	1	0.77165	.	.	1
30183	0	7.15	1	0.77165	.	.	1
30184	0	7.15	1	0.77165	.	.	1
30185	0	7.15	1	0.77165	.	.	1
30186	0	7.15	1	0.77165	.	.	1
30187	0	7.15	1	0.77165	.	.	1
30188	0	7.15	1	0.77165	.	.	1
30189	0	7.15	1	0.77165	.	.	1
30190	0	7.15	1	0.77165	.	.	1
30191	0	7.15	1	0.77165	.	.	1
30192	0	7.15	1	0.77165	.	.	1
30193	0	7.15	1	0.77165	.	.	1
30194	0	7.15	1	0.77165	.	.	1
30195	0	7.15	1	0.77165	.	.	1
30196	0	7.15	1	0.77165	.	.	1
30197	0	7.15	1	0.77165	.	.	1
30198	0	7.15	1	0.77165	.	.	1
30199	0	7.15	1	0.77165	.	.	1
30200	0	7.15	1	0.77165	.	.	1
30201	0	7.15	1	0.77165	.	.	1
30202	0	7.15	1	0.77165	.	.	1
30203	0	7.15	1	0.77165	.	.	1
30204	0	7.15	1	0.77165	.	.	1
30205	0	7.15	1	0.77165	.	.	1

Obs	Sub_group	fuptimey	_CENSOR_	SURVIVAL	SDF_LCL	SDF_UCL	STRATUM
30206	0	7.15	1	0.77165	.	.	1
30207	0	7.16	0	0.77132	0.76631	0.77624	1
30208	0	7.16	1	0.77132	.	.	1
30209	0	7.16	1	0.77132	.	.	1
30210	0	7.16	1	0.77132	.	.	1
30211	0	7.16	1	0.77132	.	.	1
30212	0	7.16	1	0.77132	.	.	1
30213	0	7.16	1	0.77132	.	.	1
30214	0	7.16	1	0.77132	.	.	1
30215	0	7.16	1	0.77132	.	.	1
30216	0	7.16	1	0.77132	.	.	1
30217	0	7.16	1	0.77132	.	.	1
30218	0	7.16	1	0.77132	.	.	1
30219	0	7.16	1	0.77132	.	.	1
30220	0	7.16	1	0.77132	.	.	1
30221	0	7.16	1	0.77132	.	.	1
30222	0	7.16	1	0.77132	.	.	1
30223	0	7.16	1	0.77132	.	.	1
30224	0	7.16	1	0.77132	.	.	1
30225	0	7.16	1	0.77132	.	.	1
30226	0	7.16	1	0.77132	.	.	1
30227	0	7.16	1	0.77132	.	.	1
30228	0	7.16	1	0.77132	.	.	1
30229	0	7.16	1	0.77132	.	.	1
30230	0	7.16	1	0.77132	.	.	1
30231	0	7.16	1	0.77132	.	.	1
30232	0	7.16	1	0.77132	.	.	1
30233	0	7.16	1	0.77132	.	.	1
30234	0	7.16	1	0.77132	.	.	1
30235	0	7.16	1	0.77132	.	.	1
30236	0	7.16	1	0.77132	.	.	1
30237	0	7.16	1	0.77132	.	.	1
30238	0	7.16	1	0.77132	.	.	1
30239	0	7.16	1	0.77132	.	.	1
30240	0	7.16	1	0.77132	.	.	1

Obs	Sub_group	fuptimey	_CENSOR_	SURVIVAL	SDF_LCL	SDF_UCL	STRATUM
**30241**	0	7.16	1	0.77132	.	.	1
**30242**	0	7.16	1	0.77132	.	.	1
**30243**	0	7.16	1	0.77132	.	.	1
**30244**	0	7.17	0	0.77107	0.76605	0.77600	1
**30245**	0	7.17	1	0.77107	.	.	1
**30246**	0	7.17	1	0.77107	.	.	1
**30247**	0	7.17	1	0.77107	.	.	1
**30248**	0	7.17	1	0.77107	.	.	1
**30249**	0	7.17	1	0.77107	.	.	1
**30250**	0	7.17	1	0.77107	.	.	1
**30251**	0	7.17	1	0.77107	.	.	1
**30252**	0	7.17	1	0.77107	.	.	1
**30253**	0	7.17	1	0.77107	.	.	1
**30254**	0	7.17	1	0.77107	.	.	1
**30255**	0	7.17	1	0.77107	.	.	1
**30256**	0	7.17	1	0.77107	.	.	1
**30257**	0	7.17	1	0.77107	.	.	1
**30258**	0	7.17	1	0.77107	.	.	1
**30259**	0	7.17	1	0.77107	.	.	1
**30260**	0	7.17	1	0.77107	.	.	1
**30261**	0	7.17	1	0.77107	.	.	1
**30262**	0	7.17	1	0.77107	.	.	1
**30263**	0	7.17	1	0.77107	.	.	1
**30264**	0	7.17	1	0.77107	.	.	1
**30265**	0	7.17	1	0.77107	.	.	1
**30266**	0	7.17	1	0.77107	.	.	1
**30267**	0	7.17	1	0.77107	.	.	1
**30268**	0	7.17	1	0.77107	.	.	1
**30269**	0	7.17	1	0.77107	.	.	1
**30270**	0	7.17	1	0.77107	.	.	1
**30271**	0	7.17	1	0.77107	.	.	1
**30272**	0	7.17	1	0.77107	.	.	1
**30273**	0	7.17	1	0.77107	.	.	1
**30274**	0	7.17	1	0.77107	.	.	1
**30275**	0	7.17	1	0.77107	.	.	1

Obs	Sub_group	fuptimey	_CENSOR_	SURVIVAL	SDF_LCL	SDF_UCL	STRATUM
**30276**	0	7.17	1	0.77107	.	.	1
**30277**	0	7.17	1	0.77107	.	.	1
**30278**	0	7.17	1	0.77107	.	.	1
**30279**	0	7.18	0	0.77082	0.76580	0.77576	1
**30280**	0	7.18	1	0.77082	.	.	1
**30281**	0	7.18	1	0.77082	.	.	1
**30282**	0	7.18	1	0.77082	.	.	1
**30283**	0	7.18	1	0.77082	.	.	1
**30284**	0	7.18	1	0.77082	.	.	1
**30285**	0	7.18	1	0.77082	.	.	1
**30286**	0	7.18	1	0.77082	.	.	1
**30287**	0	7.18	1	0.77082	.	.	1
**30288**	0	7.18	1	0.77082	.	.	1
**30289**	0	7.18	1	0.77082	.	.	1
**30290**	0	7.18	1	0.77082	.	.	1
**30291**	0	7.18	1	0.77082	.	.	1
**30292**	0	7.18	1	0.77082	.	.	1
**30293**	0	7.18	1	0.77082	.	.	1
**30294**	0	7.18	1	0.77082	.	.	1
**30295**	0	7.18	1	0.77082	.	.	1
**30296**	0	7.18	1	0.77082	.	.	1
**30297**	0	7.18	1	0.77082	.	.	1
**30298**	0	7.18	1	0.77082	.	.	1
**30299**	0	7.18	1	0.77082	.	.	1
**30300**	0	7.18	1	0.77082	.	.	1
**30301**	0	7.18	1	0.77082	.	.	1
**30302**	0	7.18	1	0.77082	.	.	1
**30303**	0	7.18	1	0.77082	.	.	1
**30304**	0	7.18	1	0.77082	.	.	1
**30305**	0	7.18	1	0.77082	.	.	1
**30306**	0	7.18	1	0.77082	.	.	1
**30307**	0	7.18	1	0.77082	.	.	1
**30308**	0	7.18	1	0.77082	.	.	1
**30309**	0	7.19	0	0.77066	0.76563	0.77560	1
**30310**	0	7.19	1	0.77066	.	.	1

Obs	Sub_group	fuptimey	_CENSOR_	SURVIVAL	SDF_LCL	SDF_UCL	STRATUM
30311	0	7.19	1	0.77066	.	.	1
30312	0	7.19	1	0.77066	.	.	1
30313	0	7.19	1	0.77066	.	.	1
30314	0	7.19	1	0.77066	.	.	1
30315	0	7.19	1	0.77066	.	.	1
30316	0	7.19	1	0.77066	.	.	1
30317	0	7.19	1	0.77066	.	.	1
30318	0	7.19	1	0.77066	.	.	1
30319	0	7.19	1	0.77066	.	.	1
30320	0	7.19	1	0.77066	.	.	1
30321	0	7.19	1	0.77066	.	.	1
30322	0	7.19	1	0.77066	.	.	1
30323	0	7.19	1	0.77066	.	.	1
30324	0	7.19	1	0.77066	.	.	1
30325	0	7.19	1	0.77066	.	.	1
30326	0	7.19	1	0.77066	.	.	1
30327	0	7.19	1	0.77066	.	.	1
30328	0	7.19	1	0.77066	.	.	1
30329	0	7.19	1	0.77066	.	.	1
30330	0	7.19	1	0.77066	.	.	1
30331	0	7.19	1	0.77066	.	.	1
30332	0	7.19	1	0.77066	.	.	1
30333	0	7.19	1	0.77066	.	.	1
30334	0	7.19	1	0.77066	.	.	1
30335	0	7.20	0	0.77041	0.76537	0.77535	1
30336	0	7.20	1	0.77041	.	.	1
30337	0	7.20	1	0.77041	.	.	1
30338	0	7.20	1	0.77041	.	.	1
30339	0	7.20	1	0.77041	.	.	1
30340	0	7.20	1	0.77041	.	.	1
30341	0	7.20	1	0.77041	.	.	1
30342	0	7.20	1	0.77041	.	.	1
30343	0	7.20	1	0.77041	.	.	1
30344	0	7.20	1	0.77041	.	.	1
30345	0	7.20	1	0.77041	.	.	1

Obs	Sub_group	fuptimey	_CENSOR_	SURVIVAL	SDF_LCL	SDF_UCL	STRATUM
30346	0	7.20	1	0.77041	.	.	1
30347	0	7.20	1	0.77041	.	.	1
30348	0	7.20	1	0.77041	.	.	1
30349	0	7.20	1	0.77041	.	.	1
30350	0	7.20	1	0.77041	.	.	1
30351	0	7.20	1	0.77041	.	.	1
30352	0	7.20	1	0.77041	.	.	1
30353	0	7.20	1	0.77041	.	.	1
30354	0	7.20	1	0.77041	.	.	1
30355	0	7.20	1	0.77041	.	.	1
30356	0	7.20	1	0.77041	.	.	1
30357	0	7.20	1	0.77041	.	.	1
30358	0	7.20	1	0.77041	.	.	1
30359	0	7.20	1	0.77041	.	.	1
30360	0	7.20	1	0.77041	.	.	1
30361	0	7.20	1	0.77041	.	.	1
30362	0	7.20	1	0.77041	.	.	1
30363	0	7.21	0	0.77016	0.76512	0.77511	1
30364	0	7.21	1	0.77016	.	.	1
30365	0	7.21	1	0.77016	.	.	1
30366	0	7.21	1	0.77016	.	.	1
30367	0	7.21	1	0.77016	.	.	1
30368	0	7.21	1	0.77016	.	.	1
30369	0	7.21	1	0.77016	.	.	1
30370	0	7.21	1	0.77016	.	.	1
30371	0	7.21	1	0.77016	.	.	1
30372	0	7.21	1	0.77016	.	.	1
30373	0	7.21	1	0.77016	.	.	1
30374	0	7.21	1	0.77016	.	.	1
30375	0	7.21	1	0.77016	.	.	1
30376	0	7.21	1	0.77016	.	.	1
30377	0	7.21	1	0.77016	.	.	1
30378	0	7.21	1	0.77016	.	.	1
30379	0	7.21	1	0.77016	.	.	1
30380	0	7.21	1	0.77016	.	.	1

Obs	Sub_group	fuptimey	_CENSOR_	SURVIVAL	SDF_LCL	SDF_UCL	STRATUM
30381	0	7.21	1	0.77016	.	.	1
30382	0	7.21	1	0.77016	.	.	1
30383	0	7.21	1	0.77016	.	.	1
30384	0	7.21	1	0.77016	.	.	1
30385	0	7.21	1	0.77016	.	.	1
30386	0	7.21	1	0.77016	.	.	1
30387	0	7.21	1	0.77016	.	.	1
30388	0	7.21	1	0.77016	.	.	1
30389	0	7.21	1	0.77016	.	.	1
30390	0	7.21	1	0.77016	.	.	1
30391	0	7.21	1	0.77016	.	.	1
30392	0	7.21	1	0.77016	.	.	1
30393	0	7.21	1	0.77016	.	.	1
30394	0	7.21	1	0.77016	.	.	1
30395	0	7.21	1	0.77016	.	.	1
30396	0	7.21	1	0.77016	.	.	1
30397	0	7.21	1	0.77016	.	.	1
30398	0	7.21	1	0.77016	.	.	1
30399	0	7.21	1	0.77016	.	.	1
30400	0	7.21	1	0.77016	.	.	1
30401	0	7.21	1	0.77016	.	.	1
30402	0	7.21	1	0.77016	.	.	1
30403	0	7.21	1	0.77016	.	.	1
30404	0	7.21	1	0.77016	.	.	1
30405	0	7.21	1	0.77016	.	.	1
30406	0	7.21	1	0.77016	.	.	1
30407	0	7.21	1	0.77016	.	.	1
30408	0	7.21	1	0.77016	.	.	1
30409	0	7.22	0	0.76974	0.76469	0.77470	1
30410	0	7.22	1	0.76974	.	.	1
30411	0	7.22	1	0.76974	.	.	1
30412	0	7.22	1	0.76974	.	.	1
30413	0	7.22	1	0.76974	.	.	1
30414	0	7.22	1	0.76974	.	.	1
30415	0	7.22	1	0.76974	.	.	1

Obs	Sub_group	fuptimey	_CENSOR_	SURVIVAL	SDF_LCL	SDF_UCL	STRATUM
30416	0	7.22	1	0.76974	.	.	1
30417	0	7.22	1	0.76974	.	.	1
30418	0	7.22	1	0.76974	.	.	1
30419	0	7.22	1	0.76974	.	.	1
30420	0	7.22	1	0.76974	.	.	1
30421	0	7.22	1	0.76974	.	.	1
30422	0	7.22	1	0.76974	.	.	1
30423	0	7.22	1	0.76974	.	.	1
30424	0	7.22	1	0.76974	.	.	1
30425	0	7.22	1	0.76974	.	.	1
30426	0	7.22	1	0.76974	.	.	1
30427	0	7.22	1	0.76974	.	.	1
30428	0	7.22	1	0.76974	.	.	1
30429	0	7.22	1	0.76974	.	.	1
30430	0	7.22	1	0.76974	.	.	1
30431	0	7.22	1	0.76974	.	.	1
30432	0	7.22	1	0.76974	.	.	1
30433	0	7.22	1	0.76974	.	.	1
30434	0	7.22	1	0.76974	.	.	1
30435	0	7.22	1	0.76974	.	.	1
30436	0	7.22	1	0.76974	.	.	1
30437	0	7.22	1	0.76974	.	.	1
30438	0	7.22	1	0.76974	.	.	1
30439	0	7.22	1	0.76974	.	.	1
30440	0	7.22	1	0.76974	.	.	1
30441	0	7.22	1	0.76974	.	.	1
30442	0	7.22	1	0.76974	.	.	1
30443	0	7.22	1	0.76974	.	.	1
30444	0	7.23	0	0.76966	0.76460	0.77462	1
30445	0	7.23	1	0.76966	.	.	1
30446	0	7.23	1	0.76966	.	.	1
30447	0	7.23	1	0.76966	.	.	1
30448	0	7.23	1	0.76966	.	.	1
30449	0	7.23	1	0.76966	.	.	1
30450	0	7.23	1	0.76966	.	.	1

Obs	Sub_group	fuptimey	_CENSOR_	SURVIVAL	SDF_LCL	SDF_UCL	STRATUM
30451	0	7.23	1	0.76966	.	.	1
30452	0	7.23	1	0.76966	.	.	1
30453	0	7.23	1	0.76966	.	.	1
30454	0	7.23	1	0.76966	.	.	1
30455	0	7.23	1	0.76966	.	.	1
30456	0	7.23	1	0.76966	.	.	1
30457	0	7.23	1	0.76966	.	.	1
30458	0	7.23	1	0.76966	.	.	1
30459	0	7.23	1	0.76966	.	.	1
30460	0	7.23	1	0.76966	.	.	1
30461	0	7.23	1	0.76966	.	.	1
30462	0	7.23	1	0.76966	.	.	1
30463	0	7.23	1	0.76966	.	.	1
30464	0	7.23	1	0.76966	.	.	1
30465	0	7.23	1	0.76966	.	.	1
30466	0	7.23	1	0.76966	.	.	1
30467	0	7.23	1	0.76966	.	.	1
30468	0	7.23	1	0.76966	.	.	1
30469	0	7.23	1	0.76966	.	.	1
30470	0	7.23	1	0.76966	.	.	1
30471	0	7.23	1	0.76966	.	.	1
30472	0	7.23	1	0.76966	.	.	1
30473	0	7.23	1	0.76966	.	.	1
30474	0	7.23	1	0.76966	.	.	1
30475	0	7.23	1	0.76966	.	.	1
30476	0	7.23	1	0.76966	.	.	1
30477	0	7.23	1	0.76966	.	.	1
30478	0	7.23	1	0.76966	.	.	1
30479	0	7.23	1	0.76966	.	.	1
30480	0	7.23	1	0.76966	.	.	1
30481	0	7.23	1	0.76966	.	.	1
30482	0	7.23	1	0.76966	.	.	1
30483	0	7.23	1	0.76966	.	.	1
30484	0	7.23	1	0.76966	.	.	1
30485	0	7.23	1	0.76966	.	.	1

Obs	Sub_group	fuptimey	_CENSOR_	SURVIVAL	SDF_LCL	SDF_UCL	STRATUM
30486	0	7.24	0	0.76949	0.76443	0.77446	1
30487	0	7.24	1	0.76949	.	.	1
30488	0	7.24	1	0.76949	.	.	1
30489	0	7.24	1	0.76949	.	.	1
30490	0	7.24	1	0.76949	.	.	1
30491	0	7.24	1	0.76949	.	.	1
30492	0	7.24	1	0.76949	.	.	1
30493	0	7.24	1	0.76949	.	.	1
30494	0	7.24	1	0.76949	.	.	1
30495	0	7.24	1	0.76949	.	.	1
30496	0	7.24	1	0.76949	.	.	1
30497	0	7.24	1	0.76949	.	.	1
30498	0	7.24	1	0.76949	.	.	1
30499	0	7.24	1	0.76949	.	.	1
30500	0	7.24	1	0.76949	.	.	1
30501	0	7.24	1	0.76949	.	.	1
30502	0	7.24	1	0.76949	.	.	1
30503	0	7.24	1	0.76949	.	.	1
30504	0	7.24	1	0.76949	.	.	1
30505	0	7.24	1	0.76949	.	.	1
30506	0	7.24	1	0.76949	.	.	1
30507	0	7.24	1	0.76949	.	.	1
30508	0	7.24	1	0.76949	.	.	1
30509	0	7.24	1	0.76949	.	.	1
30510	0	7.25	0	0.76940	0.76434	0.77437	1
30511	0	7.25	1	0.76940	.	.	1
30512	0	7.25	1	0.76940	.	.	1
30513	0	7.25	1	0.76940	.	.	1
30514	0	7.25	1	0.76940	.	.	1
30515	0	7.25	1	0.76940	.	.	1
30516	0	7.25	1	0.76940	.	.	1
30517	0	7.25	1	0.76940	.	.	1
30518	0	7.25	1	0.76940	.	.	1
30519	0	7.25	1	0.76940	.	.	1
30520	0	7.25	1	0.76940	.	.	1

Obs	Sub_group	fuptimey	_CENSOR_	SURVIVAL	SDF_LCL	SDF_UCL	STRATUM
30521	0	7.25	1	0.76940	.	.	1
30522	0	7.25	1	0.76940	.	.	1
30523	0	7.25	1	0.76940	.	.	1
30524	0	7.25	1	0.76940	.	.	1
30525	0	7.25	1	0.76940	.	.	1
30526	0	7.25	1	0.76940	.	.	1
30527	0	7.25	1	0.76940	.	.	1
30528	0	7.25	1	0.76940	.	.	1
30529	0	7.25	1	0.76940	.	.	1
30530	0	7.25	1	0.76940	.	.	1
30531	0	7.25	1	0.76940	.	.	1
30532	0	7.25	1	0.76940	.	.	1
30533	0	7.25	1	0.76940	.	.	1
30534	0	7.25	1	0.76940	.	.	1
30535	0	7.25	1	0.76940	.	.	1
30536	0	7.25	1	0.76940	.	.	1
30537	0	7.25	1	0.76940	.	.	1
30538	0	7.25	1	0.76940	.	.	1
30539	0	7.26	0	0.76915	0.76408	0.77412	1
30540	0	7.26	1	0.76915	.	.	1
30541	0	7.26	1	0.76915	.	.	1
30542	0	7.26	1	0.76915	.	.	1
30543	0	7.26	1	0.76915	.	.	1
30544	0	7.26	1	0.76915	.	.	1
30545	0	7.26	1	0.76915	.	.	1
30546	0	7.26	1	0.76915	.	.	1
30547	0	7.26	1	0.76915	.	.	1
30548	0	7.26	1	0.76915	.	.	1
30549	0	7.26	1	0.76915	.	.	1
30550	0	7.26	1	0.76915	.	.	1
30551	0	7.26	1	0.76915	.	.	1
30552	0	7.26	1	0.76915	.	.	1
30553	0	7.26	1	0.76915	.	.	1
30554	0	7.26	1	0.76915	.	.	1
30555	0	7.26	1	0.76915	.	.	1

Obs	Sub_group	fuptimey	_CENSOR_	SURVIVAL	SDF_LCL	SDF_UCL	STRATUM
30556	0	7.26	1	0.76915	.	.	1
30557	0	7.26	1	0.76915	.	.	1
30558	0	7.26	1	0.76915	.	.	1
30559	0	7.26	1	0.76915	.	.	1
30560	0	7.26	1	0.76915	.	.	1
30561	0	7.26	1	0.76915	.	.	1
30562	0	7.26	1	0.76915	.	.	1
30563	0	7.26	1	0.76915	.	.	1
30564	0	7.26	1	0.76915	.	.	1
30565	0	7.26	1	0.76915	.	.	1
30566	0	7.27	0	0.76881	0.76373	0.77379	1
30567	0	7.27	1	0.76881	.	.	1
30568	0	7.27	1	0.76881	.	.	1
30569	0	7.27	1	0.76881	.	.	1
30570	0	7.27	1	0.76881	.	.	1
30571	0	7.27	1	0.76881	.	.	1
30572	0	7.27	1	0.76881	.	.	1
30573	0	7.27	1	0.76881	.	.	1
30574	0	7.27	1	0.76881	.	.	1
30575	0	7.27	1	0.76881	.	.	1
30576	0	7.27	1	0.76881	.	.	1
30577	0	7.27	1	0.76881	.	.	1
30578	0	7.27	1	0.76881	.	.	1
30579	0	7.27	1	0.76881	.	.	1
30580	0	7.27	1	0.76881	.	.	1
30581	0	7.27	1	0.76881	.	.	1
30582	0	7.27	1	0.76881	.	.	1
30583	0	7.27	1	0.76881	.	.	1
30584	0	7.27	1	0.76881	.	.	1
30585	0	7.27	1	0.76881	.	.	1
30586	0	7.27	1	0.76881	.	.	1
30587	0	7.27	1	0.76881	.	.	1
30588	0	7.27	1	0.76881	.	.	1
30589	0	7.27	1	0.76881	.	.	1
30590	0	7.27	1	0.76881	.	.	1

Obs	Sub_group	fuptimey	_CENSOR_	SURVIVAL	SDF_LCL	SDF_UCL	STRATUM
30591	0	7.27	1	0.76881	.	.	1
30592	0	7.27	1	0.76881	.	.	1
30593	0	7.27	1	0.76881	.	.	1
30594	0	7.27	1	0.76881	.	.	1
30595	0	7.28	0	0.76855	0.76347	0.77354	1
30596	0	7.28	1	0.76855	.	.	1
30597	0	7.28	1	0.76855	.	.	1
30598	0	7.28	1	0.76855	.	.	1
30599	0	7.28	1	0.76855	.	.	1
30600	0	7.28	1	0.76855	.	.	1
30601	0	7.28	1	0.76855	.	.	1
30602	0	7.28	1	0.76855	.	.	1
30603	0	7.28	1	0.76855	.	.	1
30604	0	7.28	1	0.76855	.	.	1
30605	0	7.28	1	0.76855	.	.	1
30606	0	7.28	1	0.76855	.	.	1
30607	0	7.28	1	0.76855	.	.	1
30608	0	7.28	1	0.76855	.	.	1
30609	0	7.28	1	0.76855	.	.	1
30610	0	7.28	1	0.76855	.	.	1
30611	0	7.28	1	0.76855	.	.	1
30612	0	7.28	1	0.76855	.	.	1
30613	0	7.28	1	0.76855	.	.	1
30614	0	7.28	1	0.76855	.	.	1
30615	0	7.28	1	0.76855	.	.	1
30616	0	7.28	1	0.76855	.	.	1
30617	0	7.28	1	0.76855	.	.	1
30618	0	7.28	1	0.76855	.	.	1
30619	0	7.28	1	0.76855	.	.	1
30620	0	7.29	0	0.76838	0.76329	0.77338	1
30621	0	7.29	1	0.76838	.	.	1
30622	0	7.29	1	0.76838	.	.	1
30623	0	7.29	1	0.76838	.	.	1
30624	0	7.29	1	0.76838	.	.	1
30625	0	7.29	1	0.76838	.	.	1

Obs	Sub_group	fuptimey	_CENSOR_	SURVIVAL	SDF_LCL	SDF_UCL	STRATUM
30626	0	7.29	1	0.76838	.	.	1
30627	0	7.29	1	0.76838	.	.	1
30628	0	7.29	1	0.76838	.	.	1
30629	0	7.29	1	0.76838	.	.	1
30630	0	7.29	1	0.76838	.	.	1
30631	0	7.29	1	0.76838	.	.	1
30632	0	7.29	1	0.76838	.	.	1
30633	0	7.29	1	0.76838	.	.	1
30634	0	7.29	1	0.76838	.	.	1
30635	0	7.29	1	0.76838	.	.	1
30636	0	7.29	1	0.76838	.	.	1
30637	0	7.29	1	0.76838	.	.	1
30638	0	7.29	1	0.76838	.	.	1
30639	0	7.29	1	0.76838	.	.	1
30640	0	7.29	1	0.76838	.	.	1
30641	0	7.29	1	0.76838	.	.	1
30642	0	7.29	1	0.76838	.	.	1
30643	0	7.29	1	0.76838	.	.	1
30644	0	7.29	1	0.76838	.	.	1
30645	0	7.29	1	0.76838	.	.	1
30646	0	7.29	1	0.76838	.	.	1
30647	0	7.29	1	0.76838	.	.	1
30648	0	7.29	1	0.76838	.	.	1
30649	0	7.29	1	0.76838	.	.	1
30650	0	7.29	1	0.76838	.	.	1
30651	0	7.29	1	0.76838	.	.	1
30652	0	7.29	1	0.76838	.	.	1
30653	0	7.29	1	0.76838	.	.	1
30654	0	7.29	1	0.76838	.	.	1
30655	0	7.29	1	0.76838	.	.	1
30656	0	7.29	1	0.76838	.	.	1
30657	0	7.29	1	0.76838	.	.	1
30658	0	7.29	1	0.76838	.	.	1
30659	0	7.29	1	0.76838	.	.	1
30660	0	7.29	1	0.76838	.	.	1

Obs	Sub_group	fuptimey	_CENSOR_	SURVIVAL	SDF_LCL	SDF_UCL	STRATUM
30661	0	7.29	1	0.76838	.	.	1
30662	0	7.30	0	0.76821	0.76312	0.77321	1
30663	0	7.30	1	0.76821	.	.	1
30664	0	7.30	1	0.76821	.	.	1
30665	0	7.30	1	0.76821	.	.	1
30666	0	7.30	1	0.76821	.	.	1
30667	0	7.30	1	0.76821	.	.	1
30668	0	7.30	1	0.76821	.	.	1
30669	0	7.30	1	0.76821	.	.	1
30670	0	7.30	1	0.76821	.	.	1
30671	0	7.30	1	0.76821	.	.	1
30672	0	7.30	1	0.76821	.	.	1
30673	0	7.30	1	0.76821	.	.	1
30674	0	7.30	1	0.76821	.	.	1
30675	0	7.30	1	0.76821	.	.	1
30676	0	7.30	1	0.76821	.	.	1
30677	0	7.30	1	0.76821	.	.	1
30678	0	7.30	1	0.76821	.	.	1
30679	0	7.30	1	0.76821	.	.	1
30680	0	7.30	1	0.76821	.	.	1
30681	0	7.30	1	0.76821	.	.	1
30682	0	7.30	1	0.76821	.	.	1
30683	0	7.30	1	0.76821	.	.	1
30684	0	7.30	1	0.76821	.	.	1
30685	0	7.30	1	0.76821	.	.	1
30686	0	7.31	0	0.76804	0.76294	0.77304	1
30687	0	7.31	1	0.76804	.	.	1
30688	0	7.31	1	0.76804	.	.	1
30689	0	7.31	1	0.76804	.	.	1
30690	0	7.31	1	0.76804	.	.	1
30691	0	7.31	1	0.76804	.	.	1
30692	0	7.31	1	0.76804	.	.	1
30693	0	7.31	1	0.76804	.	.	1
30694	0	7.31	1	0.76804	.	.	1
30695	0	7.31	1	0.76804	.	.	1

Obs	Sub_group	fuptimey	_CENSOR_	SURVIVAL	SDF_LCL	SDF_UCL	STRATUM
30696	0	7.31	1	0.76804	.	.	1
30697	0	7.31	1	0.76804	.	.	1
30698	0	7.31	1	0.76804	.	.	1
30699	0	7.31	1	0.76804	.	.	1
30700	0	7.31	1	0.76804	.	.	1
30701	0	7.31	1	0.76804	.	.	1
30702	0	7.31	1	0.76804	.	.	1
30703	0	7.31	1	0.76804	.	.	1
30704	0	7.31	1	0.76804	.	.	1
30705	0	7.31	1	0.76804	.	.	1
30706	0	7.31	1	0.76804	.	.	1
30707	0	7.31	1	0.76804	.	.	1
30708	0	7.31	1	0.76804	.	.	1
30709	0	7.31	1	0.76804	.	.	1
30710	0	7.31	1	0.76804	.	.	1
30711	0	7.31	1	0.76804	.	.	1
30712	0	7.31	1	0.76804	.	.	1
30713	0	7.31	1	0.76804	.	.	1
30714	0	7.31	1	0.76804	.	.	1
30715	0	7.31	1	0.76804	.	.	1
30716	0	7.31	1	0.76804	.	.	1
30717	0	7.31	1	0.76804	.	.	1
30718	0	7.31	1	0.76804	.	.	1
30719	0	7.31	1	0.76804	.	.	1
30720	0	7.31	1	0.76804	.	.	1
30721	0	7.32	0	0.76778	0.76267	0.77279	1
30722	0	7.32	1	0.76778	.	.	1
30723	0	7.32	1	0.76778	.	.	1
30724	0	7.32	1	0.76778	.	.	1
30725	0	7.32	1	0.76778	.	.	1
30726	0	7.32	1	0.76778	.	.	1
30727	0	7.32	1	0.76778	.	.	1
30728	0	7.32	1	0.76778	.	.	1
30729	0	7.32	1	0.76778	.	.	1
30730	0	7.32	1	0.76778	.	.	1

Obs	Sub_group	fuptimey	_CENSOR_	SURVIVAL	SDF_LCL	SDF_UCL	STRATUM
30731	0	7.32	1	0.76778	.	.	1
30732	0	7.32	1	0.76778	.	.	1
30733	0	7.32	1	0.76778	.	.	1
30734	0	7.32	1	0.76778	.	.	1
30735	0	7.32	1	0.76778	.	.	1
30736	0	7.32	1	0.76778	.	.	1
30737	0	7.32	1	0.76778	.	.	1
30738	0	7.32	1	0.76778	.	.	1
30739	0	7.32	1	0.76778	.	.	1
30740	0	7.32	1	0.76778	.	.	1
30741	0	7.32	1	0.76778	.	.	1
30742	0	7.32	1	0.76778	.	.	1
30743	0	7.32	1	0.76778	.	.	1
30744	0	7.33	1	0.76778	.	.	1
30745	0	7.33	1	0.76778	.	.	1
30746	0	7.33	1	0.76778	.	.	1
30747	0	7.33	1	0.76778	.	.	1
30748	0	7.33	1	0.76778	.	.	1
30749	0	7.33	1	0.76778	.	.	1
30750	0	7.33	1	0.76778	.	.	1
30751	0	7.33	1	0.76778	.	.	1
30752	0	7.33	1	0.76778	.	.	1
30753	0	7.33	1	0.76778	.	.	1
30754	0	7.33	1	0.76778	.	.	1
30755	0	7.33	1	0.76778	.	.	1
30756	0	7.33	1	0.76778	.	.	1
30757	0	7.33	1	0.76778	.	.	1
30758	0	7.33	1	0.76778	.	.	1
30759	0	7.33	1	0.76778	.	.	1
30760	0	7.33	1	0.76778	.	.	1
30761	0	7.33	1	0.76778	.	.	1
30762	0	7.33	1	0.76778	.	.	1
30763	0	7.33	1	0.76778	.	.	1
30764	0	7.33	1	0.76778	.	.	1
30765	0	7.33	1	0.76778	.	.	1

Obs	Sub_group	fuptimey	_CENSOR_	SURVIVAL	SDF_LCL	SDF_UCL	STRATUM
30766	0	7.33	1	0.76778	.	.	1
30767	0	7.33	1	0.76778	.	.	1
30768	0	7.33	1	0.76778	.	.	1
30769	0	7.33	1	0.76778	.	.	1
30770	0	7.33	1	0.76778	.	.	1
30771	0	7.33	1	0.76778	.	.	1
30772	0	7.33	1	0.76778	.	.	1
30773	0	7.33	1	0.76778	.	.	1
30774	0	7.34	0	0.76734	0.76223	0.77236	1
30775	0	7.34	1	0.76734	.	.	1
30776	0	7.34	1	0.76734	.	.	1
30777	0	7.34	1	0.76734	.	.	1
30778	0	7.34	1	0.76734	.	.	1
30779	0	7.34	1	0.76734	.	.	1
30780	0	7.34	1	0.76734	.	.	1
30781	0	7.34	1	0.76734	.	.	1
30782	0	7.34	1	0.76734	.	.	1
30783	0	7.34	1	0.76734	.	.	1
30784	0	7.34	1	0.76734	.	.	1
30785	0	7.34	1	0.76734	.	.	1
30786	0	7.34	1	0.76734	.	.	1
30787	0	7.34	1	0.76734	.	.	1
30788	0	7.34	1	0.76734	.	.	1
30789	0	7.34	1	0.76734	.	.	1
30790	0	7.34	1	0.76734	.	.	1
30791	0	7.34	1	0.76734	.	.	1
30792	0	7.34	1	0.76734	.	.	1
30793	0	7.34	1	0.76734	.	.	1
30794	0	7.34	1	0.76734	.	.	1
30795	0	7.34	1	0.76734	.	.	1
30796	0	7.34	1	0.76734	.	.	1
30797	0	7.34	1	0.76734	.	.	1
30798	0	7.34	1	0.76734	.	.	1
30799	0	7.34	1	0.76734	.	.	1
30800	0	7.34	1	0.76734	.	.	1

Obs	Sub_group	fuptimey	_CENSOR_	SURVIVAL	SDF_LCL	SDF_UCL	STRATUM
30801	0	7.34	1	0.76734	.	.	1
30802	0	7.34	1	0.76734	.	.	1
30803	0	7.35	0	0.76717	0.76205	0.77219	1
30804	0	7.35	1	0.76717	.	.	1
30805	0	7.35	1	0.76717	.	.	1
30806	0	7.35	1	0.76717	.	.	1
30807	0	7.35	1	0.76717	.	.	1
30808	0	7.35	1	0.76717	.	.	1
30809	0	7.35	1	0.76717	.	.	1
30810	0	7.35	1	0.76717	.	.	1
30811	0	7.35	1	0.76717	.	.	1
30812	0	7.35	1	0.76717	.	.	1
30813	0	7.35	1	0.76717	.	.	1
30814	0	7.35	1	0.76717	.	.	1
30815	0	7.35	1	0.76717	.	.	1
30816	0	7.35	1	0.76717	.	.	1
30817	0	7.35	1	0.76717	.	.	1
30818	0	7.35	1	0.76717	.	.	1
30819	0	7.35	1	0.76717	.	.	1
30820	0	7.35	1	0.76717	.	.	1
30821	0	7.35	1	0.76717	.	.	1
30822	0	7.35	1	0.76717	.	.	1
30823	0	7.35	1	0.76717	.	.	1
30824	0	7.35	1	0.76717	.	.	1
30825	0	7.35	1	0.76717	.	.	1
30826	0	7.35	1	0.76717	.	.	1
30827	0	7.35	1	0.76717	.	.	1
30828	0	7.35	1	0.76717	.	.	1
30829	0	7.35	1	0.76717	.	.	1
30830	0	7.35	1	0.76717	.	.	1
30831	0	7.35	1	0.76717	.	.	1
30832	0	7.35	1	0.76717	.	.	1
30833	0	7.35	1	0.76717	.	.	1
30834	0	7.35	1	0.76717	.	.	1
30835	0	7.35	1	0.76717	.	.	1

Obs	Sub_group	fuptimey	_CENSOR_	SURVIVAL	SDF_LCL	SDF_UCL	STRATUM
30836	0	7.35	1	0.76717	.	.	1
30837	0	7.36	0	0.76690	0.76178	0.77194	1
30838	0	7.36	1	0.76690	.	.	1
30839	0	7.36	1	0.76690	.	.	1
30840	0	7.36	1	0.76690	.	.	1
30841	0	7.36	1	0.76690	.	.	1
30842	0	7.36	1	0.76690	.	.	1
30843	0	7.36	1	0.76690	.	.	1
30844	0	7.36	1	0.76690	.	.	1
30845	0	7.36	1	0.76690	.	.	1
30846	0	7.36	1	0.76690	.	.	1
30847	0	7.36	1	0.76690	.	.	1
30848	0	7.36	1	0.76690	.	.	1
30849	0	7.36	1	0.76690	.	.	1
30850	0	7.36	1	0.76690	.	.	1
30851	0	7.36	1	0.76690	.	.	1
30852	0	7.36	1	0.76690	.	.	1
30853	0	7.36	1	0.76690	.	.	1
30854	0	7.36	1	0.76690	.	.	1
30855	0	7.36	1	0.76690	.	.	1
30856	0	7.36	1	0.76690	.	.	1
30857	0	7.36	1	0.76690	.	.	1
30858	0	7.36	1	0.76690	.	.	1
30859	0	7.36	1	0.76690	.	.	1
30860	0	7.36	1	0.76690	.	.	1
30861	0	7.36	1	0.76690	.	.	1
30862	0	7.36	1	0.76690	.	.	1
30863	0	7.36	1	0.76690	.	.	1
30864	0	7.36	1	0.76690	.	.	1
30865	0	7.36	1	0.76690	.	.	1
30866	0	7.36	1	0.76690	.	.	1
30867	0	7.37	0	0.76673	0.76160	0.77177	1
30868	0	7.37	1	0.76673	.	.	1
30869	0	7.37	1	0.76673	.	.	1
30870	0	7.37	1	0.76673	.	.	1

Obs	Sub_group	fuptimey	_CENSOR_	SURVIVAL	SDF_LCL	SDF_UCL	STRATUM
30871	0	7.37	1	0.76673	.	.	1
30872	0	7.37	1	0.76673	.	.	1
30873	0	7.37	1	0.76673	.	.	1
30874	0	7.37	1	0.76673	.	.	1
30875	0	7.37	1	0.76673	.	.	1
30876	0	7.37	1	0.76673	.	.	1
30877	0	7.37	1	0.76673	.	.	1
30878	0	7.37	1	0.76673	.	.	1
30879	0	7.37	1	0.76673	.	.	1
30880	0	7.37	1	0.76673	.	.	1
30881	0	7.37	1	0.76673	.	.	1
30882	0	7.37	1	0.76673	.	.	1
30883	0	7.37	1	0.76673	.	.	1
30884	0	7.37	1	0.76673	.	.	1
30885	0	7.37	1	0.76673	.	.	1
30886	0	7.37	1	0.76673	.	.	1
30887	0	7.37	1	0.76673	.	.	1
30888	0	7.37	1	0.76673	.	.	1
30889	0	7.37	1	0.76673	.	.	1
30890	0	7.37	1	0.76673	.	.	1
30891	0	7.37	1	0.76673	.	.	1
30892	0	7.37	1	0.76673	.	.	1
30893	0	7.37	1	0.76673	.	.	1
30894	0	7.37	1	0.76673	.	.	1
30895	0	7.37	1	0.76673	.	.	1
30896	0	7.37	1	0.76673	.	.	1
30897	0	7.37	1	0.76673	.	.	1
30898	0	7.37	1	0.76673	.	.	1
30899	0	7.37	1	0.76673	.	.	1
30900	0	7.37	1	0.76673	.	.	1
30901	0	7.37	1	0.76673	.	.	1
30902	0	7.37	1	0.76673	.	.	1
30903	0	7.38	1	0.76673	.	.	1
30904	0	7.38	1	0.76673	.	.	1
30905	0	7.38	1	0.76673	.	.	1

Obs	Sub_group	fuptimey	_CENSOR_	SURVIVAL	SDF_LCL	SDF_UCL	STRATUM
30906	0	7.38	1	0.76673	.	.	1
30907	0	7.38	1	0.76673	.	.	1
30908	0	7.38	1	0.76673	.	.	1
30909	0	7.38	1	0.76673	.	.	1
30910	0	7.38	1	0.76673	.	.	1
30911	0	7.38	1	0.76673	.	.	1
30912	0	7.38	1	0.76673	.	.	1
30913	0	7.38	1	0.76673	.	.	1
30914	0	7.38	1	0.76673	.	.	1
30915	0	7.38	1	0.76673	.	.	1
30916	0	7.38	1	0.76673	.	.	1
30917	0	7.38	1	0.76673	.	.	1
30918	0	7.38	1	0.76673	.	.	1
30919	0	7.38	1	0.76673	.	.	1
30920	0	7.38	1	0.76673	.	.	1
30921	0	7.38	1	0.76673	.	.	1
30922	0	7.38	1	0.76673	.	.	1
30923	0	7.38	1	0.76673	.	.	1
30924	0	7.38	1	0.76673	.	.	1
30925	0	7.39	0	0.76664	0.76150	0.77168	1
30926	0	7.39	1	0.76664	.	.	1
30927	0	7.39	1	0.76664	.	.	1
30928	0	7.39	1	0.76664	.	.	1
30929	0	7.39	1	0.76664	.	.	1
30930	0	7.39	1	0.76664	.	.	1
30931	0	7.39	1	0.76664	.	.	1
30932	0	7.39	1	0.76664	.	.	1
30933	0	7.39	1	0.76664	.	.	1
30934	0	7.39	1	0.76664	.	.	1
30935	0	7.39	1	0.76664	.	.	1
30936	0	7.39	1	0.76664	.	.	1
30937	0	7.39	1	0.76664	.	.	1
30938	0	7.39	1	0.76664	.	.	1
30939	0	7.39	1	0.76664	.	.	1
30940	0	7.39	1	0.76664	.	.	1

Obs	Sub_group	fuptimey	_CENSOR_	SURVIVAL	SDF_LCL	SDF_UCL	STRATUM
**30941**	0	7.39	1	0.76664	.	.	1
**30942**	0	7.39	1	0.76664	.	.	1
**30943**	0	7.39	1	0.76664	.	.	1
**30944**	0	7.39	1	0.76664	.	.	1
**30945**	0	7.39	1	0.76664	.	.	1
**30946**	0	7.39	1	0.76664	.	.	1
**30947**	0	7.39	1	0.76664	.	.	1
**30948**	0	7.39	1	0.76664	.	.	1
**30949**	0	7.39	1	0.76664	.	.	1
**30950**	0	7.39	1	0.76664	.	.	1
**30951**	0	7.39	1	0.76664	.	.	1
**30952**	0	7.39	1	0.76664	.	.	1
**30953**	0	7.39	1	0.76664	.	.	1
**30954**	0	7.39	1	0.76664	.	.	1
**30955**	0	7.39	1	0.76664	.	.	1
**30956**	0	7.39	1	0.76664	.	.	1
**30957**	0	7.39	1	0.76664	.	.	1
**30958**	0	7.39	1	0.76664	.	.	1
**30959**	0	7.39	1	0.76664	.	.	1
**30960**	0	7.39	1	0.76664	.	.	1
**30961**	0	7.39	1	0.76664	.	.	1
**30962**	0	7.39	1	0.76664	.	.	1
**30963**	0	7.39	1	0.76664	.	.	1
**30964**	0	7.39	1	0.76664	.	.	1
**30965**	0	7.39	1	0.76664	.	.	1
**30966**	0	7.40	0	0.76637	0.76123	0.77142	1
**30967**	0	7.40	1	0.76637	.	.	1
**30968**	0	7.40	1	0.76637	.	.	1
**30969**	0	7.40	1	0.76637	.	.	1
**30970**	0	7.40	1	0.76637	.	.	1
**30971**	0	7.40	1	0.76637	.	.	1
**30972**	0	7.40	1	0.76637	.	.	1
**30973**	0	7.40	1	0.76637	.	.	1
**30974**	0	7.40	1	0.76637	.	.	1
**30975**	0	7.40	1	0.76637	.	.	1

Obs	Sub_group	fuptimey	_CENSOR_	SURVIVAL	SDF_LCL	SDF_UCL	STRATUM
30976	0	7.40	1	0.76637	.	.	1
30977	0	7.40	1	0.76637	.	.	1
30978	0	7.40	1	0.76637	.	.	1
30979	0	7.40	1	0.76637	.	.	1
30980	0	7.40	1	0.76637	.	.	1
30981	0	7.40	1	0.76637	.	.	1
30982	0	7.40	1	0.76637	.	.	1
30983	0	7.40	1	0.76637	.	.	1
30984	0	7.40	1	0.76637	.	.	1
30985	0	7.40	1	0.76637	.	.	1
30986	0	7.40	1	0.76637	.	.	1
30987	0	7.40	1	0.76637	.	.	1
30988	0	7.40	1	0.76637	.	.	1
30989	0	7.40	1	0.76637	.	.	1
30990	0	7.40	1	0.76637	.	.	1
30991	0	7.40	1	0.76637	.	.	1
30992	0	7.40	1	0.76637	.	.	1
30993	0	7.40	1	0.76637	.	.	1
30994	0	7.40	1	0.76637	.	.	1
30995	0	7.40	1	0.76637	.	.	1
30996	0	7.40	1	0.76637	.	.	1
30997	0	7.40	1	0.76637	.	.	1
30998	0	7.40	1	0.76637	.	.	1
30999	0	7.40	1	0.76637	.	.	1
31000	0	7.40	1	0.76637	.	.	1
31001	0	7.40	1	0.76637	.	.	1
31002	0	7.41	0	0.76610	0.76096	0.77116	1
31003	0	7.41	1	0.76610	.	.	1
31004	0	7.41	1	0.76610	.	.	1
31005	0	7.41	1	0.76610	.	.	1
31006	0	7.41	1	0.76610	.	.	1
31007	0	7.41	1	0.76610	.	.	1
31008	0	7.41	1	0.76610	.	.	1
31009	0	7.41	1	0.76610	.	.	1
31010	0	7.41	1	0.76610	.	.	1

Obs	Sub_group	fuptimey	_CENSOR_	SURVIVAL	SDF_LCL	SDF_UCL	STRATUM
31011	0	7.41	1	0.76610	.	.	1
31012	0	7.41	1	0.76610	.	.	1
31013	0	7.41	1	0.76610	.	.	1
31014	0	7.41	1	0.76610	.	.	1
31015	0	7.41	1	0.76610	.	.	1
31016	0	7.41	1	0.76610	.	.	1
31017	0	7.41	1	0.76610	.	.	1
31018	0	7.41	1	0.76610	.	.	1
31019	0	7.41	1	0.76610	.	.	1
31020	0	7.41	1	0.76610	.	.	1
31021	0	7.41	1	0.76610	.	.	1
31022	0	7.41	1	0.76610	.	.	1
31023	0	7.41	1	0.76610	.	.	1
31024	0	7.41	1	0.76610	.	.	1
31025	0	7.41	1	0.76610	.	.	1
31026	0	7.41	1	0.76610	.	.	1
31027	0	7.41	1	0.76610	.	.	1
31028	0	7.41	1	0.76610	.	.	1
31029	0	7.41	1	0.76610	.	.	1
31030	0	7.41	1	0.76610	.	.	1
31031	0	7.41	1	0.76610	.	.	1
31032	0	7.41	1	0.76610	.	.	1
31033	0	7.41	1	0.76610	.	.	1
31034	0	7.41	1	0.76610	.	.	1
31035	0	7.41	1	0.76610	.	.	1
31036	0	7.41	1	0.76610	.	.	1
31037	0	7.41	1	0.76610	.	.	1
31038	0	7.41	1	0.76610	.	.	1
31039	0	7.41	1	0.76610	.	.	1
31040	0	7.41	1	0.76610	.	.	1
31041	0	7.41	1	0.76610	.	.	1
31042	0	7.41	1	0.76610	.	.	1
31043	0	7.42	0	0.76592	0.76077	0.77098	1
31044	0	7.42	1	0.76592	.	.	1
31045	0	7.42	1	0.76592	.	.	1

Obs	Sub_group	fuptimey	_CENSOR_	SURVIVAL	SDF_LCL	SDF_UCL	STRATUM
31046	0	7.42	1	0.76592	.	.	1
31047	0	7.42	1	0.76592	.	.	1
31048	0	7.42	1	0.76592	.	.	1
31049	0	7.42	1	0.76592	.	.	1
31050	0	7.42	1	0.76592	.	.	1
31051	0	7.42	1	0.76592	.	.	1
31052	0	7.42	1	0.76592	.	.	1
31053	0	7.42	1	0.76592	.	.	1
31054	0	7.42	1	0.76592	.	.	1
31055	0	7.42	1	0.76592	.	.	1
31056	0	7.42	1	0.76592	.	.	1
31057	0	7.42	1	0.76592	.	.	1
31058	0	7.42	1	0.76592	.	.	1
31059	0	7.42	1	0.76592	.	.	1
31060	0	7.42	1	0.76592	.	.	1
31061	0	7.42	1	0.76592	.	.	1
31062	0	7.42	1	0.76592	.	.	1
31063	0	7.42	1	0.76592	.	.	1
31064	0	7.42	1	0.76592	.	.	1
31065	0	7.42	1	0.76592	.	.	1
31066	0	7.42	1	0.76592	.	.	1
31067	0	7.42	1	0.76592	.	.	1
31068	0	7.42	1	0.76592	.	.	1
31069	0	7.42	1	0.76592	.	.	1
31070	0	7.42	1	0.76592	.	.	1
31071	0	7.42	1	0.76592	.	.	1
31072	0	7.42	1	0.76592	.	.	1
31073	0	7.42	1	0.76592	.	.	1
31074	0	7.42	1	0.76592	.	.	1
31075	0	7.42	1	0.76592	.	.	1
31076	0	7.43	1	0.76592	.	.	1
31077	0	7.43	1	0.76592	.	.	1
31078	0	7.43	1	0.76592	.	.	1
31079	0	7.43	1	0.76592	.	.	1
31080	0	7.43	1	0.76592	.	.	1

Obs	Sub_group	fuptimey	_CENSOR_	SURVIVAL	SDF_LCL	SDF_UCL	STRATUM
**31081**	0	7.43	1	0.76592	.	.	1
**31082**	0	7.43	1	0.76592	.	.	1
**31083**	0	7.43	1	0.76592	.	.	1
**31084**	0	7.43	1	0.76592	.	.	1
**31085**	0	7.43	1	0.76592	.	.	1
**31086**	0	7.43	1	0.76592	.	.	1
**31087**	0	7.43	1	0.76592	.	.	1
**31088**	0	7.43	1	0.76592	.	.	1
**31089**	0	7.43	1	0.76592	.	.	1
**31090**	0	7.43	1	0.76592	.	.	1
**31091**	0	7.43	1	0.76592	.	.	1
**31092**	0	7.43	1	0.76592	.	.	1
**31093**	0	7.43	1	0.76592	.	.	1
**31094**	0	7.43	1	0.76592	.	.	1
**31095**	0	7.43	1	0.76592	.	.	1
**31096**	0	7.43	1	0.76592	.	.	1
**31097**	0	7.43	1	0.76592	.	.	1
**31098**	0	7.43	1	0.76592	.	.	1
**31099**	0	7.43	1	0.76592	.	.	1
**31100**	0	7.43	1	0.76592	.	.	1
**31101**	0	7.43	1	0.76592	.	.	1
**31102**	0	7.43	1	0.76592	.	.	1
**31103**	0	7.43	1	0.76592	.	.	1
**31104**	0	7.43	1	0.76592	.	.	1
**31105**	0	7.43	1	0.76592	.	.	1
**31106**	0	7.43	1	0.76592	.	.	1
**31107**	0	7.43	1	0.76592	.	.	1
**31108**	0	7.43	1	0.76592	.	.	1
**31109**	0	7.43	1	0.76592	.	.	1
**31110**	0	7.44	0	0.76583	0.76068	0.77090	1
**31111**	0	7.44	1	0.76583	.	.	1
**31112**	0	7.44	1	0.76583	.	.	1
**31113**	0	7.44	1	0.76583	.	.	1
**31114**	0	7.44	1	0.76583	.	.	1
**31115**	0	7.44	1	0.76583	.	.	1

Obs	Sub_group	fuptimey	_CENSOR_	SURVIVAL	SDF_LCL	SDF_UCL	STRATUM
**31116**	0	7.44	1	0.76583	.	.	1
**31117**	0	7.44	1	0.76583	.	.	1
**31118**	0	7.44	1	0.76583	.	.	1
**31119**	0	7.44	1	0.76583	.	.	1
**31120**	0	7.44	1	0.76583	.	.	1
**31121**	0	7.44	1	0.76583	.	.	1
**31122**	0	7.44	1	0.76583	.	.	1
**31123**	0	7.44	1	0.76583	.	.	1
**31124**	0	7.44	1	0.76583	.	.	1
**31125**	0	7.44	1	0.76583	.	.	1
**31126**	0	7.44	1	0.76583	.	.	1
**31127**	0	7.44	1	0.76583	.	.	1
**31128**	0	7.44	1	0.76583	.	.	1
**31129**	0	7.44	1	0.76583	.	.	1
**31130**	0	7.44	1	0.76583	.	.	1
**31131**	0	7.44	1	0.76583	.	.	1
**31132**	0	7.44	1	0.76583	.	.	1
**31133**	0	7.44	1	0.76583	.	.	1
**31134**	0	7.44	1	0.76583	.	.	1
**31135**	0	7.44	1	0.76583	.	.	1
**31136**	0	7.44	1	0.76583	.	.	1
**31137**	0	7.44	1	0.76583	.	.	1
**31138**	0	7.44	1	0.76583	.	.	1
**31139**	0	7.44	1	0.76583	.	.	1
**31140**	0	7.44	1	0.76583	.	.	1
**31141**	0	7.44	1	0.76583	.	.	1
**31142**	0	7.44	1	0.76583	.	.	1
**31143**	0	7.44	1	0.76583	.	.	1
**31144**	0	7.44	1	0.76583	.	.	1
**31145**	0	7.44	1	0.76583	.	.	1
**31146**	0	7.44	1	0.76583	.	.	1
**31147**	0	7.44	1	0.76583	.	.	1
**31148**	0	7.44	1	0.76583	.	.	1
**31149**	0	7.44	1	0.76583	.	.	1
**31150**	0	7.44	1	0.76583	.	.	1

Obs	Sub_group	fuptimey	_CENSOR_	SURVIVAL	SDF_LCL	SDF_UCL	STRATUM
31151	0	7.44	1	0.76583	.	.	1
31152	0	7.44	1	0.76583	.	.	1
31153	0	7.45	0	0.76565	0.76049	0.77072	1
31154	0	7.45	1	0.76565	.	.	1
31155	0	7.45	1	0.76565	.	.	1
31156	0	7.45	1	0.76565	.	.	1
31157	0	7.45	1	0.76565	.	.	1
31158	0	7.45	1	0.76565	.	.	1
31159	0	7.45	1	0.76565	.	.	1
31160	0	7.45	1	0.76565	.	.	1
31161	0	7.45	1	0.76565	.	.	1
31162	0	7.45	1	0.76565	.	.	1
31163	0	7.45	1	0.76565	.	.	1
31164	0	7.45	1	0.76565	.	.	1
31165	0	7.45	1	0.76565	.	.	1
31166	0	7.45	1	0.76565	.	.	1
31167	0	7.45	1	0.76565	.	.	1
31168	0	7.45	1	0.76565	.	.	1
31169	0	7.45	1	0.76565	.	.	1
31170	0	7.45	1	0.76565	.	.	1
31171	0	7.45	1	0.76565	.	.	1
31172	0	7.45	1	0.76565	.	.	1
31173	0	7.45	1	0.76565	.	.	1
31174	0	7.45	1	0.76565	.	.	1
31175	0	7.45	1	0.76565	.	.	1
31176	0	7.45	1	0.76565	.	.	1
31177	0	7.45	1	0.76565	.	.	1
31178	0	7.45	1	0.76565	.	.	1
31179	0	7.45	1	0.76565	.	.	1
31180	0	7.45	1	0.76565	.	.	1
31181	0	7.45	1	0.76565	.	.	1
31182	0	7.45	1	0.76565	.	.	1
31183	0	7.45	1	0.76565	.	.	1
31184	0	7.45	1	0.76565	.	.	1
31185	0	7.45	1	0.76565	.	.	1

Obs	Sub_group	fuptimey	_CENSOR_	SURVIVAL	SDF_LCL	SDF_UCL	STRATUM
31186	0	7.45	1	0.76565	.	.	1
31187	0	7.45	1	0.76565	.	.	1
31188	0	7.45	1	0.76565	.	.	1
31189	0	7.45	1	0.76565	.	.	1
31190	0	7.45	1	0.76565	.	.	1
31191	0	7.45	1	0.76565	.	.	1
31192	0	7.45	1	0.76565	.	.	1
31193	0	7.45	1	0.76565	.	.	1
31194	0	7.45	1	0.76565	.	.	1
31195	0	7.45	1	0.76565	.	.	1
31196	0	7.45	1	0.76565	.	.	1
31197	0	7.46	0	0.76538	0.76021	0.77045	1
31198	0	7.46	1	0.76538	.	.	1
31199	0	7.46	1	0.76538	.	.	1
31200	0	7.46	1	0.76538	.	.	1
31201	0	7.46	1	0.76538	.	.	1
31202	0	7.46	1	0.76538	.	.	1
31203	0	7.46	1	0.76538	.	.	1
31204	0	7.46	1	0.76538	.	.	1
31205	0	7.46	1	0.76538	.	.	1
31206	0	7.46	1	0.76538	.	.	1
31207	0	7.46	1	0.76538	.	.	1
31208	0	7.46	1	0.76538	.	.	1
31209	0	7.46	1	0.76538	.	.	1
31210	0	7.46	1	0.76538	.	.	1
31211	0	7.46	1	0.76538	.	.	1
31212	0	7.46	1	0.76538	.	.	1
31213	0	7.46	1	0.76538	.	.	1
31214	0	7.46	1	0.76538	.	.	1
31215	0	7.46	1	0.76538	.	.	1
31216	0	7.46	1	0.76538	.	.	1
31217	0	7.46	1	0.76538	.	.	1
31218	0	7.46	1	0.76538	.	.	1
31219	0	7.46	1	0.76538	.	.	1
31220	0	7.46	1	0.76538	.	.	1

Obs	Sub_group	fuptimey	_CENSOR_	SURVIVAL	SDF_LCL	SDF_UCL	STRATUM
31221	0	7.46	1	0.76538	.	.	1
31222	0	7.46	1	0.76538	.	.	1
31223	0	7.46	1	0.76538	.	.	1
31224	0	7.46	1	0.76538	.	.	1
31225	0	7.46	1	0.76538	.	.	1
31226	0	7.46	1	0.76538	.	.	1
31227	0	7.46	1	0.76538	.	.	1
31228	0	7.46	1	0.76538	.	.	1
31229	0	7.46	1	0.76538	.	.	1
31230	0	7.47	0	0.76529	0.76012	0.77036	1
31231	0	7.47	1	0.76529	.	.	1
31232	0	7.47	1	0.76529	.	.	1
31233	0	7.47	1	0.76529	.	.	1
31234	0	7.47	1	0.76529	.	.	1
31235	0	7.47	1	0.76529	.	.	1
31236	0	7.47	1	0.76529	.	.	1
31237	0	7.47	1	0.76529	.	.	1
31238	0	7.47	1	0.76529	.	.	1
31239	0	7.47	1	0.76529	.	.	1
31240	0	7.47	1	0.76529	.	.	1
31241	0	7.47	1	0.76529	.	.	1
31242	0	7.47	1	0.76529	.	.	1
31243	0	7.47	1	0.76529	.	.	1
31244	0	7.47	1	0.76529	.	.	1
31245	0	7.47	1	0.76529	.	.	1
31246	0	7.47	1	0.76529	.	.	1
31247	0	7.47	1	0.76529	.	.	1
31248	0	7.47	1	0.76529	.	.	1
31249	0	7.47	1	0.76529	.	.	1
31250	0	7.47	1	0.76529	.	.	1
31251	0	7.47	1	0.76529	.	.	1
31252	0	7.47	1	0.76529	.	.	1
31253	0	7.47	1	0.76529	.	.	1
31254	0	7.47	1	0.76529	.	.	1
31255	0	7.47	1	0.76529	.	.	1

Obs	Sub_group	fuptimey	_CENSOR_	SURVIVAL	SDF_LCL	SDF_UCL	STRATUM
31256	0	7.47	1	0.76529	.	.	1
31257	0	7.47	1	0.76529	.	.	1
31258	0	7.47	1	0.76529	.	.	1
31259	0	7.47	1	0.76529	.	.	1
31260	0	7.47	1	0.76529	.	.	1
31261	0	7.47	1	0.76529	.	.	1
31262	0	7.48	0	0.76510	0.75993	0.77019	1
31263	0	7.48	1	0.76510	.	.	1
31264	0	7.48	1	0.76510	.	.	1
31265	0	7.48	1	0.76510	.	.	1
31266	0	7.48	1	0.76510	.	.	1
31267	0	7.48	1	0.76510	.	.	1
31268	0	7.48	1	0.76510	.	.	1
31269	0	7.48	1	0.76510	.	.	1
31270	0	7.48	1	0.76510	.	.	1
31271	0	7.48	1	0.76510	.	.	1
31272	0	7.48	1	0.76510	.	.	1
31273	0	7.48	1	0.76510	.	.	1
31274	0	7.48	1	0.76510	.	.	1
31275	0	7.48	1	0.76510	.	.	1
31276	0	7.48	1	0.76510	.	.	1
31277	0	7.48	1	0.76510	.	.	1
31278	0	7.48	1	0.76510	.	.	1
31279	0	7.48	1	0.76510	.	.	1
31280	0	7.48	1	0.76510	.	.	1
31281	0	7.48	1	0.76510	.	.	1
31282	0	7.48	1	0.76510	.	.	1
31283	0	7.48	1	0.76510	.	.	1
31284	0	7.48	1	0.76510	.	.	1
31285	0	7.48	1	0.76510	.	.	1
31286	0	7.48	1	0.76510	.	.	1
31287	0	7.48	1	0.76510	.	.	1
31288	0	7.48	1	0.76510	.	.	1
31289	0	7.48	1	0.76510	.	.	1
31290	0	7.48	1	0.76510	.	.	1

Obs	Sub_group	fuptimey	_CENSOR_	SURVIVAL	SDF_LCL	SDF_UCL	STRATUM
31291	0	7.48	1	0.76510	.	.	1
31292	0	7.48	1	0.76510	.	.	1
31293	0	7.48	1	0.76510	.	.	1
31294	0	7.48	1	0.76510	.	.	1
31295	0	7.48	1	0.76510	.	.	1
31296	0	7.48	1	0.76510	.	.	1
31297	0	7.48	1	0.76510	.	.	1
31298	0	7.48	1	0.76510	.	.	1
31299	0	7.48	1	0.76510	.	.	1
31300	0	7.49	1	0.76510	.	.	1
31301	0	7.49	1	0.76510	.	.	1
31302	0	7.49	1	0.76510	.	.	1
31303	0	7.49	1	0.76510	.	.	1
31304	0	7.49	1	0.76510	.	.	1
31305	0	7.49	1	0.76510	.	.	1
31306	0	7.49	1	0.76510	.	.	1
31307	0	7.49	1	0.76510	.	.	1
31308	0	7.49	1	0.76510	.	.	1
31309	0	7.49	1	0.76510	.	.	1
31310	0	7.49	1	0.76510	.	.	1
31311	0	7.49	1	0.76510	.	.	1
31312	0	7.49	1	0.76510	.	.	1
31313	0	7.49	1	0.76510	.	.	1
31314	0	7.49	1	0.76510	.	.	1
31315	0	7.49	1	0.76510	.	.	1
31316	0	7.49	1	0.76510	.	.	1
31317	0	7.49	1	0.76510	.	.	1
31318	0	7.49	1	0.76510	.	.	1
31319	0	7.49	1	0.76510	.	.	1
31320	0	7.49	1	0.76510	.	.	1
31321	0	7.49	1	0.76510	.	.	1
31322	0	7.49	1	0.76510	.	.	1
31323	0	7.49	1	0.76510	.	.	1
31324	0	7.49	1	0.76510	.	.	1
31325	0	7.49	1	0.76510	.	.	1

Obs	Sub_group	fuptimey	_CENSOR_	SURVIVAL	SDF_LCL	SDF_UCL	STRATUM
31326	0	7.50	0	0.76492	0.75974	0.77000	1
31327	0	7.50	1	0.76492	.	.	1
31328	0	7.50	1	0.76492	.	.	1
31329	0	7.50	1	0.76492	.	.	1
31330	0	7.50	1	0.76492	.	.	1
31331	0	7.50	1	0.76492	.	.	1
31332	0	7.50	1	0.76492	.	.	1
31333	0	7.50	1	0.76492	.	.	1
31334	0	7.50	1	0.76492	.	.	1
31335	0	7.50	1	0.76492	.	.	1
31336	0	7.50	1	0.76492	.	.	1
31337	0	7.50	1	0.76492	.	.	1
31338	0	7.50	1	0.76492	.	.	1
31339	0	7.50	1	0.76492	.	.	1
31340	0	7.50	1	0.76492	.	.	1
31341	0	7.50	1	0.76492	.	.	1
31342	0	7.50	1	0.76492	.	.	1
31343	0	7.50	1	0.76492	.	.	1
31344	0	7.50	1	0.76492	.	.	1
31345	0	7.50	1	0.76492	.	.	1
31346	0	7.50	1	0.76492	.	.	1
31347	0	7.50	1	0.76492	.	.	1
31348	0	7.50	1	0.76492	.	.	1
31349	0	7.50	1	0.76492	.	.	1
31350	0	7.50	1	0.76492	.	.	1
31351	0	7.50	1	0.76492	.	.	1
31352	0	7.50	1	0.76492	.	.	1
31353	0	7.50	1	0.76492	.	.	1
31354	0	7.50	1	0.76492	.	.	1
31355	0	7.50	1	0.76492	.	.	1
31356	0	7.50	1	0.76492	.	.	1
31357	0	7.50	1	0.76492	.	.	1
31358	0	7.50	1	0.76492	.	.	1
31359	0	7.50	1	0.76492	.	.	1
31360	0	7.50	1	0.76492	.	.	1

Obs	Sub_group	fuptimey	_CENSOR_	SURVIVAL	SDF_LCL	SDF_UCL	STRATUM
31361	0	7.50	1	0.76492	.	.	1
31362	0	7.50	1	0.76492	.	.	1
31363	0	7.50	1	0.76492	.	.	1
31364	0	7.50	1	0.76492	.	.	1
31365	0	7.50	1	0.76492	.	.	1
31366	0	7.50	1	0.76492	.	.	1
31367	0	7.51	0	0.76464	0.75945	0.76973	1
31368	0	7.51	1	0.76464	.	.	1
31369	0	7.51	1	0.76464	.	.	1
31370	0	7.51	1	0.76464	.	.	1
31371	0	7.51	1	0.76464	.	.	1
31372	0	7.51	1	0.76464	.	.	1
31373	0	7.51	1	0.76464	.	.	1
31374	0	7.51	1	0.76464	.	.	1
31375	0	7.51	1	0.76464	.	.	1
31376	0	7.51	1	0.76464	.	.	1
31377	0	7.51	1	0.76464	.	.	1
31378	0	7.51	1	0.76464	.	.	1
31379	0	7.51	1	0.76464	.	.	1
31380	0	7.51	1	0.76464	.	.	1
31381	0	7.51	1	0.76464	.	.	1
31382	0	7.51	1	0.76464	.	.	1
31383	0	7.51	1	0.76464	.	.	1
31384	0	7.51	1	0.76464	.	.	1
31385	0	7.51	1	0.76464	.	.	1
31386	0	7.51	1	0.76464	.	.	1
31387	0	7.51	1	0.76464	.	.	1
31388	0	7.51	1	0.76464	.	.	1
31389	0	7.51	1	0.76464	.	.	1
31390	0	7.51	1	0.76464	.	.	1
31391	0	7.51	1	0.76464	.	.	1
31392	0	7.51	1	0.76464	.	.	1
31393	0	7.51	1	0.76464	.	.	1
31394	0	7.51	1	0.76464	.	.	1
31395	0	7.51	1	0.76464	.	.	1

Obs	Sub_group	fuptimey	_CENSOR_	SURVIVAL	SDF_LCL	SDF_UCL	STRATUM
31396	0	7.51	1	0.76464	.	.	1
31397	0	7.51	1	0.76464	.	.	1
31398	0	7.51	1	0.76464	.	.	1
31399	0	7.51	1	0.76464	.	.	1
31400	0	7.51	1	0.76464	.	.	1
31401	0	7.51	1	0.76464	.	.	1
31402	0	7.51	1	0.76464	.	.	1
31403	0	7.51	1	0.76464	.	.	1
31404	0	7.51	1	0.76464	.	.	1
31405	0	7.51	1	0.76464	.	.	1
31406	0	7.51	1	0.76464	.	.	1
31407	0	7.52	0	0.76417	0.75897	0.76928	1
31408	0	7.52	1	0.76417	.	.	1
31409	0	7.52	1	0.76417	.	.	1
31410	0	7.52	1	0.76417	.	.	1
31411	0	7.52	1	0.76417	.	.	1
31412	0	7.52	1	0.76417	.	.	1
31413	0	7.52	1	0.76417	.	.	1
31414	0	7.52	1	0.76417	.	.	1
31415	0	7.52	1	0.76417	.	.	1
31416	0	7.52	1	0.76417	.	.	1
31417	0	7.52	1	0.76417	.	.	1
31418	0	7.52	1	0.76417	.	.	1
31419	0	7.52	1	0.76417	.	.	1
31420	0	7.52	1	0.76417	.	.	1
31421	0	7.52	1	0.76417	.	.	1
31422	0	7.52	1	0.76417	.	.	1
31423	0	7.52	1	0.76417	.	.	1
31424	0	7.52	1	0.76417	.	.	1
31425	0	7.52	1	0.76417	.	.	1
31426	0	7.52	1	0.76417	.	.	1
31427	0	7.52	1	0.76417	.	.	1
31428	0	7.52	1	0.76417	.	.	1
31429	0	7.52	1	0.76417	.	.	1
31430	0	7.52	1	0.76417	.	.	1

Obs	Sub_group	fuptimey	_CENSOR_	SURVIVAL	SDF_LCL	SDF_UCL	STRATUM
31431	0	7.52	1	0.76417	.	.	1
31432	0	7.52	1	0.76417	.	.	1
31433	0	7.52	1	0.76417	.	.	1
31434	0	7.52	1	0.76417	.	.	1
31435	0	7.52	1	0.76417	.	.	1
31436	0	7.52	1	0.76417	.	.	1
31437	0	7.52	1	0.76417	.	.	1
31438	0	7.52	1	0.76417	.	.	1
31439	0	7.52	1	0.76417	.	.	1
31440	0	7.52	1	0.76417	.	.	1
31441	0	7.52	1	0.76417	.	.	1
31442	0	7.52	1	0.76417	.	.	1
31443	0	7.52	1	0.76417	.	.	1
31444	0	7.52	1	0.76417	.	.	1
31445	0	7.52	1	0.76417	.	.	1
31446	0	7.52	1	0.76417	.	.	1
31447	0	7.52	1	0.76417	.	.	1
31448	0	7.52	1	0.76417	.	.	1
31449	0	7.53	0	0.76389	0.75868	0.76900	1
31450	0	7.53	1	0.76389	.	.	1
31451	0	7.53	1	0.76389	.	.	1
31452	0	7.53	1	0.76389	.	.	1
31453	0	7.53	1	0.76389	.	.	1
31454	0	7.53	1	0.76389	.	.	1
31455	0	7.53	1	0.76389	.	.	1
31456	0	7.53	1	0.76389	.	.	1
31457	0	7.53	1	0.76389	.	.	1
31458	0	7.53	1	0.76389	.	.	1
31459	0	7.53	1	0.76389	.	.	1
31460	0	7.53	1	0.76389	.	.	1
31461	0	7.53	1	0.76389	.	.	1
31462	0	7.53	1	0.76389	.	.	1
31463	0	7.53	1	0.76389	.	.	1
31464	0	7.53	1	0.76389	.	.	1
31465	0	7.53	1	0.76389	.	.	1

Obs	Sub_group	fuptimey	_CENSOR_	SURVIVAL	SDF_LCL	SDF_UCL	STRATUM
31466	0	7.53	1	0.76389	.	.	1
31467	0	7.53	1	0.76389	.	.	1
31468	0	7.53	1	0.76389	.	.	1
31469	0	7.53	1	0.76389	.	.	1
31470	0	7.53	1	0.76389	.	.	1
31471	0	7.53	1	0.76389	.	.	1
31472	0	7.53	1	0.76389	.	.	1
31473	0	7.53	1	0.76389	.	.	1
31474	0	7.53	1	0.76389	.	.	1
31475	0	7.53	1	0.76389	.	.	1
31476	0	7.53	1	0.76389	.	.	1
31477	0	7.53	1	0.76389	.	.	1
31478	0	7.53	1	0.76389	.	.	1
31479	0	7.53	1	0.76389	.	.	1
31480	0	7.53	1	0.76389	.	.	1
31481	0	7.53	1	0.76389	.	.	1
31482	0	7.53	1	0.76389	.	.	1
31483	0	7.54	0	0.76370	0.75848	0.76882	1
31484	0	7.54	1	0.76370	.	.	1
31485	0	7.54	1	0.76370	.	.	1
31486	0	7.54	1	0.76370	.	.	1
31487	0	7.54	1	0.76370	.	.	1
31488	0	7.54	1	0.76370	.	.	1
31489	0	7.54	1	0.76370	.	.	1
31490	0	7.54	1	0.76370	.	.	1
31491	0	7.54	1	0.76370	.	.	1
31492	0	7.54	1	0.76370	.	.	1
31493	0	7.54	1	0.76370	.	.	1
31494	0	7.54	1	0.76370	.	.	1
31495	0	7.54	1	0.76370	.	.	1
31496	0	7.54	1	0.76370	.	.	1
31497	0	7.54	1	0.76370	.	.	1
31498	0	7.54	1	0.76370	.	.	1
31499	0	7.54	1	0.76370	.	.	1
31500	0	7.54	1	0.76370	.	.	1

Obs	Sub_group	fuptimey	_CENSOR_	SURVIVAL	SDF_LCL	SDF_UCL	STRATUM
31501	0	7.54	1	0.76370	.	.	1
31502	0	7.54	1	0.76370	.	.	1
31503	0	7.54	1	0.76370	.	.	1
31504	0	7.54	1	0.76370	.	.	1
31505	0	7.54	1	0.76370	.	.	1
31506	0	7.54	1	0.76370	.	.	1
31507	0	7.54	1	0.76370	.	.	1
31508	0	7.54	1	0.76370	.	.	1
31509	0	7.54	1	0.76370	.	.	1
31510	0	7.54	1	0.76370	.	.	1
31511	0	7.54	1	0.76370	.	.	1
31512	0	7.54	1	0.76370	.	.	1
31513	0	7.54	1	0.76370	.	.	1
31514	0	7.54	1	0.76370	.	.	1
31515	0	7.54	1	0.76370	.	.	1
31516	0	7.54	1	0.76370	.	.	1
31517	0	7.54	1	0.76370	.	.	1
31518	0	7.55	0	0.76360	0.75838	0.76873	1
31519	0	7.55	1	0.76360	.	.	1
31520	0	7.55	1	0.76360	.	.	1
31521	0	7.55	1	0.76360	.	.	1
31522	0	7.55	1	0.76360	.	.	1
31523	0	7.55	1	0.76360	.	.	1
31524	0	7.55	1	0.76360	.	.	1
31525	0	7.55	1	0.76360	.	.	1
31526	0	7.55	1	0.76360	.	.	1
31527	0	7.55	1	0.76360	.	.	1
31528	0	7.55	1	0.76360	.	.	1
31529	0	7.55	1	0.76360	.	.	1
31530	0	7.55	1	0.76360	.	.	1
31531	0	7.55	1	0.76360	.	.	1
31532	0	7.55	1	0.76360	.	.	1
31533	0	7.55	1	0.76360	.	.	1
31534	0	7.55	1	0.76360	.	.	1
31535	0	7.55	1	0.76360	.	.	1

Obs	Sub_group	fuptimey	_CENSOR_	SURVIVAL	SDF_LCL	SDF_UCL	STRATUM
31536	0	7.55	1	0.76360	.	.	1
31537	0	7.55	1	0.76360	.	.	1
31538	0	7.55	1	0.76360	.	.	1
31539	0	7.55	1	0.76360	.	.	1
31540	0	7.55	1	0.76360	.	.	1
31541	0	7.55	1	0.76360	.	.	1
31542	0	7.56	0	0.76341	0.75819	0.76854	1
31543	0	7.56	1	0.76341	.	.	1
31544	0	7.56	1	0.76341	.	.	1
31545	0	7.56	1	0.76341	.	.	1
31546	0	7.56	1	0.76341	.	.	1
31547	0	7.56	1	0.76341	.	.	1
31548	0	7.56	1	0.76341	.	.	1
31549	0	7.56	1	0.76341	.	.	1
31550	0	7.56	1	0.76341	.	.	1
31551	0	7.56	1	0.76341	.	.	1
31552	0	7.56	1	0.76341	.	.	1
31553	0	7.56	1	0.76341	.	.	1
31554	0	7.56	1	0.76341	.	.	1
31555	0	7.56	1	0.76341	.	.	1
31556	0	7.56	1	0.76341	.	.	1
31557	0	7.56	1	0.76341	.	.	1
31558	0	7.56	1	0.76341	.	.	1
31559	0	7.56	1	0.76341	.	.	1
31560	0	7.56	1	0.76341	.	.	1
31561	0	7.56	1	0.76341	.	.	1
31562	0	7.56	1	0.76341	.	.	1
31563	0	7.56	1	0.76341	.	.	1
31564	0	7.56	1	0.76341	.	.	1
31565	0	7.56	1	0.76341	.	.	1
31566	0	7.56	1	0.76341	.	.	1
31567	0	7.56	1	0.76341	.	.	1
31568	0	7.56	1	0.76341	.	.	1
31569	0	7.56	1	0.76341	.	.	1
31570	0	7.56	1	0.76341	.	.	1

Obs	Sub_group	fuptimey	_CENSOR_	SURVIVAL	SDF_LCL	SDF_UCL	STRATUM
31571	0	7.56	1	0.76341	.	.	1
31572	0	7.56	1	0.76341	.	.	1
31573	0	7.56	1	0.76341	.	.	1
31574	0	7.56	1	0.76341	.	.	1
31575	0	7.56	1	0.76341	.	.	1
31576	0	7.56	1	0.76341	.	.	1
31577	0	7.56	1	0.76341	.	.	1
31578	0	7.56	1	0.76341	.	.	1
31579	0	7.56	1	0.76341	.	.	1
31580	0	7.56	1	0.76341	.	.	1
31581	0	7.56	1	0.76341	.	.	1
31582	0	7.56	1	0.76341	.	.	1
31583	0	7.56	1	0.76341	.	.	1
31584	0	7.57	0	0.76313	0.75789	0.76826	1
31585	0	7.57	1	0.76313	.	.	1
31586	0	7.57	1	0.76313	.	.	1
31587	0	7.57	1	0.76313	.	.	1
31588	0	7.57	1	0.76313	.	.	1
31589	0	7.57	1	0.76313	.	.	1
31590	0	7.57	1	0.76313	.	.	1
31591	0	7.57	1	0.76313	.	.	1
31592	0	7.57	1	0.76313	.	.	1
31593	0	7.57	1	0.76313	.	.	1
31594	0	7.57	1	0.76313	.	.	1
31595	0	7.57	1	0.76313	.	.	1
31596	0	7.57	1	0.76313	.	.	1
31597	0	7.57	1	0.76313	.	.	1
31598	0	7.57	1	0.76313	.	.	1
31599	0	7.57	1	0.76313	.	.	1
31600	0	7.57	1	0.76313	.	.	1
31601	0	7.57	1	0.76313	.	.	1
31602	0	7.57	1	0.76313	.	.	1
31603	0	7.57	1	0.76313	.	.	1
31604	0	7.57	1	0.76313	.	.	1
31605	0	7.57	1	0.76313	.	.	1

Obs	Sub_group	fuptimey	_CENSOR_	SURVIVAL	SDF_LCL	SDF_UCL	STRATUM
31606	0	7.57	1	0.76313	.	.	1
31607	0	7.58	0	0.76293	0.75769	0.76808	1
31608	0	7.58	1	0.76293	.	.	1
31609	0	7.58	1	0.76293	.	.	1
31610	0	7.58	1	0.76293	.	.	1
31611	0	7.58	1	0.76293	.	.	1
31612	0	7.58	1	0.76293	.	.	1
31613	0	7.58	1	0.76293	.	.	1
31614	0	7.58	1	0.76293	.	.	1
31615	0	7.58	1	0.76293	.	.	1
31616	0	7.58	1	0.76293	.	.	1
31617	0	7.58	1	0.76293	.	.	1
31618	0	7.58	1	0.76293	.	.	1
31619	0	7.58	1	0.76293	.	.	1
31620	0	7.58	1	0.76293	.	.	1
31621	0	7.58	1	0.76293	.	.	1
31622	0	7.58	1	0.76293	.	.	1
31623	0	7.58	1	0.76293	.	.	1
31624	0	7.58	1	0.76293	.	.	1
31625	0	7.58	1	0.76293	.	.	1
31626	0	7.58	1	0.76293	.	.	1
31627	0	7.58	1	0.76293	.	.	1
31628	0	7.58	1	0.76293	.	.	1
31629	0	7.58	1	0.76293	.	.	1
31630	0	7.58	1	0.76293	.	.	1
31631	0	7.58	1	0.76293	.	.	1
31632	0	7.58	1	0.76293	.	.	1
31633	0	7.58	1	0.76293	.	.	1
31634	0	7.58	1	0.76293	.	.	1
31635	0	7.58	1	0.76293	.	.	1
31636	0	7.58	1	0.76293	.	.	1
31637	0	7.58	1	0.76293	.	.	1
31638	0	7.58	1	0.76293	.	.	1
31639	0	7.58	1	0.76293	.	.	1
31640	0	7.58	1	0.76293	.	.	1

Obs	Sub_group	fuptimey	_CENSOR_	SURVIVAL	SDF_LCL	SDF_UCL	STRATUM
31641	0	7.58	1	0.76293	.	.	1
31642	0	7.58	1	0.76293	.	.	1
31643	0	7.58	1	0.76293	.	.	1
31644	0	7.58	1	0.76293	.	.	1
31645	0	7.58	1	0.76293	.	.	1
31646	0	7.59	0	0.76274	0.75750	0.76789	1
31647	0	7.59	1	0.76274	.	.	1
31648	0	7.59	1	0.76274	.	.	1
31649	0	7.59	1	0.76274	.	.	1
31650	0	7.59	1	0.76274	.	.	1
31651	0	7.59	1	0.76274	.	.	1
31652	0	7.59	1	0.76274	.	.	1
31653	0	7.59	1	0.76274	.	.	1
31654	0	7.59	1	0.76274	.	.	1
31655	0	7.59	1	0.76274	.	.	1
31656	0	7.59	1	0.76274	.	.	1
31657	0	7.59	1	0.76274	.	.	1
31658	0	7.59	1	0.76274	.	.	1
31659	0	7.59	1	0.76274	.	.	1
31660	0	7.59	1	0.76274	.	.	1
31661	0	7.59	1	0.76274	.	.	1
31662	0	7.59	1	0.76274	.	.	1
31663	0	7.59	1	0.76274	.	.	1
31664	0	7.59	1	0.76274	.	.	1
31665	0	7.59	1	0.76274	.	.	1
31666	0	7.59	1	0.76274	.	.	1
31667	0	7.59	1	0.76274	.	.	1
31668	0	7.59	1	0.76274	.	.	1
31669	0	7.59	1	0.76274	.	.	1
31670	0	7.59	1	0.76274	.	.	1
31671	0	7.59	1	0.76274	.	.	1
31672	0	7.59	1	0.76274	.	.	1
31673	0	7.59	1	0.76274	.	.	1
31674	0	7.60	0	0.76245	0.75720	0.76761	1
31675	0	7.60	1	0.76245	.	.	1

Obs	Sub_group	fuptimey	_CENSOR_	SURVIVAL	SDF_LCL	SDF_UCL	STRATUM
31676	0	7.60	1	0.76245	.	.	1
31677	0	7.60	1	0.76245	.	.	1
31678	0	7.60	1	0.76245	.	.	1
31679	0	7.60	1	0.76245	.	.	1
31680	0	7.60	1	0.76245	.	.	1
31681	0	7.60	1	0.76245	.	.	1
31682	0	7.60	1	0.76245	.	.	1
31683	0	7.60	1	0.76245	.	.	1
31684	0	7.60	1	0.76245	.	.	1
31685	0	7.60	1	0.76245	.	.	1
31686	0	7.60	1	0.76245	.	.	1
31687	0	7.60	1	0.76245	.	.	1
31688	0	7.60	1	0.76245	.	.	1
31689	0	7.60	1	0.76245	.	.	1
31690	0	7.60	1	0.76245	.	.	1
31691	0	7.60	1	0.76245	.	.	1
31692	0	7.60	1	0.76245	.	.	1
31693	0	7.60	1	0.76245	.	.	1
31694	0	7.60	1	0.76245	.	.	1
31695	0	7.60	1	0.76245	.	.	1
31696	0	7.60	1	0.76245	.	.	1
31697	0	7.60	1	0.76245	.	.	1
31698	0	7.60	1	0.76245	.	.	1
31699	0	7.60	1	0.76245	.	.	1
31700	0	7.60	1	0.76245	.	.	1
31701	0	7.60	1	0.76245	.	.	1
31702	0	7.60	1	0.76245	.	.	1
31703	0	7.60	1	0.76245	.	.	1
31704	0	7.60	1	0.76245	.	.	1
31705	0	7.60	1	0.76245	.	.	1
31706	0	7.60	1	0.76245	.	.	1
31707	0	7.60	1	0.76245	.	.	1
31708	0	7.60	1	0.76245	.	.	1
31709	0	7.60	1	0.76245	.	.	1
31710	0	7.60	1	0.76245	.	.	1

Obs	Sub_group	fuptimey	_CENSOR_	SURVIVAL	SDF_LCL	SDF_UCL	STRATUM
**31711**	0	7.61	0	0.76235	0.75710	0.76751	1
**31712**	0	7.61	1	0.76235	.	.	1
**31713**	0	7.61	1	0.76235	.	.	1
**31714**	0	7.61	1	0.76235	.	.	1
**31715**	0	7.61	1	0.76235	.	.	1
**31716**	0	7.61	1	0.76235	.	.	1
**31717**	0	7.61	1	0.76235	.	.	1
**31718**	0	7.61	1	0.76235	.	.	1
**31719**	0	7.61	1	0.76235	.	.	1
**31720**	0	7.61	1	0.76235	.	.	1
**31721**	0	7.61	1	0.76235	.	.	1
**31722**	0	7.61	1	0.76235	.	.	1
**31723**	0	7.61	1	0.76235	.	.	1
**31724**	0	7.61	1	0.76235	.	.	1
**31725**	0	7.61	1	0.76235	.	.	1
**31726**	0	7.61	1	0.76235	.	.	1
**31727**	0	7.61	1	0.76235	.	.	1
**31728**	0	7.61	1	0.76235	.	.	1
**31729**	0	7.61	1	0.76235	.	.	1
**31730**	0	7.61	1	0.76235	.	.	1
**31731**	0	7.61	1	0.76235	.	.	1
**31732**	0	7.61	1	0.76235	.	.	1
**31733**	0	7.61	1	0.76235	.	.	1
**31734**	0	7.61	1	0.76235	.	.	1
**31735**	0	7.61	1	0.76235	.	.	1
**31736**	0	7.61	1	0.76235	.	.	1
**31737**	0	7.61	1	0.76235	.	.	1
**31738**	0	7.61	1	0.76235	.	.	1
**31739**	0	7.61	1	0.76235	.	.	1
**31740**	0	7.61	1	0.76235	.	.	1
**31741**	0	7.61	1	0.76235	.	.	1
**31742**	0	7.61	1	0.76235	.	.	1
**31743**	0	7.61	1	0.76235	.	.	1
**31744**	0	7.61	1	0.76235	.	.	1
**31745**	0	7.61	1	0.76235	.	.	1

Obs	Sub_group	fuptimey	_CENSOR_	SURVIVAL	SDF_LCL	SDF_UCL	STRATUM
31746	0	7.61	1	0.76235	.	.	1
31747	0	7.61	1	0.76235	.	.	1
31748	0	7.61	1	0.76235	.	.	1
31749	0	7.61	1	0.76235	.	.	1
31750	0	7.61	1	0.76235	.	.	1
31751	0	7.61	1	0.76235	.	.	1
31752	0	7.62	0	0.76226	0.75700	0.76742	1
31753	0	7.62	1	0.76226	.	.	1
31754	0	7.62	1	0.76226	.	.	1
31755	0	7.62	1	0.76226	.	.	1
31756	0	7.62	1	0.76226	.	.	1
31757	0	7.62	1	0.76226	.	.	1
31758	0	7.62	1	0.76226	.	.	1
31759	0	7.62	1	0.76226	.	.	1
31760	0	7.62	1	0.76226	.	.	1
31761	0	7.62	1	0.76226	.	.	1
31762	0	7.62	1	0.76226	.	.	1
31763	0	7.62	1	0.76226	.	.	1
31764	0	7.62	1	0.76226	.	.	1
31765	0	7.62	1	0.76226	.	.	1
31766	0	7.62	1	0.76226	.	.	1
31767	0	7.62	1	0.76226	.	.	1
31768	0	7.62	1	0.76226	.	.	1
31769	0	7.62	1	0.76226	.	.	1
31770	0	7.62	1	0.76226	.	.	1
31771	0	7.62	1	0.76226	.	.	1
31772	0	7.62	1	0.76226	.	.	1
31773	0	7.62	1	0.76226	.	.	1
31774	0	7.62	1	0.76226	.	.	1
31775	0	7.62	1	0.76226	.	.	1
31776	0	7.62	1	0.76226	.	.	1
31777	0	7.62	1	0.76226	.	.	1
31778	0	7.62	1	0.76226	.	.	1
31779	0	7.62	1	0.76226	.	.	1
31780	0	7.62	1	0.76226	.	.	1

Obs	Sub_group	fuptimey	_CENSOR_	SURVIVAL	SDF_LCL	SDF_UCL	STRATUM
31781	0	7.62	1	0.76226	.	.	1
31782	0	7.62	1	0.76226	.	.	1
31783	0	7.62	1	0.76226	.	.	1
31784	0	7.62	1	0.76226	.	.	1
31785	0	7.62	1	0.76226	.	.	1
31786	0	7.62	1	0.76226	.	.	1
31787	0	7.62	1	0.76226	.	.	1
31788	0	7.62	1	0.76226	.	.	1
31789	0	7.62	1	0.76226	.	.	1
31790	0	7.62	1	0.76226	.	.	1
31791	0	7.62	1	0.76226	.	.	1
31792	0	7.63	0	0.76206	0.75679	0.76723	1
31793	0	7.63	1	0.76206	.	.	1
31794	0	7.63	1	0.76206	.	.	1
31795	0	7.63	1	0.76206	.	.	1
31796	0	7.63	1	0.76206	.	.	1
31797	0	7.63	1	0.76206	.	.	1
31798	0	7.63	1	0.76206	.	.	1
31799	0	7.63	1	0.76206	.	.	1
31800	0	7.63	1	0.76206	.	.	1
31801	0	7.63	1	0.76206	.	.	1
31802	0	7.63	1	0.76206	.	.	1
31803	0	7.63	1	0.76206	.	.	1
31804	0	7.63	1	0.76206	.	.	1
31805	0	7.63	1	0.76206	.	.	1
31806	0	7.63	1	0.76206	.	.	1
31807	0	7.63	1	0.76206	.	.	1
31808	0	7.63	1	0.76206	.	.	1
31809	0	7.63	1	0.76206	.	.	1
31810	0	7.63	1	0.76206	.	.	1
31811	0	7.63	1	0.76206	.	.	1
31812	0	7.63	1	0.76206	.	.	1
31813	0	7.63	1	0.76206	.	.	1
31814	0	7.63	1	0.76206	.	.	1
31815	0	7.63	1	0.76206	.	.	1

Obs	Sub_group	fuptimey	_CENSOR_	SURVIVAL	SDF_LCL	SDF_UCL	STRATUM
31816	0	7.63	1	0.76206	.	.	1
31817	0	7.63	1	0.76206	.	.	1
31818	0	7.63	1	0.76206	.	.	1
31819	0	7.63	1	0.76206	.	.	1
31820	0	7.63	1	0.76206	.	.	1
31821	0	7.63	1	0.76206	.	.	1
31822	0	7.63	1	0.76206	.	.	1
31823	0	7.63	1	0.76206	.	.	1
31824	0	7.63	1	0.76206	.	.	1
31825	0	7.63	1	0.76206	.	.	1
31826	0	7.64	0	0.76176	0.75649	0.76694	1
31827	0	7.64	1	0.76176	.	.	1
31828	0	7.64	1	0.76176	.	.	1
31829	0	7.64	1	0.76176	.	.	1
31830	0	7.64	1	0.76176	.	.	1
31831	0	7.64	1	0.76176	.	.	1
31832	0	7.64	1	0.76176	.	.	1
31833	0	7.64	1	0.76176	.	.	1
31834	0	7.64	1	0.76176	.	.	1
31835	0	7.64	1	0.76176	.	.	1
31836	0	7.64	1	0.76176	.	.	1
31837	0	7.64	1	0.76176	.	.	1
31838	0	7.64	1	0.76176	.	.	1
31839	0	7.64	1	0.76176	.	.	1
31840	0	7.64	1	0.76176	.	.	1
31841	0	7.64	1	0.76176	.	.	1
31842	0	7.64	1	0.76176	.	.	1
31843	0	7.64	1	0.76176	.	.	1
31844	0	7.64	1	0.76176	.	.	1
31845	0	7.64	1	0.76176	.	.	1
31846	0	7.64	1	0.76176	.	.	1
31847	0	7.64	1	0.76176	.	.	1
31848	0	7.64	1	0.76176	.	.	1
31849	0	7.64	1	0.76176	.	.	1
31850	0	7.64	1	0.76176	.	.	1

Obs	Sub_group	fuptimey	_CENSOR_	SURVIVAL	SDF_LCL	SDF_UCL	STRATUM
**31851**	0	7.64	1	0.76176	.	.	1
**31852**	0	7.64	1	0.76176	.	.	1
**31853**	0	7.64	1	0.76176	.	.	1
**31854**	0	7.64	1	0.76176	.	.	1
**31855**	0	7.64	1	0.76176	.	.	1
**31856**	0	7.64	1	0.76176	.	.	1
**31857**	0	7.64	1	0.76176	.	.	1
**31858**	0	7.64	1	0.76176	.	.	1
**31859**	0	7.64	1	0.76176	.	.	1
**31860**	0	7.64	1	0.76176	.	.	1
**31861**	0	7.65	0	0.76166	0.75639	0.76684	1
**31862**	0	7.65	1	0.76166	.	.	1
**31863**	0	7.65	1	0.76166	.	.	1
**31864**	0	7.65	1	0.76166	.	.	1
**31865**	0	7.65	1	0.76166	.	.	1
**31866**	0	7.65	1	0.76166	.	.	1
**31867**	0	7.65	1	0.76166	.	.	1
**31868**	0	7.65	1	0.76166	.	.	1
**31869**	0	7.65	1	0.76166	.	.	1
**31870**	0	7.65	1	0.76166	.	.	1
**31871**	0	7.65	1	0.76166	.	.	1
**31872**	0	7.65	1	0.76166	.	.	1
**31873**	0	7.65	1	0.76166	.	.	1
**31874**	0	7.65	1	0.76166	.	.	1
**31875**	0	7.65	1	0.76166	.	.	1
**31876**	0	7.65	1	0.76166	.	.	1
**31877**	0	7.65	1	0.76166	.	.	1
**31878**	0	7.65	1	0.76166	.	.	1
**31879**	0	7.65	1	0.76166	.	.	1
**31880**	0	7.65	1	0.76166	.	.	1
**31881**	0	7.65	1	0.76166	.	.	1
**31882**	0	7.65	1	0.76166	.	.	1
**31883**	0	7.65	1	0.76166	.	.	1
**31884**	0	7.65	1	0.76166	.	.	1
**31885**	0	7.65	1	0.76166	.	.	1

Obs	Sub_group	fuptimey	_CENSOR_	SURVIVAL	SDF_LCL	SDF_UCL	STRATUM
31886	0	7.65	1	0.76166	.	.	1
31887	0	7.65	1	0.76166	.	.	1
31888	0	7.65	1	0.76166	.	.	1
31889	0	7.65	1	0.76166	.	.	1
31890	0	7.65	1	0.76166	.	.	1
31891	0	7.65	1	0.76166	.	.	1
31892	0	7.65	1	0.76166	.	.	1
31893	0	7.65	1	0.76166	.	.	1
31894	0	7.65	1	0.76166	.	.	1
31895	0	7.65	1	0.76166	.	.	1
31896	0	7.65	1	0.76166	.	.	1
31897	0	7.65	1	0.76166	.	.	1
31898	0	7.65	1	0.76166	.	.	1
31899	0	7.65	1	0.76166	.	.	1
31900	0	7.65	1	0.76166	.	.	1
31901	0	7.66	0	0.76137	0.75608	0.76655	1
31902	0	7.66	1	0.76137	.	.	1
31903	0	7.66	1	0.76137	.	.	1
31904	0	7.66	1	0.76137	.	.	1
31905	0	7.66	1	0.76137	.	.	1
31906	0	7.66	1	0.76137	.	.	1
31907	0	7.66	1	0.76137	.	.	1
31908	0	7.66	1	0.76137	.	.	1
31909	0	7.66	1	0.76137	.	.	1
31910	0	7.66	1	0.76137	.	.	1
31911	0	7.66	1	0.76137	.	.	1
31912	0	7.66	1	0.76137	.	.	1
31913	0	7.66	1	0.76137	.	.	1
31914	0	7.66	1	0.76137	.	.	1
31915	0	7.66	1	0.76137	.	.	1
31916	0	7.66	1	0.76137	.	.	1
31917	0	7.66	1	0.76137	.	.	1
31918	0	7.66	1	0.76137	.	.	1
31919	0	7.66	1	0.76137	.	.	1
31920	0	7.66	1	0.76137	.	.	1

Obs	Sub_group	fuptimey	_CENSOR_	SURVIVAL	SDF_LCL	SDF_UCL	STRATUM
31921	0	7.66	1	0.76137	.	.	1
31922	0	7.66	1	0.76137	.	.	1
31923	0	7.66	1	0.76137	.	.	1
31924	0	7.66	1	0.76137	.	.	1
31925	0	7.66	1	0.76137	.	.	1
31926	0	7.66	1	0.76137	.	.	1
31927	0	7.66	1	0.76137	.	.	1
31928	0	7.66	1	0.76137	.	.	1
31929	0	7.66	1	0.76137	.	.	1
31930	0	7.66	1	0.76137	.	.	1
31931	0	7.66	1	0.76137	.	.	1
31932	0	7.66	1	0.76137	.	.	1
31933	0	7.66	1	0.76137	.	.	1
31934	0	7.66	1	0.76137	.	.	1
31935	0	7.66	1	0.76137	.	.	1
31936	0	7.67	1	0.76137	.	.	1
31937	0	7.67	1	0.76137	.	.	1
31938	0	7.67	1	0.76137	.	.	1
31939	0	7.67	1	0.76137	.	.	1
31940	0	7.67	1	0.76137	.	.	1
31941	0	7.67	1	0.76137	.	.	1
31942	0	7.67	1	0.76137	.	.	1
31943	0	7.67	1	0.76137	.	.	1
31944	0	7.67	1	0.76137	.	.	1
31945	0	7.67	1	0.76137	.	.	1
31946	0	7.67	1	0.76137	.	.	1
31947	0	7.67	1	0.76137	.	.	1
31948	0	7.67	1	0.76137	.	.	1
31949	0	7.67	1	0.76137	.	.	1
31950	0	7.67	1	0.76137	.	.	1
31951	0	7.67	1	0.76137	.	.	1
31952	0	7.67	1	0.76137	.	.	1
31953	0	7.67	1	0.76137	.	.	1
31954	0	7.67	1	0.76137	.	.	1
31955	0	7.67	1	0.76137	.	.	1

Obs	Sub_group	fuptimey	_CENSOR_	SURVIVAL	SDF_LCL	SDF_UCL	STRATUM
31956	0	7.67	1	0.76137	.	.	1
31957	0	7.67	1	0.76137	.	.	1
31958	0	7.67	1	0.76137	.	.	1
31959	0	7.67	1	0.76137	.	.	1
31960	0	7.67	1	0.76137	.	.	1
31961	0	7.67	1	0.76137	.	.	1
31962	0	7.67	1	0.76137	.	.	1
31963	0	7.67	1	0.76137	.	.	1
31964	0	7.67	1	0.76137	.	.	1
31965	0	7.67	1	0.76137	.	.	1
31966	0	7.67	1	0.76137	.	.	1
31967	0	7.67	1	0.76137	.	.	1
31968	0	7.67	1	0.76137	.	.	1
31969	0	7.67	1	0.76137	.	.	1
31970	0	7.67	1	0.76137	.	.	1
31971	0	7.67	1	0.76137	.	.	1
31972	0	7.67	1	0.76137	.	.	1
31973	0	7.67	1	0.76137	.	.	1
31974	0	7.67	1	0.76137	.	.	1
31975	0	7.67	1	0.76137	.	.	1
31976	0	7.67	1	0.76137	.	.	1
31977	0	7.67	1	0.76137	.	.	1
31978	0	7.67	1	0.76137	.	.	1
31979	0	7.67	1	0.76137	.	.	1
31980	0	7.67	1	0.76137	.	.	1
31981	0	7.67	1	0.76137	.	.	1
31982	0	7.68	0	0.76106	0.75577	0.76626	1
31983	0	7.68	1	0.76106	.	.	1
31984	0	7.68	1	0.76106	.	.	1
31985	0	7.68	1	0.76106	.	.	1
31986	0	7.68	1	0.76106	.	.	1
31987	0	7.68	1	0.76106	.	.	1
31988	0	7.68	1	0.76106	.	.	1
31989	0	7.68	1	0.76106	.	.	1
31990	0	7.68	1	0.76106	.	.	1

Obs	Sub_group	fuptimey	_CENSOR_	SURVIVAL	SDF_LCL	SDF_UCL	STRATUM
31991	0	7.68	1	0.76106	.	.	1
31992	0	7.68	1	0.76106	.	.	1
31993	0	7.68	1	0.76106	.	.	1
31994	0	7.68	1	0.76106	.	.	1
31995	0	7.68	1	0.76106	.	.	1
31996	0	7.68	1	0.76106	.	.	1
31997	0	7.68	1	0.76106	.	.	1
31998	0	7.68	1	0.76106	.	.	1
31999	0	7.68	1	0.76106	.	.	1
32000	0	7.68	1	0.76106	.	.	1
32001	0	7.68	1	0.76106	.	.	1
32002	0	7.68	1	0.76106	.	.	1
32003	0	7.68	1	0.76106	.	.	1
32004	0	7.68	1	0.76106	.	.	1
32005	0	7.68	1	0.76106	.	.	1
32006	0	7.68	1	0.76106	.	.	1
32007	0	7.68	1	0.76106	.	.	1
32008	0	7.68	1	0.76106	.	.	1
32009	0	7.68	1	0.76106	.	.	1
32010	0	7.68	1	0.76106	.	.	1
32011	0	7.68	1	0.76106	.	.	1
32012	0	7.68	1	0.76106	.	.	1
32013	0	7.68	1	0.76106	.	.	1
32014	0	7.68	1	0.76106	.	.	1
32015	0	7.69	1	0.76106	.	.	1
32016	0	7.69	1	0.76106	.	.	1
32017	0	7.69	1	0.76106	.	.	1
32018	0	7.69	1	0.76106	.	.	1
32019	0	7.69	1	0.76106	.	.	1
32020	0	7.69	1	0.76106	.	.	1
32021	0	7.69	1	0.76106	.	.	1
32022	0	7.69	1	0.76106	.	.	1
32023	0	7.69	1	0.76106	.	.	1
32024	0	7.69	1	0.76106	.	.	1
32025	0	7.69	1	0.76106	.	.	1

Obs	Sub_group	fuptimey	_CENSOR_	SURVIVAL	SDF_LCL	SDF_UCL	STRATUM
32026	0	7.69	1	0.76106	.	.	1
32027	0	7.69	1	0.76106	.	.	1
32028	0	7.69	1	0.76106	.	.	1
32029	0	7.69	1	0.76106	.	.	1
32030	0	7.69	1	0.76106	.	.	1
32031	0	7.69	1	0.76106	.	.	1
32032	0	7.69	1	0.76106	.	.	1
32033	0	7.69	1	0.76106	.	.	1
32034	0	7.69	1	0.76106	.	.	1
32035	0	7.69	1	0.76106	.	.	1
32036	0	7.69	1	0.76106	.	.	1
32037	0	7.69	1	0.76106	.	.	1
32038	0	7.69	1	0.76106	.	.	1
32039	0	7.69	1	0.76106	.	.	1
32040	0	7.69	1	0.76106	.	.	1
32041	0	7.69	1	0.76106	.	.	1
32042	0	7.69	1	0.76106	.	.	1
32043	0	7.69	1	0.76106	.	.	1
32044	0	7.69	1	0.76106	.	.	1
32045	0	7.69	1	0.76106	.	.	1
32046	0	7.69	1	0.76106	.	.	1
32047	0	7.69	1	0.76106	.	.	1
32048	0	7.69	1	0.76106	.	.	1
32049	0	7.69	1	0.76106	.	.	1
32050	0	7.69	1	0.76106	.	.	1
32051	0	7.69	1	0.76106	.	.	1
32052	0	7.69	1	0.76106	.	.	1
32053	0	7.69	1	0.76106	.	.	1
32054	0	7.69	1	0.76106	.	.	1
32055	0	7.69	1	0.76106	.	.	1
32056	0	7.69	1	0.76106	.	.	1
32057	0	7.69	1	0.76106	.	.	1
32058	0	7.69	1	0.76106	.	.	1
32059	0	7.69	1	0.76106	.	.	1
32060	0	7.69	1	0.76106	.	.	1

Obs	Sub_group	fuptimey	_CENSOR_	SURVIVAL	SDF_LCL	SDF_UCL	STRATUM
32061	0	7.69	1	0.76106	.	.	1
32062	0	7.69	1	0.76106	.	.	1
32063	0	7.69	1	0.76106	.	.	1
32064	0	7.69	1	0.76106	.	.	1
32065	0	7.69	1	0.76106	.	.	1
32066	0	7.70	0	0.76096	0.75567	0.76616	1
32067	0	7.70	1	0.76096	.	.	1
32068	0	7.70	1	0.76096	.	.	1
32069	0	7.70	1	0.76096	.	.	1
32070	0	7.70	1	0.76096	.	.	1
32071	0	7.70	1	0.76096	.	.	1
32072	0	7.70	1	0.76096	.	.	1
32073	0	7.70	1	0.76096	.	.	1
32074	0	7.70	1	0.76096	.	.	1
32075	0	7.70	1	0.76096	.	.	1
32076	0	7.70	1	0.76096	.	.	1
32077	0	7.70	1	0.76096	.	.	1
32078	0	7.70	1	0.76096	.	.	1
32079	0	7.70	1	0.76096	.	.	1
32080	0	7.70	1	0.76096	.	.	1
32081	0	7.70	1	0.76096	.	.	1
32082	0	7.70	1	0.76096	.	.	1
32083	0	7.70	1	0.76096	.	.	1
32084	0	7.70	1	0.76096	.	.	1
32085	0	7.70	1	0.76096	.	.	1
32086	0	7.70	1	0.76096	.	.	1
32087	0	7.70	1	0.76096	.	.	1
32088	0	7.70	1	0.76096	.	.	1
32089	0	7.70	1	0.76096	.	.	1
32090	0	7.70	1	0.76096	.	.	1
32091	0	7.70	1	0.76096	.	.	1
32092	0	7.70	1	0.76096	.	.	1
32093	0	7.70	1	0.76096	.	.	1
32094	0	7.70	1	0.76096	.	.	1
32095	0	7.70	1	0.76096	.	.	1

Obs	Sub_group	fuptimey	_CENSOR_	SURVIVAL	SDF_LCL	SDF_UCL	STRATUM
32096	0	7.70	1	0.76096	.	.	1
32097	0	7.70	1	0.76096	.	.	1
32098	0	7.70	1	0.76096	.	.	1
32099	0	7.70	1	0.76096	.	.	1
32100	0	7.70	1	0.76096	.	.	1
32101	0	7.70	1	0.76096	.	.	1
32102	0	7.70	1	0.76096	.	.	1
32103	0	7.70	1	0.76096	.	.	1
32104	0	7.70	1	0.76096	.	.	1
32105	0	7.70	1	0.76096	.	.	1
32106	0	7.70	1	0.76096	.	.	1
32107	0	7.71	0	0.76086	0.75556	0.76606	1
32108	0	7.71	1	0.76086	.	.	1
32109	0	7.71	1	0.76086	.	.	1
32110	0	7.71	1	0.76086	.	.	1
32111	0	7.71	1	0.76086	.	.	1
32112	0	7.71	1	0.76086	.	.	1
32113	0	7.71	1	0.76086	.	.	1
32114	0	7.71	1	0.76086	.	.	1
32115	0	7.71	1	0.76086	.	.	1
32116	0	7.71	1	0.76086	.	.	1
32117	0	7.71	1	0.76086	.	.	1
32118	0	7.71	1	0.76086	.	.	1
32119	0	7.71	1	0.76086	.	.	1
32120	0	7.71	1	0.76086	.	.	1
32121	0	7.71	1	0.76086	.	.	1
32122	0	7.71	1	0.76086	.	.	1
32123	0	7.71	1	0.76086	.	.	1
32124	0	7.71	1	0.76086	.	.	1
32125	0	7.71	1	0.76086	.	.	1
32126	0	7.71	1	0.76086	.	.	1
32127	0	7.71	1	0.76086	.	.	1
32128	0	7.71	1	0.76086	.	.	1
32129	0	7.71	1	0.76086	.	.	1
32130	0	7.71	1	0.76086	.	.	1

Obs	Sub_group	fuptimey	_CENSOR_	SURVIVAL	SDF_LCL	SDF_UCL	STRATUM
32131	0	7.71	1	0.76086	.	.	1
32132	0	7.71	1	0.76086	.	.	1
32133	0	7.71	1	0.76086	.	.	1
32134	0	7.71	1	0.76086	.	.	1
32135	0	7.71	1	0.76086	.	.	1
32136	0	7.71	1	0.76086	.	.	1
32137	0	7.71	1	0.76086	.	.	1
32138	0	7.71	1	0.76086	.	.	1
32139	0	7.71	1	0.76086	.	.	1
32140	0	7.71	1	0.76086	.	.	1
32141	0	7.71	1	0.76086	.	.	1
32142	0	7.71	1	0.76086	.	.	1
32143	0	7.71	1	0.76086	.	.	1
32144	0	7.71	1	0.76086	.	.	1
32145	0	7.71	1	0.76086	.	.	1
32146	0	7.71	1	0.76086	.	.	1
32147	0	7.71	1	0.76086	.	.	1
32148	0	7.72	0	0.76065	0.75535	0.76586	1
32149	0	7.72	1	0.76065	.	.	1
32150	0	7.72	1	0.76065	.	.	1
32151	0	7.72	1	0.76065	.	.	1
32152	0	7.72	1	0.76065	.	.	1
32153	0	7.72	1	0.76065	.	.	1
32154	0	7.72	1	0.76065	.	.	1
32155	0	7.72	1	0.76065	.	.	1
32156	0	7.72	1	0.76065	.	.	1
32157	0	7.72	1	0.76065	.	.	1
32158	0	7.72	1	0.76065	.	.	1
32159	0	7.72	1	0.76065	.	.	1
32160	0	7.72	1	0.76065	.	.	1
32161	0	7.72	1	0.76065	.	.	1
32162	0	7.72	1	0.76065	.	.	1
32163	0	7.72	1	0.76065	.	.	1
32164	0	7.72	1	0.76065	.	.	1
32165	0	7.72	1	0.76065	.	.	1

Obs	Sub_group	fuptimey	_CENSOR_	SURVIVAL	SDF_LCL	SDF_UCL	STRATUM
32166	0	7.72	1	0.76065	.	.	1
32167	0	7.72	1	0.76065	.	.	1
32168	0	7.72	1	0.76065	.	.	1
32169	0	7.72	1	0.76065	.	.	1
32170	0	7.72	1	0.76065	.	.	1
32171	0	7.72	1	0.76065	.	.	1
32172	0	7.72	1	0.76065	.	.	1
32173	0	7.72	1	0.76065	.	.	1
32174	0	7.72	1	0.76065	.	.	1
32175	0	7.72	1	0.76065	.	.	1
32176	0	7.73	0	0.76045	0.75514	0.76566	1
32177	0	7.73	1	0.76045	.	.	1
32178	0	7.73	1	0.76045	.	.	1
32179	0	7.73	1	0.76045	.	.	1
32180	0	7.73	1	0.76045	.	.	1
32181	0	7.73	1	0.76045	.	.	1
32182	0	7.73	1	0.76045	.	.	1
32183	0	7.73	1	0.76045	.	.	1
32184	0	7.73	1	0.76045	.	.	1
32185	0	7.73	1	0.76045	.	.	1
32186	0	7.73	1	0.76045	.	.	1
32187	0	7.73	1	0.76045	.	.	1
32188	0	7.73	1	0.76045	.	.	1
32189	0	7.73	1	0.76045	.	.	1
32190	0	7.73	1	0.76045	.	.	1
32191	0	7.73	1	0.76045	.	.	1
32192	0	7.73	1	0.76045	.	.	1
32193	0	7.73	1	0.76045	.	.	1
32194	0	7.73	1	0.76045	.	.	1
32195	0	7.73	1	0.76045	.	.	1
32196	0	7.73	1	0.76045	.	.	1
32197	0	7.73	1	0.76045	.	.	1
32198	0	7.73	1	0.76045	.	.	1
32199	0	7.73	1	0.76045	.	.	1
32200	0	7.73	1	0.76045	.	.	1

Obs	Sub_group	fuptimey	_CENSOR_	SURVIVAL	SDF_LCL	SDF_UCL	STRATUM
32201	0	7.73	1	0.76045	.	.	1
32202	0	7.73	1	0.76045	.	.	1
32203	0	7.73	1	0.76045	.	.	1
32204	0	7.73	1	0.76045	.	.	1
32205	0	7.73	1	0.76045	.	.	1
32206	0	7.73	1	0.76045	.	.	1
32207	0	7.73	1	0.76045	.	.	1
32208	0	7.73	1	0.76045	.	.	1
32209	0	7.73	1	0.76045	.	.	1
32210	0	7.73	1	0.76045	.	.	1
32211	0	7.73	1	0.76045	.	.	1
32212	0	7.73	1	0.76045	.	.	1
32213	0	7.73	1	0.76045	.	.	1
32214	0	7.73	1	0.76045	.	.	1
32215	0	7.74	0	0.76034	0.75503	0.76556	1
32216	0	7.74	1	0.76034	.	.	1
32217	0	7.74	1	0.76034	.	.	1
32218	0	7.74	1	0.76034	.	.	1
32219	0	7.74	1	0.76034	.	.	1
32220	0	7.74	1	0.76034	.	.	1
32221	0	7.74	1	0.76034	.	.	1
32222	0	7.74	1	0.76034	.	.	1
32223	0	7.74	1	0.76034	.	.	1
32224	0	7.74	1	0.76034	.	.	1
32225	0	7.74	1	0.76034	.	.	1
32226	0	7.74	1	0.76034	.	.	1
32227	0	7.74	1	0.76034	.	.	1
32228	0	7.74	1	0.76034	.	.	1
32229	0	7.74	1	0.76034	.	.	1
32230	0	7.74	1	0.76034	.	.	1
32231	0	7.74	1	0.76034	.	.	1
32232	0	7.74	1	0.76034	.	.	1
32233	0	7.74	1	0.76034	.	.	1
32234	0	7.74	1	0.76034	.	.	1
32235	0	7.74	1	0.76034	.	.	1

Obs	Sub_group	fuptimey	_CENSOR_	SURVIVAL	SDF_LCL	SDF_UCL	STRATUM
**32236**	0	7.74	1	0.76034	.	.	1
**32237**	0	7.74	1	0.76034	.	.	1
**32238**	0	7.74	1	0.76034	.	.	1
**32239**	0	7.74	1	0.76034	.	.	1
**32240**	0	7.74	1	0.76034	.	.	1
**32241**	0	7.74	1	0.76034	.	.	1
**32242**	0	7.74	1	0.76034	.	.	1
**32243**	0	7.74	1	0.76034	.	.	1
**32244**	0	7.74	1	0.76034	.	.	1
**32245**	0	7.74	1	0.76034	.	.	1
**32246**	0	7.74	1	0.76034	.	.	1
**32247**	0	7.74	1	0.76034	.	.	1
**32248**	0	7.74	1	0.76034	.	.	1
**32249**	0	7.74	1	0.76034	.	.	1
**32250**	0	7.75	0	0.75962	0.75428	0.76486	1
**32251**	0	7.75	1	0.75962	.	.	1
**32252**	0	7.75	1	0.75962	.	.	1
**32253**	0	7.75	1	0.75962	.	.	1
**32254**	0	7.75	1	0.75962	.	.	1
**32255**	0	7.75	1	0.75962	.	.	1
**32256**	0	7.75	1	0.75962	.	.	1
**32257**	0	7.75	1	0.75962	.	.	1
**32258**	0	7.75	1	0.75962	.	.	1
**32259**	0	7.75	1	0.75962	.	.	1
**32260**	0	7.75	1	0.75962	.	.	1
**32261**	0	7.75	1	0.75962	.	.	1
**32262**	0	7.75	1	0.75962	.	.	1
**32263**	0	7.75	1	0.75962	.	.	1
**32264**	0	7.75	1	0.75962	.	.	1
**32265**	0	7.75	1	0.75962	.	.	1
**32266**	0	7.75	1	0.75962	.	.	1
**32267**	0	7.75	1	0.75962	.	.	1
**32268**	0	7.75	1	0.75962	.	.	1
**32269**	0	7.75	1	0.75962	.	.	1
**32270**	0	7.75	1	0.75962	.	.	1

Obs	Sub_group	fuptimey	_CENSOR_	SURVIVAL	SDF_LCL	SDF_UCL	STRATUM
32271	0	7.75	1	0.75962	.	.	1
32272	0	7.75	1	0.75962	.	.	1
32273	0	7.75	1	0.75962	.	.	1
32274	0	7.75	1	0.75962	.	.	1
32275	0	7.75	1	0.75962	.	.	1
32276	0	7.75	1	0.75962	.	.	1
32277	0	7.75	1	0.75962	.	.	1
32278	0	7.75	1	0.75962	.	.	1
32279	0	7.75	1	0.75962	.	.	1
32280	0	7.75	1	0.75962	.	.	1
32281	0	7.75	1	0.75962	.	.	1
32282	0	7.75	1	0.75962	.	.	1
32283	0	7.75	1	0.75962	.	.	1
32284	0	7.75	1	0.75962	.	.	1
32285	0	7.75	1	0.75962	.	.	1
32286	0	7.75	1	0.75962	.	.	1
32287	0	7.76	0	0.75920	0.75384	0.76445	1
32288	0	7.76	1	0.75920	.	.	1
32289	0	7.76	1	0.75920	.	.	1
32290	0	7.76	1	0.75920	.	.	1
32291	0	7.76	1	0.75920	.	.	1
32292	0	7.76	1	0.75920	.	.	1
32293	0	7.76	1	0.75920	.	.	1
32294	0	7.76	1	0.75920	.	.	1
32295	0	7.76	1	0.75920	.	.	1
32296	0	7.76	1	0.75920	.	.	1
32297	0	7.76	1	0.75920	.	.	1
32298	0	7.76	1	0.75920	.	.	1
32299	0	7.76	1	0.75920	.	.	1
32300	0	7.76	1	0.75920	.	.	1
32301	0	7.76	1	0.75920	.	.	1
32302	0	7.76	1	0.75920	.	.	1
32303	0	7.76	1	0.75920	.	.	1
32304	0	7.76	1	0.75920	.	.	1
32305	0	7.76	1	0.75920	.	.	1

Obs	Sub_group	fuptimey	_CENSOR_	SURVIVAL	SDF_LCL	SDF_UCL	STRATUM
32306	0	7.76	1	0.75920	.	.	1
32307	0	7.76	1	0.75920	.	.	1
32308	0	7.76	1	0.75920	.	.	1
32309	0	7.76	1	0.75920	.	.	1
32310	0	7.76	1	0.75920	.	.	1
32311	0	7.76	1	0.75920	.	.	1
32312	0	7.76	1	0.75920	.	.	1
32313	0	7.76	1	0.75920	.	.	1
32314	0	7.76	1	0.75920	.	.	1
32315	0	7.76	1	0.75920	.	.	1
32316	0	7.76	1	0.75920	.	.	1
32317	0	7.76	1	0.75920	.	.	1
32318	0	7.76	1	0.75920	.	.	1
32319	0	7.76	1	0.75920	.	.	1
32320	0	7.76	1	0.75920	.	.	1
32321	0	7.76	1	0.75920	.	.	1
32322	0	7.76	1	0.75920	.	.	1
32323	0	7.76	1	0.75920	.	.	1
32324	0	7.76	1	0.75920	.	.	1
32325	0	7.77	0	0.75878	0.75341	0.76404	1
32326	0	7.77	1	0.75878	.	.	1
32327	0	7.77	1	0.75878	.	.	1
32328	0	7.77	1	0.75878	.	.	1
32329	0	7.77	1	0.75878	.	.	1
32330	0	7.77	1	0.75878	.	.	1
32331	0	7.77	1	0.75878	.	.	1
32332	0	7.77	1	0.75878	.	.	1
32333	0	7.77	1	0.75878	.	.	1
32334	0	7.77	1	0.75878	.	.	1
32335	0	7.77	1	0.75878	.	.	1
32336	0	7.77	1	0.75878	.	.	1
32337	0	7.77	1	0.75878	.	.	1
32338	0	7.77	1	0.75878	.	.	1
32339	0	7.77	1	0.75878	.	.	1
32340	0	7.77	1	0.75878	.	.	1

Obs	Sub_group	fuptimey	_CENSOR_	SURVIVAL	SDF_LCL	SDF_UCL	STRATUM
32341	0	7.77	1	0.75878	.	.	1
32342	0	7.77	1	0.75878	.	.	1
32343	0	7.77	1	0.75878	.	.	1
32344	0	7.77	1	0.75878	.	.	1
32345	0	7.77	1	0.75878	.	.	1
32346	0	7.77	1	0.75878	.	.	1
32347	0	7.77	1	0.75878	.	.	1
32348	0	7.77	1	0.75878	.	.	1
32349	0	7.77	1	0.75878	.	.	1
32350	0	7.77	1	0.75878	.	.	1
32351	0	7.77	1	0.75878	.	.	1
32352	0	7.77	1	0.75878	.	.	1
32353	0	7.77	1	0.75878	.	.	1
32354	0	7.77	1	0.75878	.	.	1
32355	0	7.77	1	0.75878	.	.	1
32356	0	7.77	1	0.75878	.	.	1
32357	0	7.77	1	0.75878	.	.	1
32358	0	7.77	1	0.75878	.	.	1
32359	0	7.77	1	0.75878	.	.	1
32360	0	7.77	1	0.75878	.	.	1
32361	0	7.77	1	0.75878	.	.	1
32362	0	7.77	1	0.75878	.	.	1
32363	0	7.77	1	0.75878	.	.	1
32364	0	7.77	1	0.75878	.	.	1
32365	0	7.77	1	0.75878	.	.	1
32366	0	7.77	1	0.75878	.	.	1
32367	0	7.77	1	0.75878	.	.	1
32368	0	7.77	1	0.75878	.	.	1
32369	0	7.77	1	0.75878	.	.	1
32370	0	7.77	1	0.75878	.	.	1
32371	0	7.77	1	0.75878	.	.	1
32372	0	7.78	0	0.75867	0.75330	0.76394	1
32373	0	7.78	1	0.75867	.	.	1
32374	0	7.78	1	0.75867	.	.	1
32375	0	7.78	1	0.75867	.	.	1

Obs	Sub_group	fuptimey	_CENSOR_	SURVIVAL	SDF_LCL	SDF_UCL	STRATUM
32376	0	7.78	1	0.75867	.	.	1
32377	0	7.78	1	0.75867	.	.	1
32378	0	7.78	1	0.75867	.	.	1
32379	0	7.78	1	0.75867	.	.	1
32380	0	7.78	1	0.75867	.	.	1
32381	0	7.78	1	0.75867	.	.	1
32382	0	7.78	1	0.75867	.	.	1
32383	0	7.78	1	0.75867	.	.	1
32384	0	7.78	1	0.75867	.	.	1
32385	0	7.78	1	0.75867	.	.	1
32386	0	7.78	1	0.75867	.	.	1
32387	0	7.78	1	0.75867	.	.	1
32388	0	7.78	1	0.75867	.	.	1
32389	0	7.78	1	0.75867	.	.	1
32390	0	7.78	1	0.75867	.	.	1
32391	0	7.78	1	0.75867	.	.	1
32392	0	7.78	1	0.75867	.	.	1
32393	0	7.78	1	0.75867	.	.	1
32394	0	7.78	1	0.75867	.	.	1
32395	0	7.78	1	0.75867	.	.	1
32396	0	7.78	1	0.75867	.	.	1
32397	0	7.78	1	0.75867	.	.	1
32398	0	7.78	1	0.75867	.	.	1
32399	0	7.78	1	0.75867	.	.	1
32400	0	7.78	1	0.75867	.	.	1
32401	0	7.78	1	0.75867	.	.	1
32402	0	7.78	1	0.75867	.	.	1
32403	0	7.78	1	0.75867	.	.	1
32404	0	7.78	1	0.75867	.	.	1
32405	0	7.79	0	0.75846	0.75308	0.76373	1
32406	0	7.79	1	0.75846	.	.	1
32407	0	7.79	1	0.75846	.	.	1
32408	0	7.79	1	0.75846	.	.	1
32409	0	7.79	1	0.75846	.	.	1
32410	0	7.79	1	0.75846	.	.	1

Obs	Sub_group	fuptimey	_CENSOR_	SURVIVAL	SDF_LCL	SDF_UCL	STRATUM
32411	0	7.79	1	0.75846	.	.	1
32412	0	7.79	1	0.75846	.	.	1
32413	0	7.79	1	0.75846	.	.	1
32414	0	7.79	1	0.75846	.	.	1
32415	0	7.79	1	0.75846	.	.	1
32416	0	7.79	1	0.75846	.	.	1
32417	0	7.79	1	0.75846	.	.	1
32418	0	7.79	1	0.75846	.	.	1
32419	0	7.79	1	0.75846	.	.	1
32420	0	7.79	1	0.75846	.	.	1
32421	0	7.79	1	0.75846	.	.	1
32422	0	7.79	1	0.75846	.	.	1
32423	0	7.79	1	0.75846	.	.	1
32424	0	7.79	1	0.75846	.	.	1
32425	0	7.79	1	0.75846	.	.	1
32426	0	7.79	1	0.75846	.	.	1
32427	0	7.79	1	0.75846	.	.	1
32428	0	7.79	1	0.75846	.	.	1
32429	0	7.79	1	0.75846	.	.	1
32430	0	7.79	1	0.75846	.	.	1
32431	0	7.79	1	0.75846	.	.	1
32432	0	7.79	1	0.75846	.	.	1
32433	0	7.79	1	0.75846	.	.	1
32434	0	7.79	1	0.75846	.	.	1
32435	0	7.79	1	0.75846	.	.	1
32436	0	7.79	1	0.75846	.	.	1
32437	0	7.79	1	0.75846	.	.	1
32438	0	7.79	1	0.75846	.	.	1
32439	0	7.79	1	0.75846	.	.	1
32440	0	7.79	1	0.75846	.	.	1
32441	0	7.79	1	0.75846	.	.	1
32442	0	7.79	1	0.75846	.	.	1
32443	0	7.79	1	0.75846	.	.	1
32444	0	7.79	1	0.75846	.	.	1
32445	0	7.79	1	0.75846	.	.	1

Obs	Sub_group	fuptimey	_CENSOR_	SURVIVAL	SDF_LCL	SDF_UCL	STRATUM
32446	0	7.79	1	0.75846	.	.	1
32447	0	7.79	1	0.75846	.	.	1
32448	0	7.79	1	0.75846	.	.	1
32449	0	7.79	1	0.75846	.	.	1
32450	0	7.79	1	0.75846	.	.	1
32451	0	7.79	1	0.75846	.	.	1
32452	0	7.79	1	0.75846	.	.	1
32453	0	7.79	1	0.75846	.	.	1
32454	0	7.79	1	0.75846	.	.	1
32455	0	7.79	1	0.75846	.	.	1
32456	0	7.79	1	0.75846	.	.	1
32457	0	7.79	1	0.75846	.	.	1
32458	0	7.80	0	0.75835	0.75297	0.76363	1
32459	0	7.80	1	0.75835	.	.	1
32460	0	7.80	1	0.75835	.	.	1
32461	0	7.80	1	0.75835	.	.	1
32462	0	7.80	1	0.75835	.	.	1
32463	0	7.80	1	0.75835	.	.	1
32464	0	7.80	1	0.75835	.	.	1
32465	0	7.80	1	0.75835	.	.	1
32466	0	7.80	1	0.75835	.	.	1
32467	0	7.80	1	0.75835	.	.	1
32468	0	7.80	1	0.75835	.	.	1
32469	0	7.80	1	0.75835	.	.	1
32470	0	7.80	1	0.75835	.	.	1
32471	0	7.80	1	0.75835	.	.	1
32472	0	7.80	1	0.75835	.	.	1
32473	0	7.80	1	0.75835	.	.	1
32474	0	7.80	1	0.75835	.	.	1
32475	0	7.80	1	0.75835	.	.	1
32476	0	7.80	1	0.75835	.	.	1
32477	0	7.80	1	0.75835	.	.	1
32478	0	7.80	1	0.75835	.	.	1
32479	0	7.80	1	0.75835	.	.	1
32480	0	7.80	1	0.75835	.	.	1

Obs	Sub_group	fuptimey	_CENSOR_	SURVIVAL	SDF_LCL	SDF_UCL	STRATUM
32481	0	7.80	1	0.75835	.	.	1
32482	0	7.80	1	0.75835	.	.	1
32483	0	7.80	1	0.75835	.	.	1
32484	0	7.80	1	0.75835	.	.	1
32485	0	7.80	1	0.75835	.	.	1
32486	0	7.80	1	0.75835	.	.	1
32487	0	7.80	1	0.75835	.	.	1
32488	0	7.80	1	0.75835	.	.	1
32489	0	7.80	1	0.75835	.	.	1
32490	0	7.80	1	0.75835	.	.	1
32491	0	7.80	1	0.75835	.	.	1
32492	0	7.80	1	0.75835	.	.	1
32493	0	7.80	1	0.75835	.	.	1
32494	0	7.80	1	0.75835	.	.	1
32495	0	7.80	1	0.75835	.	.	1
32496	0	7.80	1	0.75835	.	.	1
32497	0	7.80	1	0.75835	.	.	1
32498	0	7.80	1	0.75835	.	.	1
32499	0	7.80	1	0.75835	.	.	1
32500	0	7.80	1	0.75835	.	.	1
32501	0	7.80	1	0.75835	.	.	1
32502	0	7.80	1	0.75835	.	.	1
32503	0	7.81	1	0.75835	.	.	1
32504	0	7.81	1	0.75835	.	.	1
32505	0	7.81	1	0.75835	.	.	1
32506	0	7.81	1	0.75835	.	.	1
32507	0	7.81	1	0.75835	.	.	1
32508	0	7.81	1	0.75835	.	.	1
32509	0	7.81	1	0.75835	.	.	1
32510	0	7.81	1	0.75835	.	.	1
32511	0	7.81	1	0.75835	.	.	1
32512	0	7.81	1	0.75835	.	.	1
32513	0	7.81	1	0.75835	.	.	1
32514	0	7.81	1	0.75835	.	.	1
32515	0	7.81	1	0.75835	.	.	1

Obs	Sub_group	fuptimey	_CENSOR_	SURVIVAL	SDF_LCL	SDF_UCL	STRATUM
32516	0	7.81	1	0.75835	.	.	1
32517	0	7.81	1	0.75835	.	.	1
32518	0	7.81	1	0.75835	.	.	1
32519	0	7.81	1	0.75835	.	.	1
32520	0	7.81	1	0.75835	.	.	1
32521	0	7.81	1	0.75835	.	.	1
32522	0	7.81	1	0.75835	.	.	1
32523	0	7.81	1	0.75835	.	.	1
32524	0	7.81	1	0.75835	.	.	1
32525	0	7.81	1	0.75835	.	.	1
32526	0	7.81	1	0.75835	.	.	1
32527	0	7.81	1	0.75835	.	.	1
32528	0	7.81	1	0.75835	.	.	1
32529	0	7.81	1	0.75835	.	.	1
32530	0	7.81	1	0.75835	.	.	1
32531	0	7.81	1	0.75835	.	.	1
32532	0	7.81	1	0.75835	.	.	1
32533	0	7.81	1	0.75835	.	.	1
32534	0	7.81	1	0.75835	.	.	1
32535	0	7.81	1	0.75835	.	.	1
32536	0	7.81	1	0.75835	.	.	1
32537	0	7.81	1	0.75835	.	.	1
32538	0	7.81	1	0.75835	.	.	1
32539	0	7.81	1	0.75835	.	.	1
32540	0	7.81	1	0.75835	.	.	1
32541	0	7.81	1	0.75835	.	.	1
32542	0	7.82	0	0.75824	0.75286	0.76353	1
32543	0	7.82	1	0.75824	.	.	1
32544	0	7.82	1	0.75824	.	.	1
32545	0	7.82	1	0.75824	.	.	1
32546	0	7.82	1	0.75824	.	.	1
32547	0	7.82	1	0.75824	.	.	1
32548	0	7.82	1	0.75824	.	.	1
32549	0	7.82	1	0.75824	.	.	1
32550	0	7.82	1	0.75824	.	.	1

Obs	Sub_group	fuptimey	_CENSOR_	SURVIVAL	SDF_LCL	SDF_UCL	STRATUM
32551	0	7.82	1	0.75824	.	.	1
32552	0	7.82	1	0.75824	.	.	1
32553	0	7.82	1	0.75824	.	.	1
32554	0	7.82	1	0.75824	.	.	1
32555	0	7.82	1	0.75824	.	.	1
32556	0	7.82	1	0.75824	.	.	1
32557	0	7.82	1	0.75824	.	.	1
32558	0	7.82	1	0.75824	.	.	1
32559	0	7.82	1	0.75824	.	.	1
32560	0	7.82	1	0.75824	.	.	1
32561	0	7.82	1	0.75824	.	.	1
32562	0	7.82	1	0.75824	.	.	1
32563	0	7.82	1	0.75824	.	.	1
32564	0	7.82	1	0.75824	.	.	1
32565	0	7.82	1	0.75824	.	.	1
32566	0	7.82	1	0.75824	.	.	1
32567	0	7.82	1	0.75824	.	.	1
32568	0	7.82	1	0.75824	.	.	1
32569	0	7.82	1	0.75824	.	.	1
32570	0	7.82	1	0.75824	.	.	1
32571	0	7.82	1	0.75824	.	.	1
32572	0	7.82	1	0.75824	.	.	1
32573	0	7.82	1	0.75824	.	.	1
32574	0	7.82	1	0.75824	.	.	1
32575	0	7.82	1	0.75824	.	.	1
32576	0	7.82	1	0.75824	.	.	1
32577	0	7.83	0	0.75813	0.75275	0.76342	1
32578	0	7.83	1	0.75813	.	.	1
32579	0	7.83	1	0.75813	.	.	1
32580	0	7.83	1	0.75813	.	.	1
32581	0	7.83	1	0.75813	.	.	1
32582	0	7.83	1	0.75813	.	.	1
32583	0	7.83	1	0.75813	.	.	1
32584	0	7.83	1	0.75813	.	.	1
32585	0	7.83	1	0.75813	.	.	1

Obs	Sub_group	fuptimey	_CENSOR_	SURVIVAL	SDF_LCL	SDF_UCL	STRATUM
32586	0	7.83	1	0.75813	.	.	1
32587	0	7.83	1	0.75813	.	.	1
32588	0	7.83	1	0.75813	.	.	1
32589	0	7.83	1	0.75813	.	.	1
32590	0	7.83	1	0.75813	.	.	1
32591	0	7.83	1	0.75813	.	.	1
32592	0	7.83	1	0.75813	.	.	1
32593	0	7.83	1	0.75813	.	.	1
32594	0	7.83	1	0.75813	.	.	1
32595	0	7.83	1	0.75813	.	.	1
32596	0	7.83	1	0.75813	.	.	1
32597	0	7.83	1	0.75813	.	.	1
32598	0	7.83	1	0.75813	.	.	1
32599	0	7.83	1	0.75813	.	.	1
32600	0	7.83	1	0.75813	.	.	1
32601	0	7.83	1	0.75813	.	.	1
32602	0	7.83	1	0.75813	.	.	1
32603	0	7.83	1	0.75813	.	.	1
32604	0	7.83	1	0.75813	.	.	1
32605	0	7.83	1	0.75813	.	.	1
32606	0	7.83	1	0.75813	.	.	1
32607	0	7.83	1	0.75813	.	.	1
32608	0	7.83	1	0.75813	.	.	1
32609	0	7.83	1	0.75813	.	.	1
32610	0	7.83	1	0.75813	.	.	1
32611	0	7.83	1	0.75813	.	.	1
32612	0	7.83	1	0.75813	.	.	1
32613	0	7.83	1	0.75813	.	.	1
32614	0	7.83	1	0.75813	.	.	1
32615	0	7.83	1	0.75813	.	.	1
32616	0	7.84	0	0.75802	0.75263	0.76331	1
32617	0	7.84	1	0.75802	.	.	1
32618	0	7.84	1	0.75802	.	.	1
32619	0	7.84	1	0.75802	.	.	1
32620	0	7.84	1	0.75802	.	.	1

Obs	Sub_group	fuptimey	_CENSOR_	SURVIVAL	SDF_LCL	SDF_UCL	STRATUM
32621	0	7.84	1	0.75802	.	.	1
32622	0	7.84	1	0.75802	.	.	1
32623	0	7.84	1	0.75802	.	.	1
32624	0	7.84	1	0.75802	.	.	1
32625	0	7.84	1	0.75802	.	.	1
32626	0	7.84	1	0.75802	.	.	1
32627	0	7.84	1	0.75802	.	.	1
32628	0	7.84	1	0.75802	.	.	1
32629	0	7.84	1	0.75802	.	.	1
32630	0	7.84	1	0.75802	.	.	1
32631	0	7.84	1	0.75802	.	.	1
32632	0	7.84	1	0.75802	.	.	1
32633	0	7.84	1	0.75802	.	.	1
32634	0	7.84	1	0.75802	.	.	1
32635	0	7.84	1	0.75802	.	.	1
32636	0	7.84	1	0.75802	.	.	1
32637	0	7.84	1	0.75802	.	.	1
32638	0	7.84	1	0.75802	.	.	1
32639	0	7.84	1	0.75802	.	.	1
32640	0	7.84	1	0.75802	.	.	1
32641	0	7.84	1	0.75802	.	.	1
32642	0	7.84	1	0.75802	.	.	1
32643	0	7.84	1	0.75802	.	.	1
32644	0	7.84	1	0.75802	.	.	1
32645	0	7.84	1	0.75802	.	.	1
32646	0	7.84	1	0.75802	.	.	1
32647	0	7.84	1	0.75802	.	.	1
32648	0	7.84	1	0.75802	.	.	1
32649	0	7.84	1	0.75802	.	.	1
32650	0	7.84	1	0.75802	.	.	1
32651	0	7.84	1	0.75802	.	.	1
32652	0	7.84	1	0.75802	.	.	1
32653	0	7.85	0	0.75791	0.75252	0.76321	1
32654	0	7.85	1	0.75791	.	.	1
32655	0	7.85	1	0.75791	.	.	1

Obs	Sub_group	fuptimey	_CENSOR_	SURVIVAL	SDF_LCL	SDF_UCL	STRATUM
32656	0	7.85	1	0.75791	.	.	1
32657	0	7.85	1	0.75791	.	.	1
32658	0	7.85	1	0.75791	.	.	1
32659	0	7.85	1	0.75791	.	.	1
32660	0	7.85	1	0.75791	.	.	1
32661	0	7.85	1	0.75791	.	.	1
32662	0	7.85	1	0.75791	.	.	1
32663	0	7.85	1	0.75791	.	.	1
32664	0	7.85	1	0.75791	.	.	1
32665	0	7.85	1	0.75791	.	.	1
32666	0	7.85	1	0.75791	.	.	1
32667	0	7.85	1	0.75791	.	.	1
32668	0	7.85	1	0.75791	.	.	1
32669	0	7.85	1	0.75791	.	.	1
32670	0	7.85	1	0.75791	.	.	1
32671	0	7.85	1	0.75791	.	.	1
32672	0	7.85	1	0.75791	.	.	1
32673	0	7.85	1	0.75791	.	.	1
32674	0	7.85	1	0.75791	.	.	1
32675	0	7.85	1	0.75791	.	.	1
32676	0	7.85	1	0.75791	.	.	1
32677	0	7.85	1	0.75791	.	.	1
32678	0	7.85	1	0.75791	.	.	1
32679	0	7.85	1	0.75791	.	.	1
32680	0	7.85	1	0.75791	.	.	1
32681	0	7.85	1	0.75791	.	.	1
32682	0	7.85	1	0.75791	.	.	1
32683	0	7.85	1	0.75791	.	.	1
32684	0	7.86	0	0.75780	0.75240	0.76310	1
32685	0	7.86	1	0.75780	.	.	1
32686	0	7.86	1	0.75780	.	.	1
32687	0	7.86	1	0.75780	.	.	1
32688	0	7.86	1	0.75780	.	.	1
32689	0	7.86	1	0.75780	.	.	1
32690	0	7.86	1	0.75780	.	.	1

Obs	Sub_group	fuptimey	_CENSOR_	SURVIVAL	SDF_LCL	SDF_UCL	STRATUM
32691	0	7.86	1	0.75780	.	.	1
32692	0	7.86	1	0.75780	.	.	1
32693	0	7.86	1	0.75780	.	.	1
32694	0	7.86	1	0.75780	.	.	1
32695	0	7.86	1	0.75780	.	.	1
32696	0	7.86	1	0.75780	.	.	1
32697	0	7.86	1	0.75780	.	.	1
32698	0	7.86	1	0.75780	.	.	1
32699	0	7.86	1	0.75780	.	.	1
32700	0	7.86	1	0.75780	.	.	1
32701	0	7.86	1	0.75780	.	.	1
32702	0	7.86	1	0.75780	.	.	1
32703	0	7.86	1	0.75780	.	.	1
32704	0	7.86	1	0.75780	.	.	1
32705	0	7.86	1	0.75780	.	.	1
32706	0	7.86	1	0.75780	.	.	1
32707	0	7.86	1	0.75780	.	.	1
32708	0	7.86	1	0.75780	.	.	1
32709	0	7.86	1	0.75780	.	.	1
32710	0	7.86	1	0.75780	.	.	1
32711	0	7.86	1	0.75780	.	.	1
32712	0	7.86	1	0.75780	.	.	1
32713	0	7.86	1	0.75780	.	.	1
32714	0	7.86	1	0.75780	.	.	1
32715	0	7.86	1	0.75780	.	.	1
32716	0	7.86	1	0.75780	.	.	1
32717	0	7.86	1	0.75780	.	.	1
32718	0	7.87	0	0.75747	0.75206	0.76278	1
32719	0	7.87	1	0.75747	.	.	1
32720	0	7.87	1	0.75747	.	.	1
32721	0	7.87	1	0.75747	.	.	1
32722	0	7.87	1	0.75747	.	.	1
32723	0	7.87	1	0.75747	.	.	1
32724	0	7.87	1	0.75747	.	.	1
32725	0	7.87	1	0.75747	.	.	1

Obs	Sub_group	fuptimey	_CENSOR_	SURVIVAL	SDF_LCL	SDF_UCL	STRATUM
32726	0	7.87	1	0.75747	.	.	1
32727	0	7.87	1	0.75747	.	.	1
32728	0	7.87	1	0.75747	.	.	1
32729	0	7.87	1	0.75747	.	.	1
32730	0	7.87	1	0.75747	.	.	1
32731	0	7.87	1	0.75747	.	.	1
32732	0	7.87	1	0.75747	.	.	1
32733	0	7.87	1	0.75747	.	.	1
32734	0	7.87	1	0.75747	.	.	1
32735	0	7.87	1	0.75747	.	.	1
32736	0	7.87	1	0.75747	.	.	1
32737	0	7.87	1	0.75747	.	.	1
32738	0	7.87	1	0.75747	.	.	1
32739	0	7.88	1	0.75747	.	.	1
32740	0	7.88	1	0.75747	.	.	1
32741	0	7.88	1	0.75747	.	.	1
32742	0	7.88	1	0.75747	.	.	1
32743	0	7.88	1	0.75747	.	.	1
32744	0	7.88	1	0.75747	.	.	1
32745	0	7.88	1	0.75747	.	.	1
32746	0	7.88	1	0.75747	.	.	1
32747	0	7.88	1	0.75747	.	.	1
32748	0	7.88	1	0.75747	.	.	1
32749	0	7.88	1	0.75747	.	.	1
32750	0	7.88	1	0.75747	.	.	1
32751	0	7.88	1	0.75747	.	.	1
32752	0	7.88	1	0.75747	.	.	1
32753	0	7.88	1	0.75747	.	.	1
32754	0	7.88	1	0.75747	.	.	1
32755	0	7.88	1	0.75747	.	.	1
32756	0	7.88	1	0.75747	.	.	1
32757	0	7.88	1	0.75747	.	.	1
32758	0	7.88	1	0.75747	.	.	1
32759	0	7.88	1	0.75747	.	.	1
32760	0	7.88	1	0.75747	.	.	1

Obs	Sub_group	fuptimey	_CENSOR_	SURVIVAL	SDF_LCL	SDF_UCL	STRATUM
32761	0	7.88	1	0.75747	.	.	1
32762	0	7.88	1	0.75747	.	.	1
32763	0	7.88	1	0.75747	.	.	1
32764	0	7.88	1	0.75747	.	.	1
32765	0	7.88	1	0.75747	.	.	1
32766	0	7.88	1	0.75747	.	.	1
32767	0	7.88	1	0.75747	.	.	1
32768	0	7.88	1	0.75747	.	.	1
32769	0	7.89	0	0.75724	0.75183	0.76256	1
32770	0	7.89	1	0.75724	.	.	1
32771	0	7.89	1	0.75724	.	.	1
32772	0	7.89	1	0.75724	.	.	1
32773	0	7.89	1	0.75724	.	.	1
32774	0	7.89	1	0.75724	.	.	1
32775	0	7.89	1	0.75724	.	.	1
32776	0	7.89	1	0.75724	.	.	1
32777	0	7.89	1	0.75724	.	.	1
32778	0	7.89	1	0.75724	.	.	1
32779	0	7.89	1	0.75724	.	.	1
32780	0	7.89	1	0.75724	.	.	1
32781	0	7.89	1	0.75724	.	.	1
32782	0	7.89	1	0.75724	.	.	1
32783	0	7.89	1	0.75724	.	.	1
32784	0	7.89	1	0.75724	.	.	1
32785	0	7.89	1	0.75724	.	.	1
32786	0	7.89	1	0.75724	.	.	1
32787	0	7.89	1	0.75724	.	.	1
32788	0	7.89	1	0.75724	.	.	1
32789	0	7.89	1	0.75724	.	.	1
32790	0	7.89	1	0.75724	.	.	1
32791	0	7.89	1	0.75724	.	.	1
32792	0	7.89	1	0.75724	.	.	1
32793	0	7.89	1	0.75724	.	.	1
32794	0	7.89	1	0.75724	.	.	1
32795	0	7.89	1	0.75724	.	.	1

Obs	Sub_group	fuptimey	_CENSOR_	SURVIVAL	SDF_LCL	SDF_UCL	STRATUM
**32796**	0	7.89	1	0.75724	.	.	1
**32797**	0	7.89	1	0.75724	.	.	1
**32798**	0	7.89	1	0.75724	.	.	1
**32799**	0	7.89	1	0.75724	.	.	1
**32800**	0	7.89	1	0.75724	.	.	1
**32801**	0	7.89	1	0.75724	.	.	1
**32802**	0	7.89	1	0.75724	.	.	1
**32803**	0	7.90	0	0.75691	0.75148	0.76223	1
**32804**	0	7.90	1	0.75691	.	.	1
**32805**	0	7.90	1	0.75691	.	.	1
**32806**	0	7.90	1	0.75691	.	.	1
**32807**	0	7.90	1	0.75691	.	.	1
**32808**	0	7.90	1	0.75691	.	.	1
**32809**	0	7.90	1	0.75691	.	.	1
**32810**	0	7.90	1	0.75691	.	.	1
**32811**	0	7.90	1	0.75691	.	.	1
**32812**	0	7.90	1	0.75691	.	.	1
**32813**	0	7.90	1	0.75691	.	.	1
**32814**	0	7.90	1	0.75691	.	.	1
**32815**	0	7.90	1	0.75691	.	.	1
**32816**	0	7.90	1	0.75691	.	.	1
**32817**	0	7.90	1	0.75691	.	.	1
**32818**	0	7.90	1	0.75691	.	.	1
**32819**	0	7.90	1	0.75691	.	.	1
**32820**	0	7.90	1	0.75691	.	.	1
**32821**	0	7.90	1	0.75691	.	.	1
**32822**	0	7.90	1	0.75691	.	.	1
**32823**	0	7.90	1	0.75691	.	.	1
**32824**	0	7.90	1	0.75691	.	.	1
**32825**	0	7.90	1	0.75691	.	.	1
**32826**	0	7.90	1	0.75691	.	.	1
**32827**	0	7.90	1	0.75691	.	.	1
**32828**	0	7.90	1	0.75691	.	.	1
**32829**	0	7.90	1	0.75691	.	.	1
**32830**	0	7.90	1	0.75691	.	.	1

Obs	Sub_group	fuptimey	_CENSOR_	SURVIVAL	SDF_LCL	SDF_UCL	STRATUM
32831	0	7.90	1	0.75691	.	.	1
32832	0	7.90	1	0.75691	.	.	1
32833	0	7.90	1	0.75691	.	.	1
32834	0	7.90	1	0.75691	.	.	1
32835	0	7.90	1	0.75691	.	.	1
32836	0	7.90	1	0.75691	.	.	1
32837	0	7.90	1	0.75691	.	.	1
32838	0	7.90	1	0.75691	.	.	1
32839	0	7.90	1	0.75691	.	.	1
32840	0	7.90	1	0.75691	.	.	1
32841	0	7.90	1	0.75691	.	.	1
32842	0	7.90	1	0.75691	.	.	1
32843	0	7.90	1	0.75691	.	.	1
32844	0	7.91	0	0.75679	0.75136	0.76213	1
32845	0	7.91	1	0.75679	.	.	1
32846	0	7.91	1	0.75679	.	.	1
32847	0	7.91	1	0.75679	.	.	1
32848	0	7.91	1	0.75679	.	.	1
32849	0	7.91	1	0.75679	.	.	1
32850	0	7.91	1	0.75679	.	.	1
32851	0	7.91	1	0.75679	.	.	1
32852	0	7.91	1	0.75679	.	.	1
32853	0	7.91	1	0.75679	.	.	1
32854	0	7.91	1	0.75679	.	.	1
32855	0	7.91	1	0.75679	.	.	1
32856	0	7.91	1	0.75679	.	.	1
32857	0	7.91	1	0.75679	.	.	1
32858	0	7.91	1	0.75679	.	.	1
32859	0	7.91	1	0.75679	.	.	1
32860	0	7.91	1	0.75679	.	.	1
32861	0	7.91	1	0.75679	.	.	1
32862	0	7.91	1	0.75679	.	.	1
32863	0	7.91	1	0.75679	.	.	1
32864	0	7.91	1	0.75679	.	.	1
32865	0	7.91	1	0.75679	.	.	1

Obs	Sub_group	fuptimey	_CENSOR_	SURVIVAL	SDF_LCL	SDF_UCL	STRATUM
32866	0	7.91	1	0.75679	.	.	1
32867	0	7.91	1	0.75679	.	.	1
32868	0	7.91	1	0.75679	.	.	1
32869	0	7.91	1	0.75679	.	.	1
32870	0	7.91	1	0.75679	.	.	1
32871	0	7.91	1	0.75679	.	.	1
32872	0	7.91	1	0.75679	.	.	1
32873	0	7.91	1	0.75679	.	.	1
32874	0	7.91	1	0.75679	.	.	1
32875	0	7.91	1	0.75679	.	.	1
32876	0	7.91	1	0.75679	.	.	1
32877	0	7.91	1	0.75679	.	.	1
32878	0	7.91	1	0.75679	.	.	1
32879	0	7.91	1	0.75679	.	.	1
32880	0	7.91	1	0.75679	.	.	1
32881	0	7.91	1	0.75679	.	.	1
32882	0	7.91	1	0.75679	.	.	1
32883	0	7.91	1	0.75679	.	.	1
32884	0	7.91	1	0.75679	.	.	1
32885	0	7.91	1	0.75679	.	.	1
32886	0	7.91	1	0.75679	.	.	1
32887	0	7.91	1	0.75679	.	.	1
32888	0	7.91	1	0.75679	.	.	1
32889	0	7.92	0	0.75634	0.75089	0.76168	1
32890	0	7.92	1	0.75634	.	.	1
32891	0	7.92	1	0.75634	.	.	1
32892	0	7.92	1	0.75634	.	.	1
32893	0	7.92	1	0.75634	.	.	1
32894	0	7.92	1	0.75634	.	.	1
32895	0	7.92	1	0.75634	.	.	1
32896	0	7.92	1	0.75634	.	.	1
32897	0	7.92	1	0.75634	.	.	1
32898	0	7.92	1	0.75634	.	.	1
32899	0	7.92	1	0.75634	.	.	1
32900	0	7.92	1	0.75634	.	.	1

Obs	Sub_group	fuptimey	_CENSOR_	SURVIVAL	SDF_LCL	SDF_UCL	STRATUM
32901	0	7.92	1	0.75634	.	.	1
32902	0	7.92	1	0.75634	.	.	1
32903	0	7.92	1	0.75634	.	.	1
32904	0	7.92	1	0.75634	.	.	1
32905	0	7.92	1	0.75634	.	.	1
32906	0	7.92	1	0.75634	.	.	1
32907	0	7.92	1	0.75634	.	.	1
32908	0	7.92	1	0.75634	.	.	1
32909	0	7.92	1	0.75634	.	.	1
32910	0	7.92	1	0.75634	.	.	1
32911	0	7.92	1	0.75634	.	.	1
32912	0	7.92	1	0.75634	.	.	1
32913	0	7.92	1	0.75634	.	.	1
32914	0	7.92	1	0.75634	.	.	1
32915	0	7.92	1	0.75634	.	.	1
32916	0	7.92	1	0.75634	.	.	1
32917	0	7.92	1	0.75634	.	.	1
32918	0	7.92	1	0.75634	.	.	1
32919	0	7.92	1	0.75634	.	.	1
32920	0	7.92	1	0.75634	.	.	1
32921	0	7.92	1	0.75634	.	.	1
32922	0	7.92	1	0.75634	.	.	1
32923	0	7.92	1	0.75634	.	.	1
32924	0	7.93	0	0.75611	0.75065	0.76146	1
32925	0	7.93	1	0.75611	.	.	1
32926	0	7.93	1	0.75611	.	.	1
32927	0	7.93	1	0.75611	.	.	1
32928	0	7.93	1	0.75611	.	.	1
32929	0	7.93	1	0.75611	.	.	1
32930	0	7.93	1	0.75611	.	.	1
32931	0	7.93	1	0.75611	.	.	1
32932	0	7.93	1	0.75611	.	.	1
32933	0	7.93	1	0.75611	.	.	1
32934	0	7.93	1	0.75611	.	.	1
32935	0	7.93	1	0.75611	.	.	1

Obs	Sub_group	fuptimey	_CENSOR_	SURVIVAL	SDF_LCL	SDF_UCL	STRATUM
32936	0	7.93	1	0.75611	.	.	1
32937	0	7.93	1	0.75611	.	.	1
32938	0	7.93	1	0.75611	.	.	1
32939	0	7.93	1	0.75611	.	.	1
32940	0	7.93	1	0.75611	.	.	1
32941	0	7.93	1	0.75611	.	.	1
32942	0	7.93	1	0.75611	.	.	1
32943	0	7.93	1	0.75611	.	.	1
32944	0	7.93	1	0.75611	.	.	1
32945	0	7.93	1	0.75611	.	.	1
32946	0	7.93	1	0.75611	.	.	1
32947	0	7.93	1	0.75611	.	.	1
32948	0	7.93	1	0.75611	.	.	1
32949	0	7.93	1	0.75611	.	.	1
32950	0	7.93	1	0.75611	.	.	1
32951	0	7.93	1	0.75611	.	.	1
32952	0	7.93	1	0.75611	.	.	1
32953	0	7.93	1	0.75611	.	.	1
32954	0	7.94	0	0.75576	0.75030	0.76113	1
32955	0	7.94	1	0.75576	.	.	1
32956	0	7.94	1	0.75576	.	.	1
32957	0	7.94	1	0.75576	.	.	1
32958	0	7.94	1	0.75576	.	.	1
32959	0	7.94	1	0.75576	.	.	1
32960	0	7.94	1	0.75576	.	.	1
32961	0	7.94	1	0.75576	.	.	1
32962	0	7.94	1	0.75576	.	.	1
32963	0	7.94	1	0.75576	.	.	1
32964	0	7.94	1	0.75576	.	.	1
32965	0	7.94	1	0.75576	.	.	1
32966	0	7.94	1	0.75576	.	.	1
32967	0	7.94	1	0.75576	.	.	1
32968	0	7.94	1	0.75576	.	.	1
32969	0	7.94	1	0.75576	.	.	1
32970	0	7.94	1	0.75576	.	.	1

Obs	Sub_group	fuptimey	_CENSOR_	SURVIVAL	SDF_LCL	SDF_UCL	STRATUM
32971	0	7.94	1	0.75576	.	.	1
32972	0	7.94	1	0.75576	.	.	1
32973	0	7.94	1	0.75576	.	.	1
32974	0	7.94	1	0.75576	.	.	1
32975	0	7.94	1	0.75576	.	.	1
32976	0	7.94	1	0.75576	.	.	1
32977	0	7.94	1	0.75576	.	.	1
32978	0	7.94	1	0.75576	.	.	1
32979	0	7.94	1	0.75576	.	.	1
32980	0	7.94	1	0.75576	.	.	1
32981	0	7.94	1	0.75576	.	.	1
32982	0	7.94	1	0.75576	.	.	1
32983	0	7.94	1	0.75576	.	.	1
32984	0	7.94	1	0.75576	.	.	1
32985	0	7.94	1	0.75576	.	.	1
32986	0	7.94	1	0.75576	.	.	1
32987	0	7.94	1	0.75576	.	.	1
32988	0	7.94	1	0.75576	.	.	1
32989	0	7.94	1	0.75576	.	.	1
32990	0	7.94	1	0.75576	.	.	1
32991	0	7.94	1	0.75576	.	.	1
32992	0	7.94	1	0.75576	.	.	1
32993	0	7.94	1	0.75576	.	.	1
32994	0	7.94	1	0.75576	.	.	1
32995	0	7.94	1	0.75576	.	.	1
32996	0	7.94	1	0.75576	.	.	1
32997	0	7.95	0	0.75542	0.74994	0.76079	1
32998	0	7.95	1	0.75542	.	.	1
32999	0	7.95	1	0.75542	.	.	1
33000	0	7.95	1	0.75542	.	.	1
33001	0	7.95	1	0.75542	.	.	1
33002	0	7.95	1	0.75542	.	.	1
33003	0	7.95	1	0.75542	.	.	1
33004	0	7.95	1	0.75542	.	.	1
33005	0	7.95	1	0.75542	.	.	1

Obs	Sub_group	fuptimey	_CENSOR_	SURVIVAL	SDF_LCL	SDF_UCL	STRATUM
33006	0	7.95	1	0.75542	.	.	1
33007	0	7.95	1	0.75542	.	.	1
33008	0	7.95	1	0.75542	.	.	1
33009	0	7.95	1	0.75542	.	.	1
33010	0	7.95	1	0.75542	.	.	1
33011	0	7.95	1	0.75542	.	.	1
33012	0	7.95	1	0.75542	.	.	1
33013	0	7.95	1	0.75542	.	.	1
33014	0	7.95	1	0.75542	.	.	1
33015	0	7.95	1	0.75542	.	.	1
33016	0	7.95	1	0.75542	.	.	1
33017	0	7.95	1	0.75542	.	.	1
33018	0	7.95	1	0.75542	.	.	1
33019	0	7.95	1	0.75542	.	.	1
33020	0	7.95	1	0.75542	.	.	1
33021	0	7.95	1	0.75542	.	.	1
33022	0	7.95	1	0.75542	.	.	1
33023	0	7.95	1	0.75542	.	.	1
33024	0	7.95	1	0.75542	.	.	1
33025	0	7.96	0	0.75507	0.74958	0.76046	1
33026	0	7.96	1	0.75507	.	.	1
33027	0	7.96	1	0.75507	.	.	1
33028	0	7.96	1	0.75507	.	.	1
33029	0	7.96	1	0.75507	.	.	1
33030	0	7.96	1	0.75507	.	.	1
33031	0	7.96	1	0.75507	.	.	1
33032	0	7.96	1	0.75507	.	.	1
33033	0	7.96	1	0.75507	.	.	1
33034	0	7.96	1	0.75507	.	.	1
33035	0	7.96	1	0.75507	.	.	1
33036	0	7.96	1	0.75507	.	.	1
33037	0	7.96	1	0.75507	.	.	1
33038	0	7.96	1	0.75507	.	.	1
33039	0	7.96	1	0.75507	.	.	1
33040	0	7.96	1	0.75507	.	.	1

Obs	Sub_group	fuptimey	_CENSOR_	SURVIVAL	SDF_LCL	SDF_UCL	STRATUM
33041	0	7.96	1	0.75507	.	.	1
33042	0	7.96	1	0.75507	.	.	1
33043	0	7.96	1	0.75507	.	.	1
33044	0	7.96	1	0.75507	.	.	1
33045	0	7.96	1	0.75507	.	.	1
33046	0	7.96	1	0.75507	.	.	1
33047	0	7.96	1	0.75507	.	.	1
33048	0	7.96	1	0.75507	.	.	1
33049	0	7.96	1	0.75507	.	.	1
33050	0	7.96	1	0.75507	.	.	1
33051	0	7.96	1	0.75507	.	.	1
33052	0	7.96	1	0.75507	.	.	1
33053	0	7.96	1	0.75507	.	.	1
33054	0	7.96	1	0.75507	.	.	1
33055	0	7.96	1	0.75507	.	.	1
33056	0	7.96	1	0.75507	.	.	1
33057	0	7.96	1	0.75507	.	.	1
33058	0	7.96	1	0.75507	.	.	1
33059	0	7.96	1	0.75507	.	.	1
33060	0	7.96	1	0.75507	.	.	1
33061	0	7.96	1	0.75507	.	.	1
33062	0	7.96	1	0.75507	.	.	1
33063	0	7.96	1	0.75507	.	.	1
33064	0	7.96	1	0.75507	.	.	1
33065	0	7.96	1	0.75507	.	.	1
33066	0	7.97	0	0.75495	0.74946	0.76034	1
33067	0	7.97	1	0.75495	.	.	1
33068	0	7.97	1	0.75495	.	.	1
33069	0	7.97	1	0.75495	.	.	1
33070	0	7.97	1	0.75495	.	.	1
33071	0	7.97	1	0.75495	.	.	1
33072	0	7.97	1	0.75495	.	.	1
33073	0	7.97	1	0.75495	.	.	1
33074	0	7.97	1	0.75495	.	.	1
33075	0	7.97	1	0.75495	.	.	1

Obs	Sub_group	fuptimey	_CENSOR_	SURVIVAL	SDF_LCL	SDF_UCL	STRATUM
33076	0	7.97	1	0.75495	.	.	1
33077	0	7.97	1	0.75495	.	.	1
33078	0	7.97	1	0.75495	.	.	1
33079	0	7.97	1	0.75495	.	.	1
33080	0	7.97	1	0.75495	.	.	1
33081	0	7.97	1	0.75495	.	.	1
33082	0	7.97	1	0.75495	.	.	1
33083	0	7.97	1	0.75495	.	.	1
33084	0	7.97	1	0.75495	.	.	1
33085	0	7.97	1	0.75495	.	.	1
33086	0	7.97	1	0.75495	.	.	1
33087	0	7.97	1	0.75495	.	.	1
33088	0	7.97	1	0.75495	.	.	1
33089	0	7.97	1	0.75495	.	.	1
33090	0	7.97	1	0.75495	.	.	1
33091	0	7.97	1	0.75495	.	.	1
33092	0	7.97	1	0.75495	.	.	1
33093	0	7.97	1	0.75495	.	.	1
33094	0	7.98	0	0.75460	0.74909	0.76000	1
33095	0	7.98	1	0.75460	.	.	1
33096	0	7.98	1	0.75460	.	.	1
33097	0	7.98	1	0.75460	.	.	1
33098	0	7.98	1	0.75460	.	.	1
33099	0	7.98	1	0.75460	.	.	1
33100	0	7.98	1	0.75460	.	.	1
33101	0	7.98	1	0.75460	.	.	1
33102	0	7.98	1	0.75460	.	.	1
33103	0	7.98	1	0.75460	.	.	1
33104	0	7.98	1	0.75460	.	.	1
33105	0	7.98	1	0.75460	.	.	1
33106	0	7.98	1	0.75460	.	.	1
33107	0	7.98	1	0.75460	.	.	1
33108	0	7.98	1	0.75460	.	.	1
33109	0	7.98	1	0.75460	.	.	1
33110	0	7.98	1	0.75460	.	.	1

Obs	Sub_group	fuptimey	_CENSOR_	SURVIVAL	SDF_LCL	SDF_UCL	STRATUM
33111	0	7.98	1	0.75460	.	.	1
33112	0	7.98	1	0.75460	.	.	1
33113	0	7.98	1	0.75460	.	.	1
33114	0	7.98	1	0.75460	.	.	1
33115	0	7.98	1	0.75460	.	.	1
33116	0	7.98	1	0.75460	.	.	1
33117	0	7.98	1	0.75460	.	.	1
33118	0	7.98	1	0.75460	.	.	1
33119	0	7.98	1	0.75460	.	.	1
33120	0	7.98	1	0.75460	.	.	1
33121	0	7.98	1	0.75460	.	.	1
33122	0	7.98	1	0.75460	.	.	1
33123	0	7.98	1	0.75460	.	.	1
33124	0	7.98	1	0.75460	.	.	1
33125	0	7.98	1	0.75460	.	.	1
33126	0	7.98	1	0.75460	.	.	1
33127	0	7.98	1	0.75460	.	.	1
33128	0	7.98	1	0.75460	.	.	1
33129	0	7.98	1	0.75460	.	.	1
33130	0	7.98	1	0.75460	.	.	1
33131	0	7.98	1	0.75460	.	.	1
33132	0	7.98	1	0.75460	.	.	1
33133	0	7.98	1	0.75460	.	.	1
33134	0	7.98	1	0.75460	.	.	1
33135	0	7.98	1	0.75460	.	.	1
33136	0	7.98	1	0.75460	.	.	1
33137	0	7.99	0	0.75424	0.74873	0.75966	1
33138	0	7.99	1	0.75424	.	.	1
33139	0	7.99	1	0.75424	.	.	1
33140	0	7.99	1	0.75424	.	.	1
33141	0	7.99	1	0.75424	.	.	1
33142	0	7.99	1	0.75424	.	.	1
33143	0	7.99	1	0.75424	.	.	1
33144	0	7.99	1	0.75424	.	.	1
33145	0	7.99	1	0.75424	.	.	1

Obs	Sub_group	fuptimey	_CENSOR_	SURVIVAL	SDF_LCL	SDF_UCL	STRATUM
33146	0	7.99	1	0.75424	.	.	1
33147	0	7.99	1	0.75424	.	.	1
33148	0	7.99	1	0.75424	.	.	1
33149	0	7.99	1	0.75424	.	.	1
33150	0	7.99	1	0.75424	.	.	1
33151	0	7.99	1	0.75424	.	.	1
33152	0	7.99	1	0.75424	.	.	1
33153	0	7.99	1	0.75424	.	.	1
33154	0	7.99	1	0.75424	.	.	1
33155	0	7.99	1	0.75424	.	.	1
33156	0	7.99	1	0.75424	.	.	1
33157	0	7.99	1	0.75424	.	.	1
33158	0	7.99	1	0.75424	.	.	1
33159	0	7.99	1	0.75424	.	.	1
33160	0	7.99	1	0.75424	.	.	1
33161	0	7.99	1	0.75424	.	.	1
33162	0	7.99	1	0.75424	.	.	1
33163	0	7.99	1	0.75424	.	.	1
33164	0	7.99	1	0.75424	.	.	1
33165	0	7.99	1	0.75424	.	.	1
33166	0	7.99	1	0.75424	.	.	1
33167	0	7.99	1	0.75424	.	.	1
33168	0	7.99	1	0.75424	.	.	1
33169	0	8.00	0	0.75377	0.74823	0.75920	1
33170	0	8.00	1	0.75377	.	.	1
33171	0	8.00	1	0.75377	.	.	1
33172	0	8.00	1	0.75377	.	.	1
33173	0	8.00	1	0.75377	.	.	1
33174	0	8.00	1	0.75377	.	.	1
33175	0	8.00	1	0.75377	.	.	1
33176	0	8.00	1	0.75377	.	.	1
33177	0	8.00	1	0.75377	.	.	1
33178	0	8.00	1	0.75377	.	.	1
33179	0	8.00	1	0.75377	.	.	1
33180	0	8.00	1	0.75377	.	.	1

Obs	Sub_group	fuptimey	_CENSOR_	SURVIVAL	SDF_LCL	SDF_UCL	STRATUM
**33181**	0	8.00	1	0.75377	.	.	1
**33182**	0	8.00	1	0.75377	.	.	1
**33183**	0	8.00	1	0.75377	.	.	1
**33184**	0	8.00	1	0.75377	.	.	1
**33185**	0	8.00	1	0.75377	.	.	1
**33186**	0	8.00	1	0.75377	.	.	1
**33187**	0	8.00	1	0.75377	.	.	1
**33188**	0	8.00	1	0.75377	.	.	1
**33189**	0	8.00	1	0.75377	.	.	1
**33190**	0	8.00	1	0.75377	.	.	1
**33191**	0	8.00	1	0.75377	.	.	1
**33192**	0	8.00	1	0.75377	.	.	1
**33193**	0	8.00	1	0.75377	.	.	1
**33194**	0	8.00	1	0.75377	.	.	1
**33195**	0	8.00	1	0.75377	.	.	1
**33196**	0	8.00	1	0.75377	.	.	1
**33197**	0	8.00	1	0.75377	.	.	1
**33198**	0	8.00	1	0.75377	.	.	1
**33199**	0	8.00	1	0.75377	.	.	1
**33200**	0	8.00	1	0.75377	.	.	1
**33201**	0	8.00	1	0.75377	.	.	1
**33202**	0	8.00	1	0.75377	.	.	1
**33203**	0	8.00	1	0.75377	.	.	1
**33204**	0	8.00	1	0.75377	.	.	1
**33205**	0	8.00	1	0.75377	.	.	1
**33206**	0	8.00	1	0.75377	.	.	1
**33207**	0	8.00	1	0.75377	.	.	1
**33208**	0	8.00	1	0.75377	.	.	1
**33209**	0	8.01	1	0.75377	.	.	1
**33210**	0	8.01	1	0.75377	.	.	1
**33211**	0	8.01	1	0.75377	.	.	1
**33212**	0	8.01	1	0.75377	.	.	1
**33213**	0	8.01	1	0.75377	.	.	1
**33214**	0	8.01	1	0.75377	.	.	1
**33215**	0	8.01	1	0.75377	.	.	1

Obs	Sub_group	fuptimey	_CENSOR_	SURVIVAL	SDF_LCL	SDF_UCL	STRATUM
33216	0	8.01	1	0.75377	.	.	1
33217	0	8.01	1	0.75377	.	.	1
33218	0	8.01	1	0.75377	.	.	1
33219	0	8.01	1	0.75377	.	.	1
33220	0	8.01	1	0.75377	.	.	1
33221	0	8.01	1	0.75377	.	.	1
33222	0	8.01	1	0.75377	.	.	1
33223	0	8.01	1	0.75377	.	.	1
33224	0	8.01	1	0.75377	.	.	1
33225	0	8.01	1	0.75377	.	.	1
33226	0	8.01	1	0.75377	.	.	1
33227	0	8.01	1	0.75377	.	.	1
33228	0	8.01	1	0.75377	.	.	1
33229	0	8.01	1	0.75377	.	.	1
33230	0	8.01	1	0.75377	.	.	1
33231	0	8.01	1	0.75377	.	.	1
33232	0	8.01	1	0.75377	.	.	1
33233	0	8.01	1	0.75377	.	.	1
33234	0	8.01	1	0.75377	.	.	1
33235	0	8.01	1	0.75377	.	.	1
33236	0	8.02	0	0.75341	0.74786	0.75885	1
33237	0	8.02	1	0.75341	.	.	1
33238	0	8.02	1	0.75341	.	.	1
33239	0	8.02	1	0.75341	.	.	1
33240	0	8.02	1	0.75341	.	.	1
33241	0	8.02	1	0.75341	.	.	1
33242	0	8.02	1	0.75341	.	.	1
33243	0	8.02	1	0.75341	.	.	1
33244	0	8.02	1	0.75341	.	.	1
33245	0	8.02	1	0.75341	.	.	1
33246	0	8.02	1	0.75341	.	.	1
33247	0	8.02	1	0.75341	.	.	1
33248	0	8.02	1	0.75341	.	.	1
33249	0	8.02	1	0.75341	.	.	1
33250	0	8.02	1	0.75341	.	.	1

Obs	Sub_group	fuptimey	_CENSOR_	SURVIVAL	SDF_LCL	SDF_UCL	STRATUM
33251	0	8.02	1	0.75341	.	.	1
33252	0	8.02	1	0.75341	.	.	1
33253	0	8.02	1	0.75341	.	.	1
33254	0	8.02	1	0.75341	.	.	1
33255	0	8.02	1	0.75341	.	.	1
33256	0	8.02	1	0.75341	.	.	1
33257	0	8.02	1	0.75341	.	.	1
33258	0	8.02	1	0.75341	.	.	1
33259	0	8.02	1	0.75341	.	.	1
33260	0	8.02	1	0.75341	.	.	1
33261	0	8.02	1	0.75341	.	.	1
33262	0	8.02	1	0.75341	.	.	1
33263	0	8.02	1	0.75341	.	.	1
33264	0	8.02	1	0.75341	.	.	1
33265	0	8.03	0	0.75329	0.74774	0.75874	1
33266	0	8.03	1	0.75329	.	.	1
33267	0	8.03	1	0.75329	.	.	1
33268	0	8.03	1	0.75329	.	.	1
33269	0	8.03	1	0.75329	.	.	1
33270	0	8.03	1	0.75329	.	.	1
33271	0	8.03	1	0.75329	.	.	1
33272	0	8.03	1	0.75329	.	.	1
33273	0	8.03	1	0.75329	.	.	1
33274	0	8.03	1	0.75329	.	.	1
33275	0	8.03	1	0.75329	.	.	1
33276	0	8.03	1	0.75329	.	.	1
33277	0	8.03	1	0.75329	.	.	1
33278	0	8.03	1	0.75329	.	.	1
33279	0	8.03	1	0.75329	.	.	1
33280	0	8.03	1	0.75329	.	.	1
33281	0	8.03	1	0.75329	.	.	1
33282	0	8.03	1	0.75329	.	.	1
33283	0	8.03	1	0.75329	.	.	1
33284	0	8.03	1	0.75329	.	.	1
33285	0	8.03	1	0.75329	.	.	1

Obs	Sub_group	fuptimey	_CENSOR_	SURVIVAL	SDF_LCL	SDF_UCL	STRATUM
33286	0	8.03	1	0.75329	.	.	1
33287	0	8.03	1	0.75329	.	.	1
33288	0	8.03	1	0.75329	.	.	1
33289	0	8.03	1	0.75329	.	.	1
33290	0	8.04	0	0.75304	0.74748	0.75850	1
33291	0	8.04	1	0.75304	.	.	1
33292	0	8.04	1	0.75304	.	.	1
33293	0	8.04	1	0.75304	.	.	1
33294	0	8.04	1	0.75304	.	.	1
33295	0	8.04	1	0.75304	.	.	1
33296	0	8.04	1	0.75304	.	.	1
33297	0	8.04	1	0.75304	.	.	1
33298	0	8.04	1	0.75304	.	.	1
33299	0	8.04	1	0.75304	.	.	1
33300	0	8.04	1	0.75304	.	.	1
33301	0	8.04	1	0.75304	.	.	1
33302	0	8.04	1	0.75304	.	.	1
33303	0	8.04	1	0.75304	.	.	1
33304	0	8.04	1	0.75304	.	.	1
33305	0	8.04	1	0.75304	.	.	1
33306	0	8.04	1	0.75304	.	.	1
33307	0	8.04	1	0.75304	.	.	1
33308	0	8.04	1	0.75304	.	.	1
33309	0	8.04	1	0.75304	.	.	1
33310	0	8.04	1	0.75304	.	.	1
33311	0	8.04	1	0.75304	.	.	1
33312	0	8.04	1	0.75304	.	.	1
33313	0	8.04	1	0.75304	.	.	1
33314	0	8.04	1	0.75304	.	.	1
33315	0	8.05	0	0.75268	0.74711	0.75815	1
33316	0	8.05	1	0.75268	.	.	1
33317	0	8.05	1	0.75268	.	.	1
33318	0	8.05	1	0.75268	.	.	1
33319	0	8.05	1	0.75268	.	.	1
33320	0	8.05	1	0.75268	.	.	1

Obs	Sub_group	fuptimey	_CENSOR_	SURVIVAL	SDF_LCL	SDF_UCL	STRATUM
33321	0	8.05	1	0.75268	.	.	1
33322	0	8.05	1	0.75268	.	.	1
33323	0	8.05	1	0.75268	.	.	1
33324	0	8.05	1	0.75268	.	.	1
33325	0	8.05	1	0.75268	.	.	1
33326	0	8.05	1	0.75268	.	.	1
33327	0	8.05	1	0.75268	.	.	1
33328	0	8.05	1	0.75268	.	.	1
33329	0	8.05	1	0.75268	.	.	1
33330	0	8.05	1	0.75268	.	.	1
33331	0	8.05	1	0.75268	.	.	1
33332	0	8.05	1	0.75268	.	.	1
33333	0	8.05	1	0.75268	.	.	1
33334	0	8.05	1	0.75268	.	.	1
33335	0	8.05	1	0.75268	.	.	1
33336	0	8.05	1	0.75268	.	.	1
33337	0	8.06	0	0.75256	0.74698	0.75803	1
33338	0	8.06	1	0.75256	.	.	1
33339	0	8.06	1	0.75256	.	.	1
33340	0	8.06	1	0.75256	.	.	1
33341	0	8.06	1	0.75256	.	.	1
33342	0	8.06	1	0.75256	.	.	1
33343	0	8.06	1	0.75256	.	.	1
33344	0	8.06	1	0.75256	.	.	1
33345	0	8.06	1	0.75256	.	.	1
33346	0	8.06	1	0.75256	.	.	1
33347	0	8.06	1	0.75256	.	.	1
33348	0	8.06	1	0.75256	.	.	1
33349	0	8.06	1	0.75256	.	.	1
33350	0	8.06	1	0.75256	.	.	1
33351	0	8.06	1	0.75256	.	.	1
33352	0	8.06	1	0.75256	.	.	1
33353	0	8.06	1	0.75256	.	.	1
33354	0	8.06	1	0.75256	.	.	1
33355	0	8.06	1	0.75256	.	.	1

Obs	Sub_group	fuptimey	_CENSOR_	SURVIVAL	SDF_LCL	SDF_UCL	STRATUM
33356	0	8.06	1	0.75256	.	.	1
33357	0	8.06	1	0.75256	.	.	1
33358	0	8.06	1	0.75256	.	.	1
33359	0	8.06	1	0.75256	.	.	1
33360	0	8.06	1	0.75256	.	.	1
33361	0	8.06	1	0.75256	.	.	1
33362	0	8.06	1	0.75256	.	.	1
33363	0	8.06	1	0.75256	.	.	1
33364	0	8.06	1	0.75256	.	.	1
33365	0	8.06	1	0.75256	.	.	1
33366	0	8.06	1	0.75256	.	.	1
33367	0	8.06	1	0.75256	.	.	1
33368	0	8.06	1	0.75256	.	.	1
33369	0	8.06	1	0.75256	.	.	1
33370	0	8.06	1	0.75256	.	.	1
33371	0	8.06	1	0.75256	.	.	1
33372	0	8.06	1	0.75256	.	.	1
33373	0	8.06	1	0.75256	.	.	1
33374	0	8.06	1	0.75256	.	.	1
33375	0	8.06	1	0.75256	.	.	1
33376	0	8.07	0	0.75244	0.74685	0.75791	1
33377	0	8.07	1	0.75244	.	.	1
33378	0	8.07	1	0.75244	.	.	1
33379	0	8.07	1	0.75244	.	.	1
33380	0	8.07	1	0.75244	.	.	1
33381	0	8.07	1	0.75244	.	.	1
33382	0	8.07	1	0.75244	.	.	1
33383	0	8.07	1	0.75244	.	.	1
33384	0	8.07	1	0.75244	.	.	1
33385	0	8.07	1	0.75244	.	.	1
33386	0	8.07	1	0.75244	.	.	1
33387	0	8.07	1	0.75244	.	.	1
33388	0	8.07	1	0.75244	.	.	1
33389	0	8.07	1	0.75244	.	.	1
33390	0	8.07	1	0.75244	.	.	1

Obs	Sub_group	fuptimey	_CENSOR_	SURVIVAL	SDF_LCL	SDF_UCL	STRATUM
33391	0	8.07	1	0.75244	.	.	1
33392	0	8.07	1	0.75244	.	.	1
33393	0	8.07	1	0.75244	.	.	1
33394	0	8.07	1	0.75244	.	.	1
33395	0	8.07	1	0.75244	.	.	1
33396	0	8.07	1	0.75244	.	.	1
33397	0	8.07	1	0.75244	.	.	1
33398	0	8.07	1	0.75244	.	.	1
33399	0	8.07	1	0.75244	.	.	1
33400	0	8.07	1	0.75244	.	.	1
33401	0	8.07	1	0.75244	.	.	1
33402	0	8.07	1	0.75244	.	.	1
33403	0	8.08	0	0.75219	0.74660	0.75768	1
33404	0	8.08	1	0.75219	.	.	1
33405	0	8.08	1	0.75219	.	.	1
33406	0	8.08	1	0.75219	.	.	1
33407	0	8.08	1	0.75219	.	.	1
33408	0	8.08	1	0.75219	.	.	1
33409	0	8.08	1	0.75219	.	.	1
33410	0	8.08	1	0.75219	.	.	1
33411	0	8.08	1	0.75219	.	.	1
33412	0	8.08	1	0.75219	.	.	1
33413	0	8.08	1	0.75219	.	.	1
33414	0	8.08	1	0.75219	.	.	1
33415	0	8.08	1	0.75219	.	.	1
33416	0	8.08	1	0.75219	.	.	1
33417	0	8.08	1	0.75219	.	.	1
33418	0	8.08	1	0.75219	.	.	1
33419	0	8.08	1	0.75219	.	.	1
33420	0	8.08	1	0.75219	.	.	1
33421	0	8.08	1	0.75219	.	.	1
33422	0	8.08	1	0.75219	.	.	1
33423	0	8.08	1	0.75219	.	.	1
33424	0	8.08	1	0.75219	.	.	1
33425	0	8.08	1	0.75219	.	.	1

Obs	Sub_group	fuptimey	_CENSOR_	SURVIVAL	SDF_LCL	SDF_UCL	STRATUM
33426	0	8.08	1	0.75219	.	.	1
33427	0	8.08	1	0.75219	.	.	1
33428	0	8.08	1	0.75219	.	.	1
33429	0	8.08	1	0.75219	.	.	1
33430	0	8.08	1	0.75219	.	.	1
33431	0	8.08	1	0.75219	.	.	1
33432	0	8.08	1	0.75219	.	.	1
33433	0	8.08	1	0.75219	.	.	1
33434	0	8.08	1	0.75219	.	.	1
33435	0	8.09	0	0.75182	0.74621	0.75732	1
33436	0	8.09	1	0.75182	.	.	1
33437	0	8.09	1	0.75182	.	.	1
33438	0	8.09	1	0.75182	.	.	1
33439	0	8.09	1	0.75182	.	.	1
33440	0	8.09	1	0.75182	.	.	1
33441	0	8.09	1	0.75182	.	.	1
33442	0	8.09	1	0.75182	.	.	1
33443	0	8.09	1	0.75182	.	.	1
33444	0	8.09	1	0.75182	.	.	1
33445	0	8.09	1	0.75182	.	.	1
33446	0	8.09	1	0.75182	.	.	1
33447	0	8.09	1	0.75182	.	.	1
33448	0	8.09	1	0.75182	.	.	1
33449	0	8.09	1	0.75182	.	.	1
33450	0	8.09	1	0.75182	.	.	1
33451	0	8.09	1	0.75182	.	.	1
33452	0	8.09	1	0.75182	.	.	1
33453	0	8.09	1	0.75182	.	.	1
33454	0	8.09	1	0.75182	.	.	1
33455	0	8.09	1	0.75182	.	.	1
33456	0	8.09	1	0.75182	.	.	1
33457	0	8.09	1	0.75182	.	.	1
33458	0	8.09	1	0.75182	.	.	1
33459	0	8.09	1	0.75182	.	.	1
33460	0	8.09	1	0.75182	.	.	1

Obs	Sub_group	fuptimey	_CENSOR_	SURVIVAL	SDF_LCL	SDF_UCL	STRATUM
33461	0	8.09	1	0.75182	.	.	1
33462	0	8.09	1	0.75182	.	.	1
33463	0	8.09	1	0.75182	.	.	1
33464	0	8.09	1	0.75182	.	.	1
33465	0	8.09	1	0.75182	.	.	1
33466	0	8.10	0	0.75157	0.74596	0.75708	1
33467	0	8.10	1	0.75157	.	.	1
33468	0	8.10	1	0.75157	.	.	1
33469	0	8.10	1	0.75157	.	.	1
33470	0	8.10	1	0.75157	.	.	1
33471	0	8.10	1	0.75157	.	.	1
33472	0	8.10	1	0.75157	.	.	1
33473	0	8.10	1	0.75157	.	.	1
33474	0	8.10	1	0.75157	.	.	1
33475	0	8.10	1	0.75157	.	.	1
33476	0	8.10	1	0.75157	.	.	1
33477	0	8.10	1	0.75157	.	.	1
33478	0	8.10	1	0.75157	.	.	1
33479	0	8.10	1	0.75157	.	.	1
33480	0	8.10	1	0.75157	.	.	1
33481	0	8.10	1	0.75157	.	.	1
33482	0	8.10	1	0.75157	.	.	1
33483	0	8.10	1	0.75157	.	.	1
33484	0	8.10	1	0.75157	.	.	1
33485	0	8.10	1	0.75157	.	.	1
33486	0	8.10	1	0.75157	.	.	1
33487	0	8.10	1	0.75157	.	.	1
33488	0	8.10	1	0.75157	.	.	1
33489	0	8.10	1	0.75157	.	.	1
33490	0	8.10	1	0.75157	.	.	1
33491	0	8.10	1	0.75157	.	.	1
33492	0	8.10	1	0.75157	.	.	1
33493	0	8.10	1	0.75157	.	.	1
33494	0	8.10	1	0.75157	.	.	1
33495	0	8.11	0	0.75144	0.74583	0.75696	1

Obs	Sub_group	fuptimey	_CENSOR_	SURVIVAL	SDF_LCL	SDF_UCL	STRATUM
33496	0	8.11	1	0.75144	.	.	1
33497	0	8.11	1	0.75144	.	.	1
33498	0	8.11	1	0.75144	.	.	1
33499	0	8.11	1	0.75144	.	.	1
33500	0	8.11	1	0.75144	.	.	1
33501	0	8.11	1	0.75144	.	.	1
33502	0	8.11	1	0.75144	.	.	1
33503	0	8.11	1	0.75144	.	.	1
33504	0	8.11	1	0.75144	.	.	1
33505	0	8.11	1	0.75144	.	.	1
33506	0	8.11	1	0.75144	.	.	1
33507	0	8.11	1	0.75144	.	.	1
33508	0	8.11	1	0.75144	.	.	1
33509	0	8.11	1	0.75144	.	.	1
33510	0	8.11	1	0.75144	.	.	1
33511	0	8.11	1	0.75144	.	.	1
33512	0	8.11	1	0.75144	.	.	1
33513	0	8.11	1	0.75144	.	.	1
33514	0	8.11	1	0.75144	.	.	1
33515	0	8.11	1	0.75144	.	.	1
33516	0	8.11	1	0.75144	.	.	1
33517	0	8.11	1	0.75144	.	.	1
33518	0	8.11	1	0.75144	.	.	1
33519	0	8.11	1	0.75144	.	.	1
33520	0	8.11	1	0.75144	.	.	1
33521	0	8.11	1	0.75144	.	.	1
33522	0	8.11	1	0.75144	.	.	1
33523	0	8.11	1	0.75144	.	.	1
33524	0	8.11	1	0.75144	.	.	1
33525	0	8.11	1	0.75144	.	.	1
33526	0	8.11	1	0.75144	.	.	1
33527	0	8.11	1	0.75144	.	.	1
33528	0	8.11	1	0.75144	.	.	1
33529	0	8.11	1	0.75144	.	.	1
33530	0	8.11	1	0.75144	.	.	1

Obs	Sub_group	fuptimey	_CENSOR_	SURVIVAL	SDF_LCL	SDF_UCL	STRATUM
33531	0	8.11	1	0.75144	.	.	1
33532	0	8.11	1	0.75144	.	.	1
33533	0	8.12	0	0.75132	0.74570	0.75684	1
33534	0	8.12	1	0.75132	.	.	1
33535	0	8.12	1	0.75132	.	.	1
33536	0	8.12	1	0.75132	.	.	1
33537	0	8.12	1	0.75132	.	.	1
33538	0	8.12	1	0.75132	.	.	1
33539	0	8.12	1	0.75132	.	.	1
33540	0	8.12	1	0.75132	.	.	1
33541	0	8.12	1	0.75132	.	.	1
33542	0	8.12	1	0.75132	.	.	1
33543	0	8.12	1	0.75132	.	.	1
33544	0	8.12	1	0.75132	.	.	1
33545	0	8.12	1	0.75132	.	.	1
33546	0	8.12	1	0.75132	.	.	1
33547	0	8.12	1	0.75132	.	.	1
33548	0	8.12	1	0.75132	.	.	1
33549	0	8.12	1	0.75132	.	.	1
33550	0	8.12	1	0.75132	.	.	1
33551	0	8.12	1	0.75132	.	.	1
33552	0	8.12	1	0.75132	.	.	1
33553	0	8.12	1	0.75132	.	.	1
33554	0	8.12	1	0.75132	.	.	1
33555	0	8.12	1	0.75132	.	.	1
33556	0	8.12	1	0.75132	.	.	1
33557	0	8.12	1	0.75132	.	.	1
33558	0	8.12	1	0.75132	.	.	1
33559	0	8.12	1	0.75132	.	.	1
33560	0	8.12	1	0.75132	.	.	1
33561	0	8.12	1	0.75132	.	.	1
33562	0	8.12	1	0.75132	.	.	1
33563	0	8.13	0	0.75119	0.74556	0.75671	1
33564	0	8.13	1	0.75119	.	.	1
33565	0	8.13	1	0.75119	.	.	1

Obs	Sub_group	fuptimey	_CENSOR_	SURVIVAL	SDF_LCL	SDF_UCL	STRATUM
33566	0	8.13	1	0.75119	.	.	1
33567	0	8.13	1	0.75119	.	.	1
33568	0	8.13	1	0.75119	.	.	1
33569	0	8.13	1	0.75119	.	.	1
33570	0	8.13	1	0.75119	.	.	1
33571	0	8.13	1	0.75119	.	.	1
33572	0	8.13	1	0.75119	.	.	1
33573	0	8.13	1	0.75119	.	.	1
33574	0	8.13	1	0.75119	.	.	1
33575	0	8.13	1	0.75119	.	.	1
33576	0	8.13	1	0.75119	.	.	1
33577	0	8.13	1	0.75119	.	.	1
33578	0	8.13	1	0.75119	.	.	1
33579	0	8.13	1	0.75119	.	.	1
33580	0	8.13	1	0.75119	.	.	1
33581	0	8.13	1	0.75119	.	.	1
33582	0	8.13	1	0.75119	.	.	1
33583	0	8.13	1	0.75119	.	.	1
33584	0	8.13	1	0.75119	.	.	1
33585	0	8.13	1	0.75119	.	.	1
33586	0	8.13	1	0.75119	.	.	1
33587	0	8.13	1	0.75119	.	.	1
33588	0	8.13	1	0.75119	.	.	1
33589	0	8.13	1	0.75119	.	.	1
33590	0	8.13	1	0.75119	.	.	1
33591	0	8.13	1	0.75119	.	.	1
33592	0	8.13	1	0.75119	.	.	1
33593	0	8.13	1	0.75119	.	.	1
33594	0	8.13	1	0.75119	.	.	1
33595	0	8.13	1	0.75119	.	.	1
33596	0	8.13	1	0.75119	.	.	1
33597	0	8.13	1	0.75119	.	.	1
33598	0	8.13	1	0.75119	.	.	1
33599	0	8.13	1	0.75119	.	.	1
33600	0	8.13	1	0.75119	.	.	1

Obs	Sub_group	fuptimey	_CENSOR_	SURVIVAL	SDF_LCL	SDF_UCL	STRATUM
33601	0	8.13	1	0.75119	.	.	1
33602	0	8.13	1	0.75119	.	.	1
33603	0	8.13	1	0.75119	.	.	1
33604	0	8.13	1	0.75119	.	.	1
33605	0	8.13	1	0.75119	.	.	1
33606	0	8.13	1	0.75119	.	.	1
33607	0	8.13	1	0.75119	.	.	1
33608	0	8.13	1	0.75119	.	.	1
33609	0	8.14	0	0.75106	0.74543	0.75659	1
33610	0	8.14	1	0.75106	.	.	1
33611	0	8.14	1	0.75106	.	.	1
33612	0	8.14	1	0.75106	.	.	1
33613	0	8.14	1	0.75106	.	.	1
33614	0	8.14	1	0.75106	.	.	1
33615	0	8.14	1	0.75106	.	.	1
33616	0	8.14	1	0.75106	.	.	1
33617	0	8.14	1	0.75106	.	.	1
33618	0	8.14	1	0.75106	.	.	1
33619	0	8.14	1	0.75106	.	.	1
33620	0	8.14	1	0.75106	.	.	1
33621	0	8.14	1	0.75106	.	.	1
33622	0	8.14	1	0.75106	.	.	1
33623	0	8.14	1	0.75106	.	.	1
33624	0	8.14	1	0.75106	.	.	1
33625	0	8.14	1	0.75106	.	.	1
33626	0	8.14	1	0.75106	.	.	1
33627	0	8.14	1	0.75106	.	.	1
33628	0	8.14	1	0.75106	.	.	1
33629	0	8.14	1	0.75106	.	.	1
33630	0	8.14	1	0.75106	.	.	1
33631	0	8.14	1	0.75106	.	.	1
33632	0	8.14	1	0.75106	.	.	1
33633	0	8.14	1	0.75106	.	.	1
33634	0	8.14	1	0.75106	.	.	1
33635	0	8.14	1	0.75106	.	.	1

Obs	Sub_group	fuptimey	_CENSOR_	SURVIVAL	SDF_LCL	SDF_UCL	STRATUM
33636	0	8.14	1	0.75106	.	.	1
33637	0	8.14	1	0.75106	.	.	1
33638	0	8.14	1	0.75106	.	.	1
33639	0	8.14	1	0.75106	.	.	1
33640	0	8.15	0	0.75055	0.74490	0.75610	1
33641	0	8.15	1	0.75055	.	.	1
33642	0	8.15	1	0.75055	.	.	1
33643	0	8.15	1	0.75055	.	.	1
33644	0	8.15	1	0.75055	.	.	1
33645	0	8.15	1	0.75055	.	.	1
33646	0	8.15	1	0.75055	.	.	1
33647	0	8.15	1	0.75055	.	.	1
33648	0	8.15	1	0.75055	.	.	1
33649	0	8.15	1	0.75055	.	.	1
33650	0	8.15	1	0.75055	.	.	1
33651	0	8.15	1	0.75055	.	.	1
33652	0	8.15	1	0.75055	.	.	1
33653	0	8.15	1	0.75055	.	.	1
33654	0	8.15	1	0.75055	.	.	1
33655	0	8.15	1	0.75055	.	.	1
33656	0	8.15	1	0.75055	.	.	1
33657	0	8.15	1	0.75055	.	.	1
33658	0	8.15	1	0.75055	.	.	1
33659	0	8.15	1	0.75055	.	.	1
33660	0	8.15	1	0.75055	.	.	1
33661	0	8.15	1	0.75055	.	.	1
33662	0	8.15	1	0.75055	.	.	1
33663	0	8.15	1	0.75055	.	.	1
33664	0	8.15	1	0.75055	.	.	1
33665	0	8.15	1	0.75055	.	.	1
33666	0	8.15	1	0.75055	.	.	1
33667	0	8.15	1	0.75055	.	.	1
33668	0	8.15	1	0.75055	.	.	1
33669	0	8.15	1	0.75055	.	.	1
33670	0	8.15	1	0.75055	.	.	1

Obs	Sub_group	fuptimey	_CENSOR_	SURVIVAL	SDF_LCL	SDF_UCL	STRATUM
33671	0	8.15	1	0.75055	.	.	1
33672	0	8.15	1	0.75055	.	.	1
33673	0	8.15	1	0.75055	.	.	1
33674	0	8.15	1	0.75055	.	.	1
33675	0	8.15	1	0.75055	.	.	1
33676	0	8.15	1	0.75055	.	.	1
33677	0	8.15	1	0.75055	.	.	1
33678	0	8.15	1	0.75055	.	.	1
33679	0	8.15	1	0.75055	.	.	1
33680	0	8.15	1	0.75055	.	.	1
33681	0	8.15	1	0.75055	.	.	1
33682	0	8.15	1	0.75055	.	.	1
33683	0	8.16	0	0.75016	0.74450	0.75572	1
33684	0	8.16	1	0.75016	.	.	1
33685	0	8.16	1	0.75016	.	.	1
33686	0	8.16	1	0.75016	.	.	1
33687	0	8.16	1	0.75016	.	.	1
33688	0	8.16	1	0.75016	.	.	1
33689	0	8.16	1	0.75016	.	.	1
33690	0	8.16	1	0.75016	.	.	1
33691	0	8.16	1	0.75016	.	.	1
33692	0	8.16	1	0.75016	.	.	1
33693	0	8.16	1	0.75016	.	.	1
33694	0	8.16	1	0.75016	.	.	1
33695	0	8.16	1	0.75016	.	.	1
33696	0	8.16	1	0.75016	.	.	1
33697	0	8.16	1	0.75016	.	.	1
33698	0	8.16	1	0.75016	.	.	1
33699	0	8.16	1	0.75016	.	.	1
33700	0	8.16	1	0.75016	.	.	1
33701	0	8.16	1	0.75016	.	.	1
33702	0	8.16	1	0.75016	.	.	1
33703	0	8.16	1	0.75016	.	.	1
33704	0	8.16	1	0.75016	.	.	1
33705	0	8.16	1	0.75016	.	.	1

Obs	Sub_group	fuptimey	_CENSOR_	SURVIVAL	SDF_LCL	SDF_UCL	STRATUM
33706	0	8.16	1	0.75016	.	.	1
33707	0	8.16	1	0.75016	.	.	1
33708	0	8.16	1	0.75016	.	.	1
33709	0	8.16	1	0.75016	.	.	1
33710	0	8.16	1	0.75016	.	.	1
33711	0	8.16	1	0.75016	.	.	1
33712	0	8.16	1	0.75016	.	.	1
33713	0	8.16	1	0.75016	.	.	1
33714	0	8.16	1	0.75016	.	.	1
33715	0	8.16	1	0.75016	.	.	1
33716	0	8.17	0	0.74990	0.74423	0.75547	1
33717	0	8.17	1	0.74990	.	.	1
33718	0	8.17	1	0.74990	.	.	1
33719	0	8.17	1	0.74990	.	.	1
33720	0	8.17	1	0.74990	.	.	1
33721	0	8.17	1	0.74990	.	.	1
33722	0	8.17	1	0.74990	.	.	1
33723	0	8.17	1	0.74990	.	.	1
33724	0	8.17	1	0.74990	.	.	1
33725	0	8.17	1	0.74990	.	.	1
33726	0	8.17	1	0.74990	.	.	1
33727	0	8.17	1	0.74990	.	.	1
33728	0	8.17	1	0.74990	.	.	1
33729	0	8.17	1	0.74990	.	.	1
33730	0	8.17	1	0.74990	.	.	1
33731	0	8.17	1	0.74990	.	.	1
33732	0	8.17	1	0.74990	.	.	1
33733	0	8.17	1	0.74990	.	.	1
33734	0	8.17	1	0.74990	.	.	1
33735	0	8.17	1	0.74990	.	.	1
33736	0	8.17	1	0.74990	.	.	1
33737	0	8.17	1	0.74990	.	.	1
33738	0	8.17	1	0.74990	.	.	1
33739	0	8.17	1	0.74990	.	.	1
33740	0	8.17	1	0.74990	.	.	1

Obs	Sub_group	fuptimey	_CENSOR_	SURVIVAL	SDF_LCL	SDF_UCL	STRATUM
33741	0	8.18	0	0.74964	0.74396	0.75522	1
33742	0	8.18	1	0.74964	.	.	1
33743	0	8.18	1	0.74964	.	.	1
33744	0	8.18	1	0.74964	.	.	1
33745	0	8.18	1	0.74964	.	.	1
33746	0	8.18	1	0.74964	.	.	1
33747	0	8.18	1	0.74964	.	.	1
33748	0	8.18	1	0.74964	.	.	1
33749	0	8.18	1	0.74964	.	.	1
33750	0	8.18	1	0.74964	.	.	1
33751	0	8.18	1	0.74964	.	.	1
33752	0	8.18	1	0.74964	.	.	1
33753	0	8.18	1	0.74964	.	.	1
33754	0	8.18	1	0.74964	.	.	1
33755	0	8.18	1	0.74964	.	.	1
33756	0	8.18	1	0.74964	.	.	1
33757	0	8.18	1	0.74964	.	.	1
33758	0	8.18	1	0.74964	.	.	1
33759	0	8.18	1	0.74964	.	.	1
33760	0	8.18	1	0.74964	.	.	1
33761	0	8.18	1	0.74964	.	.	1
33762	0	8.18	1	0.74964	.	.	1
33763	0	8.18	1	0.74964	.	.	1
33764	0	8.18	1	0.74964	.	.	1
33765	0	8.18	1	0.74964	.	.	1
33766	0	8.18	1	0.74964	.	.	1
33767	0	8.19	0	0.74938	0.74369	0.75497	1
33768	0	8.19	1	0.74938	.	.	1
33769	0	8.19	1	0.74938	.	.	1
33770	0	8.19	1	0.74938	.	.	1
33771	0	8.19	1	0.74938	.	.	1
33772	0	8.19	1	0.74938	.	.	1
33773	0	8.19	1	0.74938	.	.	1
33774	0	8.19	1	0.74938	.	.	1
33775	0	8.19	1	0.74938	.	.	1

Obs	Sub_group	fuptimey	_CENSOR_	SURVIVAL	SDF_LCL	SDF_UCL	STRATUM
**33776**	0	8.19	1	0.74938	.	.	1
**33777**	0	8.19	1	0.74938	.	.	1
**33778**	0	8.19	1	0.74938	.	.	1
**33779**	0	8.19	1	0.74938	.	.	1
**33780**	0	8.19	1	0.74938	.	.	1
**33781**	0	8.19	1	0.74938	.	.	1
**33782**	0	8.19	1	0.74938	.	.	1
**33783**	0	8.19	1	0.74938	.	.	1
**33784**	0	8.19	1	0.74938	.	.	1
**33785**	0	8.19	1	0.74938	.	.	1
**33786**	0	8.19	1	0.74938	.	.	1
**33787**	0	8.19	1	0.74938	.	.	1
**33788**	0	8.19	1	0.74938	.	.	1
**33789**	0	8.19	1	0.74938	.	.	1
**33790**	0	8.19	1	0.74938	.	.	1
**33791**	0	8.19	1	0.74938	.	.	1
**33792**	0	8.19	1	0.74938	.	.	1
**33793**	0	8.19	1	0.74938	.	.	1
**33794**	0	8.19	1	0.74938	.	.	1
**33795**	0	8.19	1	0.74938	.	.	1
**33796**	0	8.19	1	0.74938	.	.	1
**33797**	0	8.19	1	0.74938	.	.	1
**33798**	0	8.19	1	0.74938	.	.	1
**33799**	0	8.19	1	0.74938	.	.	1
**33800**	0	8.19	1	0.74938	.	.	1
**33801**	0	8.19	1	0.74938	.	.	1
**33802**	0	8.19	1	0.74938	.	.	1
**33803**	0	8.20	0	0.74912	0.74342	0.75472	1
**33804**	0	8.20	1	0.74912	.	.	1
**33805**	0	8.20	1	0.74912	.	.	1
**33806**	0	8.20	1	0.74912	.	.	1
**33807**	0	8.20	1	0.74912	.	.	1
**33808**	0	8.20	1	0.74912	.	.	1
**33809**	0	8.20	1	0.74912	.	.	1
**33810**	0	8.20	1	0.74912	.	.	1

Obs	Sub_group	fuptimey	_CENSOR_	SURVIVAL	SDF_LCL	SDF_UCL	STRATUM
**33811**	0	8.20	1	0.74912	.	.	1
**33812**	0	8.20	1	0.74912	.	.	1
**33813**	0	8.20	1	0.74912	.	.	1
**33814**	0	8.20	1	0.74912	.	.	1
**33815**	0	8.20	1	0.74912	.	.	1
**33816**	0	8.20	1	0.74912	.	.	1
**33817**	0	8.20	1	0.74912	.	.	1
**33818**	0	8.20	1	0.74912	.	.	1
**33819**	0	8.20	1	0.74912	.	.	1
**33820**	0	8.20	1	0.74912	.	.	1
**33821**	0	8.20	1	0.74912	.	.	1
**33822**	0	8.20	1	0.74912	.	.	1
**33823**	0	8.20	1	0.74912	.	.	1
**33824**	0	8.20	1	0.74912	.	.	1
**33825**	0	8.20	1	0.74912	.	.	1
**33826**	0	8.20	1	0.74912	.	.	1
**33827**	0	8.20	1	0.74912	.	.	1
**33828**	0	8.20	1	0.74912	.	.	1
**33829**	0	8.20	1	0.74912	.	.	1
**33830**	0	8.20	1	0.74912	.	.	1
**33831**	0	8.20	1	0.74912	.	.	1
**33832**	0	8.21	0	0.74899	0.74328	0.75459	1
**33833**	0	8.21	1	0.74899	.	.	1
**33834**	0	8.21	1	0.74899	.	.	1
**33835**	0	8.21	1	0.74899	.	.	1
**33836**	0	8.21	1	0.74899	.	.	1
**33837**	0	8.21	1	0.74899	.	.	1
**33838**	0	8.21	1	0.74899	.	.	1
**33839**	0	8.21	1	0.74899	.	.	1
**33840**	0	8.21	1	0.74899	.	.	1
**33841**	0	8.21	1	0.74899	.	.	1
**33842**	0	8.21	1	0.74899	.	.	1
**33843**	0	8.21	1	0.74899	.	.	1
**33844**	0	8.21	1	0.74899	.	.	1
**33845**	0	8.21	1	0.74899	.	.	1

Obs	Sub_group	fuptimey	_CENSOR_	SURVIVAL	SDF_LCL	SDF_UCL	STRATUM
**33846**	0	8.21	1	0.74899	.	.	1
**33847**	0	8.21	1	0.74899	.	.	1
**33848**	0	8.21	1	0.74899	.	.	1
**33849**	0	8.21	1	0.74899	.	.	1
**33850**	0	8.21	1	0.74899	.	.	1
**33851**	0	8.21	1	0.74899	.	.	1
**33852**	0	8.21	1	0.74899	.	.	1
**33853**	0	8.21	1	0.74899	.	.	1
**33854**	0	8.21	1	0.74899	.	.	1
**33855**	0	8.21	1	0.74899	.	.	1
**33856**	0	8.21	1	0.74899	.	.	1
**33857**	0	8.21	1	0.74899	.	.	1
**33858**	0	8.21	1	0.74899	.	.	1
**33859**	0	8.21	1	0.74899	.	.	1
**33860**	0	8.21	1	0.74899	.	.	1
**33861**	0	8.22	0	0.74885	0.74314	0.75446	1
**33862**	0	8.22	1	0.74885	.	.	1
**33863**	0	8.22	1	0.74885	.	.	1
**33864**	0	8.22	1	0.74885	.	.	1
**33865**	0	8.22	1	0.74885	.	.	1
**33866**	0	8.22	1	0.74885	.	.	1
**33867**	0	8.22	1	0.74885	.	.	1
**33868**	0	8.22	1	0.74885	.	.	1
**33869**	0	8.22	1	0.74885	.	.	1
**33870**	0	8.22	1	0.74885	.	.	1
**33871**	0	8.22	1	0.74885	.	.	1
**33872**	0	8.22	1	0.74885	.	.	1
**33873**	0	8.22	1	0.74885	.	.	1
**33874**	0	8.22	1	0.74885	.	.	1
**33875**	0	8.22	1	0.74885	.	.	1
**33876**	0	8.22	1	0.74885	.	.	1
**33877**	0	8.22	1	0.74885	.	.	1
**33878**	0	8.22	1	0.74885	.	.	1
**33879**	0	8.22	1	0.74885	.	.	1
**33880**	0	8.22	1	0.74885	.	.	1

Obs	Sub_group	fuptimey	_CENSOR_	SURVIVAL	SDF_LCL	SDF_UCL	STRATUM
33881	0	8.22	1	0.74885	.	.	1
33882	0	8.22	1	0.74885	.	.	1
33883	0	8.22	1	0.74885	.	.	1
33884	0	8.22	1	0.74885	.	.	1
33885	0	8.22	1	0.74885	.	.	1
33886	0	8.22	1	0.74885	.	.	1
33887	0	8.22	1	0.74885	.	.	1
33888	0	8.22	1	0.74885	.	.	1
33889	0	8.22	1	0.74885	.	.	1
33890	0	8.23	1	0.74885	.	.	1
33891	0	8.23	1	0.74885	.	.	1
33892	0	8.23	1	0.74885	.	.	1
33893	0	8.23	1	0.74885	.	.	1
33894	0	8.23	1	0.74885	.	.	1
33895	0	8.23	1	0.74885	.	.	1
33896	0	8.23	1	0.74885	.	.	1
33897	0	8.23	1	0.74885	.	.	1
33898	0	8.23	1	0.74885	.	.	1
33899	0	8.23	1	0.74885	.	.	1
33900	0	8.23	1	0.74885	.	.	1
33901	0	8.23	1	0.74885	.	.	1
33902	0	8.23	1	0.74885	.	.	1
33903	0	8.23	1	0.74885	.	.	1
33904	0	8.23	1	0.74885	.	.	1
33905	0	8.23	1	0.74885	.	.	1
33906	0	8.23	1	0.74885	.	.	1
33907	0	8.23	1	0.74885	.	.	1
33908	0	8.23	1	0.74885	.	.	1
33909	0	8.23	1	0.74885	.	.	1
33910	0	8.23	1	0.74885	.	.	1
33911	0	8.23	1	0.74885	.	.	1
33912	0	8.23	1	0.74885	.	.	1
33913	0	8.23	1	0.74885	.	.	1
33914	0	8.23	1	0.74885	.	.	1
33915	0	8.23	1	0.74885	.	.	1

Obs	Sub_group	fuptimey	_CENSOR_	SURVIVAL	SDF_LCL	SDF_UCL	STRATUM
33916	0	8.23	1	0.74885	.	.	1
33917	0	8.23	1	0.74885	.	.	1
33918	0	8.23	1	0.74885	.	.	1
33919	0	8.23	1	0.74885	.	.	1
33920	0	8.23	1	0.74885	.	.	1
33921	0	8.23	1	0.74885	.	.	1
33922	0	8.23	1	0.74885	.	.	1
33923	0	8.23	1	0.74885	.	.	1
33924	0	8.23	1	0.74885	.	.	1
33925	0	8.23	1	0.74885	.	.	1
33926	0	8.23	1	0.74885	.	.	1
33927	0	8.23	1	0.74885	.	.	1
33928	0	8.23	1	0.74885	.	.	1
33929	0	8.23	1	0.74885	.	.	1
33930	0	8.23	1	0.74885	.	.	1
33931	0	8.23	1	0.74885	.	.	1
33932	0	8.23	1	0.74885	.	.	1
33933	0	8.23	1	0.74885	.	.	1
33934	0	8.23	1	0.74885	.	.	1
33935	0	8.23	1	0.74885	.	.	1
33936	0	8.23	1	0.74885	.	.	1
33937	0	8.23	1	0.74885	.	.	1
33938	0	8.23	1	0.74885	.	.	1
33939	0	8.23	1	0.74885	.	.	1
33940	0	8.23	1	0.74885	.	.	1
33941	0	8.23	1	0.74885	.	.	1
33942	0	8.24	0	0.74872	0.74300	0.75433	1
33943	0	8.24	1	0.74872	.	.	1
33944	0	8.24	1	0.74872	.	.	1
33945	0	8.24	1	0.74872	.	.	1
33946	0	8.24	1	0.74872	.	.	1
33947	0	8.24	1	0.74872	.	.	1
33948	0	8.24	1	0.74872	.	.	1
33949	0	8.24	1	0.74872	.	.	1
33950	0	8.24	1	0.74872	.	.	1

Obs	Sub_group	fuptimey	_CENSOR_	SURVIVAL	SDF_LCL	SDF_UCL	STRATUM
33951	0	8.24	1	0.74872	.	.	1
33952	0	8.24	1	0.74872	.	.	1
33953	0	8.24	1	0.74872	.	.	1
33954	0	8.24	1	0.74872	.	.	1
33955	0	8.24	1	0.74872	.	.	1
33956	0	8.24	1	0.74872	.	.	1
33957	0	8.24	1	0.74872	.	.	1
33958	0	8.24	1	0.74872	.	.	1
33959	0	8.24	1	0.74872	.	.	1
33960	0	8.24	1	0.74872	.	.	1
33961	0	8.24	1	0.74872	.	.	1
33962	0	8.24	1	0.74872	.	.	1
33963	0	8.24	1	0.74872	.	.	1
33964	0	8.24	1	0.74872	.	.	1
33965	0	8.24	1	0.74872	.	.	1
33966	0	8.24	1	0.74872	.	.	1
33967	0	8.24	1	0.74872	.	.	1
33968	0	8.24	1	0.74872	.	.	1
33969	0	8.24	1	0.74872	.	.	1
33970	0	8.25	0	0.74858	0.74286	0.75420	1
33971	0	8.25	1	0.74858	.	.	1
33972	0	8.25	1	0.74858	.	.	1
33973	0	8.25	1	0.74858	.	.	1
33974	0	8.25	1	0.74858	.	.	1
33975	0	8.25	1	0.74858	.	.	1
33976	0	8.25	1	0.74858	.	.	1
33977	0	8.25	1	0.74858	.	.	1
33978	0	8.25	1	0.74858	.	.	1
33979	0	8.25	1	0.74858	.	.	1
33980	0	8.25	1	0.74858	.	.	1
33981	0	8.25	1	0.74858	.	.	1
33982	0	8.25	1	0.74858	.	.	1
33983	0	8.25	1	0.74858	.	.	1
33984	0	8.25	1	0.74858	.	.	1
33985	0	8.25	1	0.74858	.	.	1

Obs	Sub_group	fuptimey	_CENSOR_	SURVIVAL	SDF_LCL	SDF_UCL	STRATUM
33986	0	8.25	1	0.74858	.	.	1
33987	0	8.25	1	0.74858	.	.	1
33988	0	8.25	1	0.74858	.	.	1
33989	0	8.25	1	0.74858	.	.	1
33990	0	8.25	1	0.74858	.	.	1
33991	0	8.25	1	0.74858	.	.	1
33992	0	8.25	1	0.74858	.	.	1
33993	0	8.25	1	0.74858	.	.	1
33994	0	8.25	1	0.74858	.	.	1
33995	0	8.25	1	0.74858	.	.	1
33996	0	8.25	1	0.74858	.	.	1
33997	0	8.25	1	0.74858	.	.	1
33998	0	8.25	1	0.74858	.	.	1
33999	0	8.25	1	0.74858	.	.	1
34000	0	8.26	1	0.74858	.	.	1
34001	0	8.26	1	0.74858	.	.	1
34002	0	8.26	1	0.74858	.	.	1
34003	0	8.26	1	0.74858	.	.	1
34004	0	8.26	1	0.74858	.	.	1
34005	0	8.26	1	0.74858	.	.	1
34006	0	8.26	1	0.74858	.	.	1
34007	0	8.26	1	0.74858	.	.	1
34008	0	8.26	1	0.74858	.	.	1
34009	0	8.26	1	0.74858	.	.	1
34010	0	8.26	1	0.74858	.	.	1
34011	0	8.26	1	0.74858	.	.	1
34012	0	8.26	1	0.74858	.	.	1
34013	0	8.26	1	0.74858	.	.	1
34014	0	8.26	1	0.74858	.	.	1
34015	0	8.26	1	0.74858	.	.	1
34016	0	8.26	1	0.74858	.	.	1
34017	0	8.26	1	0.74858	.	.	1
34018	0	8.26	1	0.74858	.	.	1
34019	0	8.26	1	0.74858	.	.	1
34020	0	8.26	1	0.74858	.	.	1

Obs	Sub_group	fuptimey	_CENSOR_	SURVIVAL	SDF_LCL	SDF_UCL	STRATUM
34021	0	8.26	1	0.74858	.	.	1
34022	0	8.26	1	0.74858	.	.	1
34023	0	8.26	1	0.74858	.	.	1
34024	0	8.26	1	0.74858	.	.	1
34025	0	8.26	1	0.74858	.	.	1
34026	0	8.26	1	0.74858	.	.	1
34027	0	8.26	1	0.74858	.	.	1
34028	0	8.26	1	0.74858	.	.	1
34029	0	8.26	1	0.74858	.	.	1
34030	0	8.26	1	0.74858	.	.	1
34031	0	8.26	1	0.74858	.	.	1
34032	0	8.26	1	0.74858	.	.	1
34033	0	8.26	1	0.74858	.	.	1
34034	0	8.26	1	0.74858	.	.	1
34035	0	8.27	0	0.74817	0.74243	0.75381	1
34036	0	8.27	1	0.74817	.	.	1
34037	0	8.27	1	0.74817	.	.	1
34038	0	8.27	1	0.74817	.	.	1
34039	0	8.27	1	0.74817	.	.	1
34040	0	8.27	1	0.74817	.	.	1
34041	0	8.27	1	0.74817	.	.	1
34042	0	8.27	1	0.74817	.	.	1
34043	0	8.27	1	0.74817	.	.	1
34044	0	8.27	1	0.74817	.	.	1
34045	0	8.27	1	0.74817	.	.	1
34046	0	8.27	1	0.74817	.	.	1
34047	0	8.27	1	0.74817	.	.	1
34048	0	8.27	1	0.74817	.	.	1
34049	0	8.27	1	0.74817	.	.	1
34050	0	8.27	1	0.74817	.	.	1
34051	0	8.27	1	0.74817	.	.	1
34052	0	8.27	1	0.74817	.	.	1
34053	0	8.27	1	0.74817	.	.	1
34054	0	8.27	1	0.74817	.	.	1
34055	0	8.27	1	0.74817	.	.	1

Obs	Sub_group	fuptimey	_CENSOR_	SURVIVAL	SDF_LCL	SDF_UCL	STRATUM
34056	0	8.27	1	0.74817	.	.	1
34057	0	8.27	1	0.74817	.	.	1
34058	0	8.27	1	0.74817	.	.	1
34059	0	8.27	1	0.74817	.	.	1
34060	0	8.27	1	0.74817	.	.	1
34061	0	8.27	1	0.74817	.	.	1
34062	0	8.27	1	0.74817	.	.	1
34063	0	8.27	1	0.74817	.	.	1
34064	0	8.27	1	0.74817	.	.	1
34065	0	8.27	1	0.74817	.	.	1
34066	0	8.27	1	0.74817	.	.	1
34067	0	8.27	1	0.74817	.	.	1
34068	0	8.27	1	0.74817	.	.	1
34069	0	8.27	1	0.74817	.	.	1
34070	0	8.28	0	0.74776	0.74200	0.75341	1
34071	0	8.28	1	0.74776	.	.	1
34072	0	8.28	1	0.74776	.	.	1
34073	0	8.28	1	0.74776	.	.	1
34074	0	8.28	1	0.74776	.	.	1
34075	0	8.28	1	0.74776	.	.	1
34076	0	8.28	1	0.74776	.	.	1
34077	0	8.28	1	0.74776	.	.	1
34078	0	8.28	1	0.74776	.	.	1
34079	0	8.28	1	0.74776	.	.	1
34080	0	8.28	1	0.74776	.	.	1
34081	0	8.28	1	0.74776	.	.	1
34082	0	8.28	1	0.74776	.	.	1
34083	0	8.28	1	0.74776	.	.	1
34084	0	8.28	1	0.74776	.	.	1
34085	0	8.28	1	0.74776	.	.	1
34086	0	8.28	1	0.74776	.	.	1
34087	0	8.28	1	0.74776	.	.	1
34088	0	8.28	1	0.74776	.	.	1
34089	0	8.28	1	0.74776	.	.	1
34090	0	8.28	1	0.74776	.	.	1

Obs	Sub_group	fuptimey	_CENSOR_	SURVIVAL	SDF_LCL	SDF_UCL	STRATUM
**34091**	0	8.28	1	0.74776	.	.	1
**34092**	0	8.28	1	0.74776	.	.	1
**34093**	0	8.29	0	0.74762	0.74186	0.75328	1
**34094**	0	8.29	1	0.74762	.	.	1
**34095**	0	8.29	1	0.74762	.	.	1
**34096**	0	8.29	1	0.74762	.	.	1
**34097**	0	8.29	1	0.74762	.	.	1
**34098**	0	8.29	1	0.74762	.	.	1
**34099**	0	8.29	1	0.74762	.	.	1
**34100**	0	8.29	1	0.74762	.	.	1
**34101**	0	8.29	1	0.74762	.	.	1
**34102**	0	8.29	1	0.74762	.	.	1
**34103**	0	8.29	1	0.74762	.	.	1
**34104**	0	8.29	1	0.74762	.	.	1
**34105**	0	8.29	1	0.74762	.	.	1
**34106**	0	8.29	1	0.74762	.	.	1
**34107**	0	8.29	1	0.74762	.	.	1
**34108**	0	8.29	1	0.74762	.	.	1
**34109**	0	8.29	1	0.74762	.	.	1
**34110**	0	8.29	1	0.74762	.	.	1
**34111**	0	8.29	1	0.74762	.	.	1
**34112**	0	8.29	1	0.74762	.	.	1
**34113**	0	8.29	1	0.74762	.	.	1
**34114**	0	8.29	1	0.74762	.	.	1
**34115**	0	8.29	1	0.74762	.	.	1
**34116**	0	8.29	1	0.74762	.	.	1
**34117**	0	8.29	1	0.74762	.	.	1
**34118**	0	8.29	1	0.74762	.	.	1
**34119**	0	8.29	1	0.74762	.	.	1
**34120**	0	8.29	1	0.74762	.	.	1
**34121**	0	8.29	1	0.74762	.	.	1
**34122**	0	8.29	1	0.74762	.	.	1
**34123**	0	8.29	1	0.74762	.	.	1
**34124**	0	8.29	1	0.74762	.	.	1
**34125**	0	8.29	1	0.74762	.	.	1

Obs	Sub_group	fuptimey	_CENSOR_	SURVIVAL	SDF_LCL	SDF_UCL	STRATUM
**34126**	0	8.29	1	0.74762	.	.	1
**34127**	0	8.29	1	0.74762	.	.	1
**34128**	0	8.29	1	0.74762	.	.	1
**34129**	0	8.29	1	0.74762	.	.	1
**34130**	0	8.29	1	0.74762	.	.	1
**34131**	0	8.29	1	0.74762	.	.	1
**34132**	0	8.29	1	0.74762	.	.	1
**34133**	0	8.30	0	0.74720	0.74142	0.75287	1
**34134**	0	8.30	1	0.74720	.	.	1
**34135**	0	8.30	1	0.74720	.	.	1
**34136**	0	8.30	1	0.74720	.	.	1
**34137**	0	8.30	1	0.74720	.	.	1
**34138**	0	8.30	1	0.74720	.	.	1
**34139**	0	8.30	1	0.74720	.	.	1
**34140**	0	8.30	1	0.74720	.	.	1
**34141**	0	8.30	1	0.74720	.	.	1
**34142**	0	8.30	1	0.74720	.	.	1
**34143**	0	8.30	1	0.74720	.	.	1
**34144**	0	8.30	1	0.74720	.	.	1
**34145**	0	8.30	1	0.74720	.	.	1
**34146**	0	8.30	1	0.74720	.	.	1
**34147**	0	8.30	1	0.74720	.	.	1
**34148**	0	8.30	1	0.74720	.	.	1
**34149**	0	8.30	1	0.74720	.	.	1
**34150**	0	8.30	1	0.74720	.	.	1
**34151**	0	8.30	1	0.74720	.	.	1
**34152**	0	8.30	1	0.74720	.	.	1
**34153**	0	8.30	1	0.74720	.	.	1
**34154**	0	8.30	1	0.74720	.	.	1
**34155**	0	8.30	1	0.74720	.	.	1
**34156**	0	8.30	1	0.74720	.	.	1
**34157**	0	8.31	0	0.74692	0.74113	0.75260	1
**34158**	0	8.31	1	0.74692	.	.	1
**34159**	0	8.31	1	0.74692	.	.	1
**34160**	0	8.31	1	0.74692	.	.	1

Obs	Sub_group	fuptimey	_CENSOR_	SURVIVAL	SDF_LCL	SDF_UCL	STRATUM
34161	0	8.31	1	0.74692	.	.	1
34162	0	8.31	1	0.74692	.	.	1
34163	0	8.31	1	0.74692	.	.	1
34164	0	8.31	1	0.74692	.	.	1
34165	0	8.31	1	0.74692	.	.	1
34166	0	8.31	1	0.74692	.	.	1
34167	0	8.31	1	0.74692	.	.	1
34168	0	8.31	1	0.74692	.	.	1
34169	0	8.31	1	0.74692	.	.	1
34170	0	8.31	1	0.74692	.	.	1
34171	0	8.31	1	0.74692	.	.	1
34172	0	8.31	1	0.74692	.	.	1
34173	0	8.31	1	0.74692	.	.	1
34174	0	8.31	1	0.74692	.	.	1
34175	0	8.31	1	0.74692	.	.	1
34176	0	8.31	1	0.74692	.	.	1
34177	0	8.31	1	0.74692	.	.	1
34178	0	8.31	1	0.74692	.	.	1
34179	0	8.31	1	0.74692	.	.	1
34180	0	8.31	1	0.74692	.	.	1
34181	0	8.31	1	0.74692	.	.	1
34182	0	8.31	1	0.74692	.	.	1
34183	0	8.31	1	0.74692	.	.	1
34184	0	8.31	1	0.74692	.	.	1
34185	0	8.31	1	0.74692	.	.	1
34186	0	8.31	1	0.74692	.	.	1
34187	0	8.31	1	0.74692	.	.	1
34188	0	8.31	1	0.74692	.	.	1
34189	0	8.31	1	0.74692	.	.	1
34190	0	8.31	1	0.74692	.	.	1
34191	0	8.31	1	0.74692	.	.	1
34192	0	8.31	1	0.74692	.	.	1
34193	0	8.31	1	0.74692	.	.	1
34194	0	8.31	1	0.74692	.	.	1
34195	0	8.32	1	0.74692	.	.	1

Obs	Sub_group	fuptimey	_CENSOR_	SURVIVAL	SDF_LCL	SDF_UCL	STRATUM
34196	0	8.32	1	0.74692	.	.	1
34197	0	8.32	1	0.74692	.	.	1
34198	0	8.32	1	0.74692	.	.	1
34199	0	8.32	1	0.74692	.	.	1
34200	0	8.32	1	0.74692	.	.	1
34201	0	8.32	1	0.74692	.	.	1
34202	0	8.32	1	0.74692	.	.	1
34203	0	8.32	1	0.74692	.	.	1
34204	0	8.32	1	0.74692	.	.	1
34205	0	8.32	1	0.74692	.	.	1
34206	0	8.32	1	0.74692	.	.	1
34207	0	8.32	1	0.74692	.	.	1
34208	0	8.32	1	0.74692	.	.	1
34209	0	8.32	1	0.74692	.	.	1
34210	0	8.32	1	0.74692	.	.	1
34211	0	8.32	1	0.74692	.	.	1
34212	0	8.32	1	0.74692	.	.	1
34213	0	8.32	1	0.74692	.	.	1
34214	0	8.32	1	0.74692	.	.	1
34215	0	8.32	1	0.74692	.	.	1
34216	0	8.32	1	0.74692	.	.	1
34217	0	8.32	1	0.74692	.	.	1
34218	0	8.32	1	0.74692	.	.	1
34219	0	8.32	1	0.74692	.	.	1
34220	0	8.32	1	0.74692	.	.	1
34221	0	8.32	1	0.74692	.	.	1
34222	0	8.33	1	0.74692	.	.	1
34223	0	8.33	1	0.74692	.	.	1
34224	0	8.33	1	0.74692	.	.	1
34225	0	8.33	1	0.74692	.	.	1
34226	0	8.33	1	0.74692	.	.	1
34227	0	8.33	1	0.74692	.	.	1
34228	0	8.33	1	0.74692	.	.	1
34229	0	8.33	1	0.74692	.	.	1
34230	0	8.33	1	0.74692	.	.	1

Obs	Sub_group	fuptimey	_CENSOR_	SURVIVAL	SDF_LCL	SDF_UCL	STRATUM
34231	0	8.33	1	0.74692	.	.	1
34232	0	8.33	1	0.74692	.	.	1
34233	0	8.33	1	0.74692	.	.	1
34234	0	8.33	1	0.74692	.	.	1
34235	0	8.33	1	0.74692	.	.	1
34236	0	8.33	1	0.74692	.	.	1
34237	0	8.33	1	0.74692	.	.	1
34238	0	8.33	1	0.74692	.	.	1
34239	0	8.33	1	0.74692	.	.	1
34240	0	8.33	1	0.74692	.	.	1
34241	0	8.33	1	0.74692	.	.	1
34242	0	8.33	1	0.74692	.	.	1
34243	0	8.33	1	0.74692	.	.	1
34244	0	8.33	1	0.74692	.	.	1
34245	0	8.34	0	0.74649	0.74069	0.75219	1
34246	0	8.34	1	0.74649	.	.	1
34247	0	8.34	1	0.74649	.	.	1
34248	0	8.34	1	0.74649	.	.	1
34249	0	8.34	1	0.74649	.	.	1
34250	0	8.34	1	0.74649	.	.	1
34251	0	8.34	1	0.74649	.	.	1
34252	0	8.34	1	0.74649	.	.	1
34253	0	8.34	1	0.74649	.	.	1
34254	0	8.34	1	0.74649	.	.	1
34255	0	8.34	1	0.74649	.	.	1
34256	0	8.34	1	0.74649	.	.	1
34257	0	8.34	1	0.74649	.	.	1
34258	0	8.34	1	0.74649	.	.	1
34259	0	8.34	1	0.74649	.	.	1
34260	0	8.34	1	0.74649	.	.	1
34261	0	8.34	1	0.74649	.	.	1
34262	0	8.34	1	0.74649	.	.	1
34263	0	8.34	1	0.74649	.	.	1
34264	0	8.34	1	0.74649	.	.	1
34265	0	8.34	1	0.74649	.	.	1

Obs	Sub_group	fuptimey	_CENSOR_	SURVIVAL	SDF_LCL	SDF_UCL	STRATUM
**34266**	0	8.34	1	0.74649	.	.	1
**34267**	0	8.34	1	0.74649	.	.	1
**34268**	0	8.35	0	0.74621	0.74039	0.75192	1
**34269**	0	8.35	1	0.74621	.	.	1
**34270**	0	8.35	1	0.74621	.	.	1
**34271**	0	8.35	1	0.74621	.	.	1
**34272**	0	8.35	1	0.74621	.	.	1
**34273**	0	8.35	1	0.74621	.	.	1
**34274**	0	8.35	1	0.74621	.	.	1
**34275**	0	8.35	1	0.74621	.	.	1
**34276**	0	8.35	1	0.74621	.	.	1
**34277**	0	8.35	1	0.74621	.	.	1
**34278**	0	8.35	1	0.74621	.	.	1
**34279**	0	8.35	1	0.74621	.	.	1
**34280**	0	8.35	1	0.74621	.	.	1
**34281**	0	8.35	1	0.74621	.	.	1
**34282**	0	8.35	1	0.74621	.	.	1
**34283**	0	8.35	1	0.74621	.	.	1
**34284**	0	8.35	1	0.74621	.	.	1
**34285**	0	8.35	1	0.74621	.	.	1
**34286**	0	8.35	1	0.74621	.	.	1
**34287**	0	8.35	1	0.74621	.	.	1
**34288**	0	8.35	1	0.74621	.	.	1
**34289**	0	8.35	1	0.74621	.	.	1
**34290**	0	8.35	1	0.74621	.	.	1
**34291**	0	8.35	1	0.74621	.	.	1
**34292**	0	8.35	1	0.74621	.	.	1
**34293**	0	8.35	1	0.74621	.	.	1
**34294**	0	8.35	1	0.74621	.	.	1
**34295**	0	8.35	1	0.74621	.	.	1
**34296**	0	8.36	1	0.74621	.	.	1
**34297**	0	8.36	1	0.74621	.	.	1
**34298**	0	8.36	1	0.74621	.	.	1
**34299**	0	8.36	1	0.74621	.	.	1
**34300**	0	8.36	1	0.74621	.	.	1

Obs	Sub_group	fuptimey	_CENSOR_	SURVIVAL	SDF_LCL	SDF_UCL	STRATUM
34301	0	8.36	1	0.74621	.	.	1
34302	0	8.36	1	0.74621	.	.	1
34303	0	8.36	1	0.74621	.	.	1
34304	0	8.36	1	0.74621	.	.	1
34305	0	8.36	1	0.74621	.	.	1
34306	0	8.36	1	0.74621	.	.	1
34307	0	8.36	1	0.74621	.	.	1
34308	0	8.36	1	0.74621	.	.	1
34309	0	8.36	1	0.74621	.	.	1
34310	0	8.36	1	0.74621	.	.	1
34311	0	8.36	1	0.74621	.	.	1
34312	0	8.36	1	0.74621	.	.	1
34313	0	8.36	1	0.74621	.	.	1
34314	0	8.36	1	0.74621	.	.	1
34315	0	8.36	1	0.74621	.	.	1
34316	0	8.36	1	0.74621	.	.	1
34317	0	8.36	1	0.74621	.	.	1
34318	0	8.37	0	0.74577	0.73994	0.75150	1
34319	0	8.37	1	0.74577	.	.	1
34320	0	8.37	1	0.74577	.	.	1
34321	0	8.37	1	0.74577	.	.	1
34322	0	8.37	1	0.74577	.	.	1
34323	0	8.37	1	0.74577	.	.	1
34324	0	8.37	1	0.74577	.	.	1
34325	0	8.37	1	0.74577	.	.	1
34326	0	8.37	1	0.74577	.	.	1
34327	0	8.37	1	0.74577	.	.	1
34328	0	8.37	1	0.74577	.	.	1
34329	0	8.37	1	0.74577	.	.	1
34330	0	8.37	1	0.74577	.	.	1
34331	0	8.37	1	0.74577	.	.	1
34332	0	8.37	1	0.74577	.	.	1
34333	0	8.37	1	0.74577	.	.	1
34334	0	8.37	1	0.74577	.	.	1
34335	0	8.37	1	0.74577	.	.	1

Obs	Sub_group	fuptimey	_CENSOR_	SURVIVAL	SDF_LCL	SDF_UCL	STRATUM
34336	0	8.37	1	0.74577	.	.	1
34337	0	8.37	1	0.74577	.	.	1
34338	0	8.37	1	0.74577	.	.	1
34339	0	8.37	1	0.74577	.	.	1
34340	0	8.37	1	0.74577	.	.	1
34341	0	8.37	1	0.74577	.	.	1
34342	0	8.37	1	0.74577	.	.	1
34343	0	8.37	1	0.74577	.	.	1
34344	0	8.37	1	0.74577	.	.	1
34345	0	8.37	1	0.74577	.	.	1
34346	0	8.37	1	0.74577	.	.	1
34347	0	8.37	1	0.74577	.	.	1
34348	0	8.37	1	0.74577	.	.	1
34349	0	8.37	1	0.74577	.	.	1
34350	0	8.37	1	0.74577	.	.	1
34351	0	8.37	1	0.74577	.	.	1
34352	0	8.37	1	0.74577	.	.	1
34353	0	8.37	1	0.74577	.	.	1
34354	0	8.37	1	0.74577	.	.	1
34355	0	8.38	0	0.74563	0.73979	0.75136	1
34356	0	8.38	1	0.74563	.	.	1
34357	0	8.38	1	0.74563	.	.	1
34358	0	8.38	1	0.74563	.	.	1
34359	0	8.38	1	0.74563	.	.	1
34360	0	8.38	1	0.74563	.	.	1
34361	0	8.38	1	0.74563	.	.	1
34362	0	8.38	1	0.74563	.	.	1
34363	0	8.38	1	0.74563	.	.	1
34364	0	8.38	1	0.74563	.	.	1
34365	0	8.38	1	0.74563	.	.	1
34366	0	8.38	1	0.74563	.	.	1
34367	0	8.38	1	0.74563	.	.	1
34368	0	8.38	1	0.74563	.	.	1
34369	0	8.38	1	0.74563	.	.	1
34370	0	8.38	1	0.74563	.	.	1

Obs	Sub_group	fuptimey	_CENSOR_	SURVIVAL	SDF_LCL	SDF_UCL	STRATUM
34371	0	8.38	1	0.74563	.	.	1
34372	0	8.38	1	0.74563	.	.	1
34373	0	8.38	1	0.74563	.	.	1
34374	0	8.38	1	0.74563	.	.	1
34375	0	8.38	1	0.74563	.	.	1
34376	0	8.38	1	0.74563	.	.	1
34377	0	8.38	1	0.74563	.	.	1
34378	0	8.38	1	0.74563	.	.	1
34379	0	8.38	1	0.74563	.	.	1
34380	0	8.38	1	0.74563	.	.	1
34381	0	8.38	1	0.74563	.	.	1
34382	0	8.38	1	0.74563	.	.	1
34383	0	8.38	1	0.74563	.	.	1
34384	0	8.38	1	0.74563	.	.	1
34385	0	8.38	1	0.74563	.	.	1
34386	0	8.38	1	0.74563	.	.	1
34387	0	8.38	1	0.74563	.	.	1
34388	0	8.38	1	0.74563	.	.	1
34389	0	8.38	1	0.74563	.	.	1
34390	0	8.38	1	0.74563	.	.	1
34391	0	8.38	1	0.74563	.	.	1
34392	0	8.38	1	0.74563	.	.	1
34393	0	8.39	0	0.74548	0.73963	0.75122	1
34394	0	8.39	1	0.74548	.	.	1
34395	0	8.39	1	0.74548	.	.	1
34396	0	8.39	1	0.74548	.	.	1
34397	0	8.39	1	0.74548	.	.	1
34398	0	8.39	1	0.74548	.	.	1
34399	0	8.39	1	0.74548	.	.	1
34400	0	8.39	1	0.74548	.	.	1
34401	0	8.39	1	0.74548	.	.	1
34402	0	8.39	1	0.74548	.	.	1
34403	0	8.39	1	0.74548	.	.	1
34404	0	8.39	1	0.74548	.	.	1
34405	0	8.39	1	0.74548	.	.	1

Obs	Sub_group	fuptimey	_CENSOR_	SURVIVAL	SDF_LCL	SDF_UCL	STRATUM
34406	0	8.39	1	0.74548	.	.	1
34407	0	8.39	1	0.74548	.	.	1
34408	0	8.39	1	0.74548	.	.	1
34409	0	8.39	1	0.74548	.	.	1
34410	0	8.39	1	0.74548	.	.	1
34411	0	8.39	1	0.74548	.	.	1
34412	0	8.39	1	0.74548	.	.	1
34413	0	8.39	1	0.74548	.	.	1
34414	0	8.39	1	0.74548	.	.	1
34415	0	8.39	1	0.74548	.	.	1
34416	0	8.39	1	0.74548	.	.	1
34417	0	8.40	0	0.74504	0.73917	0.75080	1
34418	0	8.40	1	0.74504	.	.	1
34419	0	8.40	1	0.74504	.	.	1
34420	0	8.40	1	0.74504	.	.	1
34421	0	8.40	1	0.74504	.	.	1
34422	0	8.40	1	0.74504	.	.	1
34423	0	8.40	1	0.74504	.	.	1
34424	0	8.40	1	0.74504	.	.	1
34425	0	8.40	1	0.74504	.	.	1
34426	0	8.40	1	0.74504	.	.	1
34427	0	8.40	1	0.74504	.	.	1
34428	0	8.40	1	0.74504	.	.	1
34429	0	8.40	1	0.74504	.	.	1
34430	0	8.40	1	0.74504	.	.	1
34431	0	8.40	1	0.74504	.	.	1
34432	0	8.40	1	0.74504	.	.	1
34433	0	8.40	1	0.74504	.	.	1
34434	0	8.40	1	0.74504	.	.	1
34435	0	8.40	1	0.74504	.	.	1
34436	0	8.40	1	0.74504	.	.	1
34437	0	8.40	1	0.74504	.	.	1
34438	0	8.40	1	0.74504	.	.	1
34439	0	8.40	1	0.74504	.	.	1
34440	0	8.40	1	0.74504	.	.	1

Obs	Sub_group	fuptimey	_CENSOR_	SURVIVAL	SDF_LCL	SDF_UCL	STRATUM
**34441**	0	8.40	1	0.74504	.	.	1
**34442**	0	8.40	1	0.74504	.	.	1
**34443**	0	8.40	1	0.74504	.	.	1
**34444**	0	8.40	1	0.74504	.	.	1
**34445**	0	8.40	1	0.74504	.	.	1
**34446**	0	8.40	1	0.74504	.	.	1
**34447**	0	8.40	1	0.74504	.	.	1
**34448**	0	8.40	1	0.74504	.	.	1
**34449**	0	8.40	1	0.74504	.	.	1
**34450**	0	8.40	1	0.74504	.	.	1
**34451**	0	8.40	1	0.74504	.	.	1
**34452**	0	8.40	1	0.74504	.	.	1
**34453**	0	8.40	1	0.74504	.	.	1
**34454**	0	8.41	0	0.74460	0.73871	0.75037	1
**34455**	0	8.41	1	0.74460	.	.	1
**34456**	0	8.41	1	0.74460	.	.	1
**34457**	0	8.41	1	0.74460	.	.	1
**34458**	0	8.41	1	0.74460	.	.	1
**34459**	0	8.41	1	0.74460	.	.	1
**34460**	0	8.41	1	0.74460	.	.	1
**34461**	0	8.41	1	0.74460	.	.	1
**34462**	0	8.41	1	0.74460	.	.	1
**34463**	0	8.41	1	0.74460	.	.	1
**34464**	0	8.41	1	0.74460	.	.	1
**34465**	0	8.41	1	0.74460	.	.	1
**34466**	0	8.41	1	0.74460	.	.	1
**34467**	0	8.41	1	0.74460	.	.	1
**34468**	0	8.41	1	0.74460	.	.	1
**34469**	0	8.41	1	0.74460	.	.	1
**34470**	0	8.41	1	0.74460	.	.	1
**34471**	0	8.41	1	0.74460	.	.	1
**34472**	0	8.41	1	0.74460	.	.	1
**34473**	0	8.41	1	0.74460	.	.	1
**34474**	0	8.41	1	0.74460	.	.	1
**34475**	0	8.41	1	0.74460	.	.	1

Obs	Sub_group	fuptimey	_CENSOR_	SURVIVAL	SDF_LCL	SDF_UCL	STRATUM
**34476**	0	8.41	1	0.74460	.	.	1
**34477**	0	8.41	1	0.74460	.	.	1
**34478**	0	8.41	1	0.74460	.	.	1
**34479**	0	8.41	1	0.74460	.	.	1
**34480**	0	8.41	1	0.74460	.	.	1
**34481**	0	8.42	1	0.74460	.	.	1
**34482**	0	8.42	1	0.74460	.	.	1
**34483**	0	8.42	1	0.74460	.	.	1
**34484**	0	8.42	1	0.74460	.	.	1
**34485**	0	8.42	1	0.74460	.	.	1
**34486**	0	8.42	1	0.74460	.	.	1
**34487**	0	8.42	1	0.74460	.	.	1
**34488**	0	8.42	1	0.74460	.	.	1
**34489**	0	8.42	1	0.74460	.	.	1
**34490**	0	8.42	1	0.74460	.	.	1
**34491**	0	8.42	1	0.74460	.	.	1
**34492**	0	8.42	1	0.74460	.	.	1
**34493**	0	8.42	1	0.74460	.	.	1
**34494**	0	8.42	1	0.74460	.	.	1
**34495**	0	8.42	1	0.74460	.	.	1
**34496**	0	8.42	1	0.74460	.	.	1
**34497**	0	8.42	1	0.74460	.	.	1
**34498**	0	8.42	1	0.74460	.	.	1
**34499**	0	8.42	1	0.74460	.	.	1
**34500**	0	8.42	1	0.74460	.	.	1
**34501**	0	8.42	1	0.74460	.	.	1
**34502**	0	8.42	1	0.74460	.	.	1
**34503**	0	8.42	1	0.74460	.	.	1
**34504**	0	8.42	1	0.74460	.	.	1
**34505**	0	8.42	1	0.74460	.	.	1
**34506**	0	8.42	1	0.74460	.	.	1
**34507**	0	8.42	1	0.74460	.	.	1
**34508**	0	8.43	1	0.74460	.	.	1
**34509**	0	8.43	1	0.74460	.	.	1
**34510**	0	8.43	1	0.74460	.	.	1

Obs	Sub_group	fuptimey	_CENSOR_	SURVIVAL	SDF_LCL	SDF_UCL	STRATUM
34511	0	8.43	1	0.74460	.	.	1
34512	0	8.43	1	0.74460	.	.	1
34513	0	8.43	1	0.74460	.	.	1
34514	0	8.43	1	0.74460	.	.	1
34515	0	8.43	1	0.74460	.	.	1
34516	0	8.43	1	0.74460	.	.	1
34517	0	8.43	1	0.74460	.	.	1
34518	0	8.43	1	0.74460	.	.	1
34519	0	8.43	1	0.74460	.	.	1
34520	0	8.43	1	0.74460	.	.	1
34521	0	8.43	1	0.74460	.	.	1
34522	0	8.43	1	0.74460	.	.	1
34523	0	8.43	1	0.74460	.	.	1
34524	0	8.43	1	0.74460	.	.	1
34525	0	8.44	1	0.74460	.	.	1
34526	0	8.44	1	0.74460	.	.	1
34527	0	8.44	1	0.74460	.	.	1
34528	0	8.44	1	0.74460	.	.	1
34529	0	8.44	1	0.74460	.	.	1
34530	0	8.44	1	0.74460	.	.	1
34531	0	8.44	1	0.74460	.	.	1
34532	0	8.44	1	0.74460	.	.	1
34533	0	8.44	1	0.74460	.	.	1
34534	0	8.44	1	0.74460	.	.	1
34535	0	8.44	1	0.74460	.	.	1
34536	0	8.44	1	0.74460	.	.	1
34537	0	8.44	1	0.74460	.	.	1
34538	0	8.44	1	0.74460	.	.	1
34539	0	8.44	1	0.74460	.	.	1
34540	0	8.44	1	0.74460	.	.	1
34541	0	8.44	1	0.74460	.	.	1
34542	0	8.44	1	0.74460	.	.	1
34543	0	8.44	1	0.74460	.	.	1
34544	0	8.44	1	0.74460	.	.	1
34545	0	8.44	1	0.74460	.	.	1

Obs	Sub_group	fuptimey	_CENSOR_	SURVIVAL	SDF_LCL	SDF_UCL	STRATUM
34546	0	8.44	1	0.74460	.	.	1
34547	0	8.44	1	0.74460	.	.	1
34548	0	8.44	1	0.74460	.	.	1
34549	0	8.44	1	0.74460	.	.	1
34550	0	8.44	1	0.74460	.	.	1
34551	0	8.44	1	0.74460	.	.	1
34552	0	8.44	1	0.74460	.	.	1
34553	0	8.45	0	0.74399	0.73808	0.74979	1
34554	0	8.45	1	0.74399	.	.	1
34555	0	8.45	1	0.74399	.	.	1
34556	0	8.45	1	0.74399	.	.	1
34557	0	8.45	1	0.74399	.	.	1
34558	0	8.45	1	0.74399	.	.	1
34559	0	8.45	1	0.74399	.	.	1
34560	0	8.45	1	0.74399	.	.	1
34561	0	8.45	1	0.74399	.	.	1
34562	0	8.45	1	0.74399	.	.	1
34563	0	8.45	1	0.74399	.	.	1
34564	0	8.45	1	0.74399	.	.	1
34565	0	8.45	1	0.74399	.	.	1
34566	0	8.45	1	0.74399	.	.	1
34567	0	8.45	1	0.74399	.	.	1
34568	0	8.45	1	0.74399	.	.	1
34569	0	8.45	1	0.74399	.	.	1
34570	0	8.45	1	0.74399	.	.	1
34571	0	8.45	1	0.74399	.	.	1
34572	0	8.45	1	0.74399	.	.	1
34573	0	8.45	1	0.74399	.	.	1
34574	0	8.45	1	0.74399	.	.	1
34575	0	8.45	1	0.74399	.	.	1
34576	0	8.45	1	0.74399	.	.	1
34577	0	8.45	1	0.74399	.	.	1
34578	0	8.45	1	0.74399	.	.	1
34579	0	8.45	1	0.74399	.	.	1
34580	0	8.45	1	0.74399	.	.	1

Obs	Sub_group	fuptimey	_CENSOR_	SURVIVAL	SDF_LCL	SDF_UCL	STRATUM
34581	0	8.45	1	0.74399	.	.	1
34582	0	8.45	1	0.74399	.	.	1
34583	0	8.46	1	0.74399	.	.	1
34584	0	8.46	1	0.74399	.	.	1
34585	0	8.46	1	0.74399	.	.	1
34586	0	8.46	1	0.74399	.	.	1
34587	0	8.46	1	0.74399	.	.	1
34588	0	8.46	1	0.74399	.	.	1
34589	0	8.46	1	0.74399	.	.	1
34590	0	8.46	1	0.74399	.	.	1
34591	0	8.46	1	0.74399	.	.	1
34592	0	8.46	1	0.74399	.	.	1
34593	0	8.46	1	0.74399	.	.	1
34594	0	8.46	1	0.74399	.	.	1
34595	0	8.46	1	0.74399	.	.	1
34596	0	8.46	1	0.74399	.	.	1
34597	0	8.46	1	0.74399	.	.	1
34598	0	8.46	1	0.74399	.	.	1
34599	0	8.46	1	0.74399	.	.	1
34600	0	8.46	1	0.74399	.	.	1
34601	0	8.46	1	0.74399	.	.	1
34602	0	8.46	1	0.74399	.	.	1
34603	0	8.46	1	0.74399	.	.	1
34604	0	8.46	1	0.74399	.	.	1
34605	0	8.46	1	0.74399	.	.	1
34606	0	8.46	1	0.74399	.	.	1
34607	0	8.46	1	0.74399	.	.	1
34608	0	8.46	1	0.74399	.	.	1
34609	0	8.46	1	0.74399	.	.	1
34610	0	8.46	1	0.74399	.	.	1
34611	0	8.46	1	0.74399	.	.	1
34612	0	8.47	0	0.74369	0.73776	0.74950	1
34613	0	8.47	1	0.74369	.	.	1
34614	0	8.47	1	0.74369	.	.	1
34615	0	8.47	1	0.74369	.	.	1

Obs	Sub_group	fuptimey	_CENSOR_	SURVIVAL	SDF_LCL	SDF_UCL	STRATUM
34616	0	8.47	1	0.74369	.	.	1
34617	0	8.47	1	0.74369	.	.	1
34618	0	8.47	1	0.74369	.	.	1
34619	0	8.47	1	0.74369	.	.	1
34620	0	8.47	1	0.74369	.	.	1
34621	0	8.47	1	0.74369	.	.	1
34622	0	8.47	1	0.74369	.	.	1
34623	0	8.47	1	0.74369	.	.	1
34624	0	8.47	1	0.74369	.	.	1
34625	0	8.47	1	0.74369	.	.	1
34626	0	8.47	1	0.74369	.	.	1
34627	0	8.47	1	0.74369	.	.	1
34628	0	8.47	1	0.74369	.	.	1
34629	0	8.47	1	0.74369	.	.	1
34630	0	8.47	1	0.74369	.	.	1
34631	0	8.47	1	0.74369	.	.	1
34632	0	8.47	1	0.74369	.	.	1
34633	0	8.47	1	0.74369	.	.	1
34634	0	8.47	1	0.74369	.	.	1
34635	0	8.47	1	0.74369	.	.	1
34636	0	8.48	0	0.74353	0.73760	0.74935	1
34637	0	8.48	1	0.74353	.	.	1
34638	0	8.48	1	0.74353	.	.	1
34639	0	8.48	1	0.74353	.	.	1
34640	0	8.48	1	0.74353	.	.	1
34641	0	8.48	1	0.74353	.	.	1
34642	0	8.48	1	0.74353	.	.	1
34643	0	8.48	1	0.74353	.	.	1
34644	0	8.48	1	0.74353	.	.	1
34645	0	8.48	1	0.74353	.	.	1
34646	0	8.48	1	0.74353	.	.	1
34647	0	8.48	1	0.74353	.	.	1
34648	0	8.48	1	0.74353	.	.	1
34649	0	8.48	1	0.74353	.	.	1
34650	0	8.48	1	0.74353	.	.	1

Obs	Sub_group	fuptimey	_CENSOR_	SURVIVAL	SDF_LCL	SDF_UCL	STRATUM
34651	0	8.48	1	0.74353	.	.	1
34652	0	8.48	1	0.74353	.	.	1
34653	0	8.48	1	0.74353	.	.	1
34654	0	8.48	1	0.74353	.	.	1
34655	0	8.48	1	0.74353	.	.	1
34656	0	8.48	1	0.74353	.	.	1
34657	0	8.48	1	0.74353	.	.	1
34658	0	8.48	1	0.74353	.	.	1
34659	0	8.48	1	0.74353	.	.	1
34660	0	8.48	1	0.74353	.	.	1
34661	0	8.48	1	0.74353	.	.	1
34662	0	8.48	1	0.74353	.	.	1
34663	0	8.48	1	0.74353	.	.	1
34664	0	8.48	1	0.74353	.	.	1
34665	0	8.48	1	0.74353	.	.	1
34666	0	8.48	1	0.74353	.	.	1
34667	0	8.48	1	0.74353	.	.	1
34668	0	8.48	1	0.74353	.	.	1
34669	0	8.48	1	0.74353	.	.	1
34670	0	8.48	1	0.74353	.	.	1
34671	0	8.48	1	0.74353	.	.	1
34672	0	8.48	1	0.74353	.	.	1
34673	0	8.48	1	0.74353	.	.	1
34674	0	8.48	1	0.74353	.	.	1
34675	0	8.48	1	0.74353	.	.	1
34676	0	8.49	1	0.74353	.	.	1
34677	0	8.49	1	0.74353	.	.	1
34678	0	8.49	1	0.74353	.	.	1
34679	0	8.49	1	0.74353	.	.	1
34680	0	8.49	1	0.74353	.	.	1
34681	0	8.49	1	0.74353	.	.	1
34682	0	8.49	1	0.74353	.	.	1
34683	0	8.49	1	0.74353	.	.	1
34684	0	8.49	1	0.74353	.	.	1
34685	0	8.49	1	0.74353	.	.	1

Obs	Sub_group	fuptimey	_CENSOR_	SURVIVAL	SDF_LCL	SDF_UCL	STRATUM
34686	0	8.49	1	0.74353	.	.	1
34687	0	8.49	1	0.74353	.	.	1
34688	0	8.49	1	0.74353	.	.	1
34689	0	8.49	1	0.74353	.	.	1
34690	0	8.49	1	0.74353	.	.	1
34691	0	8.49	1	0.74353	.	.	1
34692	0	8.49	1	0.74353	.	.	1
34693	0	8.49	1	0.74353	.	.	1
34694	0	8.49	1	0.74353	.	.	1
34695	0	8.49	1	0.74353	.	.	1
34696	0	8.49	1	0.74353	.	.	1
34697	0	8.49	1	0.74353	.	.	1
34698	0	8.49	1	0.74353	.	.	1
34699	0	8.49	1	0.74353	.	.	1
34700	0	8.49	1	0.74353	.	.	1
34701	0	8.49	1	0.74353	.	.	1
34702	0	8.49	1	0.74353	.	.	1
34703	0	8.49	1	0.74353	.	.	1
34704	0	8.50	0	0.74322	0.73728	0.74905	1
34705	0	8.50	1	0.74322	.	.	1
34706	0	8.50	1	0.74322	.	.	1
34707	0	8.50	1	0.74322	.	.	1
34708	0	8.50	1	0.74322	.	.	1
34709	0	8.50	1	0.74322	.	.	1
34710	0	8.50	1	0.74322	.	.	1
34711	0	8.50	1	0.74322	.	.	1
34712	0	8.50	1	0.74322	.	.	1
34713	0	8.50	1	0.74322	.	.	1
34714	0	8.50	1	0.74322	.	.	1
34715	0	8.50	1	0.74322	.	.	1
34716	0	8.50	1	0.74322	.	.	1
34717	0	8.50	1	0.74322	.	.	1
34718	0	8.50	1	0.74322	.	.	1
34719	0	8.50	1	0.74322	.	.	1
34720	0	8.50	1	0.74322	.	.	1

Obs	Sub_group	fuptimey	_CENSOR_	SURVIVAL	SDF_LCL	SDF_UCL	STRATUM
**34721**	0	8.50	1	0.74322	.	.	1
**34722**	0	8.50	1	0.74322	.	.	1
**34723**	0	8.50	1	0.74322	.	.	1
**34724**	0	8.50	1	0.74322	.	.	1
**34725**	0	8.50	1	0.74322	.	.	1
**34726**	0	8.50	1	0.74322	.	.	1
**34727**	0	8.50	1	0.74322	.	.	1
**34728**	0	8.50	1	0.74322	.	.	1
**34729**	0	8.50	1	0.74322	.	.	1
**34730**	0	8.50	1	0.74322	.	.	1
**34731**	0	8.50	1	0.74322	.	.	1
**34732**	0	8.50	1	0.74322	.	.	1
**34733**	0	8.50	1	0.74322	.	.	1
**34734**	0	8.50	1	0.74322	.	.	1
**34735**	0	8.50	1	0.74322	.	.	1
**34736**	0	8.50	1	0.74322	.	.	1
**34737**	0	8.50	1	0.74322	.	.	1
**34738**	0	8.50	1	0.74322	.	.	1
**34739**	0	8.50	1	0.74322	.	.	1
**34740**	0	8.50	1	0.74322	.	.	1
**34741**	0	8.50	1	0.74322	.	.	1
**34742**	0	8.51	0	0.74291	0.73695	0.74875	1
**34743**	0	8.51	1	0.74291	.	.	1
**34744**	0	8.51	1	0.74291	.	.	1
**34745**	0	8.51	1	0.74291	.	.	1
**34746**	0	8.51	1	0.74291	.	.	1
**34747**	0	8.51	1	0.74291	.	.	1
**34748**	0	8.51	1	0.74291	.	.	1
**34749**	0	8.51	1	0.74291	.	.	1
**34750**	0	8.51	1	0.74291	.	.	1
**34751**	0	8.51	1	0.74291	.	.	1
**34752**	0	8.51	1	0.74291	.	.	1
**34753**	0	8.51	1	0.74291	.	.	1
**34754**	0	8.51	1	0.74291	.	.	1
**34755**	0	8.51	1	0.74291	.	.	1

Obs	Sub_group	fuptimey	_CENSOR_	SURVIVAL	SDF_LCL	SDF_UCL	STRATUM
34756	0	8.51	1	0.74291	.	.	1
34757	0	8.51	1	0.74291	.	.	1
34758	0	8.51	1	0.74291	.	.	1
34759	0	8.51	1	0.74291	.	.	1
34760	0	8.51	1	0.74291	.	.	1
34761	0	8.51	1	0.74291	.	.	1
34762	0	8.51	1	0.74291	.	.	1
34763	0	8.51	1	0.74291	.	.	1
34764	0	8.51	1	0.74291	.	.	1
34765	0	8.51	1	0.74291	.	.	1
34766	0	8.51	1	0.74291	.	.	1
34767	0	8.51	1	0.74291	.	.	1
34768	0	8.51	1	0.74291	.	.	1
34769	0	8.51	1	0.74291	.	.	1
34770	0	8.52	1	0.74291	.	.	1
34771	0	8.52	1	0.74291	.	.	1
34772	0	8.52	1	0.74291	.	.	1
34773	0	8.52	1	0.74291	.	.	1
34774	0	8.52	1	0.74291	.	.	1
34775	0	8.52	1	0.74291	.	.	1
34776	0	8.52	1	0.74291	.	.	1
34777	0	8.52	1	0.74291	.	.	1
34778	0	8.52	1	0.74291	.	.	1
34779	0	8.52	1	0.74291	.	.	1
34780	0	8.52	1	0.74291	.	.	1
34781	0	8.52	1	0.74291	.	.	1
34782	0	8.52	1	0.74291	.	.	1
34783	0	8.52	1	0.74291	.	.	1
34784	0	8.52	1	0.74291	.	.	1
34785	0	8.52	1	0.74291	.	.	1
34786	0	8.52	1	0.74291	.	.	1
34787	0	8.52	1	0.74291	.	.	1
34788	0	8.52	1	0.74291	.	.	1
34789	0	8.52	1	0.74291	.	.	1
34790	0	8.52	1	0.74291	.	.	1

Obs	Sub_group	fuptimey	_CENSOR_	SURVIVAL	SDF_LCL	SDF_UCL	STRATUM
**34791**	0	8.52	1	0.74291	.	.	1
**34792**	0	8.52	1	0.74291	.	.	1
**34793**	0	8.52	1	0.74291	.	.	1
**34794**	0	8.52	1	0.74291	.	.	1
**34795**	0	8.52	1	0.74291	.	.	1
**34796**	0	8.52	1	0.74291	.	.	1
**34797**	0	8.52	1	0.74291	.	.	1
**34798**	0	8.52	1	0.74291	.	.	1
**34799**	0	8.52	1	0.74291	.	.	1
**34800**	0	8.52	1	0.74291	.	.	1
**34801**	0	8.52	1	0.74291	.	.	1
**34802**	0	8.52	1	0.74291	.	.	1
**34803**	0	8.52	1	0.74291	.	.	1
**34804**	0	8.52	1	0.74291	.	.	1
**34805**	0	8.52	1	0.74291	.	.	1
**34806**	0	8.52	1	0.74291	.	.	1
**34807**	0	8.52	1	0.74291	.	.	1
**34808**	0	8.52	1	0.74291	.	.	1
**34809**	0	8.52	1	0.74291	.	.	1
**34810**	0	8.52	1	0.74291	.	.	1
**34811**	0	8.52	1	0.74291	.	.	1
**34812**	0	8.52	1	0.74291	.	.	1
**34813**	0	8.53	0	0.74275	0.73678	0.74860	1
**34814**	0	8.53	1	0.74275	.	.	1
**34815**	0	8.53	1	0.74275	.	.	1
**34816**	0	8.53	1	0.74275	.	.	1
**34817**	0	8.53	1	0.74275	.	.	1
**34818**	0	8.53	1	0.74275	.	.	1
**34819**	0	8.53	1	0.74275	.	.	1
**34820**	0	8.53	1	0.74275	.	.	1
**34821**	0	8.53	1	0.74275	.	.	1
**34822**	0	8.53	1	0.74275	.	.	1
**34823**	0	8.53	1	0.74275	.	.	1
**34824**	0	8.53	1	0.74275	.	.	1
**34825**	0	8.53	1	0.74275	.	.	1

Obs	Sub_group	fuptimey	_CENSOR_	SURVIVAL	SDF_LCL	SDF_UCL	STRATUM
34826	0	8.53	1	0.74275	.	.	1
34827	0	8.53	1	0.74275	.	.	1
34828	0	8.53	1	0.74275	.	.	1
34829	0	8.53	1	0.74275	.	.	1
34830	0	8.53	1	0.74275	.	.	1
34831	0	8.53	1	0.74275	.	.	1
34832	0	8.53	1	0.74275	.	.	1
34833	0	8.53	1	0.74275	.	.	1
34834	0	8.53	1	0.74275	.	.	1
34835	0	8.53	1	0.74275	.	.	1
34836	0	8.53	1	0.74275	.	.	1
34837	0	8.53	1	0.74275	.	.	1
34838	0	8.53	1	0.74275	.	.	1
34839	0	8.53	1	0.74275	.	.	1
34840	0	8.53	1	0.74275	.	.	1
34841	0	8.53	1	0.74275	.	.	1
34842	0	8.53	1	0.74275	.	.	1
34843	0	8.53	1	0.74275	.	.	1
34844	0	8.53	1	0.74275	.	.	1
34845	0	8.53	1	0.74275	.	.	1
34846	0	8.53	1	0.74275	.	.	1
34847	0	8.53	1	0.74275	.	.	1
34848	0	8.53	1	0.74275	.	.	1
34849	0	8.54	0	0.74210	0.73611	0.74799	1
34850	0	8.54	1	0.74210	.	.	1
34851	0	8.54	1	0.74210	.	.	1
34852	0	8.54	1	0.74210	.	.	1
34853	0	8.54	1	0.74210	.	.	1
34854	0	8.54	1	0.74210	.	.	1
34855	0	8.54	1	0.74210	.	.	1
34856	0	8.54	1	0.74210	.	.	1
34857	0	8.54	1	0.74210	.	.	1
34858	0	8.54	1	0.74210	.	.	1
34859	0	8.54	1	0.74210	.	.	1
34860	0	8.54	1	0.74210	.	.	1

Obs	Sub_group	fuptimey	_CENSOR_	SURVIVAL	SDF_LCL	SDF_UCL	STRATUM
34861	0	8.54	1	0.74210	.	.	1
34862	0	8.54	1	0.74210	.	.	1
34863	0	8.54	1	0.74210	.	.	1
34864	0	8.54	1	0.74210	.	.	1
34865	0	8.54	1	0.74210	.	.	1
34866	0	8.54	1	0.74210	.	.	1
34867	0	8.54	1	0.74210	.	.	1
34868	0	8.54	1	0.74210	.	.	1
34869	0	8.54	1	0.74210	.	.	1
34870	0	8.54	1	0.74210	.	.	1
34871	0	8.54	1	0.74210	.	.	1
34872	0	8.54	1	0.74210	.	.	1
34873	0	8.54	1	0.74210	.	.	1
34874	0	8.54	1	0.74210	.	.	1
34875	0	8.54	1	0.74210	.	.	1
34876	0	8.54	1	0.74210	.	.	1
34877	0	8.54	1	0.74210	.	.	1
34878	0	8.54	1	0.74210	.	.	1
34879	0	8.54	1	0.74210	.	.	1
34880	0	8.54	1	0.74210	.	.	1
34881	0	8.55	0	0.74162	0.73560	0.74752	1
34882	0	8.55	1	0.74162	.	.	1
34883	0	8.55	1	0.74162	.	.	1
34884	0	8.55	1	0.74162	.	.	1
34885	0	8.55	1	0.74162	.	.	1
34886	0	8.55	1	0.74162	.	.	1
34887	0	8.55	1	0.74162	.	.	1
34888	0	8.55	1	0.74162	.	.	1
34889	0	8.55	1	0.74162	.	.	1
34890	0	8.55	1	0.74162	.	.	1
34891	0	8.55	1	0.74162	.	.	1
34892	0	8.55	1	0.74162	.	.	1
34893	0	8.55	1	0.74162	.	.	1
34894	0	8.55	1	0.74162	.	.	1
34895	0	8.55	1	0.74162	.	.	1

Obs	Sub_group	fuptimey	_CENSOR_	SURVIVAL	SDF_LCL	SDF_UCL	STRATUM
34896	0	8.55	1	0.74162	.	.	1
34897	0	8.55	1	0.74162	.	.	1
34898	0	8.55	1	0.74162	.	.	1
34899	0	8.55	1	0.74162	.	.	1
34900	0	8.55	1	0.74162	.	.	1
34901	0	8.55	1	0.74162	.	.	1
34902	0	8.55	1	0.74162	.	.	1
34903	0	8.55	1	0.74162	.	.	1
34904	0	8.55	1	0.74162	.	.	1
34905	0	8.55	1	0.74162	.	.	1
34906	0	8.55	1	0.74162	.	.	1
34907	0	8.55	1	0.74162	.	.	1
34908	0	8.55	1	0.74162	.	.	1
34909	0	8.55	1	0.74162	.	.	1
34910	0	8.55	1	0.74162	.	.	1
34911	0	8.55	1	0.74162	.	.	1
34912	0	8.55	1	0.74162	.	.	1
34913	0	8.55	1	0.74162	.	.	1
34914	0	8.55	1	0.74162	.	.	1
34915	0	8.55	1	0.74162	.	.	1
34916	0	8.55	1	0.74162	.	.	1
34917	0	8.55	1	0.74162	.	.	1
34918	0	8.55	1	0.74162	.	.	1
34919	0	8.55	1	0.74162	.	.	1
34920	0	8.55	1	0.74162	.	.	1
34921	0	8.55	1	0.74162	.	.	1
34922	0	8.56	0	0.74146	0.73543	0.74737	1
34923	0	8.56	1	0.74146	.	.	1
34924	0	8.56	1	0.74146	.	.	1
34925	0	8.56	1	0.74146	.	.	1
34926	0	8.56	1	0.74146	.	.	1
34927	0	8.56	1	0.74146	.	.	1
34928	0	8.56	1	0.74146	.	.	1
34929	0	8.56	1	0.74146	.	.	1
34930	0	8.56	1	0.74146	.	.	1

Obs	Sub_group	fuptimey	_CENSOR_	SURVIVAL	SDF_LCL	SDF_UCL	STRATUM
**34931**	0	8.56	1	0.74146	.	.	1
**34932**	0	8.56	1	0.74146	.	.	1
**34933**	0	8.56	1	0.74146	.	.	1
**34934**	0	8.56	1	0.74146	.	.	1
**34935**	0	8.56	1	0.74146	.	.	1
**34936**	0	8.56	1	0.74146	.	.	1
**34937**	0	8.56	1	0.74146	.	.	1
**34938**	0	8.56	1	0.74146	.	.	1
**34939**	0	8.56	1	0.74146	.	.	1
**34940**	0	8.56	1	0.74146	.	.	1
**34941**	0	8.56	1	0.74146	.	.	1
**34942**	0	8.56	1	0.74146	.	.	1
**34943**	0	8.56	1	0.74146	.	.	1
**34944**	0	8.56	1	0.74146	.	.	1
**34945**	0	8.56	1	0.74146	.	.	1
**34946**	0	8.56	1	0.74146	.	.	1
**34947**	0	8.56	1	0.74146	.	.	1
**34948**	0	8.56	1	0.74146	.	.	1
**34949**	0	8.56	1	0.74146	.	.	1
**34950**	0	8.56	1	0.74146	.	.	1
**34951**	0	8.56	1	0.74146	.	.	1
**34952**	0	8.56	1	0.74146	.	.	1
**34953**	0	8.56	1	0.74146	.	.	1
**34954**	0	8.57	0	0.74129	0.73526	0.74721	1
**34955**	0	8.57	1	0.74129	.	.	1
**34956**	0	8.57	1	0.74129	.	.	1
**34957**	0	8.57	1	0.74129	.	.	1
**34958**	0	8.57	1	0.74129	.	.	1
**34959**	0	8.57	1	0.74129	.	.	1
**34960**	0	8.57	1	0.74129	.	.	1
**34961**	0	8.57	1	0.74129	.	.	1
**34962**	0	8.57	1	0.74129	.	.	1
**34963**	0	8.57	1	0.74129	.	.	1
**34964**	0	8.57	1	0.74129	.	.	1
**34965**	0	8.57	1	0.74129	.	.	1

Obs	Sub_group	fuptimey	_CENSOR_	SURVIVAL	SDF_LCL	SDF_UCL	STRATUM
34966	0	8.57	1	0.74129	.	.	1
34967	0	8.57	1	0.74129	.	.	1
34968	0	8.57	1	0.74129	.	.	1
34969	0	8.57	1	0.74129	.	.	1
34970	0	8.57	1	0.74129	.	.	1
34971	0	8.57	1	0.74129	.	.	1
34972	0	8.57	1	0.74129	.	.	1
34973	0	8.57	1	0.74129	.	.	1
34974	0	8.57	1	0.74129	.	.	1
34975	0	8.57	1	0.74129	.	.	1
34976	0	8.57	1	0.74129	.	.	1
34977	0	8.57	1	0.74129	.	.	1
34978	0	8.57	1	0.74129	.	.	1
34979	0	8.57	1	0.74129	.	.	1
34980	0	8.57	1	0.74129	.	.	1
34981	0	8.57	1	0.74129	.	.	1
34982	0	8.57	1	0.74129	.	.	1
34983	0	8.57	1	0.74129	.	.	1
34984	0	8.57	1	0.74129	.	.	1
34985	0	8.57	1	0.74129	.	.	1
34986	0	8.57	1	0.74129	.	.	1
34987	0	8.58	0	0.74096	0.73492	0.74689	1
34988	0	8.58	1	0.74096	.	.	1
34989	0	8.58	1	0.74096	.	.	1
34990	0	8.58	1	0.74096	.	.	1
34991	0	8.58	1	0.74096	.	.	1
34992	0	8.58	1	0.74096	.	.	1
34993	0	8.58	1	0.74096	.	.	1
34994	0	8.58	1	0.74096	.	.	1
34995	0	8.58	1	0.74096	.	.	1
34996	0	8.58	1	0.74096	.	.	1
34997	0	8.58	1	0.74096	.	.	1
34998	0	8.58	1	0.74096	.	.	1
34999	0	8.58	1	0.74096	.	.	1
35000	0	8.58	1	0.74096	.	.	1

Obs	Sub_group	fuptimey	_CENSOR_	SURVIVAL	SDF_LCL	SDF_UCL	STRATUM
35001	0	8.58	1	0.74096	.	.	1
35002	0	8.58	1	0.74096	.	.	1
35003	0	8.58	1	0.74096	.	.	1
35004	0	8.58	1	0.74096	.	.	1
35005	0	8.58	1	0.74096	.	.	1
35006	0	8.58	1	0.74096	.	.	1
35007	0	8.58	1	0.74096	.	.	1
35008	0	8.58	1	0.74096	.	.	1
35009	0	8.58	1	0.74096	.	.	1
35010	0	8.58	1	0.74096	.	.	1
35011	0	8.58	1	0.74096	.	.	1
35012	0	8.58	1	0.74096	.	.	1
35013	0	8.58	1	0.74096	.	.	1
35014	0	8.58	1	0.74096	.	.	1
35015	0	8.58	1	0.74096	.	.	1
35016	0	8.58	1	0.74096	.	.	1
35017	0	8.58	1	0.74096	.	.	1
35018	0	8.58	1	0.74096	.	.	1
35019	0	8.58	1	0.74096	.	.	1
35020	0	8.58	1	0.74096	.	.	1
35021	0	8.58	1	0.74096	.	.	1
35022	0	8.59	0	0.74063	0.73457	0.74657	1
35023	0	8.59	1	0.74063	.	.	1
35024	0	8.59	1	0.74063	.	.	1
35025	0	8.59	1	0.74063	.	.	1
35026	0	8.59	1	0.74063	.	.	1
35027	0	8.59	1	0.74063	.	.	1
35028	0	8.59	1	0.74063	.	.	1
35029	0	8.59	1	0.74063	.	.	1
35030	0	8.59	1	0.74063	.	.	1
35031	0	8.59	1	0.74063	.	.	1
35032	0	8.59	1	0.74063	.	.	1
35033	0	8.59	1	0.74063	.	.	1
35034	0	8.59	1	0.74063	.	.	1
35035	0	8.59	1	0.74063	.	.	1

Obs	Sub_group	fuptimey	_CENSOR_	SURVIVAL	SDF_LCL	SDF_UCL	STRATUM
35036	0	8.59	1	0.74063	.	.	1
35037	0	8.59	1	0.74063	.	.	1
35038	0	8.59	1	0.74063	.	.	1
35039	0	8.59	1	0.74063	.	.	1
35040	0	8.59	1	0.74063	.	.	1
35041	0	8.59	1	0.74063	.	.	1
35042	0	8.59	1	0.74063	.	.	1
35043	0	8.59	1	0.74063	.	.	1
35044	0	8.59	1	0.74063	.	.	1
35045	0	8.59	1	0.74063	.	.	1
35046	0	8.59	1	0.74063	.	.	1
35047	0	8.59	1	0.74063	.	.	1
35048	0	8.59	1	0.74063	.	.	1
35049	0	8.59	1	0.74063	.	.	1
35050	0	8.59	1	0.74063	.	.	1
35051	0	8.59	1	0.74063	.	.	1
35052	0	8.59	1	0.74063	.	.	1
35053	0	8.59	1	0.74063	.	.	1
35054	0	8.59	1	0.74063	.	.	1
35055	0	8.59	1	0.74063	.	.	1
35056	0	8.59	1	0.74063	.	.	1
35057	0	8.59	1	0.74063	.	.	1
35058	0	8.59	1	0.74063	.	.	1
35059	0	8.59	1	0.74063	.	.	1
35060	0	8.59	1	0.74063	.	.	1
35061	0	8.59	1	0.74063	.	.	1
35062	0	8.59	1	0.74063	.	.	1
35063	0	8.59	1	0.74063	.	.	1
35064	0	8.59	1	0.74063	.	.	1
35065	0	8.59	1	0.74063	.	.	1
35066	0	8.59	1	0.74063	.	.	1
35067	0	8.59	1	0.74063	.	.	1
35068	0	8.60	1	0.74063	.	.	1
35069	0	8.60	1	0.74063	.	.	1
35070	0	8.60	1	0.74063	.	.	1

Obs	Sub_group	fuptimey	_CENSOR_	SURVIVAL	SDF_LCL	SDF_UCL	STRATUM
35071	0	8.60	1	0.74063	.	.	1
35072	0	8.60	1	0.74063	.	.	1
35073	0	8.60	1	0.74063	.	.	1
35074	0	8.60	1	0.74063	.	.	1
35075	0	8.60	1	0.74063	.	.	1
35076	0	8.60	1	0.74063	.	.	1
35077	0	8.60	1	0.74063	.	.	1
35078	0	8.60	1	0.74063	.	.	1
35079	0	8.60	1	0.74063	.	.	1
35080	0	8.60	1	0.74063	.	.	1
35081	0	8.60	1	0.74063	.	.	1
35082	0	8.60	1	0.74063	.	.	1
35083	0	8.60	1	0.74063	.	.	1
35084	0	8.60	1	0.74063	.	.	1
35085	0	8.60	1	0.74063	.	.	1
35086	0	8.60	1	0.74063	.	.	1
35087	0	8.60	1	0.74063	.	.	1
35088	0	8.60	1	0.74063	.	.	1
35089	0	8.60	1	0.74063	.	.	1
35090	0	8.60	1	0.74063	.	.	1
35091	0	8.60	1	0.74063	.	.	1
35092	0	8.60	1	0.74063	.	.	1
35093	0	8.60	1	0.74063	.	.	1
35094	0	8.60	1	0.74063	.	.	1
35095	0	8.60	1	0.74063	.	.	1
35096	0	8.60	1	0.74063	.	.	1
35097	0	8.60	1	0.74063	.	.	1
35098	0	8.60	1	0.74063	.	.	1
35099	0	8.60	1	0.74063	.	.	1
35100	0	8.60	1	0.74063	.	.	1
35101	0	8.60	1	0.74063	.	.	1
35102	0	8.60	1	0.74063	.	.	1
35103	0	8.60	1	0.74063	.	.	1
35104	0	8.60	1	0.74063	.	.	1
35105	0	8.61	0	0.74029	0.73421	0.74625	1

Obs	Sub_group	fuptimey	_CENSOR_	SURVIVAL	SDF_LCL	SDF_UCL	STRATUM
35106	0	8.61	1	0.74029	.	.	1
35107	0	8.61	1	0.74029	.	.	1
35108	0	8.61	1	0.74029	.	.	1
35109	0	8.61	1	0.74029	.	.	1
35110	0	8.61	1	0.74029	.	.	1
35111	0	8.61	1	0.74029	.	.	1
35112	0	8.61	1	0.74029	.	.	1
35113	0	8.61	1	0.74029	.	.	1
35114	0	8.61	1	0.74029	.	.	1
35115	0	8.61	1	0.74029	.	.	1
35116	0	8.61	1	0.74029	.	.	1
35117	0	8.61	1	0.74029	.	.	1
35118	0	8.61	1	0.74029	.	.	1
35119	0	8.61	1	0.74029	.	.	1
35120	0	8.61	1	0.74029	.	.	1
35121	0	8.61	1	0.74029	.	.	1
35122	0	8.61	1	0.74029	.	.	1
35123	0	8.61	1	0.74029	.	.	1
35124	0	8.61	1	0.74029	.	.	1
35125	0	8.61	1	0.74029	.	.	1
35126	0	8.61	1	0.74029	.	.	1
35127	0	8.61	1	0.74029	.	.	1
35128	0	8.61	1	0.74029	.	.	1
35129	0	8.61	1	0.74029	.	.	1
35130	0	8.61	1	0.74029	.	.	1
35131	0	8.61	1	0.74029	.	.	1
35132	0	8.61	1	0.74029	.	.	1
35133	0	8.61	1	0.74029	.	.	1
35134	0	8.61	1	0.74029	.	.	1
35135	0	8.62	0	0.73978	0.73367	0.74576	1
35136	0	8.62	1	0.73978	.	.	1
35137	0	8.62	1	0.73978	.	.	1
35138	0	8.62	1	0.73978	.	.	1
35139	0	8.62	1	0.73978	.	.	1
35140	0	8.62	1	0.73978	.	.	1

Obs	Sub_group	fuptimey	_CENSOR_	SURVIVAL	SDF_LCL	SDF_UCL	STRATUM
35141	0	8.62	1	0.73978	.	.	1
35142	0	8.62	1	0.73978	.	.	1
35143	0	8.62	1	0.73978	.	.	1
35144	0	8.62	1	0.73978	.	.	1
35145	0	8.62	1	0.73978	.	.	1
35146	0	8.62	1	0.73978	.	.	1
35147	0	8.62	1	0.73978	.	.	1
35148	0	8.62	1	0.73978	.	.	1
35149	0	8.62	1	0.73978	.	.	1
35150	0	8.62	1	0.73978	.	.	1
35151	0	8.62	1	0.73978	.	.	1
35152	0	8.62	1	0.73978	.	.	1
35153	0	8.62	1	0.73978	.	.	1
35154	0	8.62	1	0.73978	.	.	1
35155	0	8.62	1	0.73978	.	.	1
35156	0	8.62	1	0.73978	.	.	1
35157	0	8.62	1	0.73978	.	.	1
35158	0	8.63	0	0.73960	0.73349	0.74560	1
35159	0	8.63	1	0.73960	.	.	1
35160	0	8.63	1	0.73960	.	.	1
35161	0	8.63	1	0.73960	.	.	1
35162	0	8.63	1	0.73960	.	.	1
35163	0	8.63	1	0.73960	.	.	1
35164	0	8.63	1	0.73960	.	.	1
35165	0	8.63	1	0.73960	.	.	1
35166	0	8.63	1	0.73960	.	.	1
35167	0	8.63	1	0.73960	.	.	1
35168	0	8.63	1	0.73960	.	.	1
35169	0	8.63	1	0.73960	.	.	1
35170	0	8.63	1	0.73960	.	.	1
35171	0	8.63	1	0.73960	.	.	1
35172	0	8.63	1	0.73960	.	.	1
35173	0	8.63	1	0.73960	.	.	1
35174	0	8.63	1	0.73960	.	.	1
35175	0	8.63	1	0.73960	.	.	1

Obs	Sub_group	fuptimey	_CENSOR_	SURVIVAL	SDF_LCL	SDF_UCL	STRATUM
35176	0	8.63	1	0.73960	.	.	1
35177	0	8.63	1	0.73960	.	.	1
35178	0	8.63	1	0.73960	.	.	1
35179	0	8.63	1	0.73960	.	.	1
35180	0	8.63	1	0.73960	.	.	1
35181	0	8.63	1	0.73960	.	.	1
35182	0	8.63	1	0.73960	.	.	1
35183	0	8.63	1	0.73960	.	.	1
35184	0	8.63	1	0.73960	.	.	1
35185	0	8.63	1	0.73960	.	.	1
35186	0	8.63	1	0.73960	.	.	1
35187	0	8.63	1	0.73960	.	.	1
35188	0	8.63	1	0.73960	.	.	1
35189	0	8.63	1	0.73960	.	.	1
35190	0	8.63	1	0.73960	.	.	1
35191	0	8.63	1	0.73960	.	.	1
35192	0	8.63	1	0.73960	.	.	1
35193	0	8.63	1	0.73960	.	.	1
35194	0	8.63	1	0.73960	.	.	1
35195	0	8.63	1	0.73960	.	.	1
35196	0	8.64	0	0.73943	0.73331	0.74543	1
35197	0	8.64	1	0.73943	.	.	1
35198	0	8.64	1	0.73943	.	.	1
35199	0	8.64	1	0.73943	.	.	1
35200	0	8.64	1	0.73943	.	.	1
35201	0	8.64	1	0.73943	.	.	1
35202	0	8.64	1	0.73943	.	.	1
35203	0	8.64	1	0.73943	.	.	1
35204	0	8.64	1	0.73943	.	.	1
35205	0	8.64	1	0.73943	.	.	1
35206	0	8.64	1	0.73943	.	.	1
35207	0	8.64	1	0.73943	.	.	1
35208	0	8.64	1	0.73943	.	.	1
35209	0	8.64	1	0.73943	.	.	1
35210	0	8.64	1	0.73943	.	.	1

Obs	Sub_group	fuptimey	_CENSOR_	SURVIVAL	SDF_LCL	SDF_UCL	STRATUM
35211	0	8.64	1	0.73943	.	.	1
35212	0	8.64	1	0.73943	.	.	1
35213	0	8.64	1	0.73943	.	.	1
35214	0	8.64	1	0.73943	.	.	1
35215	0	8.64	1	0.73943	.	.	1
35216	0	8.64	1	0.73943	.	.	1
35217	0	8.64	1	0.73943	.	.	1
35218	0	8.64	1	0.73943	.	.	1
35219	0	8.65	1	0.73943	.	.	1
35220	0	8.65	1	0.73943	.	.	1
35221	0	8.65	1	0.73943	.	.	1
35222	0	8.65	1	0.73943	.	.	1
35223	0	8.65	1	0.73943	.	.	1
35224	0	8.65	1	0.73943	.	.	1
35225	0	8.65	1	0.73943	.	.	1
35226	0	8.65	1	0.73943	.	.	1
35227	0	8.65	1	0.73943	.	.	1
35228	0	8.65	1	0.73943	.	.	1
35229	0	8.65	1	0.73943	.	.	1
35230	0	8.65	1	0.73943	.	.	1
35231	0	8.65	1	0.73943	.	.	1
35232	0	8.65	1	0.73943	.	.	1
35233	0	8.65	1	0.73943	.	.	1
35234	0	8.65	1	0.73943	.	.	1
35235	0	8.65	1	0.73943	.	.	1
35236	0	8.65	1	0.73943	.	.	1
35237	0	8.65	1	0.73943	.	.	1
35238	0	8.65	1	0.73943	.	.	1
35239	0	8.65	1	0.73943	.	.	1
35240	0	8.65	1	0.73943	.	.	1
35241	0	8.65	1	0.73943	.	.	1
35242	0	8.65	1	0.73943	.	.	1
35243	0	8.65	1	0.73943	.	.	1
35244	0	8.65	1	0.73943	.	.	1
35245	0	8.65	1	0.73943	.	.	1

Obs	Sub_group	fuptimey	_CENSOR_	SURVIVAL	SDF_LCL	SDF_UCL	STRATUM
35246	0	8.65	1	0.73943	.	.	1
35247	0	8.65	1	0.73943	.	.	1
35248	0	8.65	1	0.73943	.	.	1
35249	0	8.65	1	0.73943	.	.	1
35250	0	8.66	0	0.73925	0.73313	0.74526	1
35251	0	8.66	1	0.73925	.	.	1
35252	0	8.66	1	0.73925	.	.	1
35253	0	8.66	1	0.73925	.	.	1
35254	0	8.66	1	0.73925	.	.	1
35255	0	8.66	1	0.73925	.	.	1
35256	0	8.66	1	0.73925	.	.	1
35257	0	8.66	1	0.73925	.	.	1
35258	0	8.66	1	0.73925	.	.	1
35259	0	8.66	1	0.73925	.	.	1
35260	0	8.66	1	0.73925	.	.	1
35261	0	8.66	1	0.73925	.	.	1
35262	0	8.66	1	0.73925	.	.	1
35263	0	8.66	1	0.73925	.	.	1
35264	0	8.66	1	0.73925	.	.	1
35265	0	8.66	1	0.73925	.	.	1
35266	0	8.66	1	0.73925	.	.	1
35267	0	8.66	1	0.73925	.	.	1
35268	0	8.66	1	0.73925	.	.	1
35269	0	8.66	1	0.73925	.	.	1
35270	0	8.66	1	0.73925	.	.	1
35271	0	8.67	1	0.73925	.	.	1
35272	0	8.67	1	0.73925	.	.	1
35273	0	8.67	1	0.73925	.	.	1
35274	0	8.67	1	0.73925	.	.	1
35275	0	8.67	1	0.73925	.	.	1
35276	0	8.67	1	0.73925	.	.	1
35277	0	8.67	1	0.73925	.	.	1
35278	0	8.67	1	0.73925	.	.	1
35279	0	8.67	1	0.73925	.	.	1
35280	0	8.67	1	0.73925	.	.	1

Obs	Sub_group	fuptimey	_CENSOR_	SURVIVAL	SDF_LCL	SDF_UCL	STRATUM
35281	0	8.67	1	0.73925	.	.	1
35282	0	8.67	1	0.73925	.	.	1
35283	0	8.67	1	0.73925	.	.	1
35284	0	8.67	1	0.73925	.	.	1
35285	0	8.67	1	0.73925	.	.	1
35286	0	8.67	1	0.73925	.	.	1
35287	0	8.67	1	0.73925	.	.	1
35288	0	8.67	1	0.73925	.	.	1
35289	0	8.67	1	0.73925	.	.	1
35290	0	8.67	1	0.73925	.	.	1
35291	0	8.67	1	0.73925	.	.	1
35292	0	8.67	1	0.73925	.	.	1
35293	0	8.67	1	0.73925	.	.	1
35294	0	8.67	1	0.73925	.	.	1
35295	0	8.67	1	0.73925	.	.	1
35296	0	8.67	1	0.73925	.	.	1
35297	0	8.67	1	0.73925	.	.	1
35298	0	8.67	1	0.73925	.	.	1
35299	0	8.67	1	0.73925	.	.	1
35300	0	8.67	1	0.73925	.	.	1
35301	0	8.67	1	0.73925	.	.	1
35302	0	8.67	1	0.73925	.	.	1
35303	0	8.67	1	0.73925	.	.	1
35304	0	8.67	1	0.73925	.	.	1
35305	0	8.67	1	0.73925	.	.	1
35306	0	8.67	1	0.73925	.	.	1
35307	0	8.67	1	0.73925	.	.	1
35308	0	8.67	1	0.73925	.	.	1
35309	0	8.68	0	0.73908	0.73294	0.74509	1
35310	0	8.68	1	0.73908	.	.	1
35311	0	8.68	1	0.73908	.	.	1
35312	0	8.68	1	0.73908	.	.	1
35313	0	8.68	1	0.73908	.	.	1
35314	0	8.68	1	0.73908	.	.	1
35315	0	8.68	1	0.73908	.	.	1

Obs	Sub_group	fuptimey	_CENSOR_	SURVIVAL	SDF_LCL	SDF_UCL	STRATUM
35316	0	8.68	1	0.73908	.	.	1
35317	0	8.68	1	0.73908	.	.	1
35318	0	8.68	1	0.73908	.	.	1
35319	0	8.68	1	0.73908	.	.	1
35320	0	8.68	1	0.73908	.	.	1
35321	0	8.68	1	0.73908	.	.	1
35322	0	8.68	1	0.73908	.	.	1
35323	0	8.68	1	0.73908	.	.	1
35324	0	8.68	1	0.73908	.	.	1
35325	0	8.68	1	0.73908	.	.	1
35326	0	8.68	1	0.73908	.	.	1
35327	0	8.68	1	0.73908	.	.	1
35328	0	8.68	1	0.73908	.	.	1
35329	0	8.68	1	0.73908	.	.	1
35330	0	8.68	1	0.73908	.	.	1
35331	0	8.68	1	0.73908	.	.	1
35332	0	8.68	1	0.73908	.	.	1
35333	0	8.68	1	0.73908	.	.	1
35334	0	8.68	1	0.73908	.	.	1
35335	0	8.69	1	0.73908	.	.	1
35336	0	8.69	1	0.73908	.	.	1
35337	0	8.69	1	0.73908	.	.	1
35338	0	8.69	1	0.73908	.	.	1
35339	0	8.69	1	0.73908	.	.	1
35340	0	8.69	1	0.73908	.	.	1
35341	0	8.69	1	0.73908	.	.	1
35342	0	8.69	1	0.73908	.	.	1
35343	0	8.69	1	0.73908	.	.	1
35344	0	8.69	1	0.73908	.	.	1
35345	0	8.69	1	0.73908	.	.	1
35346	0	8.69	1	0.73908	.	.	1
35347	0	8.69	1	0.73908	.	.	1
35348	0	8.69	1	0.73908	.	.	1
35349	0	8.69	1	0.73908	.	.	1
35350	0	8.69	1	0.73908	.	.	1

Obs	Sub_group	fuptimey	_CENSOR_	SURVIVAL	SDF_LCL	SDF_UCL	STRATUM
35351	0	8.69	1	0.73908	.	.	1
35352	0	8.69	1	0.73908	.	.	1
35353	0	8.69	1	0.73908	.	.	1
35354	0	8.69	1	0.73908	.	.	1
35355	0	8.69	1	0.73908	.	.	1
35356	0	8.69	1	0.73908	.	.	1
35357	0	8.69	1	0.73908	.	.	1
35358	0	8.69	1	0.73908	.	.	1
35359	0	8.69	1	0.73908	.	.	1
35360	0	8.70	0	0.73890	0.73275	0.74492	1
35361	0	8.70	1	0.73890	.	.	1
35362	0	8.70	1	0.73890	.	.	1
35363	0	8.70	1	0.73890	.	.	1
35364	0	8.70	1	0.73890	.	.	1
35365	0	8.70	1	0.73890	.	.	1
35366	0	8.70	1	0.73890	.	.	1
35367	0	8.70	1	0.73890	.	.	1
35368	0	8.70	1	0.73890	.	.	1
35369	0	8.70	1	0.73890	.	.	1
35370	0	8.70	1	0.73890	.	.	1
35371	0	8.70	1	0.73890	.	.	1
35372	0	8.70	1	0.73890	.	.	1
35373	0	8.70	1	0.73890	.	.	1
35374	0	8.70	1	0.73890	.	.	1
35375	0	8.70	1	0.73890	.	.	1
35376	0	8.70	1	0.73890	.	.	1
35377	0	8.70	1	0.73890	.	.	1
35378	0	8.70	1	0.73890	.	.	1
35379	0	8.70	1	0.73890	.	.	1
35380	0	8.70	1	0.73890	.	.	1
35381	0	8.71	0	0.73835	0.73218	0.74441	1
35382	0	8.71	1	0.73835	.	.	1
35383	0	8.71	1	0.73835	.	.	1
35384	0	8.71	1	0.73835	.	.	1
35385	0	8.71	1	0.73835	.	.	1

Obs	Sub_group	fuptimey	_CENSOR_	SURVIVAL	SDF_LCL	SDF_UCL	STRATUM
35386	0	8.71	1	0.73835	.	.	1
35387	0	8.71	1	0.73835	.	.	1
35388	0	8.71	1	0.73835	.	.	1
35389	0	8.71	1	0.73835	.	.	1
35390	0	8.71	1	0.73835	.	.	1
35391	0	8.71	1	0.73835	.	.	1
35392	0	8.71	1	0.73835	.	.	1
35393	0	8.71	1	0.73835	.	.	1
35394	0	8.71	1	0.73835	.	.	1
35395	0	8.71	1	0.73835	.	.	1
35396	0	8.71	1	0.73835	.	.	1
35397	0	8.71	1	0.73835	.	.	1
35398	0	8.71	1	0.73835	.	.	1
35399	0	8.71	1	0.73835	.	.	1
35400	0	8.71	1	0.73835	.	.	1
35401	0	8.71	1	0.73835	.	.	1
35402	0	8.71	1	0.73835	.	.	1
35403	0	8.71	1	0.73835	.	.	1
35404	0	8.71	1	0.73835	.	.	1
35405	0	8.71	1	0.73835	.	.	1
35406	0	8.71	1	0.73835	.	.	1
35407	0	8.71	1	0.73835	.	.	1
35408	0	8.71	1	0.73835	.	.	1
35409	0	8.71	1	0.73835	.	.	1
35410	0	8.71	1	0.73835	.	.	1
35411	0	8.71	1	0.73835	.	.	1
35412	0	8.71	1	0.73835	.	.	1
35413	0	8.71	1	0.73835	.	.	1
35414	0	8.71	1	0.73835	.	.	1
35415	0	8.72	0	0.73817	0.73199	0.74423	1
35416	0	8.72	1	0.73817	.	.	1
35417	0	8.72	1	0.73817	.	.	1
35418	0	8.72	1	0.73817	.	.	1
35419	0	8.72	1	0.73817	.	.	1
35420	0	8.72	1	0.73817	.	.	1

Obs	Sub_group	fuptimey	_CENSOR_	SURVIVAL	SDF_LCL	SDF_UCL	STRATUM
35421	0	8.72	1	0.73817	.	.	1
35422	0	8.72	1	0.73817	.	.	1
35423	0	8.72	1	0.73817	.	.	1
35424	0	8.72	1	0.73817	.	.	1
35425	0	8.72	1	0.73817	.	.	1
35426	0	8.72	1	0.73817	.	.	1
35427	0	8.72	1	0.73817	.	.	1
35428	0	8.72	1	0.73817	.	.	1
35429	0	8.72	1	0.73817	.	.	1
35430	0	8.72	1	0.73817	.	.	1
35431	0	8.72	1	0.73817	.	.	1
35432	0	8.72	1	0.73817	.	.	1
35433	0	8.72	1	0.73817	.	.	1
35434	0	8.72	1	0.73817	.	.	1
35435	0	8.72	1	0.73817	.	.	1
35436	0	8.72	1	0.73817	.	.	1
35437	0	8.72	1	0.73817	.	.	1
35438	0	8.72	1	0.73817	.	.	1
35439	0	8.72	1	0.73817	.	.	1
35440	0	8.72	1	0.73817	.	.	1
35441	0	8.72	1	0.73817	.	.	1
35442	0	8.72	1	0.73817	.	.	1
35443	0	8.72	1	0.73817	.	.	1
35444	0	8.72	1	0.73817	.	.	1
35445	0	8.72	1	0.73817	.	.	1
35446	0	8.73	0	0.73762	0.73141	0.74371	1
35447	0	8.73	1	0.73762	.	.	1
35448	0	8.73	1	0.73762	.	.	1
35449	0	8.73	1	0.73762	.	.	1
35450	0	8.73	1	0.73762	.	.	1
35451	0	8.73	1	0.73762	.	.	1
35452	0	8.73	1	0.73762	.	.	1
35453	0	8.73	1	0.73762	.	.	1
35454	0	8.73	1	0.73762	.	.	1
35455	0	8.73	1	0.73762	.	.	1

Obs	Sub_group	fuptimey	_CENSOR_	SURVIVAL	SDF_LCL	SDF_UCL	STRATUM
35456	0	8.73	1	0.73762	.	.	1
35457	0	8.73	1	0.73762	.	.	1
35458	0	8.73	1	0.73762	.	.	1
35459	0	8.73	1	0.73762	.	.	1
35460	0	8.73	1	0.73762	.	.	1
35461	0	8.73	1	0.73762	.	.	1
35462	0	8.73	1	0.73762	.	.	1
35463	0	8.73	1	0.73762	.	.	1
35464	0	8.73	1	0.73762	.	.	1
35465	0	8.73	1	0.73762	.	.	1
35466	0	8.73	1	0.73762	.	.	1
35467	0	8.73	1	0.73762	.	.	1
35468	0	8.73	1	0.73762	.	.	1
35469	0	8.73	1	0.73762	.	.	1
35470	0	8.73	1	0.73762	.	.	1
35471	0	8.73	1	0.73762	.	.	1
35472	0	8.73	1	0.73762	.	.	1
35473	0	8.73	1	0.73762	.	.	1
35474	0	8.73	1	0.73762	.	.	1
35475	0	8.73	1	0.73762	.	.	1
35476	0	8.73	1	0.73762	.	.	1
35477	0	8.73	1	0.73762	.	.	1
35478	0	8.73	1	0.73762	.	.	1
35479	0	8.73	1	0.73762	.	.	1
35480	0	8.73	1	0.73762	.	.	1
35481	0	8.73	1	0.73762	.	.	1
35482	0	8.73	1	0.73762	.	.	1
35483	0	8.73	1	0.73762	.	.	1
35484	0	8.73	1	0.73762	.	.	1
35485	0	8.73	1	0.73762	.	.	1
35486	0	8.73	1	0.73762	.	.	1
35487	0	8.74	0	0.73743	0.73122	0.74353	1
35488	0	8.74	1	0.73743	.	.	1
35489	0	8.74	1	0.73743	.	.	1
35490	0	8.74	1	0.73743	.	.	1

Obs	Sub_group	fuptimey	_CENSOR_	SURVIVAL	SDF_LCL	SDF_UCL	STRATUM
35491	0	8.74	1	0.73743	.	.	1
35492	0	8.74	1	0.73743	.	.	1
35493	0	8.74	1	0.73743	.	.	1
35494	0	8.74	1	0.73743	.	.	1
35495	0	8.74	1	0.73743	.	.	1
35496	0	8.74	1	0.73743	.	.	1
35497	0	8.74	1	0.73743	.	.	1
35498	0	8.74	1	0.73743	.	.	1
35499	0	8.74	1	0.73743	.	.	1
35500	0	8.74	1	0.73743	.	.	1
35501	0	8.74	1	0.73743	.	.	1
35502	0	8.74	1	0.73743	.	.	1
35503	0	8.74	1	0.73743	.	.	1
35504	0	8.74	1	0.73743	.	.	1
35505	0	8.74	1	0.73743	.	.	1
35506	0	8.74	1	0.73743	.	.	1
35507	0	8.74	1	0.73743	.	.	1
35508	0	8.74	1	0.73743	.	.	1
35509	0	8.74	1	0.73743	.	.	1
35510	0	8.74	1	0.73743	.	.	1
35511	0	8.74	1	0.73743	.	.	1
35512	0	8.74	1	0.73743	.	.	1
35513	0	8.74	1	0.73743	.	.	1
35514	0	8.74	1	0.73743	.	.	1
35515	0	8.75	0	0.73706	0.73083	0.74318	1
35516	0	8.75	1	0.73706	.	.	1
35517	0	8.75	1	0.73706	.	.	1
35518	0	8.75	1	0.73706	.	.	1
35519	0	8.75	1	0.73706	.	.	1
35520	0	8.75	1	0.73706	.	.	1
35521	0	8.75	1	0.73706	.	.	1
35522	0	8.75	1	0.73706	.	.	1
35523	0	8.75	1	0.73706	.	.	1
35524	0	8.75	1	0.73706	.	.	1
35525	0	8.75	1	0.73706	.	.	1

Obs	Sub_group	fuptimey	_CENSOR_	SURVIVAL	SDF_LCL	SDF_UCL	STRATUM
35526	0	8.75	1	0.73706	.	.	1
35527	0	8.75	1	0.73706	.	.	1
35528	0	8.75	1	0.73706	.	.	1
35529	0	8.75	1	0.73706	.	.	1
35530	0	8.75	1	0.73706	.	.	1
35531	0	8.75	1	0.73706	.	.	1
35532	0	8.75	1	0.73706	.	.	1
35533	0	8.75	1	0.73706	.	.	1
35534	0	8.75	1	0.73706	.	.	1
35535	0	8.75	1	0.73706	.	.	1
35536	0	8.75	1	0.73706	.	.	1
35537	0	8.75	1	0.73706	.	.	1
35538	0	8.75	1	0.73706	.	.	1
35539	0	8.75	1	0.73706	.	.	1
35540	0	8.75	1	0.73706	.	.	1
35541	0	8.75	1	0.73706	.	.	1
35542	0	8.75	1	0.73706	.	.	1
35543	0	8.75	1	0.73706	.	.	1
35544	0	8.75	1	0.73706	.	.	1
35545	0	8.75	1	0.73706	.	.	1
35546	0	8.75	1	0.73706	.	.	1
35547	0	8.76	0	0.73687	0.73063	0.74300	1
35548	0	8.76	1	0.73687	.	.	1
35549	0	8.76	1	0.73687	.	.	1
35550	0	8.76	1	0.73687	.	.	1
35551	0	8.76	1	0.73687	.	.	1
35552	0	8.76	1	0.73687	.	.	1
35553	0	8.76	1	0.73687	.	.	1
35554	0	8.76	1	0.73687	.	.	1
35555	0	8.76	1	0.73687	.	.	1
35556	0	8.76	1	0.73687	.	.	1
35557	0	8.76	1	0.73687	.	.	1
35558	0	8.76	1	0.73687	.	.	1
35559	0	8.76	1	0.73687	.	.	1
35560	0	8.76	1	0.73687	.	.	1

Obs	Sub_group	fuptimey	_CENSOR_	SURVIVAL	SDF_LCL	SDF_UCL	STRATUM
35561	0	8.76	1	0.73687	.	.	1
35562	0	8.76	1	0.73687	.	.	1
35563	0	8.76	1	0.73687	.	.	1
35564	0	8.76	1	0.73687	.	.	1
35565	0	8.76	1	0.73687	.	.	1
35566	0	8.76	1	0.73687	.	.	1
35567	0	8.76	1	0.73687	.	.	1
35568	0	8.76	1	0.73687	.	.	1
35569	0	8.76	1	0.73687	.	.	1
35570	0	8.77	0	0.73668	0.73043	0.74282	1
35571	0	8.77	1	0.73668	.	.	1
35572	0	8.77	1	0.73668	.	.	1
35573	0	8.77	1	0.73668	.	.	1
35574	0	8.77	1	0.73668	.	.	1
35575	0	8.77	1	0.73668	.	.	1
35576	0	8.77	1	0.73668	.	.	1
35577	0	8.77	1	0.73668	.	.	1
35578	0	8.77	1	0.73668	.	.	1
35579	0	8.77	1	0.73668	.	.	1
35580	0	8.77	1	0.73668	.	.	1
35581	0	8.77	1	0.73668	.	.	1
35582	0	8.77	1	0.73668	.	.	1
35583	0	8.77	1	0.73668	.	.	1
35584	0	8.77	1	0.73668	.	.	1
35585	0	8.77	1	0.73668	.	.	1
35586	0	8.77	1	0.73668	.	.	1
35587	0	8.77	1	0.73668	.	.	1
35588	0	8.77	1	0.73668	.	.	1
35589	0	8.77	1	0.73668	.	.	1
35590	0	8.77	1	0.73668	.	.	1
35591	0	8.77	1	0.73668	.	.	1
35592	0	8.77	1	0.73668	.	.	1
35593	0	8.77	1	0.73668	.	.	1
35594	0	8.77	1	0.73668	.	.	1
35595	0	8.77	1	0.73668	.	.	1

Obs	Sub_group	fuptimey	_CENSOR_	SURVIVAL	SDF_LCL	SDF_UCL	STRATUM
35596	0	8.77	1	0.73668	.	.	1
35597	0	8.77	1	0.73668	.	.	1
35598	0	8.77	1	0.73668	.	.	1
35599	0	8.77	1	0.73668	.	.	1
35600	0	8.77	1	0.73668	.	.	1
35601	0	8.77	1	0.73668	.	.	1
35602	0	8.77	1	0.73668	.	.	1
35603	0	8.78	0	0.73630	0.73003	0.74245	1
35604	0	8.78	1	0.73630	.	.	1
35605	0	8.78	1	0.73630	.	.	1
35606	0	8.78	1	0.73630	.	.	1
35607	0	8.78	1	0.73630	.	.	1
35608	0	8.78	1	0.73630	.	.	1
35609	0	8.78	1	0.73630	.	.	1
35610	0	8.78	1	0.73630	.	.	1
35611	0	8.78	1	0.73630	.	.	1
35612	0	8.78	1	0.73630	.	.	1
35613	0	8.78	1	0.73630	.	.	1
35614	0	8.78	1	0.73630	.	.	1
35615	0	8.78	1	0.73630	.	.	1
35616	0	8.78	1	0.73630	.	.	1
35617	0	8.78	1	0.73630	.	.	1
35618	0	8.78	1	0.73630	.	.	1
35619	0	8.78	1	0.73630	.	.	1
35620	0	8.78	1	0.73630	.	.	1
35621	0	8.78	1	0.73630	.	.	1
35622	0	8.78	1	0.73630	.	.	1
35623	0	8.78	1	0.73630	.	.	1
35624	0	8.78	1	0.73630	.	.	1
35625	0	8.78	1	0.73630	.	.	1
35626	0	8.78	1	0.73630	.	.	1
35627	0	8.78	1	0.73630	.	.	1
35628	0	8.78	1	0.73630	.	.	1
35629	0	8.78	1	0.73630	.	.	1
35630	0	8.78	1	0.73630	.	.	1

Obs	Sub_group	fuptimey	_CENSOR_	SURVIVAL	SDF_LCL	SDF_UCL	STRATUM
35631	0	8.78	1	0.73630	.	.	1
35632	0	8.78	1	0.73630	.	.	1
35633	0	8.78	1	0.73630	.	.	1
35634	0	8.78	1	0.73630	.	.	1
35635	0	8.78	1	0.73630	.	.	1
35636	0	8.78	1	0.73630	.	.	1
35637	0	8.79	0	0.73611	0.72982	0.74227	1
35638	0	8.79	1	0.73611	.	.	1
35639	0	8.79	1	0.73611	.	.	1
35640	0	8.79	1	0.73611	.	.	1
35641	0	8.79	1	0.73611	.	.	1
35642	0	8.79	1	0.73611	.	.	1
35643	0	8.79	1	0.73611	.	.	1
35644	0	8.79	1	0.73611	.	.	1
35645	0	8.79	1	0.73611	.	.	1
35646	0	8.79	1	0.73611	.	.	1
35647	0	8.79	1	0.73611	.	.	1
35648	0	8.79	1	0.73611	.	.	1
35649	0	8.79	1	0.73611	.	.	1
35650	0	8.79	1	0.73611	.	.	1
35651	0	8.79	1	0.73611	.	.	1
35652	0	8.79	1	0.73611	.	.	1
35653	0	8.79	1	0.73611	.	.	1
35654	0	8.79	1	0.73611	.	.	1
35655	0	8.79	1	0.73611	.	.	1
35656	0	8.79	1	0.73611	.	.	1
35657	0	8.79	1	0.73611	.	.	1
35658	0	8.79	1	0.73611	.	.	1
35659	0	8.79	1	0.73611	.	.	1
35660	0	8.79	1	0.73611	.	.	1
35661	0	8.79	1	0.73611	.	.	1
35662	0	8.79	1	0.73611	.	.	1
35663	0	8.79	1	0.73611	.	.	1
35664	0	8.79	1	0.73611	.	.	1
35665	0	8.79	1	0.73611	.	.	1

Obs	Sub_group	fuptimey	_CENSOR_	SURVIVAL	SDF_LCL	SDF_UCL	STRATUM
35666	0	8.79	1	0.73611	.	.	1
35667	0	8.79	1	0.73611	.	.	1
35668	0	8.79	1	0.73611	.	.	1
35669	0	8.79	1	0.73611	.	.	1
35670	0	8.79	1	0.73611	.	.	1
35671	0	8.79	1	0.73611	.	.	1
35672	0	8.79	1	0.73611	.	.	1
35673	0	8.80	0	0.73572	0.72941	0.74190	1
35674	0	8.80	1	0.73572	.	.	1
35675	0	8.80	1	0.73572	.	.	1
35676	0	8.80	1	0.73572	.	.	1
35677	0	8.80	1	0.73572	.	.	1
35678	0	8.80	1	0.73572	.	.	1
35679	0	8.80	1	0.73572	.	.	1
35680	0	8.80	1	0.73572	.	.	1
35681	0	8.80	1	0.73572	.	.	1
35682	0	8.80	1	0.73572	.	.	1
35683	0	8.80	1	0.73572	.	.	1
35684	0	8.80	1	0.73572	.	.	1
35685	0	8.80	1	0.73572	.	.	1
35686	0	8.80	1	0.73572	.	.	1
35687	0	8.80	1	0.73572	.	.	1
35688	0	8.80	1	0.73572	.	.	1
35689	0	8.80	1	0.73572	.	.	1
35690	0	8.80	1	0.73572	.	.	1
35691	0	8.80	1	0.73572	.	.	1
35692	0	8.80	1	0.73572	.	.	1
35693	0	8.80	1	0.73572	.	.	1
35694	0	8.80	1	0.73572	.	.	1
35695	0	8.80	1	0.73572	.	.	1
35696	0	8.80	1	0.73572	.	.	1
35697	0	8.80	1	0.73572	.	.	1
35698	0	8.80	1	0.73572	.	.	1
35699	0	8.80	1	0.73572	.	.	1
35700	0	8.80	1	0.73572	.	.	1

Obs	Sub_group	fuptimey	_CENSOR_	SURVIVAL	SDF_LCL	SDF_UCL	STRATUM
35701	0	8.80	1	0.73572	.	.	1
35702	0	8.80	1	0.73572	.	.	1
35703	0	8.80	1	0.73572	.	.	1
35704	0	8.81	1	0.73572	.	.	1
35705	0	8.81	1	0.73572	.	.	1
35706	0	8.81	1	0.73572	.	.	1
35707	0	8.81	1	0.73572	.	.	1
35708	0	8.81	1	0.73572	.	.	1
35709	0	8.81	1	0.73572	.	.	1
35710	0	8.81	1	0.73572	.	.	1
35711	0	8.81	1	0.73572	.	.	1
35712	0	8.81	1	0.73572	.	.	1
35713	0	8.81	1	0.73572	.	.	1
35714	0	8.81	1	0.73572	.	.	1
35715	0	8.81	1	0.73572	.	.	1
35716	0	8.81	1	0.73572	.	.	1
35717	0	8.81	1	0.73572	.	.	1
35718	0	8.81	1	0.73572	.	.	1
35719	0	8.81	1	0.73572	.	.	1
35720	0	8.81	1	0.73572	.	.	1
35721	0	8.81	1	0.73572	.	.	1
35722	0	8.81	1	0.73572	.	.	1
35723	0	8.81	1	0.73572	.	.	1
35724	0	8.81	1	0.73572	.	.	1
35725	0	8.81	1	0.73572	.	.	1
35726	0	8.81	1	0.73572	.	.	1
35727	0	8.81	1	0.73572	.	.	1
35728	0	8.81	1	0.73572	.	.	1
35729	0	8.81	1	0.73572	.	.	1
35730	0	8.81	1	0.73572	.	.	1
35731	0	8.81	1	0.73572	.	.	1
35732	0	8.82	0	0.73552	0.72921	0.74171	1
35733	0	8.82	1	0.73552	.	.	1
35734	0	8.82	1	0.73552	.	.	1
35735	0	8.82	1	0.73552	.	.	1

Obs	Sub_group	fuptimey	_CENSOR_	SURVIVAL	SDF_LCL	SDF_UCL	STRATUM
35736	0	8.82	1	0.73552	.	.	1
35737	0	8.82	1	0.73552	.	.	1
35738	0	8.82	1	0.73552	.	.	1
35739	0	8.82	1	0.73552	.	.	1
35740	0	8.82	1	0.73552	.	.	1
35741	0	8.82	1	0.73552	.	.	1
35742	0	8.82	1	0.73552	.	.	1
35743	0	8.82	1	0.73552	.	.	1
35744	0	8.82	1	0.73552	.	.	1
35745	0	8.82	1	0.73552	.	.	1
35746	0	8.82	1	0.73552	.	.	1
35747	0	8.82	1	0.73552	.	.	1
35748	0	8.82	1	0.73552	.	.	1
35749	0	8.82	1	0.73552	.	.	1
35750	0	8.82	1	0.73552	.	.	1
35751	0	8.82	1	0.73552	.	.	1
35752	0	8.82	1	0.73552	.	.	1
35753	0	8.82	1	0.73552	.	.	1
35754	0	8.82	1	0.73552	.	.	1
35755	0	8.82	1	0.73552	.	.	1
35756	0	8.82	1	0.73552	.	.	1
35757	0	8.82	1	0.73552	.	.	1
35758	0	8.82	1	0.73552	.	.	1
35759	0	8.82	1	0.73552	.	.	1
35760	0	8.83	0	0.73532	0.72900	0.74153	1
35761	0	8.83	1	0.73532	.	.	1
35762	0	8.83	1	0.73532	.	.	1
35763	0	8.83	1	0.73532	.	.	1
35764	0	8.83	1	0.73532	.	.	1
35765	0	8.83	1	0.73532	.	.	1
35766	0	8.83	1	0.73532	.	.	1
35767	0	8.83	1	0.73532	.	.	1
35768	0	8.83	1	0.73532	.	.	1
35769	0	8.83	1	0.73532	.	.	1
35770	0	8.83	1	0.73532	.	.	1

Obs	Sub_group	fuptimey	_CENSOR_	SURVIVAL	SDF_LCL	SDF_UCL	STRATUM
35771	0	8.83	1	0.73532	.	.	1
35772	0	8.83	1	0.73532	.	.	1
35773	0	8.83	1	0.73532	.	.	1
35774	0	8.83	1	0.73532	.	.	1
35775	0	8.83	1	0.73532	.	.	1
35776	0	8.83	1	0.73532	.	.	1
35777	0	8.83	1	0.73532	.	.	1
35778	0	8.83	1	0.73532	.	.	1
35779	0	8.83	1	0.73532	.	.	1
35780	0	8.83	1	0.73532	.	.	1
35781	0	8.83	1	0.73532	.	.	1
35782	0	8.83	1	0.73532	.	.	1
35783	0	8.83	1	0.73532	.	.	1
35784	0	8.84	0	0.73512	0.72879	0.74134	1
35785	0	8.84	1	0.73512	.	.	1
35786	0	8.84	1	0.73512	.	.	1
35787	0	8.84	1	0.73512	.	.	1
35788	0	8.84	1	0.73512	.	.	1
35789	0	8.84	1	0.73512	.	.	1
35790	0	8.84	1	0.73512	.	.	1
35791	0	8.84	1	0.73512	.	.	1
35792	0	8.84	1	0.73512	.	.	1
35793	0	8.84	1	0.73512	.	.	1
35794	0	8.84	1	0.73512	.	.	1
35795	0	8.84	1	0.73512	.	.	1
35796	0	8.84	1	0.73512	.	.	1
35797	0	8.84	1	0.73512	.	.	1
35798	0	8.84	1	0.73512	.	.	1
35799	0	8.84	1	0.73512	.	.	1
35800	0	8.84	1	0.73512	.	.	1
35801	0	8.84	1	0.73512	.	.	1
35802	0	8.84	1	0.73512	.	.	1
35803	0	8.84	1	0.73512	.	.	1
35804	0	8.84	1	0.73512	.	.	1
35805	0	8.84	1	0.73512	.	.	1

Obs	Sub_group	fuptimey	_CENSOR_	SURVIVAL	SDF_LCL	SDF_UCL	STRATUM
35806	0	8.84	1	0.73512	.	.	1
35807	0	8.84	1	0.73512	.	.	1
35808	0	8.84	1	0.73512	.	.	1
35809	0	8.84	1	0.73512	.	.	1
35810	0	8.84	1	0.73512	.	.	1
35811	0	8.84	1	0.73512	.	.	1
35812	0	8.84	1	0.73512	.	.	1
35813	0	8.84	1	0.73512	.	.	1
35814	0	8.84	1	0.73512	.	.	1
35815	0	8.84	1	0.73512	.	.	1
35816	0	8.84	1	0.73512	.	.	1
35817	0	8.84	1	0.73512	.	.	1
35818	0	8.84	1	0.73512	.	.	1
35819	0	8.84	1	0.73512	.	.	1
35820	0	8.84	1	0.73512	.	.	1
35821	0	8.85	0	0.73472	0.72836	0.74095	1
35822	0	8.85	1	0.73472	.	.	1
35823	0	8.85	1	0.73472	.	.	1
35824	0	8.85	1	0.73472	.	.	1
35825	0	8.85	1	0.73472	.	.	1
35826	0	8.85	1	0.73472	.	.	1
35827	0	8.85	1	0.73472	.	.	1
35828	0	8.85	1	0.73472	.	.	1
35829	0	8.85	1	0.73472	.	.	1
35830	0	8.85	1	0.73472	.	.	1
35831	0	8.85	1	0.73472	.	.	1
35832	0	8.85	1	0.73472	.	.	1
35833	0	8.85	1	0.73472	.	.	1
35834	0	8.85	1	0.73472	.	.	1
35835	0	8.85	1	0.73472	.	.	1
35836	0	8.85	1	0.73472	.	.	1
35837	0	8.85	1	0.73472	.	.	1
35838	0	8.85	1	0.73472	.	.	1
35839	0	8.85	1	0.73472	.	.	1
35840	0	8.86	0	0.73431	0.72793	0.74057	1

Obs	Sub_group	fuptimey	_CENSOR_	SURVIVAL	SDF_LCL	SDF_UCL	STRATUM
35841	0	8.86	1	0.73431	.	.	1
35842	0	8.86	1	0.73431	.	.	1
35843	0	8.86	1	0.73431	.	.	1
35844	0	8.86	1	0.73431	.	.	1
35845	0	8.86	1	0.73431	.	.	1
35846	0	8.86	1	0.73431	.	.	1
35847	0	8.86	1	0.73431	.	.	1
35848	0	8.86	1	0.73431	.	.	1
35849	0	8.86	1	0.73431	.	.	1
35850	0	8.86	1	0.73431	.	.	1
35851	0	8.86	1	0.73431	.	.	1
35852	0	8.86	1	0.73431	.	.	1
35853	0	8.86	1	0.73431	.	.	1
35854	0	8.86	1	0.73431	.	.	1
35855	0	8.86	1	0.73431	.	.	1
35856	0	8.86	1	0.73431	.	.	1
35857	0	8.86	1	0.73431	.	.	1
35858	0	8.86	1	0.73431	.	.	1
35859	0	8.86	1	0.73431	.	.	1
35860	0	8.86	1	0.73431	.	.	1
35861	0	8.86	1	0.73431	.	.	1
35862	0	8.86	1	0.73431	.	.	1
35863	0	8.86	1	0.73431	.	.	1
35864	0	8.86	1	0.73431	.	.	1
35865	0	8.86	1	0.73431	.	.	1
35866	0	8.86	1	0.73431	.	.	1
35867	0	8.86	1	0.73431	.	.	1
35868	0	8.86	1	0.73431	.	.	1
35869	0	8.86	1	0.73431	.	.	1
35870	0	8.86	1	0.73431	.	.	1
35871	0	8.86	1	0.73431	.	.	1
35872	0	8.86	1	0.73431	.	.	1
35873	0	8.86	1	0.73431	.	.	1
35874	0	8.86	1	0.73431	.	.	1
35875	0	8.86	1	0.73431	.	.	1

Obs	Sub_group	fuptimey	_CENSOR_	SURVIVAL	SDF_LCL	SDF_UCL	STRATUM
35876	0	8.86	1	0.73431	.	.	1
35877	0	8.86	1	0.73431	.	.	1
35878	0	8.86	1	0.73431	.	.	1
35879	0	8.87	1	0.73431	.	.	1
35880	0	8.87	1	0.73431	.	.	1
35881	0	8.87	1	0.73431	.	.	1
35882	0	8.87	1	0.73431	.	.	1
35883	0	8.87	1	0.73431	.	.	1
35884	0	8.87	1	0.73431	.	.	1
35885	0	8.87	1	0.73431	.	.	1
35886	0	8.87	1	0.73431	.	.	1
35887	0	8.87	1	0.73431	.	.	1
35888	0	8.87	1	0.73431	.	.	1
35889	0	8.87	1	0.73431	.	.	1
35890	0	8.87	1	0.73431	.	.	1
35891	0	8.87	1	0.73431	.	.	1
35892	0	8.87	1	0.73431	.	.	1
35893	0	8.87	1	0.73431	.	.	1
35894	0	8.87	1	0.73431	.	.	1
35895	0	8.87	1	0.73431	.	.	1
35896	0	8.87	1	0.73431	.	.	1
35897	0	8.87	1	0.73431	.	.	1
35898	0	8.87	1	0.73431	.	.	1
35899	0	8.87	1	0.73431	.	.	1
35900	0	8.87	1	0.73431	.	.	1
35901	0	8.87	1	0.73431	.	.	1
35902	0	8.87	1	0.73431	.	.	1
35903	0	8.87	1	0.73431	.	.	1
35904	0	8.87	1	0.73431	.	.	1
35905	0	8.87	1	0.73431	.	.	1
35906	0	8.87	1	0.73431	.	.	1
35907	0	8.87	1	0.73431	.	.	1
35908	0	8.87	1	0.73431	.	.	1
35909	0	8.87	1	0.73431	.	.	1
35910	0	8.87	1	0.73431	.	.	1

Obs	Sub_group	fuptimey	_CENSOR_	SURVIVAL	SDF_LCL	SDF_UCL	STRATUM
35911	0	8.87	1	0.73431	.	.	1
35912	0	8.87	1	0.73431	.	.	1
35913	0	8.87	1	0.73431	.	.	1
35914	0	8.87	1	0.73431	.	.	1
35915	0	8.87	1	0.73431	.	.	1
35916	0	8.88	0	0.73410	0.72771	0.74037	1
35917	0	8.88	1	0.73410	.	.	1
35918	0	8.88	1	0.73410	.	.	1
35919	0	8.88	1	0.73410	.	.	1
35920	0	8.88	1	0.73410	.	.	1
35921	0	8.88	1	0.73410	.	.	1
35922	0	8.88	1	0.73410	.	.	1
35923	0	8.88	1	0.73410	.	.	1
35924	0	8.88	1	0.73410	.	.	1
35925	0	8.88	1	0.73410	.	.	1
35926	0	8.88	1	0.73410	.	.	1
35927	0	8.88	1	0.73410	.	.	1
35928	0	8.88	1	0.73410	.	.	1
35929	0	8.88	1	0.73410	.	.	1
35930	0	8.88	1	0.73410	.	.	1
35931	0	8.88	1	0.73410	.	.	1
35932	0	8.88	1	0.73410	.	.	1
35933	0	8.88	1	0.73410	.	.	1
35934	0	8.88	1	0.73410	.	.	1
35935	0	8.88	1	0.73410	.	.	1
35936	0	8.88	1	0.73410	.	.	1
35937	0	8.88	1	0.73410	.	.	1
35938	0	8.88	1	0.73410	.	.	1
35939	0	8.88	1	0.73410	.	.	1
35940	0	8.88	1	0.73410	.	.	1
35941	0	8.88	1	0.73410	.	.	1
35942	0	8.88	1	0.73410	.	.	1
35943	0	8.88	1	0.73410	.	.	1
35944	0	8.88	1	0.73410	.	.	1
35945	0	8.88	1	0.73410	.	.	1

Obs	Sub_group	fuptimey	_CENSOR_	SURVIVAL	SDF_LCL	SDF_UCL	STRATUM
35946	0	8.89	0	0.73368	0.72727	0.73997	1
35947	0	8.89	1	0.73368	.	.	1
35948	0	8.89	1	0.73368	.	.	1
35949	0	8.89	1	0.73368	.	.	1
35950	0	8.89	1	0.73368	.	.	1
35951	0	8.89	1	0.73368	.	.	1
35952	0	8.89	1	0.73368	.	.	1
35953	0	8.89	1	0.73368	.	.	1
35954	0	8.89	1	0.73368	.	.	1
35955	0	8.89	1	0.73368	.	.	1
35956	0	8.89	1	0.73368	.	.	1
35957	0	8.89	1	0.73368	.	.	1
35958	0	8.89	1	0.73368	.	.	1
35959	0	8.89	1	0.73368	.	.	1
35960	0	8.89	1	0.73368	.	.	1
35961	0	8.89	1	0.73368	.	.	1
35962	0	8.89	1	0.73368	.	.	1
35963	0	8.89	1	0.73368	.	.	1
35964	0	8.89	1	0.73368	.	.	1
35965	0	8.89	1	0.73368	.	.	1
35966	0	8.89	1	0.73368	.	.	1
35967	0	8.89	1	0.73368	.	.	1
35968	0	8.89	1	0.73368	.	.	1
35969	0	8.89	1	0.73368	.	.	1
35970	0	8.89	1	0.73368	.	.	1
35971	0	8.90	0	0.73347	0.72705	0.73978	1
35972	0	8.90	1	0.73347	.	.	1
35973	0	8.90	1	0.73347	.	.	1
35974	0	8.90	1	0.73347	.	.	1
35975	0	8.90	1	0.73347	.	.	1
35976	0	8.90	1	0.73347	.	.	1
35977	0	8.90	1	0.73347	.	.	1
35978	0	8.90	1	0.73347	.	.	1
35979	0	8.90	1	0.73347	.	.	1
35980	0	8.90	1	0.73347	.	.	1

Obs	Sub_group	fuptimey	_CENSOR_	SURVIVAL	SDF_LCL	SDF_UCL	STRATUM
35981	0	8.90	1	0.73347	.	.	1
35982	0	8.90	1	0.73347	.	.	1
35983	0	8.90	1	0.73347	.	.	1
35984	0	8.90	1	0.73347	.	.	1
35985	0	8.90	1	0.73347	.	.	1
35986	0	8.90	1	0.73347	.	.	1
35987	0	8.90	1	0.73347	.	.	1
35988	0	8.90	1	0.73347	.	.	1
35989	0	8.90	1	0.73347	.	.	1
35990	0	8.90	1	0.73347	.	.	1
35991	0	8.90	1	0.73347	.	.	1
35992	0	8.90	1	0.73347	.	.	1
35993	0	8.90	1	0.73347	.	.	1
35994	0	8.90	1	0.73347	.	.	1
35995	0	8.90	1	0.73347	.	.	1
35996	0	8.90	1	0.73347	.	.	1
35997	0	8.90	1	0.73347	.	.	1
35998	0	8.90	1	0.73347	.	.	1
35999	0	8.90	1	0.73347	.	.	1
36000	0	8.90	1	0.73347	.	.	1
36001	0	8.90	1	0.73347	.	.	1
36002	0	8.90	1	0.73347	.	.	1
36003	0	8.90	1	0.73347	.	.	1
36004	0	8.90	1	0.73347	.	.	1
36005	0	8.90	1	0.73347	.	.	1
36006	0	8.90	1	0.73347	.	.	1
36007	0	8.90	1	0.73347	.	.	1
36008	0	8.90	1	0.73347	.	.	1
36009	0	8.90	1	0.73347	.	.	1
36010	0	8.90	1	0.73347	.	.	1
36011	0	8.90	1	0.73347	.	.	1
36012	0	8.90	1	0.73347	.	.	1
36013	0	8.91	0	0.73305	0.72660	0.73937	1
36014	0	8.91	1	0.73305	.	.	1
36015	0	8.91	1	0.73305	.	.	1

Obs	Sub_group	fuptimey	_CENSOR_	SURVIVAL	SDF_LCL	SDF_UCL	STRATUM
36016	0	8.91	1	0.73305	.	.	1
36017	0	8.91	1	0.73305	.	.	1
36018	0	8.91	1	0.73305	.	.	1
36019	0	8.91	1	0.73305	.	.	1
36020	0	8.91	1	0.73305	.	.	1
36021	0	8.91	1	0.73305	.	.	1
36022	0	8.91	1	0.73305	.	.	1
36023	0	8.91	1	0.73305	.	.	1
36024	0	8.91	1	0.73305	.	.	1
36025	0	8.91	1	0.73305	.	.	1
36026	0	8.91	1	0.73305	.	.	1
36027	0	8.91	1	0.73305	.	.	1
36028	0	8.92	0	0.73219	0.72569	0.73856	1
36029	0	8.92	1	0.73219	.	.	1
36030	0	8.92	1	0.73219	.	.	1
36031	0	8.92	1	0.73219	.	.	1
36032	0	8.92	1	0.73219	.	.	1
36033	0	8.92	1	0.73219	.	.	1
36034	0	8.92	1	0.73219	.	.	1
36035	0	8.92	1	0.73219	.	.	1
36036	0	8.92	1	0.73219	.	.	1
36037	0	8.92	1	0.73219	.	.	1
36038	0	8.92	1	0.73219	.	.	1
36039	0	8.92	1	0.73219	.	.	1
36040	0	8.92	1	0.73219	.	.	1
36041	0	8.92	1	0.73219	.	.	1
36042	0	8.92	1	0.73219	.	.	1
36043	0	8.92	1	0.73219	.	.	1
36044	0	8.92	1	0.73219	.	.	1
36045	0	8.92	1	0.73219	.	.	1
36046	0	8.92	1	0.73219	.	.	1
36047	0	8.92	1	0.73219	.	.	1
36048	0	8.92	1	0.73219	.	.	1
36049	0	8.92	1	0.73219	.	.	1
36050	0	8.92	1	0.73219	.	.	1

Obs	Sub_group	fuptimey	_CENSOR_	SURVIVAL	SDF_LCL	SDF_UCL	STRATUM
36051	0	8.92	1	0.73219	.	.	1
36052	0	8.92	1	0.73219	.	.	1
36053	0	8.92	1	0.73219	.	.	1
36054	0	8.92	1	0.73219	.	.	1
36055	0	8.92	1	0.73219	.	.	1
36056	0	8.92	1	0.73219	.	.	1
36057	0	8.92	1	0.73219	.	.	1
36058	0	8.92	1	0.73219	.	.	1
36059	0	8.92	1	0.73219	.	.	1
36060	0	8.92	1	0.73219	.	.	1
36061	0	8.92	1	0.73219	.	.	1
36062	0	8.92	1	0.73219	.	.	1
36063	0	8.93	1	0.73219	.	.	1
36064	0	8.93	1	0.73219	.	.	1
36065	0	8.93	1	0.73219	.	.	1
36066	0	8.93	1	0.73219	.	.	1
36067	0	8.93	1	0.73219	.	.	1
36068	0	8.93	1	0.73219	.	.	1
36069	0	8.93	1	0.73219	.	.	1
36070	0	8.93	1	0.73219	.	.	1
36071	0	8.93	1	0.73219	.	.	1
36072	0	8.93	1	0.73219	.	.	1
36073	0	8.93	1	0.73219	.	.	1
36074	0	8.93	1	0.73219	.	.	1
36075	0	8.93	1	0.73219	.	.	1
36076	0	8.93	1	0.73219	.	.	1
36077	0	8.93	1	0.73219	.	.	1
36078	0	8.93	1	0.73219	.	.	1
36079	0	8.93	1	0.73219	.	.	1
36080	0	8.93	1	0.73219	.	.	1
36081	0	8.93	1	0.73219	.	.	1
36082	0	8.93	1	0.73219	.	.	1
36083	0	8.93	1	0.73219	.	.	1
36084	0	8.93	1	0.73219	.	.	1
36085	0	8.94	1	0.73219	.	.	1

Obs	Sub_group	fuptimey	_CENSOR_	SURVIVAL	SDF_LCL	SDF_UCL	STRATUM
36086	0	8.94	1	0.73219	.	.	1
36087	0	8.94	1	0.73219	.	.	1
36088	0	8.94	1	0.73219	.	.	1
36089	0	8.94	1	0.73219	.	.	1
36090	0	8.94	1	0.73219	.	.	1
36091	0	8.94	1	0.73219	.	.	1
36092	0	8.94	1	0.73219	.	.	1
36093	0	8.94	1	0.73219	.	.	1
36094	0	8.94	1	0.73219	.	.	1
36095	0	8.94	1	0.73219	.	.	1
36096	0	8.94	1	0.73219	.	.	1
36097	0	8.94	1	0.73219	.	.	1
36098	0	8.94	1	0.73219	.	.	1
36099	0	8.94	1	0.73219	.	.	1
36100	0	8.94	1	0.73219	.	.	1
36101	0	8.94	1	0.73219	.	.	1
36102	0	8.94	1	0.73219	.	.	1
36103	0	8.94	1	0.73219	.	.	1
36104	0	8.94	1	0.73219	.	.	1
36105	0	8.94	1	0.73219	.	.	1
36106	0	8.94	1	0.73219	.	.	1
36107	0	8.94	1	0.73219	.	.	1
36108	0	8.94	1	0.73219	.	.	1
36109	0	8.94	1	0.73219	.	.	1
36110	0	8.94	1	0.73219	.	.	1
36111	0	8.94	1	0.73219	.	.	1
36112	0	8.94	1	0.73219	.	.	1
36113	0	8.94	1	0.73219	.	.	1
36114	0	8.94	1	0.73219	.	.	1
36115	0	8.94	1	0.73219	.	.	1
36116	0	8.94	1	0.73219	.	.	1
36117	0	8.94	1	0.73219	.	.	1
36118	0	8.94	1	0.73219	.	.	1
36119	0	8.94	1	0.73219	.	.	1
36120	0	8.94	1	0.73219	.	.	1

Obs	Sub_group	fuptimey	_CENSOR_	SURVIVAL	SDF_LCL	SDF_UCL	STRATUM
36121	0	8.94	1	0.73219	.	.	1
36122	0	8.94	1	0.73219	.	.	1
36123	0	8.94	1	0.73219	.	.	1
36124	0	8.94	1	0.73219	.	.	1
36125	0	8.94	1	0.73219	.	.	1
36126	0	8.94	1	0.73219	.	.	1
36127	0	8.94	1	0.73219	.	.	1
36128	0	8.94	1	0.73219	.	.	1
36129	0	8.95	0	0.73197	0.72546	0.73835	1
36130	0	8.95	1	0.73197	.	.	1
36131	0	8.95	1	0.73197	.	.	1
36132	0	8.95	1	0.73197	.	.	1
36133	0	8.95	1	0.73197	.	.	1
36134	0	8.95	1	0.73197	.	.	1
36135	0	8.95	1	0.73197	.	.	1
36136	0	8.95	1	0.73197	.	.	1
36137	0	8.95	1	0.73197	.	.	1
36138	0	8.95	1	0.73197	.	.	1
36139	0	8.95	1	0.73197	.	.	1
36140	0	8.95	1	0.73197	.	.	1
36141	0	8.95	1	0.73197	.	.	1
36142	0	8.95	1	0.73197	.	.	1
36143	0	8.95	1	0.73197	.	.	1
36144	0	8.95	1	0.73197	.	.	1
36145	0	8.95	1	0.73197	.	.	1
36146	0	8.95	1	0.73197	.	.	1
36147	0	8.95	1	0.73197	.	.	1
36148	0	8.95	1	0.73197	.	.	1
36149	0	8.95	1	0.73197	.	.	1
36150	0	8.95	1	0.73197	.	.	1
36151	0	8.95	1	0.73197	.	.	1
36152	0	8.95	1	0.73197	.	.	1
36153	0	8.95	1	0.73197	.	.	1
36154	0	8.95	1	0.73197	.	.	1
36155	0	8.95	1	0.73197	.	.	1

Obs	Sub_group	fuptimey	_CENSOR_	SURVIVAL	SDF_LCL	SDF_UCL	STRATUM
36156	0	8.95	1	0.73197	.	.	1
36157	0	8.95	1	0.73197	.	.	1
36158	0	8.95	1	0.73197	.	.	1
36159	0	8.95	1	0.73197	.	.	1
36160	0	8.95	1	0.73197	.	.	1
36161	0	8.95	1	0.73197	.	.	1
36162	0	8.95	1	0.73197	.	.	1
36163	0	8.95	1	0.73197	.	.	1
36164	0	8.95	1	0.73197	.	.	1
36165	0	8.96	1	0.73197	.	.	1
36166	0	8.96	1	0.73197	.	.	1
36167	0	8.96	1	0.73197	.	.	1
36168	0	8.96	1	0.73197	.	.	1
36169	0	8.96	1	0.73197	.	.	1
36170	0	8.96	1	0.73197	.	.	1
36171	0	8.96	1	0.73197	.	.	1
36172	0	8.96	1	0.73197	.	.	1
36173	0	8.96	1	0.73197	.	.	1
36174	0	8.96	1	0.73197	.	.	1
36175	0	8.96	1	0.73197	.	.	1
36176	0	8.96	1	0.73197	.	.	1
36177	0	8.96	1	0.73197	.	.	1
36178	0	8.96	1	0.73197	.	.	1
36179	0	8.96	1	0.73197	.	.	1
36180	0	8.96	1	0.73197	.	.	1
36181	0	8.96	1	0.73197	.	.	1
36182	0	8.96	1	0.73197	.	.	1
36183	0	8.96	1	0.73197	.	.	1
36184	0	8.96	1	0.73197	.	.	1
36185	0	8.96	1	0.73197	.	.	1
36186	0	8.96	1	0.73197	.	.	1
36187	0	8.96	1	0.73197	.	.	1
36188	0	8.96	1	0.73197	.	.	1
36189	0	8.96	1	0.73197	.	.	1
36190	0	8.96	1	0.73197	.	.	1

Obs	Sub_group	fuptimey	_CENSOR_	SURVIVAL	SDF_LCL	SDF_UCL	STRATUM
36191	0	8.96	1	0.73197	.	.	1
36192	0	8.96	1	0.73197	.	.	1
36193	0	8.96	1	0.73197	.	.	1
36194	0	8.96	1	0.73197	.	.	1
36195	0	8.96	1	0.73197	.	.	1
36196	0	8.96	1	0.73197	.	.	1
36197	0	8.97	0	0.73107	0.72451	0.73751	1
36198	0	8.97	1	0.73107	.	.	1
36199	0	8.97	1	0.73107	.	.	1
36200	0	8.97	1	0.73107	.	.	1
36201	0	8.97	1	0.73107	.	.	1
36202	0	8.97	1	0.73107	.	.	1
36203	0	8.97	1	0.73107	.	.	1
36204	0	8.97	1	0.73107	.	.	1
36205	0	8.97	1	0.73107	.	.	1
36206	0	8.97	1	0.73107	.	.	1
36207	0	8.97	1	0.73107	.	.	1
36208	0	8.97	1	0.73107	.	.	1
36209	0	8.97	1	0.73107	.	.	1
36210	0	8.97	1	0.73107	.	.	1
36211	0	8.97	1	0.73107	.	.	1
36212	0	8.97	1	0.73107	.	.	1
36213	0	8.97	1	0.73107	.	.	1
36214	0	8.97	1	0.73107	.	.	1
36215	0	8.97	1	0.73107	.	.	1
36216	0	8.97	1	0.73107	.	.	1
36217	0	8.97	1	0.73107	.	.	1
36218	0	8.97	1	0.73107	.	.	1
36219	0	8.97	1	0.73107	.	.	1
36220	0	8.97	1	0.73107	.	.	1
36221	0	8.97	1	0.73107	.	.	1
36222	0	8.97	1	0.73107	.	.	1
36223	0	8.97	1	0.73107	.	.	1
36224	0	8.97	1	0.73107	.	.	1
36225	0	8.97	1	0.73107	.	.	1

Obs	Sub_group	fuptimey	_CENSOR_	SURVIVAL	SDF_LCL	SDF_UCL	STRATUM
36226	0	8.97	1	0.73107	.	.	1
36227	0	8.97	1	0.73107	.	.	1
36228	0	8.97	1	0.73107	.	.	1
36229	0	8.98	0	0.73061	0.72402	0.73708	1
36230	0	8.98	1	0.73061	.	.	1
36231	0	8.98	1	0.73061	.	.	1
36232	0	8.98	1	0.73061	.	.	1
36233	0	8.98	1	0.73061	.	.	1
36234	0	8.98	1	0.73061	.	.	1
36235	0	8.98	1	0.73061	.	.	1
36236	0	8.98	1	0.73061	.	.	1
36237	0	8.98	1	0.73061	.	.	1
36238	0	8.98	1	0.73061	.	.	1
36239	0	8.98	1	0.73061	.	.	1
36240	0	8.98	1	0.73061	.	.	1
36241	0	8.98	1	0.73061	.	.	1
36242	0	8.98	1	0.73061	.	.	1
36243	0	8.98	1	0.73061	.	.	1
36244	0	8.98	1	0.73061	.	.	1
36245	0	8.98	1	0.73061	.	.	1
36246	0	8.98	1	0.73061	.	.	1
36247	0	8.98	1	0.73061	.	.	1
36248	0	8.98	1	0.73061	.	.	1
36249	0	8.98	1	0.73061	.	.	1
36250	0	8.98	1	0.73061	.	.	1
36251	0	8.98	1	0.73061	.	.	1
36252	0	8.98	1	0.73061	.	.	1
36253	0	8.98	1	0.73061	.	.	1
36254	0	8.98	1	0.73061	.	.	1
36255	0	8.98	1	0.73061	.	.	1
36256	0	8.98	1	0.73061	.	.	1
36257	0	8.98	1	0.73061	.	.	1
36258	0	8.98	1	0.73061	.	.	1
36259	0	8.98	1	0.73061	.	.	1
36260	0	8.98	1	0.73061	.	.	1

Obs	Sub_group	fuptimey	_CENSOR_	SURVIVAL	SDF_LCL	SDF_UCL	STRATUM
36261	0	8.98	1	0.73061	.	.	1
36262	0	8.98	1	0.73061	.	.	1
36263	0	8.98	1	0.73061	.	.	1
36264	0	8.98	1	0.73061	.	.	1
36265	0	8.98	1	0.73061	.	.	1
36266	0	8.98	1	0.73061	.	.	1
36267	0	8.98	1	0.73061	.	.	1
36268	0	8.99	1	0.73061	.	.	1
36269	0	8.99	1	0.73061	.	.	1
36270	0	8.99	1	0.73061	.	.	1
36271	0	8.99	1	0.73061	.	.	1
36272	0	8.99	1	0.73061	.	.	1
36273	0	8.99	1	0.73061	.	.	1
36274	0	8.99	1	0.73061	.	.	1
36275	0	8.99	1	0.73061	.	.	1
36276	0	8.99	1	0.73061	.	.	1
36277	0	8.99	1	0.73061	.	.	1
36278	0	8.99	1	0.73061	.	.	1
36279	0	8.99	1	0.73061	.	.	1
36280	0	8.99	1	0.73061	.	.	1
36281	0	8.99	1	0.73061	.	.	1
36282	0	8.99	1	0.73061	.	.	1
36283	0	8.99	1	0.73061	.	.	1
36284	0	8.99	1	0.73061	.	.	1
36285	0	8.99	1	0.73061	.	.	1
36286	0	8.99	1	0.73061	.	.	1
36287	0	8.99	1	0.73061	.	.	1
36288	0	8.99	1	0.73061	.	.	1
36289	0	8.99	1	0.73061	.	.	1
36290	0	8.99	1	0.73061	.	.	1
36291	0	8.99	1	0.73061	.	.	1
36292	0	8.99	1	0.73061	.	.	1
36293	0	8.99	1	0.73061	.	.	1
36294	0	8.99	1	0.73061	.	.	1
36295	0	8.99	1	0.73061	.	.	1

Obs	Sub_group	fuptimey	_CENSOR_	SURVIVAL	SDF_LCL	SDF_UCL	STRATUM
36296	0	9.00	0	0.73038	0.72378	0.73686	1
36297	0	9.00	1	0.73038	.	.	1
36298	0	9.00	1	0.73038	.	.	1
36299	0	9.00	1	0.73038	.	.	1
36300	0	9.00	1	0.73038	.	.	1
36301	0	9.00	1	0.73038	.	.	1
36302	0	9.00	1	0.73038	.	.	1
36303	0	9.00	1	0.73038	.	.	1
36304	0	9.00	1	0.73038	.	.	1
36305	0	9.00	1	0.73038	.	.	1
36306	0	9.00	1	0.73038	.	.	1
36307	0	9.00	1	0.73038	.	.	1
36308	0	9.00	1	0.73038	.	.	1
36309	0	9.00	1	0.73038	.	.	1
36310	0	9.00	1	0.73038	.	.	1
36311	0	9.00	1	0.73038	.	.	1
36312	0	9.00	1	0.73038	.	.	1
36313	0	9.00	1	0.73038	.	.	1
36314	0	9.00	1	0.73038	.	.	1
36315	0	9.00	1	0.73038	.	.	1
36316	0	9.00	1	0.73038	.	.	1
36317	0	9.00	1	0.73038	.	.	1
36318	0	9.00	1	0.73038	.	.	1
36319	0	9.00	1	0.73038	.	.	1
36320	0	9.00	1	0.73038	.	.	1
36321	0	9.00	1	0.73038	.	.	1
36322	0	9.00	1	0.73038	.	.	1
36323	0	9.00	1	0.73038	.	.	1
36324	0	9.00	1	0.73038	.	.	1
36325	0	9.00	1	0.73038	.	.	1
36326	0	9.00	1	0.73038	.	.	1
36327	0	9.00	1	0.73038	.	.	1
36328	0	9.00	1	0.73038	.	.	1
36329	0	9.00	1	0.73038	.	.	1
36330	0	9.00	1	0.73038	.	.	1

Obs	Sub_group	fuptimey	_CENSOR_	SURVIVAL	SDF_LCL	SDF_UCL	STRATUM
36331	0	9.00	1	0.73038	.	.	1
36332	0	9.00	1	0.73038	.	.	1
36333	0	9.00	1	0.73038	.	.	1
36334	0	9.00	1	0.73038	.	.	1
36335	0	9.00	1	0.73038	.	.	1
36336	0	9.00	1	0.73038	.	.	1
36337	0	9.00	1	0.73038	.	.	1
36338	0	9.00	1	0.73038	.	.	1
36339	0	9.00	1	0.73038	.	.	1
36340	0	9.00	1	0.73038	.	.	1
36341	0	9.00	1	0.73038	.	.	1
36342	0	9.00	1	0.73038	.	.	1
36343	0	9.00	1	0.73038	.	.	1
36344	0	9.01	0	0.73015	0.72353	0.73664	1
36345	0	9.01	1	0.73015	.	.	1
36346	0	9.01	1	0.73015	.	.	1
36347	0	9.01	1	0.73015	.	.	1
36348	0	9.01	1	0.73015	.	.	1
36349	0	9.01	1	0.73015	.	.	1
36350	0	9.01	1	0.73015	.	.	1
36351	0	9.01	1	0.73015	.	.	1
36352	0	9.01	1	0.73015	.	.	1
36353	0	9.01	1	0.73015	.	.	1
36354	0	9.01	1	0.73015	.	.	1
36355	0	9.01	1	0.73015	.	.	1
36356	0	9.01	1	0.73015	.	.	1
36357	0	9.01	1	0.73015	.	.	1
36358	0	9.01	1	0.73015	.	.	1
36359	0	9.01	1	0.73015	.	.	1
36360	0	9.01	1	0.73015	.	.	1
36361	0	9.01	1	0.73015	.	.	1
36362	0	9.01	1	0.73015	.	.	1
36363	0	9.01	1	0.73015	.	.	1
36364	0	9.01	1	0.73015	.	.	1
36365	0	9.01	1	0.73015	.	.	1

Obs	Sub_group	fuptimey	_CENSOR_	SURVIVAL	SDF_LCL	SDF_UCL	STRATUM
36366	0	9.01	1	0.73015	.	.	1
36367	0	9.01	1	0.73015	.	.	1
36368	0	9.01	1	0.73015	.	.	1
36369	0	9.01	1	0.73015	.	.	1
36370	0	9.01	1	0.73015	.	.	1
36371	0	9.01	1	0.73015	.	.	1
36372	0	9.01	1	0.73015	.	.	1
36373	0	9.01	1	0.73015	.	.	1
36374	0	9.01	1	0.73015	.	.	1
36375	0	9.01	1	0.73015	.	.	1
36376	0	9.01	1	0.73015	.	.	1
36377	0	9.01	1	0.73015	.	.	1
36378	0	9.02	0	0.72991	0.72328	0.73641	1
36379	0	9.02	1	0.72991	.	.	1
36380	0	9.02	1	0.72991	.	.	1
36381	0	9.02	1	0.72991	.	.	1
36382	0	9.02	1	0.72991	.	.	1
36383	0	9.02	1	0.72991	.	.	1
36384	0	9.02	1	0.72991	.	.	1
36385	0	9.02	1	0.72991	.	.	1
36386	0	9.02	1	0.72991	.	.	1
36387	0	9.02	1	0.72991	.	.	1
36388	0	9.02	1	0.72991	.	.	1
36389	0	9.02	1	0.72991	.	.	1
36390	0	9.02	1	0.72991	.	.	1
36391	0	9.02	1	0.72991	.	.	1
36392	0	9.02	1	0.72991	.	.	1
36393	0	9.02	1	0.72991	.	.	1
36394	0	9.02	1	0.72991	.	.	1
36395	0	9.02	1	0.72991	.	.	1
36396	0	9.02	1	0.72991	.	.	1
36397	0	9.02	1	0.72991	.	.	1
36398	0	9.02	1	0.72991	.	.	1
36399	0	9.02	1	0.72991	.	.	1
36400	0	9.02	1	0.72991	.	.	1

Obs	Sub_group	fuptimey	_CENSOR_	SURVIVAL	SDF_LCL	SDF_UCL	STRATUM
36401	0	9.02	1	0.72991	.	.	1
36402	0	9.02	1	0.72991	.	.	1
36403	0	9.02	1	0.72991	.	.	1
36404	0	9.02	1	0.72991	.	.	1
36405	0	9.02	1	0.72991	.	.	1
36406	0	9.03	0	0.72967	0.72302	0.73619	1
36407	0	9.03	1	0.72967	.	.	1
36408	0	9.03	1	0.72967	.	.	1
36409	0	9.03	1	0.72967	.	.	1
36410	0	9.03	1	0.72967	.	.	1
36411	0	9.03	1	0.72967	.	.	1
36412	0	9.03	1	0.72967	.	.	1
36413	0	9.03	1	0.72967	.	.	1
36414	0	9.03	1	0.72967	.	.	1
36415	0	9.03	1	0.72967	.	.	1
36416	0	9.03	1	0.72967	.	.	1
36417	0	9.03	1	0.72967	.	.	1
36418	0	9.03	1	0.72967	.	.	1
36419	0	9.03	1	0.72967	.	.	1
36420	0	9.03	1	0.72967	.	.	1
36421	0	9.03	1	0.72967	.	.	1
36422	0	9.03	1	0.72967	.	.	1
36423	0	9.03	1	0.72967	.	.	1
36424	0	9.03	1	0.72967	.	.	1
36425	0	9.03	1	0.72967	.	.	1
36426	0	9.03	1	0.72967	.	.	1
36427	0	9.03	1	0.72967	.	.	1
36428	0	9.03	1	0.72967	.	.	1
36429	0	9.03	1	0.72967	.	.	1
36430	0	9.03	1	0.72967	.	.	1
36431	0	9.03	1	0.72967	.	.	1
36432	0	9.03	1	0.72967	.	.	1
36433	0	9.04	0	0.72943	0.72276	0.73596	1
36434	0	9.04	1	0.72943	.	.	1
36435	0	9.04	1	0.72943	.	.	1

Obs	Sub_group	fuptimey	_CENSOR_	SURVIVAL	SDF_LCL	SDF_UCL	STRATUM
36436	0	9.04	1	0.72943	.	.	1
36437	0	9.04	1	0.72943	.	.	1
36438	0	9.04	1	0.72943	.	.	1
36439	0	9.04	1	0.72943	.	.	1
36440	0	9.04	1	0.72943	.	.	1
36441	0	9.04	1	0.72943	.	.	1
36442	0	9.04	1	0.72943	.	.	1
36443	0	9.04	1	0.72943	.	.	1
36444	0	9.04	1	0.72943	.	.	1
36445	0	9.04	1	0.72943	.	.	1
36446	0	9.04	1	0.72943	.	.	1
36447	0	9.04	1	0.72943	.	.	1
36448	0	9.04	1	0.72943	.	.	1
36449	0	9.04	1	0.72943	.	.	1
36450	0	9.04	1	0.72943	.	.	1
36451	0	9.04	1	0.72943	.	.	1
36452	0	9.04	1	0.72943	.	.	1
36453	0	9.04	1	0.72943	.	.	1
36454	0	9.04	1	0.72943	.	.	1
36455	0	9.04	1	0.72943	.	.	1
36456	0	9.04	1	0.72943	.	.	1
36457	0	9.04	1	0.72943	.	.	1
36458	0	9.04	1	0.72943	.	.	1
36459	0	9.04	1	0.72943	.	.	1
36460	0	9.04	1	0.72943	.	.	1
36461	0	9.04	1	0.72943	.	.	1
36462	0	9.04	1	0.72943	.	.	1
36463	0	9.04	1	0.72943	.	.	1
36464	0	9.05	1	0.72943	.	.	1
36465	0	9.05	1	0.72943	.	.	1
36466	0	9.05	1	0.72943	.	.	1
36467	0	9.05	1	0.72943	.	.	1
36468	0	9.05	1	0.72943	.	.	1
36469	0	9.05	1	0.72943	.	.	1
36470	0	9.05	1	0.72943	.	.	1

Obs	Sub_group	fuptimey	_CENSOR_	SURVIVAL	SDF_LCL	SDF_UCL	STRATUM
36471	0	9.05	1	0.72943	.	.	1
36472	0	9.05	1	0.72943	.	.	1
36473	0	9.05	1	0.72943	.	.	1
36474	0	9.05	1	0.72943	.	.	1
36475	0	9.05	1	0.72943	.	.	1
36476	0	9.05	1	0.72943	.	.	1
36477	0	9.05	1	0.72943	.	.	1
36478	0	9.05	1	0.72943	.	.	1
36479	0	9.05	1	0.72943	.	.	1
36480	0	9.05	1	0.72943	.	.	1
36481	0	9.05	1	0.72943	.	.	1
36482	0	9.05	1	0.72943	.	.	1
36483	0	9.05	1	0.72943	.	.	1
36484	0	9.05	1	0.72943	.	.	1
36485	0	9.05	1	0.72943	.	.	1
36486	0	9.05	1	0.72943	.	.	1
36487	0	9.05	1	0.72943	.	.	1
36488	0	9.05	1	0.72943	.	.	1
36489	0	9.05	1	0.72943	.	.	1
36490	0	9.05	1	0.72943	.	.	1
36491	0	9.05	1	0.72943	.	.	1
36492	0	9.06	1	0.72943	.	.	1
36493	0	9.06	1	0.72943	.	.	1
36494	0	9.06	1	0.72943	.	.	1
36495	0	9.06	1	0.72943	.	.	1
36496	0	9.06	1	0.72943	.	.	1
36497	0	9.06	1	0.72943	.	.	1
36498	0	9.06	1	0.72943	.	.	1
36499	0	9.06	1	0.72943	.	.	1
36500	0	9.06	1	0.72943	.	.	1
36501	0	9.06	1	0.72943	.	.	1
36502	0	9.06	1	0.72943	.	.	1
36503	0	9.06	1	0.72943	.	.	1
36504	0	9.06	1	0.72943	.	.	1
36505	0	9.06	1	0.72943	.	.	1

Obs	Sub_group	fuptimey	_CENSOR_	SURVIVAL	SDF_LCL	SDF_UCL	STRATUM
36506	0	9.06	1	0.72943	.	.	1
36507	0	9.06	1	0.72943	.	.	1
36508	0	9.06	1	0.72943	.	.	1
36509	0	9.06	1	0.72943	.	.	1
36510	0	9.06	1	0.72943	.	.	1
36511	0	9.06	1	0.72943	.	.	1
36512	0	9.06	1	0.72943	.	.	1
36513	0	9.06	1	0.72943	.	.	1
36514	0	9.06	1	0.72943	.	.	1
36515	0	9.07	0	0.72868	0.72197	0.73526	1
36516	0	9.07	1	0.72868	.	.	1
36517	0	9.07	1	0.72868	.	.	1
36518	0	9.07	1	0.72868	.	.	1
36519	0	9.07	1	0.72868	.	.	1
36520	0	9.07	1	0.72868	.	.	1
36521	0	9.07	1	0.72868	.	.	1
36522	0	9.07	1	0.72868	.	.	1
36523	0	9.07	1	0.72868	.	.	1
36524	0	9.07	1	0.72868	.	.	1
36525	0	9.07	1	0.72868	.	.	1
36526	0	9.07	1	0.72868	.	.	1
36527	0	9.07	1	0.72868	.	.	1
36528	0	9.07	1	0.72868	.	.	1
36529	0	9.07	1	0.72868	.	.	1
36530	0	9.07	1	0.72868	.	.	1
36531	0	9.07	1	0.72868	.	.	1
36532	0	9.07	1	0.72868	.	.	1
36533	0	9.07	1	0.72868	.	.	1
36534	0	9.07	1	0.72868	.	.	1
36535	0	9.07	1	0.72868	.	.	1
36536	0	9.07	1	0.72868	.	.	1
36537	0	9.07	1	0.72868	.	.	1
36538	0	9.07	1	0.72868	.	.	1
36539	0	9.07	1	0.72868	.	.	1
36540	0	9.07	1	0.72868	.	.	1

Obs	Sub_group	fuptimey	_CENSOR_	SURVIVAL	SDF_LCL	SDF_UCL	STRATUM
36541	0	9.08	0	0.72843	0.72170	0.73502	1
36542	0	9.08	1	0.72843	.	.	1
36543	0	9.08	1	0.72843	.	.	1
36544	0	9.08	1	0.72843	.	.	1
36545	0	9.08	1	0.72843	.	.	1
36546	0	9.08	1	0.72843	.	.	1
36547	0	9.08	1	0.72843	.	.	1
36548	0	9.08	1	0.72843	.	.	1
36549	0	9.08	1	0.72843	.	.	1
36550	0	9.08	1	0.72843	.	.	1
36551	0	9.08	1	0.72843	.	.	1
36552	0	9.08	1	0.72843	.	.	1
36553	0	9.08	1	0.72843	.	.	1
36554	0	9.08	1	0.72843	.	.	1
36555	0	9.08	1	0.72843	.	.	1
36556	0	9.08	1	0.72843	.	.	1
36557	0	9.08	1	0.72843	.	.	1
36558	0	9.08	1	0.72843	.	.	1
36559	0	9.08	1	0.72843	.	.	1
36560	0	9.08	1	0.72843	.	.	1
36561	0	9.09	0	0.72818	0.72143	0.73479	1
36562	0	9.09	1	0.72818	.	.	1
36563	0	9.09	1	0.72818	.	.	1
36564	0	9.09	1	0.72818	.	.	1
36565	0	9.09	1	0.72818	.	.	1
36566	0	9.09	1	0.72818	.	.	1
36567	0	9.09	1	0.72818	.	.	1
36568	0	9.09	1	0.72818	.	.	1
36569	0	9.09	1	0.72818	.	.	1
36570	0	9.09	1	0.72818	.	.	1
36571	0	9.09	1	0.72818	.	.	1
36572	0	9.09	1	0.72818	.	.	1
36573	0	9.09	1	0.72818	.	.	1
36574	0	9.09	1	0.72818	.	.	1
36575	0	9.09	1	0.72818	.	.	1

Obs	Sub_group	fuptimey	_CENSOR_	SURVIVAL	SDF_LCL	SDF_UCL	STRATUM
36576	0	9.09	1	0.72818	.	.	1
36577	0	9.09	1	0.72818	.	.	1
36578	0	9.09	1	0.72818	.	.	1
36579	0	9.09	1	0.72818	.	.	1
36580	0	9.09	1	0.72818	.	.	1
36581	0	9.09	1	0.72818	.	.	1
36582	0	9.09	1	0.72818	.	.	1
36583	0	9.09	1	0.72818	.	.	1
36584	0	9.09	1	0.72818	.	.	1
36585	0	9.09	1	0.72818	.	.	1
36586	0	9.09	1	0.72818	.	.	1
36587	0	9.09	1	0.72818	.	.	1
36588	0	9.09	1	0.72818	.	.	1
36589	0	9.09	1	0.72818	.	.	1
36590	0	9.09	1	0.72818	.	.	1
36591	0	9.09	1	0.72818	.	.	1
36592	0	9.09	1	0.72818	.	.	1
36593	0	9.09	1	0.72818	.	.	1
36594	0	9.09	1	0.72818	.	.	1
36595	0	9.10	0	0.72766	0.72089	0.73431	1
36596	0	9.10	1	0.72766	.	.	1
36597	0	9.10	1	0.72766	.	.	1
36598	0	9.10	1	0.72766	.	.	1
36599	0	9.10	1	0.72766	.	.	1
36600	0	9.10	1	0.72766	.	.	1
36601	0	9.10	1	0.72766	.	.	1
36602	0	9.10	1	0.72766	.	.	1
36603	0	9.10	1	0.72766	.	.	1
36604	0	9.10	1	0.72766	.	.	1
36605	0	9.10	1	0.72766	.	.	1
36606	0	9.10	1	0.72766	.	.	1
36607	0	9.10	1	0.72766	.	.	1
36608	0	9.10	1	0.72766	.	.	1
36609	0	9.10	1	0.72766	.	.	1
36610	0	9.10	1	0.72766	.	.	1

Obs	Sub_group	fuptimey	_CENSOR_	SURVIVAL	SDF_LCL	SDF_UCL	STRATUM
36611	0	9.10	1	0.72766	.	.	1
36612	0	9.10	1	0.72766	.	.	1
36613	0	9.10	1	0.72766	.	.	1
36614	0	9.10	1	0.72766	.	.	1
36615	0	9.10	1	0.72766	.	.	1
36616	0	9.10	1	0.72766	.	.	1
36617	0	9.10	1	0.72766	.	.	1
36618	0	9.10	1	0.72766	.	.	1
36619	0	9.10	1	0.72766	.	.	1
36620	0	9.10	1	0.72766	.	.	1
36621	0	9.10	1	0.72766	.	.	1
36622	0	9.10	1	0.72766	.	.	1
36623	0	9.11	0	0.72715	0.72034	0.73382	1
36624	0	9.11	1	0.72715	.	.	1
36625	0	9.11	1	0.72715	.	.	1
36626	0	9.11	1	0.72715	.	.	1
36627	0	9.11	1	0.72715	.	.	1
36628	0	9.11	1	0.72715	.	.	1
36629	0	9.11	1	0.72715	.	.	1
36630	0	9.11	1	0.72715	.	.	1
36631	0	9.11	1	0.72715	.	.	1
36632	0	9.11	1	0.72715	.	.	1
36633	0	9.11	1	0.72715	.	.	1
36634	0	9.11	1	0.72715	.	.	1
36635	0	9.11	1	0.72715	.	.	1
36636	0	9.11	1	0.72715	.	.	1
36637	0	9.11	1	0.72715	.	.	1
36638	0	9.11	1	0.72715	.	.	1
36639	0	9.11	1	0.72715	.	.	1
36640	0	9.11	1	0.72715	.	.	1
36641	0	9.11	1	0.72715	.	.	1
36642	0	9.11	1	0.72715	.	.	1
36643	0	9.11	1	0.72715	.	.	1
36644	0	9.11	1	0.72715	.	.	1
36645	0	9.11	1	0.72715	.	.	1

Obs	Sub_group	fuptimey	_CENSOR_	SURVIVAL	SDF_LCL	SDF_UCL	STRATUM
36646	0	9.11	1	0.72715	.	.	1
36647	0	9.11	1	0.72715	.	.	1
36648	0	9.11	1	0.72715	.	.	1
36649	0	9.11	1	0.72715	.	.	1
36650	0	9.11	1	0.72715	.	.	1
36651	0	9.11	1	0.72715	.	.	1
36652	0	9.11	1	0.72715	.	.	1
36653	0	9.11	1	0.72715	.	.	1
36654	0	9.11	1	0.72715	.	.	1
36655	0	9.11	1	0.72715	.	.	1
36656	0	9.12	0	0.72663	0.71978	0.73334	1
36657	0	9.12	1	0.72663	.	.	1
36658	0	9.12	1	0.72663	.	.	1
36659	0	9.12	1	0.72663	.	.	1
36660	0	9.12	1	0.72663	.	.	1
36661	0	9.12	1	0.72663	.	.	1
36662	0	9.12	1	0.72663	.	.	1
36663	0	9.12	1	0.72663	.	.	1
36664	0	9.12	1	0.72663	.	.	1
36665	0	9.12	1	0.72663	.	.	1
36666	0	9.12	1	0.72663	.	.	1
36667	0	9.12	1	0.72663	.	.	1
36668	0	9.12	1	0.72663	.	.	1
36669	0	9.12	1	0.72663	.	.	1
36670	0	9.12	1	0.72663	.	.	1
36671	0	9.12	1	0.72663	.	.	1
36672	0	9.12	1	0.72663	.	.	1
36673	0	9.12	1	0.72663	.	.	1
36674	0	9.12	1	0.72663	.	.	1
36675	0	9.12	1	0.72663	.	.	1
36676	0	9.12	1	0.72663	.	.	1
36677	0	9.12	1	0.72663	.	.	1
36678	0	9.12	1	0.72663	.	.	1
36679	0	9.12	1	0.72663	.	.	1
36680	0	9.12	1	0.72663	.	.	1

Obs	Sub_group	fuptimey	_CENSOR_	SURVIVAL	SDF_LCL	SDF_UCL	STRATUM
36681	0	9.12	1	0.72663	.	.	1
36682	0	9.12	1	0.72663	.	.	1
36683	0	9.12	1	0.72663	.	.	1
36684	0	9.12	1	0.72663	.	.	1
36685	0	9.12	1	0.72663	.	.	1
36686	0	9.12	1	0.72663	.	.	1
36687	0	9.13	0	0.72610	0.71922	0.73284	1
36688	0	9.13	1	0.72610	.	.	1
36689	0	9.13	1	0.72610	.	.	1
36690	0	9.13	1	0.72610	.	.	1
36691	0	9.13	1	0.72610	.	.	1
36692	0	9.13	1	0.72610	.	.	1
36693	0	9.13	1	0.72610	.	.	1
36694	0	9.13	1	0.72610	.	.	1
36695	0	9.13	1	0.72610	.	.	1
36696	0	9.13	1	0.72610	.	.	1
36697	0	9.13	1	0.72610	.	.	1
36698	0	9.13	1	0.72610	.	.	1
36699	0	9.13	1	0.72610	.	.	1
36700	0	9.13	1	0.72610	.	.	1
36701	0	9.13	1	0.72610	.	.	1
36702	0	9.13	1	0.72610	.	.	1
36703	0	9.13	1	0.72610	.	.	1
36704	0	9.13	1	0.72610	.	.	1
36705	0	9.13	1	0.72610	.	.	1
36706	0	9.13	1	0.72610	.	.	1
36707	0	9.13	1	0.72610	.	.	1
36708	0	9.13	1	0.72610	.	.	1
36709	0	9.13	1	0.72610	.	.	1
36710	0	9.13	1	0.72610	.	.	1
36711	0	9.13	1	0.72610	.	.	1
36712	0	9.13	1	0.72610	.	.	1
36713	0	9.13	1	0.72610	.	.	1
36714	0	9.13	1	0.72610	.	.	1
36715	0	9.13	1	0.72610	.	.	1

Obs	Sub_group	fuptimey	_CENSOR_	SURVIVAL	SDF_LCL	SDF_UCL	STRATUM
36716	0	9.13	1	0.72610	.	.	1
36717	0	9.13	1	0.72610	.	.	1
36718	0	9.13	1	0.72610	.	.	1
36719	0	9.13	1	0.72610	.	.	1
36720	0	9.13	1	0.72610	.	.	1
36721	0	9.14	0	0.72583	0.71893	0.73259	1
36722	0	9.14	1	0.72583	.	.	1
36723	0	9.14	1	0.72583	.	.	1
36724	0	9.14	1	0.72583	.	.	1
36725	0	9.14	1	0.72583	.	.	1
36726	0	9.14	1	0.72583	.	.	1
36727	0	9.14	1	0.72583	.	.	1
36728	0	9.14	1	0.72583	.	.	1
36729	0	9.14	1	0.72583	.	.	1
36730	0	9.14	1	0.72583	.	.	1
36731	0	9.14	1	0.72583	.	.	1
36732	0	9.14	1	0.72583	.	.	1
36733	0	9.14	1	0.72583	.	.	1
36734	0	9.14	1	0.72583	.	.	1
36735	0	9.14	1	0.72583	.	.	1
36736	0	9.14	1	0.72583	.	.	1
36737	0	9.14	1	0.72583	.	.	1
36738	0	9.14	1	0.72583	.	.	1
36739	0	9.14	1	0.72583	.	.	1
36740	0	9.14	1	0.72583	.	.	1
36741	0	9.14	1	0.72583	.	.	1
36742	0	9.14	1	0.72583	.	.	1
36743	0	9.14	1	0.72583	.	.	1
36744	0	9.14	1	0.72583	.	.	1
36745	0	9.14	1	0.72583	.	.	1
36746	0	9.14	1	0.72583	.	.	1
36747	0	9.15	0	0.72556	0.71865	0.73234	1
36748	0	9.15	1	0.72556	.	.	1
36749	0	9.15	1	0.72556	.	.	1
36750	0	9.15	1	0.72556	.	.	1

Obs	Sub_group	fuptimey	_CENSOR_	SURVIVAL	SDF_LCL	SDF_UCL	STRATUM
36751	0	9.15	1	0.72556	.	.	1
36752	0	9.15	1	0.72556	.	.	1
36753	0	9.15	1	0.72556	.	.	1
36754	0	9.15	1	0.72556	.	.	1
36755	0	9.15	1	0.72556	.	.	1
36756	0	9.15	1	0.72556	.	.	1
36757	0	9.15	1	0.72556	.	.	1
36758	0	9.15	1	0.72556	.	.	1
36759	0	9.15	1	0.72556	.	.	1
36760	0	9.15	1	0.72556	.	.	1
36761	0	9.15	1	0.72556	.	.	1
36762	0	9.15	1	0.72556	.	.	1
36763	0	9.15	1	0.72556	.	.	1
36764	0	9.15	1	0.72556	.	.	1
36765	0	9.15	1	0.72556	.	.	1
36766	0	9.15	1	0.72556	.	.	1
36767	0	9.15	1	0.72556	.	.	1
36768	0	9.15	1	0.72556	.	.	1
36769	0	9.15	1	0.72556	.	.	1
36770	0	9.15	1	0.72556	.	.	1
36771	0	9.15	1	0.72556	.	.	1
36772	0	9.15	1	0.72556	.	.	1
36773	0	9.15	1	0.72556	.	.	1
36774	0	9.15	1	0.72556	.	.	1
36775	0	9.15	1	0.72556	.	.	1
36776	0	9.15	1	0.72556	.	.	1
36777	0	9.15	1	0.72556	.	.	1
36778	0	9.15	1	0.72556	.	.	1
36779	0	9.15	1	0.72556	.	.	1
36780	0	9.15	1	0.72556	.	.	1
36781	0	9.15	1	0.72556	.	.	1
36782	0	9.15	1	0.72556	.	.	1
36783	0	9.15	1	0.72556	.	.	1
36784	0	9.15	1	0.72556	.	.	1
36785	0	9.15	1	0.72556	.	.	1

Obs	Sub_group	fuptimey	_CENSOR_	SURVIVAL	SDF_LCL	SDF_UCL	STRATUM
36786	0	9.15	1	0.72556	.	.	1
36787	0	9.15	1	0.72556	.	.	1
36788	0	9.15	1	0.72556	.	.	1
36789	0	9.15	1	0.72556	.	.	1
36790	0	9.15	1	0.72556	.	.	1
36791	0	9.15	1	0.72556	.	.	1
36792	0	9.15	1	0.72556	.	.	1
36793	0	9.15	1	0.72556	.	.	1
36794	0	9.16	0	0.72529	0.71835	0.73208	1
36795	0	9.16	1	0.72529	.	.	1
36796	0	9.16	1	0.72529	.	.	1
36797	0	9.16	1	0.72529	.	.	1
36798	0	9.16	1	0.72529	.	.	1
36799	0	9.16	1	0.72529	.	.	1
36800	0	9.16	1	0.72529	.	.	1
36801	0	9.16	1	0.72529	.	.	1
36802	0	9.16	1	0.72529	.	.	1
36803	0	9.16	1	0.72529	.	.	1
36804	0	9.16	1	0.72529	.	.	1
36805	0	9.16	1	0.72529	.	.	1
36806	0	9.16	1	0.72529	.	.	1
36807	0	9.16	1	0.72529	.	.	1
36808	0	9.16	1	0.72529	.	.	1
36809	0	9.16	1	0.72529	.	.	1
36810	0	9.16	1	0.72529	.	.	1
36811	0	9.16	1	0.72529	.	.	1
36812	0	9.16	1	0.72529	.	.	1
36813	0	9.16	1	0.72529	.	.	1
36814	0	9.16	1	0.72529	.	.	1
36815	0	9.16	1	0.72529	.	.	1
36816	0	9.16	1	0.72529	.	.	1
36817	0	9.16	1	0.72529	.	.	1
36818	0	9.17	1	0.72529	.	.	1
36819	0	9.17	1	0.72529	.	.	1
36820	0	9.17	1	0.72529	.	.	1

Obs	Sub_group	fuptimey	_CENSOR_	SURVIVAL	SDF_LCL	SDF_UCL	STRATUM
36821	0	9.17	1	0.72529	.	.	1
36822	0	9.17	1	0.72529	.	.	1
36823	0	9.17	1	0.72529	.	.	1
36824	0	9.17	1	0.72529	.	.	1
36825	0	9.17	1	0.72529	.	.	1
36826	0	9.17	1	0.72529	.	.	1
36827	0	9.17	1	0.72529	.	.	1
36828	0	9.17	1	0.72529	.	.	1
36829	0	9.17	1	0.72529	.	.	1
36830	0	9.17	1	0.72529	.	.	1
36831	0	9.17	1	0.72529	.	.	1
36832	0	9.17	1	0.72529	.	.	1
36833	0	9.17	1	0.72529	.	.	1
36834	0	9.17	1	0.72529	.	.	1
36835	0	9.17	1	0.72529	.	.	1
36836	0	9.17	1	0.72529	.	.	1
36837	0	9.17	1	0.72529	.	.	1
36838	0	9.17	1	0.72529	.	.	1
36839	0	9.17	1	0.72529	.	.	1
36840	0	9.17	1	0.72529	.	.	1
36841	0	9.17	1	0.72529	.	.	1
36842	0	9.17	1	0.72529	.	.	1
36843	0	9.17	1	0.72529	.	.	1
36844	0	9.17	1	0.72529	.	.	1
36845	0	9.17	1	0.72529	.	.	1
36846	0	9.17	1	0.72529	.	.	1
36847	0	9.17	1	0.72529	.	.	1
36848	0	9.17	1	0.72529	.	.	1
36849	0	9.17	1	0.72529	.	.	1
36850	0	9.17	1	0.72529	.	.	1
36851	0	9.17	1	0.72529	.	.	1
36852	0	9.17	1	0.72529	.	.	1
36853	0	9.17	1	0.72529	.	.	1
36854	0	9.17	1	0.72529	.	.	1
36855	0	9.17	1	0.72529	.	.	1

Obs	Sub_group	fuptimey	_CENSOR_	SURVIVAL	SDF_LCL	SDF_UCL	STRATUM
36856	0	9.17	1	0.72529	.	.	1
36857	0	9.17	1	0.72529	.	.	1
36858	0	9.18	0	0.72501	0.71805	0.73182	1
36859	0	9.18	1	0.72501	.	.	1
36860	0	9.18	1	0.72501	.	.	1
36861	0	9.18	1	0.72501	.	.	1
36862	0	9.18	1	0.72501	.	.	1
36863	0	9.18	1	0.72501	.	.	1
36864	0	9.18	1	0.72501	.	.	1
36865	0	9.18	1	0.72501	.	.	1
36866	0	9.18	1	0.72501	.	.	1
36867	0	9.18	1	0.72501	.	.	1
36868	0	9.18	1	0.72501	.	.	1
36869	0	9.18	1	0.72501	.	.	1
36870	0	9.18	1	0.72501	.	.	1
36871	0	9.18	1	0.72501	.	.	1
36872	0	9.18	1	0.72501	.	.	1
36873	0	9.18	1	0.72501	.	.	1
36874	0	9.18	1	0.72501	.	.	1
36875	0	9.18	1	0.72501	.	.	1
36876	0	9.18	1	0.72501	.	.	1
36877	0	9.18	1	0.72501	.	.	1
36878	0	9.18	1	0.72501	.	.	1
36879	0	9.18	1	0.72501	.	.	1
36880	0	9.18	1	0.72501	.	.	1
36881	0	9.18	1	0.72501	.	.	1
36882	0	9.18	1	0.72501	.	.	1
36883	0	9.18	1	0.72501	.	.	1
36884	0	9.18	1	0.72501	.	.	1
36885	0	9.18	1	0.72501	.	.	1
36886	0	9.18	1	0.72501	.	.	1
36887	0	9.18	1	0.72501	.	.	1
36888	0	9.18	1	0.72501	.	.	1
36889	0	9.18	1	0.72501	.	.	1
36890	0	9.18	1	0.72501	.	.	1

Obs	Sub_group	fuptimey	_CENSOR_	SURVIVAL	SDF_LCL	SDF_UCL	STRATUM
36891	0	9.19	0	0.72472	0.71775	0.73156	1
36892	0	9.19	1	0.72472	.	.	1
36893	0	9.19	1	0.72472	.	.	1
36894	0	9.19	1	0.72472	.	.	1
36895	0	9.19	1	0.72472	.	.	1
36896	0	9.19	1	0.72472	.	.	1
36897	0	9.19	1	0.72472	.	.	1
36898	0	9.19	1	0.72472	.	.	1
36899	0	9.19	1	0.72472	.	.	1
36900	0	9.19	1	0.72472	.	.	1
36901	0	9.19	1	0.72472	.	.	1
36902	0	9.19	1	0.72472	.	.	1
36903	0	9.19	1	0.72472	.	.	1
36904	0	9.19	1	0.72472	.	.	1
36905	0	9.19	1	0.72472	.	.	1
36906	0	9.19	1	0.72472	.	.	1
36907	0	9.19	1	0.72472	.	.	1
36908	0	9.19	1	0.72472	.	.	1
36909	0	9.19	1	0.72472	.	.	1
36910	0	9.19	1	0.72472	.	.	1
36911	0	9.19	1	0.72472	.	.	1
36912	0	9.19	1	0.72472	.	.	1
36913	0	9.19	1	0.72472	.	.	1
36914	0	9.19	1	0.72472	.	.	1
36915	0	9.19	1	0.72472	.	.	1
36916	0	9.19	1	0.72472	.	.	1
36917	0	9.19	1	0.72472	.	.	1
36918	0	9.19	1	0.72472	.	.	1
36919	0	9.19	1	0.72472	.	.	1
36920	0	9.19	1	0.72472	.	.	1
36921	0	9.19	1	0.72472	.	.	1
36922	0	9.19	1	0.72472	.	.	1
36923	0	9.19	1	0.72472	.	.	1
36924	0	9.20	0	0.72443	0.71744	0.73129	1
36925	0	9.20	1	0.72443	.	.	1

Obs	Sub_group	fuptimey	_CENSOR_	SURVIVAL	SDF_LCL	SDF_UCL	STRATUM
36926	0	9.20	1	0.72443	.	.	1
36927	0	9.20	1	0.72443	.	.	1
36928	0	9.20	1	0.72443	.	.	1
36929	0	9.20	1	0.72443	.	.	1
36930	0	9.20	1	0.72443	.	.	1
36931	0	9.20	1	0.72443	.	.	1
36932	0	9.20	1	0.72443	.	.	1
36933	0	9.20	1	0.72443	.	.	1
36934	0	9.20	1	0.72443	.	.	1
36935	0	9.20	1	0.72443	.	.	1
36936	0	9.20	1	0.72443	.	.	1
36937	0	9.20	1	0.72443	.	.	1
36938	0	9.20	1	0.72443	.	.	1
36939	0	9.20	1	0.72443	.	.	1
36940	0	9.20	1	0.72443	.	.	1
36941	0	9.20	1	0.72443	.	.	1
36942	0	9.20	1	0.72443	.	.	1
36943	0	9.20	1	0.72443	.	.	1
36944	0	9.20	1	0.72443	.	.	1
36945	0	9.20	1	0.72443	.	.	1
36946	0	9.20	1	0.72443	.	.	1
36947	0	9.20	1	0.72443	.	.	1
36948	0	9.20	1	0.72443	.	.	1
36949	0	9.20	1	0.72443	.	.	1
36950	0	9.20	1	0.72443	.	.	1
36951	0	9.20	1	0.72443	.	.	1
36952	0	9.21	0	0.72414	0.71713	0.73102	1
36953	0	9.21	1	0.72414	.	.	1
36954	0	9.21	1	0.72414	.	.	1
36955	0	9.21	1	0.72414	.	.	1
36956	0	9.21	1	0.72414	.	.	1
36957	0	9.21	1	0.72414	.	.	1
36958	0	9.21	1	0.72414	.	.	1
36959	0	9.21	1	0.72414	.	.	1
36960	0	9.21	1	0.72414	.	.	1

Obs	Sub_group	fuptimey	_CENSOR_	SURVIVAL	SDF_LCL	SDF_UCL	STRATUM
36961	0	9.21	1	0.72414	.	.	1
36962	0	9.21	1	0.72414	.	.	1
36963	0	9.21	1	0.72414	.	.	1
36964	0	9.21	1	0.72414	.	.	1
36965	0	9.21	1	0.72414	.	.	1
36966	0	9.21	1	0.72414	.	.	1
36967	0	9.21	1	0.72414	.	.	1
36968	0	9.21	1	0.72414	.	.	1
36969	0	9.21	1	0.72414	.	.	1
36970	0	9.21	1	0.72414	.	.	1
36971	0	9.21	1	0.72414	.	.	1
36972	0	9.21	1	0.72414	.	.	1
36973	0	9.21	1	0.72414	.	.	1
36974	0	9.21	1	0.72414	.	.	1
36975	0	9.21	1	0.72414	.	.	1
36976	0	9.21	1	0.72414	.	.	1
36977	0	9.21	1	0.72414	.	.	1
36978	0	9.21	1	0.72414	.	.	1
36979	0	9.21	1	0.72414	.	.	1
36980	0	9.21	1	0.72414	.	.	1
36981	0	9.21	1	0.72414	.	.	1
36982	0	9.21	1	0.72414	.	.	1
36983	0	9.21	1	0.72414	.	.	1
36984	0	9.22	0	0.72355	0.71649	0.73047	1
36985	0	9.22	1	0.72355	.	.	1
36986	0	9.22	1	0.72355	.	.	1
36987	0	9.22	1	0.72355	.	.	1
36988	0	9.22	1	0.72355	.	.	1
36989	0	9.22	1	0.72355	.	.	1
36990	0	9.22	1	0.72355	.	.	1
36991	0	9.22	1	0.72355	.	.	1
36992	0	9.22	1	0.72355	.	.	1
36993	0	9.22	1	0.72355	.	.	1
36994	0	9.22	1	0.72355	.	.	1
36995	0	9.22	1	0.72355	.	.	1

Obs	Sub_group	fuptimey	_CENSOR_	SURVIVAL	SDF_LCL	SDF_UCL	STRATUM
36996	0	9.22	1	0.72355	.	.	1
36997	0	9.22	1	0.72355	.	.	1
36998	0	9.22	1	0.72355	.	.	1
36999	0	9.22	1	0.72355	.	.	1
37000	0	9.22	1	0.72355	.	.	1
37001	0	9.22	1	0.72355	.	.	1
37002	0	9.22	1	0.72355	.	.	1
37003	0	9.22	1	0.72355	.	.	1
37004	0	9.22	1	0.72355	.	.	1
37005	0	9.22	1	0.72355	.	.	1
37006	0	9.22	1	0.72355	.	.	1
37007	0	9.22	1	0.72355	.	.	1
37008	0	9.22	1	0.72355	.	.	1
37009	0	9.22	1	0.72355	.	.	1
37010	0	9.22	1	0.72355	.	.	1
37011	0	9.22	1	0.72355	.	.	1
37012	0	9.22	1	0.72355	.	.	1
37013	0	9.23	0	0.72325	0.71617	0.73019	1
37014	0	9.23	1	0.72325	.	.	1
37015	0	9.23	1	0.72325	.	.	1
37016	0	9.23	1	0.72325	.	.	1
37017	0	9.23	1	0.72325	.	.	1
37018	0	9.23	1	0.72325	.	.	1
37019	0	9.23	1	0.72325	.	.	1
37020	0	9.23	1	0.72325	.	.	1
37021	0	9.23	1	0.72325	.	.	1
37022	0	9.23	1	0.72325	.	.	1
37023	0	9.23	1	0.72325	.	.	1
37024	0	9.23	1	0.72325	.	.	1
37025	0	9.23	1	0.72325	.	.	1
37026	0	9.23	1	0.72325	.	.	1
37027	0	9.23	1	0.72325	.	.	1
37028	0	9.23	1	0.72325	.	.	1
37029	0	9.23	1	0.72325	.	.	1
37030	0	9.23	1	0.72325	.	.	1

Obs	Sub_group	fuptimey	_CENSOR_	SURVIVAL	SDF_LCL	SDF_UCL	STRATUM
37031	0	9.23	1	0.72325	.	.	1
37032	0	9.23	1	0.72325	.	.	1
37033	0	9.23	1	0.72325	.	.	1
37034	0	9.23	1	0.72325	.	.	1
37035	0	9.23	1	0.72325	.	.	1
37036	0	9.23	1	0.72325	.	.	1
37037	0	9.23	1	0.72325	.	.	1
37038	0	9.23	1	0.72325	.	.	1
37039	0	9.24	0	0.72295	0.71585	0.72991	1
37040	0	9.24	1	0.72295	.	.	1
37041	0	9.24	1	0.72295	.	.	1
37042	0	9.24	1	0.72295	.	.	1
37043	0	9.24	1	0.72295	.	.	1
37044	0	9.24	1	0.72295	.	.	1
37045	0	9.24	1	0.72295	.	.	1
37046	0	9.24	1	0.72295	.	.	1
37047	0	9.24	1	0.72295	.	.	1
37048	0	9.24	1	0.72295	.	.	1
37049	0	9.24	1	0.72295	.	.	1
37050	0	9.24	1	0.72295	.	.	1
37051	0	9.24	1	0.72295	.	.	1
37052	0	9.24	1	0.72295	.	.	1
37053	0	9.24	1	0.72295	.	.	1
37054	0	9.24	1	0.72295	.	.	1
37055	0	9.24	1	0.72295	.	.	1
37056	0	9.24	1	0.72295	.	.	1
37057	0	9.24	1	0.72295	.	.	1
37058	0	9.24	1	0.72295	.	.	1
37059	0	9.24	1	0.72295	.	.	1
37060	0	9.24	1	0.72295	.	.	1
37061	0	9.24	1	0.72295	.	.	1
37062	0	9.24	1	0.72295	.	.	1
37063	0	9.24	1	0.72295	.	.	1
37064	0	9.24	1	0.72295	.	.	1
37065	0	9.24	1	0.72295	.	.	1

Obs	Sub_group	fuptimey	_CENSOR_	SURVIVAL	SDF_LCL	SDF_UCL	STRATUM
37066	0	9.25	0	0.72265	0.71552	0.72963	1
37067	0	9.25	1	0.72265	.	.	1
37068	0	9.25	1	0.72265	.	.	1
37069	0	9.25	1	0.72265	.	.	1
37070	0	9.25	1	0.72265	.	.	1
37071	0	9.25	1	0.72265	.	.	1
37072	0	9.25	1	0.72265	.	.	1
37073	0	9.25	1	0.72265	.	.	1
37074	0	9.25	1	0.72265	.	.	1
37075	0	9.25	1	0.72265	.	.	1
37076	0	9.25	1	0.72265	.	.	1
37077	0	9.25	1	0.72265	.	.	1
37078	0	9.25	1	0.72265	.	.	1
37079	0	9.25	1	0.72265	.	.	1
37080	0	9.25	1	0.72265	.	.	1
37081	0	9.25	1	0.72265	.	.	1
37082	0	9.25	1	0.72265	.	.	1
37083	0	9.25	1	0.72265	.	.	1
37084	0	9.25	1	0.72265	.	.	1
37085	0	9.25	1	0.72265	.	.	1
37086	0	9.25	1	0.72265	.	.	1
37087	0	9.25	1	0.72265	.	.	1
37088	0	9.25	1	0.72265	.	.	1
37089	0	9.25	1	0.72265	.	.	1
37090	0	9.25	1	0.72265	.	.	1
37091	0	9.25	1	0.72265	.	.	1
37092	0	9.25	1	0.72265	.	.	1
37093	0	9.25	1	0.72265	.	.	1
37094	0	9.25	1	0.72265	.	.	1
37095	0	9.25	1	0.72265	.	.	1
37096	0	9.25	1	0.72265	.	.	1
37097	0	9.25	1	0.72265	.	.	1
37098	0	9.25	1	0.72265	.	.	1
37099	0	9.25	1	0.72265	.	.	1
37100	0	9.25	1	0.72265	.	.	1

Obs	Sub_group	fuptimey	_CENSOR_	SURVIVAL	SDF_LCL	SDF_UCL	STRATUM
37101	0	9.26	1	0.72265	.	.	1
37102	0	9.26	1	0.72265	.	.	1
37103	0	9.26	1	0.72265	.	.	1
37104	0	9.26	1	0.72265	.	.	1
37105	0	9.26	1	0.72265	.	.	1
37106	0	9.26	1	0.72265	.	.	1
37107	0	9.26	1	0.72265	.	.	1
37108	0	9.26	1	0.72265	.	.	1
37109	0	9.26	1	0.72265	.	.	1
37110	0	9.26	1	0.72265	.	.	1
37111	0	9.26	1	0.72265	.	.	1
37112	0	9.26	1	0.72265	.	.	1
37113	0	9.26	1	0.72265	.	.	1
37114	0	9.26	1	0.72265	.	.	1
37115	0	9.26	1	0.72265	.	.	1
37116	0	9.26	1	0.72265	.	.	1
37117	0	9.26	1	0.72265	.	.	1
37118	0	9.26	1	0.72265	.	.	1
37119	0	9.26	1	0.72265	.	.	1
37120	0	9.26	1	0.72265	.	.	1
37121	0	9.26	1	0.72265	.	.	1
37122	0	9.26	1	0.72265	.	.	1
37123	0	9.26	1	0.72265	.	.	1
37124	0	9.26	1	0.72265	.	.	1
37125	0	9.27	1	0.72265	.	.	1
37126	0	9.27	1	0.72265	.	.	1
37127	0	9.27	1	0.72265	.	.	1
37128	0	9.27	1	0.72265	.	.	1
37129	0	9.27	1	0.72265	.	.	1
37130	0	9.27	1	0.72265	.	.	1
37131	0	9.27	1	0.72265	.	.	1
37132	0	9.27	1	0.72265	.	.	1
37133	0	9.27	1	0.72265	.	.	1
37134	0	9.27	1	0.72265	.	.	1
37135	0	9.27	1	0.72265	.	.	1

Obs	Sub_group	fuptimey	_CENSOR_	SURVIVAL	SDF_LCL	SDF_UCL	STRATUM
37136	0	9.27	1	0.72265	.	.	1
37137	0	9.27	1	0.72265	.	.	1
37138	0	9.27	1	0.72265	.	.	1
37139	0	9.27	1	0.72265	.	.	1
37140	0	9.27	1	0.72265	.	.	1
37141	0	9.27	1	0.72265	.	.	1
37142	0	9.27	1	0.72265	.	.	1
37143	0	9.27	1	0.72265	.	.	1
37144	0	9.27	1	0.72265	.	.	1
37145	0	9.27	1	0.72265	.	.	1
37146	0	9.27	1	0.72265	.	.	1
37147	0	9.27	1	0.72265	.	.	1
37148	0	9.27	1	0.72265	.	.	1
37149	0	9.27	1	0.72265	.	.	1
37150	0	9.27	1	0.72265	.	.	1
37151	0	9.27	1	0.72265	.	.	1
37152	0	9.27	1	0.72265	.	.	1
37153	0	9.28	0	0.72202	0.71484	0.72905	1
37154	0	9.28	1	0.72202	.	.	1
37155	0	9.28	1	0.72202	.	.	1
37156	0	9.28	1	0.72202	.	.	1
37157	0	9.28	1	0.72202	.	.	1
37158	0	9.28	1	0.72202	.	.	1
37159	0	9.28	1	0.72202	.	.	1
37160	0	9.28	1	0.72202	.	.	1
37161	0	9.28	1	0.72202	.	.	1
37162	0	9.28	1	0.72202	.	.	1
37163	0	9.28	1	0.72202	.	.	1
37164	0	9.28	1	0.72202	.	.	1
37165	0	9.28	1	0.72202	.	.	1
37166	0	9.28	1	0.72202	.	.	1
37167	0	9.28	1	0.72202	.	.	1
37168	0	9.28	1	0.72202	.	.	1
37169	0	9.28	1	0.72202	.	.	1
37170	0	9.28	1	0.72202	.	.	1

Obs	Sub_group	fuptimey	_CENSOR_	SURVIVAL	SDF_LCL	SDF_UCL	STRATUM
37171	0	9.28	1	0.72202	.	.	1
37172	0	9.28	1	0.72202	.	.	1
37173	0	9.28	1	0.72202	.	.	1
37174	0	9.28	1	0.72202	.	.	1
37175	0	9.28	1	0.72202	.	.	1
37176	0	9.28	1	0.72202	.	.	1
37177	0	9.28	1	0.72202	.	.	1
37178	0	9.28	1	0.72202	.	.	1
37179	0	9.28	1	0.72202	.	.	1
37180	0	9.29	1	0.72202	.	.	1
37181	0	9.29	1	0.72202	.	.	1
37182	0	9.29	1	0.72202	.	.	1
37183	0	9.29	1	0.72202	.	.	1
37184	0	9.29	1	0.72202	.	.	1
37185	0	9.29	1	0.72202	.	.	1
37186	0	9.29	1	0.72202	.	.	1
37187	0	9.29	1	0.72202	.	.	1
37188	0	9.29	1	0.72202	.	.	1
37189	0	9.29	1	0.72202	.	.	1
37190	0	9.29	1	0.72202	.	.	1
37191	0	9.29	1	0.72202	.	.	1
37192	0	9.29	1	0.72202	.	.	1
37193	0	9.29	1	0.72202	.	.	1
37194	0	9.29	1	0.72202	.	.	1
37195	0	9.29	1	0.72202	.	.	1
37196	0	9.29	1	0.72202	.	.	1
37197	0	9.29	1	0.72202	.	.	1
37198	0	9.29	1	0.72202	.	.	1
37199	0	9.29	1	0.72202	.	.	1
37200	0	9.29	1	0.72202	.	.	1
37201	0	9.29	1	0.72202	.	.	1
37202	0	9.29	1	0.72202	.	.	1
37203	0	9.30	1	0.72202	.	.	1
37204	0	9.30	1	0.72202	.	.	1
37205	0	9.30	1	0.72202	.	.	1

Obs	Sub_group	fuptimey	_CENSOR_	SURVIVAL	SDF_LCL	SDF_UCL	STRATUM
37206	0	9.30	1	0.72202	.	.	1
37207	0	9.30	1	0.72202	.	.	1
37208	0	9.30	1	0.72202	.	.	1
37209	0	9.30	1	0.72202	.	.	1
37210	0	9.30	1	0.72202	.	.	1
37211	0	9.30	1	0.72202	.	.	1
37212	0	9.30	1	0.72202	.	.	1
37213	0	9.30	1	0.72202	.	.	1
37214	0	9.30	1	0.72202	.	.	1
37215	0	9.30	1	0.72202	.	.	1
37216	0	9.30	1	0.72202	.	.	1
37217	0	9.30	1	0.72202	.	.	1
37218	0	9.30	1	0.72202	.	.	1
37219	0	9.30	1	0.72202	.	.	1
37220	0	9.30	1	0.72202	.	.	1
37221	0	9.30	1	0.72202	.	.	1
37222	0	9.30	1	0.72202	.	.	1
37223	0	9.30	1	0.72202	.	.	1
37224	0	9.30	1	0.72202	.	.	1
37225	0	9.30	1	0.72202	.	.	1
37226	0	9.30	1	0.72202	.	.	1
37227	0	9.30	1	0.72202	.	.	1
37228	0	9.30	1	0.72202	.	.	1
37229	0	9.30	1	0.72202	.	.	1
37230	0	9.30	1	0.72202	.	.	1
37231	0	9.30	1	0.72202	.	.	1
37232	0	9.30	1	0.72202	.	.	1
37233	0	9.30	1	0.72202	.	.	1
37234	0	9.30	1	0.72202	.	.	1
37235	0	9.30	1	0.72202	.	.	1
37236	0	9.30	1	0.72202	.	.	1
37237	0	9.30	1	0.72202	.	.	1
37238	0	9.30	1	0.72202	.	.	1
37239	0	9.30	1	0.72202	.	.	1
37240	0	9.31	1	0.72202	.	.	1

Obs	Sub_group	fuptimey	_CENSOR_	SURVIVAL	SDF_LCL	SDF_UCL	STRATUM
37241	0	9.31	1	0.72202	.	.	1
37242	0	9.31	1	0.72202	.	.	1
37243	0	9.31	1	0.72202	.	.	1
37244	0	9.31	1	0.72202	.	.	1
37245	0	9.31	1	0.72202	.	.	1
37246	0	9.31	1	0.72202	.	.	1
37247	0	9.31	1	0.72202	.	.	1
37248	0	9.31	1	0.72202	.	.	1
37249	0	9.31	1	0.72202	.	.	1
37250	0	9.31	1	0.72202	.	.	1
37251	0	9.31	1	0.72202	.	.	1
37252	0	9.31	1	0.72202	.	.	1
37253	0	9.31	1	0.72202	.	.	1
37254	0	9.31	1	0.72202	.	.	1
37255	0	9.31	1	0.72202	.	.	1
37256	0	9.31	1	0.72202	.	.	1
37257	0	9.31	1	0.72202	.	.	1
37258	0	9.31	1	0.72202	.	.	1
37259	0	9.31	1	0.72202	.	.	1
37260	0	9.31	1	0.72202	.	.	1
37261	0	9.31	1	0.72202	.	.	1
37262	0	9.31	1	0.72202	.	.	1
37263	0	9.31	1	0.72202	.	.	1
37264	0	9.31	1	0.72202	.	.	1
37265	0	9.31	1	0.72202	.	.	1
37266	0	9.31	1	0.72202	.	.	1
37267	0	9.31	1	0.72202	.	.	1
37268	0	9.31	1	0.72202	.	.	1
37269	0	9.31	1	0.72202	.	.	1
37270	0	9.32	1	0.72202	.	.	1
37271	0	9.32	1	0.72202	.	.	1
37272	0	9.32	1	0.72202	.	.	1
37273	0	9.32	1	0.72202	.	.	1
37274	0	9.32	1	0.72202	.	.	1
37275	0	9.32	1	0.72202	.	.	1

Obs	Sub_group	fuptimey	_CENSOR_	SURVIVAL	SDF_LCL	SDF_UCL	STRATUM
37276	0	9.32	1	0.72202	.	.	1
37277	0	9.32	1	0.72202	.	.	1
37278	0	9.32	1	0.72202	.	.	1
37279	0	9.32	1	0.72202	.	.	1
37280	0	9.32	1	0.72202	.	.	1
37281	0	9.32	1	0.72202	.	.	1
37282	0	9.32	1	0.72202	.	.	1
37283	0	9.32	1	0.72202	.	.	1
37284	0	9.32	1	0.72202	.	.	1
37285	0	9.32	1	0.72202	.	.	1
37286	0	9.32	1	0.72202	.	.	1
37287	0	9.32	1	0.72202	.	.	1
37288	0	9.32	1	0.72202	.	.	1
37289	0	9.32	1	0.72202	.	.	1
37290	0	9.32	1	0.72202	.	.	1
37291	0	9.32	1	0.72202	.	.	1
37292	0	9.32	1	0.72202	.	.	1
37293	0	9.32	1	0.72202	.	.	1
37294	0	9.32	1	0.72202	.	.	1
37295	0	9.32	1	0.72202	.	.	1
37296	0	9.32	1	0.72202	.	.	1
37297	0	9.32	1	0.72202	.	.	1
37298	0	9.32	1	0.72202	.	.	1
37299	0	9.32	1	0.72202	.	.	1
37300	0	9.33	1	0.72202	.	.	1
37301	0	9.33	1	0.72202	.	.	1
37302	0	9.33	1	0.72202	.	.	1
37303	0	9.33	1	0.72202	.	.	1
37304	0	9.33	1	0.72202	.	.	1
37305	0	9.33	1	0.72202	.	.	1
37306	0	9.33	1	0.72202	.	.	1
37307	0	9.33	1	0.72202	.	.	1
37308	0	9.33	1	0.72202	.	.	1
37309	0	9.33	1	0.72202	.	.	1
37310	0	9.33	1	0.72202	.	.	1

Obs	Sub_group	fuptimey	_CENSOR_	SURVIVAL	SDF_LCL	SDF_UCL	STRATUM
37311	0	9.33	1	0.72202	.	.	1
37312	0	9.33	1	0.72202	.	.	1
37313	0	9.33	1	0.72202	.	.	1
37314	0	9.33	1	0.72202	.	.	1
37315	0	9.33	1	0.72202	.	.	1
37316	0	9.33	1	0.72202	.	.	1
37317	0	9.33	1	0.72202	.	.	1
37318	0	9.33	1	0.72202	.	.	1
37319	0	9.33	1	0.72202	.	.	1
37320	0	9.33	1	0.72202	.	.	1
37321	0	9.33	1	0.72202	.	.	1
37322	0	9.34	0	0.72167	0.71447	0.72873	1
37323	0	9.34	1	0.72167	.	.	1
37324	0	9.34	1	0.72167	.	.	1
37325	0	9.34	1	0.72167	.	.	1
37326	0	9.34	1	0.72167	.	.	1
37327	0	9.34	1	0.72167	.	.	1
37328	0	9.34	1	0.72167	.	.	1
37329	0	9.34	1	0.72167	.	.	1
37330	0	9.34	1	0.72167	.	.	1
37331	0	9.34	1	0.72167	.	.	1
37332	0	9.34	1	0.72167	.	.	1
37333	0	9.34	1	0.72167	.	.	1
37334	0	9.34	1	0.72167	.	.	1
37335	0	9.34	1	0.72167	.	.	1
37336	0	9.34	1	0.72167	.	.	1
37337	0	9.34	1	0.72167	.	.	1
37338	0	9.34	1	0.72167	.	.	1
37339	0	9.34	1	0.72167	.	.	1
37340	0	9.34	1	0.72167	.	.	1
37341	0	9.34	1	0.72167	.	.	1
37342	0	9.34	1	0.72167	.	.	1
37343	0	9.34	1	0.72167	.	.	1
37344	0	9.34	1	0.72167	.	.	1
37345	0	9.34	1	0.72167	.	.	1

Obs	Sub_group	fuptimey	_CENSOR_	SURVIVAL	SDF_LCL	SDF_UCL	STRATUM
37346	0	9.34	1	0.72167	.	.	1
37347	0	9.35	1	0.72167	.	.	1
37348	0	9.35	1	0.72167	.	.	1
37349	0	9.35	1	0.72167	.	.	1
37350	0	9.35	1	0.72167	.	.	1
37351	0	9.35	1	0.72167	.	.	1
37352	0	9.35	1	0.72167	.	.	1
37353	0	9.35	1	0.72167	.	.	1
37354	0	9.35	1	0.72167	.	.	1
37355	0	9.35	1	0.72167	.	.	1
37356	0	9.35	1	0.72167	.	.	1
37357	0	9.35	1	0.72167	.	.	1
37358	0	9.35	1	0.72167	.	.	1
37359	0	9.35	1	0.72167	.	.	1
37360	0	9.35	1	0.72167	.	.	1
37361	0	9.35	1	0.72167	.	.	1
37362	0	9.35	1	0.72167	.	.	1
37363	0	9.35	1	0.72167	.	.	1
37364	0	9.35	1	0.72167	.	.	1
37365	0	9.35	1	0.72167	.	.	1
37366	0	9.35	1	0.72167	.	.	1
37367	0	9.35	1	0.72167	.	.	1
37368	0	9.35	1	0.72167	.	.	1
37369	0	9.35	1	0.72167	.	.	1
37370	0	9.35	1	0.72167	.	.	1
37371	0	9.35	1	0.72167	.	.	1
37372	0	9.35	1	0.72167	.	.	1
37373	0	9.35	1	0.72167	.	.	1
37374	0	9.36	0	0.72062	0.71333	0.72777	1
37375	0	9.36	1	0.72062	.	.	1
37376	0	9.36	1	0.72062	.	.	1
37377	0	9.36	1	0.72062	.	.	1
37378	0	9.36	1	0.72062	.	.	1
37379	0	9.36	1	0.72062	.	.	1
37380	0	9.36	1	0.72062	.	.	1

Obs	Sub_group	fuptimey	_CENSOR_	SURVIVAL	SDF_LCL	SDF_UCL	STRATUM
37381	0	9.36	1	0.72062	.	.	1
37382	0	9.36	1	0.72062	.	.	1
37383	0	9.36	1	0.72062	.	.	1
37384	0	9.36	1	0.72062	.	.	1
37385	0	9.36	1	0.72062	.	.	1
37386	0	9.36	1	0.72062	.	.	1
37387	0	9.36	1	0.72062	.	.	1
37388	0	9.36	1	0.72062	.	.	1
37389	0	9.36	1	0.72062	.	.	1
37390	0	9.36	1	0.72062	.	.	1
37391	0	9.36	1	0.72062	.	.	1
37392	0	9.36	1	0.72062	.	.	1
37393	0	9.36	1	0.72062	.	.	1
37394	0	9.36	1	0.72062	.	.	1
37395	0	9.36	1	0.72062	.	.	1
37396	0	9.36	1	0.72062	.	.	1
37397	0	9.36	1	0.72062	.	.	1
37398	0	9.37	1	0.72062	.	.	1
37399	0	9.37	1	0.72062	.	.	1
37400	0	9.37	1	0.72062	.	.	1
37401	0	9.37	1	0.72062	.	.	1
37402	0	9.37	1	0.72062	.	.	1
37403	0	9.37	1	0.72062	.	.	1
37404	0	9.37	1	0.72062	.	.	1
37405	0	9.37	1	0.72062	.	.	1
37406	0	9.37	1	0.72062	.	.	1
37407	0	9.37	1	0.72062	.	.	1
37408	0	9.37	1	0.72062	.	.	1
37409	0	9.37	1	0.72062	.	.	1
37410	0	9.37	1	0.72062	.	.	1
37411	0	9.37	1	0.72062	.	.	1
37412	0	9.37	1	0.72062	.	.	1
37413	0	9.37	1	0.72062	.	.	1
37414	0	9.37	1	0.72062	.	.	1
37415	0	9.37	1	0.72062	.	.	1

Obs	Sub_group	fuptimey	_CENSOR_	SURVIVAL	SDF_LCL	SDF_UCL	STRATUM
37416	0	9.37	1	0.72062	.	.	1
37417	0	9.37	1	0.72062	.	.	1
37418	0	9.38	1	0.72062	.	.	1
37419	0	9.38	1	0.72062	.	.	1
37420	0	9.38	1	0.72062	.	.	1
37421	0	9.38	1	0.72062	.	.	1
37422	0	9.38	1	0.72062	.	.	1
37423	0	9.38	1	0.72062	.	.	1
37424	0	9.38	1	0.72062	.	.	1
37425	0	9.38	1	0.72062	.	.	1
37426	0	9.38	1	0.72062	.	.	1
37427	0	9.38	1	0.72062	.	.	1
37428	0	9.38	1	0.72062	.	.	1
37429	0	9.38	1	0.72062	.	.	1
37430	0	9.38	1	0.72062	.	.	1
37431	0	9.38	1	0.72062	.	.	1
37432	0	9.38	1	0.72062	.	.	1
37433	0	9.38	1	0.72062	.	.	1
37434	0	9.38	1	0.72062	.	.	1
37435	0	9.38	1	0.72062	.	.	1
37436	0	9.38	1	0.72062	.	.	1
37437	0	9.38	1	0.72062	.	.	1
37438	0	9.38	1	0.72062	.	.	1
37439	0	9.38	1	0.72062	.	.	1
37440	0	9.38	1	0.72062	.	.	1
37441	0	9.38	1	0.72062	.	.	1
37442	0	9.38	1	0.72062	.	.	1
37443	0	9.38	1	0.72062	.	.	1
37444	0	9.38	1	0.72062	.	.	1
37445	0	9.38	1	0.72062	.	.	1
37446	0	9.38	1	0.72062	.	.	1
37447	0	9.38	1	0.72062	.	.	1
37448	0	9.38	1	0.72062	.	.	1
37449	0	9.38	1	0.72062	.	.	1
37450	0	9.38	1	0.72062	.	.	1

Obs	Sub_group	fuptimey	_CENSOR_	SURVIVAL	SDF_LCL	SDF_UCL	STRATUM
37451	0	9.38	1	0.72062	.	.	1
37452	0	9.38	1	0.72062	.	.	1
37453	0	9.38	1	0.72062	.	.	1
37454	0	9.38	1	0.72062	.	.	1
37455	0	9.38	1	0.72062	.	.	1
37456	0	9.38	1	0.72062	.	.	1
37457	0	9.38	1	0.72062	.	.	1
37458	0	9.38	1	0.72062	.	.	1
37459	0	9.38	1	0.72062	.	.	1
37460	0	9.38	1	0.72062	.	.	1
37461	0	9.38	1	0.72062	.	.	1
37462	0	9.38	1	0.72062	.	.	1
37463	0	9.38	1	0.72062	.	.	1
37464	0	9.38	1	0.72062	.	.	1
37465	0	9.38	1	0.72062	.	.	1
37466	0	9.38	1	0.72062	.	.	1
37467	0	9.38	1	0.72062	.	.	1
37468	0	9.39	0	0.71989	0.71253	0.72710	1
37469	0	9.39	1	0.71989	.	.	1
37470	0	9.39	1	0.71989	.	.	1
37471	0	9.39	1	0.71989	.	.	1
37472	0	9.39	1	0.71989	.	.	1
37473	0	9.39	1	0.71989	.	.	1
37474	0	9.39	1	0.71989	.	.	1
37475	0	9.39	1	0.71989	.	.	1
37476	0	9.39	1	0.71989	.	.	1
37477	0	9.39	1	0.71989	.	.	1
37478	0	9.39	1	0.71989	.	.	1
37479	0	9.39	1	0.71989	.	.	1
37480	0	9.39	1	0.71989	.	.	1
37481	0	9.39	1	0.71989	.	.	1
37482	0	9.39	1	0.71989	.	.	1
37483	0	9.39	1	0.71989	.	.	1
37484	0	9.39	1	0.71989	.	.	1
37485	0	9.39	1	0.71989	.	.	1

Obs	Sub_group	fuptimey	_CENSOR_	SURVIVAL	SDF_LCL	SDF_UCL	STRATUM
37486	0	9.39	1	0.71989	.	.	1
37487	0	9.39	1	0.71989	.	.	1
37488	0	9.39	1	0.71989	.	.	1
37489	0	9.39	1	0.71989	.	.	1
37490	0	9.39	1	0.71989	.	.	1
37491	0	9.39	1	0.71989	.	.	1
37492	0	9.39	1	0.71989	.	.	1
37493	0	9.39	1	0.71989	.	.	1
37494	0	9.39	1	0.71989	.	.	1
37495	0	9.39	1	0.71989	.	.	1
37496	0	9.40	1	0.71989	.	.	1
37497	0	9.40	1	0.71989	.	.	1
37498	0	9.40	1	0.71989	.	.	1
37499	0	9.40	1	0.71989	.	.	1
37500	0	9.40	1	0.71989	.	.	1
37501	0	9.40	1	0.71989	.	.	1
37502	0	9.40	1	0.71989	.	.	1
37503	0	9.40	1	0.71989	.	.	1
37504	0	9.40	1	0.71989	.	.	1
37505	0	9.40	1	0.71989	.	.	1
37506	0	9.40	1	0.71989	.	.	1
37507	0	9.40	1	0.71989	.	.	1
37508	0	9.40	1	0.71989	.	.	1
37509	0	9.40	1	0.71989	.	.	1
37510	0	9.40	1	0.71989	.	.	1
37511	0	9.40	1	0.71989	.	.	1
37512	0	9.40	1	0.71989	.	.	1
37513	0	9.40	1	0.71989	.	.	1
37514	0	9.40	1	0.71989	.	.	1
37515	0	9.40	1	0.71989	.	.	1
37516	0	9.40	1	0.71989	.	.	1
37517	0	9.40	1	0.71989	.	.	1
37518	0	9.40	1	0.71989	.	.	1
37519	0	9.40	1	0.71989	.	.	1
37520	0	9.40	1	0.71989	.	.	1

Obs	Sub_group	fuptimey	_CENSOR_	SURVIVAL	SDF_LCL	SDF_UCL	STRATUM
37521	0	9.40	1	0.71989	.	.	1
37522	0	9.40	1	0.71989	.	.	1
37523	0	9.40	1	0.71989	.	.	1
37524	0	9.40	1	0.71989	.	.	1
37525	0	9.40	1	0.71989	.	.	1
37526	0	9.40	1	0.71989	.	.	1
37527	0	9.40	1	0.71989	.	.	1
37528	0	9.40	1	0.71989	.	.	1
37529	0	9.40	1	0.71989	.	.	1
37530	0	9.40	1	0.71989	.	.	1
37531	0	9.40	1	0.71989	.	.	1
37532	0	9.40	1	0.71989	.	.	1
37533	0	9.40	1	0.71989	.	.	1
37534	0	9.40	1	0.71989	.	.	1
37535	0	9.40	1	0.71989	.	.	1
37536	0	9.40	1	0.71989	.	.	1
37537	0	9.41	0	0.71951	0.71212	0.72675	1
37538	0	9.41	1	0.71951	.	.	1
37539	0	9.41	1	0.71951	.	.	1
37540	0	9.41	1	0.71951	.	.	1
37541	0	9.41	1	0.71951	.	.	1
37542	0	9.41	1	0.71951	.	.	1
37543	0	9.41	1	0.71951	.	.	1
37544	0	9.41	1	0.71951	.	.	1
37545	0	9.41	1	0.71951	.	.	1
37546	0	9.41	1	0.71951	.	.	1
37547	0	9.41	1	0.71951	.	.	1
37548	0	9.41	1	0.71951	.	.	1
37549	0	9.41	1	0.71951	.	.	1
37550	0	9.41	1	0.71951	.	.	1
37551	0	9.41	1	0.71951	.	.	1
37552	0	9.41	1	0.71951	.	.	1
37553	0	9.41	1	0.71951	.	.	1
37554	0	9.41	1	0.71951	.	.	1
37555	0	9.41	1	0.71951	.	.	1

Obs	Sub_group	fuptimey	_CENSOR_	SURVIVAL	SDF_LCL	SDF_UCL	STRATUM
37556	0	9.41	1	0.71951	.	.	1
37557	0	9.41	1	0.71951	.	.	1
37558	0	9.41	1	0.71951	.	.	1
37559	0	9.41	1	0.71951	.	.	1
37560	0	9.41	1	0.71951	.	.	1
37561	0	9.41	1	0.71951	.	.	1
37562	0	9.41	1	0.71951	.	.	1
37563	0	9.41	1	0.71951	.	.	1
37564	0	9.42	0	0.71913	0.71170	0.72640	1
37565	0	9.42	1	0.71913	.	.	1
37566	0	9.42	1	0.71913	.	.	1
37567	0	9.42	1	0.71913	.	.	1
37568	0	9.42	1	0.71913	.	.	1
37569	0	9.42	1	0.71913	.	.	1
37570	0	9.42	1	0.71913	.	.	1
37571	0	9.42	1	0.71913	.	.	1
37572	0	9.42	1	0.71913	.	.	1
37573	0	9.42	1	0.71913	.	.	1
37574	0	9.42	1	0.71913	.	.	1
37575	0	9.42	1	0.71913	.	.	1
37576	0	9.42	1	0.71913	.	.	1
37577	0	9.42	1	0.71913	.	.	1
37578	0	9.42	1	0.71913	.	.	1
37579	0	9.42	1	0.71913	.	.	1
37580	0	9.42	1	0.71913	.	.	1
37581	0	9.42	1	0.71913	.	.	1
37582	0	9.42	1	0.71913	.	.	1
37583	0	9.42	1	0.71913	.	.	1
37584	0	9.42	1	0.71913	.	.	1
37585	0	9.42	1	0.71913	.	.	1
37586	0	9.42	1	0.71913	.	.	1
37587	0	9.42	1	0.71913	.	.	1
37588	0	9.42	1	0.71913	.	.	1
37589	0	9.43	0	0.71835	0.71085	0.72569	1
37590	0	9.43	1	0.71835	.	.	1

Obs	Sub_group	fuptimey	_CENSOR_	SURVIVAL	SDF_LCL	SDF_UCL	STRATUM
**37591**	0	9.43	1	0.71835	.	.	1
**37592**	0	9.43	1	0.71835	.	.	1
**37593**	0	9.43	1	0.71835	.	.	1
**37594**	0	9.43	1	0.71835	.	.	1
**37595**	0	9.43	1	0.71835	.	.	1
**37596**	0	9.43	1	0.71835	.	.	1
**37597**	0	9.43	1	0.71835	.	.	1
**37598**	0	9.43	1	0.71835	.	.	1
**37599**	0	9.43	1	0.71835	.	.	1
**37600**	0	9.43	1	0.71835	.	.	1
**37601**	0	9.43	1	0.71835	.	.	1
**37602**	0	9.43	1	0.71835	.	.	1
**37603**	0	9.43	1	0.71835	.	.	1
**37604**	0	9.43	1	0.71835	.	.	1
**37605**	0	9.43	1	0.71835	.	.	1
**37606**	0	9.43	1	0.71835	.	.	1
**37607**	0	9.43	1	0.71835	.	.	1
**37608**	0	9.43	1	0.71835	.	.	1
**37609**	0	9.43	1	0.71835	.	.	1
**37610**	0	9.43	1	0.71835	.	.	1
**37611**	0	9.43	1	0.71835	.	.	1
**37612**	0	9.43	1	0.71835	.	.	1
**37613**	0	9.43	1	0.71835	.	.	1
**37614**	0	9.43	1	0.71835	.	.	1
**37615**	0	9.43	1	0.71835	.	.	1
**37616**	0	9.43	1	0.71835	.	.	1
**37617**	0	9.43	1	0.71835	.	.	1
**37618**	0	9.43	1	0.71835	.	.	1
**37619**	0	9.43	1	0.71835	.	.	1
**37620**	0	9.43	1	0.71835	.	.	1
**37621**	0	9.43	1	0.71835	.	.	1
**37622**	0	9.43	1	0.71835	.	.	1
**37623**	0	9.43	1	0.71835	.	.	1
**37624**	0	9.44	0	0.71795	0.71041	0.72533	1
**37625**	0	9.44	1	0.71795	.	.	1

Obs	Sub_group	fuptimey	_CENSOR_	SURVIVAL	SDF_LCL	SDF_UCL	STRATUM
37626	0	9.44	1	0.71795	.	.	1
37627	0	9.44	1	0.71795	.	.	1
37628	0	9.44	1	0.71795	.	.	1
37629	0	9.44	1	0.71795	.	.	1
37630	0	9.44	1	0.71795	.	.	1
37631	0	9.44	1	0.71795	.	.	1
37632	0	9.44	1	0.71795	.	.	1
37633	0	9.44	1	0.71795	.	.	1
37634	0	9.44	1	0.71795	.	.	1
37635	0	9.44	1	0.71795	.	.	1
37636	0	9.44	1	0.71795	.	.	1
37637	0	9.44	1	0.71795	.	.	1
37638	0	9.44	1	0.71795	.	.	1
37639	0	9.44	1	0.71795	.	.	1
37640	0	9.44	1	0.71795	.	.	1
37641	0	9.44	1	0.71795	.	.	1
37642	0	9.44	1	0.71795	.	.	1
37643	0	9.44	1	0.71795	.	.	1
37644	0	9.44	1	0.71795	.	.	1
37645	0	9.44	1	0.71795	.	.	1
37646	0	9.44	1	0.71795	.	.	1
37647	0	9.44	1	0.71795	.	.	1
37648	0	9.44	1	0.71795	.	.	1
37649	0	9.44	1	0.71795	.	.	1
37650	0	9.44	1	0.71795	.	.	1
37651	0	9.44	1	0.71795	.	.	1
37652	0	9.44	1	0.71795	.	.	1
37653	0	9.44	1	0.71795	.	.	1
37654	0	9.44	1	0.71795	.	.	1
37655	0	9.45	0	0.71714	0.70953	0.72460	1
37656	0	9.45	1	0.71714	.	.	1
37657	0	9.45	1	0.71714	.	.	1
37658	0	9.45	1	0.71714	.	.	1
37659	0	9.45	1	0.71714	.	.	1
37660	0	9.45	1	0.71714	.	.	1

Obs	Sub_group	fuptimey	_CENSOR_	SURVIVAL	SDF_LCL	SDF_UCL	STRATUM
37661	0	9.45	1	0.71714	.	.	1
37662	0	9.45	1	0.71714	.	.	1
37663	0	9.45	1	0.71714	.	.	1
37664	0	9.45	1	0.71714	.	.	1
37665	0	9.45	1	0.71714	.	.	1
37666	0	9.45	1	0.71714	.	.	1
37667	0	9.45	1	0.71714	.	.	1
37668	0	9.45	1	0.71714	.	.	1
37669	0	9.45	1	0.71714	.	.	1
37670	0	9.45	1	0.71714	.	.	1
37671	0	9.45	1	0.71714	.	.	1
37672	0	9.45	1	0.71714	.	.	1
37673	0	9.45	1	0.71714	.	.	1
37674	0	9.45	1	0.71714	.	.	1
37675	0	9.45	1	0.71714	.	.	1
37676	0	9.45	1	0.71714	.	.	1
37677	0	9.45	1	0.71714	.	.	1
37678	0	9.45	1	0.71714	.	.	1
37679	0	9.45	1	0.71714	.	.	1
37680	0	9.45	1	0.71714	.	.	1
37681	0	9.45	1	0.71714	.	.	1
37682	0	9.45	1	0.71714	.	.	1
37683	0	9.45	1	0.71714	.	.	1
37684	0	9.45	1	0.71714	.	.	1
37685	0	9.45	1	0.71714	.	.	1
37686	0	9.45	1	0.71714	.	.	1
37687	0	9.45	1	0.71714	.	.	1
37688	0	9.45	1	0.71714	.	.	1
37689	0	9.45	1	0.71714	.	.	1
37690	0	9.45	1	0.71714	.	.	1
37691	0	9.45	1	0.71714	.	.	1
37692	0	9.45	1	0.71714	.	.	1
37693	0	9.45	1	0.71714	.	.	1
37694	0	9.45	1	0.71714	.	.	1
37695	0	9.45	1	0.71714	.	.	1

Obs	Sub_group	fuptimey	_CENSOR_	SURVIVAL	SDF_LCL	SDF_UCL	STRATUM
37696	0	9.45	1	0.71714	.	.	1
37697	0	9.46	1	0.71714	.	.	1
37698	0	9.46	1	0.71714	.	.	1
37699	0	9.46	1	0.71714	.	.	1
37700	0	9.46	1	0.71714	.	.	1
37701	0	9.46	1	0.71714	.	.	1
37702	0	9.46	1	0.71714	.	.	1
37703	0	9.46	1	0.71714	.	.	1
37704	0	9.46	1	0.71714	.	.	1
37705	0	9.46	1	0.71714	.	.	1
37706	0	9.46	1	0.71714	.	.	1
37707	0	9.46	1	0.71714	.	.	1
37708	0	9.46	1	0.71714	.	.	1
37709	0	9.46	1	0.71714	.	.	1
37710	0	9.46	1	0.71714	.	.	1
37711	0	9.46	1	0.71714	.	.	1
37712	0	9.46	1	0.71714	.	.	1
37713	0	9.46	1	0.71714	.	.	1
37714	0	9.46	1	0.71714	.	.	1
37715	0	9.46	1	0.71714	.	.	1
37716	0	9.46	1	0.71714	.	.	1
37717	0	9.46	1	0.71714	.	.	1
37718	0	9.46	1	0.71714	.	.	1
37719	0	9.46	1	0.71714	.	.	1
37720	0	9.46	1	0.71714	.	.	1
37721	0	9.46	1	0.71714	.	.	1
37722	0	9.46	1	0.71714	.	.	1
37723	0	9.46	1	0.71714	.	.	1
37724	0	9.46	1	0.71714	.	.	1
37725	0	9.46	1	0.71714	.	.	1
37726	0	9.46	1	0.71714	.	.	1
37727	0	9.46	1	0.71714	.	.	1
37728	0	9.46	1	0.71714	.	.	1
37729	0	9.46	1	0.71714	.	.	1
37730	0	9.46	1	0.71714	.	.	1

Obs	Sub_group	fuptimey	_CENSOR_	SURVIVAL	SDF_LCL	SDF_UCL	STRATUM
37731	0	9.47	0	0.71672	0.70907	0.72422	1
37732	0	9.47	1	0.71672	.	.	1
37733	0	9.47	1	0.71672	.	.	1
37734	0	9.47	1	0.71672	.	.	1
37735	0	9.47	1	0.71672	.	.	1
37736	0	9.47	1	0.71672	.	.	1
37737	0	9.47	1	0.71672	.	.	1
37738	0	9.47	1	0.71672	.	.	1
37739	0	9.47	1	0.71672	.	.	1
37740	0	9.47	1	0.71672	.	.	1
37741	0	9.47	1	0.71672	.	.	1
37742	0	9.47	1	0.71672	.	.	1
37743	0	9.47	1	0.71672	.	.	1
37744	0	9.47	1	0.71672	.	.	1
37745	0	9.47	1	0.71672	.	.	1
37746	0	9.47	1	0.71672	.	.	1
37747	0	9.47	1	0.71672	.	.	1
37748	0	9.47	1	0.71672	.	.	1
37749	0	9.47	1	0.71672	.	.	1
37750	0	9.47	1	0.71672	.	.	1
37751	0	9.47	1	0.71672	.	.	1
37752	0	9.47	1	0.71672	.	.	1
37753	0	9.47	1	0.71672	.	.	1
37754	0	9.47	1	0.71672	.	.	1
37755	0	9.47	1	0.71672	.	.	1
37756	0	9.47	1	0.71672	.	.	1
37757	0	9.47	1	0.71672	.	.	1
37758	0	9.47	1	0.71672	.	.	1
37759	0	9.47	1	0.71672	.	.	1
37760	0	9.47	1	0.71672	.	.	1
37761	0	9.47	1	0.71672	.	.	1
37762	0	9.47	1	0.71672	.	.	1
37763	0	9.47	1	0.71672	.	.	1
37764	0	9.47	1	0.71672	.	.	1
37765	0	9.47	1	0.71672	.	.	1

Obs	Sub_group	fuptimey	_CENSOR_	SURVIVAL	SDF_LCL	SDF_UCL	STRATUM
37766	0	9.47	1	0.71672	.	.	1
37767	0	9.47	1	0.71672	.	.	1
37768	0	9.47	1	0.71672	.	.	1
37769	0	9.47	1	0.71672	.	.	1
37770	0	9.48	0	0.71629	0.70859	0.72383	1
37771	0	9.48	1	0.71629	.	.	1
37772	0	9.48	1	0.71629	.	.	1
37773	0	9.48	1	0.71629	.	.	1
37774	0	9.48	1	0.71629	.	.	1
37775	0	9.48	1	0.71629	.	.	1
37776	0	9.48	1	0.71629	.	.	1
37777	0	9.48	1	0.71629	.	.	1
37778	0	9.48	1	0.71629	.	.	1
37779	0	9.48	1	0.71629	.	.	1
37780	0	9.48	1	0.71629	.	.	1
37781	0	9.48	1	0.71629	.	.	1
37782	0	9.48	1	0.71629	.	.	1
37783	0	9.48	1	0.71629	.	.	1
37784	0	9.48	1	0.71629	.	.	1
37785	0	9.48	1	0.71629	.	.	1
37786	0	9.48	1	0.71629	.	.	1
37787	0	9.48	1	0.71629	.	.	1
37788	0	9.48	1	0.71629	.	.	1
37789	0	9.48	1	0.71629	.	.	1
37790	0	9.48	1	0.71629	.	.	1
37791	0	9.48	1	0.71629	.	.	1
37792	0	9.48	1	0.71629	.	.	1
37793	0	9.48	1	0.71629	.	.	1
37794	0	9.48	1	0.71629	.	.	1
37795	0	9.48	1	0.71629	.	.	1
37796	0	9.48	1	0.71629	.	.	1
37797	0	9.48	1	0.71629	.	.	1
37798	0	9.48	1	0.71629	.	.	1
37799	0	9.48	1	0.71629	.	.	1
37800	0	9.48	1	0.71629	.	.	1

Obs	Sub_group	fuptimey	_CENSOR_	SURVIVAL	SDF_LCL	SDF_UCL	STRATUM
**37801**	0	9.48	1	0.71629	.	.	1
**37802**	0	9.49	0	0.71541	0.70762	0.72303	1
**37803**	0	9.49	1	0.71541	.	.	1
**37804**	0	9.49	1	0.71541	.	.	1
**37805**	0	9.49	1	0.71541	.	.	1
**37806**	0	9.49	1	0.71541	.	.	1
**37807**	0	9.49	1	0.71541	.	.	1
**37808**	0	9.49	1	0.71541	.	.	1
**37809**	0	9.49	1	0.71541	.	.	1
**37810**	0	9.49	1	0.71541	.	.	1
**37811**	0	9.49	1	0.71541	.	.	1
**37812**	0	9.49	1	0.71541	.	.	1
**37813**	0	9.49	1	0.71541	.	.	1
**37814**	0	9.49	1	0.71541	.	.	1
**37815**	0	9.49	1	0.71541	.	.	1
**37816**	0	9.49	1	0.71541	.	.	1
**37817**	0	9.49	1	0.71541	.	.	1
**37818**	0	9.49	1	0.71541	.	.	1
**37819**	0	9.49	1	0.71541	.	.	1
**37820**	0	9.49	1	0.71541	.	.	1
**37821**	0	9.49	1	0.71541	.	.	1
**37822**	0	9.49	1	0.71541	.	.	1
**37823**	0	9.49	1	0.71541	.	.	1
**37824**	0	9.49	1	0.71541	.	.	1
**37825**	0	9.49	1	0.71541	.	.	1
**37826**	0	9.49	1	0.71541	.	.	1
**37827**	0	9.49	1	0.71541	.	.	1
**37828**	0	9.49	1	0.71541	.	.	1
**37829**	0	9.50	0	0.71407	0.70615	0.72182	1
**37830**	0	9.50	1	0.71407	.	.	1
**37831**	0	9.50	1	0.71407	.	.	1
**37832**	0	9.50	1	0.71407	.	.	1
**37833**	0	9.50	1	0.71407	.	.	1
**37834**	0	9.50	1	0.71407	.	.	1
**37835**	0	9.50	1	0.71407	.	.	1

Obs	Sub_group	fuptimey	_CENSOR_	SURVIVAL	SDF_LCL	SDF_UCL	STRATUM
**37836**	0	9.50	1	0.71407	.	.	1
**37837**	0	9.50	1	0.71407	.	.	1
**37838**	0	9.50	1	0.71407	.	.	1
**37839**	0	9.50	1	0.71407	.	.	1
**37840**	0	9.50	1	0.71407	.	.	1
**37841**	0	9.50	1	0.71407	.	.	1
**37842**	0	9.50	1	0.71407	.	.	1
**37843**	0	9.50	1	0.71407	.	.	1
**37844**	0	9.50	1	0.71407	.	.	1
**37845**	0	9.50	1	0.71407	.	.	1
**37846**	0	9.50	1	0.71407	.	.	1
**37847**	0	9.50	1	0.71407	.	.	1
**37848**	0	9.50	1	0.71407	.	.	1
**37849**	0	9.50	1	0.71407	.	.	1
**37850**	0	9.50	1	0.71407	.	.	1
**37851**	0	9.50	1	0.71407	.	.	1
**37852**	0	9.50	1	0.71407	.	.	1
**37853**	0	9.50	1	0.71407	.	.	1
**37854**	0	9.50	1	0.71407	.	.	1
**37855**	0	9.50	1	0.71407	.	.	1
**37856**	0	9.50	1	0.71407	.	.	1
**37857**	0	9.50	1	0.71407	.	.	1
**37858**	0	9.50	1	0.71407	.	.	1
**37859**	0	9.50	1	0.71407	.	.	1
**37860**	0	9.51	1	0.71407	.	.	1
**37861**	0	9.51	1	0.71407	.	.	1
**37862**	0	9.51	1	0.71407	.	.	1
**37863**	0	9.51	1	0.71407	.	.	1
**37864**	0	9.51	1	0.71407	.	.	1
**37865**	0	9.51	1	0.71407	.	.	1
**37866**	0	9.51	1	0.71407	.	.	1
**37867**	0	9.51	1	0.71407	.	.	1
**37868**	0	9.51	1	0.71407	.	.	1
**37869**	0	9.51	1	0.71407	.	.	1
**37870**	0	9.51	1	0.71407	.	.	1

Obs	Sub_group	fuptimey	_CENSOR_	SURVIVAL	SDF_LCL	SDF_UCL	STRATUM
37871	0	9.51	1	0.71407	.	.	1
37872	0	9.51	1	0.71407	.	.	1
37873	0	9.51	1	0.71407	.	.	1
37874	0	9.51	1	0.71407	.	.	1
37875	0	9.51	1	0.71407	.	.	1
37876	0	9.51	1	0.71407	.	.	1
37877	0	9.51	1	0.71407	.	.	1
37878	0	9.51	1	0.71407	.	.	1
37879	0	9.51	1	0.71407	.	.	1
37880	0	9.51	1	0.71407	.	.	1
37881	0	9.51	1	0.71407	.	.	1
37882	0	9.51	1	0.71407	.	.	1
37883	0	9.51	1	0.71407	.	.	1
37884	0	9.51	1	0.71407	.	.	1
37885	0	9.51	1	0.71407	.	.	1
37886	0	9.51	1	0.71407	.	.	1
37887	0	9.51	1	0.71407	.	.	1
37888	0	9.51	1	0.71407	.	.	1
37889	0	9.51	1	0.71407	.	.	1
37890	0	9.51	1	0.71407	.	.	1
37891	0	9.51	1	0.71407	.	.	1
37892	0	9.51	1	0.71407	.	.	1
37893	0	9.51	1	0.71407	.	.	1
37894	0	9.51	1	0.71407	.	.	1
37895	0	9.51	1	0.71407	.	.	1
37896	0	9.52	0	0.71221	0.70409	0.72015	1
37897	0	9.52	1	0.71221	.	.	1
37898	0	9.52	1	0.71221	.	.	1
37899	0	9.52	1	0.71221	.	.	1
37900	0	9.52	1	0.71221	.	.	1
37901	0	9.52	1	0.71221	.	.	1
37902	0	9.52	1	0.71221	.	.	1
37903	0	9.52	1	0.71221	.	.	1
37904	0	9.52	1	0.71221	.	.	1
37905	0	9.52	1	0.71221	.	.	1

Obs	Sub_group	fuptimey	_CENSOR_	SURVIVAL	SDF_LCL	SDF_UCL	STRATUM
37906	0	9.52	1	0.71221	.	.	1
37907	0	9.52	1	0.71221	.	.	1
37908	0	9.52	1	0.71221	.	.	1
37909	0	9.52	1	0.71221	.	.	1
37910	0	9.52	1	0.71221	.	.	1
37911	0	9.52	1	0.71221	.	.	1
37912	0	9.52	1	0.71221	.	.	1
37913	0	9.52	1	0.71221	.	.	1
37914	0	9.52	1	0.71221	.	.	1
37915	0	9.52	1	0.71221	.	.	1
37916	0	9.52	1	0.71221	.	.	1
37917	0	9.52	1	0.71221	.	.	1
37918	0	9.52	1	0.71221	.	.	1
37919	0	9.52	1	0.71221	.	.	1
37920	0	9.52	1	0.71221	.	.	1
37921	0	9.52	1	0.71221	.	.	1
37922	0	9.52	1	0.71221	.	.	1
37923	0	9.52	1	0.71221	.	.	1
37924	0	9.52	1	0.71221	.	.	1
37925	0	9.53	1	0.71221	.	.	1
37926	0	9.53	1	0.71221	.	.	1
37927	0	9.53	1	0.71221	.	.	1
37928	0	9.53	1	0.71221	.	.	1
37929	0	9.53	1	0.71221	.	.	1
37930	0	9.53	1	0.71221	.	.	1
37931	0	9.53	1	0.71221	.	.	1
37932	0	9.53	1	0.71221	.	.	1
37933	0	9.53	1	0.71221	.	.	1
37934	0	9.53	1	0.71221	.	.	1
37935	0	9.53	1	0.71221	.	.	1
37936	0	9.53	1	0.71221	.	.	1
37937	0	9.53	1	0.71221	.	.	1
37938	0	9.53	1	0.71221	.	.	1
37939	0	9.53	1	0.71221	.	.	1
37940	0	9.53	1	0.71221	.	.	1

Obs	Sub_group	fuptimey	_CENSOR_	SURVIVAL	SDF_LCL	SDF_UCL	STRATUM
37941	0	9.53	1	0.71221	.	.	1
37942	0	9.53	1	0.71221	.	.	1
37943	0	9.53	1	0.71221	.	.	1
37944	0	9.53	1	0.71221	.	.	1
37945	0	9.53	1	0.71221	.	.	1
37946	0	9.53	1	0.71221	.	.	1
37947	0	9.53	1	0.71221	.	.	1
37948	0	9.53	1	0.71221	.	.	1
37949	0	9.53	1	0.71221	.	.	1
37950	0	9.53	1	0.71221	.	.	1
37951	0	9.53	1	0.71221	.	.	1
37952	0	9.53	1	0.71221	.	.	1
37953	0	9.53	1	0.71221	.	.	1
37954	0	9.53	1	0.71221	.	.	1
37955	0	9.53	1	0.71221	.	.	1
37956	0	9.53	1	0.71221	.	.	1
37957	0	9.53	1	0.71221	.	.	1
37958	0	9.53	1	0.71221	.	.	1
37959	0	9.54	0	0.71075	0.70248	0.71884	1
37960	0	9.54	1	0.71075	.	.	1
37961	0	9.54	1	0.71075	.	.	1
37962	0	9.54	1	0.71075	.	.	1
37963	0	9.54	1	0.71075	.	.	1
37964	0	9.54	1	0.71075	.	.	1
37965	0	9.54	1	0.71075	.	.	1
37966	0	9.54	1	0.71075	.	.	1
37967	0	9.54	1	0.71075	.	.	1
37968	0	9.54	1	0.71075	.	.	1
37969	0	9.54	1	0.71075	.	.	1
37970	0	9.54	1	0.71075	.	.	1
37971	0	9.54	1	0.71075	.	.	1
37972	0	9.54	1	0.71075	.	.	1
37973	0	9.54	1	0.71075	.	.	1
37974	0	9.54	1	0.71075	.	.	1
37975	0	9.54	1	0.71075	.	.	1

Obs	Sub_group	fuptimey	_CENSOR_	SURVIVAL	SDF_LCL	SDF_UCL	STRATUM
37976	0	9.54	1	0.71075	.	.	1
37977	0	9.54	1	0.71075	.	.	1
37978	0	9.54	1	0.71075	.	.	1
37979	0	9.54	1	0.71075	.	.	1
37980	0	9.54	1	0.71075	.	.	1
37981	0	9.55	1	0.71075	.	.	1
37982	0	9.55	1	0.71075	.	.	1
37983	0	9.55	1	0.71075	.	.	1
37984	0	9.55	1	0.71075	.	.	1
37985	0	9.55	1	0.71075	.	.	1
37986	0	9.55	1	0.71075	.	.	1
37987	0	9.55	1	0.71075	.	.	1
37988	0	9.55	1	0.71075	.	.	1
37989	0	9.55	1	0.71075	.	.	1
37990	0	9.55	1	0.71075	.	.	1
37991	0	9.55	1	0.71075	.	.	1
37992	0	9.55	1	0.71075	.	.	1
37993	0	9.55	1	0.71075	.	.	1
37994	0	9.55	1	0.71075	.	.	1
37995	0	9.55	1	0.71075	.	.	1
37996	0	9.55	1	0.71075	.	.	1
37997	0	9.55	1	0.71075	.	.	1
37998	0	9.55	1	0.71075	.	.	1
37999	0	9.55	1	0.71075	.	.	1
38000	0	9.55	1	0.71075	.	.	1
38001	0	9.55	1	0.71075	.	.	1
38002	0	9.55	1	0.71075	.	.	1
38003	0	9.55	1	0.71075	.	.	1
38004	0	9.55	1	0.71075	.	.	1
38005	0	9.55	1	0.71075	.	.	1
38006	0	9.55	1	0.71075	.	.	1
38007	0	9.55	1	0.71075	.	.	1
38008	0	9.55	1	0.71075	.	.	1
38009	0	9.55	1	0.71075	.	.	1
38010	0	9.55	1	0.71075	.	.	1

Obs	Sub_group	fuptimey	_CENSOR_	SURVIVAL	SDF_LCL	SDF_UCL	STRATUM
38011	0	9.55	1	0.71075	.	.	1
38012	0	9.55	1	0.71075	.	.	1
38013	0	9.55	1	0.71075	.	.	1
38014	0	9.55	1	0.71075	.	.	1
38015	0	9.55	1	0.71075	.	.	1
38016	0	9.55	1	0.71075	.	.	1
38017	0	9.55	1	0.71075	.	.	1
38018	0	9.55	1	0.71075	.	.	1
38019	0	9.55	1	0.71075	.	.	1
38020	0	9.55	1	0.71075	.	.	1
38021	0	9.55	1	0.71075	.	.	1
38022	0	9.56	1	0.71075	.	.	1
38023	0	9.56	1	0.71075	.	.	1
38024	0	9.56	1	0.71075	.	.	1
38025	0	9.56	1	0.71075	.	.	1
38026	0	9.56	1	0.71075	.	.	1
38027	0	9.56	1	0.71075	.	.	1
38028	0	9.56	1	0.71075	.	.	1
38029	0	9.56	1	0.71075	.	.	1
38030	0	9.56	1	0.71075	.	.	1
38031	0	9.56	1	0.71075	.	.	1
38032	0	9.56	1	0.71075	.	.	1
38033	0	9.56	1	0.71075	.	.	1
38034	0	9.56	1	0.71075	.	.	1
38035	0	9.56	1	0.71075	.	.	1
38036	0	9.56	1	0.71075	.	.	1
38037	0	9.56	1	0.71075	.	.	1
38038	0	9.56	1	0.71075	.	.	1
38039	0	9.56	1	0.71075	.	.	1
38040	0	9.56	1	0.71075	.	.	1
38041	0	9.56	1	0.71075	.	.	1
38042	0	9.57	1	0.71075	.	.	1
38043	0	9.57	1	0.71075	.	.	1
38044	0	9.57	1	0.71075	.	.	1
38045	0	9.57	1	0.71075	.	.	1

Obs	Sub_group	fuptimey	_CENSOR_	SURVIVAL	SDF_LCL	SDF_UCL	STRATUM
38046	0	9.57	1	0.71075	.	.	1
38047	0	9.57	1	0.71075	.	.	1
38048	0	9.57	1	0.71075	.	.	1
38049	0	9.57	1	0.71075	.	.	1
38050	0	9.57	1	0.71075	.	.	1
38051	0	9.57	1	0.71075	.	.	1
38052	0	9.57	1	0.71075	.	.	1
38053	0	9.57	1	0.71075	.	.	1
38054	0	9.57	1	0.71075	.	.	1
38055	0	9.57	1	0.71075	.	.	1
38056	0	9.57	1	0.71075	.	.	1
38057	0	9.57	1	0.71075	.	.	1
38058	0	9.57	1	0.71075	.	.	1
38059	0	9.57	1	0.71075	.	.	1
38060	0	9.57	1	0.71075	.	.	1
38061	0	9.57	1	0.71075	.	.	1
38062	0	9.57	1	0.71075	.	.	1
38063	0	9.57	1	0.71075	.	.	1
38064	0	9.57	1	0.71075	.	.	1
38065	0	9.57	1	0.71075	.	.	1
38066	0	9.57	1	0.71075	.	.	1
38067	0	9.57	1	0.71075	.	.	1
38068	0	9.57	1	0.71075	.	.	1
38069	0	9.57	1	0.71075	.	.	1
38070	0	9.57	1	0.71075	.	.	1
38071	0	9.57	1	0.71075	.	.	1
38072	0	9.57	1	0.71075	.	.	1
38073	0	9.57	1	0.71075	.	.	1
38074	0	9.58	0	0.70970	0.70131	0.71790	1
38075	0	9.58	1	0.70970	.	.	1
38076	0	9.58	1	0.70970	.	.	1
38077	0	9.58	1	0.70970	.	.	1
38078	0	9.58	1	0.70970	.	.	1
38079	0	9.58	1	0.70970	.	.	1
38080	0	9.58	1	0.70970	.	.	1

Obs	Sub_group	fuptimey	_CENSOR_	SURVIVAL	SDF_LCL	SDF_UCL	STRATUM
38081	0	9.58	1	0.70970	.	.	1
38082	0	9.58	1	0.70970	.	.	1
38083	0	9.58	1	0.70970	.	.	1
38084	0	9.58	1	0.70970	.	.	1
38085	0	9.58	1	0.70970	.	.	1
38086	0	9.58	1	0.70970	.	.	1
38087	0	9.58	1	0.70970	.	.	1
38088	0	9.58	1	0.70970	.	.	1
38089	0	9.58	1	0.70970	.	.	1
38090	0	9.58	1	0.70970	.	.	1
38091	0	9.58	1	0.70970	.	.	1
38092	0	9.58	1	0.70970	.	.	1
38093	0	9.58	1	0.70970	.	.	1
38094	0	9.58	1	0.70970	.	.	1
38095	0	9.58	1	0.70970	.	.	1
38096	0	9.58	1	0.70970	.	.	1
38097	0	9.58	1	0.70970	.	.	1
38098	0	9.58	1	0.70970	.	.	1
38099	0	9.58	1	0.70970	.	.	1
38100	0	9.58	1	0.70970	.	.	1
38101	0	9.58	1	0.70970	.	.	1
38102	0	9.58	1	0.70970	.	.	1
38103	0	9.58	1	0.70970	.	.	1
38104	0	9.58	1	0.70970	.	.	1
38105	0	9.58	1	0.70970	.	.	1
38106	0	9.58	1	0.70970	.	.	1
38107	0	9.58	1	0.70970	.	.	1
38108	0	9.59	0	0.70862	0.70011	0.71694	1
38109	0	9.59	1	0.70862	.	.	1
38110	0	9.59	1	0.70862	.	.	1
38111	0	9.59	1	0.70862	.	.	1
38112	0	9.59	1	0.70862	.	.	1
38113	0	9.59	1	0.70862	.	.	1
38114	0	9.59	1	0.70862	.	.	1
38115	0	9.59	1	0.70862	.	.	1

Obs	Sub_group	fuptimey	_CENSOR_	SURVIVAL	SDF_LCL	SDF_UCL	STRATUM
38116	0	9.59	1	0.70862	.	.	1
38117	0	9.59	1	0.70862	.	.	1
38118	0	9.59	1	0.70862	.	.	1
38119	0	9.59	1	0.70862	.	.	1
38120	0	9.59	1	0.70862	.	.	1
38121	0	9.59	1	0.70862	.	.	1
38122	0	9.59	1	0.70862	.	.	1
38123	0	9.59	1	0.70862	.	.	1
38124	0	9.59	1	0.70862	.	.	1
38125	0	9.59	1	0.70862	.	.	1
38126	0	9.59	1	0.70862	.	.	1
38127	0	9.59	1	0.70862	.	.	1
38128	0	9.59	1	0.70862	.	.	1
38129	0	9.59	1	0.70862	.	.	1
38130	0	9.59	1	0.70862	.	.	1
38131	0	9.59	1	0.70862	.	.	1
38132	0	9.59	1	0.70862	.	.	1
38133	0	9.59	1	0.70862	.	.	1
38134	0	9.59	1	0.70862	.	.	1
38135	0	9.59	1	0.70862	.	.	1
38136	0	9.59	1	0.70862	.	.	1
38137	0	9.59	1	0.70862	.	.	1
38138	0	9.59	1	0.70862	.	.	1
38139	0	9.59	1	0.70862	.	.	1
38140	0	9.59	1	0.70862	.	.	1
38141	0	9.59	1	0.70862	.	.	1
38142	0	9.59	1	0.70862	.	.	1
38143	0	9.59	1	0.70862	.	.	1
38144	0	9.59	1	0.70862	.	.	1
38145	0	9.59	1	0.70862	.	.	1
38146	0	9.59	1	0.70862	.	.	1
38147	0	9.59	1	0.70862	.	.	1
38148	0	9.59	1	0.70862	.	.	1
38149	0	9.59	1	0.70862	.	.	1
38150	0	9.60	0	0.70806	0.69949	0.71645	1

Obs	Sub_group	fuptimey	_CENSOR_	SURVIVAL	SDF_LCL	SDF_UCL	STRATUM
38151	0	9.60	1	0.70806	.	.	1
38152	0	9.60	1	0.70806	.	.	1
38153	0	9.60	1	0.70806	.	.	1
38154	0	9.60	1	0.70806	.	.	1
38155	0	9.60	1	0.70806	.	.	1
38156	0	9.60	1	0.70806	.	.	1
38157	0	9.60	1	0.70806	.	.	1
38158	0	9.60	1	0.70806	.	.	1
38159	0	9.60	1	0.70806	.	.	1
38160	0	9.60	1	0.70806	.	.	1
38161	0	9.60	1	0.70806	.	.	1
38162	0	9.60	1	0.70806	.	.	1
38163	0	9.60	1	0.70806	.	.	1
38164	0	9.60	1	0.70806	.	.	1
38165	0	9.60	1	0.70806	.	.	1
38166	0	9.60	1	0.70806	.	.	1
38167	0	9.60	1	0.70806	.	.	1
38168	0	9.60	1	0.70806	.	.	1
38169	0	9.60	1	0.70806	.	.	1
38170	0	9.60	1	0.70806	.	.	1
38171	0	9.61	1	0.70806	.	.	1
38172	0	9.61	1	0.70806	.	.	1
38173	0	9.61	1	0.70806	.	.	1
38174	0	9.61	1	0.70806	.	.	1
38175	0	9.61	1	0.70806	.	.	1
38176	0	9.61	1	0.70806	.	.	1
38177	0	9.61	1	0.70806	.	.	1
38178	0	9.61	1	0.70806	.	.	1
38179	0	9.61	1	0.70806	.	.	1
38180	0	9.61	1	0.70806	.	.	1
38181	0	9.61	1	0.70806	.	.	1
38182	0	9.61	1	0.70806	.	.	1
38183	0	9.61	1	0.70806	.	.	1
38184	0	9.61	1	0.70806	.	.	1
38185	0	9.61	1	0.70806	.	.	1

Obs	Sub_group	fuptimey	_CENSOR_	SURVIVAL	SDF_LCL	SDF_UCL	STRATUM
38186	0	9.61	1	0.70806	.	.	1
38187	0	9.61	1	0.70806	.	.	1
38188	0	9.61	1	0.70806	.	.	1
38189	0	9.61	1	0.70806	.	.	1
38190	0	9.61	1	0.70806	.	.	1
38191	0	9.61	1	0.70806	.	.	1
38192	0	9.61	1	0.70806	.	.	1
38193	0	9.61	1	0.70806	.	.	1
38194	0	9.61	1	0.70806	.	.	1
38195	0	9.61	1	0.70806	.	.	1
38196	0	9.62	0	0.70691	0.69819	0.71542	1
38197	0	9.62	1	0.70691	.	.	1
38198	0	9.62	1	0.70691	.	.	1
38199	0	9.62	1	0.70691	.	.	1
38200	0	9.62	1	0.70691	.	.	1
38201	0	9.62	1	0.70691	.	.	1
38202	0	9.62	1	0.70691	.	.	1
38203	0	9.62	1	0.70691	.	.	1
38204	0	9.62	1	0.70691	.	.	1
38205	0	9.62	1	0.70691	.	.	1
38206	0	9.62	1	0.70691	.	.	1
38207	0	9.62	1	0.70691	.	.	1
38208	0	9.62	1	0.70691	.	.	1
38209	0	9.62	1	0.70691	.	.	1
38210	0	9.62	1	0.70691	.	.	1
38211	0	9.62	1	0.70691	.	.	1
38212	0	9.62	1	0.70691	.	.	1
38213	0	9.62	1	0.70691	.	.	1
38214	0	9.62	1	0.70691	.	.	1
38215	0	9.62	1	0.70691	.	.	1
38216	0	9.62	1	0.70691	.	.	1
38217	0	9.62	1	0.70691	.	.	1
38218	0	9.62	1	0.70691	.	.	1
38219	0	9.62	1	0.70691	.	.	1
38220	0	9.62	1	0.70691	.	.	1

Obs	Sub_group	fuptimey	_CENSOR_	SURVIVAL	SDF_LCL	SDF_UCL	STRATUM
38221	0	9.62	1	0.70691	.	.	1
38222	0	9.62	1	0.70691	.	.	1
38223	0	9.62	1	0.70691	.	.	1
38224	0	9.62	1	0.70691	.	.	1
38225	0	9.62	1	0.70691	.	.	1
38226	0	9.63	0	0.70632	0.69753	0.71490	1
38227	0	9.63	1	0.70632	.	.	1
38228	0	9.63	1	0.70632	.	.	1
38229	0	9.63	1	0.70632	.	.	1
38230	0	9.63	1	0.70632	.	.	1
38231	0	9.63	1	0.70632	.	.	1
38232	0	9.63	1	0.70632	.	.	1
38233	0	9.63	1	0.70632	.	.	1
38234	0	9.63	1	0.70632	.	.	1
38235	0	9.63	1	0.70632	.	.	1
38236	0	9.63	1	0.70632	.	.	1
38237	0	9.63	1	0.70632	.	.	1
38238	0	9.63	1	0.70632	.	.	1
38239	0	9.63	1	0.70632	.	.	1
38240	0	9.63	1	0.70632	.	.	1
38241	0	9.63	1	0.70632	.	.	1
38242	0	9.63	1	0.70632	.	.	1
38243	0	9.63	1	0.70632	.	.	1
38244	0	9.63	1	0.70632	.	.	1
38245	0	9.63	1	0.70632	.	.	1
38246	0	9.63	1	0.70632	.	.	1
38247	0	9.63	1	0.70632	.	.	1
38248	0	9.63	1	0.70632	.	.	1
38249	0	9.63	1	0.70632	.	.	1
38250	0	9.63	1	0.70632	.	.	1
38251	0	9.63	1	0.70632	.	.	1
38252	0	9.63	1	0.70632	.	.	1
38253	0	9.63	1	0.70632	.	.	1
38254	0	9.63	1	0.70632	.	.	1
38255	0	9.63	1	0.70632	.	.	1

Obs	Sub_group	fuptimey	_CENSOR_	SURVIVAL	SDF_LCL	SDF_UCL	STRATUM
38256	0	9.63	1	0.70632	.	.	1
38257	0	9.63	1	0.70632	.	.	1
38258	0	9.63	1	0.70632	.	.	1
38259	0	9.63	1	0.70632	.	.	1
38260	0	9.64	1	0.70632	.	.	1
38261	0	9.64	1	0.70632	.	.	1
38262	0	9.64	1	0.70632	.	.	1
38263	0	9.64	1	0.70632	.	.	1
38264	0	9.64	1	0.70632	.	.	1
38265	0	9.64	1	0.70632	.	.	1
38266	0	9.64	1	0.70632	.	.	1
38267	0	9.64	1	0.70632	.	.	1
38268	0	9.64	1	0.70632	.	.	1
38269	0	9.64	1	0.70632	.	.	1
38270	0	9.64	1	0.70632	.	.	1
38271	0	9.64	1	0.70632	.	.	1
38272	0	9.64	1	0.70632	.	.	1
38273	0	9.64	1	0.70632	.	.	1
38274	0	9.64	1	0.70632	.	.	1
38275	0	9.64	1	0.70632	.	.	1
38276	0	9.64	1	0.70632	.	.	1
38277	0	9.64	1	0.70632	.	.	1
38278	0	9.64	1	0.70632	.	.	1
38279	0	9.64	1	0.70632	.	.	1
38280	0	9.64	1	0.70632	.	.	1
38281	0	9.64	1	0.70632	.	.	1
38282	0	9.64	1	0.70632	.	.	1
38283	0	9.64	1	0.70632	.	.	1
38284	0	9.64	1	0.70632	.	.	1
38285	0	9.64	1	0.70632	.	.	1
38286	0	9.65	0	0.70507	0.69613	0.71381	1
38287	0	9.65	1	0.70507	.	.	1
38288	0	9.65	1	0.70507	.	.	1
38289	0	9.65	1	0.70507	.	.	1
38290	0	9.65	1	0.70507	.	.	1

Obs	Sub_group	fuptimey	_CENSOR_	SURVIVAL	SDF_LCL	SDF_UCL	STRATUM
38291	0	9.65	1	0.70507	.	.	1
38292	0	9.65	1	0.70507	.	.	1
38293	0	9.65	1	0.70507	.	.	1
38294	0	9.65	1	0.70507	.	.	1
38295	0	9.65	1	0.70507	.	.	1
38296	0	9.65	1	0.70507	.	.	1
38297	0	9.65	1	0.70507	.	.	1
38298	0	9.65	1	0.70507	.	.	1
38299	0	9.65	1	0.70507	.	.	1
38300	0	9.65	1	0.70507	.	.	1
38301	0	9.65	1	0.70507	.	.	1
38302	0	9.65	1	0.70507	.	.	1
38303	0	9.65	1	0.70507	.	.	1
38304	0	9.65	1	0.70507	.	.	1
38305	0	9.65	1	0.70507	.	.	1
38306	0	9.65	1	0.70507	.	.	1
38307	0	9.65	1	0.70507	.	.	1
38308	0	9.65	1	0.70507	.	.	1
38309	0	9.65	1	0.70507	.	.	1
38310	0	9.65	1	0.70507	.	.	1
38311	0	9.65	1	0.70507	.	.	1
38312	0	9.65	1	0.70507	.	.	1
38313	0	9.65	1	0.70507	.	.	1
38314	0	9.65	1	0.70507	.	.	1
38315	0	9.65	1	0.70507	.	.	1
38316	0	9.65	1	0.70507	.	.	1
38317	0	9.65	1	0.70507	.	.	1
38318	0	9.65	1	0.70507	.	.	1
38319	0	9.66	1	0.70507	.	.	1
38320	0	9.66	1	0.70507	.	.	1
38321	0	9.66	1	0.70507	.	.	1
38322	0	9.66	1	0.70507	.	.	1
38323	0	9.66	1	0.70507	.	.	1
38324	0	9.66	1	0.70507	.	.	1
38325	0	9.66	1	0.70507	.	.	1

Obs	Sub_group	fuptimey	_CENSOR_	SURVIVAL	SDF_LCL	SDF_UCL	STRATUM
38326	0	9.66	1	0.70507	.	.	1
38327	0	9.66	1	0.70507	.	.	1
38328	0	9.66	1	0.70507	.	.	1
38329	0	9.66	1	0.70507	.	.	1
38330	0	9.66	1	0.70507	.	.	1
38331	0	9.66	1	0.70507	.	.	1
38332	0	9.66	1	0.70507	.	.	1
38333	0	9.66	1	0.70507	.	.	1
38334	0	9.66	1	0.70507	.	.	1
38335	0	9.66	1	0.70507	.	.	1
38336	0	9.66	1	0.70507	.	.	1
38337	0	9.66	1	0.70507	.	.	1
38338	0	9.66	1	0.70507	.	.	1
38339	0	9.66	1	0.70507	.	.	1
38340	0	9.66	1	0.70507	.	.	1
38341	0	9.66	1	0.70507	.	.	1
38342	0	9.66	1	0.70507	.	.	1
38343	0	9.66	1	0.70507	.	.	1
38344	0	9.66	1	0.70507	.	.	1
38345	0	9.66	1	0.70507	.	.	1
38346	0	9.66	1	0.70507	.	.	1
38347	0	9.66	1	0.70507	.	.	1
38348	0	9.66	1	0.70507	.	.	1
38349	0	9.67	0	0.70375	0.69464	0.71266	1
38350	0	9.67	1	0.70375	.	.	1
38351	0	9.67	1	0.70375	.	.	1
38352	0	9.67	1	0.70375	.	.	1
38353	0	9.67	1	0.70375	.	.	1
38354	0	9.67	1	0.70375	.	.	1
38355	0	9.67	1	0.70375	.	.	1
38356	0	9.67	1	0.70375	.	.	1
38357	0	9.67	1	0.70375	.	.	1
38358	0	9.67	1	0.70375	.	.	1
38359	0	9.67	1	0.70375	.	.	1
38360	0	9.67	1	0.70375	.	.	1

Obs	Sub_group	fuptimey	_CENSOR_	SURVIVAL	SDF_LCL	SDF_UCL	STRATUM
38361	0	9.67	1	0.70375	.	.	1
38362	0	9.67	1	0.70375	.	.	1
38363	0	9.67	1	0.70375	.	.	1
38364	0	9.67	1	0.70375	.	.	1
38365	0	9.67	1	0.70375	.	.	1
38366	0	9.67	1	0.70375	.	.	1
38367	0	9.67	1	0.70375	.	.	1
38368	0	9.67	1	0.70375	.	.	1
38369	0	9.67	1	0.70375	.	.	1
38370	0	9.67	1	0.70375	.	.	1
38371	0	9.68	1	0.70375	.	.	1
38372	0	9.68	1	0.70375	.	.	1
38373	0	9.68	1	0.70375	.	.	1
38374	0	9.68	1	0.70375	.	.	1
38375	0	9.68	1	0.70375	.	.	1
38376	0	9.68	1	0.70375	.	.	1
38377	0	9.68	1	0.70375	.	.	1
38378	0	9.68	1	0.70375	.	.	1
38379	0	9.68	1	0.70375	.	.	1
38380	0	9.68	1	0.70375	.	.	1
38381	0	9.68	1	0.70375	.	.	1
38382	0	9.68	1	0.70375	.	.	1
38383	0	9.68	1	0.70375	.	.	1
38384	0	9.68	1	0.70375	.	.	1
38385	0	9.68	1	0.70375	.	.	1
38386	0	9.68	1	0.70375	.	.	1
38387	0	9.68	1	0.70375	.	.	1
38388	0	9.68	1	0.70375	.	.	1
38389	0	9.68	1	0.70375	.	.	1
38390	0	9.68	1	0.70375	.	.	1
38391	0	9.68	1	0.70375	.	.	1
38392	0	9.68	1	0.70375	.	.	1
38393	0	9.68	1	0.70375	.	.	1
38394	0	9.68	1	0.70375	.	.	1
38395	0	9.68	1	0.70375	.	.	1

Obs	Sub_group	fuptimey	_CENSOR_	SURVIVAL	SDF_LCL	SDF_UCL	STRATUM
38396	0	9.68	1	0.70375	.	.	1
38397	0	9.68	1	0.70375	.	.	1
38398	0	9.68	1	0.70375	.	.	1
38399	0	9.68	1	0.70375	.	.	1
38400	0	9.68	1	0.70375	.	.	1
38401	0	9.68	1	0.70375	.	.	1
38402	0	9.68	1	0.70375	.	.	1
38403	0	9.68	1	0.70375	.	.	1
38404	0	9.68	1	0.70375	.	.	1
38405	0	9.68	1	0.70375	.	.	1
38406	0	9.68	1	0.70375	.	.	1
38407	0	9.68	1	0.70375	.	.	1
38408	0	9.68	1	0.70375	.	.	1
38409	0	9.69	0	0.70236	0.69305	0.71145	1
38410	0	9.69	1	0.70236	.	.	1
38411	0	9.69	1	0.70236	.	.	1
38412	0	9.69	1	0.70236	.	.	1
38413	0	9.69	1	0.70236	.	.	1
38414	0	9.69	1	0.70236	.	.	1
38415	0	9.69	1	0.70236	.	.	1
38416	0	9.69	1	0.70236	.	.	1
38417	0	9.69	1	0.70236	.	.	1
38418	0	9.69	1	0.70236	.	.	1
38419	0	9.69	1	0.70236	.	.	1
38420	0	9.69	1	0.70236	.	.	1
38421	0	9.69	1	0.70236	.	.	1
38422	0	9.69	1	0.70236	.	.	1
38423	0	9.69	1	0.70236	.	.	1
38424	0	9.69	1	0.70236	.	.	1
38425	0	9.69	1	0.70236	.	.	1
38426	0	9.69	1	0.70236	.	.	1
38427	0	9.69	1	0.70236	.	.	1
38428	0	9.69	1	0.70236	.	.	1
38429	0	9.69	1	0.70236	.	.	1
38430	0	9.69	1	0.70236	.	.	1

Obs	Sub_group	fuptimey	_CENSOR_	SURVIVAL	SDF_LCL	SDF_UCL	STRATUM
38431	0	9.69	1	0.70236	.	.	1
38432	0	9.69	1	0.70236	.	.	1
38433	0	9.69	1	0.70236	.	.	1
38434	0	9.69	1	0.70236	.	.	1
38435	0	9.69	1	0.70236	.	.	1
38436	0	9.69	1	0.70236	.	.	1
38437	0	9.69	1	0.70236	.	.	1
38438	0	9.69	1	0.70236	.	.	1
38439	0	9.69	1	0.70236	.	.	1
38440	0	9.69	1	0.70236	.	.	1
38441	0	9.69	1	0.70236	.	.	1
38442	0	9.69	1	0.70236	.	.	1
38443	0	9.69	1	0.70236	.	.	1
38444	0	9.69	1	0.70236	.	.	1
38445	0	9.69	1	0.70236	.	.	1
38446	0	9.70	0	0.70092	0.69141	0.71020	1
38447	0	9.70	1	0.70092	.	.	1
38448	0	9.70	1	0.70092	.	.	1
38449	0	9.70	1	0.70092	.	.	1
38450	0	9.70	1	0.70092	.	.	1
38451	0	9.70	1	0.70092	.	.	1
38452	0	9.70	1	0.70092	.	.	1
38453	0	9.70	1	0.70092	.	.	1
38454	0	9.70	1	0.70092	.	.	1
38455	0	9.70	1	0.70092	.	.	1
38456	0	9.70	1	0.70092	.	.	1
38457	0	9.70	1	0.70092	.	.	1
38458	0	9.70	1	0.70092	.	.	1
38459	0	9.70	1	0.70092	.	.	1
38460	0	9.70	1	0.70092	.	.	1
38461	0	9.70	1	0.70092	.	.	1
38462	0	9.70	1	0.70092	.	.	1
38463	0	9.70	1	0.70092	.	.	1
38464	0	9.70	1	0.70092	.	.	1
38465	0	9.70	1	0.70092	.	.	1

Obs	Sub_group	fuptimey	_CENSOR_	SURVIVAL	SDF_LCL	SDF_UCL	STRATUM
38466	0	9.70	1	0.70092	.	.	1
38467	0	9.70	1	0.70092	.	.	1
38468	0	9.70	1	0.70092	.	.	1
38469	0	9.70	1	0.70092	.	.	1
38470	0	9.70	1	0.70092	.	.	1
38471	0	9.70	1	0.70092	.	.	1
38472	0	9.70	1	0.70092	.	.	1
38473	0	9.70	1	0.70092	.	.	1
38474	0	9.70	1	0.70092	.	.	1
38475	0	9.70	1	0.70092	.	.	1
38476	0	9.70	1	0.70092	.	.	1
38477	0	9.70	1	0.70092	.	.	1
38478	0	9.70	1	0.70092	.	.	1
38479	0	9.70	1	0.70092	.	.	1
38480	0	9.70	1	0.70092	.	.	1
38481	0	9.70	1	0.70092	.	.	1
38482	0	9.70	1	0.70092	.	.	1
38483	0	9.70	1	0.70092	.	.	1
38484	0	9.70	1	0.70092	.	.	1
38485	0	9.70	1	0.70092	.	.	1
38486	0	9.70	1	0.70092	.	.	1
38487	0	9.70	1	0.70092	.	.	1
38488	0	9.70	1	0.70092	.	.	1
38489	0	9.71	0	0.70016	0.69054	0.70954	1
38490	0	9.71	1	0.70016	.	.	1
38491	0	9.71	1	0.70016	.	.	1
38492	0	9.71	1	0.70016	.	.	1
38493	0	9.71	1	0.70016	.	.	1
38494	0	9.71	1	0.70016	.	.	1
38495	0	9.71	1	0.70016	.	.	1
38496	0	9.71	1	0.70016	.	.	1
38497	0	9.71	1	0.70016	.	.	1
38498	0	9.71	1	0.70016	.	.	1
38499	0	9.71	1	0.70016	.	.	1
38500	0	9.71	1	0.70016	.	.	1

Obs	Sub_group	fuptimey	_CENSOR_	SURVIVAL	SDF_LCL	SDF_UCL	STRATUM
38501	0	9.71	1	0.70016	.	.	1
38502	0	9.71	1	0.70016	.	.	1
38503	0	9.71	1	0.70016	.	.	1
38504	0	9.71	1	0.70016	.	.	1
38505	0	9.71	1	0.70016	.	.	1
38506	0	9.71	1	0.70016	.	.	1
38507	0	9.71	1	0.70016	.	.	1
38508	0	9.71	1	0.70016	.	.	1
38509	0	9.71	1	0.70016	.	.	1
38510	0	9.71	1	0.70016	.	.	1
38511	0	9.71	1	0.70016	.	.	1
38512	0	9.71	1	0.70016	.	.	1
38513	0	9.71	1	0.70016	.	.	1
38514	0	9.71	1	0.70016	.	.	1
38515	0	9.71	1	0.70016	.	.	1
38516	0	9.72	0	0.69938	0.68965	0.70888	1
38517	0	9.72	1	0.69938	.	.	1
38518	0	9.72	1	0.69938	.	.	1
38519	0	9.72	1	0.69938	.	.	1
38520	0	9.72	1	0.69938	.	.	1
38521	0	9.72	1	0.69938	.	.	1
38522	0	9.72	1	0.69938	.	.	1
38523	0	9.72	1	0.69938	.	.	1
38524	0	9.72	1	0.69938	.	.	1
38525	0	9.72	1	0.69938	.	.	1
38526	0	9.72	1	0.69938	.	.	1
38527	0	9.72	1	0.69938	.	.	1
38528	0	9.72	1	0.69938	.	.	1
38529	0	9.72	1	0.69938	.	.	1
38530	0	9.72	1	0.69938	.	.	1
38531	0	9.72	1	0.69938	.	.	1
38532	0	9.72	1	0.69938	.	.	1
38533	0	9.72	1	0.69938	.	.	1
38534	0	9.72	1	0.69938	.	.	1
38535	0	9.72	1	0.69938	.	.	1

Obs	Sub_group	fuptimey	_CENSOR_	SURVIVAL	SDF_LCL	SDF_UCL	STRATUM
38536	0	9.72	1	0.69938	.	.	1
38537	0	9.72	1	0.69938	.	.	1
38538	0	9.72	1	0.69938	.	.	1
38539	0	9.72	1	0.69938	.	.	1
38540	0	9.72	1	0.69938	.	.	1
38541	0	9.72	1	0.69938	.	.	1
38542	0	9.72	1	0.69938	.	.	1
38543	0	9.72	1	0.69938	.	.	1
38544	0	9.72	1	0.69938	.	.	1
38545	0	9.72	1	0.69938	.	.	1
38546	0	9.73	0	0.69778	0.68781	0.70750	1
38547	0	9.73	1	0.69778	.	.	1
38548	0	9.73	1	0.69778	.	.	1
38549	0	9.73	1	0.69778	.	.	1
38550	0	9.73	1	0.69778	.	.	1
38551	0	9.73	1	0.69778	.	.	1
38552	0	9.73	1	0.69778	.	.	1
38553	0	9.73	1	0.69778	.	.	1
38554	0	9.73	1	0.69778	.	.	1
38555	0	9.73	1	0.69778	.	.	1
38556	0	9.73	1	0.69778	.	.	1
38557	0	9.73	1	0.69778	.	.	1
38558	0	9.73	1	0.69778	.	.	1
38559	0	9.73	1	0.69778	.	.	1
38560	0	9.73	1	0.69778	.	.	1
38561	0	9.73	1	0.69778	.	.	1
38562	0	9.73	1	0.69778	.	.	1
38563	0	9.73	1	0.69778	.	.	1
38564	0	9.73	1	0.69778	.	.	1
38565	0	9.73	1	0.69778	.	.	1
38566	0	9.73	1	0.69778	.	.	1
38567	0	9.73	1	0.69778	.	.	1
38568	0	9.73	1	0.69778	.	.	1
38569	0	9.74	0	0.69695	0.68686	0.70679	1
38570	0	9.74	1	0.69695	.	.	1

Obs	Sub_group	fuptimey	_CENSOR_	SURVIVAL	SDF_LCL	SDF_UCL	STRATUM
38571	0	9.74	1	0.69695	.	.	1
38572	0	9.74	1	0.69695	.	.	1
38573	0	9.74	1	0.69695	.	.	1
38574	0	9.74	1	0.69695	.	.	1
38575	0	9.74	1	0.69695	.	.	1
38576	0	9.74	1	0.69695	.	.	1
38577	0	9.74	1	0.69695	.	.	1
38578	0	9.74	1	0.69695	.	.	1
38579	0	9.74	1	0.69695	.	.	1
38580	0	9.74	1	0.69695	.	.	1
38581	0	9.74	1	0.69695	.	.	1
38582	0	9.74	1	0.69695	.	.	1
38583	0	9.74	1	0.69695	.	.	1
38584	0	9.74	1	0.69695	.	.	1
38585	0	9.74	1	0.69695	.	.	1
38586	0	9.74	1	0.69695	.	.	1
38587	0	9.74	1	0.69695	.	.	1
38588	0	9.74	1	0.69695	.	.	1
38589	0	9.74	1	0.69695	.	.	1
38590	0	9.74	1	0.69695	.	.	1
38591	0	9.74	1	0.69695	.	.	1
38592	0	9.74	1	0.69695	.	.	1
38593	0	9.74	1	0.69695	.	.	1
38594	0	9.75	0	0.69611	0.68589	0.70607	1
38595	0	9.75	1	0.69611	.	.	1
38596	0	9.75	1	0.69611	.	.	1
38597	0	9.75	1	0.69611	.	.	1
38598	0	9.75	1	0.69611	.	.	1
38599	0	9.75	1	0.69611	.	.	1
38600	0	9.75	1	0.69611	.	.	1
38601	0	9.75	1	0.69611	.	.	1
38602	0	9.75	1	0.69611	.	.	1
38603	0	9.75	1	0.69611	.	.	1
38604	0	9.75	1	0.69611	.	.	1
38605	0	9.75	1	0.69611	.	.	1

Obs	Sub_group	fuptimey	_CENSOR_	SURVIVAL	SDF_LCL	SDF_UCL	STRATUM
38606	0	9.75	1	0.69611	.	.	1
38607	0	9.75	1	0.69611	.	.	1
38608	0	9.75	1	0.69611	.	.	1
38609	0	9.75	1	0.69611	.	.	1
38610	0	9.75	1	0.69611	.	.	1
38611	0	9.75	1	0.69611	.	.	1
38612	0	9.75	1	0.69611	.	.	1
38613	0	9.75	1	0.69611	.	.	1
38614	0	9.75	1	0.69611	.	.	1
38615	0	9.75	1	0.69611	.	.	1
38616	0	9.76	0	0.69524	0.68489	0.70532	1
38617	0	9.76	1	0.69524	.	.	1
38618	0	9.76	1	0.69524	.	.	1
38619	0	9.76	1	0.69524	.	.	1
38620	0	9.76	1	0.69524	.	.	1
38621	0	9.76	1	0.69524	.	.	1
38622	0	9.76	1	0.69524	.	.	1
38623	0	9.76	1	0.69524	.	.	1
38624	0	9.76	1	0.69524	.	.	1
38625	0	9.76	1	0.69524	.	.	1
38626	0	9.76	1	0.69524	.	.	1
38627	0	9.76	1	0.69524	.	.	1
38628	0	9.76	1	0.69524	.	.	1
38629	0	9.76	1	0.69524	.	.	1
38630	0	9.76	1	0.69524	.	.	1
38631	0	9.76	1	0.69524	.	.	1
38632	0	9.76	1	0.69524	.	.	1
38633	0	9.76	1	0.69524	.	.	1
38634	0	9.76	1	0.69524	.	.	1
38635	0	9.76	1	0.69524	.	.	1
38636	0	9.76	1	0.69524	.	.	1
38637	0	9.76	1	0.69524	.	.	1
38638	0	9.76	1	0.69524	.	.	1
38639	0	9.76	1	0.69524	.	.	1
38640	0	9.76	1	0.69524	.	.	1

Obs	Sub_group	fuptimey	_CENSOR_	SURVIVAL	SDF_LCL	SDF_UCL	STRATUM
38641	0	9.76	1	0.69524	.	.	1
38642	0	9.76	1	0.69524	.	.	1
38643	0	9.76	1	0.69524	.	.	1
38644	0	9.77	1	0.69524	.	.	1
38645	0	9.77	1	0.69524	.	.	1
38646	0	9.77	1	0.69524	.	.	1
38647	0	9.77	1	0.69524	.	.	1
38648	0	9.77	1	0.69524	.	.	1
38649	0	9.77	1	0.69524	.	.	1
38650	0	9.77	1	0.69524	.	.	1
38651	0	9.77	1	0.69524	.	.	1
38652	0	9.77	1	0.69524	.	.	1
38653	0	9.77	1	0.69524	.	.	1
38654	0	9.77	1	0.69524	.	.	1
38655	0	9.77	1	0.69524	.	.	1
38656	0	9.77	1	0.69524	.	.	1
38657	0	9.77	1	0.69524	.	.	1
38658	0	9.77	1	0.69524	.	.	1
38659	0	9.77	1	0.69524	.	.	1
38660	0	9.77	1	0.69524	.	.	1
38661	0	9.77	1	0.69524	.	.	1
38662	0	9.77	1	0.69524	.	.	1
38663	0	9.77	1	0.69524	.	.	1
38664	0	9.77	1	0.69524	.	.	1
38665	0	9.78	1	0.69524	.	.	1
38666	0	9.78	1	0.69524	.	.	1
38667	0	9.78	1	0.69524	.	.	1
38668	0	9.78	1	0.69524	.	.	1
38669	0	9.78	1	0.69524	.	.	1
38670	0	9.78	1	0.69524	.	.	1
38671	0	9.78	1	0.69524	.	.	1
38672	0	9.78	1	0.69524	.	.	1
38673	0	9.78	1	0.69524	.	.	1
38674	0	9.78	1	0.69524	.	.	1
38675	0	9.78	1	0.69524	.	.	1

Obs	Sub_group	fuptimey	_CENSOR_	SURVIVAL	SDF_LCL	SDF_UCL	STRATUM
38676	0	9.78	1	0.69524	.	.	1
38677	0	9.78	1	0.69524	.	.	1
38678	0	9.78	1	0.69524	.	.	1
38679	0	9.78	1	0.69524	.	.	1
38680	0	9.78	1	0.69524	.	.	1
38681	0	9.78	1	0.69524	.	.	1
38682	0	9.78	1	0.69524	.	.	1
38683	0	9.78	1	0.69524	.	.	1
38684	0	9.78	1	0.69524	.	.	1
38685	0	9.78	1	0.69524	.	.	1
38686	0	9.78	1	0.69524	.	.	1
38687	0	9.78	1	0.69524	.	.	1
38688	0	9.78	1	0.69524	.	.	1
38689	0	9.78	1	0.69524	.	.	1
38690	0	9.78	1	0.69524	.	.	1
38691	0	9.78	1	0.69524	.	.	1
38692	0	9.78	1	0.69524	.	.	1
38693	0	9.78	1	0.69524	.	.	1
38694	0	9.78	1	0.69524	.	.	1
38695	0	9.78	1	0.69524	.	.	1
38696	0	9.78	1	0.69524	.	.	1
38697	0	9.78	1	0.69524	.	.	1
38698	0	9.78	1	0.69524	.	.	1
38699	0	9.78	1	0.69524	.	.	1
38700	0	9.79	0	0.69426	0.68375	0.70451	1
38701	0	9.79	1	0.69426	.	.	1
38702	0	9.79	1	0.69426	.	.	1
38703	0	9.79	1	0.69426	.	.	1
38704	0	9.79	1	0.69426	.	.	1
38705	0	9.79	1	0.69426	.	.	1
38706	0	9.79	1	0.69426	.	.	1
38707	0	9.79	1	0.69426	.	.	1
38708	0	9.79	1	0.69426	.	.	1
38709	0	9.79	1	0.69426	.	.	1
38710	0	9.79	1	0.69426	.	.	1

Obs	Sub_group	fuptimey	_CENSOR_	SURVIVAL	SDF_LCL	SDF_UCL	STRATUM
38711	0	9.79	1	0.69426	.	.	1
38712	0	9.79	1	0.69426	.	.	1
38713	0	9.79	1	0.69426	.	.	1
38714	0	9.79	1	0.69426	.	.	1
38715	0	9.79	1	0.69426	.	.	1
38716	0	9.79	1	0.69426	.	.	1
38717	0	9.79	1	0.69426	.	.	1
38718	0	9.79	1	0.69426	.	.	1
38719	0	9.79	1	0.69426	.	.	1
38720	0	9.79	1	0.69426	.	.	1
38721	0	9.79	1	0.69426	.	.	1
38722	0	9.79	1	0.69426	.	.	1
38723	0	9.79	1	0.69426	.	.	1
38724	0	9.79	1	0.69426	.	.	1
38725	0	9.79	1	0.69426	.	.	1
38726	0	9.80	1	0.69426	.	.	1
38727	0	9.80	1	0.69426	.	.	1
38728	0	9.80	1	0.69426	.	.	1
38729	0	9.80	1	0.69426	.	.	1
38730	0	9.80	1	0.69426	.	.	1
38731	0	9.80	1	0.69426	.	.	1
38732	0	9.80	1	0.69426	.	.	1
38733	0	9.80	1	0.69426	.	.	1
38734	0	9.80	1	0.69426	.	.	1
38735	0	9.80	1	0.69426	.	.	1
38736	0	9.80	1	0.69426	.	.	1
38737	0	9.80	1	0.69426	.	.	1
38738	0	9.80	1	0.69426	.	.	1
38739	0	9.80	1	0.69426	.	.	1
38740	0	9.80	1	0.69426	.	.	1
38741	0	9.80	1	0.69426	.	.	1
38742	0	9.80	1	0.69426	.	.	1
38743	0	9.80	1	0.69426	.	.	1
38744	0	9.80	1	0.69426	.	.	1
38745	0	9.80	1	0.69426	.	.	1

Obs	Sub_group	fuptimey	_CENSOR_	SURVIVAL	SDF_LCL	SDF_UCL	STRATUM
38746	0	9.80	1	0.69426	.	.	1
38747	0	9.80	1	0.69426	.	.	1
38748	0	9.80	1	0.69426	.	.	1
38749	0	9.80	1	0.69426	.	.	1
38750	0	9.80	1	0.69426	.	.	1
38751	0	9.80	1	0.69426	.	.	1
38752	0	9.80	1	0.69426	.	.	1
38753	0	9.80	1	0.69426	.	.	1
38754	0	9.80	1	0.69426	.	.	1
38755	0	9.80	1	0.69426	.	.	1
38756	0	9.80	1	0.69426	.	.	1
38757	0	9.80	1	0.69426	.	.	1
38758	0	9.80	1	0.69426	.	.	1
38759	0	9.80	1	0.69426	.	.	1
38760	0	9.81	1	0.69426	.	.	1
38761	0	9.81	1	0.69426	.	.	1
38762	0	9.81	1	0.69426	.	.	1
38763	0	9.81	1	0.69426	.	.	1
38764	0	9.81	1	0.69426	.	.	1
38765	0	9.81	1	0.69426	.	.	1
38766	0	9.81	1	0.69426	.	.	1
38767	0	9.81	1	0.69426	.	.	1
38768	0	9.81	1	0.69426	.	.	1
38769	0	9.81	1	0.69426	.	.	1
38770	0	9.81	1	0.69426	.	.	1
38771	0	9.81	1	0.69426	.	.	1
38772	0	9.81	1	0.69426	.	.	1
38773	0	9.81	1	0.69426	.	.	1
38774	0	9.81	1	0.69426	.	.	1
38775	0	9.81	1	0.69426	.	.	1
38776	0	9.81	1	0.69426	.	.	1
38777	0	9.81	1	0.69426	.	.	1
38778	0	9.81	1	0.69426	.	.	1
38779	0	9.81	1	0.69426	.	.	1
38780	0	9.81	1	0.69426	.	.	1

Obs	Sub_group	fuptimey	_CENSOR_	SURVIVAL	SDF_LCL	SDF_UCL	STRATUM
38781	0	9.81	1	0.69426	.	.	1
38782	0	9.81	1	0.69426	.	.	1
38783	0	9.81	1	0.69426	.	.	1
38784	0	9.81	1	0.69426	.	.	1
38785	0	9.81	1	0.69426	.	.	1
38786	0	9.81	1	0.69426	.	.	1
38787	0	9.82	1	0.69426	.	.	1
38788	0	9.82	1	0.69426	.	.	1
38789	0	9.82	1	0.69426	.	.	1
38790	0	9.82	1	0.69426	.	.	1
38791	0	9.82	1	0.69426	.	.	1
38792	0	9.82	1	0.69426	.	.	1
38793	0	9.82	1	0.69426	.	.	1
38794	0	9.82	1	0.69426	.	.	1
38795	0	9.82	1	0.69426	.	.	1
38796	0	9.82	1	0.69426	.	.	1
38797	0	9.82	1	0.69426	.	.	1
38798	0	9.82	1	0.69426	.	.	1
38799	0	9.82	1	0.69426	.	.	1
38800	0	9.82	1	0.69426	.	.	1
38801	0	9.82	1	0.69426	.	.	1
38802	0	9.82	1	0.69426	.	.	1
38803	0	9.82	1	0.69426	.	.	1
38804	0	9.82	1	0.69426	.	.	1
38805	0	9.82	1	0.69426	.	.	1
38806	0	9.82	1	0.69426	.	.	1
38807	0	9.82	1	0.69426	.	.	1
38808	0	9.82	1	0.69426	.	.	1
38809	0	9.82	1	0.69426	.	.	1
38810	0	9.82	1	0.69426	.	.	1
38811	0	9.82	1	0.69426	.	.	1
38812	0	9.82	1	0.69426	.	.	1
38813	0	9.82	1	0.69426	.	.	1
38814	0	9.82	1	0.69426	.	.	1
38815	0	9.82	1	0.69426	.	.	1

Obs	Sub_group	fuptimey	_CENSOR_	SURVIVAL	SDF_LCL	SDF_UCL	STRATUM
38816	0	9.82	1	0.69426	.	.	1
38817	0	9.82	1	0.69426	.	.	1
38818	0	9.83	0	0.69194	0.68096	0.70263	1
38819	0	9.83	1	0.69194	.	.	1
38820	0	9.83	1	0.69194	.	.	1
38821	0	9.83	1	0.69194	.	.	1
38822	0	9.83	1	0.69194	.	.	1
38823	0	9.83	1	0.69194	.	.	1
38824	0	9.83	1	0.69194	.	.	1
38825	0	9.83	1	0.69194	.	.	1
38826	0	9.83	1	0.69194	.	.	1
38827	0	9.83	1	0.69194	.	.	1
38828	0	9.83	1	0.69194	.	.	1
38829	0	9.83	1	0.69194	.	.	1
38830	0	9.83	1	0.69194	.	.	1
38831	0	9.83	1	0.69194	.	.	1
38832	0	9.83	1	0.69194	.	.	1
38833	0	9.83	1	0.69194	.	.	1
38834	0	9.83	1	0.69194	.	.	1
38835	0	9.83	1	0.69194	.	.	1
38836	0	9.83	1	0.69194	.	.	1
38837	0	9.83	1	0.69194	.	.	1
38838	0	9.83	1	0.69194	.	.	1
38839	0	9.83	1	0.69194	.	.	1
38840	0	9.83	1	0.69194	.	.	1
38841	0	9.83	1	0.69194	.	.	1
38842	0	9.83	1	0.69194	.	.	1
38843	0	9.83	1	0.69194	.	.	1
38844	0	9.84	1	0.69194	.	.	1
38845	0	9.84	1	0.69194	.	.	1
38846	0	9.84	1	0.69194	.	.	1
38847	0	9.84	1	0.69194	.	.	1
38848	0	9.84	1	0.69194	.	.	1
38849	0	9.84	1	0.69194	.	.	1
38850	0	9.84	1	0.69194	.	.	1

Obs	Sub_group	fuptimey	_CENSOR_	SURVIVAL	SDF_LCL	SDF_UCL	STRATUM
38851	0	9.84	1	0.69194	.	.	1
38852	0	9.84	1	0.69194	.	.	1
38853	0	9.84	1	0.69194	.	.	1
38854	0	9.84	1	0.69194	.	.	1
38855	0	9.84	1	0.69194	.	.	1
38856	0	9.84	1	0.69194	.	.	1
38857	0	9.84	1	0.69194	.	.	1
38858	0	9.84	1	0.69194	.	.	1
38859	0	9.84	1	0.69194	.	.	1
38860	0	9.84	1	0.69194	.	.	1
38861	0	9.84	1	0.69194	.	.	1
38862	0	9.84	1	0.69194	.	.	1
38863	0	9.84	1	0.69194	.	.	1
38864	0	9.84	1	0.69194	.	.	1
38865	0	9.84	1	0.69194	.	.	1
38866	0	9.84	1	0.69194	.	.	1
38867	0	9.84	1	0.69194	.	.	1
38868	0	9.84	1	0.69194	.	.	1
38869	0	9.84	1	0.69194	.	.	1
38870	0	9.84	1	0.69194	.	.	1
38871	0	9.84	1	0.69194	.	.	1
38872	0	9.84	1	0.69194	.	.	1
38873	0	9.84	1	0.69194	.	.	1
38874	0	9.84	1	0.69194	.	.	1
38875	0	9.84	1	0.69194	.	.	1
38876	0	9.84	1	0.69194	.	.	1
38877	0	9.84	1	0.69194	.	.	1
38878	0	9.84	1	0.69194	.	.	1
38879	0	9.84	1	0.69194	.	.	1
38880	0	9.85	0	0.69065	0.67939	0.70160	1
38881	0	9.85	1	0.69065	.	.	1
38882	0	9.85	1	0.69065	.	.	1
38883	0	9.85	1	0.69065	.	.	1
38884	0	9.85	1	0.69065	.	.	1
38885	0	9.85	1	0.69065	.	.	1

Obs	Sub_group	fuptimey	_CENSOR_	SURVIVAL	SDF_LCL	SDF_UCL	STRATUM
38886	0	9.85	1	0.69065	.	.	1
38887	0	9.85	1	0.69065	.	.	1
38888	0	9.85	1	0.69065	.	.	1
38889	0	9.85	1	0.69065	.	.	1
38890	0	9.85	1	0.69065	.	.	1
38891	0	9.85	1	0.69065	.	.	1
38892	0	9.85	1	0.69065	.	.	1
38893	0	9.85	1	0.69065	.	.	1
38894	0	9.85	1	0.69065	.	.	1
38895	0	9.85	1	0.69065	.	.	1
38896	0	9.85	1	0.69065	.	.	1
38897	0	9.85	1	0.69065	.	.	1
38898	0	9.85	1	0.69065	.	.	1
38899	0	9.85	1	0.69065	.	.	1
38900	0	9.85	1	0.69065	.	.	1
38901	0	9.85	1	0.69065	.	.	1
38902	0	9.85	1	0.69065	.	.	1
38903	0	9.85	1	0.69065	.	.	1
38904	0	9.85	1	0.69065	.	.	1
38905	0	9.85	1	0.69065	.	.	1
38906	0	9.86	1	0.69065	.	.	1
38907	0	9.86	1	0.69065	.	.	1
38908	0	9.86	1	0.69065	.	.	1
38909	0	9.86	1	0.69065	.	.	1
38910	0	9.86	1	0.69065	.	.	1
38911	0	9.86	1	0.69065	.	.	1
38912	0	9.86	1	0.69065	.	.	1
38913	0	9.86	1	0.69065	.	.	1
38914	0	9.86	1	0.69065	.	.	1
38915	0	9.86	1	0.69065	.	.	1
38916	0	9.86	1	0.69065	.	.	1
38917	0	9.86	1	0.69065	.	.	1
38918	0	9.86	1	0.69065	.	.	1
38919	0	9.86	1	0.69065	.	.	1
38920	0	9.86	1	0.69065	.	.	1

Obs	Sub_group	fuptimey	_CENSOR_	SURVIVAL	SDF_LCL	SDF_UCL	STRATUM
38921	0	9.86	1	0.69065	.	.	1
38922	0	9.86	1	0.69065	.	.	1
38923	0	9.86	1	0.69065	.	.	1
38924	0	9.86	1	0.69065	.	.	1
38925	0	9.86	1	0.69065	.	.	1
38926	0	9.86	1	0.69065	.	.	1
38927	0	9.86	1	0.69065	.	.	1
38928	0	9.86	1	0.69065	.	.	1
38929	0	9.86	1	0.69065	.	.	1
38930	0	9.86	1	0.69065	.	.	1
38931	0	9.86	1	0.69065	.	.	1
38932	0	9.86	1	0.69065	.	.	1
38933	0	9.86	1	0.69065	.	.	1
38934	0	9.86	1	0.69065	.	.	1
38935	0	9.86	1	0.69065	.	.	1
38936	0	9.86	1	0.69065	.	.	1
38937	0	9.86	1	0.69065	.	.	1
38938	0	9.86	1	0.69065	.	.	1
38939	0	9.86	1	0.69065	.	.	1
38940	0	9.87	0	0.68920	0.67759	0.70048	1
38941	0	9.87	1	0.68920	.	.	1
38942	0	9.87	1	0.68920	.	.	1
38943	0	9.87	1	0.68920	.	.	1
38944	0	9.87	1	0.68920	.	.	1
38945	0	9.87	1	0.68920	.	.	1
38946	0	9.87	1	0.68920	.	.	1
38947	0	9.87	1	0.68920	.	.	1
38948	0	9.87	1	0.68920	.	.	1
38949	0	9.87	1	0.68920	.	.	1
38950	0	9.87	1	0.68920	.	.	1
38951	0	9.87	1	0.68920	.	.	1
38952	0	9.87	1	0.68920	.	.	1
38953	0	9.87	1	0.68920	.	.	1
38954	0	9.87	1	0.68920	.	.	1
38955	0	9.87	1	0.68920	.	.	1

Obs	Sub_group	fuptimey	_CENSOR_	SURVIVAL	SDF_LCL	SDF_UCL	STRATUM
38956	0	9.87	1	0.68920	.	.	1
38957	0	9.87	1	0.68920	.	.	1
38958	0	9.87	1	0.68920	.	.	1
38959	0	9.87	1	0.68920	.	.	1
38960	0	9.87	1	0.68920	.	.	1
38961	0	9.87	1	0.68920	.	.	1
38962	0	9.87	1	0.68920	.	.	1
38963	0	9.87	1	0.68920	.	.	1
38964	0	9.87	1	0.68920	.	.	1
38965	0	9.87	1	0.68920	.	.	1
38966	0	9.87	1	0.68920	.	.	1
38967	0	9.87	1	0.68920	.	.	1
38968	0	9.87	1	0.68920	.	.	1
38969	0	9.87	1	0.68920	.	.	1
38970	0	9.87	1	0.68920	.	.	1
38971	0	9.88	0	0.68764	0.67566	0.69929	1
38972	0	9.88	1	0.68764	.	.	1
38973	0	9.88	1	0.68764	.	.	1
38974	0	9.88	1	0.68764	.	.	1
38975	0	9.88	1	0.68764	.	.	1
38976	0	9.88	1	0.68764	.	.	1
38977	0	9.88	1	0.68764	.	.	1
38978	0	9.88	1	0.68764	.	.	1
38979	0	9.88	1	0.68764	.	.	1
38980	0	9.88	1	0.68764	.	.	1
38981	0	9.88	1	0.68764	.	.	1
38982	0	9.88	1	0.68764	.	.	1
38983	0	9.88	1	0.68764	.	.	1
38984	0	9.88	1	0.68764	.	.	1
38985	0	9.88	1	0.68764	.	.	1
38986	0	9.88	1	0.68764	.	.	1
38987	0	9.88	1	0.68764	.	.	1
38988	0	9.88	1	0.68764	.	.	1
38989	0	9.88	1	0.68764	.	.	1
38990	0	9.88	1	0.68764	.	.	1

Obs	Sub_group	fuptimey	_CENSOR_	SURVIVAL	SDF_LCL	SDF_UCL	STRATUM
38991	0	9.88	1	0.68764	.	.	1
38992	0	9.88	1	0.68764	.	.	1
38993	0	9.88	1	0.68764	.	.	1
38994	0	9.88	1	0.68764	.	.	1
38995	0	9.88	1	0.68764	.	.	1
38996	0	9.88	1	0.68764	.	.	1
38997	0	9.88	1	0.68764	.	.	1
38998	0	9.88	1	0.68764	.	.	1
38999	0	9.88	1	0.68764	.	.	1
39000	0	9.88	1	0.68764	.	.	1
39001	0	9.88	1	0.68764	.	.	1
39002	0	9.88	1	0.68764	.	.	1
39003	0	9.88	1	0.68764	.	.	1
39004	0	9.88	1	0.68764	.	.	1
39005	0	9.88	1	0.68764	.	.	1
39006	0	9.89	0	0.68428	0.67145	0.69673	1
39007	0	9.89	1	0.68428	.	.	1
39008	0	9.89	1	0.68428	.	.	1
39009	0	9.89	1	0.68428	.	.	1
39010	0	9.89	1	0.68428	.	.	1
39011	0	9.89	1	0.68428	.	.	1
39012	0	9.89	1	0.68428	.	.	1
39013	0	9.89	1	0.68428	.	.	1
39014	0	9.89	1	0.68428	.	.	1
39015	0	9.89	1	0.68428	.	.	1
39016	0	9.89	1	0.68428	.	.	1
39017	0	9.89	1	0.68428	.	.	1
39018	0	9.89	1	0.68428	.	.	1
39019	0	9.89	1	0.68428	.	.	1
39020	0	9.89	1	0.68428	.	.	1
39021	0	9.89	1	0.68428	.	.	1
39022	0	9.89	1	0.68428	.	.	1
39023	0	9.89	1	0.68428	.	.	1
39024	0	9.89	1	0.68428	.	.	1
39025	0	9.89	1	0.68428	.	.	1

Obs	Sub_group	fuptimey	_CENSOR_	SURVIVAL	SDF_LCL	SDF_UCL	STRATUM
39026	0	9.89	1	0.68428	.	.	1
39027	0	9.89	1	0.68428	.	.	1
39028	0	9.89	1	0.68428	.	.	1
39029	0	9.89	1	0.68428	.	.	1
39030	0	9.89	1	0.68428	.	.	1
39031	0	9.89	1	0.68428	.	.	1
39032	0	9.89	1	0.68428	.	.	1
39033	0	9.89	1	0.68428	.	.	1
39034	0	9.90	0	0.68248	0.66919	0.69537	1
39035	0	9.90	1	0.68248	.	.	1
39036	0	9.90	1	0.68248	.	.	1
39037	0	9.90	1	0.68248	.	.	1
39038	0	9.90	1	0.68248	.	.	1
39039	0	9.90	1	0.68248	.	.	1
39040	0	9.90	1	0.68248	.	.	1
39041	0	9.90	1	0.68248	.	.	1
39042	0	9.90	1	0.68248	.	.	1
39043	0	9.90	1	0.68248	.	.	1
39044	0	9.90	1	0.68248	.	.	1
39045	0	9.90	1	0.68248	.	.	1
39046	0	9.90	1	0.68248	.	.	1
39047	0	9.90	1	0.68248	.	.	1
39048	0	9.90	1	0.68248	.	.	1
39049	0	9.90	1	0.68248	.	.	1
39050	0	9.90	1	0.68248	.	.	1
39051	0	9.90	1	0.68248	.	.	1
39052	0	9.90	1	0.68248	.	.	1
39053	0	9.90	1	0.68248	.	.	1
39054	0	9.90	1	0.68248	.	.	1
39055	0	9.90	1	0.68248	.	.	1
39056	0	9.90	1	0.68248	.	.	1
39057	0	9.90	1	0.68248	.	.	1
39058	0	9.90	1	0.68248	.	.	1
39059	0	9.90	1	0.68248	.	.	1
39060	0	9.90	1	0.68248	.	.	1

Obs	Sub_group	fuptimey	_CENSOR_	SURVIVAL	SDF_LCL	SDF_UCL	STRATUM
39061	0	9.90	1	0.68248	.	.	1
39062	0	9.90	1	0.68248	.	.	1
39063	0	9.90	1	0.68248	.	.	1
39064	0	9.90	1	0.68248	.	.	1
39065	0	9.90	1	0.68248	.	.	1
39066	0	9.91	1	0.68248	.	.	1
39067	0	9.91	1	0.68248	.	.	1
39068	0	9.91	1	0.68248	.	.	1
39069	0	9.91	1	0.68248	.	.	1
39070	0	9.91	1	0.68248	.	.	1
39071	0	9.91	1	0.68248	.	.	1
39072	0	9.91	1	0.68248	.	.	1
39073	0	9.91	1	0.68248	.	.	1
39074	0	9.91	1	0.68248	.	.	1
39075	0	9.91	1	0.68248	.	.	1
39076	0	9.91	1	0.68248	.	.	1
39077	0	9.91	1	0.68248	.	.	1
39078	0	9.91	1	0.68248	.	.	1
39079	0	9.91	1	0.68248	.	.	1
39080	0	9.91	1	0.68248	.	.	1
39081	0	9.91	1	0.68248	.	.	1
39082	0	9.91	1	0.68248	.	.	1
39083	0	9.91	1	0.68248	.	.	1
39084	0	9.91	1	0.68248	.	.	1
39085	0	9.91	1	0.68248	.	.	1
39086	0	9.91	1	0.68248	.	.	1
39087	0	9.92	0	0.68039	0.66650	0.69385	1
39088	0	9.92	1	0.68039	.	.	1
39089	0	9.92	1	0.68039	.	.	1
39090	0	9.92	1	0.68039	.	.	1
39091	0	9.92	1	0.68039	.	.	1
39092	0	9.92	1	0.68039	.	.	1
39093	0	9.92	1	0.68039	.	.	1
39094	0	9.92	1	0.68039	.	.	1
39095	0	9.92	1	0.68039	.	.	1

Obs	Sub_group	fuptimey	_CENSOR_	SURVIVAL	SDF_LCL	SDF_UCL	STRATUM
39096	0	9.92	1	0.68039	.	.	1
39097	0	9.92	1	0.68039	.	.	1
39098	0	9.92	1	0.68039	.	.	1
39099	0	9.92	1	0.68039	.	.	1
39100	0	9.92	1	0.68039	.	.	1
39101	0	9.92	1	0.68039	.	.	1
39102	0	9.92	1	0.68039	.	.	1
39103	0	9.92	1	0.68039	.	.	1
39104	0	9.92	1	0.68039	.	.	1
39105	0	9.92	1	0.68039	.	.	1
39106	0	9.92	1	0.68039	.	.	1
39107	0	9.92	1	0.68039	.	.	1
39108	0	9.92	1	0.68039	.	.	1
39109	0	9.92	1	0.68039	.	.	1
39110	0	9.92	1	0.68039	.	.	1
39111	0	9.92	1	0.68039	.	.	1
39112	0	9.92	1	0.68039	.	.	1
39113	0	9.92	1	0.68039	.	.	1
39114	0	9.92	1	0.68039	.	.	1
39115	0	9.93	0	0.67812	0.66354	0.69222	1
39116	0	9.93	1	0.67812	.	.	1
39117	0	9.93	1	0.67812	.	.	1
39118	0	9.93	1	0.67812	.	.	1
39119	0	9.93	1	0.67812	.	.	1
39120	0	9.93	1	0.67812	.	.	1
39121	0	9.93	1	0.67812	.	.	1
[truncated: 684,956 more chars]
